# Spatiotemporal dynamics of the postnatal developing primate brain transcriptome

**DOI:** 10.1093/hmg/ddv166

**Published:** 2015-05-07

**Authors:** Trygve E. Bakken, Jeremy A. Miller, Rui Luo, Amy Bernard, Jeffrey L. Bennett, Chang-Kyu Lee, Darren Bertagnolli, Neelroop N. Parikshak, Kimberly A. Smith, Susan M. Sunkin, David G. Amaral, Daniel H. Geschwind, Ed S. Lein

**Affiliations:** 1 Allen Institute for Brain Science, Seattle, WA, USA,; 2 Human Genetics Program, Department of Neurology and Semel Institute, David Geffen School of Medicine, UC, Los Angeles, Los Angeles, CA, USA and; 3 Department of Psychiatry and Behavioral Science and M.I.N.D. Institute, UC Davis, Sacramento, CA, USA

## Abstract

Developmental changes in the temporal and spatial regulation of gene expression drive the emergence of normal mature brain function, while disruptions in these processes underlie many neurodevelopmental abnormalities. To solidify our foundational knowledge of such changes in a primate brain with an extended period of postnatal maturation like in human, we investigated the whole-genome transcriptional profiles of rhesus monkey brains from birth to adulthood. We found that gene expression dynamics are largest from birth through infancy, after which gene expression profiles transition to a relatively stable state by young adulthood. Biological pathway enrichment analysis revealed that genes more highly expressed at birth are associated with cell adhesion and neuron differentiation, while genes more highly expressed in juveniles and adults are associated with cell death. Neocortex showed significantly greater differential expression over time than subcortical structures, and this trend likely reflects the protracted postnatal development of the cortex. Using network analysis, we identified 27 co-expression modules containing genes with highly correlated expression patterns that are associated with specific brain regions, ages or both. In particular, one module with high expression in neonatal cortex and striatum that decreases during infancy and juvenile development was significantly enriched for autism spectrum disorder (ASD)-related genes. This network was enriched for genes associated with axon guidance and interneuron differentiation, consistent with a disruption in the formation of functional cortical circuitry in ASD.

## Introduction

Human and non-human primate brain development requires the complex coordination of genetic and environmental cues that start during early embryogenesis and continue throughout adulthood. After birth, there is a protracted period of axon myelination and circuit development: synapses are overproduced during infancy, pruned during juvenile development and show cortical layer specificity (1,2). Juvenile growth is also characterized by immense cognitive development and susceptibility to neuropsychiatric disease (3). Correlated with these processes are highly dynamic changes in gene expression in multiple human brain regions from early fetal life through adulthood (4,5). Many genes associated with neurodevelopmental disorders, including ASD, are co-expressed during human fetal brain development, affecting specific developmental pathways and brain circuits (6,7).

Animal model systems of brain development allow for controlled experimental designs that include a healthy, age-matched cohort of individuals raised in similar environments, which can mitigate some of the potential limitations of studying postmortem human brain, such as variation in agonal state and postmortem tissue artifacts that may reduce RNA integrity and alter gene expression (8,9). Although mice have provided insights into global and cortical laminar patterns of gene expression in the adult and developing brain (10–12), there are major aspects of these gene expression patterns that differ between mouse and human (13,14). These differences reflect differences in both neurons and glia, and many primate-specific features of cortical development: a protracted developmental period (15–17), specific molecular pathways (13,18), expansion of frontal lobe and other association areas (19,20), and increased corticocortical connectivity (21).

Non-human primates, including rhesus monkeys, provide a complementary approach to understanding human brain development as they are an anatomically well-characterized model system for primate cortical development (22–24). The frontal and temporal lobes, which are important for neuropsychiatric disorders, show significant expansion in rhesus monkeys relative to mice (25,26). Similarly, many behaviors and cognitive functions are shared between rhesus monkeys and humans (27,28), including tool use and aspects of social organization (reviewed in 29).

Recent work has characterized cortical gene expression patterns in adult rhesus monkey (30), but there has not been a study of brain gene expression changes during early postnatal development through young adulthood, a critical period for neural circuit formation and behavioral changes that may be especially relevant to neuropsychiatric disease (31,32). In this study, we measured genome-wide gene expression by microarray in rhesus monkey from five brain regions—visual and prefrontal cortex, hippocampus, amygdala and ventral striatum—at birth, infancy, childhood and young adulthood (0, 3, 12 and 48 months after birth). We identified regional and temporal expression patterns during postnatal development and identified specific patterns of co-expressed genes associated with ASD.

## Results

### Transcriptome dynamics across development and brain regions

To analyze the transcriptome across rhesus brain development, we performed microarray analysis on medial prefrontal cortex, primary visual cortex, hippocampus, amygdala and ventral striatum from newborn, infant, juvenile and young adult male monkeys (*T* = 0, 3, 12 and 48 postnatal months, *N* = 3 per timepoint). After subjecting the data to strict quality control assessments (see Materials and Methods), one outlier sample from *T* = 0 striatum was removed, leaving 59 samples for downstream analysis. Overall, 32 217 probes (mapping to 14 754 genes; see Supplementary Material, Table S1) were robustly expressed in at least one brain region or developmental stage.

**Figure 1.**
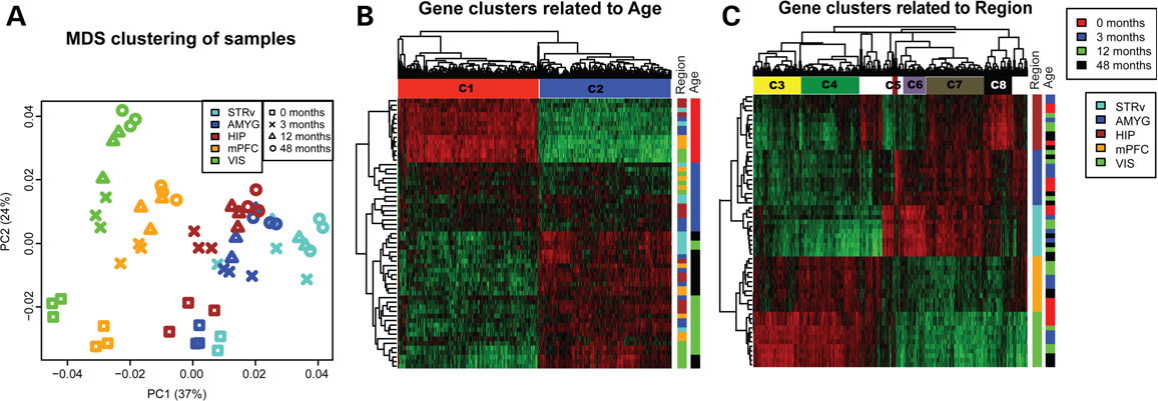
Gene expression is associated with both brain region and development stages. (**A**) MDS using all genes shows that samples cluster by both region (*x*-axis) and age (*y*-axis). Brain regions are illustrated by different shapes, while ages are labeled by different colors. The *x*- and *y*-axes represent the first and second PC, respectively, with the percent variance explained by each coordinate in parentheses. (**B** and **C**) Heatmap of top 1000 ANOVA genes for age (B) and region (C). Genes are hierarchically clustered along the *x*-axis, and gene clusters associated with different ages and regions are labeled by horizontal color bars. Samples are clustered along the *y*-axis and are color coded based both on region (left vertical bars) and age (right vertical bars).

To explore the relationship between gene expression and spatiotemporal dynamics in the rhesus monkey brain, we calculated the distance between brain samples based on their correlated expression patterns and performed classical multidimensional scaling (MDS) to represent these distances in two dimensions. We found that the first two principal coordinates (PCs) corresponded to brain region and age (Fig. 1A), and explained more than half of the transcriptional variation between brain samples (37 and 24%). To identify specific genes differentially expressed (DE) between brain regions or changing across postnatal development, we performed two one-way ANOVAs on all samples, using age and brain region as the two factors. We found that 10 338 of 32 217 (32%) probes were DE [false discovery rate (FDR) < 0.01] across developmental stages, and 13 527 (42%) probes were DE across brain regions. A total of 2967 probes (representing 2141 genes) were DE across both development and brain regions (Supplementary Material, Table S2) and were significantly enriched for several neurodevelopmental gene ontology (GO) categories including axon guidance (nominal *P* = 3.4 × 10^−7^), neuron projection (*P* = 1.8 × 10^−6^) and synapse (*P* = 2.0 × 10^−5^). Next, we searched for age-specific changes in expression and found 5935 DE probes (FDR < 0.05, >2 fold-change between pairs of ages for any region). The vast majority were DE at birth (3492 probes) or adulthood (1569) with many fewer at intermediate ages (149 at *T* = 3, 725 at *T* = 12; for a full list of age-related genes, see Supplementary Material, Table S2).

To further focus our analysis on the genes most significantly changing with age, we next considered only the top 1000 most DE probes (i.e. lowest *P*-value) identified through one-way ANOVA to find age-related clusters of both genes and samples. The most distinct transition occurred between 0 months and later development (Fig. 1B, rows), where virtually all DE genes show a complete reversal of expression between birth and later stages; those that are high at birth decrease later, and those that are low, increase. This dramatic neonatal shift in expression is consistent with what has been previously observed in human prefrontal cortex (5).

Genes clustered into two patterns with relatively equal proportions—those decreasing with age (cluster C1) and those increasing with age (C2)—with few genes peaking in infants or juveniles (Fig. 1B). Genes decreasing with age (C1) showed GO enrichment in cell adhesion (*P* = 2.9 × 10^−7^), extracellular matrix (*P* = 7.4 × 10^−5^) and neuron differentiation (*P* = 2.7 × 10^−3^), while genes increasing with age (C2) were associated with cell death (*P* = 1.4 × 10^−4^) (Table 1). This postnatal trend likely represents the tail end of a longer developmental trajectory that begins prenatally as described previously in human brain (4,5), including a transition from prenatal neurogenesis to postnatal cell death and synaptic pruning of redundant connections (33).

**Table 1.** Functional enrichment of differentially expressed genes

Cluster	Trait	DAVID GO	IPA canonical pathways	Top 10 DE genes
C1	Age 0	Cell adhesion (*P* = 2.9e−07) extracellular matrix part (*P* = 7.4e−05)	Agrin interactions as neuromuscular junction (*P* = 1.5e−03)	LPA, MYD88, DOK4, CIAPIN1, PARP6, NKAIN1, HAPLN1, COL4A1, APRT, ZDHHC2
		Neuron differentiation (*P* = 2.7e−03)	Semaphorin signaling in neurons (*P* = 2.6e−03)	
C2	Age 3–48	Cell death (*P* = 1.4e−04)	Sphingolipid metabolism (*P* = 1.3e−03)	*LRRC32, DNAJB6, IL6ST, CD74, PRUNE2, PPID, RICTOR, NPL2, DSTYK, CCDC28A*
		Apoptosis (*P* = 3.0e−04)	NRF2-mediated oxidative stress response (*P* = 3.7e−03)	
		Negative regulation of cell growth (*P* = 1.5e−03)	p53 signaling (*P* = 5.2e−03)	
C3	VIS	di-, tri-valent inorganic cation transport (*P* = 2.5e−04)	Calcium signaling (*P* = 2.4e−04)	*ESRRG, ATP2B2, KIAA0802, SEMA7A, CUX1, RORB, OSBPL1A, ZADH2, MAP3K13, EFNA5*
		Cell junction (*P* = 8.7e−03)	VDR/RXR activation (*P* = 5.9e−04)	
		Axon guidance (*P* = 9.8e−03)	Ephrin A signaling (*P* = 1.7e−03)	
C4	Cortex	Voltage-gated channel activity (*P* = 6.3e−05)	ErbB signaling (*P* = 7.4e−04)	*ARNTL, PCSK1, PLCH1, KIAA1107, VSNL1, FBLN7, SATB2, NCK2, RGS6, hCG_1776007*
		Regulation of synaptic plasticity (*P* = 5.0e−03)	Paxillin signaling (*P* = 1.4e−03)	
		Regulation of transmission of nerve impulse (*P* = 6.2e−03)	axonal guidance signaling (*P* = 2.6e−03)	
C5	AMYG	Membrane organization (*P* = 1.1e−02)	Role of Oct4 in mammalian embryonic stem cell pluripotency (*P* = 2.6e−02)	*LRMP, NR2F2, LOC644192, HS6ST2, OTOF, SLC27A6, PAPPA2, LIN28B, FIGN, LOC100132798*
		Membrane fusion (*P* = 2.3e−02)		
C6	STRv	Behavior (*P* = 8.0e−08)	G-Protein coupled receptor signaling	*NKX2-1, ZFHX3, DIRAS3, CHODL, PBX3, DRD2, ADCY5, RP11-327P2.4, PENK, DLX6*
		G-protein signaling, coupled to cAMP nucleotide second messenger (*P* = 7.1e−05)	Protein kinase A signaling (*P* = 8.1e−09)	
		Locomotory behavior (7.3e−05)	Dopamine-DARPP32 Feedback in cAMP signaling (*P* = 1.1e−04)	
C7	Non-cortical region	Neuron projection development (*P* = 1.0e−04)	Neuroprotective role of THOP1 in Alzheilmer’'s disease (*P* = 7.7e−05)	*BCL11B, CXorf57, SULF2, DACH2, TRIM36, ZCCHC12, RBP4, RGS14, NTS, LOC646576*
		Axonogenesis (*P* = 1.1e−04)	Glycosaminoglycan degradation (*P* = 1.1e−02)	
		Cell morphogenesis involved in neuron differentiation (*P* = 1.8e−04)		
C8	HIP	Actin binding (*P* = 2.4e−02)	Cdc42 signaling (*P* = 5.0e−03)	*CABP7, TSPAN18, TDRD12, CIB2, VWA5A, ARHGEF6, LASS3, C14orf132, ZBTB20, NELL1*
		Regulation of actin cytoskeleton (*P* = 4.2e−02)	Paxillin signaling (*P* = 2.0e−02)	

We took a similar strategy to search for region-specific changes in expression using the top 1000 most DE probes. Samples from each of the five assayed brain regions could be perfectly hierarchically clustered on the basis of gene expression. The two neocortical regions were most similar to one another and clearly distinct from non-neocortical regions (Fig. 1C, rows). Each region had a distinct gene expression signature (clusters C3–C8) and enriched GO and pathway analysis terms that reflected region-specific functions (Table 1). For example, genes DE in neocortex (C4) were enriched for voltage-gated channel activity (*P* = 6 × 10^−5^), while genes enriched in the non-cortical cluster C7 showed GO enrichment for neuron projection development (*P* = 1 × 10^−4^). The Ephrin A signaling pathway was statistically over-represented in the visual cortex enriched cluster C3 (*P* = 1.7 × 10^−3^) consistent with deficits in the functional mapping of primary visual cortex reported in mice lacking Ephrin-A2, -A3 and -A5 (34).

### Regional specificity of expression patterns across development

**Figure 2.**
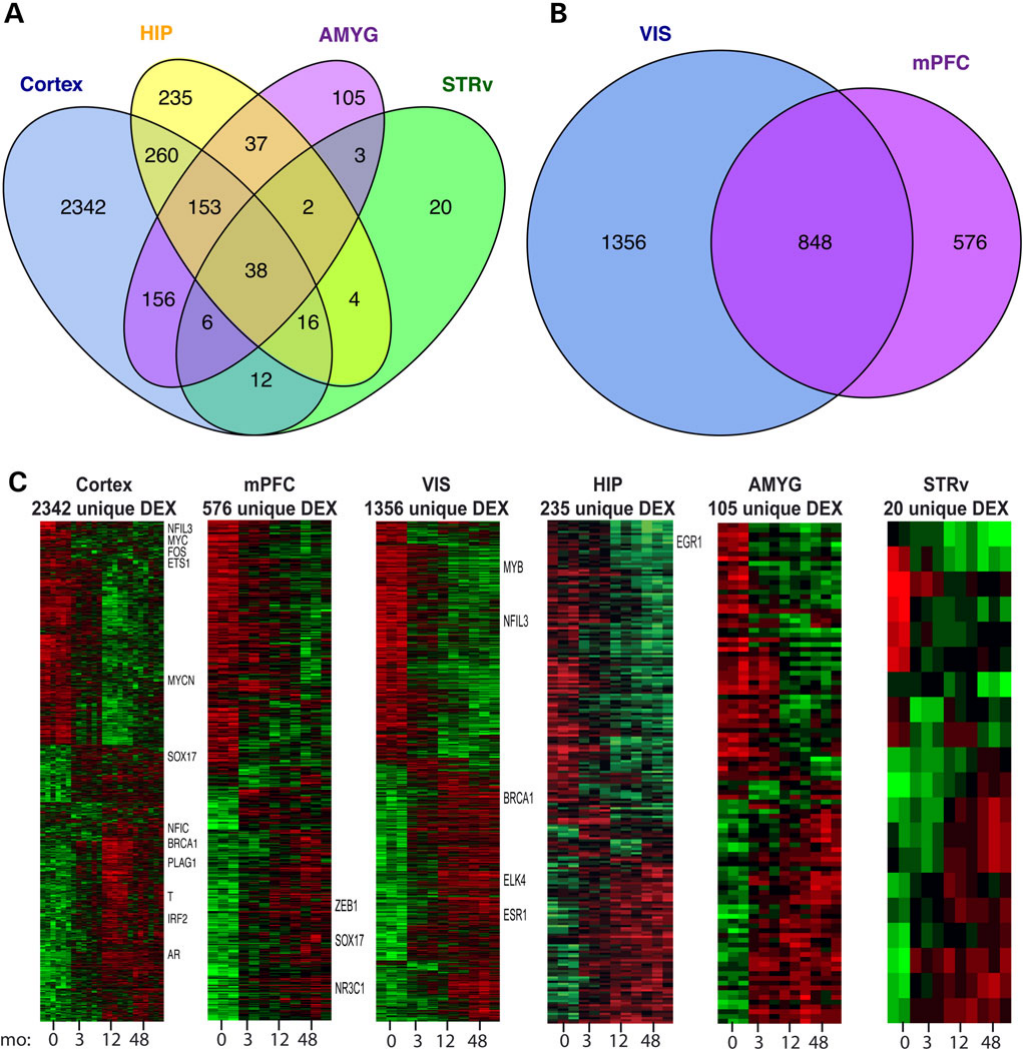
Developmental transcriptional profiles common to and unique between brain regions. (**A**) Venn-diagram shows the overlap of age-associated genes identified in neocortex and the three non-neocortical areas. (**B**) Overlap between genes DE in visual cortex and medial prefrontal cortex. (**C**) Heatmaps show age-associated genes unique to each brain region. Samples are in columns and ordered by age. Genes are presented on the rows, sorted such that genes decreasing with age are on top and genes increasing with age are at the bottom. Gene symbols for selected transcription factors are displayed in the right margins. Red indicates high expression and green indicates low expression.

We next sought to understand how gene expression dynamics vary between different brain regions. We conducted five one-way ANOVAs—one for each region—to identify genes DE with age (FDR < 0.05, fold-change >2 in any pair of four ages; see Materials and Methods and Supplemental Material, Table S3). The largest number of DE probes was found in neocortex (2983 probes combining visual and medial prefrontal areas) and hippocampus (745). In contrast, 500 probes were DE in amygdala and only 101 probes in ventral striatum, perhaps reflecting relatively earlier prenatal genesis of these regions. Surprisingly, ∼80% of DE probes were specific to a region, while only 20% were shared between any pair of regions (Fig. 2A). For example, 2342 probes were changing with age selectively in neocortex and GO enrichment analysis suggested significant involvement of cell adhesion (*P* = 1.8 × 10^−11^) and calcium ion binding (*P* = 5.5 × 10^−9^) (see Supplementary Material, Table S3 for a full list of GO enrichment terms).

The different cortical areas had unique temporal profiles (Fig. 2B), with 1356 probes uniquely DE in visual cortex and 576 in medial prefrontal cortex with 848 shared probes. However, the magnitude of differential gene expression was not constant over time, but rather increased over postnatal development so that twice as many probes were DE at 48 months (721, FDR *q* < 0.05) compared with 0 months (362 genes). Genes changing with age in visual cortex were enriched for neuron projection (*P* = 1.4 × 10^−6^), while genes changing with age in medial prefrontal cortex were enriched for myelin sheath (*P* = 1.7 × 10^−4^). Since these processes undoubtedly occur in both regions, it is likely this difference reflects a difference in timing of early developmental events between rostral and caudal cortex (24).

Next, we focused on the 38 probes that showed consistent changes with age in all regions (Fig. 2A). This shared list included five extracellular matrix genes (*HAPLN1*, *GPC2*, *TNC*, *EMID2* and *PRELP*). *HAPLN1* was reported to trigger the formation of perineuronal nets (35), which are extracellular matrix structures that surround many neurons, may regulate experience-dependent cortical plasticity, and increase in number during postnatal development (36). The transcription factor *SOX11*, which is highly expressed in proliferating and differentiating neural progenitor cells in mouse (37), decreased across all structures during postnatal development. This down-regulation likely reflects the maturation of neurons and decreased numbers of progenitors throughout postnatal neurodevelopment, as we previously described in the neurogenic hippocampal dentate subgranular zone (15).

Although different brain regions had largely different sets of genes changing over time, the temporal profile for each region was remarkably similar (Fig. 2C). Although these trends of high early/low late or vice versa were shown earlier for genes common to all regions (see Fig. 1B), the same trends were seen for those genes uniquely age-regulated in each region. Interestingly, a number of transcription factors were region-specific at early or late stages, potentially regulating maturation of different cell types in those regions.

### Co-expression networks define brain regions and developmental stages

**Figure 3.**
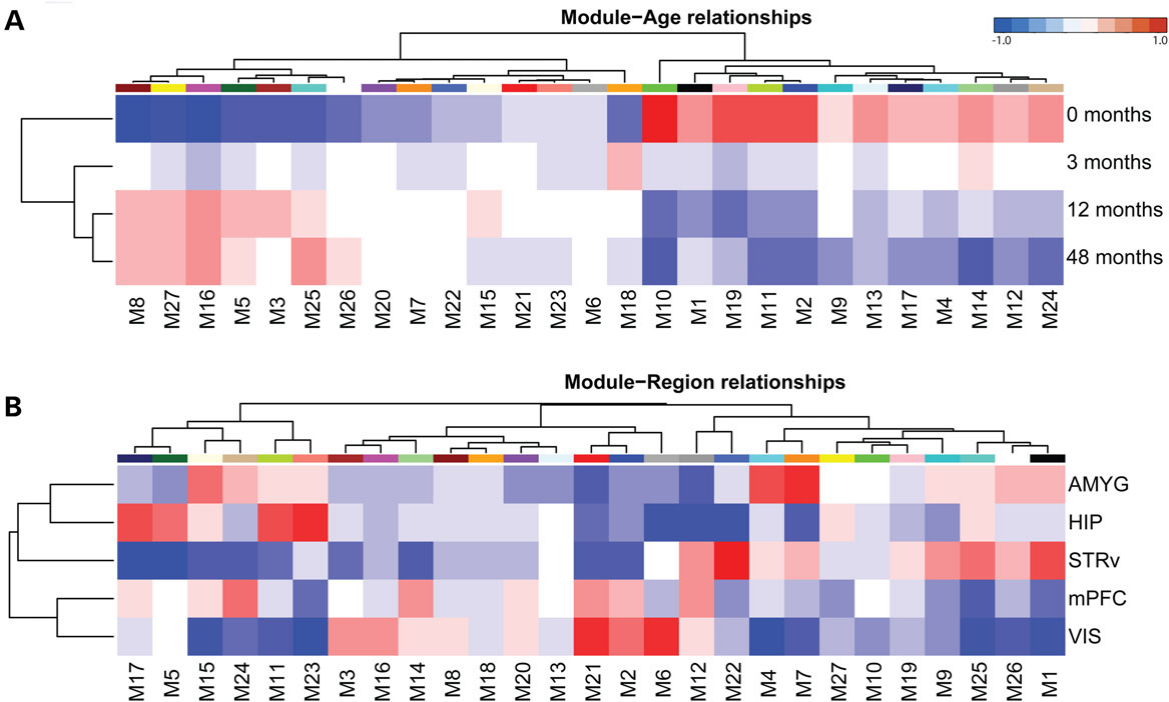
WGCNA identified modules predominately associated with region and age. Heatmap plot shows the correlation between MEs and age (**A**) or region (**B**). Modules were hierarchically clustered and sorted along the *x*-axis based on their eigengene–trait relationships. Traits were likewise sorted along the *y*-axis. Red indicates positive correlation, while blue shows negative correlation.

To identify transcriptional mechanisms in an unbiased manner, we applied weighted gene co-expression network analysis (WGCNA) on the 20 000 most variable probes to identify groups (or ‘modules’) of genes with similar patterns of expression across samples (38) (see Supplementary Material, Table S4 for module assignments and Supplementary Material, Fig. S1). We identified 27 modules that spanned a wide range of spatiotemporal patterns and each was represented using the module eigengene (ME) (the first principle component of each module). To relate these modules to developmental stage or brain region, we correlated each ME against age and region (see Materials and Methods) and hierarchically clustered modules to highlight shared temporal (Fig. 3A) and regional (Fig. 3B) profiles. Consistent with our differential expression analyses, samples from 0 months were most distinct from other ages, with a gross division between modules enriched at early versus late ages (Fig. 3A). Also consistent with our DE analysis, the two neocortical regions clustered closely together and distinctly from the other three brain regions (Fig. 3B). However, the association of modules with regions was complex, and individual modules showed enriched expression in distinct combinations of regions.

**Figure 4.**
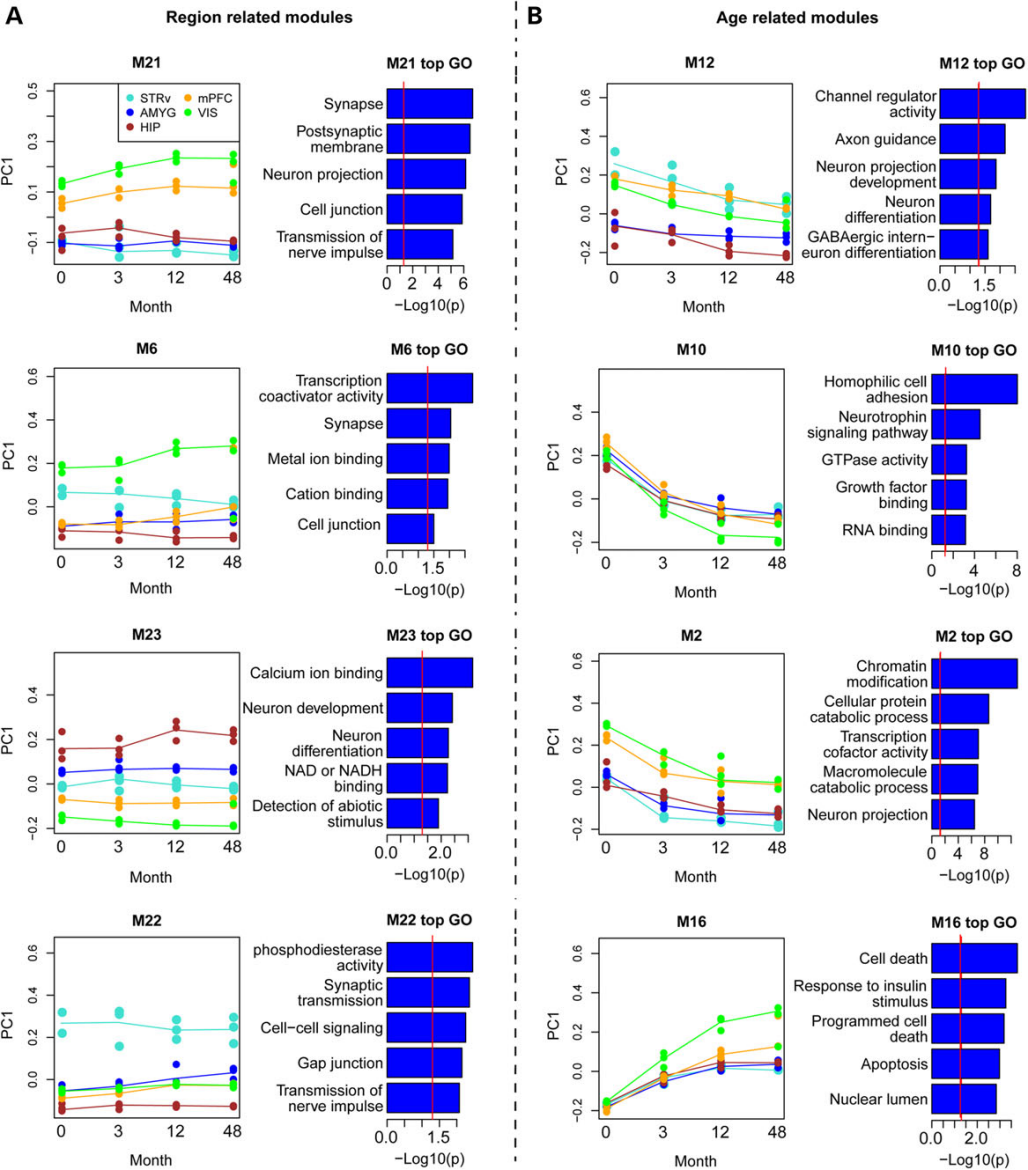
Expression patterns of modules associated with ages and regions. Line plot for four modules related to region (**A**) and four related to age (**B**). The left plots show the developmental profiles of each module, plotted separately for each brain region (using different colors). *X*-axes show the developmental stages, while *y*-axes show the value of eigengene (PC1) for each module. Adjacent plots show the top 5 GO pathways associated with each module, with some redundant categories filtered. Bars show the significance of each category. Vertical red lines indicate nominal *P* = 0.05.

We identified individual modules that showed strong associations with age and brain region, and were associated with specific developmental processes or cellular functions (Fig. 4). In many cases, the regional patterning was already established at birth and persisted through development. For example, module M21 was highly expressed in neocortical regions, and showed a slight increase in expression with time (Fig. 4A). The dense neuronal make-up of the cortex was reflected in GO enrichment of genes in this module including synapse, neuron projection and other neuron-associated GO categories. Genes associated with M21 were significantly enriched for transcription factors (17 genes, *P* = 0.008). ‘Hub’ genes of a module are often highly biologically relevant, either by playing a functional role (39,40) or by marking a cell type or cellular process with high specificity (41). Hub genes for this module included *CUX2* and *SATB2*, two key transcription factors for the proper specification of upper cortical layer neurons (42).

We also identified evidence for cortical area-specific expression, namely a module enriched selectively in primary visual cortex (M6). *SYT6*, which is selectively enriched in Layer 6 of primary visual cortex (30), was one of the hub genes in module M6. Gene expression in primary visual cortex has been shown to be highly distinct from other cortical regions in rhesus monkey (30) and adult human brain (43), reflecting the cytoarchitectural specialization of this region in primates. To determine if these genes were associated with cortical areal identity or more broadly associated with visual processing, we surveyed the expression of module M6 genes across the primate visual system using another rhesus dataset containing multiple cortical regions and visual thalamic regions (30). We confirmed high expression in primary visual cortex, even compared with its neighboring target region V2, and also found high expression in the dorsal lateral geniculate nucleus (Supplementary Material, Fig. S2). This suggested a role for this gene network in thalamocortical, but not higher order corticocortical, visual pathways.

We identified several groups of genes enriched in subcortical structures and that have conserved patterns between rhesus monkey and human. For example, module M22 showed particular enrichment in striatum. Twelve of the top 15 hub genes in this module show similar striatal enrichment in human brain (43), including, as expected, genes associated with dopaminergic signaling (*ARPP-21* and *PPP1R1B*) and GABAergic (*GAD2*/*GAD65* and *SLC31A1*/*VGAT*) neurons. Similarly, M23 showed enriched expression in hippocampus throughout postnatal development. The top two hub genes (*FZD7* and *CNIH2*) are also enriched in human hippocampus (43) and *CNIH2* has been shown to functionally interact with hippocampal AMPA receptor complexes, modulating receptor gating and trafficking (44,45). Together these results suggest that at least some elements of regional molecular specification are established by birth and conserved between primates.

In addition, we identified a number of modules that were primarily age-related and illustrate the gradual shift in gene expression patterning from birth to adulthood (Fig. 4B) as opposed to sharp transitions between developmental epochs. For example, modules M12, M10 and M2 all showed decreasing expression with age in all structures, although with subtle variations in regional enrichments. GO enrichments for these modules reflect the down-regulation of pathways associated with early developmental events such as neuronal differentiation and axonal path-finding. Interestingly, M2 was enriched for terms associated with chromatin modification, suggesting a developmental alteration of epigenetic regulation. Module M10 appeared to represent the core set of developmentally regulated genes identified earlier through differential expression analysis across regions. Genes in module M10 showed very similar temporal down-regulation across brain regions and included several canonical markers of early neural progenitors including *DCX*, *SOX4* and *SOX11* mentioned earlier, and showed GO enrichment for cell adhesion and neurotrophin signaling.

Finally, we identified several modules that showed increasing expression with age. For example, M16 showed progressive enrichment in all structures but particularly in primary visual cortex (Fig. 4B). The primary GO enrichments relate to programed cell death, although why developmental cell death is maintained into adulthood and why it is particularly enriched in visual cortex is unclear and may reflect an important difference between primates and rodents. For example, cell death in rat visual cortex peaks a week after birth, and there is minimal death in adulthood (46). We also see evidence for genes associated with gliogenesis increasing across postnatal development. Module M25 increases with age in subcortical structures (see Fig. 3) and contained markers for astrocytes (*AQP4*, *GFAP*), oligodendrocytes (*MAG*, *MOG*) and microglia (*TYROBP*, *LY86*). Finally, we identified a small module (M26) with similar expression as M25 that contained a disproportionately large proportion of genes (10/17 genes, *P* = 0.00023) essential for embryonic development and in which mutations cause disease phenotypes in humans and/or mouse (47,48). In particular, this module included several genes associated with Alzheimer’'s disease—including *APOE*, *CLU* and two genes in the S100-mediated signal transduction family (*S100A1*, *S100A13*) (49).

### ASD candidate genes are enriched in early postnatal cortex and striatum

Recent studies have mapped enrichment of genes associated with ASD and intellectual disability (ID) onto particular spatiotemporal patterns of gene expression and cell types (6,7). These studies demonstrated that prenatal neocortex was a common locus of action for pathways involving ASD genes, while ID genes showed non-specific developmental patterns (6). In this study, we extended these analyses to test for specific gene co-expression patterns associated with three neurodevelopmental disorders—ASD, ID and schizophrenia (SCZ)—in rhesus monkey. Putative disease gene lists were derived from candidate gene studies (Simons Foundation Autism Research Initiative (SFARI) Base (50)), a large-scale genome-wide association study of SCZ that identified 108 significant loci (51), and rare *de novo* likely gene-disrupting (LGD) variants enriched in ASD, ID and SCZ (gene lists from 52). An additional gene list was taken from a study of postmortem human brains that identified a module (asdM12) of co-expressed genes that were down-regulated in the frontotemporal neocortex of ASD compared with age-matched controls (53). Finally, genes with silent coding variants and LGD variants in unaffected siblings of individuals with ASD were not expected to show developmental expression patterns and were included as negative controls. Gene lists (Supplementary Material, Table S5) were tested for enrichment in 23 of 27 WGCNA co-expression modules identified earlier (Fig. 3) that included at least 20 genes.

We found significant enrichment (FDR < 0.05; see Supplementary Material, Table S4) of SFARI ASD genes in three modules (M2, M12 and M21) with specific spatiotemporal patterning. All three modules were strongly enriched in neocortex, while M12 was additionally enriched in striatum. Genes in M21 increased expression after birth and were associated with synaptic transmission (Fig. 4A), while M2 and M12 decreased expression and were associated with chromatin modification and axon guidance, respectively (Fig. 4B). Genes down-regulated in ASD frontotemporal cortex were also significantly enriched in modules M2 and M21, indicating a disruption in ASD of genes that are both up-regulated (M21) and down-regulated (M2) over postnatal development. In contrast, genes associated with SCZ GWAS loci were significantly enriched only in M21 that increases into adulthood. Finally, there were no specific spatiotemporal patterns enriched in postnatal development for genes with rare *de novo* LGD variants in ASD, ID or SCZ. This is consistent with previous observations that ASD LGD genes only show enrichment in transcriptional regulatory pathways early in fetal development (6).

To determine if these gene modules (M2, M12 and M21) corresponded to the SFARI-associated modules identified by Parikshak *et al*. (pM13, pM16 and pM17), we used a hypergeometric test to test for significant overlap of genes. All three modules from Parikshak *et al*. increased expression through prenatal development were associated with synaptic transmission and, as expected, significantly overlapped (*P* < 0.0001) with M21. Somewhat surprisingly, two of these three modules significantly overlapped (*P* < 0.0001) with M2, which shows decreasing postnatal expression, and genes in common between these modules were also enriched for synaptic transmission. This gene set likely represents early-peaking genes associated with synaptogenesis that peak prenatally and decrease postnatally, whereas M21 represents later peaking synapse-related genes.

**Figure 5.**
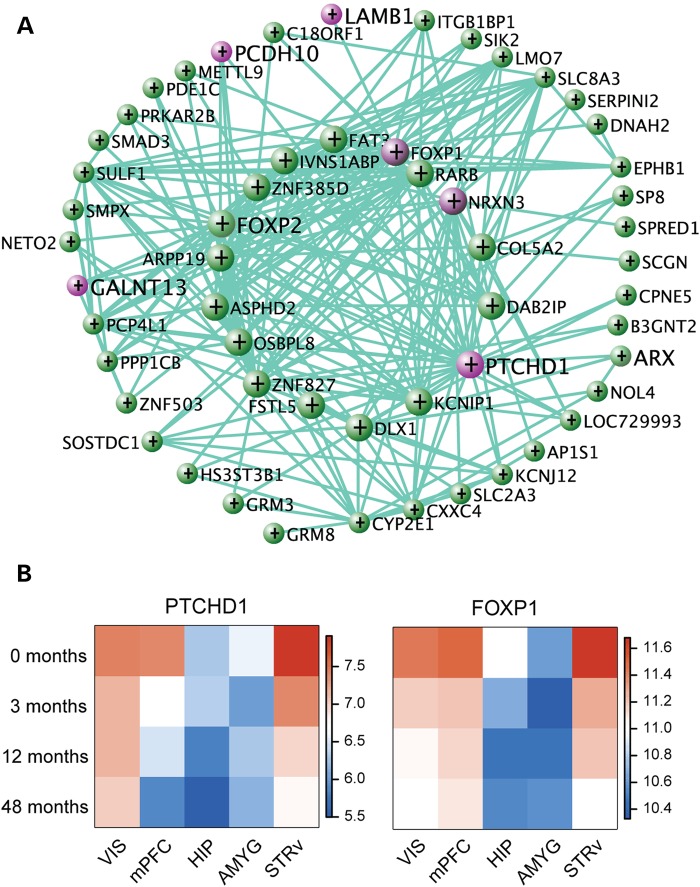
Module M12 is associated with ASD candidate genes. (**A**) VisANT plot showing the top 200 connections in M12. The hub genes, which have more connections, are displayed in the inner circle. Purple nodes indicate genes identified as ASD candidate genes from SFARI Base, with the remaining nodes shown in green. (**B**) Heatmap of the expression patterns for two hub genes in module M12. Cell colors show the average expression of samples from the specified age (*y*-axis) and region (*x*-axis).

Intriguingly, module M12 did not show even nominally significant overlap with ASD modules from Parikshak *et al*., suggesting a novel role for striatum in the neuropathology of ASD. To understand the co-expression relationships of the genes within M12, we created a network diagram based on the top 200 connections (based on co-expression) between pairs of genes (Fig. 5A). This network included six genes from SFARI (*PTCHD1*, *FOXP1*, *NRXN3*, *LAMB1*, *PCDH10* and *GALNT13*), two of which are hub genes. Figure 5B shows decreasing expression with age in neocortex and striatum for the hub genes *PTCHD1*, a receptor for sonic hedgehog, and *FOXP1*, a forkhead box transcription factor (Fig. 5B). Rare mutations in *FOXP1* have been linked to ASD and other disorders associated with ID and mental retardation (54). Together this demonstrates that a number of genes associated with ASD are coordinately expressed across postnatal development, both in the neocortex and the striatum.

## Discussion

Here, we describe genome-wide transcriptional profiling in rhesus monkey brain that spans four developmental periods (neonatal, infant, juvenile and young adult) in five brain regions (medial frontal cortex, visual cortex, hippocampus, amygdala and ventral striatum). We find similar spatiotemporal gene expression patterns in rhesus compared with recent studies in human neocortex (4,5,55). As in the human studies, we find that the most striking transcriptional changes occur early in postnatal development. Neonatal brain has a distinct gene expression signature from later ages, and the two most common temporal expression patterns are a dramatic increase or decrease in expression after birth (Figs 1–4). Since the rate of gene expression changes in developing human brain is highest prenatally and decreases rapidly during early postnatal development (5), the early postnatal dynamics we observe likely reflect the latter phase of a transition from a prenatal program of neurogenesis and gliogenesis to one of circuit formation and maturation. From the perspective of cognitive function, this period marks a transition from mostly reflex subcortical processing of sensory stimuli, to more conscious cortical processing that occurs in the first several months postnatally in humans (56,57). The pattern of gene expression shifts between birth and other postnatal periods is consistent with the neonatal pattern of cortical activation, which involves incomplete networks and primarily sensory cortex activation, in contrast to the emergence of more mature cortical networks that more clearly engage heteromodal association areas (58).

Remarkably, we did not observe significant spikes in gene expression for infant and juvenile phases, but rather a smooth transition from the neonate to the adult. Furthermore, despite the large phenomenological changes that occur between the juvenile (12 months) to the post-pubertal young adult (48 months), the gene expression signatures were nearly adult-like by 12 months. These findings are consistent with those observed in human brain development (4,5), indicating that rhesus monkey serves as a useful model system to learn about how genes shape primate brain development over extended developmental periods through expression of specific spatiotemporal patterns.

Interestingly, subcortical regions that have a more ancient evolutionary origin and earlier age of peak neurogenesis in rhesus macaque (17) had fewer genes changing expression after birth (Fig. 2). Although these regions such as thalamus continue to increase in volume after birth, this is due at least in part to increased myelination (59), and therefore it is less surprising that this volume change is not associated with altered gene expression. In contrast, neocortex develops later, expands dramatically after birth and shows a large increase in synaptic density. Functional imaging studies reveal similar trajectories of cortical networks (58). We find that these large anatomical changes in neocortex are reflected in thousands of DE genes that are highly significantly enriched for synapse-related categories such as cell adhesion and calcium ion binding. Moreover, we find that genes DE in primary visual cortex compared with prefrontal cortex are enriched for neuron projection that may reflect the specificity of visual pathway connections from the dorsal lateral geniculate nucleus to layer 4. Genes DE in prefrontal cortex were enriched for myelin sheath, potentially reflecting the protracted myelination of white matter in frontal compared with occipital cortex during primate postnatal development (33).

In this study, we leveraged the developmental time series of gene expression in primate brain to investigate the expression of genes associated with neurodevelopmental disorders including ASD, ID and SCZ. Recent evidence from studies of postmortem human brain (6,7,53,60) and normal human neuronal progenitors (61,62) suggests that many ASD susceptibility genes are co-expressed in the brain and represent convergent molecular pathways (63). These genes are most significantly co-expressed in cortical projection neurons in cortex during mid-fetal development (6,7,60), and are co-expressed with markers for neuron projection and other neuron-associated gene categories, which tend to show decreased transcription in autistic individuals (53). Here, we show that ASD susceptibility genes are enriched in gene co-expression networks during postnatal development, with distinct patterns both increasing and decreasing with age from birth to adulthood. In addition to the neocortex, we find one of these gene networks is highly enriched in the developing striatum, potentially contributing to the reported disrupted growth rate (64) and functional connectivity (65) of striatum in children with autism and to abnormal cortico-striatal circuits in mouse models of ASD (66,67). SCZ-associated genes showed overlap with ASD-associated genes in neocortical enriched, postnatally increasing gene modules, but not with postnatally decreasing or striatal enrichment. Consistent with prior observations (6), there was no enrichment in postnatal coexpression networks for rare variants associated with ASD. Overall, these results support the hypothesis that ASD-related genes may be part of the same developmental pathway (6,7,53), which is distinct from other developmental disorders and can be studied in a non-human primate model system. However, these results are correlative in nature and suggest complex regulatory mechanisms, as this co-expressed gene module contains genes expressed both by excitatory projection neurons (e.g. *FOXP2*) as well as inhibitory neurons (*ARX*, *DLX1*). More work is needed to understand mechanisms of trans-regulation among different cell types during cortical development and how they are impacted in ASD.

These data represent the first look at gene expression in the developing rhesus brain at a coarse level, and serve as a foundation for assessing the utility of rhesus monkey as a model system for studying human neurodevelopment and dysfunction. In the future, these data can be enhanced by including prenatal time points when it is expected that even more dynamic gene expression changes are occurring (4,5) and with RNA sequencing to characterize expression of non-coding transcripts. In addition, inclusion of female rhesus monkeys may reveal further insights into sex-related expression differences that have been observed for a small set of genes in developing postnatal human (4) and in adult rhesus (30), particularly in combination with differential splicing information. Finally, brain structures can be sampled at a finer anatomical resolution to examine the gene expression differences between cortical layers and, ultimately, between individual cells. These improvements will allow for a more detailed comparison between human and non-human primate species, and will increase our understanding of the role of cell types in the development of healthy and diseased brains.

## Materials and Methods

### Animals

Frozen postmortem tissue samples from 12 male rhesus macaque (*Macaca mulatta*) monkeys (*N* = 3 from *T* = 0, 3, 12 and 48 months) were provided by the Time-Mated Breeding Program at the California National Primate Research Center (CNPRC; http://www.cnprc.ucdavis.edu/). All monkeys were predominantly of Indian origin, including nine monkeys of less than one-sixteenth Chinese origin and three monkeys of no more than one-fourth Chinese origin. Rhesus monkeys were born and raised at the CNPRC in outdoor, half-acre enclosures that provide a naturalistic setting and normal social environment. Extensive health, family lineage and dominance information are maintained on all animals in the outdoor enclosures. All procedures were approved by the IACUC at UC Davis.

### Brain tissue collection

After dissection, brains were sectioned into coronal slabs ∼1–1.5 cm in thickness. Individual brain slabs were marked on the rostral surface with India ink (BD Biosciences) to identify rostral-caudal orientation of the slab. Rostral and caudal surfaces of all slabs were photographed digitally and then each slab was placed onto a metal disk that was embedded in dry ice. The mean (±SEM) time between euthanasia and freezing of tissue was 49 ± 2.1 min. Once frozen, sections were placed individually into bar-coded bags and then stored at −80°C. Structures of interest for microarray analysis were scalpel dissected from the right hemisphere slabs, and these samples were then frozen at −80°C until further processed. Structures were isolated with the greatest precision possible based on gross anatomical structure, with minimal white matter inclusion for cortical structures. These structures included ventral striatum, hippocampus, amygdala, primary visual cortex and medial prefrontal cortex.

### RNA collection and microarray generation

Tissue structures were homogenized in TRIZOL. The aqueous phase was removed and further processed for RNA isolation, using a modified version of Ambion's bead-based MagMAX-96 Total RNA Isolation kit, done on the MagMAX Express instrument. RNA samples were examined using a Bioanalyzer to assess RNA quality and concentration. Microarray data generation was performed by Covance Genomics Laboratory (now Laboratory Corporation of America Holdings (LabCorp)) in Seattle, WA using 50 ng total RNA starting material and Affymetrix GeneChip Rhesus Macaque Genome Arrays.

### Microarray data normalization and analysis

*SampleNetwork* function (68) was used for microarray data pre-processing. In this function, inter-array correlations (IACs) between each pair of samples (chips) were calculated using expression levels of all probe sets. Samples were hierarchically clustered using 1-IAC as a distance metric to visually identify outliers, and the average IACs value for each sample was calculated to quantitatively identify outliers (mean *R* < 0.95) (41). Samples whose IAC were 2SD away from other samples were defined as outlier samples. One outlier sample from *T* = 0 striatum was removed as failing both metrics, after which quantile normalization was performed on the remaining 59 samples in R. Log 2 transformation was applied to the data before downstream analysis.

### Probe set filtering and gene symbol assignments

Probe sets were initially filtered on the basis of gene expression using the present/marginal/absent calls reported on each chip. We selected probes with evidence of robust expression (75% of samples) at least in one region or one age. After filtering, 32 217 probe sets remained for different expression analysis. For WGCNA, which is more computationally intensive, we included the top 20 000 of these probe sets based on variance. We then mapped rhesus probe sets to human genes using the file ‘HG-U133 Plus 2.0 to Rhesus Best Match’ provided on Affymetrix website (http://www.affymetrix.com/Auth/support/downloads/comparisons/U133PlusVsRhesus_BestMatch.zip, last accessed on 24 July 2013). The probe information is included in Supplementary Material, Table S1.

### Differential expression analysis

ANOVA, multidimensional scaling and hierarchical clustering were performed using R. The gene expression distances between samples were calculated based on 1 – Pearson correlation, and classical MDS was applied with two components to visualize the relative distances. Two one-way ANOVA *F*-tests were performed with region or age as a factor, and probes passing a Benjamini–Hochberg corrected FDR < 0.01 were defined as region- or age-enriched. DE genes at specific ages were defined as genes with a relaxed FDR < 0.05 and fold difference >2 between any pair of ages in a region. The top 1000 DE genes from the age and region ANOVAs were hierarchically (agglomerative) clustered and visualized as heatmaps to identify sets of genes with similar age or region enrichment. A secondary analysis of region-specific expression dynamics included five one-way ANOVA tests performed separately for each brain region with age as the factor. GO enrichment analysis was performed using DAVID software (http://david.abcc.ncifcrf.gov/) (69).

### Weighted gene co-expression network analysis

To identify clusters of co-expressed genes, WGCNA was applied to all 59 samples following the standard procedure for generating a signed network (41,70). In short, pairwise Pearson correlation coefficients were calculated for the 20 000 most variable probes across all samples, and converted to connection strengths, defined as [(1 + correlation)/2]*^β^*, where *β* = 16 (70). These adjacency matrices were then used as the basis for defining topological overlap (TO), which measures the common connections between a pair of genes. For each network, we then used average linkage hierarchical clustering to group genes on the basis of TO dissimilarity measure (1 – TO). Finally, modules were defined using a dynamic tree-cutting algorithm (41). The ME, defined as the first principle component of a given module, was used to represent characteristic expression patterns of individual modules. Hub genes were defined as having a module membership value (kME) > 0.7, while non-hub genes had a kME < 0.3. VisAnt was used to visualize a sub-network of co-expressed genes (71).

### Neurodevelopmental disease gene spatiotemporal pattern enrichment analysis

To find the modules that are associated with ASD candidate genes, we compiled the SFARI ASD set (50) by using the online SFARI gene database (https://gene.sfari.org/autdb/Welcome.do, last accessed on 20 November 2014), AutDB. We used the ‘Gene Score,’ which classifies evidence levels, to restrict our set to those categorized as S (Syndromic) and evidence levels 1–4 (high confidence–minimal evidence). Two hundred and fifty genes were included when we downloaded the data on November 20, 2014. Three hundred and thirty-two genes located within 108 loci significant associated with SCZ were obtained from a recent large-scale GWAS (51). Rare *de novo* LGD and silent variants enriched in ASD (including unaffected siblings where available), ID and SCZ were significantly enriched in genes and were obtained from Ref. (52). Co-expressed genes that were down-regulated in the frontotemporal neocortex of ASD compared with age-matched controls were obtained from Ref. (53). See Supplementary Material, Table S5 for all gene lists. Hypergeometric enrichment analysis was performed for all combinations of gene lists and modules (for modules containing at least 20 unique genes), and *P*-values were corrected for multiple comparisons via the Benjamini–Hochberg method.

### Data availability

Detailed technical white papers describing tissue processing and microarray profiling are available at the Allen Brain Atlas portal (http://brain-map.org) through the Non-Human Primate link, or directly from the NIH Blueprint NHP Atlas website (http://www.blueprintnhpatlas.org/), under the ‘Documentation’ tab. Microarray data can be viewed online by selecting ‘Macrodissection’ under the ‘Microarray’ tab and can be downloaded under the ‘Download’ tab.

## Supplementary Material

Supplementary Material is available at *HMG* online.

## Funding

The National Institutes of Health Blueprint project described was supported by contract HHSN-271-2008-0047 from the National Institute of Mental Health. Its contents are solely the responsibility of the authors and do not necessarily represent the official views of the National Institutes of Health or the National Institute of Mental Health. Funding to pay the Open Access publication charges for this article was provided by the Allen Institute for Brain Science.

## Supplementary Material

Supplementary Data
