# Supplementary material for: Spatiotemporal dynamics of the postnatal developing primate brain transcriptome
Source: Hum Mol Genet. 2015 May 7;24(15):4327–39. doi: 10.1093/hmg/ddv166 (PMC4492396; doi:10.1093/hmg/ddv166)
Supplement: Supplementary Data [file supp_24_15_4327__index.html]

Spatiotemporal dynamics of the postnatal developing primate brain transcriptome — Spatiotemporal dynamics of the postnatal developing primate brain transcriptome — Spatiotemporal dynamics of the postnatal developing primate brain transcriptome — Supplementary Data 

# Spatiotemporal dynamics of the postnatal developing primate brain transcriptome

## Supplementary Data

Supplementary Data

- Supplementary Data - Doc file
- Supplementary Table 1 - pdf file
- Supplementary Table 2 - xlsx file
- Supplementary Table 3 - xlsx file
- Supplementary Table 4 - xlsx file
- Supplementary Table 5 - xlsx file
