# Supplementary material for: Spatiotemporal dynamics of the postnatal developing primate brain transcriptome
Source: Hum Mol Genet. 2015 May 7;24(15):4327–39. doi: 10.1093/hmg/ddv166 (PMC4492396; doi:10.1093/hmg/ddv166)
Supplement: Supplementary Data [file supp_ddv166_ddv166supp.doc]

**Supplementary Information**


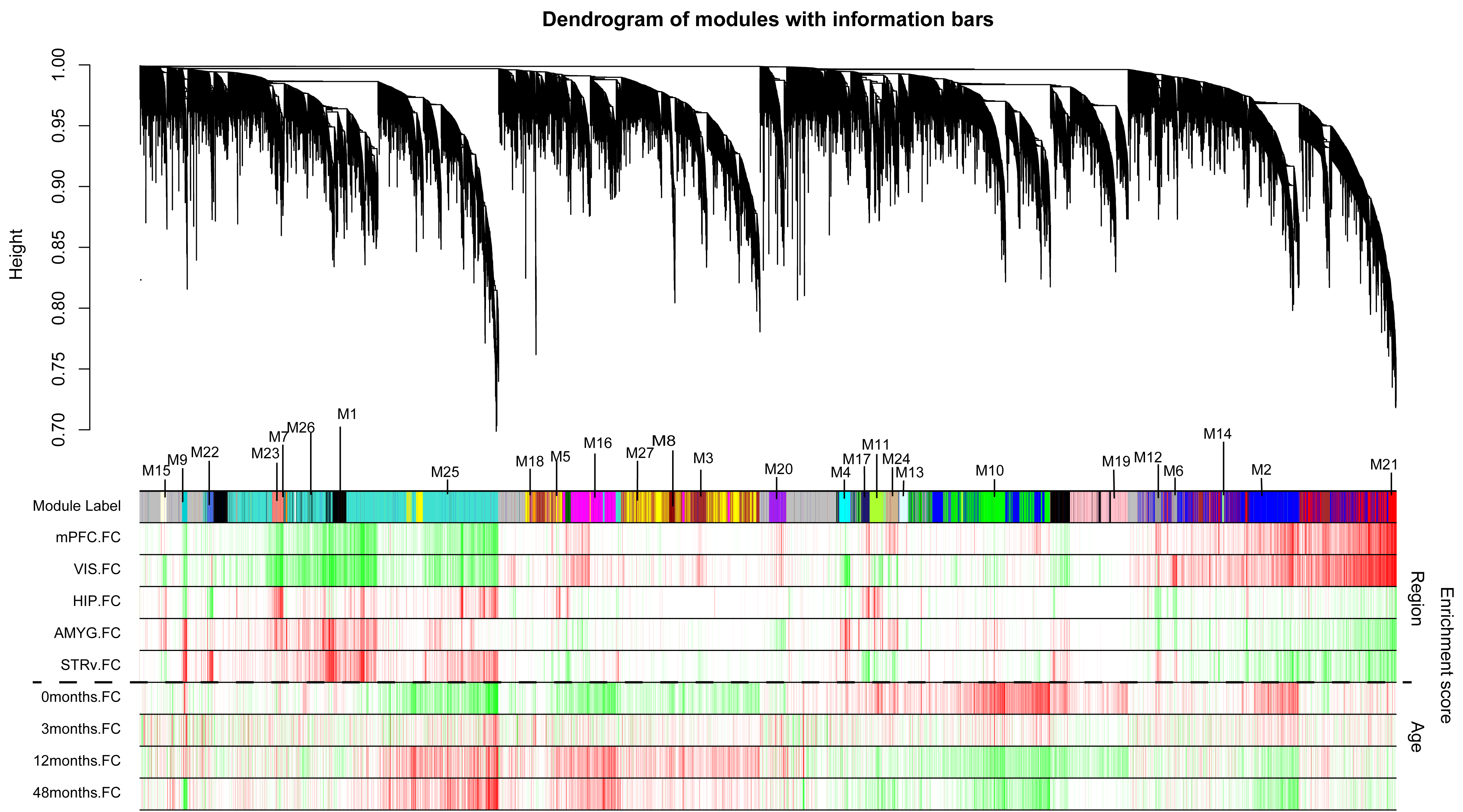


Figure S1. Dendrogram and module assignment of WGCNA network. Genes were hierarchically clustered and are presented in that order along the horizontal. The first color bar (and associated labels) indicates the module to which each gene is assigned. The next nine horizontal bars show the fold change of genes in each region (top five bars) or age (next four bars) versus remaining ages or regions. Red indicates positive fold change (enrichment), while green shows negative fold change.


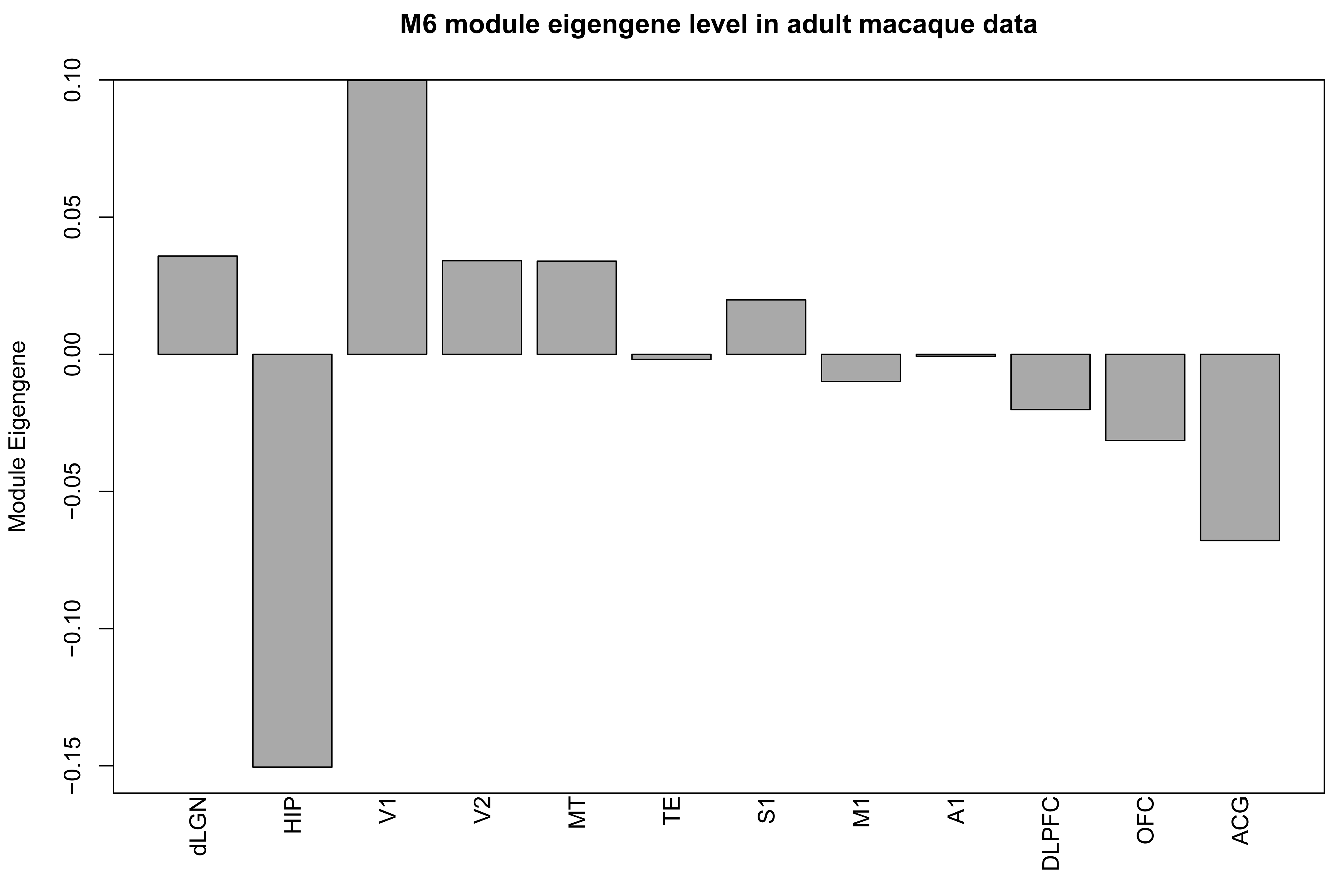


Figure S2. Bar plot showing relative expression of genes in module M6 across different cortical and sub-cortical brain regions in adult (~8 years old) rhesus macaque. dLGN – dorsal lateral geniculate nucleus; HIP – hippocampus; V1 – primary visual cortex; V2 – secondary visual cortex; MT – middle temporal area; TE – temporal area; S1 – primary somatosensory cortex; M1 – primary motor cortex; A1 – primary auditory cortex; DLPFC – dorsolateral prefrontal cortex; OFC – orbitofrontal cortex; ACG – anterior cingulate gyrus. See Bernard et al. 2012 for further details on brain regions sampled.

**Tables**

Table S1. Probe annotation file.

Table S2. Cluster summary of ANOVA analysis for region and age.

Table S3. Differentially expressed genes across developmental stages in each brain region, and GO pathways of specific DE genes across developmental stages in each brain region.

Table S4. WGCNA module information.

Table S5. Gene lists used for neurodevelopmental gene enrichment analysis.
