# Supplementary material for: Spatiotemporal dynamics of the postnatal developing primate brain transcriptome
Source: Hum Mol Genet. 2015 May 7;24(15):4327–39. doi: 10.1093/hmg/ddv166 (PMC4492396; doi:10.1093/hmg/ddv166)
Supplement: Supplementary Data [file supp_ddv166_ddv166supp_table1.pdf]

macaque.Probe	human.Probe	Gene.Symbol.Human	Gene.Title.Human	Chromosomal.Location.Human	Entrez.Gene.Human	Gene.Symbol	Gene.Title.mac	Chromosomal.Location.mac	Entrez.Gene.mac	name
Mmu.10002.1.S1_at	NA	NA	NA	NA	NA	NA	NA	NA	NA	Mmu.10002.1.S1_at_NA
Mmu.10002.1.S1_s_at	NA	NA	NA	NA	NA	NA	NA	NA	NA	Mmu.10002.1.S1_s_at_NA
Mmu.10014.1.S1_at	NA	NA	NA	NA	NA	NA	NA	NA	NA	Mmu.10014.1.S1_at_NA
Mmu.10025.1.S1_at	NA	NA	NA	NA	NA	NA	NA	NA	NA	Mmu.10025.1.S1_at_NA
Mmu.10030.1.S1_at	NA	NA	NA	NA	NA	NA	NA	NA	NA	Mmu.10030.1.S1_at_NA
Mmu.10039.1.S1_at	NA	NA	NA	NA	NA	NA	NA	NA	NA	Mmu.10039.1.S1_at_NA
Mmu.10045.2.S1_at	211656_x_at	HLA-DQB1	major histocompatibility complex, class II, DQ beta 1	chr6p21.3	3119	MAMU-DQB1	major histocompatibility complex, class II, DQ beta 1	---	716823	Mmu.10045.2.S1_at_HLA-DQB1
Mmu.10045.2.S1_x_at	NA	NA	NA	NA	NA	NA	NA	NA	NA	Mmu.10045.2.S1_x_at_NA
Mmu.10062.1.S1_s_at	202110_at	COX7B	cytochrome c oxidase subunit VIIb	chrXq21.1	1349	LOC706099	similar to cytochrome c oxidase subunit VIIb precursor	---	706099	Mmu.10062.1.S1_s_at_COX7B
Mmu.10083.1.A1_at	NA	NA	NA	NA	NA	NA	NA	NA	NA	Mmu.10083.1.A1_at_NA
Mmu.10083.1.S1_s_at	NA	NA	NA	NA	NA	NA	NA	NA	NA	Mmu.10083.1.S1_s_at_NA
Mmu.10088.2.S1_at	NA	NA	NA	NA	NA	NA	NA	NA	NA	Mmu.10088.2.S1_at_NA
Mmu.10114.1.S1_at	NA	NA	NA	NA	NA	NA	NA	NA	NA	Mmu.10114.1.S1_at_NA
Mmu.10116.1.S1_at	NA	NA	NA	NA	NA	NA	NA	NA	NA	Mmu.10116.1.S1_at_NA
Mmu.101.1.S1_at	NA	NA	NA	NA	NA	NA	NA	NA	NA	Mmu.101.1.S1_at_NA
Mmu.10122.1.S1_at	NA	NA	NA	NA	NA	NA	NA	NA	NA	Mmu.10122.1.S1_at_NA
Mmu.10126.1.S1_at	229856_s_at	C1orf128	Chromosome 1 open reading frame 128	chr1p36.11	57095	EIF4A2	Eukaryotic translation initiation factor 4A, isoform 2	---	707775	Mmu.10126.1.S1_at_C1orf128
Mmu.10134.1.S1_at	217868_s_at	METTL9	methyltransferase like 9	chr16p13-p12	51108	METTL9	methyltransferase like 9	---	698159	Mmu.10134.1.S1_at_METTL9
Mmu.10135.1.S1_at	204093_at	CCNH	cyclin H	chr5q13.3-q14	902	CCNH	cyclin H	---	694224	Mmu.10135.1.S1_at_CCNH
Mmu.10139.2.S1_at	NA	NA	NA	NA	NA	NA	NA	NA	NA	Mmu.10139.2.S1_at_NA
Mmu.10150.1.S1_at	NA	NA	NA	NA	NA	NA	NA	NA	NA	Mmu.10150.1.S1_at_NA
Mmu.1015.1.S1_s_at	221943_x_at	RPL38	Ribosomal protein L38	chr17q23-q25	6169	LOC710665	similar to 60S ribosomal protein L38	---	710665	Mmu.1015.1.S1_s_at_RPL38
Mmu.10158.1.S1_at	NA	NA	NA	NA	NA	NA	NA	NA	NA	Mmu.10158.1.S1_at_NA
Mmu.10176.1.S1_at	NA	NA	NA	NA	NA	NA	NA	NA	NA	Mmu.10176.1.S1_at_NA
Mmu.10185.1.S1_at	NA	NA	NA	NA	NA	NA	NA	NA	NA	Mmu.10185.1.S1_at_NA
Mmu.10185.1.S1_s_at	NA	NA	NA	NA	NA	NA	NA	NA	NA	Mmu.10185.1.S1_s_at_NA
Mmu.10186.1.S1_at	NA	NA	NA	NA	NA	NA	NA	NA	NA	Mmu.10186.1.S1_at_NA
Mmu.10187.1.S1_at	NA	NA	NA	NA	NA	NA	NA	NA	NA	Mmu.10187.1.S1_at_NA
Mmu.10195.2.S1_x_at	NA	NA	NA	NA	NA	NA	NA	NA	NA	Mmu.10195.2.S1_x_at_NA
Mmu.10197.1.S1_at	NA	NA	NA	NA	NA	NA	NA	NA	NA	Mmu.10197.1.S1_at_NA
Mmu.1020.1.S1_s_at	NA	NA	NA	NA	NA	NA	NA	NA	NA	Mmu.1020.1.S1_s_at_NA
Mmu.10202.1.S1_at	NA	NA	NA	NA	NA	NA	NA	NA	NA	Mmu.10202.1.S1_at_NA
Mmu.10209.1.S1_s_at	NA	NA	NA	NA	NA	NA	NA	NA	NA	Mmu.10209.1.S1_s_at_NA
Mmu.10209.2.S1_at	NA	NA	NA	NA	NA	NA	NA	NA	NA	Mmu.10209.2.S1_at_NA
Mmu.10212.1.S1_at	NA	NA	NA	NA	NA	NA	NA	NA	NA	Mmu.10212.1.S1_at_NA
Mmu.10214.1.S1_at	NA	NA	NA	NA	NA	NA	NA	NA	NA	Mmu.10214.1.S1_at_NA
Mmu.10221.1.S1_at	224689_at	MANBAL	mannosidase, beta A, lysosomal-like	chr20q11.23-q12	63905	LOC701167	similar to mannosidase, beta A, lysosomal-like	---	701167	Mmu.10221.1.S1_at_MANBAL
Mmu.10229.1.S1_at	NA	NA	NA	NA	NA	NA	NA	NA	NA	Mmu.10229.1.S1_at_NA
Mmu.10237.1.S1_at	NA	NA	NA	NA	NA	NA	NA	NA	NA	Mmu.10237.1.S1_at_NA
Mmu.10239.1.S1_at	NA	NA	NA	NA	NA	NA	NA	NA	NA	Mmu.10239.1.S1_at_NA
Mmu.10240.1.S1_at	NA	NA	NA	NA	NA	NA	NA	NA	NA	Mmu.10240.1.S1_at_NA
Mmu.10246.2.A1_x_at	NA	NA	NA	NA	NA	NA	NA	NA	NA	Mmu.10246.2.A1_x_at_NA
Mmu.10246.2.S1_x_at	NA	NA	NA	NA	NA	NA	NA	NA	NA	Mmu.10246.2.S1_x_at_NA
Mmu.10266.1.A1_at	NA	NA	NA	NA	NA	NA	NA	NA	NA	Mmu.10266.1.A1_at_NA
Mmu.1028.1.S1_at	NA	NA	NA	NA	NA	NA	NA	NA	NA	Mmu.1028.1.S1_at_NA
Mmu.10293.1.S1_s_at	NA	NA	NA	NA	NA	NA	NA	NA	NA	Mmu.10293.1.S1_s_at_NA
Mmu.10300.2.S1_at	NA	NA	NA	NA	NA	NA	NA	NA	NA	Mmu.10300.2.S1_at_NA
Mmu.10318.1.S1_at	NA	NA	NA	NA	NA	NA	NA	NA	NA	Mmu.10318.1.S1_at_NA
Mmu.10318.1.S1_s_at	NA	NA	NA	NA	NA	NA	NA	NA	NA	Mmu.10318.1.S1_s_at_NA
Mmu.1032.1.S1_at	NA	NA	NA	NA	NA	NA	NA	NA	NA	Mmu.1032.1.S1_at_NA
Mmu.10327.1.S1_at	NA	NA	NA	NA	NA	NA	NA	NA	NA	Mmu.10327.1.S1_at_NA
Mmu.10346.1.S1_at	NA	NA	NA	NA	NA	NA	NA	NA	NA	Mmu.10346.1.S1_at_NA
Mmu.1036.1.S1_at	NA	NA	NA	NA	NA	NA	NA	NA	NA	Mmu.1036.1.S1_at_NA
Mmu.10362.1.S1_at	NA	NA	NA	NA	NA	NA	NA	NA	NA	Mmu.10362.1.S1_at_NA
Mmu.10377.1.S1_x_at	NA	NA	NA	NA	NA	NA	NA	NA	NA	Mmu.10377.1.S1_x_at_NA
Mmu.10380.1.S1_at	NA	NA	NA	NA	NA	NA	NA	NA	NA	Mmu.10380.1.S1_at_NA
Mmu.10380.2.S1_s_at	NA	NA	NA	NA	NA	NA	NA	NA	NA	Mmu.10380.2.S1_s_at_NA
Mmu.10383.1.S1_at	NA	NA	NA	NA	NA	NA	NA	NA	NA	Mmu.10383.1.S1_at_NA
Mmu.10390.1.S1_at	NA	NA	NA	NA	NA	NA	NA	NA	NA	Mmu.10390.1.S1_at_NA
Mmu.10393.1.S1_x_at	210699_at	---	---	---	---	LOC702809	similar to eukaryotic translation elongation factor 1 alpha 2	---	702809	Mmu.10393.1.S1_x_at_---
Mmu.10420.1.S1_at	NA	NA	NA	NA	NA	NA	NA	NA	NA	Mmu.10420.1.S1_at_NA
Mmu.10433.1.S1_at	NA	NA	NA	NA	NA	NA	NA	NA	NA	Mmu.10433.1.S1_at_NA
Mmu.10436.1.S1_at	NA	NA	NA	NA	NA	NA	NA	NA	NA	Mmu.10436.1.S1_at_NA
Mmu.10443.1.S1_at	NA	NA	NA	NA	NA	NA	NA	NA	NA	Mmu.10443.1.S1_at_NA
Mmu.10451.1.S1_at	NA	NA	NA	NA	NA	NA	NA	NA	NA	Mmu.10451.1.S1_at_NA
Mmu.10452.1.A1_at	NA	NA	NA	NA	NA	NA	NA	NA	NA	Mmu.10452.1.A1_at_NA
Mmu.10452.1.S1_at	NA	NA	NA	NA	NA	NA	NA	NA	NA	Mmu.10452.1.S1_at_NA
Mmu.10472.1.S1_at	NA	NA	NA	NA	NA	NA	NA	NA	NA	Mmu.10472.1.S1_at_NA
Mmu.10481.1.S1_at	NA	NA	NA	NA	NA	NA	NA	NA	NA	Mmu.10481.1.S1_at_NA
Mmu.10483.1.S1_x_at	NA	NA	NA	NA	NA	NA	NA	NA	NA	Mmu.10483.1.S1_x_at_NA
Mmu.10484.1.A1_at	NA	NA	NA	NA	NA	NA	NA	NA	NA	Mmu.10484.1.A1_at_NA
Mmu.10484.1.S1_at	NA	NA	NA	NA	NA	NA	NA	NA	NA	Mmu.10484.1.S1_at_NA
Mmu.10484.2.A1_at	217390_x_at	---	---	---	---	---	---	---	---	Mmu.10484.2.A1_at_---
Mmu.10484.2.S1_x_at	NA	NA	NA	NA	NA	NA	NA	NA	NA	Mmu.10484.2.S1_x_at_NA
Mmu.10488.1.S1_at	NA	NA	NA	NA	NA	NA	NA	NA	NA	Mmu.10488.1.S1_at_NA
Mmu.10506.1.S1_at	NA	NA	NA	NA	NA	NA	NA	NA	NA	Mmu.10506.1.S1_at_NA
Mmu.10506.1.S1_s_at	NA	NA	NA	NA	NA	NA	NA	NA	NA	Mmu.10506.1.S1_s_at_NA
Mmu.10507.1.S1_at	214150_x_at	ATP6V0E1	ATPase, H+ transporting, lysosomal 9kDa, V0 subunit e1	chr5q35.2	8992	ATP6V0E1	ATPase, H+ transporting, lysosomal 9kDa, V0 subunit e1	---	703342	Mmu.10507.1.S1_at_ATP6V0E1
Mmu.1051.1.S1_at	NA	NA	NA	NA	NA	NA	NA	NA	NA	Mmu.1051.1.S1_at_NA
Mmu.105.1.S1_at	NA	NA	NA	NA	NA	NA	NA	NA	NA	Mmu.105.1.S1_at_NA
Mmu.105.1.S1_s_at	NA	NA	NA	NA	NA	NA	NA	NA	NA	Mmu.105.1.S1_s_at_NA
Mmu.105.1.S1_x_at	NA	NA	NA	NA	NA	NA	NA	NA	NA	Mmu.105.1.S1_x_at_NA
Mmu.10533.2.S1_s_at	NA	NA	NA	NA	NA	NA	NA	NA	NA	Mmu.10533.2.S1_s_at_NA
Mmu.10543.1.S1_at	NA	NA	NA	NA	NA	NA	NA	NA	NA	Mmu.10543.1.S1_at_NA
Mmu.10543.1.S1_x_at	NA	NA	NA	NA	NA	NA	NA	NA	NA	Mmu.10543.1.S1_x_at_NA
Mmu.10544.1.S1_at	NA	NA	NA	NA	NA	NA	NA	NA	NA	Mmu.10544.1.S1_at_NA
Mmu.10545.1.S1_at	NA	NA	NA	NA	NA	NA	NA	NA	NA	Mmu.10545.1.S1_at_NA
Mmu.10556.1.S1_at	NA	NA	NA	NA	NA	NA	NA	NA	NA	Mmu.10556.1.S1_at_NA
Mmu.10556.1.S1_s_at	NA	NA	NA	NA	NA	NA	NA	NA	NA	Mmu.10556.1.S1_s_at_NA
Mmu.10570.1.S1_at	NA	NA	NA	NA	NA	NA	NA	NA	NA	Mmu.10570.1.S1_at_NA
Mmu.10576.1.S1_at	NA	NA	NA	NA	NA	NA	NA	NA	NA	Mmu.10576.1.S1_at_NA
Mmu.10576.1.S1_s_at	NA	NA	NA	NA	NA	NA	NA	NA	NA	Mmu.10576.1.S1_s_at_NA
Mmu.10581.1.S2_at	NA	NA	NA	NA	NA	NA	NA	NA	NA	Mmu.10581.1.S2_at_NA
Mmu.10602.2.S1_at	NA	NA	NA	NA	NA	NA	NA	NA	NA	Mmu.10602.2.S1_at_NA
Mmu.10608.1.S1_s_at	222083_at	GLYAT	glycine-N-acyltransferase	chr11q12.1	10249	GLYAT	glycine-N-acyltransferase	---	701659	Mmu.10608.1.S1_s_at_GLYAT
Mmu.10608.1.S1_x_at	NA	NA	NA	NA	NA	NA	NA	NA	NA	Mmu.10608.1.S1_x_at_NA
Mmu.10626.1.S1_at	NA	NA	NA	NA	NA	NA	NA	NA	NA	Mmu.10626.1.S1_at_NA
Mmu.10627.1.S1_at	230116_at	KRT8P12	Keratin 8 pseudogene 12	chr3q26.1	90133	KRT8	keratin 8	---	700449	Mmu.10627.1.S1_at_KRT8P12
Mmu.1063.2.S1_at	NA	NA	NA	NA	NA	NA	NA	NA	NA	Mmu.1063.2.S1_at_NA
Mmu.10646.1.S1_at	NA	NA	NA	NA	NA	NA	NA	NA	NA	Mmu.10646.1.S1_at_NA
Mmu.10665.1.S1_at	NA	NA	NA	NA	NA	NA	NA	NA	NA	Mmu.10665.1.S1_at_NA
Mmu.10677.1.S1_at	NA	NA	NA	NA	NA	NA	NA	NA	NA	Mmu.10677.1.S1_at_NA
Mmu.10692.1.A1_at	NA	NA	NA	NA	NA	NA	NA	NA	NA	Mmu.10692.1.A1_at_NA
Mmu.10692.1.S1_at	NA	NA	NA	NA	NA	NA	NA	NA	NA	Mmu.10692.1.S1_at_NA
Mmu.10706.1.S1_at	206341_at	IL2RA	interleukin 2 receptor, alpha	chr10p15-p14	3559	IL2RA	interleukin 2 receptor, alpha	---	574300	Mmu.10706.1.S1_at_IL2RA
Mmu.1071.1.S1_at	NA	NA	NA	NA	NA	NA	NA	NA	NA	Mmu.1071.1.S1_at_NA
Mmu.10769.1.S1_at	NA	NA	NA	NA	NA	NA	NA	NA	NA	Mmu.10769.1.S1_at_NA

Mmu.10771.1.S1_at	NA	NA	NA	NA	NA	NA	NA	NA	NA	Mmu.10771.1.S1_at_NA
Mmu.10780.1.S1_at	NA	NA	NA	NA	NA	NA	NA	NA	NA	Mmu.10780.1.S1_at_NA
Mmu.10797.1.S1_at	NA	NA	NA	NA	NA	NA	NA	NA	NA	Mmu.10797.1.S1_at_NA
Mmu.10798.1.S1_at	NA	NA	NA	NA	NA	NA	NA	NA	NA	Mmu.10798.1.S1_at_NA
Mmu.10798.1.S1_s_at	NA	NA	NA	NA	NA	NA	NA	NA	NA	Mmu.10798.1.S1_s_at_NA
Mmu.1081.1.S1_at	NA	NA	NA	NA	NA	NA	NA	NA	NA	Mmu.1081.1.S1_at_NA
Mmu.10820.1.S1_at	NA	NA	NA	NA	NA	NA	NA	NA	NA	Mmu.10820.1.S1_at_NA
Mmu.10820.1.S1_s_at	NA	NA	NA	NA	NA	NA	NA	NA	NA	Mmu.10820.1.S1_s_at_NA
Mmu.10830.1.S1_at	NA	NA	NA	NA	NA	NA	NA	NA	NA	Mmu.10830.1.S1_at_NA
Mmu.10843.1.S1_at	NA	NA	NA	NA	NA	NA	NA	NA	NA	Mmu.10843.1.S1_at_NA
Mmu.10853.1.S1_at	NA	NA	NA	NA	NA	NA	NA	NA	NA	Mmu.10853.1.S1_at_NA
Mmu.10858.1.S1_s_at	NA	NA	NA	NA	NA	NA	NA	NA	NA	Mmu.10858.1.S1_s_at_NA
Mmu.10874.1.S1_at	NA	NA	NA	NA	NA	NA	NA	NA	NA	Mmu.10874.1.S1_at_NA
Mmu.10885.1.S1_at	NA	NA	NA	NA	NA	NA	NA	NA	NA	Mmu.10885.1.S1_at_NA
Mmu.10886.1.S1_at	NA	NA	NA	NA	NA	NA	NA	NA	NA	Mmu.10886.1.S1_at_NA
Mmu.10905.1.S1_at	NA	NA	NA	NA	NA	NA	NA	NA	NA	Mmu.10905.1.S1_at_NA
Mmu.10913.1.S1_at	NA	NA	NA	NA	NA	NA	NA	NA	NA	Mmu.10913.1.S1_at_NA
Mmu.10924.1.S1_a_at	NA	NA	NA	NA	NA	NA	NA	NA	NA	Mmu.10924.1.S1_a_at_NA
Mmu.10938.1.S1_at	NA	NA	NA	NA	NA	NA	NA	NA	NA	Mmu.10938.1.S1_at_NA
Mmu.10939.1.S1_at	NA	NA	NA	NA	NA	NA	NA	NA	NA	Mmu.10939.1.S1_at_NA
Mmu.10941.2.S1_at	NA	NA	NA	NA	NA	NA	NA	NA	NA	Mmu.10941.2.S1_at_NA
Mmu.10947.1.A1_at	NA	NA	NA	NA	NA	NA	NA	NA	NA	Mmu.10947.1.A1_at_NA
Mmu.10973.1.S1_at	NA	NA	NA	NA	NA	NA	NA	NA	NA	Mmu.10973.1.S1_at_NA
Mmu.10979.1.S1_at	NA	NA	NA	NA	NA	NA	NA	NA	NA	Mmu.10979.1.S1_at_NA
Mmu.1098.1.A1_at	NA	NA	NA	NA	NA	NA	NA	NA	NA	Mmu.1098.1.A1_at_NA
Mmu.10988.1.S1_at	NA	NA	NA	NA	NA	NA	NA	NA	NA	Mmu.10988.1.S1_at_NA
Mmu.10994.1.S1_at	NA	NA	NA	NA	NA	NA	NA	NA	NA	Mmu.10994.1.S1_at_NA
Mmu.11000.1.S1_at	NA	NA	NA	NA	NA	NA	NA	NA	NA	Mmu.11000.1.S1_at_NA
Mmu.11032.1.A1_at	NA	NA	NA	NA	NA	NA	NA	NA	NA	Mmu.11032.1.A1_at_NA
Mmu.11047.2.S1_s_at	NA	NA	NA	NA	NA	NA	NA	NA	NA	Mmu.11047.2.S1_s_at_NA
Mmu.11049.1.S1_s_at	NA	NA	NA	NA	NA	NA	NA	NA	NA	Mmu.11049.1.S1_s_at_NA
Mmu.11053.1.S1_s_at	NA	NA	NA	NA	NA	NA	NA	NA	NA	Mmu.11053.1.S1_s_at_NA
Mmu.11074.1.S1_at	NA	NA	NA	NA	NA	NA	NA	NA	NA	Mmu.11074.1.S1_at_NA
Mmu.11077.2.S1_at	NA	NA	NA	NA	NA	NA	NA	NA	NA	Mmu.11077.2.S1_at_NA
Mmu.11093.1.S1_at	NA	NA	NA	NA	NA	NA	NA	NA	NA	Mmu.11093.1.S1_at_NA
Mmu.11097.1.S1_at	NA	NA	NA	NA	NA	NA	NA	NA	NA	Mmu.11097.1.S1_at_NA
Mmu.11135.1.S1_at	NA	NA	NA	NA	NA	NA	NA	NA	NA	Mmu.11135.1.S1_at_NA
Mmu.11137.1.S1_x_at	NA	NA	NA	NA	NA	NA	NA	NA	NA	Mmu.11137.1.S1_x_at_NA
Mmu.11137.2.S1_at	NA	NA	NA	NA	NA	NA	NA	NA	NA	Mmu.11137.2.S1_at_NA
Mmu.11141.1.S1_at	NA	NA	NA	NA	NA	NA	NA	NA	NA	Mmu.11141.1.S1_at_NA
Mmu.11151.1.S1_at	231897_at	PTGR1	prostaglandin reductase 1	chr9q31.3	22949	LTB4DH /// LTB4DH	NADP-dependent leukotriene B4 12-hydroxydehydrogenase /// similar to NADP-dependent leukotrien	---	695105 /// 708725	Mmu.11151.1.S1_at_PTGR1
Mmu.11151.1.S1_s_at	NA	NA	NA	NA	NA	NA	NA	NA	NA	Mmu.11151.1.S1_s_at_NA
Mmu.11154.1.A1_at	NA	NA	NA	NA	NA	NA	NA	NA	NA	Mmu.11154.1.A1_at_NA
Mmu.11154.2.S1_at	NA	NA	NA	NA	NA	NA	NA	NA	NA	Mmu.11154.2.S1_at_NA
Mmu.11164.1.S1_at	NA	NA	NA	NA	NA	NA	NA	NA	NA	Mmu.11164.1.S1_at_NA
Mmu.11165.2.S1_at	NA	NA	NA	NA	NA	NA	NA	NA	NA	Mmu.11165.2.S1_at_NA
Mmu.11167.1.A1_at	NA	NA	NA	NA	NA	NA	NA	NA	NA	Mmu.11167.1.A1_at_NA
Mmu.1117.1.S1_at	NA	NA	NA	NA	NA	NA	NA	NA	NA	Mmu.1117.1.S1_at_NA
Mmu.11179.1.S1_at	NA	NA	NA	NA	NA	NA	NA	NA	NA	Mmu.11179.1.S1_at_NA
Mmu.11185.2.S1_at	NA	NA	NA	NA	NA	NA	NA	NA	NA	Mmu.11185.2.S1_at_NA
Mmu.11188.1.S1_at	NA	NA	NA	NA	NA	NA	NA	NA	NA	Mmu.11188.1.S1_at_NA
Mmu.11192.1.S1_at	NA	NA	NA	NA	NA	NA	NA	NA	NA	Mmu.11192.1.S1_at_NA
Mmu.11195.1.S1_at	NA	NA	NA	NA	NA	NA	NA	NA	NA	Mmu.11195.1.S1_at_NA
Mmu.11197.1.S1_at	NA	NA	NA	NA	NA	NA	NA	NA	NA	Mmu.11197.1.S1_at_NA
Mmu.11201.1.S1_at	NA	NA	NA	NA	NA	NA	NA	NA	NA	Mmu.11201.1.S1_at_NA
Mmu.11204.1.S1_s_at	NA	NA	NA	NA	NA	NA	NA	NA	NA	Mmu.11204.1.S1_s_at_NA
Mmu.11208.1.S1_at	214702_at	FN1	fibronectin 1	chr2q34	2335	FN1	fibronectin 1	---	613269	Mmu.11208.1.S1_at_FN1
Mmu.11220.1.S1_at	NA	NA	NA	NA	NA	NA	NA	NA	NA	Mmu.11220.1.S1_at_NA
Mmu.11224.1.S1_at	212470_at	SPAG9	sperm associated antigen 9	chr17q21.33	9043	---	---	---	---	Mmu.11224.1.S1_at_SPAG9
Mmu.11229.1.S1_at	NA	NA	NA	NA	NA	NA	NA	NA	NA	Mmu.11229.1.S1_at_NA
Mmu.11231.1.A1_at	NA	NA	NA	NA	NA	NA	NA	NA	NA	Mmu.11231.1.A1_at_NA
Mmu.11231.1.S1_at	NA	NA	NA	NA	NA	NA	NA	NA	NA	Mmu.11231.1.S1_at_NA
Mmu.11243.2.S1_at	NA	NA	NA	NA	NA	NA	NA	NA	NA	Mmu.11243.2.S1_at_NA
Mmu.11250.2.S1_at	NA	NA	NA	NA	NA	NA	NA	NA	NA	Mmu.11250.2.S1_at_NA
Mmu.11268.1.S1_at	NA	NA	NA	NA	NA	NA	NA	NA	NA	Mmu.11268.1.S1_at_NA
Mmu.11289.1.S1_at	NA	NA	NA	NA	NA	NA	NA	NA	NA	Mmu.11289.1.S1_at_NA
Mmu.11306.1.S1_at	NA	NA	NA	NA	NA	NA	NA	NA	NA	Mmu.11306.1.S1_at_NA
Mmu.11314.1.S1_x_at	NA	NA	NA	NA	NA	NA	NA	NA	NA	Mmu.11314.1.S1_x_at_NA
Mmu.11342.1.S1_at	NA	NA	NA	NA	NA	NA	NA	NA	NA	Mmu.11342.1.S1_at_NA
Mmu.11343.1.S1_at	NA	NA	NA	NA	NA	NA	NA	NA	NA	Mmu.11343.1.S1_at_NA
Mmu.11358.1.S1_at	NA	NA	NA	NA	NA	NA	NA	NA	NA	Mmu.11358.1.S1_at_NA
Mmu.11363.1.S1_at	NA	NA	NA	NA	NA	NA	NA	NA	NA	Mmu.11363.1.S1_at_NA
Mmu.11366.1.S1_at	NA	NA	NA	NA	NA	NA	NA	NA	NA	Mmu.11366.1.S1_at_NA
Mmu.11367.1.S1_at	NA	NA	NA	NA	NA	NA	NA	NA	NA	Mmu.11367.1.S1_at_NA
Mmu.11369.1.S1_at	NA	NA	NA	NA	NA	NA	NA	NA	NA	Mmu.11369.1.S1_at_NA
Mmu.1137.1.S1_at	216920_s_at	TARP /// TRGC2	TCR gamma alternate reading frame protein /// T cell receptor gamma constant 2	chr7p14 /// chr7p15-p14	445347 /// 6967	LOC720538	Similar to T-cell receptor gamma chain C region PT-gamma-1/2	---	720538	Mmu.1137.1.S1_at_TARP /// TRGC2
Mmu.1137.1.S1_s_at	NA	NA	NA	NA	NA	NA	NA	NA	NA	Mmu.1137.1.S1_s_at_NA
Mmu.11373.1.S1_at	NA	NA	NA	NA	NA	NA	NA	NA	NA	Mmu.11373.1.S1_at_NA
Mmu.1138.1.S1_at	NA	NA	NA	NA	NA	NA	NA	NA	NA	Mmu.1138.1.S1_at_NA
Mmu.11384.1.S1_at	NA	NA	NA	NA	NA	NA	NA	NA	NA	Mmu.11384.1.S1_at_NA
Mmu.11403.1.S1_at	NA	NA	NA	NA	NA	NA	NA	NA	NA	Mmu.11403.1.S1_at_NA
Mmu.11403.1.S1_x_at	NA	NA	NA	NA	NA	NA	NA	NA	NA	Mmu.11403.1.S1_x_at_NA
Mmu.11412.1.S1_at	NA	NA	NA	NA	NA	NA	NA	NA	NA	Mmu.11412.1.S1_at_NA
Mmu.11413.1.S1_at	NA	NA	NA	NA	NA	NA	NA	NA	NA	Mmu.11413.1.S1_at_NA
Mmu.11414.1.S1_a_at	NA	NA	NA	NA	NA	NA	NA	NA	NA	Mmu.11414.1.S1_a_at_NA
Mmu.11416.1.A1_at	NA	NA	NA	NA	NA	NA	NA	NA	NA	Mmu.11416.1.A1_at_NA
Mmu.11416.1.S1_at	NA	NA	NA	NA	NA	NA	NA	NA	NA	Mmu.11416.1.S1_at_NA
Mmu.11417.1.S1_at	NA	NA	NA	NA	NA	NA	NA	NA	NA	Mmu.11417.1.S1_at_NA
Mmu.11419.1.S1_at	NA	NA	NA	NA	NA	NA	NA	NA	NA	Mmu.11419.1.S1_at_NA
Mmu.11420.1.S1_at	NA	NA	NA	NA	NA	NA	NA	NA	NA	Mmu.11420.1.S1_at_NA
Mmu.11421.1.S1_a_at	NA	NA	NA	NA	NA	NA	NA	NA	NA	Mmu.11421.1.S1_a_at_NA
Mmu.11423.1.S1_a_at	NA	NA	NA	NA	NA	NA	NA	NA	NA	Mmu.11423.1.S1_a_at_NA
Mmu.11429.1.A1_at	NA	NA	NA	NA	NA	NA	NA	NA	NA	Mmu.11429.1.A1_at_NA
Mmu.11473.1.S1_at	NA	NA	NA	NA	NA	NA	NA	NA	NA	Mmu.11473.1.S1_at_NA
Mmu.11473.1.S1_s_at	NA	NA	NA	NA	NA	NA	NA	NA	NA	Mmu.11473.1.S1_s_at_NA
Mmu.11492.1.S1_x_at	NA	NA	NA	NA	NA	NA	NA	NA	NA	Mmu.11492.1.S1_x_at_NA
Mmu.11516.1.S1_at	NA	NA	NA	NA	NA	NA	NA	NA	NA	Mmu.11516.1.S1_at_NA
Mmu.11526.1.S1_at	NA	NA	NA	NA	NA	NA	NA	NA	NA	Mmu.11526.1.S1_at_NA
Mmu.11557.1.S1_at	NA	NA	NA	NA	NA	NA	NA	NA	NA	Mmu.11557.1.S1_at_NA
Mmu.11562.1.S1_at	NA	NA	NA	NA	NA	NA	NA	NA	NA	Mmu.11562.1.S1_at_NA
Mmu.11565.1.S1_at	NA	NA	NA	NA	NA	NA	NA	NA	NA	Mmu.11565.1.S1_at_NA
Mmu.11584.1.S1_at	NA	NA	NA	NA	NA	NA	NA	NA	NA	Mmu.11584.1.S1_at_NA
Mmu.11607.1.S1_at	NA	NA	NA	NA	NA	NA	NA	NA	NA	Mmu.11607.1.S1_at_NA
Mmu.1161.1.S1_at	NA	NA	NA	NA	NA	NA	NA	NA	NA	Mmu.1161.1.S1_at_NA
Mmu.116.1.S1_s_at	218007_s_at	RPS27L	ribosomal protein S27-like	chr15q22.2	51065	LOC705483 /// LOC7084	similar to 40S ribosomal protein S27-like protein /// similar to 40S ribosomal protein S27-like protein	---	705483 /// 708492	Mmu.116.1.S1_s_at_RPS27L
Mmu.1162.1.S1_at	NA	NA	NA	NA	NA	NA	NA	NA	NA	Mmu.1162.1.S1_at_NA
Mmu.11635.1.S1_at	NA	NA	NA	NA	NA	NA	NA	NA	NA	Mmu.11635.1.S1_at_NA
Mmu.11636.1.S1_at	NA	NA	NA	NA	NA	NA	NA	NA	NA	Mmu.11636.1.S1_at_NA

Mmu.11638.2.S1_at	NA	NA	NA	NA	NA	NA	NA	NA	NA	Mmu.11638.2.S1_at_NA
Mmu.11640.1.S1_at	NA	NA	NA	NA	NA	NA	NA	NA	NA	Mmu.11640.1.S1_at_NA
Mmu.11645.2.S1_at	NA	NA	NA	NA	NA	NA	NA	NA	NA	Mmu.11645.2.S1_at_NA
Mmu.11646.1.S1_at	NA	NA	NA	NA	NA	NA	NA	NA	NA	Mmu.11646.1.S1_at_NA
Mmu.11647.1.A1_at	NA	NA	NA	NA	NA	NA	NA	NA	NA	Mmu.11647.1.A1_at_NA
Mmu.11662.1.S1_at	NA	NA	NA	NA	NA	NA	NA	NA	NA	Mmu.11662.1.S1_at_NA
Mmu.11663.1.S1_at	NA	NA	NA	NA	NA	NA	NA	NA	NA	Mmu.11663.1.S1_at_NA
Mmu.11667.1.S1_at	NA	NA	NA	NA	NA	NA	NA	NA	NA	Mmu.11667.1.S1_at_NA
Mmu.11667.1.S1_x_at	NA	NA	NA	NA	NA	NA	NA	NA	NA	Mmu.11667.1.S1_x_at_NA
Mmu.11668.1.S1_at	NA	NA	NA	NA	NA	NA	NA	NA	NA	Mmu.11668.1.S1_at_NA
Mmu.1167.1.S1_at	NA	NA	NA	NA	NA	NA	NA	NA	NA	Mmu.1167.1.S1_at_NA
Mmu.11672.1.S1_at	NA	NA	NA	NA	NA	NA	NA	NA	NA	Mmu.11672.1.S1_at_NA
Mmu.11693.1.S1_at	NA	NA	NA	NA	NA	NA	NA	NA	NA	Mmu.11693.1.S1_at_NA
Mmu.11695.1.S1_at	NA	NA	NA	NA	NA	NA	NA	NA	NA	Mmu.11695.1.S1_at_NA
Mmu.11700.1.S1_at	NA	NA	NA	NA	NA	NA	NA	NA	NA	Mmu.11700.1.S1_at_NA
Mmu.11707.1.S1_at	229401_at	IL17RE	Interleukin 17 receptor E	chr3p25.3	132014	IL17RE	interleukin 17 receptor E	---	698972	Mmu.11707.1.S1_at_IL17RE
Mmu.11727.1.S1_at	NA	NA	NA	NA	NA	NA	NA	NA	NA	Mmu.11727.1.S1_at_NA
Mmu.11729.1.S1_s_at	NA	NA	NA	NA	NA	NA	NA	NA	NA	Mmu.11729.1.S1_s_at_NA
Mmu.11739.1.S1_at	NA	NA	NA	NA	NA	NA	NA	NA	NA	Mmu.11739.1.S1_at_NA
Mmu.11741.1.S1_at	NA	NA	NA	NA	NA	NA	NA	NA	NA	Mmu.11741.1.S1_at_NA
Mmu.11741.2.S1_a_at	NA	NA	NA	NA	NA	NA	NA	NA	NA	Mmu.11741.2.S1_a_at_NA
Mmu.11745.1.A1_at	NA	NA	NA	NA	NA	NA	NA	NA	NA	Mmu.11745.1.A1_at_NA
Mmu.11775.1.S1_at	212124_at	ZMIZ1	zinc finger, MIZ-type containing 1	chr10q22.3	57178	ZMIZ1	zinc finger, MIZ-type containing 1	---	702311	Mmu.11775.1.S1_at_ZMIZ1
Mmu.11792.1.S1_at	NA	NA	NA	NA	NA	NA	NA	NA	NA	Mmu.11792.1.S1_at_NA
Mmu.11804.2.S1_at	NA	NA	NA	NA	NA	NA	NA	NA	NA	Mmu.11804.2.S1_at_NA
Mmu.1181.1.S1_at	NA	NA	NA	NA	NA	NA	NA	NA	NA	Mmu.1181.1.S1_at_NA
Mmu.11815.1.S1_s_at	NA	NA	NA	NA	NA	NA	NA	NA	NA	Mmu.11815.1.S1_s_at_NA
Mmu.11819.2.S1_at	NA	NA	NA	NA	NA	NA	NA	NA	NA	Mmu.11819.2.S1_at_NA
Mmu.11820.13.S1_a_at	NA	NA	NA	NA	NA	NA	NA	NA	NA	Mmu.11820.13.S1_a_at_NA
Mmu.11826.1.S1_at	208826_x_at	HINT1	histidine triad nucleotide binding protein 1	chr5q31.2	3094	HINT1	histidine triad nucleotide binding protein 1	---	706083	Mmu.11826.1.S1_at_HINT1
Mmu.11827.1.A1_at	NA	NA	NA	NA	NA	NA	NA	NA	NA	Mmu.11827.1.A1_at_NA
Mmu.11827.1.S1_at	NA	NA	NA	NA	NA	NA	NA	NA	NA	Mmu.11827.1.S1_at_NA
Mmu.11828.1.S1_at	NA	NA	NA	NA	NA	NA	NA	NA	NA	Mmu.11828.1.S1_at_NA
Mmu.1183.2.S1_at	NA	NA	NA	NA	NA	NA	NA	NA	NA	Mmu.1183.2.S1_at_NA
Mmu.1183.4.A1_at	NA	NA	NA	NA	NA	NA	NA	NA	NA	Mmu.1183.4.A1_at_NA
Mmu.1183.4.S1_at	NA	NA	NA	NA	NA	NA	NA	NA	NA	Mmu.1183.4.S1_at_NA
Mmu.11837.1.S1_at	NA	NA	NA	NA	NA	NA	NA	NA	NA	Mmu.11837.1.S1_at_NA
Mmu.11839.1.S1_at	NA	NA	NA	NA	NA	NA	NA	NA	NA	Mmu.11839.1.S1_at_NA
Mmu.11841.1.S1_s_at	NA	NA	NA	NA	NA	NA	NA	NA	NA	Mmu.11841.1.S1_s_at_NA
Mmu.1184.1.S1_at	NA	NA	NA	NA	NA	NA	NA	NA	NA	Mmu.1184.1.S1_at_NA
Mmu.11859.1.S1_at	NA	NA	NA	NA	NA	NA	NA	NA	NA	Mmu.11859.1.S1_at_NA
Mmu.11863.1.S1_at	NA	NA	NA	NA	NA	NA	NA	NA	NA	Mmu.11863.1.S1_at_NA
Mmu.11884.1.S1_at	NA	NA	NA	NA	NA	NA	NA	NA	NA	Mmu.11884.1.S1_at_NA
Mmu.11885.2.S1_at	NA	NA	NA	NA	NA	NA	NA	NA	NA	Mmu.11885.2.S1_at_NA
Mmu.11893.1.S1_at	NA	NA	NA	NA	NA	NA	NA	NA	NA	Mmu.11893.1.S1_at_NA
Mmu.1189.5.S1_x_at	NA	NA	NA	NA	NA	NA	NA	NA	NA	Mmu.1189.5.S1_x_at_NA
Mmu.11899.1.S1_at	NA	NA	NA	NA	NA	NA	NA	NA	NA	Mmu.11899.1.S1_at_NA
Mmu.11912.1.S1_at	NA	NA	NA	NA	NA	NA	NA	NA	NA	Mmu.11912.1.S1_at_NA
Mmu.11914.1.S1_at	NA	NA	NA	NA	NA	NA	NA	NA	NA	Mmu.11914.1.S1_at_NA
Mmu.11943.1.S1_at	NA	NA	NA	NA	NA	NA	NA	NA	NA	Mmu.11943.1.S1_at_NA
Mmu.11959.1.A1_at	NA	NA	NA	NA	NA	NA	NA	NA	NA	Mmu.11959.1.A1_at_NA
Mmu.11960.1.S1_at	NA	NA	NA	NA	NA	NA	NA	NA	NA	Mmu.11960.1.S1_at_NA
Mmu.11960.1.S1_x_at	NA	NA	NA	NA	NA	NA	NA	NA	NA	Mmu.11960.1.S1_x_at_NA
Mmu.11961.1.S1_at	NA	NA	NA	NA	NA	NA	NA	NA	NA	Mmu.11961.1.S1_at_NA
Mmu.11961.1.S1_x_at	NA	NA	NA	NA	NA	NA	NA	NA	NA	Mmu.11961.1.S1_x_at_NA
Mmu.11962.1.S1_at	NA	NA	NA	NA	NA	NA	NA	NA	NA	Mmu.11962.1.S1_at_NA
Mmu.11980.1.S1_at	NA	NA	NA	NA	NA	NA	NA	NA	NA	Mmu.11980.1.S1_at_NA
Mmu.1198.1.S1_at	NA	NA	NA	NA	NA	NA	NA	NA	NA	Mmu.1198.1.S1_at_NA
Mmu.11986.2.S1_at	NA	NA	NA	NA	NA	NA	NA	NA	NA	Mmu.11986.2.S1_at_NA
Mmu.11987.1.S1_at	NA	NA	NA	NA	NA	NA	NA	NA	NA	Mmu.11987.1.S1_at_NA
Mmu.11995.1.S1_at	NA	NA	NA	NA	NA	NA	NA	NA	NA	Mmu.11995.1.S1_at_NA
Mmu.11997.1.S1_at	200655_s_at	CALM1	calmodulin 1 (phosphorylase kinase, delta)	chr14q24-q31	801	LOC720263	Similar to protein kinase C, gamma	---	720263	Mmu.11997.1.S1_at_CALM1
Mmu.12003.1.S1_s_at	NA	NA	NA	NA	NA	NA	NA	NA	NA	Mmu.12003.1.S1_s_at_NA
Mmu.12005.1.S1_at	NA	NA	NA	NA	NA	NA	NA	NA	NA	Mmu.12005.1.S1_at_NA
Mmu.12007.1.S1_s_at	NA	NA	NA	NA	NA	NA	NA	NA	NA	Mmu.12007.1.S1_s_at_NA
Mmu.12009.1.S1_at	NA	NA	NA	NA	NA	NA	NA	NA	NA	Mmu.12009.1.S1_at_NA
Mmu.12027.2.S1_at	NA	NA	NA	NA	NA	NA	NA	NA	NA	Mmu.12027.2.S1_at_NA
Mmu.12027.3.S1_at	NA	NA	NA	NA	NA	NA	NA	NA	NA	Mmu.12027.3.S1_at_NA
Mmu.12045.1.S1_at	NA	NA	NA	NA	NA	NA	NA	NA	NA	Mmu.12045.1.S1_at_NA
Mmu.12046.1.S1_at	NA	NA	NA	NA	NA	NA	NA	NA	NA	Mmu.12046.1.S1_at_NA
Mmu.12059.1.S1_at	NA	NA	NA	NA	NA	NA	NA	NA	NA	Mmu.12059.1.S1_at_NA
Mmu.12077.1.S1_at	NA	NA	NA	NA	NA	NA	NA	NA	NA	Mmu.12077.1.S1_at_NA
Mmu.12078.1.S1_at	NA	NA	NA	NA	NA	NA	NA	NA	NA	Mmu.12078.1.S1_at_NA
Mmu.12079.1.A1_at	NA	NA	NA	NA	NA	NA	NA	NA	NA	Mmu.12079.1.A1_at_NA
Mmu.12087.1.S1_x_at	NA	NA	NA	NA	NA	NA	NA	NA	NA	Mmu.12087.1.S1_x_at_NA
Mmu.12088.1.S1_at	NA	NA	NA	NA	NA	NA	NA	NA	NA	Mmu.12088.1.S1_at_NA
Mmu.12088.1.S1_x_at	NA	NA	NA	NA	NA	NA	NA	NA	NA	Mmu.12088.1.S1_x_at_NA
Mmu.12088.2.A1_s_at	NA	NA	NA	NA	NA	NA	NA	NA	NA	Mmu.12088.2.A1_s_at_NA
Mmu.12088.2.S1_s_at	NA	NA	NA	NA	NA	NA	NA	NA	NA	Mmu.12088.2.S1_s_at_NA
Mmu.12088.3.A1_at	NA	NA	NA	NA	NA	NA	NA	NA	NA	Mmu.12088.3.A1_at_NA
Mmu.12088.3.A1_x_at	NA	NA	NA	NA	NA	NA	NA	NA	NA	Mmu.12088.3.A1_x_at_NA
Mmu.12088.3.S1_at	NA	NA	NA	NA	NA	NA	NA	NA	NA	Mmu.12088.3.S1_at_NA
Mmu.12088.3.S1_x_at	NA	NA	NA	NA	NA	NA	NA	NA	NA	Mmu.12088.3.S1_x_at_NA
Mmu.12088.4.A1_at	NA	NA	NA	NA	NA	NA	NA	NA	NA	Mmu.12088.4.A1_at_NA
Mmu.12088.4.A1_x_at	NA	NA	NA	NA	NA	NA	NA	NA	NA	Mmu.12088.4.A1_x_at_NA
Mmu.12088.4.S1_at	NA	NA	NA	NA	NA	NA	NA	NA	NA	Mmu.12088.4.S1_at_NA
Mmu.12088.4.S1_x_at	NA	NA	NA	NA	NA	NA	NA	NA	NA	Mmu.12088.4.S1_x_at_NA
Mmu.12089.1.S1_at	NA	NA	NA	NA	NA	NA	NA	NA	NA	Mmu.12089.1.S1_at_NA
Mmu.12089.1.S1_x_at	NA	NA	NA	NA	NA	NA	NA	NA	NA	Mmu.12089.1.S1_x_at_NA
Mmu.12090.1.S1_at	NA	NA	NA	NA	NA	NA	NA	NA	NA	Mmu.12090.1.S1_at_NA
Mmu.1209.5.S1_x_at	208357_x_at	CSH1	chorionic somatomammotropin hormone 1 (placental lactogen)	chr17q24.2	1442	CSH-4	chorionic somatommamotropin hormone 4	---	718116	Mmu.1209.5.S1_x_at_CSH1
Mmu.12096.1.S1_at	NA	NA	NA	NA	NA	NA	NA	NA	NA	Mmu.12096.1.S1_at_NA
Mmu.12098.1.S1_x_at	NA	NA	NA	NA	NA	NA	NA	NA	NA	Mmu.12098.1.S1_x_at_NA
Mmu.12098.2.S1_x_at	NA	NA	NA	NA	NA	NA	NA	NA	NA	Mmu.12098.2.S1_x_at_NA
Mmu.12104.1.A1_at	NA	NA	NA	NA	NA	NA	NA	NA	NA	Mmu.12104.1.A1_at_NA
Mmu.12104.1.S1_at	NA	NA	NA	NA	NA	NA	NA	NA	NA	Mmu.12104.1.S1_at_NA
Mmu.12105.1.S1_a_at	NA	NA	NA	NA	NA	NA	NA	NA	NA	Mmu.12105.1.S1_a_at_NA
Mmu.12110.1.S1_at	NA	NA	NA	NA	NA	NA	NA	NA	NA	Mmu.12110.1.S1_at_NA
Mmu.12129.1.S1_at	NA	NA	NA	NA	NA	NA	NA	NA	NA	Mmu.12129.1.S1_at_NA
Mmu.12135.1.S1_at	NA	NA	NA	NA	NA	NA	NA	NA	NA	Mmu.12135.1.S1_at_NA
Mmu.12136.2.S1_at	NA	NA	NA	NA	NA	NA	NA	NA	NA	Mmu.12136.2.S1_at_NA
Mmu.12145.1.S1_at	NA	NA	NA	NA	NA	NA	NA	NA	NA	Mmu.12145.1.S1_at_NA
Mmu.12145.1.S1_s_at	200032_s_at	RPL9	ribosomal protein L9	chr4p13	6133	LOC694093 /// LOC6993	similar to 60S ribosomal protein L9 /// similar to ribosomal protein L9 /// similar to ribosomal protein L9	---	694093 /// 699362 /// 7165	Mmu.12145.1.S1_s_at_RPL9
Mmu.1215.1.S1_at	NA	NA	NA	NA	NA	NA	NA	NA	NA	Mmu.1215.1.S1_at_NA
Mmu.12174.1.S1_at	NA	NA	NA	NA	NA	NA	NA	NA	NA	Mmu.12174.1.S1_at_NA
Mmu.1218.1.S1_at	NA	NA	NA	NA	NA	NA	NA	NA	NA	Mmu.1218.1.S1_at_NA
Mmu.12187.1.S1_at	NA	NA	NA	NA	NA	NA	NA	NA	NA	Mmu.12187.1.S1_at_NA
Mmu.12196.1.S1_at	NA	NA	NA	NA	NA	NA	NA	NA	NA	Mmu.12196.1.S1_at_NA

Mmu.12.1.S1_at	NA	NA	NA	NA	NA	NA	NA	NA	NA	Mmu.12.1.S1_at_NA
Mmu.12206.2.S1_at	NA	NA	NA	NA	NA	NA	NA	NA	NA	Mmu.12206.2.S1_at_NA
Mmu.12206.3.S1_at	NA	NA	NA	NA	NA	NA	NA	NA	NA	Mmu.12206.3.S1_at_NA
Mmu.12206.3.S1_x_at	NA	NA	NA	NA	NA	NA	NA	NA	NA	Mmu.12206.3.S1_x_at_NA
Mmu.12211.1.A1_at	NA	NA	NA	NA	NA	NA	NA	NA	NA	Mmu.12211.1.A1_at_NA
Mmu.12211.1.S1_at	NA	NA	NA	NA	NA	NA	NA	NA	NA	Mmu.12211.1.S1_at_NA
Mmu.12227.1.S1_at	NA	NA	NA	NA	NA	NA	NA	NA	NA	Mmu.12227.1.S1_at_NA
Mmu.12235.2.S1_at	NA	NA	NA	NA	NA	NA	NA	NA	NA	Mmu.12235.2.S1_at_NA
Mmu.12240.1.S1_at	NA	NA	NA	NA	NA	NA	NA	NA	NA	Mmu.12240.1.S1_at_NA
Mmu.12241.1.S1_at	NA	NA	NA	NA	NA	NA	NA	NA	NA	Mmu.12241.1.S1_at_NA
Mmu.12242.1.S1_at	NA	NA	NA	NA	NA	NA	NA	NA	NA	Mmu.12242.1.S1_at_NA
Mmu.12242.1.S1_x_at	NA	NA	NA	NA	NA	NA	NA	NA	NA	Mmu.12242.1.S1_x_at_NA
Mmu.12243.1.A1_at	NA	NA	NA	NA	NA	NA	NA	NA	NA	Mmu.12243.1.A1_at_NA
Mmu.12243.1.S1_at	NA	NA	NA	NA	NA	NA	NA	NA	NA	Mmu.12243.1.S1_at_NA
Mmu.12249.1.S1_at	NA	NA	NA	NA	NA	NA	NA	NA	NA	Mmu.12249.1.S1_at_NA
Mmu.12251.1.S1_s_at	NA	NA	NA	NA	NA	NA	NA	NA	NA	Mmu.12251.1.S1_s_at_NA
Mmu.12263.1.S1_at	217919_s_at	MRPL42	mitochondrial ribosomal protein L42	chr12q22	28977	LOC715676	similar to Mitochondrial 28S ribosomal protein S32 (S32mt) (MRP-S32)	---	715676	Mmu.12263.1.S1_at_MRPL42
Mmu.12264.1.S1_at	NA	NA	NA	NA	NA	NA	NA	NA	NA	Mmu.12264.1.S1_at_NA
Mmu.12266.1.S1_at	NA	NA	NA	NA	NA	NA	NA	NA	NA	Mmu.12266.1.S1_at_NA
Mmu.12268.1.S1_at	NA	NA	NA	NA	NA	NA	NA	NA	NA	Mmu.12268.1.S1_at_NA
Mmu.12272.2.A1_at	NA	NA	NA	NA	NA	NA	NA	NA	NA	Mmu.12272.2.A1_at_NA
Mmu.12272.2.S1_at	NA	NA	NA	NA	NA	NA	NA	NA	NA	Mmu.12272.2.S1_at_NA
Mmu.12281.1.S1_at	NA	NA	NA	NA	NA	NA	NA	NA	NA	Mmu.12281.1.S1_at_NA
Mmu.12285.1.S1_at	NA	NA	NA	NA	NA	NA	NA	NA	NA	Mmu.12285.1.S1_at_NA
Mmu.12305.1.S1_at	NA	NA	NA	NA	NA	NA	NA	NA	NA	Mmu.12305.1.S1_at_NA
Mmu.12307.1.S1_at	NA	NA	NA	NA	NA	NA	NA	NA	NA	Mmu.12307.1.S1_at_NA
Mmu.12320.1.S1_at	NA	NA	NA	NA	NA	NA	NA	NA	NA	Mmu.12320.1.S1_at_NA
Mmu.12324.1.S1_at	NA	NA	NA	NA	NA	NA	NA	NA	NA	Mmu.12324.1.S1_at_NA
Mmu.12330.1.S1_at	NA	NA	NA	NA	NA	NA	NA	NA	NA	Mmu.12330.1.S1_at_NA
Mmu.12345.2.S1_at	NA	NA	NA	NA	NA	NA	NA	NA	NA	Mmu.12345.2.S1_at_NA
Mmu.12356.1.S1_at	NA	NA	NA	NA	NA	NA	NA	NA	NA	Mmu.12356.1.S1_at_NA
Mmu.12356.1.S1_s_at	NA	NA	NA	NA	NA	NA	NA	NA	NA	Mmu.12356.1.S1_s_at_NA
Mmu.12364.1.S1_at	NA	NA	NA	NA	NA	NA	NA	NA	NA	Mmu.12364.1.S1_at_NA
Mmu.12364.1.S1_x_at	NA	NA	NA	NA	NA	NA	NA	NA	NA	Mmu.12364.1.S1_x_at_NA
Mmu.12381.1.S1_at	NA	NA	NA	NA	NA	NA	NA	NA	NA	Mmu.12381.1.S1_at_NA
Mmu.12382.1.S1_s_at	209224_s_at	NDUFA2	NADH dehydrogenase (ubiquinone) 1 alpha subcomplex, 2, 8kDa	chr5q31	4695	LOC697736	similar to NADH dehydrogenase (ubiquinone) 1 alpha subcomplex, 2, 8kDa	---	697736	Mmu.12382.1.S1_s_at_NDUFA2
Mmu.12383.1.S1_at	200066_at	IK	IK cytokine, down-regulator of HLA II	chr2p15-p14|5q31.3	3550	LOC696887	similar to WD repeat domain 55	---	696887	Mmu.12383.1.S1_at_IK
Mmu.12385.2.S1_x_at	214459_x_at	HLA-C	major histocompatibility complex, class I, C	chr6p21.3	3107	LOC696824	similar to HLA class I histocompatibility antigen, A-2 alpha chain precursor (MHC class I antigen A*2)	---	696824	Mmu.12385.2.S1_x_at_HLA-C
Mmu.12385.4.S1_x_at	209140_x_at	HLA-B	major histocompatibility complex, class I, B	chr6p21.3	3106	KRT18 /// LOC693294 ///	Keratin 18 /// Similar to 60S ribosomal protein L8 /// Similar to Glutamate dehydrogenase 1, mitocho	---	693294 /// 693843 /// 694	Mmu.12385.4.S1_x_at_HLA-B
Mmu.12385.5.S1_x_at	210514_x_at	HLA-G	major histocompatibility complex, class I, G	chr6p21.3	3135	LOC708939	Similar to splicing factor 3b, subunit 3	---	708939	Mmu.12385.5.S1_x_at_HLA-G
Mmu.12391.1.S1_at	216554_s_at	---	---	---	---	EDARADD	EDAR-associated death domain	---	710637	Mmu.12391.1.S1_at_---
Mmu.12394.1.S1_at	NA	NA	NA	NA	NA	NA	NA	NA	NA	Mmu.12394.1.S1_at_NA
Mmu.12401.1.S1_at	NA	NA	NA	NA	NA	NA	NA	NA	NA	Mmu.12401.1.S1_at_NA
Mmu.12401.1.S1_s_at	NA	NA	NA	NA	NA	NA	NA	NA	NA	Mmu.12401.1.S1_s_at_NA
Mmu.12405.1.S1_at	NA	NA	NA	NA	NA	NA	NA	NA	NA	Mmu.12405.1.S1_at_NA
Mmu.12407.1.S1_at	NA	NA	NA	NA	NA	NA	NA	NA	NA	Mmu.12407.1.S1_at_NA
Mmu.12414.1.S1_at	NA	NA	NA	NA	NA	NA	NA	NA	NA	Mmu.12414.1.S1_at_NA
Mmu.12423.1.S1_at	NA	NA	NA	NA	NA	NA	NA	NA	NA	Mmu.12423.1.S1_at_NA
Mmu.12425.1.S1_at	207349_s_at	UCP3	uncoupling protein 3 (mitochondrial, proton carrier)	chr11q13	7352	UCP3	uncoupling protein 3	---	574125	Mmu.12425.1.S1_at_UCP3
Mmu.12426.1.S1_at	NA	NA	NA	NA	NA	NA	NA	NA	NA	Mmu.12426.1.S1_at_NA
Mmu.12439.1.S1_s_at	204565_at	ACOT13	acyl-CoA thioesterase 13	chr6p22.2	55856	---	---	---	---	Mmu.12439.1.S1_s_at_ACOT13
Mmu.12441.1.S1_at	NA	NA	NA	NA	NA	NA	NA	NA	NA	Mmu.12441.1.S1_at_NA
Mmu.12449.1.S1_at	NA	NA	NA	NA	NA	NA	NA	NA	NA	Mmu.12449.1.S1_at_NA
Mmu.12457.1.S1_at	NA	NA	NA	NA	NA	NA	NA	NA	NA	Mmu.12457.1.S1_at_NA
Mmu.12457.1.S1_s_at	NA	NA	NA	NA	NA	NA	NA	NA	NA	Mmu.12457.1.S1_s_at_NA
Mmu.12458.2.S1_at	NA	NA	NA	NA	NA	NA	NA	NA	NA	Mmu.12458.2.S1_at_NA
Mmu.12458.2.S1_x_at	NA	NA	NA	NA	NA	NA	NA	NA	NA	Mmu.12458.2.S1_x_at_NA
Mmu.12467.1.S1_at	NA	NA	NA	NA	NA	NA	NA	NA	NA	Mmu.12467.1.S1_at_NA
Mmu.12468.1.S1_at	NA	NA	NA	NA	NA	NA	NA	NA	NA	Mmu.12468.1.S1_at_NA
Mmu.12476.2.S1_a_at	NA	NA	NA	NA	NA	NA	NA	NA	NA	Mmu.12476.2.S1_a_at_NA
Mmu.1249.1.S1_at	NA	NA	NA	NA	NA	NA	NA	NA	NA	Mmu.1249.1.S1_at_NA
Mmu.12497.1.S1_at	NA	NA	NA	NA	NA	NA	NA	NA	NA	Mmu.12497.1.S1_at_NA
Mmu.12516.1.A1_at	NA	NA	NA	NA	NA	NA	NA	NA	NA	Mmu.12516.1.A1_at_NA
Mmu.12516.1.S1_at	204406_at	FLT1	fms-related tyrosine kinase 1 (vascular endothelial growth factor/vascular permeability factor receptor	chr13q12	2321	FLT1	fms-related tyrosine kinase 1	---	574133	Mmu.12516.1.S1_at_FLT1
Mmu.125.1.S1_at	NA	NA	NA	NA	NA	NA	NA	NA	NA	Mmu.125.1.S1_at_NA
Mmu.12522.1.S1_at	NA	NA	NA	NA	NA	NA	NA	NA	NA	Mmu.12522.1.S1_at_NA
Mmu.12522.1.S1_x_at	200035_at	DULLARD	dullard homolog (Xenopus laevis)	chr17p13	23399	---	---	---	---	Mmu.12522.1.S1_x_at_DULLARD
Mmu.12524.1.S1_at	NA	NA	NA	NA	NA	NA	NA	NA	NA	Mmu.12524.1.S1_at_NA
Mmu.1257.1.S1_at	NA	NA	NA	NA	NA	NA	NA	NA	NA	Mmu.1257.1.S1_at_NA
Mmu.12585.1.S1_at	NA	NA	NA	NA	NA	NA	NA	NA	NA	Mmu.12585.1.S1_at_NA
Mmu.12596.1.S1_at	NA	NA	NA	NA	NA	NA	NA	NA	NA	Mmu.12596.1.S1_at_NA
Mmu.12603.1.S1_a_at	NA	NA	NA	NA	NA	NA	NA	NA	NA	Mmu.12603.1.S1_a_at_NA
Mmu.12603.1.S1_at	NA	NA	NA	NA	NA	NA	NA	NA	NA	Mmu.12603.1.S1_at_NA
Mmu.12603.2.S1_at	NA	NA	NA	NA	NA	NA	NA	NA	NA	Mmu.12603.2.S1_at_NA
Mmu.12611.1.S1_at	NA	NA	NA	NA	NA	NA	NA	NA	NA	Mmu.12611.1.S1_at_NA
Mmu.1261.1.S1_at	NA	NA	NA	NA	NA	NA	NA	NA	NA	Mmu.1261.1.S1_at_NA
Mmu.126.1.S1_a_at	NA	NA	NA	NA	NA	NA	NA	NA	NA	Mmu.126.1.S1_a_at_NA
Mmu.1262.1.A1_at	NA	NA	NA	NA	NA	NA	NA	NA	NA	Mmu.1262.1.A1_at_NA
Mmu.12628.1.S1_at	NA	NA	NA	NA	NA	NA	NA	NA	NA	Mmu.12628.1.S1_at_NA
Mmu.126.2.A1_at	NA	NA	NA	NA	NA	NA	NA	NA	NA	Mmu.126.2.A1_at_NA
Mmu.12633.1.S1_at	NA	NA	NA	NA	NA	NA	NA	NA	NA	Mmu.12633.1.S1_at_NA
Mmu.12638.1.S1_at	NA	NA	NA	NA	NA	NA	NA	NA	NA	Mmu.12638.1.S1_at_NA
Mmu.12642.1.S1_at	NA	NA	NA	NA	NA	NA	NA	NA	NA	Mmu.12642.1.S1_at_NA
Mmu.12644.1.S1_at	NA	NA	NA	NA	NA	NA	NA	NA	NA	Mmu.12644.1.S1_at_NA
Mmu.12647.1.S1_at	NA	NA	NA	NA	NA	NA	NA	NA	NA	Mmu.12647.1.S1_at_NA
Mmu.12651.3.S1_at	NA	NA	NA	NA	NA	NA	NA	NA	NA	Mmu.12651.3.S1_at_NA
Mmu.1265.1.S1_at	NA	NA	NA	NA	NA	NA	NA	NA	NA	Mmu.1265.1.S1_at_NA
Mmu.12653.1.S1_at	NA	NA	NA	NA	NA	NA	NA	NA	NA	Mmu.12653.1.S1_at_NA
Mmu.12672.1.S1_at	NA	NA	NA	NA	NA	NA	NA	NA	NA	Mmu.12672.1.S1_at_NA
Mmu.12672.1.S1_s_at	NA	NA	NA	NA	NA	NA	NA	NA	NA	Mmu.12672.1.S1_s_at_NA
Mmu.12708.1.S1_at	NA	NA	NA	NA	NA	NA	NA	NA	NA	Mmu.12708.1.S1_at_NA
Mmu.127.1.A1_at	NA	NA	NA	NA	NA	NA	NA	NA	NA	Mmu.127.1.A1_at_NA
Mmu.127.1.S1_at	234865_at	TRBV25-1	T cell receptor beta variable 25-1	chr7q34	28562	LOC702680	similar to T-cell receptor beta chain V region C5 precursor	---	702680	Mmu.127.1.S1_at_TRBV25-1
Mmu.12740.1.S1_at	NA	NA	NA	NA	NA	NA	NA	NA	NA	Mmu.12740.1.S1_at_NA
Mmu.12743.1.S1_at	NA	NA	NA	NA	NA	NA	NA	NA	NA	Mmu.12743.1.S1_at_NA
Mmu.12751.1.S1_at	NA	NA	NA	NA	NA	NA	NA	NA	NA	Mmu.12751.1.S1_at_NA
Mmu.12759.1.S1_at	NA	NA	NA	NA	NA	NA	NA	NA	NA	Mmu.12759.1.S1_at_NA
Mmu.1276.1.S1_at	210715_s_at	SPINT2	serine peptidase inhibitor, Kunitz type, 2	chr19q13.1	10653	LOC714755	similar to serine protease inhibitor, Kunitz type, 2	---	714755	Mmu.1276.1.S1_at_SPINT2
Mmu.1278.1.S1_s_at	NA	NA	NA	NA	NA	NA	NA	NA	NA	Mmu.1278.1.S1_s_at_NA
Mmu.12784.1.S1_at	NA	NA	NA	NA	NA	NA	NA	NA	NA	Mmu.12784.1.S1_at_NA
Mmu.12802.1.S1_at	NA	NA	NA	NA	NA	NA	NA	NA	NA	Mmu.12802.1.S1_at_NA
Mmu.12802.2.S1_a_at	NA	NA	NA	NA	NA	NA	NA	NA	NA	Mmu.12802.2.S1_a_at_NA
Mmu.12802.2.S1_at	NA	NA	NA	NA	NA	NA	NA	NA	NA	Mmu.12802.2.S1_at_NA
Mmu.12806.1.S1_at	203719_at	ERCC1	excision repair cross-complementing rodent repair deficiency, complementation group 1 (includes ove	chr19q13.2-q13.3	2067	ERCC1	excision repair cross-complementing rodent repair deficiency, complementation group 1 (includes ove	---	574267	Mmu.12806.1.S1_at_ERCC1
Mmu.12819.1.S1_at	NA	NA	NA	NA	NA	NA	NA	NA	NA	Mmu.12819.1.S1_at_NA
Mmu.12819.1.S1_s_at	NA	NA	NA	NA	NA	NA	NA	NA	NA	Mmu.12819.1.S1_s_at_NA
Mmu.128.1.S1_at	NA	NA	NA	NA	NA	NA	NA	NA	NA	Mmu.128.1.S1_at_NA
Mmu.12831.2.S1_at	NA	NA	NA	NA	NA	NA	NA	NA	NA	Mmu.12831.2.S1_at_NA

Mmu.12852.1.S1_at	NA	NA	NA	NA	NA	NA	NA	NA	NA	Mmu.12852.1.S1_at_NA
Mmu.12860.1.S1_at	NA	NA	NA	NA	NA	NA	NA	NA	NA	Mmu.12860.1.S1_at_NA
Mmu.12861.1.S1_at	224642_at	FYTTD1	forty-two-three domain containing 1	chr3q29	84248	LOC714705	similar to forty-two-three domain containing 1 isoform 1	---	714705	Mmu.12861.1.S1_at_FYTTD1
Mmu.12861.2.S1_at	NA	NA	NA	NA	NA	NA	NA	NA	NA	Mmu.12861.2.S1_at_NA
Mmu.12863.1.S1_at	NA	NA	NA	NA	NA	NA	NA	NA	NA	Mmu.12863.1.S1_at_NA
Mmu.12864.1.S1_at	NA	NA	NA	NA	NA	NA	NA	NA	NA	Mmu.12864.1.S1_at_NA
Mmu.12865.1.S1_s_at	NA	NA	NA	NA	NA	NA	NA	NA	NA	Mmu.12865.1.S1_s_at_NA
Mmu.12870.1.S1_at	200620_at	TMEM59	transmembrane protein 59	chr1p36-p31	9528	LOC700669	Similar to protocadherin gamma subfamily B, 8	---	700669	Mmu.12870.1.S1_at_TMEM59
Mmu.12873.1.S1_at	NA	NA	NA	NA	NA	NA	NA	NA	NA	Mmu.12873.1.S1_at_NA
Mmu.12875.1.S1_at	NA	NA	NA	NA	NA	NA	NA	NA	NA	Mmu.12875.1.S1_at_NA
Mmu.12880.1.S1_at	NA	NA	NA	NA	NA	NA	NA	NA	NA	Mmu.12880.1.S1_at_NA
Mmu.12897.1.A1_at	NA	NA	NA	NA	NA	NA	NA	NA	NA	Mmu.12897.1.A1_at_NA
Mmu.12897.1.S1_at	NA	NA	NA	NA	NA	NA	NA	NA	NA	Mmu.12897.1.S1_at_NA
Mmu.12899.1.S1_at	NA	NA	NA	NA	NA	NA	NA	NA	NA	Mmu.12899.1.S1_at_NA
Mmu.12915.1.A1_at	NA	NA	NA	NA	NA	NA	NA	NA	NA	Mmu.12915.1.A1_at_NA
Mmu.12915.1.S1_at	NA	NA	NA	NA	NA	NA	NA	NA	NA	Mmu.12915.1.S1_at_NA
Mmu.12921.1.S1_at	NA	NA	NA	NA	NA	NA	NA	NA	NA	Mmu.12921.1.S1_at_NA
Mmu.12925.2.S1_at	228544_s_at	CSRP2BP	CSRP2 binding protein	chr20p11.23	57325	---	---	---	---	Mmu.12925.2.S1_at_CSRP2BP
Mmu.12935.1.S1_at	NA	NA	NA	NA	NA	NA	NA	NA	NA	Mmu.12935.1.S1_at_NA
Mmu.12948.1.S1_at	NA	NA	NA	NA	NA	NA	NA	NA	NA	Mmu.12948.1.S1_at_NA
Mmu.12952.1.S1_at	NA	NA	NA	NA	NA	NA	NA	NA	NA	Mmu.12952.1.S1_at_NA
Mmu.12953.1.A1_at	NA	NA	NA	NA	NA	NA	NA	NA	NA	Mmu.12953.1.A1_at_NA
Mmu.12959.1.S1_at	NA	NA	NA	NA	NA	NA	NA	NA	NA	Mmu.12959.1.S1_at_NA
Mmu.12966.1.S1_at	NA	NA	NA	NA	NA	NA	NA	NA	NA	Mmu.12966.1.S1_at_NA
Mmu.12981.1.S1_at	223578_x_at	MALAT1	metastasis associated lung adenocarcinoma transcript 1 (non-protein coding)	chr11q13.1	378938	---	---	---	---	Mmu.12981.1.S1_at_MALAT1
Mmu.12981.1.S1_s_at	1558678_s_at	MALAT1	metastasis associated lung adenocarcinoma transcript 1 (non-protein coding)	chr11q13.1	378938	---	---	---	---	Mmu.12981.1.S1_s_at_MALAT1
Mmu.12987.1.S1_at	NA	NA	NA	NA	NA	NA	NA	NA	NA	Mmu.12987.1.S1_at_NA
Mmu.12990.1.S1_x_at	NA	NA	NA	NA	NA	NA	NA	NA	NA	Mmu.12990.1.S1_x_at_NA
Mmu.12999.1.S1_at	NA	NA	NA	NA	NA	NA	NA	NA	NA	Mmu.12999.1.S1_at_NA
Mmu.1300.1.S1_at	NA	NA	NA	NA	NA	NA	NA	NA	NA	Mmu.1300.1.S1_at_NA
Mmu.13011.1.S1_at	NA	NA	NA	NA	NA	NA	NA	NA	NA	Mmu.13011.1.S1_at_NA
Mmu.13012.1.S1_at	231418_at	---	---	---	---	MS4A1	membrane-spanning 4-domains, subfamily A, member 1	---	696843	Mmu.13012.1.S1_at_---
Mmu.13013.1.S1_at	205387_s_at	CGB7	chorionic gonadotropin, beta polypeptide /// chorionic gonadotropin, beta polypeptide 5 /// chorionic g	chr19q13.32	1082 /// 93659 /// 9402	CGB8	chorionic gonadotropin, beta polypeptide 8	---	574324	Mmu.13013.1.S1_at_CGB /// CGB5 /// C
Mmu.13013.1.S1_x_at	NA	NA	NA	NA	NA	NA	NA	NA	NA	Mmu.13013.1.S1_x_at_NA
Mmu.13018.1.S1_at	NA	NA	NA	NA	NA	NA	NA	NA	NA	Mmu.13018.1.S1_at_NA
Mmu.13018.2.S1_a_at	NA	NA	NA	NA	NA	NA	NA	NA	NA	Mmu.13018.2.S1_a_at_NA
Mmu.13021.1.S1_at	NA	NA	NA	NA	NA	NA	NA	NA	NA	Mmu.13021.1.S1_at_NA
Mmu.1303.1.A1_at	NA	NA	NA	NA	NA	NA	NA	NA	NA	Mmu.1303.1.A1_at_NA
Mmu.1303.1.S1_at	NA	NA	NA	NA	NA	NA	NA	NA	NA	Mmu.1303.1.S1_at_NA
Mmu.13051.1.S1_at	234377_at	TRBV7-8	T cell receptor beta variable 7-8	chr7q34	28590	LOC699298 /// LOC6995	similar to T-cell receptor beta chain V region CTL-L17 precursor /// similar to T-cell receptor beta chai	---	699298 /// 699543	Mmu.13051.1.S1_at_TRBV7-8
Mmu.13059.1.S1_at	NA	NA	NA	NA	NA	NA	NA	NA	NA	Mmu.13059.1.S1_at_NA
Mmu.13079.1.S1_at	NA	NA	NA	NA	NA	NA	NA	NA	NA	Mmu.13079.1.S1_at_NA
Mmu.13079.1.S1_s_at	NA	NA	NA	NA	NA	NA	NA	NA	NA	Mmu.13079.1.S1_s_at_NA
Mmu.13084.1.S1_x_at	211741_x_at	PSG3	pregnancy specific beta-1-glycoprotein 3	chr19q13.2	5671	---	---	---	---	Mmu.13084.1.S1_x_at_PSG3
Mmu.1309.1.S1_at	NA	NA	NA	NA	NA	NA	NA	NA	NA	Mmu.1309.1.S1_at_NA
Mmu.13092.1.S1_s_at	NA	NA	NA	NA	NA	NA	NA	NA	NA	Mmu.13092.1.S1_s_at_NA
Mmu.13096.1.S1_at	NA	NA	NA	NA	NA	NA	NA	NA	NA	Mmu.13096.1.S1_at_NA
Mmu.1310.2.S1_at	NA	NA	NA	NA	NA	NA	NA	NA	NA	Mmu.1310.2.S1_at_NA
Mmu.13104.1.S1_at	NA	NA	NA	NA	NA	NA	NA	NA	NA	Mmu.13104.1.S1_at_NA
Mmu.13105.1.S1_at	NA	NA	NA	NA	NA	NA	NA	NA	NA	Mmu.13105.1.S1_at_NA
Mmu.13110.1.S1_at	NA	NA	NA	NA	NA	NA	NA	NA	NA	Mmu.13110.1.S1_at_NA
Mmu.13110.1.S1_s_at	NA	NA	NA	NA	NA	NA	NA	NA	NA	Mmu.13110.1.S1_s_at_NA
Mmu.13111.1.S1_at	NA	NA	NA	NA	NA	NA	NA	NA	NA	Mmu.13111.1.S1_at_NA
Mmu.1314.2.S1_a_at	NA	NA	NA	NA	NA	NA	NA	NA	NA	Mmu.1314.2.S1_a_at_NA
Mmu.13156.2.A1_x_at	NA	NA	NA	NA	NA	NA	NA	NA	NA	Mmu.13156.2.A1_x_at_NA
Mmu.13156.2.S1_x_at	NA	NA	NA	NA	NA	NA	NA	NA	NA	Mmu.13156.2.S1_x_at_NA
Mmu.13156.3.A1_at	NA	NA	NA	NA	NA	NA	NA	NA	NA	Mmu.13156.3.A1_at_NA
Mmu.13156.3.S1_at	NA	NA	NA	NA	NA	NA	NA	NA	NA	Mmu.13156.3.S1_at_NA
Mmu.13156.4.S1_x_at	NA	NA	NA	NA	NA	NA	NA	NA	NA	Mmu.13156.4.S1_x_at_NA
Mmu.13156.5.A1_x_at	NA	NA	NA	NA	NA	NA	NA	NA	NA	Mmu.13156.5.A1_x_at_NA
Mmu.13156.5.S1_x_at	NA	NA	NA	NA	NA	NA	NA	NA	NA	Mmu.13156.5.S1_x_at_NA
Mmu.13168.1.S1_at	NA	NA	NA	NA	NA	NA	NA	NA	NA	Mmu.13168.1.S1_at_NA
Mmu.13168.2.S1_at	NA	NA	NA	NA	NA	NA	NA	NA	NA	Mmu.13168.2.S1_at_NA
Mmu.13181.1.S1_at	NA	NA	NA	NA	NA	NA	NA	NA	NA	Mmu.13181.1.S1_at_NA
Mmu.13184.1.S1_x_at	NA	NA	NA	NA	NA	NA	NA	NA	NA	Mmu.13184.1.S1_x_at_NA
Mmu.13185.1.S1_s_at	NA	NA	NA	NA	NA	NA	NA	NA	NA	Mmu.13185.1.S1_s_at_NA
Mmu.132.1.S1_at	NA	NA	NA	NA	NA	NA	NA	NA	NA	Mmu.132.1.S1_at_NA
Mmu.13251.1.A1_at	231559_at	NNMT	Nicotinamide N-methyltransferase	chr11q23.1	4837	LOC695185	Similar to nicotinamide N-methyltransferase	---	695185	Mmu.13251.1.A1_at_NNMT
Mmu.13254.1.S1_at	NA	NA	NA	NA	NA	NA	NA	NA	NA	Mmu.13254.1.S1_at_NA
Mmu.13271.1.S1_s_at	NA	NA	NA	NA	NA	NA	NA	NA	NA	Mmu.13271.1.S1_s_at_NA
Mmu.13281.1.S1_at	NA	NA	NA	NA	NA	NA	NA	NA	NA	Mmu.13281.1.S1_at_NA
Mmu.13282.1.A1_at	NA	NA	NA	NA	NA	NA	NA	NA	NA	Mmu.13282.1.A1_at_NA
Mmu.13282.1.S1_at	216510_x_at	IGHA1 /// IGHD /// IGH	immunoglobulin heavy constant alpha 1 /// immunoglobulin heavy constant delta /// immunoglobulin h	chr14q32.33	28396 /// 28442 /// 349	---	---	---	---	Mmu.13282.1.S1_at_IGHA1 /// IGHD ///
Mmu.13291.1.S1_at	NA	NA	NA	NA	NA	NA	NA	NA	NA	Mmu.13291.1.S1_at_NA
Mmu.13294.1.S1_at	NA	NA	NA	NA	NA	NA	NA	NA	NA	Mmu.13294.1.S1_at_NA
Mmu.13324.1.S1_at	NA	NA	NA	NA	NA	NA	NA	NA	NA	Mmu.13324.1.S1_at_NA
Mmu.13335.1.S1_at	NA	NA	NA	NA	NA	NA	NA	NA	NA	Mmu.13335.1.S1_at_NA
Mmu.13339.1.S1_at	NA	NA	NA	NA	NA	NA	NA	NA	NA	Mmu.13339.1.S1_at_NA
Mmu.13340.1.S1_at	NA	NA	NA	NA	NA	NA	NA	NA	NA	Mmu.13340.1.S1_at_NA
Mmu.13354.1.A1_at	NA	NA	NA	NA	NA	NA	NA	NA	NA	Mmu.13354.1.A1_at_NA
Mmu.13354.1.S1_at	208860_s_at	ATRX	alpha thalassemia/mental retardation syndrome X-linked (RAD54 homolog, S. cerevisiae)	chrXq13.1-q21.1	546	LOC705735	Similar to transcriptional regulator ATRX isoform 2	---	705735	Mmu.13354.1.S1_at_ATRX
Mmu.13356.1.S1_at	NA	NA	NA	NA	NA	NA	NA	NA	NA	Mmu.13356.1.S1_at_NA
Mmu.13360.1.S1_at	NA	NA	NA	NA	NA	NA	NA	NA	NA	Mmu.13360.1.S1_at_NA
Mmu.13368.1.S1_at	NA	NA	NA	NA	NA	NA	NA	NA	NA	Mmu.13368.1.S1_at_NA
Mmu.13380.2.S1_at	NA	NA	NA	NA	NA	NA	NA	NA	NA	Mmu.13380.2.S1_at_NA
Mmu.13384.1.S1_at	NA	NA	NA	NA	NA	NA	NA	NA	NA	Mmu.13384.1.S1_at_NA
Mmu.13405.1.S1_at	NA	NA	NA	NA	NA	NA	NA	NA	NA	Mmu.13405.1.S1_at_NA
Mmu.13435.1.S1_at	NA	NA	NA	NA	NA	NA	NA	NA	NA	Mmu.13435.1.S1_at_NA
Mmu.13445.1.S1_at	NA	NA	NA	NA	NA	NA	NA	NA	NA	Mmu.13445.1.S1_at_NA
Mmu.13450.1.S1_s_at	213350_at	RPS11	Ribosomal protein S11	chr19q13.3	6205	RPS11	ribosomal protein S11	---	718844	Mmu.13450.1.S1_s_at_RPS11
Mmu.13463.1.S1_at	NA	NA	NA	NA	NA	NA	NA	NA	NA	Mmu.13463.1.S1_at_NA
Mmu.13476.1.A1_at	NA	NA	NA	NA	NA	NA	NA	NA	NA	Mmu.13476.1.A1_at_NA
Mmu.13476.1.S1_at	NA	NA	NA	NA	NA	NA	NA	NA	NA	Mmu.13476.1.S1_at_NA
Mmu.13482.1.S1_at	NA	NA	NA	NA	NA	NA	NA	NA	NA	Mmu.13482.1.S1_at_NA
Mmu.13488.1.S1_at	NA	NA	NA	NA	NA	NA	NA	NA	NA	Mmu.13488.1.S1_at_NA
Mmu.13493.1.S1_at	NA	NA	NA	NA	NA	NA	NA	NA	NA	Mmu.13493.1.S1_at_NA
Mmu.13511.1.S1_at	NA	NA	NA	NA	NA	NA	NA	NA	NA	Mmu.13511.1.S1_at_NA
Mmu.1351.1.S1_at	NA	NA	NA	NA	NA	NA	NA	NA	NA	Mmu.1351.1.S1_at_NA
Mmu.13514.1.S1_s_at	NA	NA	NA	NA	NA	NA	NA	NA	NA	Mmu.13514.1.S1_s_at_NA
Mmu.13516.1.S1_at	NA	NA	NA	NA	NA	NA	NA	NA	NA	Mmu.13516.1.S1_at_NA
Mmu.13540.1.A1_at	NA	NA	NA	NA	NA	NA	NA	NA	NA	Mmu.13540.1.A1_at_NA
Mmu.13540.1.S1_at	NA	NA	NA	NA	NA	NA	NA	NA	NA	Mmu.13540.1.S1_at_NA
Mmu.13562.1.S1_at	NA	NA	NA	NA	NA	NA	NA	NA	NA	Mmu.13562.1.S1_at_NA
Mmu.13562.1.S1_x_at	200846_s_at	PPP1CA	protein phosphatase 1, catalytic subunit, alpha isoform	chr11q13	5499	LOC721735	similar to protein phosphatase 1, catalytic subunit, alpha	---	721735	Mmu.13562.1.S1_x_at_PPP1CA
Mmu.1356.2.S1_at	NA	NA	NA	NA	NA	NA	NA	NA	NA	Mmu.1356.2.S1_at_NA
Mmu.13564.1.S1_at	NA	NA	NA	NA	NA	NA	NA	NA	NA	Mmu.13564.1.S1_at_NA
Mmu.13566.1.A1_at	NA	NA	NA	NA	NA	NA	NA	NA	NA	Mmu.13566.1.A1_at_NA
Mmu.13566.1.S1_at	NA	NA	NA	NA	NA	NA	NA	NA	NA	Mmu.13566.1.S1_at_NA

Mmu.13573.1.S1_at	NA	NA	NA	NA	NA	NA	NA	NA	NA	Mmu.13573.1.S1_at_NA
Mmu.1358.2.S1_at	NA	NA	NA	NA	NA	NA	NA	NA	NA	Mmu.1358.2.S1_at_NA
Mmu.13595.1.S1_at	225153_at	GFM1	G elongation factor, mitochondrial 1	chr3q25.1-q26.2	85476	GFM1	G elongation factor, mitochondrial 1	---	706456	Mmu.13595.1.S1_at_GFM1
Mmu.13611.1.S1_at	NA	NA	NA	NA	NA	NA	NA	NA	NA	Mmu.13611.1.S1_at_NA
Mmu.13628.1.S1_s_at	NA	NA	NA	NA	NA	NA	NA	NA	NA	Mmu.13628.1.S1_s_at_NA
Mmu.13628.1.S1_x_at	223263_s_at	FGFR1OP2	FGFR1 oncogene partner 2	chr12p11.23	26127	LOC708905	similar to FGFR1 oncogene partner 2	---	708905	Mmu.13628.1.S1_x_at_FGFR1OP2
Mmu.13644.1.A1_at	NA	NA	NA	NA	NA	NA	NA	NA	NA	Mmu.13644.1.A1_at_NA
Mmu.13644.1.S1_at	NA	NA	NA	NA	NA	NA	NA	NA	NA	Mmu.13644.1.S1_at_NA
Mmu.13651.1.S1_s_at	NA	NA	NA	NA	NA	NA	NA	NA	NA	Mmu.13651.1.S1_s_at_NA
Mmu.13667.1.S1_s_at	NA	NA	NA	NA	NA	NA	NA	NA	NA	Mmu.13667.1.S1_s_at_NA
Mmu.1367.1.S1_at	NA	NA	NA	NA	NA	NA	NA	NA	NA	Mmu.1367.1.S1_at_NA
Mmu.1367.1.S1_x_at	NA	NA	NA	NA	NA	NA	NA	NA	NA	Mmu.1367.1.S1_x_at_NA
Mmu.13672.1.S1_s_at	NA	NA	NA	NA	NA	NA	NA	NA	NA	Mmu.13672.1.S1_s_at_NA
Mmu.13676.1.S1_at	NA	NA	NA	NA	NA	NA	NA	NA	NA	Mmu.13676.1.S1_at_NA
Mmu.13676.1.S1_s_at	NA	NA	NA	NA	NA	NA	NA	NA	NA	Mmu.13676.1.S1_s_at_NA
Mmu.13692.1.S1_at	NA	NA	NA	NA	NA	NA	NA	NA	NA	Mmu.13692.1.S1_at_NA
Mmu.13696.1.S1_at	NA	NA	NA	NA	NA	NA	NA	NA	NA	Mmu.13696.1.S1_at_NA
Mmu.13707.1.S1_at	NA	NA	NA	NA	NA	NA	NA	NA	NA	Mmu.13707.1.S1_at_NA
Mmu.13713.1.S1_s_at	NA	NA	NA	NA	NA	NA	NA	NA	NA	Mmu.13713.1.S1_s_at_NA
Mmu.13716.1.S1_at	NA	NA	NA	NA	NA	NA	NA	NA	NA	Mmu.13716.1.S1_at_NA
Mmu.13728.1.S1_a_at	NA	NA	NA	NA	NA	NA	NA	NA	NA	Mmu.13728.1.S1_a_at_NA
Mmu.13729.1.S1_at	NA	NA	NA	NA	NA	NA	NA	NA	NA	Mmu.13729.1.S1_at_NA
Mmu.13736.1.S1_at	NA	NA	NA	NA	NA	NA	NA	NA	NA	Mmu.13736.1.S1_at_NA
Mmu.13746.1.S1_at	NA	NA	NA	NA	NA	NA	NA	NA	NA	Mmu.13746.1.S1_at_NA
Mmu.13750.1.S1_at	NA	NA	NA	NA	NA	NA	NA	NA	NA	Mmu.13750.1.S1_at_NA
Mmu.13764.1.S1_s_at	NA	NA	NA	NA	NA	NA	NA	NA	NA	Mmu.13764.1.S1_s_at_NA
Mmu.13773.1.S1_at	NA	NA	NA	NA	NA	NA	NA	NA	NA	Mmu.13773.1.S1_at_NA
Mmu.13782.1.S1_at	NA	NA	NA	NA	NA	NA	NA	NA	NA	Mmu.13782.1.S1_at_NA
Mmu.13787.1.S1_at	NA	NA	NA	NA	NA	NA	NA	NA	NA	Mmu.13787.1.S1_at_NA
Mmu.13791.1.S1_at	NA	NA	NA	NA	NA	NA	NA	NA	NA	Mmu.13791.1.S1_at_NA
Mmu.1379.1.S1_at	NA	NA	NA	NA	NA	NA	NA	NA	NA	Mmu.1379.1.S1_at_NA
Mmu.13792.1.S1_at	NA	NA	NA	NA	NA	NA	NA	NA	NA	Mmu.13792.1.S1_at_NA
Mmu.13799.1.S1_at	NA	NA	NA	NA	NA	NA	NA	NA	NA	Mmu.13799.1.S1_at_NA
Mmu.13800.1.S1_at	NA	NA	NA	NA	NA	NA	NA	NA	NA	Mmu.13800.1.S1_at_NA
Mmu.13804.1.S1_s_at	208680_at	PRDX1	peroxiredoxin 1	chr1p34.1	5052	IL8RB	Similar to High affinity interleukin-8 receptor B (IL-8R B) (CXCR-2) (GRO/MGSA receptor) (IL-8 recep	---	697193	Mmu.13804.1.S1_s_at_PRDX1
Mmu.1384.2.S1_at	1568752_s_at	RGS13	regulator of G-protein signaling 13	chr1q31.2	6003	LOC712688	similar to regulator of G-protein signalling 13	---	712688	Mmu.1384.2.S1_at_RGS13
Mmu.13843.2.S1_s_at	NA	NA	NA	NA	NA	NA	NA	NA	NA	Mmu.13843.2.S1_s_at_NA
Mmu.13854.1.S1_at	NA	NA	NA	NA	NA	NA	NA	NA	NA	Mmu.13854.1.S1_at_NA
Mmu.13858.1.S1_at	NA	NA	NA	NA	NA	NA	NA	NA	NA	Mmu.13858.1.S1_at_NA
Mmu.13865.1.S1_s_at	NA	NA	NA	NA	NA	NA	NA	NA	NA	Mmu.13865.1.S1_s_at_NA
Mmu.13868.1.S1_at	NA	NA	NA	NA	NA	NA	NA	NA	NA	Mmu.13868.1.S1_at_NA
Mmu.1387.1.A1_at	NA	NA	NA	NA	NA	NA	NA	NA	NA	Mmu.1387.1.A1_at_NA
Mmu.13876.1.S1_at	NA	NA	NA	NA	NA	NA	NA	NA	NA	Mmu.13876.1.S1_at_NA
Mmu.13883.1.S1_s_at	NA	NA	NA	NA	NA	NA	NA	NA	NA	Mmu.13883.1.S1_s_at_NA
Mmu.13908.1.S1_at	NA	NA	NA	NA	NA	NA	NA	NA	NA	Mmu.13908.1.S1_at_NA
Mmu.13908.2.A1_at	NA	NA	NA	NA	NA	NA	NA	NA	NA	Mmu.13908.2.A1_at_NA
Mmu.13917.1.S1_at	NA	NA	NA	NA	NA	NA	NA	NA	NA	Mmu.13917.1.S1_at_NA
Mmu.13923.1.S1_at	NA	NA	NA	NA	NA	NA	NA	NA	NA	Mmu.13923.1.S1_at_NA
Mmu.1393.1.S1_s_at	200936_at	RPL8	ribosomal protein L8	chr8q24.3	6132	LOC708535	similar to 60S ribosomal protein L8	---	708535	Mmu.1393.1.S1_s_at_RPL8
Mmu.13937.1.S1_s_at	216559_x_at	HNRNPA1 /// HNRNPA	heterogeneous nuclear ribonucleoprotein A1 /// heterogeneous nuclear ribonucleoprotein A1-like 2 ///	chr10q11.22 /// chr11p15.4 /// ch	100128836 /// 120364 /	HNRPA1 /// HNRPA1 /// I	similar to Heterogeneous nuclear ribonucleoprotein A1 (Helix-destabilizing protein) (Single-strand RN	---	693386 /// 693967 /// 6974	Mmu.13937.1.S1_s_at_HNRNPA1 /// HI
Mmu.1394.1.S1_s_at	NA	NA	NA	NA	NA	NA	NA	NA	NA	Mmu.1394.1.S1_s_at_NA
Mmu.13944.1.A1_at	NA	NA	NA	NA	NA	NA	NA	NA	NA	Mmu.13944.1.A1_at_NA
Mmu.13944.1.S1_at	NA	NA	NA	NA	NA	NA	NA	NA	NA	Mmu.13944.1.S1_at_NA
Mmu.13947.1.S1_at	NA	NA	NA	NA	NA	NA	NA	NA	NA	Mmu.13947.1.S1_at_NA
Mmu.13948.1.S1_at	NA	NA	NA	NA	NA	NA	NA	NA	NA	Mmu.13948.1.S1_at_NA
Mmu.13956.1.S1_at	NA	NA	NA	NA	NA	NA	NA	NA	NA	Mmu.13956.1.S1_at_NA
Mmu.13959.1.S1_at	NA	NA	NA	NA	NA	NA	NA	NA	NA	Mmu.13959.1.S1_at_NA
Mmu.13961.1.S1_at	224436_s_at	NIPSNAP3A	nipsnap homolog 3A (C. elegans)	chr9q31.1	25934	LOC716188	similar to Protein NipSnap3A (NipSnap4) (Target for Salmonella secreted protein C) (TassC)	---	716188	Mmu.13961.1.S1_at_NIPSNAP3A
Mmu.1396.1.S1_at	NA	NA	NA	NA	NA	NA	NA	NA	NA	Mmu.1396.1.S1_at_NA
Mmu.13982.2.S1_at	NA	NA	NA	NA	NA	NA	NA	NA	NA	Mmu.13982.2.S1_at_NA
Mmu.13989.1.S1_at	NA	NA	NA	NA	NA	NA	NA	NA	NA	Mmu.13989.1.S1_at_NA
Mmu.13993.1.S1_at	NA	NA	NA	NA	NA	NA	NA	NA	NA	Mmu.13993.1.S1_at_NA
Mmu.13995.1.A1_at	NA	NA	NA	NA	NA	NA	NA	NA	NA	Mmu.13995.1.A1_at_NA
Mmu.14024.1.S1_at	NA	NA	NA	NA	NA	NA	NA	NA	NA	Mmu.14024.1.S1_at_NA
Mmu.14035.1.S1_at	NA	NA	NA	NA	NA	NA	NA	NA	NA	Mmu.14035.1.S1_at_NA
Mmu.14041.1.S1_at	NA	NA	NA	NA	NA	NA	NA	NA	NA	Mmu.14041.1.S1_at_NA
Mmu.14057.1.S1_at	NA	NA	NA	NA	NA	NA	NA	NA	NA	Mmu.14057.1.S1_at_NA
Mmu.14060.1.A1_at	NA	NA	NA	NA	NA	NA	NA	NA	NA	Mmu.14060.1.A1_at_NA
Mmu.14063.1.S1_at	NA	NA	NA	NA	NA	NA	NA	NA	NA	Mmu.14063.1.S1_at_NA
Mmu.14068.1.S1_at	NA	NA	NA	NA	NA	NA	NA	NA	NA	Mmu.14068.1.S1_at_NA
Mmu.14068.2.A1_at	NA	NA	NA	NA	NA	NA	NA	NA	NA	Mmu.14068.2.A1_at_NA
Mmu.14082.1.S1_at	NA	NA	NA	NA	NA	NA	NA	NA	NA	Mmu.14082.1.S1_at_NA
Mmu.14100.1.S1_at	NA	NA	NA	NA	NA	NA	NA	NA	NA	Mmu.14100.1.S1_at_NA
Mmu.14121.1.S1_at	NA	NA	NA	NA	NA	NA	NA	NA	NA	Mmu.14121.1.S1_at_NA
Mmu.1412.1.S1_at	NA	NA	NA	NA	NA	NA	NA	NA	NA	Mmu.1412.1.S1_at_NA
Mmu.14152.1.S1_at	NA	NA	NA	NA	NA	NA	NA	NA	NA	Mmu.14152.1.S1_at_NA
Mmu.14166.1.S1_at	NA	NA	NA	NA	NA	NA	NA	NA	NA	Mmu.14166.1.S1_at_NA
Mmu.14167.1.S1_at	NA	NA	NA	NA	NA	NA	NA	NA	NA	Mmu.14167.1.S1_at_NA
Mmu.14173.1.S1_at	NA	NA	NA	NA	NA	NA	NA	NA	NA	Mmu.14173.1.S1_at_NA
Mmu.14174.1.S1_at	NA	NA	NA	NA	NA	NA	NA	NA	NA	Mmu.14174.1.S1_at_NA
Mmu.14177.1.S1_at	NA	NA	NA	NA	NA	NA	NA	NA	NA	Mmu.14177.1.S1_at_NA
Mmu.14179.2.S1_at	NA	NA	NA	NA	NA	NA	NA	NA	NA	Mmu.14179.2.S1_at_NA
Mmu.14179.2.S1_x_at	NA	NA	NA	NA	NA	NA	NA	NA	NA	Mmu.14179.2.S1_x_at_NA
Mmu.14180.1.S1_at	NA	NA	NA	NA	NA	NA	NA	NA	NA	Mmu.14180.1.S1_at_NA
Mmu.14181.1.S1_at	NA	NA	NA	NA	NA	NA	NA	NA	NA	Mmu.14181.1.S1_at_NA
Mmu.14202.1.S1_at	201096_s_at	ARF4	ADP-ribosylation factor 4	chr3p21.2-p21.1	378	---	---	---	---	Mmu.14202.1.S1_at_ARF4
Mmu.14209.1.A1_at	NA	NA	NA	NA	NA	NA	NA	NA	NA	Mmu.14209.1.A1_at_NA
Mmu.14209.1.S1_at	NA	NA	NA	NA	NA	NA	NA	NA	NA	Mmu.14209.1.S1_at_NA
Mmu.14221.2.S1_at	NA	NA	NA	NA	NA	NA	NA	NA	NA	Mmu.14221.2.S1_at_NA
Mmu.14247.1.S1_at	NA	NA	NA	NA	NA	NA	NA	NA	NA	Mmu.14247.1.S1_at_NA
Mmu.14249.1.S1_at	NA	NA	NA	NA	NA	NA	NA	NA	NA	Mmu.14249.1.S1_at_NA
Mmu.1425.1.S1_at	NA	NA	NA	NA	NA	NA	NA	NA	NA	Mmu.1425.1.S1_at_NA
Mmu.1426.1.S1_at	NA	NA	NA	NA	NA	NA	NA	NA	NA	Mmu.1426.1.S1_at_NA
Mmu.14263.2.S1_at	NA	NA	NA	NA	NA	NA	NA	NA	NA	Mmu.14263.2.S1_at_NA
Mmu.14275.1.S1_at	NA	NA	NA	NA	NA	NA	NA	NA	NA	Mmu.14275.1.S1_at_NA
Mmu.14298.1.S1_at	NA	NA	NA	NA	NA	NA	NA	NA	NA	Mmu.14298.1.S1_at_NA
Mmu.14306.1.S1_s_at	NA	NA	NA	NA	NA	NA	NA	NA	NA	Mmu.14306.1.S1_s_at_NA
Mmu.14311.1.S1_at	NA	NA	NA	NA	NA	NA	NA	NA	NA	Mmu.14311.1.S1_at_NA
Mmu.14315.1.S1_at	NA	NA	NA	NA	NA	NA	NA	NA	NA	Mmu.14315.1.S1_at_NA
Mmu.14317.2.S1_at	NA	NA	NA	NA	NA	NA	NA	NA	NA	Mmu.14317.2.S1_at_NA
Mmu.14327.1.S1_at	NA	NA	NA	NA	NA	NA	NA	NA	NA	Mmu.14327.1.S1_at_NA
Mmu.14341.1.S1_at	NA	NA	NA	NA	NA	NA	NA	NA	NA	Mmu.14341.1.S1_at_NA
Mmu.14369.1.S1_at	NA	NA	NA	NA	NA	NA	NA	NA	NA	Mmu.14369.1.S1_at_NA
Mmu.14389.1.S1_at	NA	NA	NA	NA	NA	NA	NA	NA	NA	Mmu.14389.1.S1_at_NA
Mmu.14395.1.S1_at	NA	NA	NA	NA	NA	NA	NA	NA	NA	Mmu.14395.1.S1_at_NA
Mmu.14396.1.S1_at	NA	NA	NA	NA	NA	NA	NA	NA	NA	Mmu.14396.1.S1_at_NA
Mmu.1440.1.S1_at	NA	NA	NA	NA	NA	NA	NA	NA	NA	Mmu.1440.1.S1_at_NA
Mmu.14402.2.A1_at	210915_x_at	TRBC1	T cell receptor beta constant 1	chr7q34	28639	LOC702904	Similar to T-cell receptor beta chain C region	---	702904	Mmu.14402.2.A1_at_TRBC1

Mmu.14402.2.S1_at	NA	NA	NA	NA	NA	NA	NA	NA	NA	Mmu.14402.2.S1_at_NA
Mmu.14426.1.S1_at	NA	NA	NA	NA	NA	NA	NA	NA	NA	Mmu.14426.1.S1_at_NA
Mmu.14429.1.A1_at	NA	NA	NA	NA	NA	NA	NA	NA	NA	Mmu.14429.1.A1_at_NA
Mmu.14429.1.S1_at	NA	NA	NA	NA	NA	NA	NA	NA	NA	Mmu.14429.1.S1_at_NA
Mmu.14431.1.S1_at	NA	NA	NA	NA	NA	NA	NA	NA	NA	Mmu.14431.1.S1_at_NA
Mmu.14436.1.S1_at	NA	NA	NA	NA	NA	NA	NA	NA	NA	Mmu.14436.1.S1_at_NA
Mmu.14440.1.S1_at	NA	NA	NA	NA	NA	NA	NA	NA	NA	Mmu.14440.1.S1_at_NA
Mmu.14441.1.S1_at	NA	NA	NA	NA	NA	NA	NA	NA	NA	Mmu.14441.1.S1_at_NA
Mmu.14442.2.S1_at	NA	NA	NA	NA	NA	NA	NA	NA	NA	Mmu.14442.2.S1_at_NA
Mmu.14456.1.S1_at	NA	NA	NA	NA	NA	NA	NA	NA	NA	Mmu.14456.1.S1_at_NA
Mmu.14457.1.S1_at	203207_s_at	MTFR1	mitochondrial fission regulator 1	chr8q13.1	9650	MTFR1	Mitochondrial fission regulator 1	---	711143	Mmu.14457.1.S1_at_MTFR1
Mmu.1447.1.A1_at	NA	NA	NA	NA	NA	NA	NA	NA	NA	Mmu.1447.1.A1_at_NA
Mmu.1447.1.S1_at	NA	NA	NA	NA	NA	NA	NA	NA	NA	Mmu.1447.1.S1_at_NA
Mmu.14488.1.S1_x_at	NA	NA	NA	NA	NA	NA	NA	NA	NA	Mmu.14488.1.S1_x_at_NA
Mmu.14495.1.S1_at	NA	NA	NA	NA	NA	NA	NA	NA	NA	Mmu.14495.1.S1_at_NA
Mmu.14509.2.S1_at	NA	NA	NA	NA	NA	NA	NA	NA	NA	Mmu.14509.2.S1_at_NA
Mmu.14510.1.S2_at	NA	NA	NA	NA	NA	NA	NA	NA	NA	Mmu.14510.1.S2_at_NA
Mmu.1451.1.S1_at	200006_at	PARK7	Parkinson disease (autosomal recessive, early onset) 7	chr1p36.23	11315	PARK7	similar to DJ-1 protein	---	708377	Mmu.1451.1.S1_at_PARK7
Mmu.14519.1.S1_at	NA	NA	NA	NA	NA	NA	NA	NA	NA	Mmu.14519.1.S1_at_NA
Mmu.14534.1.S1_at	NA	NA	NA	NA	NA	NA	NA	NA	NA	Mmu.14534.1.S1_at_NA
Mmu.14535.1.S1_at	NA	NA	NA	NA	NA	NA	NA	NA	NA	Mmu.14535.1.S1_at_NA
Mmu.14551.1.S1_at	NA	NA	NA	NA	NA	NA	NA	NA	NA	Mmu.14551.1.S1_at_NA
Mmu.14552.1.S1_a_at	NA	NA	NA	NA	NA	NA	NA	NA	NA	Mmu.14552.1.S1_a_at_NA
Mmu.14560.3.S1_at	NA	NA	NA	NA	NA	NA	NA	NA	NA	Mmu.14560.3.S1_at_NA
Mmu.14562.1.S1_at	NA	NA	NA	NA	NA	NA	NA	NA	NA	Mmu.14562.1.S1_at_NA
Mmu.14571.1.S1_at	NA	NA	NA	NA	NA	NA	NA	NA	NA	Mmu.14571.1.S1_at_NA
Mmu.1457.1.S1_at	NA	NA	NA	NA	NA	NA	NA	NA	NA	Mmu.1457.1.S1_at_NA
Mmu.1457.1.S1_x_at	NA	NA	NA	NA	NA	NA	NA	NA	NA	Mmu.1457.1.S1_x_at_NA
Mmu.14583.1.S1_at	NA	NA	NA	NA	NA	NA	NA	NA	NA	Mmu.14583.1.S1_at_NA
Mmu.14589.1.A1_at	NA	NA	NA	NA	NA	NA	NA	NA	NA	Mmu.14589.1.A1_at_NA
Mmu.14593.1.S1_at	NA	NA	NA	NA	NA	NA	NA	NA	NA	Mmu.14593.1.S1_at_NA
Mmu.14635.1.S1_s_at	NA	NA	NA	NA	NA	NA	NA	NA	NA	Mmu.14635.1.S1_s_at_NA
Mmu.14640.1.S1_x_at	NA	NA	NA	NA	NA	NA	NA	NA	NA	Mmu.14640.1.S1_x_at_NA
Mmu.14669.1.S1_at	NA	NA	NA	NA	NA	NA	NA	NA	NA	Mmu.14669.1.S1_at_NA
Mmu.14676.1.S1_at	NA	NA	NA	NA	NA	NA	NA	NA	NA	Mmu.14676.1.S1_at_NA
Mmu.14695.1.A1_at	NA	NA	NA	NA	NA	NA	NA	NA	NA	Mmu.14695.1.A1_at_NA
Mmu.14710.1.S1_at	NA	NA	NA	NA	NA	NA	NA	NA	NA	Mmu.14710.1.S1_at_NA
Mmu.14712.1.S1_at	NA	NA	NA	NA	NA	NA	NA	NA	NA	Mmu.14712.1.S1_at_NA
Mmu.14715.2.S1_at	NA	NA	NA	NA	NA	NA	NA	NA	NA	Mmu.14715.2.S1_at_NA
Mmu.14728.1.S1_at	NA	NA	NA	NA	NA	NA	NA	NA	NA	Mmu.14728.1.S1_at_NA
Mmu.14737.1.S1_s_at	201441_at	COX6B1	cytochrome c oxidase subunit Vib polypeptide 1 (ubiquitous)	chr19q13.1	1340	COX6B	cytochrome c oxidase subunit 6B	---	692062	Mmu.14737.1.S1_s_at_COX6B1
Mmu.14749.1.S1_s_at	NA	NA	NA	NA	NA	NA	NA	NA	NA	Mmu.14749.1.S1_s_at_NA
Mmu.14771.1.S1_at	NA	NA	NA	NA	NA	NA	NA	NA	NA	Mmu.14771.1.S1_at_NA
Mmu.1477.1.S1_at	NA	NA	NA	NA	NA	NA	NA	NA	NA	Mmu.1477.1.S1_at_NA
Mmu.14774.1.S1_at	NA	NA	NA	NA	NA	NA	NA	NA	NA	Mmu.14774.1.S1_at_NA
Mmu.14785.1.S1_at	NA	NA	NA	NA	NA	NA	NA	NA	NA	Mmu.14785.1.S1_at_NA
Mmu.14786.1.S1_at	NA	NA	NA	NA	NA	NA	NA	NA	NA	Mmu.14786.1.S1_at_NA
Mmu.14787.1.S1_at	NA	NA	NA	NA	NA	NA	NA	NA	NA	Mmu.14787.1.S1_at_NA
Mmu.14792.1.S1_at	NA	NA	NA	NA	NA	NA	NA	NA	NA	Mmu.14792.1.S1_at_NA
Mmu.14795.1.S1_at	NA	NA	NA	NA	NA	NA	NA	NA	NA	Mmu.14795.1.S1_at_NA
Mmu.14798.1.S1_s_at	203835_at	LRRC32	leucine rich repeat containing 32	chr11q13.5-q14	2615	LRRC32	leucine rich repeat containing 32	---	697438	Mmu.14798.1.S1_s_at_LRRC32
Mmu.14801.1.S1_at	NA	NA	NA	NA	NA	NA	NA	NA	NA	Mmu.14801.1.S1_at_NA
Mmu.14807.1.S1_s_at	NA	NA	NA	NA	NA	NA	NA	NA	NA	Mmu.14807.1.S1_s_at_NA
Mmu.14809.1.S1_at	NA	NA	NA	NA	NA	NA	NA	NA	NA	Mmu.14809.1.S1_at_NA
Mmu.1481.2.S1_at	NA	NA	NA	NA	NA	NA	NA	NA	NA	Mmu.1481.2.S1_at_NA
Mmu.1481.2.S1_s_at	NA	NA	NA	NA	NA	NA	NA	NA	NA	Mmu.1481.2.S1_s_at_NA
Mmu.14814.1.S1_at	NA	NA	NA	NA	NA	NA	NA	NA	NA	Mmu.14814.1.S1_at_NA
Mmu.14836.1.S1_at	NA	NA	NA	NA	NA	NA	NA	NA	NA	Mmu.14836.1.S1_at_NA
Mmu.14838.1.S1_at	NA	NA	NA	NA	NA	NA	NA	NA	NA	Mmu.14838.1.S1_at_NA
Mmu.1485.1.S1_at	NA	NA	NA	NA	NA	NA	NA	NA	NA	Mmu.1485.1.S1_at_NA
Mmu.14856.1.A1_at	NA	NA	NA	NA	NA	NA	NA	NA	NA	Mmu.14856.1.A1_at_NA
Mmu.14866.1.S1_at	NA	NA	NA	NA	NA	NA	NA	NA	NA	Mmu.14866.1.S1_at_NA
Mmu.14874.1.S1_at	228215_at	---	---	---	---	ADD3	adducin 3 (gamma)	---	677712	Mmu.14874.1.S1_at_---
Mmu.14893.1.S1_x_at	NA	NA	NA	NA	NA	NA	NA	NA	NA	Mmu.14893.1.S1_x_at_NA
Mmu.14901.1.S1_at	NA	NA	NA	NA	NA	NA	NA	NA	NA	Mmu.14901.1.S1_at_NA
Mmu.14917.1.S1_s_at	NA	NA	NA	NA	NA	NA	NA	NA	NA	Mmu.14917.1.S1_s_at_NA
Mmu.14917.2.S1_at	NA	NA	NA	NA	NA	NA	NA	NA	NA	Mmu.14917.2.S1_at_NA
Mmu.14931.1.A1_at	NA	NA	NA	NA	NA	NA	NA	NA	NA	Mmu.14931.1.A1_at_NA
Mmu.14932.1.S1_at	NA	NA	NA	NA	NA	NA	NA	NA	NA	Mmu.14932.1.S1_at_NA
Mmu.14942.1.S1_at	NA	NA	NA	NA	NA	NA	NA	NA	NA	Mmu.14942.1.S1_at_NA
Mmu.14945.2.S1_at	NA	NA	NA	NA	NA	NA	NA	NA	NA	Mmu.14945.2.S1_at_NA
Mmu.14958.1.S1_at	NA	NA	NA	NA	NA	NA	NA	NA	NA	Mmu.14958.1.S1_at_NA
Mmu.14962.1.S1_at	NA	NA	NA	NA	NA	NA	NA	NA	NA	Mmu.14962.1.S1_at_NA
Mmu.14966.1.S1_at	NA	NA	NA	NA	NA	NA	NA	NA	NA	Mmu.14966.1.S1_at_NA
Mmu.1499.1.S1_at	NA	NA	NA	NA	NA	NA	NA	NA	NA	Mmu.1499.1.S1_at_NA
Mmu.14994.1.S1_at	NA	NA	NA	NA	NA	NA	NA	NA	NA	Mmu.14994.1.S1_at_NA
Mmu.15003.1.S1_at	NA	NA	NA	NA	NA	NA	NA	NA	NA	Mmu.15003.1.S1_at_NA
Mmu.15003.1.S1_s_at	NA	NA	NA	NA	NA	NA	NA	NA	NA	Mmu.15003.1.S1_s_at_NA
Mmu.15003.1.S1_x_at	NA	NA	NA	NA	NA	NA	NA	NA	NA	Mmu.15003.1.S1_x_at_NA
Mmu.15008.1.S1_at	NA	NA	NA	NA	NA	NA	NA	NA	NA	Mmu.15008.1.S1_at_NA
Mmu.15009.1.S1_at	NA	NA	NA	NA	NA	NA	NA	NA	NA	Mmu.15009.1.S1_at_NA
Mmu.150.1.S1_at	NA	NA	NA	NA	NA	NA	NA	NA	NA	Mmu.150.1.S1_at_NA
Mmu.15028.1.S1_at	NA	NA	NA	NA	NA	NA	NA	NA	NA	Mmu.15028.1.S1_at_NA
Mmu.15029.1.A1_at	NA	NA	NA	NA	NA	NA	NA	NA	NA	Mmu.15029.1.A1_at_NA
Mmu.15029.1.S1_at	NA	NA	NA	NA	NA	NA	NA	NA	NA	Mmu.15029.1.S1_at_NA
Mmu.15032.1.S1_at	NA	NA	NA	NA	NA	NA	NA	NA	NA	Mmu.15032.1.S1_at_NA
Mmu.15035.1.S1_at	NA	NA	NA	NA	NA	NA	NA	NA	NA	Mmu.15035.1.S1_at_NA
Mmu.15041.1.S1_at	NA	NA	NA	NA	NA	NA	NA	NA	NA	Mmu.15041.1.S1_at_NA
Mmu.15054.1.S1_at	209015_s_at	DNAJB6	DnaJ (Hsp40) homolog, subfamily B, member 6	chr7q36.3	10049	DNAJB6	DnaJ (Hsp40) homolog, subfamily B, member 6	---	693305	Mmu.15054.1.S1_at_DNAJB6
Mmu.15056.1.S2_at	NA	NA	NA	NA	NA	NA	NA	NA	NA	Mmu.15056.1.S2_at_NA
Mmu.15059.1.S1_at	NA	NA	NA	NA	NA	NA	NA	NA	NA	Mmu.15059.1.S1_at_NA
Mmu.15062.1.S1_at	NA	NA	NA	NA	NA	NA	NA	NA	NA	Mmu.15062.1.S1_at_NA
Mmu.15077.1.S1_at	1554365_a_at	PPP2R5C	protein phosphatase 2, regulatory subunit B', gamma isoform	chr14q32	5527	LOC706718	similar to gamma isoform of regulatory subunit B56, protein phosphatase 2A isoform b	---	706718	Mmu.15077.1.S1_at_PPP2R5C
Mmu.15102.1.S1_at	NA	NA	NA	NA	NA	NA	NA	NA	NA	Mmu.15102.1.S1_at_NA
Mmu.15103.2.A1_at	NA	NA	NA	NA	NA	NA	NA	NA	NA	Mmu.15103.2.A1_at_NA
Mmu.15103.2.S1_at	NA	NA	NA	NA	NA	NA	NA	NA	NA	Mmu.15103.2.S1_at_NA
Mmu.15115.1.S1_at	231705_at	HRSP12	Heat-responsive protein 12	chr8q22	10247	LOC705533	similar to Ribonuclease UK114 (14.5 kDa translational inhibitor protein) (p14.5) (UK114 antigen homo	---	705533	Mmu.15115.1.S1_at_HRSP12
Mmu.15125.1.S1_at	NA	NA	NA	NA	NA	NA	NA	NA	NA	Mmu.15125.1.S1_at_NA
Mmu.15143.1.S1_at	NA	NA	NA	NA	NA	NA	NA	NA	NA	Mmu.15143.1.S1_at_NA
Mmu.15145.1.A1_at	NA	NA	NA	NA	NA	NA	NA	NA	NA	Mmu.15145.1.A1_at_NA
Mmu.15145.1.A1_x_at	NA	NA	NA	NA	NA	NA	NA	NA	NA	Mmu.15145.1.A1_x_at_NA
Mmu.15162.1.S1_at	NA	NA	NA	NA	NA	NA	NA	NA	NA	Mmu.15162.1.S1_at_NA
Mmu.1520.2.S1_at	NA	NA	NA	NA	NA	NA	NA	NA	NA	Mmu.1520.2.S1_at_NA
Mmu.15219.1.S1_at	NA	NA	NA	NA	NA	NA	NA	NA	NA	Mmu.15219.1.S1_at_NA
Mmu.15231.1.S1_at	NA	NA	NA	NA	NA	NA	NA	NA	NA	Mmu.15231.1.S1_at_NA
Mmu.15242.1.S1_at	NA	NA	NA	NA	NA	NA	NA	NA	NA	Mmu.15242.1.S1_at_NA
Mmu.15247.11.S1_s_at	NA	NA	NA	NA	NA	NA	NA	NA	NA	Mmu.15247.11.S1_s_at_NA
Mmu.15247.13.S1_at	NA	NA	NA	NA	NA	NA	NA	NA	NA	Mmu.15247.13.S1_at_NA

Mmu.15247.13.S1_x_at	NA	NA	NA	NA	NA	NA	NA	NA	NA	Mmu.15247.13.S1_x_at_NA
Mmu.15247.15.S1_x_at	NA	NA	NA	NA	NA	NA	NA	NA	NA	Mmu.15247.15.S1_x_at_NA
Mmu.15247.16.S1_x_at	NA	NA	NA	NA	NA	NA	NA	NA	NA	Mmu.15247.16.S1_x_at_NA
Mmu.15247.18.S1_at	NA	NA	NA	NA	NA	NA	NA	NA	NA	Mmu.15247.18.S1_at_NA
Mmu.15247.1.S1_s_at	NA	NA	NA	NA	NA	NA	NA	NA	NA	Mmu.15247.1.S1_s_at_NA
Mmu.15247.21.S1_x_at	NA	NA	NA	NA	NA	NA	NA	NA	NA	Mmu.15247.21.S1_x_at_NA
Mmu.15247.23.S1_x_at	NA	NA	NA	NA	NA	NA	NA	NA	NA	Mmu.15247.23.S1_x_at_NA
Mmu.15247.25.S1_x_at	NA	NA	NA	NA	NA	NA	NA	NA	NA	Mmu.15247.25.S1_x_at_NA
Mmu.15247.26.S1_x_at	NA	NA	NA	NA	NA	NA	NA	NA	NA	Mmu.15247.26.S1_x_at_NA
Mmu.15247.33.S1_at	NA	NA	NA	NA	NA	NA	NA	NA	NA	Mmu.15247.33.S1_at_NA
Mmu.15247.37.S1_s_at	NA	NA	NA	NA	NA	NA	NA	NA	NA	Mmu.15247.37.S1_s_at_NA
Mmu.15247.37.S1_x_at	NA	NA	NA	NA	NA	NA	NA	NA	NA	Mmu.15247.37.S1_x_at_NA
Mmu.15247.3.S1_x_at	NA	NA	NA	NA	NA	NA	NA	NA	NA	Mmu.15247.3.S1_x_at_NA
Mmu.15247.40.S1_at	NA	NA	NA	NA	NA	NA	NA	NA	NA	Mmu.15247.40.S1_at_NA
Mmu.15247.42.S1_s_at	NA	NA	NA	NA	NA	NA	NA	NA	NA	Mmu.15247.42.S1_s_at_NA
Mmu.15247.43.S1_at	NA	NA	NA	NA	NA	NA	NA	NA	NA	Mmu.15247.43.S1_at_NA
Mmu.15247.44.S1_x_at	NA	NA	NA	NA	NA	NA	NA	NA	NA	Mmu.15247.44.S1_x_at_NA
Mmu.15247.45.S1_x_at	NA	NA	NA	NA	NA	NA	NA	NA	NA	Mmu.15247.45.S1_x_at_NA
Mmu.15247.7.S1_s_at	NA	NA	NA	NA	NA	NA	NA	NA	NA	Mmu.15247.7.S1_s_at_NA
Mmu.15247.8.S1_x_at	NA	NA	NA	NA	NA	NA	NA	NA	NA	Mmu.15247.8.S1_x_at_NA
Mmu.15249.2.S1_at	NA	NA	NA	NA	NA	NA	NA	NA	NA	Mmu.15249.2.S1_at_NA
Mmu.1526.1.S1_a_at	211816_x_at	FCAR	Fc fragment of IgA, receptor for	chr19q13.2-q13.4	2204	FCAR	Fc fragment of IgA, receptor for	---	664734	Mmu.1526.1.S1_a_at_FCAR
Mmu.1527.1.S1_at	NA	NA	NA	NA	NA	NA	NA	NA	NA	Mmu.1527.1.S1_at_NA
Mmu.15276.1.S1_s_at	NA	NA	NA	NA	NA	NA	NA	NA	NA	Mmu.15276.1.S1_s_at_NA
Mmu.15280.1.S1_at	NA	NA	NA	NA	NA	NA	NA	NA	NA	Mmu.15280.1.S1_at_NA
Mmu.15281.1.S1_at	NA	NA	NA	NA	NA	NA	NA	NA	NA	Mmu.15281.1.S1_at_NA
Mmu.15288.1.S1_at	NA	NA	NA	NA	NA	NA	NA	NA	NA	Mmu.15288.1.S1_at_NA
Mmu.15288.1.S1_s_at	NA	NA	NA	NA	NA	NA	NA	NA	NA	Mmu.15288.1.S1_s_at_NA
Mmu.1530.1.S1_at	NA	NA	NA	NA	NA	NA	NA	NA	NA	Mmu.1530.1.S1_at_NA
Mmu.15331.1.S1_at	NA	NA	NA	NA	NA	NA	NA	NA	NA	Mmu.15331.1.S1_at_NA
Mmu.15347.1.S1_s_at	213875_x_at	C6orf62	chromosome 6 open reading frame 62	chr6p22.2	81688	C4H6orf62	similar to chromosome 6 open reading frame 62	---	708218	Mmu.15347.1.S1_s_at_C6orf62
Mmu.15352.1.S1_at	NA	NA	NA	NA	NA	NA	NA	NA	NA	Mmu.15352.1.S1_at_NA
Mmu.15359.1.S1_s_at	NA	NA	NA	NA	NA	NA	NA	NA	NA	Mmu.15359.1.S1_s_at_NA
Mmu.1538.1.S1_at	NA	NA	NA	NA	NA	NA	NA	NA	NA	Mmu.1538.1.S1_at_NA
Mmu.15402.10.S1_s_at	NA	NA	NA	NA	NA	NA	NA	NA	NA	Mmu.15402.10.S1_s_at_NA
Mmu.15402.11.S1_at	NA	NA	NA	NA	NA	NA	NA	NA	NA	Mmu.15402.11.S1_at_NA
Mmu.15402.1.A1_at	1553570_x_at	COX2 /// ZFYVE20	cytochrome c oxidase II /// zinc finger, FYVE domain containing 20	chr3p24.3	4513 /// 64145	---	---	---	---	Mmu.15402.1.A1_at_COX2 /// ZFYVE2
Mmu.15433.1.S1_at	NA	NA	NA	NA	NA	NA	NA	NA	NA	Mmu.15433.1.S1_at_NA
Mmu.15440.1.S1_at	NA	NA	NA	NA	NA	NA	NA	NA	NA	Mmu.15440.1.S1_at_NA
Mmu.15443.1.S1_x_at	NA	NA	NA	NA	NA	NA	NA	NA	NA	Mmu.15443.1.S1_x_at_NA
Mmu.15490.1.A1_at	NA	NA	NA	NA	NA	NA	NA	NA	NA	Mmu.15490.1.A1_at_NA
Mmu.15491.1.S1_s_at	NA	NA	NA	NA	NA	NA	NA	NA	NA	Mmu.15491.1.S1_s_at_NA
Mmu.15494.1.S1_at	226334_s_at	AHSA2	AHA1, activator of heat shock 90kDa protein ATPase homolog 2 (yeast)	chr2p15	130872	LOC719540	similar to RIKEN cDNA 1110064P04	---	719540	Mmu.15494.1.S1_at_AHSA2
Mmu.15499.1.S1_s_at	222808_at	ALG13	asparagine-linked glycosylation 13 homolog (S. cerevisiae)	chrXq23	79868	LOC706863	similar to glycosyltransferase 28 domain containing 1	---	706863	Mmu.15499.1.S1_s_at_ALG13
Mmu.15501.1.S1_s_at	NA	NA	NA	NA	NA	NA	NA	NA	NA	Mmu.15501.1.S1_s_at_NA
Mmu.15507.1.A1_at	NA	NA	NA	NA	NA	NA	NA	NA	NA	Mmu.15507.1.A1_at_NA
Mmu.15507.1.S1_x_at	NA	NA	NA	NA	NA	NA	NA	NA	NA	Mmu.15507.1.S1_x_at_NA
Mmu.15516.1.S1_at	NA	NA	NA	NA	NA	NA	NA	NA	NA	Mmu.15516.1.S1_at_NA
Mmu.15516.1.S1_x_at	NA	NA	NA	NA	NA	NA	NA	NA	NA	Mmu.15516.1.S1_x_at_NA
Mmu.15555.1.S1_at	NA	NA	NA	NA	NA	NA	NA	NA	NA	Mmu.15555.1.S1_at_NA
Mmu.15572.1.S1_at	NA	NA	NA	NA	NA	NA	NA	NA	NA	Mmu.15572.1.S1_at_NA
Mmu.15592.2.S1_at	205078_at	PIGF	phosphatidylinositol glycan anchor biosynthesis, class F	chr2p21-p16	5281	LOC714844	similar to phosphatidylinositol glycan, class F isoform 1	---	714844	Mmu.15592.2.S1_at_PIGF
Mmu.15601.1.S2_at	NA	NA	NA	NA	NA	NA	NA	NA	NA	Mmu.15601.1.S2_at_NA
Mmu.15601.1.S2_s_at	215125_s_at	UGT1A1 /// UGT1A10 /	UDP glucuronosyltransferase 1 family, polypeptide A1 /// UDP glucuronosyltransferase 1 family, polyp	chr2q37	54575 /// 54576 /// 545	ALPI	Intestinal alkaline phosphatase	---	721052	Mmu.15601.1.S2_s_at_UGT1A1 /// UGT
Mmu.15609.1.S1_at	NA	NA	NA	NA	NA	NA	NA	NA	NA	Mmu.15609.1.S1_at_NA
Mmu.15616.1.S1_at	NA	NA	NA	NA	NA	NA	NA	NA	NA	Mmu.15616.1.S1_at_NA
Mmu.15616.1.S1_x_at	NA	NA	NA	NA	NA	NA	NA	NA	NA	Mmu.15616.1.S1_x_at_NA
Mmu.15620.1.S1_at	NA	NA	NA	NA	NA	NA	NA	NA	NA	Mmu.15620.1.S1_at_NA
Mmu.156.2.S1_at	NA	NA	NA	NA	NA	NA	NA	NA	NA	Mmu.156.2.S1_at_NA
Mmu.156.2.S1_x_at	NA	NA	NA	NA	NA	NA	NA	NA	NA	Mmu.156.2.S1_x_at_NA
Mmu.1563.1.S1_at	224054_at	---	---	---	---	ARMCX3	armadillo repeat containing, X-linked 3	---	704035	Mmu.1563.1.S1_at_---
Mmu.15662.1.S1_at	NA	NA	NA	NA	NA	NA	NA	NA	NA	Mmu.15662.1.S1_at_NA
Mmu.15690.2.S1_at	NA	NA	NA	NA	NA	NA	NA	NA	NA	Mmu.15690.2.S1_at_NA
Mmu.15700.2.S1_at	210979_at	ASAH1	N-acylsphingosine amidohydrolase (acid ceramidase) 1	chr8p22-p21.3	427	ASAH1	N-acylsphingosine amidohydrolase (acid ceramidase) 1	---	703699	Mmu.15700.2.S1_at_ASAH1
Mmu.15708.2.S1_at	NA	NA	NA	NA	NA	NA	NA	NA	NA	Mmu.15708.2.S1_at_NA
Mmu.15723.1.S1_s_at	NA	NA	NA	NA	NA	NA	NA	NA	NA	Mmu.15723.1.S1_s_at_NA
Mmu.15723.2.S1_at	NA	NA	NA	NA	NA	NA	NA	NA	NA	Mmu.15723.2.S1_at_NA
Mmu.15730.2.S1_at	NA	NA	NA	NA	NA	NA	NA	NA	NA	Mmu.15730.2.S1_at_NA
Mmu.15738.1.S1_at	NA	NA	NA	NA	NA	NA	NA	NA	NA	Mmu.15738.1.S1_at_NA
Mmu.15748.1.S1_s_at	NA	NA	NA	NA	NA	NA	NA	NA	NA	Mmu.15748.1.S1_s_at_NA
Mmu.15752.2.S1_at	NA	NA	NA	NA	NA	NA	NA	NA	NA	Mmu.15752.2.S1_at_NA
Mmu.15755.1.S1_at	NA	NA	NA	NA	NA	NA	NA	NA	NA	Mmu.15755.1.S1_at_NA
Mmu.15770.1.S1_at	NA	NA	NA	NA	NA	NA	NA	NA	NA	Mmu.15770.1.S1_at_NA
Mmu.15774.1.S1_at	NA	NA	NA	NA	NA	NA	NA	NA	NA	Mmu.15774.1.S1_at_NA
Mmu.15814.5.S1_at	NA	NA	NA	NA	NA	NA	NA	NA	NA	Mmu.15814.5.S1_at_NA
Mmu.15827.1.S1_at	NA	NA	NA	NA	NA	NA	NA	NA	NA	Mmu.15827.1.S1_at_NA
Mmu.15835.1.S1_at	NA	NA	NA	NA	NA	NA	NA	NA	NA	Mmu.15835.1.S1_at_NA
Mmu.15843.1.S1_at	NA	NA	NA	NA	NA	NA	NA	NA	NA	Mmu.15843.1.S1_at_NA
Mmu.15848.1.S1_at	NA	NA	NA	NA	NA	NA	NA	NA	NA	Mmu.15848.1.S1_at_NA
Mmu.15849.1.S1_a_at	NA	NA	NA	NA	NA	NA	NA	NA	NA	Mmu.15849.1.S1_a_at_NA
Mmu.15849.1.S1_at	NA	NA	NA	NA	NA	NA	NA	NA	NA	Mmu.15849.1.S1_at_NA
Mmu.15849.2.S1_at	NA	NA	NA	NA	NA	NA	NA	NA	NA	Mmu.15849.2.S1_at_NA
Mmu.15852.1.S1_s_at	NA	NA	NA	NA	NA	NA	NA	NA	NA	Mmu.15852.1.S1_s_at_NA
Mmu.15853.1.S1_s_at	NA	NA	NA	NA	NA	NA	NA	NA	NA	Mmu.15853.1.S1_s_at_NA
Mmu.15853.1.S1_x_at	NA	NA	NA	NA	NA	NA	NA	NA	NA	Mmu.15853.1.S1_x_at_NA
Mmu.15859.1.S1_at	204050_s_at	CLTA	clathrin, light chain (Lca)	chr9p13	1211	CLTA	clathrin, light chain (Lca)	---	693659	Mmu.15859.1.S1_at_CLTA
Mmu.1586.1.S1_at	NA	NA	NA	NA	NA	NA	NA	NA	NA	Mmu.1586.1.S1_at_NA
Mmu.15886.1.S1_s_at	NA	NA	NA	NA	NA	NA	NA	NA	NA	Mmu.15886.1.S1_s_at_NA
Mmu.15890.1.S1_s_at	227971_at	NRK	Nik related kinase	chrXq22.3	203447	---	---	---	---	Mmu.15890.1.S1_s_at_NRK
Mmu.15894.3.S1_at	NA	NA	NA	NA	NA	NA	NA	NA	NA	Mmu.15894.3.S1_at_NA
Mmu.15897.1.S1_at	NA	NA	NA	NA	NA	NA	NA	NA	NA	Mmu.15897.1.S1_at_NA
Mmu.15902.2.S1_at	NA	NA	NA	NA	NA	NA	NA	NA	NA	Mmu.15902.2.S1_at_NA
Mmu.15921.1.S1_at	NA	NA	NA	NA	NA	NA	NA	NA	NA	Mmu.15921.1.S1_at_NA
Mmu.15931.1.S1_at	NA	NA	NA	NA	NA	NA	NA	NA	NA	Mmu.15931.1.S1_at_NA
Mmu.15937.1.S1_at	NA	NA	NA	NA	NA	NA	NA	NA	NA	Mmu.15937.1.S1_at_NA
Mmu.15939.2.S1_at	NA	NA	NA	NA	NA	NA	NA	NA	NA	Mmu.15939.2.S1_at_NA
Mmu.15951.1.A1_at	NA	NA	NA	NA	NA	NA	NA	NA	NA	Mmu.15951.1.A1_at_NA
Mmu.15951.1.S1_at	NA	NA	NA	NA	NA	NA	NA	NA	NA	Mmu.15951.1.S1_at_NA
Mmu.1595.1.S1_at	NA	NA	NA	NA	NA	NA	NA	NA	NA	Mmu.1595.1.S1_at_NA
Mmu.15952.1.A1_at	NA	NA	NA	NA	NA	NA	NA	NA	NA	Mmu.15952.1.A1_at_NA
Mmu.15952.1.S1_at	NA	NA	NA	NA	NA	NA	NA	NA	NA	Mmu.15952.1.S1_at_NA
Mmu.15953.1.A1_at	NA	NA	NA	NA	NA	NA	NA	NA	NA	Mmu.15953.1.A1_at_NA
Mmu.15953.1.S1_at	NA	NA	NA	NA	NA	NA	NA	NA	NA	Mmu.15953.1.S1_at_NA
Mmu.15956.1.A1_at	NA	NA	NA	NA	NA	NA	NA	NA	NA	Mmu.15956.1.A1_at_NA
Mmu.15956.1.S1_at	NA	NA	NA	NA	NA	NA	NA	NA	NA	Mmu.15956.1.S1_at_NA
Mmu.15966.1.S1_at	NA	NA	NA	NA	NA	NA	NA	NA	NA	Mmu.15966.1.S1_at_NA
Mmu.15973.14.S1_at	NA	NA	NA	NA	NA	NA	NA	NA	NA	Mmu.15973.14.S1_at_NA
Mmu.15973.16.S1_at	NA	NA	NA	NA	NA	NA	NA	NA	NA	Mmu.15973.16.S1_at_NA

Mmu.15973.16.S1_x_at	NA	NA	NA	NA	NA	NA	NA	NA	NA	Mmu.15973.16.S1_x_at_NA
Mmu.15973.17.S1_x_at	211739_x_at	CSH1	chorionic somatomammotropin hormone 1 (placental lactogen)	chr17q24.2	1442	CSH-3 /// CSH-4 /// GH-2	chorionic somatommamotropin hormone 3 /// chorionic somatommamotropin hormone 4 /// growth ho	---	700885 /// 718116 /// 718	Mmu.15973.17.S1_x_at_CSH1
Mmu.15973.19.S1_at	NA	NA	NA	NA	NA	NA	NA	NA	NA	Mmu.15973.19.S1_at_NA
Mmu.15973.19.S1_x_at	NA	NA	NA	NA	NA	NA	NA	NA	NA	Mmu.15973.19.S1_x_at_NA
Mmu.15973.1.S1_a_at	NA	NA	NA	NA	NA	NA	NA	NA	NA	Mmu.15973.1.S1_a_at_NA
Mmu.15973.2.S1_x_at	NA	NA	NA	NA	NA	NA	NA	NA	NA	Mmu.15973.2.S1_x_at_NA
Mmu.15973.50.S1_x_at	NA	NA	NA	NA	NA	NA	NA	NA	NA	Mmu.15973.50.S1_x_at_NA
Mmu.15976.1.S1_at	NA	NA	NA	NA	NA	NA	NA	NA	NA	Mmu.15976.1.S1_at_NA
Mmu.15983.1.S1_at	NA	NA	NA	NA	NA	NA	NA	NA	NA	Mmu.15983.1.S1_at_NA
Mmu.15987.2.S1_at	NA	NA	NA	NA	NA	NA	NA	NA	NA	Mmu.15987.2.S1_at_NA
Mmu.15993.1.S1_s_at	NA	NA	NA	NA	NA	NA	NA	NA	NA	Mmu.15993.1.S1_s_at_NA
Mmu.15999.1.S1_at	NA	NA	NA	NA	NA	NA	NA	NA	NA	Mmu.15999.1.S1_at_NA
Mmu.16001.1.S1_at	1554679_a_at	LAPTM4B	lysosomal protein transmembrane 4 beta	chr8q22.1	55353	LYAR	Ly1 antibody reactive homolog (mouse)	---	712499	Mmu.16001.1.S1_at_LAPTM4B
Mmu.16022.1.S1_at	NA	NA	NA	NA	NA	NA	NA	NA	NA	Mmu.16022.1.S1_at_NA
Mmu.16023.1.S1_at	NA	NA	NA	NA	NA	NA	NA	NA	NA	Mmu.16023.1.S1_at_NA
Mmu.16024.1.S1_at	NA	NA	NA	NA	NA	NA	NA	NA	NA	Mmu.16024.1.S1_at_NA
Mmu.16030.2.S1_a_at	NA	NA	NA	NA	NA	NA	NA	NA	NA	Mmu.16030.2.S1_a_at_NA
Mmu.16043.1.S1_at	NA	NA	NA	NA	NA	NA	NA	NA	NA	Mmu.16043.1.S1_at_NA
Mmu.1605.1.S1_at	NA	NA	NA	NA	NA	NA	NA	NA	NA	Mmu.1605.1.S1_at_NA
Mmu.16061.1.S1_at	NA	NA	NA	NA	NA	NA	NA	NA	NA	Mmu.16061.1.S1_at_NA
Mmu.16078.1.S1_at	NA	NA	NA	NA	NA	NA	NA	NA	NA	Mmu.16078.1.S1_at_NA
Mmu.16091.1.A1_at	NA	NA	NA	NA	NA	NA	NA	NA	NA	Mmu.16091.1.A1_at_NA
Mmu.16091.1.S1_at	NA	NA	NA	NA	NA	NA	NA	NA	NA	Mmu.16091.1.S1_at_NA
Mmu.16093.1.S1_at	NA	NA	NA	NA	NA	NA	NA	NA	NA	Mmu.16093.1.S1_at_NA
Mmu.16103.1.S1_s_at	211995_x_at	ACTG1	actin, gamma 1	chr17q25	71	LOC706459 /// LOC7105	similar to cytoplasmic beta-actin /// similar to Actin, cytoplasmic 2 (Gamma-actin) /// similar to Actin, c	---	706459 /// 710533 /// 713	Mmu.16103.1.S1_s_at_ACTG1
Mmu.16106.1.S1_at	205480_s_at	UGP2	UDP-glucose pyrophosphorylase 2	chr2p14-p13	7360	UGP2	UDP-glucose pyrophosphorylase 2	---	695178	Mmu.16106.1.S1_at_UGP2
Mmu.16109.1.S1_at	NA	NA	NA	NA	NA	NA	NA	NA	NA	Mmu.16109.1.S1_at_NA
Mmu.16110.1.S1_s_at	NA	NA	NA	NA	NA	NA	NA	NA	NA	Mmu.16110.1.S1_s_at_NA
Mmu.16117.1.S1_at	210551_s_at	ASMT	acetylserotonin O-methyltransferase	chrXp22.3 or Yp11.3	438	HIOMT	hydroxyindole O-methyltransferase	---	574350	Mmu.16117.1.S1_at_ASMT
Mmu.16121.1.S1_at	NA	NA	NA	NA	NA	NA	NA	NA	NA	Mmu.16121.1.S1_at_NA
Mmu.16121.1.S1_s_at	NA	NA	NA	NA	NA	NA	NA	NA	NA	Mmu.16121.1.S1_s_at_NA
Mmu.1612.1.S1_at	NA	NA	NA	NA	NA	NA	NA	NA	NA	Mmu.1612.1.S1_at_NA
Mmu.16123.2.S1_at	NA	NA	NA	NA	NA	NA	NA	NA	NA	Mmu.16123.2.S1_at_NA
Mmu.16124.1.S1_at	NA	NA	NA	NA	NA	NA	NA	NA	NA	Mmu.16124.1.S1_at_NA
Mmu.16174.1.S1_at	204427_s_at	TMED2	transmembrane emp24 domain trafficking protein 2	chr12q24.31	10959	LOC700505	Hypothetical protein LOC700505	---	700505	Mmu.16174.1.S1_at_TMED2
Mmu.16174.1.S1_x_at	NA	NA	NA	NA	NA	NA	NA	NA	NA	Mmu.16174.1.S1_x_at_NA
Mmu.16175.1.S1_at	NA	NA	NA	NA	NA	NA	NA	NA	NA	Mmu.16175.1.S1_at_NA
Mmu.16205.1.S1_at	NA	NA	NA	NA	NA	NA	NA	NA	NA	Mmu.16205.1.S1_at_NA
Mmu.16211.1.S1_at	NA	NA	NA	NA	NA	NA	NA	NA	NA	Mmu.16211.1.S1_at_NA
Mmu.16215.1.S1_at	NA	NA	NA	NA	NA	NA	NA	NA	NA	Mmu.16215.1.S1_at_NA
Mmu.16226.1.S1_at	NA	NA	NA	NA	NA	NA	NA	NA	NA	Mmu.16226.1.S1_at_NA
Mmu.16226.1.S1_x_at	NA	NA	NA	NA	NA	NA	NA	NA	NA	Mmu.16226.1.S1_x_at_NA
Mmu.16227.2.S1_at	NA	NA	NA	NA	NA	NA	NA	NA	NA	Mmu.16227.2.S1_at_NA
Mmu.16227.3.S1_at	NA	NA	NA	NA	NA	NA	NA	NA	NA	Mmu.16227.3.S1_at_NA
Mmu.16235.1.S1_at	NA	NA	NA	NA	NA	NA	NA	NA	NA	Mmu.16235.1.S1_at_NA
Mmu.16242.1.S1_at	NA	NA	NA	NA	NA	NA	NA	NA	NA	Mmu.16242.1.S1_at_NA
Mmu.16242.1.S1_s_at	NA	NA	NA	NA	NA	NA	NA	NA	NA	Mmu.16242.1.S1_s_at_NA
Mmu.16247.1.S1_at	NA	NA	NA	NA	NA	NA	NA	NA	NA	Mmu.16247.1.S1_at_NA
Mmu.1625.1.S1_at	NA	NA	NA	NA	NA	NA	NA	NA	NA	Mmu.1625.1.S1_at_NA
Mmu.16255.1.S1_at	225638_at	C1orf31	chromosome 1 open reading frame 31	chr1q42.2	388753	LOC718775	similar to Molybdenum cofactor synthesis protein cinnamon	---	718775	Mmu.16255.1.S1_at_C1orf31
Mmu.16260.1.S1_at	NA	NA	NA	NA	NA	NA	NA	NA	NA	Mmu.16260.1.S1_at_NA
Mmu.16261.1.S1_at	NA	NA	NA	NA	NA	NA	NA	NA	NA	Mmu.16261.1.S1_at_NA
Mmu.16278.1.S1_at	NA	NA	NA	NA	NA	NA	NA	NA	NA	Mmu.16278.1.S1_at_NA
Mmu.16285.1.A1_at	NA	NA	NA	NA	NA	NA	NA	NA	NA	Mmu.16285.1.A1_at_NA
Mmu.16290.1.S1_at	NA	NA	NA	NA	NA	NA	NA	NA	NA	Mmu.16290.1.S1_at_NA
Mmu.16292.1.S1_at	NA	NA	NA	NA	NA	NA	NA	NA	NA	Mmu.16292.1.S1_at_NA
Mmu.16297.1.S1_s_at	NA	NA	NA	NA	NA	NA	NA	NA	NA	Mmu.16297.1.S1_s_at_NA
Mmu.16299.1.S1_at	NA	NA	NA	NA	NA	NA	NA	NA	NA	Mmu.16299.1.S1_at_NA
Mmu.16305.1.S1_at	NA	NA	NA	NA	NA	NA	NA	NA	NA	Mmu.16305.1.S1_at_NA
Mmu.16311.1.S1_at	NA	NA	NA	NA	NA	NA	NA	NA	NA	Mmu.16311.1.S1_at_NA
Mmu.16317.1.S1_at	NA	NA	NA	NA	NA	NA	NA	NA	NA	Mmu.16317.1.S1_at_NA
Mmu.16324.1.S1_at	NA	NA	NA	NA	NA	NA	NA	NA	NA	Mmu.16324.1.S1_at_NA
Mmu.16330.1.S1_at	NA	NA	NA	NA	NA	NA	NA	NA	NA	Mmu.16330.1.S1_at_NA
Mmu.16335.1.S1_at	NA	NA	NA	NA	NA	NA	NA	NA	NA	Mmu.16335.1.S1_at_NA
Mmu.16349.1.S1_at	NA	NA	NA	NA	NA	NA	NA	NA	NA	Mmu.16349.1.S1_at_NA
Mmu.16352.2.S1_at	NA	NA	NA	NA	NA	NA	NA	NA	NA	Mmu.16352.2.S1_at_NA
Mmu.16352.3.S1_at	NA	NA	NA	NA	NA	NA	NA	NA	NA	Mmu.16352.3.S1_at_NA
Mmu.16358.1.S1_at	NA	NA	NA	NA	NA	NA	NA	NA	NA	Mmu.16358.1.S1_at_NA
Mmu.16365.1.S1_at	211090_s_at	PRPF4B	PRP4 pre-mRNA processing factor 4 homolog B (yeast)	chr6p25.2	8899	LOC710193	similar to PRP4 pre-mRNA processing factor 4 homolog B (yeast) (predicted)	---	710193	Mmu.16365.1.S1_at_PRPF4B
Mmu.16379.1.S1_at	NA	NA	NA	NA	NA	NA	NA	NA	NA	Mmu.16379.1.S1_at_NA
Mmu.16380.2.S1_at	NA	NA	NA	NA	NA	NA	NA	NA	NA	Mmu.16380.2.S1_at_NA
Mmu.16385.1.S1_a_at	NA	NA	NA	NA	NA	NA	NA	NA	NA	Mmu.16385.1.S1_a_at_NA
Mmu.16385.1.S1_at	NA	NA	NA	NA	NA	NA	NA	NA	NA	Mmu.16385.1.S1_at_NA
Mmu.16385.2.S1_at	NA	NA	NA	NA	NA	NA	NA	NA	NA	Mmu.16385.2.S1_at_NA
Mmu.16390.2.S1_at	NA	NA	NA	NA	NA	NA	NA	NA	NA	Mmu.16390.2.S1_at_NA
Mmu.16391.1.S1_at	NA	NA	NA	NA	NA	NA	NA	NA	NA	Mmu.16391.1.S1_at_NA
Mmu.1639.1.S1_at	NA	NA	NA	NA	NA	NA	NA	NA	NA	Mmu.1639.1.S1_at_NA
Mmu.16393.1.S1_at	NA	NA	NA	NA	NA	NA	NA	NA	NA	Mmu.16393.1.S1_at_NA
Mmu.16400.1.S1_at	NA	NA	NA	NA	NA	NA	NA	NA	NA	Mmu.16400.1.S1_at_NA
Mmu.16411.1.S1_at	NA	NA	NA	NA	NA	NA	NA	NA	NA	Mmu.16411.1.S1_at_NA
Mmu.16415.2.S1_at	NA	NA	NA	NA	NA	NA	NA	NA	NA	Mmu.16415.2.S1_at_NA
Mmu.16418.1.A1_at	NA	NA	NA	NA	NA	NA	NA	NA	NA	Mmu.16418.1.A1_at_NA
Mmu.16421.1.S1_at	NA	NA	NA	NA	NA	NA	NA	NA	NA	Mmu.16421.1.S1_at_NA
Mmu.16432.1.S1_at	NA	NA	NA	NA	NA	NA	NA	NA	NA	Mmu.16432.1.S1_at_NA
Mmu.16433.2.S1_at	NA	NA	NA	NA	NA	NA	NA	NA	NA	Mmu.16433.2.S1_at_NA
Mmu.16444.1.S1_at	NA	NA	NA	NA	NA	NA	NA	NA	NA	Mmu.16444.1.S1_at_NA
Mmu.16444.2.S1_a_at	NA	NA	NA	NA	NA	NA	NA	NA	NA	Mmu.16444.2.S1_a_at_NA
Mmu.16445.1.S1_at	NA	NA	NA	NA	NA	NA	NA	NA	NA	Mmu.16445.1.S1_at_NA
Mmu.16454.1.S1_at	208851_s_at	THY1	Thy-1 cell surface antigen	chr11q22.3-q23	7070	THY1	Thy-1 cell surface antigen	---	705321	Mmu.16454.1.S1_at_THY1
Mmu.16454.1.S2_at	NA	NA	NA	NA	NA	NA	NA	NA	NA	Mmu.16454.1.S2_at_NA
Mmu.16465.1.S1_at	NA	NA	NA	NA	NA	NA	NA	NA	NA	Mmu.16465.1.S1_at_NA
Mmu.16468.1.S1_at	NA	NA	NA	NA	NA	NA	NA	NA	NA	Mmu.16468.1.S1_at_NA
Mmu.16475.1.S1_at	202190_at	CSTF1	cleavage stimulation factor, 3' pre-RNA, subunit 1, 50kDa	chr20q13.31	1477	PX19	Similar to Preli	---	698007	Mmu.16475.1.S1_at_CSTF1
Mmu.16489.1.S1_at	NA	NA	NA	NA	NA	NA	NA	NA	NA	Mmu.16489.1.S1_at_NA
Mmu.16497.1.S1_at	NA	NA	NA	NA	NA	NA	NA	NA	NA	Mmu.16497.1.S1_at_NA
Mmu.16501.1.S1_at	NA	NA	NA	NA	NA	NA	NA	NA	NA	Mmu.16501.1.S1_at_NA
Mmu.16504.1.S1_at	NA	NA	NA	NA	NA	NA	NA	NA	NA	Mmu.16504.1.S1_at_NA
Mmu.16530.1.S1_at	NA	NA	NA	NA	NA	NA	NA	NA	NA	Mmu.16530.1.S1_at_NA
Mmu.1653.1.S1_at	NA	NA	NA	NA	NA	NA	NA	NA	NA	Mmu.1653.1.S1_at_NA
Mmu.16536.10.S1_at	NA	NA	NA	NA	NA	NA	NA	NA	NA	Mmu.16536.10.S1_at_NA
Mmu.16547.1.S1_at	NA	NA	NA	NA	NA	NA	NA	NA	NA	Mmu.16547.1.S1_at_NA
Mmu.16551.1.S1_at	NA	NA	NA	NA	NA	NA	NA	NA	NA	Mmu.16551.1.S1_at_NA
Mmu.16556.1.S1_at	NA	NA	NA	NA	NA	NA	NA	NA	NA	Mmu.16556.1.S1_at_NA
Mmu.1671.1.S1_at	NA	NA	NA	NA	NA	NA	NA	NA	NA	Mmu.1671.1.S1_at_NA
Mmu.1700.1.S1_x_at	NA	NA	NA	NA	NA	NA	NA	NA	NA	Mmu.1700.1.S1_x_at_NA
Mmu.1716.2.S1_at	NA	NA	NA	NA	NA	NA	NA	NA	NA	Mmu.1716.2.S1_at_NA
Mmu.1719.1.S1_at	NA	NA	NA	NA	NA	NA	NA	NA	NA	Mmu.1719.1.S1_at_NA
Mmu.1726.1.S1_at	NA	NA	NA	NA	NA	NA	NA	NA	NA	Mmu.1726.1.S1_at_NA

Mmu.1730.1.S1_at	NA	NA	NA	NA	NA	NA	NA	NA	NA	Mmu.1730.1.S1_at_NA
Mmu.1734.1.S1_at	NA	NA	NA	NA	NA	NA	NA	NA	NA	Mmu.1734.1.S1_at_NA
Mmu.1738.2.S1_at	NA	NA	NA	NA	NA	NA	NA	NA	NA	Mmu.1738.2.S1_at_NA
Mmu.1748.1.A1_at	NA	NA	NA	NA	NA	NA	NA	NA	NA	Mmu.1748.1.A1_at_NA
Mmu.1748.1.S1_at	NA	NA	NA	NA	NA	NA	NA	NA	NA	Mmu.1748.1.S1_at_NA
Mmu.175.1.A1_at	NA	NA	NA	NA	NA	NA	NA	NA	NA	Mmu.175.1.A1_at_NA
Mmu.175.1.A1_x_at	NA	NA	NA	NA	NA	NA	NA	NA	NA	Mmu.175.1.A1_x_at_NA
Mmu.175.1.S1_at	NA	NA	NA	NA	NA	NA	NA	NA	NA	Mmu.175.1.S1_at_NA
Mmu.1757.2.S1_at	NA	NA	NA	NA	NA	NA	NA	NA	NA	Mmu.1757.2.S1_at_NA
Mmu.1773.1.S1_at	NA	NA	NA	NA	NA	NA	NA	NA	NA	Mmu.1773.1.S1_at_NA
Mmu.1775.1.S1_at	NA	NA	NA	NA	NA	NA	NA	NA	NA	Mmu.1775.1.S1_at_NA
Mmu.181.1.S1_at	209927_s_at	C1orf77	chromosome 1 open reading frame 77	chr1q21.3	26097	LOC715501	similar to Protein C1orf77 homolog	---	715501	Mmu.181.1.S1_at_C1orf77
Mmu.1815.1.S1_at	NA	NA	NA	NA	NA	NA	NA	NA	NA	Mmu.1815.1.S1_at_NA
Mmu.1817.1.S1_at	NA	NA	NA	NA	NA	NA	NA	NA	NA	Mmu.1817.1.S1_at_NA
Mmu.1818.1.S1_at	NA	NA	NA	NA	NA	NA	NA	NA	NA	Mmu.1818.1.S1_at_NA
Mmu.1819.1.S1_at	NA	NA	NA	NA	NA	NA	NA	NA	NA	Mmu.1819.1.S1_at_NA
Mmu.1836.1.S1_at	NA	NA	NA	NA	NA	NA	NA	NA	NA	Mmu.1836.1.S1_at_NA
Mmu.1837.2.S1_at	NA	NA	NA	NA	NA	NA	NA	NA	NA	Mmu.1837.2.S1_at_NA
Mmu.1854.1.S1_at	215450_at	---	---	---	---	LOC701418	similar to small nuclear ribonucleoprotein E	---	701418	Mmu.1854.1.S1_at_---
Mmu.1855.1.S1_at	NA	NA	NA	NA	NA	NA	NA	NA	NA	Mmu.1855.1.S1_at_NA
Mmu.1884.1.S1_at	NA	NA	NA	NA	NA	NA	NA	NA	NA	Mmu.1884.1.S1_at_NA
Mmu.1900.1.S1_s_at	NA	NA	NA	NA	NA	NA	NA	NA	NA	Mmu.1900.1.S1_s_at_NA
Mmu.190.1.S1_at	NA	NA	NA	NA	NA	NA	NA	NA	NA	Mmu.190.1.S1_at_NA
Mmu.190.2.S1_at	NA	NA	NA	NA	NA	NA	NA	NA	NA	Mmu.190.2.S1_at_NA
Mmu.1903.1.S1_at	200786_at	PSMB7	proteasome (prosome, macropain) subunit, beta type, 7	chr9q34.11-q34.12	5695	---	---	---	---	Mmu.1903.1.S1_at_PSMB7
Mmu.1906.10.S1_a_at	NA	NA	NA	NA	NA	NA	NA	NA	NA	Mmu.1906.10.S1_a_at_NA
Mmu.1906.10.S1_s_at	NA	NA	NA	NA	NA	NA	NA	NA	NA	Mmu.1906.10.S1_s_at_NA
Mmu.1906.11.S1_a_at	NA	NA	NA	NA	NA	NA	NA	NA	NA	Mmu.1906.11.S1_a_at_NA
Mmu.1906.11.S1_x_at	NA	NA	NA	NA	NA	NA	NA	NA	NA	Mmu.1906.11.S1_x_at_NA
Mmu.1906.13.S1_at	NA	NA	NA	NA	NA	NA	NA	NA	NA	Mmu.1906.13.S1_at_NA
Mmu.1906.3.S1_x_at	NA	NA	NA	NA	NA	NA	NA	NA	NA	Mmu.1906.3.S1_x_at_NA
Mmu.1906.5.S1_s_at	NA	NA	NA	NA	NA	NA	NA	NA	NA	Mmu.1906.5.S1_s_at_NA
Mmu.1906.7.S1_at	206785_s_at	KLRC1 /// KLRC2	killer cell lectin-like receptor subfamily C, member 1 /// killer cell lectin-like receptor subfamily C, mem	chr12p13	3821 /// 3822	KLRC3	killer cell lectin-like receptor subfamily C, member 3	---	696590	Mmu.1906.7.S1_at_KLRC1 /// KLRC2
Mmu.1906.9.S1_s_at	NA	NA	NA	NA	NA	NA	NA	NA	NA	Mmu.1906.9.S1_s_at_NA
Mmu.1907.1.S1_at	NA	NA	NA	NA	NA	NA	NA	NA	NA	Mmu.1907.1.S1_at_NA
Mmu.1914.1.S1_s_at	NA	NA	NA	NA	NA	NA	NA	NA	NA	Mmu.1914.1.S1_s_at_NA
Mmu.19.1.S1_at	NA	NA	NA	NA	NA	NA	NA	NA	NA	Mmu.19.1.S1_at_NA
Mmu.1942.1.S1_at	NA	NA	NA	NA	NA	NA	NA	NA	NA	Mmu.1942.1.S1_at_NA
Mmu.1942.1.S1_x_at	NA	NA	NA	NA	NA	NA	NA	NA	NA	Mmu.1942.1.S1_x_at_NA
Mmu.1943.1.S1_at	NA	NA	NA	NA	NA	NA	NA	NA	NA	Mmu.1943.1.S1_at_NA
Mmu.1946.1.S1_at	NA	NA	NA	NA	NA	NA	NA	NA	NA	Mmu.1946.1.S1_at_NA
Mmu.1956.1.S1_at	NA	NA	NA	NA	NA	NA	NA	NA	NA	Mmu.1956.1.S1_at_NA
Mmu.1976.1.A1_at	NA	NA	NA	NA	NA	NA	NA	NA	NA	Mmu.1976.1.A1_at_NA
Mmu.1976.1.S1_at	NA	NA	NA	NA	NA	NA	NA	NA	NA	Mmu.1976.1.S1_at_NA
Mmu.1995.1.S1_s_at	NA	NA	NA	NA	NA	NA	NA	NA	NA	Mmu.1995.1.S1_s_at_NA
Mmu.2010.1.S1_s_at	NA	NA	NA	NA	NA	NA	NA	NA	NA	Mmu.2010.1.S1_s_at_NA
Mmu.2013.1.S1_at	NA	NA	NA	NA	NA	NA	NA	NA	NA	Mmu.2013.1.S1_at_NA
Mmu.2018.1.S1_at	NA	NA	NA	NA	NA	NA	NA	NA	NA	Mmu.2018.1.S1_at_NA
Mmu.2031.1.S1_at	NA	NA	NA	NA	NA	NA	NA	NA	NA	Mmu.2031.1.S1_at_NA
Mmu.2033.2.S1_at	NA	NA	NA	NA	NA	NA	NA	NA	NA	Mmu.2033.2.S1_at_NA
Mmu.2043.1.S1_at	NA	NA	NA	NA	NA	NA	NA	NA	NA	Mmu.2043.1.S1_at_NA
Mmu.2046.1.S1_at	NA	NA	NA	NA	NA	NA	NA	NA	NA	Mmu.2046.1.S1_at_NA
Mmu.2048.1.S1_at	NA	NA	NA	NA	NA	NA	NA	NA	NA	Mmu.2048.1.S1_at_NA
Mmu.2050.1.S1_s_at	NA	NA	NA	NA	NA	NA	NA	NA	NA	Mmu.2050.1.S1_s_at_NA
Mmu.2053.1.S1_s_at	205114_s_at	CCL3 /// CCL3L1 /// CC	chemokine (C-C motif) ligand 3 /// chemokine (C-C motif) ligand 3-like 1 /// chemokine (C-C motif) lig	chr17q11-q21 /// chr17q21.1	414062 /// 6348 /// 634	CCL3	chemokine (C-C motif) ligand 3	---	619514	Mmu.2053.1.S1_s_at_CCL3 /// CCL3L1
Mmu.2056.1.S1_at	NA	NA	NA	NA	NA	NA	NA	NA	NA	Mmu.2056.1.S1_at_NA
Mmu.2057.1.S1_at	NA	NA	NA	NA	NA	NA	NA	NA	NA	Mmu.2057.1.S1_at_NA
Mmu.2057.1.S1_s_at	NA	NA	NA	NA	NA	NA	NA	NA	NA	Mmu.2057.1.S1_s_at_NA
Mmu.2059.1.S1_at	NA	NA	NA	NA	NA	NA	NA	NA	NA	Mmu.2059.1.S1_at_NA
Mmu.2066.1.A1_at	NA	NA	NA	NA	NA	NA	NA	NA	NA	Mmu.2066.1.A1_at_NA
Mmu.2074.1.S1_at	NA	NA	NA	NA	NA	NA	NA	NA	NA	Mmu.2074.1.S1_at_NA
Mmu.2083.1.S1_at	NA	NA	NA	NA	NA	NA	NA	NA	NA	Mmu.2083.1.S1_at_NA
Mmu.2088.1.S1_at	NA	NA	NA	NA	NA	NA	NA	NA	NA	Mmu.2088.1.S1_at_NA
Mmu.2091.1.S1_x_at	NA	NA	NA	NA	NA	NA	NA	NA	NA	Mmu.2091.1.S1_x_at_NA
Mmu.2091.3.S1_at	NA	NA	NA	NA	NA	NA	NA	NA	NA	Mmu.2091.3.S1_at_NA
Mmu.2091.3.S1_x_at	NA	NA	NA	NA	NA	NA	NA	NA	NA	Mmu.2091.3.S1_x_at_NA
Mmu.2091.5.S1_a_at	NA	NA	NA	NA	NA	NA	NA	NA	NA	Mmu.2091.5.S1_a_at_NA
Mmu.2091.5.S1_x_at	NA	NA	NA	NA	NA	NA	NA	NA	NA	Mmu.2091.5.S1_x_at_NA
Mmu.2104.1.S1_at	NA	NA	NA	NA	NA	NA	NA	NA	NA	Mmu.2104.1.S1_at_NA
Mmu.2107.1.S1_at	NA	NA	NA	NA	NA	NA	NA	NA	NA	Mmu.2107.1.S1_at_NA
Mmu.211.1.S1_at	225541_at	RPL22L1	ribosomal protein L22-like 1	chr3q26.2	200916	LOC695815	similar to ribosomal protein L22 like 1	---	695815	Mmu.211.1.S1_at_RPL22L1
Mmu.2114.1.A1_at	NA	NA	NA	NA	NA	NA	NA	NA	NA	Mmu.2114.1.A1_at_NA
Mmu.2114.1.S1_at	217323_at	HLA-DRB6	major histocompatibility complex, class II, DR beta 6 (pseudogene)	chr6p21.3	3128	---	---	---	---	Mmu.2114.1.S1_at_HLA-DRB6
Mmu.2120.1.A1_at	NA	NA	NA	NA	NA	NA	NA	NA	NA	Mmu.2120.1.A1_at_NA
Mmu.2120.1.S1_s_at	NA	NA	NA	NA	NA	NA	NA	NA	NA	Mmu.2120.1.S1_s_at_NA
Mmu.2126.1.S1_at	NA	NA	NA	NA	NA	NA	NA	NA	NA	Mmu.2126.1.S1_at_NA
Mmu.2126.1.S1_s_at	NA	NA	NA	NA	NA	NA	NA	NA	NA	Mmu.2126.1.S1_s_at_NA
Mmu.2128.1.S1_x_at	NA	NA	NA	NA	NA	NA	NA	NA	NA	Mmu.2128.1.S1_x_at_NA
Mmu.2136.1.S1_at	NA	NA	NA	NA	NA	NA	NA	NA	NA	Mmu.2136.1.S1_at_NA
Mmu.2142.1.S1_at	NA	NA	NA	NA	NA	NA	NA	NA	NA	Mmu.2142.1.S1_at_NA
Mmu.2143.1.S1_x_at	1553882_at	---	---	---	---	---	---	---	---	Mmu.2143.1.S1_x_at_---
Mmu.2174.1.S1_at	NA	NA	NA	NA	NA	NA	NA	NA	NA	Mmu.2174.1.S1_at_NA
Mmu.2177.1.S1_x_at	NA	NA	NA	NA	NA	NA	NA	NA	NA	Mmu.2177.1.S1_x_at_NA
Mmu.2196.1.A1_at	NA	NA	NA	NA	NA	NA	NA	NA	NA	Mmu.2196.1.A1_at_NA
Mmu.2196.1.S1_at	NA	NA	NA	NA	NA	NA	NA	NA	NA	Mmu.2196.1.S1_at_NA
Mmu.2224.1.A1_at	NA	NA	NA	NA	NA	NA	NA	NA	NA	Mmu.2224.1.A1_at_NA
Mmu.2226.2.S1_at	NA	NA	NA	NA	NA	NA	NA	NA	NA	Mmu.2226.2.S1_at_NA
Mmu.22.2.S1_at	NA	NA	NA	NA	NA	NA	NA	NA	NA	Mmu.22.2.S1_at_NA
Mmu.2231.1.S1_at	NA	NA	NA	NA	NA	NA	NA	NA	NA	Mmu.2231.1.S1_at_NA
Mmu.2238.1.S1_at	NA	NA	NA	NA	NA	NA	NA	NA	NA	Mmu.2238.1.S1_at_NA
Mmu.2240.1.A1_at	NA	NA	NA	NA	NA	NA	NA	NA	NA	Mmu.2240.1.A1_at_NA
Mmu.2243.1.S1_at	208756_at	EIF3I	eukaryotic translation initiation factor 3, subunit I	chr1p34.1	8668	EIF3I	eukaryotic translation initiation factor 3, subunit I	---	708145	Mmu.2243.1.S1_at_EIF3I
Mmu.2250.1.S1_at	NA	NA	NA	NA	NA	NA	NA	NA	NA	Mmu.2250.1.S1_at_NA
Mmu.225.1.S1_at	NA	NA	NA	NA	NA	NA	NA	NA	NA	Mmu.225.1.S1_at_NA
Mmu.2252.1.S1_at	NA	NA	NA	NA	NA	NA	NA	NA	NA	Mmu.2252.1.S1_at_NA
Mmu.2256.2.S1_at	NA	NA	NA	NA	NA	NA	NA	NA	NA	Mmu.2256.2.S1_at_NA
Mmu.2276.1.S1_at	NA	NA	NA	NA	NA	NA	NA	NA	NA	Mmu.2276.1.S1_at_NA
Mmu.2277.1.S1_at	NA	NA	NA	NA	NA	NA	NA	NA	NA	Mmu.2277.1.S1_at_NA
Mmu.2297.1.S1_at	NA	NA	NA	NA	NA	NA	NA	NA	NA	Mmu.2297.1.S1_at_NA
Mmu.2302.1.S1_at	NA	NA	NA	NA	NA	NA	NA	NA	NA	Mmu.2302.1.S1_at_NA
Mmu.2304.1.S1_at	NA	NA	NA	NA	NA	NA	NA	NA	NA	Mmu.2304.1.S1_at_NA
Mmu.2305.1.S1_at	NA	NA	NA	NA	NA	NA	NA	NA	NA	Mmu.2305.1.S1_at_NA
Mmu.2313.1.S1_at	NA	NA	NA	NA	NA	NA	NA	NA	NA	Mmu.2313.1.S1_at_NA
Mmu.2323.2.S1_at	NA	NA	NA	NA	NA	NA	NA	NA	NA	Mmu.2323.2.S1_at_NA
Mmu.2333.1.S1_at	NA	NA	NA	NA	NA	NA	NA	NA	NA	Mmu.2333.1.S1_at_NA
Mmu.234.1.A1_at	NA	NA	NA	NA	NA	NA	NA	NA	NA	Mmu.234.1.A1_at_NA
Mmu.2342.1.S1_at	NA	NA	NA	NA	NA	NA	NA	NA	NA	Mmu.2342.1.S1_at_NA
Mmu.2344.1.A1_at	NA	NA	NA	NA	NA	NA	NA	NA	NA	Mmu.2344.1.A1_at_NA

Mmu.2344.1.S1_at	NA	NA	NA	NA	NA	NA	NA	NA	NA	Mmu.2344.1.S1_at_NA
Mmu.235.1.S1_at	NA	NA	NA	NA	NA	NA	NA	NA	NA	Mmu.235.1.S1_at_NA
Mmu.2375.1.S1_at	NA	NA	NA	NA	NA	NA	NA	NA	NA	Mmu.2375.1.S1_at_NA
Mmu.2391.1.S1_at	NA	NA	NA	NA	NA	NA	NA	NA	NA	Mmu.2391.1.S1_at_NA
Mmu.2392.1.S1_at	NA	NA	NA	NA	NA	NA	NA	NA	NA	Mmu.2392.1.S1_at_NA
Mmu.2411.1.S1_at	NA	NA	NA	NA	NA	NA	NA	NA	NA	Mmu.2411.1.S1_at_NA
Mmu.2416.1.S1_at	NA	NA	NA	NA	NA	NA	NA	NA	NA	Mmu.2416.1.S1_at_NA
Mmu.2442.1.S1_at	NA	NA	NA	NA	NA	NA	NA	NA	NA	Mmu.2442.1.S1_at_NA
Mmu.245.1.S1_x_at	200016_x_at	HNRNPA1	heterogeneous nuclear ribonucleoprotein A1	chr12q13.1	3178	LOC702977	heterogeneous nuclear ribonucleoprotein A1	---	702977	Mmu.245.1.S1_x_at_HNRNPA1
Mmu.2452.1.S1_at	224707_at	C5orf32	chromosome 5 open reading frame 32	chr5q31.3	84418	LOC697656	hypothetical protein LOC697656	---	697656	Mmu.2452.1.S1_at_C5orf32
Mmu.2461.3.S1_at	NA	NA	NA	NA	NA	NA	NA	NA	NA	Mmu.2461.3.S1_at_NA
Mmu.2484.1.S1_at	NA	NA	NA	NA	NA	NA	NA	NA	NA	Mmu.2484.1.S1_at_NA
Mmu.249.1.S1_at	NA	NA	NA	NA	NA	NA	NA	NA	NA	Mmu.249.1.S1_at_NA
Mmu.2499.1.S1_at	NA	NA	NA	NA	NA	NA	NA	NA	NA	Mmu.2499.1.S1_at_NA
Mmu.2514.1.S1_at	202453_s_at	GTF2H1	general transcription factor IIH, polypeptide 1, 62kDa	chr11p15.1-p14	2965	GTF2H1	general transcription factor IIH, polypeptide 1, 62kDa	---	694564	Mmu.2514.1.S1_at_GTF2H1
Mmu.2523.1.S1_at	NA	NA	NA	NA	NA	NA	NA	NA	NA	Mmu.2523.1.S1_at_NA
Mmu.2524.1.S1_at	NA	NA	NA	NA	NA	NA	NA	NA	NA	Mmu.2524.1.S1_at_NA
Mmu.2541.1.S1_at	NA	NA	NA	NA	NA	NA	NA	NA	NA	Mmu.2541.1.S1_at_NA
Mmu.2554.1.A1_at	232337_at	B3GNT7	UDP-GlcNAc:betaGal beta-1,3-N-acetylglucosaminyltransferase 7	chr2q37.1	93010	LOC716017	Similar to UDP-GlcNAc:betaGal beta-1,3-N-acetylglucosaminyltransferase 7	---	716017	Mmu.2554.1.A1_at_B3GNT7
Mmu.2556.1.S1_at	NA	NA	NA	NA	NA	NA	NA	NA	NA	Mmu.2556.1.S1_at_NA
Mmu.2571.1.S1_at	216576_x_at	IGKC /// IGKV1-5 /// LO	immunoglobulin kappa constant /// immunoglobulin kappa variable 1-5 /// similar to Ig kappa chain V-I	chr2p12	28299 /// 3514 /// 6475	IGKC /// IGKC /// IGKV1-	similar to Ig kappa chain V-I region Walker precursor /// similar to Ig kappa chain V-I region HK101 pr	---	701600 /// 701734 /// 7179	Mmu.2571.1.S1_at_IGKC /// IGKV1-5 ///
Mmu.2576.1.S1_at	NA	NA	NA	NA	NA	NA	NA	NA	NA	Mmu.2576.1.S1_at_NA
Mmu.258.1.S1_s_at	NA	NA	NA	NA	NA	NA	NA	NA	NA	Mmu.258.1.S1_s_at_NA
Mmu.2594.1.S1_at	NA	NA	NA	NA	NA	NA	NA	NA	NA	Mmu.2594.1.S1_at_NA
Mmu.2601.1.S1_at	NA	NA	NA	NA	NA	NA	NA	NA	NA	Mmu.2601.1.S1_at_NA
Mmu.2604.1.S1_s_at	200820_at	PSMD8	proteasome (prosome, macropain) 26S subunit, non-ATPase, 8	chr19q13.2	5714	LOC721897	similar to 26S proteasome non-ATPase regulatory subunit 8 (26S proteasome regulatory subunit S14	---	721897	Mmu.2604.1.S1_s_at_PSMD8
Mmu.2612.1.S1_at	NA	NA	NA	NA	NA	NA	NA	NA	NA	Mmu.2612.1.S1_at_NA
Mmu.2615.1.S1_at	NA	NA	NA	NA	NA	NA	NA	NA	NA	Mmu.2615.1.S1_at_NA
Mmu.2633.1.S1_at	NA	NA	NA	NA	NA	NA	NA	NA	NA	Mmu.2633.1.S1_at_NA
Mmu.2659.1.S1_at	NA	NA	NA	NA	NA	NA	NA	NA	NA	Mmu.2659.1.S1_at_NA
Mmu.2665.1.S1_at	NA	NA	NA	NA	NA	NA	NA	NA	NA	Mmu.2665.1.S1_at_NA
Mmu.2673.1.S1_at	NA	NA	NA	NA	NA	NA	NA	NA	NA	Mmu.2673.1.S1_at_NA
Mmu.2694.1.S1_at	NA	NA	NA	NA	NA	NA	NA	NA	NA	Mmu.2694.1.S1_at_NA
Mmu.2694.1.S1_s_at	NA	NA	NA	NA	NA	NA	NA	NA	NA	Mmu.2694.1.S1_s_at_NA
Mmu.2695.1.S1_at	231715_s_at	PYCR2	pyrroline-5-carboxylate reductase family, member 2	chr1q42.12	29920	PYCR2	pyrroline-5-carboxylate reductase family, member 2	---	699808	Mmu.2695.1.S1_at_PYCR2
Mmu.2704.1.S1_at	NA	NA	NA	NA	NA	NA	NA	NA	NA	Mmu.2704.1.S1_at_NA
Mmu.2718.1.S1_at	NA	NA	NA	NA	NA	NA	NA	NA	NA	Mmu.2718.1.S1_at_NA
Mmu.2721.1.S1_at	NA	NA	NA	NA	NA	NA	NA	NA	NA	Mmu.2721.1.S1_at_NA
Mmu.2724.1.S1_at	NA	NA	NA	NA	NA	NA	NA	NA	NA	Mmu.2724.1.S1_at_NA
Mmu.2746.2.S1_at	NA	NA	NA	NA	NA	NA	NA	NA	NA	Mmu.2746.2.S1_at_NA
Mmu.2749.1.S1_at	NA	NA	NA	NA	NA	NA	NA	NA	NA	Mmu.2749.1.S1_at_NA
Mmu.2756.1.S1_at	NA	NA	NA	NA	NA	NA	NA	NA	NA	Mmu.2756.1.S1_at_NA
Mmu.2761.1.S1_at	NA	NA	NA	NA	NA	NA	NA	NA	NA	Mmu.2761.1.S1_at_NA
Mmu.2763.1.S1_at	NA	NA	NA	NA	NA	NA	NA	NA	NA	Mmu.2763.1.S1_at_NA
Mmu.2776.1.S1_at	NA	NA	NA	NA	NA	NA	NA	NA	NA	Mmu.2776.1.S1_at_NA
Mmu.2787.1.S1_at	224932_at	CHCHD10	coiled-coil-helix-coiled-coil-helix domain containing 10	chr22q11.23	400916	LOC720205	similar to chromosome 22 open reading frame 16	---	720205	Mmu.2787.1.S1_at_CHCHD10
Mmu.2787.1.S1_x_at	NA	NA	NA	NA	NA	NA	NA	NA	NA	Mmu.2787.1.S1_x_at_NA
Mmu.2788.1.S1_at	NA	NA	NA	NA	NA	NA	NA	NA	NA	Mmu.2788.1.S1_at_NA
Mmu.2788.1.S1_s_at	242400_at	ZFP90	zinc finger protein 90 homolog (mouse)	chr16q22.1	146198	---	---	---	---	Mmu.2788.1.S1_s_at_ZFP90
Mmu.2800.1.S1_at	NA	NA	NA	NA	NA	NA	NA	NA	NA	Mmu.2800.1.S1_at_NA
Mmu.2804.1.S1_at	NA	NA	NA	NA	NA	NA	NA	NA	NA	Mmu.2804.1.S1_at_NA
Mmu.2810.1.S1_at	NA	NA	NA	NA	NA	NA	NA	NA	NA	Mmu.2810.1.S1_at_NA
Mmu.2851.1.S1_at	207509_s_at	LAIR2	leukocyte-associated immunoglobulin-like receptor 2	chr19q13.4	3904	LOC696069	similar to leukocyte-associated immunoglobulin-like receptor 1 isoform b precursor	---	696069	Mmu.2851.1.S1_at_LAIR2
Mmu.2852.1.S1_at	NA	NA	NA	NA	NA	NA	NA	NA	NA	Mmu.2852.1.S1_at_NA
Mmu.2852.1.S1_x_at	NA	NA	NA	NA	NA	NA	NA	NA	NA	Mmu.2852.1.S1_x_at_NA
Mmu.2856.1.S1_at	NA	NA	NA	NA	NA	NA	NA	NA	NA	Mmu.2856.1.S1_at_NA
Mmu.2860.1.S1_at	NA	NA	NA	NA	NA	NA	NA	NA	NA	Mmu.2860.1.S1_at_NA
Mmu.2868.1.S1_at	NA	NA	NA	NA	NA	NA	NA	NA	NA	Mmu.2868.1.S1_at_NA
Mmu.2883.1.S1_at	NA	NA	NA	NA	NA	NA	NA	NA	NA	Mmu.2883.1.S1_at_NA
Mmu.2887.1.S1_at	NA	NA	NA	NA	NA	NA	NA	NA	NA	Mmu.2887.1.S1_at_NA
Mmu.289.1.S1_at	NA	NA	NA	NA	NA	NA	NA	NA	NA	Mmu.289.1.S1_at_NA
Mmu.2918.1.S1_at	NA	NA	NA	NA	NA	NA	NA	NA	NA	Mmu.2918.1.S1_at_NA
Mmu.2925.1.S1_at	NA	NA	NA	NA	NA	NA	NA	NA	NA	Mmu.2925.1.S1_at_NA
Mmu.2935.1.S1_at	NA	NA	NA	NA	NA	NA	NA	NA	NA	Mmu.2935.1.S1_at_NA
Mmu.2935.2.S1_at	NA	NA	NA	NA	NA	NA	NA	NA	NA	Mmu.2935.2.S1_at_NA
Mmu.2935.3.A1_a_at	NA	NA	NA	NA	NA	NA	NA	NA	NA	Mmu.2935.3.A1_a_at_NA
Mmu.2935.3.S1_s_at	NA	NA	NA	NA	NA	NA	NA	NA	NA	Mmu.2935.3.S1_s_at_NA
Mmu.2935.4.S1_x_at	215313_x_at	HLA-A	major histocompatibility complex, class I, A	chr6p21.3	3105	MAMU-A	major histocompatibility complex, class I, A	---	711832	Mmu.2935.4.S1_x_at_HLA-A
Mmu.2975.1.S1_at	NA	NA	NA	NA	NA	NA	NA	NA	NA	Mmu.2975.1.S1_at_NA
Mmu.2976.1.S1_at	NA	NA	NA	NA	NA	NA	NA	NA	NA	Mmu.2976.1.S1_at_NA
Mmu.2984.1.S1_at	NA	NA	NA	NA	NA	NA	NA	NA	NA	Mmu.2984.1.S1_at_NA
Mmu.2985.1.S1_at	NA	NA	NA	NA	NA	NA	NA	NA	NA	Mmu.2985.1.S1_at_NA
Mmu.2989.1.S1_s_at	NA	NA	NA	NA	NA	NA	NA	NA	NA	Mmu.2989.1.S1_s_at_NA
Mmu.3027.1.S1_at	NA	NA	NA	NA	NA	NA	NA	NA	NA	Mmu.3027.1.S1_at_NA
Mmu.303.1.S1_at	NA	NA	NA	NA	NA	NA	NA	NA	NA	Mmu.303.1.S1_at_NA
Mmu.3035.1.S1_x_at	NA	NA	NA	NA	NA	NA	NA	NA	NA	Mmu.3035.1.S1_x_at_NA
Mmu.3041.1.S1_at	NA	NA	NA	NA	NA	NA	NA	NA	NA	Mmu.3041.1.S1_at_NA
Mmu.3044.12.S1_s_at	NA	NA	NA	NA	NA	NA	NA	NA	NA	Mmu.3044.12.S1_s_at_NA
Mmu.3044.13.S1_x_at	NA	NA	NA	NA	NA	NA	NA	NA	NA	Mmu.3044.13.S1_x_at_NA
Mmu.3044.15.S1_at	NA	NA	NA	NA	NA	NA	NA	NA	NA	Mmu.3044.15.S1_at_NA
Mmu.3044.17.S1_x_at	NA	NA	NA	NA	NA	NA	NA	NA	NA	Mmu.3044.17.S1_x_at_NA
Mmu.3044.1.S1_x_at	NA	NA	NA	NA	NA	NA	NA	NA	NA	Mmu.3044.1.S1_x_at_NA
Mmu.3044.6.S1_at	NA	NA	NA	NA	NA	NA	NA	NA	NA	Mmu.3044.6.S1_at_NA
Mmu.3053.1.S1_at	NA	NA	NA	NA	NA	NA	NA	NA	NA	Mmu.3053.1.S1_at_NA
Mmu.3054.2.S1_at	NA	NA	NA	NA	NA	NA	NA	NA	NA	Mmu.3054.2.S1_at_NA
Mmu.3058.1.S1_at	NA	NA	NA	NA	NA	NA	NA	NA	NA	Mmu.3058.1.S1_at_NA
Mmu.3064.1.S1_s_at	NA	NA	NA	NA	NA	NA	NA	NA	NA	Mmu.3064.1.S1_s_at_NA
Mmu.3064.4.S1_s_at	NA	NA	NA	NA	NA	NA	NA	NA	NA	Mmu.3064.4.S1_s_at_NA
Mmu.3073.1.A1_s_at	NA	NA	NA	NA	NA	NA	NA	NA	NA	Mmu.3073.1.A1_s_at_NA
Mmu.3073.1.S1_x_at	NA	NA	NA	NA	NA	NA	NA	NA	NA	Mmu.3073.1.S1_x_at_NA
Mmu.3075.1.S1_at	NA	NA	NA	NA	NA	NA	NA	NA	NA	Mmu.3075.1.S1_at_NA
Mmu.3079.1.S1_at	NA	NA	NA	NA	NA	NA	NA	NA	NA	Mmu.3079.1.S1_at_NA
Mmu.309.1.S1_s_at	NA	NA	NA	NA	NA	NA	NA	NA	NA	Mmu.309.1.S1_s_at_NA
Mmu.3094.1.S1_at	NA	NA	NA	NA	NA	NA	NA	NA	NA	Mmu.3094.1.S1_at_NA
Mmu.3117.1.S1_at	NA	NA	NA	NA	NA	NA	NA	NA	NA	Mmu.3117.1.S1_at_NA
Mmu.312.1.S1_at	NA	NA	NA	NA	NA	NA	NA	NA	NA	Mmu.312.1.S1_at_NA
Mmu.3126.23.S1_at	NA	NA	NA	NA	NA	NA	NA	NA	NA	Mmu.3126.23.S1_at_NA
Mmu.3126.28.S1_at	NA	NA	NA	NA	NA	NA	NA	NA	NA	Mmu.3126.28.S1_at_NA
Mmu.3126.4.S1_at	NA	NA	NA	NA	NA	NA	NA	NA	NA	Mmu.3126.4.S1_at_NA
Mmu.3126.6.S1_x_at	NA	NA	NA	NA	NA	NA	NA	NA	NA	Mmu.3126.6.S1_x_at_NA
Mmu.3127.1.S1_at	NA	NA	NA	NA	NA	NA	NA	NA	NA	Mmu.3127.1.S1_at_NA
Mmu.3131.1.S1_at	NA	NA	NA	NA	NA	NA	NA	NA	NA	Mmu.3131.1.S1_at_NA
Mmu.3131.1.S1_x_at	NA	NA	NA	NA	NA	NA	NA	NA	NA	Mmu.3131.1.S1_x_at_NA
Mmu.313.1.S1_at	202301_s_at	RSRC2	arginine/serine-rich coiled-coil 2	chr12q24.31	65117	---	---	---	---	Mmu.313.1.S1_at_RSRC2
Mmu.3132.1.S1_at	NA	NA	NA	NA	NA	NA	NA	NA	NA	Mmu.3132.1.S1_at_NA
Mmu.3140.1.A1_at	NA	NA	NA	NA	NA	NA	NA	NA	NA	Mmu.3140.1.A1_at_NA
Mmu.3140.1.S1_at	NA	NA	NA	NA	NA	NA	NA	NA	NA	Mmu.3140.1.S1_at_NA
Mmu.3149.1.S1_at	NA	NA	NA	NA	NA	NA	NA	NA	NA	Mmu.3149.1.S1_at_NA

Mmu.3173.1.S1_at	NA	NA	NA	NA	NA	NA	NA	NA	NA	Mmu.3173.1.S1_at_NA
Mmu.3176.1.S1_at	NA	NA	NA	NA	NA	NA	NA	NA	NA	Mmu.3176.1.S1_at_NA
Mmu.3186.1.S1_s_at	NA	NA	NA	NA	NA	NA	NA	NA	NA	Mmu.3186.1.S1_s_at_NA
Mmu.3186.3.S1_at	NA	NA	NA	NA	NA	NA	NA	NA	NA	Mmu.3186.3.S1_at_NA
Mmu.3186.4.S1_at	NA	NA	NA	NA	NA	NA	NA	NA	NA	Mmu.3186.4.S1_at_NA
Mmu.3186.4.S1_x_at	NA	NA	NA	NA	NA	NA	NA	NA	NA	Mmu.3186.4.S1_x_at_NA
Mmu.3187.1.S1_at	NA	NA	NA	NA	NA	NA	NA	NA	NA	Mmu.3187.1.S1_at_NA
Mmu.3193.1.S1_at	202222_s_at	DES	desmin	chr2q35	1674	LOC710096	Similar to Keratin, type II cytoskeletal 8 (Cytokeratin-8) (CK-8) (Keratin-8) (K8)	---	710096	Mmu.3193.1.S1_at_DES
Mmu.3203.2.S1_at	NA	NA	NA	NA	NA	NA	NA	NA	NA	Mmu.3203.2.S1_at_NA
Mmu.3223.1.S1_at	NA	NA	NA	NA	NA	NA	NA	NA	NA	Mmu.3223.1.S1_at_NA
Mmu.3224.1.S1_at	NA	NA	NA	NA	NA	NA	NA	NA	NA	Mmu.3224.1.S1_at_NA
Mmu.3235.1.S1_at	NA	NA	NA	NA	NA	NA	NA	NA	NA	Mmu.3235.1.S1_at_NA
Mmu.3243.1.S1_at	NA	NA	NA	NA	NA	NA	NA	NA	NA	Mmu.3243.1.S1_at_NA
Mmu.3265.1.S1_at	NA	NA	NA	NA	NA	NA	NA	NA	NA	Mmu.3265.1.S1_at_NA
Mmu.3265.1.S1_s_at	NA	NA	NA	NA	NA	NA	NA	NA	NA	Mmu.3265.1.S1_s_at_NA
Mmu.3271.1.A1_at	NA	NA	NA	NA	NA	NA	NA	NA	NA	Mmu.3271.1.A1_at_NA
Mmu.3271.1.S1_at	NA	NA	NA	NA	NA	NA	NA	NA	NA	Mmu.3271.1.S1_at_NA
Mmu.3274.1.S1_at	NA	NA	NA	NA	NA	NA	NA	NA	NA	Mmu.3274.1.S1_at_NA
Mmu.3276.1.S1_at	NA	NA	NA	NA	NA	NA	NA	NA	NA	Mmu.3276.1.S1_at_NA
Mmu.3280.1.S1_at	NA	NA	NA	NA	NA	NA	NA	NA	NA	Mmu.3280.1.S1_at_NA
Mmu.328.1.S1_at	NA	NA	NA	NA	NA	NA	NA	NA	NA	Mmu.328.1.S1_at_NA
Mmu.3285.1.S1_s_at	NA	NA	NA	NA	NA	NA	NA	NA	NA	Mmu.3285.1.S1_s_at_NA
Mmu.3298.1.S1_at	NA	NA	NA	NA	NA	NA	NA	NA	NA	Mmu.3298.1.S1_at_NA
Mmu.3308.1.S1_at	NA	NA	NA	NA	NA	NA	NA	NA	NA	Mmu.3308.1.S1_at_NA
Mmu.3320.1.S1_at	NA	NA	NA	NA	NA	NA	NA	NA	NA	Mmu.3320.1.S1_at_NA
Mmu.3343.2.S1_at	NA	NA	NA	NA	NA	NA	NA	NA	NA	Mmu.3343.2.S1_at_NA
Mmu.3346.2.S1_at	NA	NA	NA	NA	NA	NA	NA	NA	NA	Mmu.3346.2.S1_at_NA
Mmu.3350.1.S1_at	NA	NA	NA	NA	NA	NA	NA	NA	NA	Mmu.3350.1.S1_at_NA
Mmu.335.1.S1_at	NA	NA	NA	NA	NA	NA	NA	NA	NA	Mmu.335.1.S1_at_NA
Mmu.3361.2.S1_s_at	200010_at	RPL11	ribosomal protein L11	chr1p36.1-p35	6135	TCEB3	transcription elongation factor B (SIII), polypeptide 3 (110kDa, elongin A)	---	710467	Mmu.3361.2.S1_s_at_RPL11
Mmu.3375.1.S1_at	NA	NA	NA	NA	NA	NA	NA	NA	NA	Mmu.3375.1.S1_at_NA
Mmu.3378.1.S1_at	NA	NA	NA	NA	NA	NA	NA	NA	NA	Mmu.3378.1.S1_at_NA
Mmu.3384.1.S1_at	NA	NA	NA	NA	NA	NA	NA	NA	NA	Mmu.3384.1.S1_at_NA
Mmu.3395.1.S1_at	NA	NA	NA	NA	NA	NA	NA	NA	NA	Mmu.3395.1.S1_at_NA
Mmu.3395.1.S1_s_at	NA	NA	NA	NA	NA	NA	NA	NA	NA	Mmu.3395.1.S1_s_at_NA
Mmu.3404.1.S1_at	NA	NA	NA	NA	NA	NA	NA	NA	NA	Mmu.3404.1.S1_at_NA
Mmu.3412.1.A1_at	NA	NA	NA	NA	NA	NA	NA	NA	NA	Mmu.3412.1.A1_at_NA
Mmu.3418.2.S1_at	NA	NA	NA	NA	NA	NA	NA	NA	NA	Mmu.3418.2.S1_at_NA
Mmu.3422.1.A1_at	NA	NA	NA	NA	NA	NA	NA	NA	NA	Mmu.3422.1.A1_at_NA
Mmu.3422.1.S1_at	NA	NA	NA	NA	NA	NA	NA	NA	NA	Mmu.3422.1.S1_at_NA
Mmu.3435.1.S1_at	NA	NA	NA	NA	NA	NA	NA	NA	NA	Mmu.3435.1.S1_at_NA
Mmu.3463.1.S1_at	NA	NA	NA	NA	NA	NA	NA	NA	NA	Mmu.3463.1.S1_at_NA
Mmu.3466.1.S1_at	NA	NA	NA	NA	NA	NA	NA	NA	NA	Mmu.3466.1.S1_at_NA
Mmu.3474.1.S1_at	NA	NA	NA	NA	NA	NA	NA	NA	NA	Mmu.3474.1.S1_at_NA
Mmu.3483.1.S1_at	NA	NA	NA	NA	NA	NA	NA	NA	NA	Mmu.3483.1.S1_at_NA
Mmu.3486.1.S1_at	NA	NA	NA	NA	NA	NA	NA	NA	NA	Mmu.3486.1.S1_at_NA
Mmu.3486.2.S1_at	NA	NA	NA	NA	NA	NA	NA	NA	NA	Mmu.3486.2.S1_at_NA
Mmu.3500.1.S1_at	NA	NA	NA	NA	NA	NA	NA	NA	NA	Mmu.3500.1.S1_at_NA
Mmu.3505.2.S1_s_at	NA	NA	NA	NA	NA	NA	NA	NA	NA	Mmu.3505.2.S1_s_at_NA
Mmu.3513.1.S1_at	NA	NA	NA	NA	NA	NA	NA	NA	NA	Mmu.3513.1.S1_at_NA
Mmu.3521.1.A1_at	NA	NA	NA	NA	NA	NA	NA	NA	NA	Mmu.3521.1.A1_at_NA
Mmu.3521.1.S1_at	NA	NA	NA	NA	NA	NA	NA	NA	NA	Mmu.3521.1.S1_at_NA
Mmu.3524.1.S1_at	NA	NA	NA	NA	NA	NA	NA	NA	NA	Mmu.3524.1.S1_at_NA
Mmu.3529.1.S1_at	NA	NA	NA	NA	NA	NA	NA	NA	NA	Mmu.3529.1.S1_at_NA
Mmu.3531.1.A1_at	NA	NA	NA	NA	NA	NA	NA	NA	NA	Mmu.3531.1.A1_at_NA
Mmu.3531.1.S1_at	NA	NA	NA	NA	NA	NA	NA	NA	NA	Mmu.3531.1.S1_at_NA
Mmu.3541.1.S1_at	NA	NA	NA	NA	NA	NA	NA	NA	NA	Mmu.3541.1.S1_at_NA
Mmu.3553.1.A1_at	NA	NA	NA	NA	NA	NA	NA	NA	NA	Mmu.3553.1.A1_at_NA
Mmu.3554.1.S1_at	NA	NA	NA	NA	NA	NA	NA	NA	NA	Mmu.3554.1.S1_at_NA
Mmu.3556.1.S1_s_at	224779_s_at	FAM96A	family with sequence similarity 96, member A	chr15q22.31	84191	LOC714217	similar to family with sequence similarity 96, member A isoform a	---	714217	Mmu.3556.1.S1_s_at_FAM96A
Mmu.3567.1.S1_at	NA	NA	NA	NA	NA	NA	NA	NA	NA	Mmu.3567.1.S1_at_NA
Mmu.357.1.S1_at	NA	NA	NA	NA	NA	NA	NA	NA	NA	Mmu.357.1.S1_at_NA
Mmu.357.1.S1_x_at	217126_at	---	---	---	---	---	---	---	---	Mmu.357.1.S1_x_at_---
Mmu.3573.1.S1_at	NA	NA	NA	NA	NA	NA	NA	NA	NA	Mmu.3573.1.S1_at_NA
Mmu.359.1.S1_at	NA	NA	NA	NA	NA	NA	NA	NA	NA	Mmu.359.1.S1_at_NA
Mmu.3604.1.S1_s_at	NA	NA	NA	NA	NA	NA	NA	NA	NA	Mmu.3604.1.S1_s_at_NA
Mmu.3605.1.S1_at	NA	NA	NA	NA	NA	NA	NA	NA	NA	Mmu.3605.1.S1_at_NA
Mmu.3605.1.S1_s_at	202143_s_at	COPS8	COP9 constitutive photomorphogenic homolog subunit 8 (Arabidopsis)	chr2q37.3	10920	LOC702097	similar to COP9 signalosome complex subunit 8 (Signalosome subunit 8) (SGN8) (JAB1-containing s	---	702097	Mmu.3605.1.S1_s_at_COPS8
Mmu.3611.1.S1_at	NA	NA	NA	NA	NA	NA	NA	NA	NA	Mmu.3611.1.S1_at_NA
Mmu.3625.1.S1_at	NA	NA	NA	NA	NA	NA	NA	NA	NA	Mmu.3625.1.S1_at_NA
Mmu.3626.1.S1_at	NA	NA	NA	NA	NA	NA	NA	NA	NA	Mmu.3626.1.S1_at_NA
Mmu.3634.1.S1_at	NA	NA	NA	NA	NA	NA	NA	NA	NA	Mmu.3634.1.S1_at_NA
Mmu.364.1.S1_at	NA	NA	NA	NA	NA	NA	NA	NA	NA	Mmu.364.1.S1_at_NA
Mmu.367.1.S1_at	NA	NA	NA	NA	NA	NA	NA	NA	NA	Mmu.367.1.S1_at_NA
Mmu.3675.1.S1_at	202410_x_at	IGF2 /// INS-IGF2	insulin-like growth factor 2 (somatomedin A) /// INS-IGF2 readthrough transcript	chr11p15.5	3481 /// 723961	LOC710802	similar to Insulin-like growth factor II precursor (IGF-II) (Somatomedin A)	---	710802	Mmu.3675.1.S1_at_IGF2 /// INS-IGF2
Mmu.3690.1.S1_x_at	NA	NA	NA	NA	NA	NA	NA	NA	NA	Mmu.3690.1.S1_x_at_NA
Mmu.3691.1.A1_at	NA	NA	NA	NA	NA	NA	NA	NA	NA	Mmu.3691.1.A1_at_NA
Mmu.3691.1.S1_at	NA	NA	NA	NA	NA	NA	NA	NA	NA	Mmu.3691.1.S1_at_NA
Mmu.3692.1.S1_at	NA	NA	NA	NA	NA	NA	NA	NA	NA	Mmu.3692.1.S1_at_NA
Mmu.3693.1.A1_at	NA	NA	NA	NA	NA	NA	NA	NA	NA	Mmu.3693.1.A1_at_NA
Mmu.3693.1.S1_at	NA	NA	NA	NA	NA	NA	NA	NA	NA	Mmu.3693.1.S1_at_NA
Mmu.3695.1.S1_s_at	NA	NA	NA	NA	NA	NA	NA	NA	NA	Mmu.3695.1.S1_s_at_NA
Mmu.3696.1.S1_at	NA	NA	NA	NA	NA	NA	NA	NA	NA	Mmu.3696.1.S1_at_NA
Mmu.3708.1.S1_at	NA	NA	NA	NA	NA	NA	NA	NA	NA	Mmu.3708.1.S1_at_NA
Mmu.3718.1.S1_at	NA	NA	NA	NA	NA	NA	NA	NA	NA	Mmu.3718.1.S1_at_NA
Mmu.3719.1.S1_at	NA	NA	NA	NA	NA	NA	NA	NA	NA	Mmu.3719.1.S1_at_NA
Mmu.374.1.S1_at	NA	NA	NA	NA	NA	NA	NA	NA	NA	Mmu.374.1.S1_at_NA
Mmu.3743.1.S1_s_at	NA	NA	NA	NA	NA	NA	NA	NA	NA	Mmu.3743.1.S1_s_at_NA
Mmu.3745.1.S1_at	NA	NA	NA	NA	NA	NA	NA	NA	NA	Mmu.3745.1.S1_at_NA
Mmu.3750.1.S1_at	NA	NA	NA	NA	NA	NA	NA	NA	NA	Mmu.3750.1.S1_at_NA
Mmu.3756.1.S1_at	NA	NA	NA	NA	NA	NA	NA	NA	NA	Mmu.3756.1.S1_at_NA
Mmu.3779.1.S1_at	NA	NA	NA	NA	NA	NA	NA	NA	NA	Mmu.3779.1.S1_at_NA
Mmu.3782.2.S1_a_at	NA	NA	NA	NA	NA	NA	NA	NA	NA	Mmu.3782.2.S1_a_at_NA
Mmu.3793.1.S1_at	NA	NA	NA	NA	NA	NA	NA	NA	NA	Mmu.3793.1.S1_at_NA
Mmu.3794.1.S1_at	NA	NA	NA	NA	NA	NA	NA	NA	NA	Mmu.3794.1.S1_at_NA
Mmu.3795.1.S1_at	229137_at	FUCA1	Fucosidase, alpha-L- 1, tissue	chr1p34	2517	FUCA1	fucosidase, alpha-L- 1, tissue	---	710993	Mmu.3795.1.S1_at_FUCA1
Mmu.3798.1.S1_at	NA	NA	NA	NA	NA	NA	NA	NA	NA	Mmu.3798.1.S1_at_NA
Mmu.380.1.S1_at	NA	NA	NA	NA	NA	NA	NA	NA	NA	Mmu.380.1.S1_at_NA
Mmu.3806.2.S1_at	NA	NA	NA	NA	NA	NA	NA	NA	NA	Mmu.3806.2.S1_at_NA
Mmu.3811.1.S1_at	NA	NA	NA	NA	NA	NA	NA	NA	NA	Mmu.3811.1.S1_at_NA
Mmu.3814.1.S1_at	NA	NA	NA	NA	NA	NA	NA	NA	NA	Mmu.3814.1.S1_at_NA
Mmu.3820.1.S1_at	NA	NA	NA	NA	NA	NA	NA	NA	NA	Mmu.3820.1.S1_at_NA
Mmu.3845.1.S1_at	NA	NA	NA	NA	NA	NA	NA	NA	NA	Mmu.3845.1.S1_at_NA
Mmu.3845.1.S1_x_at	NA	NA	NA	NA	NA	NA	NA	NA	NA	Mmu.3845.1.S1_x_at_NA
Mmu.3847.1.S1_at	NA	NA	NA	NA	NA	NA	NA	NA	NA	Mmu.3847.1.S1_at_NA
Mmu.3853.1.S1_at	NA	NA	NA	NA	NA	NA	NA	NA	NA	Mmu.3853.1.S1_at_NA
Mmu.3865.1.A1_at	NA	NA	NA	NA	NA	NA	NA	NA	NA	Mmu.3865.1.A1_at_NA
Mmu.3886.1.S1_at	NA	NA	NA	NA	NA	NA	NA	NA	NA	Mmu.3886.1.S1_at_NA

Mmu.3898.1.A1_at	NA	NA	NA	NA	NA	NA	NA	NA	NA	Mmu.3898.1.A1_at_NA
Mmu.3902.1.S1_at	NA	NA	NA	NA	NA	NA	NA	NA	NA	Mmu.3902.1.S1_at_NA
Mmu.3906.1.S1_at	NA	NA	NA	NA	NA	NA	NA	NA	NA	Mmu.3906.1.S1_at_NA
Mmu.3907.1.S1_at	227538_at	MED26	mediator complex subunit 26	chr19p13.11	9441	LOC719559	similar to solute carrier family 35, member E1	---	719559	Mmu.3907.1.S1_at_MED26
Mmu.3908.1.S1_s_at	NA	NA	NA	NA	NA	NA	NA	NA	NA	Mmu.3908.1.S1_s_at_NA
Mmu.3911.1.S1_at	NA	NA	NA	NA	NA	NA	NA	NA	NA	Mmu.3911.1.S1_at_NA
Mmu.3918.1.S1_at	NA	NA	NA	NA	NA	NA	NA	NA	NA	Mmu.3918.1.S1_at_NA
Mmu.3931.1.S1_at	220386_s_at	EML4	echinoderm microtubule associated protein like 4	chr2p22-p21	27436	EML4	echinoderm microtubule associated protein like 4	---	715091	Mmu.3931.1.S1_at_EML4
Mmu.3936.1.S1_at	NA	NA	NA	NA	NA	NA	NA	NA	NA	Mmu.3936.1.S1_at_NA
Mmu.3937.1.S1_at	NA	NA	NA	NA	NA	NA	NA	NA	NA	Mmu.3937.1.S1_at_NA
Mmu.394.1.S1_at	NA	NA	NA	NA	NA	NA	NA	NA	NA	Mmu.394.1.S1_at_NA
Mmu.3952.1.A1_at	NA	NA	NA	NA	NA	NA	NA	NA	NA	Mmu.3952.1.A1_at_NA
Mmu.3952.1.S1_at	NA	NA	NA	NA	NA	NA	NA	NA	NA	Mmu.3952.1.S1_at_NA
Mmu.3978.1.S1_at	NA	NA	NA	NA	NA	NA	NA	NA	NA	Mmu.3978.1.S1_at_NA
Mmu.4005.1.S1_s_at	NA	NA	NA	NA	NA	NA	NA	NA	NA	Mmu.4005.1.S1_s_at_NA
Mmu.4013.1.S1_at	NA	NA	NA	NA	NA	NA	NA	NA	NA	Mmu.4013.1.S1_at_NA
Mmu.4026.1.S1_at	NA	NA	NA	NA	NA	NA	NA	NA	NA	Mmu.4026.1.S1_at_NA
Mmu.4039.1.S1_at	NA	NA	NA	NA	NA	NA	NA	NA	NA	Mmu.4039.1.S1_at_NA
Mmu.4060.1.S1_at	NA	NA	NA	NA	NA	NA	NA	NA	NA	Mmu.4060.1.S1_at_NA
Mmu.408.1.S1_at	NA	NA	NA	NA	NA	NA	NA	NA	NA	Mmu.408.1.S1_at_NA
Mmu.4085.1.A1_x_at	NA	NA	NA	NA	NA	NA	NA	NA	NA	Mmu.4085.1.A1_x_at_NA
Mmu.4088.1.S1_at	NA	NA	NA	NA	NA	NA	NA	NA	NA	Mmu.4088.1.S1_at_NA
Mmu.4098.1.S1_at	NA	NA	NA	NA	NA	NA	NA	NA	NA	Mmu.4098.1.S1_at_NA
Mmu.4110.1.S1_s_at	200888_s_at	RPL23	ribosomal protein L23	chr17q	9349	RPL23	ribosomal protein L23	---	694719	Mmu.4110.1.S1_s_at_RPL23
Mmu.4117.1.S1_at	NA	NA	NA	NA	NA	NA	NA	NA	NA	Mmu.4117.1.S1_at_NA
Mmu.4118.1.S1_s_at	NA	NA	NA	NA	NA	NA	NA	NA	NA	Mmu.4118.1.S1_s_at_NA
Mmu.4118.1.S1_x_at	NA	NA	NA	NA	NA	NA	NA	NA	NA	Mmu.4118.1.S1_x_at_NA
Mmu.4119.1.S1_x_at	NA	NA	NA	NA	NA	NA	NA	NA	NA	Mmu.4119.1.S1_x_at_NA
Mmu.4119.2.S1_a_at	NA	NA	NA	NA	NA	NA	NA	NA	NA	Mmu.4119.2.S1_a_at_NA
Mmu.4119.2.S1_s_at	NA	NA	NA	NA	NA	NA	NA	NA	NA	Mmu.4119.2.S1_s_at_NA
Mmu.4119.3.S1_a_at	NA	NA	NA	NA	NA	NA	NA	NA	NA	Mmu.4119.3.S1_a_at_NA
Mmu.4119.3.S1_at	NA	NA	NA	NA	NA	NA	NA	NA	NA	Mmu.4119.3.S1_at_NA
Mmu.4119.3.S1_x_at	NA	NA	NA	NA	NA	NA	NA	NA	NA	Mmu.4119.3.S1_x_at_NA
Mmu.4120.1.S1_x_at	NA	NA	NA	NA	NA	NA	NA	NA	NA	Mmu.4120.1.S1_x_at_NA
Mmu.4122.1.A1_at	NA	NA	NA	NA	NA	NA	NA	NA	NA	Mmu.4122.1.A1_at_NA
Mmu.4122.1.S1_x_at	NA	NA	NA	NA	NA	NA	NA	NA	NA	Mmu.4122.1.S1_x_at_NA
Mmu.4123.1.S1_x_at	NA	NA	NA	NA	NA	NA	NA	NA	NA	Mmu.4123.1.S1_x_at_NA
Mmu.4124.1.S1_a_at	NA	NA	NA	NA	NA	NA	NA	NA	NA	Mmu.4124.1.S1_a_at_NA
Mmu.4125.1.A1_at	NA	NA	NA	NA	NA	NA	NA	NA	NA	Mmu.4125.1.A1_at_NA
Mmu.4125.1.S1_at	NA	NA	NA	NA	NA	NA	NA	NA	NA	Mmu.4125.1.S1_at_NA
Mmu.4125.1.S1_x_at	NA	NA	NA	NA	NA	NA	NA	NA	NA	Mmu.4125.1.S1_x_at_NA
Mmu.4129.1.S1_at	NA	NA	NA	NA	NA	NA	NA	NA	NA	Mmu.4129.1.S1_at_NA
Mmu.4140.1.S1_at	NA	NA	NA	NA	NA	NA	NA	NA	NA	Mmu.4140.1.S1_at_NA
Mmu.414.1.S1_at	NA	NA	NA	NA	NA	NA	NA	NA	NA	Mmu.414.1.S1_at_NA
Mmu.4152.2.S1_at	NA	NA	NA	NA	NA	NA	NA	NA	NA	Mmu.4152.2.S1_at_NA
Mmu.4154.1.S1_at	NA	NA	NA	NA	NA	NA	NA	NA	NA	Mmu.4154.1.S1_at_NA
Mmu.4184.1.A1_at	NA	NA	NA	NA	NA	NA	NA	NA	NA	Mmu.4184.1.A1_at_NA
Mmu.4184.1.S1_at	234849_at	TRA@	T cell receptor alpha locus	chr14q11.2	6955	LOC709909	similar to T-cell receptor alpha chain V region CTL-L17 precursor	---	709909	Mmu.4184.1.S1_at_TRA@
Mmu.4189.1.S1_at	NA	NA	NA	NA	NA	NA	NA	NA	NA	Mmu.4189.1.S1_at_NA
Mmu.4194.1.S1_at	NA	NA	NA	NA	NA	NA	NA	NA	NA	Mmu.4194.1.S1_at_NA
Mmu.4204.1.S1_at	NA	NA	NA	NA	NA	NA	NA	NA	NA	Mmu.4204.1.S1_at_NA
Mmu.421.1.S1_at	NA	NA	NA	NA	NA	NA	NA	NA	NA	Mmu.421.1.S1_at_NA
Mmu.4246.1.S1_at	NA	NA	NA	NA	NA	NA	NA	NA	NA	Mmu.4246.1.S1_at_NA
Mmu.4246.1.S1_s_at	NA	NA	NA	NA	NA	NA	NA	NA	NA	Mmu.4246.1.S1_s_at_NA
Mmu.4266.1.S1_at	NA	NA	NA	NA	NA	NA	NA	NA	NA	Mmu.4266.1.S1_at_NA
Mmu.4269.1.S1_at	NA	NA	NA	NA	NA	NA	NA	NA	NA	Mmu.4269.1.S1_at_NA
Mmu.4271.1.S1_at	NA	NA	NA	NA	NA	NA	NA	NA	NA	Mmu.4271.1.S1_at_NA
Mmu.4290.1.S1_at	NA	NA	NA	NA	NA	NA	NA	NA	NA	Mmu.4290.1.S1_at_NA
Mmu.4305.1.S1_at	NA	NA	NA	NA	NA	NA	NA	NA	NA	Mmu.4305.1.S1_at_NA
Mmu.4317.1.S1_at	NA	NA	NA	NA	NA	NA	NA	NA	NA	Mmu.4317.1.S1_at_NA
Mmu.4317.2.S1_s_at	NA	NA	NA	NA	NA	NA	NA	NA	NA	Mmu.4317.2.S1_s_at_NA
Mmu.4328.1.S1_at	NA	NA	NA	NA	NA	NA	NA	NA	NA	Mmu.4328.1.S1_at_NA
Mmu.4331.1.S1_s_at	NA	NA	NA	NA	NA	NA	NA	NA	NA	Mmu.4331.1.S1_s_at_NA
Mmu.4348.1.S1_at	NA	NA	NA	NA	NA	NA	NA	NA	NA	Mmu.4348.1.S1_at_NA
Mmu.436.1.S1_at	NA	NA	NA	NA	NA	NA	NA	NA	NA	Mmu.436.1.S1_at_NA
Mmu.4374.2.S1_s_at	NA	NA	NA	NA	NA	NA	NA	NA	NA	Mmu.4374.2.S1_s_at_NA
Mmu.4374.2.S1_x_at	NA	NA	NA	NA	NA	NA	NA	NA	NA	Mmu.4374.2.S1_x_at_NA
Mmu.4376.1.A1_at	NA	NA	NA	NA	NA	NA	NA	NA	NA	Mmu.4376.1.A1_at_NA
Mmu.4376.1.S1_at	NA	NA	NA	NA	NA	NA	NA	NA	NA	Mmu.4376.1.S1_at_NA
Mmu.4380.1.S1_at	NA	NA	NA	NA	NA	NA	NA	NA	NA	Mmu.4380.1.S1_at_NA
Mmu.4380.1.S1_s_at	201825_s_at	SCCPDH	saccharopine dehydrogenase (putative)	chr1q44	51097	LOC702924	similar to saccharopine dehydrogenase (putative)	---	702924	Mmu.4380.1.S1_s_at_SCCPDH
Mmu.4385.1.S1_at	NA	NA	NA	NA	NA	NA	NA	NA	NA	Mmu.4385.1.S1_at_NA
Mmu.4390.1.S1_at	NA	NA	NA	NA	NA	NA	NA	NA	NA	Mmu.4390.1.S1_at_NA
Mmu.439.1.S1_x_at	NA	NA	NA	NA	NA	NA	NA	NA	NA	Mmu.439.1.S1_x_at_NA
Mmu.4398.1.S1_s_at	NA	NA	NA	NA	NA	NA	NA	NA	NA	Mmu.4398.1.S1_s_at_NA
Mmu.4403.1.S1_at	NA	NA	NA	NA	NA	NA	NA	NA	NA	Mmu.4403.1.S1_at_NA
Mmu.4413.1.S1_at	NA	NA	NA	NA	NA	NA	NA	NA	NA	Mmu.4413.1.S1_at_NA
Mmu.4422.1.S1_s_at	NA	NA	NA	NA	NA	NA	NA	NA	NA	Mmu.4422.1.S1_s_at_NA
Mmu.4423.1.S1_at	NA	NA	NA	NA	NA	NA	NA	NA	NA	Mmu.4423.1.S1_at_NA
Mmu.4425.1.S1_at	NA	NA	NA	NA	NA	NA	NA	NA	NA	Mmu.4425.1.S1_at_NA
Mmu.443.1.S1_at	NA	NA	NA	NA	NA	NA	NA	NA	NA	Mmu.443.1.S1_at_NA
Mmu.4434.1.S1_at	NA	NA	NA	NA	NA	NA	NA	NA	NA	Mmu.4434.1.S1_at_NA
Mmu.4437.1.S1_at	NA	NA	NA	NA	NA	NA	NA	NA	NA	Mmu.4437.1.S1_at_NA
Mmu.4440.1.S1_at	NA	NA	NA	NA	NA	NA	NA	NA	NA	Mmu.4440.1.S1_at_NA
Mmu.4442.1.S1_at	NA	NA	NA	NA	NA	NA	NA	NA	NA	Mmu.4442.1.S1_at_NA
Mmu.4442.1.S1_s_at	1562106_at	---	---	---	---	LOC720120	Similar to ribosomal protein S18	---	720120	Mmu.4442.1.S1_s_at_---
Mmu.4443.6.S1_at	NA	NA	NA	NA	NA	NA	NA	NA	NA	Mmu.4443.6.S1_at_NA
Mmu.4443.8.S1_at	NA	NA	NA	NA	NA	NA	NA	NA	NA	Mmu.4443.8.S1_at_NA
Mmu.4443.9.S1_at	NA	NA	NA	NA	NA	NA	NA	NA	NA	Mmu.4443.9.S1_at_NA
Mmu.4443.9.S1_x_at	NA	NA	NA	NA	NA	NA	NA	NA	NA	Mmu.4443.9.S1_x_at_NA
Mmu.4444.2.S1_at	NA	NA	NA	NA	NA	NA	NA	NA	NA	Mmu.4444.2.S1_at_NA
Mmu.4448.1.S1_at	NA	NA	NA	NA	NA	NA	NA	NA	NA	Mmu.4448.1.S1_at_NA
Mmu.4450.1.S1_x_at	NA	NA	NA	NA	NA	NA	NA	NA	NA	Mmu.4450.1.S1_x_at_NA
Mmu.4459.1.S1_at	NA	NA	NA	NA	NA	NA	NA	NA	NA	Mmu.4459.1.S1_at_NA
Mmu.4460.1.S1_at	NA	NA	NA	NA	NA	NA	NA	NA	NA	Mmu.4460.1.S1_at_NA
Mmu.4465.1.S1_s_at	NA	NA	NA	NA	NA	NA	NA	NA	NA	Mmu.4465.1.S1_s_at_NA
Mmu.4493.1.S1_at	NA	NA	NA	NA	NA	NA	NA	NA	NA	Mmu.4493.1.S1_at_NA
Mmu.4504.1.S1_at	201084_s_at	BCLAF1	BCL2-associated transcription factor 1	chr6q22-q23	9774	SAR1A	SAR1 homolog A (S. cerevisiae)	---	711277	Mmu.4504.1.S1_at_BCLAF1
Mmu.4512.2.S1_at	NA	NA	NA	NA	NA	NA	NA	NA	NA	Mmu.4512.2.S1_at_NA
Mmu.4519.1.S1_x_at	NA	NA	NA	NA	NA	NA	NA	NA	NA	Mmu.4519.1.S1_x_at_NA
Mmu.452.1.S1_at	229868_s_at	GDF15	Growth differentiation factor 15	chr19p13.11	9518	GDF15	growth differentiation factor 15	---	719466	Mmu.452.1.S1_at_GDF15
Mmu.4523.1.S1_at	NA	NA	NA	NA	NA	NA	NA	NA	NA	Mmu.4523.1.S1_at_NA
Mmu.4529.1.S1_at	NA	NA	NA	NA	NA	NA	NA	NA	NA	Mmu.4529.1.S1_at_NA
Mmu.4533.1.S1_at	219788_at	PILRA	paired immunoglobin-like type 2 receptor alpha	chr7q22.1	29992	LOC711746	similar to paired immunoglobulin-like type 2 receptor alpha isoform 2 precursor	---	711746	Mmu.4533.1.S1_at_PILRA
Mmu.4535.1.S1_at	NA	NA	NA	NA	NA	NA	NA	NA	NA	Mmu.4535.1.S1_at_NA
Mmu.4542.1.A1_at	NA	NA	NA	NA	NA	NA	NA	NA	NA	Mmu.4542.1.A1_at_NA
Mmu.4542.1.S1_at	NA	NA	NA	NA	NA	NA	NA	NA	NA	Mmu.4542.1.S1_at_NA
Mmu.4542.2.A1_s_at	NA	NA	NA	NA	NA	NA	NA	NA	NA	Mmu.4542.2.A1_s_at_NA

Mmu.4542.2.A1_x_at	NA	NA	NA	NA	NA	NA	NA	NA	NA	Mmu.4542.2.A1_x_at_NA
Mmu.4542.2.S1_s_at	NA	NA	NA	NA	NA	NA	NA	NA	NA	Mmu.4542.2.S1_s_at_NA
Mmu.4542.2.S1_x_at	NA	NA	NA	NA	NA	NA	NA	NA	NA	Mmu.4542.2.S1_x_at_NA
Mmu.4580.1.S1_at	201077_s_at	NHP2L1	NHP2 non-histone chromosome protein 2-like 1 (S. cerevisiae)	chr22q13.2-q13.31	4809	---	---	---	---	Mmu.4580.1.S1_at_NHP2L1
Mmu.4582.1.S1_at	NA	NA	NA	NA	NA	NA	NA	NA	NA	Mmu.4582.1.S1_at_NA
Mmu.4582.2.S1_x_at	215821_x_at	PSG3	pregnancy specific beta-1-glycoprotein 3	chr19q13.2	5671	LOC722256	similar to pregnancy specific beta-1-glycoprotein 7	---	722256	Mmu.4582.2.S1_x_at_PSG3
Mmu.4582.3.S1_s_at	NA	NA	NA	NA	NA	NA	NA	NA	NA	Mmu.4582.3.S1_s_at_NA
Mmu.4582.4.S1_at	NA	NA	NA	NA	NA	NA	NA	NA	NA	Mmu.4582.4.S1_at_NA
Mmu.4594.1.S1_s_at	NA	NA	NA	NA	NA	NA	NA	NA	NA	Mmu.4594.1.S1_s_at_NA
Mmu.460.1.S1_s_at	NA	NA	NA	NA	NA	NA	NA	NA	NA	Mmu.460.1.S1_s_at_NA
Mmu.4602.1.S1_at	NA	NA	NA	NA	NA	NA	NA	NA	NA	Mmu.4602.1.S1_at_NA
Mmu.4615.1.S1_s_at	NA	NA	NA	NA	NA	NA	NA	NA	NA	Mmu.4615.1.S1_s_at_NA
Mmu.4627.1.S1_at	NA	NA	NA	NA	NA	NA	NA	NA	NA	Mmu.4627.1.S1_at_NA
Mmu.4647.4.S1_s_at	NA	NA	NA	NA	NA	NA	NA	NA	NA	Mmu.4647.4.S1_s_at_NA
Mmu.4660.1.A1_at	NA	NA	NA	NA	NA	NA	NA	NA	NA	Mmu.4660.1.A1_at_NA
Mmu.4662.5.S1_at	NA	NA	NA	NA	NA	NA	NA	NA	NA	Mmu.4662.5.S1_at_NA
Mmu.4677.1.S1_s_at	NA	NA	NA	NA	NA	NA	NA	NA	NA	Mmu.4677.1.S1_s_at_NA
Mmu.4682.1.S1_at	211363_s_at	MTAP	methylthioadenosine phosphorylase	chr9p21	4507	---	---	---	---	Mmu.4682.1.S1_at_MTAP
Mmu.4689.1.S1_at	NA	NA	NA	NA	NA	NA	NA	NA	NA	Mmu.4689.1.S1_at_NA
Mmu.4690.1.S1_at	223187_s_at	ORMDL1	ORM1-like 1 (S. cerevisiae)	chr2q32	94101	ORMDL1	ORM1-like 1 (S. cerevisiae)	---	709832	Mmu.4690.1.S1_at_ORMDL1
Mmu.4697.1.S1_at	NA	NA	NA	NA	NA	NA	NA	NA	NA	Mmu.4697.1.S1_at_NA
Mmu.4703.1.S1_at	NA	NA	NA	NA	NA	NA	NA	NA	NA	Mmu.4703.1.S1_at_NA
Mmu.4705.1.S1_at	NA	NA	NA	NA	NA	NA	NA	NA	NA	Mmu.4705.1.S1_at_NA
Mmu.4707.1.S1_at	NA	NA	NA	NA	NA	NA	NA	NA	NA	Mmu.4707.1.S1_at_NA
Mmu.4717.1.S1_at	229162_s_at	ABTB1	Ankyrin repeat and BTB (POZ) domain containing 1	chr3q21	80325	LOC710603	similar to ankyrin repeat and BTB (POZ) domain containing 1 isoform 2	---	710603	Mmu.4717.1.S1_at_ABTB1
Mmu.4722.1.S1_at	NA	NA	NA	NA	NA	NA	NA	NA	NA	Mmu.4722.1.S1_at_NA
Mmu.4737.1.S1_at	NA	NA	NA	NA	NA	NA	NA	NA	NA	Mmu.4737.1.S1_at_NA
Mmu.4754.1.S1_at	NA	NA	NA	NA	NA	NA	NA	NA	NA	Mmu.4754.1.S1_at_NA
Mmu.4761.1.S1_at	NA	NA	NA	NA	NA	NA	NA	NA	NA	Mmu.4761.1.S1_at_NA
Mmu.4778.1.S1_at	NA	NA	NA	NA	NA	NA	NA	NA	NA	Mmu.4778.1.S1_at_NA
Mmu.4786.2.S1_at	NA	NA	NA	NA	NA	NA	NA	NA	NA	Mmu.4786.2.S1_at_NA
Mmu.4786.2.S1_s_at	NA	NA	NA	NA	NA	NA	NA	NA	NA	Mmu.4786.2.S1_s_at_NA
Mmu.4813.1.S1_at	NA	NA	NA	NA	NA	NA	NA	NA	NA	Mmu.4813.1.S1_at_NA
Mmu.4815.2.S1_at	NA	NA	NA	NA	NA	NA	NA	NA	NA	Mmu.4815.2.S1_at_NA
Mmu.483.1.S1_at	NA	NA	NA	NA	NA	NA	NA	NA	NA	Mmu.483.1.S1_at_NA
Mmu.4846.1.S1_at	227310_at	ADSS	Adenylosuccinate synthase	chr1cen-q12	159	ADSS	adenylosuccinate synthase	---	713580	Mmu.4846.1.S1_at_ADSS
Mmu.4846.2.S1_at	NA	NA	NA	NA	NA	NA	NA	NA	NA	Mmu.4846.2.S1_at_NA
Mmu.4853.1.S1_at	NA	NA	NA	NA	NA	NA	NA	NA	NA	Mmu.4853.1.S1_at_NA
Mmu.488.1.S1_at	NA	NA	NA	NA	NA	NA	NA	NA	NA	Mmu.488.1.S1_at_NA
Mmu.4882.1.S1_at	NA	NA	NA	NA	NA	NA	NA	NA	NA	Mmu.4882.1.S1_at_NA
Mmu.4888.1.S1_at	NA	NA	NA	NA	NA	NA	NA	NA	NA	Mmu.4888.1.S1_at_NA
Mmu.4889.1.S1_at	NA	NA	NA	NA	NA	NA	NA	NA	NA	Mmu.4889.1.S1_at_NA
Mmu.4898.1.S1_at	NA	NA	NA	NA	NA	NA	NA	NA	NA	Mmu.4898.1.S1_at_NA
Mmu.4904.1.S1_at	NA	NA	NA	NA	NA	NA	NA	NA	NA	Mmu.4904.1.S1_at_NA
Mmu.4923.1.S1_at	210948_s_at	LEF1	lymphoid enhancer-binding factor 1	chr4q23-q25	51176	LOC708226	Similar to keratin 8	---	708226	Mmu.4923.1.S1_at_LEF1
Mmu.4926.1.S1_at	NA	NA	NA	NA	NA	NA	NA	NA	NA	Mmu.4926.1.S1_at_NA
Mmu.4940.1.S1_at	NA	NA	NA	NA	NA	NA	NA	NA	NA	Mmu.4940.1.S1_at_NA
Mmu.4943.1.S1_at	NA	NA	NA	NA	NA	NA	NA	NA	NA	Mmu.4943.1.S1_at_NA
Mmu.4946.1.S1_at	NA	NA	NA	NA	NA	NA	NA	NA	NA	Mmu.4946.1.S1_at_NA
Mmu.4947.1.S1_at	NA	NA	NA	NA	NA	NA	NA	NA	NA	Mmu.4947.1.S1_at_NA
Mmu.4949.1.A1_at	NA	NA	NA	NA	NA	NA	NA	NA	NA	Mmu.4949.1.A1_at_NA
Mmu.4957.1.S1_at	NA	NA	NA	NA	NA	NA	NA	NA	NA	Mmu.4957.1.S1_at_NA
Mmu.4958.1.S1_at	207584_at	LPA	lipoprotein, Lp(a)	chr6q26	4018	LPA	lipoprotein, Lp(a)	---	709528	Mmu.4958.1.S1_at_LPA
Mmu.4959.1.S1_at	NA	NA	NA	NA	NA	NA	NA	NA	NA	Mmu.4959.1.S1_at_NA
Mmu.497.1.A1_at	NA	NA	NA	NA	NA	NA	NA	NA	NA	Mmu.497.1.A1_at_NA
Mmu.4984.1.S1_at	NA	NA	NA	NA	NA	NA	NA	NA	NA	Mmu.4984.1.S1_at_NA
Mmu.4985.1.S1_at	NA	NA	NA	NA	NA	NA	NA	NA	NA	Mmu.4985.1.S1_at_NA
Mmu.4997.1.S1_at	NA	NA	NA	NA	NA	NA	NA	NA	NA	Mmu.4997.1.S1_at_NA
Mmu.5019.1.S1_s_at	NA	NA	NA	NA	NA	NA	NA	NA	NA	Mmu.5019.1.S1_s_at_NA
Mmu.5026.1.S1_at	NA	NA	NA	NA	NA	NA	NA	NA	NA	Mmu.5026.1.S1_at_NA
Mmu.5028.1.S1_at	NA	NA	NA	NA	NA	NA	NA	NA	NA	Mmu.5028.1.S1_at_NA
Mmu.5034.1.A1_at	NA	NA	NA	NA	NA	NA	NA	NA	NA	Mmu.5034.1.A1_at_NA
Mmu.5034.1.S1_s_at	NA	NA	NA	NA	NA	NA	NA	NA	NA	Mmu.5034.1.S1_s_at_NA
Mmu.5034.1.S1_x_at	NA	NA	NA	NA	NA	NA	NA	NA	NA	Mmu.5034.1.S1_x_at_NA
Mmu.5060.1.S1_at	NA	NA	NA	NA	NA	NA	NA	NA	NA	Mmu.5060.1.S1_at_NA
Mmu.506.1.A1_at	NA	NA	NA	NA	NA	NA	NA	NA	NA	Mmu.506.1.A1_at_NA
Mmu.506.1.S1_at	NA	NA	NA	NA	NA	NA	NA	NA	NA	Mmu.506.1.S1_at_NA
Mmu.5062.1.S1_at	NA	NA	NA	NA	NA	NA	NA	NA	NA	Mmu.5062.1.S1_at_NA
Mmu.5066.1.S1_at	NA	NA	NA	NA	NA	NA	NA	NA	NA	Mmu.5066.1.S1_at_NA
Mmu.5073.1.S1_at	NA	NA	NA	NA	NA	NA	NA	NA	NA	Mmu.5073.1.S1_at_NA
Mmu.5090.1.S1_at	NA	NA	NA	NA	NA	NA	NA	NA	NA	Mmu.5090.1.S1_at_NA
Mmu.5094.1.S1_at	NA	NA	NA	NA	NA	NA	NA	NA	NA	Mmu.5094.1.S1_at_NA
Mmu.511.1.S1_at	NA	NA	NA	NA	NA	NA	NA	NA	NA	Mmu.511.1.S1_at_NA
Mmu.512.1.S1_at	NA	NA	NA	NA	NA	NA	NA	NA	NA	Mmu.512.1.S1_at_NA
Mmu.5124.1.S1_at	223993_s_at	CNIH4	cornichon homolog 4 (Drosophila)	chr1q42.11	29097	CNIH4	cornichon homolog 4 (Drosophila)	---	701767	Mmu.5124.1.S1_at_CNIH4
Mmu.5133.1.S1_at	NA	NA	NA	NA	NA	NA	NA	NA	NA	Mmu.5133.1.S1_at_NA
Mmu.5150.1.A1_at	NA	NA	NA	NA	NA	NA	NA	NA	NA	Mmu.5150.1.A1_at_NA
Mmu.5150.1.S1_at	NA	NA	NA	NA	NA	NA	NA	NA	NA	Mmu.5150.1.S1_at_NA
Mmu.5151.1.S1_s_at	NA	NA	NA	NA	NA	NA	NA	NA	NA	Mmu.5151.1.S1_s_at_NA
Mmu.5152.1.S1_at	NA	NA	NA	NA	NA	NA	NA	NA	NA	Mmu.5152.1.S1_at_NA
Mmu.5152.1.S1_x_at	NA	NA	NA	NA	NA	NA	NA	NA	NA	Mmu.5152.1.S1_x_at_NA
Mmu.5155.1.S1_s_at	NA	NA	NA	NA	NA	NA	NA	NA	NA	Mmu.5155.1.S1_s_at_NA
Mmu.5166.1.S1_at	NA	NA	NA	NA	NA	NA	NA	NA	NA	Mmu.5166.1.S1_at_NA
Mmu.518.1.S1_at	NA	NA	NA	NA	NA	NA	NA	NA	NA	Mmu.518.1.S1_at_NA
Mmu.5186.1.S1_at	NA	NA	NA	NA	NA	NA	NA	NA	NA	Mmu.5186.1.S1_at_NA
Mmu.5207.1.S1_at	NA	NA	NA	NA	NA	NA	NA	NA	NA	Mmu.5207.1.S1_at_NA
Mmu.5234.1.S1_at	NA	NA	NA	NA	NA	NA	NA	NA	NA	Mmu.5234.1.S1_at_NA
Mmu.5240.1.S1_at	207278_s_at	CD209	CD209 molecule	chr19p13	30835	CD209L2	C-type lectin CD209L2	---	574373	Mmu.5240.1.S1_at_CD209
Mmu.524.1.S1_at	NA	NA	NA	NA	NA	NA	NA	NA	NA	Mmu.524.1.S1_at_NA
Mmu.5247.1.A1_at	NA	NA	NA	NA	NA	NA	NA	NA	NA	Mmu.5247.1.A1_at_NA
Mmu.5251.1.S1_at	NA	NA	NA	NA	NA	NA	NA	NA	NA	Mmu.5251.1.S1_at_NA
Mmu.5257.1.S1_at	203663_s_at	COX5A	cytochrome c oxidase subunit Va	chr15q24.1	9377	COX5A	cytochrome c oxidase subunit 5A	---	692061	Mmu.5257.1.S1_at_COX5A
Mmu.5288.1.S1_at	NA	NA	NA	NA	NA	NA	NA	NA	NA	Mmu.5288.1.S1_at_NA
Mmu.5300.1.S1_at	NA	NA	NA	NA	NA	NA	NA	NA	NA	Mmu.5300.1.S1_at_NA
Mmu.5303.1.S1_at	NA	NA	NA	NA	NA	NA	NA	NA	NA	Mmu.5303.1.S1_at_NA
Mmu.53.1.S1_at	NA	NA	NA	NA	NA	NA	NA	NA	NA	Mmu.53.1.S1_at_NA
Mmu.5321.1.S1_at	NA	NA	NA	NA	NA	NA	NA	NA	NA	Mmu.5321.1.S1_at_NA
Mmu.5328.1.S1_x_at	NA	NA	NA	NA	NA	NA	NA	NA	NA	Mmu.5328.1.S1_x_at_NA
Mmu.5329.1.S1_at	NA	NA	NA	NA	NA	NA	NA	NA	NA	Mmu.5329.1.S1_at_NA
Mmu.5333.1.S1_at	NA	NA	NA	NA	NA	NA	NA	NA	NA	Mmu.5333.1.S1_at_NA
Mmu.5339.1.S1_at	NA	NA	NA	NA	NA	NA	NA	NA	NA	Mmu.5339.1.S1_at_NA
Mmu.534.1.S1_at	NA	NA	NA	NA	NA	NA	NA	NA	NA	Mmu.534.1.S1_at_NA
Mmu.535.1.S1_at	NA	NA	NA	NA	NA	NA	NA	NA	NA	Mmu.535.1.S1_at_NA
Mmu.5354.1.S1_at	NA	NA	NA	NA	NA	NA	NA	NA	NA	Mmu.5354.1.S1_at_NA
Mmu.5356.1.S1_at	NA	NA	NA	NA	NA	NA	NA	NA	NA	Mmu.5356.1.S1_at_NA
Mmu.5357.1.S1_at	NA	NA	NA	NA	NA	NA	NA	NA	NA	Mmu.5357.1.S1_at_NA
Mmu.5363.1.S1_at	NA	NA	NA	NA	NA	NA	NA	NA	NA	Mmu.5363.1.S1_at_NA
Mmu.5373.1.S1_s_at	NA	NA	NA	NA	NA	NA	NA	NA	NA	Mmu.5373.1.S1_s_at_NA

Mmu.5386.1.S1_at	NA	NA	NA	NA	NA	NA	NA	NA	NA	Mmu.5386.1.S1_at_NA
Mmu.5392.1.S1_at	201909_at	RPS4Y1	ribosomal protein S4, Y-linked 1	chrYp11.3	6192	RPS4Y1	ribosomal protein S4, Y-linked 1	---	100270700	Mmu.5392.1.S1_at_RPS4Y1
Mmu.5406.1.S1_at	NA	NA	NA	NA	NA	NA	NA	NA	NA	Mmu.5406.1.S1_at_NA
Mmu.5409.1.S1_at	NA	NA	NA	NA	NA	NA	NA	NA	NA	Mmu.5409.1.S1_at_NA
Mmu.5423.1.S1_at	NA	NA	NA	NA	NA	NA	NA	NA	NA	Mmu.5423.1.S1_at_NA
Mmu.5427.1.S1_at	NA	NA	NA	NA	NA	NA	NA	NA	NA	Mmu.5427.1.S1_at_NA
Mmu.5431.1.S1_at	NA	NA	NA	NA	NA	NA	NA	NA	NA	Mmu.5431.1.S1_at_NA
Mmu.5435.1.S1_at	NA	NA	NA	NA	NA	NA	NA	NA	NA	Mmu.5435.1.S1_at_NA
Mmu.5448.1.S1_at	NA	NA	NA	NA	NA	NA	NA	NA	NA	Mmu.5448.1.S1_at_NA
Mmu.5468.1.S1_at	202621_at	IRF3	interferon regulatory factor 3	chr19q13.3-q13.4	3661	IRF3	interferon regulatory factor 3	---	718970	Mmu.5468.1.S1_at_IRF3
Mmu.5484.1.S1_s_at	NA	NA	NA	NA	NA	NA	NA	NA	NA	Mmu.5484.1.S1_s_at_NA
Mmu.5491.1.S1_at	NA	NA	NA	NA	NA	NA	NA	NA	NA	Mmu.5491.1.S1_at_NA
Mmu.549.1.S1_x_at	NA	NA	NA	NA	NA	NA	NA	NA	NA	Mmu.549.1.S1_x_at_NA
Mmu.5499.1.S1_at	NA	NA	NA	NA	NA	NA	NA	NA	NA	Mmu.5499.1.S1_at_NA
Mmu.5502.1.S1_at	NA	NA	NA	NA	NA	NA	NA	NA	NA	Mmu.5502.1.S1_at_NA
Mmu.5514.1.S1_at	NA	NA	NA	NA	NA	NA	NA	NA	NA	Mmu.5514.1.S1_at_NA
Mmu.5523.1.S1_at	NA	NA	NA	NA	NA	NA	NA	NA	NA	Mmu.5523.1.S1_at_NA
Mmu.5524.1.S1_at	NA	NA	NA	NA	NA	NA	NA	NA	NA	Mmu.5524.1.S1_at_NA
Mmu.5534.1.S1_at	NA	NA	NA	NA	NA	NA	NA	NA	NA	Mmu.5534.1.S1_at_NA
Mmu.5541.2.S1_at	NA	NA	NA	NA	NA	NA	NA	NA	NA	Mmu.5541.2.S1_at_NA
Mmu.5569.1.S1_at	NA	NA	NA	NA	NA	NA	NA	NA	NA	Mmu.5569.1.S1_at_NA
Mmu.5587.1.S1_at	NA	NA	NA	NA	NA	NA	NA	NA	NA	Mmu.5587.1.S1_at_NA
Mmu.5593.1.S1_at	NA	NA	NA	NA	NA	NA	NA	NA	NA	Mmu.5593.1.S1_at_NA
Mmu.5631.1.A1_at	NA	NA	NA	NA	NA	NA	NA	NA	NA	Mmu.5631.1.A1_at_NA
Mmu.5646.1.A1_at	NA	NA	NA	NA	NA	NA	NA	NA	NA	Mmu.5646.1.A1_at_NA
Mmu.5654.1.S1_at	NA	NA	NA	NA	NA	NA	NA	NA	NA	Mmu.5654.1.S1_at_NA
Mmu.5655.1.S1_s_at	NA	NA	NA	NA	NA	NA	NA	NA	NA	Mmu.5655.1.S1_s_at_NA
Mmu.5673.1.S1_at	NA	NA	NA	NA	NA	NA	NA	NA	NA	Mmu.5673.1.S1_at_NA
Mmu.5674.1.A1_at	NA	NA	NA	NA	NA	NA	NA	NA	NA	Mmu.5674.1.A1_at_NA
Mmu.5674.1.S1_at	NA	NA	NA	NA	NA	NA	NA	NA	NA	Mmu.5674.1.S1_at_NA
Mmu.5677.4.S1_at	NA	NA	NA	NA	NA	NA	NA	NA	NA	Mmu.5677.4.S1_at_NA
Mmu.5680.1.S1_at	NA	NA	NA	NA	NA	NA	NA	NA	NA	Mmu.5680.1.S1_at_NA
Mmu.5689.1.S1_at	NA	NA	NA	NA	NA	NA	NA	NA	NA	Mmu.5689.1.S1_at_NA
Mmu.5691.1.S1_at	NA	NA	NA	NA	NA	NA	NA	NA	NA	Mmu.5691.1.S1_at_NA
Mmu.5692.1.S1_at	NA	NA	NA	NA	NA	NA	NA	NA	NA	Mmu.5692.1.S1_at_NA
Mmu.5693.1.S1_at	NA	NA	NA	NA	NA	NA	NA	NA	NA	Mmu.5693.1.S1_at_NA
Mmu.5696.1.S1_at	NA	NA	NA	NA	NA	NA	NA	NA	NA	Mmu.5696.1.S1_at_NA
Mmu.5709.1.S1_x_at	201550_x_at	ACTG1	actin, gamma 1	chr17q25	71	LOC713687	similar to Actin, cytoplasmic 2 (Gamma-actin)	---	713687	Mmu.5709.1.S1_x_at_ACTG1
Mmu.5709.2.S1_s_at	NA	NA	NA	NA	NA	NA	NA	NA	NA	Mmu.5709.2.S1_s_at_NA
Mmu.5714.1.S1_at	NA	NA	NA	NA	NA	NA	NA	NA	NA	Mmu.5714.1.S1_at_NA
Mmu.5725.1.S1_at	NA	NA	NA	NA	NA	NA	NA	NA	NA	Mmu.5725.1.S1_at_NA
Mmu.5727.1.S1_at	NA	NA	NA	NA	NA	NA	NA	NA	NA	Mmu.5727.1.S1_at_NA
Mmu.5757.1.S1_at	64883_at	MOSPD2	motile sperm domain containing 2	chrXp22.2	158747	LOC712225	similar to motile sperm domain containing 2	---	712225	Mmu.5757.1.S1_at_MOSPD2
Mmu.5766.1.S1_at	224437_s_at	VTA1	Vps20-associated 1 homolog (S. cerevisiae)	chr6q24.1	51534	VTA1	Vps20-associated 1 homolog (S. cerevisiae)	---	701578	Mmu.5766.1.S1_at_VTA1
Mmu.5767.1.A1_at	NA	NA	NA	NA	NA	NA	NA	NA	NA	Mmu.5767.1.A1_at_NA
Mmu.577.1.S1_at	NA	NA	NA	NA	NA	NA	NA	NA	NA	Mmu.577.1.S1_at_NA
Mmu.5782.1.S1_at	NA	NA	NA	NA	NA	NA	NA	NA	NA	Mmu.5782.1.S1_at_NA
Mmu.5802.2.S1_at	NA	NA	NA	NA	NA	NA	NA	NA	NA	Mmu.5802.2.S1_at_NA
Mmu.5808.1.S1_at	NA	NA	NA	NA	NA	NA	NA	NA	NA	Mmu.5808.1.S1_at_NA
Mmu.5811.1.A1_at	NA	NA	NA	NA	NA	NA	NA	NA	NA	Mmu.5811.1.A1_at_NA
Mmu.5814.1.S1_at	NA	NA	NA	NA	NA	NA	NA	NA	NA	Mmu.5814.1.S1_at_NA
Mmu.5825.1.S1_s_at	NA	NA	NA	NA	NA	NA	NA	NA	NA	Mmu.5825.1.S1_s_at_NA
Mmu.5849.1.S1_at	NA	NA	NA	NA	NA	NA	NA	NA	NA	Mmu.5849.1.S1_at_NA
Mmu.586.1.S1_at	NA	NA	NA	NA	NA	NA	NA	NA	NA	Mmu.586.1.S1_at_NA
Mmu.586.2.S1_at	NA	NA	NA	NA	NA	NA	NA	NA	NA	Mmu.586.2.S1_at_NA
Mmu.5870.1.S1_at	NA	NA	NA	NA	NA	NA	NA	NA	NA	Mmu.5870.1.S1_at_NA
Mmu.5871.1.S1_x_at	213614_x_at	EEF1A1	eukaryotic translation elongation factor 1 alpha 1	chr6q14.1	1915	LOC702809	similar to eukaryotic translation elongation factor 1 alpha 2	---	702809	Mmu.5871.1.S1_x_at_EEF1A1
Mmu.5875.1.S1_at	NA	NA	NA	NA	NA	NA	NA	NA	NA	Mmu.5875.1.S1_at_NA
Mmu.5879.1.S1_at	NA	NA	NA	NA	NA	NA	NA	NA	NA	Mmu.5879.1.S1_at_NA
Mmu.5899.1.S1_at	214205_x_at	GLRX3	glutaredoxin 3	chr10q26	10539	---	---	---	---	Mmu.5899.1.S1_at_GLRX3
Mmu.5901.1.S1_at	NA	NA	NA	NA	NA	NA	NA	NA	NA	Mmu.5901.1.S1_at_NA
Mmu.5908.1.S1_at	NA	NA	NA	NA	NA	NA	NA	NA	NA	Mmu.5908.1.S1_at_NA
Mmu.5912.1.S1_at	NA	NA	NA	NA	NA	NA	NA	NA	NA	Mmu.5912.1.S1_at_NA
Mmu.5921.1.S1_at	NA	NA	NA	NA	NA	NA	NA	NA	NA	Mmu.5921.1.S1_at_NA
Mmu.5967.1.S1_s_at	209080_x_at	GLRX3	glutaredoxin 3	chr10q26	10539	GLRX3	glutaredoxin 3	---	698891	Mmu.5967.1.S1_s_at_GLRX3
Mmu.5974.1.S1_at	NA	NA	NA	NA	NA	NA	NA	NA	NA	Mmu.5974.1.S1_at_NA
Mmu.5978.1.S1_at	NA	NA	NA	NA	NA	NA	NA	NA	NA	Mmu.5978.1.S1_at_NA
Mmu.5991.1.S1_at	NA	NA	NA	NA	NA	NA	NA	NA	NA	Mmu.5991.1.S1_at_NA
Mmu.5996.1.S1_s_at	NA	NA	NA	NA	NA	NA	NA	NA	NA	Mmu.5996.1.S1_s_at_NA
Mmu.5996.1.S1_x_at	NA	NA	NA	NA	NA	NA	NA	NA	NA	Mmu.5996.1.S1_x_at_NA
Mmu.5996.2.S1_at	NA	NA	NA	NA	NA	NA	NA	NA	NA	Mmu.5996.2.S1_at_NA
Mmu.5996.2.S1_x_at	234852_at	TRD@	T cell receptor delta locus	chr14q11.2	6964	LOC711141	hypothetical protein LOC711141	---	711141	Mmu.5996.2.S1_x_at_TRD@
Mmu.6007.1.S1_at	NA	NA	NA	NA	NA	NA	NA	NA	NA	Mmu.6007.1.S1_at_NA
Mmu.6016.1.S1_at	212085_at	SLC25A6	solute carrier family 25 (mitochondrial carrier; adenine nucleotide translocator), member 6	chrXp22.32 and Yp11.3	293	SLC25A6	solute carrier family 25 (mitochondrial carrier; adenine nucleotide translocator), member 6	---	719811	Mmu.6016.1.S1_at_SLC25A6
Mmu.6025.1.S1_at	NA	NA	NA	NA	NA	NA	NA	NA	NA	Mmu.6025.1.S1_at_NA
Mmu.6027.1.S1_at	NA	NA	NA	NA	NA	NA	NA	NA	NA	Mmu.6027.1.S1_at_NA
Mmu.6042.1.S1_at	NA	NA	NA	NA	NA	NA	NA	NA	NA	Mmu.6042.1.S1_at_NA
Mmu.6048.1.S1_s_at	NA	NA	NA	NA	NA	NA	NA	NA	NA	Mmu.6048.1.S1_s_at_NA
Mmu.6048.2.S1_at	NA	NA	NA	NA	NA	NA	NA	NA	NA	Mmu.6048.2.S1_at_NA
Mmu.6050.1.S1_at	NA	NA	NA	NA	NA	NA	NA	NA	NA	Mmu.6050.1.S1_at_NA
Mmu.6053.1.S1_at	NA	NA	NA	NA	NA	NA	NA	NA	NA	Mmu.6053.1.S1_at_NA
Mmu.6061.1.A1_at	NA	NA	NA	NA	NA	NA	NA	NA	NA	Mmu.6061.1.A1_at_NA
Mmu.6061.1.S1_at	NA	NA	NA	NA	NA	NA	NA	NA	NA	Mmu.6061.1.S1_at_NA
Mmu.6083.1.S1_at	212195_at	IL6ST	interleukin 6 signal transducer (gp130, oncostatin M receptor)	chr5q11	3572	---	---	---	---	Mmu.6083.1.S1_at_IL6ST
Mmu.6085.1.S1_x_at	208812_x_at	HLA-C	major histocompatibility complex, class I, C	chr6p21.3	3107	LOC720369 /// MAMU-B	hypothetical protein LOC720369 /// MHC class I antigen	---	700391 /// 720369	Mmu.6085.1.S1_x_at_HLA-C
Mmu.6085.2.S1_x_at	NA	NA	NA	NA	NA	NA	NA	NA	NA	Mmu.6085.2.S1_x_at_NA
Mmu.6118.1.S1_at	NA	NA	NA	NA	NA	NA	NA	NA	NA	Mmu.6118.1.S1_at_NA
Mmu.6125.1.A1_at	NA	NA	NA	NA	NA	NA	NA	NA	NA	Mmu.6125.1.A1_at_NA
Mmu.6125.1.S1_at	NA	NA	NA	NA	NA	NA	NA	NA	NA	Mmu.6125.1.S1_at_NA
Mmu.6135.1.S1_at	NA	NA	NA	NA	NA	NA	NA	NA	NA	Mmu.6135.1.S1_at_NA
Mmu.6139.1.S1_s_at	NA	NA	NA	NA	NA	NA	NA	NA	NA	Mmu.6139.1.S1_s_at_NA
Mmu.6141.1.A1_at	NA	NA	NA	NA	NA	NA	NA	NA	NA	Mmu.6141.1.A1_at_NA
Mmu.6141.1.S1_at	NA	NA	NA	NA	NA	NA	NA	NA	NA	Mmu.6141.1.S1_at_NA
Mmu.616.1.S1_at	NA	NA	NA	NA	NA	NA	NA	NA	NA	Mmu.616.1.S1_at_NA
Mmu.6177.1.A1_at	NA	NA	NA	NA	NA	NA	NA	NA	NA	Mmu.6177.1.A1_at_NA
Mmu.6180.1.S1_s_at	NA	NA	NA	NA	NA	NA	NA	NA	NA	Mmu.6180.1.S1_s_at_NA
Mmu.6187.1.S1_at	NA	NA	NA	NA	NA	NA	NA	NA	NA	Mmu.6187.1.S1_at_NA
Mmu.6201.1.S1_at	NA	NA	NA	NA	NA	NA	NA	NA	NA	Mmu.6201.1.S1_at_NA
Mmu.6263.1.S1_s_at	NA	NA	NA	NA	NA	NA	NA	NA	NA	Mmu.6263.1.S1_s_at_NA
Mmu.6269.1.S1_at	NA	NA	NA	NA	NA	NA	NA	NA	NA	Mmu.6269.1.S1_at_NA
Mmu.6271.1.S1_at	NA	NA	NA	NA	NA	NA	NA	NA	NA	Mmu.6271.1.S1_at_NA
Mmu.6271.2.S1_a_at	NA	NA	NA	NA	NA	NA	NA	NA	NA	Mmu.6271.2.S1_a_at_NA
Mmu.6272.2.S1_at	NA	NA	NA	NA	NA	NA	NA	NA	NA	Mmu.6272.2.S1_at_NA
Mmu.6282.1.S1_at	NA	NA	NA	NA	NA	NA	NA	NA	NA	Mmu.6282.1.S1_at_NA
Mmu.629.1.S1_s_at	NA	NA	NA	NA	NA	NA	NA	NA	NA	Mmu.629.1.S1_s_at_NA
Mmu.629.2.S1_a_at	NA	NA	NA	NA	NA	NA	NA	NA	NA	Mmu.629.2.S1_a_at_NA
Mmu.629.2.S1_at	225647_s_at	CTSC	cathepsin C	chr11q14.1-q14.3	1075	LOC701346	Similar to 60 kDa heat shock protein, mitochondrial precursor (Hsp60) (60 kDa chaperonin) (CPN60)	---	701346	Mmu.629.2.S1_at_CTSC
Mmu.6308.1.S1_at	NA	NA	NA	NA	NA	NA	NA	NA	NA	Mmu.6308.1.S1_at_NA

Mmu.6310.1.S1_at	NA	NA	NA	NA	NA	NA	NA	NA	NA	Mmu.6310.1.S1_at_NA
Mmu.6311.1.S1_at	NA	NA	NA	NA	NA	NA	NA	NA	NA	Mmu.6311.1.S1_at_NA
Mmu.6318.1.S1_at	NA	NA	NA	NA	NA	NA	NA	NA	NA	Mmu.6318.1.S1_at_NA
Mmu.6342.1.S1_at	NA	NA	NA	NA	NA	NA	NA	NA	NA	Mmu.6342.1.S1_at_NA
Mmu.6352.1.S1_at	NA	NA	NA	NA	NA	NA	NA	NA	NA	Mmu.6352.1.S1_at_NA
Mmu.6387.1.S1_s_at	216338_s_at	YIPF3	Yip1 domain family, member 3	chr6p21.1	25844	YIPF3	Yip1 domain family, member 3	---	700228	Mmu.6387.1.S1_s_at_YIPF3
Mmu.6397.2.S1_at	NA	NA	NA	NA	NA	NA	NA	NA	NA	Mmu.6397.2.S1_at_NA
Mmu.6399.1.S1_at	NA	NA	NA	NA	NA	NA	NA	NA	NA	Mmu.6399.1.S1_at_NA
Mmu.6412.1.S1_at	NA	NA	NA	NA	NA	NA	NA	NA	NA	Mmu.6412.1.S1_at_NA
Mmu.6416.1.S1_at	NA	NA	NA	NA	NA	NA	NA	NA	NA	Mmu.6416.1.S1_at_NA
Mmu.6423.1.S1_at	NA	NA	NA	NA	NA	NA	NA	NA	NA	Mmu.6423.1.S1_at_NA
Mmu.6425.1.S1_at	NA	NA	NA	NA	NA	NA	NA	NA	NA	Mmu.6425.1.S1_at_NA
Mmu.6430.2.S1_at	NA	NA	NA	NA	NA	NA	NA	NA	NA	Mmu.6430.2.S1_at_NA
Mmu.6446.1.S1_at	NA	NA	NA	NA	NA	NA	NA	NA	NA	Mmu.6446.1.S1_at_NA
Mmu.6446.1.S1_x_at	NA	NA	NA	NA	NA	NA	NA	NA	NA	Mmu.6446.1.S1_x_at_NA
Mmu.6455.1.S1_at	NA	NA	NA	NA	NA	NA	NA	NA	NA	Mmu.6455.1.S1_at_NA
Mmu.6457.1.A1_at	NA	NA	NA	NA	NA	NA	NA	NA	NA	Mmu.6457.1.A1_at_NA
Mmu.6457.1.S1_at	NA	NA	NA	NA	NA	NA	NA	NA	NA	Mmu.6457.1.S1_at_NA
Mmu.6469.1.S1_at	NA	NA	NA	NA	NA	NA	NA	NA	NA	Mmu.6469.1.S1_at_NA
Mmu.6470.1.S1_at	NA	NA	NA	NA	NA	NA	NA	NA	NA	Mmu.6470.1.S1_at_NA
Mmu.6471.1.S1_at	NA	NA	NA	NA	NA	NA	NA	NA	NA	Mmu.6471.1.S1_at_NA
Mmu.6471.1.S1_x_at	NA	NA	NA	NA	NA	NA	NA	NA	NA	Mmu.6471.1.S1_x_at_NA
Mmu.6481.1.S1_at	NA	NA	NA	NA	NA	NA	NA	NA	NA	Mmu.6481.1.S1_at_NA
Mmu.6504.1.S1_at	NA	NA	NA	NA	NA	NA	NA	NA	NA	Mmu.6504.1.S1_at_NA
Mmu.6513.1.S1_at	NA	NA	NA	NA	NA	NA	NA	NA	NA	Mmu.6513.1.S1_at_NA
Mmu.6514.1.S1_at	NA	NA	NA	NA	NA	NA	NA	NA	NA	Mmu.6514.1.S1_at_NA
Mmu.6522.1.S1_at	NA	NA	NA	NA	NA	NA	NA	NA	NA	Mmu.6522.1.S1_at_NA
Mmu.6550.1.S1_at	NA	NA	NA	NA	NA	NA	NA	NA	NA	Mmu.6550.1.S1_at_NA
Mmu.6551.1.S1_at	NA	NA	NA	NA	NA	NA	NA	NA	NA	Mmu.6551.1.S1_at_NA
Mmu.6554.1.S1_at	203203_s_at	KRR1	KRR1, small subunit (SSU) processome component, homolog (yeast)	chr12q21.2	11103	BCOR	BCL6 co-repressor	---	698644	Mmu.6554.1.S1_at_KRR1
Mmu.6556.1.S1_at	NA	NA	NA	NA	NA	NA	NA	NA	NA	Mmu.6556.1.S1_at_NA
Mmu.6559.1.S1_s_at	NA	NA	NA	NA	NA	NA	NA	NA	NA	Mmu.6559.1.S1_s_at_NA
Mmu.656.1.S1_at	NA	NA	NA	NA	NA	NA	NA	NA	NA	Mmu.656.1.S1_at_NA
Mmu.6573.1.S1_at	NA	NA	NA	NA	NA	NA	NA	NA	NA	Mmu.6573.1.S1_at_NA
Mmu.6593.2.S1_x_at	1553518_at	DEFT1P	defensin, theta 1 pseudogene	chr8p23.1	170949	LOC574123	demidefensin 1	---	574123	Mmu.6593.2.S1_x_at_DEFT1P
Mmu.6609.1.S1_at	NA	NA	NA	NA	NA	NA	NA	NA	NA	Mmu.6609.1.S1_at_NA
Mmu.6623.1.S1_at	NA	NA	NA	NA	NA	NA	NA	NA	NA	Mmu.6623.1.S1_at_NA
Mmu.6637.1.S1_at	NA	NA	NA	NA	NA	NA	NA	NA	NA	Mmu.6637.1.S1_at_NA
Mmu.6642.1.S1_at	NA	NA	NA	NA	NA	NA	NA	NA	NA	Mmu.6642.1.S1_at_NA
Mmu.6646.1.S1_at	NA	NA	NA	NA	NA	NA	NA	NA	NA	Mmu.6646.1.S1_at_NA
Mmu.666.1.S1_at	NA	NA	NA	NA	NA	NA	NA	NA	NA	Mmu.666.1.S1_at_NA
Mmu.6678.1.S1_at	213629_x_at	MT1F	metallothionein 1F	chr16q13	4494	LOC709619	similar to Metallothionein-1I (MT-1I) (Metallothionein-II)	---	709619	Mmu.6678.1.S1_at_MT1F
Mmu.6678.1.S1_x_at	NA	NA	NA	NA	NA	NA	NA	NA	NA	Mmu.6678.1.S1_x_at_NA
Mmu.6687.1.S1_at	NA	NA	NA	NA	NA	NA	NA	NA	NA	Mmu.6687.1.S1_at_NA
Mmu.6716.1.S1_at	NA	NA	NA	NA	NA	NA	NA	NA	NA	Mmu.6716.1.S1_at_NA
Mmu.6730.1.S1_at	219014_at	PLAC8	placenta-specific 8	chr4q21.22	51316	---	---	---	---	Mmu.6730.1.S1_at_PLAC8
Mmu.6738.1.S1_at	NA	NA	NA	NA	NA	NA	NA	NA	NA	Mmu.6738.1.S1_at_NA
Mmu.6738.1.S1_s_at	NA	NA	NA	NA	NA	NA	NA	NA	NA	Mmu.6738.1.S1_s_at_NA
Mmu.6739.1.S1_at	NA	NA	NA	NA	NA	NA	NA	NA	NA	Mmu.6739.1.S1_at_NA
Mmu.6744.2.S1_at	NA	NA	NA	NA	NA	NA	NA	NA	NA	Mmu.6744.2.S1_at_NA
Mmu.6763.1.S1_at	NA	NA	NA	NA	NA	NA	NA	NA	NA	Mmu.6763.1.S1_at_NA
Mmu.6769.1.S1_at	NA	NA	NA	NA	NA	NA	NA	NA	NA	Mmu.6769.1.S1_at_NA
Mmu.6773.1.S1_at	NA	NA	NA	NA	NA	NA	NA	NA	NA	Mmu.6773.1.S1_at_NA
Mmu.6776.1.A1_at	NA	NA	NA	NA	NA	NA	NA	NA	NA	Mmu.6776.1.A1_at_NA
Mmu.6776.1.S1_at	NA	NA	NA	NA	NA	NA	NA	NA	NA	Mmu.6776.1.S1_at_NA
Mmu.6784.1.S1_at	NA	NA	NA	NA	NA	NA	NA	NA	NA	Mmu.6784.1.S1_at_NA
Mmu.6788.1.S1_at	NA	NA	NA	NA	NA	NA	NA	NA	NA	Mmu.6788.1.S1_at_NA
Mmu.6796.1.S1_at	NA	NA	NA	NA	NA	NA	NA	NA	NA	Mmu.6796.1.S1_at_NA
Mmu.6800.1.S1_at	NA	NA	NA	NA	NA	NA	NA	NA	NA	Mmu.6800.1.S1_at_NA
Mmu.6832.1.S1_at	218281_at	MRPL48	mitochondrial ribosomal protein L48	chr11q13.4	51642	LOC718240	similar to mitochondrial ribosomal protein L48 isoform 1	---	718240	Mmu.6832.1.S1_at_MRPL48
Mmu.6835.1.S1_at	NA	NA	NA	NA	NA	NA	NA	NA	NA	Mmu.6835.1.S1_at_NA
Mmu.6867.1.A1_at	NA	NA	NA	NA	NA	NA	NA	NA	NA	Mmu.6867.1.A1_at_NA
Mmu.6867.1.S1_at	NA	NA	NA	NA	NA	NA	NA	NA	NA	Mmu.6867.1.S1_at_NA
Mmu.6867.1.S1_s_at	NA	NA	NA	NA	NA	NA	NA	NA	NA	Mmu.6867.1.S1_s_at_NA
Mmu.6867.2.S1_at	NA	NA	NA	NA	NA	NA	NA	NA	NA	Mmu.6867.2.S1_at_NA
Mmu.6867.3.S1_s_at	NA	NA	NA	NA	NA	NA	NA	NA	NA	Mmu.6867.3.S1_s_at_NA
Mmu.6887.1.S1_at	NA	NA	NA	NA	NA	NA	NA	NA	NA	Mmu.6887.1.S1_at_NA
Mmu.6892.1.S1_at	NA	NA	NA	NA	NA	NA	NA	NA	NA	Mmu.6892.1.S1_at_NA
Mmu.6898.1.S1_at	NA	NA	NA	NA	NA	NA	NA	NA	NA	Mmu.6898.1.S1_at_NA
Mmu.6922.1.A1_at	NA	NA	NA	NA	NA	NA	NA	NA	NA	Mmu.6922.1.A1_at_NA
Mmu.6922.1.S1_at	NA	NA	NA	NA	NA	NA	NA	NA	NA	Mmu.6922.1.S1_at_NA
Mmu.6936.1.S1_at	NA	NA	NA	NA	NA	NA	NA	NA	NA	Mmu.6936.1.S1_at_NA
Mmu.6961.1.S1_at	NA	NA	NA	NA	NA	NA	NA	NA	NA	Mmu.6961.1.S1_at_NA
Mmu.6961.1.S1_s_at	NA	NA	NA	NA	NA	NA	NA	NA	NA	Mmu.6961.1.S1_s_at_NA
Mmu.6977.1.S1_at	NA	NA	NA	NA	NA	NA	NA	NA	NA	Mmu.6977.1.S1_at_NA
Mmu.6979.1.S1_at	NA	NA	NA	NA	NA	NA	NA	NA	NA	Mmu.6979.1.S1_at_NA
Mmu.6994.1.S1_at	NA	NA	NA	NA	NA	NA	NA	NA	NA	Mmu.6994.1.S1_at_NA
Mmu.7027.1.S1_at	NA	NA	NA	NA	NA	NA	NA	NA	NA	Mmu.7027.1.S1_at_NA
Mmu.7028.1.S1_at	NA	NA	NA	NA	NA	NA	NA	NA	NA	Mmu.7028.1.S1_at_NA
Mmu.7032.1.S1_s_at	213011_s_at	TPI1	triosephosphate isomerase 1	chr12p13	7167	TPI1	triosephosphate isomerase 1	---	714090	Mmu.7032.1.S1_s_at_TPI1
Mmu.7046.1.S1_at	NA	NA	NA	NA	NA	NA	NA	NA	NA	Mmu.7046.1.S1_at_NA
Mmu.7053.1.S1_at	NA	NA	NA	NA	NA	NA	NA	NA	NA	Mmu.7053.1.S1_at_NA
Mmu.7057.1.S1_at	NA	NA	NA	NA	NA	NA	NA	NA	NA	Mmu.7057.1.S1_at_NA
Mmu.7075.1.S1_at	NA	NA	NA	NA	NA	NA	NA	NA	NA	Mmu.7075.1.S1_at_NA
Mmu.7075.1.S2_s_at	NA	NA	NA	NA	NA	NA	NA	NA	NA	Mmu.7075.1.S2_s_at_NA
Mmu.7078.1.S1_s_at	212857_x_at	SUB1	SUB1 homolog (S. cerevisiae)	chr5p13.3	10923	LOC701555	Similar to spermatid-specific linker histone H1-like protein	---	701555	Mmu.7078.1.S1_s_at_SUB1
Mmu.7081.1.S1_at	NA	NA	NA	NA	NA	NA	NA	NA	NA	Mmu.7081.1.S1_at_NA
Mmu.7083.1.S1_at	219761_at	CLEC1A	C-type lectin domain family 1, member A	chr12p13.2	51267	CLEC1A	C-type lectin domain family 1, member A	---	717526	Mmu.7083.1.S1_at_CLEC1A
Mmu.7088.1.S1_at	NA	NA	NA	NA	NA	NA	NA	NA	NA	Mmu.7088.1.S1_at_NA
Mmu.7098.1.S1_at	NA	NA	NA	NA	NA	NA	NA	NA	NA	Mmu.7098.1.S1_at_NA
Mmu.7120.1.S1_at	NA	NA	NA	NA	NA	NA	NA	NA	NA	Mmu.7120.1.S1_at_NA
Mmu.7135.1.S1_at	NA	NA	NA	NA	NA	NA	NA	NA	NA	Mmu.7135.1.S1_at_NA
Mmu.7150.1.S1_at	NA	NA	NA	NA	NA	NA	NA	NA	NA	Mmu.7150.1.S1_at_NA
Mmu.7164.1.S1_at	NA	NA	NA	NA	NA	NA	NA	NA	NA	Mmu.7164.1.S1_at_NA
Mmu.7173.1.S1_at	NA	NA	NA	NA	NA	NA	NA	NA	NA	Mmu.7173.1.S1_at_NA
Mmu.7179.1.S1_at	NA	NA	NA	NA	NA	NA	NA	NA	NA	Mmu.7179.1.S1_at_NA
Mmu.7190.1.A1_at	NA	NA	NA	NA	NA	NA	NA	NA	NA	Mmu.7190.1.A1_at_NA
Mmu.7191.1.S1_at	NA	NA	NA	NA	NA	NA	NA	NA	NA	Mmu.7191.1.S1_at_NA
Mmu.7193.1.S1_at	NA	NA	NA	NA	NA	NA	NA	NA	NA	Mmu.7193.1.S1_at_NA
Mmu.7205.1.A1_at	NA	NA	NA	NA	NA	NA	NA	NA	NA	Mmu.7205.1.A1_at_NA
Mmu.7207.1.S1_at	NA	NA	NA	NA	NA	NA	NA	NA	NA	Mmu.7207.1.S1_at_NA
Mmu.7208.1.S1_at	NA	NA	NA	NA	NA	NA	NA	NA	NA	Mmu.7208.1.S1_at_NA
Mmu.722.1.S1_at	NA	NA	NA	NA	NA	NA	NA	NA	NA	Mmu.722.1.S1_at_NA
Mmu.7225.1.S1_at	NA	NA	NA	NA	NA	NA	NA	NA	NA	Mmu.7225.1.S1_at_NA
Mmu.7236.1.S1_at	NA	NA	NA	NA	NA	NA	NA	NA	NA	Mmu.7236.1.S1_at_NA
Mmu.7236.1.S1_s_at	NA	NA	NA	NA	NA	NA	NA	NA	NA	Mmu.7236.1.S1_s_at_NA
Mmu.7267.1.S1_at	228491_at	KRT19	Keratin 19	chr17q21.2	3880	LOC707129	similar to keratin 19	---	707129	Mmu.7267.1.S1_at_KRT19
Mmu.7278.1.A1_at	NA	NA	NA	NA	NA	NA	NA	NA	NA	Mmu.7278.1.A1_at_NA

Mmu.7278.1.S1_at	NA	NA	NA	NA	NA	NA	NA	NA	NA	Mmu.7278.1.S1_at_NA
Mmu.7280.1.S1_at	NA	NA	NA	NA	NA	NA	NA	NA	NA	Mmu.7280.1.S1_at_NA
Mmu.7281.1.S1_s_at	NA	NA	NA	NA	NA	NA	NA	NA	NA	Mmu.7281.1.S1_s_at_NA
Mmu.7287.1.S1_at	NA	NA	NA	NA	NA	NA	NA	NA	NA	Mmu.7287.1.S1_at_NA
Mmu.729.1.S1_at	NA	NA	NA	NA	NA	NA	NA	NA	NA	Mmu.729.1.S1_at_NA
Mmu.7319.1.S1_at	NA	NA	NA	NA	NA	NA	NA	NA	NA	Mmu.7319.1.S1_at_NA
Mmu.7346.1.S1_at	NA	NA	NA	NA	NA	NA	NA	NA	NA	Mmu.7346.1.S1_at_NA
Mmu.7348.2.S1_a_at	NA	NA	NA	NA	NA	NA	NA	NA	NA	Mmu.7348.2.S1_a_at_NA
Mmu.7348.2.S1_at	NA	NA	NA	NA	NA	NA	NA	NA	NA	Mmu.7348.2.S1_at_NA
Mmu.737.1.S1_s_at	NA	NA	NA	NA	NA	NA	NA	NA	NA	Mmu.737.1.S1_s_at_NA
Mmu.7379.1.A1_at	NA	NA	NA	NA	NA	NA	NA	NA	NA	Mmu.7379.1.A1_at_NA
Mmu.7380.1.S1_at	NA	NA	NA	NA	NA	NA	NA	NA	NA	Mmu.7380.1.S1_at_NA
Mmu.7385.1.S1_at	NA	NA	NA	NA	NA	NA	NA	NA	NA	Mmu.7385.1.S1_at_NA
Mmu.7390.1.S1_at	214792_x_at	VAMP2	vesicle-associated membrane protein 2 (synaptobrevin 2)	chr17p13.1	6844	LOC574134	VAMP-2	---	574134	Mmu.7390.1.S1_at_VAMP2
Mmu.7394.1.A1_at	NA	NA	NA	NA	NA	NA	NA	NA	NA	Mmu.7394.1.A1_at_NA
Mmu.7399.1.S1_at	201322_at	ATP5B	ATP synthase, H+ transporting, mitochondrial F1 complex, beta polypeptide	chr12q13.13	506	ATP5B	ATP synthase, H+ transporting, mitochondrial F1 complex, beta polypeptide	---	705465	Mmu.7399.1.S1_at_ATP5B
Mmu.7434.1.S1_at	NA	NA	NA	NA	NA	NA	NA	NA	NA	Mmu.7434.1.S1_at_NA
Mmu.7445.1.S1_at	NA	NA	NA	NA	NA	NA	NA	NA	NA	Mmu.7445.1.S1_at_NA
Mmu.7448.1.S1_at	NA	NA	NA	NA	NA	NA	NA	NA	NA	Mmu.7448.1.S1_at_NA
Mmu.7453.1.S1_at	NA	NA	NA	NA	NA	NA	NA	NA	NA	Mmu.7453.1.S1_at_NA
Mmu.7454.1.S1_at	NA	NA	NA	NA	NA	NA	NA	NA	NA	Mmu.7454.1.S1_at_NA
Mmu.7458.1.S1_at	NA	NA	NA	NA	NA	NA	NA	NA	NA	Mmu.7458.1.S1_at_NA
Mmu.7460.10.S1_x_at	NA	NA	NA	NA	NA	NA	NA	NA	NA	Mmu.7460.10.S1_x_at_NA
Mmu.7460.12.S1_at	208122_x_at	KIR2DS3	killer cell immunoglobulin-like receptor, two domains, short cytoplasmic tail, 3	chr19q13.4	3808	KIR3DL10	killer immunoglobulin-like receptor KIR3DL10	---	100125558	Mmu.7460.12.S1_at_KIR2DS3
Mmu.7460.12.S1_x_at	NA	NA	NA	NA	NA	NA	NA	NA	NA	Mmu.7460.12.S1_x_at_NA
Mmu.7460.14.S1_s_at	208179_x_at	KIR2DL3	killer cell immunoglobulin-like receptor, two domains, long cytoplasmic tail, 3	chr19q13.4	3804	KIR3DL /// KIR3DL10 /// K	killer immunoglobulin-like receptor KIR3DL /// killer immunoglobulin-like receptor KIR3DL10 /// killer in	---	100125558 /// 100125560	Mmu.7460.14.S1_s_at_KIR2DL3
Mmu.7460.14.S1_x_at	211242_x_at	KIR2DL4	killer cell immunoglobulin-like receptor, two domains, long cytoplasmic tail, 4	chr19q13.4	3805	KIR2DL5 /// KIR3DL /// KI	killer immunoglobulin-like receptor KIR2DL5 /// killer immunoglobulin-like receptor KIR3DL /// killer im	---	100125559 /// 100125575	Mmu.7460.14.S1_x_at_KIR2DL4
Mmu.7460.15.S1_x_at	211687_x_at	KIR3DL1	killer cell immunoglobulin-like receptor, three domains, long cytoplasmic tail, 1	chr19q13.4	3811	KIR3DL17	killer-cell Ig-like receptor KIR3DL17	---	100125564	Mmu.7460.15.S1_x_at_KIR3DL1
Mmu.7460.17.S1_s_at	NA	NA	NA	NA	NA	NA	NA	NA	NA	Mmu.7460.17.S1_s_at_NA
Mmu.7460.17.S1_x_at	NA	NA	NA	NA	NA	NA	NA	NA	NA	Mmu.7460.17.S1_x_at_NA
Mmu.7460.18.S1_x_at	NA	NA	NA	NA	NA	NA	NA	NA	NA	Mmu.7460.18.S1_x_at_NA
Mmu.7460.1.S1_x_at	NA	NA	NA	NA	NA	NA	NA	NA	NA	Mmu.7460.1.S1_x_at_NA
Mmu.7460.2.S1_x_at	NA	NA	NA	NA	NA	NA	NA	NA	NA	Mmu.7460.2.S1_x_at_NA
Mmu.7460.4.S1_a_at	NA	NA	NA	NA	NA	NA	NA	NA	NA	Mmu.7460.4.S1_a_at_NA
Mmu.7460.4.S1_x_at	NA	NA	NA	NA	NA	NA	NA	NA	NA	Mmu.7460.4.S1_x_at_NA
Mmu.7460.6.S1_x_at	208426_x_at	KIR2DL4	killer cell immunoglobulin-like receptor, two domains, long cytoplasmic tail, 4	chr19q13.4	3805	KIR3DH5	Killer-cell Ig-like receptor KIR3DH5	---	100125563	Mmu.7460.6.S1_x_at_KIR2DL4
Mmu.7460.7.S1_x_at	210890_x_at	KIR2DL1	killer cell immunoglobulin-like receptor, two domains, long cytoplasmic tail, 1	chr19q13.4	3802	KIR3DL	killer immunoglobulin-like receptor KIR3DL	---	100125576	Mmu.7460.7.S1_x_at_KIR2DL1
Mmu.7469.1.S1_at	NA	NA	NA	NA	NA	NA	NA	NA	NA	Mmu.7469.1.S1_at_NA
Mmu.7474.1.S1_at	NA	NA	NA	NA	NA	NA	NA	NA	NA	Mmu.7474.1.S1_at_NA
Mmu.7474.1.S1_x_at	NA	NA	NA	NA	NA	NA	NA	NA	NA	Mmu.7474.1.S1_x_at_NA
Mmu.7476.1.S1_s_at	200869_at	RPL18A /// RPL18AP3	ribosomal protein L18a /// ribosomal protein L18a pseudogene 3	chr12q23.3 /// chr19p13	390354 /// 6142	MFAP5	Microfibrillar associated protein 5	---	716459	Mmu.7476.1.S1_s_at_RPL18A /// RPL1
Mmu.7481.1.A1_at	NA	NA	NA	NA	NA	NA	NA	NA	NA	Mmu.7481.1.A1_at_NA
Mmu.749.1.S1_at	NA	NA	NA	NA	NA	NA	NA	NA	NA	Mmu.749.1.S1_at_NA
Mmu.7507.2.S1_at	NA	NA	NA	NA	NA	NA	NA	NA	NA	Mmu.7507.2.S1_at_NA
Mmu.7520.1.S1_x_at	207529_at	DEFA5	defensin, alpha 5, Paneth cell-specific	chr8pter-p21	1670	LOC574310	alpha-defensin 3 precursor	---	574310	Mmu.7520.1.S1_x_at_DEFA5
Mmu.7528.1.S1_at	NA	NA	NA	NA	NA	NA	NA	NA	NA	Mmu.7528.1.S1_at_NA
Mmu.7531.1.S1_at	NA	NA	NA	NA	NA	NA	NA	NA	NA	Mmu.7531.1.S1_at_NA
Mmu.7535.1.S1_at	NA	NA	NA	NA	NA	NA	NA	NA	NA	Mmu.7535.1.S1_at_NA
Mmu.7543.1.S1_at	NA	NA	NA	NA	NA	NA	NA	NA	NA	Mmu.7543.1.S1_at_NA
Mmu.7546.1.S1_at	NA	NA	NA	NA	NA	NA	NA	NA	NA	Mmu.7546.1.S1_at_NA
Mmu.7561.1.S1_at	NA	NA	NA	NA	NA	NA	NA	NA	NA	Mmu.7561.1.S1_at_NA
Mmu.7599.1.S1_at	NA	NA	NA	NA	NA	NA	NA	NA	NA	Mmu.7599.1.S1_at_NA
Mmu.7601.1.S1_at	NA	NA	NA	NA	NA	NA	NA	NA	NA	Mmu.7601.1.S1_at_NA
Mmu.7605.1.S1_at	NA	NA	NA	NA	NA	NA	NA	NA	NA	Mmu.7605.1.S1_at_NA
Mmu.7638.1.S1_at	NA	NA	NA	NA	NA	NA	NA	NA	NA	Mmu.7638.1.S1_at_NA
Mmu.7639.1.S1_at	NA	NA	NA	NA	NA	NA	NA	NA	NA	Mmu.7639.1.S1_at_NA
Mmu.7643.1.S1_at	NA	NA	NA	NA	NA	NA	NA	NA	NA	Mmu.7643.1.S1_at_NA
Mmu.7649.1.S1_at	NA	NA	NA	NA	NA	NA	NA	NA	NA	Mmu.7649.1.S1_at_NA
Mmu.7660.1.S1_at	NA	NA	NA	NA	NA	NA	NA	NA	NA	Mmu.7660.1.S1_at_NA
Mmu.7663.1.S1_at	NA	NA	NA	NA	NA	NA	NA	NA	NA	Mmu.7663.1.S1_at_NA
Mmu.7667.1.S1_at	NA	NA	NA	NA	NA	NA	NA	NA	NA	Mmu.7667.1.S1_at_NA
Mmu.7673.1.S1_at	NA	NA	NA	NA	NA	NA	NA	NA	NA	Mmu.7673.1.S1_at_NA
Mmu.7683.1.S1_at	NA	NA	NA	NA	NA	NA	NA	NA	NA	Mmu.7683.1.S1_at_NA
Mmu.7710.1.S1_at	NA	NA	NA	NA	NA	NA	NA	NA	NA	Mmu.7710.1.S1_at_NA
Mmu.7716.1.S1_at	NA	NA	NA	NA	NA	NA	NA	NA	NA	Mmu.7716.1.S1_at_NA
Mmu.7717.1.S1_at	NA	NA	NA	NA	NA	NA	NA	NA	NA	Mmu.7717.1.S1_at_NA
Mmu.7730.1.S1_at	NA	NA	NA	NA	NA	NA	NA	NA	NA	Mmu.7730.1.S1_at_NA
Mmu.7746.1.S1_at	NA	NA	NA	NA	NA	NA	NA	NA	NA	Mmu.7746.1.S1_at_NA
Mmu.7752.1.S1_at	NA	NA	NA	NA	NA	NA	NA	NA	NA	Mmu.7752.1.S1_at_NA
Mmu.7758.1.S1_at	NA	NA	NA	NA	NA	NA	NA	NA	NA	Mmu.7758.1.S1_at_NA
Mmu.7792.1.S1_at	NA	NA	NA	NA	NA	NA	NA	NA	NA	Mmu.7792.1.S1_at_NA
Mmu.7801.1.S1_at	NA	NA	NA	NA	NA	NA	NA	NA	NA	Mmu.7801.1.S1_at_NA
Mmu.7805.1.S1_at	NA	NA	NA	NA	NA	NA	NA	NA	NA	Mmu.7805.1.S1_at_NA
Mmu.7831.2.S1_at	NA	NA	NA	NA	NA	NA	NA	NA	NA	Mmu.7831.2.S1_at_NA
Mmu.7836.1.S1_at	NA	NA	NA	NA	NA	NA	NA	NA	NA	Mmu.7836.1.S1_at_NA
Mmu.7836.1.S1_x_at	210564_x_at	CFLAR	CASP8 and FADD-like apoptosis regulator	chr2q33-q34	8837	CFLAR	CASP8 and FADD-like apoptosis regulator	---	574234	Mmu.7836.1.S1_x_at_CFLAR
Mmu.7841.1.S1_at	NA	NA	NA	NA	NA	NA	NA	NA	NA	Mmu.7841.1.S1_at_NA
Mmu.7842.1.S1_at	NA	NA	NA	NA	NA	NA	NA	NA	NA	Mmu.7842.1.S1_at_NA
Mmu.7847.1.S1_s_at	214381_at	LOC441601	septin 7 pseudogene	chr11p11.12	441601	SEPT7	septin 7	---	677666	Mmu.7847.1.S1_s_at_LOC441601
Mmu.7855.1.S1_at	NA	NA	NA	NA	NA	NA	NA	NA	NA	Mmu.7855.1.S1_at_NA
Mmu.7856.1.S1_at	NA	NA	NA	NA	NA	NA	NA	NA	NA	Mmu.7856.1.S1_at_NA
Mmu.7863.1.S1_at	NA	NA	NA	NA	NA	NA	NA	NA	NA	Mmu.7863.1.S1_at_NA
Mmu.7865.1.S1_at	NA	NA	NA	NA	NA	NA	NA	NA	NA	Mmu.7865.1.S1_at_NA
Mmu.7878.1.S1_at	NA	NA	NA	NA	NA	NA	NA	NA	NA	Mmu.7878.1.S1_at_NA
Mmu.7883.1.S1_x_at	201492_s_at	RPL41	ribosomal protein L41	chr12q13	6171	---	---	---	---	Mmu.7883.1.S1_x_at_RPL41
Mmu.7886.1.S1_at	NA	NA	NA	NA	NA	NA	NA	NA	NA	Mmu.7886.1.S1_at_NA
Mmu.7889.1.S1_at	NA	NA	NA	NA	NA	NA	NA	NA	NA	Mmu.7889.1.S1_at_NA
Mmu.789.1.S1_at	NA	NA	NA	NA	NA	NA	NA	NA	NA	Mmu.789.1.S1_at_NA
Mmu.7914.1.S1_at	NA	NA	NA	NA	NA	NA	NA	NA	NA	Mmu.7914.1.S1_at_NA
Mmu.792.1.S1_at	NA	NA	NA	NA	NA	NA	NA	NA	NA	Mmu.792.1.S1_at_NA
Mmu.7922.1.S1_at	NA	NA	NA	NA	NA	NA	NA	NA	NA	Mmu.7922.1.S1_at_NA
Mmu.7922.1.S1_x_at	NA	NA	NA	NA	NA	NA	NA	NA	NA	Mmu.7922.1.S1_x_at_NA
Mmu.7935.1.S1_at	NA	NA	NA	NA	NA	NA	NA	NA	NA	Mmu.7935.1.S1_at_NA
Mmu.7940.1.A1_at	NA	NA	NA	NA	NA	NA	NA	NA	NA	Mmu.7940.1.A1_at_NA
Mmu.7944.1.S1_at	NA	NA	NA	NA	NA	NA	NA	NA	NA	Mmu.7944.1.S1_at_NA
Mmu.7947.1.S1_at	NA	NA	NA	NA	NA	NA	NA	NA	NA	Mmu.7947.1.S1_at_NA
Mmu.7958.1.S1_at	NA	NA	NA	NA	NA	NA	NA	NA	NA	Mmu.7958.1.S1_at_NA
Mmu.7958.1.S1_s_at	NA	NA	NA	NA	NA	NA	NA	NA	NA	Mmu.7958.1.S1_s_at_NA
Mmu.7978.1.S1_at	NA	NA	NA	NA	NA	NA	NA	NA	NA	Mmu.7978.1.S1_at_NA
Mmu.799.1.S1_at	NA	NA	NA	NA	NA	NA	NA	NA	NA	Mmu.799.1.S1_at_NA
Mmu.800.1.S1_at	NA	NA	NA	NA	NA	NA	NA	NA	NA	Mmu.800.1.S1_at_NA
Mmu.8008.1.S1_at	NA	NA	NA	NA	NA	NA	NA	NA	NA	Mmu.8008.1.S1_at_NA
Mmu.8044.1.S1_at	NA	NA	NA	NA	NA	NA	NA	NA	NA	Mmu.8044.1.S1_at_NA
Mmu.8046.1.S1_at	NA	NA	NA	NA	NA	NA	NA	NA	NA	Mmu.8046.1.S1_at_NA
Mmu.8048.1.S1_at	NA	NA	NA	NA	NA	NA	NA	NA	NA	Mmu.8048.1.S1_at_NA
Mmu.8051.1.S1_at	NA	NA	NA	NA	NA	NA	NA	NA	NA	Mmu.8051.1.S1_at_NA
Mmu.8060.2.S1_at	NA	NA	NA	NA	NA	NA	NA	NA	NA	Mmu.8060.2.S1_at_NA
Mmu.8061.1.S1_at	231669_at	SEPP1	Selenoprotein P, plasma, 1	chr5q31	6414	SEPP1	selenoprotein P	---	698917	Mmu.8061.1.S1_at_SEPP1

Mmu.8065.1.S1_at	207641_at	TNFRSF13B	tumor necrosis factor receptor superfamily, member 13B	chr17p11.2		23495	NF-AT	transmembrane activator	---	574200	Mmu.8065.1.S1_at_TNFRSF13B
Mmu.8073.1.S1_at	NA	NA	NA	NA	NA		NA	NA	NA	NA	Mmu.8073.1.S1_at_NA
Mmu.8078.2.S1_at	NA	NA	NA	NA	NA		NA	NA	NA	NA	Mmu.8078.2.S1_at_NA
Mmu.8081.1.S1_at	NA	NA	NA	NA	NA		NA	NA	NA	NA	Mmu.8081.1.S1_at_NA
Mmu.8081.1.S1_x_at	NA	NA	NA	NA	NA		NA	NA	NA	NA	Mmu.8081.1.S1_x_at_NA
Mmu.8084.1.S1_at	NA	NA	NA	NA	NA		NA	NA	NA	NA	Mmu.8084.1.S1_at_NA
Mmu.8090.1.S1_at	NA	NA	NA	NA	NA		NA	NA	NA	NA	Mmu.8090.1.S1_at_NA
Mmu.8100.1.S1_at	NA	NA	NA	NA	NA		NA	NA	NA	NA	Mmu.8100.1.S1_at_NA
Mmu.8117.1.S1_at	NA	NA	NA	NA	NA		NA	NA	NA	NA	Mmu.8117.1.S1_at_NA
Mmu.8122.1.A1_at	NA	NA	NA	NA	NA		NA	NA	NA	NA	Mmu.8122.1.A1_at_NA
Mmu.8122.1.S1_at	NA	NA	NA	NA	NA		NA	NA	NA	NA	Mmu.8122.1.S1_at_NA
Mmu.8123.1.S1_at	NA	NA	NA	NA	NA		NA	NA	NA	NA	Mmu.8123.1.S1_at_NA
Mmu.8137.1.S1_at	NA	NA	NA	NA	NA		NA	NA	NA	NA	Mmu.8137.1.S1_at_NA
Mmu.8143.1.S1_at	NA	NA	NA	NA	NA		NA	NA	NA	NA	Mmu.8143.1.S1_at_NA
Mmu.8145.1.S1_at	NA	NA	NA	NA	NA		NA	NA	NA	NA	Mmu.8145.1.S1_at_NA
Mmu.8147.1.A1_at	NA	NA	NA	NA	NA		NA	NA	NA	NA	Mmu.8147.1.A1_at_NA
Mmu.8149.1.S1_s_at	NA	NA	NA	NA	NA		NA	NA	NA	NA	Mmu.8149.1.S1_s_at_NA
Mmu.8156.1.S1_at	NA	NA	NA	NA	NA		NA	NA	NA	NA	Mmu.8156.1.S1_at_NA
Mmu.8163.1.S1_at	NA	NA	NA	NA	NA		NA	NA	NA	NA	Mmu.8163.1.S1_at_NA
Mmu.8192.1.S1_at	NA	NA	NA	NA	NA		NA	NA	NA	NA	Mmu.8192.1.S1_at_NA
Mmu.8197.1.S1_at	NA	NA	NA	NA	NA		NA	NA	NA	NA	Mmu.8197.1.S1_at_NA
Mmu.8209.1.S1_at	NA	NA	NA	NA	NA		NA	NA	NA	NA	Mmu.8209.1.S1_at_NA
Mmu.8218.1.S1_at	NA	NA	NA	NA	NA		NA	NA	NA	NA	Mmu.8218.1.S1_at_NA
Mmu.8222.1.S1_at	NA	NA	NA	NA	NA		NA	NA	NA	NA	Mmu.8222.1.S1_at_NA
Mmu.8245.1.S1_at	NA	NA	NA	NA	NA		NA	NA	NA	NA	Mmu.8245.1.S1_at_NA
Mmu.826.1.S1_at	NA	NA	NA	NA	NA		NA	NA	NA	NA	Mmu.826.1.S1_at_NA
Mmu.8262.1.S1_at	NA	NA	NA	NA	NA		NA	NA	NA	NA	Mmu.8262.1.S1_at_NA
Mmu.8265.1.S1_at	NA	NA	NA	NA	NA		NA	NA	NA	NA	Mmu.8265.1.S1_at_NA
Mmu.8273.1.S1_at	NA	NA	NA	NA	NA		NA	NA	NA	NA	Mmu.8273.1.S1_at_NA
Mmu.8277.1.S1_at	NA	NA	NA	NA	NA		NA	NA	NA	NA	Mmu.8277.1.S1_at_NA
Mmu.8277.2.S1_at	NA	NA	NA	NA	NA		NA	NA	NA	NA	Mmu.8277.2.S1_at_NA
Mmu.828.1.S1_at	NA	NA	NA	NA	NA		NA	NA	NA	NA	Mmu.828.1.S1_at_NA
Mmu.8286.1.S1_s_at	211969_at	HSP90AA1	heat shock protein 90kDa alpha (cytosolic), class A member 1	chr14q32.33		3320	LOC701865	Similar to kallikrein 13 precursor	---	701865	Mmu.8286.1.S1_s_at_HSP90AA1
Mmu.8299.1.S1_at	NA	NA	NA	NA	NA		NA	NA	NA	NA	Mmu.8299.1.S1_at_NA
Mmu.8299.1.S1_s_at	NA	NA	NA	NA	NA		NA	NA	NA	NA	Mmu.8299.1.S1_s_at_NA
Mmu.8323.1.S1_at	231590_at	GATM	Glycine amidinotransferase (L-arginine:glycine amidinotransferase)	chr15q21.1		2628	GATM	glycine amidinotransferase (L-arginine:glycine amidinotransferase)	---	713306	Mmu.8323.1.S1_at_GATM
Mmu.8324.2.S1_at	NA	NA	NA	NA	NA		NA	NA	NA	NA	Mmu.8324.2.S1_at_NA
Mmu.8324.2.S1_s_at	NA	NA	NA	NA	NA		NA	NA	NA	NA	Mmu.8324.2.S1_s_at_NA
Mmu.8335.1.S1_s_at	NA	NA	NA	NA	NA		NA	NA	NA	NA	Mmu.8335.1.S1_s_at_NA
Mmu.8339.1.S1_at	NA	NA	NA	NA	NA		NA	NA	NA	NA	Mmu.8339.1.S1_at_NA
Mmu.8361.1.S1_at	NA	NA	NA	NA	NA		NA	NA	NA	NA	Mmu.8361.1.S1_at_NA
Mmu.8365.1.S1_at	NA	NA	NA	NA	NA		NA	NA	NA	NA	Mmu.8365.1.S1_at_NA
Mmu.8365.1.S1_s_at	NA	NA	NA	NA	NA		NA	NA	NA	NA	Mmu.8365.1.S1_s_at_NA
Mmu.8378.1.S1_at	NA	NA	NA	NA	NA		NA	NA	NA	NA	Mmu.8378.1.S1_at_NA
Mmu.8380.1.S1_at	NA	NA	NA	NA	NA		NA	NA	NA	NA	Mmu.8380.1.S1_at_NA
Mmu.8395.1.S1_at	NA	NA	NA	NA	NA		NA	NA	NA	NA	Mmu.8395.1.S1_at_NA
Mmu.8402.1.S1_at	NA	NA	NA	NA	NA		NA	NA	NA	NA	Mmu.8402.1.S1_at_NA
Mmu.8410.1.S1_at	NA	NA	NA	NA	NA		NA	NA	NA	NA	Mmu.8410.1.S1_at_NA
Mmu.8428.1.S1_at	NA	NA	NA	NA	NA		NA	NA	NA	NA	Mmu.8428.1.S1_at_NA
Mmu.8473.1.A1_at	NA	NA	NA	NA	NA		NA	NA	NA	NA	Mmu.8473.1.A1_at_NA
Mmu.8473.1.S1_x_at	234883_x_at	TRBV7-3	T cell receptor beta variable 7-3	chr7q34		28595	LOC698913 /// LOC6992	similar to T-cell receptor beta chain V region CTL-L17 precursor /// similar to T-cell receptor beta chai	---	698913 /// 699298	Mmu.8473.1.S1_x_at_TRBV7-3
Mmu.8487.1.S1_at	NA	NA	NA	NA	NA		NA	NA	NA	NA	Mmu.8487.1.S1_at_NA
Mmu.849.1.S1_at	NA	NA	NA	NA	NA		NA	NA	NA	NA	Mmu.849.1.S1_at_NA
Mmu.85.1.S1_at	NA	NA	NA	NA	NA		NA	NA	NA	NA	Mmu.85.1.S1_at_NA
Mmu.8527.2.A1_at	NA	NA	NA	NA	NA		NA	NA	NA	NA	Mmu.8527.2.A1_at_NA
Mmu.8527.2.A1_s_at	219892_at	TM6SF1	transmembrane 6 superfamily member 1	chr15q24-q26		53346	LOC700147	similar to transmembrane 6 superfamily member 1	---	700147	Mmu.8527.2.A1_s_at_TM6SF1
Mmu.8548.1.S1_at	NA	NA	NA	NA	NA		NA	NA	NA	NA	Mmu.8548.1.S1_at_NA
Mmu.8566.2.S1_at	NA	NA	NA	NA	NA		NA	NA	NA	NA	Mmu.8566.2.S1_at_NA
Mmu.8574.2.S1_at	NA	NA	NA	NA	NA		NA	NA	NA	NA	Mmu.8574.2.S1_at_NA
Mmu.8581.1.A1_at	NA	NA	NA	NA	NA		NA	NA	NA	NA	Mmu.8581.1.A1_at_NA
Mmu.8587.1.S1_at	NA	NA	NA	NA	NA		NA	NA	NA	NA	Mmu.8587.1.S1_at_NA
Mmu.8604.1.A1_at	224359_s_at	HOOK3	hook homolog 3 (Drosophila)	chr8p11.21		84376	LOC706093	Similar to golgi-associated microtubule-binding protein HOOK3	---	706093	Mmu.8604.1.A1_at_HOOK3
Mmu.8608.1.S2_at	1405_i_at	CCL5	chemokine (C-C motif) ligand 5	chr17q11.2-q12		6352	CCL5	chemokine (C-C motif) ligand 5	---	574178	Mmu.8608.1.S2_at_CCL5
Mmu.8637.1.S1_at	201525_at	APOD	apolipoprotein D	chr3q26.2-qter		347	---	---	---	---	Mmu.8637.1.S1_at_APOD
Mmu.865.1.S1_at	NA	NA	NA	NA	NA		NA	NA	NA	NA	Mmu.865.1.S1_at_NA
Mmu.8677.1.S1_at	NA	NA	NA	NA	NA		NA	NA	NA	NA	Mmu.8677.1.S1_at_NA
Mmu.8707.1.S1_s_at	NA	NA	NA	NA	NA		NA	NA	NA	NA	Mmu.8707.1.S1_s_at_NA
Mmu.8713.1.S1_at	NA	NA	NA	NA	NA		NA	NA	NA	NA	Mmu.8713.1.S1_at_NA
Mmu.8713.1.S1_s_at	NA	NA	NA	NA	NA		NA	NA	NA	NA	Mmu.8713.1.S1_s_at_NA
Mmu.8716.1.S1_at	NA	NA	NA	NA	NA		NA	NA	NA	NA	Mmu.8716.1.S1_at_NA
Mmu.8731.1.S1_at	NA	NA	NA	NA	NA		NA	NA	NA	NA	Mmu.8731.1.S1_at_NA
Mmu.8731.1.S1_x_at	223042_s_at	FUNDC2	FUN14 domain containing 2	chrXq28		65991	LOC707539	Similar to myeloid cell leukemia sequence 1 isoform 1	---	707539	Mmu.8731.1.S1_x_at_FUNDC2
Mmu.8739.1.S1_at	NA	NA	NA	NA	NA		NA	NA	NA	NA	Mmu.8739.1.S1_at_NA
Mmu.874.1.S1_at	218468_s_at	GREM1	gremlin 1, cysteine knot superfamily, homolog (Xenopus laevis)	chr15q13-q15		26585	GREM1	gremlin	---	574176	Mmu.874.1.S1_at_GREM1
Mmu.8747.1.S1_at	NA	NA	NA	NA	NA		NA	NA	NA	NA	Mmu.8747.1.S1_at_NA
Mmu.8757.1.S1_at	NA	NA	NA	NA	NA		NA	NA	NA	NA	Mmu.8757.1.S1_at_NA
Mmu.8766.1.S1_at	NA	NA	NA	NA	NA		NA	NA	NA	NA	Mmu.8766.1.S1_at_NA
Mmu.8769.1.S1_s_at	NA	NA	NA	NA	NA		NA	NA	NA	NA	Mmu.8769.1.S1_s_at_NA
Mmu.8777.1.S1_s_at	NA	NA	NA	NA	NA		NA	NA	NA	NA	Mmu.8777.1.S1_s_at_NA
Mmu.8778.1.S1_at	NA	NA	NA	NA	NA		NA	NA	NA	NA	Mmu.8778.1.S1_at_NA
Mmu.8787.1.S1_at	NA	NA	NA	NA	NA		NA	NA	NA	NA	Mmu.8787.1.S1_at_NA
Mmu.8794.2.S1_s_at	NA	NA	NA	NA	NA		NA	NA	NA	NA	Mmu.8794.2.S1_s_at_NA
Mmu.8798.1.S1_at	NA	NA	NA	NA	NA		NA	NA	NA	NA	Mmu.8798.1.S1_at_NA
Mmu.8798.1.S1_s_at	NA	NA	NA	NA	NA		NA	NA	NA	NA	Mmu.8798.1.S1_s_at_NA
Mmu.8810.2.S1_at	NA	NA	NA	NA	NA		NA	NA	NA	NA	Mmu.8810.2.S1_at_NA
Mmu.8849.1.S1_at	NA	NA	NA	NA	NA		NA	NA	NA	NA	Mmu.8849.1.S1_at_NA
Mmu.8855.1.S1_at	NA	NA	NA	NA	NA		NA	NA	NA	NA	Mmu.8855.1.S1_at_NA
Mmu.8859.1.S1_at	NA	NA	NA	NA	NA		NA	NA	NA	NA	Mmu.8859.1.S1_at_NA
Mmu.886.1.S1_at	NA	NA	NA	NA	NA		NA	NA	NA	NA	Mmu.886.1.S1_at_NA
Mmu.8876.1.S1_at	NA	NA	NA	NA	NA		NA	NA	NA	NA	Mmu.8876.1.S1_at_NA
Mmu.8887.1.S1_at	NA	NA	NA	NA	NA		NA	NA	NA	NA	Mmu.8887.1.S1_at_NA
Mmu.8893.1.S1_s_at	NA	NA	NA	NA	NA		NA	NA	NA	NA	Mmu.8893.1.S1_s_at_NA
Mmu.8901.1.S1_at	NA	NA	NA	NA	NA		NA	NA	NA	NA	Mmu.8901.1.S1_at_NA
Mmu.8905.1.S1_at	NA	NA	NA	NA	NA		NA	NA	NA	NA	Mmu.8905.1.S1_at_NA
Mmu.891.1.S1_at	NA	NA	NA	NA	NA		NA	NA	NA	NA	Mmu.891.1.S1_at_NA
Mmu.8925.1.S1_at	NA	NA	NA	NA	NA		NA	NA	NA	NA	Mmu.8925.1.S1_at_NA
Mmu.8934.1.S1_at	NA	NA	NA	NA	NA		NA	NA	NA	NA	Mmu.8934.1.S1_at_NA
Mmu.8939.2.S1_at	NA	NA	NA	NA	NA		NA	NA	NA	NA	Mmu.8939.2.S1_at_NA
Mmu.8945.1.S1_at	NA	NA	NA	NA	NA		NA	NA	NA	NA	Mmu.8945.1.S1_at_NA
Mmu.8948.1.S1_at	NA	NA	NA	NA	NA		NA	NA	NA	NA	Mmu.8948.1.S1_at_NA
Mmu.8970.1.S1_at	NA	NA	NA	NA	NA		NA	NA	NA	NA	Mmu.8970.1.S1_at_NA
Mmu.8971.1.S1_at	221726_at	RPL22	ribosomal protein L22	chr1p36.3-p36.2		6146	LOC703050	similar to 60S ribosomal protein L22 (Heparin-binding protein HBp15)	---	703050	Mmu.8971.1.S1_at_RPL22
Mmu.8980.1.S1_at	NA	NA	NA	NA	NA		NA	NA	NA	NA	Mmu.8980.1.S1_at_NA
Mmu.8980.1.S1_s_at	211710_x_at	RPL4	ribosomal protein L4	chr15q22		6124	LOC693472	similar to ribosomal protein L4	---	693472	Mmu.8980.1.S1_s_at_RPL4
Mmu.8984.1.S1_at	NA	NA	NA	NA	NA		NA	NA	NA	NA	Mmu.8984.1.S1_at_NA
Mmu.8988.1.S1_at	NA	NA	NA	NA	NA		NA	NA	NA	NA	Mmu.8988.1.S1_at_NA
Mmu.8999.1.S1_at	NA	NA	NA	NA	NA		NA	NA	NA	NA	Mmu.8999.1.S1_at_NA
Mmu.9008.1.S1_at	NA	NA	NA	NA	NA		NA	NA	NA	NA	Mmu.9008.1.S1_at_NA

Mmu.9015.1.S1_at	NA	NA	NA	NA	NA	NA	NA	NA	NA	Mmu.9015.1.S1_at_NA
Mmu.9017.1.S1_at	NA	NA	NA	NA	NA	NA	NA	NA	NA	Mmu.9017.1.S1_at_NA
Mmu.90.1.S1_at	NA	NA	NA	NA	NA	NA	NA	NA	NA	Mmu.90.1.S1_at_NA
Mmu.9020.1.S1_at	NA	NA	NA	NA	NA	NA	NA	NA	NA	Mmu.9020.1.S1_at_NA
Mmu.9040.1.S1_at	NA	NA	NA	NA	NA	NA	NA	NA	NA	Mmu.9040.1.S1_at_NA
Mmu.9070.1.S1_at	NA	NA	NA	NA	NA	NA	NA	NA	NA	Mmu.9070.1.S1_at_NA
Mmu.9097.1.S1_at	NA	NA	NA	NA	NA	NA	NA	NA	NA	Mmu.9097.1.S1_at_NA
Mmu.9109.1.A1_at	NA	NA	NA	NA	NA	NA	NA	NA	NA	Mmu.9109.1.A1_at_NA
Mmu.9109.1.A1_x_at	NA	NA	NA	NA	NA	NA	NA	NA	NA	Mmu.9109.1.A1_x_at_NA
Mmu.9109.1.S1_at	NA	NA	NA	NA	NA	NA	NA	NA	NA	Mmu.9109.1.S1_at_NA
Mmu.9118.1.S1_at	NA	NA	NA	NA	NA	NA	NA	NA	NA	Mmu.9118.1.S1_at_NA
Mmu.912.1.S1_at	NA	NA	NA	NA	NA	NA	NA	NA	NA	Mmu.912.1.S1_at_NA
Mmu.9127.1.S1_a_at	NA	NA	NA	NA	NA	NA	NA	NA	NA	Mmu.9127.1.S1_a_at_NA
Mmu.915.1.S1_at	NA	NA	NA	NA	NA	NA	NA	NA	NA	Mmu.915.1.S1_at_NA
Mmu.9154.1.S1_at	NA	NA	NA	NA	NA	NA	NA	NA	NA	Mmu.9154.1.S1_at_NA
Mmu.9155.1.S1_at	NA	NA	NA	NA	NA	NA	NA	NA	NA	Mmu.9155.1.S1_at_NA
Mmu.919.1.S1_at	NA	NA	NA	NA	NA	NA	NA	NA	NA	Mmu.919.1.S1_at_NA
Mmu.9198.1.S1_at	NA	NA	NA	NA	NA	NA	NA	NA	NA	Mmu.9198.1.S1_at_NA
Mmu.92.1.S1_at	NA	NA	NA	NA	NA	NA	NA	NA	NA	Mmu.92.1.S1_at_NA
Mmu.9230.1.S1_at	NA	NA	NA	NA	NA	NA	NA	NA	NA	Mmu.9230.1.S1_at_NA
Mmu.9231.1.S1_s_at	NA	NA	NA	NA	NA	NA	NA	NA	NA	Mmu.9231.1.S1_s_at_NA
Mmu.923.1.S1_at	NA	NA	NA	NA	NA	NA	NA	NA	NA	Mmu.923.1.S1_at_NA
Mmu.9239.1.S1_at	NA	NA	NA	NA	NA	NA	NA	NA	NA	Mmu.9239.1.S1_at_NA
Mmu.9241.2.S1_at	209619_at	CD74	CD74 molecule, major histocompatibility complex, class II invariant chain	chr5q32	972	CD74	CD74 molecule, major histocompatibility complex, class II invariant chain	---	710820	Mmu.9241.2.S1_at_CD74
Mmu.924.1.S1_at	NA	NA	NA	NA	NA	NA	NA	NA	NA	Mmu.924.1.S1_at_NA
Mmu.9242.10.S1_x_at	209738_x_at	PSG6	pregnancy specific beta-1-glycoprotein 6	chr19q13.2	5675	PSG2	pregnancy specific beta-1-glycoprotein 2	---	722249	Mmu.9242.10.S1_x_at_PSG6
Mmu.9242.11.S1_x_at	NA	NA	NA	NA	NA	NA	NA	NA	NA	Mmu.9242.11.S1_x_at_NA
Mmu.9242.12.S1_at	NA	NA	NA	NA	NA	NA	NA	NA	NA	Mmu.9242.12.S1_at_NA
Mmu.9242.12.S1_x_at	NA	NA	NA	NA	NA	NA	NA	NA	NA	Mmu.9242.12.S1_x_at_NA
Mmu.9242.1.S1_x_at	NA	NA	NA	NA	NA	NA	NA	NA	NA	Mmu.9242.1.S1_x_at_NA
Mmu.9242.2.S1_at	210196_s_at	PSG1	pregnancy specific beta-1-glycoprotein 1	chr19q13.2	5669	LOC710169 /// LOC7110	similar to pregnancy specific beta-1-glycoprotein 11 isoform 1 /// similar to pregnancy specific beta-1-	---	710169 /// 711053 /// 7222	Mmu.9242.2.S1_at_PSG1
Mmu.9242.2.S1_x_at	NA	NA	NA	NA	NA	NA	NA	NA	NA	Mmu.9242.2.S1_x_at_NA
Mmu.9242.6.S1_x_at	NA	NA	NA	NA	NA	NA	NA	NA	NA	Mmu.9242.6.S1_x_at_NA
Mmu.9242.8.S1_at	NA	NA	NA	NA	NA	NA	NA	NA	NA	Mmu.9242.8.S1_at_NA
Mmu.9244.1.S1_at	NA	NA	NA	NA	NA	NA	NA	NA	NA	Mmu.9244.1.S1_at_NA
Mmu.926.1.S1_at	201931_at	ETFA	electron-transfer-flavoprotein, alpha polypeptide	chr15q23-q25	2108	LOC714874	Hypothetical protein LOC714874	---	714874	Mmu.926.1.S1_at_ETFA
Mmu.9266.1.S1_x_at	NA	NA	NA	NA	NA	NA	NA	NA	NA	Mmu.9266.1.S1_x_at_NA
Mmu.9306.1.S1_at	NA	NA	NA	NA	NA	NA	NA	NA	NA	Mmu.9306.1.S1_at_NA
Mmu.9308.1.S1_at	NA	NA	NA	NA	NA	NA	NA	NA	NA	Mmu.9308.1.S1_at_NA
Mmu.9309.1.S1_at	NA	NA	NA	NA	NA	NA	NA	NA	NA	Mmu.9309.1.S1_at_NA
Mmu.9314.1.S1_at	NA	NA	NA	NA	NA	NA	NA	NA	NA	Mmu.9314.1.S1_at_NA
Mmu.9316.1.S1_at	NA	NA	NA	NA	NA	NA	NA	NA	NA	Mmu.9316.1.S1_at_NA
Mmu.9324.1.S1_at	NA	NA	NA	NA	NA	NA	NA	NA	NA	Mmu.9324.1.S1_at_NA
Mmu.9324.2.S1_a_at	NA	NA	NA	NA	NA	NA	NA	NA	NA	Mmu.9324.2.S1_a_at_NA
Mmu.9324.2.S1_at	NA	NA	NA	NA	NA	NA	NA	NA	NA	Mmu.9324.2.S1_at_NA
Mmu.9329.1.S1_at	NA	NA	NA	NA	NA	NA	NA	NA	NA	Mmu.9329.1.S1_at_NA
Mmu.9335.1.S1_at	NA	NA	NA	NA	NA	NA	NA	NA	NA	Mmu.9335.1.S1_at_NA
Mmu.9362.1.S1_at	NA	NA	NA	NA	NA	NA	NA	NA	NA	Mmu.9362.1.S1_at_NA
Mmu.937.1.S1_at	NA	NA	NA	NA	NA	NA	NA	NA	NA	Mmu.937.1.S1_at_NA
Mmu.9375.1.S1_at	NA	NA	NA	NA	NA	NA	NA	NA	NA	Mmu.9375.1.S1_at_NA
Mmu.9409.1.S1_at	NA	NA	NA	NA	NA	NA	NA	NA	NA	Mmu.9409.1.S1_at_NA
Mmu.9420.1.S1_at	NA	NA	NA	NA	NA	NA	NA	NA	NA	Mmu.9420.1.S1_at_NA
Mmu.9425.2.S1_at	NA	NA	NA	NA	NA	NA	NA	NA	NA	Mmu.9425.2.S1_at_NA
Mmu.9437.1.S1_at	NA	NA	NA	NA	NA	NA	NA	NA	NA	Mmu.9437.1.S1_at_NA
Mmu.9444.1.S1_at	NA	NA	NA	NA	NA	NA	NA	NA	NA	Mmu.9444.1.S1_at_NA
Mmu.9460.1.S1_s_at	NA	NA	NA	NA	NA	NA	NA	NA	NA	Mmu.9460.1.S1_s_at_NA
Mmu.9470.1.A1_x_at	NA	NA	NA	NA	NA	NA	NA	NA	NA	Mmu.9470.1.A1_x_at_NA
Mmu.9470.1.S1_x_at	NA	NA	NA	NA	NA	NA	NA	NA	NA	Mmu.9470.1.S1_x_at_NA
Mmu.9470.2.A1_at	NA	NA	NA	NA	NA	NA	NA	NA	NA	Mmu.9470.2.A1_at_NA
Mmu.9470.2.S1_at	NA	NA	NA	NA	NA	NA	NA	NA	NA	Mmu.9470.2.S1_at_NA
Mmu.9470.4.A1_x_at	NA	NA	NA	NA	NA	NA	NA	NA	NA	Mmu.9470.4.A1_x_at_NA
Mmu.9470.4.S1_x_at	NA	NA	NA	NA	NA	NA	NA	NA	NA	Mmu.9470.4.S1_x_at_NA
Mmu.9481.2.S1_a_at	NA	NA	NA	NA	NA	NA	NA	NA	NA	Mmu.9481.2.S1_a_at_NA
Mmu.9497.1.S1_at	NA	NA	NA	NA	NA	NA	NA	NA	NA	Mmu.9497.1.S1_at_NA
Mmu.9501.1.S1_at	NA	NA	NA	NA	NA	NA	NA	NA	NA	Mmu.9501.1.S1_at_NA
Mmu.952.1.S1_at	NA	NA	NA	NA	NA	NA	NA	NA	NA	Mmu.952.1.S1_at_NA
Mmu.9527.1.S1_s_at	232224_at	MASP1	mannan-binding lectin serine peptidase 1 (C4/C2 activating component of Ra-reactive factor)	chr3q27-q28	5648	LOC708206	similar to mannan-binding lectin serine protease 1 isoform 2 precursor	---	708206	Mmu.9527.1.S1_s_at_MASP1
Mmu.954.1.S1_at	NA	NA	NA	NA	NA	NA	NA	NA	NA	Mmu.954.1.S1_at_NA
Mmu.9543.1.S1_at	NA	NA	NA	NA	NA	NA	NA	NA	NA	Mmu.9543.1.S1_at_NA
Mmu.9549.1.S1_at	NA	NA	NA	NA	NA	NA	NA	NA	NA	Mmu.9549.1.S1_at_NA
Mmu.9550.1.S1_at	NA	NA	NA	NA	NA	NA	NA	NA	NA	Mmu.9550.1.S1_at_NA
Mmu.9550.2.S1_at	NA	NA	NA	NA	NA	NA	NA	NA	NA	Mmu.9550.2.S1_at_NA
Mmu.9557.1.S1_at	NA	NA	NA	NA	NA	NA	NA	NA	NA	Mmu.9557.1.S1_at_NA
Mmu.9567.1.S1_at	NA	NA	NA	NA	NA	NA	NA	NA	NA	Mmu.9567.1.S1_at_NA
Mmu.9589.1.S1_at	NA	NA	NA	NA	NA	NA	NA	NA	NA	Mmu.9589.1.S1_at_NA
Mmu.9596.1.S1_at	NA	NA	NA	NA	NA	NA	NA	NA	NA	Mmu.9596.1.S1_at_NA
Mmu.9614.1.A1_at	NA	NA	NA	NA	NA	NA	NA	NA	NA	Mmu.9614.1.A1_at_NA
Mmu.9614.1.S1_at	211146_at	HLA-C	Major histocompatibility complex, class I, C	chr6p21.3	3107	LOC701546 /// LOC7068	similar to Ig kappa chain V-II region RPMI 6410 precursor /// similar to Ig kappa chain V-II region Cur	---	701546 /// 706869 /// 7086	Mmu.9614.1.S1_at_HLA-C
Mmu.9632.1.A1_at	NA	NA	NA	NA	NA	NA	NA	NA	NA	Mmu.9632.1.A1_at_NA
Mmu.964.1.S1_at	NA	NA	NA	NA	NA	NA	NA	NA	NA	Mmu.964.1.S1_at_NA
Mmu.9654.1.S1_at	NA	NA	NA	NA	NA	NA	NA	NA	NA	Mmu.9654.1.S1_at_NA
Mmu.9655.1.S1_at	NA	NA	NA	NA	NA	NA	NA	NA	NA	Mmu.9655.1.S1_at_NA
Mmu.9670.2.S1_at	NA	NA	NA	NA	NA	NA	NA	NA	NA	Mmu.9670.2.S1_at_NA
Mmu.9670.2.S1_x_at	NA	NA	NA	NA	NA	NA	NA	NA	NA	Mmu.9670.2.S1_x_at_NA
Mmu.967.1.S1_s_at	NA	NA	NA	NA	NA	NA	NA	NA	NA	Mmu.967.1.S1_s_at_NA
Mmu.9683.1.S1_at	NA	NA	NA	NA	NA	NA	NA	NA	NA	Mmu.9683.1.S1_at_NA
Mmu.9686.1.S1_at	NA	NA	NA	NA	NA	NA	NA	NA	NA	Mmu.9686.1.S1_at_NA
Mmu.9689.2.S1_at	NA	NA	NA	NA	NA	NA	NA	NA	NA	Mmu.9689.2.S1_at_NA
Mmu.9711.1.S1_at	NA	NA	NA	NA	NA	NA	NA	NA	NA	Mmu.9711.1.S1_at_NA
Mmu.971.2.S1_at	NA	NA	NA	NA	NA	NA	NA	NA	NA	Mmu.971.2.S1_at_NA
Mmu.9720.1.S1_at	NA	NA	NA	NA	NA	NA	NA	NA	NA	Mmu.9720.1.S1_at_NA
Mmu.9731.1.S1_at	NA	NA	NA	NA	NA	NA	NA	NA	NA	Mmu.9731.1.S1_at_NA
Mmu.9738.1.S1_at	NA	NA	NA	NA	NA	NA	NA	NA	NA	Mmu.9738.1.S1_at_NA
Mmu.9738.1.S1_s_at	NA	NA	NA	NA	NA	NA	NA	NA	NA	Mmu.9738.1.S1_s_at_NA
Mmu.9769.1.S1_s_at	229505_at	---	---	---	---	NAP1L4	nucleosome assembly protein 1-like 4	---	706266	Mmu.9769.1.S1_s_at_---
Mmu.9771.1.S1_at	NA	NA	NA	NA	NA	NA	NA	NA	NA	Mmu.9771.1.S1_at_NA
Mmu.9773.1.S1_at	NA	NA	NA	NA	NA	NA	NA	NA	NA	Mmu.9773.1.S1_at_NA
Mmu.9777.1.S1_a_at	NA	NA	NA	NA	NA	NA	NA	NA	NA	Mmu.9777.1.S1_a_at_NA
Mmu.9792.1.S1_at	NA	NA	NA	NA	NA	NA	NA	NA	NA	Mmu.9792.1.S1_at_NA
Mmu.9793.1.S1_at	NA	NA	NA	NA	NA	NA	NA	NA	NA	Mmu.9793.1.S1_at_NA
Mmu.9798.1.S1_at	NA	NA	NA	NA	NA	NA	NA	NA	NA	Mmu.9798.1.S1_at_NA
Mmu.9802.1.S1_at	NA	NA	NA	NA	NA	NA	NA	NA	NA	Mmu.9802.1.S1_at_NA
Mmu.9805.1.S1_at	NA	NA	NA	NA	NA	NA	NA	NA	NA	Mmu.9805.1.S1_at_NA
Mmu.9808.1.S1_s_at	NA	NA	NA	NA	NA	NA	NA	NA	NA	Mmu.9808.1.S1_s_at_NA
Mmu.9831.1.S1_at	NA	NA	NA	NA	NA	NA	NA	NA	NA	Mmu.9831.1.S1_at_NA
Mmu.9837.1.S1_at	NA	NA	NA	NA	NA	NA	NA	NA	NA	Mmu.9837.1.S1_at_NA
Mmu.9862.2.S1_at	NA	NA	NA	NA	NA	NA	NA	NA	NA	Mmu.9862.2.S1_at_NA
Mmu.9866.1.S1_s_at	NA	NA	NA	NA	NA	NA	NA	NA	NA	Mmu.9866.1.S1_s_at_NA

Mmu.9876.2.S1_at	NA	NA	NA	NA	NA	NA	NA	NA	NA	Mmu.9876.2.S1_at_NA
Mmu.9888.1.S1_at	NA	NA	NA	NA	NA	NA	NA	NA	NA	Mmu.9888.1.S1_at_NA
Mmu.9888.1.S1_s_at	NA	NA	NA	NA	NA	NA	NA	NA	NA	Mmu.9888.1.S1_s_at_NA
Mmu.9951.1.S1_at	NA	NA	NA	NA	NA	NA	NA	NA	NA	Mmu.9951.1.S1_at_NA
Mmu.9961.1.S1_at	NA	NA	NA	NA	NA	NA	NA	NA	NA	Mmu.9961.1.S1_at_NA
Mmu.9964.1.S1_at	NA	NA	NA	NA	NA	NA	NA	NA	NA	Mmu.9964.1.S1_at_NA
Mmu.9975.10.S1_x_at	NA	NA	NA	NA	NA	NA	NA	NA	NA	Mmu.9975.10.S1_x_at_NA
Mmu.9975.17.S1_at	NA	NA	NA	NA	NA	NA	NA	NA	NA	Mmu.9975.17.S1_at_NA
Mmu.9989.1.S1_s_at	NA	NA	NA	NA	NA	NA	NA	NA	NA	Mmu.9989.1.S1_s_at_NA
MmuAffx.1008.1.S1_at	NA	NA	NA	NA	NA	NA	NA	NA	NA	MmuAffx.1008.1.S1_at_NA
MmuAffx.10.1.A1_x_at	NA	NA	NA	NA	NA	NA	NA	NA	NA	MmuAffx.10.1.A1_x_at_NA
MmuAffx.11.1.A1_at	NA	NA	NA	NA	NA	NA	NA	NA	NA	MmuAffx.11.1.A1_at_NA
MmuAffx.11.1.S1_x_at	NA	NA	NA	NA	NA	NA	NA	NA	NA	MmuAffx.11.1.S1_x_at_NA
MmuAffx.1125.1.S1_at	NA	NA	NA	NA	NA	NA	NA	NA	NA	MmuAffx.1125.1.S1_at_NA
MmuAffx.1186.1.S1_at	NA	NA	NA	NA	NA	NA	NA	NA	NA	MmuAffx.1186.1.S1_at_NA
MmuAffx.1.1.S1_at	207893_at	SRY	sex determining region Y	chrYp11.3	6736	SRY	sex determining region Y	---	574155	MmuAffx.1.1.S1_at_SRY
MmuAffx.12.1.A1_at	NA	NA	NA	NA	NA	NA	NA	NA	NA	MmuAffx.12.1.A1_at_NA
MmuAffx.12.1.S1_x_at	NA	NA	NA	NA	NA	NA	NA	NA	NA	MmuAffx.12.1.S1_x_at_NA
MmuAffx.1252.1.A1_at	NA	NA	NA	NA	NA	NA	NA	NA	NA	MmuAffx.1252.1.A1_at_NA
MmuAffx.1533.1.S1_at	NA	NA	NA	NA	NA	NA	NA	NA	NA	MmuAffx.1533.1.S1_at_NA
MmuAffx.161.1.S1_at	204416_x_at	APOC1	apolipoprotein C-I	chr19q13.2	341	LOC706963	Similar to profilin 1	---	706963	MmuAffx.161.1.S1_at_APOC1
MmuAffx.18.1.A1_at	NA	NA	NA	NA	NA	NA	NA	NA	NA	MmuAffx.18.1.A1_at_NA
MmuAffx.18.1.S1_x_at	NA	NA	NA	NA	NA	NA	NA	NA	NA	MmuAffx.18.1.S1_x_at_NA
MmuAffx.19.1.A1_at	NA	NA	NA	NA	NA	NA	NA	NA	NA	MmuAffx.19.1.A1_at_NA
MmuAffx.19.1.S1_s_at	NA	NA	NA	NA	NA	NA	NA	NA	NA	MmuAffx.19.1.S1_s_at_NA
MmuAffx.204.1.A1_at	NA	NA	NA	NA	NA	NA	NA	NA	NA	MmuAffx.204.1.A1_at_NA
MmuAffx.204.1.S1_at	NA	NA	NA	NA	NA	NA	NA	NA	NA	MmuAffx.204.1.S1_at_NA
MmuAffx.2095.1.S1_at	NA	NA	NA	NA	NA	NA	NA	NA	NA	MmuAffx.2095.1.S1_at_NA
MmuAffx.21.1.A1_at	NA	NA	NA	NA	NA	NA	NA	NA	NA	MmuAffx.21.1.A1_at_NA
MmuAffx.21.1.S1_at	NA	NA	NA	NA	NA	NA	NA	NA	NA	MmuAffx.21.1.S1_at_NA
MmuAffx.2165.1.A1_at	NA	NA	NA	NA	NA	NA	NA	NA	NA	MmuAffx.2165.1.A1_at_NA
MmuAffx.2165.1.S1_at	NA	NA	NA	NA	NA	NA	NA	NA	NA	MmuAffx.2165.1.S1_at_NA
MmuAffx.2166.1.A1_a_at	NA	NA	NA	NA	NA	NA	NA	NA	NA	MmuAffx.2166.1.A1_a_at_NA
MmuAffx.2166.1.S1_at	NA	NA	NA	NA	NA	NA	NA	NA	NA	MmuAffx.2166.1.S1_at_NA
MmuAffx.2166.1.S1_x_at	234396_at	---	---	---	---	LOC710951 /// LOC7113	similar to T-cell receptor alpha chain C region /// similar to T-cell receptor alpha chain V region CTL-L	---	710951 /// 711302 /// 711	MmuAffx.2166.1.S1_x_at_---
MmuAffx.2166.2.S1_at	NA	NA	NA	NA	NA	NA	NA	NA	NA	MmuAffx.2166.2.S1_at_NA
MmuAffx.2166.2.S1_x_at	NA	NA	NA	NA	NA	NA	NA	NA	NA	MmuAffx.2166.2.S1_x_at_NA
MmuAffx.2168.1.A1_at	NA	NA	NA	NA	NA	NA	NA	NA	NA	MmuAffx.2168.1.A1_at_NA
MmuAffx.2168.1.S1_at	NA	NA	NA	NA	NA	NA	NA	NA	NA	MmuAffx.2168.1.S1_at_NA
MmuAffx.2168.1.S1_x_at	NA	NA	NA	NA	NA	NA	NA	NA	NA	MmuAffx.2168.1.S1_x_at_NA
MmuAffx.2169.1.S1_at	NA	NA	NA	NA	NA	NA	NA	NA	NA	MmuAffx.2169.1.S1_at_NA
MmuAffx.2170.1.A1_at	NA	NA	NA	NA	NA	NA	NA	NA	NA	MmuAffx.2170.1.A1_at_NA
MmuAffx.2170.1.S1_at	NA	NA	NA	NA	NA	NA	NA	NA	NA	MmuAffx.2170.1.S1_at_NA
MmuAffx.2181.1.S1_s_at	NA	NA	NA	NA	NA	NA	NA	NA	NA	MmuAffx.2181.1.S1_s_at_NA
MmuAffx.2.1.S1_at	207356_at	DEFB4	defensin, beta 4	chr8p23.1-p22	1673	DEFB4	defensin, beta 4	---	574144	MmuAffx.2.1.S1_at_DEFB4
MmuAffx.22.1.S1_at	NA	NA	NA	NA	NA	NA	NA	NA	NA	MmuAffx.22.1.S1_at_NA
MmuAffx.225.1.A1_x_at	NA	NA	NA	NA	NA	NA	NA	NA	NA	MmuAffx.225.1.A1_x_at_NA
MmuAffx.225.1.S1_at	NA	NA	NA	NA	NA	NA	NA	NA	NA	MmuAffx.225.1.S1_at_NA
MmuAffx.228.1.A1_at	NA	NA	NA	NA	NA	NA	NA	NA	NA	MmuAffx.228.1.A1_at_NA
MmuAffx.228.1.S1_x_at	NA	NA	NA	NA	NA	NA	NA	NA	NA	MmuAffx.228.1.S1_x_at_NA
MmuAffx.23.12.S1_at	202409_at	IGF2 /// INS-IGF2	insulin-like growth factor 2 (somatomedin A) /// INS-IGF2 readthrough transcript	chr11p15.5	3481 /// 723961	---	---	---	---	MmuAffx.23.12.S1_at_IGF2 /// INS-IGF2
MmuAffx.23.12.S1_x_at	NA	NA	NA	NA	NA	NA	NA	NA	NA	MmuAffx.23.12.S1_x_at_NA
MmuAffx.23.13.S1_s_at	224646_x_at	H19	H19, imprinted maternally expressed transcript (non-protein coding)	chr11p15.5	283120	H19	H19, imprinted maternally expressed transcript (non-protein coding)	---	704308	MmuAffx.23.13.S1_s_at_H19
MmuAffx.23.14.S1_at	NA	NA	NA	NA	NA	NA	NA	NA	NA	MmuAffx.23.14.S1_at_NA
MmuAffx.23.14.S1_s_at	NA	NA	NA	NA	NA	NA	NA	NA	NA	MmuAffx.23.14.S1_s_at_NA
MmuAffx.23.16.S1_s_at	NA	NA	NA	NA	NA	NA	NA	NA	NA	MmuAffx.23.16.S1_s_at_NA
MmuAffx.23.18.S1_s_at	NA	NA	NA	NA	NA	NA	NA	NA	NA	MmuAffx.23.18.S1_s_at_NA
MmuAffx.23.21.S1_s_at	NA	NA	NA	NA	NA	NA	NA	NA	NA	MmuAffx.23.21.S1_s_at_NA
MmuAffx.23.22.S1_at	NA	NA	NA	NA	NA	NA	NA	NA	NA	MmuAffx.23.22.S1_at_NA
MmuAffx.23.23.S1_s_at	NA	NA	NA	NA	NA	NA	NA	NA	NA	MmuAffx.23.23.S1_s_at_NA
MmuAffx.23.30.S1_at	NA	NA	NA	NA	NA	NA	NA	NA	NA	MmuAffx.23.30.S1_at_NA
MmuAffx.23.31.S1_at	NA	NA	NA	NA	NA	NA	NA	NA	NA	MmuAffx.23.31.S1_at_NA
MmuAffx.23.32.S1_at	NA	NA	NA	NA	NA	NA	NA	NA	NA	MmuAffx.23.32.S1_at_NA
MmuAffx.23.33.S1_at	NA	NA	NA	NA	NA	NA	NA	NA	NA	MmuAffx.23.33.S1_at_NA
MmuAffx.23.35.S1_at	NA	NA	NA	NA	NA	NA	NA	NA	NA	MmuAffx.23.35.S1_at_NA
MmuAffx.23.3.S1_s_at	NA	NA	NA	NA	NA	NA	NA	NA	NA	MmuAffx.23.3.S1_s_at_NA
MmuAffx.23.8.S1_s_at	NA	NA	NA	NA	NA	NA	NA	NA	NA	MmuAffx.23.8.S1_s_at_NA
MmuAffx.259.1.A1_at	NA	NA	NA	NA	NA	NA	NA	NA	NA	MmuAffx.259.1.A1_at_NA
MmuAffx.259.1.S1_x_at	NA	NA	NA	NA	NA	NA	NA	NA	NA	MmuAffx.259.1.S1_x_at_NA
MmuAffx.396.1.A1_at	NA	NA	NA	NA	NA	NA	NA	NA	NA	MmuAffx.396.1.A1_at_NA
MmuAffx.52.1.A1_at	NA	NA	NA	NA	NA	NA	NA	NA	NA	MmuAffx.52.1.A1_at_NA
MmuAffx.52.1.S1_s_at	204103_at	CCL4	chemokine (C-C motif) ligand 4	chr17q12	6351	CCL4L1	chemokine (C-C motif) ligand 4-like 1	---	574217	MmuAffx.52.1.S1_s_at_CCL4
MmuAffx.730.1.A1_at	NA	NA	NA	NA	NA	NA	NA	NA	NA	MmuAffx.730.1.A1_at_NA
MmuAffx.738.1.S1_at	NA	NA	NA	NA	NA	NA	NA	NA	NA	MmuAffx.738.1.S1_at_NA
MmuAffx.78.1.S1_s_at	NA	NA	NA	NA	NA	NA	NA	NA	NA	MmuAffx.78.1.S1_s_at_NA
MmuAffx.8.1.A1_at	NA	NA	NA	NA	NA	NA	NA	NA	NA	MmuAffx.8.1.A1_at_NA
MmuAffx.8.1.S1_at	NA	NA	NA	NA	NA	NA	NA	NA	NA	MmuAffx.8.1.S1_at_NA
MmuAffx.84.1.S1_at	NA	NA	NA	NA	NA	NA	NA	NA	NA	MmuAffx.84.1.S1_at_NA
MmuAffx.873.1.A1_at	NA	NA	NA	NA	NA	NA	NA	NA	NA	MmuAffx.873.1.A1_at_NA
MmuAffx.873.1.S1_at	202367_at	CUX1	cut-like homeobox 1	chr7q22.1	1523	CUTL1	cut-like 1	---	574390	MmuAffx.873.1.S1_at_CUX1
MmuAffx.885.1.S1_at	NA	NA	NA	NA	NA	NA	NA	NA	NA	MmuAffx.885.1.S1_at_NA
MmuAffx.888.1.A1_at	NA	NA	NA	NA	NA	NA	NA	NA	NA	MmuAffx.888.1.A1_at_NA
MmuAffx.888.1.S1_at	NA	NA	NA	NA	NA	NA	NA	NA	NA	MmuAffx.888.1.S1_at_NA
MmuAffx.914.1.S1_at	NA	NA	NA	NA	NA	NA	NA	NA	NA	MmuAffx.914.1.S1_at_NA
MmuAffx.949.1.A1_at	NA	NA	NA	NA	NA	NA	NA	NA	NA	MmuAffx.949.1.A1_at_NA
MmuAffx.949.1.S1_x_at	NA	NA	NA	NA	NA	NA	NA	NA	NA	MmuAffx.949.1.S1_x_at_NA
MmuAffx.951.1.S1_at	NA	NA	NA	NA	NA	NA	NA	NA	NA	MmuAffx.951.1.S1_at_NA
MmuAffx.951.1.S1_x_at	NA	NA	NA	NA	NA	NA	NA	NA	NA	MmuAffx.951.1.S1_x_at_NA
MmuAffx.956.1.S1_at	208675_s_at	DDOST	dolichyl-diphosphooligosaccharide-protein glycosyltransferase	chr1p36.1	1650	---	---	---	---	MmuAffx.956.1.S1_at_DDOST
MmugDNA.10000.1.S1_at	208829_at	TAPBP	TAP binding protein (tapasin)	chr6p21.3	6892	TAPBP	TAP binding protein (tapasin)	---	718235	MmugDNA.10000.1.S1_at_TAPBP
MmugDNA.10001.1.S1_at	225205_at	KIF3B	kinesin family member 3B	chr20q11.21	9371	KIF3B	kinesin family member 3B	---	711883	MmugDNA.10001.1.S1_at_KIF3B
MmugDNA.1000.1.S1_at	234442_at	---	---	---	---	---	---	---	---	MmugDNA.1000.1.S1_at_---
MmugDNA.10002.1.S1_at	1566042_at	---	---	---	---	---	---	---	---	MmugDNA.10002.1.S1_at_---
MmugDNA.10003.1.S1_at	1556107_at	---	---	---	---	---	---	---	---	MmugDNA.10003.1.S1_at_---
MmugDNA.10004.1.S1_at	1553850_at	C21orf88	chromosome 21 open reading frame 88	chr21q22.2	114041	---	---	---	---	MmugDNA.10004.1.S1_at_C21orf88
MmugDNA.10005.1.S1_at	207838_x_at	PBXIP1	pre-B-cell leukemia homeobox interacting protein 1	chr1q21.3	57326	LOC717036	similar to pre-B-cell leukemia transcription factor interacting protein 1	---	717036	MmugDNA.10005.1.S1_at_PBXIP1
MmugDNA.10006.1.S1_at	200058_s_at	SNRNP200	small nuclear ribonucleoprotein 200kDa (U5)	chr2q11.2	23020	SNRNP200	small nuclear ribonucleoprotein 200kDa (U5)	---	705184	MmugDNA.10006.1.S1_at_SNRNP200
MmugDNA.10007.1.S1_at	221165_s_at	IL22	interleukin 22	chr12q15	50616	LOC718047	similar to interleukin 22	---	718047	MmugDNA.10007.1.S1_at_IL22
MmugDNA.10008.1.S1_at	209124_at	MYD88	myeloid differentiation primary response gene (88)	chr3p22	4615	ABP1	Amiloride binding protein 1	---	714112	MmugDNA.10008.1.S1_at_MYD88
MmugDNA.10009.1.S1_at	235615_at	PGGT1B	protein geranylgeranyltransferase type I, beta subunit	chr5q22.3	5229	LOC696437	similar to Geranylgeranyl transferase type-1 beta subunit (Geranylgeranyl transferase type I beta sub	---	696437	MmugDNA.10009.1.S1_at_PGGT1B
MmugDNA.10010.1.S1_at	240685_at	---	---	---	---	---	---	---	---	MmugDNA.10010.1.S1_at_---
MmugDNA.10011.1.S1_at	235178_x_at	ESCO2	establishment of cohesion 1 homolog 2 (S. cerevisiae)	chr8p21.1	157570	LOC713186	similar to establishment of cohesion 1 homolog 2	---	713186	MmugDNA.10011.1.S1_at_ESCO2
MmugDNA.1001.1.S1_s_at	205391_x_at	ANK1	ankyrin 1, erythrocytic	chr8p11.1	286	LOC710904	similar to ankyrin 1 isoform 1	---	710904	MmugDNA.1001.1.S1_s_at_ANK1
MmugDNA.10012.1.S1_at	239883_s_at	ANO4	Anoctamin 4	chr12q23.1-q23.2	121601	CXCL13	Chemokine (C-X-C motif) ligand 13	---	574224	MmugDNA.10012.1.S1_at_ANO4
MmugDNA.10013.1.S1_s_at	211282_x_at	TNFRSF25	tumor necrosis factor receptor superfamily, member 25	chr1p36.2	8718	TNFRSF25	tumor necrosis factor receptor superfamily, member 25	---	703744	MmugDNA.10013.1.S1_s_at_TNFRSF2
MmugDNA.10014.1.S1_s_at	213118_at	UHRF1BP1L	UHRF1 binding protein 1-like	chr12q23.1	23074	UHRF1BP1L	UHRF1 binding protein 1-like	---	694816	MmugDNA.10014.1.S1_s_at_UHRF1BP
MmugDNA.10015.1.S1_at	213120_at	UHRF1BP1L	UHRF1 binding protein 1-like	chr12q23.1	23074	---	---	---	---	MmugDNA.10015.1.S1_at_UHRF1BP1L
MmugDNA.10016.1.S1_at	214398_s_at	IKBKE	inhibitor of kappa light polypeptide gene enhancer in B-cells, kinase epsilon	chr1q32.1	9641	IKBKE	inhibitor of kappa light polypeptide gene enhancer in B-cells, kinase epsilon	---	695607	MmugDNA.10016.1.S1_at_IKBKE

MmugDNA.10017.1.S1_at	241114_s_at	---	---	---	---	---	---	---	---	MmugDNA.10017.1.S1_at_---
MmugDNA.10018.1.S1_at	203608_at	ALDH5A1	aldehyde dehydrogenase 5 family, member A1	chr6p22.2-p22.3	7915	---	---	---	---	MmugDNA.10018.1.S1_at_ALDH5A1
MmugDNA.10019.1.S1_at	209852_x_at	PSME3	proteasome (prosome, macropain) activator subunit 3 (PA28 gamma; Ki)	chr17q21	10197	NUTF2	Similar to Nuclear transport factor 2 (NTF-2)	---	695785	MmugDNA.10019.1.S1_at_PSME3
MmugDNA.100.1.S1_at	212806_at	PRUNE2	prune homolog 2 (Drosophila)	chr9q21.13	158471	---	---	---	---	MmugDNA.100.1.S1_at_PRUNE2
MmugDNA.10020.1.S1_at	1554634_at	PDS5B	PDS5, regulator of cohesion maintenance, homolog B (S. cerevisiae)	chr13q12.3	23047	---	---	---	---	MmugDNA.10020.1.S1_at_PDS5B
MmugDNA.10021.1.S1_at	221159_at	---	---	---	---	---	---	---	---	MmugDNA.10021.1.S1_at_---
MmugDNA.1002.1.S1_at	1554513_s_at	CCDC123	coiled-coil domain containing 123	chr19q13.11	84902	LOC703199	similar to citron	---	703199	MmugDNA.1002.1.S1_at_CCDC123
MmugDNA.10022.1.S1_at	1566541_at	---	---	---	---	---	---	---	---	MmugDNA.10022.1.S1_at_---
MmugDNA.10023.1.S1_at	208278_s_at	---	---	---	---	---	---	---	---	MmugDNA.10023.1.S1_at_---
MmugDNA.10024.1.S1_at	235287_at	CDK6	cyclin-dependent kinase 6	chr7q21-q22	1021	---	---	---	---	MmugDNA.10024.1.S1_at_CDK6
MmugDNA.10025.1.S1_at	202552_s_at	CRIM1	cysteine rich transmembrane BMP regulator 1 (chordin-like)	chr2p21	51232	LOC705063	Similar to nitric oxide synthase trafficking isoform 1	---	705063	MmugDNA.10025.1.S1_at_CRIM1
MmugDNA.10026.1.S1_at	238047_at	RP13-102H20.1	hypothetical protein FLJ30058	chrXq26.1	158763	LOC705650	similar to Rho-GTPase-activating protein 6 (Rho-type GTPase-activating protein RhoGAPX-1)	---	705650	MmugDNA.10026.1.S1_at_RP13-102H2
MmugDNA.10027.1.S1_at	240606_at	---	---	---	---	---	---	---	---	MmugDNA.10027.1.S1_at_---
MmugDNA.10028.1.S1_s_at	209615_s_at	PAK1	p21 protein (Cdc42/Rac)-activated kinase 1	chr11q13-q14	5058	LOC698585	similar to p21-activated kinase 1	---	698585	MmugDNA.10028.1.S1_s_at_PAK1
MmugDNA.10029.1.S1_at	1555388_s_at	SNX25	sorting nexin 25	chr4q35.1	83891	LOC699548	similar to sorting nexin 25	---	699548	MmugDNA.10029.1.S1_at_SNX25
MmugDNA.10030.1.S1_at	244139_s_at	---	---	---	---	---	---	---	---	MmugDNA.10030.1.S1_at_---
MmugDNA.10031.1.S1_at	209691_s_at	DOK4	docking protein 4	chr16q13	55715	DOK4	docking protein 4	---	704443	MmugDNA.10031.1.S1_at_DOK4
MmugDNA.1003.1.S1_at	222799_at	WDR91	WD repeat domain 91	chr7q33	29062	LOC707674	similar to HSPC049 protein	---	707674	MmugDNA.1003.1.S1_at_WDR91
MmugDNA.10032.1.S1_at	231757_at	TAS2R5	taste receptor, type 2, member 5	chr7q31.3-q32	54429	TAS2R5	taste receptor, type 2, member 5	---	694858	MmugDNA.10032.1.S1_at_TAS2R5
MmugDNA.10033.1.S1_at	212662_at	PVR	poliovirus receptor	chr19q13.2	5817	LOC714190	similar to poliovirus receptor	---	714190	MmugDNA.10033.1.S1_at_PVR
MmugDNA.10034.1.S1_at	207220_at	ART4	ADP-ribosyltransferase 4 (Dombrock blood group)	chr12p13-p12	420	ART4	ADP-ribosyltransferase 4	---	699934	MmugDNA.10034.1.S1_at_ART4
MmugDNA.10035.1.S1_at	NA	NA	NA	NA	NA	NA	NA	NA	NA	MmugDNA.10035.1.S1_at_NA
MmugDNA.10035.1.S1_s_at	234306_s_at	SLAMF7	SLAM family member 7	chr1q23.1-q24.1	57823	LOC719781	similar to SLAM family member 7	---	719781	MmugDNA.10035.1.S1_s_at_SLAMF7
MmugDNA.10036.1.S1_at	218560_s_at	JMJD4	jumonji domain containing 4	chr1q42.13	65094	JMJD4	jumonji domain containing 4	---	696580	MmugDNA.10036.1.S1_at_JMJD4
MmugDNA.10037.1.S1_at	222325_at	---	---	---	---	---	---	---	---	MmugDNA.10037.1.S1_at_---
MmugDNA.10038.1.S1_at	232997_at	---	---	---	---	---	---	---	---	MmugDNA.10038.1.S1_at_---
MmugDNA.10040.1.S1_at	201896_s_at	PSRC1	proline/serine-rich coiled-coil 1	chr1p13.3	84722	LOC711521	Similar to chromosome 22 open reading frame 13	---	711521	MmugDNA.10040.1.S1_at_PSRC1
MmugDNA.10041.1.S1_at	1569676_at	IFRG15 /// TOR1AIP2	interferon responsive gene 15 /// torsin A interacting protein 2	chr1q25.2	163590 /// 64163	---	---	---	---	MmugDNA.10041.1.S1_at_IFRG15 /// T
MmugDNA.1004.1.S1_at	236895_at	---	---	---	---	---	---	---	---	MmugDNA.1004.1.S1_at_---
MmugDNA.10042.1.S1_at	243516_at	---	---	---	---	---	---	---	---	MmugDNA.10042.1.S1_at_---
MmugDNA.10043.1.S1_at	215154_at	ULK2	unc-51-like kinase 2 (C. elegans)	chr17p11.2	9706	LOC708268	Similar to chondroitin sulfate proteoglycan 4	---	708268	MmugDNA.10043.1.S1_at_ULK2
MmugDNA.10044.1.S1_at	222647_at	SLC35C1	solute carrier family 35, member C1	chr11p11.2	55343	SLC35C1	solute carrier family 35, member C1	---	715064	MmugDNA.10044.1.S1_at_SLC35C1
MmugDNA.10045.1.S1_at	237459_at	---	---	---	---	---	---	---	---	MmugDNA.10045.1.S1_at_---
MmugDNA.10046.1.S1_s_at	233564_s_at	CDADC1	cytidine and dCMP deaminase domain containing 1	chr13q14.2	81602	LOC705941	similar to cytidine and dCMP deaminase domain containing 1	---	705941	MmugDNA.10046.1.S1_s_at_CDADC1
MmugDNA.10047.1.S1_at	224121_x_at	PLEKHB2	pleckstrin homology domain containing, family B (evectins) member 2	chr2q21.1	55041	---	---	---	---	MmugDNA.10047.1.S1_at_PLEKHB2
MmugDNA.10048.1.S1_at	240982_at	CARS	cysteinyl-tRNA synthetase	chr11p15.5	833	LOC696937	Similar to keratin 8	---	696937	MmugDNA.10048.1.S1_at_CARS
MmugDNA.10049.1.S1_at	241246_at	---	---	---	---	---	---	---	---	MmugDNA.10049.1.S1_at_---
MmugDNA.10050.1.S1_at	229107_at	---	---	---	---	---	---	---	---	MmugDNA.10050.1.S1_at_---
MmugDNA.10051.1.S1_at	244292_at	---	---	---	---	---	---	---	---	MmugDNA.10051.1.S1_at_---
MmugDNA.1005.1.S1_at	236680_at	---	---	---	---	---	---	---	---	MmugDNA.1005.1.S1_at_---
MmugDNA.10052.1.S1_at	226697_at	FAM114A1	family with sequence similarity 114, member A1	chr4p14	92689	LOC704283	similar to CG9590-PA	---	704283	MmugDNA.10052.1.S1_at_FAM114A1
MmugDNA.10053.1.S1_at	231095_at	---	---	---	---	---	---	---	---	MmugDNA.10053.1.S1_at_---
MmugDNA.10054.1.S1_at	218798_at	KRI1	KRI1 homolog (S. cerevisiae)	chr19p13.2	65095	LOC713047	similar to CG5645-PA	---	713047	MmugDNA.10054.1.S1_at_KRI1
MmugDNA.10055.1.S1_at	231171_at	---	---	---	---	---	---	---	---	MmugDNA.10055.1.S1_at_---
MmugDNA.10056.1.S1_at	233824_at	---	---	---	---	---	---	---	---	MmugDNA.10056.1.S1_at_---
MmugDNA.10057.1.S1_at	208661_s_at	TTC3	tetratricopeptide repeat domain 3	chr21q22.2	7267	TTC3	tetratricopeptide repeat domain 3	---	704690	MmugDNA.10057.1.S1_at_TTC3
MmugDNA.10058.1.S1_at	208662_s_at	TTC3	tetratricopeptide repeat domain 3	chr21q22.2	7267	TTC3	tetratricopeptide repeat domain 3	---	694206	MmugDNA.10058.1.S1_at_TTC3
MmugDNA.10059.1.S1_at	218757_s_at	UPF3B	UPF3 regulator of nonsense transcripts homolog B (yeast)	chrXq25-q26	65109	UPF3B	UPF3 regulator of nonsense transcripts homolog B	---	694247	MmugDNA.10059.1.S1_at_UPF3B
MmugDNA.10060.1.S1_at	208663_s_at	TTC3	tetratricopeptide repeat domain 3	chr21q22.2	7267	TTC3	tetratricopeptide repeat domain 3	---	694206	MmugDNA.10060.1.S1_at_TTC3
MmugDNA.10060.1.S1_s_at	NA	NA	NA	NA	NA	NA	NA	NA	NA	MmugDNA.10060.1.S1_s_at_NA
MmugDNA.10061.1.S1_s_at	239859_x_at	---	---	---	---	LOC710290	Similar to inhibitor of DNA binding 3	---	710290	MmugDNA.10061.1.S1_s_at_---
MmugDNA.1006.1.S1_at	240428_at	LOC285000	hypothetical protein LOC285000	chr2q12.2	285000	---	---	---	---	MmugDNA.1006.1.S1_at_LOC285000
MmugDNA.10062.1.S1_at	208664_s_at	TTC3	tetratricopeptide repeat domain 3	chr21q22.2	7267	TTC3	tetratricopeptide repeat domain 3	---	694206	MmugDNA.10062.1.S1_at_TTC3
MmugDNA.10063.1.S1_at	206959_s_at	UPF3A	UPF3 regulator of nonsense transcripts homolog A (yeast)	chr13q34	65110	LOC716601	similar to UPF3 regulator of nonsense transcripts homolog A isoform hUpf3p	---	716601	MmugDNA.10063.1.S1_at_UPF3A
MmugDNA.10064.1.S1_at	NA	NA	NA	NA	NA	NA	NA	NA	NA	MmugDNA.10064.1.S1_at_NA
MmugDNA.10065.1.S1_at	240092_at	---	---	---	---	---	---	---	---	MmugDNA.10065.1.S1_at_---
MmugDNA.10066.1.S1_at	202809_s_at	INTS3	integrator complex subunit 3	chr1q21.3	65123	LOC715645 /// LOC7184	similar to CG17665-PA.3 /// similar to solute carrier family 27 member 3	---	715645 /// 718424	MmugDNA.10066.1.S1_at_INTS3
MmugDNA.10067.1.S1_at	217699_at	---	---	---	---	---	---	---	---	MmugDNA.10067.1.S1_at_---
MmugDNA.10068.1.S1_at	1559635_at	CASC4	cancer susceptibility candidate 4	chr15q15.3	113201	---	---	---	---	MmugDNA.10068.1.S1_at_CASC4
MmugDNA.10069.1.S1_at	209482_at	POP7	processing of precursor 7, ribonuclease P/MRP subunit (S. cerevisiae)	chr7q22	10248	POP7	processing of precursor 7, ribonuclease P/MRP subunit (S. cerevisiae)	---	712859	MmugDNA.10069.1.S1_at_POP7
MmugDNA.10070.1.S1_at	200898_s_at	MGEA5	meningioma expressed antigen 5 (hyaluronidase)	chr10q24.1-q24.3	10724	LOC712370	similar to meningioma expressed antigen 5 (hyaluronidase)	---	712370	MmugDNA.10070.1.S1_at_MGEA5
MmugDNA.10071.1.S1_at	235632_at	---	---	---	---	---	---	---	---	MmugDNA.10071.1.S1_at_---
MmugDNA.1007.1.S1_at	1562158_at	---	---	---	---	---	---	---	---	MmugDNA.1007.1.S1_at_---
MmugDNA.10072.1.S1_at	231164_at	ABCA17P	ATP-binding cassette, sub-family A (ABC1), member 17 (pseudogene)	chr16p13.3	650655	---	---	---	---	MmugDNA.10072.1.S1_at_ABCA17P
MmugDNA.10073.1.S1_s_at	217865_at	RNF130	ring finger protein 130	chr5q35.3	55819	LOC715356	similar to ring finger protein 130	---	715356	MmugDNA.10073.1.S1_s_at_RNF130
MmugDNA.10074.1.S1_at	210894_s_at	CEP250	centrosomal protein 250kDa	chr20q11.22-q12	11190	CEP250	centrosomal protein 250kDa	---	705173	MmugDNA.10074.1.S1_at_CEP250
MmugDNA.10075.1.S1_at	221637_s_at	C11orf48	chromosome 11 open reading frame 48	chr11q12.3	79081	LOC705526	hypothetical protein LOC705526	---	705526	MmugDNA.10075.1.S1_at_C11orf48
MmugDNA.10076.1.S1_at	NA	NA	NA	NA	NA	NA	NA	NA	NA	MmugDNA.10076.1.S1_at_NA
MmugDNA.10077.1.S1_at	232185_at	C20orf132	chromosome 20 open reading frame 132	chr20q11.22	140699	---	---	---	---	MmugDNA.10077.1.S1_at_C20orf132
MmugDNA.10078.1.S1_at	220365_at	ALLC	allantoicase	chr2q35	55821	LOC710183	Similar to T-cell receptor alpha chain V region PY14 precursor	---	710183	MmugDNA.10078.1.S1_at_ALLC
MmugDNA.10079.1.S1_at	231374_at	---	---	---	---	---	---	---	---	MmugDNA.10079.1.S1_at_---
MmugDNA.10080.1.S1_at	1553706_at	HTRA4	HtrA serine peptidase 4	chr8p11.23	203100	HTRA4	HtrA serine peptidase 4	---	700875	MmugDNA.10080.1.S1_at_HTRA4
MmugDNA.10081.1.S1_at	223188_at	C19orf61	chromosome 19 open reading frame 61	chr19q13.31	56006	LOC710940	hypothetical protein LOC710940	---	710940	MmugDNA.10081.1.S1_at_C19orf61
MmugDNA.1008.1.S1_at	1555726_at	GAFA3	FGF-2 activity-associated protein 3	chr4p14	100128236	---	---	---	---	MmugDNA.1008.1.S1_at_GAFA3
MmugDNA.10082.1.S1_s_at	219974_x_at	ECHDC1	enoyl Coenzyme A hydratase domain containing 1	chr6q22.33	55862	LOC712072	similar to enoyl Coenzyme A hydratase domain containing 1	---	712072	MmugDNA.10082.1.S1_s_at_ECHDC1
MmugDNA.10083.1.S1_at	242892_at	---	---	---	---	---	---	---	---	MmugDNA.10083.1.S1_at_---
MmugDNA.10084.1.S1_at	228627_at	TLK2	tousled-like kinase 2	chr17q23	11011	---	---	---	---	MmugDNA.10084.1.S1_at_TLK2
MmugDNA.10085.1.S1_at	NA	NA	NA	NA	NA	NA	NA	NA	NA	MmugDNA.10085.1.S1_at_NA
MmugDNA.10086.1.S1_at	223811_s_at	C7orf20	chromosome 7 open reading frame 20	chr7p22.3	51608	LOC696126	similar to CG9853-PA	---	696126	MmugDNA.10086.1.S1_at_C7orf20
MmugDNA.10087.1.S1_at	242619_x_at	---	---	---	---	---	---	---	---	MmugDNA.10087.1.S1_at_---
MmugDNA.10088.1.S1_at	229219_s_at	---	---	---	---	---	---	---	---	MmugDNA.10088.1.S1_at_---
MmugDNA.10089.1.S1_at	1555875_at	SRGAP1	SLIT-ROBO Rho GTPase activating protein 1	chr12q14.2	57522	LOC717169	similar to SLIT-ROBO Rho GTPase-activating protein 1	---	717169	MmugDNA.10089.1.S1_at_SRGAP1
MmugDNA.10090.1.S1_at	1555876_at	SRGAP1	SLIT-ROBO Rho GTPase activating protein 1	chr12q14.2	57522	LOC717169	similar to SLIT-ROBO Rho GTPase-activating protein 1	---	717169	MmugDNA.10090.1.S1_at_SRGAP1
MmugDNA.10091.1.S1_at	1569609_at	---	---	---	---	---	---	---	---	MmugDNA.10091.1.S1_at_---
MmugDNA.1009.1.S1_at	209038_s_at	EHD1	EH-domain containing 1	chr11q13	10938	LOC702128	Similar to degenerative spermatocyte homolog 1, lipid desaturase	---	702128	MmugDNA.1009.1.S1_at_EHD1
MmugDNA.10092.1.S1_at	237064_x_at	---	---	---	---	---	---	---	---	MmugDNA.10092.1.S1_at_---
MmugDNA.10093.1.S1_at	216575_at	---	---	---	---	---	---	---	---	MmugDNA.10093.1.S1_at_---
MmugDNA.10094.1.S1_at	223845_at	FLJ20674	hypothetical protein FLJ20674	chr12q24.23	54621	LOC694552	hypothetical protein LOC694552	---	694552	MmugDNA.10094.1.S1_at_FLJ20674
MmugDNA.10095.1.S1_at	237318_at	---	---	---	---	---	---	---	---	MmugDNA.10095.1.S1_at_---
MmugDNA.10096.1.S1_at	223714_at	ZNF256	zinc finger protein 256	chr19q13.43	10172	LOC709486	similar to zinc finger protein 256	---	709486	MmugDNA.10096.1.S1_at_ZNF256
MmugDNA.10097.1.S1_at	237590_at	---	---	---	---	---	---	---	---	MmugDNA.10097.1.S1_at_---
MmugDNA.10098.1.S1_at	243288_at	SMYD2	SET and MYND domain containing 2	chr1q41	56950	---	---	---	---	MmugDNA.10098.1.S1_at_SMYD2
MmugDNA.10099.1.S1_at	244122_at	MGST3	Microsomal glutathione S-transferase 3	chr1q23	4259	---	---	---	---	MmugDNA.10099.1.S1_at_MGST3
MmugDNA.10100.1.S1_at	1565829_at	KIAA1731	KIAA1731	chr11q21	85459	LOC722637	hypothetical protein LOC722637	---	722637	MmugDNA.10100.1.S1_at_KIAA1731
MmugDNA.10101.1.S1_at	221409_at	OR2S2	olfactory receptor, family 2, subfamily S, member 2	chr9p13.3-p13.1	56656	---	---	---	---	MmugDNA.10101.1.S1_at_OR2S2
MmugDNA.1010.1.S1_at	241878_at	---	---	---	---	---	---	---	---	MmugDNA.1010.1.S1_at_---
MmugDNA.10102.1.S1_s_at	208687_x_at	HSPA8	heat shock 70kDa protein 8	chr11q24.1	3312	LOC701711	Similar to plasma glutamate carboxypeptidase	---	701711	MmugDNA.10102.1.S1_s_at_HSPA8
MmugDNA.10103.1.S1_at	225553_at	---	---	---	---	---	---	---	---	MmugDNA.10103.1.S1_at_---
MmugDNA.10104.1.S1_at	1562880_at	---	---	---	---	LOC710439	hypothetical protein LOC710439	---	710439	MmugDNA.10104.1.S1_at_---
MmugDNA.10105.1.S1_at	1569585_at	---	---	---	---	---	---	---	---	MmugDNA.10105.1.S1_at_---
MmugDNA.10106.1.S1_at	244568_at	---	---	---	---	---	---	---	---	MmugDNA.10106.1.S1_at_---
MmugDNA.10107.1.S1_at	216413_at	---	---	---	---	---	---	---	---	MmugDNA.10107.1.S1_at_---
MmugDNA.10108.1.S1_at	211003_x_at	TGM2	transglutaminase 2 (C polypeptide, protein-glutamine-gamma-glutamyltransferase)	chr20q12	7052	TGM2	transglutaminase 2	---	706777	MmugDNA.10108.1.S1_at_TGM2
MmugDNA.10109.1.S1_at	203937_s_at	TAF1C	TATA box binding protein (TBP)-associated factor, RNA polymerase I, C, 110kDa	chr16q24	9013	LOC695318	Similar to N-acyl-phosphatidylethanolamine-hydrolyzing phospholipase D	---	695318	MmugDNA.10109.1.S1_at_TAF1C
MmugDNA.10110.1.S1_at	213566_at	RNASE6	ribonuclease, RNase A family, k6	chr14q11.2	6039	RNASE6	ribonuclease, RNase A family, k6	---	704579	MmugDNA.10110.1.S1_at_RNASE6
MmugDNA.10111.1.S1_at	226242_at	C1orf131	chromosome 1 open reading frame 131	chr1q42.2	128061	LOC719141	hypothetical protein LOC719141	---	719141	MmugDNA.10111.1.S1_at_C1orf131
MmugDNA.1011.1.S1_at	231078_at	---	---	---	---	---	---	---	---	MmugDNA.1011.1.S1_at_---
MmugDNA.10112.1.S1_at	NA	NA	NA	NA	NA	NA	NA	NA	NA	MmugDNA.10112.1.S1_at_NA

MmugDNA.10112.1.S1_s_at	1554021_a_at	ZNF12	zinc finger protein 12	chr7p22.1	7559	ZNF12	zinc finger protein 12	---	705346	MmugDNA.10112.1.S1_s_at_ZNF12
MmugDNA.10113.1.S1_at	217653_x_at	---	---	---	---	---	---	---	---	MmugDNA.10113.1.S1_at_---
MmugDNA.10114.1.S1_at	213273_at	ODZ4	odz, odd Oz/ten-m homolog 4 (Drosophila)	chr11q14.1	26011	LOC701138	similar to odd Oz/ten-m homolog 4	---	701138	MmugDNA.10114.1.S1_at_ODZ4
MmugDNA.10115.1.S1_at	236126_at	ACVR2B	activin A receptor, type IIB	chr3p22	93	---	---	---	---	MmugDNA.10115.1.S1_at_ACVR2B
MmugDNA.10116.1.S1_at	209478_at	STRA13	stimulated by retinoic acid 13 homolog (mouse)	chr17q25.3	201254	LOC719290	similar to stimulated by retinoic acid 13	---	719290	MmugDNA.10116.1.S1_at_STRA13
MmugDNA.10117.1.S1_s_at	210567_s_at	SKP2	S-phase kinase-associated protein 2 (p45)	chr5p13	6502	SKP2	S-phase kinase-associated protein 2 (p45)	---	700617	MmugDNA.10117.1.S1_s_at_SKP2
MmugDNA.10118.1.S1_at	240684_at	---	---	---	---	---	---	---	---	MmugDNA.10118.1.S1_at_---
MmugDNA.10119.1.S1_at	1553828_at	FAM55A	family with sequence similarity 55, member A	chr11q23.2	120400	LOC695630	hypothetical protein LOC695630	---	695630	MmugDNA.10119.1.S1_at_FAM55A
MmugDNA.101.1.S1_at	222882_s_at	C17orf59	chromosome 17 open reading frame 59	chr17p13.1	54785	LOC716995	hypothetical protein LOC716995	---	716995	MmugDNA.101.1.S1_at_C17orf59
MmugDNA.10120.1.S1_at	230408_at	---	---	---	---	---	---	---	---	MmugDNA.10120.1.S1_at_---
MmugDNA.10121.1.S1_s_at	201138_s_at	SSB	Sjogren syndrome antigen B (autoantigen La)	chr2q31.1	6741	LOC710344	similar to autoantigen La	---	710344	MmugDNA.10121.1.S1_s_at_SSB
MmugDNA.1012.1.S1_at	208901_s_at	TOP1	topoisomerase (DNA) I	chr20q12-q13.1	7150	---	---	---	---	MmugDNA.1012.1.S1_at_TOP1
MmugDNA.10122.1.S1_at	208424_s_at	CIAPIN1	cytokine induced apoptosis inhibitor 1	chr16q13-q21	57019	CIAPIN1	similar to Anamorsin (Cytokine-induced apoptosis inhibitor 1) (CUA001)	---	704770	MmugDNA.10122.1.S1_at_CIAPIN1
MmugDNA.10123.1.S1_at	240231_at	---	---	---	---	---	---	---	---	MmugDNA.10123.1.S1_at_---
MmugDNA.10124.1.S1_at	207449_s_at	POFUT2	protein O-fucosyltransferase 2	chr21q22.3	23275	---	---	---	---	MmugDNA.10124.1.S1_at_POFUT2
MmugDNA.10125.1.S1_at	1564851_at	---	---	---	---	---	---	---	---	MmugDNA.10125.1.S1_at_---
MmugDNA.10126.1.S1_s_at	219954_s_at	GBA3	glucosidase, beta, acid 3 (cytosolic)	chr4p15.31	57733	LOC715716	similar to cytosolic beta-glucosidase	---	715716	MmugDNA.10126.1.S1_s_at_GBA3
MmugDNA.10127.1.S1_at	217996_at	PHLDA1	pleckstrin homology-like domain, family A, member 1	chr12q15	22822	LOC719036	similar to T-cell death associated gene	---	719036	MmugDNA.10127.1.S1_at_PHLDA1
MmugDNA.10128.1.S1_at	217997_at	PHLDA1	pleckstrin homology-like domain, family A, member 1	chr12q15	22822	LOC719036	similar to T-cell death associated gene	---	719036	MmugDNA.10128.1.S1_at_PHLDA1
MmugDNA.10129.1.S1_at	221278_at	HOXB8	homeobox B8	chr17q21.3	3218	LOC697211	similar to homeobox B9	---	697211	MmugDNA.10129.1.S1_at_HOXB8
MmugDNA.10130.1.S1_at	217999_s_at	PHLDA1	pleckstrin homology-like domain, family A, member 1	chr12q15	22822	LOC719036	similar to T-cell death associated gene	---	719036	MmugDNA.10130.1.S1_at_PHLDA1
MmugDNA.10131.1.S1_at	1565848_at	ZNF428	zinc finger protein 428	chr19q13.31	126299	---	---	---	---	MmugDNA.10131.1.S1_at_ZNF428
MmugDNA.1013.1.S1_at	241692_at	---	---	---	---	---	---	---	---	MmugDNA.1013.1.S1_at_---
MmugDNA.10132.1.S1_at	1557771_at	FHAD1	forkhead-associated (FHA) phosphopeptide binding domain 1	chr1p36.21	114827	---	---	---	---	MmugDNA.10132.1.S1_at_FHAD1
MmugDNA.10133.1.S1_at	240934_at	---	---	---	---	---	---	---	---	MmugDNA.10133.1.S1_at_---
MmugDNA.10134.1.S1_at	201671_x_at	USP14	ubiquitin specific peptidase 14 (tRNA-guanine transglycosylase)	chr18p11.32	9097	---	---	---	---	MmugDNA.10134.1.S1_at_USP14
MmugDNA.10135.1.S1_at	234710_s_at	PARP6	poly (ADP-ribose) polymerase family, member 6	chr15q23	56965	LOC697993	similar to poly (ADP-ribose) polymerase family, member 16	---	697993	MmugDNA.10135.1.S1_at_PARP6
MmugDNA.10136.1.S1_at	229794_at	ZNF585A	zinc finger protein 585A	chr19q13.12	199704	---	---	---	---	MmugDNA.10136.1.S1_at_ZNF585A
MmugDNA.10137.1.S1_at	215274_at	SLC12A3	solute carrier family 12 (sodium/chloride transporters), member 3	chr16q13	6559	---	---	---	---	MmugDNA.10137.1.S1_at_SLC12A3
MmugDNA.10138.1.S1_at	209520_s_at	NCBP1	nuclear cap binding protein subunit 1, 80kDa	chr9q34.1	4686	NCBP1	nuclear cap binding protein subunit 1, 80kDa	---	718256	MmugDNA.10138.1.S1_at_NCBP1
MmugDNA.10139.1.S1_at	216607_s_at	CYP51A1	cytochrome P450, family 51, subfamily A, polypeptide 1	chr7q21.2-q21.3|7q21.2	1595	LOC703490	cytochrome P450, family 51	---	703490	MmugDNA.10139.1.S1_at_CYP51A1
MmugDNA.10140.1.S1_at	202719_s_at	TES	testis derived transcript (3 LIM domains)	chr7q31.2	26136	TES	testis derived transcript (3 LIM domains)	---	706879	MmugDNA.10140.1.S1_at_TES
MmugDNA.10141.1.S1_at	241041_at	---	---	---	---	---	---	---	---	MmugDNA.10141.1.S1_at_---
MmugDNA.1014.1.S1_at	236319_at	---	---	---	---	---	---	---	---	MmugDNA.1014.1.S1_at_---
MmugDNA.10142.1.S1_s_at	221311_x_at	LYRM2	LYR motif containing 2	chr6q14.2-q16.1	57226	LOC701575	hypothetical protein LOC701575	---	701575	MmugDNA.10142.1.S1_s_at_LYRM2
MmugDNA.10143.1.S1_at	239928_at	TCTN2	tectonic family member 2	chr12q24.31	79867	LOC721415	similar to TECT2	---	721415	MmugDNA.10143.1.S1_at_TCTN2
MmugDNA.10144.1.S1_at	1553772_at	GK5	glycerol kinase 5 (putative)	chr3q23	256356	LOC714604	similar to CG1271-PA, isoform A	---	714604	MmugDNA.10144.1.S1_at_GK5
MmugDNA.10145.1.S1_at	1561236_at	---	---	---	---	---	---	---	---	MmugDNA.10145.1.S1_at_---
MmugDNA.10146.1.S1_at	224806_at	TRIM25	tripartite motif-containing 25	chr17q23.2	7706	LOC712588	similar to tripartite motif-containing 25	---	712588	MmugDNA.10146.1.S1_at_TRIM25
MmugDNA.10147.1.S1_at	NA	NA	NA	NA	NA	NA	NA	NA	NA	MmugDNA.10147.1.S1_at_NA
MmugDNA.10148.1.S1_at	215058_at	DENND5B	DENN/MADD domain containing 5B	chr12p11.21	160518	---	---	---	---	MmugDNA.10148.1.S1_at_DENND5B
MmugDNA.10149.1.S1_at	240916_x_at	---	---	---	---	---	---	---	---	MmugDNA.10149.1.S1_at_---
MmugDNA.10150.1.S1_s_at	221259_s_at	TEX11	testis expressed 11	chrXq13.1	56159	LOC697414	similar to testis expressed sequence 11 isoform 1	---	697414	MmugDNA.10150.1.S1_s_at_TEX11
MmugDNA.10151.1.S1_at	232412_at	FBXL20	F-box and leucine-rich repeat protein 20	chr17q12	84961	---	---	---	---	MmugDNA.10151.1.S1_at_FBXL20
MmugDNA.1015.1.S1_at	243886_at	---	---	---	---	---	---	---	---	MmugDNA.1015.1.S1_at_---
MmugDNA.10152.1.S1_at	236766_at	---	---	---	---	---	---	---	---	MmugDNA.10152.1.S1_at_---
MmugDNA.10153.1.S1_at	1560734_at	LOC727924 /// OR4N4	hypothetical LOC727924 /// olfactory receptor, family 4, subfamily N, member 4	chr15q11.2	283694 /// 727924	LOC699628	similar to Olfactory receptor 4N4	---	699628	MmugDNA.10153.1.S1_at_LOC727924
MmugDNA.10154.1.S1_at	241076_at	---	---	---	---	---	---	---	---	MmugDNA.10154.1.S1_at_---
MmugDNA.10155.1.S1_at	1569339_s_at	---	---	---	---	---	---	---	---	MmugDNA.10155.1.S1_at_---
MmugDNA.10156.1.S1_at	209531_at	GSTZ1	glutathione transferase zeta 1	chr14q24.3	2954	LOC705723	similar to glutathione transferase zeta 1 isoform 1	---	705723	MmugDNA.10156.1.S1_at_GSTZ1
MmugDNA.10157.1.S1_at	230859_at	---	---	---	---	---	---	---	---	MmugDNA.10157.1.S1_at_---
MmugDNA.10158.1.S1_at	223109_at	TRUB2	TruB pseudouridine (psi) synthase homolog 2 (E. coli)	chr9q34.11	26995	LOC717619	similar to TruB pseudouridine (psi) synthase homolog 2	---	717619	MmugDNA.10158.1.S1_at_TRUB2
MmugDNA.10159.1.S1_at	222841_s_at	TIMM22	translocase of inner mitochondrial membrane 22 homolog (yeast)	chr17p13	29928	LOC721162	similar to Mitochondrial import inner membrane translocase subunit Tim22	---	721162	MmugDNA.10159.1.S1_at_TIMM22
MmugDNA.10160.1.S1_at	213355_at	ST3GAL6	ST3 beta-galactoside alpha-2,3-sialyltransferase 6	chr3q12.1	10402	ST3GAL6	ST3 beta-galactoside alpha-2,3-sialyltransferase 6	---	699096	MmugDNA.10160.1.S1_at_ST3GAL6
MmugDNA.10161.1.S1_s_at	204186_s_at	PPID	peptidylprolyl isomerase D	chr4q31.3	5481	LOC705500	similar to peptidylprolyl isomerase D	---	705500	MmugDNA.10161.1.S1_s_at_PPID
MmugDNA.10161.1.S1_x_at	NA	NA	NA	NA	NA	NA	NA	NA	NA	MmugDNA.10161.1.S1_x_at_NA
MmugDNA.1016.1.S1_at	238549_at	CBFA2T2	core-binding factor, runt domain, alpha subunit 2; translocated to, 2	chr20q11	9139	---	---	---	---	MmugDNA.1016.1.S1_at_CBFA2T2
MmugDNA.10162.1.S1_at	213335_s_at	ST3GAL6	ST3 beta-galactoside alpha-2,3-sialyltransferase 6	chr3q12.1	10402	---	---	---	---	MmugDNA.10162.1.S1_at_ST3GAL6
MmugDNA.10163.1.S1_at	NA	NA	NA	NA	NA	NA	NA	NA	NA	MmugDNA.10163.1.S1_at_NA
MmugDNA.10164.1.S1_at	1557197_a_at	LGALS3	lectin, galactoside-binding, soluble, 3	chr14q21-q22	3958	---	---	---	---	MmugDNA.10164.1.S1_at_LGALS3
MmugDNA.10165.1.S1_at	228948_at	EPHA4	EPH receptor A4	chr2q36.1	2043	HN1	Hematological and neurological expressed 1	---	664729	MmugDNA.10165.1.S1_at_EPHA4
MmugDNA.10166.1.S1_at	242611_at	---	---	---	---	---	---	---	---	MmugDNA.10166.1.S1_at_---
MmugDNA.10167.1.S1_at	238906_s_at	RHOJ	ras homolog gene family, member J	chr14q23.2	57381	RHOJ	ras homolog gene family, member J	---	705603	MmugDNA.10167.1.S1_at_RHOJ
MmugDNA.10168.1.S1_at	234456_at	TRAPPC4	trafficking protein particle complex 4	chr11q23.3	51399	---	---	---	---	MmugDNA.10168.1.S1_at_TRAPPC4
MmugDNA.10169.1.S1_at	1559341_at	---	---	---	---	---	---	---	---	MmugDNA.10169.1.S1_at_---
MmugDNA.10170.1.S1_at	242219_at	---	---	---	---	---	---	---	---	MmugDNA.10170.1.S1_at_---
MmugDNA.10171.1.S1_at	228045_at	---	---	---	---	---	---	---	---	MmugDNA.10171.1.S1_at_---
MmugDNA.1017.1.S1_at	229715_at	DKFZp686O24166	Hypothetical protein DKFZp686O24166	chr11p15.1	374383	---	---	---	---	MmugDNA.1017.1.S1_at_DKFZp686O2
MmugDNA.10172.1.S1_at	227051_at	---	---	---	---	---	---	---	---	MmugDNA.10172.1.S1_at_---
MmugDNA.10173.1.S1_at	235631_at	DDR2	Discoidin domain receptor tyrosine kinase 2	chr1q23.3	4921	LOC720124	similar to discoidin domain receptor family, member 2 precursor	---	720124	MmugDNA.10173.1.S1_at_DDR2
MmugDNA.10174.1.S1_at	230483_at	---	---	---	---	---	---	---	---	MmugDNA.10174.1.S1_at_---
MmugDNA.10175.1.S1_at	1569484_s_at	MDN1	MDN1, midasin homolog (yeast)	chr6q15	23195	---	---	---	---	MmugDNA.10175.1.S1_at_MDN1
MmugDNA.10176.1.S1_at	234101_at	---	---	---	---	---	---	---	---	MmugDNA.10176.1.S1_at_---
MmugDNA.10177.1.S1_at	221571_at	TRAF3	TNF receptor-associated factor 3	chr14q32.32	7187	TRAF3	TNF receptor-associated factor 3	---	693961	MmugDNA.10177.1.S1_at_TRAF3
MmugDNA.10178.1.S1_at	235166_at	ZNF148	zinc finger protein 148	chr3q21	7707	ZNF148	zinc finger protein 148	---	715925	MmugDNA.10178.1.S1_at_ZNF148
MmugDNA.10179.1.S1_at	232509_at	PDE4DIP	phosphodiesterase 4D interacting protein	chr1q12	9659	---	---	---	---	MmugDNA.10179.1.S1_at_PDE4DIP
MmugDNA.10180.1.S1_at	1563099_at	---	---	---	---	---	---	---	---	MmugDNA.10180.1.S1_at_---
MmugDNA.10181.1.S1_at	1554145_a_at	KLRAQ1	KLRAQ motif containing 1	chr2p16.3	129285	LOC715917	similar to smooth muscle myosin heavy chain 11 isoform SM1-like	---	715917	MmugDNA.10181.1.S1_at_KLRAQ1
MmugDNA.1018.1.S1_at	235813_at	---	---	---	---	---	---	---	---	MmugDNA.1018.1.S1_at_---
MmugDNA.10182.1.S1_at	233660_at	EHD4	EH-domain containing 4	chr15q11.1	30844	EHD4	EH-domain containing 4	---	707376	MmugDNA.10182.1.S1_at_EHD4
MmugDNA.10183.1.S1_at	1561009_at	MAOB	Monoamine oxidase B	chrXp11.23	4129	---	---	---	---	MmugDNA.10183.1.S1_at_MAOB
MmugDNA.10184.1.S1_at	1562691_at	---	---	---	---	---	---	---	---	MmugDNA.10184.1.S1_at_---
MmugDNA.10185.1.S1_at	1552526_at	FAM71C	family with sequence similarity 71, member C	chr12q23.1	196472	LOC694296	hypothetical protein LOC694296	---	694296	MmugDNA.10185.1.S1_at_FAM71C
MmugDNA.10186.1.S1_at	228323_at	CASC5	cancer susceptibility candidate 5	chr15q14	57082	---	---	---	---	MmugDNA.10186.1.S1_at_CASC5
MmugDNA.10187.1.S1_at	223523_at	TMEM108	transmembrane protein 108	chr3q21	66000	LOC717579	similar to mucin and cadherin like	---	717579	MmugDNA.10187.1.S1_at_TMEM108
MmugDNA.10188.1.S1_at	244309_at	---	---	---	---	---	---	---	---	MmugDNA.10188.1.S1_at_---
MmugDNA.10189.1.S1_at	230711_at	---	---	---	---	---	---	---	---	MmugDNA.10189.1.S1_at_---
MmugDNA.10190.1.S1_at	244537_at	---	---	---	---	---	---	---	---	MmugDNA.10190.1.S1_at_---
MmugDNA.10191.1.S1_at	238850_at	LOC645323	hypothetical LOC645323	chr5q14.3	645323	---	---	---	---	MmugDNA.10191.1.S1_at_LOC645323
MmugDNA.1019.1.S1_at	1564674_a_at	CDC20B	cell division cycle 20 homolog B (S. cerevisiae)	chr5q11.2	166979	CDC20B	cell division cycle 20 homolog B (S. cerevisiae)	---	705833	MmugDNA.1019.1.S1_at_CDC20B
MmugDNA.10192.1.S1_at	238934_at	---	---	---	---	---	---	---	---	MmugDNA.10192.1.S1_at_---
MmugDNA.10193.1.S1_at	NA	NA	NA	NA	NA	NA	NA	NA	NA	MmugDNA.10193.1.S1_at_NA
MmugDNA.10194.1.S1_at	226310_at	RICTOR	rapamycin-insensitive companion of mTOR	chr5p13.1	253260	RICTOR	rapamycin-insensitive companion of mTOR	---	694915	MmugDNA.10194.1.S1_at_RICTOR
MmugDNA.10195.1.S1_at	213427_at	RPP40	ribonuclease P/MRP 40kDa subunit	chr6p25.1	10799	LOC710505	similar to ribonuclease P 40kDa subunit	---	710505	MmugDNA.10195.1.S1_at_RPP40
MmugDNA.10196.1.S1_at	226312_at	RICTOR	rapamycin-insensitive companion of mTOR	chr5p13.1	253260	RICTOR	rapamycin-insensitive companion of mTOR	---	694915	MmugDNA.10196.1.S1_at_RICTOR
MmugDNA.10197.1.S1_at	211178_s_at	PSTPIP1	proline-serine-threonine phosphatase interacting protein 1	chr15q24-q25.1	9051	PSTPIP2	similar to proline-serine-threonine phosphatase interacting protein 1	---	708327	MmugDNA.10197.1.S1_at_PSTPIP1
MmugDNA.10198.1.S1_at	202824_s_at	TCEB1	transcription elongation factor B (SIII), polypeptide 1 (15kDa, elongin C)	chr8q21.11	6921	---	---	---	---	MmugDNA.10198.1.S1_at_TCEB1
MmugDNA.10199.1.S1_at	242285_at	---	---	---	---	---	---	---	---	MmugDNA.10199.1.S1_at_---
MmugDNA.10.1.S1_s_at	NA	NA	NA	NA	NA	NA	NA	NA	NA	MmugDNA.10.1.S1_s_at_NA
MmugDNA.10200.1.S1_at	239093_at	NPL2	N-acetylneuraminate pyruvate lyase 2 (putative)	chr10q24.1	112817	---	---	---	---	MmugDNA.10200.1.S1_at_NPL2
MmugDNA.10201.1.S1_at	240937_at	LOC389662	similar to hCG1984745	chr8q12.1	389662	---	---	---	---	MmugDNA.10201.1.S1_at_LOC389662
MmugDNA.1020.1.S1_at	220076_at	ANKH	ankylosis, progressive homolog (mouse)	chr5p15.1	56172	---	---	---	---	MmugDNA.1020.1.S1_at_ANKH
MmugDNA.10202.1.S1_at	216847_at	---	---	---	---	---	---	---	---	MmugDNA.10202.1.S1_at_---
MmugDNA.10203.1.S1_at	243434_at	---	---	---	---	---	---	---	---	MmugDNA.10203.1.S1_at_---
MmugDNA.10204.1.S1_at	234144_at	---	---	---	---	---	---	---	---	MmugDNA.10204.1.S1_at_---
MmugDNA.10205.1.S1_at	243970_at	LOC100130642	hypothetical LOC100130642	chr2p21	100130642	---	---	---	---	MmugDNA.10205.1.S1_at_LOC1001306
MmugDNA.10206.1.S1_at	239890_at	SERP2	Stress-associated endoplasmic reticulum protein family member 2	chr13q14.11	387923	---	---	---	---	MmugDNA.10206.1.S1_at_SERP2
MmugDNA.10207.1.S1_at	239445_at	---	---	---	---	---	---	---	---	MmugDNA.10207.1.S1_at_---

MmugDNA.10208.1.S1_s_at	222056_s_at	FAHD2A	fumarylacetoacetate hydrolase domain containing 2A	chr2p24.3-p11.2	51011	LOC704637 /// LOC7069	similar to fumarylacetoacetate hydrolase domain containing 2A /// similar to fumarylacetoacetate hydr	---	704637 /// 706967	MmugDNA.10208.1.S1_s_at_FAHD2A
MmugDNA.10209.1.S1_at	204525_at	PHF14	PHD finger protein 14	chr7p21.3	9678	PHF14	PHD finger protein 14	---	694855	MmugDNA.10209.1.S1_at_PHF14
MmugDNA.10210.1.S1_at	207637_at	LOC100129448 /// WSC	hypothetical protein LOC100129448 /// WSC domain containing 2	chr12q23.3	100129448 /// 9671	---	---	---	---	MmugDNA.10210.1.S1_at_LOC1001294
MmugDNA.10211.1.S1_at	1558530_at	LRTM2	leucine-rich repeats and transmembrane domains 2	chr12p13.33	654429	LOC707976	similar to slit CG8355-PA, isoform A	---	707976	MmugDNA.10211.1.S1_at_LRTM2
MmugDNA.1021.1.S1_at	234636_at	---	---	---	---	---	---	---	---	MmugDNA.1021.1.S1_at_---
MmugDNA.10212.1.S1_at	1552999_a_at	WFDC10B	WAP four-disulfide core domain 10B	chr20q13.12	280664	---	---	---	---	MmugDNA.10212.1.S1_at_WFDC10B
MmugDNA.10213.1.S1_at	211522_s_at	GNRHR	gonadotropin-releasing hormone receptor	chr4q21.2	2798	---	---	---	---	MmugDNA.10213.1.S1_at_GNRHR
MmugDNA.10214.1.S1_at	202700_s_at	TMEM63A	transmembrane protein 63A	chr1q42.12	9725	---	---	---	---	MmugDNA.10214.1.S1_at_TMEM63A
MmugDNA.10215.1.S1_at	211159_s_at	PPP2R5D	protein phosphatase 2, regulatory subunit B', delta isoform	chr6p21.1	5528	LOC695982	Hypothetical protein LOC695982	---	695982	MmugDNA.10215.1.S1_at_PPP2R5D
MmugDNA.10216.1.S1_at	1557621_at	KCP	kielin/chordin-like protein	chr7q32.1	375616	---	---	---	---	MmugDNA.10216.1.S1_at_KCP
MmugDNA.10217.1.S1_at	NA	NA	NA	NA	NA	NA	NA	NA	NA	MmugDNA.10217.1.S1_at_NA
MmugDNA.10218.1.S1_at	204511_at	FARP2	FERM, RhoGEF and pleckstrin domain protein 2	chr2q37.3	9855	FARP2	FERM, RhoGEF and pleckstrin domain protein 2	---	701179	MmugDNA.10218.1.S1_at_FARP2
MmugDNA.10219.1.S1_at	244384_at	---	---	---	---	---	---	---	---	MmugDNA.10219.1.S1_at_---
MmugDNA.102.1.S1_at	217903_at	STRN4	striatin, calmodulin binding protein 4	chr19q13.2	29888	LOC696958	Similar to ribosomal protein L10	---	696958	MmugDNA.102.1.S1_at_STRN4
MmugDNA.10220.1.S1_at	204257_at	FADS3	fatty acid desaturase 3	chr11q12-q13.1	3995	FADS3	fatty acid desaturase 3	---	722337	MmugDNA.10220.1.S1_at_FADS3
MmugDNA.10221.1.S1_at	224223_s_at	PDE11A	phosphodiesterase 11A	chr2q31.2	50940	PDE11A	phosphodiesterase 11A	---	702458	MmugDNA.10221.1.S1_at_PDE11A
MmugDNA.1022.1.S1_s_at	238900_at	HLA-DRB1 /// HLA-DRE	major histocompatibility complex, class II, DR beta 1 /// major histocompatibility complex, class II, DR	chr6p21.3	3123 /// 3125 /// 3126	---	---	---	---	MmugDNA.1022.1.S1_s_at_HLA-DRB1
MmugDNA.10222.1.S1_at	200862_at	DHCR24	24-dehydrocholesterol reductase	chr1p33-p31.1	1718	DCLRE1C	Artemis protein	---	697493	MmugDNA.10222.1.S1_at_DHCR24
MmugDNA.10223.1.S1_at	209527_at	EXOSC2	exosome component 2	chr9q34	23404	EXOSC2	exosome component 2	---	715960	MmugDNA.10223.1.S1_at_EXOSC2
MmugDNA.10224.1.S1_at	207190_at	ZZEF1	zinc finger, ZZ-type with EF-hand domain 1	chr17p13.2	23140	LOC722400	Similar to NADH-ubiquinone oxidoreductase flavoprotein 3 isoform a precursor	---	722400	MmugDNA.10224.1.S1_at_ZZEF1
MmugDNA.10225.1.S1_at	229795_at	---	---	---	---	---	---	---	---	MmugDNA.10225.1.S1_at_---
MmugDNA.10226.1.S1_at	226995_at	LOC642852	hypothetical LOC642852	chr21q22.3	642852	---	---	---	---	MmugDNA.10226.1.S1_at_LOC642852
MmugDNA.10227.1.S1_at	236175_at	TRIM55	tripartite motif-containing 55	chr8q13.1	84675	LOC711707	similar to ring finger protein 29 isoform 2	---	711707	MmugDNA.10227.1.S1_at_TRIM55
MmugDNA.10228.1.S1_at	1570516_s_at	OR51B5	olfactory receptor, family 51, subfamily B, member 5	chr11p15.4	282763	LOC696727	similar to olfactory receptor, family 51, subfamily B, member 5	---	696727	MmugDNA.10228.1.S1_at_OR51B5
MmugDNA.10229.1.S1_at	238375_at	RPL22	Ribosomal protein L22	chr1p36.3-p36.2	6146	---	---	---	---	MmugDNA.10229.1.S1_at_RPL22
MmugDNA.10230.1.S1_at	227836_at	UTP23	UTP23, small subunit (SSU) processome component, homolog (yeast)	chr8q24.11	84294	---	---	---	---	MmugDNA.10230.1.S1_at_UTP23
MmugDNA.10231.1.S1_at	231564_at	MANEAL	Mannosidase, endo-alpha-like	chr1p34.3	149175	LOC716710	similar to mannosidase, endo-alpha	---	716710	MmugDNA.10231.1.S1_at_MANEAL
MmugDNA.1023.1.S1_at	236909_at	C2orf77	chromosome 2 open reading frame 77	chr2q31.1	129881	LOC709886	similar to SH2 domain containing 4B	---	709886	MmugDNA.1023.1.S1_at_C2orf77
MmugDNA.10232.1.S1_at	219848_s_at	ZNF432	zinc finger protein 432	chr19q13.33	9668	---	---	---	---	MmugDNA.10232.1.S1_at_ZNF432
MmugDNA.10233.1.S1_s_at	223083_s_at	EGLN2	egl nine homolog 2 (C. elegans)	chr19q13.2	112398	EGLN2	egl nine homolog 2 (C. elegans)	---	703083	MmugDNA.10233.1.S1_s_at_EGLN2
MmugDNA.10234.1.S1_at	204377_s_at	VPRBP	Vpr (HIV-1) binding protein	chr3p21.2	9730	LOC700596	similar to VprBP protein	---	700596	MmugDNA.10234.1.S1_at_VPRBP
MmugDNA.10235.1.S1_at	202462_s_at	DDX46	DEAD (Asp-Glu-Ala-Asp) box polypeptide 46	chr5q31.1	9879	LOC711026	similar to DEAD (Asp-Glu-Ala-Asp) box polypeptide 46	---	711026	MmugDNA.10235.1.S1_at_DDX46
MmugDNA.10236.1.S1_at	208657_s_at	SEPT9	septin 9	chr17q25	10801	LOC720811	similar to Septin-9 (MLL septin-like fusion protein) (MLL septin-like fusion protein MSF-A) (Ovarian/Br	---	720811	MmugDNA.10236.1.S1_at_SEPT9
MmugDNA.10237.1.S1_at	208202_s_at	PHF15	PHD finger protein 15	chr5q31.1	23338	LOC713338	similar to PHD finger protein 15	---	713338	MmugDNA.10237.1.S1_at_PHF15
MmugDNA.10238.1.S1_at	243198_at	TEX9	testis expressed 9	chr15q21.3	374618	TEX9	testis expressed 9	---	699256	MmugDNA.10238.1.S1_at_TEX9
MmugDNA.10239.1.S1_at	237640_at	C14orf138	chromosome 14 open reading frame 138	chr14q22.1	79609	LOC713211	Similar to eukaryotic translation initiation factor 3, subunit 10 theta, 150/170kDa	---	713211	MmugDNA.10239.1.S1_at_C14orf138
MmugDNA.10240.1.S1_at	208303_s_at	CRLF2	cytokine receptor-like factor 2	chrXp22.3; Yp11.3	64109	---	---	---	---	MmugDNA.10240.1.S1_at_CRLF2
MmugDNA.10241.1.S1_at	NA	NA	NA	NA	NA	NA	NA	NA	NA	MmugDNA.10241.1.S1_at_NA
MmugDNA.1024.1.S1_s_at	224494_x_at	HSD17B14	hydroxysteroid (17-beta) dehydrogenase 14	chr19q13.33	51171	LOC718891	similar to pleckstrin homology domain containing, family A (phosphoinositide binding specific) membe	---	718891	MmugDNA.1024.1.S1_s_at_HSD17B14
MmugDNA.10242.1.S1_s_at	213753_x_at	EIF5A	eukaryotic translation initiation factor 5A	chr17p13-p12	1984	---	---	---	---	MmugDNA.10242.1.S1_s_at_EIF5A
MmugDNA.10243.1.S1_at	202167_s_at	MMS19	MMS19 nucleotide excision repair homolog (S. cerevisiae)	chr10q24-q25	64210	MMS19	MMS19 nucleotide excision repair homolog (S. cerevisiae)	---	706888	MmugDNA.10243.1.S1_at_MMS19
MmugDNA.10244.1.S1_at	1560334_at	MEGF11	multiple EGF-like-domains 11	chr15q22.31	84465	---	---	---	---	MmugDNA.10244.1.S1_at_MEGF11
MmugDNA.10245.1.S1_at	239476_at	---	---	---	---	---	---	---	---	MmugDNA.10245.1.S1_at_---
MmugDNA.10246.1.S1_at	214663_at	DSTYK	dual serine/threonine and tyrosine protein kinase	chr1q32.1	25778	DSTYK	dual serine/threonine and tyrosine protein kinase	---	664732	MmugDNA.10246.1.S1_at_DSTYK
MmugDNA.10247.1.S1_at	1561155_at	---	---	---	---	---	---	---	---	MmugDNA.10247.1.S1_at_---
MmugDNA.10248.1.S1_at	1563455_at	KIAA0999	KIAA0999 protein	chr11q23.3	23387	---	---	---	---	MmugDNA.10248.1.S1_at_KIAA0999
MmugDNA.10249.1.S1_at	219497_s_at	BCL11A	B-cell CLL/lymphoma 11A (zinc finger protein)	chr2p16.1	53335	---	---	---	---	MmugDNA.10249.1.S1_at_BCL11A
MmugDNA.10250.1.S1_at	217091_at	---	---	---	---	---	---	---	---	MmugDNA.10250.1.S1_at_---
MmugDNA.10251.1.S1_at	209473_at	ENTPD1	ectonucleoside triphosphate diphosphohydrolase 1	chr10q24	953	ENTPD1	ectonucleoside triphosphate diphosphohydrolase 1	---	703591	MmugDNA.10251.1.S1_at_ENTPD1
MmugDNA.1025.1.S1_at	1556551_s_at	SLC39A6	solute carrier family 39 (zinc transporter), member 6	chr18q12.2	25800	LOC709313	similar to solute carrier family 39 (zinc transporter), member 6	---	709313	MmugDNA.1025.1.S1_at_SLC39A6
MmugDNA.10252.1.S1_at	241861_at	SYCP3	Synaptonemal complex protein 3	chr12q	50511	---	---	---	---	MmugDNA.10252.1.S1_at_SYCP3
MmugDNA.10253.1.S1_at	221941_at	PAOX	polyamine oxidase (exo-N4-amino)	chr10q26.3	196743	LOC719884	similar to polyamine oxidase (exo-N4-amino) isoform 4	---	719884	MmugDNA.10253.1.S1_at_PAOX
MmugDNA.10254.1.S1_at	241309_at	---	---	---	---	---	---	---	---	MmugDNA.10254.1.S1_at_---
MmugDNA.10255.1.S1_at	236374_at	CTXN3	cortexin 3	chr5q23.2	613212	LOC704352	similar to Cortexin-1	---	704352	MmugDNA.10255.1.S1_at_CTXN3
MmugDNA.10256.1.S1_at	1556153_s_at	---	---	---	---	---	---	---	---	MmugDNA.10256.1.S1_at_---
MmugDNA.10257.1.S1_at	204530_s_at	TOX	thymocyte selection-associated high mobility group box	chr8q12.1	9760	TOX	thymocyte selection-associated high mobility group box	---	698759	MmugDNA.10257.1.S1_at_TOX
MmugDNA.10258.1.S1_at	NA	NA	NA	NA	NA	NA	NA	NA	NA	MmugDNA.10258.1.S1_at_NA
MmugDNA.10259.1.S1_at	204568_at	KIAA0831	KIAA0831	chr14q22.3	22863	---	---	---	---	MmugDNA.10259.1.S1_at_KIAA0831
MmugDNA.10260.1.S1_at	203159_at	GLS	glutaminase	chr2q32-q34	2744	---	---	---	---	MmugDNA.10260.1.S1_at_GLS
MmugDNA.10261.1.S1_at	223252_at	HDGF2	hepatoma-derived growth factor-related protein 2	chr19p13.3	84717	LOC693476	similar to hepatoma-derived growth factor-related protein 2 isoform 1	---	693476	MmugDNA.10261.1.S1_at_HDGF2
MmugDNA.1026.1.S1_at	1556288_at	C18orf62	chromosome 18 open reading frame 62	chr18q22.3	284274	---	---	---	---	MmugDNA.1026.1.S1_at_C18orf62
MmugDNA.10262.1.S1_at	NA	NA	NA	NA	NA	NA	NA	NA	NA	MmugDNA.10262.1.S1_at_NA
MmugDNA.10262.1.S1_s_at	220042_x_at	HIVEP3	human immunodeficiency virus type I enhancer binding protein 3	chr1p34	59269	HIVEP3	human immunodeficiency virus type I enhancer binding protein 3	---	696084	MmugDNA.10262.1.S1_s_at_HIVEP3
MmugDNA.10263.1.S1_at	1569811_at	LOC729307	hypothetical protein LOC729307	chr4q28.3	729307	---	---	---	---	MmugDNA.10263.1.S1_at_LOC729307
MmugDNA.10264.1.S1_at	223743_s_at	MRPL4	mitochondrial ribosomal protein L4	chr19p13.2	51073	LOC715100	similar to mitochondrial ribosomal protein L4 isoform a	---	715100	MmugDNA.10264.1.S1_at_MRPL4
MmugDNA.10265.1.S1_at	232228_at	ZNF530	zinc finger protein 530	chr19q13.43	348327	---	---	---	---	MmugDNA.10265.1.S1_at_ZNF530
MmugDNA.10266.1.S1_at	241051_at	---	---	---	---	---	---	---	---	MmugDNA.10266.1.S1_at_---
MmugDNA.10267.1.S1_at	220641_at	NOX5	NADPH oxidase, EF-hand calcium binding domain 5	chr15q23	79400	NOX5	NADPH oxidase, EF-hand calcium binding domain 5	---	694875	MmugDNA.10267.1.S1_at_NOX5
MmugDNA.10269.1.S1_at	209479_at	CCDC28A	coiled-coil domain containing 28A	chr6q23.1-q24.1	25901	LOC703104	hypothetical protein LOC703104	---	703104	MmugDNA.10269.1.S1_at_CCDC28A
MmugDNA.10270.1.S1_at	211448_s_at	RGS6	regulator of G-protein signaling 6	chr14q24.3	9628	LOC698400	Hypothetical protein LOC698400	---	698400	MmugDNA.10270.1.S1_at_RGS6
MmugDNA.10271.1.S1_at	223678_s_at	SFTPA1 /// SFTPA1B //	surfactant protein A1 /// surfactant protein A1B /// surfactant protein A2 /// surfactant protein A2B	chr10q22.3	6435 /// 6436 /// 65350	SFTPA2	surfactant protein A2	---	701715	MmugDNA.10271.1.S1_at_SFTPA1 /// S
MmugDNA.1027.1.S1_at	214509_at	HIST1H3I	histone cluster 1, H3i	chr6p22-p21.3	8354	LOC705220	similar to histone 1, H2ai (predicted)	---	705220	MmugDNA.1027.1.S1_at_HIST1H3I
MmugDNA.10272.1.S1_at	226707_at	NAPRT1	nicotinate phosphoribosyltransferase domain containing 1	chr8q24.3	93100	LOC708992	similar to nicotinate phosphoribosyltransferase domain containing 1	---	708992	MmugDNA.10272.1.S1_at_NAPRT1
MmugDNA.10273.1.S1_at	222393_s_at	NAT13	N-acetyltransferase 13 (GCN5-related)	chr3q13.2	80218	LOC700650	Similar to Ect3 CG3132-PA	---	700650	MmugDNA.10273.1.S1_at_NAT13
MmugDNA.10274.1.S1_at	233178_at	TGIF2LY	TGFB-induced factor homeobox 2-like, Y-linked	chrYp11.2	90655	TGIFLX	TGIF-like protein on the X	---	574239	MmugDNA.10274.1.S1_at_TGIF2LY
MmugDNA.10275.1.S1_x_at	NA	NA	NA	NA	NA	NA	NA	NA	NA	MmugDNA.10275.1.S1_x_at_NA
MmugDNA.10276.1.S1_at	206448_at	ZNF365	zinc finger protein 365	chr10q21.2	22891	SH3GLB1	SH3-domain GRB2-like endophilin B1	---	712393	MmugDNA.10276.1.S1_at_ZNF365
MmugDNA.10277.1.S1_at	222397_at	EFTUD2	elongation factor Tu GTP binding domain containing 2	chr17q21.31	9343	EFTUD2	elongation factor Tu GTP binding domain containing 2	---	715512	MmugDNA.10277.1.S1_at_EFTUD2
MmugDNA.10278.1.S1_at	206732_at	SLITRK3	SLIT and NTRK-like family, member 3	chr3q26.1	22865	SLITRK3	similar to slit and trk like 3 protein	---	700660	MmugDNA.10278.1.S1_at_SLITRK3
MmugDNA.10279.1.S1_at	1570237_at	---	---	---	---	---	---	---	---	MmugDNA.10279.1.S1_at_---
MmugDNA.10280.1.S1_at	1564511_a_at	FSTL4	follistatin-like 4	chr5q31.1	23105	LOC709868	similar to follistatin-like 4	---	709868	MmugDNA.10280.1.S1_at_FSTL4
MmugDNA.10281.1.S1_s_at	1562876_s_at	LOC541471	Hypothetical LOC541471	chr2q13	541471	---	---	---	---	MmugDNA.10281.1.S1_s_at_LOC54147
MmugDNA.1028.1.S1_at	NA	NA	NA	NA	NA	NA	NA	NA	NA	MmugDNA.1028.1.S1_at_NA
MmugDNA.10282.1.S1_at	204626_s_at	ITGB3	integrin, beta 3 (platelet glycoprotein IIIa, antigen CD61)	chr17q21.32	3690	LOC717379	similar to integrin beta chain, beta 3 precursor	---	717379	MmugDNA.10282.1.S1_at_ITGB3
MmugDNA.10283.1.S1_at	219692_at	KREMEN2	kringle containing transmembrane protein 2	chr16p13.3	79412	KREMEN2	similar to kringle-containing transmembrane protein 2 isoform b precursor	---	701257	MmugDNA.10283.1.S1_at_KREMEN2
MmugDNA.10284.1.S1_at	203956_at	MORC2	MORC family CW-type zinc finger 2	chr22q12.2	22880	MORC2	MORC family CW-type zinc finger 2	---	718224	MmugDNA.10284.1.S1_at_MORC2
MmugDNA.10285.1.S1_at	219836_at	ZBED2	zinc finger, BED-type containing 2	chr3q13.13	79413	ZBED2	zinc finger, BED-type containing 2	---	706929	MmugDNA.10285.1.S1_at_ZBED2
MmugDNA.10286.1.S1_at	223066_at	SNAPIN	SNAP-associated protein	chr1q21.3	23557	LOC715546	similar to SNARE-associated protein Snapin (Synaptosomal-associated protein 25-binding protein) (S	---	715546	MmugDNA.10286.1.S1_at_SNAPIN
MmugDNA.10287.1.S1_at	219346_at	LRFN3	leucine rich repeat and fibronectin type III domain containing 3	chr19q13.12	79414	LOC711565	similar to leucine rich repeat and fibronectin type III domain containing 3	---	711565	MmugDNA.10287.1.S1_at_LRFN3
MmugDNA.10288.1.S1_at	232018_at	LENG1	leukocyte receptor cluster (LRC) member 1	chr19q13.4	79165	LENG1	leukocyte receptor cluster (LRC) member 1	---	693417	MmugDNA.10288.1.S1_at_LENG1
MmugDNA.10289.1.S1_at	218130_at	C17orf62	chromosome 17 open reading frame 62	chr17q25.3	79415	LOC716212	hypothetical protein LOC716212	---	716212	MmugDNA.10289.1.S1_at_C17orf62
MmugDNA.10290.1.S1_at	213644_at	CCDC46	coiled-coil domain containing 46	chr17q24.1-q24.2	201134	LOC718585	similar to CG3493-PA	---	718585	MmugDNA.10290.1.S1_at_CCDC46
MmugDNA.10291.1.S1_at	207786_at	CYP2R1	cytochrome P450, family 2, subfamily R, polypeptide 1	chr11p15.2	120227	---	---	---	---	MmugDNA.10291.1.S1_at_CYP2R1
MmugDNA.1029.1.S1_at	233449_at	---	---	---	---	---	---	---	---	MmugDNA.1029.1.S1_at_---
MmugDNA.10292.1.S1_at	240170_at	---	---	---	---	---	---	---	---	MmugDNA.10292.1.S1_at_---
MmugDNA.10293.1.S1_at	1554784_at	CNTN1	contactin 1	chr12q11-q12	1272	---	---	---	---	MmugDNA.10293.1.S1_at_CNTN1
MmugDNA.10294.1.S1_at	239499_at	DNAH2	dynein, axonemal, heavy chain 2	chr17p13.1	146754	---	---	---	---	MmugDNA.10294.1.S1_at_DNAH2
MmugDNA.10295.1.S1_at	1561389_at	---	---	---	---	---	---	---	---	MmugDNA.10295.1.S1_at_---
MmugDNA.10296.1.S1_at	240547_at	---	---	---	---	---	---	---	---	MmugDNA.10296.1.S1_at_---
MmugDNA.10297.1.S1_at	213288_at	MBOAT2	membrane bound O-acyltransferase domain containing 2	chr2p25.1	129642	---	---	---	---	MmugDNA.10297.1.S1_at_MBOAT2
MmugDNA.10298.1.S1_at	237301_at	---	---	---	---	---	---	---	---	MmugDNA.10298.1.S1_at_---
MmugDNA.10299.1.S1_at	217321_x_at	ATXN3	Ataxin 3	chr14q24.3-q32.2	4287	---	---	---	---	MmugDNA.10299.1.S1_at_ATXN3
MmugDNA.10300.1.S1_at	240099_at	---	---	---	---	---	---	---	---	MmugDNA.10300.1.S1_at_---
MmugDNA.10301.1.S1_at	231969_at	STOX2	storkhead box 2	chr4q35.1	56977	STOX2	storkhead box 2	---	693692	MmugDNA.10301.1.S1_at_STOX2
MmugDNA.1030.1.S1_at	225303_at	KIRREL	kin of IRRE like (Drosophila)	chr1q21-q25	55243	KIRREL	kin of IRRE like	---	718870	MmugDNA.1030.1.S1_at_KIRREL
MmugDNA.10302.1.S1_at	203556_at	ZHX2	zinc fingers and homeoboxes 2	chr8q24.13	22882	---	---	---	---	MmugDNA.10302.1.S1_at_ZHX2
MmugDNA.10303.1.S1_at	238356_at	DOCK11	dedicator of cytokinesis 11	chrXq24	139818	LOC710690	similar to dedicator of cytokinesis 11	---	710690	MmugDNA.10303.1.S1_at_DOCK11
MmugDNA.10304.1.S1_at	234361_at	CREB3L3	cAMP responsive element binding protein 3-like 3	chr19p13.3	84699	LOC721825	similar to cAMP responsive element binding protein 3-like 3	---	721825	MmugDNA.10304.1.S1_at_CREB3L3

MmugDNA.10304.1.S1_s_at	NA	NA	NA	NA	NA	NA	NA	NA	NA	MmugDNA.10304.1.S1_s_at_NA
MmugDNA.10305.1.S1_at	223145_s_at	AKIRIN2	akirin 2	chr6q15	55122	LOC700914	similar to CG8580-PA, isoform A	---	700914	MmugDNA.10305.1.S1_at_AKIRIN2
MmugDNA.10306.1.S1_at	202519_at	MLXIP	MLX interacting protein	chr12q24.31	22877	MLXIP	MLX interacting protein	---	705874	MmugDNA.10306.1.S1_at_MLXIP
MmugDNA.10307.1.S1_at	219609_at	WDR25	WD repeat domain 25	chr14q32.2	79446	LOC717690	similar to pre-mRNA splicing factor-like	---	717690	MmugDNA.10307.1.S1_at_WDR25
MmugDNA.10308.1.S1_at	217163_at	ESR1	estrogen receptor 1	chr6q25.1	2099	---	---	---	---	MmugDNA.10308.1.S1_at_ESR1
MmugDNA.10309.1.S1_at	232394_at	---	---	---	---	LOC720779	Similar to Plakophilin-3	---	720779	MmugDNA.10309.1.S1_at_---
MmugDNA.10310.1.S1_at	215640_at	TBC1D2B	TBC1 domain family, member 2B	chr15q24.3-q25.1	23102	---	---	---	---	MmugDNA.10310.1.S1_at_TBC1D2B
MmugDNA.10311.1.S1_at	219302_s_at	CNTNAP2	contactin associated protein-like 2	chr7q35-q36	26047	---	---	---	---	MmugDNA.10311.1.S1_at_CNTNAP2
MmugDNA.1031.1.S1_at	1562440_at	MAP3K13	Mitogen-activated protein kinase kinase kinase 13	chr3q27	9175	---	---	---	---	MmugDNA.1031.1.S1_at_MAP3K13
MmugDNA.10312.1.S1_at	218300_at	C16orf53	chromosome 16 open reading frame 53	chr16p11.2	79447	LOC706839	hypothetical protein LOC706839	---	706839	MmugDNA.10312.1.S1_at_C16orf53
MmugDNA.10313.1.S1_at	236850_at	CAPRIN1	Cell cycle associated protein 1	chr11p13	4076	---	---	---	---	MmugDNA.10313.1.S1_at_CAPRIN1
MmugDNA.10314.1.S1_at	239000_at	BRD4	Bromodomain containing 4	chr19p13.1	23476	---	---	---	---	MmugDNA.10314.1.S1_at_BRD4
MmugDNA.10315.1.S1_at	1565765_x_at	C13orf33	Chromosome 13 open reading frame 33	chr13q12.3	84935	---	---	---	---	MmugDNA.10315.1.S1_at_C13orf33
MmugDNA.10316.1.S1_s_at	224057_s_at	THAP4	THAP domain containing 4	chr2q37.3	51078	THAP4	THAP domain containing 4	---	701543	MmugDNA.10316.1.S1_s_at_THAP4
MmugDNA.10317.1.S1_at	218029_at	FAM65A	family with sequence similarity 65, member A	chr16q22.1	79567	LOC699910	similar to C27H2.3	---	699910	MmugDNA.10317.1.S1_at_FAM65A
MmugDNA.10318.1.S1_at	219176_at	C2orf47	chromosome 2 open reading frame 47	chr2q33.1	79568	C12H2orf47	chromosome 2 open reading frame 47 ortholog	---	699322	MmugDNA.10318.1.S1_at_C2orf47
MmugDNA.10319.1.S1_at	1566142_at	---	---	---	---	---	---	---	---	MmugDNA.10319.1.S1_at_---
MmugDNA.103.1.S1_at	214121_x_at	PDLIM7	PDZ and LIM domain 7 (enigma)	chr5q35.3	9260	PDLIM7	PDZ and LIM domain 7 (enigma)	---	706581	MmugDNA.103.1.S1_at_PDLIM7
MmugDNA.10320.1.S1_at	228090_at	NMNAT3	nicotinamide nucleotide adenylyltransferase 3	chr3q23	349565	NMNAT3	nicotinamide nucleotide adenylyltransferase 3	---	715614	MmugDNA.10320.1.S1_at_NMNAT3
MmugDNA.10321.1.S1_at	225519_at	PPP4R2	protein phosphatase 4, regulatory subunit 2	chr3p13	151987	LOC697207	similar to protein phosphatase 4, regulatory subunit 2	---	697207	MmugDNA.10321.1.S1_at_PPP4R2
MmugDNA.1032.1.S1_at	213821_s_at	---	---	---	---	---	---	---	---	MmugDNA.1032.1.S1_at_---
MmugDNA.10322.1.S1_at	209614_at	ADH1B	alcohol dehydrogenase 1B (class I), beta polypeptide	chr4q21-q23	125	---	---	---	---	MmugDNA.10322.1.S1_at_ADH1B
MmugDNA.10323.1.S1_s_at	212028_at	RBM25	RNA binding motif protein 25	chr14q24.3	58517	RBM25	RNA binding motif protein 25	---	695911	MmugDNA.10323.1.S1_s_at_RBM25
MmugDNA.10325.1.S1_at	212031_at	RBM25	RNA binding motif protein 25	chr14q24.3	58517	KPNA1	Karyopherin alpha 1	---	692082	MmugDNA.10325.1.S1_at_RBM25
MmugDNA.10326.1.S1_at	212030_at	RBM25	RNA binding motif protein 25	chr14q24.3	58517	KPNA1	Karyopherin alpha 1	---	692082	MmugDNA.10326.1.S1_at_RBM25
MmugDNA.10327.1.S1_at	203724_s_at	RUFY3	RUN and FYVE domain containing 3	chr4q13.3	22902	---	---	---	---	MmugDNA.10327.1.S1_at_RUFY3
MmugDNA.10328.1.S1_at	212027_at	RBM25	RNA binding motif protein 25	chr14q24.3	58517	---	---	---	---	MmugDNA.10328.1.S1_at_RBM25
MmugDNA.10329.1.S1_at	240385_at	---	---	---	---	---	---	---	---	MmugDNA.10329.1.S1_at_---
MmugDNA.10330.1.S1_at	1561609_at	---	---	---	---	---	---	---	---	MmugDNA.10330.1.S1_at_---
MmugDNA.10331.1.S1_at	202976_s_at	RHOBTB3	Rho-related BTB domain containing 3	chr5q15	22836	LOC716022	Similar to Vasodilator-stimulated phosphoprotein (VASP)	---	716022	MmugDNA.10331.1.S1_at_RHOBTB3
MmugDNA.1033.1.S1_at	1556700_a_at	---	---	---	---	---	---	---	---	MmugDNA.1033.1.S1_at_---
MmugDNA.10332.1.S1_at	1554140_at	WDR78	WD repeat domain 78	chr1p31.3	79819	LOC699921	similar to WD repeat domain 78 isoform 2	---	699921	MmugDNA.10332.1.S1_at_WDR78
MmugDNA.10333.1.S1_at	221573_at	C7orf25	chromosome 7 open reading frame 25	chr7p14-p11.2	79020	LOC708455	hypothetical protein LOC708455	---	708455	MmugDNA.10333.1.S1_at_C7orf25
MmugDNA.10334.1.S1_at	204161_s_at	ENPP4	ectonucleotide pyrophosphatase/phosphodiesterase 4 (putative function)	chr6p12.3	22875	LOC699511	Similar to CTAGE family, member 5 isoform 1	---	699511	MmugDNA.10334.1.S1_at_ENPP4
MmugDNA.10335.1.S1_at	221206_at	PMS2 /// PMS2CL	PMS2 postmeiotic segregation increased 2 (S. cerevisiae) /// PMS2 C-terminal like pseudogene	chr7p22.1 /// chr7p22.2	441194 /// 5395	---	---	---	---	MmugDNA.10335.1.S1_at_PMS2 /// PM
MmugDNA.10336.1.S1_at	NA	NA	NA	NA	NA	NA	NA	NA	NA	MmugDNA.10336.1.S1_at_NA
MmugDNA.10337.1.S1_at	219438_at	NKAIN1	Na+/K+ transporting ATPase interacting 1	chr1p35.2	79570	LOC704595	similar to CG9047-PA, isoform A	---	704595	MmugDNA.10337.1.S1_at_NKAIN1
MmugDNA.10338.1.S1_at	218912_at	GCC1	GRIP and coiled-coil domain containing 1	chr7q32.1	79571	GCC1	GRIP and coiled-coil domain containing 1	---	697975	MmugDNA.10338.1.S1_at_GCC1
MmugDNA.10339.1.S1_at	210922_at	---	---	---	---	---	---	---	---	MmugDNA.10339.1.S1_at_---
MmugDNA.10340.1.S1_at	227864_s_at	FAM125A	family with sequence similarity 125, member A	chr19p13.11	93343	---	---	---	---	MmugDNA.10340.1.S1_at_FAM125A
MmugDNA.10341.1.S1_at	1556413_a_at	---	---	---	---	---	---	---	---	MmugDNA.10341.1.S1_at_---
MmugDNA.1034.1.S1_at	231848_x_at	ZNF207	zinc finger protein 207	chr17q11.2	7756	LOC697319	Similar to EMSY protein	---	697319	MmugDNA.1034.1.S1_at_ZNF207
MmugDNA.10342.1.S1_at	219558_at	ATP13A3	ATPase type 13A3	chr3q29	79572	ATP13A3	ATPase type 13A3	---	707893	MmugDNA.10342.1.S1_at_ATP13A3
MmugDNA.10343.1.S1_at	230731_x_at	ZDHHC8	zinc finger, DHHC-type containing 8	chr22q11.21	29801	LOC712832	similar to zinc finger, DHHC domain containing 8	---	712832	MmugDNA.10343.1.S1_at_ZDHHC8
MmugDNA.10344.1.S1_at	239479_x_at	---	---	---	---	---	---	---	---	MmugDNA.10344.1.S1_at_---
MmugDNA.10345.1.S1_at	219481_at	TTC13	tetratricopeptide repeat domain 13	chr1q42.2	79573	TTC13	tetratricopeptide repeat domain 13	---	714027	MmugDNA.10345.1.S1_at_TTC13
MmugDNA.10346.1.S1_at	238147_at	TRIM46	tripartite motif-containing 46	chr1q22	80128	LOC717498	similar to tripartite motif-containing 46	---	717498	MmugDNA.10346.1.S1_at_TRIM46
MmugDNA.10347.1.S1_at	211421_s_at	RET	ret proto-oncogene	chr10q11.2	5979	---	---	---	---	MmugDNA.10347.1.S1_at_RET
MmugDNA.10348.1.S1_s_at	208319_s_at	RBM3	RNA binding motif (RNP1, RRM) protein 3	chrXp11.2	5935	RBM3	RNA binding motif (RNP1, RRM) protein 3	---	710689	MmugDNA.10348.1.S1_s_at_RBM3
MmugDNA.10349.1.S1_at	223152_at	PPP1R12C	protein phosphatase 1, regulatory (inhibitor) subunit 12C	chr19q13.42	54776	PPP1R12C	protein phosphatase 1, regulatory (inhibitor) subunit 12C	---	698538	MmugDNA.10349.1.S1_at_PPP1R12C
MmugDNA.10350.1.S1_at	203694_s_at	DHX16	DEAH (Asp-Glu-Ala-His) box polypeptide 16	chr6p21.3	8449	DHX16	DEAH (Asp-Glu-Ala-His) box polypeptide 16	---	713836	MmugDNA.10350.1.S1_at_DHX16
MmugDNA.10351.1.S1_at	1562976_at	---	---	---	---	---	---	---	---	MmugDNA.10351.1.S1_at_---
MmugDNA.1035.1.S1_at	NA	NA	NA	NA	NA	NA	NA	NA	NA	MmugDNA.1035.1.S1_at_NA
MmugDNA.10352.1.S1_at	208151_x_at	DDX17	DEAD (Asp-Glu-Ala-Asp) box polypeptide 17	chr22q13.1	10521	---	---	---	---	MmugDNA.10352.1.S1_at_DDX17
MmugDNA.10353.1.S1_at	237221_at	---	---	---	---	---	---	---	---	MmugDNA.10353.1.S1_at_---
MmugDNA.10354.1.S1_at	205763_s_at	DDX18	DEAD (Asp-Glu-Ala-Asp) box polypeptide 18	chr2q14.1	8886	DDX18	DEAD (Asp-Glu-Ala-Asp) box polypeptide 18	---	693278	MmugDNA.10354.1.S1_at_DDX18
MmugDNA.10355.1.S1_at	225439_at	NUDCD1	NudC domain containing 1	chr8q23	84955	LOC698632	similar to NudC domain containing 1	---	698632	MmugDNA.10355.1.S1_at_NUDCD1
MmugDNA.10356.1.S1_at	225438_at	NUDCD1	NudC domain containing 1	chr8q23	84955	LOC698632	similar to NudC domain containing 1	---	698632	MmugDNA.10356.1.S1_at_NUDCD1
MmugDNA.10357.1.S1_at	233110_s_at	BCL2L12	BCL2-like 12 (proline rich)	chr19q13.3	83596	LOC718981	similar to BCL2-like 12 isoform 1	---	718981	MmugDNA.10357.1.S1_at_BCL2L12
MmugDNA.10358.1.S1_at	NA	NA	NA	NA	NA	NA	NA	NA	NA	MmugDNA.10358.1.S1_at_NA
MmugDNA.10359.1.S1_at	204355_at	DHX30	DEAH (Asp-Glu-Ala-His) box polypeptide 30	chr3p21.31	22907	LOC711178	similar to DEAH (Asp-Glu-Ala-His) box polypeptide 30 isoform 1	---	711178	MmugDNA.10359.1.S1_at_DHX30
MmugDNA.10359.1.S1_s_at	NA	NA	NA	NA	NA	NA	NA	NA	NA	MmugDNA.10359.1.S1_s_at_NA
MmugDNA.10360.1.S1_at	214252_s_at	CLN5	ceroid-lipofuscinosis, neuronal 5	chr13q21.1-q32	1203	---	---	---	---	MmugDNA.10360.1.S1_at_CLN5
MmugDNA.10361.1.S1_at	205274_at	GTPBP1	GTP binding protein 1	chr22q13.1	9567	---	---	---	---	MmugDNA.10361.1.S1_at_GTPBP1
MmugDNA.1036.1.S1_at	244856_at	---	---	---	---	---	---	---	---	MmugDNA.1036.1.S1_at_---
MmugDNA.10362.1.S1_at	236089_at	---	---	---	---	---	---	---	---	MmugDNA.10362.1.S1_at_---
MmugDNA.10363.1.S1_at	203855_at	WDR47	WD repeat domain 47	chr1p13.3	22911	LOC711452	Similar to Kelch repeat and BTB domain-containing protein 4 (BTB and kelch domain-containing prot	---	711452	MmugDNA.10363.1.S1_at_WDR47
MmugDNA.10365.1.S1_at	234164_at	---	---	---	---	---	---	---	---	MmugDNA.10365.1.S1_at_---
MmugDNA.10366.1.S1_at	207436_x_at	KIAA0894	KIAA0894 protein	chr10q23.33	22833	---	---	---	---	MmugDNA.10366.1.S1_at_KIAA0894
MmugDNA.10367.1.S1_at	229086_at	C1orf213	chromosome 1 open reading frame 213	chr1p36.12	148898	---	---	---	---	MmugDNA.10367.1.S1_at_C1orf213
MmugDNA.10368.1.S1_at	206731_at	CNKSR2	connector enhancer of kinase suppressor of Ras 2	chrXp22.12	22866	CNKSR2	connector enhancer of kinase suppressor of Ras 2	---	696046	MmugDNA.10368.1.S1_at_CNKSR2
MmugDNA.10369.1.S1_at	219404_at	EPS8L3	EPS8-like 3	chr1p13.3	79574	EPS8L3	EPS8-like 3	---	700441	MmugDNA.10369.1.S1_at_EPS8L3
MmugDNA.10370.1.S1_at	223223_at	ARV1	ARV1 homolog (S. cerevisiae)	chr1q42.2	64801	LOC713975	similar to ARV1 homolog	---	713975	MmugDNA.10370.1.S1_at_ARV1
MmugDNA.10371.1.S1_at	222240_s_at	ISYNA1	inositol-3-phosphate synthase 1	chr19p13.11	51477	---	---	---	---	MmugDNA.10371.1.S1_at_ISYNA1
MmugDNA.1037.1.S1_at	239870_at	SPATS1 /// TMEM151B	spermatogenesis associated, serine-rich 1 /// transmembrane protein 151B	chr6p21.1	221409 /// 441151	SPATS1	spermatogenesis associated, serine-rich 1	---	703218	MmugDNA.1037.1.S1_at_SPATS1 /// TI
MmugDNA.10372.1.S1_at	227728_at	PPM1A	protein phosphatase 1A (formerly 2C), magnesium-dependent, alpha isoform	chr14q23.1	5494	LOC697300	Similar to DNA topoisomerase I	---	697300	MmugDNA.10372.1.S1_at_PPM1A
MmugDNA.10373.1.S1_at	200860_s_at	CNOT1	CCR4-NOT transcription complex, subunit 1	chr16q21	23019	LOC712880	similar to CCR4-NOT transcription complex, subunit 1 isoform a	---	712880	MmugDNA.10373.1.S1_at_CNOT1
MmugDNA.10374.1.S1_at	205396_at	SMAD3	SMAD family member 3	chr15q22.33	4088	SMAD3	SMAD family member 3	---	711355	MmugDNA.10374.1.S1_at_SMAD3
MmugDNA.10376.1.S1_at	218881_s_at	FOSL2	FOS-like antigen 2	chr2p23.3	2355	---	---	---	---	MmugDNA.10376.1.S1_at_FOSL2
MmugDNA.10377.1.S1_at	231113_at	---	---	---	---	---	---	---	---	MmugDNA.10377.1.S1_at_---
MmugDNA.10378.1.S1_at	1568660_a_at	---	---	---	---	---	---	---	---	MmugDNA.10378.1.S1_at_---
MmugDNA.10379.1.S1_at	200694_s_at	DDX24	DEAD (Asp-Glu-Ala-Asp) box polypeptide 24	chr14q32	57062	LOC699931	Similar to Crooked neck-like protein 1 (Crooked neck homolog) (hCrn)	---	699931	MmugDNA.10379.1.S1_at_DDX24
MmugDNA.10380.1.S1_at	219185_at	SIRT5	sirtuin (silent mating type information regulation 2 homolog) 5 (S. cerevisiae)	chr6p23	23408	SIRT5	sirtuin (silent mating type information regulation 2 homolog) 5 (S. cerevisiae)	---	700855	MmugDNA.10380.1.S1_at_SIRT5
MmugDNA.10381.1.S1_at	1559060_a_at	FNIP1	Folliculin interacting protein 1	chr5q23.3	96459	---	---	---	---	MmugDNA.10381.1.S1_at_FNIP1
MmugDNA.1038.1.S1_at	244616_x_at	MDM2	Mdm2 p53 binding protein homolog (mouse)	chr12q14.3-q15	4193	---	---	---	---	MmugDNA.1038.1.S1_at_MDM2
MmugDNA.10382.1.S1_at	1567281_at	---	---	---	---	---	---	---	---	MmugDNA.10382.1.S1_at_---
MmugDNA.10383.1.S1_at	210689_at	CLDN14	claudin 14	chr21q22.3	23562	CLDN2	similar to claudin 14	---	695087	MmugDNA.10383.1.S1_at_CLDN14
MmugDNA.10384.1.S1_at	1557502_at	PCCB	Propionyl Coenzyme A carboxylase, beta polypeptide	chr3q21-q22	5096	---	---	---	---	MmugDNA.10384.1.S1_at_PCCB
MmugDNA.10385.1.S1_at	1554486_a_at	C6orf114	chromosome 6 open reading frame 114	chr6p23	85411	---	---	---	---	MmugDNA.10385.1.S1_at_C6orf114
MmugDNA.10386.1.S1_at	203727_at	SKIV2L	superkiller viralicidic activity 2-like (S. cerevisiae)	chr6p21	6499	LOC715987	similar to superkiller viralicidic activity 2-like homolog	---	715987	MmugDNA.10386.1.S1_at_SKIV2L
MmugDNA.10387.1.S1_at	244712_at	MEX3D	mex-3 homolog D (C. elegans)	chr19p13.3	399664	MEX3D	mex-3 homolog D (C. elegans)	---	721304	MmugDNA.10387.1.S1_at_MEX3D
MmugDNA.10388.1.S1_at	1561303_at	---	---	---	---	---	---	---	---	MmugDNA.10388.1.S1_at_---
MmugDNA.10389.1.S1_at	202220_at	KIAA0907	KIAA0907	chr1q22	22889	LOC718005	hypothetical protein LOC718005	---	718005	MmugDNA.10389.1.S1_at_KIAA0907
MmugDNA.10390.1.S1_at	201561_s_at	CLSTN1	calsyntenin 1	chr1p36.22	22883	CLSTN1	Calsyntenin 1	---	722304	MmugDNA.10390.1.S1_at_CLSTN1
MmugDNA.10391.1.S1_at	1556797_at	LOC386597	hypothetical protein LOC386597	chr2p25.2	386597	---	---	---	---	MmugDNA.10391.1.S1_at_LOC386597
MmugDNA.1039.1.S1_at	242095_at	FLJ45825	hypothetical protein LOC646888	chr6p21.2	646888	---	---	---	---	MmugDNA.1039.1.S1_at_FLJ45825
MmugDNA.10392.1.S1_at	NA	NA	NA	NA	NA	NA	NA	NA	NA	MmugDNA.10392.1.S1_at_NA
MmugDNA.10392.1.S1_s_at	202973_x_at	FAM13A	family with sequence similarity 13, member A	chr4q22.1	10144	FAM13A	family with sequence similarity 13, member A	---	703335	MmugDNA.10392.1.S1_s_at_FAM13A
MmugDNA.10393.1.S1_at	1552457_a_at	ADAMTSL1	ADAMTS-like 1	chr9p22.2-p22.1	92949	ADAMTSL1	ADAMTS-like 1	---	712909	MmugDNA.10393.1.S1_at_ADAMTSL1
MmugDNA.10394.1.S1_at	229285_at	RNASEL	ribonuclease L (2',5'-oligoisoadenylate synthetase-dependent)	chr1q25	6041	---	---	---	---	MmugDNA.10394.1.S1_at_RNASEL
MmugDNA.10395.1.S1_at	226101_at	PRKCE	protein kinase C, epsilon	chr2p21	5581	DPCR1	Diffuse panbronchiolitis critical region 1 protein	---	714446	MmugDNA.10395.1.S1_at_PRKCE
MmugDNA.10396.1.S1_at	209573_s_at	C18orf1	chromosome 18 open reading frame 1	chr18p11.2	753	LOC701919	similar to complement component 6	---	701919	MmugDNA.10396.1.S1_at_C18orf1
MmugDNA.10397.1.S1_at	205507_at	ARHGEF15	Rho guanine nucleotide exchange factor (GEF) 15	chr17p13.1	22899	ARHGEF15	Rho guanine nucleotide exchange factor (GEF) 15	---	721968	MmugDNA.10397.1.S1_at_ARHGEF15
MmugDNA.10398.1.S1_at	NA	NA	NA	NA	NA	NA	NA	NA	NA	MmugDNA.10398.1.S1_at_NA
MmugDNA.10398.1.S1_s_at	218151_x_at	GPR172A	G protein-coupled receptor 172A	chr8q24.3	79581	GPR172A	G protein-coupled receptor 172A	---	701712	MmugDNA.10398.1.S1_s_at_GPR172A
MmugDNA.10399.1.S1_at	229124_at	PROK1	prokineticin 1	chr1p21	84432	PROK1	prokineticin 1	---	701766	MmugDNA.10399.1.S1_at_PROK1
MmugDNA.10400.1.S1_at	209574_s_at	C18orf1	chromosome 18 open reading frame 1	chr18p11.2	753	LOC701919	similar to complement component 6	---	701919	MmugDNA.10400.1.S1_at_C18orf1
MmugDNA.10401.1.S1_at	226203_at	---	---	---	---	---	---	---	---	MmugDNA.10401.1.S1_at_---

MmugDNA.1040.1.S1_at	243038_at	RBM43	RNA binding motif protein 43	chr2q23.3	375287	LOC694426	hypothetical protein LOC694426	---	694426	MmugDNA.1040.1.S1_at_RBM43
MmugDNA.10402.1.S1_at	1562598_at	LOC100128292	hypothetical LOC100128292	chr10q22.3	100128292	---	---	---	---	MmugDNA.10402.1.S1_at_LOC1001282
MmugDNA.10403.1.S1_at	201960_s_at	MYCBP2	MYC binding protein 2	chr13q22	23077	LOC705868	Hypothetical protein LOC705868	---	705868	MmugDNA.10403.1.S1_at_MYCBP2
MmugDNA.10404.1.S1_at	219109_at	SPAG16	sperm associated antigen 16	chr2q34	79582	---	---	---	---	MmugDNA.10404.1.S1_at_SPAG16
MmugDNA.10405.1.S1_at	224692_at	PPP1R15B	protein phosphatase 1, regulatory (inhibitor) subunit 15B	chr1q32.1	84919	---	---	---	---	MmugDNA.10405.1.S1_at_PPP1R15B
MmugDNA.10406.1.S1_at	64900_at	FLJ22167	hypothetical protein FLJ22167	chr16q23.1	79583	LOC698122	Hypothetical protein LOC698122	---	698122	MmugDNA.10406.1.S1_at_FLJ22167
MmugDNA.10407.1.S1_at	236169_at	---	---	---	---	---	---	---	---	MmugDNA.10407.1.S1_at_---
MmugDNA.10408.1.S1_at	219987_at	---	---	---	---	---	---	---	---	MmugDNA.10408.1.S1_at_---
MmugDNA.10409.1.S1_at	1554782_at	C2orf19	chromosome 2 open reading frame 19	chr2q37.3	394261	---	---	---	---	MmugDNA.10409.1.S1_at_C2orf19
MmugDNA.10410.1.S1_at	219040_at	CORO7	coronin 7	chr16p13.3	79585	CORO7	coronin 7	---	708677	MmugDNA.10410.1.S1_at_CORO7
MmugDNA.10411.1.S1_at	208715_at	TMCO1	transmembrane and coiled-coil domains 1	chr1q22-q25	54499	---	---	---	---	MmugDNA.10411.1.S1_at_TMCO1
MmugDNA.1041.1.S1_s_at	200641_s_at	YWHAZ	tyrosine 3-monooxygenase/tryptophan 5-monooxygenase activation protein, zeta polypeptide	chr8q23.1	7534	LOC709723	similar to tyrosine 3/tryptophan 5 -monooxygenase activation protein, zeta polypeptide	---	709723	MmugDNA.1041.1.S1_s_at_YWHAZ
MmugDNA.10412.1.S1_at	NA	NA	NA	NA	NA	NA	NA	NA	NA	MmugDNA.10412.1.S1_at_NA
MmugDNA.10412.1.S1_x_at	217179_x_at	---	---	---	---	LOC704375	similar to Ig lambda chain V-I region BL2 precursor	---	704375	MmugDNA.10412.1.S1_x_at_---
MmugDNA.10413.1.S1_at	215242_at	PIGC	phosphatidylinositol glycan anchor biosynthesis, class C	chr1q23-q25	5279	---	---	---	---	MmugDNA.10413.1.S1_at_PIGC
MmugDNA.10414.1.S1_at	243634_at	---	---	---	---	LOC704834	hypothetical protein LOC704834	---	704834	MmugDNA.10414.1.S1_at_---
MmugDNA.10415.1.S1_at	214147_at	C1orf175	chromosome 1 open reading frame 175	chr1p32.3	374977	LOC711105	Hypothetical protein LOC711105	---	711105	MmugDNA.10415.1.S1_at_C1orf175
MmugDNA.10416.1.S1_at	NA	NA	NA	NA	NA	NA	NA	NA	NA	MmugDNA.10416.1.S1_at_NA
MmugDNA.10417.1.S1_at	1553564_at	MACROD2	MACRO domain containing 2	chr20p12.1	140733	---	---	---	---	MmugDNA.10417.1.S1_at_MACROD2
MmugDNA.10418.1.S1_at	1560763_at	---	---	---	---	---	---	---	---	MmugDNA.10418.1.S1_at_---
MmugDNA.10419.1.S1_at	202206_at	ARL4C	ADP-ribosylation factor-like 4C	chr2q37.1	10123	ARL4C	ADP-ribosylation factor-like 4C	---	715121	MmugDNA.10419.1.S1_at_ARL4C
MmugDNA.104.1.S1_at	242396_at	LOC100132798	Similar to hCG1774772	chr15q26.2	100132798	---	---	---	---	MmugDNA.104.1.S1_at_LOC10013279
MmugDNA.10420.1.S1_at	238170_at	---	---	---	---	---	---	---	---	MmugDNA.10420.1.S1_at_---
MmugDNA.10421.1.S1_at	202207_at	ARL4C	ADP-ribosylation factor-like 4C	chr2q37.1	10123	ARL4C	ADP-ribosylation factor-like 4C	---	715121	MmugDNA.10421.1.S1_at_ARL4C
MmugDNA.1042.1.S1_s_at	221875_x_at	HLA-F	major histocompatibility complex, class I, F	chr6p21.3	3134	MAMU-F	major histocompatibility complex, class I, F	---	709076	MmugDNA.1042.1.S1_s_at_HLA-F
MmugDNA.10422.1.S1_s_at	210871_x_at	SSX2IP	synovial sarcoma, X breakpoint 2 interacting protein	chr1p22.3	117178	SSX2IP	synovial sarcoma, X breakpoint 2 interacting protein	---	710730	MmugDNA.10422.1.S1_s_at_SSX2IP
MmugDNA.10423.1.S1_at	205594_at	ZNF652	zinc finger protein 652	chr17q21.32	22834	ZNF652	zinc finger protein 652	---	699084	MmugDNA.10423.1.S1_at_ZNF652
MmugDNA.10424.1.S1_at	1554640_at	PALM2	paralemmin 2	chr9q31-q33	114299	---	---	---	---	MmugDNA.10424.1.S1_at_PALM2
MmugDNA.10425.1.S1_at	202175_at	CHPF	chondroitin polymerizing factor	chr2q35	79586	LOC707783	Hypothetical protein LOC707783	---	707783	MmugDNA.10425.1.S1_at_CHPF
MmugDNA.10426.1.S1_at	204823_at	NAV3	neuron navigator 3	chr12q14.3	89795	LOC695754	Similar to component of oligomeric golgi complex 1	---	695754	MmugDNA.10426.1.S1_at_NAV3
MmugDNA.10427.1.S1_at	218153_at	CARS2	cysteinyl-tRNA synthetase 2, mitochondrial (putative)	chr13q34	79587	CARS2	cysteinyl-tRNA synthetase 2, mitochondrial (putative)	---	694840	MmugDNA.10427.1.S1_at_CARS2
MmugDNA.10428.1.S1_at	236614_at	LOC729683	hypothetical protein LOC729683	chr17q23.3	729683	---	---	---	---	MmugDNA.10428.1.S1_at_LOC729683
MmugDNA.10429.1.S1_at	213204_at	CUL9	cullin 9	chr6p21.1	23113	LOC698984	similar to p53-associated parkin-like cytoplasmic protein	---	698984	MmugDNA.10429.1.S1_at_CUL9
MmugDNA.10430.1.S1_at	227339_at	RGMB	RGM domain family, member B	chr5q21.1	285704	RGMB	RGM domain family, member B	---	701700	MmugDNA.10430.1.S1_at_RGMB
MmugDNA.10431.1.S1_at	227340_s_at	RGMB	RGM domain family, member B	chr5q21.1	285704	RGMB	RGM domain family, member B	---	701700	MmugDNA.10431.1.S1_at_RGMB
MmugDNA.10432.1.S1_at	218270_at	MRPL24	mitochondrial ribosomal protein L24	chr1q21-q22	79590	LOC702055	similar to mitochondrial ribosomal protein L24	---	702055	MmugDNA.10432.1.S1_at_MRPL24
MmugDNA.10433.1.S1_at	1569494_at	---	---	---	---	---	---	---	---	MmugDNA.10433.1.S1_at_---
MmugDNA.10434.1.S1_at	221766_s_at	FAM46A	family with sequence similarity 46, member A	chr6q14	55603	LOC693306	similar to family with sequence similarity 46, member A	---	693306	MmugDNA.10434.1.S1_at_FAM46A
MmugDNA.10435.1.S1_at	224973_at	FAM46A	family with sequence similarity 46, member A	chr6q14	55603	LOC693306	similar to family with sequence similarity 46, member A	---	693306	MmugDNA.10435.1.S1_at_FAM46A
MmugDNA.10436.1.S1_at	1570163_at	---	---	---	---	---	---	---	---	MmugDNA.10436.1.S1_at_---
MmugDNA.10437.1.S1_at	229530_at	GUCY1A3	guanylate cyclase 1, soluble, alpha 3	chr4q31.3-q33|4q31.1-q31.2	2982	---	---	---	---	MmugDNA.10437.1.S1_at_GUCY1A3
MmugDNA.10438.1.S1_at	239254_at	CCDC40	coiled-coil domain containing 40	chr17q25.3	55036	LOC717836	similar to coiled-coil domain containing 40	---	717836	MmugDNA.10438.1.S1_at_CCDC40
MmugDNA.10439.1.S1_at	241047_at	FLJ22536	hypothetical locus LOC401237	chr6p22.3	401237	LOC706743	hypothetical protein LOC706743	---	706743	MmugDNA.10439.1.S1_at_FLJ22536
MmugDNA.10440.1.S1_at	224470_at	SEC22C	SEC22 vesicle trafficking protein homolog C (S. cerevisiae)	chr3p22.1	9117	LOC716351	similar to SEC22 vesicle trafficking protein homolog C isoform b	---	716351	MmugDNA.10440.1.S1_at_SEC22C
MmugDNA.10441.1.S1_at	236200_at	---	---	---	---	---	---	---	---	MmugDNA.10441.1.S1_at_---
MmugDNA.1044.1.S1_at	227185_at	LOC643988	hypothetical LOC643988	chr1p36.33	643988	---	---	---	---	MmugDNA.1044.1.S1_at_LOC643988
MmugDNA.10442.1.S1_s_at	216899_s_at	SKAP2	src kinase associated phosphoprotein 2	chr7p21-p15	8935	---	---	---	---	MmugDNA.10442.1.S1_s_at_SKAP2
MmugDNA.10443.1.S1_s_at	205773_at	CPEB3	cytoplasmic polyadenylation element binding protein 3	chr10q23.32	22849	CPEB3	cytoplasmic polyadenylation element binding protein 3	---	698133	MmugDNA.10443.1.S1_s_at_CPEB3
MmugDNA.10444.1.S1_at	211784_s_at	SFRS1	splicing factor, arginine/serine-rich 1	chr17q21.3-q22	6426	LOC713776	similar to splicing factor, arginine/serine-rich 1 (ASF/SF2)	---	713776	MmugDNA.10444.1.S1_at_SFRS1
MmugDNA.10445.1.S1_at	203884_s_at	RAB11FIP2	RAB11 family interacting protein 2 (class I)	chr10q26.11	22841	RAB11FIP2	RAB11 family interacting protein 2 (class I)	---	711468	MmugDNA.10445.1.S1_at_RAB11FIP2
MmugDNA.10446.1.S1_at	203355_s_at	PSD3	pleckstrin and Sec7 domain containing 3	chr8pter-p23.3	23362	LOC712115	similar to ADP-ribosylation factor guanine nucleotide factor 6 isoform a	---	712115	MmugDNA.10446.1.S1_at_PSD3
MmugDNA.10447.1.S1_at	232434_at	DIRC3	disrupted in renal carcinoma 3	chr2q35	729582	---	---	---	---	MmugDNA.10447.1.S1_at_DIRC3
MmugDNA.10448.1.S1_at	221547_at	PRPF18	PRP18 pre-mRNA processing factor 18 homolog (S. cerevisiae)	chr10p13	8559	PRPF18	PRP18 pre-mRNA processing factor 18 homolog	---	696401	MmugDNA.10448.1.S1_at_PRPF18
MmugDNA.10449.1.S1_at	229319_at	---	---	---	---	---	---	---	---	MmugDNA.10449.1.S1_at_---
MmugDNA.10450.1.S1_at	221610_s_at	STAP2	signal transducing adaptor family member 2	chr19p13.3	55620	LOC718949	similar to signal-transducing adaptor protein-2 isoform 2	---	718949	MmugDNA.10450.1.S1_at_STAP2
MmugDNA.10451.1.S1_at	NA	NA	NA	NA	NA	NA	NA	NA	NA	MmugDNA.10451.1.S1_at_NA
MmugDNA.1045.1.S1_at	239950_at	HOXA11AS	HOXA11 antisense RNA (non-protein coding)	chr7p15.2	221883	---	---	---	---	MmugDNA.1045.1.S1_at_HOXA11AS
MmugDNA.10452.1.S1_at	1554655_a_at	RPRML	reprimo-like	chr17q21.32	388394	LOC716801	similar to reprimo-like	---	716801	MmugDNA.10452.1.S1_at_RPRML
MmugDNA.10453.1.S1_at	1552368_at	CTCFL	CCCTC-binding factor (zinc finger protein)-like	chr20q13.31	140690	CTCFL	CCCTC-binding factor (zinc finger protein)-like	---	701500	MmugDNA.10453.1.S1_at_CTCFL
MmugDNA.10454.1.S1_at	1559880_at	---	---	---	---	---	---	---	---	MmugDNA.10454.1.S1_at_---
MmugDNA.10455.1.S1_at	1557777_at	PDE6B	phosphodiesterase 6B, cGMP-specific, rod, beta	chr4p16.3	5158	---	---	---	---	MmugDNA.10455.1.S1_at_PDE6B
MmugDNA.10456.1.S1_at	232530_at	PLD1	phospholipase D1, phosphatidylcholine-specific	chr3q26	5337	---	---	---	---	MmugDNA.10456.1.S1_at_PLD1
MmugDNA.10457.1.S1_at	240817_at	---	---	---	---	LOC709504	hypothetical protein LOC709504	---	709504	MmugDNA.10457.1.S1_at_---
MmugDNA.10458.1.S1_at	226463_at	ATP6V1C1	ATPase, H+ transporting, lysosomal 42kDa, V1 subunit C1	chr8q22.3	528	---	---	---	---	MmugDNA.10458.1.S1_at_ATP6V1C1
MmugDNA.10459.1.S1_at	239468_at	MKX	mohawk homeobox	chr10p12.1	283078	---	---	---	---	MmugDNA.10459.1.S1_at_MKX
MmugDNA.10460.1.S1_at	242856_at	---	---	---	---	---	---	---	---	MmugDNA.10460.1.S1_at_---
MmugDNA.10461.1.S1_at	1566037_at	LOC729314	similar to POM121-like 2	chr22q11.21	729314	---	---	---	---	MmugDNA.10461.1.S1_at_LOC729314
MmugDNA.1046.1.S1_at	NA	NA	NA	NA	NA	NA	NA	NA	NA	MmugDNA.1046.1.S1_at_NA
MmugDNA.1046.1.S1_s_at	210982_s_at	HLA-DRA	major histocompatibility complex, class II, DR alpha	chr6p21.3	3122	MAMU-DRA	major histocompatibility complex, class II, DR alpha	---	100187587	MmugDNA.1046.1.S1_s_at_HLA-DRA
MmugDNA.10462.1.S1_at	230204_at	HAPLN1	hyaluronan and proteoglycan link protein 1	chr5q14.3	1404	HAPLN1	hyaluronan and proteoglycan link protein 1	---	712501	MmugDNA.10462.1.S1_at_HAPLN1
MmugDNA.10463.1.S1_at	1554085_at	DDX51	DEAD (Asp-Glu-Ala-Asp) box polypeptide 51	chr12q24.33	317781	LOC710113	similar to DEAD (Asp-Glu-Ala-Asp) box polypeptide 51	---	710113	MmugDNA.10463.1.S1_at_DDX51
MmugDNA.10464.1.S1_at	211049_at	TLX2	T-cell leukemia homeobox 2	chr2p13.1-p12	3196	TLX2	T-cell leukemia homeobox 2	---	709649	MmugDNA.10464.1.S1_at_TLX2
MmugDNA.10465.1.S1_at	235046_at	---	---	---	---	---	---	---	---	MmugDNA.10465.1.S1_at_---
MmugDNA.10466.1.S1_at	229204_at	HP1BP3	Heterochromatin protein 1, binding protein 3	chr1p36.12	50809	---	---	---	---	MmugDNA.10466.1.S1_at_HP1BP3
MmugDNA.10467.1.S1_x_at	213737_x_at	GOLGA9P	golgi autoantigen, golgin subfamily a, 9 pseudogene	chr15q11.2	283796	---	---	---	---	MmugDNA.10467.1.S1_x_at_GOLGA9P
MmugDNA.10468.1.S1_at	241998_at	C2orf80	chromosome 2 open reading frame 80	chr2q33.3	389073	LOC709833	hypothetical protein LOC709833	---	709833	MmugDNA.10468.1.S1_at_C2orf80
MmugDNA.10469.1.S1_at	211050_x_at	DKFZP434B2016 /// LO	similar to hypothetical protein LOC284701 /// similar to hypothetical protein LOC284701	chr16q24.3 /// chr1p36.33	642780 /// 643313	---	---	---	---	MmugDNA.10469.1.S1_at_DKFZP434B
MmugDNA.10470.1.S1_at	229973_at	C1orf173	chromosome 1 open reading frame 173	chr1p31.1	127254	LOC708851	similar to cyclic nucleotide gated channel beta 1	---	708851	MmugDNA.10470.1.S1_at_C1orf173
MmugDNA.10471.1.S1_at	240676_at	---	---	---	---	---	---	---	---	MmugDNA.10471.1.S1_at_---
MmugDNA.1047.1.S1_at	240696_at	---	---	---	---	---	---	---	---	MmugDNA.1047.1.S1_at_---
MmugDNA.10472.1.S1_at	238838_at	TCERG1	Transcription elongation regulator 1	chr5q31	10915	---	---	---	---	MmugDNA.10472.1.S1_at_TCERG1
MmugDNA.10473.1.S1_at	1566678_at	MMP2	Matrix metallopeptidase 2 (gelatinase A, 72kDa gelatinase, 72kDa type IV collagenase)	chr16q13-q21	4313	MMP2	matrix metallopeptidase 2 (gelatinase A, 72kDa gelatinase, 72kDa type IV collagenase)	---	698478	MmugDNA.10473.1.S1_at_MMP2
MmugDNA.10474.1.S1_at	233804_at	LOC440131	similar to bA90M5.1 (novel protein)	chr13q12.3	440131	---	---	---	---	MmugDNA.10474.1.S1_at_LOC440131
MmugDNA.10476.1.S1_at	1553333_at	C1orf161	chromosome 1 open reading frame 161	chr1p13.1	126868	---	---	---	---	MmugDNA.10476.1.S1_at_C1orf161
MmugDNA.10477.1.S1_at	224506_s_at	PPAPDC3	phosphatidic acid phosphatase type 2 domain containing 3	chr9q34.13	84814	LOC713200	Similar to zinc finger protein 317	---	713200	MmugDNA.10477.1.S1_at_PPAPDC3
MmugDNA.10478.1.S1_at	1562712_at	---	---	---	---	---	---	---	---	MmugDNA.10478.1.S1_at_---
MmugDNA.10479.1.S1_at	211051_s_at	EXTL3	exostoses (multiple)-like 3	chr8p21	2137	LOC695335	Similar to nuclear factor of kappa light polypeptide gene enhancer in B-cells inhibitor, alpha	---	695335	MmugDNA.10479.1.S1_at_EXTL3
MmugDNA.10480.1.S1_at	211052_s_at	TBCD	tubulin folding cofactor D	chr17q25.3	6904	LOC719682	similar to beta-tubulin cofactor D isoform 1	---	719682	MmugDNA.10480.1.S1_at_TBCD
MmugDNA.10481.1.S1_at	209606_at	CYTIP	cytohesin 1 interacting protein	chr2q11.2	9595	CYTIP	cytohesin 1 interacting protein	---	697677	MmugDNA.10481.1.S1_at_CYTIP
MmugDNA.1048.1.S1_at	1562308_at	LOC283682	hypothetical protein LOC283682	chr15q26.1-q26.2	283682	---	---	---	---	MmugDNA.1048.1.S1_at_LOC283682
MmugDNA.10482.1.S1_at	211053_at	KCNG1	potassium voltage-gated channel, subfamily G, member 1	chr20q13	3755	---	---	---	---	MmugDNA.10482.1.S1_at_KCNG1
MmugDNA.10483.1.S1_at	1560258_a_at	---	---	---	---	---	---	---	---	MmugDNA.10483.1.S1_at_---
MmugDNA.10484.1.S1_at	1560896_a_at	---	---	---	---	---	---	---	---	MmugDNA.10484.1.S1_at_---
MmugDNA.10485.1.S1_at	NA	NA	NA	NA	NA	NA	NA	NA	NA	MmugDNA.10485.1.S1_at_NA
MmugDNA.10486.1.S1_at	235272_at	SBSN	suprabasin	chr19q13.13	374897	LOC718724	similar to suprabasin	---	718724	MmugDNA.10486.1.S1_at_SBSN
MmugDNA.10487.1.S1_at	241181_x_at	---	---	---	---	---	---	---	---	MmugDNA.10487.1.S1_at_---
MmugDNA.10488.1.S1_at	1563878_a_at	LOC338963	hypothetical protein LOC338963	chr15q25.2	338963	---	---	---	---	MmugDNA.10488.1.S1_at_LOC338963
MmugDNA.10489.1.S1_at	229915_at	FAM26F	family with sequence similarity 26, member F	chr6q22.1	441168	LOC715967	hypothetical protein LOC715967	---	715967	MmugDNA.10489.1.S1_at_FAM26F
MmugDNA.10489.1.S1_s_at	NA	NA	NA	NA	NA	NA	NA	NA	NA	MmugDNA.10489.1.S1_s_at_NA
MmugDNA.10490.1.S1_at	1561657_at	---	---	---	---	---	---	---	---	MmugDNA.10490.1.S1_at_---
MmugDNA.10491.1.S1_at	226629_at	SLC43A2	solute carrier family 43, member 2	chr17p13.3	124935	SLC43A2	solute carrier family 43, member 2	---	701666	MmugDNA.10491.1.S1_at_SLC43A2
MmugDNA.1049.1.S1_at	240410_at	---	---	---	---	---	---	---	---	MmugDNA.1049.1.S1_at_---
MmugDNA.10492.1.S1_at	230496_at	FAM123A	family with sequence similarity 123A	chr13q12.13	219287	LOC708730	hypothetical protein LOC708730	---	708730	MmugDNA.10492.1.S1_at_FAM123A
MmugDNA.10493.1.S1_at	241075_at	RIMKLA	ribosomal modification protein rimK-like family member A	chr1p34.2	284716	---	---	---	---	MmugDNA.10493.1.S1_at_RIMKLA
MmugDNA.10494.1.S1_at	203327_at	IDE	insulin-degrading enzyme	chr10q23-q25	3416	IDE	insulin-degrading enzyme	---	698640	MmugDNA.10494.1.S1_at_IDE
MmugDNA.10495.1.S1_at	209704_at	MTF2	metal response element binding transcription factor 2	chr1p22.1	22823	---	---	---	---	MmugDNA.10495.1.S1_at_MTF2
MmugDNA.10496.1.S1_at	209705_at	MTF2	metal response element binding transcription factor 2	chr1p22.1	22823	---	---	---	---	MmugDNA.10496.1.S1_at_MTF2
MmugDNA.10497.1.S1_at	237709_at	---	---	---	---	---	---	---	---	MmugDNA.10497.1.S1_at_---

MmugDNA.10498.1.S1_at	1566176_at	---	---	---	---		---	---	---	---		MmugDNA.10498.1.S1_at_---
MmugDNA.10499.1.S1_at	211054_at	INVS	inversin	chr9q31		27130	---	---	---	---		MmugDNA.10499.1.S1_at_INVS
MmugDNA.10500.1.S1_at	235605_at	CASZ1	castor zinc finger 1	chr1p36.22		54897	---	---	---	---		MmugDNA.10500.1.S1_at_CASZ1
MmugDNA.10501.1.S1_at	244270_at	---	---	---	---		---	---	---	---		MmugDNA.10501.1.S1_at_---
MmugDNA.1050.1.S1_s_at	220191_at	GKN1	gastrokine 1	chr2p14		56287	LOC713197	similar to gastrokine 1	---		713197	MmugDNA.1050.1.S1_s_at_GKN1
MmugDNA.10502.1.S1_at	226121_at	DHRS13	dehydrogenase/reductase (SDR family) member 13	chr17q11.2		147015	LOC710755	similar to retinol dehydrogenase 11 (predicted)	---		710755	MmugDNA.10502.1.S1_at_DHRS13
MmugDNA.10503.1.S1_at	239760_at	---	---	---	---		---	---	---	---		MmugDNA.10503.1.S1_at_---
MmugDNA.10504.1.S1_at	225204_at	PPTC7	PTC7 protein phosphatase homolog (S. cerevisiae)	chr12q24.11		160760	LOC709471	similar to T-cell activation protein phosphatase 2C	---		709471	MmugDNA.10504.1.S1_at_PPTC7
MmugDNA.10505.1.S1_at	NA	NA	NA	NA	NA		NA	NA	NA	NA		MmugDNA.10505.1.S1_at_NA
MmugDNA.10505.1.S1_s_at	211056_s_at	SRD5A1	steroid-5-alpha-reductase, alpha polypeptide 1 (3-oxo-5 alpha-steroid delta 4-dehydrogenase alpha 1	chr5p15		6715	LOC712079	Similar to barrier to autointegration factor 1	---		712079	MmugDNA.10505.1.S1_s_at_SRD5A1
MmugDNA.10506.1.S1_at	225213_at	PPTC7	PTC7 protein phosphatase homolog (S. cerevisiae)	chr12q24.11		160760	LOC709471	similar to T-cell activation protein phosphatase 2C	---		709471	MmugDNA.10506.1.S1_at_PPTC7
MmugDNA.10507.1.S1_at	NA	NA	NA	NA	NA		NA	NA	NA	NA		MmugDNA.10507.1.S1_at_NA
MmugDNA.10508.1.S1_at	1561196_at	---	---	---	---		---	---	---	---		MmugDNA.10508.1.S1_at_---
MmugDNA.10509.1.S1_s_at	231647_s_at	FCRL5	Fc receptor-like 5	chr1q21		83416	---	---	---	---		MmugDNA.10509.1.S1_s_at_FCRL5
MmugDNA.10510.1.S1_at	231685_at	---	---	---	---		---	---	---	---		MmugDNA.10510.1.S1_at_---
MmugDNA.10511.1.S1_at	227241_at	MUC15	mucin 15, cell surface associated	chr11p14.3		143662	MUC15	mucin 15, cell surface associated	---		700194	MmugDNA.10511.1.S1_at_MUC15
MmugDNA.1051.1.S1_at	229730_at	SMTNL2	smoothelin-like 2	chr17p13.2		342527	LOC709114	similar to smoothelin, like	---		709114	MmugDNA.1051.1.S1_at_SMTNL2
MmugDNA.10512.1.S1_at	227238_at	MUC15	mucin 15, cell surface associated	chr11p14.3		143662	MUC15	mucin 15, cell surface associated	---		700194	MmugDNA.10512.1.S1_at_MUC15
MmugDNA.10513.1.S1_at	241491_at	---	---	---	---		---	---	---	---		MmugDNA.10513.1.S1_at_---
MmugDNA.10514.1.S1_at	243836_at	C2orf21	chromosome 2 open reading frame 21	chr2q35		285175	LOC713007	similar to CG18437-PA	---		713007	MmugDNA.10514.1.S1_at_C2orf21
MmugDNA.10515.1.S1_at	239560_at	---	---	---	---		---	---	---	---		MmugDNA.10515.1.S1_at_---
MmugDNA.10516.1.S1_at	214452_at	BCAT1	branched chain aminotransferase 1, cytosolic	chr12p12.1		586	LOC707321	similar to branched chain aminotransferase 1, cytosolic	---		707321	MmugDNA.10516.1.S1_at_BCAT1
MmugDNA.10517.1.S1_at	1565830_at	---	---	---	---		---	---	---	---		MmugDNA.10517.1.S1_at_---
MmugDNA.10518.1.S1_at	1562817_at	---	---	---	---		---	---	---	---		MmugDNA.10518.1.S1_at_---
MmugDNA.10519.1.S1_at	236817_at	ADAT2	adenosine deaminase, tRNA-specific 2, TAD2 homolog (S. cerevisiae)	chr6q24.2		134637	---	---	---	---		MmugDNA.10519.1.S1_at_ADAT2
MmugDNA.105.1.S1_at	236167_at	---	---	---	---		---	---	---	---		MmugDNA.105.1.S1_at_---
MmugDNA.10520.1.S1_at	241300_at	---	---	---	---		---	---	---	---		MmugDNA.10520.1.S1_at_---
MmugDNA.10521.1.S1_at	211057_at	ROR1	receptor tyrosine kinase-like orphan receptor 1	chr1p32-p31		4919	---	---	---	---		MmugDNA.10521.1.S1_at_ROR1
MmugDNA.1052.1.S1_at	1559653_at	---	---	---	---		---	---	---	---		MmugDNA.1052.1.S1_at_---
MmugDNA.10522.1.S1_at	1561481_at	---	---	---	---		---	---	---	---		MmugDNA.10522.1.S1_at_---
MmugDNA.10523.1.S1_at	232438_at	EPS15L1	epidermal growth factor receptor pathway substrate 15-like 1	chr19p13.11		58513	LOC719502	similar to epidermal growth factor receptor pathway substrate 15-like 1	---		719502	MmugDNA.10523.1.S1_at_EPS15L1
MmugDNA.10524.1.S1_at	233089_at	QRSL1	glutaminyl-tRNA synthase (glutamine-hydrolyzing)-like 1	chr6q21		55278	QRSL1	glutaminyl-tRNA synthase (glutamine-hydrolyzing)-like 1	---		697354	MmugDNA.10524.1.S1_at_QRSL1
MmugDNA.10525.1.S1_at	213965_s_at	CHD5	chromodomain helicase DNA binding protein 5	chr1p36.31		26038	LOC720712	similar to chromodomain helicase DNA binding protein 5	---		720712	MmugDNA.10525.1.S1_at_CHD5
MmugDNA.10526.1.S1_at	201012_at	ANXA1	annexin A1	chr9q12-q21.2|9q12-q21.2		301	ANXA1	annexin A1	---		702946	MmugDNA.10526.1.S1_at_ANXA1
MmugDNA.10527.1.S1_at	1570486_at	SOX6	SRY (sex determining region Y)-box 6	chr11p15.3		55553	SOX6	SRY (sex determining region Y)-box 6	---		697831	MmugDNA.10527.1.S1_at_SOX6
MmugDNA.10528.1.S1_at	216331_at	ITGA7	integrin, alpha 7	chr12q13		3679	LOC707279	similar to integrin alpha 7 precursor	---		707279	MmugDNA.10528.1.S1_at_ITGA7
MmugDNA.10529.1.S1_at	244618_at	---	---	---	---		---	---	---	---		MmugDNA.10529.1.S1_at_---
MmugDNA.10530.1.S1_at	242882_at	RNF207	ring finger protein 207	chr1p36.31		388591	---	---	---	---		MmugDNA.10530.1.S1_at_RNF207
MmugDNA.10531.1.S1_at	227717_at	FLJ41603	FLJ41603 protein	chr5q33.1		389337	LOC710950	similar to Cdc42 guanine nucleotide exchange factor (GEF) 9	---		710950	MmugDNA.10531.1.S1_at_FLJ41603
MmugDNA.1053.1.S1_at	202656_s_at	SERTAD2	SERTA domain containing 2	chr2p14		9792	---	---	---	---		MmugDNA.1053.1.S1_at_SERTAD2
MmugDNA.10532.1.S1_s_at	228009_x_at	ZNRD1	zinc ribbon domain containing 1	chr6p21.3		30834	ZNRD1	zinc ribbon domain containing 1	---		712151	MmugDNA.10532.1.S1_s_at_ZNRD1
MmugDNA.10533.1.S1_at	242054_s_at	---	---	---	---		---	---	---	---		MmugDNA.10533.1.S1_at_---
MmugDNA.10534.1.S1_at	1565849_a_at	LOC100170229	hypothetical protein LOC100170229	---		100170229	LOC711598	Similar to Transmembrane anchor protein 1	---		711598	MmugDNA.10534.1.S1_at_LOC10017022
MmugDNA.10536.1.S1_at	242812_at	TRIM26	Tripartite motif-containing 26	chr6p21.3		7726	---	---	---	---		MmugDNA.10536.1.S1_at_TRIM26
MmugDNA.10537.1.S1_at	231152_at	INO80D	INO80 complex subunit D	chr2q33.3		54891	---	---	---	---		MmugDNA.10537.1.S1_at_INO80D
MmugDNA.10538.1.S1_at	211059_s_at	GOLGA2	golgi autoantigen, golgin subfamily a, 2	chr9q34.11		2801	LOC717677	similar to Golgi autoantigen, golgin subfamily a, 2	---		717677	MmugDNA.10538.1.S1_at_GOLGA2
MmugDNA.10538.1.S1_s_at	35436_at	GOLGA2	golgi autoantigen, golgin subfamily a, 2	chr9q34.11		2801	LOC717677	similar to Golgi autoantigen, golgin subfamily a, 2	---		717677	MmugDNA.10538.1.S1_s_at_GOLGA2
MmugDNA.10539.1.S1_s_at	215690_x_at	GPAA1	glycosylphosphatidylinositol anchor attachment protein 1 homolog (yeast)	chr8q24.3		8733	GPAA1	glycosylphosphatidylinositol anchor attachment protein 1	---		699887	MmugDNA.10539.1.S1_s_at_GPAA1
MmugDNA.10540.1.S1_at	224508_at	MGC12916	hypothetical protein MGC12916	chr17p11.2		84815	LOC718334	hypothetical protein LOC718334	---		718334	MmugDNA.10540.1.S1_at_MGC12916
MmugDNA.10541.1.S1_at	1561084_at	---	---	---	---		---	---	---	---		MmugDNA.10541.1.S1_at_---
MmugDNA.1054.1.S1_at	1562961_at	---	---	---	---		---	---	---	---		MmugDNA.1054.1.S1_at_---
MmugDNA.10542.1.S1_at	229164_s_at	ABTB1	ankyrin repeat and BTB (POZ) domain containing 1	chr3q21		80325	LOC710603	similar to ankyrin repeat and BTB (POZ) domain containing 1 isoform 2	---		710603	MmugDNA.10542.1.S1_at_ABTB1
MmugDNA.10543.1.S1_at	238433_at	SNX5	sorting nexin 5	chr20p11		27131	---	---	---	---		MmugDNA.10543.1.S1_at_SNX5
MmugDNA.10544.1.S1_at	1566937_at	---	---	---	---		---	---	---	---		MmugDNA.10544.1.S1_at_---
MmugDNA.10545.1.S1_at	236540_at	---	---	---	---		---	---	---	---		MmugDNA.10545.1.S1_at_---
MmugDNA.10546.1.S1_at	238143_at	LOC646627	phospholipase inhibitor	chr1q44		646627	LOC710465	hypothetical protein LOC710465	---		710465	MmugDNA.10546.1.S1_at_LOC646627
MmugDNA.10547.1.S1_at	233764_s_at	LARP2	La ribonucleoprotein domain family, member 2	chr4q28.2		55132	LARP2	La ribonucleoprotein domain family, member 2	---		693747	MmugDNA.10547.1.S1_at_LARP2
MmugDNA.10548.1.S1_at	227251_at	WDR22	WD repeat domain 22	chr14q23-q24.1		8816	---	---	---	---		MmugDNA.10548.1.S1_at_WDR22
MmugDNA.10549.1.S1_at	1553063_at	GPR78	G protein-coupled receptor 78	chr4p16.1		27201	LOC708831	similar to G protein-coupled receptor 78	---		708831	MmugDNA.10549.1.S1_at_GPR78
MmugDNA.10550.1.S1_at	1569960_at	BRD7P3	bromodomain containing 7 pseudogene 3	chr6q22.1-q22.33		23629	---	---	---	---		MmugDNA.10550.1.S1_at_BRD7P3
MmugDNA.10551.1.S1_at	211061_s_at	MGAT2	mannosyl (alpha-1,6-)-glycoprotein beta-1,2-N-acetylglucosaminyltransferase	chr14q21		4247	MGAT2	similar to Alpha-1,6-mannosyl-glycoprotein 2-beta-N-acetylglucosaminyltransferase (Mannoside acety	---		706426	MmugDNA.10551.1.S1_at_MGAT2
MmugDNA.1055.1.S1_at	236681_at	HOXD13	homeobox D13	chr2q31.1		3239	LOC703825	similar to Homeobox protein Hox-D13 (Hox-4.8)	---		703825	MmugDNA.1055.1.S1_at_HOXD13
MmugDNA.10552.1.S1_at	1560968_at	---	---	---	---		---	---	---	---		MmugDNA.10552.1.S1_at_---
MmugDNA.10553.1.S1_at	232560_at	UROS	uroporphyrinogen III synthase	chr10q25.2-q26.3		7390	---	---	---	---		MmugDNA.10553.1.S1_at_UROS
MmugDNA.10554.1.S1_at	1569659_at	---	---	---	---		---	---	---	---		MmugDNA.10554.1.S1_at_---
MmugDNA.10555.1.S1_at	229449_at	---	---	---	---		---	---	---	---		MmugDNA.10555.1.S1_at_---
MmugDNA.10556.1.S1_at	236812_at	STMN4	stathmin-like 4	chr8p21.2		81551	---	---	---	---		MmugDNA.10556.1.S1_at_STMN4
MmugDNA.10557.1.S1_at	235403_at	---	---	---	---		---	---	---	---		MmugDNA.10557.1.S1_at_---
MmugDNA.10558.1.S1_at	225131_at	ZRANB1	zinc finger, RAN-binding domain containing 1	chr10q26.13		54764	ZRANB1	similar to TRAF-binding protein	---		697541	MmugDNA.10558.1.S1_at_ZRANB1
MmugDNA.10559.1.S1_at	215877_at	C14orf56	chromosome 14 open reading frame 56	chr14q24.2		89919	---	---	---	---		MmugDNA.10559.1.S1_at_C14orf56
MmugDNA.10560.1.S1_at	225138_at	ZRANB1	zinc finger, RAN-binding domain containing 1	chr10q26.13		54764	ZRANB1	similar to TRAF-binding protein	---		697541	MmugDNA.10560.1.S1_at_ZRANB1
MmugDNA.10561.1.S1_at	233457_at	---	---	---	---		---	---	---	---		MmugDNA.10561.1.S1_at_---
MmugDNA.1056.1.S1_at	1553957_at	ZNF564	zinc finger protein 564	chr19p13.2		163050	---	---	---	---		MmugDNA.1056.1.S1_at_ZNF564
MmugDNA.10562.1.S1_at	225130_at	ZRANB1	zinc finger, RAN-binding domain containing 1	chr10q26.13		54764	---	---	---	---		MmugDNA.10562.1.S1_at_ZRANB1
MmugDNA.10563.1.S1_at	244660_at	ELAVL1	ELAV (embryonic lethal, abnormal vision, Drosophila)-like 1 (Hu antigen R)	chr19p13.2		1994	---	---	---	---		MmugDNA.10563.1.S1_at_ELAVL1
MmugDNA.10564.1.S1_at	226475_at	FAM118A	family with sequence similarity 118, member A	chr22q13		55007	LOC713415	similar to Protein FAM118A	---		713415	MmugDNA.10564.1.S1_at_FAM118A
MmugDNA.10565.1.S1_at	239861_at	---	---	---	---		---	---	---	---		MmugDNA.10565.1.S1_at_---
MmugDNA.10566.1.S1_at	1560485_at	HIVEP1	human immunodeficiency virus type I enhancer binding protein 1	chr6p24-p22.3		3096	---	---	---	---		MmugDNA.10566.1.S1_at_HIVEP1
MmugDNA.10567.1.S1_at	243841_at	SYNE2	spectrin repeat containing, nuclear envelope 2	chr14q23.2		23224	LOC706759	similar to spectrin repeat containing, nuclear envelope 2 isoform e	---		706759	MmugDNA.10567.1.S1_at_SYNE2
MmugDNA.10568.1.S1_at	235801_at	---	---	---	---		---	---	---	---		MmugDNA.10568.1.S1_at_---
MmugDNA.10569.1.S1_at	NA	NA	NA	NA	NA		NA	NA	NA	NA		MmugDNA.10569.1.S1_at_NA
MmugDNA.10570.1.S1_at	211062_s_at	CPZ	carboxypeptidase Z	chr4p16.1		8532	---	---	---	---		MmugDNA.10570.1.S1_at_CPZ
MmugDNA.10571.1.S1_at	1560739_a_at	---	---	---	---		---	---	---	---		MmugDNA.10571.1.S1_at_---
MmugDNA.1057.1.S1_at	200880_at	DNAJA1	DnaJ (Hsp40) homolog, subfamily A, member 1	chr9p13-p12		3301	DNAJA1	DnaJ (Hsp40) homolog, subfamily A, member 1	---		703765	MmugDNA.1057.1.S1_at_DNAJA1
MmugDNA.10572.1.S1_at	227181_at	LNP1	leukemia NUP98 fusion partner 1	chr3q12.2		348801	LOC700480	hypothetical protein LOC700480	---		700480	MmugDNA.10572.1.S1_at_LNP1
MmugDNA.10573.1.S1_at	211584_s_at	NPAT	nuclear protein, ataxia-telangiectasia locus	chr11q22-q23		4863	NPAT	nuclear protein, ataxia-telangiectasia locus	---		707873	MmugDNA.10573.1.S1_at_NPAT
MmugDNA.10574.1.S1_at	202652_at	APBB1	amyloid beta (A4) precursor protein-binding, family B, member 1 (Fe65)	chr11p15		322	LOC712585	similar to amyloid beta A4 precursor protein-binding, family B, member 1 isoform delta E9	---		712585	MmugDNA.10574.1.S1_at_APBB1
MmugDNA.10575.1.S1_at	224509_s_at	RTN4IP1	reticulon 4 interacting protein 1	chr6q21		84816	LOC697236	similar to reticulon 4 interacting protein 1	---		697236	MmugDNA.10575.1.S1_at_RTN4IP1
MmugDNA.10576.1.S1_at	211829_s_at	GPER	G protein-coupled estrogen receptor 1	chr7p22.3		2852	GPER	G protein-coupled estrogen receptor 1	---		696625	MmugDNA.10576.1.S1_at_GPER
MmugDNA.10577.1.S1_at	242066_at	---	---	---	---		---	---	---	---		MmugDNA.10577.1.S1_at_---
MmugDNA.10578.1.S1_at	213440_at	RAB1A	RAB1A, member RAS oncogene family	chr2p14		5861	RAB1A	RAB1A, member RAS oncogene family	---		696962	MmugDNA.10578.1.S1_at_RAB1A
MmugDNA.10579.1.S1_at	236783_at	KCNIP4	Kv channel interacting protein 4	chr4p15.31		80333	LOC695613	Similar to dopamine receptor interacting protein	---		695613	MmugDNA.10579.1.S1_at_KCNIP4
MmugDNA.10580.1.S1_at	203346_s_at	MTF2	metal response element binding transcription factor 2	chr1p22.1		22823	LOC702671	Similar to Aprataxin (Forkhead-associated domain histidine triad-like protein) (FHA-HIT)	---		702671	MmugDNA.10580.1.S1_at_MTF2
MmugDNA.10581.1.S1_s_at	217368_at	ATP5G2	ATP synthase, H+ transporting, mitochondrial F0 complex, subunit C2 (subunit 9)	chr12q13.13		517	LOC713542	similar to ATP synthase lipid-binding protein, mitochondrial precursor (ATP synthase proteolipid P2) (	---		713542	MmugDNA.10581.1.S1_s_at_ATP5G2
MmugDNA.1058.1.S1_at	239095_at	---	---	---	---		---	---	---	---		MmugDNA.1058.1.S1_at_---
MmugDNA.10582.1.S1_at	217202_s_at	GLUL	glutamate-ammonia ligase (glutamine synthetase)	chr1q31		2752	GLUL	similar to Glutamine synthetase (Glutamate--ammonia ligase) (GS)	---		703535	MmugDNA.10582.1.S1_at_GLUL
MmugDNA.10583.1.S1_at	1555291_at	TRPV3	transient receptor potential cation channel, subfamily V, member 3	chr17p13.3		162514	LOC721450	similar to transient receptor potential cation channel, subfamily V, member 3	---		721450	MmugDNA.10583.1.S1_at_TRPV3
MmugDNA.10584.1.S1_s_at	228019_s_at	MRPS18C	mitochondrial ribosomal protein S18C	chr4q21.23		51023	MRPS18C	mitochondrial ribosomal protein S18C	---		707257	MmugDNA.10584.1.S1_s_at_MRPS18C
MmugDNA.10585.1.S1_at	NA	NA	NA	NA	NA		NA	NA	NA	NA		MmugDNA.10585.1.S1_at_NA
MmugDNA.10586.1.S1_at	1553072_at	BNIPL	BCL2/adenovirus E1B 19kD interacting protein like	chr1q21.2		149428	LOC716312	similar to BCL2/adenovirus E1B 19kD interacting protein like isoform 1	---		716312	MmugDNA.10586.1.S1_at_BNIPL
MmugDNA.10587.1.S1_at	211063_s_at	NCK1	NCK adaptor protein 1	chr3q21		4690	NCK1	NCK adaptor protein 1	---		716807	MmugDNA.10587.1.S1_at_NCK1
MmugDNA.10588.1.S1_at	213249_at	FBXL7	F-box and leucine-rich repeat protein 7	chr5p15.1		23194	FBXL7	F-box and leucine-rich repeat protein 7	---		699367	MmugDNA.10588.1.S1_at_FBXL7
MmugDNA.10589.1.S1_at	224510_s_at	CLPB	ClpB caseinolytic peptidase B homolog (E. coli)	chr11q13.4		81570	---	---	---	---		MmugDNA.10589.1.S1_at_CLPB
MmugDNA.10590.1.S1_at	206537_at	XIAP	X-linked inhibitor of apoptosis	chrXq25		331	---	---	---	---		MmugDNA.10590.1.S1_at_XIAP
MmugDNA.10591.1.S1_at	1566481_at	---	---	---	---		---	---	---	---		MmugDNA.10591.1.S1_at_---
MmugDNA.1059.1.S1_at	238510_at	ZNF720	zinc finger protein 720	chr16p11.2		124411	LOC714758	similar to Zinc finger protein 267 (Zinc finger protein HZF2)	---		714758	MmugDNA.1059.1.S1_at_ZNF720
MmugDNA.10592.1.S1_at	237365_at	---	---	---	---		---	---	---	---		MmugDNA.10592.1.S1_at_---
MmugDNA.10593.1.S1_at	227861_at	TMEM161B	transmembrane protein 161B	chr5q14.3		153396	TMEM161B	transmembrane protein 161B	---		694351	MmugDNA.10593.1.S1_at_TMEM161B

MmugDNA.10594.1.S1_at	221809_at	RANBP10	RAN binding protein 10	chr16q22.1		57610	LOC700911	similar to Ran-binding protein 10	---		700911	MmugDNA.10594.1.S1_at_RANBP10
MmugDNA.10595.1.S1_at	201964_at	SETX	senataxin	chr9q34.13		23064	---	---	---	---		MmugDNA.10595.1.S1_at_SETX
MmugDNA.10596.1.S1_s_at	208704_x_at	APLP2	amyloid beta (A4) precursor-like protein 2	chr11q23-q25|11q24		334	LOC710658	Similar to TBP-1 interacting protein isoform 1	---		710658	MmugDNA.10596.1.S1_s_at_APLP2
MmugDNA.10597.1.S1_at	236368_at	KIAA0368	KIAA0368	chr9q31.3		23392	---	---	---	---		MmugDNA.10597.1.S1_at_KIAA0368
MmugDNA.10598.1.S1_s_at	219465_at	APOA2	apolipoprotein A-II	chr1q21-q23		336	APOA2	apolipoprotein A-II	---		719946	MmugDNA.10598.1.S1_s_at_APOA2
MmugDNA.10599.1.S1_at	237314_at	ENKUR	enkurin, TRPC channel interacting protein	chr10p12.1		219670	---	---	---	---		MmugDNA.10599.1.S1_at_ENKUR
MmugDNA.10600.1.S1_at	1560149_at	SLC29A2	solute carrier family 29 (nucleoside transporters), member 2	chr11q13		3177	---	---	---	---		MmugDNA.10600.1.S1_at_SLC29A2
MmugDNA.10601.1.S1_at	NA	NA	NA	NA	NA		NA	NA	NA	NA		MmugDNA.10601.1.S1_at_NA
MmugDNA.10601.1.S1_s_at	209536_s_at	EHD4	EH-domain containing 4	chr15q11.1		30844	EHD4	EH-domain containing 4	---		707376	MmugDNA.10601.1.S1_s_at_EHD4
MmugDNA.1060.1.S1_at	1554185_at	LOC554206	hypothetical LOC554206	chr16p12.1		554206	---	---	---	---		MmugDNA.1060.1.S1_at_LOC554206
MmugDNA.10602.1.S1_at	211981_at	COL4A1	collagen, type IV, alpha 1	chr13q34		1282	---	---	---	---		MmugDNA.10602.1.S1_at_COL4A1
MmugDNA.10603.1.S1_at	1564050_at	LOC642808	similar to RAD52 motif 1	chr17q12-q21.2		642808	---	---	---	---		MmugDNA.10603.1.S1_at_LOC642808
MmugDNA.10604.1.S1_at	1560024_at	---	---	---	---		---	---	---	---		MmugDNA.10604.1.S1_at_---
MmugDNA.10606.1.S1_at	NA	NA	NA	NA	NA		NA	NA	NA	NA		MmugDNA.10606.1.S1_at_NA
MmugDNA.10607.1.S1_s_at	224511_s_at	TXNDC17	thioredoxin domain containing 17	chr17p13.2		84817	LOC712910	similar to thioredoxin-like 5	---		712910	MmugDNA.10607.1.S1_s_at_TXNDC17
MmugDNA.10608.1.S1_s_at	224512_s_at	LSMD1	LSM domain containing 1	chr17p13.1		84316	LOC716462	similar to MAK31-like protein	---		716462	MmugDNA.10608.1.S1_s_at_LSMD1
MmugDNA.10609.1.S1_at	211064_at	ZNF493	zinc finger protein 493	chr19p12		284443	---	---	---	---		MmugDNA.10609.1.S1_at_ZNF493
MmugDNA.10610.1.S1_s_at	224513_s_at	UBQLN4	ubiquilin 4	chr1q21		56893	LOC721305	similar to ataxin-1 ubiquitin-like interacting protein	---		721305	MmugDNA.10610.1.S1_s_at_UBQLN4
MmugDNA.10611.1.S1_at	233654_at	PIGG	phosphatidylinositol glycan anchor biosynthesis, class G	chr4p16.3		54872	---	---	---	---		MmugDNA.10611.1.S1_at_PIGG
MmugDNA.1061.1.S1_at	1554187_at	LOC554206	hypothetical LOC554206	chr16p12.1		554206	---	---	---	---		MmugDNA.1061.1.S1_at_LOC554206
MmugDNA.10612.1.S1_at	237076_at	NCSTN	nicastrin	chr1q22-q23		23385	NCSTN	nicastrin	---		719696	MmugDNA.10612.1.S1_at_NCSTN
MmugDNA.10613.1.S1_at	1552258_at	NCRNA00152	non-protein coding RNA 152	chr2p11.2		112597	LOC701028	hypothetical protein LOC701028	---		701028	MmugDNA.10613.1.S1_at_NCRNA0015
MmugDNA.10614.1.S1_at	1562637_at	SAMD12	sterile alpha motif domain containing 12	chr8q24.12		401474	---	---	---	---		MmugDNA.10614.1.S1_at_SAMD12
MmugDNA.10615.1.S1_at	240897_at	---	---	---	---		---	---	---	---		MmugDNA.10615.1.S1_at_---
MmugDNA.10616.1.S1_at	236358_at	---	---	---	---		---	---	---	---		MmugDNA.10616.1.S1_at_---
MmugDNA.10617.1.S1_s_at	235580_at	ZNF141	zinc finger protein 141	chr4p16.3		7700	---	---	---	---		MmugDNA.10617.1.S1_s_at_ZNF141
MmugDNA.10618.1.S1_at	225173_at	ARHGAP18	Rho GTPase activating protein 18	chr6q22.33		93663	LOC711107	similar to Rho GTPase activating protein 18	---		711107	MmugDNA.10618.1.S1_at_ARHGAP18
MmugDNA.10619.1.S1_at	231687_at	---	---	---	---		---	---	---	---		MmugDNA.10619.1.S1_at_---
MmugDNA.106.1.S1_at	233636_at	MIRHG1	microRNA host gene 1 (non-protein coding)	chr13q31.3		407975	---	---	---	---		MmugDNA.106.1.S1_at_MIRHG1
MmugDNA.10620.1.S1_at	225166_at	ARHGAP18	Rho GTPase activating protein 18	chr6q22.33		93663	LOC711107	similar to Rho GTPase activating protein 18	---		711107	MmugDNA.10620.1.S1_at_ARHGAP18
MmugDNA.10621.1.S1_at	226025_at	ANKRD28	ankyrin repeat domain 28	chr3p24.3		23243	---	---	---	---		MmugDNA.10621.1.S1_at_ANKRD28
MmugDNA.1062.1.S1_at	NA	NA	NA	NA	NA		NA	NA	NA	NA		MmugDNA.1062.1.S1_at_NA
MmugDNA.10622.1.S1_at	216454_at	TRMT1	TRM1 tRNA methyltransferase 1 homolog (S. cerevisiae)	chr19p13.13		55621	---	---	---	---		MmugDNA.10622.1.S1_at_TRMT1
MmugDNA.10623.1.S1_at	216702_x_at	---	---	---	---		---	---	---	---		MmugDNA.10623.1.S1_at_---
MmugDNA.10624.1.S1_at	215423_at	---	---	---	---		---	---	---	---		MmugDNA.10624.1.S1_at_---
MmugDNA.10625.1.S1_at	230180_at	---	---	---	---		---	---	---	---		MmugDNA.10625.1.S1_at_---
MmugDNA.10626.1.S1_at	238370_x_at	RPL22	Ribosomal protein L22	chr1p36.3-p36.2		6146	---	---	---	---		MmugDNA.10626.1.S1_at_RPL22
MmugDNA.10627.1.S1_at	202053_s_at	ALDH3A2	aldehyde dehydrogenase 3 family, member A2	chr17p11.2		224	ALDH3A2	aldehyde dehydrogenase 3 family, member A2	---		703661	MmugDNA.10627.1.S1_at_ALDH3A2
MmugDNA.10628.1.S1_at	211023_at	PDHB	pyruvate dehydrogenase (lipoamide) beta	chr3p21.1-p14.2		5162	---	---	---	---		MmugDNA.10628.1.S1_at_PDHB
MmugDNA.10629.1.S1_at	215171_s_at	TIMM17A	translocase of inner mitochondrial membrane 17 homolog A (yeast)	chr1q32.1		10440	TIMM17A	translocase of inner mitochondrial membrane 17 A-like protein	---		707001	MmugDNA.10629.1.S1_at_TIMM17A
MmugDNA.10630.1.S1_s_at	NA	NA	NA	NA	NA		NA	NA	NA	NA		MmugDNA.10630.1.S1_s_at_NA
MmugDNA.10631.1.S1_at	222594_s_at	SPATS2	spermatogenesis associated, serine-rich 2	chr12q13.12		65244	---	---	---	---		MmugDNA.10631.1.S1_at_SPATS2
MmugDNA.1063.1.S1_at	1554667_s_at	METTL8	methyltransferase like 8	chr2q31.1		79828	LOC695361	similar to Methyltransferase-like protein	---		695361	MmugDNA.1063.1.S1_at_METTL8
MmugDNA.10632.1.S1_at	230278_at	---	---	---	---		---	---	---	---		MmugDNA.10632.1.S1_at_---
MmugDNA.10633.1.S1_s_at	242372_s_at	MFSD4	major facilitator superfamily domain containing 4	chr1q32.1		148808	MFSD4	major facilitator superfamily domain containing 4	---		697813	MmugDNA.10633.1.S1_s_at_MFSD4
MmugDNA.10634.1.S1_at	228296_at	YPEL1	yippee-like 1 (Drosophila)	chr22q11.2		29799	---	---	---	---		MmugDNA.10634.1.S1_at_YPEL1
MmugDNA.10635.1.S1_at	212098_at	LOC151162 /// MGAT5	hypothetical LOC151162 /// mannosyl (alpha-1,6-)-glycoprotein beta-1,6-N-acetyl-glucosaminyltransfe	chr2q21 /// chr2q21.3	151162 /// 4249		---	---	---	---		MmugDNA.10635.1.S1_at_LOC151162
MmugDNA.10637.1.S1_at	224514_x_at	IL17RC	interleukin 17 receptor C	chr3p25.3|3p25.3-p24.1		84818	IL17RC	interleukin 17 receptor C	---		699223	MmugDNA.10637.1.S1_at_IL17RC
MmugDNA.10638.1.S1_at	224840_at	FKBP5	FK506 binding protein 5	chr6p21.3-p21.2		2289	---	---	---	---		MmugDNA.10638.1.S1_at_FKBP5
MmugDNA.10639.1.S1_at	241955_at	HECTD1	HECT domain containing 1	chr14q12		25831	LOC717177	similar to HECT domain containing 1	---		717177	MmugDNA.10639.1.S1_at_HECTD1
MmugDNA.10640.1.S1_s_at	206738_at	APOC4	apolipoprotein C-IV	chr19q13.2		346	APOC4	apolipoprotein C-IV	---		718319	MmugDNA.10640.1.S1_s_at_APOC4
MmugDNA.10641.1.S1_at	1557081_at	RBM25	RNA binding motif protein 25	chr14q24.3		58517	RBM25	RNA binding motif protein 25	---		695911	MmugDNA.10641.1.S1_at_RBM25
MmugDNA.1064.1.S1_at	1563728_at	NCRNA00032	non-protein coding RNA 32	chr9p21		158035	---	---	---	---		MmugDNA.1064.1.S1_at_NCRNA00032
MmugDNA.10642.1.S1_at	NA	NA	NA	NA	NA		NA	NA	NA	NA		MmugDNA.10642.1.S1_at_NA
MmugDNA.10643.1.S1_s_at	NA	NA	NA	NA	NA		NA	NA	NA	NA		MmugDNA.10643.1.S1_s_at_NA
MmugDNA.10644.1.S1_at	1560137_at	---	---	---	---		---	---	---	---		MmugDNA.10644.1.S1_at_---
MmugDNA.10645.1.S1_at	202327_s_at	PKD1	polycystic kidney disease 1 (autosomal dominant)	chr16p13.3		5310	PKD1	polycystic kidney disease 1 (autosomal dominant)	---		694905	MmugDNA.10645.1.S1_at_PKD1
MmugDNA.10646.1.S1_at	236963_at	---	---	---	---		---	---	---	---		MmugDNA.10646.1.S1_at_---
MmugDNA.10647.1.S1_at	207262_at	APOF	apolipoprotein F	chr12q13.2		319	LOC712761	similar to apolipoprotein F precursor	---		712761	MmugDNA.10647.1.S1_at_APOF
MmugDNA.10648.1.S1_at	1562788_at	LOC254099	hypothetical protein LOC254099	chr1p36.33		254099	---	---	---	---		MmugDNA.10648.1.S1_at_LOC254099
MmugDNA.10649.1.S1_at	202268_s_at	NAE1	NEDD8 activating enzyme E1 subunit 1	chr16q22		8883	MRPS22	Mitochondrial ribosomal protein S22	---		715845	MmugDNA.10649.1.S1_at_NAE1
MmugDNA.10650.1.S1_at	1564231_at	IFT80	intraflagellar transport 80 homolog (Chlamydomonas)	chr3q26.1		57560	---	---	---	---		MmugDNA.10650.1.S1_at_IFT80
MmugDNA.10651.1.S1_at	237442_at	---	---	---	---		---	---	---	---		MmugDNA.10651.1.S1_at_---
MmugDNA.1065.1.S1_at	1556571_at	C8orf38	Chromosome 8 open reading frame 38	chr8q22.1		137682	---	---	---	---		MmugDNA.1065.1.S1_at_C8orf38
MmugDNA.10652.1.S1_at	1561293_at	---	---	---	---		---	---	---	---		MmugDNA.10652.1.S1_at_---
MmugDNA.10653.1.S1_at	1561555_at	---	---	---	---		---	---	---	---		MmugDNA.10653.1.S1_at_---
MmugDNA.10654.1.S1_at	233007_at	---	---	---	---		---	---	---	---		MmugDNA.10654.1.S1_at_---
MmugDNA.10655.1.S1_at	238392_at	---	---	---	---		---	---	---	---		MmugDNA.10655.1.S1_at_---
MmugDNA.10656.1.S1_at	243370_at	CAPRIN1	cell cycle associated protein 1	chr11p13		4076	LOC717473	similar to membrane component chromosome 11 surface marker 1 isoform 1	---		717473	MmugDNA.10656.1.S1_at_CAPRIN1
MmugDNA.10657.1.S1_at	236180_at	---	---	---	---		---	---	---	---		MmugDNA.10657.1.S1_at_---
MmugDNA.10658.1.S1_at	237509_at	---	---	---	---		---	---	---	---		MmugDNA.10658.1.S1_at_---
MmugDNA.10659.1.S1_at	244485_at	---	---	---	---		---	---	---	---		MmugDNA.10659.1.S1_at_---
MmugDNA.10660.1.S1_at	224864_at	SRA1	steroid receptor RNA activator 1	chr5q31.3		10011	LOC698169	similar to steroid receptor RNA activator 1	---		698169	MmugDNA.10660.1.S1_at_SRA1
MmugDNA.10661.1.S1_at	233962_at	---	---	---	---		---	---	---	---		MmugDNA.10661.1.S1_at_---
MmugDNA.1066.1.S1_at	236980_at	---	---	---	---		---	---	---	---		MmugDNA.1066.1.S1_at_---
MmugDNA.10662.1.S1_at	238200_at	---	---	---	---		---	---	---	---		MmugDNA.10662.1.S1_at_---
MmugDNA.10663.1.S1_at	227725_at	ST6GALNAC1	ST6 (alpha-N-acetyl-neuraminyl-2,3-beta-galactosyl-1,3)-N-acetylgalactosaminide alpha-2,6-sialyltran	chr17q25.1		55808	ST6GALNAC1	ST6 (alpha-N-acetyl-neuraminyl-2,3-beta-galactosyl-1,3)-N-acetylgalactosaminide alpha-2,6-sialyltran	---		707339	MmugDNA.10663.1.S1_at_ST6GALNAC
MmugDNA.10664.1.S1_at	216022_at	---	---	---	---		---	---	---	---		MmugDNA.10664.1.S1_at_---
MmugDNA.10665.1.S1_at	226883_at	---	---	---	---		LOC707676	Similar to MRS2-like, magnesium homeostasis factor	---		707676	MmugDNA.10665.1.S1_at_---
MmugDNA.10666.1.S1_at	203219_s_at	APRT	adenine phosphoribosyltransferase	chr16q24		353	APRT	adenine phosphoribosyltransferase	---		697715	MmugDNA.10666.1.S1_at_APRT
MmugDNA.10667.1.S1_at	213399_x_at	RPN2	ribophorin II	chr20q12-q13.1		6185	LOC708971	similar to ribophorin II precursor	---		708971	MmugDNA.10667.1.S1_at_RPN2
MmugDNA.10668.1.S1_at	231629_x_at	KLK3	Kallikrein-related peptidase 3	chr19q13.41		354	LOC719444	prostate specific antigen	---		719444	MmugDNA.10668.1.S1_at_KLK3
MmugDNA.10670.1.S1_at	239301_at	---	---	---	---		---	---	---	---		MmugDNA.10670.1.S1_at_---
MmugDNA.10671.1.S1_at	239561_at	---	---	---	---		---	---	---	---		MmugDNA.10671.1.S1_at_---
MmugDNA.1067.1.S1_s_at	208649_s_at	VCP	valosin-containing protein	chr9p13.3		7415	---	---	---	---		MmugDNA.1067.1.S1_s_at_VCP
MmugDNA.10672.1.S1_at	1564385_at	LOC219688	hypothetical protein LOC219688	chr10p12.33		219688	---	---	---	---		MmugDNA.10672.1.S1_at_LOC219688
MmugDNA.10673.1.S1_at	236147_at	---	---	---	---		---	---	---	---		MmugDNA.10673.1.S1_at_---
MmugDNA.10674.1.S1_at	229488_at	OTUD7B	OTU domain containing 7B	chr1q21.2		56957	OTUD7B	OTU domain containing 7B	---		705774	MmugDNA.10674.1.S1_at_OTUD7B
MmugDNA.10675.1.S1_at	209635_at	AP1S1	adaptor-related protein complex 1, sigma 1 subunit	chr7q22.1		1174	LOC719569	similar to AP-1 complex subunit sigma-1A (Adapter-related protein complex 1 sigma-1A subunit) (Sig	---		719569	MmugDNA.10675.1.S1_at_AP1S1
MmugDNA.10676.1.S1_s_at	203791_at	DMXL1	Dmx-like 1	chr5q22		1657	LOC698117	similar to Dmx-like 1	---		698117	MmugDNA.10676.1.S1_s_at_DMXL1
MmugDNA.10677.1.S1_at	234947_s_at	C10orf84	chromosome 10 open reading frame 84	chr10q26.11		63877	LOC701856	hypothetical protein LOC701856	---		701856	MmugDNA.10677.1.S1_at_C10orf84
MmugDNA.10678.1.S1_at	242912_at	P704P	prostate-specific P704P	chr14q11.2		641455	---	---	---	---		MmugDNA.10678.1.S1_at_P704P
MmugDNA.10679.1.S1_at	208737_at	ATP6V1G1	ATPase, H+ transporting, lysosomal 13kDa, V1 subunit G1	chr9q32		9550	LOC699522	similar to vacuolar H+ ATPase G1	---		699522	MmugDNA.10679.1.S1_at_ATP6V1G1
MmugDNA.10680.1.S1_at	202668_at	EFNB2	ephrin-B2	chr13q33		1948	EFNB2	ephrin-B2	---		711288	MmugDNA.10680.1.S1_at_EFNB2
MmugDNA.10681.1.S1_at	208435_s_at	AQP6	aquaporin 6, kidney specific	chr12q13		363	LOC711968	similar to aquaporin 6	---		711968	MmugDNA.10681.1.S1_at_AQP6
MmugDNA.1068.1.S1_at	201626_at	INSIG1	insulin induced gene 1	chr7q36		3638	LOC718513	similar to insulin induced gene 1 isoform 2	---		718513	MmugDNA.1068.1.S1_at_INSIG1
MmugDNA.10682.1.S1_at	206955_at	AQP7	aquaporin 7	chr9p13		364	AQP7	aquaporin 7	---		702836	MmugDNA.10682.1.S1_at_AQP7
MmugDNA.10683.1.S1_at	209534_x_at	AKAP13	A kinase (PRKA) anchor protein 13	chr15q24-q25		11214	---	---	---	---		MmugDNA.10683.1.S1_at_AKAP13
MmugDNA.10684.1.S1_at	206784_at	AQP8	aquaporin 8	chr16p12		343	AQP8	aquaporin 8	---		702167	MmugDNA.10684.1.S1_at_AQP8
MmugDNA.10685.1.S1_at	202586_at	POLR2L	polymerase (RNA) II (DNA directed) polypeptide L, 7.6kDa	chr11p15		5441	LOC717808	Similar to Protein GPR137B (Transmembrane 7 superfamily member 1)	---		717808	MmugDNA.10685.1.S1_at_POLR2L
MmugDNA.10686.1.S1_at	226471_at	GGT7	gamma-glutamyltransferase 7	chr20q11.22		2686	GGT7	gamma-glutamyltransferase 7	---		706813	MmugDNA.10686.1.S1_at_GGT7
MmugDNA.10687.1.S1_s_at	210966_x_at	LARP1	La ribonucleoprotein domain family, member 1	chr5q33.2		23367	LOC714714	similar to la related protein isoform 1	---		714714	MmugDNA.10687.1.S1_s_at_LARP1
MmugDNA.10688.1.S1_at	226470_at	GGT7	gamma-glutamyltransferase 7	chr20q11.22		2686	GGT7	gamma-glutamyltransferase 7	---		706813	MmugDNA.10688.1.S1_at_GGT7
MmugDNA.10689.1.S1_at	236138_at	---	---	---	---		---	---	---	---		MmugDNA.10689.1.S1_at_---
MmugDNA.10690.1.S1_at	1562810_at	---	---	---	---		---	---	---	---		MmugDNA.10690.1.S1_at_---
MmugDNA.10691.1.S1_at	213933_at	PTGER3	prostaglandin E receptor 3 (subtype EP3)	chr1p31.2		5733	---	---	---	---		MmugDNA.10691.1.S1_at_PTGER3
MmugDNA.1069.1.S1_at	243511_at	---	---	---	---		---	---	---	---		MmugDNA.1069.1.S1_at_---
MmugDNA.10692.1.S1_at	222986_s_at	SHISA5	shisa homolog 5 (Xenopus laevis)	chr3p21.31		51246	LOC702542	Similar to ribosomal protein L19	---		702542	MmugDNA.10692.1.S1_at_SHISA5

MmugDNA.10693.1.S1_at	206791_s_at	PDE4C	phosphodiesterase 4C, cAMP-specific (phosphodiesterase E1 dunce homolog, Drosophila)	chr19p13.11		5143	PDE4C	phosphodiesterase 4C, cAMP-specific (phosphodiesterase E1 dunce homolog, Drosophila)	---		720171	MmugDNA.10693.1.S1_at_PDE4C
MmugDNA.10694.1.S1_at	234690_at	---	---	---	---		---	---	---	---		MmugDNA.10694.1.S1_at_---
MmugDNA.10695.1.S1_at	205620_at	F10	coagulation factor X	chr13q34		2159	F10	coagulation factor X	---		714132	MmugDNA.10695.1.S1_at_F10
MmugDNA.10696.1.S1_at	1558238_at	TNRC18	trinucleotide repeat containing 18	chr7p22.1		84629	---	---	---	---		MmugDNA.10696.1.S1_at_TNRC18
MmugDNA.10697.1.S1_at	240989_at	---	---	---	---		---	---	---	---		MmugDNA.10697.1.S1_at_---
MmugDNA.10698.1.S1_at	205774_at	F12	coagulation factor XII (Hageman factor)	chr5q33-qter		2161	F12	coagulation factor XII	---		699875	MmugDNA.10698.1.S1_at_F12
MmugDNA.10699.1.S1_at	207810_at	F13B	coagulation factor XIII, B polypeptide	chr1q31-q32.1		2165	F13B	coagulation factor XIII, B polypeptide	---		711237	MmugDNA.10699.1.S1_at_F13B
MmugDNA.10700.1.S1_at	226810_at	OGFRL1	opioid growth factor receptor-like 1	chr6q13		79627	---	---	---	---		MmugDNA.10700.1.S1_at_OGFRL1
MmugDNA.10701.1.S1_at	240497_at	---	---	---	---		---	---	---	---		MmugDNA.10701.1.S1_at_---
MmugDNA.1070.1.S1_at	228106_at	C4orf30	chromosome 4 open reading frame 30	chr4p15.32		54876	---	---	---	---		MmugDNA.1070.1.S1_at_C4orf30
MmugDNA.1070.1.S1_s_at	NA	NA	NA	NA	NA		NA	NA	NA	NA		MmugDNA.1070.1.S1_s_at_NA
MmugDNA.10702.1.S1_at	1553175_s_at	PDE5A	phosphodiesterase 5A, cGMP-specific	chr4q25-q27		8654	PDE5A	phosphodiesterase 5A, cGMP-specific	---		705592	MmugDNA.10702.1.S1_at_PDE5A
MmugDNA.10703.1.S1_at	237234_at	CYTH2	cytohesin 2	chr19q13.33		9266	---	---	---	---		MmugDNA.10703.1.S1_at_CYTH2
MmugDNA.10704.1.S1_at	1559579_at	LOC283688	Hypothetical protein LOC283688	chr15q25.1		283688	---	---	---	---		MmugDNA.10704.1.S1_at_LOC283688
MmugDNA.10705.1.S1_at	203247_s_at	ZNF24	zinc finger protein 24	chr18q12		7572	LOC702374	Similar to Protein KIAA0195 (Transmembrane protein 94)	---		702374	MmugDNA.10705.1.S1_at_ZNF24
MmugDNA.10706.1.S1_at	222730_s_at	ZDHHC2	zinc finger, DHHC-type containing 2	chr8p21.3-p22		51201	ZDHHC2	zinc finger, DHHC-type containing 2	---		710001	MmugDNA.10706.1.S1_at_ZDHHC2
MmugDNA.10707.1.S1_at	222731_at	ZDHHC2	zinc finger, DHHC-type containing 2	chr8p21.3-p22		51201	ZDHHC2	zinc finger, DHHC-type containing 2	---		710001	MmugDNA.10707.1.S1_at_ZDHHC2
MmugDNA.10708.1.S1_at	225032_at	FNDC3B	fibronectin type III domain containing 3B	chr3q26.31		64778	---	---	---	---		MmugDNA.10708.1.S1_at_FNDC3B
MmugDNA.10709.1.S1_at	238645_at	---	---	---	---		---	---	---	---		MmugDNA.10709.1.S1_at_---
MmugDNA.10710.1.S1_at	1553148_a_at	SNX13	sorting nexin 13	chr7p21.1		23161	SNX13	sorting nexin 13	---		714019	MmugDNA.10710.1.S1_at_SNX13
MmugDNA.10711.1.S1_s_at	217106_x_at	DIMT1L	DIM1 dimethyladenosine transferase 1-like (S. cerevisiae)	chr5q11-q14		27292	LOC697690	Similar to small acidic protein	---		697690	MmugDNA.10711.1.S1_s_at_DIMT1L
MmugDNA.1071.1.S1_s_at	209067_s_at	HNRPDL	heterogeneous nuclear ribonucleoprotein D-like	chr4q13-q21		9987	HNRPDL	heterogeneous nuclear ribonucleoprotein D-like	---		694603	MmugDNA.1071.1.S1_s_at_HNRPDL
MmugDNA.10712.1.S1_at	205239_at	AREG	amphiregulin	chr4q13-q21		374	AREGB	amphiregulin B	---		702396	MmugDNA.10712.1.S1_at_AREG
MmugDNA.10713.1.S1_at	236474_at	---	---	---	---		---	---	---	---		MmugDNA.10713.1.S1_at_---
MmugDNA.10714.1.S1_at	244015_at	---	---	---	---		---	---	---	---		MmugDNA.10714.1.S1_at_---
MmugDNA.10715.1.S1_at	1558983_at	LRRC41	leucine rich repeat containing 41	chr1p33		10489	---	---	---	---		MmugDNA.10715.1.S1_at_LRRC41
MmugDNA.10716.1.S1_at	209316_s_at	HBS1L	HBS1-like (S. cerevisiae)	chr6q23-q24		10767	HBS1L	HBS1-like (S. cerevisiae)	---		706748	MmugDNA.10716.1.S1_at_HBS1L
MmugDNA.10717.1.S1_at	1570038_at	ZNF595	Zinc finger protein 595	chr4p16.3		152687	---	---	---	---		MmugDNA.10717.1.S1_at_ZNF595
MmugDNA.10718.1.S1_at	222400_s_at	ADI1	acireductone dioxygenase 1	chr2p25.3		55256	LOC722050	similar to 1,2-dihydroxy-3-keto-5-methylthiopentene dioxygenase (Aci-reductone dioxygenase) (ARD)	---		722050	MmugDNA.10718.1.S1_at_ADI1
MmugDNA.10718.1.S1_s_at	NA	NA	NA	NA	NA		NA	NA	NA	NA		MmugDNA.10718.1.S1_s_at_NA
MmugDNA.10719.1.S1_at	235954_at	---	---	---	---		---	---	---	---		MmugDNA.10719.1.S1_at_---
MmugDNA.107.1.S1_at	219992_at	TAC3	tachykinin 3	chr12q13-q21		6866	TAC3	tachykinin 3	---		713978	MmugDNA.107.1.S1_at_TAC3
MmugDNA.10720.1.S1_at	231423_s_at	ANKRD16	ankyrin repeat domain 16	chr10p15.1		54522	---	---	---	---		MmugDNA.10720.1.S1_at_ANKRD16
MmugDNA.10721.1.S1_at	240266_at	---	---	---	---		---	---	---	---		MmugDNA.10721.1.S1_at_---
MmugDNA.1072.1.S1_at	221280_s_at	PARD3	par-3 partitioning defective 3 homolog (C. elegans)	chr10p11.22-p11.21		56288	LOC706472	similar to partitioning-defective protein 3 homolog	---		706472	MmugDNA.1072.1.S1_at_PARD3
MmugDNA.10722.1.S1_at	241693_at	---	---	---	---		---	---	---	---		MmugDNA.10722.1.S1_at_---
MmugDNA.10723.1.S1_at	230123_at	NECAP2	NECAP endocytosis associated 2	chr1p36.13		55707	NECAP2	NECAP endocytosis associated 2	---		698687	MmugDNA.10723.1.S1_at_NECAP2
MmugDNA.10724.1.S1_at	237552_at	---	---	---	---		---	---	---	---		MmugDNA.10724.1.S1_at_---
MmugDNA.10726.1.S1_at	229637_at	RIC8B	resistance to inhibitors of cholinesterase 8 homolog B (C. elegans)	chr12q23.3		55188	---	---	---	---		MmugDNA.10726.1.S1_at_RIC8B
MmugDNA.10727.1.S1_at	227823_at	RGAG4	retrotransposon gag domain containing 4	chrXq13.1		340526	LOC713023	Similar to ribosomal protein L7-like 1	---		713023	MmugDNA.10727.1.S1_at_RGAG4
MmugDNA.10728.1.S1_at	201336_at	VAMP3	vesicle-associated membrane protein 3 (cellubrevin)	chr1p36.23		9341	LOC693564	Similar to Insulin-like growth factor-binding protein 7 precursor (IGFBP-7) (IBP-7) (IGF-binding protei	---		693564	MmugDNA.10728.1.S1_at_VAMP3
MmugDNA.10729.1.S1_at	244490_at	---	---	---	---		---	---	---	---		MmugDNA.10729.1.S1_at_---
MmugDNA.10730.1.S1_at	243259_at	ATXN7	ataxin 7	chr3p21.1-p12		6314	---	---	---	---		MmugDNA.10730.1.S1_at_ATXN7
MmugDNA.10731.1.S1_at	1561080_at	TXNRD1	thioredoxin reductase 1	chr12q23-q24.1		7296	---	---	---	---		MmugDNA.10731.1.S1_at_TXNRD1
MmugDNA.1073.1.S1_at	241996_at	---	---	---	---		---	---	---	---		MmugDNA.1073.1.S1_at_---
MmugDNA.10732.1.S1_at	1557558_s_at	LOC100129196	similar to hCG2033298	chr1p35.2		100129196	---	---	---	---		MmugDNA.10732.1.S1_at_LOC100129
MmugDNA.10733.1.S1_s_at	208611_s_at	SPTAN1	spectrin, alpha, non-erythrocytic 1 (alpha-fodrin)	chr9q33-q34		6709	LOC712202	Similar to MIS12 homolog	---		712202	MmugDNA.10733.1.S1_s_at_SPTAN1
MmugDNA.10734.1.S1_at	235431_s_at	PELI3	pellino homolog 3 (Drosophila)	chr11q13.1		246330	PELI3	pellino homolog 3 (Drosophila)	---		714037	MmugDNA.10734.1.S1_at_PELI3
MmugDNA.10735.1.S1_at	225016_at	APCDD1	adenomatosis polyposis coli down-regulated 1	chr18p11.22		147495	LOC722280	similar to adenomatosis polyposis coli down-regulated 1	---		722280	MmugDNA.10735.1.S1_at_APCDD1
MmugDNA.10736.1.S1_at	225333_at	ZNF496	zinc finger protein 496	chr1q44		84838	LOC709694	similar to zinc finger protein 496	---		709694	MmugDNA.10736.1.S1_at_ZNF496
MmugDNA.10737.1.S1_at	225335_at	ZNF496	zinc finger protein 496	chr1q44		84838	LOC709694	similar to zinc finger protein 496	---		709694	MmugDNA.10737.1.S1_at_ZNF496
MmugDNA.10738.1.S1_s_at	214326_x_at	JUND	jun D proto-oncogene	chr19p13.2		3727	LOC720182	similar to jun D proto-oncogene	---		720182	MmugDNA.10738.1.S1_s_at_JUND
MmugDNA.10739.1.S1_at	225349_at	ZNF496	zinc finger protein 496	chr1q44		84838	LOC709694	similar to zinc finger protein 496	---		709694	MmugDNA.10739.1.S1_at_ZNF496
MmugDNA.10740.1.S1_at	239241_at	LOC727869	hypothetical LOC727869	chr11q22.2		727869	LOC704704	hypothetical protein LOC704704	---		704704	MmugDNA.10740.1.S1_at_LOC727869
MmugDNA.10741.1.S1_s_at	212371_at	PPPDE1	PPPDE peptidase domain containing 1	chr1q44		51029	LOC713515	similar to CG7222-PA	---		713515	MmugDNA.10741.1.S1_s_at_PPPDE1
MmugDNA.1074.1.S1_at	216298_at	TRGV5	T cell receptor gamma variable 5	chr7p14		6978	LOC712882	similar to Ig kappa chain V-II region 17S29.1	---		712882	MmugDNA.1074.1.S1_at_TRGV5
MmugDNA.10742.1.S1_at	210950_s_at	FDFT1	farnesyl-diphosphate farnesyltransferase 1	chr8p23.1-p22		2222	FDFT1	farnesyl-diphosphate farnesyltransferase 1	---		696396	MmugDNA.10742.1.S1_at_FDFT1
MmugDNA.10743.1.S1_at	208763_s_at	TSC22D3	TSC22 domain family, member 3	chrXq22.3		1831	TSC22D3	TSC22 domain family, member 3	---		702656	MmugDNA.10743.1.S1_at_TSC22D3
MmugDNA.10744.1.S1_at	NA	NA	NA	NA	NA		NA	NA	NA	NA		MmugDNA.10744.1.S1_at_NA
MmugDNA.10744.1.S1_s_at	NA	NA	NA	NA	NA		NA	NA	NA	NA		MmugDNA.10744.1.S1_s_at_NA
MmugDNA.10745.1.S1_at	231096_at	GDEP	gene differentially expressed in prostate	chr4q21.1		118425	---	---	---	---		MmugDNA.10745.1.S1_at_GDEP
MmugDNA.10746.1.S1_at	242975_s_at	---	---	---	---		---	---	---	---		MmugDNA.10746.1.S1_at_---
MmugDNA.10747.1.S1_at	211472_at	PLXNB2	Plexin B2	chr22q13.33		23654	LOC702350	Similar to CD53 antigen	---		702350	MmugDNA.10747.1.S1_at_PLXNB2
MmugDNA.10748.1.S1_at	1569670_at	LOC100192379	hypothetical LOC100192379	---		100192379	LOC707155	hypothetical protein LOC707155	---		707155	MmugDNA.10748.1.S1_at_LOC100192
MmugDNA.10749.1.S1_at	242040_at	GCNT7	Glucosaminyl (N-acetyl) transferase family member 7	chr20q13.2		140687	BPGM	2,3-bisphosphoglycerate mutase	---		706741	MmugDNA.10749.1.S1_at_GCNT7
MmugDNA.10750.1.S1_at	208637_x_at	ACTN1	actinin, alpha 1	chr14q24.1-q24.2|14q24|14q22-c		87	LOC718007	Similar to Cytochrome c oxidase polypeptide VIII-liver/heart, mitochondrial precursor (Cytochrome c o	---		718007	MmugDNA.10750.1.S1_at_ACTN1
MmugDNA.10750.1.S1_s_at	211160_x_at	ACTN1	actinin, alpha 1	chr14q24.1-q24.2|14q24|14q22-c		87	LOC700044 /// LOC7180	Similar to Protein C14orf111 /// Similar to Cytochrome c oxidase polypeptide VIII-liver/heart, mitochon	---	700044 /// 718007		MmugDNA.10750.1.S1_s_at_ACTN1
MmugDNA.10751.1.S1_at	214277_at	COX11	COX11 homolog, cytochrome c oxidase assembly protein (yeast)	chr17q22		1353	---	---	---	---		MmugDNA.10751.1.S1_at_COX11
MmugDNA.1075.1.S1_at	NA	NA	NA	NA	NA		NA	NA	NA	NA		MmugDNA.1075.1.S1_at_NA
MmugDNA.10752.1.S1_at	213193_x_at	TRBC1	T cell receptor beta constant 1	chr7q34		28639	LOC702904	similar to T-cell receptor beta chain C region	---		702904	MmugDNA.10752.1.S1_at_TRBC1
MmugDNA.10753.1.S1_at	232552_at	DAAM1	Dishevelled associated activator of morphogenesis 1	chr14q23.1		23002	---	---	---	---		MmugDNA.10753.1.S1_at_DAAM1
MmugDNA.10754.1.S1_at	223795_at	TSPAN10	tetraspanin 10	chr17q25.3		83882	LOC714103	similar to tetraspanin 10	---		714103	MmugDNA.10754.1.S1_at_TSPAN10
MmugDNA.10755.1.S1_at	236906_x_at	IPO5	Importin 5	chr13q32.2		3843	---	---	---	---		MmugDNA.10755.1.S1_at_IPO5
MmugDNA.10756.1.S1_at	215560_x_at	MTRF1L	Mitochondrial translational release factor 1-like	chr6q25-q26		54516	---	---	---	---		MmugDNA.10756.1.S1_at_MTRF1L
MmugDNA.10757.1.S1_at	1569810_at	---	---	---	---		---	---	---	---		MmugDNA.10757.1.S1_at_---
MmugDNA.10758.1.S1_at	1557835_at	---	---	---	---		---	---	---	---		MmugDNA.10758.1.S1_at_---
MmugDNA.10759.1.S1_at	NA	NA	NA	NA	NA		NA	NA	NA	NA		MmugDNA.10759.1.S1_at_NA
MmugDNA.10760.1.S1_at	1566527_at	---	---	---	---		---	---	---	---		MmugDNA.10760.1.S1_at_---
MmugDNA.10761.1.S1_at	1569074_at	FLJ37078	hypothetical protein FLJ37078	chr7q11.23		222183	LOC719961	hypothetical protein LOC719961	---		719961	MmugDNA.10761.1.S1_at_FLJ37078
MmugDNA.1076.1.S1_at	221531_at	WDR61	WD repeat domain 61	chr15q25.1		80349	LOC709971	similar to WD repeat domain 61	---		709971	MmugDNA.1076.1.S1_at_WDR61
MmugDNA.10762.1.S1_at	211610_at	KLF6	Kruppel-like factor 6	chr10p15		1316	LOC710784	similar to Kruppel-like factor 6	---		710784	MmugDNA.10762.1.S1_at_KLF6
MmugDNA.10763.1.S1_at	1552964_at	C10orf93	chromosome 10 open reading frame 93	chr10q26.3		255352	C9H10orf93	similar to Protein C10orf93	---		701229	MmugDNA.10763.1.S1_at_C10orf93
MmugDNA.10764.1.S1_at	224289_s_at	FKSG83	FKSG83	chr6p22.1		83954	---	---	---	---		MmugDNA.10764.1.S1_at_FKSG83
MmugDNA.10765.1.S1_at	1561207_at	---	---	---	---		---	---	---	---		MmugDNA.10765.1.S1_at_---
MmugDNA.10766.1.S1_at	237744_at	---	---	---	---		---	---	---	---		MmugDNA.10766.1.S1_at_---
MmugDNA.10767.1.S1_at	1554168_a_at	SH3KBP1	SH3-domain kinase binding protein 1	chrXp22.1-p21.3		30011	SH3KBP1	SH3-domain kinase binding protein 1	---		694764	MmugDNA.10767.1.S1_at_SH3KBP1
MmugDNA.10768.1.S1_at	244173_at	C14orf106	chromosome 14 open reading frame 106	chr14q21.3		55320	---	---	---	---		MmugDNA.10768.1.S1_at_C14orf106
MmugDNA.10769.1.S1_at	1553074_at	ASB11	ankyrin repeat and SOCS box-containing 11	chrXp22.31		140456	LOC712435	similar to ankyrin repeat and SOCS box-containing protein 11 isoform b	---		712435	MmugDNA.10769.1.S1_at_ASB11
MmugDNA.10770.1.S1_at	1561006_at	---	---	---	---		---	---	---	---		MmugDNA.10770.1.S1_at_---
MmugDNA.10771.1.S1_at	227766_at	LIG4	ligase IV, DNA, ATP-dependent	chr13q33-q34		3981	LOC705748	Similar to SH2 domain binding protein 1	---		705748	MmugDNA.10771.1.S1_at_LIG4
MmugDNA.1077.1.S1_at	236570_at	ZNF366	zinc finger protein 366	chr5q13.2|5q13.2		167465	---	---	---	---		MmugDNA.1077.1.S1_at_ZNF366
MmugDNA.10772.1.S1_at	240837_at	FNDC7	fibronectin type III domain containing 7	chr1p13.3		163479	LOC699018	similar to fibronectin type III domain containing 7	---		699018	MmugDNA.10772.1.S1_at_FNDC7
MmugDNA.10773.1.S1_at	223563_at	GNB1L	guanine nucleotide binding protein (G protein), beta polypeptide 1-like	chr22q11.2		54584	---	---	---	---		MmugDNA.10773.1.S1_at_GNB1L
MmugDNA.10774.1.S1_at	243060_at	---	---	---	---		---	---	---	---		MmugDNA.10774.1.S1_at_---
MmugDNA.10775.1.S1_at	223674_s_at	CDC42SE1	CDC42 small effector 1	chr1q21.2		56882	LOC713638	Similar to S100 calcium binding protein A16	---		713638	MmugDNA.10775.1.S1_at_CDC42SE1
MmugDNA.10775.1.S1_s_at	NA	NA	NA	NA	NA		NA	NA	NA	NA		MmugDNA.10775.1.S1_s_at_NA
MmugDNA.10776.1.S1_at	231475_at	TBC1D21	TBC1 domain family, member 21	chr15q24.1		161514	TBC1D21	TBC1 domain family, member 21	---		700124	MmugDNA.10776.1.S1_at_TBC1D21
MmugDNA.10777.1.S1_at	217220_at	---	---	---	---		---	---	---	---		MmugDNA.10777.1.S1_at_---
MmugDNA.10778.1.S1_at	209714_s_at	CDKN3	cyclin-dependent kinase inhibitor 3	chr14q22		1033	CDKN3	cyclin-dependent kinase inhibitor 3	---		694877	MmugDNA.10778.1.S1_at_CDKN3
MmugDNA.10779.1.S1_at	234225_at	---	---	---	---		---	---	---	---		MmugDNA.10779.1.S1_at_---
MmugDNA.10780.1.S1_at	234236_at	AMBRA1	autophagy/beclin-1 regulator 1	chr11p11.2		55626	LOC714434	similar to WD repeat domain 5B	---		714434	MmugDNA.10780.1.S1_at_AMBRA1
MmugDNA.10781.1.S1_at	222735_at	TMEM38B	transmembrane protein 38B	chr9q31.2		55151	LOC715679	similar to transmembrane protein 38B	---		715679	MmugDNA.10781.1.S1_at_TMEM38B
MmugDNA.1078.1.S1_at	1562703_at	LOC157381	hypothetical LOC157381	chr8q24.13		157381	---	---	---	---		MmugDNA.1078.1.S1_at_LOC157381
MmugDNA.10782.1.S1_at	244626_at	---	---	---	---		---	---	---	---		MmugDNA.10782.1.S1_at_---
MmugDNA.10783.1.S1_at	224859_at	CD276	CD276 molecule	chr15q23-q24		80381	CD276	CD276 molecule	---		699877	MmugDNA.10783.1.S1_at_CD276
MmugDNA.10784.1.S1_at	234149_at	---	---	---	---		---	---	---	---		MmugDNA.10784.1.S1_at_---
MmugDNA.10785.1.S1_at	230060_at	CDCA7	cell division cycle associated 7	chr2q31		83879	---	---	---	---		MmugDNA.10785.1.S1_at_CDCA7
MmugDNA.10786.1.S1_at	226287_at	CCDC34	coiled-coil domain containing 34	chr11p14.1		91057	LOC699208	hypothetical protein LOC699208	---		699208	MmugDNA.10786.1.S1_at_CCDC34

MmugDNA.10787.1.S1_at	212707_s_at	LOC100133005 /// LOC	RAS p21 protein activator 4 pseudogene /// similar to HSPC047 protein /// RAS p21 protein activator	chr7p13 /// chr7q22 /// chr7q22.1	100133005 /// 1001347	RASA4	RAS p21 protein activator 4	---	719871	MmugDNA.10787.1.S1_at_LOC100133(
MmugDNA.10788.1.S1_at	234721_s_at	CYP26B1	cytochrome P450, family 26, subfamily B, polypeptide 1	chr2p13.3	56603	LOC704750	cytochrome P450, family 26, subfamily b, polypeptide 1	---	704750	MmugDNA.10788.1.S1_at_CYP26B1
MmugDNA.10789.1.S1_at	1556267_at	C12orf28	chromosome 12 open reading frame 28	chr12q15	196446	LOC717240	similar to CG3328-PA	---	717240	MmugDNA.10789.1.S1_at_C12orf28
MmugDNA.10790.1.S1_at	209511_at	POLR2F	polymerase (RNA) II (DNA directed) polypeptide F	chr22q13.1	5435	POLR2F	polymerase (RNA) II (DNA directed) polypeptide F	---	699566	MmugDNA.10790.1.S1_at_POLR2F
MmugDNA.10791.1.S1_at	1552325_at	CCDC11	coiled-coil domain containing 11	chr18q21.1	220136	CCDC11	similar to Coiled-coil domain-containing protein 11	---	700084	MmugDNA.10791.1.S1_at_CCDC11
MmugDNA.1079.1.S1_at	221137_at	---	---	---	---	---	---	---	---	MmugDNA.1079.1.S1_at_---
MmugDNA.10792.1.S1_at	202533_s_at	DHFR	dihydrofolate reductase	chr5q11.2-q13.2	1719	DHFR /// LOC695198 /// I	dihydrofolate reductase /// similar to dihydrofolate reductase /// similar to Dihydrofolate reductase	---	695198 /// 702446 /// 7112	MmugDNA.10792.1.S1_at_DHFR
MmugDNA.10793.1.S1_at	230961_at	---	---	---	---	---	---	---	---	MmugDNA.10793.1.S1_at_---
MmugDNA.10794.1.S1_at	1561402_at	LOC339894	hypothetical protein LOC339894	chr3q25.31	339894	---	---	---	---	MmugDNA.10794.1.S1_at_LOC339894
MmugDNA.10795.1.S1_at	223328_at	ARMC10	armadillo repeat containing 10	chr7q22.1	83787	LOC695210	similar to SVH protein	---	695210	MmugDNA.10795.1.S1_at_ARMC10
MmugDNA.10796.1.S1_at	215410_at	LOC100132832 /// LOC	postmeiotic segregation increased 2-like 5-like /// PMS2 postmeiotic segregation increased 2 (S. cere	chr7q11-q22 /// chr7q11.23 /// ch	100132832 /// 441259 /	---	---	---	---	MmugDNA.10796.1.S1_at_LOC100132(
MmugDNA.10797.1.S1_at	204277_s_at	TK2	thymidine kinase 2, mitochondrial	chr16q22-q23.1	7084	---	---	---	---	MmugDNA.10797.1.S1_at_TK2
MmugDNA.10798.1.S1_at	1561114_a_at	DEPDC4	DEP domain containing 4	chr12q23.1	120863	---	---	---	---	MmugDNA.10798.1.S1_at_DEPDC4
MmugDNA.10799.1.S1_at	232363_at	---	---	---	---	---	---	---	---	MmugDNA.10799.1.S1_at_---
MmugDNA.10800.1.S1_at	242526_at	---	---	---	---	---	---	---	---	MmugDNA.10800.1.S1_at_---
MmugDNA.10801.1.S1_s_at	224284_x_at	FKSG49	FKSG49	chr5q13.3	400949	---	---	---	---	MmugDNA.10801.1.S1_s_at_FKSG49
MmugDNA.1080.1.S1_at	244464_at	---	---	---	---	---	---	---	---	MmugDNA.1080.1.S1_at_---
MmugDNA.10802.1.S1_at	1557618_at	LOC285768	hypothetical LOC285768	chr6p25.3	285768	---	---	---	---	MmugDNA.10802.1.S1_at_LOC285768
MmugDNA.10803.1.S1_s_at	204361_s_at	SKAP2	src kinase associated phosphoprotein 2	chr7p21-p15	8935	---	---	---	---	MmugDNA.10803.1.S1_s_at_SKAP2
MmugDNA.10804.1.S1_at	241251_at	---	---	---	---	---	---	---	---	MmugDNA.10804.1.S1_at_---
MmugDNA.10805.1.S1_at	NA	NA	NA	NA	NA	NA	NA	NA	NA	MmugDNA.10805.1.S1_at_NA
MmugDNA.10805.1.S1_s_at	243835_at	ZDHHC21	Zinc finger, DHHC-type containing 21	chr9p22.3	340481	---	---	---	---	MmugDNA.10805.1.S1_s_at_ZDHHC21
MmugDNA.10806.1.S1_at	1566038_at	DGCR7	DiGeorge syndrome critical region gene 7	chr22q11	266621	---	---	---	---	MmugDNA.10806.1.S1_at_DGCR7
MmugDNA.10807.1.S1_at	NA	NA	NA	NA	NA	NA	NA	NA	NA	MmugDNA.10807.1.S1_at_NA
MmugDNA.10808.1.S1_at	240519_at	---	---	---	---	---	---	---	---	MmugDNA.10808.1.S1_at_---
MmugDNA.10809.1.S1_at	1552665_at	LOC84989	hypothetical LOC84989	chr10q21.3	84989	---	---	---	---	MmugDNA.10809.1.S1_at_LOC84989
MmugDNA.10810.1.S1_at	231354_at	LOC780529	hypothetical LOC780529	chr13q31	780529	---	---	---	---	MmugDNA.10810.1.S1_at_LOC780529
MmugDNA.10811.1.S1_at	236734_at	SLITRK1	SLIT and NTRK-like family, member 1	chr13q31.1	114798	SLITRK1	SLIT and NTRK-like family, member 1	---	699217	MmugDNA.10811.1.S1_at_SLITRK1
MmugDNA.1081.1.S1_at	202307_s_at	TAP1	transporter 1, ATP-binding cassette, sub-family B (MDR/TAP)	chr6p21.3	6890	LOC717726	similar to transporter 1, ATP-binding cassette, sub-family B	---	717726	MmugDNA.1081.1.S1_at_TAP1
MmugDNA.10812.1.S1_at	202092_s_at	ARL2BP	ADP-ribosylation factor-like 2 binding protein	chr16q13	23568	ARL2BP	ADP-ribosylation factor-like 2 binding protein	---	703999	MmugDNA.10812.1.S1_at_ARL2BP
MmugDNA.10813.1.S1_at	229569_at	---	---	---	---	---	---	---	---	MmugDNA.10813.1.S1_at_---
MmugDNA.10814.1.S1_at	224116_at	---	---	---	---	---	---	---	---	MmugDNA.10814.1.S1_at_---
MmugDNA.10815.1.S1_at	201693_s_at	EGR1	early growth response 1	chr5q31.1	1958	---	---	---	---	MmugDNA.10815.1.S1_at_EGR1
MmugDNA.10816.1.S1_at	227745_at	---	---	---	---	---	---	---	---	MmugDNA.10816.1.S1_at_---
MmugDNA.10817.1.S1_at	236622_at	PIGM	phosphatidylinositol glycan anchor biosynthesis, class M	chr1q23.2	93183	---	---	---	---	MmugDNA.10817.1.S1_at_PIGM
MmugDNA.10818.1.S1_at	242806_at	---	---	---	---	---	---	---	---	MmugDNA.10818.1.S1_at_---
MmugDNA.10819.1.S1_at	236421_at	ANKRD45	ankyrin repeat domain 45	chr1q25.1	339416	ANKRD45	ankyrin repeat domain 45	---	706697	MmugDNA.10819.1.S1_at_ANKRD45
MmugDNA.108.1.S1_at	1564656_at	---	---	---	---	---	---	---	---	MmugDNA.108.1.S1_at_---
MmugDNA.10820.1.S1_at	238914_at	---	---	---	---	---	---	---	---	MmugDNA.10820.1.S1_at_---
MmugDNA.10821.1.S1_at	234059_at	---	---	---	---	---	---	---	---	MmugDNA.10821.1.S1_at_---
MmugDNA.1082.1.S1_at	1563052_at	---	---	---	---	---	---	---	---	MmugDNA.1082.1.S1_at_---
MmugDNA.10822.1.S1_at	241638_at	---	---	---	---	---	---	---	---	MmugDNA.10822.1.S1_at_---
MmugDNA.10823.1.S1_at	1557756_a_at	C14orf145	chromosome 14 open reading frame 145	chr14q31.1	145508	---	---	---	---	MmugDNA.10823.1.S1_at_C14orf145
MmugDNA.10824.1.S1_at	230087_at	PRIMA1	proline rich membrane anchor 1	chr14q32.13	145270	---	---	---	---	MmugDNA.10824.1.S1_at_PRIMA1
MmugDNA.10825.1.S1_at	228426_at	CLEC2D	C-type lectin domain family 2, member D	chr12p13	29121	LOC717228	similar to osteoclast inhibitory lectin isoform 2	---	717228	MmugDNA.10825.1.S1_at_CLEC2D
MmugDNA.10826.1.S1_at	241330_x_at	---	---	---	---	---	---	---	---	MmugDNA.10826.1.S1_at_---
MmugDNA.10827.1.S1_at	235203_at	---	---	---	---	---	---	---	---	MmugDNA.10827.1.S1_at_---
MmugDNA.10828.1.S1_at	233233_at	---	---	---	---	---	---	---	---	MmugDNA.10828.1.S1_at_---
MmugDNA.10829.1.S1_at	216522_at	OR2B6	olfactory receptor, family 2, subfamily B, member 6	chr6p21.3	26212	---	---	---	---	MmugDNA.10829.1.S1_at_OR2B6
MmugDNA.10830.1.S1_at	1570090_at	LOC100129166	hypothetical protein LOC100129166	chr2q37.1	100129166	---	---	---	---	MmugDNA.10830.1.S1_at_LOC100129(
MmugDNA.10831.1.S1_at	201959_s_at	MYCBP2	MYC binding protein 2	chr13q22	23077	---	---	---	---	MmugDNA.10831.1.S1_at_MYCBP2
MmugDNA.1083.1.S1_at	236583_at	---	---	---	---	---	---	---	---	MmugDNA.1083.1.S1_at_---
MmugDNA.10832.1.S1_at	214507_s_at	EXOSC2	exosome component 2	chr9q34	23404	EXOSC2	exosome component 2	---	715960	MmugDNA.10832.1.S1_at_EXOSC2
MmugDNA.10833.1.S1_at	224015_s_at	MRPS25	mitochondrial ribosomal protein S25	chr3p25	64432	MRPS25	mitochondrial ribosomal protein S25	---	703261	MmugDNA.10833.1.S1_at_MRPS25
MmugDNA.10834.1.S1_at	209901_x_at	AIF1	allograft inflammatory factor 1	chr6p21.3	199	AIF1	allograft inflammatory factor 1	---	574115	MmugDNA.10834.1.S1_at_AIF1
MmugDNA.10835.1.S1_at	243146_at	---	---	---	---	---	---	---	---	MmugDNA.10835.1.S1_at_---
MmugDNA.10836.1.S1_at	241554_at	---	---	---	---	---	---	---	---	MmugDNA.10836.1.S1_at_---
MmugDNA.10837.1.S1_at	235763_at	SLC44A5	solute carrier family 44, member 5	chr1p31.1	204962	---	---	---	---	MmugDNA.10837.1.S1_at_SLC44A5
MmugDNA.10838.1.S1_at	237128_at	---	---	---	---	---	---	---	---	MmugDNA.10838.1.S1_at_---
MmugDNA.10839.1.S1_at	210355_at	PTHLH	parathyroid hormone-like hormone	chr12p12.1-p11.2	5744	PTHLH	parathyroid hormone-like hormone	---	710295	MmugDNA.10839.1.S1_at_PTHLH
MmugDNA.10840.1.S1_at	1555471_a_at	FMN2	formin 2	chr1q43	56776	FMN1	similar to formin 2	---	708376	MmugDNA.10840.1.S1_at_FMN2
MmugDNA.10841.1.S1_at	237229_at	JMJD5	Jumonji domain containing 5	chr16p12.1	79831	---	---	---	---	MmugDNA.10841.1.S1_at_JMJD5
MmugDNA.1084.1.S1_at	222265_at	TNS4	tensin 4	chr17q21.2	84951	TNS4	tensin 4	---	701075	MmugDNA.1084.1.S1_at_TNS4
MmugDNA.10842.1.S1_at	228866_at	---	---	---	---	---	---	---	---	MmugDNA.10842.1.S1_at_---
MmugDNA.10843.1.S1_at	242024_at	---	---	---	---	---	---	---	---	MmugDNA.10843.1.S1_at_---
MmugDNA.10844.1.S1_at	225047_at	NUPL1	nucleoporin like 1	chr13q12.13	9818	LOC709215	similar to nucleoporin like 1 isoform a	---	709215	MmugDNA.10844.1.S1_at_NUPL1
MmugDNA.10845.1.S1_at	209768_s_at	GP1BB /// SEPT5	glycoprotein Ib (platelet), beta polypeptide /// septin 5	chr22q11.21 /// chr22q11.21-q11	2812 /// 5413	---	---	---	---	MmugDNA.10845.1.S1_at_GP1BB /// SI
MmugDNA.10846.1.S1_at	232196_at	LCA5L	Leber congenital amaurosis 5-like	chr21q22.2	150082	LOC715569	similar to CG6652-PB, isoform B	---	715569	MmugDNA.10846.1.S1_at_LCA5L
MmugDNA.10847.1.S1_at	1566178_x_at	---	---	---	---	---	---	---	---	MmugDNA.10847.1.S1_at_---
MmugDNA.10848.1.S1_at	240047_at	---	---	---	---	---	---	---	---	MmugDNA.10848.1.S1_at_---
MmugDNA.10849.1.S1_at	237166_at	---	---	---	---	---	---	---	---	MmugDNA.10849.1.S1_at_---
MmugDNA.10850.1.S1_at	209769_s_at	GP1BB	glycoprotein Ib (platelet), beta polypeptide	chr22q11.21-q11.23|22q11.21	2812	---	---	---	---	MmugDNA.10850.1.S1_at_GP1BB
MmugDNA.10851.1.S1_at	1561208_at	---	---	---	---	---	---	---	---	MmugDNA.10851.1.S1_at_---
MmugDNA.1085.1.S1_at	1555799_at	FCRL5	Fc receptor-like 5	chr1q21	83416	---	---	---	---	MmugDNA.1085.1.S1_at_FCRL5
MmugDNA.10852.1.S1_at	241046_at	---	---	---	---	---	---	---	---	MmugDNA.10852.1.S1_at_---
MmugDNA.10853.1.S1_at	202721_s_at	GFPT1	glutamine-fructose-6-phosphate transaminase 1	chr2p13	2673	---	---	---	---	MmugDNA.10853.1.S1_at_GFPT1
MmugDNA.10854.1.S1_at	1561166_a_at	---	---	---	---	---	---	---	---	MmugDNA.10854.1.S1_at_---
MmugDNA.10855.1.S1_at	216952_s_at	LMNB2	lamin B2	chr19p13.3	84823	---	---	---	---	MmugDNA.10855.1.S1_at_LMNB2
MmugDNA.10856.1.S1_at	241478_at	MICALL2	MICAL-like 2	chr7p22.3	79778	---	---	---	---	MmugDNA.10856.1.S1_at_MICALL2
MmugDNA.10857.1.S1_at	229539_at	ABCB8	ATP-binding cassette, sub-family B (MDR/TAP), member 8	chr7q36	11194	---	---	---	---	MmugDNA.10857.1.S1_at_ABCB8
MmugDNA.10858.1.S1_at	NA	NA	NA	NA	NA	NA	NA	NA	NA	MmugDNA.10858.1.S1_at_NA
MmugDNA.10859.1.S1_at	243865_x_at	---	---	---	---	---	---	---	---	MmugDNA.10859.1.S1_at_---
MmugDNA.10860.1.S1_s_at	209980_s_at	SHMT1	serine hydroxymethyltransferase 1 (soluble)	chr17p11.2	6470	SHMT1	serine hydroxymethyltransferase 1 (soluble)	---	701186	MmugDNA.10860.1.S1_s_at_SHMT1
MmugDNA.10861.1.S1_at	1557761_s_at	LOC400794	hypothetical LOC400794	chr1q23.2	400794	---	---	---	---	MmugDNA.10861.1.S1_at_LOC400794
MmugDNA.1086.1.S1_at	234423_x_at	---	---	---	---	---	---	---	---	MmugDNA.1086.1.S1_at_---
MmugDNA.10862.1.S1_at	1570318_at	---	---	---	---	---	---	---	---	MmugDNA.10862.1.S1_at_---
MmugDNA.10863.1.S1_at	229726_at	GRAP	GRB2-related adaptor protein	chr17p11.2	10750	GRAP	GRB2-related adaptor protein	---	702473	MmugDNA.10863.1.S1_at_GRAP
MmugDNA.10864.1.S1_at	1552295_a_at	SLC39A13	solute carrier family 39 (zinc transporter), member 13	chr11p11.2	91252	LOC713420	similar to solute carrier family 39 (zinc transporter), member 13	---	713420	MmugDNA.10864.1.S1_at_SLC39A13
MmugDNA.10864.1.S1_s_at	NA	NA	NA	NA	NA	NA	NA	NA	NA	MmugDNA.10864.1.S1_s_at_NA
MmugDNA.10865.1.S1_at	214578_s_at	ROCK1	Rho-associated, coiled-coil containing protein kinase 1	chr18q11.1	6093	ROCK1	Rho-associated, coiled-coil containing protein kinase 1	---	698223	MmugDNA.10865.1.S1_at_ROCK1
MmugDNA.10866.1.S1_at	200776_s_at	BZW1 /// BZW1L1	basic leucine zipper and W2 domains 1 /// basic leucine zipper and W2 domains 1 like 1	chr2q33 /// chr3q13.31	151579 /// 9689	LOC694783	similar to basic leucine zipper and W2 domains 1	---	694783	MmugDNA.10866.1.S1_at_BZW1 /// BZ
MmugDNA.10867.1.S1_at	1556529_a_at	LOC285326	hypothetical protein LOC285326	chr3p24.2	285326	---	---	---	---	MmugDNA.10867.1.S1_at_LOC285326
MmugDNA.10868.1.S1_at	1565559_at	MAGIX	MAGI family member, X-linked	chrXp11.23	79917	---	---	---	---	MmugDNA.10868.1.S1_at_MAGIX
MmugDNA.10869.1.S1_at	229431_at	RFXAP	regulatory factor X-associated protein	chr13q14	5994	RFXAP	regulatory factor X-associated protein	---	694441	MmugDNA.10869.1.S1_at_RFXAP
MmugDNA.10870.1.S1_at	1553023_a_at	NOX5	NADPH oxidase, EF-hand calcium binding domain 5	chr15q23	79400	NOX5	NADPH oxidase, EF-hand calcium binding domain 5	---	694875	MmugDNA.10870.1.S1_at_NOX5
MmugDNA.10871.1.S1_at	1559148_at	---	---	---	---	---	---	---	---	MmugDNA.10871.1.S1_at_---
MmugDNA.1087.1.S1_at	228505_s_at	TMEM170A	transmembrane protein 170A	chr16q23.1	124491	---	---	---	---	MmugDNA.1087.1.S1_at_TMEM170A
MmugDNA.10872.1.S1_at	213551_x_at	PCGF2	polycomb group ring finger 2	chr17q12	7703	PCGF2	polycomb group ring finger 2	---	694187	MmugDNA.10872.1.S1_at_PCGF2
MmugDNA.10873.1.S1_at	237223_at	---	---	---	---	---	---	---	---	MmugDNA.10873.1.S1_at_---
MmugDNA.10874.1.S1_s_at	231982_at	LOC284422	similar to HSPC323	chr19p13.3	284422	LOC721706	hypothetical protein LOC721706	---	721706	MmugDNA.10874.1.S1_s_at_LOC28442
MmugDNA.10875.1.S1_at	204772_s_at	TTF1	transcription termination factor, RNA polymerase I	chr9q34.13	7270	LOC715002	similar to transcription termination factor, RNA polymerase I	---	715002	MmugDNA.10875.1.S1_at_TTF1
MmugDNA.10876.1.S1_at	218891_at	C10orf76	chromosome 10 open reading frame 76	chr10q24.32	79591	LOC712512	similar to CG8379-PA, isoform A	---	712512	MmugDNA.10876.1.S1_at_C10orf76
MmugDNA.10877.1.S1_at	1559705_s_at	PHKA2	phosphorylase kinase, alpha 2 (liver)	chrXp22.2-p22.1	5256	PHKA2	phosphorylase kinase, alpha 2 (liver)	---	694376	MmugDNA.10877.1.S1_at_PHKA2
MmugDNA.10878.1.S1_at	227029_at	FAM177A1	family with sequence similarity 177, member A1	chr14q13.2	283635	LOC694483	hypothetical protein LOC694483	---	694483	MmugDNA.10878.1.S1_at_FAM177A1
MmugDNA.10879.1.S1_at	214292_at	ITGB4	integrin, beta 4	chr17q25	3691	LOC703770	similar to integrin beta 4 isoform 1 precursor	---	703770	MmugDNA.10879.1.S1_at_ITGB4
MmugDNA.10880.1.S1_at	218246_at	MUL1	mitochondrial E3 ubiquitin ligase 1	chr1p36.12	79594	MUL1	mitochondrial E3 ubiquitin ligase 1	---	705429	MmugDNA.10880.1.S1_at_MUL1
MmugDNA.10881.1.S1_at	210561_s_at	WSB1	WD repeat and SOCS box-containing 1	chr17q11.1	26118	WSB1	WD repeat and SOCS box-containing 1	---	706172	MmugDNA.10881.1.S1_at_WSB1
MmugDNA.1088.1.S1_at	240857_at	DNAH9	dynein, axonemal, heavy chain 9	chr17p12	1770	---	---	---	---	MmugDNA.1088.1.S1_at_DNAH9

MmugDNA.10882.1.S1_at	216733_s_at	GATM	glycine amidinotransferase (L-arginine:glycine amidinotransferase)	chr15q21.1	2628	GATM	glycine amidinotransferase (L-arginine:glycine amidinotransferase)	---	713306	MmugDNA.10882.1.S1_at_GATM
MmugDNA.10883.1.S1_at	220367_s_at	SAP130	Sin3A-associated protein, 130kDa	chr2q14.3	79595	LOC715151	similar to mSin3A-associated protein 130	---	715151	MmugDNA.10883.1.S1_at_SAP130
MmugDNA.10884.1.S1_at	219303_at	RNF219	ring finger protein 219	chr13q31.1	79596	RNF219	ring finger protein 219	---	697577	MmugDNA.10884.1.S1_at_RNF219
MmugDNA.10885.1.S1_at	NA	NA	NA	NA	NA	NA	NA	NA	NA	MmugDNA.10885.1.S1_at_NA
MmugDNA.10886.1.S1_at	235558_at	RBMS2	RNA binding motif, single stranded interacting protein 2	chr12q13.3	5939	LOC713163	similar to RNA binding motif, single stranded interacting protein 2	---	713163	MmugDNA.10886.1.S1_at_RBMS2
MmugDNA.10887.1.S1_at	1563247_at	---	---	---	---	---	---	---	---	MmugDNA.10887.1.S1_at_---
MmugDNA.10888.1.S1_at	243172_at	---	---	---	---	---	---	---	---	MmugDNA.10888.1.S1_at_---
MmugDNA.10889.1.S1_at	NA	NA	NA	NA	NA	NA	NA	NA	NA	MmugDNA.10889.1.S1_at_NA
MmugDNA.10890.1.S1_at	217187_at	MUC5AC	mucin 5AC, oligomeric mucus/gel-forming	chr11p15.5	4586	---	---	---	---	MmugDNA.10890.1.S1_at_MUC5AC
MmugDNA.10891.1.S1_at	1562671_s_at	---	---	---	---	---	---	---	---	MmugDNA.10891.1.S1_at_---
MmugDNA.1089.1.S1_at	202316_x_at	UBE4B	ubiquitination factor E4B (UFD2 homolog, yeast)	chr1p36.3	10277	---	---	---	---	MmugDNA.1089.1.S1_at_UBE4B
MmugDNA.1089.1.S1_s_at	215533_s_at	UBE4B	ubiquitination factor E4B (UFD2 homolog, yeast)	chr1p36.3	10277	---	---	---	---	MmugDNA.1089.1.S1_s_at_UBE4B
MmugDNA.10892.1.S1_at	216682_s_at	FAM48A	Family with sequence similarity 48, member A	chr13q13.3	55578	---	---	---	---	MmugDNA.10892.1.S1_at_FAM48A
MmugDNA.10893.1.S1_at	238823_at	FMNL3	formin-like 3	chr12q13.12	91010	---	---	---	---	MmugDNA.10893.1.S1_at_FMNL3
MmugDNA.10894.1.S1_at	1558237_x_at	---	---	---	---	---	---	---	---	MmugDNA.10894.1.S1_at_---
MmugDNA.10895.1.S1_at	234641_at	---	---	---	---	---	---	---	---	MmugDNA.10895.1.S1_at_---
MmugDNA.10896.1.S1_at	NA	NA	NA	NA	NA	NA	NA	NA	NA	MmugDNA.10896.1.S1_at_NA
MmugDNA.10897.1.S1_at	222133_s_at	PHF20L1	PHD finger protein 20-like 1	chr8q24.22	51105	PHF20L1	PHD finger protein 20-like 1	---	701406	MmugDNA.10897.1.S1_at_PHF20L1
MmugDNA.10897.1.S1_s_at	227523_s_at	PHF20L1	PHD finger protein 20-like 1	chr8q24.22	51105	PHF20L1	PHD finger protein 20-like 1	---	701406	MmugDNA.10897.1.S1_s_at_PHF20L1
MmugDNA.10898.1.S1_at	231926_at	EPS15L1	epidermal growth factor receptor pathway substrate 15-like 1	chr19p13.11	58513	---	---	---	---	MmugDNA.10898.1.S1_at_EPS15L1
MmugDNA.10899.1.S1_at	NA	NA	NA	NA	NA	NA	NA	NA	NA	MmugDNA.10899.1.S1_at_NA
MmugDNA.10899.1.S1_s_at	204672_s_at	ANKRD6	ankyrin repeat domain 6	chr6q14.2-q16.1	22881	ANKRD6	ankyrin repeat domain 6	---	701339	MmugDNA.10899.1.S1_s_at_ANKRD6
MmugDNA.10900.1.S1_at	207090_x_at	ZFP30	zinc finger protein 30 homolog (mouse)	chr19q13.12	22835	---	---	---	---	MmugDNA.10900.1.S1_at_ZFP30
MmugDNA.10901.1.S1_at	1560821_at	ARHGAP22	Rho GTPase activating protein 22	chr10q11.22	58504	---	---	---	---	MmugDNA.10901.1.S1_at_ARHGAP22
MmugDNA.1090.1.S1_at	226484_at	ZBTB47	zinc finger and BTB domain containing 47	chr3p22.1	92999	LOC717392	similar to natural killer-tumor recognition sequence isoform a	---	717392	MmugDNA.1090.1.S1_at_ZBTB47
MmugDNA.10902.1.S1_at	219886_at	CEP97	centrosomal protein 97kDa	chr3q12.3	79598	CEP97	centrosomal protein 97kDa	---	702482	MmugDNA.10902.1.S1_at_CEP97
MmugDNA.10903.1.S1_at	1569039_s_at	ZNF677	zinc finger protein 677	chr19q13.41	342926	LOC720163	similar to zinc finger protein 677	---	720163	MmugDNA.10903.1.S1_at_ZNF677
MmugDNA.10904.1.S1_at	1570274_at	UNQ9391	tryptophan/serine protease	chr8p23.1	203074	LOC698379	similar to Plasma kallikrein precursor (Plasma prekallikrein) (Kininogenin) (Fletcher factor)	---	698379	MmugDNA.10904.1.S1_at_UNQ9391
MmugDNA.10905.1.S1_at	218584_at	TCTN1	tectonic family member 1	chr12q24.11	79600	---	---	---	---	MmugDNA.10905.1.S1_at_TCTN1
MmugDNA.10906.1.S1_at	204166_at	SBNO2	strawberry notch homolog 2 (Drosophila)	chr19p13.3	22904	---	---	---	---	MmugDNA.10906.1.S1_at_SBNO2
MmugDNA.10907.1.S1_at	201346_at	ADIPOR2	adiponectin receptor 2	chr12p13.31	79602	LOC707547	similar to adiponectin receptor 2	---	707547	MmugDNA.10907.1.S1_at_ADIPOR2
MmugDNA.10908.1.S1_at	226631_at	METTL10	methyltransferase like 10	chr10q26.13	399818	LOC697177	similar to CG9643-PA	---	697177	MmugDNA.10908.1.S1_at_METTL10
MmugDNA.10909.1.S1_at	226634_at	METTL10	methyltransferase like 10	chr10q26.13	399818	---	---	---	---	MmugDNA.10909.1.S1_at_METTL10
MmugDNA.10910.1.S1_at	1553024_at	G30	protein LG30-like	chr13q33.2|13q34	282706	---	---	---	---	MmugDNA.10910.1.S1_at_G30
MmugDNA.10911.1.S1_at	219225_at	LOC100134440 /// PGB	similar to PGBD5 protein /// piggyBac transposable element derived 5	chr1q42.13	100134440 /// 79605	---	---	---	---	MmugDNA.10911.1.S1_at_LOC100134
MmugDNA.1091.1.S1_at	226500_at	ZBTB47	zinc finger and BTB domain containing 47	chr3p22.1	92999	LOC717392	similar to natural killer-tumor recognition sequence isoform a	---	717392	MmugDNA.1091.1.S1_at_ZBTB47
MmugDNA.10912.1.S1_at	226213_at	ERBB3	v-erb-b2 erythroblastic leukemia viral oncogene homolog 3 (avian)	chr12q13	2065	ERBB3	v-erb-b2 erythroblastic leukemia viral oncogene homolog 3 (avian)	---	711407	MmugDNA.10912.1.S1_at_ERBB3
MmugDNA.10913.1.S1_at	243699_at	---	---	---	---	---	---	---	---	MmugDNA.10913.1.S1_at_---
MmugDNA.10914.1.S1_at	1554871_at	---	---	---	---	---	---	---	---	MmugDNA.10914.1.S1_at_---
MmugDNA.10915.1.S1_at	1563827_at	LOC158434	hypothetical protein LOC158434	chr9q22.33	158434	---	---	---	---	MmugDNA.10915.1.S1_at_LOC158434
MmugDNA.10916.1.S1_at	244143_at	---	---	---	---	---	---	---	---	MmugDNA.10916.1.S1_at_---
MmugDNA.10917.1.S1_at	230924_at	TTLL6	tubulin tyrosine ligase-like family, member 6	chr17q21.32	284076	---	---	---	---	MmugDNA.10917.1.S1_at_TTLL6
MmugDNA.10918.1.S1_at	243779_at	GALNT13	UDP-N-acetyl-alpha-D-galactosamine:polypeptide N-acetylgalactosaminyltransferase 13 (GalNAc-T1	chr2q23.3-q24.1	114805	LOC696337	similar to UDP-N-acetyl-alpha-D-galactosamine:polypeptide N-acetylgalactosaminyltransferase 13	---	696337	MmugDNA.10918.1.S1_at_GALNT13
MmugDNA.10919.1.S1_at	231250_at	---	---	---	---	LOC698777	hypothetical protein LOC698777	---	698777	MmugDNA.10919.1.S1_at_---
MmugDNA.109.1.S1_at	222329_x_at	---	---	---	---	---	---	---	---	MmugDNA.109.1.S1_at_---
MmugDNA.10920.1.S1_at	235599_at	LOC339535	hypothetical LOC339535	chr1q43	339535	---	---	---	---	MmugDNA.10920.1.S1_at_LOC339535
MmugDNA.10921.1.S1_at	202570_s_at	DLGAP4	discs, large (Drosophila) homolog-associated protein 4	chr20q11.23	22839	DLGAP4	discs, large (Drosophila) homolog-associated protein 4	---	702928	MmugDNA.10921.1.S1_at_DLGAP4
MmugDNA.1092.1.S1_at	210050_at	TPI1	triosephosphate isomerase 1	chr12p13	7167	TPI1	triosephosphate isomerase 1	---	714090	MmugDNA.1092.1.S1_at_TPI1
MmugDNA.10922.1.S1_at	NA	NA	NA	NA	NA	NA	NA	NA	NA	MmugDNA.10922.1.S1_at_NA
MmugDNA.10923.1.S1_at	204627_s_at	ITGB3	integrin, beta 3 (platelet glycoprotein IIIa, antigen CD61)	chr17q21.32	3690	LOC717379	similar to integrin beta chain, beta 3 precursor	---	717379	MmugDNA.10923.1.S1_at_ITGB3
MmugDNA.10924.1.S1_at	205093_at	PLEKHA6	pleckstrin homology domain containing, family A member 6	chr1q32.1	22874	---	---	---	---	MmugDNA.10924.1.S1_at_PLEKHA6
MmugDNA.10925.1.S1_at	220282_at	RIC3	resistance to inhibitors of cholinesterase 3 homolog (C. elegans)	chr11p15.4	79608	LMO1	LIM domain only 1 (rhombotin 1)	---	706162	MmugDNA.10925.1.S1_at_RIC3
MmugDNA.10926.1.S1_at	202304_at	FNDC3A	fibronectin type III domain containing 3A	chr13q14.2	22862	LOC705570	similar to Fibronectin type-III domain-containing protein 3a	---	705570	MmugDNA.10926.1.S1_at_FNDC3A
MmugDNA.10927.1.S1_at	1555731_a_at	AP1S3	adaptor-related protein complex 1, sigma 3 subunit	chr2q36.1	130340	LOC710257	similar to adaptor-related protein complex AP-1, sigma 3	---	710257	MmugDNA.10927.1.S1_at_AP1S3
MmugDNA.10928.1.S1_at	218940_at	C14orf138	chromosome 14 open reading frame 138	chr14q22.1	79609	LOC713211	Similar to eukaryotic translation initiation factor 3, subunit 10 theta, 150/170kDa	---	713211	MmugDNA.10928.1.S1_at_C14orf138
MmugDNA.10929.1.S1_at	219616_at	ACSS3	acyl-CoA synthetase short-chain family member 3	chr12q21.31	79611	---	---	---	---	MmugDNA.10929.1.S1_at_ACSS3
MmugDNA.10930.1.S1_s_at	213756_s_at	HSF1	heat shock transcription factor 1	chr8q24.3	3297	HSF1	heat shock transcription factor 1	---	714078	MmugDNA.10930.1.S1_s_at_HSF1
MmugDNA.10931.1.S1_at	219378_at	NARG1L	NMDA receptor regulated 1-like	chr13q14.11	79612	NARG1L	NMDA receptor regulated 1-like	---	698843	MmugDNA.10931.1.S1_at_NARG1L
MmugDNA.1093.1.S1_at	218203_at	ALG5	asparagine-linked glycosylation 5, dolichyl-phosphate beta-glucosyltransferase homolog (S. cerevisia	chr13q13.3	29880	ALG5	asparagine-linked glycosylation 5	---	694836	MmugDNA.1093.1.S1_at_ALG5
MmugDNA.10932.1.S1_at	243695_at	---	---	---	---	---	---	---	---	MmugDNA.10932.1.S1_at_---
MmugDNA.10933.1.S1_at	231844_at	MGC27345	hypothetical protein MGC27345	chr7q32.1	157247	---	---	---	---	MmugDNA.10933.1.S1_at_MGC27345
MmugDNA.10934.1.S1_at	222168_at	---	---	---	---	---	---	---	---	MmugDNA.10934.1.S1_at_---
MmugDNA.10935.1.S1_at	231087_at	---	---	---	---	---	---	---	---	MmugDNA.10935.1.S1_at_---
MmugDNA.10936.1.S1_at	237647_at	GHRL	Ghrelin/obestatin prepropeptide	chr3p26-p25	51738	---	---	---	---	MmugDNA.10936.1.S1_at_GHRL
MmugDNA.10937.1.S1_at	1552957_at	DNAH6	dynein, axonemal, heavy chain 6	chr2p11.2	1768	LOC694115	similar to Dynein heavy chain at 16F CG7092-PA	---	694115	MmugDNA.10937.1.S1_at_DNAH6
MmugDNA.10938.1.S1_at	1563612_at	---	---	---	---	---	---	---	---	MmugDNA.10938.1.S1_at_---
MmugDNA.10939.1.S1_at	215319_at	ATP8B3	ATPase, class I, type 8B, member 3	chr19p13.3	148229	ATP8B3	ATPase, Class I, type 8B, member 3	---	721348	MmugDNA.10939.1.S1_at_ATP8B3
MmugDNA.10940.1.S1_at	237472_at	SOX1	SRY (sex determining region Y)-box 1	chr13q34	6656	---	---	---	---	MmugDNA.10940.1.S1_at_SOX1
MmugDNA.10941.1.S1_at	205976_at	FASTKD2	FAST kinase domains 2	chr2q33.3	22868	LOC709702	hypothetical protein LOC709702	---	709702	MmugDNA.10941.1.S1_at_FASTKD2
MmugDNA.1094.1.S1_at	221114_at	AMBN	ameloblastin (enamel matrix protein)	chr4q21	258	AMBN	ameloblastin	---	706845	MmugDNA.1094.1.S1_at_AMBN
MmugDNA.10942.1.S1_at	210038_at	PRKCQ	protein kinase C, theta	chr10p15	5588	---	---	---	---	MmugDNA.10942.1.S1_at_PRKCQ
MmugDNA.10943.1.S1_at	206053_at	ZNF510	zinc finger protein 510	chr9q22.33	22869	---	---	---	---	MmugDNA.10943.1.S1_at_ZNF510
MmugDNA.10944.1.S1_at	214855_s_at	GARNL1	GTPase activating Rap/RanGAP domain-like 1	chr14q13.2	253959	GARNL1	GTPase activating Rap/RanGAP domain-like 1	---	695674	MmugDNA.10944.1.S1_at_GARNL1
MmugDNA.10946.1.S1_at	235205_at	LOC100128259 /// LOC	hypothetical protein LOC100128259 /// similar to solute carrier family 16 (monocarboxylic acid transp	chr8q23.1	100128259 /// 346887	---	---	---	---	MmugDNA.10946.1.S1_at_LOC100128
MmugDNA.10947.1.S1_at	206713_at	NTNG1	netrin G1	chr1p13.3	22854	NTNG2	netrin G2	---	694410	MmugDNA.10947.1.S1_at_NTNG1
MmugDNA.10948.1.S1_at	235447_at	TRUB1	TruB pseudouridine (psi) synthase homolog 1 (E. coli)	chr10q25.3	142940	---	---	---	---	MmugDNA.10948.1.S1_at_TRUB1
MmugDNA.10949.1.S1_at	219054_at	C5orf23	chromosome 5 open reading frame 23	chr5p13.3	79614	---	---	---	---	MmugDNA.10949.1.S1_at_C5orf23
MmugDNA.10950.1.S1_at	203642_s_at	COBLL1	COBL-like 1	chr2q24.3	22837	COBLL1	COBL-like 1	---	702934	MmugDNA.10950.1.S1_at_COBLL1
MmugDNA.10951.1.S1_at	1566482_at	---	---	---	---	---	---	---	---	MmugDNA.10951.1.S1_at_---
MmugDNA.1095.1.S1_at	238761_at	---	---	---	---	---	---	---	---	MmugDNA.1095.1.S1_at_---
MmugDNA.10952.1.S1_at	220296_at	GALNT10	UDP-N-acetyl-alpha-D-galactosamine:polypeptide N-acetylgalactosaminyltransferase 10 (GalNAc-T1	chr5q33.2	55568	---	---	---	---	MmugDNA.10952.1.S1_at_GALNT10
MmugDNA.10953.1.S1_at	219227_at	CCNJL	cyclin J-like	chr5q33.3	79616	CCNJL	similar to cyclin J	---	695560	MmugDNA.10953.1.S1_at_CCNJL
MmugDNA.10954.1.S1_at	1554721_a_at	TAF2	TAF2 RNA polymerase II, TATA box binding protein (TBP)-associated factor, 150kDa	chr8q24.12	6873	TAF2	TAF2 RNA polymerase II, TATA box binding protein (TBP)-associated factor, 150kDa	---	702411	MmugDNA.10954.1.S1_at_TAF2
MmugDNA.10955.1.S1_at	219269_at	HMBOX1	homeobox containing 1	chr8p21.1	79618	---	---	---	---	MmugDNA.10955.1.S1_at_HMBOX1
MmugDNA.10956.1.S1_at	226545_at	CD109	CD109 molecule	chr6q13	135228	---	---	---	---	MmugDNA.10956.1.S1_at_CD109
MmugDNA.10958.1.S1_at	1558249_s_at	STX16	syntaxin 16	chr20q13.32	8675	STX16	syntaxin 16	---	694811	MmugDNA.10958.1.S1_at_STX16
MmugDNA.10959.1.S1_at	1553129_at	SVEP1	sushi, von Willebrand factor type A, EGF and pentraxin domain containing 1	chr9q32	79987	---	---	---	---	MmugDNA.10959.1.S1_at_SVEP1
MmugDNA.10960.1.S1_at	1568913_at	NSUN3	NOL1/NOP2/Sun domain family, member 3	chr3q11.2	63899	---	---	---	---	MmugDNA.10960.1.S1_at_NSUN3
MmugDNA.10961.1.S1_at	1562510_at	LOC339442	hypothetical protein LOC339442	chr1p34.3	339442	---	---	---	---	MmugDNA.10961.1.S1_at_LOC339442
MmugDNA.1096.1.S1_at	202180_s_at	MVP	major vault protein	chr16p13.1-p11.2	9961	LOC695727	Similar to filamin-binding LIM protein-1 isoform a	---	695727	MmugDNA.1096.1.S1_at_MVP
MmugDNA.10962.1.S1_at	229291_at	---	---	---	---	---	---	---	---	MmugDNA.10962.1.S1_at_---
MmugDNA.10963.1.S1_at	1555636_at	CD300LG	CD300 molecule-like family member g	chr17q21.31	146894	---	---	---	---	MmugDNA.10963.1.S1_at_CD300LG
MmugDNA.10964.1.S1_at	243391_x_at	---	---	---	---	---	---	---	---	MmugDNA.10964.1.S1_at_---
MmugDNA.10965.1.S1_at	1555400_at	LOC645261	PP565	chr5q14.3	645261	---	---	---	---	MmugDNA.10965.1.S1_at_LOC645261
MmugDNA.10966.1.S1_at	236609_at	LOC100129592	Hypothetical protein LOC100129592	chr2q32.2	100129592	LOC709937	hypothetical protein LOC709937	---	709937	MmugDNA.10966.1.S1_at_LOC100129
MmugDNA.10967.1.S1_at	215345_x_at	---	---	---	---	LOC712811	similar to Ig kappa chain V-II region 17S29.1	---	712811	MmugDNA.10967.1.S1_at_---
MmugDNA.10968.1.S1_at	211905_s_at	ITGB4	integrin, beta 4	chr17q25	3691	LOC703770	similar to integrin beta 4 isoform 1 precursor	---	703770	MmugDNA.10968.1.S1_at_ITGB4
MmugDNA.10969.1.S1_at	1552919_at	C4orf36	chromosome 4 open reading frame 36	chr4q21.3	132989	LOC700500	hypothetical protein LOC700500	---	700500	MmugDNA.10969.1.S1_at_C4orf36
MmugDNA.10970.1.S1_at	227792_at	ITPRIPL2	inositol 1,4,5-triphosphate receptor interacting protein-like 2	chr16p12.3	162073	---	---	---	---	MmugDNA.10970.1.S1_at_ITPRIPL2
MmugDNA.10971.1.S1_at	228006_at	---	---	---	---	---	---	---	---	MmugDNA.10971.1.S1_at_---
MmugDNA.1097.1.S1_at	240478_at	---	---	---	---	---	---	---	---	MmugDNA.1097.1.S1_at_---
MmugDNA.10972.1.S1_at	231765_at	ZFYVE20	zinc finger, FYVE domain containing 20	chr3p24.3	64145	LOC702356	similar to FYVE-finger-containing Rab5 effector protein rabenosyn-5	---	702356	MmugDNA.10972.1.S1_at_ZFYVE20
MmugDNA.10973.1.S1_at	214757_at	PMS2L2	postmeiotic segregation increased 2-like 2 pseudogene	chr7q11-q22	5380	---	---	---	---	MmugDNA.10973.1.S1_at_PMS2L2
MmugDNA.10974.1.S1_at	216260_at	DICER1	dicer 1, ribonuclease type III	chr14q32.13	23405	DICER1	dicer 1, ribonuclease type III	---	702516	MmugDNA.10974.1.S1_at_DICER1
MmugDNA.10975.1.S1_at	205230_at	RPH3A	rabphilin 3A homolog (mouse)	chr12q24.13	22895	LOC714096	Similar to Heterogeneous nuclear ribonucleoprotein A1 (Helix-destabilizing protein) (Single-strand RN	---	714096	MmugDNA.10975.1.S1_at_RPH3A
MmugDNA.10976.1.S1_at	203044_at	CHSY1	chondroitin sulfate synthase 1	chr15q26.3	22856	---	---	---	---	MmugDNA.10976.1.S1_at_CHSY1
MmugDNA.10977.1.S1_at	204557_s_at	DZIP1	DAZ interacting protein 1	chr13q32.1	22873	DZIP1	DAZ interacting protein 1	---	695529	MmugDNA.10977.1.S1_at_DZIP1
MmugDNA.10978.1.S1_at	1569885_at	---	---	---	---	---	---	---	---	MmugDNA.10978.1.S1_at_---

MmugDNA.10979.1.S1_at	205092_x_at	ZBTB1	zinc finger and BTB domain containing 1	chr14q23.3	22890	---	---	---	---		MmugDNA.10979.1.S1_at_ZBTB1
MmugDNA.10980.1.S1_at	219056_at	RNASEH2B	ribonuclease H2, subunit B	chr13q14.3	79621	LOC707995	similar to CG11164-PA	---		707995	MmugDNA.10980.1.S1_at_RNASEH2B
MmugDNA.10981.1.S1_at	218195_at	C6orf211	chromosome 6 open reading frame 211	chr6q25.1	79624	LOC697395	similar to F31D4.2	---		697395	MmugDNA.10981.1.S1_at_C6orf211
MmugDNA.1098.1.S1_at	1553042_a_at	NFKBID	nuclear factor of kappa light polypeptide gene enhancer in B-cells inhibitor, delta	chr19q13.12	84807	LOC711338	similar to T-cell activation NFKB-like protein	---		711338	MmugDNA.1098.1.S1_at_NFKBID
MmugDNA.10982.1.S1_at	219747_at	C4orf31	chromosome 4 open reading frame 31	chr4q27	79625	LOC706304	hypothetical protein LOC706304	---		706304	MmugDNA.10982.1.S1_at_C4orf31
MmugDNA.10983.1.S1_at	214720_x_at	SEPT10	septin 10	chr2q13	151011	LOC711008	Similar to peptidylprolyl isomerase H	---		711008	MmugDNA.10983.1.S1_at_SEPT10
MmugDNA.10984.1.S1_at	216286_at	---	---	---	---	---	---	---	---		MmugDNA.10984.1.S1_at_---
MmugDNA.10985.1.S1_at	216028_at	---	---	---	---	---	---	---	---		MmugDNA.10985.1.S1_at_---
MmugDNA.10986.1.S1_s_at	NA	NA	NA	NA	NA	NA	NA	NA	NA		MmugDNA.10986.1.S1_s_at_NA
MmugDNA.10987.1.S1_at	235280_at	---	---	---	---	---	---	---	---		MmugDNA.10987.1.S1_at_---
MmugDNA.10988.1.S1_at	241435_at	---	---	---	---	---	---	---	---		MmugDNA.10988.1.S1_at_---
MmugDNA.10989.1.S1_at	1554117_at	CCDC60	coiled-coil domain containing 60	chr12q24.23	160777	LOC697641	hypothetical protein LOC697641	---		697641	MmugDNA.10989.1.S1_at_CCDC60
MmugDNA.10990.1.S1_at	226754_at	ZNF251	zinc finger protein 251	chr8q24.3	90987	LOC715399	similar to zinc finger protein 251	---		715399	MmugDNA.10990.1.S1_at_ZNF251
MmugDNA.10991.1.S1_at	238355_at	RBM39	RNA binding motif protein 39	chr20q11.22	9584	---	---	---	---		MmugDNA.10991.1.S1_at_RBM39
MmugDNA.1099.1.S1_at	239381_at	KLK7	kallikrein-related peptidase 7	chr19q13.33	5650	---	---	---	---		MmugDNA.1099.1.S1_at_KLK7
MmugDNA.10992.1.S1_at	1566265_at	---	---	---	---	---	---	---	---		MmugDNA.10992.1.S1_at_---
MmugDNA.10993.1.S1_at	240372_at	---	---	---	---	---	---	---	---		MmugDNA.10993.1.S1_at_---
MmugDNA.10994.1.S1_at	1564886_at	---	---	---	---	---	---	---	---		MmugDNA.10994.1.S1_at_---
MmugDNA.10995.1.S1_at	203831_at	R3HDM2	R3H domain containing 2	chr12q13.3	22864	LOC711448	similar to R3H domain-containing protein 2	---		711448	MmugDNA.10995.1.S1_at_R3HDM2
MmugDNA.10996.1.S1_at	218362_s_at	DIS3	DIS3 mitotic control homolog (S. cerevisiae)	chr13q22.1	22894	DIS3	DIS3 mitotic control homolog (S. cerevisiae)	---		694048	MmugDNA.10996.1.S1_at_DIS3
MmugDNA.10997.1.S1_at	231061_at	---	---	---	---	---	---	---	---		MmugDNA.10997.1.S1_at_---
MmugDNA.10998.1.S1_at	206006_s_at	KIAA1009	KIAA1009	chr6q14.3	22832	KIAA1009	KIAA1009	---		695775	MmugDNA.10998.1.S1_at_KIAA1009
MmugDNA.10999.1.S1_at	243780_at	---	---	---	---	---	---	---	---		MmugDNA.10999.1.S1_at_---
MmugDNA.11000.1.S1_at	238591_at	---	---	---	---	---	---	---	---		MmugDNA.11000.1.S1_at_---
MmugDNA.11001.1.S1_at	207305_s_at	KIAA1012	KIAA1012	chr18q12.1	22878	LOC705283	Similar to transcription factor-like nuclear regulator	---		705283	MmugDNA.11001.1.S1_at_KIAA1012
MmugDNA.1100.1.S1_at	203990_s_at	KDM6A	lysine (K)-specific demethylase 6A	chrXp11.2	7403	LOC709727	similar to ubiquitously transcribed tetratricopeptide repeat, X chromosome	---		709727	MmugDNA.1100.1.S1_at_KDM6A
MmugDNA.11002.1.S1_at	219582_at	OGFRL1	opioid growth factor receptor-like 1	chr6q13	79627	---	---	---	---		MmugDNA.11002.1.S1_at_OGFRL1
MmugDNA.11003.1.S1_at	203678_at	MTMR15	myotubularin related protein 15	chr15q13.2-q13.3	22909	LOC713125	similar to C01G5.8	---		713125	MmugDNA.11003.1.S1_at_MTMR15
MmugDNA.11004.1.S1_at	219710_at	SH3TC2	SH3 domain and tetratricopeptide repeats 2	chr5q32	79628	LOC709970	similar to SH3 domain and tetratricopeptide repeats 2	---		709970	MmugDNA.11004.1.S1_at_SH3TC2
MmugDNA.11005.1.S1_at	1564459_at	LOC100129427	hypothetical protein LOC100129427	chr7p12.2	100129427	---	---	---	---		MmugDNA.11005.1.S1_at_LOC1001294
MmugDNA.11006.1.S1_at	205441_at	OCEL1	occludin/ELL domain containing 1	chr19p13.11	79629	LOC718921	hypothetical protein LOC718921	---		718921	MmugDNA.11006.1.S1_at_OCEL1
MmugDNA.11007.1.S1_at	219506_at	C1orf54	chromosome 1 open reading frame 54	chr1q21.2	79630	---	---	---	---		MmugDNA.11007.1.S1_at_C1orf54
MmugDNA.11008.1.S1_at	243795_s_at	LOC440900	hypothetical LOC440900	chr2q14.1	440900	---	---	---	---		MmugDNA.11008.1.S1_at_LOC440900
MmugDNA.11009.1.S1_at	218973_at	EFTUD1	elongation factor Tu GTP binding domain containing 1	chr15q25.2	79631	LOC713570	similar to elongation factor Tu GTP binding domain containing 1	---		713570	MmugDNA.11009.1.S1_at_EFTUD1
MmugDNA.11010.1.S1_at	238395_at	---	---	---	---	---	---	---	---		MmugDNA.11010.1.S1_at_---
MmugDNA.11011.1.S1_at	228670_at	TEP1	telomerase-associated protein 1	chr14q11.2	7011	---	---	---	---		MmugDNA.11011.1.S1_at_TEP1
MmugDNA.1101.1.S1_at	215635_at	---	---	---	---	---	---	---	---		MmugDNA.1101.1.S1_at_---
MmugDNA.11012.1.S1_at	242819_at	---	---	---	---	---	---	---	---		MmugDNA.11012.1.S1_at_---
MmugDNA.11013.1.S1_at	NA	NA	NA	NA	NA	NA	NA	NA	NA		MmugDNA.11013.1.S1_at_NA
MmugDNA.11014.1.S1_at	243831_at	---	---	---	---	---	---	---	---		MmugDNA.11014.1.S1_at_---
MmugDNA.11015.1.S1_at	233673_at	LOC339524	Hypothetical LOC339524	chr1p22.3	339524	---	---	---	---		MmugDNA.11015.1.S1_at_LOC339524
MmugDNA.11016.1.S1_at	229833_at	---	---	---	---	---	---	---	---		MmugDNA.11016.1.S1_at_---
MmugDNA.11017.1.S1_at	237836_at	---	---	---	---	---	---	---	---		MmugDNA.11017.1.S1_at_---
MmugDNA.11018.1.S1_at	1558577_at	LOC148709	actin pseudogene	chr1q32.1	148709	---	---	---	---		MmugDNA.11018.1.S1_at_LOC148709
MmugDNA.11019.1.S1_at	1553039_a_at	ASB10	ankyrin repeat and SOCS box-containing 10	chr7q36.1	136371	LOC714306	similar to ankyrin repeat and SOCS box-containing protein 10	---		714306	MmugDNA.11019.1.S1_at_ASB10
MmugDNA.11019.1.S1_s_at	NA	NA	NA	NA	NA	NA	NA	NA	NA		MmugDNA.11019.1.S1_s_at_NA
MmugDNA.110.1.S1_at	210338_s_at	HSPA8	heat shock 70kDa protein 8	chr11q24.1	3312	LOC709412	similar to Heat Shock Protein family member (hsp-1)	---		709412	MmugDNA.110.1.S1_at_HSPA8
MmugDNA.11020.1.S1_at	203940_s_at	VASH1	vasohibin 1	chr14q24.3	22846	VASH1	vasohibin 1	---		703906	MmugDNA.11020.1.S1_at_VASH1
MmugDNA.11021.1.S1_at	1566673_at	---	---	---	---	---	---	---	---		MmugDNA.11021.1.S1_at_---
MmugDNA.1102.1.S1_at	238265_x_at	---	---	---	---	---	---	---	---		MmugDNA.1102.1.S1_at_---
MmugDNA.11022.1.S1_at	227976_at	LOC644538	hypothetical protein LOC644538	chrXq26.3	644538	LOC710283	hypothetical protein LOC710283	---		710283	MmugDNA.11022.1.S1_at_LOC644538
MmugDNA.11023.1.S1_at	202080_s_at	TRAK1	trafficking protein, kinesin binding 1	chr3p25.3-p24.1	22906	---	---	---	---		MmugDNA.11023.1.S1_at_TRAK1
MmugDNA.11024.1.S1_at	220150_s_at	FAM184A	family with sequence similarity 184, member A	chr6q22.31	79632	LOC714658	similar to Temporarily Assigned Gene name family member (tag-278)	---		714658	MmugDNA.11024.1.S1_at_FAM184A
MmugDNA.11025.1.S1_at	208911_s_at	PDHB	pyruvate dehydrogenase (lipoamide) beta	chr3p21.1-p14.2	5162	---	---	---	---		MmugDNA.11025.1.S1_at_PDHB
MmugDNA.11026.1.S1_at	203480_s_at	OTUD4	OTU domain containing 4	chr4q31.21	54726	OTUD4	OTU domain containing 4	---		701837	MmugDNA.11026.1.S1_at_OTUD4
MmugDNA.11027.1.S1_at	219427_at	FAT4	FAT tumor suppressor homolog 4 (Drosophila)	chr4q28.1	79633	LOC711867	similar to FAT tumor suppressor homolog 4	---		711867	MmugDNA.11027.1.S1_at_FAT4
MmugDNA.11028.1.S1_at	1554758_a_at	CD99L2	CD99 molecule-like 2	chrXq28	83692	---	---	---	---		MmugDNA.11028.1.S1_at_CD99L2
MmugDNA.11029.1.S1_at	235879_at	MBNL1	Muscleblind-like (Drosophila)	chr3q25	4154	---	---	---	---		MmugDNA.11029.1.S1_at_MBNL1
MmugDNA.11030.1.S1_s_at	NA	NA	NA	NA	NA	NA	NA	NA	NA		MmugDNA.11030.1.S1_s_at_NA
MmugDNA.11031.1.S1_at	1560509_at	---	---	---	---	---	---	---	---		MmugDNA.11031.1.S1_at_---
MmugDNA.1103.1.S1_at	205018_s_at	MBNL2	muscleblind-like 2 (Drosophila)	chr13q32.1	10150	LOC697097	similar to muscleblind-like 2 isoform 1	---		697097	MmugDNA.1103.1.S1_at_MBNL2
MmugDNA.11032.1.S1_at	219234_x_at	SCRN3	secernin 3	chr2q31.1	79634	SCRN3	secernin 3	---		698199	MmugDNA.11032.1.S1_at_SCRN3
MmugDNA.11033.1.S1_at	220321_s_at	CCDC121	coiled-coil domain containing 121	chr2p23.2	79635	---	---	---	---		MmugDNA.11033.1.S1_at_CCDC121
MmugDNA.11034.1.S1_at	239903_at	---	---	---	---	---	---	---	---		MmugDNA.11034.1.S1_at_---
MmugDNA.11035.1.S1_at	219096_at	ARMC7	armadillo repeat containing 7	chr17q25.1	79637	---	---	---	---		MmugDNA.11035.1.S1_at_ARMC7
MmugDNA.11036.1.S1_at	226867_at	DENND4C	DENN/MADD domain containing 4C	chr9p22.1	55667	LOC712447	similar to Protein C9orf55	---		712447	MmugDNA.11036.1.S1_at_DENND4C
MmugDNA.11037.1.S1_at	NA	NA	NA	NA	NA	NA	NA	NA	NA		MmugDNA.11037.1.S1_at_NA
MmugDNA.11037.1.S1_s_at	231777_at	LY6G5B	lymphocyte antigen 6 complex, locus G5B	chr6p21.3	58496	LOC715929	similar to lymphocyte antigen 6 complex G5B	---		715929	MmugDNA.11037.1.S1_s_at_LY6G5B
MmugDNA.11038.1.S1_at	NA	NA	NA	NA	NA	NA	NA	NA	NA		MmugDNA.11038.1.S1_at_NA
MmugDNA.11038.1.S1_s_at	NA	NA	NA	NA	NA	NA	NA	NA	NA		MmugDNA.11038.1.S1_s_at_NA
MmugDNA.11039.1.S1_at	229952_at	---	---	---	---	---	---	---	---		MmugDNA.11039.1.S1_at_---
MmugDNA.11040.1.S1_at	222465_at	RSL24D1	ribosomal L24 domain containing 1	chr15q21	51187	LOC703098	similar to ribosomal protein L24-like	---		703098	MmugDNA.11040.1.S1_at_RSL24D1
MmugDNA.11041.1.S1_at	229187_at	LOC283788	FSHD region gene 1 pseudogene	---	283788	---	---	---	---		MmugDNA.11041.1.S1_at_LOC283788
MmugDNA.1104.1.S1_at	234241_at	SCAMP5	secretory carrier membrane protein 5	chr15q24.1-q24.2	192683	---	---	---	---		MmugDNA.1104.1.S1_at_SCAMP5
MmugDNA.11042.1.S1_at	241155_at	---	---	---	---	---	---	---	---		MmugDNA.11042.1.S1_at_---
MmugDNA.11043.1.S1_at	NA	NA	NA	NA	NA	NA	NA	NA	NA		MmugDNA.11043.1.S1_at_NA
MmugDNA.11043.1.S1_s_at	202889_x_at	MAP7	microtubule-associated protein 7	chr6q23.3	9053	LOC707739	Similar to serine/threonine protein kinase MASK	---		707739	MmugDNA.11043.1.S1_s_at_MAP7
MmugDNA.11044.1.S1_at	205435_s_at	AAK1	AP2 associated kinase 1	chr2p14	22848	---	---	---	---		MmugDNA.11044.1.S1_at_AAK1
MmugDNA.11045.1.S1_at	NA	NA	NA	NA	NA	NA	NA	NA	NA		MmugDNA.11045.1.S1_at_NA
MmugDNA.11045.1.S1_s_at	202890_at	MAP7	microtubule-associated protein 7	chr6q23.3	9053	LOC707739	Similar to serine/threonine protein kinase MASK	---		707739	MmugDNA.11045.1.S1_s_at_MAP7
MmugDNA.11046.1.S1_at	212591_at	RBM34	RNA binding motif protein 34	chr1q42.3	23029	RBM34	RNA binding motif protein 34	---		711962	MmugDNA.11046.1.S1_at_RBM34
MmugDNA.11047.1.S1_at	204251_s_at	CEP164	centrosomal protein 164kDa	chr11q23.3	22897	CEP164	centrosomal protein 164kDa	---		697948	MmugDNA.11047.1.S1_at_CEP164
MmugDNA.11048.1.S1_at	1554386_at	CST9	cystatin 9 (testatin)	chr20p11.21	128822	CST9	cystatin 9 (testatin)	---		703522	MmugDNA.11048.1.S1_at_CST9
MmugDNA.11049.1.S1_at	207699_at	ZFHX2	zinc finger homeobox 2	chr14q11.2	85446	---	---	---	---		MmugDNA.11049.1.S1_at_ZFHX2
MmugDNA.11050.1.S1_at	242699_at	---	---	---	---	---	---	---	---		MmugDNA.11050.1.S1_at_---
MmugDNA.11051.1.S1_at	203464_s_at	EPN2	epsin 2	chr17p11.2	22905	EPN2	epsin 2	---		702600	MmugDNA.11051.1.S1_at_EPN2
MmugDNA.1105.1.S1_at	242841_at	---	---	---	---	---	---	---	---		MmugDNA.1105.1.S1_at_---
MmugDNA.11052.1.S1_at	218047_at	OSBPL9	oxysterol binding protein-like 9	chr1p32.3	114883	---	---	---	---		MmugDNA.11052.1.S1_at_OSBPL9
MmugDNA.11053.1.S1_at	226273_at	CLCN5	chloride channel 5	chrXp11.23	1184	---	---	---	---		MmugDNA.11053.1.S1_at_CLCN5
MmugDNA.11054.1.S1_at	219462_at	TMEM53	transmembrane protein 53	chr1p34.1	79639	LOC705023	similar to transmembrane protein 53	---		705023	MmugDNA.11054.1.S1_at_TMEM53
MmugDNA.11055.1.S1_at	226274_at	CLCN5	chloride channel 5	chrXp11.23	1184	---	---	---	---		MmugDNA.11055.1.S1_at_CLCN5
MmugDNA.11056.1.S1_at	236434_at	PES1	pescadillo homolog 1, containing BRCT domain (zebrafish)	chr22q12.1	23481	PES1	pescadillo homolog 1, containing BRCT domain	---		718122	MmugDNA.11056.1.S1_at_PES1
MmugDNA.11057.1.S1_at	227800_at	FAM110B	family with sequence similarity 110, member B	chr8q12.1	90362	LOC703840	hypothetical protein LOC703840	---		703840	MmugDNA.11057.1.S1_at_FAM110B
MmugDNA.11058.1.S1_at	224819_at	TCEAL8	transcription elongation factor A (SII)-like 8	chrXq22.1	90843	LOC696504	similar to transcription elongation factor A (SII)-like 8	---		696504	MmugDNA.11058.1.S1_at_TCEAL8
MmugDNA.11059.1.S1_at	219309_at	CTA-216E10.6	hypothetical FLJ23584	chr22q13.2	79640	LOC712231	similar to Ribosome-binding protein 1 (Ribosome receptor protein) (mRRp)	---		712231	MmugDNA.11059.1.S1_at_CTA-216E1(
MmugDNA.11060.1.S1_at	221959_at	FAM110B	family with sequence similarity 110, member B	chr8q12.1	90362	LOC704185	hypothetical protein LOC704185	---		704185	MmugDNA.11060.1.S1_at_FAM110B
MmugDNA.11061.1.S1_at	236663_at	---	---	---	---	---	---	---	---		MmugDNA.11061.1.S1_at_---
MmugDNA.1106.1.S1_at	203389_at	KIF3C	kinesin family member 3C	chr2p23	3797	KIF3C	kinesin family member 3C	---		695287	MmugDNA.1106.1.S1_at_KIF3C
MmugDNA.11062.1.S1_at	227668_at	C17orf56	chromosome 17 open reading frame 56	chr17q25.3	146705	LOC718666	hypothetical protein LOC718666	---		718666	MmugDNA.11062.1.S1_at_C17orf56
MmugDNA.11063.1.S1_at	221773_at	ELK3	ELK3, ETS-domain protein (SRF accessory protein 2)	chr12q23	2004	LOC713100	similar to ELK3 protein	---		713100	MmugDNA.11063.1.S1_at_ELK3
MmugDNA.11064.1.S1_at	218394_at	ROGDI	rogdi homolog (Drosophila)	chr16p13.3	79641	ROGDI	rogdi homolog (Drosophila)	---		706735	MmugDNA.11064.1.S1_at_ROGDI
MmugDNA.11065.1.S1_at	232849_at	LOC100128988	similar to hCG2018847	chr20q12	100128988	---	---	---	---		MmugDNA.11065.1.S1_at_LOC100128(
MmugDNA.11066.1.S1_at	212771_at	FAM171A1	family with sequence similarity 171, member A1	chr10p13	221061	LOC703843	hypothetical protein LOC703843	---		703843	MmugDNA.11066.1.S1_at_FAM171A1
MmugDNA.11067.1.S1_at	240174_at	---	---	---	---	---	---	---	---		MmugDNA.11067.1.S1_at_---
MmugDNA.11068.1.S1_at	222927_s_at	CPLX3 /// LMAN1L	complexin 3 /// lectin, mannose-binding, 1 like	chr15q24.1	594855 /// 79748	---	---	---	---		MmugDNA.11068.1.S1_at_CPLX3 /// LM
MmugDNA.11069.1.S1_at	210798_x_at	MASP2	mannan-binding lectin serine peptidase 2	chr1p36.3-p36.2	10747	LOC722714	similar to mannan-binding lectin serine protease 2 isoform 2 precursor	---		722714	MmugDNA.11069.1.S1_at_MASP2
MmugDNA.11070.1.S1_at	244144_at	SYNE1	spectrin repeat containing, nuclear envelope 1	chr6q25	23345	LOC711609	similar to nesprin 1 longest	---		711609	MmugDNA.11070.1.S1_at_SYNE1
MmugDNA.11071.1.S1_at	242052_at	---	---	---	---	---	---	---	---		MmugDNA.11071.1.S1_at_---

MmugDNA.1107.1.S1_at	242531_at	RRAGC	Ras-related GTP binding C	chr1p34		64121	---	---	---	---		MmugDNA.1107.1.S1_at_RRAGC
MmugDNA.11072.1.S1_at	209284_s_at	C3orf63	chromosome 3 open reading frame 63	chr3p14.3		23272	RB1	similar to retinoblastoma-associated protein 140	---		708452	MmugDNA.11072.1.S1_at_C3orf63
MmugDNA.11073.1.S1_at	1559571_a_at	ATP13A4	ATPase type 13A4	chr3q29		84239	ATP13A4	ATPase type 13A4	---		698284	MmugDNA.11073.1.S1_at_ATP13A4
MmugDNA.11074.1.S1_at	209285_s_at	C3orf63	chromosome 3 open reading frame 63	chr3p14.3		23272	RB1	similar to retinoblastoma-associated protein 140	---		708452	MmugDNA.11074.1.S1_at_C3orf63
MmugDNA.11075.1.S1_at	NA	NA	NA	NA	NA		NA	NA	NA	NA		MmugDNA.11075.1.S1_at_NA
MmugDNA.11076.1.S1_at	205706_s_at	ANKRD26	ankyrin repeat domain 26	chr10pter-q22.1		22852	LOC707795	similar to ankyrin repeat domain 26	---		707795	MmugDNA.11076.1.S1_at_ANKRD26
MmugDNA.11077.1.S1_at	206223_at	LMTK2	lemur tyrosine kinase 2	chr7q21.3		22853	LOC718147	similar to lemur tyrosine kinase 2	---		718147	MmugDNA.11077.1.S1_at_LMTK2
MmugDNA.11078.1.S1_at	NA	NA	NA	NA	NA		NA	NA	NA	NA		MmugDNA.11078.1.S1_at_NA
MmugDNA.11078.1.S1_s_at	221529_s_at	PLVAP	plasmalemma vesicle associated protein	chr19p13.2		83483	---	---	---	---		MmugDNA.11078.1.S1_s_at_PLVAP
MmugDNA.11079.1.S1_at	219973_at	ARSJ	arylsulfatase family, member J	chr4q26		79642	ARSJ	arylsulfatase family, member J	---		702633	MmugDNA.11079.1.S1_at_ARSJ
MmugDNA.11080.1.S1_at	242194_at	CUL4A	Cullin 4A	chr13q34		8451	---	---	---	---		MmugDNA.11080.1.S1_at_CUL4A
MmugDNA.11081.1.S1_at	NA	NA	NA	NA	NA		NA	NA	NA	NA		MmugDNA.11081.1.S1_at_NA
MmugDNA.11081.1.S1_x_at	211825_s_at	EWSR1 /// FLI1	Ewing sarcoma breakpoint region 1 /// Friend leukemia virus integration 1	chr11q24.1-q24.3 /// chr22q12.2	2130 /// 2313		FLI1	Friend leukemia virus integration 1	---		714748	MmugDNA.11081.1.S1_x_at_EWSR1 //
MmugDNA.1108.1.S1_at	222623_s_at	ZNF639	zinc finger protein 639	chr3q26.32		51193	ZNF639	zinc finger protein 639	---		711860	MmugDNA.1108.1.S1_at_ZNF639
MmugDNA.11082.1.S1_at	218743_at	CHMP6	chromatin modifying protein 6	chr17q25.3		79643	---	---	---	---		MmugDNA.11082.1.S1_at_CHMP6
MmugDNA.11083.1.S1_at	206225_at	ZNF507	zinc finger protein 507	chr19q13.11		22847	---	---	---	---		MmugDNA.11083.1.S1_at_ZNF507
MmugDNA.11084.1.S1_at	218800_at	SRD5A3	steroid 5 alpha-reductase 3	chr4q12		79644	SRD5A3	steroid 5 alpha-reductase 3	---		696381	MmugDNA.11084.1.S1_at_SRD5A3
MmugDNA.11085.1.S1_at	220156_at	EFCAB1	EF-hand calcium binding domain 1	chr8q11.21		79645	LOC708842	similar to EF-hand calcium binding domain 1	---		708842	MmugDNA.11085.1.S1_at_EFCAB1
MmugDNA.11086.1.S1_at	217500_at	TIAL1	TIA1 cytotoxic granule-associated RNA binding protein-like 1	chr10q		7073	TIAL1	TIA1 cytotoxic granule-associated RNA binding protein-like 1	---		703249	MmugDNA.11086.1.S1_at_TIAL1
MmugDNA.11087.1.S1_s_at	NA	NA	NA	NA	NA		NA	NA	NA	NA		MmugDNA.11087.1.S1_s_at_NA
MmugDNA.11088.1.S1_at	218433_at	PANK3	pantothenate kinase 3	chr5q34		79646	---	---	---	---		MmugDNA.11088.1.S1_at_PANK3
MmugDNA.11089.1.S1_s_at	211076_x_at	ATN1	Atrophin 1	chr12p13.31		1822	ATN1	atrophin 1	---		722114	MmugDNA.11089.1.S1_s_at_ATN1
MmugDNA.11090.1.S1_at	230546_at	VASH1	vasohibin 1	chr14q24.3		22846	VASH1	vasohibin 1	---		703906	MmugDNA.11090.1.S1_at_VASH1
MmugDNA.11091.1.S1_at	1557639_at	NFIA	Nuclear factor I/A	chr1p31.3-p31.2		4774	---	---	---	---		MmugDNA.11091.1.S1_at_NFIA
MmugDNA.1109.1.S1_at	222624_s_at	ZNF639	zinc finger protein 639	chr3q26.32		51193	ZNF639	similar to Zinc finger protein 639 (Zinc finger protein ZASC1) (Zinc finger protein ANC_2H01)	---		716299	MmugDNA.1109.1.S1_at_ZNF639
MmugDNA.11092.1.S1_at	1558523_at	FAM184A	family with sequence similarity 184, member A	chr6q22.31		79632	LOC714658	similar to Temporarily Assigned Gene name family member (tag-278)	---		714658	MmugDNA.11092.1.S1_at_FAM184A
MmugDNA.11093.1.S1_at	1563360_at	---	---	---	---		---	---	---	---		MmugDNA.11093.1.S1_at_---
MmugDNA.11094.1.S1_at	NA	NA	NA	NA	NA		NA	NA	NA	NA		MmugDNA.11094.1.S1_at_NA
MmugDNA.11095.1.S1_at	1552726_at	ADAMTS17	ADAM metallopeptidase with thrombospondin type 1 motif, 17	chr15q24		170691	ADAMTS17	ADAM metallopeptidase with thrombospondin type 1 motif, 17	---		693447	MmugDNA.11095.1.S1_at_ADAMTS17
MmugDNA.11096.1.S1_at	1552727_s_at	ADAMTS17	ADAM metallopeptidase with thrombospondin type 1 motif, 17	chr15q24		170691	---	---	---	---		MmugDNA.11096.1.S1_at_ADAMTS17
MmugDNA.11097.1.S1_at	227649_s_at	SRGAP2	SLIT-ROBO Rho GTPase activating protein 2	chr1q32.1		23380	LOC704579	Similar to Ribonuclease K6 precursor (RNase K6)	---		704579	MmugDNA.11097.1.S1_at_SRGAP2
MmugDNA.11098.1.S1_at	231667_at	SLC39A5	solute carrier family 39 (metal ion transporter), member 5	chr12q13.2		283375	LOC712131	similar to solute carrier family 39 (metal ion transporter), member 5	---		712131	MmugDNA.11098.1.S1_at_SLC39A5
MmugDNA.11099.1.S1_at	242558_at	---	---	---	---		---	---	---	---		MmugDNA.11099.1.S1_at_---
MmugDNA.11100.1.S1_at	230534_at	ZNF678	zinc finger protein 678	chr1q42.13		339500	---	---	---	---		MmugDNA.11100.1.S1_at_ZNF678
MmugDNA.11101.1.S1_at	229064_s_at	RCAN3	RCAN family member 3	chr1p35.3-p33		11123	---	---	---	---		MmugDNA.11101.1.S1_at_RCAN3
MmugDNA.1110.1.S1_at	242679_at	LOC100131039	Hypothetical protein LOC100131039	chr2q31.2		100131039	---	---	---	---		MmugDNA.1110.1.S1_at_LOC1001310
MmugDNA.11102.1.S1_at	222286_at	SNAPC3	small nuclear RNA activating complex, polypeptide 3, 50kDa	chr9p22.3		6619	---	---	---	---		MmugDNA.11102.1.S1_at_SNAPC3
MmugDNA.11103.1.S1_at	243954_at	LOC285286	hypothetical protein LOC285286	chr3p14.2		285286	---	---	---	---		MmugDNA.11103.1.S1_at_LOC285286
MmugDNA.11104.1.S1_at	216397_s_at	BOP1	block of proliferation 1	chr8q24.3		23246	LOC714024	similar to block of proliferation 1	---		714024	MmugDNA.11104.1.S1_at_BOP1
MmugDNA.11105.1.S1_at	204066_s_at	AGAP1	ArfGAP with GTPase domain, ankyrin repeat and PH domain 1	chr2q37		116987	AGAP1	ArfGAP with GTPase domain, ankyrin repeat and PH domain 1	---		693652	MmugDNA.11105.1.S1_at_AGAP1
MmugDNA.11106.1.S1_at	213472_at	HNRNPH1	heterogeneous nuclear ribonucleoprotein H1 (H)	chr5q35.3		3187	HNRPH1	heterogeneous nuclear ribonucleoprotein H1	---		704469	MmugDNA.11106.1.S1_at_HNRNPH1
MmugDNA.11107.1.S1_at	213470_s_at	HNRNPH1	heterogeneous nuclear ribonucleoprotein H1 (H)	chr5q35.3		3187	HNRPH1	heterogeneous nuclear ribonucleoprotein H1	---		704469	MmugDNA.11107.1.S1_at_HNRNPH1
MmugDNA.11108.1.S1_at	203286_at	RNF44	ring finger protein 44	chr5q35.2		22838	LOC702911	similar to ring finger protein 44	---		702911	MmugDNA.11108.1.S1_at_RNF44
MmugDNA.11109.1.S1_at	238055_at	ATP8B1	ATPase, class I, type 8B, member 1	chr18q21-q22|18q21.31		5205	---	---	---	---		MmugDNA.11109.1.S1_at_ATP8B1
MmugDNA.11110.1.S1_at	217893_s_at	AKIRIN1	akirin 1	chr1p34.3		79647	LOC718520	similar to CG8580-PA, isoform A	---		718520	MmugDNA.11110.1.S1_at_AKIRIN1
MmugDNA.11111.1.S1_at	205273_s_at	PITRM1	pitrilysin metallopeptidase 1	chr10p15.2		10531	LOC710684	similar to metalloprotease 1	---		710684	MmugDNA.11111.1.S1_at_PITRM1
MmugDNA.1111.1.S1_at	241141_at	BMP6	Bone morphogenetic protein 6	chr6p24-p23		654	---	---	---	---		MmugDNA.1111.1.S1_at_BMP6
MmugDNA.11112.1.S1_at	219592_at	MCPH1	microcephalin 1	chr8p23.1		79648	---	---	---	---		MmugDNA.11112.1.S1_at_MCPH1
MmugDNA.11113.1.S1_at	219626_at	MAP7D3	MAP7 domain containing 3	chrXq26.3		79649	LOC712823	hypothetical protein LOC712823	---		712823	MmugDNA.11113.1.S1_at_MAP7D3
MmugDNA.11114.1.S1_s_at	1554016_a_at	C16orf57	chromosome 16 open reading frame 57	chr16q13		79650	---	---	---	---		MmugDNA.11114.1.S1_s_at_C16orf57
MmugDNA.11115.1.S1_at	NA	NA	NA	NA	NA		NA	NA	NA	NA		MmugDNA.11115.1.S1_at_NA
MmugDNA.11115.1.S1_s_at	219202_at	RHBDF2	rhomboid 5 homolog 2 (Drosophila)	chr17q25.1		79651	LOC707033	similar to rhomboid-like protein 6	---		707033	MmugDNA.11115.1.S1_s_at_RHBDF2
MmugDNA.11116.1.S1_at	237202_at	PGPEP1	pyroglutamyl-peptidase I	chr19p13.11		54858	PGPEP1	pyroglutamyl-peptidase I	---		720200	MmugDNA.11116.1.S1_at_PGPEP1
MmugDNA.11117.1.S1_at	1553810_a_at	KIAA1524	KIAA1524	chr3q13.13		57650	KIAA1524	KIAA1524	---		705690	MmugDNA.11117.1.S1_at_KIAA1524
MmugDNA.11118.1.S1_at	234661_at	CCDC57	coiled-coil domain containing 57	chr17q25.3		284001	---	---	---	---		MmugDNA.11118.1.S1_at_CCDC57
MmugDNA.11119.1.S1_at	232880_at	---	---	---	---		---	---	---	---		MmugDNA.11119.1.S1_at_---
MmugDNA.111.1.S1_at	219495_s_at	ZNF180	zinc finger protein 180	chr19q13.2		7733	ZNF180	zinc finger protein 180	---		712493	MmugDNA.111.1.S1_at_ZNF180
MmugDNA.11120.1.S1_at	228419_at	METRN	meteorin, glial cell differentiation regulator	chr16p13.3		79006	---	---	---	---		MmugDNA.11120.1.S1_at_METRN
MmugDNA.11121.1.S1_at	1562950_at	---	---	---	---		---	---	---	---		MmugDNA.11121.1.S1_at_---
MmugDNA.1112.1.S1_at	1560901_at	---	---	---	---		---	---	---	---		MmugDNA.1112.1.S1_at_---
MmugDNA.11122.1.S1_at	240709_at	SEZ6L	seizure related 6 homolog (mouse)-like	chr22q12.1		23544	SEZ6L	seizure related 6 homolog (mouse)-like	---		714925	MmugDNA.11122.1.S1_at_SEZ6L
MmugDNA.11123.1.S1_at	228838_at	---	---	---	---		---	---	---	---		MmugDNA.11123.1.S1_at_---
MmugDNA.11124.1.S1_at	1562112_at	---	---	---	---		---	---	---	---		MmugDNA.11124.1.S1_at_---
MmugDNA.11125.1.S1_at	1564200_at	LOC646324	hypothetical LOC646324	chr2q35		646324	---	---	---	---		MmugDNA.11125.1.S1_at_LOC646324
MmugDNA.11126.1.S1_at	1563319_at	---	---	---	---		---	---	---	---		MmugDNA.11126.1.S1_at_---
MmugDNA.11127.1.S1_at	1563154_at	---	---	---	---		---	---	---	---		MmugDNA.11127.1.S1_at_---
MmugDNA.11128.1.S1_at	230631_s_at	---	---	---	---		---	---	---	---		MmugDNA.11128.1.S1_at_---
MmugDNA.11129.1.S1_at	235256_s_at	GALM	galactose mutarotase (aldose 1-epimerase)	chr2p22.1		130589	LOC713322	similar to galactose mutarotase (aldose 1-epimerase)	---		713322	MmugDNA.11129.1.S1_at_GALM
MmugDNA.11130.1.S1_at	243256_at	MKNK1	MAP kinase interacting serine/threonine kinase 1	chr1p33		8569	---	---	---	---		MmugDNA.11130.1.S1_at_MKNK1
MmugDNA.11131.1.S1_at	236801_at	---	---	---	---		---	---	---	---		MmugDNA.11131.1.S1_at_---
MmugDNA.1113.1.S1_at	238247_at	---	---	---	---		---	---	---	---		MmugDNA.1113.1.S1_at_---
MmugDNA.11132.1.S1_at	1553082_at	CRYGN	crystallin, gamma N	chr7q36.1		155051	LOC715040	similar to crystallin, gamma N	---		715040	MmugDNA.11132.1.S1_at_CRYGN
MmugDNA.11133.1.S1_at	1559986_at	---	---	---	---		---	---	---	---		MmugDNA.11133.1.S1_at_---
MmugDNA.11134.1.S1_at	217525_at	OLFML1	olfactomedin-like 1	chr11p15.4		283298	---	---	---	---		MmugDNA.11134.1.S1_at_OLFML1
MmugDNA.11135.1.S1_at	225654_at	NSD1	nuclear receptor binding SET domain protein 1	chr5q35.2-q35.3		64324	NSD1	nuclear receptor binding SET domain protein 1	---		706131	MmugDNA.11135.1.S1_at_NSD1
MmugDNA.11136.1.S1_at	221302_at	KLF15	Kruppel-like factor 15	chr3q13-q21		28999	KLF15	Kruppel-like factor 15	---		716386	MmugDNA.11136.1.S1_at_KLF15
MmugDNA.11137.1.S1_at	211605_s_at	RARA	retinoic acid receptor, alpha	chr17q21		5914	RARA	retinoic acid receptor, alpha	---		700206	MmugDNA.11137.1.S1_at_RARA
MmugDNA.11138.1.S1_at	1555958_at	CRTAC1	cartilage acidic protein 1	chr10q22		55118	CRTAC1	cartilage acidic protein 1	---		707486	MmugDNA.11138.1.S1_at_CRTAC1
MmugDNA.11139.1.S1_at	1563465_at	PKD1L1	polycystic kidney disease 1 like 1	chr7p12.3		168507	---	---	---	---		MmugDNA.11139.1.S1_at_PKD1L1
MmugDNA.11140.1.S1_at	239552_at	VWDE	von Willebrand factor D and EGF domains	chr7p21.3		221806	LOC694004	similar to notch homolog 5	---		694004	MmugDNA.11140.1.S1_at_VWDE
MmugDNA.11141.1.S1_at	239062_at	LOC100131096	hypothetical LOC100131096	chr17q25.3		100131096	---	---	---	---		MmugDNA.11141.1.S1_at_LOC100131(
MmugDNA.1114.1.S1_at	1555357_at	DDX53	DEAD (Asp-Glu-Ala-Asp) box polypeptide 53	chrXp22.11		168400	LOC699052	similar to DEAD (Asp-Glu-Ala-Asp) box polypeptide 53	---		699052	MmugDNA.1114.1.S1_at_DDX53
MmugDNA.11142.1.S1_at	244516_at	---	---	---	---		---	---	---	---		MmugDNA.11142.1.S1_at_---
MmugDNA.11143.1.S1_at	202741_at	PRKACB	protein kinase, cAMP-dependent, catalytic, beta	chr1p36.1		5567	PRKACB	protein kinase, cAMP-dependent, catalytic, beta	---		709728	MmugDNA.11143.1.S1_at_PRKACB
MmugDNA.11144.1.S1_s_at	234899_at	H2BFM	H2B histone family, member M	chrXq22.2		286436	LOC698930	similar to H2B histone family, member W, testis-specific	---		698930	MmugDNA.11144.1.S1_s_at_H2BFM
MmugDNA.11145.1.S1_at	215394_at	PIK3C3	phosphoinositide-3-kinase, class 3	chr18q12.3		5289	---	---	---	---		MmugDNA.11145.1.S1_at_PIK3C3
MmugDNA.11146.1.S1_at	231404_at	---	---	---	---		LOC693403	hypothetical protein LOC693403	---		693403	MmugDNA.11146.1.S1_at_---
MmugDNA.11146.1.S1_s_at	NA	NA	NA	NA	NA		NA	NA	NA	NA		MmugDNA.11146.1.S1_s_at_NA
MmugDNA.11147.1.S1_at	244264_at	KLRG2	killer cell lectin-like receptor subfamily G, member 2	chr7q34		346689	---	---	---	---		MmugDNA.11147.1.S1_at_KLRG2
MmugDNA.11148.1.S1_at	224515_at	---	---	---	---		---	---	---	---		MmugDNA.11148.1.S1_at_---
MmugDNA.11149.1.S1_at	1559537_at	---	---	---	---		---	---	---	---		MmugDNA.11149.1.S1_at_---
MmugDNA.11150.1.S1_at	1562854_at	---	---	---	---		---	---	---	---		MmugDNA.11150.1.S1_at_---
MmugDNA.11151.1.S1_s_at	201102_s_at	PFKL	phosphofructokinase, liver	chr21q22.3		5211	LOC722173	similar to liver phosphofructokinase isoform b	---		722173	MmugDNA.11151.1.S1_s_at_PFKL
MmugDNA.1115.1.S1_at	240662_at	---	---	---	---		---	---	---	---		MmugDNA.1115.1.S1_at_---
MmugDNA.11152.1.S1_at	1556638_at	PAX7	paired box 7	chr1p36.13		5081	---	---	---	---		MmugDNA.11152.1.S1_at_PAX7
MmugDNA.11153.1.S1_at	214874_at	PKP4	plakophilin 4	chr2q23-q31		8502	---	---	---	---		MmugDNA.11153.1.S1_at_PKP4
MmugDNA.11154.1.S1_at	NA	NA	NA	NA	NA		NA	NA	NA	NA		MmugDNA.11154.1.S1_at_NA
MmugDNA.11155.1.S1_at	241453_at	PTK2	PTK2 protein tyrosine kinase 2	chr8q24-qter		5747	PTK2	PTK2 protein tyrosine kinase 2	---		700634	MmugDNA.11155.1.S1_at_PTK2
MmugDNA.11156.1.S1_at	243551_at	---	---	---	---		---	---	---	---		MmugDNA.11156.1.S1_at_---
MmugDNA.11157.1.S1_at	NA	NA	NA	NA	NA		NA	NA	NA	NA		MmugDNA.11157.1.S1_at_NA
MmugDNA.11157.1.S1_s_at	213589_s_at	B3GNTL1	UDP-GlcNAc:betaGal beta-1,3-N-acetylglucosaminyltransferase-like 1	chr17q25.3		146712	LOC719689	similar to UDP-GlcNAc:betaGal beta-1,3-N-acetylglucosaminyltransferase-like 1	---		719689	MmugDNA.11157.1.S1_s_at_B3GNTL1
MmugDNA.11158.1.S1_at	NA	NA	NA	NA	NA		NA	NA	NA	NA		MmugDNA.11158.1.S1_at_NA
MmugDNA.11159.1.S1_at	228675_at	LOC100131733	hypothetical protein LOC100131733	chr12q24.11		100131733	---	---	---	---		MmugDNA.11159.1.S1_at_LOC100131
MmugDNA.11160.1.S1_at	NA	NA	NA	NA	NA		NA	NA	NA	NA		MmugDNA.11160.1.S1_at_NA
MmugDNA.11161.1.S1_at	244562_s_at	---	---	---	---		---	---	---	---		MmugDNA.11161.1.S1_at_---
MmugDNA.1116.1.S1_at	236202_at	---	---	---	---		---	---	---	---		MmugDNA.1116.1.S1_at_---
MmugDNA.11162.1.S1_at	230485_at	LOC644844	hypothetical protein LOC644844	chr15q15.1		644844	LOC707422	hypothetical protein LOC707422	---		707422	MmugDNA.11162.1.S1_at_LOC644844
MmugDNA.11163.1.S1_at	229533_x_at	ZNF680	zinc finger protein 680	chr7q11.21		340252	LOC708683	similar to zinc finger protein 92 (HTF12) isoform 2	---		708683	MmugDNA.11163.1.S1_at_ZNF680

MmugDNA.11164.1.S1_at	NA	NA	NA	NA	NA	NA	NA	NA	NA	MmugDNA.11164.1.S1_at_NA
MmugDNA.11165.1.S1_at	210956_at	PPYR1	pancreatic polypeptide receptor 1	chr10q11.2	5540	PPYR1	pancreatic polypeptide receptor 1	---	694492	MmugDNA.11165.1.S1_at_PPYR1
MmugDNA.11166.1.S1_s_at	215737_x_at	USF2	upstream transcription factor 2, c-fos interacting	chr19q13	7392	LOC718527	similar to LISCH protein isoform 1	---	718527	MmugDNA.11166.1.S1_s_at_USF2
MmugDNA.11167.1.S1_at	242636_at	---	---	---	---	PRCP	prolylcarboxypeptidase	---	701305	MmugDNA.11167.1.S1_at_---
MmugDNA.11168.1.S1_at	209702_at	FTO	fat mass and obesity associated	chr16q12.2	79068	FTO	fat mass and obesity associated	---	703724	MmugDNA.11168.1.S1_at_FTO
MmugDNA.11169.1.S1_at	215094_at	CCDC52	coiled-coil domain containing 52	chr3q13.2	152185	LOC709409	similar to coiled-coil domain containing 52	---	709409	MmugDNA.11169.1.S1_at_CCDC52
MmugDNA.11170.1.S1_at	240078_at	SFRS8	splicing factor, arginine/serine-rich 8 (suppressor-of-white-apricot homolog, Drosophila)	chr12q24.33	6433	---	---	---	---	MmugDNA.11170.1.S1_at_SFRS8
MmugDNA.11171.1.S1_at	224516_s_at	CXXC5	CXXC finger 5	chr5q31.3	51523	CXXC5	CXXC finger 5	---	694746	MmugDNA.11171.1.S1_at_CXXC5
MmugDNA.1117.1.S1_at	244592_at	---	---	---	---	---	---	---	---	MmugDNA.1117.1.S1_at_---
MmugDNA.11172.1.S1_s_at	233588_x_at	PFDN6	prefoldin subunit 6	chr6p21.3	10471	LOC718253	similar to MHC class II region expressed gene KE2	---	718253	MmugDNA.11172.1.S1_s_at_PFDN6
MmugDNA.11173.1.S1_at	237522_at	FAS	Fas (TNF receptor superfamily, member 6)	chr10q24.1	355	---	---	---	---	MmugDNA.11173.1.S1_at_FAS
MmugDNA.11174.1.S1_at	1558858_at	---	---	---	---	LOC713260	hypothetical protein LOC713260	---	713260	MmugDNA.11174.1.S1_at_---
MmugDNA.11175.1.S1_at	1562276_at	---	---	---	---	---	---	---	---	MmugDNA.11175.1.S1_at_---
MmugDNA.11176.1.S1_at	1553526_at	NLRP8	NLR family, pyrin domain containing 8	chr19q13.42	126205	NLRP8	NLR family, pyrin domain containing 8	---	100135419	MmugDNA.11176.1.S1_at_NLRP8
MmugDNA.11177.1.S1_at	241594_at	LOC643449	similar to hCG2041756	chr4p12	643449	---	---	---	---	MmugDNA.11177.1.S1_at_LOC643449
MmugDNA.11178.1.S1_at	234082_at	---	---	---	---	---	---	---	---	MmugDNA.11178.1.S1_at_---
MmugDNA.11179.1.S1_at	238860_at	C6orf130	chromosome 6 open reading frame 130	chr6p21.1	221443	LOC719685	similar to Protein C6orf130 homolog	---	719685	MmugDNA.11179.1.S1_at_C6orf130
MmugDNA.11180.1.S1_at	215863_at	TFR2	transferrin receptor 2	chr7q22	7036	---	---	---	---	MmugDNA.11180.1.S1_at_TFR2
MmugDNA.11181.1.S1_at	236316_at	FAM3C	family with sequence similarity 3, member C	chr7q31	10447	LOC694209	similar to Protein FAM3C precursor (Protein GS3786)	---	694209	MmugDNA.11181.1.S1_at_FAM3C
MmugDNA.1118.1.S1_at	1557613_at	FLJ39534	hypothetical FLJ39534	chr3p21.31	285352	---	---	---	---	MmugDNA.1118.1.S1_at_FLJ39534
MmugDNA.11182.1.S1_at	1562476_at	---	---	---	---	---	---	---	---	MmugDNA.11182.1.S1_at_---
MmugDNA.11183.1.S1_at	244821_at	---	---	---	---	---	---	---	---	MmugDNA.11183.1.S1_at_---
MmugDNA.11184.1.S1_at	233745_at	---	---	---	---	---	---	---	---	MmugDNA.11184.1.S1_at_---
MmugDNA.11185.1.S1_at	205528_s_at	RUNX1T1	runt-related transcription factor 1; translocated to, 1 (cyclin D-related)	chr8q22	862	LOC696934	hypothetical protein LOC696934	---	696934	MmugDNA.11185.1.S1_at_RUNX1T1
MmugDNA.11186.1.S1_at	NA	NA	NA	NA	NA	NA	NA	NA	NA	MmugDNA.11186.1.S1_at_NA
MmugDNA.11186.1.S1_s_at	204152_s_at	MFNG	MFNG O-fucosylpeptide 3-beta-N-acetylglucosaminyltransferase	chr22q12	4242	LOC714101	Similar to LIM/homeobox protein Lhx1 (LIM homeobox protein 1) (Homeobox protein Lim-1)	---	714101	MmugDNA.11186.1.S1_s_at_MFNG
MmugDNA.11187.1.S1_at	1559194_a_at	CLEC4GP1	C-type lectin domain family 4, member G pseudogene 1	chr19p13.2	440508	LOC705334	similar to C-type lectin superfamily 4, member G	---	705334	MmugDNA.11187.1.S1_at_CLEC4GP1
MmugDNA.11188.1.S1_at	222829_s_at	IL20RA	interleukin 20 receptor, alpha	chr6q22.33-q23.1	53832	IL20RA	interleukin 20 receptor, alpha	---	704890	MmugDNA.11188.1.S1_at_IL20RA
MmugDNA.11189.1.S1_at	221713_s_at	MAP6D1	MAP6 domain containing 1	chr3q27.1	79929	LOC714058	Cytochrome b5 reductase	---	714058	MmugDNA.11189.1.S1_at_MAP6D1
MmugDNA.11190.1.S1_at	238100_at	AAK1	AP2 associated kinase 1	chr2p14	22848	---	---	---	---	MmugDNA.11190.1.S1_at_AAK1
MmugDNA.11191.1.S1_at	1554203_at	NCRNA00110	non-protein coding RNA 110	chr21q22.11	642976	---	---	---	---	MmugDNA.11191.1.S1_at_NCRNA0011
MmugDNA.1119.1.S1_at	215754_at	SCARB2	scavenger receptor class B, member 2	chr4q21.1	950	---	---	---	---	MmugDNA.1119.1.S1_at_SCARB2
MmugDNA.11192.1.S1_at	1569256_a_at	FAM43B	family with sequence similarity 43, member B	chr1p36.12	163933	LOC697015	Similar to OTU domain containing 6B	---	697015	MmugDNA.11192.1.S1_at_FAM43B
MmugDNA.11193.1.S1_s_at	228685_at	---	---	---	---	LOC710934	Similar to tripartite motif-containing 37 protein	---	710934	MmugDNA.11193.1.S1_s_at_---
MmugDNA.11194.1.S1_at	240654_at	---	---	---	---	---	---	---	---	MmugDNA.11194.1.S1_at_---
MmugDNA.11195.1.S1_at	239982_at	---	---	---	---	---	---	---	---	MmugDNA.11195.1.S1_at_---
MmugDNA.11196.1.S1_at	228709_at	TPR	translocated promoter region (to activated MET oncogene)	chr1q25	7175	TPR	translocated promoter region (to activated MET oncogene)	---	713910	MmugDNA.11196.1.S1_at_TPR
MmugDNA.11197.1.S1_at	212959_s_at	GNPTAB	N-acetylglucosamine-1-phosphate transferase, alpha and beta subunits	chr12q23.2	79158	---	---	---	---	MmugDNA.11197.1.S1_at_GNPTAB
MmugDNA.11198.1.S1_at	237451_x_at	---	---	---	---	---	---	---	---	MmugDNA.11198.1.S1_at_---
MmugDNA.11199.1.S1_at	215883_at	CTNNA1	Catenin (cadherin-associated protein), alpha 1, 102kDa	chr5q31	1495	---	---	---	---	MmugDNA.11199.1.S1_at_CTNNA1
MmugDNA.11.1.S1_at	222911_s_at	CXorf36	chromosome X open reading frame 36	chrXp11.3	79742	LOC709814	similar to Protein CXorf36 precursor	---	709814	MmugDNA.11.1.S1_at_CXorf36
MmugDNA.11200.1.S1_at	211659_at	GPR135	G protein-coupled receptor 135	chr14q23.1	64582	GPR135	G protein-coupled receptor 135	---	701843	MmugDNA.11200.1.S1_at_GPR135
MmugDNA.11201.1.S1_at	243663_at	---	---	---	---	---	---	---	---	MmugDNA.11201.1.S1_at_---
MmugDNA.1120.1.S1_at	201843_s_at	EFEMP1	EGF-containing fibulin-like extracellular matrix protein 1	chr2p16	2202	LOC718984	similar to EGF-containing fibulin-like extracellular matrix protein 1 precursor	---	718984	MmugDNA.1120.1.S1_at_EFEMP1
MmugDNA.11202.1.S1_at	1561880_a_at	SIGLEC16	sialic acid binding Ig-like lectin 16 (gene/pseudogene)	chr19q13.33	400709	---	---	---	---	MmugDNA.11202.1.S1_at_SIGLEC16
MmugDNA.11204.1.S1_at	213547_at	CAND2	cullin-associated and neddylation-dissociated 2 (putative)	chr3p25.1	23066	CAND2	cullin-associated and neddylation-dissociated 2 (putative)	---	694326	MmugDNA.11204.1.S1_at_CAND2
MmugDNA.11205.1.S1_at	224517_at	POLR2J4	polymerase (RNA) II (DNA directed) polypeptide J4, pseudogene	chr7p13	84820	---	---	---	---	MmugDNA.11205.1.S1_at_POLR2J4
MmugDNA.11206.1.S1_at	NA	NA	NA	NA	NA	NA	NA	NA	NA	MmugDNA.11206.1.S1_at_NA
MmugDNA.11207.1.S1_at	224518_s_at	ZNF559	zinc finger protein 559	chr19p13.2	84527	LOC709955	similar to zinc finger protein 559	---	709955	MmugDNA.11207.1.S1_at_ZNF559
MmugDNA.11208.1.S1_at	NA	NA	NA	NA	NA	NA	NA	NA	NA	MmugDNA.11208.1.S1_at_NA
MmugDNA.11208.1.S1_s_at	201629_s_at	ACP1	acid phosphatase 1, soluble	chr2p25	52	LOC721397	similar to Low molecular weight phosphotyrosine protein phosphatase (LMW-PTP) (Low molecular we	---	721397	MmugDNA.11208.1.S1_s_at_ACP1
MmugDNA.11209.1.S1_at	224519_at	LOC100132167 /// RPL	similar to hCG1993567 /// ribosomal protein L7a pseudogene 45	chr9p11.2 /// chr9p12	100132167 /// 1001326	LOC696077	hypothetical protein LOC696077	---	696077	MmugDNA.11209.1.S1_at_LOC100132
MmugDNA.11210.1.S1_s_at	211066_x_at	PCDHGA1 /// PCDHGA	protocadherin gamma subfamily A, 1 /// protocadherin gamma subfamily A, 10 /// protocadherin gamn	chr5q31	26025 /// 5098 /// 5609	LOC702071	Similar to protocadherin gamma subfamily A, 12 isoform 2 precursor	---	702071	MmugDNA.11210.1.S1_s_at_PCDHGA1
MmugDNA.11211.1.S1_at	229371_at	---	---	---	---	---	---	---	---	MmugDNA.11211.1.S1_at_---
MmugDNA.1121.1.S1_at	1552913_at	MAGEB18	melanoma antigen family B, 18	chrXp21.3	286514	LOC700524	similar to melanoma antigen family B, 18	---	700524	MmugDNA.1121.1.S1_at_MAGEB18
MmugDNA.11212.1.S1_s_at	215718_s_at	PHF3	PHD finger protein 3	chr6q12	23469	PHF3	PHD finger protein 3	---	716282	MmugDNA.11212.1.S1_s_at_PHF3
MmugDNA.11213.1.S1_at	1556800_a_at	---	---	---	---	---	---	---	---	MmugDNA.11213.1.S1_at_---
MmugDNA.11214.1.S1_at	210681_s_at	USP15	ubiquitin specific peptidase 15	chr12q14	9958	USP15	ubiquitin specific peptidase 15	---	716793	MmugDNA.11214.1.S1_at_USP15
MmugDNA.11215.1.S1_at	NA	NA	NA	NA	NA	NA	NA	NA	NA	MmugDNA.11215.1.S1_at_NA
MmugDNA.11216.1.S1_at	224619_at	CASC4	cancer susceptibility candidate 4	chr15q15.3	113201	---	---	---	---	MmugDNA.11216.1.S1_at_CASC4
MmugDNA.11217.1.S1_at	223032_x_at	PRELID1	PRELI domain containing 1	chr5q35.3	27166	PRELID1	PRELI domain containing 1	---	699254	MmugDNA.11217.1.S1_at_PRELID1
MmugDNA.11218.1.S1_at	221965_at	MPHOSPH9	M-phase phosphoprotein 9	chr12q24.31	10198	---	---	---	---	MmugDNA.11218.1.S1_at_MPHOSPH9
MmugDNA.11219.1.S1_at	222391_at	TMEM30A	transmembrane protein 30A	chr6q14.1	55754	LOC717879	hypothetical protein LOC717879	---	717879	MmugDNA.11219.1.S1_at_TMEM30A
MmugDNA.112.1.S1_at	1554233_at	C1QTNF9	C1q and tumor necrosis factor related protein 9	chr13q12.12	338872	C1QTNF9	C1q and tumor necrosis factor related protein 9	---	707884	MmugDNA.112.1.S1_at_C1QTNF9
MmugDNA.11220.1.S1_at	232797_at	---	---	---	---	---	---	---	---	MmugDNA.11220.1.S1_at_---
MmugDNA.11221.1.S1_s_at	1554411_at	CTNNB1	catenin (cadherin-associated protein), beta 1, 88kDa	chr3p21	1499	---	---	---	---	MmugDNA.11221.1.S1_s_at_CTNNB1
MmugDNA.1122.1.S1_at	NA	NA	NA	NA	NA	NA	NA	NA	NA	MmugDNA.1122.1.S1_at_NA
MmugDNA.1122.1.S1_s_at	206399_x_at	CACNA1A	calcium channel, voltage-dependent, P/Q type, alpha 1A subunit	chr19p13.2-p13.1	773	LOC720692	similar to calcium channel, alpha 1A subunit isoform 2	---	720692	MmugDNA.1122.1.S1_s_at_CACNA1A
MmugDNA.11222.1.S1_at	224520_s_at	BEST3	bestrophin 3	chr12q14.2-q15	144453	---	---	---	---	MmugDNA.11222.1.S1_at_BEST3
MmugDNA.11223.1.S1_at	221714_s_at	LOC730092	RRN3 RNA polymerase I transcription factor homolog (S. cerevisiae) pseudogene	chr16p12.1	730092	---	---	---	---	MmugDNA.11223.1.S1_at_LOC730092
MmugDNA.11224.1.S1_at	224521_s_at	CCDC77	coiled-coil domain containing 77	chr12p13.33	84318	LOC721604	hypothetical protein LOC721604	---	721604	MmugDNA.11224.1.S1_at_CCDC77
MmugDNA.11225.1.S1_s_at	211126_s_at	CSRP2	cysteine and glycine-rich protein 2	chr12q21.1	1466	LOC712494	similar to Cysteine and glycine-rich protein 2 (Cysteine-rich protein 2) (CRP2) (Smooth muscle cell LII	---	712494	MmugDNA.11225.1.S1_s_at_CSRP2
MmugDNA.11226.1.S1_at	244410_at	PKHD1	polycystic kidney and hepatic disease 1 (autosomal recessive)	chr6p12.2	5314	---	---	---	---	MmugDNA.11226.1.S1_at_PKHD1
MmugDNA.11227.1.S1_at	244051_at	---	---	---	---	---	---	---	---	MmugDNA.11227.1.S1_at_---
MmugDNA.11228.1.S1_at	231999_at	ANKRD11	ankyrin repeat domain 11	chr16q24.3	29123	ANKRD11	ankyrin repeat domain 11	---	698722	MmugDNA.11228.1.S1_at_ANKRD11
MmugDNA.11229.1.S1_at	241903_at	---	---	---	---	---	---	---	---	MmugDNA.11229.1.S1_at_---
MmugDNA.11230.1.S1_at	239004_at	SQSTM1	Sequestosome 1	chr5q35	8878	---	---	---	---	MmugDNA.11230.1.S1_at_SQSTM1
MmugDNA.11231.1.S1_at	243783_at	---	---	---	---	---	---	---	---	MmugDNA.11231.1.S1_at_---
MmugDNA.1123.1.S1_at	233531_at	SLC46A1	Solute carrier family 46 (folate transporter), member 1	chr17q11.2	113235	LOC708727	similar to heme carrier protein 1	---	708727	MmugDNA.1123.1.S1_at_SLC46A1
MmugDNA.11232.1.S1_at	217254_s_at	EPO	erythropoietin	chr7q22	2056	EPO	erythropoietin	---	719294	MmugDNA.11232.1.S1_at_EPO
MmugDNA.11233.1.S1_at	242605_at	---	---	---	---	---	---	---	---	MmugDNA.11233.1.S1_at_---
MmugDNA.11234.1.S1_at	1554268_at	MORN1	MORN repeat containing 1	chr1p36.33-p36.32	79906	MORN1	MORN repeat containing 1	---	694285	MmugDNA.11234.1.S1_at_MORN1
MmugDNA.11235.1.S1_at	1569729_a_at	ASZ1	ankyrin repeat, SAM and basic leucine zipper domain containing 1	chr7q31.2	136991	ASZ1	ankyrin repeat, SAM and basic leucine zipper domain containing 1	---	705097	MmugDNA.11235.1.S1_at_ASZ1
MmugDNA.11236.1.S1_at	222382_x_at	NUP205	nucleoporin 205kDa	chr7q33	23165	NUP205	nucleoporin 205kDa	---	708216	MmugDNA.11236.1.S1_at_NUP205
MmugDNA.11237.1.S1_at	229009_at	SIX5	SIX homeobox 5	chr19q13.32	147912	LOC715373	similar to sine oculis homeobox homolog 5	---	715373	MmugDNA.11237.1.S1_at_SIX5
MmugDNA.11238.1.S1_at	226209_at	NDUFV3	NADH dehydrogenase (ubiquinone) flavoprotein 3, 10kDa	chr21q22.3	4731	LOC722400	similar to NADH-ubiquinone oxidoreductase flavoprotein 3 isoform a precursor	---	722400	MmugDNA.11238.1.S1_at_NDUFV3
MmugDNA.11239.1.S1_at	1561902_at	---	---	---	---	---	---	---	---	MmugDNA.11239.1.S1_at_---
MmugDNA.11240.1.S1_at	1561528_at	---	---	---	---	---	---	---	---	MmugDNA.11240.1.S1_at_---
MmugDNA.11241.1.S1_at	226983_at	ZNF777	zinc finger protein 777	chr7q36.1	27153	LOC711987	similar to zinc finger protein 629	---	711987	MmugDNA.11241.1.S1_at_ZNF777
MmugDNA.1124.1.S1_at	237499_at	---	---	---	---	---	---	---	---	MmugDNA.1124.1.S1_at_---
MmugDNA.11242.1.S1_at	1554757_a_at	INPP5A	inositol polyphosphate-5-phosphatase, 40kDa	chr10q26.3	3632	LOC719508	similar to Type I inositol-1,4,5-trisphosphate 5-phosphatase (5PTase)	---	719508	MmugDNA.11242.1.S1_at_INPP5A
MmugDNA.11243.1.S1_at	201224_s_at	SRRM1	serine/arginine repetitive matrix 1	chr1p36.11	10250	LOC694800	similar to Ser/Arg-related nuclear matrix protein	---	694800	MmugDNA.11243.1.S1_at_SRRM1
MmugDNA.11244.1.S1_at	217240_at	LOC654056 /// SIRPB1	similar to Signal-regulatory protein beta-1 precursor (SIRP-beta-1) /// signal-regulatory protein beta 1	chr20p13	10326 /// 654056	---	---	---	---	MmugDNA.11244.1.S1_at_LOC654056
MmugDNA.11245.1.S1_at	1564665_at	---	---	---	---	---	---	---	---	MmugDNA.11245.1.S1_at_---
MmugDNA.11246.1.S1_at	242248_at	PHKB	phosphorylase kinase, beta	chr16q12-q13	5257	---	---	---	---	MmugDNA.11246.1.S1_at_PHKB
MmugDNA.11247.1.S1_at	1560791_at	---	---	---	---	---	---	---	---	MmugDNA.11247.1.S1_at_---
MmugDNA.11248.1.S1_s_at	211067_s_at	GAS7	growth arrest-specific 7	chr17p13.1	8522	LOC717827	similar to Growth-arrest-specific protein 7 (GAS-7)	---	717827	MmugDNA.11248.1.S1_s_at_GAS7
MmugDNA.11249.1.S1_at	212188_at	KCTD12	potassium channel tetramerisation domain containing 12	chr13q22.3	115207	NGFR	Nerve growth factor receptor	---	574304	MmugDNA.11249.1.S1_at_KCTD12
MmugDNA.11250.1.S1_at	1558449_at	---	---	---	---	---	---	---	---	MmugDNA.11250.1.S1_at_---
MmugDNA.11251.1.S1_at	212192_at	KCTD12	potassium channel tetramerisation domain containing 12	chr13q22.3	115207	---	---	---	---	MmugDNA.11251.1.S1_at_KCTD12
MmugDNA.1125.1.S1_at	230715_at	ZNF518B	zinc finger protein 518B	chr4p16.1	85460	---	---	---	---	MmugDNA.1125.1.S1_at_ZNF518B
MmugDNA.11252.1.S1_at	NA	NA	NA	NA	NA	NA	NA	NA	NA	MmugDNA.11252.1.S1_at_NA
MmugDNA.11252.1.S1_s_at	209782_s_at	DBP	D site of albumin promoter (albumin D-box) binding protein	chr19q13.3	1628	CA11	carbonic anhydrase XI	---	718066	MmugDNA.11252.1.S1_s_at_DBP
MmugDNA.11253.1.S1_at	222057_at	NOL12	nucleolar protein 12	chr22q13.1	79159	---	---	---	---	MmugDNA.11253.1.S1_at_NOL12
MmugDNA.11254.1.S1_at	NA	NA	NA	NA	NA	NA	NA	NA	NA	MmugDNA.11254.1.S1_at_NA
MmugDNA.11255.1.S1_at	209051_s_at	RALGDS	ral guanine nucleotide dissociation stimulator	chr9q34.3	5900	RALGDS	ral guanine nucleotide dissociation stimulator	---	714436	MmugDNA.11255.1.S1_at_RALGDS
MmugDNA.11257.1.S1_s_at	202564_x_at	ARL2	ADP-ribosylation factor-like 2	chr11q13	402	LOC722013	similar to ADP-ribosylation factor-like protein 2	---	722013	MmugDNA.11257.1.S1_s_at_ARL2
MmugDNA.11258.1.S1_at	233614_at	---	---	---	---	---	---	---	---	MmugDNA.11258.1.S1_at_---

MmugDNA.11259.1.S1_at	214566_at	SMR3A /// SMR3B	submaxillary gland androgen regulated protein 3A /// submaxillary gland androgen regulated protein 3	chr4q13.3	10879 /// 26952	SMR3A	submaxillary gland androgen regulated protein 3 homolog A (mouse)	---		707477	MmugDNA.11259.1.S1_at_SMR3A /// S
MmugDNA.11260.1.S1_at	237747_at	---	---	---	---	---	---	---	---		MmugDNA.11260.1.S1_at_---
MmugDNA.11261.1.S1_at	236712_at	---	---	---	---	---	---	---	---		MmugDNA.11261.1.S1_at_---
MmugDNA.1126.1.S1_at	1557845_at	---	---	---	---	---	---	---	---		MmugDNA.1126.1.S1_at_---
MmugDNA.11262.1.S1_at	228615_at	LOC286161	hypothetical protein LOC286161	chr8p23.3	286161	---	---	---	---		MmugDNA.11262.1.S1_at_LOC286161
MmugDNA.11263.1.S1_at	231930_at	ELMOD1	ELMO/CED-12 domain containing 1	chr11q22.3	55531	ELMOD1	ELMO/CED-12 domain containing 1	---		707026	MmugDNA.11263.1.S1_at_ELMOD1
MmugDNA.11264.1.S1_at	230451_at	---	---	---	---	---	---	---	---		MmugDNA.11264.1.S1_at_---
MmugDNA.11265.1.S1_at	242630_at	---	---	---	---	---	---	---	---		MmugDNA.11265.1.S1_at_---
MmugDNA.11266.1.S1_s_at	211068_x_at	FAM21C /// FAM21D	family with sequence similarity 21, member C /// family with sequence similarity 21, member D	chr10q11.1 /// chr10q11.23	253725 /// 653450	LOC719082	hypothetical protein LOC719082	---		719082	MmugDNA.11266.1.S1_s_at_FAM21C /
MmugDNA.11267.1.S1_at	210384_at	PRMT2	protein arginine methyltransferase 2	chr21q22.3	3275	PRMT2	protein arginine methyltransferase 2	---		708006	MmugDNA.11267.1.S1_at_PRMT2
MmugDNA.11268.1.S1_at	234128_at	---	---	---	---	---	---	---	---		MmugDNA.11268.1.S1_at_---
MmugDNA.11269.1.S1_at	218222_x_at	ARNT	aryl hydrocarbon receptor nuclear translocator	chr1q21	405	ARNT	aryl hydrocarbon receptor nuclear translocator	---		715971	MmugDNA.11269.1.S1_at_ARNT
MmugDNA.11270.1.S1_at	212194_s_at	TM9SF4	transmembrane 9 superfamily protein member 4	chr20q11.21	9777	TM9SF4	transmembrane 9 superfamily protein member 4	---		712260	MmugDNA.11270.1.S1_at_TM9SF4
MmugDNA.11271.1.S1_at	239587_at	---	---	---	---	---	---	---	---		MmugDNA.11271.1.S1_at_---
MmugDNA.1127.1.S1_at	242944_at	FAM83A	family with sequence similarity 83, member A	chr8q24.13	84985	LOC704023	hypothetical protein LOC704023	---		704023	MmugDNA.1127.1.S1_at_FAM83A
MmugDNA.11272.1.S1_at	1565562_at	MAGIX	MAGI family member, X-linked	chrXp11.23	79917	---	---	---	---		MmugDNA.11272.1.S1_at_MAGIX
MmugDNA.11273.1.S1_at	212198_s_at	TM9SF4	transmembrane 9 superfamily protein member 4	chr20q11.21	9777	TM9SF4	transmembrane 9 superfamily protein member 4	---		712260	MmugDNA.11273.1.S1_at_TM9SF4
MmugDNA.11274.1.S1_at	227258_at	C10orf46	chromosome 10 open reading frame 46	chr10q26.11	143384	C9H10orf46	chromosome 10 open reading frame 46 ortholog	---		702200	MmugDNA.11274.1.S1_at_C10orf46
MmugDNA.11275.1.S1_at	206831_s_at	ARSD	arylsulfatase D	chrXp22.3	414	ARSD	similar to arylsulfatase D isoform a precursor	---		704070	MmugDNA.11275.1.S1_at_ARSD
MmugDNA.11276.1.S1_at	213364_s_at	SNX1	sorting nexin 1	chr15q22.31	6642	---	---	---	---		MmugDNA.11276.1.S1_at_SNX1
MmugDNA.11277.1.S1_at	227137_at	C10orf46	Chromosome 10 open reading frame 46	chr10q26.11	143384	C9H10orf46	chromosome 10 open reading frame 46 ortholog	---		702200	MmugDNA.11277.1.S1_at_C10orf46
MmugDNA.11278.1.S1_at	241543_at	---	---	---	---	---	---	---	---		MmugDNA.11278.1.S1_at_---
MmugDNA.11279.1.S1_at	225753_at	ZNF513	zinc finger protein 513	chr2p23.3	130557	---	---	---	---		MmugDNA.11279.1.S1_at_ZNF513
MmugDNA.11280.1.S1_at	1557793_at	FAM62C	family with sequence similarity 62 (C2 domain containing), member C	chr3q22.3	83850	LOC716304	similar to chr3 synaptotagmin	---		716304	MmugDNA.11280.1.S1_at_FAM62C
MmugDNA.11281.1.S1_at	215354_s_at	PELP1	proline, glutamate and leucine rich protein 1	chr17p13.2	27043	LOC709306	similar to proline-, glutamic acid-, leucine-rich protein 1	---		709306	MmugDNA.11281.1.S1_at_PELP1
MmugDNA.1128.1.S1_at	1558445_at	---	---	---	---	---	---	---	---		MmugDNA.1128.1.S1_at_---
MmugDNA.11282.1.S1_at	228453_at	KIAA1632	KIAA1632	chr18q12.3-q21.1	57724	LOC700766	similar to CG14299-PA, isoform A	---		700766	MmugDNA.11282.1.S1_at_KIAA1632
MmugDNA.11283.1.S1_at	1570345_at	C9orf27	Chromosome 9 open reading frame 27	chr9q31.3	58483	---	---	---	---		MmugDNA.11283.1.S1_at_C9orf27
MmugDNA.11284.1.S1_s_at	211069_s_at	SUMO1	SMT3 suppressor of mif two 3 homolog 1 (S. cerevisiae)	chr2q33	7341	---	---	---	---		MmugDNA.11284.1.S1_s_at_SUMO1
MmugDNA.11285.1.S1_at	237599_at	---	---	---	---	---	---	---	---		MmugDNA.11285.1.S1_at_---
MmugDNA.11286.1.S1_at	225025_at	IGSF8	immunoglobulin superfamily, member 8	chr1q23.1	93185	LOC720186	similar to immunoglobulin superfamily, member 8	---		720186	MmugDNA.11286.1.S1_at_IGSF8
MmugDNA.11287.1.S1_s_at	NA	NA	NA	NA	NA	NA	NA	NA	NA		MmugDNA.11287.1.S1_s_at_NA
MmugDNA.11288.1.S1_s_at	207675_x_at	ARTN	artemin	chr1p33-p32	9048	ARTN	artemin	---		701641	MmugDNA.11288.1.S1_s_at_ARTN
MmugDNA.11289.1.S1_at	240893_at	---	---	---	---	---	---	---	---		MmugDNA.11289.1.S1_at_---
MmugDNA.11290.1.S1_at	201101_s_at	BCLAF1	BCL2-associated transcription factor 1	chr6q22-q23	9774	LOC704656	Similar to linker for activation of T cells isoform b	---		704656	MmugDNA.11290.1.S1_at_BCLAF1
MmugDNA.11291.1.S1_s_at	201083_s_at	BCLAF1	BCL2-associated transcription factor 1	chr6q22-q23	9774	---	---	---	---		MmugDNA.11291.1.S1_s_at_BCLAF1
MmugDNA.11291.1.S1_x_at	NA	NA	NA	NA	NA	NA	NA	NA	NA		MmugDNA.11291.1.S1_x_at_NA
MmugDNA.1129.1.S1_at	239881_at	---	---	---	---	---	---	---	---		MmugDNA.1129.1.S1_at_---
MmugDNA.11293.1.S1_at	223493_at	FBXO4	F-box protein 4	chr5p12	26272	FBXO4	F-box protein 4	---		697007	MmugDNA.11293.1.S1_at_FBXO4
MmugDNA.11294.1.S1_at	222842_at	EIF2C4	eukaryotic translation initiation factor 2C, 4	chr1p34.3	192670	LOC713134	similar to eukaryotic translation initiation factor 2C, 4	---		713134	MmugDNA.11294.1.S1_at_EIF2C4
MmugDNA.11295.1.S1_at	1560014_s_at	PDXDC1	pyridoxal-dependent decarboxylase domain containing 1	chr16p13.11	23042	---	---	---	---		MmugDNA.11295.1.S1_at_PDXDC1
MmugDNA.11296.1.S1_at	227533_at	---	---	---	---	---	---	---	---		MmugDNA.11296.1.S1_at_---
MmugDNA.11297.1.S1_at	231143_at	LOC100129514	hypothetical protein LOC100129514	chr7q34	100129514	LOC693815	hypothetical protein LOC693815	---		693815	MmugDNA.11297.1.S1_at_LOC100129
MmugDNA.11298.1.S1_at	238122_at	RBM12B	RNA binding motif protein 12B	chr8q22.1	389677	LOC698382	similar to swan	---		698382	MmugDNA.11298.1.S1_at_RBM12B
MmugDNA.11299.1.S1_at	243438_at	PDE7B	phosphodiesterase 7B	chr6q23-q24	27115	---	---	---	---		MmugDNA.11299.1.S1_at_PDE7B
MmugDNA.11300.1.S1_at	239101_at	ITCH	itchy E3 ubiquitin protein ligase homolog (mouse)	chr20q11.22-q11.23	83737	---	---	---	---		MmugDNA.11300.1.S1_at_ITCH
MmugDNA.11301.1.S1_at	1552736_a_at	NETO1	neuropilin (NRP) and tolloid (TLL)-like 1	chr18q22-q23	81832	LOC722206	Similar to Protein FAM38B	---		722206	MmugDNA.11301.1.S1_at_NETO1
MmugDNA.1130.1.S1_at	216186_at	---	---	---	---	---	---	---	---		MmugDNA.1130.1.S1_at_---
MmugDNA.11302.1.S1_at	239429_at	---	---	---	---	---	---	---	---		MmugDNA.11302.1.S1_at_---
MmugDNA.11304.1.S1_at	229196_at	FAM111A	family with sequence similarity 111, member A	chr11q12.1	63901	LOC701070	hypothetical protein LOC701070	---		701070	MmugDNA.11304.1.S1_at_FAM111A
MmugDNA.11305.1.S1_at	241168_at	---	---	---	---	---	---	---	---		MmugDNA.11305.1.S1_at_---
MmugDNA.11306.1.S1_at	233456_at	---	---	---	---	---	---	---	---		MmugDNA.11306.1.S1_at_---
MmugDNA.11307.1.S1_at	224522_s_at	DCAKD	dephospho-CoA kinase domain containing	chr17q21.31	79877	LOC715841	similar to dephospho-CoA kinase domain containing	---		715841	MmugDNA.11307.1.S1_at_DCAKD
MmugDNA.11308.1.S1_at	1568821_at	TTC23	tetratricopeptide repeat domain 23	chr15q26.3	64927	---	---	---	---		MmugDNA.11308.1.S1_at_TTC23
MmugDNA.11309.1.S1_at	234775_at	OR51B5	olfactory receptor, family 51, subfamily B, member 5	chr11p15.4	282763	LOC696727	similar to olfactory receptor, family 51, subfamily B, member 5	---		696727	MmugDNA.11309.1.S1_at_OR51B5
MmugDNA.11310.1.S1_at	206130_s_at	ASGR2	asialoglycoprotein receptor 2	chr17p	433	LOC698405	similar to Asialoglycoprotein receptor 2 (ASGP-R 2) (ASGPR 2) (Hepatic lectin H2)	---		698405	MmugDNA.11310.1.S1_at_ASGR2
MmugDNA.11311.1.S1_at	204608_at	ASL	argininosuccinate lyase	chr7cen-q11.2	435	ASL	argininosuccinate lyase	---		697109	MmugDNA.11311.1.S1_at_ASL
MmugDNA.1131.1.S1_at	214826_at	PDE12	Phosphodiesterase 12	chr3p14.3	201626	PDE12	phosphodiesterase 12	---		703552	MmugDNA.1131.1.S1_at_PDE12
MmugDNA.11312.1.S1_at	1554618_at	AGFG2	ArfGAP with FG repeats 2	chr7q22.1	3268	---	---	---	---		MmugDNA.11312.1.S1_at_AGFG2
MmugDNA.11313.1.S1_at	231324_at	---	---	---	---	---	---	---	---		MmugDNA.11313.1.S1_at_---
MmugDNA.11314.1.S1_at	1556199_a_at	RGS9BP	regulator of G protein signaling 9 binding protein	chr19q13.11	388531	RGS9BP	regulator of G protein signaling 9 binding protein	---		702728	MmugDNA.11314.1.S1_at_RGS9BP
MmugDNA.11315.1.S1_at	213883_s_at	TM2D1	TM2 domain containing 1	chr1p31.3	83941	TM2D1	TM2 domain containing 1	---		694282	MmugDNA.11315.1.S1_at_TM2D1
MmugDNA.11316.1.S1_at	227040_at	NHLRC3	NHL repeat containing 3	chr13q13.3	387921	NHLRC3	NHL repeat containing 3	---		696852	MmugDNA.11316.1.S1_at_NHLRC3
MmugDNA.11317.1.S1_at	236296_x_at	C8orf58	chromosome 8 open reading frame 58	chr8p21	541565	LOC708961	hypothetical protein LOC708961	---		708961	MmugDNA.11317.1.S1_at_C8orf58
MmugDNA.11318.1.S1_at	209954_x_at	SS18	synovial sarcoma translocation, chromosome 18	chr18q11.2	6760	LOC709897	similar to SSXT protein (Synovial sarcoma, translocated to X chromosome) (SYT protein)	---		709897	MmugDNA.11318.1.S1_at_SS18
MmugDNA.11319.1.S1_at	225731_at	ANKRD50	ankyrin repeat domain 50	chr4q28.1	57182	LOC709327	similar to ankyrin repeat domain 50	---		709327	MmugDNA.11319.1.S1_at_ANKRD50
MmugDNA.113.1.S1_at	223261_at	POLK	polymerase (DNA directed) kappa	chr5q13	51426	POLK	polymerase (DNA directed) kappa	---		705713	MmugDNA.113.1.S1_at_POLK
MmugDNA.11320.1.S1_at	225735_at	ANKRD50	ankyrin repeat domain 50	chr4q28.1	57182	LOC709327	similar to ankyrin repeat domain 50	---		709327	MmugDNA.11320.1.S1_at_ANKRD50
MmugDNA.11321.1.S1_at	235346_at	FUNDC1	FUN14 domain containing 1	chrXp11.3	139341	LOC696024	similar to FUN14 domain containing 1	---		696024	MmugDNA.11321.1.S1_at_FUNDC1
MmugDNA.1132.1.S1_at	NA	NA	NA	NA	NA	NA	NA	NA	NA		MmugDNA.1132.1.S1_at_NA
MmugDNA.11322.1.S1_at	232866_at	ZSCAN18	zinc finger and SCAN domain containing 18	chr19q13.43	65982	---	---	---	---		MmugDNA.11322.1.S1_at_ZSCAN18
MmugDNA.11323.1.S1_at	237749_at	---	---	---	---	---	---	---	---		MmugDNA.11323.1.S1_at_---
MmugDNA.11324.1.S1_at	224523_s_at	C3orf26	chromosome 3 open reading frame 26	chr3q12.1	84319	LOC699717	hypothetical protein LOC699717	---		699717	MmugDNA.11324.1.S1_at_C3orf26
MmugDNA.11325.1.S1_at	1555256_at	EVC2	Ellis van Creveld syndrome 2	chr4p16.2-p16.1	132884	---	---	---	---		MmugDNA.11325.1.S1_at_EVC2
MmugDNA.11326.1.S1_at	242832_at	PER1	period homolog 1 (Drosophila)	chr17p13.1-p12	5187	---	---	---	---		MmugDNA.11326.1.S1_at_PER1
MmugDNA.11327.1.S1_s_at	225271_at	TMEM63B	transmembrane protein 63B	chr6p21.1	55362	LOC702797	similar to transmembrane protein 63B	---		702797	MmugDNA.11327.1.S1_s_at_TMEM63B
MmugDNA.11328.1.S1_at	202672_s_at	ATF3	activating transcription factor 3	chr1q32.3	467	ATF3	activating transcription factor 3	---		710832	MmugDNA.11328.1.S1_at_ATF3
MmugDNA.11329.1.S1_at	208442_s_at	ATM	ataxia telangiectasia mutated	chr11q22-q23	472	---	---	---	---		MmugDNA.11329.1.S1_at_ATM
MmugDNA.11330.1.S1_at	1560226_at	---	---	---	---	---	---	---	---		MmugDNA.11330.1.S1_at_---
MmugDNA.11331.1.S1_at	NA	NA	NA	NA	NA	NA	NA	NA	NA		MmugDNA.11331.1.S1_at_NA
MmugDNA.1133.1.S1_at	239188_at	PPP2R3C	protein phosphatase 2 (formerly 2A), regulatory subunit B", gamma	chr14q13.2	55012	---	---	---	---		MmugDNA.1133.1.S1_at_PPP2R3C
MmugDNA.11332.1.S1_at	238067_at	TBC1D8B	TBC1 domain family, member 8B (with GRAM domain)	chrXq22.3	54885	LOC701409	similar to TBC1 domain family, member 8	---		701409	MmugDNA.11332.1.S1_at_TBC1D8B
MmugDNA.11333.1.S1_at	241231_at	---	---	---	---	---	---	---	---		MmugDNA.11333.1.S1_at_---
MmugDNA.11334.1.S1_at	1568696_at	ARMETL1	arginine-rich, mutated in early stage tumors-like 1	chr10p13	441549	LOC697138	similar to arginine-rich, mutated in early stage tumors-like 1	---		697138	MmugDNA.11334.1.S1_at_ARMETL1
MmugDNA.11335.1.S1_at	243660_at	CHD9	Chromodomain helicase DNA binding protein 9	chr16q12.2	80205	---	---	---	---		MmugDNA.11335.1.S1_at_CHD9
MmugDNA.11336.1.S1_at	NA	NA	NA	NA	NA	NA	NA	NA	NA		MmugDNA.11336.1.S1_at_NA
MmugDNA.11337.1.S1_at	234959_at	---	---	---	---	---	---	---	---		MmugDNA.11337.1.S1_at_---
MmugDNA.11338.1.S1_at	1552811_at	WFIKKN1	WAP, follistatin/kazal, immunoglobulin, kunitz and netrin domain containing 1	chr16p13.3	117166	LOC722338	similar to WAP, follistatin/kazal, immunoglobulin, kunitz and netrin domain containing 1 precursor	---		722338	MmugDNA.11338.1.S1_at_WFIKKN1
MmugDNA.11339.1.S1_at	227256_at	USP31	ubiquitin specific peptidase 31	chr16p12.1	57478	---	---	---	---		MmugDNA.11339.1.S1_at_USP31
MmugDNA.11340.1.S1_at	215975_x_at	GK	glycerol kinase	chrXp21.3	2710	GK	glycerol kinase	---		705001	MmugDNA.11340.1.S1_at_GK
MmugDNA.11341.1.S1_s_at	212670_at	ELN	elastin	chr7q11.23	2006	---	---	---	---		MmugDNA.11341.1.S1_s_at_ELN
MmugDNA.1134.1.S1_at	233111_at	---	---	---	---	---	---	---	---		MmugDNA.1134.1.S1_at_---
MmugDNA.11342.1.S1_at	203296_s_at	ATP1A2	ATPase, Na+/K+ transporting, alpha 2 (+) polypeptide	chr1q21-q23	477	ATP1A2	ATPase, Na+/K+ transporting, alpha 2 (+) polypeptide	---		720191	MmugDNA.11342.1.S1_at_ATP1A2
MmugDNA.11343.1.S1_s_at	221545_x_at	MED16	mediator complex subunit 16	chr19p13.3	10025	LOC721141	similar to thyroid hormone receptor-associated protein 5	---		721141	MmugDNA.11343.1.S1_s_at_MED16
MmugDNA.11344.1.S1_at	221644_s_at	SLC45A2	solute carrier family 45, member 2	chr5p13.3	51151	SLC45A2	membrane-associated transporter protein	---		698246	MmugDNA.11344.1.S1_at_SLC45A2
MmugDNA.11345.1.S1_at	229005_at	MCTP2	multiple C2 domains, transmembrane 2	chr15q26.2	55784	MCTP2	multiple C2 domains, transmembrane 2	---		706949	MmugDNA.11345.1.S1_at_MCTP2
MmugDNA.11346.1.S1_at	228676_at	ORAOV1	oral cancer overexpressed 1	chr11q13.2	220064	---	---	---	---		MmugDNA.11346.1.S1_at_ORAOV1
MmugDNA.11347.1.S1_at	1554496_at	RAD51L1	RAD51-like 1 (S. cerevisiae)	chr14q23-q24.2	5890	RAD51L1	RAD51-like 1	---		711358	MmugDNA.11347.1.S1_at_RAD51L1
MmugDNA.11348.1.S1_at	229021_at	MCTP2	multiple C2 domains, transmembrane 2	chr15q26.2	55784	---	---	---	---		MmugDNA.11348.1.S1_at_MCTP2
MmugDNA.11349.1.S1_at	201243_s_at	ATP1B1	ATPase, Na+/K+ transporting, beta 1 polypeptide	chr1q24	481	ATP1B1	ATPase, Na+/K+ transporting, beta 1 polypeptide	---		700443	MmugDNA.11349.1.S1_at_ATP1B1
MmugDNA.11350.1.S1_at	204311_at	ATP1B2	ATPase, Na+/K+ transporting, beta 2 polypeptide	chr17p13.1	482	---	---	---	---		MmugDNA.11350.1.S1_at_ATP1B2
MmugDNA.11351.1.S1_at	207531_at	CRYGC	crystallin, gamma C	chr2q33-q35	1420	CRYGC	crystallin, gamma C	---		709553	MmugDNA.11351.1.S1_at_CRYGC
MmugDNA.1135.1.S1_at	241721_at	---	---	---	---	---	---	---	---		MmugDNA.1135.1.S1_at_---
MmugDNA.11352.1.S1_at	232366_at	KIAA0232	KIAA0232	chr4p16.1	9778	LOC710141	similar to Protein KIAA0232	---		710141	MmugDNA.11352.1.S1_at_KIAA0232
MmugDNA.11353.1.S1_at	222838_at	SLAMF7	SLAM family member 7	chr1q23.1-q24.1	57823	---	---	---	---		MmugDNA.11353.1.S1_at_SLAMF7
MmugDNA.11354.1.S1_at	229609_at	LOC728190	hypothetical LOC728190	chr10q23.2	728190	---	---	---	---		MmugDNA.11354.1.S1_at_LOC728190
MmugDNA.11355.1.S1_at	222956_at	FIGN	fidgetin	chr2q24.3	55137	---	---	---	---		MmugDNA.11355.1.S1_at_FIGN
MmugDNA.11356.1.S1_at	233946_at	SMU1	smu-1 suppressor of mec-8 and unc-52 homolog (C. elegans)	chr9p12	55234	---	---	---	---		MmugDNA.11356.1.S1_at_SMU1

MmugDNA.11357.1.S1_at	1570026_at	CPXM2	carboxypeptidase X (M14 family), member 2	chr10q26.13		119587	---	---	---	---		MmugDNA.11357.1.S1_at_CPXM2
MmugDNA.11358.1.S1_at	226752_at	FAM174A	family with sequence similarity 174, member A	chr5q21.1		345757	LOC702300	hypothetical protein LOC702300	---		702300	MmugDNA.11358.1.S1_at_FAM174A
MmugDNA.11359.1.S1_at	233773_at	---	---	---	---		---	---	---	---		MmugDNA.11359.1.S1_at_---
MmugDNA.11360.1.S1_at	205075_at	SERPINF2	serpin peptidase inhibitor, clade F (alpha-2 antiplasmin, pigment epithelium derived factor), member 2	chr17p13		5345	---	---	---	---		MmugDNA.11360.1.S1_at_SERPINF2
MmugDNA.11361.1.S1_at	205410_s_at	ATP2B4	ATPase, Ca++ transporting, plasma membrane 4	chr1q32.1		493	ATP2B4	ATPase, Ca++ transporting, plasma membrane 4	---		574317	MmugDNA.11361.1.S1_at_ATP2B4
MmugDNA.1136.1.S1_at	201166_s_at	PUM1	pumilio homolog 1 (Drosophila)	chr1p35.2		9698	PUM1	pumilio homolog 1	---		704848	MmugDNA.1136.1.S1_at_PUM1
MmugDNA.11362.1.S1_at	223786_at	CHST6	carbohydrate (N-acetylglucosamine 6-O) sulfotransferase 6	chr16q22		4166	---	---	---	---		MmugDNA.11362.1.S1_at_CHST6
MmugDNA.11363.1.S1_at	1561216_at	---	---	---	---		---	---	---	---		MmugDNA.11363.1.S1_at_---
MmugDNA.11364.1.S1_at	202580_x_at	FOXM1	forkhead box M1	chr12p13		2305	LOC708805	similar to forkhead box M1 isoform 3	---		708805	MmugDNA.11364.1.S1_at_FOXM1
MmugDNA.11365.1.S1_at	200871_s_at	PSAP	prosaposin	chr10q21-q22		5660	LOC722479	Similar to Nucleoside diphosphate kinase 3 (NDK 3) (NDP kinase 3) (Nucleoside diphosphate kinase	---		722479	MmugDNA.11365.1.S1_at_PSAP
MmugDNA.11366.1.S1_at	203029_s_at	PTPRN2	protein tyrosine phosphatase, receptor type, N polypeptide 2	chr7q36		5799	LOC694661	similar to protein tyrosine phosphatase, receptor type, N polypeptide 2 isoform 3 precursor	---		694661	MmugDNA.11366.1.S1_at_PTPRN2
MmugDNA.11367.1.S1_at	1561121_at	---	---	---	---		---	---	---	---		MmugDNA.11367.1.S1_at_---
MmugDNA.11368.1.S1_at	204869_at	PCSK2	proprotein convertase subtilisin/kexin type 2	chr20p11.2		5126	PCSK2	proprotein convertase subtilisin/kexin type 2	---		695730	MmugDNA.11368.1.S1_at_PCSK2
MmugDNA.11369.1.S1_at	233910_at	TMEFF2	transmembrane protein with EGF-like and two follistatin-like domains 2	chr2q32.3		23671	TMEFF2	transmembrane protein with EGF-like and two follistatin-like domains 2	---		694555	MmugDNA.11369.1.S1_at_TMEFF2
MmugDNA.11370.1.S1_at	228008_at	---	---	---	---		---	---	---	---		MmugDNA.11370.1.S1_at_---
MmugDNA.11371.1.S1_at	244128_x_at	GLIS1	GLIS family zinc finger 1	chr1p32.3		148979	---	---	---	---		MmugDNA.11371.1.S1_at_GLIS1
MmugDNA.1137.1.S1_at	1557406_s_at	LOC283697	hypothetical protein LOC283697	chr15q13.1		283697	---	---	---	---		MmugDNA.1137.1.S1_at_LOC283697
MmugDNA.11372.1.S1_at	236875_at	---	---	---	---		---	---	---	---		MmugDNA.11372.1.S1_at_---
MmugDNA.11373.1.S1_at	238357_at	RBM39	RNA binding motif protein 39	chr20q11.22		9584	---	---	---	---		MmugDNA.11373.1.S1_at_RBM39
MmugDNA.11374.1.S1_at	213620_s_at	ICAM2	intercellular adhesion molecule 2	chr17q23-q25		3384	ICAM2	intercellular adhesion molecule 2	---		574158	MmugDNA.11374.1.S1_at_ICAM2
MmugDNA.11374.1.S1_s_at	NA	NA	NA	NA	NA		NA	NA	NA	NA		MmugDNA.11374.1.S1_s_at_NA
MmugDNA.11375.1.S1_at	236600_at	SPG20	spastic paraplegia 20 (Troyer syndrome)	chr13q13.3		23111	SPG20	spastic paraplegia 20 (Troyer syndrome)	---		693663	MmugDNA.11375.1.S1_at_SPG20
MmugDNA.11376.1.S1_at	243026_x_at	XIAP	X-linked inhibitor of apoptosis	chrXq25		331	LOC697821	Similar to heme binding protein 1	---		697821	MmugDNA.11376.1.S1_at_XIAP
MmugDNA.11377.1.S1_s_at	NA	NA	NA	NA	NA		NA	NA	NA	NA		MmugDNA.11377.1.S1_s_at_NA
MmugDNA.11378.1.S1_at	212673_at	METAP1	methionyl aminopeptidase 1	chr4q23		23173	METAP1	methionyl aminopeptidase 1	---		710537	MmugDNA.11378.1.S1_at_METAP1
MmugDNA.11379.1.S1_at	202325_s_at	ATP5J	ATP synthase, H+ transporting, mitochondrial F0 complex, subunit F6	chr21q21.1		522	ATP5J	ATP synthase, H+ transporting, mitochondrial F0 complex, subunit F6	---		708411	MmugDNA.11379.1.S1_at_ATP5J
MmugDNA.11380.1.S1_at	NA	NA	NA	NA	NA		NA	NA	NA	NA		MmugDNA.11380.1.S1_at_NA
MmugDNA.11381.1.S1_at	1556609_at	LOC401098	hypothetical LOC401098	chr3q26.1		401098	LOC704710	hypothetical protein LOC704710	---		704710	MmugDNA.11381.1.S1_at_LOC401098
MmugDNA.1138.1.S1_at	1566150_at	CALML4	Calmodulin-like 4	chr15q23		91860	---	---	---	---		MmugDNA.1138.1.S1_at_CALML4
MmugDNA.11382.1.S1_at	NA	NA	NA	NA	NA		NA	NA	NA	NA		MmugDNA.11382.1.S1_at_NA
MmugDNA.11382.1.S1_s_at	213041_s_at	ATP5D	ATP synthase, H+ transporting, mitochondrial F1 complex, delta subunit	chr19p13.3		513	ATP5D	ATP synthase, H+ transporting, mitochondrial F1 complex, delta subunit	---		705944	MmugDNA.11382.1.S1_s_at_ATP5D
MmugDNA.11383.1.S1_at	1558502_s_at	DNM3	dynamin 3	chr1q24.3		26052	LOC705300	similar to dynamin 3	---		705300	MmugDNA.11383.1.S1_at_DNM3
MmugDNA.11384.1.S1_at	234758_at	---	---	---	---		---	---	---	---		MmugDNA.11384.1.S1_at_---
MmugDNA.11385.1.S1_at	207026_s_at	ATP2B3	ATPase, Ca++ transporting, plasma membrane 3	chrXq28		492	LOC695803	similar to plasma membrane calcium ATPase 3 isoform 3b	---		695803	MmugDNA.11385.1.S1_at_ATP2B3
MmugDNA.11386.1.S1_at	224900_at	ANKFY1	ankyrin repeat and FYVE domain containing 1	chr17p13.3		51479	ANKFY1	similar to ankyrin repeat and FYVE domain containing 1 isoform 1	---		708297	MmugDNA.11386.1.S1_at_ANKFY1
MmugDNA.11387.1.S1_at	214524_at	GHRH	growth hormone releasing hormone	chr20q11.2		2691	GHRH	growth hormone releasing hormone	---		701284	MmugDNA.11387.1.S1_at_GHRH
MmugDNA.11388.1.S1_at	212026_s_at	EXOC7	exocyst complex component 7	chr17q25.1		23265	---	---	---	---		MmugDNA.11388.1.S1_at_EXOC7
MmugDNA.11389.1.S1_at	205627_at	CDA	cytidine deaminase	chr1p36.2-p35		978	CDA	cytidine deaminase	---		705913	MmugDNA.11389.1.S1_at_CDA
MmugDNA.11390.1.S1_at	244620_at	LOC100128590	hypothetical protein LOC100128590	chr2p22.1		100128590	---	---	---	---		MmugDNA.11390.1.S1_at_LOC100128
MmugDNA.11391.1.S1_at	212034_s_at	EXOC7	exocyst complex component 7	chr17q25.1		23265	---	---	---	---		MmugDNA.11391.1.S1_at_EXOC7
MmugDNA.1139.1.S1_at	220483_s_at	RNF19A	ring finger protein 19A	chr8q22		25897	LOC708264	Similar to 60S ribosomal protein L32	---		708264	MmugDNA.1139.1.S1_at_RNF19A
MmugDNA.11392.1.S1_at	244494_at	ZDHHC1	Zinc finger, DHHC-type containing 1	chr16q22.1		29800	---	---	---	---		MmugDNA.11392.1.S1_at_ZDHHC1
MmugDNA.11393.1.S1_at	232478_at	---	---	---	---		---	---	---	---		MmugDNA.11393.1.S1_at_---
MmugDNA.11394.1.S1_at	232799_at	---	---	---	---		---	---	---	---		MmugDNA.11394.1.S1_at_---
MmugDNA.11395.1.S1_at	232628_at	---	---	---	---		---	---	---	---		MmugDNA.11395.1.S1_at_---
MmugDNA.11396.1.S1_s_at	235912_at	---	---	---	---		---	---	---	---		MmugDNA.11396.1.S1_s_at_---
MmugDNA.11397.1.S1_at	214921_at	KCNMA1	potassium large conductance calcium-activated channel, subfamily M, alpha member 1	chr10q22.3		3778	KCNMA1	potassium large conductance calcium-activated channel, subfamily M, alpha member 1	---		574103	MmugDNA.11397.1.S1_at_KCNMA1
MmugDNA.11398.1.S1_s_at	221677_s_at	DONSON	downstream neighbor of SON	chr21q22.1		29980	DONSON	downstream neighbor of SON	---		698853	MmugDNA.11398.1.S1_s_at_DONSON
MmugDNA.11399.1.S1_at	1553725_s_at	ZNF644	zinc finger protein 644	chr1p22.2		84146	LOC697066	similar to zinc finger protein 644 isoform 1	---		697066	MmugDNA.11399.1.S1_at_ZNF644
MmugDNA.11400.1.S1_at	232975_at	HCG18	HLA complex group 18	chr6p21.3		414777	---	---	---	---		MmugDNA.11400.1.S1_at_HCG18
MmugDNA.11401.1.S1_at	233064_at	ZFR2	zinc finger RNA binding protein 2	chr19p13.3		23217	---	---	---	---		MmugDNA.11401.1.S1_at_ZFR2
MmugDNA.1140.1.S1_at	1561611_at	---	---	---	---		---	---	---	---		MmugDNA.1140.1.S1_at_---
MmugDNA.11402.1.S1_at	NA	NA	NA	NA	NA		NA	NA	NA	NA		MmugDNA.11402.1.S1_at_NA
MmugDNA.11403.1.S1_at	230457_at	C6orf35	chromosome 6 open reading frame 35	chr6q25.3		729515	---	---	---	---		MmugDNA.11403.1.S1_at_C6orf35
MmugDNA.11404.1.S1_at	1554069_at	EPHA8	EPH receptor A8	chr1p36.12		2046	LOC708378	similar to EPH receptor A8 isoform 1 precursor	---		708378	MmugDNA.11404.1.S1_at_EPHA8
MmugDNA.11405.1.S1_s_at	202884_s_at	PPP2R1B	protein phosphatase 2 (formerly 2A), regulatory subunit A, beta isoform	chr11q23.2		5519	LOC710387	similar to beta isoform of regulatory subunit A, protein phosphatase 2 isoform b	---		710387	MmugDNA.11405.1.S1_s_at_PPP2R1B
MmugDNA.11406.1.S1_at	204944_at	PTPRG	protein tyrosine phosphatase, receptor type, G	chr3p21-p14		5793	LOC703937	similar to protein tyrosine phosphatase, receptor type, G precursor	---		703937	MmugDNA.11406.1.S1_at_PTPRG
MmugDNA.11407.1.S1_at	242048_at	PFDN6	prefoldin subunit 6	chr6p21.3		10471	---	---	---	---		MmugDNA.11407.1.S1_at_PFDN6
MmugDNA.11408.1.S1_at	224543_at	SVEP1	sushi, von Willebrand factor type A, EGF and pentraxin domain containing 1	chr9q32		79987	LOC709478	similar to sushi, von Willebrand factor type A, EGF and pentraxin domain containing 1	---		709478	MmugDNA.11408.1.S1_at_SVEP1
MmugDNA.11409.1.S1_at	237569_at	---	---	---	---		---	---	---	---		MmugDNA.11409.1.S1_at_---
MmugDNA.11410.1.S1_at	222389_s_at	WAC	WW domain containing adaptor with coiled-coil	---		51322	LOC716453	Similar to Protein C1orf43 (Hepatitis C virus NS5A-transactivated protein 4) (Protein NICE-3) (S863-3	---		716453	MmugDNA.11410.1.S1_at_WAC
MmugDNA.11411.1.S1_at	228309_at	---	---	---	---		---	---	---	---		MmugDNA.11411.1.S1_at_---
MmugDNA.1141.1.S1_at	236865_at	---	---	---	---		---	---	---	---		MmugDNA.1141.1.S1_at_---
MmugDNA.11412.1.S1_at	222390_at	WAC	WW domain containing adaptor with coiled-coil	---		51322	---	---	---	---		MmugDNA.11412.1.S1_at_WAC
MmugDNA.11413.1.S1_at	214777_at	IGKV4-1	immunoglobulin kappa variable 4-1	chr2p12		28908	IGKV4-1	similar to Ig kappa chain V-IV region precursor	---		700696	MmugDNA.11413.1.S1_at_IGKV4-1
MmugDNA.11413.1.S1_s_at	NA	NA	NA	NA	NA		NA	NA	NA	NA		MmugDNA.11413.1.S1_s_at_NA
MmugDNA.11414.1.S1_at	232715_at	---	---	---	---		---	---	---	---		MmugDNA.11414.1.S1_at_---
MmugDNA.11415.1.S1_at	233575_s_at	TLE4	transducin-like enhancer of split 4 (E(sp1) homolog, Drosophila)	chr9q21.31		7091	TLE4	transducin-like enhancer of split 4 (E(sp1) homolog, Drosophila)	---		706619	MmugDNA.11415.1.S1_at_TLE4
MmugDNA.11415.1.S1_s_at	216997_x_at	TLE4	transducin-like enhancer of split 4 (E(sp1) homolog, Drosophila)	chr9q21.31		7091	TLE4	transducin-like enhancer of split 4 (E(sp1) homolog, Drosophila)	---		706619	MmugDNA.11415.1.S1_s_at_TLE4
MmugDNA.11416.1.S1_at	214589_at	FGF12	fibroblast growth factor 12	chr3q28		2257	LOC705813	similar to Fibroblast growth factor 12 (FGF-12) (Fibroblast growth factor homologous factor 1) (FHF-1	---		705813	MmugDNA.11416.1.S1_at_FGF12
MmugDNA.11417.1.S1_at	228141_at	GPX8	glutathione peroxidase 8 (putative)	chr5q11.2		493869	GPX8	glutathione peroxidase 8 (putative)	---		705965	MmugDNA.11417.1.S1_at_GPX8
MmugDNA.11418.1.S1_at	1562512_at	SMPDL3A	Sphingomyelin phosphodiesterase, acid-like 3A	chr6q22.31		10924	---	---	---	---		MmugDNA.11418.1.S1_at_SMPDL3A
MmugDNA.11419.1.S1_at	55081_at	MICALL1	MICAL-like 1	chr22q13.1-q13.2		85377	MICAL-L1	molecule interacting with Rab13	---		702213	MmugDNA.11419.1.S1_at_MICALL1
MmugDNA.114.1.S1_at	1561368_at	---	---	---	---		LOC705029	hypothetical protein LOC705029	---		705029	MmugDNA.114.1.S1_at_---
MmugDNA.11420.1.S1_at	244527_at	TRIO	Triple functional domain (PTPRF interacting)	chr5p15.2		7204	---	---	---	---		MmugDNA.11420.1.S1_at_TRIO
MmugDNA.11421.1.S1_at	227781_x_at	FAM57B	family with sequence similarity 57, member B	chr16p11.2		83723	FAM57B	similar to Protein FAM57B	---		708210	MmugDNA.11421.1.S1_at_FAM57B
MmugDNA.1142.1.S1_at	223422_s_at	ARHGAP24	Rho GTPase activating protein 24	chr4q21.23-q21.3		83478	NUCKS1	Nuclear casein kinase and cyclin-dependent kinase substrate 1	---		697298	MmugDNA.1142.1.S1_at_ARHGAP24
MmugDNA.11422.1.S1_at	216503_s_at	MLLT10	myeloid/lymphoid or mixed-lineage leukemia (trithorax homolog, Drosophila); translocated to, 10	chr10p12		8028	LOC709083	similar to myeloid/lymphoid or mixed lineage-leukemia translocation to 10 homolog	---		709083	MmugDNA.11422.1.S1_at_MLLT10
MmugDNA.11423.1.S1_at	1561691_at	LOC285735	hypothetical LOC285735	chr6q23.2		285735	LOC708124	hypothetical protein LOC708124	---		708124	MmugDNA.11423.1.S1_at_LOC285735
MmugDNA.11424.1.S1_at	213438_at	NFASC	neurofascin homolog (chicken)	chr1q32.1		23114	---	---	---	---		MmugDNA.11424.1.S1_at_NFASC
MmugDNA.11425.1.S1_at	232270_at	C9orf3	Chromosome 9 open reading frame 3	chr9q22.32		84909	---	---	---	---		MmugDNA.11425.1.S1_at_C9orf3
MmugDNA.11426.1.S1_at	201884_at	CEACAM5	carcinoembryonic antigen-related cell adhesion molecule 5	chr19q13.1-q13.2		1048	---	---	---	---		MmugDNA.11426.1.S1_at_CEACAM5
MmugDNA.11427.1.S1_at	207166_at	GNGT1	guanine nucleotide binding protein (G protein), gamma transducing activity polypeptide 1	chr7q21.3		2792	GNGT1	guanine nucleotide binding protein (G protein), gamma transducing activity polypeptide 1	---		700724	MmugDNA.11427.1.S1_at_GNGT1
MmugDNA.11428.1.S1_at	227610_at	TSPAN11	tetraspanin 11	chr12p11.21		441631	---	---	---	---		MmugDNA.11428.1.S1_at_TSPAN11
MmugDNA.11429.1.S1_at	228360_at	LYPD6B	LY6/PLAUR domain containing 6B	chr2q23.2		130576	LOC713216	Similar to minor histocompatibility antigen 13 isoform 3	---		713216	MmugDNA.11429.1.S1_at_LYPD6B
MmugDNA.11430.1.S1_at	226875_at	DOCK11	dedicator of cytokinesis 11	chrXq24		139818	LOC710690	similar to dedicator of cytokinesis 11	---		710690	MmugDNA.11430.1.S1_at_DOCK11
MmugDNA.11431.1.S1_at	215715_at	SLC6A2	solute carrier family 6 (neurotransmitter transporter, noradrenalin), member 2	chr16q12.2		6530	---	---	---	---		MmugDNA.11431.1.S1_at_SLC6A2
MmugDNA.1143.1.S1_at	213463_s_at	FAM149B1	family with sequence similarity 149, member B1	chr10q22.2		317662	---	---	---	---		MmugDNA.1143.1.S1_at_FAM149B1
MmugDNA.11432.1.S1_at	226777_at	---	---	---	---		ADAM12	ADAM metallopeptidase domain 12	---		696405	MmugDNA.11432.1.S1_at_---
MmugDNA.11433.1.S1_at	210704_at	FEZ2	fasciculation and elongation protein zeta 2 (zygin II)	chr2p21		9637	---	---	---	---		MmugDNA.11433.1.S1_at_FEZ2
MmugDNA.11434.1.S1_at	225719_s_at	MRPL55	mitochondrial ribosomal protein L55	chr1q42.13		128308	MRPL55	mitochondrial ribosomal protein L55	---		695962	MmugDNA.11434.1.S1_at_MRPL55
MmugDNA.11435.1.S1_at	237181_at	---	---	---	---		---	---	---	---		MmugDNA.11435.1.S1_at_---
MmugDNA.11436.1.S1_at	213027_at	TROVE2	TROVE domain family, member 2	chr1q31		6738	---	---	---	---		MmugDNA.11436.1.S1_at_TROVE2
MmugDNA.11437.1.S1_at	242667_at	---	---	---	---		---	---	---	---		MmugDNA.11437.1.S1_at_---
MmugDNA.11438.1.S1_at	213660_s_at	TOP3B	topoisomerase (DNA) III beta	chr22q11.22		8940	TOP3B	topoisomerase (DNA) III beta	---		698317	MmugDNA.11438.1.S1_at_TOP3B
MmugDNA.11439.1.S1_at	241962_at	---	---	---	---		---	---	---	---		MmugDNA.11439.1.S1_at_---
MmugDNA.11440.1.S1_at	231210_at	C11orf85	chromosome 11 open reading frame 85	chr11q13.1		283129	---	---	---	---		MmugDNA.11440.1.S1_at_C11orf85
MmugDNA.11441.1.S1_at	243840_at	---	---	---	---		---	---	---	---		MmugDNA.11441.1.S1_at_---
MmugDNA.1144.1.S1_at	213458_at	FAM149B1	family with sequence similarity 149, member B1	chr10q22.2		317662	---	---	---	---		MmugDNA.1144.1.S1_at_FAM149B1
MmugDNA.11442.1.S1_at	236639_at	---	---	---	---		---	---	---	---		MmugDNA.11442.1.S1_at_---
MmugDNA.11443.1.S1_at	1555153_s_at	FCHO2	FCH domain only 2	chr5q13.2		115548	---	---	---	---		MmugDNA.11443.1.S1_at_FCHO2
MmugDNA.11444.1.S1_at	232919_at	AFG3L2	AFG3 ATPase family gene 3-like 2 (yeast)	chr18p11		10939	---	---	---	---		MmugDNA.11444.1.S1_at_AFG3L2
MmugDNA.11445.1.S1_at	221751_at	SLC2A3P1	Solute carrier family 2 (facilitated glucose transporter), member 3 pseudogene 1	chr5q35.1		100128062	---	---	---	---		MmugDNA.11445.1.S1_at_SLC2A3P1
MmugDNA.11446.1.S1_at	229199_at	SCN9A	sodium channel, voltage-gated, type IX, alpha subunit	chr2q24		6335	---	---	---	---		MmugDNA.11446.1.S1_at_SCN9A
MmugDNA.11447.1.S1_at	229201_at	---	---	---	---		LOC715923	Similar to CD68 antigen	---		715923	MmugDNA.11447.1.S1_at_---
MmugDNA.11448.1.S1_at	242141_at	HDAC2	histone deacetylase 2	chr6q21		3066	---	---	---	---		MmugDNA.11448.1.S1_at_HDAC2
MmugDNA.11449.1.S1_at	230333_at	---	---	---	---		---	---	---	---		MmugDNA.11449.1.S1_at_---
MmugDNA.11450.1.S1_at	213067_at	MYH10	myosin, heavy chain 10, non-muscle	chr17p13		4628	MYH10	myosin, heavy chain 10, non-muscle	---		721979	MmugDNA.11450.1.S1_at_MYH10

MmugDNA.11451.1.S1_at	236289_at	---	---	---	---	---	---	---	---		MmugDNA.11451.1.S1_at_---
MmugDNA.1145.1.S1_at	NA	NA	NA	NA	NA	NA	NA	NA	NA		MmugDNA.1145.1.S1_at_NA
MmugDNA.1145.1.S1_x_at	238722_x_at	NAPEPLD	N-acyl phosphatidylethanolamine phospholipase D	chr7q22.1	222236	NAPEPLD	N-acyl phosphatidylethanolamine phospholipase D	---		695318	MmugDNA.1145.1.S1_x_at_NAPEPLD
MmugDNA.11452.1.S1_at	233367_at	---	---	---	---	---	---	---	---		MmugDNA.11452.1.S1_at_---
MmugDNA.11453.1.S1_at	241549_at	---	---	---	---	---	---	---	---		MmugDNA.11453.1.S1_at_---
MmugDNA.11454.1.S1_at	242805_at	---	---	---	---	---	---	---	---		MmugDNA.11454.1.S1_at_---
MmugDNA.11455.1.S1_s_at	1553486_a_at	C17orf78	chromosome 17 open reading frame 78	chr17q12	284099	---	---	---	---		MmugDNA.11455.1.S1_s_at_C17orf78
MmugDNA.11456.1.S1_at	218253_s_at	LGTN	ligatin	chr1q31-q32	1939	LGTN	ligatin	---		695274	MmugDNA.11456.1.S1_at_LGTN
MmugDNA.11457.1.S1_at	229942_at	BNC2	basonuclin 2	chr9p22.3-p22.2	54796	---	---	---	---		MmugDNA.11457.1.S1_at_BNC2
MmugDNA.11458.1.S1_at	239229_at	---	---	---	---	---	---	---	---		MmugDNA.11458.1.S1_at_---
MmugDNA.11459.1.S1_at	239291_at	---	---	---	---	---	---	---	---		MmugDNA.11459.1.S1_at_---
MmugDNA.11460.1.S1_at	215268_at	KIAA0754	hypothetical LOC643314	chr1p34.2	643314	---	---	---	---		MmugDNA.11460.1.S1_at_KIAA0754
MmugDNA.11461.1.S1_at	208513_at	FOXB1	forkhead box B1	chr15q21-q26	27023	FOXB1	similar to Forkhead box protein B1 (Transcription factor FKH-5)	---		703577	MmugDNA.11461.1.S1_at_FOXB1
MmugDNA.1146.1.S1_at	225528_at	IPO8	importin 8	chr12p11.21	10526	LOC693514	similar to importin 8	---		693514	MmugDNA.1146.1.S1_at_IPO8
MmugDNA.11462.1.S1_at	52169_at	STRADA	STE20-related kinase adaptor alpha	chr17q23.3	92335	LOC717927	similar to protein kinase LYK5 isoform 1	---		717927	MmugDNA.11462.1.S1_at_STRADA
MmugDNA.11463.1.S1_at	220621_at	FOXE3	forkhead box E3	chr1p32	2301	---	---	---	---		MmugDNA.11463.1.S1_at_FOXE3
MmugDNA.11464.1.S1_at	1555688_s_at	SLA2	Src-like-adaptor 2	chr20q11.23	84174	SLA2	Src-like-adaptor 2	---		702574	MmugDNA.11464.1.S1_at_SLA2
MmugDNA.11465.1.S1_at	1552657_a_at	TXNDC2	thioredoxin domain containing 2 (spermatozoa)	---	84203	HNRPA1	Similar to Heterogeneous nuclear ribonucleoprotein A1 (Helix-destabilizing protein) (Single-strand RN	---		709393	MmugDNA.11465.1.S1_at_TXNDC2
MmugDNA.11466.1.S1_at	231070_at	IYD	iodotyrosine deiodinase	chr6q25.1	389434	IYD	iodotyrosine deiodinase	---		705471	MmugDNA.11466.1.S1_at_IYD
MmugDNA.11467.1.S1_at	232754_at	---	---	---	---	---	---	---	---		MmugDNA.11467.1.S1_at_---
MmugDNA.11468.1.S1_at	232402_at	LOC642346	hypothetical LOC642346	chr15q25.2	642346	LOC721534	hypothetical protein LOC721534	---		721534	MmugDNA.11468.1.S1_at_LOC642346
MmugDNA.11469.1.S1_at	230128_at	IGL@	Immunoglobulin lambda locus	chr22q11.1-q11.2	3535	---	---	---	---		MmugDNA.11469.1.S1_at_IGL@
MmugDNA.11470.1.S1_at	NA	NA	NA	NA	NA	NA	NA	NA	NA		MmugDNA.11470.1.S1_at_NA
MmugDNA.11470.1.S1_s_at	213864_s_at	NAP1L1	nucleosome assembly protein 1-like 1	chr12q21.2	4673	PLK2	Polo-like kinase 2	---		708835	MmugDNA.11470.1.S1_s_at_NAP1L1
MmugDNA.11471.1.S1_at	227585_at	---	---	---	---	ATAD1	ATPase family, AAA domain containing 1	---		704809	MmugDNA.11471.1.S1_at_---
MmugDNA.1147.1.S1_at	229741_at	MAVS	mitochondrial antiviral signaling protein	chr20p13	57506	---	---	---	---		MmugDNA.1147.1.S1_at_MAVS
MmugDNA.11472.1.S1_at	241348_at	ZNF654	zinc finger protein 654	chr3p11.2	55279	LOC694002	hypothetical protein LOC694002	---		694002	MmugDNA.11472.1.S1_at_ZNF654
MmugDNA.11473.1.S1_at	236304_at	---	---	---	---	---	---	---	---		MmugDNA.11473.1.S1_at_---
MmugDNA.11474.1.S1_at	1566032_at	---	---	---	---	---	---	---	---		MmugDNA.11474.1.S1_at_---
MmugDNA.11475.1.S1_at	234686_at	SUGT1 /// SUGT1P	SGT1, suppressor of G2 allele of SKP1 (S. cerevisiae) /// suppressor of G2 allele of SKP1 pseudoge	chr13q14.3 /// chr9p13.3	10910 /// 441394	SUGT1	SGT1, suppressor of G2 allele of SKP1 (S. cerevisiae)	---		694046	MmugDNA.11475.1.S1_at_SUGT1 /// S
MmugDNA.11476.1.S1_at	238284_at	---	---	---	---	---	---	---	---		MmugDNA.11476.1.S1_at_---
MmugDNA.11477.1.S1_at	1554456_a_at	LINS1	lines homolog 1 (Drosophila)	chr15q26.3	55180	LOC693833	similar to Wins2 protein	---		693833	MmugDNA.11477.1.S1_at_LINS1
MmugDNA.11478.1.S1_at	233324_at	TRERF1	transcriptional regulating factor 1	chr6p21.1-p12.1	55809	LOC696006	similar to transcriptional regulating factor 1 isoform 1	---		696006	MmugDNA.11478.1.S1_at_TRERF1
MmugDNA.11479.1.S1_at	215663_at	MBNL1	muscleblind-like (Drosophila)	chr3q25	4154	---	---	---	---		MmugDNA.11479.1.S1_at_MBNL1
MmugDNA.11480.1.S1_at	230105_at	HOXB13	homeobox B13	chr17q21.2	10481	---	---	---	---		MmugDNA.11480.1.S1_at_HOXB13
MmugDNA.11481.1.S1_at	239729_at	---	---	---	---	---	---	---	---		MmugDNA.11481.1.S1_at_---
MmugDNA.1148.1.S1_at	222452_s_at	GPBP1L1	GC-rich promoter binding protein 1-like 1	chr1p34.1	60313	GPBP1L1	GC-rich promoter binding protein 1-like 1	---		706690	MmugDNA.1148.1.S1_at_GPBP1L1
MmugDNA.11482.1.S1_at	236411_at	---	---	---	---	---	---	---	---		MmugDNA.11482.1.S1_at_---
MmugDNA.11483.1.S1_at	1555349_a_at	ITGB2	integrin, beta 2 (complement component 3 receptor 3 and 4 subunit)	chr21q22.3	3689	ITGB2	integrin, beta 2	---		710577	MmugDNA.11483.1.S1_at_ITGB2
MmugDNA.11484.1.S1_at	243208_x_at	MGC33407	hypothetical protein MGC33407	chr19p13.2	284382	LOC708303	similar to Actin-like protein 7A (Actin-like-7-alpha) (Actin-like 7A) (T-actin-2) (Testis-specific actin-2)	---		708303	MmugDNA.11484.1.S1_at_MGC33407
MmugDNA.11485.1.S1_at	232865_at	AFF4	AF4/FMR2 family, member 4	chr5q31	27125	LOC709137	similar to ALL1 fused gene from 5q31	---		709137	MmugDNA.11485.1.S1_at_AFF4
MmugDNA.11486.1.S1_at	222957_at	NEU4	sialidase 4	chr2q37.3	129807	NEU4	sialidase 4	---		702264	MmugDNA.11486.1.S1_at_NEU4
MmugDNA.11487.1.S1_at	1558077_s_at	MDH1B	malate dehydrogenase 1B, NAD (soluble)	chr2q33.3	130752	LOC708188	similar to malate dehydrogenase 1B, NAD (soluble)	---		708188	MmugDNA.11487.1.S1_at_MDH1B
MmugDNA.11488.1.S1_at	240806_at	RPL15	Ribosomal protein L15	chr3p24.2	6138	---	---	---	---		MmugDNA.11488.1.S1_at_RPL15
MmugDNA.11489.1.S1_at	242426_at	NRG4	neuregulin 4	chr15q24.2	145957	NRG4	neuregulin 4	---		707378	MmugDNA.11489.1.S1_at_NRG4
MmugDNA.11490.1.S1_at	209827_s_at	IL16	interleukin 16 (lymphocyte chemoattractant factor)	chr15q26.3	3603	LOC574100	IL-16 precursor	---		574100	MmugDNA.11490.1.S1_at_IL16
MmugDNA.11491.1.S1_at	1569365_a_at	KIAA1656	KIAA1656 protein	---	85371	---	---	---	---		MmugDNA.11491.1.S1_at_KIAA1656
MmugDNA.1149.1.S1_at	209456_s_at	FBXW11	F-box and WD repeat domain containing 11	chr5q35.1	23291	LOC708990	similar to F-box and WD-40 domain protein 11	---		708990	MmugDNA.1149.1.S1_at_FBXW11
MmugDNA.11492.1.S1_at	1556393_at	---	---	---	---	---	---	---	---		MmugDNA.11492.1.S1_at_---
MmugDNA.11493.1.S1_at	1556350_a_at	EIF4A2	eukaryotic translation initiation factor 4A, isoform 2	chr3q28	1974	---	---	---	---		MmugDNA.11493.1.S1_at_EIF4A2
MmugDNA.11494.1.S1_at	237284_at	DNAJB8	DnaJ (Hsp40) homolog, subfamily B, member 8	chr3q21.3	165721	DNAJB8	DnaJ (Hsp40) homolog, subfamily B, member 8	---		705667	MmugDNA.11494.1.S1_at_DNAJB8
MmugDNA.11495.1.S1_at	233015_at	MBNL1	muscleblind-like (Drosophila)	chr3q25	4154	---	---	---	---		MmugDNA.11495.1.S1_at_MBNL1
MmugDNA.11496.1.S1_at	216893_s_at	COL4A3	collagen, type IV, alpha 3 (Goodpasture antigen)	chr2q36-q37	1285	COL4A3	collagen, type IV, alpha 3 (Goodpasture antigen)	---		712444	MmugDNA.11496.1.S1_at_COL4A3
MmugDNA.11497.1.S1_at	239911_at	ONECUT2	one cut homeobox 2	chr18q21.1-q21.2	9480	WDR18	WD repeat domain 18	---		704547	MmugDNA.11497.1.S1_at_ONECUT2
MmugDNA.11498.1.S1_at	238022_at	hCG_1815491	hCG1815491	chr16q12.2	643911	---	---	---	---		MmugDNA.11498.1.S1_at_hCG_18154
MmugDNA.11499.1.S1_at	236603_at	---	---	---	---	---	---	---	---		MmugDNA.11499.1.S1_at_---
MmugDNA.11500.1.S1_at	222947_at	---	---	---	---	---	---	---	---		MmugDNA.11500.1.S1_at_---
MmugDNA.11501.1.S1_at	215186_at	FLJ41278	hypothetical gene supported by AK123272	chr12q14.3	400046	---	---	---	---		MmugDNA.11501.1.S1_at_FLJ41278
MmugDNA.11502.1.S1_at	241971_at	---	---	---	---	---	---	---	---		MmugDNA.11502.1.S1_at_---
MmugDNA.11503.1.S1_at	1554584_at	LRRC48	leucine rich repeat containing 48	chr17p11.2	83450	---	---	---	---		MmugDNA.11503.1.S1_at_LRRC48
MmugDNA.11504.1.S1_at	232056_at	SCEL	sciellin	chr13q22	8796	SCEL	sciellin	---		696980	MmugDNA.11504.1.S1_at_SCEL
MmugDNA.11505.1.S1_at	242590_at	---	---	---	---	---	---	---	---		MmugDNA.11505.1.S1_at_---
MmugDNA.11506.1.S1_at	NA	NA	NA	NA	NA	NA	NA	NA	NA		MmugDNA.11506.1.S1_at_NA
MmugDNA.11507.1.S1_at	232362_at	CCDC18	coiled-coil domain containing 18	chr1p22.1	343099	LOC713445	similar to sarcoma antigen NY-SAR-41	---		713445	MmugDNA.11507.1.S1_at_CCDC18
MmugDNA.11508.1.S1_at	220048_at	EDAR	ectodysplasin A receptor	chr2q11-q13	10913	EDAR	ectodysplasin A receptor	---		695059	MmugDNA.11508.1.S1_at_EDAR
MmugDNA.11509.1.S1_at	220587_s_at	GBL	G protein beta subunit-like	chr16p13.3	64223	GBL	G protein beta subunit-like	---		695650	MmugDNA.11509.1.S1_at_GBL
MmugDNA.11510.1.S1_at	230071_at	SEPT11	septin 11	chr4q21.1	55752	SEPT11	septin 11	---		698867	MmugDNA.11510.1.S1_at_SEPT11
MmugDNA.11511.1.S1_s_at	222521_x_at	NDUFC2	NADH dehydrogenase (ubiquinone) 1, subcomplex unknown, 2, 14.5kDa	chr11q14.1	4718	---	---	---	---		MmugDNA.11511.1.S1_s_at_NDUFC2
MmugDNA.1151.1.S1_at	243097_x_at	---	---	---	---	---	---	---	---		MmugDNA.1151.1.S1_at_---
MmugDNA.11512.1.S1_at	242837_at	SFRS4	Splicing factor, arginine/serine-rich 4	chr1p35.3	6429	---	---	---	---		MmugDNA.11512.1.S1_at_SFRS4
MmugDNA.11513.1.S1_at	232544_at	---	---	---	---	---	---	---	---		MmugDNA.11513.1.S1_at_---
MmugDNA.11514.1.S1_at	1561895_at	---	---	---	---	---	---	---	---		MmugDNA.11514.1.S1_at_---
MmugDNA.11515.1.S1_at	205849_s_at	UQCRB	ubiquinol-cytochrome c reductase binding protein	chr8q22	7381	UQCRB	ubiquinol-cytochrome c reductase binding protein	---		701223	MmugDNA.11515.1.S1_at_UQCRB
MmugDNA.11516.1.S1_at	218299_at	C11orf24	chromosome 11 open reading frame 24	chr11q13	53838	LOC721633	hypothetical protein LOC721633	---		721633	MmugDNA.11516.1.S1_at_C11orf24
MmugDNA.11517.1.S1_at	233375_at	EFCAB2	EF-hand calcium binding domain 2	chr1q44	84288	---	---	---	---		MmugDNA.11517.1.S1_at_EFCAB2
MmugDNA.11518.1.S1_at	NA	NA	NA	NA	NA	NA	NA	NA	NA		MmugDNA.11518.1.S1_at_NA
MmugDNA.11518.1.S1_s_at	222281_s_at	---	---	---	---	---	---	---	---		MmugDNA.11518.1.S1_s_at_---
MmugDNA.11519.1.S1_at	226602_s_at	BCR /// FLJ42953 /// LC	breakpoint cluster region /// breakpoint cluster region pseudogene /// breakpoint cluster region pseud	chr22q11.21 /// chr22q11|22q11.	100133163 /// 400892 /	LOC709258	similar to breakpoint cluster region isoform 1	---		709258	MmugDNA.11519.1.S1_at_BCR /// FLJ4
MmugDNA.115.1.S1_at	241888_at	---	---	---	---	---	---	---	---		MmugDNA.115.1.S1_at_---
MmugDNA.11520.1.S1_at	219763_at	DENND1A	DENN/MADD domain containing 1A	chr9q33.2	57706	---	---	---	---		MmugDNA.11520.1.S1_at_DENND1A
MmugDNA.11521.1.S1_at	207757_at	ZFP2	zinc finger protein 2 homolog (mouse)	chr5q35.3	80108	LOC703114	similar to zinc finger protein 2 homolog	---		703114	MmugDNA.11521.1.S1_at_ZFP2
MmugDNA.1152.1.S1_at	221455_s_at	WNT3	wingless-type MMTV integration site family, member 3	chr17q21	7473	LOC716909	similar to wingless-type MMTV integration site family, member 3	---		716909	MmugDNA.1152.1.S1_at_WNT3
MmugDNA.11522.1.S1_at	236675_at	RPA1	replication protein A1, 70kDa	chr17p13.3	6117	---	---	---	---		MmugDNA.11522.1.S1_at_RPA1
MmugDNA.11523.1.S1_at	233453_at	---	---	---	---	---	---	---	---		MmugDNA.11523.1.S1_at_---
MmugDNA.11524.1.S1_at	1565867_a_at	LOC100131402	hypothetical protein LOC100131402	chr1q32.1	100131402	ZC3H11A	zinc finger CCCH-type containing 11A	---		701537	MmugDNA.11524.1.S1_at_LOC100131
MmugDNA.11525.1.S1_at	220721_at	ZNF614	zinc finger protein 614	chr19q13.33	80110	LOC720110	similar to zinc finger protein 614	---		720110	MmugDNA.11525.1.S1_at_ZNF614
MmugDNA.11526.1.S1_at	238128_at	TTMA	Two transmembrane domain family member A	chr18p11.31	645369	---	---	---	---		MmugDNA.11526.1.S1_at_TTMA
MmugDNA.11527.1.S1_at	209425_at	C1QTNF3	C1q and tumor necrosis factor related protein 3	chr5p13	114899	---	---	---	---		MmugDNA.11527.1.S1_at_C1QTNF3
MmugDNA.11528.1.S1_at	241434_at	---	---	---	---	IPP	intracisternal A particle-promoted polypeptide	---		706893	MmugDNA.11528.1.S1_at_---
MmugDNA.11529.1.S1_at	1552476_s_at	PLCD3	phospholipase C, delta 3	chr17q21.31	113026	LOC715924	similar to phospholipase C delta 3	---		715924	MmugDNA.11529.1.S1_at_PLCD3
MmugDNA.11530.1.S1_at	229557_at	MEG3	maternally expressed 3 (non-protein coding)	chr14q32	55384	---	---	---	---		MmugDNA.11530.1.S1_at_MEG3
MmugDNA.11531.1.S1_at	227520_at	CXorf15	chromosome X open reading frame 15	chrXp22.2	55787	LOC713769	gamma-taxilin	---		713769	MmugDNA.11531.1.S1_at_CXorf15
MmugDNA.1153.1.S1_at	222751_at	HERPUD2	HERPUD family member 2	chr7p14.2	64224	---	---	---	---		MmugDNA.1153.1.S1_at_HERPUD2
MmugDNA.11532.1.S1_at	225224_at	C20orf112	chromosome 20 open reading frame 112	chr20q11.1-q11.23	140688	---	---	---	---		MmugDNA.11532.1.S1_at_C20orf112
MmugDNA.11533.1.S1_at	1552299_at	AKD2	adenylate kinase domain containing 2	chr6q21	221264	---	---	---	---		MmugDNA.11533.1.S1_at_AKD2
MmugDNA.11534.1.S1_at	229876_at	PHKA1	phosphorylase kinase, alpha 1 (muscle)	chrXq12-q13	5255	PHKA1	phosphorylase kinase, alpha 1 (muscle)	---		699764	MmugDNA.11534.1.S1_at_PHKA1
MmugDNA.11535.1.S1_at	219575_s_at	COG8 /// PDF	component of oligomeric golgi complex 8 /// peptide deformylase (mitochondrial)	chr16q22.1	64146 /// 84342	COG8	similar to component of oligomeric golgi complex 8	---		704556	MmugDNA.11535.1.S1_at_COG8 /// PD
MmugDNA.11536.1.S1_at	229615_at	---	---	---	---	---	---	---	---		MmugDNA.11536.1.S1_at_---
MmugDNA.11537.1.S1_at	230342_at	FOXK1	forkhead box K1	chr7p22.1	221937	LOC715686	similar to Forkhead box protein K1 (Myocyte nuclear factor) (MNF)	---		715686	MmugDNA.11537.1.S1_at_FOXK1
MmugDNA.11538.1.S1_at	231228_at	BCL2L1	BCL2-like 1	chr20q11.21	598	LOC713035	similar to BCL2-like 1 isoform 1	---		713035	MmugDNA.11538.1.S1_at_BCL2L1
MmugDNA.11539.1.S1_at	203830_at	C17orf75	chromosome 17 open reading frame 75	chr17q11.2	64149	LOC703888	Similar to nucleoporin 153kDa	---		703888	MmugDNA.11539.1.S1_at_C17orf75
MmugDNA.11541.1.S1_at	220940_at	ANKRD36B	ankyrin repeat domain 36B	chr2q11.2	57730	---	---	---	---		MmugDNA.11541.1.S1_at_ANKRD36B
MmugDNA.1154.1.S1_at	208078_s_at	SIK1	salt-inducible kinase 1	chr21q22.3	150094	SIK1	salt-inducible kinase 1	---		722303	MmugDNA.1154.1.S1_at_SIK1
MmugDNA.11542.1.S1_at	224966_s_at	DUS3L	dihydrouridine synthase 3-like (S. cerevisiae)	chr19p13.3	56931	LOC719524	similar to dihydrouridine synthase 3-like	---		719524	MmugDNA.11542.1.S1_at_DUS3L
MmugDNA.11543.1.S1_at	1560305_x_at	FKBP4	FK506 binding protein 4, 59kDa	chr12p13.33	2288	FKBP4	FK506 binding protein 4, 59kDa	---		708481	MmugDNA.11543.1.S1_at_FKBP4
MmugDNA.11544.1.S1_at	1569666_s_at	---	---	---	---	---	---	---	---		MmugDNA.11544.1.S1_at_---
MmugDNA.11545.1.S1_at	230063_at	ZNF264	zinc finger protein 264	chr19q13.4	9422	---	---	---	---		MmugDNA.11545.1.S1_at_ZNF264
MmugDNA.11546.1.S1_at	211510_s_at	CRHR2	corticotropin releasing hormone receptor 2	chr7p15.1	1395	LOC697404	similar to corticotropin releasing hormone receptor 2	---		697404	MmugDNA.11546.1.S1_at_CRHR2

MmugDNA.11547.1.S1_at	227351_at	C16orf52	chromosome 16 open reading frame 52	chr16p12.1	730094	---	---	---	---	MmugDNA.11547.1.S1_at_C16orf52
MmugDNA.11548.1.S1_at	1559522_at	---	---	---	---	---	---	---	---	MmugDNA.11548.1.S1_at_---
MmugDNA.11549.1.S1_at	223212_at	ZDHHC16	zinc finger, DHHC-type containing 16	chr10q24.1	84287	ZDHHC16	zinc finger, DHHC-type containing 16	---	705982	MmugDNA.11549.1.S1_at_ZDHHC16
MmugDNA.11550.1.S1_at	1555865_at	LOC255512	hypothetical protein LOC255512	chr11p15.5	255512	---	---	---	---	MmugDNA.11550.1.S1_at_LOC255512
MmugDNA.11551.1.S1_at	225846_at	ESRP1	epithelial splicing regulatory protein 1	chr8q22.1	54845	---	---	---	---	MmugDNA.11551.1.S1_at_ESRP1
MmugDNA.1155.1.S1_at	1554594_at	SH3D20	SH3 domain containing 20	chr17q21.31	201175	---	---	---	---	MmugDNA.1155.1.S1_at_SH3D20
MmugDNA.11552.1.S1_at	215351_at	RTCD1	RNA terminal phosphate cyclase domain 1	chr1p21.2	8634	LOC700540	Similar to ribosomal protein L24	---	700540	MmugDNA.11552.1.S1_at_RTCD1
MmugDNA.11553.1.S1_at	1561278_at	---	---	---	---	---	---	---	---	MmugDNA.11553.1.S1_at_---
MmugDNA.11554.1.S1_at	243323_s_at	ZFHX3	Zinc finger homeobox 3	chr16q22.3-q23.1	463	---	---	---	---	MmugDNA.11554.1.S1_at_ZFHX3
MmugDNA.11555.1.S1_at	227021_at	AOF1	amine oxidase (flavin containing) domain 1	chr6p22.3	221656	AOF1	similar to amine oxidase, flavin containing 1	---	704663	MmugDNA.11555.1.S1_at_AOF1
MmugDNA.11556.1.S1_at	1564391_at	---	---	---	---	---	---	---	---	MmugDNA.11556.1.S1_at_---
MmugDNA.11557.1.S1_at	218663_at	NCAPG	non-SMC condensin I complex, subunit G	chr4p15.33	64151	LOC715235	similar to chromosome condensation protein G	---	715235	MmugDNA.11557.1.S1_at_NCAPG
MmugDNA.11558.1.S1_at	1562823_at	---	---	---	---	---	---	---	---	MmugDNA.11558.1.S1_at_---
MmugDNA.11559.1.S1_at	1561517_at	SSPN	Sarcospan (Kras oncogene-associated gene)	chr12p11.2	8082	---	---	---	---	MmugDNA.11559.1.S1_at_SSPN
MmugDNA.11560.1.S1_at	227797_x_at	LYRM2	LYR motif containing 2	chr6q14.2-q16.1	57226	---	---	---	---	MmugDNA.11560.1.S1_at_LYRM2
MmugDNA.11561.1.S1_at	1562727_at	---	---	---	---	---	---	---	---	MmugDNA.11561.1.S1_at_---
MmugDNA.1156.1.S1_at	NA	NA	NA	NA	NA	NA	NA	NA	NA	MmugDNA.1156.1.S1_at_NA
MmugDNA.11562.1.S1_at	217216_x_at	MLH3	mutL homolog 3 (E. coli)	chr14q24.3	27030	MLH3	mutL homolog 3	---	701482	MmugDNA.11562.1.S1_at_MLH3
MmugDNA.11563.1.S1_at	219666_at	MS4A6A	membrane-spanning 4-domains, subfamily A, member 6A	chr11q12.1	64231	MS4A6A	membrane-spanning 4-domains, subfamily A, member 6A	---	697689	MmugDNA.11563.1.S1_at_MS4A6A
MmugDNA.11564.1.S1_at	1553427_at	ADAMTS15	ADAM metallopeptidase with thrombospondin type 1 motif, 15	chr11q25	170689	LOC715608	similar to a disintegrin-like and metalloprotease (reprolysin type) with thrombospondin type 1 motif, 15	---	715608	MmugDNA.11564.1.S1_at_ADAMTS15
MmugDNA.11565.1.S1_at	1556078_at	---	---	---	---	---	---	---	---	MmugDNA.11565.1.S1_at_---
MmugDNA.11566.1.S1_at	223736_at	IFT81	intraflagellar transport 81 homolog (Chlamydomonas)	chr12q24.13	28981	---	---	---	---	MmugDNA.11566.1.S1_at_IFT81
MmugDNA.11567.1.S1_s_at	NA	NA	NA	NA	NA	NA	NA	NA	NA	MmugDNA.11567.1.S1_s_at_NA
MmugDNA.11568.1.S1_at	1556259_at	---	---	---	---	---	---	---	---	MmugDNA.11568.1.S1_at_---
MmugDNA.11569.1.S1_at	208469_s_at	EGFL8 /// PPT2	EGF-like-domain, multiple 8 /// palmitoyl-protein thioesterase 2	chr6p21.3 /// chr6p21.32	80864 /// 9374	LOC717145	similar to NG3 protein	---	717145	MmugDNA.11569.1.S1_at_EGFL8 /// PI
MmugDNA.11570.1.S1_at	229145_at	C10orf104	chromosome 10 open reading frame 104	chr10q22.1	119504	---	---	---	---	MmugDNA.11570.1.S1_at_C10orf104
MmugDNA.11571.1.S1_at	227584_at	NAV1	neuron navigator 1	chr1q32.3	89796	---	---	---	---	MmugDNA.11571.1.S1_at_NAV1
MmugDNA.1157.1.S1_at	236009_at	PERP	PERP, TP53 apoptosis effector	chr6q24	64065	---	---	---	---	MmugDNA.1157.1.S1_at_PERP
MmugDNA.11572.1.S1_at	209687_at	CXCL12	chemokine (C-X-C motif) ligand 12 (stromal cell-derived factor 1)	chr10q11.1	6387	---	---	---	---	MmugDNA.11572.1.S1_at_CXCL12
MmugDNA.11573.1.S1_at	206061_s_at	DICER1	dicer 1, ribonuclease type III	chr14q32.13	23405	DICER1	dicer 1, ribonuclease type III	---	702516	MmugDNA.11573.1.S1_at_DICER1
MmugDNA.11574.1.S1_at	240034_at	---	---	---	---	---	---	---	---	MmugDNA.11574.1.S1_at_---
MmugDNA.11575.1.S1_at	213981_at	COMT	catechol-O-methyltransferase	chr22q11.21-q11.23|22q11.21	1312	LOC712548	similar to Catechol O-methyltransferase	---	712548	MmugDNA.11575.1.S1_at_COMT
MmugDNA.11576.1.S1_at	240467_at	---	---	---	---	---	---	---	---	MmugDNA.11576.1.S1_at_---
MmugDNA.11577.1.S1_at	1557374_at	ABCC9	ATP-binding cassette, sub-family C (CFTR/MRP), member 9	chr12p12.1	10060	---	---	---	---	MmugDNA.11577.1.S1_at_ABCC9
MmugDNA.11578.1.S1_at	239759_at	---	---	---	---	---	---	---	---	MmugDNA.11578.1.S1_at_---
MmugDNA.11579.1.S1_at	1554830_a_at	STEAP3	STEAP family member 3	chr2q14.2	55240	---	---	---	---	MmugDNA.11579.1.S1_at_STEAP3
MmugDNA.11580.1.S1_at	219315_s_at	TMEM204	transmembrane protein 204	chr16p13.3	79652	LOC701926	similar to Claudin-like protein 24	---	701926	MmugDNA.11580.1.S1_at_TMEM204
MmugDNA.11581.1.S1_at	214738_s_at	NEK9	NIMA (never in mitosis gene a)- related kinase 9	chr14q24.3	91754	NEK9	NIMA (never in mitosis gene a)- related kinase 9	---	701707	MmugDNA.11581.1.S1_at_NEK9
MmugDNA.1158.1.S1_at	209554_at	CD36	CD36 molecule (thrombospondin receptor)	chr7q11.2	948	---	---	---	---	MmugDNA.1158.1.S1_at_CD36
MmugDNA.11582.1.S1_at	220162_s_at	CARD9	caspase recruitment domain family, member 9	chr9q34.3	64170	LOC710485	similar to caspase recruitment domain protein 9	---	710485	MmugDNA.11582.1.S1_at_CARD9
MmugDNA.11583.1.S1_at	208598_s_at	HUWE1	HECT, UBA and WWE domain containing 1	chrXp11.22	10075	HUWE1	HECT, UBA and WWE domain containing 1	---	700136	MmugDNA.11583.1.S1_at_HUWE1
MmugDNA.11584.1.S1_at	220033_at	---	---	---	---	---	---	---	---	MmugDNA.11584.1.S1_at_---
MmugDNA.11585.1.S1_at	220631_at	OSGEPL1	O-sialoglycoprotein endopeptidase-like 1	chr2q32.2	64172	LOC709747	similar to O-sialoglycoprotein endopeptidase-like 1	---	709747	MmugDNA.11585.1.S1_at_OSGEPL1
MmugDNA.11586.1.S1_at	237435_at	---	---	---	---	---	---	---	---	MmugDNA.11586.1.S1_at_---
MmugDNA.11587.1.S1_at	233578_at	C1orf101	chromosome 1 open reading frame 101	chr1q44	257044	LOC704627	hypothetical protein LOC704627	---	704627	MmugDNA.11587.1.S1_at_C1orf101
MmugDNA.11588.1.S1_at	218632_at	HECTD3	HECT domain containing 3	chr1p34.1	79654	HECTD3	HECT domain containing 3	---	705051	MmugDNA.11588.1.S1_at_HECTD3
MmugDNA.11589.1.S1_at	221057_at	SPATA1	spermatogenesis associated 1	chr1p22.3	64173	LOC710462	similar to spermatogenesis associated 1	---	710462	MmugDNA.11589.1.S1_at_SPATA1
MmugDNA.11590.1.S1_at	219670_at	BEND5	BEN domain containing 5	chr1p33	79656	LOC711150	hypothetical protein LOC711150	---	711150	MmugDNA.11590.1.S1_at_BEND5
MmugDNA.11591.1.S1_s_at	219452_at	DPEP2	dipeptidase 2	chr16q22.1	64174	LOC701570	similar to dipeptidase 2	---	701570	MmugDNA.11591.1.S1_s_at_DPEP2
MmugDNA.1159.1.S1_at	NA	NA	NA	NA	NA	NA	NA	NA	NA	MmugDNA.1159.1.S1_at_NA
MmugDNA.11592.1.S1_at	NA	NA	NA	NA	NA	NA	NA	NA	NA	MmugDNA.11592.1.S1_at_NA
MmugDNA.11592.1.S1_s_at	1554530_at	VSTM2A	V-set and transmembrane domain containing 2A	chr7p11.2	222008	LOC705954	hypothetical protein LOC705954	---	705954	MmugDNA.11592.1.S1_s_at_VSTM2A
MmugDNA.11593.1.S1_at	218842_at	RPAP3	RNA polymerase II associated protein 3	chr12q13.11	79657	RPAP3	RNA polymerase II associated protein 3	---	703749	MmugDNA.11593.1.S1_at_RPAP3
MmugDNA.11594.1.S1_at	223534_s_at	RPS6KL1	ribosomal protein S6 kinase-like 1	chr14q24.3	83694	RPS6KL1	ribosomal protein S6 kinase-like 1	---	701119	MmugDNA.11594.1.S1_at_RPS6KL1
MmugDNA.11595.1.S1_at	227651_at	NACC1	nucleus accumbens associated 1, BEN and BTB (POZ) domain containing	chr19p13.13	112939	---	---	---	---	MmugDNA.11595.1.S1_at_NACC1
MmugDNA.11596.1.S1_at	1563907_at	GARNL3	GTPase activating Rap/RanGAP domain-like 3	chr9q33.3	84253	---	---	---	---	MmugDNA.11596.1.S1_at_GARNL3
MmugDNA.11597.1.S1_at	204138_s_at	MZF1	myeloid zinc finger 1	chr19q13.4	7593	LOC712144	similar to zinc finger protein 42 isoform 2	---	712144	MmugDNA.11597.1.S1_at_MZF1
MmugDNA.11598.1.S1_at	1563993_at	---	---	---	---	---	---	---	---	MmugDNA.11598.1.S1_at_---
MmugDNA.11599.1.S1_at	235506_at	---	---	---	---	---	---	---	---	MmugDNA.11599.1.S1_at_---
MmugDNA.11600.1.S1_at	1553386_at	MFSD2	major facilitator superfamily domain containing 2	chr1p34.2	84879	---	---	---	---	MmugDNA.11600.1.S1_at_MFSD2
MmugDNA.11601.1.S1_at	208890_s_at	PLXNB2	plexin B2	chr22q13.33	23654	PLXNB2	plexin B2	---	720646	MmugDNA.11601.1.S1_at_PLXNB2
MmugDNA.1160.1.S1_at	220024_s_at	PRX	periaxin	chr19q13.13-q13.2	57716	LOC702329	similar to Periaxin	---	702329	MmugDNA.1160.1.S1_at_PRX
MmugDNA.11602.1.S1_at	1554489_a_at	CEP70	centrosomal protein 70kDa	chr3q22-q23	80321	---	---	---	---	MmugDNA.11602.1.S1_at_CEP70
MmugDNA.11603.1.S1_at	231271_x_at	NMRAL1	NmrA-like family domain containing 1	chr16p13.3	57407	---	---	---	---	MmugDNA.11603.1.S1_at_NMRAL1
MmugDNA.11603.1.S1_s_at	NA	NA	NA	NA	NA	NA	NA	NA	NA	MmugDNA.11603.1.S1_s_at_NA
MmugDNA.11604.1.S1_at	221856_s_at	FAM63A	family with sequence similarity 63, member A	chr1q21.2	55793	---	---	---	---	MmugDNA.11604.1.S1_at_FAM63A
MmugDNA.11605.1.S1_at	1556737_at	LOC388387	hypothetical LOC388387	chr17q21.31	388387	---	---	---	---	MmugDNA.11605.1.S1_at_LOC388387
MmugDNA.11606.1.S1_at	226062_x_at	FAM63A	family with sequence similarity 63, member A	chr1q21.2	55793	---	---	---	---	MmugDNA.11606.1.S1_at_FAM63A
MmugDNA.11607.1.S1_at	240867_at	---	---	---	---	---	---	---	---	MmugDNA.11607.1.S1_at_---
MmugDNA.11608.1.S1_at	217305_s_at	ADCY10	adenylate cyclase 10 (soluble)	chr1q24	55811	LOC698566	similar to soluble adenylyl cyclase	---	698566	MmugDNA.11608.1.S1_at_ADCY10
MmugDNA.11609.1.S1_at	1561099_at	FLJ32756	hypothetical LOC642757	chr22q13.31	642757	---	---	---	---	MmugDNA.11609.1.S1_at_FLJ32756
MmugDNA.11610.1.S1_at	218163_at	MCTS1	malignant T cell amplified sequence 1	chrXq22-q24	28985	MCTS1	malignant T cell amplified sequence 1	---	696543	MmugDNA.11610.1.S1_at_MCTS1
MmugDNA.11611.1.S1_at	1563245_at	---	---	---	---	---	---	---	---	MmugDNA.11611.1.S1_at_---
MmugDNA.1161.1.S1_at	1556029_s_at	NMNAT2	nicotinamide nucleotide adenylyltransferase 2	chr1q25	23057	LOC717648	similar to nicotinamide mononucleotide adenylyltransferase 2 isoform 1	---	717648	MmugDNA.1161.1.S1_at_NMNAT2
MmugDNA.11612.1.S1_at	1570351_at	ADAMTS6	ADAM metallopeptidase with thrombospondin type 1 motif, 6	chr5q12	11174	---	---	---	---	MmugDNA.11612.1.S1_at_ADAMTS6
MmugDNA.11613.1.S1_at	241804_at	---	---	---	---	---	---	---	---	MmugDNA.11613.1.S1_at_---
MmugDNA.11614.1.S1_at	206584_at	LY96	lymphocyte antigen 96	chr8q21.11	23643	LY96	lymphocyte antigen 96	---	696778	MmugDNA.11614.1.S1_at_LY96
MmugDNA.11615.1.S1_at	1557571_at	VPS13D	Vacuolar protein sorting 13 homolog D (S. cerevisiae)	chr1p36.22	55187	---	---	---	---	MmugDNA.11615.1.S1_at_VPS13D
MmugDNA.11616.1.S1_at	225877_at	TYSND1	trypsin domain containing 1	chr10q22.1	219743	---	---	---	---	MmugDNA.11616.1.S1_at_TYSND1
MmugDNA.11617.1.S1_at	217955_at	BCL2L13	BCL2-like 13 (apoptosis facilitator)	chr22q11.1	23786	BCL2L13	BCL2-like 13 (apoptosis facilitator)	---	709825	MmugDNA.11617.1.S1_at_BCL2L13
MmugDNA.11618.1.S1_at	NA	NA	NA	NA	NA	NA	NA	NA	NA	MmugDNA.11618.1.S1_at_NA
MmugDNA.11618.1.S1_s_at	225036_at	SHB /// TOMM5	Src homology 2 domain containing adaptor protein B /// translocase of outer mitochondrial membrane	chr9p13.2 /// chr9p13.2-p13.1	401505 /// 6461	LOC716461	hypothetical protein LOC716461	---	716461	MmugDNA.11618.1.S1_s_at_SHB /// TC
MmugDNA.11619.1.S1_s_at	232826_at	---	---	---	---	---	---	---	---	MmugDNA.11619.1.S1_at_---
MmugDNA.116.1.S1_at	215451_s_at	AFF1	AF4/FMR2 family, member 1	chr4q21	4299	AFF1	AF4/FMR2 family, member 1	---	700733	MmugDNA.116.1.S1_at_AFF1
MmugDNA.11620.1.S1_at	222065_s_at	FLII	flightless I homolog (Drosophila)	chr17p11.2	2314	---	---	---	---	MmugDNA.11620.1.S1_at_FLII
MmugDNA.11621.1.S1_at	229736_at	TMEM86B	transmembrane protein 86B	chr19q13.42	255043	LOC699222	hypothetical protein LOC699222	---	699222	MmugDNA.11621.1.S1_at_TMEM86B
MmugDNA.1162.1.S1_at	200644_at	MARCKSL1	MARCKS-like 1	chr1p35.1	65108	---	---	---	---	MmugDNA.1162.1.S1_at_MARCKSL1
MmugDNA.11622.1.S1_at	NA	NA	NA	NA	NA	NA	NA	NA	NA	MmugDNA.11622.1.S1_at_NA
MmugDNA.11623.1.S1_at	204715_at	PANX1	pannexin 1	chr11q21	24145	PANX1	pannexin 1	---	697204	MmugDNA.11623.1.S1_at_PANX1
MmugDNA.11624.1.S1_at	220179_at	DPEP3	dipeptidase 3	chr16q22.1	64180	LOC710176	Hypothetical protein LOC710176	---	710176	MmugDNA.11624.1.S1_at_DPEP3
MmugDNA.11625.1.S1_at	236133_x_at	ZNF254	zinc finger protein 254	chr19p12	9534	LOC699094	similar to zinc finger protein 539	---	699094	MmugDNA.11625.1.S1_at_ZNF254
MmugDNA.11626.1.S1_at	219469_at	DYNC2H1	dynein, cytoplasmic 2, heavy chain 1	chr11q21-q22.1	79659	---	---	---	---	MmugDNA.11626.1.S1_at_DYNC2H1
MmugDNA.11627.1.S1_at	NA	NA	NA	NA	NA	NA	NA	NA	NA	MmugDNA.11627.1.S1_at_NA
MmugDNA.11628.1.S1_at	240614_at	KCNC2	potassium voltage-gated channel, Shaw-related subfamily, member 2	chr12q14.1	3747	---	---	---	---	MmugDNA.11628.1.S1_at_KCNC2
MmugDNA.11629.1.S1_at	211480_s_at	SLCO1A2	solute carrier organic anion transporter family, member 1A2	chr12p12	6579	SLCO1A2	solute carrier organic anion transporter family, member 1A2	---	705434	MmugDNA.11629.1.S1_at_SLCO1A2
MmugDNA.11630.1.S1_at	213271_s_at	DOPEY1	dopey family member 1	chr6q15	23033	LOC694339	similar to pad-1-like isoform 1	---	694339	MmugDNA.11630.1.S1_at_DOPEY1
MmugDNA.11631.1.S1_s_at	204470_at	CXCL1	chemokine (C-X-C motif) ligand 1 (melanoma growth stimulating activity, alpha)	chr4q21	2919	---	---	---	---	MmugDNA.11631.1.S1_s_at_CXCL1
MmugDNA.1163.1.S1_a_at	234421_s_at	EVC	Ellis van Creveld syndrome	chr4p16	2121	EVC	Ellis van Creveld syndrome	---	711266	MmugDNA.1163.1.S1_a_at_EVC
MmugDNA.1163.1.S1_at	NA	NA	NA	NA	NA	NA	NA	NA	NA	MmugDNA.1163.1.S1_at_NA
MmugDNA.11632.1.S1_at	205872_x_at	PDE4DIP	phosphodiesterase 4D interacting protein	chr1q12	9659	LOC716104	similar to phosphodiesterase 4D interacting protein isoform 1	---	716104	MmugDNA.11632.1.S1_at_PDE4DIP
MmugDNA.11633.1.S1_at	213267_at	DOPEY1	dopey family member 1	chr6q15	23033	---	---	---	---	MmugDNA.11633.1.S1_at_DOPEY1
MmugDNA.11634.1.S1_at	219396_s_at	NEIL1	nei endonuclease VIII-like 1 (E. coli)	chr15q24.2	79661	NEIL1	nei endonuclease VIII-like 1	---	705484	MmugDNA.11634.1.S1_at_NEIL1
MmugDNA.11635.1.S1_at	220759_at	FAM12B	family with sequence similarity 12, member B (epididymal)	chr14q11.2	64184	LOC704471	similar to human epididymis-specific 3 beta precursor	---	704471	MmugDNA.11635.1.S1_at_FAM12B
MmugDNA.11636.1.S1_at	240269_at	---	---	---	---	---	---	---	---	MmugDNA.11636.1.S1_at_---
MmugDNA.11637.1.S1_at	218678_at	NES	nestin	chr1q23.1	10763	---	---	---	---	MmugDNA.11637.1.S1_at_NES
MmugDNA.11637.1.S1_s_at	NA	NA	NA	NA	NA	NA	NA	NA	NA	MmugDNA.11637.1.S1_s_at_NA
MmugDNA.11638.1.S1_at	1556997_a_at	---	---	---	---	---	---	---	---	MmugDNA.11638.1.S1_at_---
MmugDNA.11639.1.S1_at	209486_at	UTP3	UTP3, small subunit (SSU) processome component, homolog (S. cerevisiae)	chr4q13.3	57050	SAS10	disrupter of silencing 10	---	706536	MmugDNA.11639.1.S1_at_UTP3

MmugDNA.11640.1.S1_at	244168_s_at	ULK4	unc-51-like kinase 4 (C. elegans)	chr3p22.1		54986	LOC717956	similar to serine/threonine kinase 36 (fused homolog, Drosophila)	---		717956	MmugDNA.11640.1.S1_at_ULK4
MmugDNA.11641.1.S1_s_at	210257_x_at	CUL4B	cullin 4B	chrXq23		8450	CUL4B	cullin 4B	---		696410	MmugDNA.11641.1.S1_s_at_CUL4B
MmugDNA.1164.1.S1_at	229235_at	ZCCHC11	zinc finger, CCHC domain containing 11	chr1p32.3		23318	---	---	---	---		MmugDNA.1164.1.S1_at_ZCCHC11
MmugDNA.11642.1.S1_at	1562684_at	---	---	---	---		---	---	---	---		MmugDNA.11642.1.S1_at_---
MmugDNA.11643.1.S1_at	209029_at	COPS7A	COP9 constitutive photomorphogenic homolog subunit 7A (Arabidopsis)	chr12p13.31		50813	COPS7A	COP9 constitutive photomorphogenic homolog subunit 7A (Arabidopsis)	---		713607	MmugDNA.11643.1.S1_at_COPS7A
MmugDNA.11644.1.S1_at	1569470_a_at	FRMD5	FERM domain containing 5	chr15q15.3		84978	LOC711670	similar to CG5022-PA	---		711670	MmugDNA.11644.1.S1_at_FRMD5
MmugDNA.11645.1.S1_at	217666_at	---	---	---	---		---	---	---	---		MmugDNA.11645.1.S1_at_---
MmugDNA.11646.1.S1_at	1557451_at	DGCR10	DiGeorge syndrome critical region gene 10	chr22q11		26222	---	---	---	---		MmugDNA.11646.1.S1_at_DGCR10
MmugDNA.11647.1.S1_at	230489_at	CD5	CD5 molecule	chr11q13		921	CD5	CD5 molecule	---		695249	MmugDNA.11647.1.S1_at_CD5
MmugDNA.11648.1.S1_at	214635_at	CLDN9	claudin 9	chr16p13.3		9080	LOC699721	similar to claudin 9	---		699721	MmugDNA.11648.1.S1_at_CLDN9
MmugDNA.11649.1.S1_at	218259_at	MKL2	MKL/myocardin-like 2	chr16p13.12		57496	LOC712856	similar to megakaryoblastic leukemia 2 protein	---		712856	MmugDNA.11649.1.S1_at_MKL2
MmugDNA.11650.1.S1_at	228873_at	COL22A1	collagen, type XXII, alpha 1	chr8q24.23		169044	---	---	---	---		MmugDNA.11650.1.S1_at_COL22A1
MmugDNA.11651.1.S1_at	1555545_at	KLK2	kallikrein-related peptidase 2	chr19q13.41		3817	---	---	---	---		MmugDNA.11651.1.S1_at_KLK2
MmugDNA.1165.1.S1_at	211305_x_at	FCAR	Fc fragment of IgA, receptor for	chr19q13.2-q13.4		2204	---	---	---	---		MmugDNA.1165.1.S1_at_FCAR
MmugDNA.11652.1.S1_at	203115_at	FECH	ferrochelatase (protoporphyria)	chr18q21.3		2235	FECH	ferrochelatase (protoporphyria)	---		695759	MmugDNA.11652.1.S1_at_FECH
MmugDNA.11653.1.S1_at	219284_at	HSPBAP1	HSPB (heat shock 27kDa) associated protein 1	chr3q21.1		79663	LOC715073	similar to Hspb associated protein 1	---		715073	MmugDNA.11653.1.S1_at_HSPBAP1
MmugDNA.11654.1.S1_at	1552596_at	GAS2L2	growth arrest-specific 2 like 2	chr17q12		246176	LOC716209	similar to growth arrest-specific 2 like 2	---		716209	MmugDNA.11654.1.S1_at_GAS2L2
MmugDNA.11655.1.S1_at	208333_at	LHX5	LIM homeobox 5	chr12q24		64211	LOC713417	similar to LIM homeobox protein 5	---		713417	MmugDNA.11655.1.S1_at_LHX5
MmugDNA.11656.1.S1_at	218713_at	NARG2	NMDA receptor regulated 2	chr15q22.2		79664	---	---	---	---		MmugDNA.11656.1.S1_at_NARG2
MmugDNA.11657.1.S1_at	221130_s_at	---	---	---	---		---	---	---	---		MmugDNA.11657.1.S1_at_---
MmugDNA.11658.1.S1_at	218277_s_at	DHX40	DEAH (Asp-Glu-Ala-His) box polypeptide 40	chr17q23.1		79665	LOC715202	similar to DEAH (Asp-Glu-Ala-His) box polypeptide 40	---		715202	MmugDNA.11658.1.S1_at_DHX40
MmugDNA.11659.1.S1_s_at	212128_s_at	DAG1	dystroglycan 1 (dystrophin-associated glycoprotein 1)	chr3p21		1605	DAG1	dystroglycan 1	---		706179	MmugDNA.11659.1.S1_s_at_DAG1
MmugDNA.11660.1.S1_at	218409_s_at	DNAJC1	DnaJ (Hsp40) homolog, subfamily C, member 1	chr10p12.31		64215	DNAJC1	DnaJ (Hsp40) homolog, subfamily C, member 1	---		704151	MmugDNA.11660.1.S1_at_DNAJC1
MmugDNA.11661.1.S1_at	218640_s_at	PLEKHF2	pleckstrin homology domain containing, family F (with FYVE domain) member 2	chr8q22.1		79666	LOC701884	hypothetical protein LOC701884	---		701884	MmugDNA.11661.1.S1_at_PLEKHF2
MmugDNA.1166.1.S1_at	220382_s_at	ARHGAP28	Rho GTPase activating protein 28	chr18p11.31		79822	LOC707240	similar to Rho GTPase activating protein 28 isoform a	---		707240	MmugDNA.1166.1.S1_at_ARHGAP28
MmugDNA.11662.1.S1_at	218605_at	TFB2M	transcription factor B2, mitochondrial	chr1q44		64216	LOC710669	similar to transcription factor B2, mitochondrial	---		710669	MmugDNA.11662.1.S1_at_TFB2M
MmugDNA.11663.1.S1_at	219871_at	FLJ13197	hypothetical FLJ13197	chr4p14		79667	---	---	---	---		MmugDNA.11663.1.S1_at_FLJ13197
MmugDNA.11664.1.S1_at	230121_at	C1orf133	chromosome 1 open reading frame 133	chr1q32.2		574036	LOC712687	hypothetical protein LOC712687	---		712687	MmugDNA.11664.1.S1_at_C1orf133
MmugDNA.11665.1.S1_at	1558941_at	ZNF704	zinc finger protein 704	chr8q21.13		619279	ZNF704	zinc finger protein 704	---		700514	MmugDNA.11665.1.S1_at_ZNF704
MmugDNA.11666.1.S1_at	1561572_at	---	---	---	---		---	---	---	---		MmugDNA.11666.1.S1_at_---
MmugDNA.11667.1.S1_at	236192_at	HOOK3	hook homolog 3 (Drosophila)	chr8p11.21		84376	---	---	---	---		MmugDNA.11667.1.S1_at_HOOK3
MmugDNA.11668.1.S1_at	244326_at	---	---	---	---		---	---	---	---		MmugDNA.11668.1.S1_at_---
MmugDNA.11669.1.S1_at	243192_at	---	---	---	---		---	---	---	---		MmugDNA.11669.1.S1_at_---
MmugDNA.11670.1.S1_at	1560883_s_at	---	---	---	---		---	---	---	---		MmugDNA.11670.1.S1_at_---
MmugDNA.11671.1.S1_at	227501_at	---	---	---	---		---	---	---	---		MmugDNA.11671.1.S1_at_---
MmugDNA.1167.1.S1_at	220741_s_at	PPA2	pyrophosphatase (inorganic) 2	chr4q25		27068	PPA2	pyrophosphatase (inorganic) 2	---		694267	MmugDNA.1167.1.S1_at_PPA2
MmugDNA.11672.1.S1_at	236248_x_at	TADA2B	transcriptional adaptor 2 (ADA2 homolog, yeast)-beta	chr4p16.1		93624	LOC722715	similar to Ada2b CG9638-PA, isoform A	---		722715	MmugDNA.11672.1.S1_at_TADA2B
MmugDNA.11673.1.S1_at	231336_at	CPNE4	copine IV	chr3q22.1		131034	LOC717868 /// LOC7192	similar to copine IV /// similar to copine IV	---	717868 /// 719231		MmugDNA.11673.1.S1_at_CPNE4
MmugDNA.11674.1.S1_at	220691_at	---	---	---	---		---	---	---	---		MmugDNA.11674.1.S1_at_---
MmugDNA.11675.1.S1_at	244805_at	---	---	---	---		---	---	---	---		MmugDNA.11675.1.S1_at_---
MmugDNA.11676.1.S1_at	240632_at	---	---	---	---		---	---	---	---		MmugDNA.11676.1.S1_at_---
MmugDNA.11677.1.S1_at	242367_at	SLC38A1	solute carrier family 38, member 1	chr12q13.11		81539	LOC695736	Similar to phosphatidylinositol N-acetylglucosaminyltransferase subunit A isoform 2	---		695736	MmugDNA.11677.1.S1_at_SLC38A1
MmugDNA.11678.1.S1_at	227135_at	NAAA	N-acylethanolamine acid amidase	chr4q21.1		27163	LOC711283	Similar to 3(2),5-bisphosphate nucleotidase 1 (Bisphosphate 3-nucleotidase 1) (PAP-inositol-1,4-phos	---		711283	MmugDNA.11678.1.S1_at_NAAA
MmugDNA.11679.1.S1_at	220479_at	LOC29034	hypothetical LOC29034	chr2q34		29034	---	---	---	---		MmugDNA.11679.1.S1_at_LOC29034
MmugDNA.11680.1.S1_at	219259_at	SEMA4A	sema domain, immunoglobulin domain (Ig), transmembrane domain (TM) and short cytoplasmic doma	chr1q22		64218	LOC718191	similar to semaphorin B	---		718191	MmugDNA.11680.1.S1_at_SEMA4A
MmugDNA.11681.1.S1_at	229772_at	DEFB123	defensin, beta 123	chr20q11.1		245936	DEFB123	defensin, beta 123	---		619504	MmugDNA.11681.1.S1_at_DEFB123
MmugDNA.1168.1.S1_at	NA	NA	NA	NA	NA		NA	NA	NA	NA		MmugDNA.1168.1.S1_at_NA
MmugDNA.1168.1.S1_s_at	232937_at	---	---	---	---		---	---	---	---		MmugDNA.1168.1.S1_s_at_---
MmugDNA.11682.1.S1_at	242792_at	---	---	---	---		---	---	---	---		MmugDNA.11682.1.S1_at_---
MmugDNA.11683.1.S1_at	220743_at	---	---	---	---		---	---	---	---		MmugDNA.11683.1.S1_at_---
MmugDNA.11684.1.S1_at	218667_at	PJA1	praja ring finger 1	chrXq13.1		64219	LOC695038	similar to Ubiquitin protein ligase Praja1 (RING finger protein 70)	---		695038	MmugDNA.11684.1.S1_at_PJA1
MmugDNA.11685.1.S1_at	219033_at	PARP8	poly (ADP-ribose) polymerase family, member 8	chr5q11.1		79668	PARP8	poly (ADP-ribose) polymerase family, member 8	---		702637	MmugDNA.11685.1.S1_at_PARP8
MmugDNA.11686.1.S1_at	NA	NA	NA	NA	NA		NA	NA	NA	NA		MmugDNA.11686.1.S1_at_NA
MmugDNA.11687.1.S1_at	220853_at	GTDC1	glycosyltransferase-like domain containing 1	chr2q22.2-q22.3		79712	---	---	---	---		MmugDNA.11687.1.S1_at_GTDC1
MmugDNA.11688.1.S1_at	219474_at	C3orf52	chromosome 3 open reading frame 52	chr3q13.2		79669	---	---	---	---		MmugDNA.11688.1.S1_at_C3orf52
MmugDNA.11689.1.S1_at	242276_at	---	---	---	---		---	---	---	---		MmugDNA.11689.1.S1_at_---
MmugDNA.11690.1.S1_at	239934_x_at	---	---	---	---		---	---	---	---		MmugDNA.11690.1.S1_at_---
MmugDNA.11691.1.S1_at	219550_at	ROBO3	roundabout, axon guidance receptor, homolog 3 (Drosophila)	chr11q24.2		64221	LOC716274	similar to roundabout, axon guidance receptor, homolog 3	---		716274	MmugDNA.11691.1.S1_at_ROBO3
MmugDNA.1169.1.S1_at	1554984_a_at	HLA-DOB	major histocompatibility complex, class II, DO beta	chr6p21.3		3112	LOC717702	similar to HLA class II histocompatibility antigen, DO beta chain precursor (MHC class II antigen DOB	---		717702	MmugDNA.1169.1.S1_at_HLA-DOB
MmugDNA.11692.1.S1_at	220933_s_at	ZCCHC6	zinc finger, CCHC domain containing 6	chr9q21		79670	ZCCHC6	zinc finger, CCHC domain containing 6	---		693660	MmugDNA.11692.1.S1_at_ZCCHC6
MmugDNA.11693.1.S1_at	218459_at	TOR3A	torsin family 3, member A	chr1q25.2		64222	LOC717100	similar to torsin family 3, member A	---		717100	MmugDNA.11693.1.S1_at_TOR3A
MmugDNA.11694.1.S1_at	219680_at	NLRX1	NLR family member X1	chr11q23.3		79671	NLRX1	NLR family member X1	---		703981	MmugDNA.11694.1.S1_at_NLRX1
MmugDNA.11695.1.S1_at	NA	NA	NA	NA	NA		NA	NA	NA	NA		MmugDNA.11695.1.S1_at_NA
MmugDNA.11696.1.S1_at	203129_s_at	KIF5C	kinesin family member 5C	chr2q23.1		3800	KIF5C	kinesin family member 5C	---		693340	MmugDNA.11696.1.S1_at_KIF5C
MmugDNA.11697.1.S1_at	215584_at	HECW1	HECT, C2 and WW domain containing E3 ubiquitin protein ligase 1	chr7p14.1-p13		23072	---	---	---	---		MmugDNA.11697.1.S1_at_HECW1
MmugDNA.11698.1.S1_at	204188_s_at	RARG	retinoic acid receptor, gamma	chr12q13		5916	RARG	retinoic acid receptor, gamma	---		699532	MmugDNA.11698.1.S1_at_RARG
MmugDNA.11699.1.S1_at	218210_at	FN3KRP	fructosamine 3 kinase related protein	chr17q25.3		79672	---	---	---	---		MmugDNA.11699.1.S1_at_FN3KRP
MmugDNA.11700.1.S1_at	225489_at	TMEM18	transmembrane protein 18	chr2p25.3		129787	LOC704209	similar to transmembrane protein 18	---		704209	MmugDNA.11700.1.S1_at_TMEM18
MmugDNA.11701.1.S1_at	225487_at	TMEM18	transmembrane protein 18	chr2p25.3		129787	---	---	---	---		MmugDNA.11701.1.S1_at_TMEM18
MmugDNA.1170.1.S1_at	236295_s_at	NLRC3	NLR family, CARD domain containing 3	chr16p13.3		197358	---	---	---	---		MmugDNA.1170.1.S1_at_NLRC3
MmugDNA.11702.1.S1_at	226067_at	C20orf114	chromosome 20 open reading frame 114	chr20q11.21		92747	LOC710014	LPLUNC1 protein	---		710014	MmugDNA.11702.1.S1_at_C20orf114
MmugDNA.11703.1.S1_at	211215_x_at	DIO2	deiodinase, iodothyronine, type II	chr14q24.2-q24.3		1734	DIO2	deiodinase, iodothyronine, type II	---		707484	MmugDNA.11703.1.S1_at_DIO2
MmugDNA.11704.1.S1_at	239645_at	---	---	---	---		---	---	---	---		MmugDNA.11704.1.S1_at_---
MmugDNA.11705.1.S1_at	236974_at	---	---	---	---		---	---	---	---		MmugDNA.11705.1.S1_at_---
MmugDNA.11706.1.S1_at	210819_x_at	DIO2	deiodinase, iodothyronine, type II	chr14q24.2-q24.3		1734	DIO2	deiodinase, iodothyronine, type II	---		707484	MmugDNA.11706.1.S1_at_DIO2
MmugDNA.11707.1.S1_at	1565700_at	---	---	---	---		---	---	---	---		MmugDNA.11707.1.S1_at_---
MmugDNA.11708.1.S1_at	207855_s_at	CLCC1	chloride channel CLIC-like 1	chr1p13.3		23155	---	---	---	---		MmugDNA.11708.1.S1_at_CLCC1
MmugDNA.11709.1.S1_at	231750_at	PCDHB4	protocadherin beta 4	chr5q31		56131	PCDHB4	protocadherin beta 4	---		700373	MmugDNA.11709.1.S1_at_PCDHB4
MmugDNA.11710.1.S1_at	236764_at	---	---	---	---		---	---	---	---		MmugDNA.11710.1.S1_at_---
MmugDNA.1171.1.S1_at	217664_at	---	---	---	---		---	---	---	---		MmugDNA.1171.1.S1_at_---
MmugDNA.11712.1.S1_at	202962_at	KIF13B	kinesin family member 13B	chr8p21.1		23303	LOC702877	Similar to Corticoliberin precursor (Corticotropin-releasing factor) (CRF) (Corticotropin-releasing horm	---		702877	MmugDNA.11712.1.S1_at_KIF13B
MmugDNA.11713.1.S1_at	234329_at	CLIC5	chloride intracellular channel 5	chr6p21.1-p12.1		53405	CLIC5	chloride intracellular channel 5	---		703565	MmugDNA.11713.1.S1_at_CLIC5
MmugDNA.11714.1.S1_at	NA	NA	NA	NA	NA		NA	NA	NA	NA		MmugDNA.11714.1.S1_at_NA
MmugDNA.11715.1.S1_at	236788_at	---	---	---	---		---	---	---	---		MmugDNA.11715.1.S1_at_---
MmugDNA.11716.1.S1_s_at	213775_x_at	ZNF638	zinc finger protein 638	chr2p13.2-p13.1		27332	LOC704418	similar to zinc finger protein 638	---		704418	MmugDNA.11716.1.S1_s_at_ZNF638
MmugDNA.11717.1.S1_at	233460_at	---	---	---	---		---	---	---	---		MmugDNA.11717.1.S1_at_---
MmugDNA.11718.1.S1_at	207464_at	AHCYL1	S-adenosylhomocysteine hydrolase-like 1	chr1p13.2		10768	LOC700681	similar to S-adenosylhomocysteine hydrolase-like 1	---		700681	MmugDNA.11718.1.S1_at_AHCYL1
MmugDNA.11719.1.S1_at	207748_at	---	---	---	---		---	---	---	---		MmugDNA.11719.1.S1_at_---
MmugDNA.117.1.S1_at	224060_s_at	DPH5	DPH5 homolog (S. cerevisiae)	chr1p21.2		51611	DPH5	DPH5 homolog	---		712253	MmugDNA.117.1.S1_at_DPH5
MmugDNA.11720.1.S1_at	219765_at	ZNF329	zinc finger protein 329	chr19q13.43		79673	---	---	---	---		MmugDNA.11720.1.S1_at_ZNF329
MmugDNA.11721.1.S1_a_at	220854_at	---	---	---	---		---	---	---	---		MmugDNA.11721.1.S1_a_at_---
MmugDNA.1172.1.S1_at	241216_at	---	---	---	---		---	---	---	---		MmugDNA.1172.1.S1_at_---
MmugDNA.11722.1.S1_at	219002_at	FASTKD1	FAST kinase domains 1	chr2q31		79675	LOC706154	similar to CG31643-PA	---		706154	MmugDNA.11722.1.S1_at_FASTKD1
MmugDNA.11723.1.S1_at	239256_at	---	---	---	---		---	---	---	---		MmugDNA.11723.1.S1_at_---
MmugDNA.11724.1.S1_at	219246_s_at	OGFOD2	2-oxoglutarate and iron-dependent oxygenase domain containing 2	chr12q24.31		79676	---	---	---	---		MmugDNA.11724.1.S1_at_OGFOD2
MmugDNA.11725.1.S1_at	230932_at	---	---	---	---		---	---	---	---		MmugDNA.11725.1.S1_at_---
MmugDNA.11726.1.S1_at	218781_at	SMC6	structural maintenance of chromosomes 6	chr2p24.2		79677	LOC699570	similar to SMC6 protein	---		699570	MmugDNA.11726.1.S1_at_SMC6
MmugDNA.11727.1.S1_at	239541_at	---	---	---	---		---	---	---	---		MmugDNA.11727.1.S1_at_---
MmugDNA.11728.1.S1_at	219768_at	VTCN1	V-set domain containing T cell activation inhibitor 1	chr1p13.1		79679	LOC714123	similar to V-set domain containing T cell activation inhibitor 1	---		714123	MmugDNA.11728.1.S1_at_VTCN1
MmugDNA.11729.1.S1_at	242036_x_at	ATP2B3	ATPase, Ca++ transporting, plasma membrane 3	chrXq28		492	---	---	---	---		MmugDNA.11729.1.S1_at_ATP2B3
MmugDNA.11730.1.S1_at	1557745_at	---	---	---	---		---	---	---	---		MmugDNA.11730.1.S1_at_---
MmugDNA.11731.1.S1_at	217610_at	---	---	---	---		---	---	---	---		MmugDNA.11731.1.S1_at_---
MmugDNA.1173.1.S1_at	238397_at	---	---	---	---		---	---	---	---		MmugDNA.1173.1.S1_at_---
MmugDNA.11732.1.S1_at	241461_at	---	---	---	---		---	---	---	---		MmugDNA.11732.1.S1_at_---
MmugDNA.11733.1.S1_at	1561471_at	LOC441009	Hypothetical LOC441009	chr4p15.33		441009	---	---	---	---		MmugDNA.11733.1.S1_at_LOC441009
MmugDNA.11734.1.S1_at	230737_s_at	LOC387647	patched domain containing 3 pseudogene	chr10p11.23		387647	---	---	---	---		MmugDNA.11734.1.S1_at_LOC387647
MmugDNA.11735.1.S1_at	222793_at	DDX58	DEAD (Asp-Glu-Ala-Asp) box polypeptide 58	chr9p12		23586	---	---	---	---		MmugDNA.11735.1.S1_at_DDX58
MmugDNA.11736.1.S1_at	243707_at	---	---	---	---		---	---	---	---		MmugDNA.11736.1.S1_at_---

MmugDNA.11737.1.S1_at	207744_at	---	---	---	---		---	---	---	---		MmugDNA.11737.1.S1_at_---
MmugDNA.11738.1.S1_at	200733_s_at	PTP4A1	protein tyrosine phosphatase type IVA, member 1	chr6q12		7803	LOC721040	Similar to gene trap ROSA b-geo 22	---		721040	MmugDNA.11738.1.S1_at_PTP4A1
MmugDNA.11739.1.S1_s_at	208616_s_at	PTP4A2	protein tyrosine phosphatase type IVA, member 2	chr1p35		8073	LOC706489	similar to Protein tyrosine phosphatase type IVA protein 2 (Protein-tyrosine phosphatase 4a2) (Protei	---		706489	MmugDNA.11739.1.S1_s_at_PTP4A2
MmugDNA.11740.1.S1_at	1557950_at	SDCCAG1	serologically defined colon cancer antigen 1	chr14q22		9147	---	---	---	---		MmugDNA.11740.1.S1_at_SDCCAG1
MmugDNA.11741.1.S1_at	227790_at	UBE2CBP	ubiquitin-conjugating enzyme E2C binding protein	chr6q14.1		90025	LOC701568	Similar to intraflagellar transport 140	---		701568	MmugDNA.11741.1.S1_at_UBE2CBP
MmugDNA.1174.1.S1_at	215583_at	TMEM63A	Transmembrane protein 63A	chr1q42.12		9725	TMEM63A	transmembrane protein 63A	---		701499	MmugDNA.1174.1.S1_at_TMEM63A
MmugDNA.11742.1.S1_at	207746_at	POLQ	polymerase (DNA directed), theta	chr3q13.33		10721	---	---	---	---		MmugDNA.11742.1.S1_at_POLQ
MmugDNA.11743.1.S1_at	210912_x_at	GSTM4	glutathione S-transferase mu 4	chr1p13.3		2948	GSTM4	glutathione S-transferase mu 4	---		699923	MmugDNA.11743.1.S1_at_GSTM4
MmugDNA.11744.1.S1_at	208077_at	C9orf38	chromosome 9 open reading frame 38	chr9p24.1		29044	---	---	---	---		MmugDNA.11744.1.S1_at_C9orf38
MmugDNA.11745.1.S1_at	219560_at	C22orf29	chromosome 22 open reading frame 29	chr22q11.21		79680	LOC712339	hypothetical protein LOC712339	---		712339	MmugDNA.11745.1.S1_at_C22orf29
MmugDNA.11746.1.S1_at	219874_at	SLC12A8	solute carrier family 12 (potassium/chloride transporters), member 8	chr3q21.2		84561	LOC716531	hypothetical protein LOC716531	---		716531	MmugDNA.11746.1.S1_at_SLC12A8
MmugDNA.11747.1.S1_at	218883_s_at	MLF1IP	MLF1 interacting protein	chr4q35.1		79682	LOC696925	similar to MLF1 interacting protein	---		696925	MmugDNA.11747.1.S1_at_MLF1IP
MmugDNA.11748.1.S1_at	219247_s_at	ZDHHC14	zinc finger, DHHC-type containing 14	chr6q25.3		79683	ZDHHC14	zinc finger, DHHC-type containing 14	---		700610	MmugDNA.11748.1.S1_at_ZDHHC14
MmugDNA.11749.1.S1_at	236665_at	CCDC18	coiled-coil domain containing 18	chr1p22.1		343099	LOC713129	similar to sarcoma antigen NY-SAR-41	---		713129	MmugDNA.11749.1.S1_at_CCDC18
MmugDNA.11750.1.S1_at	231350_at	---	---	---	---		---	---	---	---		MmugDNA.11750.1.S1_at_---
MmugDNA.11751.1.S1_at	1570146_at	---	---	---	---		---	---	---	---		MmugDNA.11751.1.S1_at_---
MmugDNA.11752.1.S1_at	221208_s_at	C11orf61	chromosome 11 open reading frame 61	chr11q24.2		79684	LOC712347	hypothetical protein LOC712347	---		712347	MmugDNA.11752.1.S1_at_C11orf61
MmugDNA.11753.1.S1_at	NA	NA	NA	NA	NA		NA	NA	NA	NA		MmugDNA.11753.1.S1_at_NA
MmugDNA.11754.1.S1_at	204809_at	CLPX	ClpX caseinolytic peptidase X homolog (E. coli)	chr15q22.2-q22.3		10845	CLPX	ClpX caseinolytic protease X homolog	---		716946	MmugDNA.11754.1.S1_at_CLPX
MmugDNA.11755.1.S1_s_at	234735_s_at	USP21	ubiquitin specific peptidase 21	chr1q22		27005	USP21	ubiquitin specific peptidase 21	---		719894	MmugDNA.11755.1.S1_s_at_USP21
MmugDNA.11756.1.S1_at	1555245_s_at	RP1L1	retinitis pigmentosa 1-like 1	chr8p23		94137	---	---	---	---		MmugDNA.11756.1.S1_at_RP1L1
MmugDNA.11757.1.S1_at	1559495_at	---	---	---	---		---	---	---	---		MmugDNA.11757.1.S1_at_---
MmugDNA.11758.1.S1_at	204998_s_at	ATF5	activating transcription factor 5	chr19q13.3		22809	LOC719150	similar to activating transcription factor 5	---		719150	MmugDNA.11758.1.S1_at_ATF5
MmugDNA.11759.1.S1_at	218224_at	PNMA1	paraneoplastic antigen MA1	chr14q24.3		9240	PNMA1	paraneoplastic antigen MA1	---		697618	MmugDNA.11759.1.S1_at_PNMA1
MmugDNA.11760.1.S1_at	1555587_at	PDZRN3	PDZ domain containing ring finger 3	chr3p13		23024	LOC709901	similar to PDZ domain containing RING finger 3	---		709901	MmugDNA.11760.1.S1_at_PDZRN3
MmugDNA.11761.1.S1_at	NA	NA	NA	NA	NA		NA	NA	NA	NA		MmugDNA.11761.1.S1_at_NA
MmugDNA.1176.1.S1_at	227044_at	---	---	---	---		---	---	---	---		MmugDNA.1176.1.S1_at_---
MmugDNA.11762.1.S1_at	239974_at	---	---	---	---		---	---	---	---		MmugDNA.11762.1.S1_at_---
MmugDNA.11763.1.S1_at	219160_s_at	PAPOLG	poly(A) polymerase gamma	chr2p16.1		64895	LOC717781	similar to poly(A) polymerase gamma	---		717781	MmugDNA.11763.1.S1_at_PAPOLG
MmugDNA.11764.1.S1_at	230309_at	---	---	---	---		---	---	---	---		MmugDNA.11764.1.S1_at_---
MmugDNA.11765.1.S1_at	238439_at	ANKRD22	ankyrin repeat domain 22	chr10q23.31		118932	ANKRD22	ankyrin repeat domain 22	---		694240	MmugDNA.11765.1.S1_at_ANKRD22
MmugDNA.11766.1.S1_at	1554360_at	FCHSD2	FCH and double SH3 domains 2	chr11q13.4		9873	LOC717996	similar to FCH and double SH3 domains 2	---		717996	MmugDNA.11766.1.S1_at_FCHSD2
MmugDNA.11767.1.S1_at	225312_at	COMMD6	COMM domain containing 6	chr13q22		170622	COMMD6	COMM domain containing 6	---		695191	MmugDNA.11767.1.S1_at_COMMD6
MmugDNA.11768.1.S1_at	1560306_at	---	---	---	---		---	---	---	---		MmugDNA.11768.1.S1_at_---
MmugDNA.11769.1.S1_at	1553322_s_at	TEAD1	TEA domain family member 1 (SV40 transcriptional enhancer factor)	chr11p15.2		7003	LOC700954	similar to TEA domain family member 1	---		700954	MmugDNA.11769.1.S1_at_TEAD1
MmugDNA.11770.1.S1_at	229019_at	ZNF385B	zinc finger protein 385B	chr2q31.2-q31.3		151126	LOC704204	similar to zinc finger protein 533	---		704204	MmugDNA.11770.1.S1_at_ZNF385B
MmugDNA.11771.1.S1_at	1569959_at	---	---	---	---		---	---	---	---		MmugDNA.11771.1.S1_at_---
MmugDNA.1177.1.S1_at	NA	NA	NA	NA	NA		NA	NA	NA	NA		MmugDNA.1177.1.S1_at_NA
MmugDNA.11772.1.S1_at	220855_at	---	---	---	---		---	---	---	---		MmugDNA.11772.1.S1_at_---
MmugDNA.11773.1.S1_at	229878_at	KIAA1731	KIAA1731	chr11q21		85459	LOC722637	hypothetical protein LOC722637	---		722637	MmugDNA.11773.1.S1_at_KIAA1731
MmugDNA.11774.1.S1_at	231145_at	---	---	---	---		---	---	---	---		MmugDNA.11774.1.S1_at_---
MmugDNA.11775.1.S1_at	201882_x_at	B4GALT1	UDP-Gal:betaGlcNAc beta 1,4- galactosyltransferase, polypeptide 1	chr9p13		2683	B4GALT1	UDP-Gal:betaGlcNAc beta 1,4- galactosyltransferase, polypeptide 1	---		703541	MmugDNA.11775.1.S1_at_B4GALT1
MmugDNA.11776.1.S1_at	220856_x_at	---	---	---	---		---	---	---	---		MmugDNA.11776.1.S1_at_---
MmugDNA.11776.1.S1_x_at	NA	NA	NA	NA	NA		NA	NA	NA	NA		MmugDNA.11776.1.S1_x_at_NA
MmugDNA.11777.1.S1_at	231944_at	ERO1LB	ERO1-like beta (S. cerevisiae)	chr1q42.2-q43		56605	---	---	---	---		MmugDNA.11777.1.S1_at_ERO1LB
MmugDNA.11778.1.S1_at	230795_at	---	---	---	---		---	---	---	---		MmugDNA.11778.1.S1_at_---
MmugDNA.11779.1.S1_at	226772_s_at	SAP30L	SAP30-like	chr5q33.2		79685	LOC714400	similar to Sin3A associated protein p30-like	---		714400	MmugDNA.11779.1.S1_at_SAP30L
MmugDNA.11781.1.S1_at	219563_at	C14orf139	chromosome 14 open reading frame 139	chr14q32.13		79686	---	---	---	---		MmugDNA.11781.1.S1_at_C14orf139
MmugDNA.1178.1.S1_at	242354_at	---	---	---	---		---	---	---	---		MmugDNA.1178.1.S1_at_---
MmugDNA.11782.1.S1_s_at	216611_s_at	SLC6A2	solute carrier family 6 (neurotransmitter transporter, noradrenalin), member 2	chr16q12.2		6530	---	---	---	---		MmugDNA.11782.1.S1_s_at_SLC6A2
MmugDNA.11783.1.S1_at	219362_at	MAK10	MAK10 homolog, amino-acid N-acetyltransferase subunit (S. cerevisiae)	chr9q21.33		60560	LOC709758	Hypothetical protein LOC709758	---		709758	MmugDNA.11783.1.S1_at_MAK10
MmugDNA.11784.1.S1_s_at	223942_x_at	CHST5	carbohydrate (N-acetylglucosamine 6-O) sulfotransferase 5	chr16q22.3		23563	LOC711744	similar to carbohydrate (N-acetylglucosamine 6-O) sulfotransferase 5 isoform 2	---		711744	MmugDNA.11784.1.S1_s_at_CHST5
MmugDNA.11785.1.S1_at	220187_at	STEAP4	STEAP family member 4	chr7q21.12		79689	STEAP4	STEAP family member 4	---		705096	MmugDNA.11785.1.S1_at_STEAP4
MmugDNA.11786.1.S1_at	1556049_at	RTN4	reticulon 4	chr2p16.3		57142	---	---	---	---		MmugDNA.11786.1.S1_at_RTN4
MmugDNA.11787.1.S1_at	219815_at	GAL3ST4	galactose-3-O-sulfotransferase 4	chr7q22.1		79690	LOC711504	similar to galactose-3-O-sulfotransferase 4	---		711504	MmugDNA.11787.1.S1_at_GAL3ST4
MmugDNA.11788.1.S1_at	203292_s_at	VPS11	vacuolar protein sorting 11 homolog (S. cerevisiae)	chr11q23		55823	---	---	---	---		MmugDNA.11788.1.S1_at_VPS11
MmugDNA.11789.1.S1_at	219022_at	C12orf43	chromosome 12 open reading frame 43	chr12q		64897	LOC698821	hypothetical protein LOC698821	---		698821	MmugDNA.11789.1.S1_at_C12orf43
MmugDNA.11790.1.S1_at	216294_s_at	KIAA1109	KIAA1109	chr4q27		84162	LOC707785	similar to CG15133-PA	---		707785	MmugDNA.11790.1.S1_at_KIAA1109
MmugDNA.11791.1.S1_at	219661_at	RANBP17	RAN binding protein 17	chr5q34		64901	LOC701214	similar to RAN binding protein 17	---		701214	MmugDNA.11791.1.S1_at_RANBP17
MmugDNA.1179.1.S1_at	221232_s_at	ANKRD2	ankyrin repeat domain 2 (stretch responsive muscle)	chr10q23.31-q23.32		26287	ANKRD2	ankyrin repeat domain 2 (stretch responsive muscle)	---		707097	MmugDNA.1179.1.S1_at_ANKRD2
MmugDNA.11792.1.S1_at	224182_x_at	SEMA6B	sema domain, transmembrane domain (TM), and cytoplasmic domain, (semaphorin) 6B	chr19p13.3		10501	LOC694125	similar to semaphorin 6B isoform 3 precursor	---		694125	MmugDNA.11792.1.S1_at_SEMA6B
MmugDNA.11793.1.S1_at	226957_x_at	RALBP1	ralA binding protein 1	chr18p11.3		10928	RALBP1	ralA binding protein 1	---		705571	MmugDNA.11793.1.S1_at_RALBP1
MmugDNA.11794.1.S1_at	1556636_at	---	---	---	---		---	---	---	---		MmugDNA.11794.1.S1_at_---
MmugDNA.11795.1.S1_at	219528_s_at	BCL11B	B-cell CLL/lymphoma 11B (zinc finger protein)	chr14q32.2		64919	LOC708930	Similar to polypeptide N-acetylgalactosaminyltransferase 1	---		708930	MmugDNA.11795.1.S1_at_BCL11B
MmugDNA.11796.1.S1_at	214999_s_at	RAB11FIP3	RAB11 family interacting protein 3 (class II)	chr16p13.3		9727	---	---	---	---		MmugDNA.11796.1.S1_at_RAB11FIP3
MmugDNA.11797.1.S1_at	218658_s_at	ACTR8	ARP8 actin-related protein 8 homolog (yeast)	---		93973	ACTR8	ARP8 actin-related protein 8 homolog (yeast)	---		693539	MmugDNA.11797.1.S1_at_ACTR8
MmugDNA.11798.1.S1_at	238189_at	SIN3A	SIN3 homolog A, transcription regulator (yeast)	chr15q24.2		25942	---	---	---	---		MmugDNA.11798.1.S1_at_SIN3A
MmugDNA.11799.1.S1_at	219342_at	CASD1	CAS1 domain containing 1	chr7q21.3		64921	CASD1	CAS1 domain containing 1	---		700222	MmugDNA.11799.1.S1_at_CASD1
MmugDNA.11800.1.S1_at	232304_at	PELI1	Pellino homolog 1 (Drosophila)	chr2p13.3		57162	---	---	---	---		MmugDNA.11800.1.S1_at_PELI1
MmugDNA.11801.1.S1_at	1553457_at	CXorf62	chromosome X open reading frame 62	chrXq13.1		439943	---	---	---	---		MmugDNA.11801.1.S1_at_CXorf62
MmugDNA.1180.1.S1_at	244001_at	NAP1L4	nucleosome assembly protein 1-like 4	chr11p15.5		4676	LOC698864	Similar to FUN14 domain containing 2	---		698864	MmugDNA.1180.1.S1_at_NAP1L4
MmugDNA.11802.1.S1_at	1555673_at	KRTAP2-4	keratin associated protein 2-4	chr17q12-q21		85294	LOC703663	similar to keratin associated protein 2-4	---		703663	MmugDNA.11802.1.S1_at_KRTAP2-4
MmugDNA.11803.1.S1_at	242672_at	---	---	---	---		---	---	---	---		MmugDNA.11803.1.S1_at_---
MmugDNA.11804.1.S1_at	211944_at	BAT2D1	BAT2 domain containing 1	chr1q23.3		23215	BAT2D1	BAT2 domain containing 1	---		704297	MmugDNA.11804.1.S1_at_BAT2D1
MmugDNA.11805.1.S1_at	220857_at	---	---	---	---		---	---	---	---		MmugDNA.11805.1.S1_at_---
MmugDNA.11806.1.S1_at	1554785_at	CCDC82	coiled-coil domain containing 82	chr11q21		79780	---	---	---	---		MmugDNA.11806.1.S1_at_CCDC82
MmugDNA.11807.1.S1_at	NA	NA	NA	NA	NA		NA	NA	NA	NA		MmugDNA.11807.1.S1_at_NA
MmugDNA.11807.1.S1_s_at	214055_x_at	BAT2D1	BAT2 domain containing 1	chr1q23.3		23215	BAT2D1	BAT2 domain containing 1	---		704297	MmugDNA.11807.1.S1_s_at_BAT2D1
MmugDNA.11808.1.S1_at	211947_s_at	BAT2D1	BAT2 domain containing 1	chr1q23.3		23215	BAT2D1	BAT2 domain containing 1	---		704297	MmugDNA.11808.1.S1_at_BAT2D1
MmugDNA.11809.1.S1_at	211946_s_at	BAT2D1	BAT2 domain containing 1	chr1q23.3		23215	BAT2D1	BAT2 domain containing 1	---		704297	MmugDNA.11809.1.S1_at_BAT2D1
MmugDNA.11810.1.S1_at	233827_s_at	SUPT16H	suppressor of Ty 16 homolog (S. cerevisiae)	chr14q11.2		11198	LOC708066	similar to chromatin-specific transcription elongation factor large subunit	---		708066	MmugDNA.11810.1.S1_at_SUPT16H
MmugDNA.11811.1.S1_at	219178_at	QTRTD1	queuine tRNA-ribosyltransferase domain containing 1	chr3q13.31		79691	LOC694354	Similar to Trc8 CG2304-PA, isoform A	---		694354	MmugDNA.11811.1.S1_at_QTRTD1
MmugDNA.1181.1.S1_at	220977_x_at	EPB41L5	erythrocyte membrane protein band 4.1 like 5	chr2q14.2		57669	---	---	---	---		MmugDNA.1181.1.S1_at_EPB41L5
MmugDNA.11812.1.S1_x_at	1553136_at	KCTD18	potassium channel tetramerisation domain containing 18	chr2q33.1		130535	---	---	---	---		MmugDNA.11812.1.S1_x_at_KCTD18
MmugDNA.11813.1.S1_at	236787_at	---	---	---	---		---	---	---	---		MmugDNA.11813.1.S1_at_---
MmugDNA.11814.1.S1_at	219376_at	ZNF322B	zinc finger protein 322B	chr9q22.33		387328	ZNF322A	zinc finger protein 322A	---		701098	MmugDNA.11814.1.S1_at_ZNF322B
MmugDNA.11815.1.S1_at	1569418_at	---	---	---	---		---	---	---	---		MmugDNA.11815.1.S1_at_---
MmugDNA.11816.1.S1_at	1569114_at	---	---	---	---		---	---	---	---		MmugDNA.11816.1.S1_at_---
MmugDNA.11817.1.S1_at	218647_s_at	YRDC	yrdC domain containing (E. coli)	chr1p34.3		79693	---	---	---	---		MmugDNA.11817.1.S1_at_YRDC
MmugDNA.11818.1.S1_at	230469_at	RTKN2	rhotekin 2	chr10q21.2		219790	---	---	---	---		MmugDNA.11818.1.S1_at_RTKN2
MmugDNA.11819.1.S1_at	203511_s_at	TRAPPC3	trafficking protein particle complex 3	chr1p34.3		27095	LOC713705	similar to Trafficking protein particle complex subunit 3 (BET3 homolog)	---		713705	MmugDNA.11819.1.S1_at_TRAPPC3
MmugDNA.118.1.S1_at	243590_at	---	---	---	---		---	---	---	---		MmugDNA.118.1.S1_at_---
MmugDNA.11820.1.S1_at	218976_at	DNAJC12	DnaJ (Hsp40) homolog, subfamily C, member 12	chr10q22.1		56521	DNAJC12	DnaJ (Hsp40) homolog, subfamily C, member 12	---		695795	MmugDNA.11820.1.S1_at_DNAJC12
MmugDNA.11821.1.S1_at	219003_s_at	MANEA	mannosidase, endo-alpha	chr6q16.1		79694	---	---	---	---		MmugDNA.11821.1.S1_at_MANEA
MmugDNA.1182.1.S1_at	224340_at	---	---	---	---		---	---	---	---		MmugDNA.1182.1.S1_at_---
MmugDNA.11822.1.S1_at	239094_at	LOC730961	hypothetical protein LOC730961	---		730961	---	---	---	---		MmugDNA.11822.1.S1_at_LOC730961
MmugDNA.11823.1.S1_at	242701_at	TBRG1	transforming growth factor beta regulator 1	chr11q24.2		84897	LOC711895	similar to transforming growth factor beta regulated gene 1	---		711895	MmugDNA.11823.1.S1_at_TBRG1
MmugDNA.11824.1.S1_at	220376_at	LRRC19	leucine rich repeat containing 19	chr9p21.2		64922	LRRC19	leucine rich repeat containing 19	---		707657	MmugDNA.11824.1.S1_at_LRRC19
MmugDNA.11825.1.S1_at	221271_at	IL21	interleukin 21	chr4q26-q27		59067	LOC708125	similar to interleukin 21	---		708125	MmugDNA.11825.1.S1_at_IL21
MmugDNA.11826.1.S1_at	NA	NA	NA	NA	NA		NA	NA	NA	NA		MmugDNA.11826.1.S1_at_NA
MmugDNA.11827.1.S1_at	1566844_at	PER4	period 4 pseudogene	chr7p21.3		168741	---	---	---	---		MmugDNA.11827.1.S1_at_PER4
MmugDNA.11828.1.S1_at	NA	NA	NA	NA	NA		NA	NA	NA	NA		MmugDNA.11828.1.S1_at_NA
MmugDNA.11828.1.S1_s_at	218989_x_at	SLC30A5	solute carrier family 30 (zinc transporter), member 5	chr5q12.1		64924	---	---	---	---		MmugDNA.11828.1.S1_s_at_SLC30A5
MmugDNA.11829.1.S1_at	219893_at	CCDC71	coiled-coil domain containing 71	chr3p21.31		64925	---	---	---	---		MmugDNA.11829.1.S1_at_CCDC71
MmugDNA.11830.1.S1_at	219838_at	TTC23	tetratricopeptide repeat domain 23	chr15q26.3		64927	LOC708530	similar to tetratricopeptide repeat domain 23	---		708530	MmugDNA.11830.1.S1_at_TTC23
MmugDNA.11831.1.S1_at	218994_s_at	STAG3L4	stromal antigen 3-like 4	chr7p11.2-q11.2		64940	---	---	---	---		MmugDNA.11831.1.S1_at_STAG3L4
MmugDNA.1183.1.S1_at	1552501_a_at	GPBAR1	G protein-coupled bile acid receptor 1	chr2q35		151306	GPBAR1	G protein-coupled bile acid receptor 1	---		697937	MmugDNA.1183.1.S1_at_GPBAR1
MmugDNA.11832.1.S1_at	1562935_at	---	---	---	---		---	---	---	---		MmugDNA.11832.1.S1_at_---

MmugDNA.11833.1.S1_at	1569296_a_at	LOC493754	RAB guanine nucleotide exchange factor (GEF) 1 pseudogene	chr7q11.21	493754	---	---	---	---	MmugDNA.11833.1.S1_at_LOC493754
MmugDNA.11834.1.S1_at	213105_s_at	C16orf42	chromosome 16 open reading frame 42	chr16p13.3	115939	LOC677691	hypothetical protein MGC24381	---	677691	MmugDNA.11834.1.S1_at_C16orf42
MmugDNA.11835.1.S1_at	237631_at	---	---	---	---	---	---	---	---	MmugDNA.11835.1.S1_at_---
MmugDNA.11836.1.S1_at	213961_s_at	---	---	---	---	---	---	---	---	MmugDNA.11836.1.S1_at_---
MmugDNA.11837.1.S1_at	220404_at	GPR97	G protein-coupled receptor 97	chr16q13	222487	---	---	---	---	MmugDNA.11837.1.S1_at_GPR97
MmugDNA.11838.1.S1_at	220858_at	SORBS2	sorbin and SH3 domain containing 2	chr4q35.1	8470	---	---	---	---	MmugDNA.11838.1.S1_at_SORBS2
MmugDNA.11839.1.S1_at	243379_at	---	---	---	---	---	---	---	---	MmugDNA.11839.1.S1_at_---
MmugDNA.11840.1.S1_at	207465_at	---	---	---	---	---	---	---	---	MmugDNA.11840.1.S1_at_---
MmugDNA.11841.1.S1_at	234081_at	---	---	---	---	---	---	---	---	MmugDNA.11841.1.S1_at_---
MmugDNA.1184.1.S1_at	1560253_at	LHX9	LIM homeobox 9	chr1q31-q32	56956	LHX9	LIM homeobox 9	---	710636	MmugDNA.1184.1.S1_at_LHX9
MmugDNA.11842.1.S1_at	242023_at	ABHD4	Abhydrolase domain containing 4	chr14q11.2	63874	---	---	---	---	MmugDNA.11842.1.S1_at_ABHD4
MmugDNA.11843.1.S1_at	218885_s_at	GALNT12	UDP-N-acetyl-alpha-D-galactosamine:polypeptide N-acetylgalactosaminyltransferase 12 (GalNAc-T1	chr9q22.33	79695	LOC715128	similar to UDP-N-acetyl-alpha-D-galactosamine:polypeptide N-acetylgalactosaminyltransferase 12 (G	---	715128	MmugDNA.11843.1.S1_at_GALNT12
MmugDNA.11844.1.S1_at	243249_at	---	---	---	---	---	---	---	---	MmugDNA.11844.1.S1_at_---
MmugDNA.11845.1.S1_at	220612_at	---	---	---	---	---	---	---	---	MmugDNA.11845.1.S1_at_---
MmugDNA.11846.1.S1_at	220107_s_at	FAM164C	family with sequence similarity 164, member C	chr14q24.3	79696	FAM164C	family with sequence similarity 164, member C	---	701845	MmugDNA.11846.1.S1_at_FAM164C
MmugDNA.11847.1.S1_at	1566469_at	---	---	---	---	---	---	---	---	MmugDNA.11847.1.S1_at_---
MmugDNA.11848.1.S1_at	219526_at	C14orf169	chromosome 14 open reading frame 169	chr14q24.3	79697	C7H14orf169	chromosome 14 open reading frame 169 ortholog	---	696773	MmugDNA.11848.1.S1_at_C14orf169
MmugDNA.11849.1.S1_at	1563657_at	PLXND1	plexin D1	chr3q21.3	23129	---	---	---	---	MmugDNA.11849.1.S1_at_PLXND1
MmugDNA.11850.1.S1_at	219877_at	ZMAT4	zinc finger, matrin type 4	chr8p11.21	79698	ZMAT4	similar to zinc finger, matrin type 4	---	708069	MmugDNA.11850.1.S1_at_ZMAT4
MmugDNA.11851.1.S1_at	NA	NA	NA	NA	NA	NA	NA	NA	NA	MmugDNA.11851.1.S1_at_NA
MmugDNA.11851.1.S1_x_at	NA	NA	NA	NA	NA	NA	NA	NA	NA	MmugDNA.11851.1.S1_x_at_NA
MmugDNA.1185.1.S1_at	211306_s_at	FCAR	Fc fragment of IgA, receptor for	chr19q13.2-q13.4	2204	FCAR	Fc fragment of IgA, receptor for	---	664734	MmugDNA.1185.1.S1_at_FCAR
MmugDNA.11852.1.S1_at	215429_s_at	ZNF428	zinc finger protein 428	chr19q13.31	126299	LOC710496	hypothetical protein LOC710496	---	710496	MmugDNA.11852.1.S1_at_ZNF428
MmugDNA.11853.1.S1_at	220772_at	BPESC1	blepharophimosis, epicanthus inversus and ptosis, candidate 1	chr3q23	60467	---	---	---	---	MmugDNA.11853.1.S1_at_BPESC1
MmugDNA.11854.1.S1_at	240101_at	---	---	---	---	---	---	---	---	MmugDNA.11854.1.S1_at_---
MmugDNA.11855.1.S1_at	219881_s_at	---	---	---	---	LOC708645	hypothetical protein LOC708645	---	708645	MmugDNA.11855.1.S1_at_---
MmugDNA.11856.1.S1_at	220722_s_at	SLC5A7	solute carrier family 5 (choline transporter), member 7	chr2q12	60482	LOC714093	similar to solute carrier family 5 (choline transporter), member 7	---	714093	MmugDNA.11856.1.S1_at_SLC5A7
MmugDNA.11857.1.S1_at	1568592_at	TRIM69	tripartite motif-containing 69	chr15q21.1	140691	---	---	---	---	MmugDNA.11857.1.S1_at_TRIM69
MmugDNA.11858.1.S1_at	220142_at	HAPLN2	hyaluronan and proteoglycan link protein 2	chr1q23.1	60484	LOC718500	similar to brain link protein-1	---	718500	MmugDNA.11858.1.S1_at_HAPLN2
MmugDNA.11859.1.S1_at	218051_s_at	NT5DC2	5'-nucleotidase domain containing 2	chr3p21.1	64943	NT5DC2	5'-nucleotidase domain containing 2	---	696679	MmugDNA.11859.1.S1_at_NT5DC2
MmugDNA.11860.1.S1_at	237967_at	HAL	Histidine ammonia-lyase	chr12q22-q24.1	3034	---	---	---	---	MmugDNA.11860.1.S1_at_HAL
MmugDNA.11861.1.S1_at	218276_s_at	SAV1	salvador homolog 1 (Drosophila)	chr14q13-q23	60485	SAV1	salvador homolog 1 (Drosophila)	---	706424	MmugDNA.11861.1.S1_at_SAV1
MmugDNA.1186.1.S1_at	233142_at	---	---	---	---	---	---	---	---	MmugDNA.1186.1.S1_at_---
MmugDNA.11862.1.S1_at	238782_at	---	---	---	---	---	---	---	---	MmugDNA.11862.1.S1_at_---
MmugDNA.11863.1.S1_at	204365_s_at	REEP1	receptor accessory protein 1	chr2p11.2	65055	LOC697390	similar to receptor expression enhancing protein 1	---	697390	MmugDNA.11863.1.S1_at_REEP1
MmugDNA.11864.1.S1_at	204617_s_at	ACD	adrenocortical dysplasia homolog (mouse)	chr16q22.1	65057	ACD	adrenocortical dysplasia homolog (mouse)	---	699230	MmugDNA.11864.1.S1_at_ACD
MmugDNA.11865.1.S1_at	211261_at	NUP214	nucleoporin 214kDa	chr9q34.1	8021	LOC710478	Similar to 60S ribosomal protein L7	---	710478	MmugDNA.11865.1.S1_at_NUP214
MmugDNA.11866.1.S1_at	241686_x_at	---	---	---	---	---	---	---	---	MmugDNA.11866.1.S1_at_---
MmugDNA.11867.1.S1_at	1556645_s_at	---	---	---	---	---	---	---	---	MmugDNA.11867.1.S1_at_---
MmugDNA.11868.1.S1_at	224622_at	TBC1D14	TBC1 domain family, member 14	chr4p16.1	57533	LOC722719	similar to TBC1 domain family, member 14	---	722719	MmugDNA.11868.1.S1_at_TBC1D14
MmugDNA.11869.1.S1_at	224799_at	NDFIP2	Nedd4 family interacting protein 2	chr13q31.1	54602	NDFIP2	Nedd4 family interacting protein 2	---	698464	MmugDNA.11869.1.S1_at_NDFIP2
MmugDNA.11870.1.S1_at	NA	NA	NA	NA	NA	NA	NA	NA	NA	MmugDNA.11870.1.S1_at_NA
MmugDNA.11871.1.S1_at	224802_at	NDFIP2	Nedd4 family interacting protein 2	chr13q31.1	54602	NDFIP2	Nedd4 family interacting protein 2	---	698464	MmugDNA.11871.1.S1_at_NDFIP2
MmugDNA.1187.1.S1_at	1557433_at	---	---	---	---	---	---	---	---	MmugDNA.1187.1.S1_at_---
MmugDNA.11872.1.S1_at	1558732_at	MAP4K4	mitogen-activated protein kinase kinase kinase kinase 4	chr2q11.2-q12	9448	---	---	---	---	MmugDNA.11872.1.S1_at_MAP4K4
MmugDNA.11873.1.S1_s_at	224801_at	NDFIP2	Nedd4 family interacting protein 2	chr13q31.1	54602	NDFIP2	Nedd4 family interacting protein 2	---	698464	MmugDNA.11873.1.S1_s_at_NDFIP2
MmugDNA.11874.1.S1_at	220644_at	---	---	---	---	LOC700929	hypothetical protein LOC700929	---	700929	MmugDNA.11874.1.S1_at_---
MmugDNA.11875.1.S1_at	233338_at	FLJ26056 /// LOC28486	hypothetical protein LOC375127 /// hypothetical LOC284861 /// hypothetical LOC729461	chr22q11.21	284861 /// 375127 /// 7	---	---	---	---	MmugDNA.11875.1.S1_at_FLJ26056 ///
MmugDNA.11876.1.S1_at	227464_at	ACSF3	acyl-CoA synthetase family member 3	chr16q24.3	197322	---	---	---	---	MmugDNA.11876.1.S1_at_ACSF3
MmugDNA.11877.1.S1_at	219254_at	C17orf101	chromosome 17 open reading frame 101	chr17q25.3	79701	LOC716172	hypothetical protein LOC716172	---	716172	MmugDNA.11877.1.S1_at_C17orf101
MmugDNA.11878.1.S1_at	242814_at	SERPINB9	serpin peptidase inhibitor, clade B (ovalbumin), member 9	chr6p25	5272	SERPINB9	serpin peptidase inhibitor, clade B (ovalbumin), member 9	---	707580	MmugDNA.11878.1.S1_at_SERPINB9
MmugDNA.11879.1.S1_at	218471_s_at	BBS1	Bardet-Biedl syndrome 1	chr11q13.1	582	LOC709730	Similar to vang-like 1	---	709730	MmugDNA.11879.1.S1_at_BBS1
MmugDNA.11880.1.S1_at	228207_at	---	---	---	---	---	---	---	---	MmugDNA.11880.1.S1_at_---
MmugDNA.11881.1.S1_at	229445_at	CYBA	cytochrome b-245, alpha polypeptide	chr16q24	1535	LOC696748	similar to cytochrome b, alpha polypeptide	---	696748	MmugDNA.11881.1.S1_at_CYBA
MmugDNA.1188.1.S1_at	232881_at	GNASAS	GNAS antisense RNA (non-protein coding)	chr20q13.32	149775	---	---	---	---	MmugDNA.1188.1.S1_at_GNASAS
MmugDNA.11882.1.S1_at	204922_at	C11orf80	chromosome 11 open reading frame 80	chr11q13.1	79703	LOC721782	hypothetical protein LOC721782	---	721782	MmugDNA.11882.1.S1_at_C11orf80
MmugDNA.11883.1.S1_at	1553336_a_at	MIER3	mesoderm induction early response 1, family member 3	chr5q11.2	166968	LOC711063	similar to CG1620-PA	---	711063	MmugDNA.11883.1.S1_at_MIER3
MmugDNA.11884.1.S1_at	220420_at	LMAN1L	lectin, mannose-binding, 1 like	chr15q24.1	79748	LOC709457	similar to ERGIC-53-like protein precursor (ERGIC53-like protein) (Lectin, mannose-binding 1-like) (L	---	709457	MmugDNA.11884.1.S1_at_LMAN1L
MmugDNA.11885.1.S1_at	236664_at	AKT2	v-akt murine thymoma viral oncogene homolog 2	chr19q13.1-q13.2	208	---	---	---	---	MmugDNA.11885.1.S1_at_AKT2
MmugDNA.11886.1.S1_at	218877_s_at	TRMT11	tRNA methyltransferase 11 homolog (S. cerevisiae)	chr6q11.1-q22.33	60487	LOC712712	similar to RIKEN cDNA 2410075D05	---	712712	MmugDNA.11886.1.S1_at_TRMT11
MmugDNA.11887.1.S1_at	1566001_at	---	---	---	---	---	---	---	---	MmugDNA.11887.1.S1_at_---
MmugDNA.11888.1.S1_at	239674_at	---	---	---	---	---	---	---	---	MmugDNA.11888.1.S1_at_---
MmugDNA.11889.1.S1_at	218133_s_at	NIF3L1	NIF3 NGG1 interacting factor 3-like 1 (S. pombe)	chr2q33	60491	LOC713997	Similar to LR8 protein	---	713997	MmugDNA.11889.1.S1_at_NIF3L1
MmugDNA.11890.1.S1_at	218199_s_at	NOL6	nucleolar protein family 6 (RNA-associated)	chr9p13.3	65083	NOL6	nucleolar protein family 6 (RNA-associated)	---	702596	MmugDNA.11890.1.S1_at_NOL6
MmugDNA.11891.1.S1_at	225632_s_at	RAB43	RAB43, member RAS oncogene family	chr3q21.3	339122	---	---	---	---	MmugDNA.11891.1.S1_at_RAB43
MmugDNA.1189.1.S1_at	238688_at	TPM1	Tropomyosin 1 (alpha)	chr15q22.1	7168	TPM1	tropomyosin 1 (alpha)	---	705236	MmugDNA.1189.1.S1_at_TPM1
MmugDNA.11892.1.S1_at	215588_x_at	RIOK3	RIO kinase 3 (yeast)	chr18q11.2	8780	---	---	---	---	MmugDNA.11892.1.S1_at_RIOK3
MmugDNA.11893.1.S1_at	228135_at	C1orf52	chromosome 1 open reading frame 52	chr1p22.3	148423	LOC711232	hypothetical protein LOC711232	---	711232	MmugDNA.11893.1.S1_at_C1orf52
MmugDNA.11894.1.S1_at	237160_at	CCDC83	coiled-coil domain containing 83	chr11q14.1-q14.2	220047	CCDC83	coiled-coil domain containing 83	---	703306	MmugDNA.11894.1.S1_at_CCDC83
MmugDNA.11895.1.S1_at	208656_s_at	CCNI	cyclin I	chr4q21.1	10983	CCNI	cyclin I	---	677687	MmugDNA.11895.1.S1_at_CCNI
MmugDNA.11896.1.S1_at	240192_at	FLJ45983	hypothetical LOC399717	chr10p14	399717	---	---	---	---	MmugDNA.11896.1.S1_at_FLJ45983
MmugDNA.11897.1.S1_at	1568957_x_at	SRGAP2P1	SLIT-ROBO Rho GTPase activating protein 2 pseudogene 1	chr1p11.2	653464	---	---	---	---	MmugDNA.11897.1.S1_at_SRGAP2P1
MmugDNA.11898.1.S1_at	213131_at	OLFM1	olfactomedin 1	chr9q34.3	10439	LOC712348	similar to olfactomedin related ER localized protein isoform 1	---	712348	MmugDNA.11898.1.S1_at_OLFM1
MmugDNA.11899.1.S1_at	220729_at	---	---	---	---	---	---	---	---	MmugDNA.11899.1.S1_at_---
MmugDNA.11900.1.S1_at	226587_at	SNRPN	small nuclear ribonucleoprotein polypeptide N	chr15q11.2	6638	---	---	---	---	MmugDNA.11900.1.S1_at_SNRPN
MmugDNA.11901.1.S1_at	225793_at	LIX1L	Lix1 homolog (mouse)-like	chr1q21.1	128077	---	---	---	---	MmugDNA.11901.1.S1_at_LIX1L
MmugDNA.1190.1.S1_at	238784_at	DPY19L2	dpy-19-like 2 (C. elegans)	chr12q14.2	283417	LOC699789 /// LOC7078	similar to dpy-19-like 1, like /// similar to dpy-19-like 1, like /// similar to dpy-19-like 1, like	---	699789 /// 707898 /// 7093	MmugDNA.1190.1.S1_at_DPY19L2
MmugDNA.11902.1.S1_at	220862_s_at	USP22	ubiquitin specific peptidase 22	chr17p11.2	23326	---	---	---	---	MmugDNA.11902.1.S1_at_USP22
MmugDNA.11903.1.S1_at	226591_at	SNRPN	small nuclear ribonucleoprotein polypeptide N	chr15q11.2	6638	PTTG1	Similar to Securin (Pituitary tumor-transforming protein 1) (Tumor transforming protein 1) (Esp1-asso	---	697601	MmugDNA.11903.1.S1_at_SNRPN
MmugDNA.11904.1.S1_at	1560472_at	LOC338588	hypothetical LOC338588	chr10p15.1	338588	---	---	---	---	MmugDNA.11904.1.S1_at_LOC338588
MmugDNA.11905.1.S1_at	222284_at	---	---	---	---	---	---	---	---	MmugDNA.11905.1.S1_at_---
MmugDNA.11906.1.S1_at	213121_at	SNRNP70	small nuclear ribonucleoprotein 70kDa (U1)	chr19q13.3	6625	---	---	---	---	MmugDNA.11906.1.S1_at_SNRNP70
MmugDNA.11907.1.S1_s_at	NA	NA	NA	NA	NA	NA	NA	NA	NA	MmugDNA.11907.1.S1_s_at_NA
MmugDNA.11908.1.S1_s_at	200008_s_at	GDI2	GDP dissociation inhibitor 2	chr10p15	2665	GDI2	GDP dissociation inhibitor 2	---	712510	MmugDNA.11908.1.S1_s_at_GDI2
MmugDNA.11909.1.S1_s_at	201167_x_at	ARHGDIA	Rho GDP dissociation inhibitor (GDI) alpha	chr17q25.3	396	NECAP2	NECAP endocytosis associated 2	---	698687	MmugDNA.11909.1.S1_s_at_ARHGDIA
MmugDNA.11911.1.S1_at	214646_at	HIST1H3J	Histone cluster 1, H3j	chr6p22-p21.3	8356	---	---	---	---	MmugDNA.11911.1.S1_at_HIST1H3J
MmugDNA.1191.1.S1_at	1559949_at	---	---	---	---	---	---	---	---	MmugDNA.1191.1.S1_at_---
MmugDNA.11912.1.S1_at	215110_at	MBL1P1	mannose-binding lectin (protein A) 1, pseudogene 1	chr10q22.2-q22.3	8512	MBP-A	mannose-binding protein A	---	707965	MmugDNA.11912.1.S1_at_MBL1P1
MmugDNA.11913.1.S1_at	214069_at	ACSM2A /// ACSM2B	acyl-CoA synthetase medium-chain family member 2A /// acyl-CoA synthetase medium-chain family n	chr16p12.3	123876 /// 348158	LOC696244	similar to xenobiotic/medium-chain fatty acid:CoA ligase	---	696244	MmugDNA.11913.1.S1_at_ACSM2A ///
MmugDNA.11914.1.S1_at	200076_s_at	C19orf50	chromosome 19 open reading frame 50	chr19p13.11	79036	LOC720235	similar to CG10681-PA	---	720235	MmugDNA.11914.1.S1_at_C19orf50
MmugDNA.11915.1.S1_at	227285_at	C1orf51	chromosome 1 open reading frame 51	chr1q21.2	148523	LOC706693	hypothetical protein LOC706693	---	706693	MmugDNA.11915.1.S1_at_C1orf51
MmugDNA.11916.1.S1_s_at	NA	NA	NA	NA	NA	NA	NA	NA	NA	MmugDNA.11916.1.S1_s_at_NA
MmugDNA.11918.1.S1_s_at	225980_at	C14orf43	chromosome 14 open reading frame 43	chr14q24.3	91748	---	---	---	---	MmugDNA.11918.1.S1_s_at_C14orf43
MmugDNA.11919.1.S1_at	205555_s_at	MSX2	msh homeobox 2	chr5q34-q35	4488	LOC694227	similar to msh homeobox 2	---	694227	MmugDNA.11919.1.S1_at_MSX2
MmugDNA.119.1.S1_at	239250_at	ZNF542	zinc finger protein 542	chr19q13.43	147947	---	---	---	---	MmugDNA.119.1.S1_at_ZNF542
MmugDNA.11920.1.S1_at	237249_at	KCNQ1OT1	KCNQ1 overlapping transcript 1 (non-protein coding)	chr11p15	10984	---	---	---	---	MmugDNA.11920.1.S1_at_KCNQ1OT1
MmugDNA.11921.1.S1_at	225375_at	TMEM199	transmembrane protein 199	chr17q11.2	147007	LOC708298	similar to CG7071-PA, isoform A	---	708298	MmugDNA.11921.1.S1_at_TMEM199
MmugDNA.1192.1.S1_at	1562754_at	LOC339260	hypothetical protein LOC339260	chr17p11.2	339260	---	---	---	---	MmugDNA.1192.1.S1_at_LOC339260
MmugDNA.11922.1.S1_at	1558452_at	TMEM144	transmembrane protein 144	chr4q32.1	55314	---	---	---	---	MmugDNA.11922.1.S1_at_TMEM144
MmugDNA.11923.1.S1_at	225374_at	TMEM199	transmembrane protein 199	chr17q11.2	147007	LOC708298	similar to CG7071-PA, isoform A	---	708298	MmugDNA.11923.1.S1_at_TMEM199
MmugDNA.11924.1.S1_at	1564169_at	---	---	---	---	---	---	---	---	MmugDNA.11924.1.S1_at_---
MmugDNA.11925.1.S1_at	234302_s_at	ALKBH5	alkB, alkylation repair homolog 5 (E. coli)	chr17p11.2	54890	LOC700205	hypothetical protein LOC700205	---	700205	MmugDNA.11925.1.S1_at_ALKBH5
MmugDNA.11926.1.S1_at	240148_at	MSH6	MutS homolog 6 (E. coli)	chr2p16	2956	---	---	---	---	MmugDNA.11926.1.S1_at_MSH6
MmugDNA.11927.1.S1_at	1557996_at	LOC100132832 /// PMS	postmeiotic segregation increased 2-like 5-like /// postmeiotic segregation increased 2-like 1 pseudog	chr7q11-q22 /// chr7q11.23 /// ch	100132832 /// 5379 ///	---	---	---	---	MmugDNA.11927.1.S1_at_LOC100132
MmugDNA.11928.1.S1_at	234443_at	---	---	---	---	---	---	---	---	MmugDNA.11928.1.S1_at_---
MmugDNA.11929.1.S1_at	227817_at	PRKCB	protein kinase C, beta	chr16p11.2	5579	LOC696005	Similar to Histone H1.2 (H1d)	---	696005	MmugDNA.11929.1.S1_at_PRKCB
MmugDNA.11930.1.S1_at	1552862_at	C1orf104	chromosome 1 open reading frame 104	chr1q22	284618	---	---	---	---	MmugDNA.11930.1.S1_at_C1orf104
MmugDNA.11931.1.S1_at	227824_at	PRKCB	protein kinase C, beta	chr16p11.2	5579	AMMECR1	Alport syndrome, mental retardation, midface hypoplasia and elliptocytosis chromosomal region gene	---	705858	MmugDNA.11931.1.S1_at_PRKCB

MmugDNA.1193.1.S1_at	1553285_s_at	RAD9B	RAD9 homolog B (S. pombe)	chr12q24.11	144715	LOC711050	similar to RAD9 homolog B	---	711050	MmugDNA.1193.1.S1_at_RAD9B
MmugDNA.11932.1.S1_at	231037_at	---	---	---	---	---	---	---	---	MmugDNA.11932.1.S1_at_---
MmugDNA.11933.1.S1_at	230507_at	LOC100130360	hypothetical protein LOC100130360	chr6p22.3	100130360	---	---	---	---	MmugDNA.11933.1.S1_at_LOC100130
MmugDNA.11934.1.S1_at	208631_s_at	HADHA	hydroxyacyl-Coenzyme A dehydrogenase/3-ketoacyl-Coenzyme A thiolase/enoyl-Coenzyme A hydrat	chr2p23	3030	HADHA	hydroxyacyl-Coenzyme A dehydrogenase/3-ketoacyl-Coenzyme A thiolase/enoyl-Coenzyme A hydrat	---	695975	MmugDNA.11934.1.S1_at_HADHA
MmugDNA.11935.1.S1_x_at	217598_at	---	---	---	---	---	---	---	---	MmugDNA.11935.1.S1_x_at_---
MmugDNA.11936.1.S1_at	229939_at	---	---	---	---	GHRL	Ghrelin	---	574280	MmugDNA.11936.1.S1_at_---
MmugDNA.11937.1.S1_at	1553998_at	DMRTC1 /// DMRTC1B	DMRT-like family C1 /// DMRT-like family C1B	chrXq13.1	63947 /// 728656	DMRTC1	similar to DMRT-like family C1	---	704619	MmugDNA.11937.1.S1_at_DMRTC1 ///
MmugDNA.11938.1.S1_at	236119_s_at	SPRR2G	small proline-rich protein 2G	chr1q21-q22	6706	---	---	---	---	MmugDNA.11938.1.S1_at_SPRR2G
MmugDNA.11939.1.S1_at	1560716_at	---	---	---	---	---	---	---	---	MmugDNA.11939.1.S1_at_---
MmugDNA.11940.1.S1_at	224524_s_at	ASB3	ankyrin repeat and SOCS box-containing 3	chr2p16-p14	51130	ASB3	ankyrin repeat and SOCS box-containing 3	---	716503	MmugDNA.11940.1.S1_at_ASB3
MmugDNA.11941.1.S1_at	240358_at	---	---	---	---	---	---	---	---	MmugDNA.11941.1.S1_at_---
MmugDNA.1194.1.S1_at	232771_at	NRK	Nik related kinase	chrXq22.3	203447	LOC700137	similar to Nik related kinase	---	700137	MmugDNA.1194.1.S1_at_NRK
MmugDNA.11942.1.S1_at	240645_at	---	---	---	---	---	---	---	---	MmugDNA.11942.1.S1_at_---
MmugDNA.11943.1.S1_at	237404_at	---	---	---	---	---	---	---	---	MmugDNA.11943.1.S1_at_---
MmugDNA.11944.1.S1_at	212202_s_at	TMEM87A	transmembrane protein 87A	chr15q15.1	25963	---	---	---	---	MmugDNA.11944.1.S1_at_TMEM87A
MmugDNA.11945.1.S1_at	212204_at	TMEM87A	transmembrane protein 87A	chr15q15.1	25963	---	---	---	---	MmugDNA.11945.1.S1_at_TMEM87A
MmugDNA.11946.1.S1_at	243416_at	---	---	---	---	---	---	---	---	MmugDNA.11946.1.S1_at_---
MmugDNA.11947.1.S1_at	1554245_x_at	ARL17 /// ARL17P1	ADP-ribosylation factor-like 17 /// ADP-ribosylation factor-like 17 pseudogene 1	chr17q21.31 /// chr17q21.32	51326 /// 641522	---	---	---	---	MmugDNA.11947.1.S1_at_ARL17 /// A
MmugDNA.11948.1.S1_at	1558648_at	C15orf57	chromosome 15 open reading frame 57	chr15q15.1	90416	---	---	---	---	MmugDNA.11948.1.S1_at_C15orf57
MmugDNA.11949.1.S1_at	216766_at	---	---	---	---	---	---	---	---	MmugDNA.11949.1.S1_at_---
MmugDNA.11950.1.S1_at	200688_at	SF3B3	splicing factor 3b, subunit 3, 130kDa	chr16q22.1	23450	LOC708939	similar to splicing factor 3b, subunit 3	---	708939	MmugDNA.11950.1.S1_at_SF3B3
MmugDNA.11951.1.S1_at	232566_at	NOL6	nucleolar protein family 6 (RNA-associated)	chr9p13.3	65083	NOL6	nucleolar protein family 6 (RNA-associated)	---	702596	MmugDNA.11951.1.S1_at_NOL6
MmugDNA.1195.1.S1_at	240060_at	---	---	---	---	---	---	---	---	MmugDNA.1195.1.S1_at_---
MmugDNA.11952.1.S1_at	228306_at	CNIH4	cornichon homolog 4 (Drosophila)	chr1q42.11	29097	---	---	---	---	MmugDNA.11952.1.S1_at_CNIH4
MmugDNA.11953.1.S1_at	231873_at	BMPR2	bone morphogenetic protein receptor, type II (serine/threonine kinase)	chr2q33-q34	659	---	---	---	---	MmugDNA.11953.1.S1_at_BMPR2
MmugDNA.11954.1.S1_at	1561026_a_at	---	---	---	---	---	---	---	---	MmugDNA.11954.1.S1_at_---
MmugDNA.11955.1.S1_at	209996_x_at	PCM1	pericentriolar material 1	chr8p22-p21.3	5108	PCM1	pericentriolar material 1	---	703580	MmugDNA.11955.1.S1_at_PCM1
MmugDNA.11956.1.S1_at	1561670_at	---	---	---	---	---	---	---	---	MmugDNA.11956.1.S1_at_---
MmugDNA.11957.1.S1_at	201844_s_at	RYBP	RING1 and YY1 binding protein	chr3p13	23429	LOC694390	similar to RING1 and YY1 binding protein	---	694390	MmugDNA.11957.1.S1_at_RYBP
MmugDNA.11958.1.S1_at	224538_s_at	PARD6G	par-6 partitioning defective 6 homolog gamma (C. elegans)	chr18q23	84552	PARD6G	par-6 partitioning defective 6 homolog gamma (C. elegans)	---	699479	MmugDNA.11958.1.S1_at_PARD6G
MmugDNA.11959.1.S1_at	237078_at	---	---	---	---	---	---	---	---	MmugDNA.11959.1.S1_at_---
MmugDNA.11960.1.S1_at	239504_at	---	---	---	---	---	---	---	---	MmugDNA.11960.1.S1_at_---
MmugDNA.11961.1.S1_at	1566033_at	---	---	---	---	---	---	---	---	MmugDNA.11961.1.S1_at_---
MmugDNA.1196.1.S1_s_at	200095_x_at	RPS10	ribosomal protein S10	chr6p21.31	6204	LOC694687 /// LOC6969	similar to 40S ribosomal protein S10 /// similar to 40S ribosomal protein S10 /// similar to 40S ribosom	---	694687 /// 696921 /// 708	MmugDNA.1196.1.S1_s_at_RPS10
MmugDNA.11962.1.S1_at	225062_at	LOC100132181 /// LOC	hypothetical protein LOC100132181 /// hypothetical gene supported by AL713796	---	100132181 /// 389831	---	---	---	---	MmugDNA.11962.1.S1_at_LOC100132
MmugDNA.11964.1.S1_at	225046_at	LOC100132181	hypothetical protein LOC100132181	---	100132181	---	---	---	---	MmugDNA.11964.1.S1_at_LOC100132
MmugDNA.11965.1.S1_x_at	NA	NA	NA	NA	NA	NA	NA	NA	NA	MmugDNA.11965.1.S1_x_at_NA
MmugDNA.11966.1.S1_at	217983_s_at	RNASET2	ribonuclease T2	chr6q27	8635	---	---	---	---	MmugDNA.11966.1.S1_at_RNASET2
MmugDNA.11967.1.S1_at	1558354_s_at	---	---	---	---	---	---	---	---	MmugDNA.11967.1.S1_at_---
MmugDNA.11968.1.S1_at	242507_at	---	---	---	---	---	---	---	---	MmugDNA.11968.1.S1_at_---
MmugDNA.11969.1.S1_at	204445_s_at	ALOX5	arachidonate 5-lipoxygenase	chr10q11.2	240	ALOX5	arachidonate 5-lipoxygenase	---	707926	MmugDNA.11969.1.S1_at_ALOX5
MmugDNA.11970.1.S1_at	204176_at	KLHL20	kelch-like 20 (Drosophila)	chr1q24.1-q24.3	27252	KLHL20	similar to kelch-like 20	---	708546	MmugDNA.11970.1.S1_at_KLHL20
MmugDNA.11971.1.S1_at	1560870_a_at	---	---	---	---	---	---	---	---	MmugDNA.11971.1.S1_at_---
MmugDNA.1197.1.S1_at	238753_at	FREQ	frequenin homolog (Drosophila)	chr9q34	23413	LOC722507	similar to neuronal calcium sensor-1	---	722507	MmugDNA.1197.1.S1_at_FREQ
MmugDNA.11972.1.S1_at	240140_s_at	LRIG1	leucine-rich repeats and immunoglobulin-like domains 1	chr3p14	26018	LRIG1	leucine-rich repeats and immunoglobulin-like domains 1	---	700033	MmugDNA.11972.1.S1_at_LRIG1
MmugDNA.11973.1.S1_at	237169_at	---	---	---	---	---	---	---	---	MmugDNA.11973.1.S1_at_---
MmugDNA.11974.1.S1_at	229415_at	CYCS	cytochrome c, somatic	chr7p15.2	54205	---	---	---	---	MmugDNA.11974.1.S1_at_CYCS
MmugDNA.11975.1.S1_at	242900_at	ALG10B	asparagine-linked glycosylation 10, alpha-1,2-glucosyltransferase homolog B (yeast)	chr12q12	144245	---	---	---	---	MmugDNA.11975.1.S1_at_ALG10B
MmugDNA.11976.1.S1_s_at	217197_x_at	N4BP2L1	NEDD4 binding protein 2-like 1	chr13q12-q13	90634	LOC707686	Similar to angiogenic factor VG5Q	---	707686	MmugDNA.11976.1.S1_s_at_N4BP2L1
MmugDNA.11977.1.S1_at	214396_s_at	MBD2	methyl-CpG binding domain protein 2	chr18q21	8932	MBD2	methyl-CpG binding domain protein 2	---	693801	MmugDNA.11977.1.S1_at_MBD2
MmugDNA.11978.1.S1_at	207507_s_at	ATP5G3	ATP synthase, H+ transporting, mitochondrial F0 complex, subunit C3 (subunit 9)	chr2q31.1	518	ATP5G3	ATP synthase, H+ transporting, mitochondrial F0 complex, subunit C3 (subunit 9)	---	702893	MmugDNA.11978.1.S1_at_ATP5G3
MmugDNA.11979.1.S1_at	200818_at	ATP5O	ATP synthase, H+ transporting, mitochondrial F1 complex, O subunit	chr21q22.1-q22.2|21q22.11	539	ATP5O	ATP synthase, H+ transporting, mitochondrial F1 complex, O subunit (oligomycin sensitivity conferrin	---	698480	MmugDNA.11979.1.S1_at_ATP5O
MmugDNA.11980.1.S1_at	1559322_at	LOC727916	hypothetical protein LOC727916	chr6q12	727916	---	---	---	---	MmugDNA.11980.1.S1_at_LOC727916
MmugDNA.11981.1.S1_x_at	NA	NA	NA	NA	NA	NA	NA	NA	NA	MmugDNA.11981.1.S1_x_at_NA
MmugDNA.1198.1.S1_at	214434_at	HSPA12A	heat shock 70kDa protein 12A	chr10q26.12	259217	HSPA12A	heat shock 70kDa protein 12A	---	707202	MmugDNA.1198.1.S1_at_HSPA12A
MmugDNA.11982.1.S1_at	237814_at	---	---	---	---	---	---	---	---	MmugDNA.11982.1.S1_at_---
MmugDNA.11983.1.S1_s_at	201971_s_at	ATP6V1A	ATPase, H+ transporting, lysosomal 70kDa, V1 subunit A	chr3q13.2-q13.31	523	LOC696878	similar to ATPase, H+ transporting, lysosomal 70kD, V1 subunit A, isoform 1	---	696878	MmugDNA.11983.1.S1_s_at_ATP6V1A
MmugDNA.11984.1.S1_at	230939_at	LOC100131813	similar to hCG2041218	chr8q22.3	100131813	---	---	---	---	MmugDNA.11984.1.S1_at_LOC100131
MmugDNA.11985.1.S1_at	225621_at	ALG2	asparagine-linked glycosylation 2, alpha-1,3-mannosyltransferase homolog (S. cerevisiae)	chr9q22.33	85365	LOC714850	similar to asparagine-linked glycosylation 2	---	714850	MmugDNA.11985.1.S1_at_ALG2
MmugDNA.11986.1.S1_at	1558552_s_at	C3orf55	chromosome 3 open reading frame 55	chr3q25.32	152078	---	---	---	---	MmugDNA.11986.1.S1_at_C3orf55
MmugDNA.11987.1.S1_at	228329_at	DAB1	disabled homolog 1 (Drosophila)	chr1p32-p31	1600	LOC716200	similar to Disabled homolog 1	---	716200	MmugDNA.11987.1.S1_at_DAB1
MmugDNA.11988.1.S1_at	1570085_at	TTLL1	tubulin tyrosine ligase-like family, member 1	chr22q13.1	25809	---	---	---	---	MmugDNA.11988.1.S1_at_TTLL1
MmugDNA.11989.1.S1_at	230349_at	XKRX	XK, Kell blood group complex subunit-related, X-linked	chrXq22.1	402415	XKRX	XK, Kell blood group complex subunit-related, X-linked	---	701721	MmugDNA.11989.1.S1_at_XKRX
MmugDNA.11990.1.S1_at	222772_at	MYEF2	myelin expression factor 2	chr15q21.1	50804	LOC716153	similar to myelin gene expression factor 2	---	716153	MmugDNA.11990.1.S1_at_MYEF2
MmugDNA.11991.1.S1_at	230643_at	WNT9A	wingless-type MMTV integration site family, member 9A	chr1q42	7483	---	---	---	---	MmugDNA.11991.1.S1_at_WNT9A
MmugDNA.1199.1.S1_at	242167_at	---	---	---	---	---	---	---	---	MmugDNA.1199.1.S1_at_---
MmugDNA.11992.1.S1_at	212432_at	GRPEL1	GrpE-like 1, mitochondrial (E. coli)	chr4p16	80273	---	---	---	---	MmugDNA.11992.1.S1_at_GRPEL1
MmugDNA.11993.1.S1_at	212434_at	GRPEL1	GrpE-like 1, mitochondrial (E. coli)	chr4p16	80273	---	---	---	---	MmugDNA.11993.1.S1_at_GRPEL1
MmugDNA.11994.1.S1_at	1557765_at	LOC643401	hypothetical protein LOC643401	chr5p14.1	643401	---	---	---	---	MmugDNA.11994.1.S1_at_LOC643401
MmugDNA.11995.1.S1_at	1561229_at	---	---	---	---	---	---	---	---	MmugDNA.11995.1.S1_at_---
MmugDNA.11996.1.S1_at	NA	NA	NA	NA	NA	NA	NA	NA	NA	MmugDNA.11996.1.S1_at_NA
MmugDNA.11996.1.S1_s_at	212466_at	SPRED2	sprouty-related, EVH1 domain containing 2	chr2p14	200734	SPRED2	sprouty-related, EVH1 domain containing 2	---	697196	MmugDNA.11996.1.S1_s_at_SPRED2
MmugDNA.11997.1.S1_at	222835_at	THSD4	thrombospondin, type I, domain containing 4	chr15q23	79875	LOC700919	similar to thrombospondin repeat protein 1	---	700919	MmugDNA.11997.1.S1_at_THSD4
MmugDNA.11998.1.S1_at	212458_at	SPRED2	sprouty-related, EVH1 domain containing 2	chr2p14	200734	SPRED2	sprouty-related, EVH1 domain containing 2	---	697196	MmugDNA.11998.1.S1_at_SPRED2
MmugDNA.11999.1.S1_at	239420_at	---	---	---	---	---	---	---	---	MmugDNA.11999.1.S1_at_---
MmugDNA.1.1.S1_at	240236_at	STXBP5L	syntaxin binding protein 5-like	chr3q13.33	9515	---	---	---	---	MmugDNA.1.1.S1_at_STXBP5L
MmugDNA.12000.1.S1_at	1561919_at	---	---	---	---	---	---	---	---	MmugDNA.12000.1.S1_at_---
MmugDNA.12001.1.S1_at	242075_at	---	---	---	---	---	---	---	---	MmugDNA.12001.1.S1_at_---
MmugDNA.1200.1.S1_at	1563121_at	---	---	---	---	---	---	---	---	MmugDNA.1200.1.S1_at_---
MmugDNA.12002.1.S1_at	202874_s_at	ATP6V1C1	ATPase, H+ transporting, lysosomal 42kDa, V1 subunit C1	chr8q22.3	528	LOC698077	Hypothetical protein LOC698077	---	698077	MmugDNA.12002.1.S1_at_ATP6V1C1
MmugDNA.12003.1.S1_s_at	NA	NA	NA	NA	NA	NA	NA	NA	NA	MmugDNA.12003.1.S1_s_at_NA
MmugDNA.12004.1.S1_at	1561256_at	---	---	---	---	---	---	---	---	MmugDNA.12004.1.S1_at_---
MmugDNA.12005.1.S1_at	1562771_at	---	---	---	---	---	---	---	---	MmugDNA.12005.1.S1_at_---
MmugDNA.12006.1.S1_at	1565866_a_at	---	---	---	---	---	---	---	---	MmugDNA.12006.1.S1_at_---
MmugDNA.12007.1.S1_at	226308_at	HAUS8	HAUS augmin-like complex, subunit 8	chr19p13.11	93323	LOC718874	similar to sarcoma antigen NY-SAR-48 isoform a	---	718874	MmugDNA.12007.1.S1_at_HAUS8
MmugDNA.12008.1.S1_at	236767_at	IQCF2	IQ motif containing F2	chr3p21.1	389123	IQCF5	similar to IQ motif containing F4	---	699538	MmugDNA.12008.1.S1_at_IQCF2
MmugDNA.12009.1.S1_at	244802_at	GLUD1	glutamate dehydrogenase 1	chr10q23.3	2746	---	---	---	---	MmugDNA.12009.1.S1_at_GLUD1
MmugDNA.12010.1.S1_at	231437_at	SLC35D2	solute carrier family 35, member D2	chr9q22.32	11046	---	---	---	---	MmugDNA.12010.1.S1_at_SLC35D2
MmugDNA.1201.1.S1_at	228403_at	ENHO	energy homeostasis associated	chr9p13.3	375704	LOC700537	hypothetical protein LOC700537	---	700537	MmugDNA.1201.1.S1_at_ENHO
MmugDNA.12012.1.S1_at	1559350_at	---	---	---	---	---	---	---	---	MmugDNA.12012.1.S1_at_---
MmugDNA.12013.1.S1_at	232155_at	KIAA1618	KIAA1618	chr17q25.3	57714	---	---	---	---	MmugDNA.12013.1.S1_at_KIAA1618
MmugDNA.12014.1.S1_at	216420_at	TARDBPL	TAR DNA binding protein-like	chr20p12.3	643503	---	---	---	---	MmugDNA.12014.1.S1_at_TARDBPL
MmugDNA.12015.1.S1_at	235345_at	RUFY2	RUN and FYVE domain containing 2	chr10q21.3	55680	LOC694919	similar to RUN and FYVE domain-containing 2	---	694919	MmugDNA.12015.1.S1_at_RUFY2
MmugDNA.12016.1.S1_at	205198_s_at	ATP7A	ATPase, Cu++ transporting, alpha polypeptide	chrXq13.2-q13.3	538	---	---	---	---	MmugDNA.12016.1.S1_at_ATP7A
MmugDNA.12017.1.S1_at	232968_at	FANK1	fibronectin type III and ankyrin repeat domains 1	chr10q26.2	92565	FANK1	fibronectin type III and ankyrin repeat domains 1	---	696281	MmugDNA.12017.1.S1_at_FANK1
MmugDNA.12018.1.S1_at	232666_at	OAS3	2'-5'-oligoadenylate synthetase 3, 100kDa	chr12q24.2	4940	LOC712342	similar to 2-5oligoadenylate synthetase 3	---	712342	MmugDNA.12018.1.S1_at_OAS3
MmugDNA.12019.1.S1_at	205052_at	AUH	AU RNA binding protein/enoyl-Coenzyme A hydratase	chr9q22.31	549	AUH	AU RNA binding protein/enoyl-Coenzyme A hydratase	---	704542	MmugDNA.12019.1.S1_at_AUH
MmugDNA.120.1.S1_at	213144_at	GOSR2	golgi SNAP receptor complex member 2	chr17q21	9570	LOC716841	similar to golgi SNAP receptor complex member 2 isoform A	---	716841	MmugDNA.120.1.S1_at_GOSR2
MmugDNA.12020.1.S1_at	1564053_a_at	YTHDF3	YTH domain family, member 3	chr8q12.3	253943	LOC708171	similar to YTH domain family, member 3	---	708171	MmugDNA.12020.1.S1_at_YTHDF3
MmugDNA.12021.1.S1_s_at	205989_s_at	MOG	myelin oligodendrocyte glycoprotein	chr6p22.1	4340	---	---	---	---	MmugDNA.12021.1.S1_s_at_MOG
MmugDNA.1202.1.S1_at	1556362_at	---	---	---	---	CRKL	V-crk sarcoma virus CT10 oncogene homolog (avian)-like	---	696584	MmugDNA.1202.1.S1_at_---
MmugDNA.12022.1.S1_at	232202_at	---	---	---	---	---	---	---	---	MmugDNA.12022.1.S1_at_---
MmugDNA.12023.1.S1_at	NA	NA	NA	NA	NA	NA	NA	NA	NA	MmugDNA.12023.1.S1_at_NA
MmugDNA.12023.1.S1_s_at	1555407_s_at	FGD3	FYVE, RhoGEF and PH domain containing 3	---	89846	FGD3	FYVE, RhoGEF and PH domain containing 3	---	706720	MmugDNA.12023.1.S1_s_at_FGD3
MmugDNA.12024.1.S1_s_at	234427_at	TRAJ17	T cell receptor alpha joining 17	chr14q11	28738	LOC710093	similar to T-cell receptor alpha chain V region CTL-L17 precursor	---	710093	MmugDNA.12024.1.S1_s_at_TRAJ17
MmugDNA.12025.1.S1_at	212731_at	ANKRD46	ankyrin repeat domain 46	chr8q22.3	157567	ANKRD46	ankyrin repeat domain 46	---	705491	MmugDNA.12025.1.S1_at_ANKRD46
MmugDNA.12026.1.S1_at	1554039_at	C14orf159	chromosome 14 open reading frame 159	chr14q32.12	80017	C7H14orf159	chromosome 14 open reading frame 159 ortholog	---	700168	MmugDNA.12026.1.S1_at_C14orf159
MmugDNA.12027.1.S1_at	232595_at	---	---	---	---	---	---	---	---	MmugDNA.12027.1.S1_at_---

MmugDNA.12028.1.S1_at	235483_at	---	---	---	---		LOC698953	similar to syntaxin 3A	---		698953	MmugDNA.12028.1.S1_at_---
MmugDNA.12029.1.S1_at	213575_at	TRA2A	transformer 2 alpha homolog (Drosophila)	chr7p15.3		29896	EXOC4	Exocyst complex component 4	---		712458	MmugDNA.12029.1.S1_at_TRA2A
MmugDNA.12030.1.S1_at	227939_s_at	TRA2A	Transformer 2 alpha homolog (Drosophila)	chr7p15.3		29896	EXOC4	Exocyst complex component 4	---		712458	MmugDNA.12030.1.S1_at_TRA2A
MmugDNA.12031.1.S1_at	236022_at	MYO19	myosin XIX	chr17q12		80179	---	---	---	---		MmugDNA.12031.1.S1_at_MYO19
MmugDNA.1203.1.S1_s_at	233152_x_at	---	---	---	---		---	---	---	---		MmugDNA.1203.1.S1_s_at_---
MmugDNA.12033.1.S1_at	228039_at	DDX46	DEAD (Asp-Glu-Ala-Asp) box polypeptide 46	chr5q31.1		9879	LOC711026	similar to DEAD (Asp-Glu-Ala-Asp) box polypeptide 46	---		711026	MmugDNA.12033.1.S1_at_DDX46
MmugDNA.12034.1.S1_at	1564051_at	---	---	---	---		---	---	---	---		MmugDNA.12034.1.S1_at_---
MmugDNA.12035.1.S1_at	232994_s_at	RGNEF	Rho-guanine nucleotide exchange factor	chr5q13.2		64283	---	---	---	---		MmugDNA.12035.1.S1_at_RGNEF
MmugDNA.12036.1.S1_at	1569830_at	PTPRC	protein tyrosine phosphatase, receptor type, C	chr1q31-q32		5788	---	---	---	---		MmugDNA.12036.1.S1_at_PTPRC
MmugDNA.12037.1.S1_at	238779_at	DCP2	DCP2 decapping enzyme homolog (S. cerevisiae)	chr5q22.2		167227	DCP2	DCP2 decapping enzyme homolog (S. cerevisiae)	---		694225	MmugDNA.12037.1.S1_at_DCP2
MmugDNA.12038.1.S1_at	1570210_x_at	SAPS2	SAPS domain family, member 2	chr22q13.33		9701	---	---	---	---		MmugDNA.12038.1.S1_at_SAPS2
MmugDNA.12038.1.S1_x_at	NA	NA	NA	NA	NA		NA	NA	NA	NA		MmugDNA.12038.1.S1_x_at_NA
MmugDNA.12039.1.S1_at	1554460_at	ST8SIA4	ST8 alpha-N-acetyl-neuraminide alpha-2,8-sialyltransferase 4	chr5q21		7903	---	---	---	---		MmugDNA.12039.1.S1_at_ST8SIA4
MmugDNA.12040.1.S1_at	1554908_at	HYDIN	hydrocephalus inducing homolog (mouse)	chr16q22.1-q22.3		54768	---	---	---	---		MmugDNA.12040.1.S1_at_HYDIN
MmugDNA.12041.1.S1_at	1559096_x_at	FBXO9	F-box protein 9	chr6p12.3-p11.2		26268	LOC702354	Similar to solute carrier family 38, member 4	---		702354	MmugDNA.12041.1.S1_at_FBXO9
MmugDNA.1204.1.S1_at	240145_at	---	---	---	---		---	---	---	---		MmugDNA.1204.1.S1_at_---
MmugDNA.12042.1.S1_at	1566222_at	---	---	---	---		---	---	---	---		MmugDNA.12042.1.S1_at_---
MmugDNA.12043.1.S1_at	235643_at	SAMD9L	sterile alpha motif domain containing 9-like	chr7q21.2-q21.3		219285	LOC701572	similar to sterile alpha motif domain containing 9-like	---		701572	MmugDNA.12043.1.S1_at_SAMD9L
MmugDNA.12044.1.S1_at	236137_at	---	---	---	---		---	---	---	---		MmugDNA.12044.1.S1_at_---
MmugDNA.12045.1.S1_at	234993_at	ABHD13	abhydrolase domain containing 13	chr13q33.3		84945	PDE6A	Phosphodiesterase 6A, cGMP-specific, rod, alpha	---		677688	MmugDNA.12045.1.S1_at_ABHD13
MmugDNA.12046.1.S1_at	234684_s_at	KRTAP4-4	keratin associated protein 4-4	chr17q12-q21		84616	LOC704430	similar to keratin associated protein 4-12	---		704430	MmugDNA.12046.1.S1_at_KRTAP4-4
MmugDNA.12047.1.S1_at	212843_at	NCAM1	neural cell adhesion molecule 1	chr11q23.1		4684	NCAM1	neural cell adhesion molecule 1	---		693789	MmugDNA.12047.1.S1_at_NCAM1
MmugDNA.12048.1.S1_at	1568648_a_at	---	---	---	---		---	---	---	---		MmugDNA.12048.1.S1_at_---
MmugDNA.12049.1.S1_at	1554828_at	PDGFRA	platelet-derived growth factor receptor, alpha polypeptide	chr4q11-q13		5156	IPO13	Importin 13	---		702007	MmugDNA.12049.1.S1_at_PDGFRA
MmugDNA.12050.1.S1_at	226276_at	TMEM167A	transmembrane protein 167A	chr5q14.2		153339	LOC712218	similar to CG14199-PA	---		712218	MmugDNA.12050.1.S1_at_TMEM167A
MmugDNA.12051.1.S1_at	215962_at	---	---	---	---		LOC709592	hypothetical protein LOC709592	---		709592	MmugDNA.12051.1.S1_at_---
MmugDNA.1205.1.S1_at	221919_at	HNRNPA1 /// LOC7288	heterogeneous nuclear ribonucleoprotein A1 /// hypothetical LOC728844	chr12q13.1 /// chr12q13.13	3178 /// 728844		---	---	---	---		MmugDNA.1205.1.S1_at_HNRNPA1 ///
MmugDNA.12052.1.S1_at	209231_s_at	DCTN5	dynactin 5 (p25)	chr16p12.1		84516	DCTN5	dynactin 5 (p25)	---		698277	MmugDNA.12052.1.S1_at_DCTN5
MmugDNA.12053.1.S1_at	233786_at	---	---	---	---		---	---	---	---		MmugDNA.12053.1.S1_at_---
MmugDNA.12054.1.S1_at	233767_at	HHLA1	HERV-H LTR-associating 1	chr8q24		10086	---	---	---	---		MmugDNA.12054.1.S1_at_HHLA1
MmugDNA.12055.1.S1_at	238836_at	---	---	---	---		TFAM	transcription factor A, mitochondrial	---		701368	MmugDNA.12055.1.S1_at_---
MmugDNA.12056.1.S1_at	233195_at	DNAI1	dynein, axonemal, intermediate chain 1	chr9p21-p13		27019	---	---	---	---		MmugDNA.12056.1.S1_at_DNAI1
MmugDNA.12057.1.S1_at	240298_at	LOC147791	hypothetical protein LOC147791	chr19p13.3		147791	LOC708157	hypothetical protein LOC708157	---		708157	MmugDNA.12057.1.S1_at_LOC147791
MmugDNA.12058.1.S1_at	241650_x_at	HMCN2	hemicentin 2	chr9q34.11		256158	---	---	---	---		MmugDNA.12058.1.S1_at_HMCN2
MmugDNA.12059.1.S1_at	1559932_at	---	---	---	---		---	---	---	---		MmugDNA.12059.1.S1_at_---
MmugDNA.12060.1.S1_at	218801_at	UGCGL2	UDP-glucose ceramide glucosyltransferase-like 2	chr13q32.1		55757	LOC697784	similar to UDP-glucose:glycoprotein glucosyltransferase 2	---		697784	MmugDNA.12060.1.S1_at_UGCGL2
MmugDNA.12061.1.S1_at	241925_x_at	---	---	---	---		---	---	---	---		MmugDNA.12061.1.S1_at_---
MmugDNA.1206.1.S1_at	227592_at	ALDH16A1	aldehyde dehydrogenase 16 family, member A1	chr19q13.33		126133	---	---	---	---		MmugDNA.1206.1.S1_at_ALDH16A1
MmugDNA.12062.1.S1_at	1553635_s_at	TCTEX1D1	Tctex1 domain containing 1	chr1p31.3		200132	TCTEX1D1	Tctex1 domain containing 1	---		699673	MmugDNA.12062.1.S1_at_TCTEX1D1
MmugDNA.12063.1.S1_at	216556_x_at	---	---	---	---		LOC697073 /// LOC7093	similar to SMT3 suppressor of mif two 3 homolog 1 /// similar to SMT3 suppressor of mif two 3 homol	---	697073 /// 709360		MmugDNA.12063.1.S1_at_---
MmugDNA.12064.1.S1_at	NA	NA	NA	NA	NA		NA	NA	NA	NA		MmugDNA.12064.1.S1_at_NA
MmugDNA.12064.1.S1_s_at	207530_s_at	CDKN2B	cyclin-dependent kinase inhibitor 2B (p15, inhibits CDK4)	chr9p21		1030	TPT1	Similar to Translationally-controlled tumor protein (TCTP) (p23) (Histamine-releasing factor) (HRF) (F	---		711851	MmugDNA.12064.1.S1_s_at_CDKN2B
MmugDNA.12065.1.S1_at	209596_at	MXRA5	matrix-remodelling associated 5	chrXp22.33		25878	MXRA5	matrix-remodelling associated 5	---		706214	MmugDNA.12065.1.S1_at_MXRA5
MmugDNA.12066.1.S1_at	216012_at	---	---	---	---		---	---	---	---		MmugDNA.12066.1.S1_at_---
MmugDNA.12067.1.S1_at	209968_s_at	NCAM1	neural cell adhesion molecule 1	chr11q23.1		4684	NCAM1	neural cell adhesion molecule 1	---		693789	MmugDNA.12067.1.S1_at_NCAM1
MmugDNA.12068.1.S1_at	1559282_at	---	---	---	---		---	---	---	---		MmugDNA.12068.1.S1_at_---
MmugDNA.12069.1.S1_at	227822_at	ZNF605	zinc finger protein 605	chr12q24.33		90462	LOC694553	similar to zinc finger protein 605	---		694553	MmugDNA.12069.1.S1_at_ZNF605
MmugDNA.12070.1.S1_at	206913_at	BAAT	bile acid Coenzyme A: amino acid N-acyltransferase (glycine N-choloyltransferase)	chr9q22.3		570	LOC713983	similar to bile acid Coenzyme A: amino acid N-acyltransferase	---		713983	MmugDNA.12070.1.S1_at_BAAT
MmugDNA.12071.1.S1_at	204194_at	BACH1	BTB and CNC homology 1, basic leucine zipper transcription factor 1	chr21q22.11		571	BACH1	BTB and CNC homology 1, basic leucine zipper transcription factor 1	---		705817	MmugDNA.12071.1.S1_at_BACH1
MmugDNA.1207.1.S1_at	243246_at	NAT12	N-acetyltransferase 12 (GCN5-related, putative)	chr14q23.1		122830	---	---	---	---		MmugDNA.1207.1.S1_at_NAT12
MmugDNA.12072.1.S1_at	238767_at	---	---	---	---		---	---	---	---		MmugDNA.12072.1.S1_at_---
MmugDNA.12073.1.S1_at	206083_at	BAI1	brain-specific angiogenesis inhibitor 1	chr8q24		575	BAI1	brain-specific angiogenesis inhibitor 1	---		694367	MmugDNA.12073.1.S1_at_BAI1
MmugDNA.12074.1.S1_at	224231_at	PRO0471	hypothetical LOC28994	chr3p13-q26.1		28994	---	---	---	---		MmugDNA.12074.1.S1_at_PRO0471
MmugDNA.12075.1.S1_at	222931_s_at	THNSL1	threonine synthase-like 1 (S. cerevisiae)	chr10p12.1		79896	LOC696314	Similar to tumor necrosis factor, alpha-induced protein 2	---		696314	MmugDNA.12075.1.S1_at_THNSL1
MmugDNA.12076.1.S1_at	1562346_at	---	---	---	---		---	---	---	---		MmugDNA.12076.1.S1_at_---
MmugDNA.12077.1.S1_at	NA	NA	NA	NA	NA		NA	NA	NA	NA		MmugDNA.12077.1.S1_at_NA
MmugDNA.12077.1.S1_s_at	238725_at	IRF1	interferon regulatory factor 1	chr5q31.1		3659	---	---	---	---		MmugDNA.12077.1.S1_s_at_IRF1
MmugDNA.12078.1.S1_at	224016_at	HIPK2	homeodomain interacting protein kinase 2	chr7q32-q34		28996	---	---	---	---		MmugDNA.12078.1.S1_at_HIPK2
MmugDNA.12079.1.S1_at	1556075_at	---	---	---	---		ZNF133	zinc finger protein 133	---		700023	MmugDNA.12079.1.S1_at_---
MmugDNA.12080.1.S1_at	1555347_at	PDXDC1	pyridoxal-dependent decarboxylase domain containing 1	chr16p13.11		23042	---	---	---	---		MmugDNA.12080.1.S1_at_PDXDC1
MmugDNA.12081.1.S1_at	235681_at	---	---	---	---		---	---	---	---		MmugDNA.12081.1.S1_at_---
MmugDNA.1208.1.S1_at	221235_s_at	LOC644617	hypothetical LOC644617	chr2q12.1		644617	LOC713102	similar to TGF beta receptor associated protein -1	---		713102	MmugDNA.1208.1.S1_at_LOC644617
MmugDNA.12082.1.S1_at	214221_at	ALMS1	Alstrom syndrome 1	chr2p13		7840	ALMS1	Alstrom syndrome 1	---		706612	MmugDNA.12082.1.S1_at_ALMS1
MmugDNA.12083.1.S1_at	240995_at	---	---	---	---		---	---	---	---		MmugDNA.12083.1.S1_at_---
MmugDNA.12084.1.S1_at	238568_s_at	---	---	---	---		---	---	---	---		MmugDNA.12084.1.S1_at_---
MmugDNA.12085.1.S1_at	215355_at	POU2F3	POU class 2 homeobox 3	chr11q23.3		25833	---	---	---	---		MmugDNA.12085.1.S1_at_POU2F3
MmugDNA.12086.1.S1_at	237958_at	---	---	---	---		---	---	---	---		MmugDNA.12086.1.S1_at_---
MmugDNA.12087.1.S1_at	228014_at	PTRH1	peptidyl-tRNA hydrolase 1 homolog (S. cerevisiae)	chr9q34.11		138428	LOC700335	hypothetical protein LOC700335	---		700335	MmugDNA.12087.1.S1_at_PTRH1
MmugDNA.12088.1.S1_at	238720_at	OMG	Oligodendrocyte myelin glycoprotein	chr17q11.2		4974	---	---	---	---		MmugDNA.12088.1.S1_at_OMG
MmugDNA.12089.1.S1_at	1554015_a_at	CHD2	chromodomain helicase DNA binding protein 2	chr15q26		1106	CHD2	chromodomain helicase DNA binding protein 2	---		706310	MmugDNA.12089.1.S1_at_CHD2
MmugDNA.12090.1.S1_at	236917_at	LRRC34	leucine rich repeat containing 34	chr3q26.2		151827	LRRC34	leucine rich repeat containing 34	---		697963	MmugDNA.12090.1.S1_at_LRRC34
MmugDNA.12091.1.S1_at	203728_at	BAK1	BCL2-antagonist/killer 1	chr6p21.3		578	BAK1	BCL2-antagonist/killer 1	---		706534	MmugDNA.12091.1.S1_at_BAK1
MmugDNA.1209.1.S1_at	242238_at	---	---	---	---		---	---	---	---		MmugDNA.1209.1.S1_at_---
MmugDNA.12092.1.S1_at	238436_s_at	ZNF805	zinc finger protein 805	chr19q13.43		390980	---	---	---	---		MmugDNA.12092.1.S1_at_ZNF805
MmugDNA.12093.1.S1_s_at	1555639_a_at	RBM14	RNA binding motif protein 14	chr11q13.1		10432	RBM4	RNA binding motif protein 4	---		713490	MmugDNA.12093.1.S1_s_at_RBM14
MmugDNA.12094.1.S1_at	205363_at	BBOX1	butyrobetaine (gamma), 2-oxoglutarate dioxygenase (gamma-butyrobetaine hydroxylase) 1	chr11p14.2		8424	BBOX1	butyrobetaine (gamma), 2-oxoglutarate dioxygenase (gamma-butyrobetaine hydroxylase) 1	---		699465	MmugDNA.12094.1.S1_at_BBOX1
MmugDNA.12095.1.S1_at	1553521_at	DEFB104A /// DEFB104	defensin, beta 104A /// defensin, beta 104B	chr8p23.1	140596 /// 503618		---	---	---	---		MmugDNA.12095.1.S1_at_DEFB104A /
MmugDNA.12096.1.S1_at	241910_x_at	ARL17P1	ADP-ribosylation factor-like 17 pseudogene 1	chr17q21.32		51326	---	---	---	---		MmugDNA.12096.1.S1_at_ARL17P1
MmugDNA.12097.1.S1_at	233763_at	LYRM2	LYR motif containing 2	chr6q14.2-q16.1		57226	---	---	---	---		MmugDNA.12097.1.S1_at_LYRM2
MmugDNA.12098.1.S1_at	237413_at	---	---	---	---		MAPK10	mitogen-activated protein kinase 10	---		699736	MmugDNA.12098.1.S1_at_---
MmugDNA.12099.1.S1_at	228834_at	TOB1	transducer of ERBB2, 1	chr17q21		10140	---	---	---	---		MmugDNA.12099.1.S1_at_TOB1
MmugDNA.12100.1.S1_at	244299_at	---	---	---	---		---	---	---	---		MmugDNA.12100.1.S1_at_---
MmugDNA.12101.1.S1_at	236991_at	---	---	---	---		---	---	---	---		MmugDNA.12101.1.S1_at_---
MmugDNA.1210.1.S1_at	1561507_at	FFAR1	Free fatty acid receptor 1	chr19q13.1		2864	FFAR1	free fatty acid receptor 1	---		708620	MmugDNA.1210.1.S1_at_FFAR1
MmugDNA.12102.1.S1_at	238064_at	---	---	---	---		---	---	---	---		MmugDNA.12102.1.S1_at_---
MmugDNA.12103.1.S1_at	NA	NA	NA	NA	NA		NA	NA	NA	NA		MmugDNA.12103.1.S1_at_NA
MmugDNA.12104.1.S1_at	222616_s_at	USP16	ubiquitin specific peptidase 16	chr21q22.11		10600	USP16	ubiquitin specific peptidase 16	---		706403	MmugDNA.12104.1.S1_at_USP16
MmugDNA.12104.1.S1_s_at	NA	NA	NA	NA	NA		NA	NA	NA	NA		MmugDNA.12104.1.S1_s_at_NA
MmugDNA.12105.1.S1_at	228107_at	LOC100127983	hypothetical protein LOC100127983	chr8q21.3		100127983	LOC696190	hypothetical protein LOC696190	---		696190	MmugDNA.12105.1.S1_at_LOC100127
MmugDNA.12106.1.S1_at	203994_s_at	C21orf2	chromosome 21 open reading frame 2	chr21q22.3		755	LOC722168	similar to Protein C21orf2 (C21orf-HUMF09G8.5) (YF5/A2)	---		722168	MmugDNA.12106.1.S1_at_C21orf2
MmugDNA.12107.1.S1_at	1563064_at	---	---	---	---		---	---	---	---		MmugDNA.12107.1.S1_at_---
MmugDNA.12108.1.S1_at	242254_at	---	---	---	---		---	---	---	---		MmugDNA.12108.1.S1_at_---
MmugDNA.12109.1.S1_at	204032_at	BCAR3	breast cancer anti-estrogen resistance 3	chr1p22.1		8412	LOC713636	similar to breast cancer antiestrogen resistance 3	---		713636	MmugDNA.12109.1.S1_at_BCAR3
MmugDNA.12110.1.S1_at	204378_at	BCAS1	breast carcinoma amplified sequence 1	chr20q13.2		8537	BCAS1	breast carcinoma amplified sequence 1	---		699063	MmugDNA.12110.1.S1_at_BCAS1
MmugDNA.12111.1.S1_at	1569029_at	MAP1D	methionine aminopeptidase 1D	chr2q31.1		254042	LOC695392	similar to methionine aminopeptidase 1D	---		695392	MmugDNA.12111.1.S1_at_MAP1D
MmugDNA.1211.1.S1_s_at	NA	NA	NA	NA	NA		NA	NA	NA	NA		MmugDNA.1211.1.S1_s_at_NA
MmugDNA.1211.1.S1_x_at	226006_at	LOC100131801	similar to hCG2036585	chr19p13.2		100131801	STXBP3	Syntaxin binding protein 3	---		699144	MmugDNA.1211.1.S1_x_at_LOC10013
MmugDNA.12112.1.S1_at	228995_at	CAMSAP1	calmodulin regulated spectrin-associated protein 1	chr9q34.3		157922	LOC721823	similar to calmodulin regulated spectrin-associated protein 1	---		721823	MmugDNA.12112.1.S1_at_CAMSAP1
MmugDNA.12113.1.S1_s_at	212125_at	RANGAP1	Ran GTPase activating protein 1	chr22q13		5905	RANGAP1	Ran GTPase activating protein 1	---		710698	MmugDNA.12113.1.S1_s_at_RANGAP1
MmugDNA.12114.1.S1_at	212127_at	RANGAP1	Ran GTPase activating protein 1	chr22q13		5905	---	---	---	---		MmugDNA.12114.1.S1_at_RANGAP1
MmugDNA.12115.1.S1_at	1562288_at	---	---	---	---		---	---	---	---		MmugDNA.12115.1.S1_at_---
MmugDNA.12116.1.S1_at	1558867_at	DSE	Dermatan sulfate epimerase	chr6q22		29940	---	---	---	---		MmugDNA.12116.1.S1_at_DSE
MmugDNA.12117.1.S1_at	1562966_at	KIAA1217	KIAA1217	chr10p12.2-p12.1		56243	LOC705851	similar to p130Cas-associated protein (p140Cap) (SNAP-25-interacting protein) (SNIP)	---		705851	MmugDNA.12117.1.S1_at_KIAA1217
MmugDNA.12118.1.S1_at	227123_at	RAB3B	RAB3B, member RAS oncogene family	chr1p32-p31		5865	---	---	---	---		MmugDNA.12118.1.S1_at_RAB3B
MmugDNA.12119.1.S1_s_at	233864_s_at	VPS35	vacuolar protein sorting 35 homolog (S. cerevisiae)	chr16q12		55737	LOC698981	Hypothetical protein LOC698981	---		698981	MmugDNA.12119.1.S1_s_at_VPS35
MmugDNA.121.1.S1_at	NA	NA	NA	NA	NA		NA	NA	NA	NA		MmugDNA.121.1.S1_at_NA
MmugDNA.121.1.S1_s_at	NA	NA	NA	NA	NA		NA	NA	NA	NA		MmugDNA.121.1.S1_s_at_NA
MmugDNA.12120.1.S1_at	1561966_at	---	---	---	---		---	---	---	---		MmugDNA.12120.1.S1_at_---

MmugDNA.12121.1.S1_at	1565701_at	---	---	---	---	---	---	---	---	MmugDNA.12121.1.S1_at_---
MmugDNA.1212.1.S1_at	234480_at	---	---	---	---	---	---	---	---	MmugDNA.1212.1.S1_at_---
MmugDNA.12122.1.S1_s_at	218303_x_at	KRCC1	lysine-rich coiled-coil 1	chr2p11.2	51315	---	---	---	---	MmugDNA.12122.1.S1_s_at_KRCC1
MmugDNA.12123.1.S1_at	226981_at	MLL	Myeloid/lymphoid or mixed-lineage leukemia (trithorax homolog, Drosophila)	chr11q23	4297	MLL	myeloid/lymphoid or mixed-lineage leukemia (trithorax homolog, Drosophila)	---	705511	MmugDNA.12123.1.S1_at_MLL
MmugDNA.12125.1.S1_at	243109_at	MCTP2	multiple C2 domains, transmembrane 2	chr15q26.2	55784	---	---	---	---	MmugDNA.12125.1.S1_at_MCTP2
MmugDNA.12126.1.S1_at	232246_at	LOC284900	hypothetical LOC284900	chr22q12.1	284900	---	---	---	---	MmugDNA.12126.1.S1_at_LOC284900
MmugDNA.12127.1.S1_at	238919_at	---	---	---	---	---	---	---	---	MmugDNA.12127.1.S1_at_---
MmugDNA.12128.1.S1_at	225277_at	SLC39A13	solute carrier family 39 (zinc transporter), member 13	chr11p11.2	91252	LOC713420	similar to solute carrier family 39 (zinc transporter), member 13	---	713420	MmugDNA.12128.1.S1_at_SLC39A13
MmugDNA.12129.1.S1_at	211021_s_at	RGS14	regulator of G-protein signaling 14	chr5q35.3	10636	RGS14	regulator of G-protein signaling 14	---	699625	MmugDNA.12129.1.S1_at_RGS14
MmugDNA.12130.1.S1_at	234386_s_at	C15orf49	chromosome 15 open reading frame 49	chr15q11.2	63969	---	---	---	---	MmugDNA.12130.1.S1_at_C15orf49
MmugDNA.12131.1.S1_at	237754_at	---	---	---	---	---	---	---	---	MmugDNA.12131.1.S1_at_---
MmugDNA.1213.1.S1_at	240788_at	---	---	---	---	---	---	---	---	MmugDNA.1213.1.S1_at_---
MmugDNA.12132.1.S1_at	229690_at	FAM109A	family with sequence similarity 109, member A	chr12q24.12	144717	---	---	---	---	MmugDNA.12132.1.S1_at_FAM109A
MmugDNA.12133.1.S1_at	206665_s_at	BCL2L1	BCL2-like 1	chr20q11.21	598	LOC713035	similar to BCL2-like 1 isoform 1	---	713035	MmugDNA.12133.1.S1_at_BCL2L1
MmugDNA.12133.1.S1_s_at	NA	NA	NA	NA	NA	NA	NA	NA	NA	MmugDNA.12133.1.S1_s_at_NA
MmugDNA.12134.1.S1_at	203140_at	BCL6	B-cell CLL/lymphoma 6	chr3q27	604	LOC708736	similar to B-cell lymphoma 6 protein	---	708736	MmugDNA.12134.1.S1_at_BCL6
MmugDNA.12135.1.S1_at	226102_at	---	---	---	---	ZNF192	zinc finger protein 192	---	706188	MmugDNA.12135.1.S1_at_---
MmugDNA.12136.1.S1_at	233477_at	KLK15	kallikrein-related peptidase 15	chr19q13.41	55554	KLK15 /// LOC719422	kallikrein-related peptidase 15 /// similar to kallikrein 4	---	719422 /// 719456	MmugDNA.12136.1.S1_at_KLK15
MmugDNA.12137.1.S1_at	236792_at	---	---	---	---	LOC721893	similar to CG32662-PA	---	721893	MmugDNA.12137.1.S1_at_---
MmugDNA.12138.1.S1_at	241701_at	ARHGAP21	Rho GTPase activating protein 21	chr10p12.1|10p12.3	57584	ARHGAP21	Rho GTPase activating protein 21	---	706428	MmugDNA.12138.1.S1_at_ARHGAP21
MmugDNA.12139.1.S1_at	208036_at	OPN1SW	opsin 1 (cone pigments), short-wave-sensitive	chr7q32.1	611	LOC715800	Similar to protein phosphatase 4, regulatory subunit 2	---	715800	MmugDNA.12139.1.S1_at_OPN1SW
MmugDNA.12140.1.S1_at	235089_at	FBXL20	F-box and leucine-rich repeat protein 20	chr17q12	84961	FBXL20	F-box and leucine-rich repeat protein 20	---	697394	MmugDNA.12140.1.S1_at_FBXL20
MmugDNA.12141.1.S1_at	1557544_at	CCDC147	coiled-coil domain containing 147	chr10q25.1	159686	LOC710977	Similar to 60S ribosomal protein L12	---	710977	MmugDNA.12141.1.S1_at_CCDC147
MmugDNA.1214.1.S1_at	1560994_x_at	LOC400590	hypothetical LOC400590	chr17q11.2	400590	---	---	---	---	MmugDNA.1214.1.S1_at_LOC400590
MmugDNA.12142.1.S1_s_at	1554691_a_at	PACSIN2	protein kinase C and casein kinase substrate in neurons 2	chr22q13.2-q13.33	11252	PACSIN2	protein kinase C and casein kinase substrate in neurons 2	---	711242	MmugDNA.12142.1.S1_s_at_PACSIN2
MmugDNA.12143.1.S1_at	243760_at	LOC650794	similar to FRAS1 related extracellular matrix protein 2	chr13q12.11	650794	---	---	---	---	MmugDNA.12143.1.S1_at_LOC650794
MmugDNA.12144.1.S1_at	215895_x_at	ADFP	Adipose differentiation-related protein	chr9p22.1	123	---	---	---	---	MmugDNA.12144.1.S1_at_ADFP
MmugDNA.12145.1.S1_at	216788_at	---	---	---	---	---	---	---	---	MmugDNA.12145.1.S1_at_---
MmugDNA.12146.1.S1_at	214334_x_at	DAZAP2	DAZ associated protein 2	chr12q12	9802	LOC701019	Similar to actin-related protein T1	---	701019	MmugDNA.12146.1.S1_at_DAZAP2
MmugDNA.12147.1.S1_at	236561_at	TGFBR1	Transforming growth factor, beta receptor 1	chr9q22	7046	---	---	---	---	MmugDNA.12147.1.S1_at_TGFBR1
MmugDNA.12148.1.S1_at	229012_at	C9orf24	chromosome 9 open reading frame 24	chr9p13.3	84688	LOC712602	Similar to tau tubulin kinase 2	---	712602	MmugDNA.12148.1.S1_at_C9orf24
MmugDNA.12149.1.S1_at	1555820_a_at	MKS1	Meckel syndrome, type 1	chr17q22	54903	LOC714187	similar to Meckel syndrome, type 1	---	714187	MmugDNA.12149.1.S1_at_MKS1
MmugDNA.12150.1.S1_at	207510_at	BDKRB1	bradykinin receptor B1	chr14q32.1-q32.2	623	LOC712251	similar to bradykinin receptor B1	---	712251	MmugDNA.12150.1.S1_at_BDKRB1
MmugDNA.12151.1.S1_at	244324_at	C18orf54	chromosome 18 open reading frame 54	chr18q21.2	162681	LOC694192	similar to Protein C18orf54 precursor	---	694192	MmugDNA.12151.1.S1_at_C18orf54
MmugDNA.1215.1.S1_at	242362_at	---	---	---	---	---	---	---	---	MmugDNA.1215.1.S1_at_---
MmugDNA.12152.1.S1_at	236473_at	CC2D2A	coiled-coil and C2 domain containing 2A	chr4p15.33	57545	---	---	---	---	MmugDNA.12152.1.S1_at_CC2D2A
MmugDNA.12153.1.S1_at	202357_s_at	C2 /// CFB	complement component 2 /// complement factor B	chr6p21.3	629 /// 717	CFB	complement factor B	---	716809	MmugDNA.12153.1.S1_at_C2 /// CFB
MmugDNA.12154.1.S1_at	241751_at	OFD1	oral-facial-digital syndrome 1	chrXp22	8481	OFD1	oral-facial-digital syndrome 1	---	711677	MmugDNA.12154.1.S1_at_OFD1
MmugDNA.12155.1.S1_at	1558295_a_at	PPFIA2	protein tyrosine phosphatase, receptor type, f polypeptide (PTPRF), interacting protein (liprin), alpha	chr12q21.31	8499	PPFIA2	protein tyrosine phosphatase, receptor type, f polypeptide (PTPRF), interacting protein (liprin), alpha	---	698065	MmugDNA.12155.1.S1_at_PPFIA2
MmugDNA.12156.1.S1_at	227222_at	FBXO10	F-box protein 10	chr9p13.2	26267	FBXO10	F-box protein 10	---	718443	MmugDNA.12156.1.S1_at_FBXO10
MmugDNA.12157.1.S1_at	240786_at	NOTCH4	Notch homolog 4 (Drosophila)	chr6p21.3	4855	NOTCH4	notch4	---	717323	MmugDNA.12157.1.S1_at_NOTCH4
MmugDNA.12158.1.S1_at	1559566_at	FBXO42	F-box protein 42	chr1p36.23-p36.11	54455	---	---	---	---	MmugDNA.12158.1.S1_at_FBXO42
MmugDNA.12159.1.S1_at	212283_at	AGRN	agrin	chr1p36.33	375790	AGRN	similar to agrin	---	700400	MmugDNA.12159.1.S1_at_AGRN
MmugDNA.12160.1.S1_at	236168_at	---	---	---	---	---	---	---	---	MmugDNA.12160.1.S1_at_---
MmugDNA.12161.1.S1_at	224700_at	STT3B	STT3, subunit of the oligosaccharyltransferase complex, homolog B (S. cerevisiae)	chr3p23	201595	LOC703434	similar to source of immunodominant MHC-associated peptides	---	703434	MmugDNA.12161.1.S1_at_STT3B
MmugDNA.1216.1.S1_at	243169_at	---	---	---	---	---	---	---	---	MmugDNA.1216.1.S1_at_---
MmugDNA.12162.1.S1_s_at	211574_s_at	CD46	CD46 molecule, complement regulatory protein	chr1q32	4179	CD46	CD46 molecule, complement regulatory protein	---	714082	MmugDNA.12162.1.S1_s_at_CD46
MmugDNA.12163.1.S1_at	216826_at	---	---	---	---	---	---	---	---	MmugDNA.12163.1.S1_at_---
MmugDNA.12164.1.S1_at	NA	NA	NA	NA	NA	NA	NA	NA	NA	MmugDNA.12164.1.S1_at_NA
MmugDNA.12164.1.S1_s_at	212226_s_at	PPAP2B	phosphatidic acid phosphatase type 2B	chr1pter-p22.1	8613	LOC715993	similar to phosphatidic acid phosphatase type 2B	---	715993	MmugDNA.12164.1.S1_s_at_PPAP2B
MmugDNA.12165.1.S1_at	229043_at	PAPD5	PAP associated domain containing 5	chr16q12.1	64282	PAPD5	PAP associated domain containing 5	---	694589	MmugDNA.12165.1.S1_at_PAPD5
MmugDNA.12166.1.S1_at	212230_at	PPAP2B	phosphatidic acid phosphatase type 2B	chr1pter-p22.1	8613	LOC715993	similar to phosphatidic acid phosphatase type 2B	---	715993	MmugDNA.12166.1.S1_at_PPAP2B
MmugDNA.12167.1.S1_at	215608_at	---	---	---	---	---	---	---	---	MmugDNA.12167.1.S1_at_---
MmugDNA.12168.1.S1_at	243745_at	---	---	---	---	---	---	---	---	MmugDNA.12168.1.S1_at_---
MmugDNA.12169.1.S1_at	213289_at	APOOL	Apolipoprotein O-like	chrXq21.1	139322	---	---	---	---	MmugDNA.12169.1.S1_at_APOOL
MmugDNA.12170.1.S1_at	1562269_at	---	---	---	---	---	---	---	---	MmugDNA.12170.1.S1_at_---
MmugDNA.12171.1.S1_at	213282_at	APOOL	Apolipoprotein O-like	chrXq21.1	139322	---	---	---	---	MmugDNA.12171.1.S1_at_APOOL
MmugDNA.1217.1.S1_at	215228_at	NHLH2	nescient helix loop helix 2	chr1p12-p11	4808	LOC709920	similar to Helix-loop-helix protein 2 (HEN2) (Nescient helix loop helix 2) (NSCL-2)	---	709920	MmugDNA.1217.1.S1_at_NHLH2
MmugDNA.12172.1.S1_at	228289_at	---	---	---	---	---	---	---	---	MmugDNA.12172.1.S1_at_---
MmugDNA.12173.1.S1_at	213392_at	IQCK	IQ motif containing K	chr16p12.3	124152	LOC695417	hypothetical protein LOC695417	---	695417	MmugDNA.12173.1.S1_at_IQCK
MmugDNA.12174.1.S1_at	244102_at	---	---	---	---	LOC707817	hypothetical protein LOC707817	---	707817	MmugDNA.12174.1.S1_at_---
MmugDNA.12175.1.S1_at	212949_at	NCAPH	non-SMC condensin I complex, subunit H	chr2q11.2	23397	LOC697443	Hypothetical protein LOC697443	---	697443	MmugDNA.12175.1.S1_at_NCAPH
MmugDNA.12176.1.S1_at	236472_at	---	---	---	---	---	---	---	---	MmugDNA.12176.1.S1_at_---
MmugDNA.12177.1.S1_at	239475_at	ACPL2	acid phosphatase-like 2	chr3q23	92370	LOC715229	similar to acid phosphatase-like 2	---	715229	MmugDNA.12177.1.S1_at_ACPL2
MmugDNA.12178.1.S1_s_at	NA	NA	NA	NA	NA	NA	NA	NA	NA	MmugDNA.12178.1.S1_s_at_NA
MmugDNA.12179.1.S1_at	230178_s_at	---	---	---	---	LOC709483	similar to elongator protein 2	---	709483	MmugDNA.12179.1.S1_at_---
MmugDNA.12180.1.S1_at	1552626_a_at	TMEM163	transmembrane protein 163	chr2q21.3	81615	LOC709015	hypothetical protein LOC709015	---	709015	MmugDNA.12180.1.S1_at_TMEM163
MmugDNA.12181.1.S1_at	212628_at	PKN2	protein kinase N2	chr1p22.2	5586	---	---	---	---	MmugDNA.12181.1.S1_at_PKN2
MmugDNA.1218.1.S1_at	1568922_at	SLC24A4	solute carrier family 24 (sodium/potassium/calcium exchanger), member 4	chr14q32.12	123041	---	---	---	---	MmugDNA.1218.1.S1_at_SLC24A4
MmugDNA.12182.1.S1_at	1569544_at	---	---	---	---	---	---	---	---	MmugDNA.12182.1.S1_at_---
MmugDNA.12183.1.S1_at	233911_s_at	PPM1H	protein phosphatase 1H (PP2C domain containing)	chr12q14.1-q14.2	57460	LOC716958	similar to protein phosphatase 1H (PP2C domain containing)	---	716958	MmugDNA.12183.1.S1_at_PPM1H
MmugDNA.12184.1.S1_at	221723_s_at	SLC4A5	solute carrier family 4, sodium bicarbonate cotransporter, member 5	chr2p13	57835	LOC715025	similar to sodium bicarbonate transporter 4 isoform a	---	715025	MmugDNA.12184.1.S1_at_SLC4A5
MmugDNA.12185.1.S1_at	242235_x_at	---	---	---	---	---	---	---	---	MmugDNA.12185.1.S1_at_---
MmugDNA.12186.1.S1_at	229580_at	---	---	---	---	LOC715374	Similar to protein tyrosine phosphatase-like (proline instead of catalytic arginine), member b	---	715374	MmugDNA.12186.1.S1_at_---
MmugDNA.12187.1.S1_at	230907_at	GPRC5C	G protein-coupled receptor, family C, group 5, member C	chr17q25	55890	---	---	---	---	MmugDNA.12187.1.S1_at_GPRC5C
MmugDNA.12188.1.S1_at	238441_at	PRKAA2	protein kinase, AMP-activated, alpha 2 catalytic subunit	chr1p31	5563	---	---	---	---	MmugDNA.12188.1.S1_at_PRKAA2
MmugDNA.12189.1.S1_at	223560_s_at	C2orf56	chromosome 2 open reading frame 56	chr2p22.2	55471	LOC709473	similar to CG17726-PA	---	709473	MmugDNA.12189.1.S1_at_C2orf56
MmugDNA.12190.1.S1_at	224527_at	CDH23	cadherin-like 23	chr10q21-q22	64072	CDH23	cadherin related 23	---	709433	MmugDNA.12190.1.S1_at_CDH23
MmugDNA.12191.1.S1_at	244785_at	---	---	---	---	---	---	---	---	MmugDNA.12191.1.S1_at_---
MmugDNA.1219.1.S1_at	240111_at	RHOBTB3	Rho-related BTB domain containing 3	chr5q15	22836	---	---	---	---	MmugDNA.1219.1.S1_at_RHOBTB3
MmugDNA.12192.1.S1_at	216460_at	FLJ00049	FLJ00049 protein	chr7p22.2	645372	---	---	---	---	MmugDNA.12192.1.S1_at_FLJ00049
MmugDNA.12193.1.S1_at	213385_at	CHN2	chimerin (chimaerin) 2	chr7p15.3	1124	CHN2	chimerin (chimaerin) 2	---	697231	MmugDNA.12193.1.S1_at_CHN2
MmugDNA.12195.1.S1_at	241773_at	---	---	---	---	---	---	---	---	MmugDNA.12195.1.S1_at_---
MmugDNA.12196.1.S1_at	1566442_at	---	---	---	---	---	---	---	---	MmugDNA.12196.1.S1_at_---
MmugDNA.12197.1.S1_at	1560689_s_at	AKT2	V-akt murine thymoma viral oncogene homolog 2	chr19q13.1-q13.2	208	---	---	---	---	MmugDNA.12197.1.S1_at_AKT2
MmugDNA.12198.1.S1_at	215378_at	---	---	---	---	---	---	---	---	MmugDNA.12198.1.S1_at_---
MmugDNA.12199.1.S1_at	236362_at	---	---	---	---	---	---	---	---	MmugDNA.12199.1.S1_at_---
MmugDNA.12.1.S1_at	1562251_a_at	LOC574538	hypothetical protein LOC574538	chr12p13.33	574538	---	---	---	---	MmugDNA.12.1.S1_at_LOC574538
MmugDNA.12200.1.S1_at	231286_at	C9orf47	chromosome 9 open reading frame 47	chr9q22.1	286223	---	---	---	---	MmugDNA.12200.1.S1_at_C9orf47
MmugDNA.12201.1.S1_at	229872_s_at	LOC100132999	hypothetical protein LOC100132999	chr1q21.1	100132999	LOC702746	hypothetical protein LOC702746	---	702746	MmugDNA.12201.1.S1_at_LOC1001329
MmugDNA.1220.1.S1_at	1556815_at	---	---	---	---	---	---	---	---	MmugDNA.1220.1.S1_at_---
MmugDNA.12202.1.S1_at	227738_s_at	ARMC5	armadillo repeat containing 5	chr16p11.2	79798	LOC715103	similar to armadillo repeat containing 5	---	715103	MmugDNA.12202.1.S1_at_ARMC5
MmugDNA.12203.1.S1_at	1557954_at	CXorf15	chromosome X open reading frame 15	chrXp22.2	55787	LOC713769	gamma-taxilin	---	713769	MmugDNA.12203.1.S1_at_CXorf15
MmugDNA.12204.1.S1_x_at	215383_x_at	SPG21	spastic paraplegia 21 (autosomal recessive, Mast syndrome)	chr15q21-q22	51324	---	---	---	---	MmugDNA.12204.1.S1_x_at_SPG21
MmugDNA.12205.1.S1_at	240859_at	ZFYVE16	zinc finger, FYVE domain containing 16	chr5q14	9765	---	---	---	---	MmugDNA.12205.1.S1_at_ZFYVE16
MmugDNA.12206.1.S1_at	209694_at	PTS	6-pyruvoyltetrahydropterin synthase	chr11q22.3-q23.3	5805	PTS	6-pyruvoyltetrahydropterin synthase	---	711642	MmugDNA.12206.1.S1_at_PTS
MmugDNA.12207.1.S1_at	242376_at	---	---	---	---	---	---	---	---	MmugDNA.12207.1.S1_at_---
MmugDNA.12208.1.S1_at	237948_at	---	---	---	---	---	---	---	---	MmugDNA.12208.1.S1_at_---
MmugDNA.12209.1.S1_at	1559258_a_at	CXorf61	chromosome X open reading frame 61	chrXq23	203413	LOC709686	hypothetical protein LOC709686	---	709686	MmugDNA.12209.1.S1_at_CXorf61
MmugDNA.12210.1.S1_at	239295_at	SRrp35	serine-arginine repressor protein (35 kDa)	chr6q15	135295	SRRP35	serine-arginine repressor protein (35 kDa)	---	700497	MmugDNA.12210.1.S1_at_SRrp35
MmugDNA.12211.1.S1_at	210603_at	ARD1B	ARD1 homolog B (S. cerevisiae)	chr4q21.21	84779	---	---	---	---	MmugDNA.12211.1.S1_at_ARD1B
MmugDNA.1221.1.S1_at	200079_s_at	KARS	lysyl-tRNA synthetase	chr16q23-q24	3735	KARS	lysyl-tRNA synthetase	---	714516	MmugDNA.1221.1.S1_at_KARS
MmugDNA.1221.1.S1_s_at	NA	NA	NA	NA	NA	NA	NA	NA	NA	MmugDNA.1221.1.S1_s_at_NA
MmugDNA.12212.1.S1_at	203898_at	CRCP	CGRP receptor component	chr7q11.21	27297	LOC704831	similar to Calcitonin gene-related peptide-receptor component protein (CGRP-receptor component pr	---	704831	MmugDNA.12212.1.S1_at_CRCP
MmugDNA.12213.1.S1_at	241783_at	---	---	---	---	---	---	---	---	MmugDNA.12213.1.S1_at_---
MmugDNA.12214.1.S1_at	231804_at	RXFP1	relaxin/insulin-like family peptide receptor 1	chr4q32.1	59350	---	---	---	---	MmugDNA.12214.1.S1_at_RXFP1
MmugDNA.12215.1.S1_at	233082_at	ZNF630	zinc finger protein 630	chrXp11.3-p11.1	57232	LOC708541	similar to zinc finger protein 300	---	708541	MmugDNA.12215.1.S1_at_ZNF630
MmugDNA.12216.1.S1_at	244310_at	---	---	---	---	---	---	---	---	MmugDNA.12216.1.S1_at_---

MmugDNA.12217.1.S1_at	203836_s_at	MAP3K5	mitogen-activated protein kinase kinase kinase 5	chr6q22.33	4217	LOC720673	Similar to K11G12.6	---	720673	MmugDNA.12217.1.S1_at_MAP3K5
MmugDNA.12218.1.S1_at	228446_at	KIAA2026	KIAA2026	chr9p24.1	158358	KIAA2026	KIAA2026 protein	---	717388	MmugDNA.12218.1.S1_at_KIAA2026
MmugDNA.12219.1.S1_at	210690_at	KLRC4	killer cell lectin-like receptor subfamily C, member 4	chr12p13.2-p12.3	8302	---	---	---	---	MmugDNA.12219.1.S1_at_KLRC4
MmugDNA.122.1.S1_at	242094_at	---	---	---	---	---	---	---	---	MmugDNA.122.1.S1_at_---
MmugDNA.12220.1.S1_at	223440_at	C16orf70	chromosome 16 open reading frame 70	chr16q22.1	80262	C16orf70	chromosome 16 open reading frame 70	---	695885	MmugDNA.12220.1.S1_at_C16orf70
MmugDNA.12221.1.S1_at	222268_x_at	MUC5AC /// MUC5B	mucin 5AC, oligomeric mucus/gel-forming /// mucin 5B, oligomeric mucus/gel-forming	chr11p15.5	4586 /// 727897	---	---	---	---	MmugDNA.12221.1.S1_at_MUC5AC ///
MmugDNA.1222.1.S1_at	1555348_at	TFAP2E	transcription factor AP-2 epsilon (activating enhancer binding protein 2 epsilon)	chr1p34.3	339488	---	---	---	---	MmugDNA.1222.1.S1_at_TFAP2E
MmugDNA.12222.1.S1_at	210107_at	CLCA1	chloride channel accessory 1	chr1p31-p22	1179	CLCA1	chloride channel accessory 1	---	574295	MmugDNA.12222.1.S1_at_CLCA1
MmugDNA.12223.1.S1_at	236223_s_at	RIT1	Ras-like without CAAX 1	chr1q22	6016	---	---	---	---	MmugDNA.12223.1.S1_at_RIT1
MmugDNA.12224.1.S1_at	239343_at	LOC728705	hypothetical protein LOC728705	chr12q23.3	728705	---	---	---	---	MmugDNA.12224.1.S1_at_LOC728705
MmugDNA.12225.1.S1_at	205799_s_at	SLC3A1	solute carrier family 3 (cystine, dibasic and neutral amino acid transporters, activator of cystine, dibas	chr2p16.3	6519	LOC714035	similar to solute carrier family 3, member 1	---	714035	MmugDNA.12225.1.S1_at_SLC3A1
MmugDNA.12226.1.S1_at	227370_at	FAM171B	family with sequence similarity 171, member B	chr2q32.1	165215	FH	Fumarate hydratase	---	707542	MmugDNA.12226.1.S1_at_FAM171B
MmugDNA.12227.1.S1_at	222662_at	PPP1R3B	protein phosphatase 1, regulatory (inhibitor) subunit 3B	chr8p23.1	79660	---	---	---	---	MmugDNA.12227.1.S1_at_PPP1R3B
MmugDNA.12228.1.S1_at	NA	NA	NA	NA	NA	NA	NA	NA	NA	MmugDNA.12228.1.S1_at_NA
MmugDNA.12229.1.S1_at	224315_at	DDX20	DEAD (Asp-Glu-Ala-Asp) box polypeptide 20	chr1p21.1-p13.2	11218	DDX20	DEAD (Asp-Glu-Ala-Asp) box polypeptide 20	---	704731	MmugDNA.12229.1.S1_at_DDX20
MmugDNA.12230.1.S1_at	NA	NA	NA	NA	NA	NA	NA	NA	NA	MmugDNA.12230.1.S1_at_NA
MmugDNA.12230.1.S1_x_at	244755_at	XPR1	Xenotropic and polytropic retrovirus receptor	chr1q25.1	9213	---	---	---	---	MmugDNA.12230.1.S1_x_at_XPR1
MmugDNA.12231.1.S1_at	NA	NA	NA	NA	NA	NA	NA	NA	NA	MmugDNA.12231.1.S1_at_NA
MmugDNA.12231.1.S1_s_at	NA	NA	NA	NA	NA	NA	NA	NA	NA	MmugDNA.12231.1.S1_s_at_NA
MmugDNA.1223.1.S1_at	224416_s_at	MED28	mediator complex subunit 28	chr4p16	80306	---	---	---	---	MmugDNA.1223.1.S1_at_MED28
MmugDNA.12232.1.S1_at	224612_s_at	DNAJC5	DnaJ (Hsp40) homolog, subfamily C, member 5	chr20q13.33	80331	---	---	---	---	MmugDNA.12232.1.S1_at_DNAJC5
MmugDNA.12233.1.S1_at	235715_at	FLJ44653	FLJ44653 protein	chr10q26.3	399833	---	---	---	---	MmugDNA.12233.1.S1_at_FLJ44653
MmugDNA.12234.1.S1_at	209649_at	STAM2	signal transducing adaptor molecule (SH3 domain and ITAM motif) 2	chr2q23.3	10254	STAM2	signal transducing adaptor molecule 2	---	695286	MmugDNA.12234.1.S1_at_STAM2
MmugDNA.12235.1.S1_at	243756_at	---	---	---	---	---	---	---	---	MmugDNA.12235.1.S1_at_---
MmugDNA.12236.1.S1_at	1561332_at	ATP13A5	ATPase type 13A5	chr3q29	344905	LOC715046	Hypothetical protein LOC715046	---	715046	MmugDNA.12236.1.S1_at_ATP13A5
MmugDNA.12237.1.S1_at	242227_at	---	---	---	---	---	---	---	---	MmugDNA.12237.1.S1_at_---
MmugDNA.12238.1.S1_at	210883_x_at	EFNB3	ephrin-B3	chr17p13.1-p11.2	1949	EFNB3	ephrin-B3	---	716257	MmugDNA.12238.1.S1_at_EFNB3
MmugDNA.12239.1.S1_at	209163_at	CYB561	cytochrome b-561	chr17q11-qter	1534	---	---	---	---	MmugDNA.12239.1.S1_at_CYB561
MmugDNA.12240.1.S1_at	233481_at	---	---	---	---	---	---	---	---	MmugDNA.12240.1.S1_at_---
MmugDNA.12241.1.S1_at	241538_at	CXorf30	chromosome X open reading frame 30	chrXp21.1	645090	LOC695721	hypothetical protein LOC695721	---	695721	MmugDNA.12241.1.S1_at_CXorf30
MmugDNA.1224.1.S1_s_at	210967_x_at	CACNB1	calcium channel, voltage-dependent, beta 1 subunit	chr17q21-q22	782	CACNB1	calcium channel, voltage-dependent, beta 1 subunit	---	695864	MmugDNA.1224.1.S1_s_at_CACNB1
MmugDNA.12242.1.S1_at	243703_x_at	---	---	---	---	---	---	---	---	MmugDNA.12242.1.S1_at_---
MmugDNA.12243.1.S1_at	235196_at	---	---	---	---	---	---	---	---	MmugDNA.12243.1.S1_at_---
MmugDNA.12244.1.S1_at	241055_at	EPAS1	Endothelial PAS domain protein 1	chr2p21-p16	2034	---	---	---	---	MmugDNA.12244.1.S1_at_EPAS1
MmugDNA.12245.1.S1_at	1556176_at	TAF8	TAF8 RNA polymerase II, TATA box binding protein (TBP)-associated factor, 43kDa	chr6p21.1	129685	---	---	---	---	MmugDNA.12245.1.S1_at_TAF8
MmugDNA.12246.1.S1_at	214900_at	ZKSCAN1	zinc finger with KRAB and SCAN domains 1	chr7q21.3-q22.1	7586	LOC710407	similar to zinc finger protein 36	---	710407	MmugDNA.12246.1.S1_at_ZKSCAN1
MmugDNA.12247.1.S1_at	211075_s_at	CD47	CD47 molecule	chr3q13.1-q13.2	961	CD47	CD47 molecule	---	704980	MmugDNA.12247.1.S1_at_CD47
MmugDNA.12248.1.S1_at	1562634_at	---	---	---	---	---	---	---	---	MmugDNA.12248.1.S1_at_---
MmugDNA.12249.1.S1_at	NA	NA	NA	NA	NA	NA	NA	NA	NA	MmugDNA.12249.1.S1_at_NA
MmugDNA.12249.1.S1_s_at	201752_s_at	ADD3	adducin 3 (gamma)	chr10q24.2-q24.3	120	ADD3	adducin 3 (gamma)	---	677712	MmugDNA.12249.1.S1_s_at_ADD3
MmugDNA.12250.1.S1_at	1554043_a_at	---	---	---	---	---	---	---	---	MmugDNA.12250.1.S1_at_---
MmugDNA.12251.1.S1_at	225732_at	KLHDC5	kelch domain containing 5	chr12p11.22	57542	KLHDC5	kelch domain containing 5	---	711593	MmugDNA.12251.1.S1_at_KLHDC5
MmugDNA.1225.1.S1_x_at	NA	NA	NA	NA	NA	NA	NA	NA	NA	MmugDNA.1225.1.S1_x_at_NA
MmugDNA.12252.1.S1_at	235539_at	NUMA1	Nuclear mitotic apparatus protein 1	chr11q13	4926	---	---	---	---	MmugDNA.12252.1.S1_at_NUMA1
MmugDNA.12253.1.S1_at	225183_at	C16orf72	chromosome 16 open reading frame 72	chr16p13.2	29035	LOC709573	similar to CG4768-PA	---	709573	MmugDNA.12253.1.S1_at_C16orf72
MmugDNA.12254.1.S1_at	233602_at	---	---	---	---	---	---	---	---	MmugDNA.12254.1.S1_at_---
MmugDNA.12255.1.S1_at	1559057_at	---	---	---	---	---	---	---	---	MmugDNA.12255.1.S1_at_---
MmugDNA.12256.1.S1_at	211391_s_at	PATZ1	POZ (BTB) and AT hook containing zinc finger 1	chr22q12.2	23598	LOC718381	similar to zinc finger protein 278 long C isoform	---	718381	MmugDNA.12256.1.S1_at_PATZ1
MmugDNA.12256.1.S1_s_at	210581_x_at	PATZ1	POZ (BTB) and AT hook containing zinc finger 1	chr22q12.2	23598	LOC718381	similar to zinc finger protein 278 long C isoform	---	718381	MmugDNA.12256.1.S1_s_at_PATZ1
MmugDNA.12257.1.S1_at	210555_s_at	NFATC3	nuclear factor of activated T-cells, cytoplasmic, calcineurin-dependent 3	chr16q22.2	4775	LOC702390	similar to cytoplasmic nuclear factor of activated T-cells 3 isoform 1	---	702390	MmugDNA.12257.1.S1_at_NFATC3
MmugDNA.12259.1.S1_at	1558578_a_at	SLC13A4	solute carrier family 13 (sodium/sulfate symporters), member 4	chr7q33	26266	---	---	---	---	MmugDNA.12259.1.S1_at_SLC13A4
MmugDNA.12260.1.S1_at	217248_s_at	SLC7A8	solute carrier family 7 (cationic amino acid transporter, y+ system), member 8	chr14q11.2	23428	SLC7A8	solute carrier family 7 (cationic amino acid transporter, y+ system), member 8	---	713511	MmugDNA.12260.1.S1_at_SLC7A8
MmugDNA.12261.1.S1_at	236809_at	---	---	---	---	---	---	---	---	MmugDNA.12261.1.S1_at_---
MmugDNA.1226.1.S1_at	1563392_at	---	---	---	---	---	---	---	---	MmugDNA.1226.1.S1_at_---
MmugDNA.12262.1.S1_at	213313_at	RABGAP1	RAB GTPase activating protein 1	chr9q33.2	23637	---	---	---	---	MmugDNA.12262.1.S1_at_RABGAP1
MmugDNA.12263.1.S1_at	1562280_at	---	---	---	---	---	---	---	---	MmugDNA.12263.1.S1_at_---
MmugDNA.12264.1.S1_at	213953_at	KRT20	keratin 20	chr17q21.2	54474	KRT20	keratin 20	---	702602	MmugDNA.12264.1.S1_at_KRT20
MmugDNA.12265.1.S1_at	1552960_at	LRRC15	leucine rich repeat containing 15	chr3q29	131578	LRRC15	leucine rich repeat containing 15	---	707668	MmugDNA.12265.1.S1_at_LRRC15
MmugDNA.12266.1.S1_at	1561187_at	---	---	---	---	---	---	---	---	MmugDNA.12266.1.S1_at_---
MmugDNA.12267.1.S1_at	232251_at	NUDT16P	nudix (nucleoside diphosphate linked moiety X)-type motif 16 pseudogene	chr3q22.1	152195	LOC717946	similar to nudix-type motif 16	---	717946	MmugDNA.12267.1.S1_at_NUDT16P
MmugDNA.12268.1.S1_at	1553629_a_at	FAM71B	family with sequence similarity 71, member B	chr5q33.3	153745	---	---	---	---	MmugDNA.12268.1.S1_at_FAM71B
MmugDNA.12269.1.S1_s_at	216536_at	OR7E19P	olfactory receptor, family 7, subfamily E, member 19 pseudogene	chr19p13.2	26651	LOC713326	similar to olfactory receptor Olr1166	---	713326	MmugDNA.12269.1.S1_s_at_OR7E19P
MmugDNA.12270.1.S1_at	1570326_at	---	---	---	---	---	---	---	---	MmugDNA.12270.1.S1_at_---
MmugDNA.12271.1.S1_at	NA	NA	NA	NA	NA	NA	NA	NA	NA	MmugDNA.12271.1.S1_at_NA
MmugDNA.1227.1.S1_s_at	209882_at	RIT1	Ras-like without CAAX 1	chr1q22	6016	RIT1	Ras-like without CAAX 1	---	717985	MmugDNA.1227.1.S1_s_at_RIT1
MmugDNA.12272.1.S1_at	237355_at	---	---	---	---	---	---	---	---	MmugDNA.12272.1.S1_at_---
MmugDNA.12273.1.S1_at	201291_s_at	TOP2A	topoisomerase (DNA) II alpha 170kDa	chr17q21-q22	7153	TOP2A	DNA topoisomerase II, alpha isozyme	---	700707	MmugDNA.12273.1.S1_at_TOP2A
MmugDNA.12274.1.S1_at	223553_s_at	DOK3	docking protein 3	chr5q35.3	79930	---	---	---	---	MmugDNA.12274.1.S1_at_DOK3
MmugDNA.12275.1.S1_at	243611_at	MICALCL	MICAL C-terminal like	chr11p15.3	84953	MICALCL	MICAL C-terminal like	---	701303	MmugDNA.12275.1.S1_at_MICALCL
MmugDNA.12276.1.S1_at	215739_s_at	TUBGCP3	tubulin, gamma complex associated protein 3	chr13q34	10426	---	---	---	---	MmugDNA.12276.1.S1_at_TUBGCP3
MmugDNA.12277.1.S1_at	201292_at	TOP2A	topoisomerase (DNA) II alpha 170kDa	chr17q21-q22	7153	TOP2A	DNA topoisomerase II, alpha isozyme	---	700707	MmugDNA.12277.1.S1_at_TOP2A
MmugDNA.12278.1.S1_at	1561857_at	---	---	---	---	---	---	---	---	MmugDNA.12278.1.S1_at_---
MmugDNA.12279.1.S1_at	223932_at	---	---	---	---	---	---	---	---	MmugDNA.12279.1.S1_at_---
MmugDNA.12280.1.S1_at	235674_at	KIAA0922	KIAA0922	chr4q31.3	23240	---	---	---	---	MmugDNA.12280.1.S1_at_KIAA0922
MmugDNA.12281.1.S1_at	241091_at	---	---	---	---	---	---	---	---	MmugDNA.12281.1.S1_at_---
MmugDNA.12281.1.S1_x_at	NA	NA	NA	NA	NA	NA	NA	NA	NA	MmugDNA.12281.1.S1_x_at_NA
MmugDNA.1228.1.S1_at	1557481_a_at	C21orf131	chromosome 21 open reading frame 131	---	387486	---	---	---	---	MmugDNA.1228.1.S1_at_C21orf131
MmugDNA.12282.1.S1_at	1555468_at	NRP2	neuropilin 2	chr2q33.3	8828	---	---	---	---	MmugDNA.12282.1.S1_at_NRP2
MmugDNA.12283.1.S1_at	1554354_at	SIAE	sialic acid acetylesterase	chr11q24	54414	---	---	---	---	MmugDNA.12283.1.S1_at_SIAE
MmugDNA.12284.1.S1_at	234607_at	ARRDC1	arrestin domain containing 1	chr9q34.3	92714	---	---	---	---	MmugDNA.12284.1.S1_at_ARRDC1
MmugDNA.12285.1.S1_at	240582_x_at	---	---	---	---	---	---	---	---	MmugDNA.12285.1.S1_at_---
MmugDNA.12286.1.S1_at	232798_at	DNAJC5B	DnaJ (Hsp40) homolog, subfamily C, member 5 beta	chr8q13.1	85479	DNAJC5B	DnaJ (Hsp40) homolog, subfamily C, member 5 beta	---	702408	MmugDNA.12286.1.S1_at_DNAJC5B
MmugDNA.12287.1.S1_at	239036_at	---	---	---	---	---	---	---	---	MmugDNA.12287.1.S1_at_---
MmugDNA.12288.1.S1_at	226594_at	---	---	---	---	---	---	---	---	MmugDNA.12288.1.S1_at_---
MmugDNA.12289.1.S1_at	244691_at	SETD5	SET domain containing 5	chr3p25.3	55209	SETD5	SET domain containing 5	---	704174	MmugDNA.12289.1.S1_at_SETD5
MmugDNA.12290.1.S1_at	232428_at	MOGAT2	monoacylglycerol O-acyltransferase 2	chr11q13.5	80168	---	---	---	---	MmugDNA.12290.1.S1_at_MOGAT2
MmugDNA.12291.1.S1_at	1558041_a_at	KIAA0895L	KIAA0895-like	chr16q22.1	653319	LOC696623	hypothetical protein LOC696623	---	696623	MmugDNA.12291.1.S1_at_KIAA0895L
MmugDNA.1229.1.S1_s_at	200653_s_at	CALM1	calmodulin 1 (phosphorylase kinase, delta)	chr14q24-q31	801	LOC720263	Similar to protein kinase C, gamma	---	720263	MmugDNA.1229.1.S1_s_at_CALM1
MmugDNA.12292.1.S1_at	1554251_at	HP1BP3	heterochromatin protein 1, binding protein 3	chr1p36.12	50809	---	---	---	---	MmugDNA.12292.1.S1_at_HP1BP3
MmugDNA.12293.1.S1_at	222997_s_at	MRPS21	mitochondrial ribosomal protein S21	chr1q21	54460	---	---	---	---	MmugDNA.12293.1.S1_at_MRPS21
MmugDNA.12294.1.S1_at	1556435_at	LOC400622	hypothetical LOC400622	chr17q25.3	400622	---	---	---	---	MmugDNA.12294.1.S1_at_LOC400622
MmugDNA.12295.1.S1_at	215766_at	GSTA1	Glutathione S-transferase alpha 1	chr6p12.1	2938	---	---	---	---	MmugDNA.12295.1.S1_at_GSTA1
MmugDNA.12296.1.S1_at	222206_s_at	NCLN	nicalin homolog (zebrafish)	chr19p13.3	56926	LOC712703	similar to nicalin	---	712703	MmugDNA.12296.1.S1_at_NCLN
MmugDNA.12297.1.S1_at	NA	NA	NA	NA	NA	NA	NA	NA	NA	MmugDNA.12297.1.S1_at_NA
MmugDNA.12298.1.S1_x_at	NA	NA	NA	NA	NA	NA	NA	NA	NA	MmugDNA.12298.1.S1_x_at_NA
MmugDNA.12299.1.S1_at	1554648_a_at	DUOXA1	dual oxidase maturation factor 1	chr15q21.1	90527	LOC713058	similar to Numb-interacting protein	---	713058	MmugDNA.12299.1.S1_at_DUOXA1
MmugDNA.12300.1.S1_at	240825_at	---	---	---	---	---	---	---	---	MmugDNA.12300.1.S1_at_---
MmugDNA.12301.1.S1_at	1570411_at	LOC100133131	hypothetical protein LOC100133131	chr4p16.1	100133131	---	---	---	---	MmugDNA.12301.1.S1_at_LOC100133
MmugDNA.1230.1.S1_at	236098_at	hCG_1643808	hypothetical LOC100130933	---	100130933	LOC703664	hypothetical protein LOC703664	---	703664	MmugDNA.1230.1.S1_at_hCG_164380
MmugDNA.12302.1.S1_at	222637_at	COMMD10	COMM domain containing 10	chr5q23.1	51397	---	---	---	---	MmugDNA.12302.1.S1_at_COMMD10
MmugDNA.12303.1.S1_at	233417_at	---	---	---	---	---	---	---	---	MmugDNA.12303.1.S1_at_---
MmugDNA.12304.1.S1_at	235133_at	---	---	---	---	---	---	---	---	MmugDNA.12304.1.S1_at_---
MmugDNA.12305.1.S1_at	202804_at	ABCC1	ATP-binding cassette, sub-family C (CFTR/MRP), member 1	chr16p13.1	4363	LOC717857	similar to ATP-binding cassette, sub-family C, member 1 isoform 6	---	717857	MmugDNA.12305.1.S1_at_ABCC1
MmugDNA.12306.1.S1_at	243003_at	---	---	---	---	---	---	---	---	MmugDNA.12306.1.S1_at_---
MmugDNA.12308.1.S1_at	237296_at	---	---	---	---	---	---	---	---	MmugDNA.12308.1.S1_at_---
MmugDNA.12309.1.S1_at	237838_at	---	---	---	---	---	---	---	---	MmugDNA.12309.1.S1_at_---
MmugDNA.12310.1.S1_at	227369_at	SERBP1	SERPINE1 mRNA binding protein 1	chr1p31	26135	---	---	---	---	MmugDNA.12310.1.S1_at_SERBP1
MmugDNA.12311.1.S1_at	230508_at	DKK3	dickkopf homolog 3 (Xenopus laevis)	chr11p15.2	27122	---	---	---	---	MmugDNA.12311.1.S1_at_DKK3

MmugDNA.1231.1.S1_at	240260_at	---	---	---	---	LOC700866	Hypothetical protein LOC700866	---	700866	MmugDNA.1231.1.S1_at_---
MmugDNA.12312.1.S1_at	241097_at	---	---	---	---	---	---	---	---	MmugDNA.12312.1.S1_at_---
MmugDNA.12313.1.S1_at	212396_s_at	KIAA0090	KIAA0090	chr1p36.13	23065	---	---	---	---	MmugDNA.12313.1.S1_at_KIAA0090
MmugDNA.12314.1.S1_at	212395_s_at	KIAA0090	KIAA0090	chr1p36.13	23065	---	---	---	---	MmugDNA.12314.1.S1_at_KIAA0090
MmugDNA.12315.1.S1_at	214229_at	DNAH17	dynein, axonemal, heavy chain 17	chr17q25.3	8632	LOC715728	similar to dynein, axonemal, heavy polypeptide 17	---	715728	MmugDNA.12315.1.S1_at_DNAH17
MmugDNA.12316.1.S1_at	NA	NA	NA	NA	NA	NA	NA	NA	NA	MmugDNA.12316.1.S1_at_NA
MmugDNA.12317.1.S1_at	237606_at	CD53	CD53 molecule	chr1p13	963	---	---	---	---	MmugDNA.12317.1.S1_at_CD53
MmugDNA.12318.1.S1_at	228754_at	SLC6A6	solute carrier family 6 (neurotransmitter transporter, taurine), member 6	chr3p25-p24	6533	---	---	---	---	MmugDNA.12318.1.S1_at_SLC6A6
MmugDNA.12319.1.S1_at	239382_at	HPS1	Hermansky-Pudlak syndrome 1	chr10q23.1-q23.3	3257	LOC705948	Similar to N-acylaminoacyl-peptide hydrolase	---	705948	MmugDNA.12319.1.S1_at_HPS1
MmugDNA.123.1.S1_at	244131_at	HNRNPA2B1	heterogeneous nuclear ribonucleoprotein A2/B1	chr7p15	3181	---	---	---	---	MmugDNA.123.1.S1_at_HNRNPA2B1
MmugDNA.12320.1.S1_at	1561906_at	---	---	---	---	---	---	---	---	MmugDNA.12320.1.S1_at_---
MmugDNA.12321.1.S1_at	223040_at	NAT5	N-acetyltransferase 5 (GCN5-related, putative)	chr20p11.23	51126	---	---	---	---	MmugDNA.12321.1.S1_at_NAT5
MmugDNA.1232.1.S1_at	230906_at	GALNT10	UDP-N-acetyl-alpha-D-galactosamine:polypeptide N-acetylgalactosaminyltransferase 10 (GalNAc-T1	chr5q33.2	55568	---	---	---	---	MmugDNA.1232.1.S1_at_GALNT10
MmugDNA.12322.1.S1_at	1561421_a_at	---	---	---	---	---	---	---	---	MmugDNA.12322.1.S1_at_---
MmugDNA.12323.1.S1_at	208742_s_at	SAP18	Sin3A-associated protein, 18kDa	chr13q12.11	10284	SAP18	Sin3A-associated protein, 18kDa	---	703078	MmugDNA.12323.1.S1_at_SAP18
MmugDNA.12324.1.S1_at	1561651_s_at	TAL1	T-cell acute lymphocytic leukemia 1	chr1p32	6886	---	---	---	---	MmugDNA.12324.1.S1_at_TAL1
MmugDNA.12325.1.S1_at	234922_s_at	NHSL1	NHS-like 1	chr6q23.3	57224	NHS	similar to NHS-like 1	---	703448	MmugDNA.12325.1.S1_at_NHSL1
MmugDNA.12326.1.S1_at	1560257_at	---	---	---	---	---	---	---	---	MmugDNA.12326.1.S1_at_---
MmugDNA.12327.1.S1_at	235660_at	---	---	---	---	---	---	---	---	MmugDNA.12327.1.S1_at_---
MmugDNA.12328.1.S1_at	225617_at	ODF2	outer dense fiber of sperm tails 2	chr9q34.11	4957	LOC717505	similar to outer dense fiber of sperm tails 2 isoform 1	---	717505	MmugDNA.12328.1.S1_at_ODF2
MmugDNA.12329.1.S1_at	208853_s_at	CANX	calnexin	chr5q35	821	CYP2A23	Cytochrome P450, family 2, subfamily A, polypeptide 23	---	678690	MmugDNA.12329.1.S1_at_CANX
MmugDNA.12330.1.S1_at	1559631_at	---	---	---	---	---	---	---	---	MmugDNA.12330.1.S1_at_---
MmugDNA.12331.1.S1_x_at	NA	NA	NA	NA	NA	NA	NA	NA	NA	MmugDNA.12331.1.S1_x_at_NA
MmugDNA.1233.1.S1_at	214030_at	CRYBG3	beta-gamma crystallin domain containing 3	chr3q11.2	131544	GAPDH	Similar to Glyceraldehyde-3-phosphate dehydrogenase (GAPDH)	---	715293	MmugDNA.1233.1.S1_at_CRYBG3
MmugDNA.12332.1.S1_at	239340_at	---	---	---	---	LOC706403	Similar to ubiquitin specific protease 16 isoform a	---	706403	MmugDNA.12332.1.S1_at_---
MmugDNA.12333.1.S1_at	235181_at	C2orf60	chromosome 2 open reading frame 60	chr2q33.1	129450	JMJD5	similar to CG13902-PA	---	699197	MmugDNA.12333.1.S1_at_C2orf60
MmugDNA.12334.1.S1_at	214706_at	ZNF200	zinc finger protein 200	chr16p13.3	7752	---	---	---	---	MmugDNA.12334.1.S1_at_ZNF200
MmugDNA.12335.1.S1_at	1564630_at	EDN1	endothelin 1	chr6p24.1	1906	---	---	---	---	MmugDNA.12335.1.S1_at_EDN1
MmugDNA.12336.1.S1_at	1563894_at	LOC441178	hypothetical LOC441178	chr6q27	441178	---	---	---	---	MmugDNA.12336.1.S1_at_LOC441178
MmugDNA.12337.1.S1_at	225350_s_at	ZYG11B	zyg-11 homolog B (C. elegans)	chr1p32.3	79699	---	---	---	---	MmugDNA.12337.1.S1_at_ZYG11B
MmugDNA.12338.1.S1_at	225338_at	ZYG11B	zyg-11 homolog B (C. elegans)	chr1p32.3	79699	---	---	---	---	MmugDNA.12338.1.S1_at_ZYG11B
MmugDNA.12339.1.S1_at	234121_at	LOC100129069	hypothetical protein LOC100129069	chr11q24.2	100129069	LOC712271	hypothetical protein LOC712271	---	712271	MmugDNA.12339.1.S1_at_LOC100129
MmugDNA.12340.1.S1_at	223814_at	TRNT1	tRNA nucleotidyl transferase, CCA-adding, 1	---	51095	---	---	---	---	MmugDNA.12340.1.S1_at_TRNT1
MmugDNA.12341.1.S1_at	241688_at	---	---	---	---	---	---	---	---	MmugDNA.12341.1.S1_at_---
MmugDNA.1234.1.S1_at	241728_at	---	---	---	---	---	---	---	---	MmugDNA.1234.1.S1_at_---
MmugDNA.12342.1.S1_at	1552532_a_at	ATP6V1C2	ATPase, H+ transporting, lysosomal 42kDa, V1 subunit C2	---	245973	---	---	---	---	MmugDNA.12342.1.S1_at_ATP6V1C2
MmugDNA.12343.1.S1_at	236874_at	LOC100128071	Similar to hCG41624	chr1p35.1	100128071	---	---	---	---	MmugDNA.12343.1.S1_at_LOC100128
MmugDNA.12344.1.S1_at	217270_s_at	DYRK1B	dual-specificity tyrosine-(Y)-phosphorylation regulated kinase 1B	chr19q12-q13.1	9149	LOC698848	similar to dual-specificity tyrosine-(Y)-phosphorylation regulated kinase 1B isoform b	---	698848	MmugDNA.12344.1.S1_at_DYRK1B
MmugDNA.12345.1.S1_at	222760_at	ZNF703	zinc finger protein 703	chr8p12	80139	ZNF703	zinc finger protein 703	---	698000	MmugDNA.12345.1.S1_at_ZNF703
MmugDNA.12346.1.S1_at	243861_at	---	---	---	---	---	---	---	---	MmugDNA.12346.1.S1_at_---
MmugDNA.12347.1.S1_at	244312_at	---	---	---	---	---	---	---	---	MmugDNA.12347.1.S1_at_---
MmugDNA.12348.1.S1_at	242379_at	---	---	---	---	---	---	---	---	MmugDNA.12348.1.S1_at_---
MmugDNA.12349.1.S1_at	236461_at	ABHD1	abhydrolase domain containing 1	chr2p23.3	84696	ABHD1	abhydrolase domain containing 1	---	698700	MmugDNA.12349.1.S1_at_ABHD1
MmugDNA.12350.1.S1_at	211174_s_at	CCKAR	cholecystokinin A receptor	chr4p15.1-p15.2	886	CCKAR	cholecystokinin A receptor	---	694742	MmugDNA.12350.1.S1_at_CCKAR
MmugDNA.12351.1.S1_at	230971_x_at	GLTPD2	glycolipid transfer protein domain containing 2	chr17p13.2	388323	LOC709758	hypothetical protein LOC709758	---	709758	MmugDNA.12351.1.S1_at_GLTPD2
MmugDNA.1235.1.S1_at	1558534_at	LOC440354 /// LOC641	PI-3-kinase-related kinase SMG-1 pseudogene /// SMG1 homolog, phosphatidylinositol 3-kinase-relat	chr16p11.2 /// chr16p12.2	440354 /// 641298	LOC693542	similar to PI-3-kinase-related kinase SMG-1	---	693542	MmugDNA.1235.1.S1_at_LOC440354 //
MmugDNA.12352.1.S1_at	237711_at	ZNF705G	zinc finger protein 705G-like	chr8p23.1	100131980	---	---	---	---	MmugDNA.12352.1.S1_at_ZNF705G
MmugDNA.12353.1.S1_at	238424_at	ADAL	adenosine deaminase-like	chr15q15.3	161823	---	---	---	---	MmugDNA.12353.1.S1_at_ADAL
MmugDNA.12354.1.S1_at	214575_s_at	AZU1	azurocidin 1	chr19p13.3	566	---	---	---	---	MmugDNA.12354.1.S1_at_AZU1
MmugDNA.12355.1.S1_at	242639_at	NARG2	NMDA receptor regulated 2	chr15q22.2	79664	---	---	---	---	MmugDNA.12355.1.S1_at_NARG2
MmugDNA.12356.1.S1_at	215023_s_at	PEX1	peroxisomal biogenesis factor 1	chr7q21.2	5189	PEX1	peroxisomal biogenesis factor 1	---	702392	MmugDNA.12356.1.S1_at_PEX1
MmugDNA.12357.1.S1_at	1562342_at	---	---	---	---	---	---	---	---	MmugDNA.12357.1.S1_at_---
MmugDNA.12358.1.S1_at	241225_at	---	---	---	---	---	---	---	---	MmugDNA.12358.1.S1_at_---
MmugDNA.12359.1.S1_at	238153_at	PDE6B	phosphodiesterase 6B, cGMP-specific, rod, beta	chr4p16.3	5158	---	---	---	---	MmugDNA.12359.1.S1_at_PDE6B
MmugDNA.12360.1.S1_at	1556493_a_at	KDM4C	lysine (K)-specific demethylase 4C	chr9p24.1	23081	---	---	---	---	MmugDNA.12360.1.S1_at_KDM4C
MmugDNA.12361.1.S1_at	241918_at	ANO7	anoctamin 7	chr2q37.3	50636	LOC700819	similar to transmembrane protein 16G isoform NGEP long	---	700819	MmugDNA.12361.1.S1_at_ANO7
MmugDNA.1236.1.S1_at	222839_s_at	PAPOLG	poly(A) polymerase gamma	chr2p16.1	64895	LOC717781	similar to poly(A) polymerase gamma	---	717781	MmugDNA.1236.1.S1_at_PAPOLG
MmugDNA.12362.1.S1_at	240520_at	---	---	---	---	---	---	---	---	MmugDNA.12362.1.S1_at_---
MmugDNA.12363.1.S1_at	225704_at	FBRSL1	fibrosin-like 1	chr12q24.33	57666	---	---	---	---	MmugDNA.12363.1.S1_at_FBRSL1
MmugDNA.12364.1.S1_at	NA	NA	NA	NA	NA	NA	NA	NA	NA	MmugDNA.12364.1.S1_at_NA
MmugDNA.12365.1.S1_at	NA	NA	NA	NA	NA	NA	NA	NA	NA	MmugDNA.12365.1.S1_at_NA
MmugDNA.12366.1.S1_at	237417_at	RCBTB1	Regulator of chromosome condensation (RCC1) and BTB (POZ) domain containing protein 1	chr13q14	55213	---	---	---	---	MmugDNA.12366.1.S1_at_RCBTB1
MmugDNA.12367.1.S1_at	239418_x_at	---	---	---	---	---	---	---	---	MmugDNA.12367.1.S1_at_---
MmugDNA.12368.1.S1_at	238740_at	AARSD1	alanyl-tRNA synthetase domain containing 1	chr17q21.31	80755	---	---	---	---	MmugDNA.12368.1.S1_at_AARSD1
MmugDNA.12369.1.S1_at	235251_at	---	---	---	---	---	---	---	---	MmugDNA.12369.1.S1_at_---
MmugDNA.12370.1.S1_at	227356_at	---	---	---	---	---	---	---	---	MmugDNA.12370.1.S1_at_---
MmugDNA.12371.1.S1_at	223692_at	NMNAT1	nicotinamide nucleotide adenylyltransferase 1	chr1p32-p35	64802	LOC722599	similar to nicotinamide nucleotide adenylyltransferase 1	---	722599	MmugDNA.12371.1.S1_at_NMNAT1
MmugDNA.1237.1.S1_at	242347_at	---	---	---	---	---	---	---	---	MmugDNA.1237.1.S1_at_---
MmugDNA.12372.1.S1_at	233188_at	GALNTL4	UDP-N-acetyl-alpha-D-galactosamine:polypeptide N-acetylgalactosaminyltransferase-like 4	chr11p15.3	374378	---	---	---	---	MmugDNA.12372.1.S1_at_GALNTL4
MmugDNA.12373.1.S1_at	NA	NA	NA	NA	NA	NA	NA	NA	NA	MmugDNA.12373.1.S1_at_NA
MmugDNA.12374.1.S1_at	223298_s_at	NT5C3	5'-nucleotidase, cytosolic III	chr7p14.3	51251	LOC708936	Similar to protein phosphatase 1, regulatory (inhibitor) subunit 2	---	708936	MmugDNA.12374.1.S1_at_NT5C3
MmugDNA.12375.1.S1_at	218754_at	NOL9	nucleolar protein 9	chr1p36.31	79707	---	---	---	---	MmugDNA.12375.1.S1_at_NOL9
MmugDNA.12376.1.S1_at	226195_at	C14orf179	chromosome 14 open reading frame 179	chr14q24.3	112752	LOC703348	hypothetical protein LOC703348	---	703348	MmugDNA.12376.1.S1_at_C14orf179
MmugDNA.12377.1.S1_at	1553389_at	NPHP3	nephronophthisis 3 (adolescent)	chr3q22.1	27031	---	---	---	---	MmugDNA.12377.1.S1_at_NPHP3
MmugDNA.12378.1.S1_at	1570393_at	EML5	echinoderm microtubule associated protein like 5	chr14q31.3	161436	LOC694095	similar to echinoderm microtubule associated protein like 5	---	694095	MmugDNA.12378.1.S1_at_EML5
MmugDNA.12379.1.S1_at	215822_x_at	MYT1	myelin transcription factor 1	chr20q13.33	4661	---	---	---	---	MmugDNA.12379.1.S1_at_MYT1
MmugDNA.12380.1.S1_at	217365_at	PRAMEF11	PRAME family member 11	chr1p36.21	440560	LOC715996	similar to PRAME family member 5	---	715996	MmugDNA.12380.1.S1_at_PRAMEF11
MmugDNA.12381.1.S1_s_at	232245_at	SLC25A34	solute carrier family 25, member 34	chr1p36.21	284723	SLC25A34	solute carrier family 25, member 34	---	695610	MmugDNA.12381.1.S1_s_at_SLC25A3
MmugDNA.1238.1.S1_at	1565608_at	---	---	---	---	---	---	---	---	MmugDNA.1238.1.S1_at_---
MmugDNA.12382.1.S1_at	218473_s_at	GLT25D1	glycosyltransferase 25 domain containing 1	chr19p13.11	79709	LOC719980	similar to glycosyltransferase 25 domain containing 1	---	719980	MmugDNA.12382.1.S1_at_GLT25D1
MmugDNA.12383.1.S1_at	219038_at	MORC4	MORC family CW-type zinc finger 4	chrXq22.3	79710	MORC4	MORC family CW-type zinc finger 4	---	701136	MmugDNA.12383.1.S1_at_MORC4
MmugDNA.12384.1.S1_at	228587_at	FAM83G	family with sequence similarity 83, member G	chr17p11.2	644815	---	---	---	---	MmugDNA.12384.1.S1_at_FAM83G
MmugDNA.12385.1.S1_at	218288_s_at	CCDC90B	coiled-coil domain containing 90B	chr11q14.1	60492	LOC702035	hypothetical protein LOC702035	---	702035	MmugDNA.12385.1.S1_at_CCDC90B
MmugDNA.12386.1.S1_at	218305_at	IPO4	importin 4	chr14q12	79711	IPO4	importin 4	---	715380	MmugDNA.12386.1.S1_at_IPO4
MmugDNA.12387.1.S1_at	219016_at	FASTKD5	FAST kinase domains 5	chr20p13	60493	LOC718041	Similar to karyopherin alpha 5 (importin alpha 6)	---	718041	MmugDNA.12387.1.S1_at_FASTKD5
MmugDNA.12388.1.S1_at	222502_s_at	UFM1	ubiquitin-fold modifier 1	chr13q13.3	51569	---	---	---	---	MmugDNA.12388.1.S1_at_UFM1
MmugDNA.12389.1.S1_at	220389_at	CCDC81	coiled-coil domain containing 81	chr11q14.2	60494	CCDC81	coiled-coil domain containing 81	---	704214	MmugDNA.12389.1.S1_at_CCDC81
MmugDNA.12390.1.S1_s_at	202522_at	PITPNB	phosphatidylinositol transfer protein, beta	chr22q12.1	23760	PITPNB	phosphatidylinositol transfer protein, beta	---	713414	MmugDNA.12390.1.S1_s_at_PITPNB
MmugDNA.12391.1.S1_at	220927_s_at	HPSE2 /// LOC1001338	heparanase 2 /// hypothetical protein LOC100133895	chr10q23-q24	100133895 /// 60495	LOC708795	similar to Heparanase-2 (Hpa2)	---	708795	MmugDNA.12391.1.S1_at_HPSE2 /// L
MmugDNA.1239.1.S1_at	234125_at	---	---	---	---	---	---	---	---	MmugDNA.1239.1.S1_at_---
MmugDNA.12392.1.S1_at	1557366_at	CCDC144A /// CCDC14	coiled-coil domain containing 144A /// coiled-coil domain containing 144B /// coiled-coil domain contai	chr17p11.2	100134159 /// 284047 /	CCDC144A	coiled-coil domain containing 144A	---	704543	MmugDNA.12392.1.S1_at_CCDC144A /
MmugDNA.12393.1.S1_at	218590_at	C10orf2	chromosome 10 open reading frame 2	chr10q23.3-q24.3	56652	LOC710958	similar to twinkle	---	710958	MmugDNA.12393.1.S1_at_C10orf2
MmugDNA.12394.1.S1_at	226392_at	---	---	---	---	LOC715074	similar to RAS p21 protein activator 2	---	715074	MmugDNA.12394.1.S1_at_---
MmugDNA.12395.1.S1_s_at	221987_s_at	TSR1	TSR1, 20S rRNA accumulation, homolog (S. cerevisiae)	chr17p13.3	55720	TSR1	TSR1, 20S rRNA accumulation, homolog (S. cerevisiae)	---	703311	MmugDNA.12395.1.S1_s_at_TSR1
MmugDNA.12396.1.S1_at	228721_at	C3orf41	chromosome 3 open reading frame 41	chr3p22.1	26172	LOC717189	similar to zinc finger protein 621	---	717189	MmugDNA.12396.1.S1_at_C3orf41
MmugDNA.12397.1.S1_at	228803_at	LOC100129592	Hypothetical protein LOC100129592	chr2q32.2	100129592	LOC709937	hypothetical protein LOC709937	---	709937	MmugDNA.12397.1.S1_at_LOC1001295
MmugDNA.12398.1.S1_at	225716_at	---	---	---	---	LOC723437	Similar to SH3 domain and tetratricopeptide repeats 1	---	723437	MmugDNA.12398.1.S1_at_---
MmugDNA.12399.1.S1_at	209476_at	TMX1	thioredoxin-related transmembrane protein 1	chr14q22.1	81542	TMX1	thioredoxin-related transmembrane protein 1	---	707057	MmugDNA.12399.1.S1_at_TMX1
MmugDNA.12400.1.S1_at	1553583_a_at	THRSP	thyroid hormone responsive (SPOT14 homolog, rat)	chr11q13.5	7069	THRSP	thyroid hormone responsive (SPOT14 homolog, rat)	---	699701	MmugDNA.12400.1.S1_at_THRSP
MmugDNA.12401.1.S1_at	1561107_at	---	---	---	---	---	---	---	---	MmugDNA.12401.1.S1_at_---
MmugDNA.1240.1.S1_at	234140_s_at	STIM2	stromal interaction molecule 2	chr4p15.2	57620	STIM2	stromal interaction molecule 2	---	694980	MmugDNA.1240.1.S1_at_STIM2
MmugDNA.12402.1.S1_at	243325_at	GSTK1	Glutathione S-transferase kappa 1	chr7q35	373156	---	---	---	---	MmugDNA.12402.1.S1_at_GSTK1
MmugDNA.12403.1.S1_at	213567_at	---	---	---	---	---	---	---	---	MmugDNA.12403.1.S1_at_---
MmugDNA.12404.1.S1_at	220851_at	---	---	---	---	---	---	---	---	MmugDNA.12404.1.S1_at_---
MmugDNA.12405.1.S1_at	NA	NA	NA	NA	NA	NA	NA	NA	NA	MmugDNA.12405.1.S1_at_NA
MmugDNA.12405.1.S1_s_at	203336_s_at	ITGB1BP1	integrin beta 1 binding protein 1	chr2p25.2	9270	ITGB1BP1	similar to Integrin beta-1-binding protein 1 (Integrin cytoplasmic domain-associated protein 1) (ICAP-1	---	699902	MmugDNA.12405.1.S1_s_at_ITGB1BP
MmugDNA.12406.1.S1_s_at	209881_s_at	LAT /// SPNS1	linker for activation of T cells /// spinster homolog 1 (Drosophila)	chr16p11.2	27040 /// 83985	LAT	linker for activation of T cells	---	704656	MmugDNA.12406.1.S1_s_at_LAT /// SP
MmugDNA.12407.1.S1_at	215290_at	---	---	---	---	---	---	---	---	MmugDNA.12407.1.S1_at_---

MmugDNA.12408.1.S1_at	211005_at	LAT /// SPNS1	linker for activation of T cells /// spinster homolog 1 (Drosophila)	chr16p11.2	27040 /// 83985	---	---	---	---	MmugDNA.12408.1.S1_at_LAT /// SPNS
MmugDNA.12409.1.S1_at	1566840_at	LOC283674	hypothetical protein LOC283674	chr15q22.32	283674	---	---	---	---	MmugDNA.12409.1.S1_at_LOC283674
MmugDNA.12410.1.S1_at	221138_s_at	---	---	---	---	---	---	---	---	MmugDNA.12410.1.S1_at_---
MmugDNA.12411.1.S1_at	240022_at	ZC3H4	Zinc finger CCCH-type containing 4	chr19q13.32	23211	---	---	---	---	MmugDNA.12411.1.S1_at_ZC3H4
MmugDNA.1241.1.S1_at	234166_at	UBE3A	Ubiquitin protein ligase E3A	chr15q11-q13	7337	---	---	---	---	MmugDNA.1241.1.S1_at_UBE3A
MmugDNA.12412.1.S1_s_at	219770_at	GTDC1	glycosyltransferase-like domain containing 1	chr2q22.2-q22.3	79712	LOC720926	Similar to Beta-1,3-N-acetylglucosaminyltransferase manic fringe (O-fucosylpeptide 3-beta-N-acetylgl	---	720926	MmugDNA.12412.1.S1_s_at_GTDC1
MmugDNA.12413.1.S1_at	222847_s_at	EGLN3	egl nine homolog 3 (C. elegans)	chr14q13.1	112399	---	---	---	---	MmugDNA.12413.1.S1_at_EGLN3
MmugDNA.12414.1.S1_at	238241_at	LOC100144602	hypothetical	---	100144602	---	---	---	---	MmugDNA.12414.1.S1_at_LOC100144
MmugDNA.12415.1.S1_at	219690_at	TMEM149	transmembrane protein 149	chr19q13.12	79713	LOC710396	similar to transmembrane protein 149	---	710396	MmugDNA.12415.1.S1_at_TMEM149
MmugDNA.12416.1.S1_at	218722_s_at	CCDC51	coiled-coil domain containing 51	chr3p21.31	79714	LOC710311	similar to ZC477.3a	---	710311	MmugDNA.12416.1.S1_at_CCDC51
MmugDNA.12417.1.S1_at	89476_r_at	NPEPL1	aminopeptidase-like 1	chr20q13.32	79716	---	---	---	---	MmugDNA.12417.1.S1_at_NPEPL1
MmugDNA.12418.1.S1_at	218480_at	AGBL5	ATP/GTP binding protein-like 5	chr2p23.3	60509	AGBL5	ATP/GTP binding protein-like 5	---	697940	MmugDNA.12418.1.S1_at_AGBL5
MmugDNA.12419.1.S1_at	229528_at	LOC283378	hypothetical protein LOC283378	chr12q24.31	283378	---	---	---	---	MmugDNA.12419.1.S1_at_LOC283378
MmugDNA.124.1.S1_at	228930_at	SCARNA15	Small Cajal body-specific RNA 15	chr15q25.2	677778	---	---	---	---	MmugDNA.124.1.S1_at_SCARNA15
MmugDNA.12420.1.S1_at	235552_at	METTL14	methyltransferase like 14	chr4q26	57721	METTL14	methyltransferase like 14	---	704667	MmugDNA.12420.1.S1_at_METTL14
MmugDNA.12421.1.S1_at	241707_at	LOC388630	UPF0632 protein A	chr1p33	388630	LOC712301	similar to C05G5.5	---	712301	MmugDNA.12421.1.S1_at_LOC388630
MmugDNA.1242.1.S1_at	1556128_a_at	RASGRF2	Ras protein-specific guanine nucleotide-releasing factor 2	chr5q13	5924	---	---	---	---	MmugDNA.1242.1.S1_at_RASGRF2
MmugDNA.12422.1.S1_at	NA	NA	NA	NA	NA	NA	NA	NA	NA	MmugDNA.12422.1.S1_at_NA
MmugDNA.12422.1.S1_s_at	218341_at	PPCS	phosphopantothenoylcysteine synthetase	chr1p34.2	79717	PPCS	phosphopantothenoylcysteine synthetase	---	697064	MmugDNA.12422.1.S1_s_at_PPCS
MmugDNA.12423.1.S1_at	1557666_s_at	C9orf98	chromosome 9 open reading frame 98	chr9q34.13	158067	---	---	---	---	MmugDNA.12423.1.S1_at_C9orf98
MmugDNA.12424.1.S1_at	210339_s_at	KLK2	kallikrein-related peptidase 2	chr19q13.41	3817	KLK2	similar to Kallikrein-2 precursor (Tissue kallikrein-2) (Glandular kallikrein-1) (hGK-1)	---	719436	MmugDNA.12424.1.S1_at_KLK2
MmugDNA.12425.1.S1_at	237978_at	---	---	---	---	---	---	---	---	MmugDNA.12425.1.S1_at_---
MmugDNA.12426.1.S1_at	224152_s_at	PBRM1	polybromo 1	chr3p21	55193	PB1	polybromo 1	---	696361	MmugDNA.12426.1.S1_at_PBRM1
MmugDNA.12427.1.S1_at	232800_at	---	---	---	---	---	---	---	---	MmugDNA.12427.1.S1_at_---
MmugDNA.12428.1.S1_at	238595_at	---	---	---	---	---	---	---	---	MmugDNA.12428.1.S1_at_---
MmugDNA.12429.1.S1_at	220852_at	PRO1768	PRO1768	chr14q32.11	29018	---	---	---	---	MmugDNA.12429.1.S1_at_PRO1768
MmugDNA.12430.1.S1_at	242618_at	HCG18	HLA complex group 18	chr6p21.3	414777	---	---	---	---	MmugDNA.12430.1.S1_at_HCG18
MmugDNA.12431.1.S1_at	213786_at	TAX1BP1	Tax1 (human T-cell leukemia virus type I) binding protein 1	chr7p15	8887	---	---	---	---	MmugDNA.12431.1.S1_at_TAX1BP1
MmugDNA.1243.1.S1_at	237950_s_at	---	---	---	---	---	---	---	---	MmugDNA.1243.1.S1_at_---
MmugDNA.12432.1.S1_at	221076_at	---	---	---	---	---	---	---	---	MmugDNA.12432.1.S1_at_---
MmugDNA.12433.1.S1_s_at	224279_s_at	CABYR	calcium binding tyrosine-(Y)-phosphorylation regulated	chr18q11.2	26256	---	---	---	---	MmugDNA.12433.1.S1_s_at_CABYR
MmugDNA.12434.1.S1_at	1557474_at	LOC284578	hypothetical LOC284578	chr1q32.1	284578	---	---	---	---	MmugDNA.12434.1.S1_at_LOC284578
MmugDNA.12435.1.S1_at	NA	NA	NA	NA	NA	NA	NA	NA	NA	MmugDNA.12435.1.S1_at_NA
MmugDNA.12436.1.S1_at	215184_at	DAPK2	death-associated protein kinase 2	chr15q22.31	23604	DAPK2	death-associated protein kinase 2	---	706421	MmugDNA.12436.1.S1_at_DAPK2
MmugDNA.12437.1.S1_at	230968_at	---	---	---	---	LOC706735	Similar to leucine zipper domain protein	---	706735	MmugDNA.12437.1.S1_at_---
MmugDNA.12438.1.S1_at	201633_s_at	CYB5B	cytochrome b5 type B (outer mitochondrial membrane)	chr16q22.1	80777	LOC705093	similar to Cytochrome b5 outer mitochondrial membrane isoform precursor	---	705093	MmugDNA.12438.1.S1_at_CYB5B
MmugDNA.12439.1.S1_at	202852_s_at	FLJ11506	alpha- and gamma-adaptin-binding protein p34	chr15q22.33-q23	79719	LOC711436	similar to Alpha- and gamma-adaptin-binding protein p34	---	711436	MmugDNA.12439.1.S1_at_FLJ11506
MmugDNA.12440.1.S1_s_at	235535_x_at	FRG1 /// FRG1B /// LO	FSHD region gene 1 /// FSHD region gene 1 family, member B /// hypothetical protein LOC10013409	chr20q11.1 /// chr4q35 /// chr9q1	100134091 /// 2483 ///	FRG1 /// LOC695817	FSHD region gene 1 /// similar to FRG1 protein (FSHD region gene 1 protein)	---	695817 /// 698743	MmugDNA.12440.1.S1_s_at_FRG1 /// F
MmugDNA.12441.1.S1_at	220112_at	ANKRD55	ankyrin repeat domain 55	chr5q11.2	79722	LOC709997	hypothetical protein LOC709997	---	709997	MmugDNA.12441.1.S1_at_ANKRD55
MmugDNA.1244.1.S1_at	218735_s_at	ZNF544	zinc finger protein 544	chr19q13.43	27300	LOC712453	similar to zinc finger protein 544	---	712453	MmugDNA.1244.1.S1_at_ZNF544
MmugDNA.12442.1.S1_at	238656_at	---	---	---	---	---	---	---	---	MmugDNA.12442.1.S1_at_---
MmugDNA.12443.1.S1_at	219262_at	SUV39H2	suppressor of variegation 3-9 homolog 2 (Drosophila)	chr10p13	79723	SUV39H2	suppressor of variegation 3-9 homolog 2	---	697378	MmugDNA.12443.1.S1_at_SUV39H2
MmugDNA.12444.1.S1_at	232223_at	---	---	---	---	---	---	---	---	MmugDNA.12444.1.S1_at_---
MmugDNA.12445.1.S1_at	1561672_at	---	---	---	---	---	---	---	---	MmugDNA.12445.1.S1_at_---
MmugDNA.12446.1.S1_at	209242_at	PEG3	paternally expressed 3	chr19q13.4	5178	---	---	---	---	MmugDNA.12446.1.S1_at_PEG3
MmugDNA.12447.1.S1_at	232533_at	METTL8	methyltransferase like 8	chr2q31.1	79828	---	---	---	---	MmugDNA.12447.1.S1_at_METTL8
MmugDNA.12448.1.S1_at	1569809_at	---	---	---	---	---	---	---	---	MmugDNA.12448.1.S1_at_---
MmugDNA.12449.1.S1_at	207743_at	---	---	---	---	---	---	---	---	MmugDNA.12449.1.S1_at_---
MmugDNA.12450.1.S1_s_at	224262_at	IL1F10	interleukin 1 family, member 10 (theta)	chr2q13	84639	---	---	---	---	MmugDNA.12450.1.S1_s_at_IL1F10
MmugDNA.12450.1.S1_x_at	NA	NA	NA	NA	NA	NA	NA	NA	NA	MmugDNA.12450.1.S1_x_at_NA
MmugDNA.12451.1.S1_at	216638_s_at	PRLR	prolactin receptor	chr5p13.2	5618	PRLR	prolactin receptor	---	699369	MmugDNA.12451.1.S1_at_PRLR
MmugDNA.1245.1.S1_at	226997_at	ADAMTS12	ADAM metallopeptidase with thrombospondin type 1 motif, 12	chr5q35	81792	ASB3	Ankyrin repeat and SOCS box-containing 3	---	716503	MmugDNA.1245.1.S1_at_ADAMTS12
MmugDNA.12452.1.S1_at	65588_at	LOC388796	hypothetical LOC388796	chr20q11.23	388796	LOC699186	hypothetical protein LOC699186	---	699186	MmugDNA.12452.1.S1_at_LOC388796
MmugDNA.12453.1.S1_at	220608_s_at	ZNF770	zinc finger protein 770	chr15q14	54989	---	---	---	---	MmugDNA.12453.1.S1_at_ZNF770
MmugDNA.12454.1.S1_at	NA	NA	NA	NA	NA	NA	NA	NA	NA	MmugDNA.12454.1.S1_at_NA
MmugDNA.12455.1.S1_at	207472_at	RARS2	arginyl-tRNA synthetase 2, mitochondrial	chr6q16.1	57038	RARS2	arginyl-tRNA synthetase 2, mitochondrial	---	699240	MmugDNA.12455.1.S1_at_RARS2
MmugDNA.12456.1.S1_at	218916_at	ZNF768	zinc finger protein 768	chr16p11.2	79724	LOC712020	similar to zinc finger protein 239 (predicted)	---	712020	MmugDNA.12456.1.S1_at_ZNF768
MmugDNA.12457.1.S1_at	232898_at	DAB2	disabled homolog 2, mitogen-responsive phosphoprotein (Drosophila)	chr5p13	1601	---	---	---	---	MmugDNA.12457.1.S1_at_DAB2
MmugDNA.12458.1.S1_at	1554377_a_at	CNTNAP4	contactin associated protein-like 4	chr16q23.1	85445	CNTNAP4	contactin associated protein-like 4	---	574170	MmugDNA.12458.1.S1_at_CNTNAP4
MmugDNA.12459.1.S1_at	220360_at	THAP9	THAP domain containing 9	chr4q21.22	79725	THAP9	THAP domain containing 9	---	693821	MmugDNA.12459.1.S1_at_THAP9
MmugDNA.12460.1.S1_s_at	214909_s_at	DDAH2	dimethylarginine dimethylaminohydrolase 2	chr6p21.3	23564	DDAH2	dimethylarginine dimethylaminohydrolase 2	---	716356	MmugDNA.12460.1.S1_s_at_DDAH2
MmugDNA.12461.1.S1_at	218505_at	WDR59	WD repeat domain 59	chr16q23.1	79726	LOC710576	similar to WD repeat domain 59	---	710576	MmugDNA.12461.1.S1_at_WDR59
MmugDNA.1246.1.S1_at	1565694_at	DTYMK /// LOC727761	deoxythymidylate kinase (thymidylate kinase) /// similar to Deoxythymidylate kinase (thymidylate kinas	chr2q37.3	1841 /// 727761	LOC701781	similar to deoxythymidylate kinase (thymidylate kinase)	---	701781	MmugDNA.1246.1.S1_at_DTYMK /// LO
MmugDNA.12462.1.S1_at	237589_at	---	---	---	---	---	---	---	---	MmugDNA.12462.1.S1_at_---
MmugDNA.12463.1.S1_at	231938_at	SGOL1	Shugoshin-like 1 (S. pombe)	chr3p24.3	151648	---	---	---	---	MmugDNA.12463.1.S1_at_SGOL1
MmugDNA.12464.1.S1_at	215923_s_at	PSD4	pleckstrin and Sec7 domain containing 4	chr2q13	23550	---	---	---	---	MmugDNA.12464.1.S1_at_PSD4
MmugDNA.12465.1.S1_at	219823_at	LIN28	lin-28 homolog (C. elegans)	chr1p36.11	79727	---	---	---	---	MmugDNA.12465.1.S1_at_LIN28
MmugDNA.12466.1.S1_at	211540_s_at	RB1	retinoblastoma 1	chr13q14.2	5925	RB1	retinoblastoma 1	---	704871	MmugDNA.12466.1.S1_at_RB1
MmugDNA.12467.1.S1_at	219530_at	PALB2	partner and localizer of BRCA2	chr16p12.1	79728	LOC700843	hypothetical protein LOC700843	---	700843	MmugDNA.12467.1.S1_at_PALB2
MmugDNA.12468.1.S1_s_at	208794_s_at	SMARCA4	SWI/SNF related, matrix associated, actin dependent regulator of chromatin, subfamily a, member 4	chr19p13.2	6597	---	---	---	---	MmugDNA.12468.1.S1_s_at_SMARCA
MmugDNA.12469.1.S1_at	239522_at	IL12RB1	interleukin 12 receptor, beta 1	chr19p13.1	3594	---	---	---	---	MmugDNA.12469.1.S1_at_IL12RB1
MmugDNA.12470.1.S1_at	225348_at	FUSIP1	FUS interacting protein (serine/arginine-rich) 1	chr1p36.11	10772	---	---	---	---	MmugDNA.12470.1.S1_at_FUSIP1
MmugDNA.12471.1.S1_at	1564610_at	---	---	---	---	---	---	---	---	MmugDNA.12471.1.S1_at_---
MmugDNA.1247.1.S1_s_at	NA	NA	NA	NA	NA	NA	NA	NA	NA	MmugDNA.1247.1.S1_s_at_NA
MmugDNA.12472.1.S1_at	1563842_at	PIGG	phosphatidylinositol glycan anchor biosynthesis, class G	chr4p16.3	54872	---	---	---	---	MmugDNA.12472.1.S1_at_PIGG
MmugDNA.12473.1.S1_at	221994_at	PDLIM5	PDZ and LIM domain 5	chr4q22	10611	LOC705358	similar to PDZ and LIM domain 5 isoform a	---	705358	MmugDNA.12473.1.S1_at_PDLIM5
MmugDNA.12474.1.S1_at	229574_at	TRA2A	transformer 2 alpha homolog (Drosophila)	chr7p15.3	29896	---	---	---	---	MmugDNA.12474.1.S1_at_TRA2A
MmugDNA.12475.1.S1_s_at	218782_s_at	ATAD2	ATPase family, AAA domain containing 2	chr8q24.13	29028	LOC704478	similar to two AAA domain containing protein	---	704478	MmugDNA.12475.1.S1_s_at_ATAD2
MmugDNA.12476.1.S1_at	1562943_at	---	---	---	---	---	---	---	---	MmugDNA.12476.1.S1_at_---
MmugDNA.12477.1.S1_at	220109_at	TF	transferrin	chr3q22.1	7018	---	---	---	---	MmugDNA.12477.1.S1_at_TF
MmugDNA.12478.1.S1_at	209210_s_at	FERMT2	fermitin family homolog 2 (Drosophila)	chr14q22.2	10979	FERMT2	fermitin family homolog 2 (Drosophila)	---	693958	MmugDNA.12478.1.S1_at_FERMT2
MmugDNA.12479.1.S1_at	220193_at	C1orf113	chromosome 1 open reading frame 113	chr1p34.3	79729	LOC713094	similar to CD2-associated protein	---	713094	MmugDNA.12479.1.S1_at_C1orf113
MmugDNA.12480.1.S1_at	218477_at	TMEM14A	transmembrane protein 14A	chr6p12.1	28978	LOC708829	similar to transmembrane protein 14A	---	708829	MmugDNA.12480.1.S1_at_TMEM14A
MmugDNA.12481.1.S1_at	220194_at	NSUN7	NOL1/NOP2/Sun domain family, member 7	chr4p14	79730	NSUN7	NOL1/NOP2/Sun domain family, member 7	---	701102	MmugDNA.12481.1.S1_at_NSUN7
MmugDNA.1248.1.S1_at	235074_at	SPRED1	sprouty-related, EVH1 domain containing 1	chr15q14	161742	---	---	---	---	MmugDNA.1248.1.S1_at_SPRED1
MmugDNA.12482.1.S1_at	241070_at	---	---	---	---	---	---	---	---	MmugDNA.12482.1.S1_at_---
MmugDNA.12483.1.S1_at	1559050_at	HCG27	HLA complex group 27	chr6p21.33	253018	---	---	---	---	MmugDNA.12483.1.S1_at_HCG27
MmugDNA.12484.1.S1_at	209219_at	RDBP	RD RNA binding protein	chr6p21.3	7936	RDBP	RD RNA binding protein	---	716913	MmugDNA.12484.1.S1_at_RDBP
MmugDNA.12485.1.S1_at	219217_at	NARS2	asparaginyl-tRNA synthetase 2, mitochondrial (putative)	chr11q14.1	79731	NARS2	asparaginyl-tRNA synthetase 2, mitochondrial (putative)	---	700326	MmugDNA.12485.1.S1_at_NARS2
MmugDNA.12486.1.S1_at	1558202_at	LOC145783	hypothetical LOC145783	chr15q21.3	145783	---	---	---	---	MmugDNA.12486.1.S1_at_LOC145783
MmugDNA.12487.1.S1_at	47560_at	LPHN1	latrophilin 1	chr19p13.2	22859	---	---	---	---	MmugDNA.12487.1.S1_at_LPHN1
MmugDNA.12488.1.S1_at	212687_at	LIMS1	LIM and senescent cell antigen-like domains 1	chr2q12.3-q13	3987	---	---	---	---	MmugDNA.12488.1.S1_at_LIMS1
MmugDNA.12489.1.S1_at	226760_at	MBTPS2	membrane-bound transcription factor peptidase, site 2	chrXp22.13	51360	MBTPS2	membrane-bound transcription factor peptidase, site 2	---	696540	MmugDNA.12489.1.S1_at_MBTPS2
MmugDNA.12490.1.S1_at	219990_at	E2F8	E2F transcription factor 8	chr11p15.1	79733	E2F8	E2F transcription factor 8	---	702368	MmugDNA.12490.1.S1_at_E2F8
MmugDNA.12491.1.S1_at	227754_at	---	---	---	---	---	---	---	---	MmugDNA.12491.1.S1_at_---
MmugDNA.1249.1.S1_at	239226_at	RTKN	Rhotekin	chr2p13.1	6242	---	---	---	---	MmugDNA.1249.1.S1_at_RTKN
MmugDNA.12492.1.S1_at	1555788_a_at	TRIB3	tribbles homolog 3 (Drosophila)	chr20p13-p12.2	57761	TRIB3	tribbles homolog 3 (Drosophila)	---	714636	MmugDNA.12492.1.S1_at_TRIB3
MmugDNA.12493.1.S1_at	229688_at	---	---	---	---	LOC715319	hypothetical protein LOC715319	---	715319	MmugDNA.12493.1.S1_at_---
MmugDNA.12494.1.S1_at	232221_x_at	RSBN1L	round spermatid basic protein 1-like	chr7q11.23	222194	RSBN1L	round spermatid basic protein 1-like	---	693814	MmugDNA.12494.1.S1_at_RSBN1L
MmugDNA.12495.1.S1_at	1563181_a_at	---	---	---	---	---	---	---	---	MmugDNA.12495.1.S1_at_---
MmugDNA.12496.1.S1_at	235270_at	ZNF397	zinc finger protein 397	chr18q12.2	84307	ZNF397	zinc finger protein 397	---	714878	MmugDNA.12496.1.S1_at_ZNF397
MmugDNA.12497.1.S1_at	1559997_x_at	SAMD14	Sterile alpha motif domain containing 14	chr17q21.33	201191	LOC704275	similar to protein phosphatase 1, regulatory subunit 9B	---	704275	MmugDNA.12497.1.S1_at_SAMD14
MmugDNA.12498.1.S1_at	230928_at	---	---	---	---	---	---	---	---	MmugDNA.12498.1.S1_at_---
MmugDNA.12500.1.S1_at	244432_at	---	---	---	---	---	---	---	---	MmugDNA.12500.1.S1_at_---
MmugDNA.12501.1.S1_at	218466_at	TBC1D17	TBC1 domain family, member 17	chr19q13.33	79735	LOC719093	similar to TBC1 domain family, member 17	---	719093	MmugDNA.12501.1.S1_at_TBC1D17
MmugDNA.1250.1.S1_at	211384_s_at	CASR	calcium-sensing receptor	chr3q13	846	LOC708986	Similar to ribosomal protein L27	---	708986	MmugDNA.1250.1.S1_at_CASR
MmugDNA.12502.1.S1_at	229094_at	LOC401431	hypothetical LOC401431	chr7q36.1	401431	LOC712385	hypothetical protein LOC712385	---	712385	MmugDNA.12502.1.S1_at_LOC401431
MmugDNA.12503.1.S1_at	243143_at	FAM24A	family with sequence similarity 24, member A	chr10q26.13	118670	LOC707274	similar to family with sequence similarity 24, member A	---	707274	MmugDNA.12503.1.S1_at_FAM24A

MmugDNA.12504.1.S1_at	219146_at	C17orf42	chromosome 17 open reading frame 42	---	79736	LOC713684	hypothetical protein LOC713684	---	713684	MmugDNA.12504.1.S1_at_C17orf42
MmugDNA.12505.1.S1_at	201460_at	MAPKAPK2	mitogen-activated protein kinase-activated protein kinase 2	chr1q32	9261	MAPKAPK2	mitogen-activated protein kinase-activated protein kinase 2	---	695044	MmugDNA.12505.1.S1_at_MAPKAPK2
MmugDNA.12506.1.S1_at	240807_at	---	---	---	---	---	---	---	---	MmugDNA.12506.1.S1_at_---
MmugDNA.12507.1.S1_at	219487_at	BBS10	Bardet-Biedl syndrome 10	chr12q21.2	79738	LOC693270	similar to chaperonin containing TCP1, subunit 3 (gamma)	---	693270	MmugDNA.12507.1.S1_at_BBS10
MmugDNA.12508.1.S1_at	229829_at	C18orf18	chromosome 18 open reading frame 18	chr18p11.31	147525	---	---	---	---	MmugDNA.12508.1.S1_at_C18orf18
MmugDNA.12509.1.S1_at	219882_at	TTLL7	tubulin tyrosine ligase-like family, member 7	chr1p31.1	79739	---	---	---	---	MmugDNA.12509.1.S1_at_TTLL7
MmugDNA.12510.1.S1_at	231723_at	SNX12	sorting nexin 12	chrXq13.1	29934	LOC696408	similar to sorting nexin 12	---	696408	MmugDNA.12510.1.S1_at_SNX12
MmugDNA.12511.1.S1_at	231442_at	ZPBP2	zona pellucida binding protein 2	chr17q12	124626	ZPBP2	zona pellucida binding protein 2	---	698217	MmugDNA.12511.1.S1_at_ZPBP2
MmugDNA.1251.1.S1_at	209317_at	POLR1C	polymerase (RNA) I polypeptide C, 30kDa	chr6p21.1	9533	POLR1C	polymerase (RNA) I polypeptide C, 30kDa	---	700727	MmugDNA.1251.1.S1_at_POLR1C
MmugDNA.12512.1.S1_at	220269_at	ZBBX	zinc finger, B-box domain containing	chr3q26.1	79740	LOC700091	hypothetical protein LOC700091	---	700091	MmugDNA.12512.1.S1_at_ZBBX
MmugDNA.12513.1.S1_at	241230_at	---	---	---	---	---	---	---	---	MmugDNA.12513.1.S1_at_---
MmugDNA.12514.1.S1_at	237295_at	---	---	---	---	---	---	---	---	MmugDNA.12514.1.S1_at_---
MmugDNA.12515.1.S1_at	204762_s_at	GNAO1	guanine nucleotide binding protein (G protein), alpha activating activity polypeptide O	chr16q13	2775	GNAO1	guanine nucleotide binding protein (G protein), alpha activating activity polypeptide O	---	574394	MmugDNA.12515.1.S1_at_GNAO1
MmugDNA.12517.1.S1_at	244387_at	---	---	---	---	---	---	---	---	MmugDNA.12517.1.S1_at_---
MmugDNA.12518.1.S1_at	243403_x_at	CPM	carboxypeptidase M	chr12q14.3	1368	---	---	---	---	MmugDNA.12518.1.S1_at_CPM
MmugDNA.12519.1.S1_at	238572_at	STK16	serine/threonine kinase 16	chr2q35	8576	STK16	serine/threonine kinase 16	---	702715	MmugDNA.12519.1.S1_at_STK16
MmugDNA.125.1.S1_at	220497_at	ZNF214	zinc finger protein 214	chr11p15.4	7761	ZNF214	zinc finger protein 214	---	708389	MmugDNA.125.1.S1_at_ZNF214
MmugDNA.12520.1.S1_at	227570_at	TMEM86A	transmembrane protein 86A	chr11p15.1	144110	TMEM86A	transmembrane protein 86A	---	703759	MmugDNA.12520.1.S1_at_TMEM86A
MmugDNA.12521.1.S1_at	202254_at	SIPA1L1	Signal-induced proliferation-associated 1 like 1	chr14q24.2	26037	SIPA1L1	signal-induced proliferation-associated 1 like 1	---	694485	MmugDNA.12521.1.S1_at_SIPA1L1
MmugDNA.1252.1.S1_at	238692_at	BTBD11	BTB (POZ) domain containing 11	chr12q23.3	121551	BTBD11	BTB (POZ) domain containing 11	---	704916	MmugDNA.1252.1.S1_at_BTBD11
MmugDNA.1252.1.S1_s_at	NA	NA	NA	NA	NA	NA	NA	NA	NA	MmugDNA.1252.1.S1_s_at_NA
MmugDNA.12522.1.S1_at	1556284_at	PPA2	Pyrophosphatase (inorganic) 2	chr4q25	27068	PPA2	pyrophosphatase (inorganic) 2	---	694267	MmugDNA.12522.1.S1_at_PPA2
MmugDNA.12523.1.S1_at	235422_at	---	---	---	---	---	---	---	---	MmugDNA.12523.1.S1_at_---
MmugDNA.12524.1.S1_at	228193_s_at	C13orf15	Chromosome 13 open reading frame 15	chr13q14.11	28984	C17H13orf15	chromosome 13 open reading frame 15 ortholog	---	698962	MmugDNA.12524.1.S1_at_C13orf15
MmugDNA.12525.1.S1_at	235279_at	---	---	---	---	---	---	---	---	MmugDNA.12525.1.S1_at_---
MmugDNA.12526.1.S1_at	230392_at	---	---	---	---	---	---	---	---	MmugDNA.12526.1.S1_at_---
MmugDNA.12527.1.S1_at	1559205_s_at	---	---	---	---	---	---	---	---	MmugDNA.12527.1.S1_at_---
MmugDNA.12528.1.S1_at	207801_s_at	RNF10	ring finger protein 10	chr12q24.31	9921	---	---	---	---	MmugDNA.12528.1.S1_at_RNF10
MmugDNA.12529.1.S1_at	220749_at	C10orf68	chromosome 10 open reading frame 68	chr10p11.22	79741	C9H10orf68	chromosome 10 open reading frame 68 ortholog	---	703164	MmugDNA.12529.1.S1_at_C10orf68
MmugDNA.12530.1.S1_at	202375_at	SEC24D	SEC24 family, member D (S. cerevisiae)	chr4q26	9871	LOC707521	hypothetical protein LOC707521	---	707521	MmugDNA.12530.1.S1_at_SEC24D
MmugDNA.12531.1.S1_at	1557463_at	---	---	---	---	---	---	---	---	MmugDNA.12531.1.S1_at_---
MmugDNA.1253.1.S1_at	1554887_at	---	---	---	---	---	---	---	---	MmugDNA.1253.1.S1_at_---
MmugDNA.12532.1.S1_at	239972_at	---	---	---	---	---	---	---	---	MmugDNA.12532.1.S1_at_---
MmugDNA.12533.1.S1_at	219652_s_at	CXorf36	chromosome X open reading frame 36	chrXp11.3	79742	---	---	---	---	MmugDNA.12533.1.S1_at_CXorf36
MmugDNA.12534.1.S1_at	220196_at	MUC16	mucin 16, cell surface associated	chr19p13.2	94025	LOC712735	hypothetical protein LOC712735	---	712735	MmugDNA.12534.1.S1_at_MUC16
MmugDNA.12535.1.S1_at	58367_s_at	ZNF419	zinc finger protein 419	chr19q13.43	79744	LOC710445	similar to zinc finger protein 749	---	710445	MmugDNA.12535.1.S1_at_ZNF419
MmugDNA.12536.1.S1_at	244351_at	RBM46	RNA binding motif protein 46	chr4q32.1	166863	RBM46	RNA binding motif protein 46	---	698990	MmugDNA.12536.1.S1_at_RBM46
MmugDNA.12537.1.S1_at	230685_at	FLJ33630	hypothetical LOC644873	chr5q23.3	644873	---	---	---	---	MmugDNA.12537.1.S1_at_FLJ33630
MmugDNA.12538.1.S1_at	239048_at	---	---	---	---	---	---	---	---	MmugDNA.12538.1.S1_at_---
MmugDNA.12539.1.S1_s_at	228258_at	TBC1D10C	TBC1 domain family, member 10C	chr11q13.1	374403	LOC712270	similar to TBC1 domain family, member 10C	---	712270	MmugDNA.12539.1.S1_s_at_TBC1D10
MmugDNA.12540.1.S1_at	213111_at	PIKFYVE	phosphoinositide kinase, FYVE finger containing	chr2q33.3	200576	---	---	---	---	MmugDNA.12540.1.S1_at_PIKFYVE
MmugDNA.12541.1.S1_at	1553388_at	FAM26D	family with sequence similarity 26, member D	chr6q22.1	221301	---	---	---	---	MmugDNA.12541.1.S1_at_FAM26D
MmugDNA.1254.1.S1_at	1568680_s_at	YTHDC2	YTH domain containing 2	chr5q22.2	64848	LOC694612	similar to YTH domain containing 2	---	694612	MmugDNA.1254.1.S1_at_YTHDC2
MmugDNA.12542.1.S1_at	222860_s_at	PDGFD	platelet derived growth factor D	chr11q22.3	80310	---	---	---	---	MmugDNA.12542.1.S1_at_PDGFD
MmugDNA.12543.1.S1_at	225325_at	MFSD6	major facilitator superfamily domain containing 6	chr2q32.2	54842	LOC713392	Similar to Keratin, type I cytoskeletal 18 (Cytokeratin-18) (CK-18) (Keratin-18) (K18)	---	713392	MmugDNA.12543.1.S1_at_MFSD6
MmugDNA.12544.1.S1_at	201661_s_at	ACSL3	acyl-CoA synthetase long-chain family member 3	chr2q34-q35	2181	LOC703458	Similar to Histidine acid phosphatase domain containing 1	---	703458	MmugDNA.12544.1.S1_at_ACSL3
MmugDNA.12545.1.S1_at	242573_at	---	---	---	---	---	---	---	---	MmugDNA.12545.1.S1_at_---
MmugDNA.12546.1.S1_at	202422_s_at	ACSL4	acyl-CoA synthetase long-chain family member 4	chrXq22.3-q23	2182	LOC695987	Similar to ribosomal protein L19	---	695987	MmugDNA.12546.1.S1_at_ACSL4
MmugDNA.12547.1.S1_at	NA	NA	NA	NA	NA	NA	NA	NA	NA	MmugDNA.12547.1.S1_at_NA
MmugDNA.12548.1.S1_at	225268_at	KPNA4	karyopherin alpha 4 (importin alpha 3)	chr3q25.33	3840	KPNA4	karyopherin alpha 4	---	702486	MmugDNA.12548.1.S1_at_KPNA4
MmugDNA.12549.1.S1_at	209579_s_at	MBD4	methyl-CpG binding domain protein 4	chr3q21-q22	8930	LOC720557	similar to methyl-CpG binding domain protein 4	---	720557	MmugDNA.12549.1.S1_at_MBD4
MmugDNA.12550.1.S1_at	220362_at	PSORS1C1	psoriasis susceptibility 1 candidate 1	chr6p21.3	170679	LOC713967	similar to DNA-directed RNA polymerase II 7.6 kDa polypeptide (RPB10) (RPB7.6) (RPABC5)	---	713967	MmugDNA.12550.1.S1_at_PSORS1C1
MmugDNA.12551.1.S1_at	NA	NA	NA	NA	NA	NA	NA	NA	NA	MmugDNA.12551.1.S1_at_NA
MmugDNA.12551.1.S1_s_at	228170_at	OLIG1	oligodendrocyte transcription factor 1	chr21q22.11	116448	---	---	---	---	MmugDNA.12551.1.S1_s_at_OLIG1
MmugDNA.1255.1.S1_at	215760_s_at	SBNO2	strawberry notch homolog 2 (Drosophila)	chr19p13.3	22904	LOC716193	similar to KIAA0963	---	716193	MmugDNA.1255.1.S1_at_SBNO2
MmugDNA.12552.1.S1_at	220560_at	C11orf21	chromosome 11 open reading frame 21	chr11p15.5	29125	---	---	---	---	MmugDNA.12552.1.S1_at_C11orf21
MmugDNA.12553.1.S1_at	NA	NA	NA	NA	NA	NA	NA	NA	NA	MmugDNA.12553.1.S1_at_NA
MmugDNA.12553.1.S1_s_at	1557786_s_at	CHIC1	cysteine-rich hydrophobic domain 1	chrXq13-q21	53344	CHIC1	cysteine-rich hydrophobic domain 1	---	701720	MmugDNA.12553.1.S1_s_at_CHIC1
MmugDNA.12554.1.S1_at	1562482_at	---	---	---	---	---	---	---	---	MmugDNA.12554.1.S1_at_---
MmugDNA.12555.1.S1_at	220635_at	PSORS1C2	psoriasis susceptibility 1 candidate 2	chr6p21.3	170680	---	---	---	---	MmugDNA.12555.1.S1_at_PSORS1C2
MmugDNA.12556.1.S1_at	216408_at	OR2B2	olfactory receptor, family 2, subfamily B, member 2	chr6p22.3-p21.3	81697	LOC705704	similar to olfactory receptor, family 2, subfamily B, member 2	---	705704	MmugDNA.12556.1.S1_at_OR2B2
MmugDNA.12557.1.S1_at	221100_at	C6orf15	chromosome 6 open reading frame 15	chr6p21.3	29113	C4H6orf15	chromosome 6 open reading frame 15 ortholog	---	100141389	MmugDNA.12557.1.S1_at_C6orf15
MmugDNA.12558.1.S1_at	219944_at	CLIP4	CAP-GLY domain containing linker protein family, member 4	chr2p23.2	79745	CLIP4	CAP-GLY domain containing linker protein family, member 4	---	704092	MmugDNA.12558.1.S1_at_CLIP4
MmugDNA.12559.1.S1_at	203776_at	GPKOW	G patch domain and KOW motifs	chrXp11.23	27238	LOC715449	similar to G patch domain and KOW motifs	---	715449	MmugDNA.12559.1.S1_at_GPKOW
MmugDNA.12560.1.S1_at	219298_at	ECHDC3	enoyl Coenzyme A hydratase domain containing 3	chr10p14	79746	ECHDC3	enoyl Coenzyme A hydratase domain containing 3	---	693583	MmugDNA.12560.1.S1_at_ECHDC3
MmugDNA.12561.1.S1_at	230912_at	ASPDH	aspartate dehydrogenase domain containing	chr19q13.33	554235	LOC719326	similar to RIKEN cDNA 0610012D14	---	719326	MmugDNA.12561.1.S1_at_ASPDH
MmugDNA.1256.1.S1_at	1570024_at	---	---	---	---	---	---	---	---	MmugDNA.1256.1.S1_at_---
MmugDNA.12562.1.S1_at	1554564_a_at	UNQ1887	signal peptide peptidase 3	chr12q24.31	121665	---	---	---	---	MmugDNA.12562.1.S1_at_UNQ1887
MmugDNA.12563.1.S1_at	220614_s_at	C6orf103	chromosome 6 open reading frame 103	chr6q24.3	79747	LOC698358	hypothetical protein LOC698358	---	698358	MmugDNA.12563.1.S1_at_C6orf103
MmugDNA.12564.1.S1_at	226288_s_at	NLGN2	neuroligin 2	chr17p13.1	57555	---	---	---	---	MmugDNA.12564.1.S1_at_NLGN2
MmugDNA.12565.1.S1_at	242361_at	IMMT	Inner membrane protein, mitochondrial (mitofilin)	chr2p11.2|2	10989	---	---	---	---	MmugDNA.12565.1.S1_at_IMMT
MmugDNA.12566.1.S1_at	219775_s_at	CPLX3 /// LMAN1L	complexin 3 /// lectin, mannose-binding, 1 like	chr15q24.1	594855 /// 79748	---	---	---	---	MmugDNA.12566.1.S1_at_CPLX3 /// LM
MmugDNA.12567.1.S1_at	225413_at	USMG5	up-regulated during skeletal muscle growth 5 homolog (mouse)	chr10q24.33	84833	---	---	---	---	MmugDNA.12567.1.S1_at_USMG5
MmugDNA.12568.1.S1_at	219218_at	BAHCC1	BAH domain and coiled-coil containing 1	chr17q25.3	57597	---	---	---	---	MmugDNA.12568.1.S1_at_BAHCC1
MmugDNA.12569.1.S1_at	NA	NA	NA	NA	NA	NA	NA	NA	NA	MmugDNA.12569.1.S1_at_NA
MmugDNA.12570.1.S1_at	204947_at	E2F1	E2F transcription factor 1	chr20q11.2	1869	LOC714126	similar to E2F transcription factor 1	---	714126	MmugDNA.12570.1.S1_at_E2F1
MmugDNA.12571.1.S1_at	227859_at	DNAJC27	DnaJ (Hsp40) homolog, subfamily C, member 27	chr2p23.3	51277	DNAJC27	DnaJ (Hsp40) homolog, subfamily C, member 27	---	694037	MmugDNA.12571.1.S1_at_DNAJC27
MmugDNA.1257.1.S1_at	222500_at	PPIL1	peptidylprolyl isomerase (cyclophilin)-like 1	chr6p21.1	51645	PPIL1	peptidylprolyl isomerase (cyclophilin)-like 1	---	707647	MmugDNA.1257.1.S1_at_PPIL1
MmugDNA.12572.1.S1_at	233812_at	NCRNA00028	non-protein coding RNA 28	---	140875	---	---	---	---	MmugDNA.12572.1.S1_at_NCRNA0002
MmugDNA.12573.1.S1_at	203693_s_at	E2F3	E2F transcription factor 3	chr6p22	1871	LOC713904	similar to Transcription factor E2F3 (E2F-3)	---	713904	MmugDNA.12573.1.S1_at_E2F3
MmugDNA.12574.1.S1_s_at	214164_x_at	CA12	carbonic anhydrase XII	chr15q22	771	CA12	carbonic anhydrase XII	---	705838	MmugDNA.12574.1.S1_s_at_CA12
MmugDNA.12575.1.S1_at	215493_x_at	BTN2A1	butyrophilin, subfamily 2, member A1	chr6p22.1	11120	LOC700495	similar to butyrophilin, subfamily 2, member A1 isoform 1 precursor	---	700495	MmugDNA.12575.1.S1_at_BTN2A1
MmugDNA.12576.1.S1_at	240864_at	---	---	---	---	---	---	---	---	MmugDNA.12576.1.S1_at_---
MmugDNA.12577.1.S1_at	203957_at	E2F6	E2F transcription factor 6	chr2p25.1	1876	LOC574147	UDP-Glucuronosyltransferase UGT2B33	---	574147	MmugDNA.12577.1.S1_at_E2F6
MmugDNA.12578.1.S1_at	1566294_at	---	---	---	---	---	---	---	---	MmugDNA.12578.1.S1_at_---
MmugDNA.12579.1.S1_at	223445_at	DTNBP1	dystrobrevin binding protein 1	chr6p22.3	84062	LOC709358	similar to dystrobrevin binding protein 1 isoform a	---	709358	MmugDNA.12579.1.S1_at_DTNBP1
MmugDNA.12580.1.S1_at	239946_at	---	---	---	---	---	---	---	---	MmugDNA.12580.1.S1_at_---
MmugDNA.12581.1.S1_at	224062_x_at	KLK4	kallikrein-related peptidase 4	chr19q13.41	9622	LOC719422	similar to kallikrein 4	---	719422	MmugDNA.12581.1.S1_at_KLK4
MmugDNA.12581.1.S1_s_at	1555737_a_at	KLK4	kallikrein-related peptidase 4	chr19q13.41	9622	LOC719422	similar to kallikrein 4	---	719422	MmugDNA.12581.1.S1_s_at_KLK4
MmugDNA.1258.1.S1_at	214593_at	PIAS2	protein inhibitor of activated STAT, 2	chr18q21.1	9063	PIAS2	protein inhibitor of activated STAT, 2	---	696737	MmugDNA.1258.1.S1_at_PIAS2
MmugDNA.12582.1.S1_at	230595_at	LOC572558	hypothetical locus LOC572558	chr9q13	572558	---	---	---	---	MmugDNA.12582.1.S1_at_LOC572558
MmugDNA.12583.1.S1_at	226399_at	---	---	---	---	---	---	---	---	MmugDNA.12583.1.S1_at_---
MmugDNA.12584.1.S1_at	224030_s_at	LOC100134743	similar to PRO2751	---	100134743	LOC701181	Similar to DNA-directed RNA polymerase II 7.6 kDa polypeptide (RPB10) (RPB7.6) (RPABC5)	---	701181	MmugDNA.12584.1.S1_at_LOC1001347
MmugDNA.12585.1.S1_at	1563023_at	---	---	---	---	---	---	---	---	MmugDNA.12585.1.S1_at_---
MmugDNA.12586.1.S1_at	212979_s_at	FAM115A	family with sequence similarity 115, member A	chr7q35	9747	---	---	---	---	MmugDNA.12586.1.S1_at_FAM115A
MmugDNA.12587.1.S1_at	1552608_at	WFDC11	WAP four-disulfide core domain 11	chr20q13.12	259239	LOC709928	similar to WAP four-disulfide core domain 11 precursor	---	709928	MmugDNA.12587.1.S1_at_WFDC11
MmugDNA.12588.1.S1_at	239888_at	---	---	---	---	---	---	---	---	MmugDNA.12588.1.S1_at_---
MmugDNA.12589.1.S1_at	215889_at	SKIL	SKI-like oncogene	chr3q26	6498	---	---	---	---	MmugDNA.12589.1.S1_at_SKIL
MmugDNA.12590.1.S1_at	1569600_at	DLEU2	Deleted in lymphocytic leukemia 2 (non-protein coding)	chr13q14.3	8847	---	---	---	---	MmugDNA.12590.1.S1_at_DLEU2
MmugDNA.12591.1.S1_s_at	224834_at	UBTD2	ubiquitin domain containing 2	chr5q35.1	92181	LOC709455	similar to dendritic cell-derived ubiquitin-like protein	---	709455	MmugDNA.12591.1.S1_s_at_UBTD2
MmugDNA.1259.1.S1_at	209669_s_at	SERBP1	SERPINE1 mRNA binding protein 1	chr1p31	26135	SERBP1	SERPINE1 mRNA binding protein 1	---	700797	MmugDNA.1259.1.S1_at_SERBP1
MmugDNA.1259.1.S1_s_at	NA	NA	NA	NA	NA	NA	NA	NA	NA	MmugDNA.1259.1.S1_s_at_NA
MmugDNA.12592.1.S1_at	224827_at	UBTD2	ubiquitin domain containing 2	chr5q35.1	92181	LOC709455	similar to dendritic cell-derived ubiquitin-like protein	---	709455	MmugDNA.12592.1.S1_at_UBTD2
MmugDNA.12593.1.S1_at	201519_at	TOMM70A	translocase of outer mitochondrial membrane 70 homolog A (S. cerevisiae)	chr3q12.2	9868	---	---	---	---	MmugDNA.12593.1.S1_at_TOMM70A
MmugDNA.12594.1.S1_at	237407_at	HS1BP3	HCLS1 binding protein 3	chr2p24.1	64342	---	---	---	---	MmugDNA.12594.1.S1_at_HS1BP3
MmugDNA.12595.1.S1_at	201922_at	TINP1	TGF beta-inducible nuclear protein 1	chr5q13.3	10412	TINP1	similar to TGF-beta-inducible nuclear protein 1 (Hairy cell leukemia protein 1)	---	697182	MmugDNA.12595.1.S1_at_TINP1
MmugDNA.12596.1.S1_at	220117_at	ZNF385D	zinc finger protein 385D	chr3p24.3	79750	---	---	---	---	MmugDNA.12596.1.S1_at_ZNF385D
MmugDNA.12597.1.S1_at	1569712_at	LOC729725	hypothetical LOC729725	chr4q32.2	729725	---	---	---	---	MmugDNA.12597.1.S1_at_LOC729725

MmugDNA.12598.1.S1_at	218725_at	SLC25A22	solute carrier family 25 (mitochondrial carrier: glutamate), member 22	chr11p15.5	79751	SLC25A22	solute carrier family 25 (mitochondrial carrier: glutamate), member 22	---	700463	MmugDNA.12598.1.S1_at_SLC25A22
MmugDNA.12599.1.S1_at	214431_at	GMPS	guanine monphosphate synthetase	chr3q24	8833	GMPS	guanine monphosphate synthetase	---	706932	MmugDNA.12599.1.S1_at_GMPS
MmugDNA.12600.1.S1_at	243018_at	---	---	---	---	---	---	---	---	MmugDNA.12600.1.S1_at_---
MmugDNA.12601.1.S1_at	218919_at	ZFAND1	zinc finger, AN1-type domain 1	chr8q21.13	79752	ZFAND1	zinc finger, AN1-type domain 1	---	702195	MmugDNA.12601.1.S1_at_ZFAND1
MmugDNA.1260.1.S1_at	226394_at	MARCH5	membrane-associated ring finger (C3HC4) 5	chr10q23.32	54708	MARCH5	membrane-associated ring finger (C3HC4) 5	---	698517	MmugDNA.1260.1.S1_at_MARCH5
MmugDNA.12602.1.S1_at	219409_at	SNIP1	Smad nuclear interacting protein 1	chr1p34.3	79753	LOC713733	similar to Smad nuclear interacting protein	---	713733	MmugDNA.12602.1.S1_at_SNIP1
MmugDNA.12603.1.S1_at	243877_at	---	---	---	---	---	---	---	---	MmugDNA.12603.1.S1_at_---
MmugDNA.12604.1.S1_at	218862_at	ASB13	ankyrin repeat and SOCS box-containing 13	chr10p15.1	79754	LOC710765	Similar to synaptonemal complex protein 3	---	710765	MmugDNA.12604.1.S1_at_ASB13
MmugDNA.12605.1.S1_at	211815_s_at	GGA3	golgi associated, gamma adaptin ear containing, ARF binding protein 3	chr17q25.1	23163	LOC703459	Similar to CG13624-PC, isoform C	---	703459	MmugDNA.12605.1.S1_at_GGA3
MmugDNA.12606.1.S1_at	1556898_at	LOC200830	hypothetical protein LOC200830	chr3q26.1	200830	---	---	---	---	MmugDNA.12606.1.S1_at_LOC200830
MmugDNA.12607.1.S1_at	221850_x_at	AGAP11 /// AGAP4 /// A	ankyrin repeat and GTPase domain Arf GTPase activating protein 11 /// ArfGAP with GTPase domai	chr10q11.21 /// chr10q11.23 /// c	119016 /// 119385 /// 4	AGAP1	ArfGAP with GTPase domain, ankyrin repeat and PH domain 1	---	693652	MmugDNA.12607.1.S1_at_AGAP11 /// A
MmugDNA.12607.1.S1_x_at	NA	NA	NA	NA	NA	NA	NA	NA	NA	MmugDNA.12607.1.S1_x_at_NA
MmugDNA.12608.1.S1_at	1559648_at	RP3-527F8.2	Hypothetical protein LOC100128420	chrXq26.3	100128420	---	---	---	---	MmugDNA.12608.1.S1_at_RP3-527F8.
MmugDNA.12609.1.S1_at	209264_s_at	TSPAN4	tetraspanin 4	chr11p15.5	7106	---	---	---	---	MmugDNA.12609.1.S1_at_TSPAN4
MmugDNA.12610.1.S1_at	209583_s_at	CD200	CD200 molecule	chr3q12-q13	4345	CD200	CD200 molecule	---	708110	MmugDNA.12610.1.S1_at_CD200
MmugDNA.12611.1.S1_at	204022_at	WWP2	WW domain containing E3 ubiquitin protein ligase 2	chr16q22.1	11060	WWP2	WW domain containing E3 ubiquitin protein ligase 2	---	705464	MmugDNA.12611.1.S1_at_WWP2
MmugDNA.1261.1.S1_at	203849_s_at	KIF1A	kinesin family member 1A	chr2q37.3	547	LOC716889	similar to axonal transport of synaptic vesicles	---	716889	MmugDNA.1261.1.S1_at_KIF1A
MmugDNA.12612.1.S1_s_at	224036_s_at	LMBR1	limb region 1 homolog (mouse)	chr7q36	64327	LOC695544	Similar to ribosomal protein L3 isoform a	---	695544	MmugDNA.12612.1.S1_s_at_LMBR1
MmugDNA.12613.1.S1_at	222047_s_at	SRRT	serrate RNA effector molecule homolog (Arabidopsis)	chr7q21	51593	ARS2	arsenate resistance protein ARS2	---	713235	MmugDNA.12613.1.S1_at_SRRT
MmugDNA.12614.1.S1_at	231387_at	C6orf145	Chromosome 6 open reading frame 145	chr6p25.2	221749	LOC716603	hypothetical protein LOC716603	---	716603	MmugDNA.12614.1.S1_at_C6orf145
MmugDNA.12615.1.S1_at	1558846_at	PNLIPRP3	pancreatic lipase-related protein 3	chr10q25.3	119548	LOC699762	similar to Pancreatic lipase-related protein 1 precursor	---	699762	MmugDNA.12615.1.S1_at_PNLIPRP3
MmugDNA.12616.1.S1_at	202065_s_at	PPFIA1	protein tyrosine phosphatase, receptor type, f polypeptide (PTPRF), interacting protein (liprin), alpha	chr11q13.3	8500	---	---	---	---	MmugDNA.12616.1.S1_at_PPFIA1
MmugDNA.12617.1.S1_at	202066_at	PPFIA1	protein tyrosine phosphatase, receptor type, f polypeptide (PTPRF), interacting protein (liprin), alpha	chr11q13.3	8500	LOC718671	Hypothetical protein LOC718671	---	718671	MmugDNA.12617.1.S1_at_PPFIA1
MmugDNA.12618.1.S1_at	1559288_at	---	---	---	---	---	---	---	---	MmugDNA.12618.1.S1_at_---
MmugDNA.12619.1.S1_at	1565581_at	---	---	---	---	---	---	---	---	MmugDNA.12619.1.S1_at_---
MmugDNA.126.1.S1_at	220214_at	ZNF215	zinc finger protein 215	chr11p15.4	7762	ZNF215	zinc finger protein 215	---	708494	MmugDNA.126.1.S1_at_ZNF215
MmugDNA.12620.1.S1_at	219995_s_at	ZNF750	zinc finger protein 750	chr17q25.3	79755	LOC716544	hypothetical protein LOC716544	---	716544	MmugDNA.12620.1.S1_at_ZNF750
MmugDNA.12621.1.S1_at	1564654_at	---	---	---	---	---	---	---	---	MmugDNA.12621.1.S1_at_---
MmugDNA.1262.1.S1_at	1553006_at	GPR112	G protein-coupled receptor 112	chrXq26.3	139378	LOC693375	similar to G-protein coupled receptor 112	---	693375	MmugDNA.1262.1.S1_at_GPR112
MmugDNA.12622.1.S1_at	219695_at	SMPD3	sphingomyelin phosphodiesterase 3, neutral membrane (neutral sphingomyelinase II)	chr16q22.1	55512	---	---	---	---	MmugDNA.12622.1.S1_at_SMPD3
MmugDNA.12623.1.S1_at	221820_s_at	MYST1	MYST histone acetyltransferase 1	chr16p11.2	84148	MYST1	MYST histone acetyltransferase 1	---	713117	MmugDNA.12623.1.S1_at_MYST1
MmugDNA.12624.1.S1_at	219570_at	KIF16B	kinesin family member 16B	chr20p11.23	55614	LOC711996	Similar to F-box and WD-40 domain protein 4	---	711996	MmugDNA.12624.1.S1_at_KIF16B
MmugDNA.12625.1.S1_at	228109_at	RASGRF2	Ras protein-specific guanine nucleotide-releasing factor 2	chr5q13	5924	LOC711350	similar to Ras protein-specific guanine nucleotide-releasing factor 2	---	711350	MmugDNA.12625.1.S1_at_RASGRF2
MmugDNA.12626.1.S1_s_at	NA	NA	NA	NA	NA	NA	NA	NA	NA	MmugDNA.12626.1.S1_s_at_NA
MmugDNA.12627.1.S1_at	206690_at	ACCN1	amiloride-sensitive cation channel 1, neuronal	chr17q12	40	LOC718204	similar to amiloride-sensitive cation channel 1, neuronal (degenerin) isoform a	---	718204	MmugDNA.12627.1.S1_at_ACCN1
MmugDNA.12628.1.S1_at	1561414_at	LOC401497	similar to PRO2738	chr9p21.1	401497	---	---	---	---	MmugDNA.12628.1.S1_at_LOC401497
MmugDNA.12629.1.S1_at	204801_s_at	DHRS12	dehydrogenase/reductase (SDR family) member 12	chr13q14.3	79758	---	---	---	---	MmugDNA.12629.1.S1_at_DHRS12
MmugDNA.12630.1.S1_at	1561639_at	---	---	---	---	---	---	---	---	MmugDNA.12630.1.S1_at_---
MmugDNA.12631.1.S1_at	241267_at	EHD3	EH-domain containing 3	chr2p21	30845	---	---	---	---	MmugDNA.12631.1.S1_at_EHD3
MmugDNA.1263.1.S1_at	209414_at	FZR1	fizzy/cell division cycle 20 related 1 (Drosophila)	chr19p13.3	51343	LOC721711	similar to Fzr1 protein	---	721711	MmugDNA.1263.1.S1_at_FZR1
MmugDNA.12632.1.S1_at	234796_at	CABP7	calcium binding protein 7	chr22q12.2	164633	---	---	---	---	MmugDNA.12632.1.S1_at_CABP7
MmugDNA.12633.1.S1_at	219047_s_at	ZNF668	zinc finger protein 668	chr16p11.2	79759	LOC712709	similar to zinc finger protein 668	---	712709	MmugDNA.12633.1.S1_at_ZNF668
MmugDNA.12634.1.S1_at	207561_s_at	ACCN3	amiloride-sensitive cation channel 3	chr7q35	9311	LOC714396	similar to amiloride-sensitive cation channel 3 isoform c	---	714396	MmugDNA.12634.1.S1_at_ACCN3
MmugDNA.12635.1.S1_at	1564598_a_at	VWA3B	von Willebrand factor A domain containing 3B	chr2q11.2	200403	LOC707760	hypothetical protein LOC707760	---	707760	MmugDNA.12635.1.S1_at_VWA3B
MmugDNA.12636.1.S1_at	231818_x_at	---	---	---	---	---	---	---	---	MmugDNA.12636.1.S1_at_---
MmugDNA.12637.1.S1_at	233472_at	TCP11L1	t-complex 11 (mouse)-like 1	chr11p13	55346	---	---	---	---	MmugDNA.12637.1.S1_at_TCP11L1
MmugDNA.12638.1.S1_at	223802_s_at	RBBP6	retinoblastoma binding protein 6	chr16p12.2	5930	RBBP6	retinoblastoma binding protein 6	---	701569	MmugDNA.12638.1.S1_at_RBBP6
MmugDNA.12639.1.S1_at	243467_at	---	---	---	---	---	---	---	---	MmugDNA.12639.1.S1_at_---
MmugDNA.12640.1.S1_at	223630_at	C7orf13	chromosome 7 open reading frame 13	chr7q36	129790	LOC716668	hypothetical protein LOC716668	---	716668	MmugDNA.12640.1.S1_at_C7orf13
MmugDNA.12641.1.S1_at	236728_at	LNPEP	leucyl/cystinyl aminopeptidase	chr5q15	4012	---	---	---	---	MmugDNA.12641.1.S1_at_LNPEP
MmugDNA.1264.1.S1_at	1562821_a_at	---	---	---	---	---	---	---	---	MmugDNA.1264.1.S1_at_---
MmugDNA.12642.1.S1_at	243274_x_at	---	---	---	---	---	---	---	---	MmugDNA.12642.1.S1_at_---
MmugDNA.12643.1.S1_at	224276_at	ZNF33A	zinc finger protein 33A	chr10p11.2	7581	---	---	---	---	MmugDNA.12643.1.S1_at_ZNF33A
MmugDNA.12644.1.S1_at	226005_at	UBE2G1	ubiquitin-conjugating enzyme E2G 1 (UBC7 homolog, yeast)	chr1q42|17p13.2	7326	---	---	---	---	MmugDNA.12644.1.S1_at_UBE2G1
MmugDNA.12645.1.S1_at	1554080_at	RQCD1	RCD1 required for cell differentiation1 homolog (S. pombe)	chr2q35	9125	---	---	---	---	MmugDNA.12645.1.S1_at_RQCD1
MmugDNA.12646.1.S1_at	215004_s_at	SF4	splicing factor 4	chr19p13.11	57794	LOC719772	similar to splicing factor 4	---	719772	MmugDNA.12646.1.S1_at_SF4
MmugDNA.12647.1.S1_at	230519_at	FAM124A	family with sequence similarity 124A	chr13q14.3	220108	LOC708395	hypothetical protein LOC708395	---	708395	MmugDNA.12647.1.S1_at_FAM124A
MmugDNA.12648.1.S1_at	213293_s_at	TRIM22	tripartite motif-containing 22	chr11p15	10346	---	---	---	---	MmugDNA.12648.1.S1_at_TRIM22
MmugDNA.12649.1.S1_at	1559856_s_at	MLL	myeloid/lymphoid or mixed-lineage leukemia (trithorax homolog, Drosophila)	chr11q23	4297	MLL	myeloid/lymphoid or mixed-lineage leukemia (trithorax homolog, Drosophila)	---	705511	MmugDNA.12649.1.S1_at_MLL
MmugDNA.12650.1.S1_at	238453_at	FGFBP3	fibroblast growth factor binding protein 3	chr10q23.32	143282	LOC697881	hypothetical protein LOC697881	---	697881	MmugDNA.12650.1.S1_at_FGFBP3
MmugDNA.12651.1.S1_at	240396_at	IL20RA	Interleukin 20 receptor, alpha	chr6q22.33-q23.1	53832	---	---	---	---	MmugDNA.12651.1.S1_at_IL20RA
MmugDNA.1265.1.S1_at	209415_at	FZR1	fizzy/cell division cycle 20 related 1 (Drosophila)	chr19p13.3	51343	LOC721711	similar to Fzr1 protein	---	721711	MmugDNA.1265.1.S1_at_FZR1
MmugDNA.12652.1.S1_at	1565698_at	HECTD2	HECT domain containing 2	chr10q23.32	143279	---	---	---	---	MmugDNA.12652.1.S1_at_HECTD2
MmugDNA.12653.1.S1_at	NA	NA	NA	NA	NA	NA	NA	NA	NA	MmugDNA.12653.1.S1_at_NA
MmugDNA.12653.1.S1_s_at	223448_x_at	MRPS22	mitochondrial ribosomal protein S22	chr3q23	56945	MRPS22 /// MRPS22	similar to Mitochondrial 28S ribosomal protein S22 (S22mt) (MRP-S22) /// mitochondrial ribosomal pr	---	703571 /// 715845	MmugDNA.12653.1.S1_s_at_MRPS22
MmugDNA.12654.1.S1_at	234839_at	---	---	---	---	---	---	---	---	MmugDNA.12654.1.S1_at_---
MmugDNA.12655.1.S1_at	1568887_at	---	---	---	---	---	---	---	---	MmugDNA.12655.1.S1_at_---
MmugDNA.12656.1.S1_at	237206_at	MYOCD	myocardin	chr17p11.2	93649	LOC718193	similar to myocardin	---	718193	MmugDNA.12656.1.S1_at_MYOCD
MmugDNA.12657.1.S1_at	215809_at	CYP2D6	cytochrome P450, family 2, subfamily D, polypeptide 6	chr22q13.1	1565	---	---	---	---	MmugDNA.12657.1.S1_at_CYP2D6
MmugDNA.12658.1.S1_at	1557326_at	---	---	---	---	---	---	---	---	MmugDNA.12658.1.S1_at_---
MmugDNA.12659.1.S1_s_at	215948_x_at	ZMYM5	zinc finger, MYM-type 5	chr13q12	9205	CYP2B6	Cytochrome P450, family 2, subfamily B, polypeptide 6	---	678687	MmugDNA.12659.1.S1_s_at_ZMYM5
MmugDNA.12660.1.S1_at	226905_at	FAM101B	family with sequence similarity 101, member B	chr17p13	359845	---	---	---	---	MmugDNA.12660.1.S1_at_FAM101B
MmugDNA.12661.1.S1_at	1554972_at	---	---	---	---	---	---	---	---	MmugDNA.12661.1.S1_at_---
MmugDNA.1266.1.S1_at	240392_at	---	---	---	---	---	---	---	---	MmugDNA.1266.1.S1_at_---
MmugDNA.12662.1.S1_at	242291_at	EIF4ENIF1	eukaryotic translation initiation factor 4E nuclear import factor 1	chr22q11.2	56478	---	---	---	---	MmugDNA.12662.1.S1_at_EIF4ENIF1
MmugDNA.12663.1.S1_at	221902_at	GPR153	G protein-coupled receptor 153	chr1p36.31	387509	GPR153	G protein-coupled receptor 153	---	703169	MmugDNA.12663.1.S1_at_GPR153
MmugDNA.12664.1.S1_at	1554415_at	TAF5L	TAF5-like RNA polymerase II, p300/CBP-associated factor (PCAF)-associated factor, 65kDa	chr1q42.13	27097	TAF5L	TAF5-like RNA polymerase II, p300/CBP-associated factor (PCAF)-associated factor, 65kDa	---	693770	MmugDNA.12664.1.S1_at_TAF5L
MmugDNA.12665.1.S1_at	242230_at	ATXN1	ataxin 1	chr6p23	6310	---	---	---	---	MmugDNA.12665.1.S1_at_ATXN1
MmugDNA.12666.1.S1_at	212672_at	ATM	ataxia telangiectasia mutated	chr11q22-q23	472	---	---	---	---	MmugDNA.12666.1.S1_at_ATM
MmugDNA.12666.1.S1_s_at	NA	NA	NA	NA	NA	NA	NA	NA	NA	MmugDNA.12666.1.S1_s_at_NA
MmugDNA.12667.1.S1_at	240739_at	---	---	---	---	---	---	---	---	MmugDNA.12667.1.S1_at_---
MmugDNA.12668.1.S1_at	NA	NA	NA	NA	NA	NA	NA	NA	NA	MmugDNA.12668.1.S1_at_NA
MmugDNA.12668.1.S1_s_at	237955_at	LOC133874	hypothetical protein LOC133874	chr5q35.1	133874	---	---	---	---	MmugDNA.12668.1.S1_s_at_LOC13387
MmugDNA.12669.1.S1_at	222193_at	C2orf43	chromosome 2 open reading frame 43	chr2p24.1	60526	LOC717510	similar to CG9186-PB, isoform B	---	717510	MmugDNA.12669.1.S1_at_C2orf43
MmugDNA.12670.1.S1_at	223933_at	KIF5A	kinesin family member 5A	chr12q13.13	3798	LOC720348	Similar to actin related protein 2/3 complex subunit 1A	---	720348	MmugDNA.12670.1.S1_at_KIF5A
MmugDNA.12671.1.S1_at	230590_at	---	---	---	---	---	---	---	---	MmugDNA.12671.1.S1_at_---
MmugDNA.1267.1.S1_s_at	NA	NA	NA	NA	NA	NA	NA	NA	NA	MmugDNA.1267.1.S1_s_at_NA
MmugDNA.12672.1.S1_s_at	241874_at	C5orf53	chromosome 5 open reading frame 53	chr5q31	492311	---	---	---	---	MmugDNA.12672.1.S1_s_at_C5orf53
MmugDNA.12673.1.S1_at	234171_at	---	---	---	---	---	---	---	---	MmugDNA.12673.1.S1_at_---
MmugDNA.12674.1.S1_at	229948_at	---	---	---	---	---	---	---	---	MmugDNA.12674.1.S1_at_---
MmugDNA.12675.1.S1_at	1554842_at	SLC12A1	solute carrier family 12 (sodium/potassium/chloride transporters), member 1	chr15q15-q21.1	6557	SLC12A1	solute carrier family 12 (sodium/potassium/chloride transporters), member 1	---	714289	MmugDNA.12675.1.S1_at_SLC12A1
MmugDNA.12676.1.S1_at	238636_at	CACNA1C	calcium channel, voltage-dependent, L type, alpha 1C subunit	chr12p13.3	775	---	---	---	---	MmugDNA.12676.1.S1_at_CACNA1C
MmugDNA.12677.1.S1_at	239453_at	---	---	---	---	GAD2	Glutamate decarboxylase 2	---	613029	MmugDNA.12677.1.S1_at_---
MmugDNA.12678.1.S1_at	215418_at	PARVA	parvin, alpha	chr11p15.3	55742	---	---	---	---	MmugDNA.12678.1.S1_at_PARVA
MmugDNA.12679.1.S1_at	207422_at	ADAM20	ADAM metallopeptidase domain 20	chr14q24.1	8748	LOC715827	similar to ADAM metallopeptidase domain 20 preproprotein	---	715827	MmugDNA.12679.1.S1_at_ADAM20
MmugDNA.12680.1.S1_at	207665_at	ADAM21 /// ADAM21P	ADAM metallopeptidase domain 21 /// ADAM metallopeptidase domain 21 pseudogene	chr14q24.1 /// chr14q24.2	145241 /// 8747	LOC715542	similar to ADAM metallopeptidase domain 21 preproprotein	---	715542	MmugDNA.12680.1.S1_at_ADAM21 ///
MmugDNA.12681.1.S1_at	237383_at	---	---	---	---	---	---	---	---	MmugDNA.12681.1.S1_at_---
MmugDNA.1268.1.S1_at	230562_at	LOC100131112	similar to hCG2002332	chr8p23.1	100131112	---	---	---	---	MmugDNA.1268.1.S1_at_LOC1001311
MmugDNA.12682.1.S1_at	241243_at	---	---	---	---	---	---	---	---	MmugDNA.12682.1.S1_at_---
MmugDNA.12683.1.S1_at	241607_at	LOC730102	hypothetical protein LOC730102	chr1q25.2	730102	---	---	---	---	MmugDNA.12683.1.S1_at_LOC730102
MmugDNA.12684.1.S1_at	240271_at	---	---	---	---	---	---	---	---	MmugDNA.12684.1.S1_at_---
MmugDNA.12685.1.S1_at	204062_s_at	ULK2	unc-51-like kinase 2 (C. elegans)	chr17p11.2	9706	LOC695942	Similar to proteasome (prosome, macropain) 26S subunit, ATPase 2	---	695942	MmugDNA.12685.1.S1_at_ULK2
MmugDNA.12686.1.S1_at	244699_at	AHI1	Abelson helper integration site 1	chr6q23.3	54806	AHI1	Abelson helper integration site 1	---	706412	MmugDNA.12686.1.S1_at_AHI1
MmugDNA.12687.1.S1_at	239763_at	LOC100129965 /// PRD	hypothetical protein LOC100129965 /// PR domain containing 11	chr11p11 /// chr11p11.2	100129965 /// 56981	LOC714723	similar to PR domain containing 11	---	714723	MmugDNA.12687.1.S1_at_LOC100129
MmugDNA.12688.1.S1_at	221534_at	C11orf68	chromosome 11 open reading frame 68	chr11q13.1	83638	LOC715318	similar to basophilic leukemia expressed protein BLES03	---	715318	MmugDNA.12688.1.S1_at_C11orf68
MmugDNA.12689.1.S1_at	1569688_at	FMO5	flavin containing monooxygenase 5	chr1q21.1	2330	---	---	---	---	MmugDNA.12689.1.S1_at_FMO5
MmugDNA.12690.1.S1_at	230368_at	ERF	Ets2 repressor factor	chr19q13	2077	---	---	---	---	MmugDNA.12690.1.S1_at_ERF
MmugDNA.12691.1.S1_at	226583_at	C12orf76	chromosome 12 open reading frame 76	chr12q24.11	400073	LOC708599	hypothetical protein LOC708599	---	708599	MmugDNA.12691.1.S1_at_C12orf76

MmugDNA.1269.1.S1_at	214273_x_at	C16orf35	chromosome 16 open reading frame 35	chr16p13.3	8131	LOC722264	conserved gene telomeric to alpha globin cluster	---	722264	MmugDNA.1269.1.S1_at_C16orf35
MmugDNA.12692.1.S1_at	NA	NA	NA	NA	NA	NA	NA	NA	NA	MmugDNA.12692.1.S1_at_NA
MmugDNA.12693.1.S1_at	235893_at	---	---	---	---	---	---	---	---	MmugDNA.12693.1.S1_at_---
MmugDNA.12694.1.S1_at	238204_at	---	---	---	---	---	---	---	---	MmugDNA.12694.1.S1_at_---
MmugDNA.12696.1.S1_at	241690_at	C9orf144	chromosome 9 open reading frame 144	chr9p13.2	389715	LOC699468	hypothetical protein LOC699468	---	699468	MmugDNA.12696.1.S1_at_C9orf144
MmugDNA.12697.1.S1_at	208227_x_at	ADAM22	ADAM metallopeptidase domain 22	chr7q21	53616	ADAM22	ADAM metallopeptidase domain 22	---	705350	MmugDNA.12697.1.S1_at_ADAM22
MmugDNA.12698.1.S1_at	237881_at	---	---	---	---	---	---	---	---	MmugDNA.12698.1.S1_at_---
MmugDNA.12699.1.S1_at	244575_at	POLA2	Polymerase (DNA directed), alpha 2 (70kD subunit)	chr11q13.1	23649	---	---	---	---	MmugDNA.12699.1.S1_at_POLA2
MmugDNA.12700.1.S1_at	231664_at	SLC25A29	solute carrier family 25, member 29	chr14q32.2	123096	---	---	---	---	MmugDNA.12700.1.S1_at_SLC25A29
MmugDNA.12701.1.S1_at	1561963_at	---	---	---	---	---	---	---	---	MmugDNA.12701.1.S1_at_---
MmugDNA.1270.1.S1_at	216779_at	CYLC1	cylicin, basic protein of sperm head cytoskeleton 1	chrXq21.1	1538	LOC699976	Similar to Elongation factor 1-delta (EF-1-delta) (Antigen NY-CO-4)	---	699976	MmugDNA.1270.1.S1_at_CYLC1
MmugDNA.12702.1.S1_at	1557486_at	---	---	---	---	---	---	---	---	MmugDNA.12702.1.S1_at_---
MmugDNA.12703.1.S1_at	203170_at	RRP8	ribosomal RNA processing 8, methyltransferase, homolog (yeast)	chr11p15.4	23378	LOC712011	similar to T07A9.8	---	712011	MmugDNA.12703.1.S1_at_RRP8
MmugDNA.12704.1.S1_s_at	229748_x_at	LOC100132288	hypothetical protein LOC100132288	---	100132288	LOC708925	similar to Tektin-1	---	708925	MmugDNA.12704.1.S1_s_at_LOC10013
MmugDNA.12705.1.S1_at	235375_x_at	TTC9B	tetratricopeptide repeat domain 9B	chr19q13.2	148014	LOC701620	hypothetical protein LOC701620	---	701620	MmugDNA.12705.1.S1_at_TTC9B
MmugDNA.12706.1.S1_at	228987_at	---	---	---	---	LOC695449	similar to CG32066-PB, isoform B	---	695449	MmugDNA.12706.1.S1_at_---
MmugDNA.12707.1.S1_at	1554929_at	KIAA0999	KIAA0999 protein	chr11q23.3	23387	---	---	---	---	MmugDNA.12707.1.S1_at_KIAA0999
MmugDNA.12708.1.S1_at	1568924_a_at	IQUB	IQ motif and ubiquitin domain containing	chr7q31.32	154865	IQUB	IQ motif and ubiquitin domain containing	---	695424	MmugDNA.12708.1.S1_at_IQUB
MmugDNA.12709.1.S1_at	243612_at	NSD1	nuclear receptor binding SET domain protein 1	chr5q35.2-q35.3	64324	LOC706022	similar to nuclear receptor binding SET domain protein 1 isoform b	---	706022	MmugDNA.12709.1.S1_at_NSD1
MmugDNA.12710.1.S1_at	233034_at	---	---	---	---	---	---	---	---	MmugDNA.12710.1.S1_at_---
MmugDNA.12711.1.S1_at	206956_at	BGLAP	bone gamma-carboxyglutamate (gla) protein	chr1q25-q31	632	BGLAP	bone gamma-carboxyglutamate (gla) protein	---	718296	MmugDNA.12711.1.S1_at_BGLAP
MmugDNA.1271.1.S1_at	207609_s_at	CYP1A2	cytochrome P450, family 1, subfamily A, polypeptide 2	chr15q24.1	1544	LOC721176	Similar to active breakpoint cluster region-related protein isoform b	---	721176	MmugDNA.1271.1.S1_at_CYP1A2
MmugDNA.12712.1.S1_s_at	235082_at	PCNX	pecanex homolog (Drosophila)	chr14q24.2	22990	---	---	---	---	MmugDNA.12712.1.S1_s_at_PCNX
MmugDNA.12713.1.S1_at	238789_at	KANK1	KN motif and ankyrin repeat domains 1	chr9p24.3	23189	KANK1	similar to ankyrin repeat domain protein 15 isoform a	---	697569	MmugDNA.12713.1.S1_at_KANK1
MmugDNA.12714.1.S1_at	201262_s_at	BGN	biglycan	chrXq28	633	BGN	biglycan	---	697544	MmugDNA.12714.1.S1_at_BGN
MmugDNA.12715.1.S1_s_at	1554148_a_at	SLC33A1	solute carrier family 33 (acetyl-CoA transporter), member 1	chr3q25.31	9197	LOC721420	similar to acetyl-coenzyme A transporter	---	721420	MmugDNA.12715.1.S1_s_at_SLC33A1
MmugDNA.12716.1.S1_at	222508_s_at	ARGLU1	arginine and glutamate rich 1	chr13q33.3	55082	LOC702840	hypothetical protein LOC702840	---	702840	MmugDNA.12716.1.S1_at_ARGLU1
MmugDNA.12717.1.S1_at	206576_s_at	CEACAM1	carcinoembryonic antigen-related cell adhesion molecule 1 (biliary glycoprotein)	chr19q13.2	634	LOC708200	similar to carcinoembryonic antigen-related cell adhesion molecule 1 isoform 1 precursor	---	708200	MmugDNA.12717.1.S1_at_CEACAM1
MmugDNA.12718.1.S1_at	1552558_a_at	RAI1	retinoic acid induced 1	chr17p11.2	10743	RAI1	retinoic acid induced 1	---	699215	MmugDNA.12718.1.S1_at_RAI1
MmugDNA.12719.1.S1_at	238827_at	---	---	---	---	---	---	---	---	MmugDNA.12719.1.S1_at_---
MmugDNA.127.1.S1_at	NA	NA	NA	NA	NA	NA	NA	NA	NA	MmugDNA.127.1.S1_at_NA
MmugDNA.127.1.S1_x_at	217322_x_at	---	---	---	---	LOC705493	hypothetical protein LOC705493	---	705493	MmugDNA.127.1.S1_x_at_---
MmugDNA.12721.1.S1_at	229821_at	---	---	---	---	---	---	---	---	MmugDNA.12721.1.S1_at_---
MmugDNA.1272.1.S1_at	1555943_at	PGAM5	phosphoglycerate mutase family member 5	chr12q24.33	192111	PGAM5	phosphoglycerate mutase family member 5	---	693782	MmugDNA.1272.1.S1_at_PGAM5
MmugDNA.12722.1.S1_at	1570102_at	---	---	---	---	---	---	---	---	MmugDNA.12722.1.S1_at_---
MmugDNA.12723.1.S1_at	224551_s_at	SPTBN4	spectrin, beta, non-erythrocytic 4	chr19q13.13	57731	SPTBN4	spectrin, beta, non-erythrocytic 4	---	701922	MmugDNA.12723.1.S1_at_SPTBN4
MmugDNA.12724.1.S1_at	240110_at	HMGCS2	3-hydroxy-3-methylglutaryl-Coenzyme A synthase 2 (mitochondrial)	chr1p13-p12	3158	HMGCS2	3-hydroxy-3-methylglutaryl-Coenzyme A synthase 2 (mitochondrial)	---	713541	MmugDNA.12724.1.S1_at_HMGCS2
MmugDNA.12725.1.S1_at	213338_at	TMEM158	transmembrane protein 158	chr3p21.3	25907	---	---	---	---	MmugDNA.12725.1.S1_at_TMEM158
MmugDNA.12726.1.S1_at	224297_s_at	SPTBN4	spectrin, beta, non-erythrocytic 4	chr19q13.13	57731	SPTBN4	spectrin, beta, non-erythrocytic 4	---	701922	MmugDNA.12726.1.S1_at_SPTBN4
MmugDNA.12727.1.S1_at	239262_at	---	---	---	---	---	---	---	---	MmugDNA.12727.1.S1_at_---
MmugDNA.12728.1.S1_at	242807_at	FSD1L	fibronectin type III and SPRY domain containing 1-like	chr9q31	83856	LOC712272	similar to fibronectin type 3 and SPRY domain-containing protein	---	712272	MmugDNA.12728.1.S1_at_FSD1L
MmugDNA.12729.1.S1_at	1566623_at	---	---	---	---	---	---	---	---	MmugDNA.12729.1.S1_at_---
MmugDNA.12730.1.S1_at	215427_s_at	ZCCHC14	zinc finger, CCHC domain containing 14	chr16q24.2	23174	TAF6	TBP-associated factor 6	---	710863	MmugDNA.12730.1.S1_at_ZCCHC14
MmugDNA.12731.1.S1_at	240224_at	---	---	---	---	---	---	---	---	MmugDNA.12731.1.S1_at_---
MmugDNA.1273.1.S1_at	209491_s_at	AMPD3	adenosine monophosphate deaminase (isoform E)	chr11p15	272	AMPD3	adenosine monophosphate deaminase (isoform E)	---	703186	MmugDNA.1273.1.S1_at_AMPD3
MmugDNA.12732.1.S1_at	236984_at	C4orf26	chromosome 4 open reading frame 26	chr4q21.1	152816	---	---	---	---	MmugDNA.12732.1.S1_at_C4orf26
MmugDNA.12733.1.S1_at	215365_at	CACNB2	calcium channel, voltage-dependent, beta 2 subunit	chr10p12	783	---	---	---	---	MmugDNA.12733.1.S1_at_CACNB2
MmugDNA.12734.1.S1_at	210039_s_at	PRKCQ	protein kinase C, theta	chr10p15	5588	---	---	---	---	MmugDNA.12734.1.S1_at_PRKCQ
MmugDNA.12735.1.S1_at	215820_x_at	SNX13	sorting nexin 13	chr7p21.1	23161	SNX13	sorting nexin 13	---	714019	MmugDNA.12735.1.S1_at_SNX13
MmugDNA.12736.1.S1_at	206255_at	BLK	B lymphoid tyrosine kinase	chr8p23-p22	640	LOC696892	similar to B lymphoid tyrosine kinase	---	696892	MmugDNA.12736.1.S1_at_BLK
MmugDNA.12737.1.S1_at	212165_at	TMEM183A /// TMEM18	transmembrane protein 183A /// transmembrane protein 183B	chr1q32.1 /// chr3q25.1	653659 /// 92703	LOC704195	similar to CG12765-PA	---	704195	MmugDNA.12737.1.S1_at_TMEM183A
MmugDNA.12738.1.S1_at	214974_x_at	CXCL5	chemokine (C-X-C motif) ligand 5	chr4q12-q13	6374	---	---	---	---	MmugDNA.12738.1.S1_at_CXCL5
MmugDNA.12739.1.S1_at	212164_at	TMEM183A	transmembrane protein 183A	chr1q32.1	92703	---	---	---	---	MmugDNA.12739.1.S1_at_TMEM183A
MmugDNA.12740.1.S1_at	206126_at	CXCR5	chemokine (C-X-C motif) receptor 5	chr11q23.3	643	---	---	---	---	MmugDNA.12740.1.S1_at_CXCR5
MmugDNA.1274.1.S1_at	239713_at	CASC2	cancer susceptibility candidate 2	chr10q26.11	255082	---	---	---	---	MmugDNA.1274.1.S1_at_CASC2
MmugDNA.12742.1.S1_at	1561493_at	---	---	---	---	---	---	---	---	MmugDNA.12742.1.S1_at_---
MmugDNA.12743.1.S1_at	202201_at	BLVRB	biliverdin reductase B (flavin reductase (NADPH))	chr19q13.1-q13.2	645	BLVRB	biliverdin reductase B (flavin reductase (NADPH))	---	703020	MmugDNA.12743.1.S1_at_BLVRB
MmugDNA.12744.1.S1_at	1570303_at	---	---	---	---	---	---	---	---	MmugDNA.12744.1.S1_at_---
MmugDNA.12745.1.S1_at	214265_at	ITGA8	integrin, alpha 8	chr10p13	8516	ITGA8	integrin, alpha 8	---	698888	MmugDNA.12745.1.S1_at_ITGA8
MmugDNA.12746.1.S1_at	NA	NA	NA	NA	NA	NA	NA	NA	NA	MmugDNA.12746.1.S1_at_NA
MmugDNA.12747.1.S1_at	1559976_at	---	---	---	---	---	---	---	---	MmugDNA.12747.1.S1_at_---
MmugDNA.12748.1.S1_at	NA	NA	NA	NA	NA	NA	NA	NA	NA	MmugDNA.12748.1.S1_at_NA
MmugDNA.12749.1.S1_s_at	209146_at	SC4MOL	sterol-C4-methyl oxidase-like	chr4q32-q34	6307	LOC701512	Similar to ataxin 7	---	701512	MmugDNA.12749.1.S1_s_at_SC4MOL
MmugDNA.12750.1.S1_at	235864_at	MPP5	Membrane protein, palmitoylated 5 (MAGUK p55 subfamily member 5)	chr14q23.3	64398	---	---	---	---	MmugDNA.12750.1.S1_at_MPP5
MmugDNA.12751.1.S1_at	233795_at	ODF3	outer dense fiber of sperm tails 3	chr11p15.5	113746	ODF3	outer dense fiber of sperm tails 3	---	696480	MmugDNA.12751.1.S1_at_ODF3
MmugDNA.1275.1.S1_at	NA	NA	NA	NA	NA	NA	NA	NA	NA	MmugDNA.1275.1.S1_at_NA
MmugDNA.1275.1.S1_x_at	243662_at	---	---	---	---	---	---	---	---	MmugDNA.1275.1.S1_x_at_---
MmugDNA.12752.1.S1_s_at	212526_at	SPG20	spastic paraplegia 20 (Troyer syndrome)	chr13q13.3	23111	SPG20	spastic paraplegia 20 (Troyer syndrome)	---	693663	MmugDNA.12752.1.S1_s_at_SPG20
MmugDNA.12753.1.S1_at	1561871_at	---	---	---	---	---	---	---	---	MmugDNA.12753.1.S1_at_---
MmugDNA.12754.1.S1_s_at	210356_x_at	MS4A1	membrane-spanning 4-domains, subfamily A, member 1	chr11q12	931	MS4A1	membrane-spanning 4-domains, subfamily A, member 1	---	696843	MmugDNA.12754.1.S1_s_at_MS4A1
MmugDNA.12755.1.S1_s_at	205574_x_at	BMP1	bone morphogenetic protein 1	chr8p21.3	649	BMP1	bone morphogenetic protein 1	---	707596	MmugDNA.12755.1.S1_s_at_BMP1
MmugDNA.12756.1.S1_at	231346_s_at	---	---	---	---	---	---	---	---	MmugDNA.12756.1.S1_at_---
MmugDNA.12757.1.S1_s_at	209407_s_at	DEAF1	deformed epidermal autoregulatory factor 1 (Drosophila)	chr11p15.5	10522	DEAF1	deformed epidermal autoregulatory factor 1	---	720821	MmugDNA.12757.1.S1_s_at_DEAF1
MmugDNA.12758.1.S1_at	243560_at	---	---	---	---	---	---	---	---	MmugDNA.12758.1.S1_at_---
MmugDNA.12759.1.S1_at	207865_s_at	BMP8B	bone morphogenetic protein 8b	chr1p35-p32	656	LOC718677	similar to 3-oxoacid CoA transferase 2	---	718677	MmugDNA.12759.1.S1_at_BMP8B
MmugDNA.12760.1.S1_at	230288_at	FGF14	fibroblast growth factor 14	chr13q34	2259	FGF14	fibroblast growth factor 14	---	701077	MmugDNA.12760.1.S1_at_FGF14
MmugDNA.12761.1.S1_at	233527_at	---	---	---	---	---	---	---	---	MmugDNA.12761.1.S1_at_---
MmugDNA.1276.1.S1_at	1561612_at	---	---	---	---	---	---	---	---	MmugDNA.1276.1.S1_at_---
MmugDNA.12762.1.S1_s_at	1555278_a_at	CKAP5	cytoskeleton associated protein 5	chr11p11.2	9793	CKAP5	colonic and hepatic tumor over-expressed protein	---	714095	MmugDNA.12762.1.S1_s_at_CKAP5
MmugDNA.12763.1.S1_at	238496_at	---	---	---	---	---	---	---	---	MmugDNA.12763.1.S1_at_---
MmugDNA.12764.1.S1_at	215993_at	---	---	---	---	---	---	---	---	MmugDNA.12764.1.S1_at_---
MmugDNA.12765.1.S1_at	229914_at	FLJ38717	FLJ38717 protein	chr6p21.1	401261	---	---	---	---	MmugDNA.12765.1.S1_at_FLJ38717
MmugDNA.12766.1.S1_at	NA	NA	NA	NA	NA	NA	NA	NA	NA	MmugDNA.12766.1.S1_at_NA
MmugDNA.12766.1.S1_s_at	217159_x_at	SIGLEC7	sialic acid binding Ig-like lectin 7	chr19q13.3	27036	LOC719831	similar to sialic acid binding Ig-like lectin 9	---	719831	MmugDNA.12766.1.S1_s_at_SIGLEC7
MmugDNA.12767.1.S1_at	237903_at	---	---	---	---	---	---	---	---	MmugDNA.12767.1.S1_at_---
MmugDNA.12768.1.S1_at	1569268_at	GRIN2C	glutamate receptor, ionotropic, N-methyl D-aspartate 2C	chr17q25	2905	GRIN2C	N-methyl-D-aspartate receptor subunit 2C	---	699216	MmugDNA.12768.1.S1_at_GRIN2C
MmugDNA.12769.1.S1_at	224149_x_at	SLMAP	sarcolemma associated protein	chr3p21.2-p14.3	7871	SLMAP	sarcolemma associated protein	---	703086	MmugDNA.12769.1.S1_at_SLMAP
MmugDNA.12770.1.S1_at	233484_at	---	---	---	---	---	---	---	---	MmugDNA.12770.1.S1_at_---
MmugDNA.12771.1.S1_at	1562319_at	FAM169B	family with sequence similarity 169, member B	chr15q26.3	283777	---	---	---	---	MmugDNA.12771.1.S1_at_FAM169B
MmugDNA.1277.1.S1_at	235621_at	LOC285014	hypothetical protein LOC285014	chr2q11.2	285014	---	---	---	---	MmugDNA.1277.1.S1_at_LOC285014
MmugDNA.12772.1.S1_at	227764_at	LYPD6	LY6/PLAUR domain containing 6	chr2q23.2	130574	---	---	---	---	MmugDNA.12772.1.S1_at_LYPD6
MmugDNA.12773.1.S1_at	227763_at	LYPD6	LY6/PLAUR domain containing 6	chr2q23.2	130574	---	---	---	---	MmugDNA.12773.1.S1_at_LYPD6
MmugDNA.12774.1.S1_at	204358_s_at	FLRT2	fibronectin leucine rich transmembrane protein 2	chr14q24-q32	23768	FLRT2	fibronectin leucine rich transmembrane protein 2	---	709329	MmugDNA.12774.1.S1_at_FLRT2
MmugDNA.12775.1.S1_at	229353_s_at	NUCKS1	nuclear casein kinase and cyclin-dependent kinase substrate 1	chr1q32.1	64710	LOC704875	Similar to ubiquitin-activating enzyme E1-like	---	704875	MmugDNA.12775.1.S1_at_NUCKS1
MmugDNA.12775.1.S1_x_at	NA	NA	NA	NA	NA	NA	NA	NA	NA	MmugDNA.12775.1.S1_x_at_NA
MmugDNA.12776.1.S1_at	222540_s_at	RSF1	remodeling and spacing factor 1	chr11q14.1	51773	RSF1	remodeling and spacing factor 1	---	699078	MmugDNA.12776.1.S1_at_RSF1
MmugDNA.12777.1.S1_at	233293_at	---	---	---	---	---	---	---	---	MmugDNA.12777.1.S1_at_---
MmugDNA.12778.1.S1_at	204678_s_at	KCNK1	potassium channel, subfamily K, member 1	chr1q42-q43	3775	KCNK1	potassium channel, subfamily K, member 1	---	718815	MmugDNA.12778.1.S1_at_KCNK1
MmugDNA.12779.1.S1_at	222541_at	RSF1	remodeling and spacing factor 1	chr11q14.1	51773	RSF1	remodeling and spacing factor 1	---	699078	MmugDNA.12779.1.S1_at_RSF1
MmugDNA.12780.1.S1_at	206464_at	BMX	BMX non-receptor tyrosine kinase	chrXp22.2	660	BMX	BMX non-receptor tyrosine kinase	---	712722	MmugDNA.12780.1.S1_at_BMX
MmugDNA.12781.1.S1_at	239132_at	NOS1	Nitric oxide synthase 1 (neuronal)	chr12q24.2-q24.31	4842	---	---	---	---	MmugDNA.12781.1.S1_at_NOS1
MmugDNA.1278.1.S1_at	230782_at	SORD	sorbitol dehydrogenase	chr15q15.3	6652	---	---	---	---	MmugDNA.1278.1.S1_at_SORD
MmugDNA.12782.1.S1_at	213632_at	DHODH	dihydroorotate dehydrogenase	chr16q22	1723	DHODH	dihydroorotate dehydrogenase	---	706936	MmugDNA.12782.1.S1_at_DHODH
MmugDNA.12783.1.S1_at	235033_at	NPEPL1	Aminopeptidase-like 1	chr20q13.32	79716	---	---	---	---	MmugDNA.12783.1.S1_at_NPEPL1
MmugDNA.12784.1.S1_at	224930_x_at	RPL7A	ribosomal protein L7a	chr9q34	6130	LOC693416	similar to 60S ribosomal protein L7a	---	693416	MmugDNA.12784.1.S1_at_RPL7A
MmugDNA.12785.1.S1_at	224409_s_at	TSSK6	testis-specific serine kinase 6	chr19p13.11	83983	LOC716627	similar to serine/threonine protein kinase SSTK	---	716627	MmugDNA.12785.1.S1_at_TSSK6
MmugDNA.12786.1.S1_at	208798_x_at	GOLGA8A	golgi autoantigen, golgin subfamily a, 8A	chr15q11.2	23015	LOC695266	similar to golgi autoantigen, golgin subfamily a, 8A	---	695266	MmugDNA.12786.1.S1_at_GOLGA8A
MmugDNA.12787.1.S1_at	221748_s_at	TNS1	tensin 1	chr2q35-q36	7145	---	---	---	---	MmugDNA.12787.1.S1_at_TNS1

MmugDNA.12788.1.S1_at	231813_s_at	COG1	component of oligomeric golgi complex 1	chr17q25.1		9382	COG1	component of oligomeric golgi complex 1	---		695754	MmugDNA.12788.1.S1_at_COG1
MmugDNA.12789.1.S1_s_at	NA	NA	NA	NA	NA		NA	NA	NA	NA		MmugDNA.12789.1.S1_s_at_NA
MmugDNA.12790.1.S1_at	204455_at	DST	dystonin	chr6p12.1		667	---	---	---	---		MmugDNA.12790.1.S1_at_DST
MmugDNA.12791.1.S1_at	203502_at	BPGM	2,3-bisphosphoglycerate mutase	chr7q31-q34		669	LIPF	Lipase, gastric	---		693586	MmugDNA.12791.1.S1_at_BPGM
MmugDNA.1279.1.S1_at	243222_at	---	---	---	---		---	---	---	---		MmugDNA.1279.1.S1_at_---
MmugDNA.12792.1.S1_at	NA	NA	NA	NA	NA		NA	NA	NA	NA		MmugDNA.12792.1.S1_at_NA
MmugDNA.12793.1.S1_at	226260_x_at	ZNF358	zinc finger protein 358	---		140467	LOC707823	similar to Zinc finger protein 358	---		707823	MmugDNA.12793.1.S1_at_ZNF358
MmugDNA.12795.1.S1_at	209096_at	UBE2V2	ubiquitin-conjugating enzyme E2 variant 2	chr8q11.21		7336	LOC695773	Similar to 60S ribosomal protein L23a	---		695773	MmugDNA.12795.1.S1_at_UBE2V2
MmugDNA.12796.1.S1_at	227242_s_at	EBF3	early B-cell factor 3	chr10q26.3		253738	LOC713536	similar to early B-cell factor 3	---		713536	MmugDNA.12796.1.S1_at_EBF3
MmugDNA.12797.1.S1_at	227243_s_at	EBF3	early B-cell factor 3	chr10q26.3		253738	LOC713536	similar to early B-cell factor 3	---		713536	MmugDNA.12797.1.S1_at_EBF3
MmugDNA.12798.1.S1_at	235786_at	---	---	---	---		---	---	---	---		MmugDNA.12798.1.S1_at_---
MmugDNA.12799.1.S1_at	228801_at	ORMDL1	ORM1-like 1 (S. cerevisiae)	chr2q32		94101	ORMDL1	ORM1-like 1 (S. cerevisiae)	---		709832	MmugDNA.12799.1.S1_at_ORMDL1
MmugDNA.12800.1.S1_at	1564083_at	---	---	---	---		---	---	---	---		MmugDNA.12800.1.S1_at_---
MmugDNA.12801.1.S1_at	229547_s_at	WNK2	WNK lysine deficient protein kinase 2	chr9q22.3		65268	LOC717275	similar to WNK lysine deficient protein kinase 2	---		717275	MmugDNA.12801.1.S1_at_WNK2
MmugDNA.1280.1.S1_at	244066_at	---	---	---	---		---	---	---	---		MmugDNA.1280.1.S1_at_---
MmugDNA.12802.1.S1_at	1558613_at	---	---	---	---		---	---	---	---		MmugDNA.12802.1.S1_at_---
MmugDNA.12803.1.S1_s_at	214280_x_at	HNRNPA1	heterogeneous nuclear ribonucleoprotein A1	chr12q13.1		3178	HNRPA1 /// HNRPA1 /// I	similar to Heterogeneous nuclear ribonucleoprotein A1 (Helix-destabilizing protein) (Single-strand RN	---		693386 /// 693712 /// 7007	MmugDNA.12803.1.S1_s_at_HNRNPA1
MmugDNA.12804.1.S1_at	1556439_at	---	---	---	---		---	---	---	---		MmugDNA.12804.1.S1_at_---
MmugDNA.12805.1.S1_at	241228_at	---	---	---	---		---	---	---	---		MmugDNA.12805.1.S1_at_---
MmugDNA.12806.1.S1_at	242120_at	---	---	---	---		---	---	---	---		MmugDNA.12806.1.S1_at_---
MmugDNA.12807.1.S1_at	1561322_at	---	---	---	---		---	---	---	---		MmugDNA.12807.1.S1_at_---
MmugDNA.12808.1.S1_at	227840_at	C2orf76	chromosome 2 open reading frame 76	chr2q14.2		130355	LOC694824	hypothetical protein LOC694824	---		694824	MmugDNA.12808.1.S1_at_C2orf76
MmugDNA.12809.1.S1_at	230985_at	C9orf131	chromosome 9 open reading frame 131	chr9p13.3		138724	---	---	---	---		MmugDNA.12809.1.S1_at_C9orf131
MmugDNA.12810.1.S1_at	208368_s_at	BRCA2	breast cancer 2, early onset	chr13q12.3		675	BRCA2	breast cancer 2, early onset	---		721981	MmugDNA.12810.1.S1_at_BRCA2
MmugDNA.12811.1.S1_at	206787_at	BRDT	bromodomain, testis-specific	chr1p22.1		676	LOC711998	similar to testis-specific bromodomain protein	---		711998	MmugDNA.12811.1.S1_at_BRDT
MmugDNA.1281.1.S1_at	240461_at	---	---	---	---		---	---	---	---		MmugDNA.1281.1.S1_at_---
MmugDNA.12812.1.S1_at	222575_at	SETD5	SET domain containing 5	chr3p25.3		55209	SETD5	SET domain containing 5	---		704174	MmugDNA.12812.1.S1_at_SETD5
MmugDNA.12813.1.S1_at	229049_at	---	---	---	---		---	---	---	---		MmugDNA.12813.1.S1_at_---
MmugDNA.12814.1.S1_at	1560031_at	FRMD4A	FERM domain containing 4A	chr10p13		55691	---	---	---	---		MmugDNA.12814.1.S1_at_FRMD4A
MmugDNA.12815.1.S1_at	240891_at	FSTL1	Follistatin-like 1	chr3q13.33		11167	---	---	---	---		MmugDNA.12815.1.S1_at_FSTL1
MmugDNA.12816.1.S1_at	231388_at	ZNF318	Zinc finger protein 318	chr6pter-p12.1		24149	---	---	---	---		MmugDNA.12816.1.S1_at_ZNF318
MmugDNA.12817.1.S1_at	226483_at	TMEM68	transmembrane protein 68	chr8q12.1		137695	LOC695447	similar to transmembrane protein 68	---		695447	MmugDNA.12817.1.S1_at_TMEM68
MmugDNA.12818.1.S1_at	237044_s_at	C10orf41	chromosome 10 open reading frame 41	chr10q22.2		100131213	---	---	---	---		MmugDNA.12818.1.S1_at_C10orf41
MmugDNA.12819.1.S1_at	1570597_at	---	---	---	---		---	---	---	---		MmugDNA.12819.1.S1_at_---
MmugDNA.128.1.S1_at	231133_at	C2orf39	chromosome 2 open reading frame 39	chr2p23.3		92749	LOC696596	similar to CG10958-PA	---		696596	MmugDNA.128.1.S1_at_C2orf39
MmugDNA.12820.1.S1_at	1558756_at	C2CD3	C2 calcium-dependent domain containing 3	chr11q13.4		26005	LOC718842	hypothetical protein LOC718842	---		718842	MmugDNA.12820.1.S1_at_C2CD3
MmugDNA.12821.1.S1_at	236038_at	---	---	---	---		---	---	---	---		MmugDNA.12821.1.S1_at_---
MmugDNA.1282.1.S1_at	1553911_at	ZNF663	zinc finger protein 663	chr20q13.12		100130934	---	---	---	---		MmugDNA.1282.1.S1_at_ZNF663
MmugDNA.12822.1.S1_at	1555341_at	UNC5C	unc-5 homolog C (C. elegans)	chr4q21-q23		8633	LOC705591	similar to unc5C	---		705591	MmugDNA.12822.1.S1_at_UNC5C
MmugDNA.12823.1.S1_at	227678_at	XRCC6BP1	XRCC6 binding protein 1	chr12q14.1		91419	LOC712932	similar to Ku70-binding protein 3	---		712932	MmugDNA.12823.1.S1_at_XRCC6BP1
MmugDNA.12824.1.S1_at	233271_at	---	---	---	---		---	---	---	---		MmugDNA.12824.1.S1_at_---
MmugDNA.12825.1.S1_at	240719_at	---	---	---	---		---	---	---	---		MmugDNA.12825.1.S1_at_---
MmugDNA.12826.1.S1_x_at	208517_x_at	BTF3	basic transcription factor 3	chr5q13.2		689	BTF3	basic transcription factor 3	---		703339	MmugDNA.12826.1.S1_x_at_BTF3
MmugDNA.12827.1.S1_at	208529_at	BTF3L1	basic transcription factor 3, like 1 pseudogene	chr13q22		690	LOC695868	similar to Transcription factor BTF3 homolog 1 (Basic transcription factor 3-like 1)	---		695868	MmugDNA.12827.1.S1_at_BTF3L1
MmugDNA.12828.1.S1_at	205699_at	MAP2K6	mitogen-activated protein kinase kinase 6	chr17q24.3		5608	---	---	---	---		MmugDNA.12828.1.S1_at_MAP2K6
MmugDNA.12829.1.S1_at	243509_at	---	---	---	---		---	---	---	---		MmugDNA.12829.1.S1_at_---
MmugDNA.12830.1.S1_at	228687_at	ZNF777	Zinc finger protein 777	chr7q36.1		27153	---	---	---	---		MmugDNA.12830.1.S1_at_ZNF777
MmugDNA.1283.1.S1_at	1566428_at	---	---	---	---		---	---	---	---		MmugDNA.1283.1.S1_at_---
MmugDNA.12832.1.S1_at	240851_at	---	---	---	---		---	---	---	---		MmugDNA.12832.1.S1_at_---
MmugDNA.12833.1.S1_at	239053_at	CIAO1	cytosolic iron-sulfur protein assembly 1 homolog (S. cerevisiae)	chr2q11.2		9391	---	---	---	---		MmugDNA.12833.1.S1_at_CIAO1
MmugDNA.12834.1.S1_at	239782_at	RBP1	Retinol binding protein 1, cellular	chr3q23		5947	---	---	---	---		MmugDNA.12834.1.S1_at_RBP1
MmugDNA.12835.1.S1_at	224327_s_at	DGAT2	diacylglycerol O-acyltransferase homolog 2 (mouse)	chr11q13.5		84649	LOC696549	similar to diacylglycerol O-acyltransferase homolog 2	---		696549	MmugDNA.12835.1.S1_at_DGAT2
MmugDNA.12836.1.S1_at	210407_at	PPM1A	protein phosphatase 1A (formerly 2C), magnesium-dependent, alpha isoform	chr14q23.1		5494	LOC702987	similar to protein phosphatase 1A isoform 1	---		702987	MmugDNA.12836.1.S1_at_PPM1A
MmugDNA.12837.1.S1_at	1552662_a_at	PCDHGB7	protocadherin gamma subfamily B, 7	chr5q31		56099	LOC700430	similar to protocadherin gamma subfamily B, 7 isoform 1 precursor	---		700430	MmugDNA.12837.1.S1_at_PCDHGB7
MmugDNA.12838.1.S1_at	244165_at	C10orf18	chromosome 10 open reading frame 18	chr10p15.1		54906	---	---	---	---		MmugDNA.12838.1.S1_at_C10orf18
MmugDNA.12839.1.S1_at	240595_at	---	---	---	---		---	---	---	---		MmugDNA.12839.1.S1_at_---
MmugDNA.12840.1.S1_at	NA	NA	NA	NA	NA		NA	NA	NA	NA		MmugDNA.12840.1.S1_at_NA
MmugDNA.12840.1.S1_s_at	NA	NA	NA	NA	NA		NA	NA	NA	NA		MmugDNA.12840.1.S1_s_at_NA
MmugDNA.12841.1.S1_at	222912_at	ARRB1	arrestin, beta 1	chr11q13		408	LOC695250	similar to arrestin beta 1 isoform A	---		695250	MmugDNA.12841.1.S1_at_ARRB1
MmugDNA.1284.1.S1_s_at	223819_x_at	COMMD5	COMM domain containing 5	chr8q24-qter		28991	LOC704586	similar to hypertension-related calcium-regulated gene	---		704586	MmugDNA.1284.1.S1_s_at_COMMD5
MmugDNA.12842.1.S1_at	1560775_at	---	---	---	---		---	---	---	---		MmugDNA.12842.1.S1_at_---
MmugDNA.12843.1.S1_at	233580_at	---	---	---	---		LOC705912	hypothetical protein LOC705912	---		705912	MmugDNA.12843.1.S1_at_---
MmugDNA.12844.1.S1_at	230785_at	---	---	---	---		---	---	---	---		MmugDNA.12844.1.S1_at_---
MmugDNA.12845.1.S1_at	241142_at	---	---	---	---		---	---	---	---		MmugDNA.12845.1.S1_at_---
MmugDNA.12846.1.S1_s_at	225156_at	ELOF1	elongation factor 1 homolog (S. cerevisiae)	chr19p13.2		84337	LOC715007	similar to elongation factor 1 homolog (ELF1, S. cerevisiae)	---		715007	MmugDNA.12846.1.S1_s_at_ELOF1
MmugDNA.12848.1.S1_at	210787_s_at	CAMKK2	calcium/calmodulin-dependent protein kinase kinase 2, beta	chr12q24.2		10645	CAMKK2	calcium/calmodulin-dependent protein kinase kinase 2, beta	---		699685	MmugDNA.12848.1.S1_at_CAMKK2
MmugDNA.12849.1.S1_at	225860_at	LOC729580	hypothetical LOC729580	chr20q11.22		729580	EIF6	eukaryotic translation initiation factor 6	---		711933	MmugDNA.12849.1.S1_at_LOC729580
MmugDNA.12850.1.S1_at	232702_at	RABGAP1L	RAB GTPase activating protein 1-like	chr1q24		9910	---	---	---	---		MmugDNA.12850.1.S1_at_RABGAP1L
MmugDNA.12851.1.S1_at	209197_at	SYT11	synaptotagmin XI	chr1q21.2		23208	---	---	---	---		MmugDNA.12851.1.S1_at_SYT11
MmugDNA.1285.1.S1_at	214310_s_at	ZFPL1	zinc finger protein-like 1	chr11q13		7542	LOC716932	similar to zinc finger protein-like 1	---		716932	MmugDNA.1285.1.S1_at_ZFPL1
MmugDNA.12852.1.S1_at	233556_at	CRYBB2P1	Crystallin, beta B2 pseudogene 1	chr22q11.2-q12.1		1416	---	---	---	---		MmugDNA.12852.1.S1_at_CRYBB2P1
MmugDNA.12853.1.S1_at	241771_at	RIMBP2	RIMS binding protein 2	chr12q24.33		23504	---	---	---	---		MmugDNA.12853.1.S1_at_RIMBP2
MmugDNA.12854.1.S1_at	226872_at	RFX2	regulatory factor X, 2 (influences HLA class II expression)	chr19p13.3-p13.2		5990	---	---	---	---		MmugDNA.12854.1.S1_at_RFX2
MmugDNA.12855.1.S1_at	40359_at	RASSF7	Ras association (RalGDS/AF-6) domain family (N-terminal) member 7	chr11p15.5		8045	RASSF7	similar to Ras association domain-containing protein 7 (HRAS1-related cluster protein 1)	---		699206	MmugDNA.12855.1.S1_at_RASSF7
MmugDNA.12856.1.S1_at	232450_at	LOC149351	hypothetical protein LOC149351	chr1p22.2		149351	LOC697267	hypothetical protein LOC697267	---		697267	MmugDNA.12856.1.S1_at_LOC149351
MmugDNA.12857.1.S1_at	205413_at	MPPED2	metallophosphoesterase domain containing 2	chr11p13		744	MPPED2	metallophosphoesterase domain containing 2	---		696968	MmugDNA.12857.1.S1_at_MPPED2
MmugDNA.12858.1.S1_at	234923_at	GARNL1	GTPase activating Rap/RanGAP domain-like 1	chr14q13.2		253959	---	---	---	---		MmugDNA.12858.1.S1_at_GARNL1
MmugDNA.12859.1.S1_at	207615_s_at	C16orf3	chromosome 16 open reading frame 3	chr16q24.3		750	---	---	---	---		MmugDNA.12859.1.S1_at_C16orf3
MmugDNA.12860.1.S1_at	230228_at	LOC284297	hypothetical LOC284297	chr19q13.42		284297	LOC701740	similar to deleted in malignant brain tumors 1 isoform b precursor	---		701740	MmugDNA.12860.1.S1_at_LOC284297
MmugDNA.12861.1.S1_at	235257_at	ODF3B	outer dense fiber of sperm tails 3B	chr22q13.33		440836	LOC720683	similar to sperm tail protein SHIPPO1	---		720683	MmugDNA.12861.1.S1_at_ODF3B
MmugDNA.1286.1.S1_at	209155_s_at	NT5C2	5'-nucleotidase, cytosolic II	chr10q24.32-q24.33		22978	---	---	---	---		MmugDNA.1286.1.S1_at_NT5C2
MmugDNA.12862.1.S1_at	228174_at	C9orf126	chromosome 9 open reading frame 126	chr9q33.3		286205	LOC714763	Similar to CG1998-PA	---		714763	MmugDNA.12862.1.S1_at_C9orf126
MmugDNA.12863.1.S1_at	212689_s_at	KDM3A	lysine (K)-specific demethylase 3A	chr2p11.2		55818	KDM3A	lysine (K)-specific demethylase 3A	---		697775	MmugDNA.12863.1.S1_at_KDM3A
MmugDNA.12864.1.S1_x_at	NA	NA	NA	NA	NA		NA	NA	NA	NA		MmugDNA.12864.1.S1_x_at_NA
MmugDNA.12865.1.S1_at	227701_at	C10orf118	chromosome 10 open reading frame 118	chr10q25.3		55088	LOC722518	similar to CTCL tumor antigen L14-2	---		722518	MmugDNA.12865.1.S1_at_C10orf118
MmugDNA.12866.1.S1_at	233983_at	TGM6	transglutaminase 6	chr20p13		343641	---	---	---	---		MmugDNA.12866.1.S1_at_TGM6
MmugDNA.12867.1.S1_at	222482_at	LOC100131851 /// LOC	hypothetical protein LOC100131851 /// hypothetical protein LOC100134497 /// similar to single strand	chr1p32.3 /// chr2q21.1 /// chr7p1		100131851 /// 1001344	LOC715114	similar to single-stranded DNA-binding protein isoform a	---		715114	MmugDNA.12867.1.S1_at_LOC1001318
MmugDNA.12868.1.S1_at	212103_at	KPNA6	karyopherin alpha 6 (importin alpha 7)	chr1p35.1-p34.3		23633	KPNA6	karyopherin alpha 6	---		706812	MmugDNA.12868.1.S1_at_KPNA6
MmugDNA.12869.1.S1_at	NA	NA	NA	NA	NA		NA	NA	NA	NA		MmugDNA.12869.1.S1_at_NA
MmugDNA.12870.1.S1_at	244589_at	---	---	---	---		---	---	---	---		MmugDNA.12870.1.S1_at_---
MmugDNA.12871.1.S1_at	244715_at	---	---	---	---		---	---	---	---		MmugDNA.12871.1.S1_at_---
MmugDNA.1287.1.S1_s_at	224916_at	TMEM173	transmembrane protein 173	chr5q31.2		340061	LOC694353	hypothetical protein LOC694353	---		694353	MmugDNA.1287.1.S1_s_at_TMEM173
MmugDNA.12872.1.S1_at	242774_at	SYNE2	spectrin repeat containing, nuclear envelope 2	chr14q23.2		23224	LOC706759	similar to spectrin repeat containing, nuclear envelope 2 isoform e	---		706759	MmugDNA.12872.1.S1_at_SYNE2
MmugDNA.12873.1.S1_at	227308_x_at	LTBP3	latent transforming growth factor beta binding protein 3	chr11q13.1		4054	---	---	---	---		MmugDNA.12873.1.S1_at_LTBP3
MmugDNA.12874.1.S1_at	215102_at	DPY19L1P1	dpy-19-like 1 pseudogene 1 (C. elegans)	chr7p14.3		100129460	---	---	---	---		MmugDNA.12874.1.S1_at_DPY19L1P1
MmugDNA.12875.1.S1_at	1560198_at	C14orf70	chromosome 14 open reading frame 70	chr14q32.2		283601	---	---	---	---		MmugDNA.12875.1.S1_at_C14orf70
MmugDNA.12876.1.S1_at	1563529_at	HYDIN2	hydrocephalus inducing homolog 2 (mouse)	chr1q21.1		790898	---	---	---	---		MmugDNA.12876.1.S1_at_HYDIN2
MmugDNA.12877.1.S1_at	1561719_at	---	---	---	---		---	---	---	---		MmugDNA.12877.1.S1_at_---
MmugDNA.12878.1.S1_at	227326_at	MXRA7	matrix-remodelling associated 7	chr17q25.1-q25.2		439921	LOC711598	similar to Transmembrane anchor protein 1	---		711598	MmugDNA.12878.1.S1_at_MXRA7
MmugDNA.12879.1.S1_at	1554294_s_at	TTBK2	tau tubulin kinase 2	chr15q15.2		146057	LOC712520	Similar to plakophilin 4 isoform b	---		712520	MmugDNA.12879.1.S1_at_TTBK2
MmugDNA.12880.1.S1_at	206436_at	MPPED1	metallophosphoesterase domain containing 1	chr22q13.31		758	LOC714104	Similar to START domain containing 13 isoform beta	---		714104	MmugDNA.12880.1.S1_at_MPPED1
MmugDNA.12881.1.S1_at	206932_at	CH25H	cholesterol 25-hydroxylase	chr10q23		9023	CH25H	cholesterol 25-hydroxylase	---		694532	MmugDNA.12881.1.S1_at_CH25H
MmugDNA.1288.1.S1_at	224929_at	TMEM173	transmembrane protein 173	chr5q31.2		340061	LOC694353	hypothetical protein LOC694353	---		694353	MmugDNA.1288.1.S1_at_TMEM173
MmugDNA.12882.1.S1_at	1564590_a_at	OFCC1	orofacial cleft 1 candidate 1	chr6p24.3		266553	---	---	---	---		MmugDNA.12882.1.S1_at_OFCC1
MmugDNA.12883.1.S1_at	1557042_at	---	---	---	---		---	---	---	---		MmugDNA.12883.1.S1_at_---
MmugDNA.12884.1.S1_at	231466_at	FAM71F1	family with sequence similarity 71, member F1	chr7q32.1		84691	---	---	---	---		MmugDNA.12884.1.S1_at_FAM71F1
MmugDNA.12885.1.S1_at	1558334_a_at	C22orf15	chromosome 22 open reading frame 15	chr22q11.23		150248	LOC698813	similar to Protein C22orf15 (N27C7-3 protein)	---		698813	MmugDNA.12885.1.S1_at_C22orf15
MmugDNA.12886.1.S1_at	217317_s_at	HERC2P2 /// HERC2P3	hect domain and RLD 2 pseudogene 2 /// hect domain and RLD 2 pseudogene 3 /// hect domain and	chr15q11.2 /// chr15q13.1		283755 /// 400322 /// 4	---	---	---	---		MmugDNA.12886.1.S1_at_HERC2P2 //

MmugDNA.12887.1.S1_at	209078_s_at	TXN2	thioredoxin 2	chr22q13.1	25828	LOC709917	similar to Thioredoxin, mitochondrial precursor (Mt-Trx) (MTRX) (Thioredoxin-2)	---	709917	MmugDNA.12887.1.S1_at_TXN2
MmugDNA.12888.1.S1_at	1556346_at	---	---	---	---	---	---	---	---	MmugDNA.12888.1.S1_at_---
MmugDNA.12889.1.S1_at	221907_at	TRMT61A	tRNA methyltransferase 61 homolog A (S. cerevisiae)	chr14q32.33	115708	TRMT61A	tRNA methyltransferase 61 homolog A (S. cerevisiae)	---	695566	MmugDNA.12889.1.S1_at_TRMT61A
MmugDNA.12890.1.S1_at	1554241_at	COCH	coagulation factor C homolog, cochlin (Limulus polyphemus)	chr14q12-q13	1690	LOC717114	similar to coagulation factor C homolog, cochlin precursor	---	717114	MmugDNA.12890.1.S1_at_COCH
MmugDNA.12891.1.S1_at	240048_at	STRC	stereocilin	chr15q15.3	161497	STRC	stereocilin	---	677720	MmugDNA.12891.1.S1_at_STRC
MmugDNA.1289.1.S1_at	216114_at	NCKIPSD	NCK interacting protein with SH3 domain	chr3p21	51517	---	---	---	---	MmugDNA.1289.1.S1_at_NCKIPSD
MmugDNA.12892.1.S1_at	214453_s_at	IFI44	interferon-induced protein 44	chr1p31.1	10561	IFI44	interferon-induced protein 44	---	707854	MmugDNA.12892.1.S1_at_IFI44
MmugDNA.12893.1.S1_at	227862_at	TRNP1	TMF1-regulated nuclear protein 1	chr1p36.11	388610	---	---	---	---	MmugDNA.12893.1.S1_at_TRNP1
MmugDNA.12894.1.S1_at	234987_at	---	---	---	---	LOC697228	Similar to HSPC171 protein	---	697228	MmugDNA.12894.1.S1_at_---
MmugDNA.12895.1.S1_s_at	221286_s_at	MGC29506	hypothetical protein MGC29506	chr5q23-q31	51237	LOC693572	similar to proapoptotic caspase adaptor protein	---	693572	MmugDNA.12895.1.S1_s_at_MGC2950
MmugDNA.12896.1.S1_at	212925_at	C19orf21	chromosome 19 open reading frame 21	chr19p13.3	126353	---	---	---	---	MmugDNA.12896.1.S1_at_C19orf21
MmugDNA.12897.1.S1_at	212890_at	SLC38A10	solute carrier family 38, member 10	chr17q25.3	124565	LOC713556	similar to CG30394-PB, isoform B	---	713556	MmugDNA.12897.1.S1_at_SLC38A10
MmugDNA.12898.1.S1_at	227952_at	---	---	---	---	---	---	---	---	MmugDNA.12898.1.S1_at_---
MmugDNA.12899.1.S1_at	227176_at	SLC2A13	solute carrier family 2 (facilitated glucose transporter), member 13	chr12q12	114134	LOC699362	Similar to ribosomal protein L9	---	699362	MmugDNA.12899.1.S1_at_SLC2A13
MmugDNA.12900.1.S1_at	213000_at	MORC3	MORC family CW-type zinc finger 3	chr21q22.13	23515	MORC3	MORC family CW-type zinc finger 3	---	695314	MmugDNA.12900.1.S1_at_MORC3
MmugDNA.12901.1.S1_at	1563364_at	---	---	---	---	---	---	---	---	MmugDNA.12901.1.S1_at_---
MmugDNA.1290.1.S1_at	200068_s_at	CANX	calnexin	chr5q35	821	---	---	---	---	MmugDNA.1290.1.S1_at_CANX
MmugDNA.12902.1.S1_at	232127_at	CLCN5	chloride channel 5	chrXp11.23	1184	CLCN5	chloride channel 5	---	694497	MmugDNA.12902.1.S1_at_CLCN5
MmugDNA.12903.1.S1_at	241198_s_at	C11orf70	chromosome 11 open reading frame 70	chr11q22.1	85016	LOC704382	hypothetical protein LOC704382	---	704382	MmugDNA.12903.1.S1_at_C11orf70
MmugDNA.12903.1.S1_s_at	NA	NA	NA	NA	NA	NA	NA	NA	NA	MmugDNA.12903.1.S1_s_at_NA
MmugDNA.12904.1.S1_at	228363_at	XIAP	X-linked inhibitor of apoptosis	chrXq25	331	---	---	---	---	MmugDNA.12904.1.S1_at_XIAP
MmugDNA.12904.1.S1_x_at	NA	NA	NA	NA	NA	NA	NA	NA	NA	MmugDNA.12904.1.S1_x_at_NA
MmugDNA.12905.1.S1_at	241291_at	---	---	---	---	---	---	---	---	MmugDNA.12905.1.S1_at_---
MmugDNA.12906.1.S1_at	205835_s_at	YTHDC2	YTH domain containing 2	chr5q22.2	64848	LOC694612	similar to YTH domain containing 2	---	694612	MmugDNA.12906.1.S1_at_YTHDC2
MmugDNA.12907.1.S1_at	214997_at	C9orf126	chromosome 9 open reading frame 126	chr9q33.3	286205	---	---	---	---	MmugDNA.12907.1.S1_at_C9orf126
MmugDNA.12908.1.S1_at	210341_at	MYT1	myelin transcription factor 1	chr20q13.33	4661	---	---	---	---	MmugDNA.12908.1.S1_at_MYT1
MmugDNA.12909.1.S1_at	235987_at	PRKXP1	protein kinase, X-linked, pseudogene 1	chr15q26.3	441733	PRKX	protein kinase, X-linked	---	706432	MmugDNA.12909.1.S1_at_PRKXP1
MmugDNA.12910.1.S1_at	NA	NA	NA	NA	NA	NA	NA	NA	NA	MmugDNA.12910.1.S1_at_NA
MmugDNA.12911.1.S1_at	227588_s_at	C7orf20	Chromosome 7 open reading frame 20	chr7p22.3	51608	---	---	---	---	MmugDNA.12911.1.S1_at_C7orf20
MmugDNA.1291.1.S1_at	217143_s_at	TRA@ /// TRD@	T cell receptor alpha locus /// T cell receptor delta locus	chr14q11.2	6955 /// 6964	LOC711031	Hypothetical protein LOC711031	---	711031	MmugDNA.1291.1.S1_at_TRA@ /// TR
MmugDNA.12912.1.S1_at	230183_at	EXT1	exostoses (multiple) 1	chr8q24.11-q24.13	2131	---	---	---	---	MmugDNA.12912.1.S1_at_EXT1
MmugDNA.12913.1.S1_at	236148_at	---	---	---	---	---	---	---	---	MmugDNA.12913.1.S1_at_---
MmugDNA.12914.1.S1_at	229464_at	MYEF2	myelin expression factor 2	chr15q21.1	50804	LOC716153	similar to myelin gene expression factor 2	---	716153	MmugDNA.12914.1.S1_at_MYEF2
MmugDNA.12915.1.S1_at	240962_at	---	---	---	---	---	---	---	---	MmugDNA.12915.1.S1_at_---
MmugDNA.12916.1.S1_at	212052_s_at	TBC1D9B	TBC1 domain family, member 9B (with GRAM domain)	chr5q35.3	23061	LOC703999	Similar to binder of Arl Two	---	703999	MmugDNA.12916.1.S1_at_TBC1D9B
MmugDNA.12917.1.S1_at	206653_at	POLR3G	polymerase (RNA) III (DNA directed) polypeptide G (32kD)	chr5q14.3	10622	LOC693918	Similar to phosphoinositide-3-kinase, class 3	---	693918	MmugDNA.12917.1.S1_at_POLR3G
MmugDNA.12919.1.S1_s_at	NA	NA	NA	NA	NA	NA	NA	NA	NA	MmugDNA.12919.1.S1_s_at_NA
MmugDNA.129.1.S1_s_at	217123_x_at	PMCHL1	pro-melanin-concentrating hormone-like 1	chr5p14.3	5369	---	---	---	---	MmugDNA.129.1.S1_s_at_PMCHL1
MmugDNA.12920.1.S1_at	1560543_at	---	---	---	---	---	---	---	---	MmugDNA.12920.1.S1_at_---
MmugDNA.12921.1.S1_at	1558431_at	FLJ36208	hypothetical protein FLJ36208	chr16p13.3	283948	---	---	---	---	MmugDNA.12921.1.S1_at_FLJ36208
MmugDNA.1292.1.S1_at	235353_at	KIAA0746	KIAA0746 protein	chr4p15.2	23231	---	---	---	---	MmugDNA.1292.1.S1_at_KIAA0746
MmugDNA.12922.1.S1_at	244234_at	FAM62A	Family with sequence similarity 62 (C2 domain containing), member A	chr12q13.2	23344	---	---	---	---	MmugDNA.12922.1.S1_at_FAM62A
MmugDNA.12923.1.S1_at	212694_s_at	PCCB	propionyl Coenzyme A carboxylase, beta polypeptide	chr3q21-q22	5096	LOC700636	Similar to myosin, heavy polypeptide 11, smooth muscle	---	700636	MmugDNA.12923.1.S1_at_PCCB
MmugDNA.12924.1.S1_at	230070_at	CNIH2	cornichon homolog 2 (Drosophila)	chr11q13.1	254263	LOC721848	similar to cornichon homolog 2	---	721848	MmugDNA.12924.1.S1_at_CNIH2
MmugDNA.12925.1.S1_at	1561662_at	---	---	---	---	---	---	---	---	MmugDNA.12925.1.S1_at_---
MmugDNA.12926.1.S1_at	1556304_s_at	hCG_2039027	hypothetical LOC100271722	---	100271722	---	---	---	---	MmugDNA.12926.1.S1_at_hCG_20390
MmugDNA.12927.1.S1_at	228919_at	---	---	---	---	---	---	---	---	MmugDNA.12927.1.S1_at_---
MmugDNA.12928.1.S1_at	1555163_at	---	---	---	---	---	---	---	---	MmugDNA.12928.1.S1_at_---
MmugDNA.12929.1.S1_at	222650_s_at	SLC2A4RG	SLC2A4 regulator	chr20q13.33	56731	LOC695278	similar to SLC2A4 regulator	---	695278	MmugDNA.12929.1.S1_at_SLC2A4RG
MmugDNA.12930.1.S1_at	1560455_at	LOC339166	hypothetical protein LOC339166	chr17p13.3-p13.2	339166	---	---	---	---	MmugDNA.12930.1.S1_at_LOC339166
MmugDNA.12931.1.S1_at	239818_x_at	TRIB1	tribbles homolog 1 (Drosophila)	chr8q24.13	10221	LOC693459	similar to G-protein-coupled receptor induced protein	---	693459	MmugDNA.12931.1.S1_at_TRIB1
MmugDNA.12932.1.S1_at	1566973_at	---	---	---	---	---	---	---	---	MmugDNA.12932.1.S1_at_---
MmugDNA.12933.1.S1_at	242296_x_at	---	---	---	---	---	---	---	---	MmugDNA.12933.1.S1_at_---
MmugDNA.12934.1.S1_at	236261_at	---	---	---	---	---	---	---	---	MmugDNA.12934.1.S1_at_---
MmugDNA.12935.1.S1_at	1560987_a_at	---	---	---	---	---	---	---	---	MmugDNA.12935.1.S1_at_---
MmugDNA.12936.1.S1_at	1570517_at	FHL5	four and a half LIM domains 5	chr6q16.1-q16.3	9457	---	---	---	---	MmugDNA.12936.1.S1_at_FHL5
MmugDNA.12937.1.S1_at	232188_at	AKAP13	A kinase (PRKA) anchor protein 13	chr15q24-q25	11214	---	---	---	---	MmugDNA.12937.1.S1_at_AKAP13
MmugDNA.12938.1.S1_at	244090_at	---	---	---	---	---	---	---	---	MmugDNA.12938.1.S1_at_---
MmugDNA.12939.1.S1_at	NA	NA	NA	NA	NA	NA	NA	NA	NA	MmugDNA.12939.1.S1_at_NA
MmugDNA.12940.1.S1_at	203157_s_at	GLS	glutaminase	chr2q32-q34	2744	PCMT1	Protein-L-isoaspartate (D-aspartate) O-methyltransferase	---	695893	MmugDNA.12940.1.S1_at_GLS
MmugDNA.12941.1.S1_at	235396_at	C22orf25	chromosome 22 open reading frame 25	chr22q11.21	128989	LOC719416	similar to Ser/Thr-rich protein T10 in DGCR region	---	719416	MmugDNA.12941.1.S1_at_C22orf25
MmugDNA.1294.1.S1_s_at	1431_at	CYP2E1	cytochrome P450, family 2, subfamily E, polypeptide 1	chr10q24.3-qter	1571	CYP2E1	cytochrome P450, family 2, subfamily E, polypeptide 1	---	678688	MmugDNA.1294.1.S1_s_at_CYP2E1
MmugDNA.12942.1.S1_at	243368_at	---	---	---	---	---	---	---	---	MmugDNA.12942.1.S1_at_---
MmugDNA.12943.1.S1_at	213667_at	SRCAP	Snf2-related CREBBP activator protein	chr16p11.2	10847	LOC711500	hypothetical protein LOC711500	---	711500	MmugDNA.12943.1.S1_at_SRCAP
MmugDNA.12944.1.S1_at	223764_x_at	NIPSNAP3B	nipsnap homolog 3B (C. elegans)	chr9q31.1	55335	NIPSNAP3B	nipsnap homolog 3B (C. elegans)	---	712487	MmugDNA.12944.1.S1_at_NIPSNAP3B
MmugDNA.12945.1.S1_at	237300_at	PSMA3	Proteasome (prosome, macropain) subunit, alpha type, 3	chr14q23	5684	---	---	---	---	MmugDNA.12945.1.S1_at_PSMA3
MmugDNA.12946.1.S1_at	239582_at	PML	promyelocytic leukemia	chr15q22	5371	---	---	---	---	MmugDNA.12946.1.S1_at_PML
MmugDNA.12947.1.S1_at	241666_at	C3orf23	chromosome 3 open reading frame 23	chr3p21.33-p21.32	285343	LOC715979	similar to CG13295-PA	---	715979	MmugDNA.12947.1.S1_at_C3orf23
MmugDNA.12948.1.S1_at	201259_s_at	SYPL1	synaptophysin-like 1	chr7q22.2	6856	SYPL1	synaptophysin-like 1	---	698350	MmugDNA.12948.1.S1_at_SYPL1
MmugDNA.12949.1.S1_at	239033_at	---	---	---	---	---	---	---	---	MmugDNA.12949.1.S1_at_---
MmugDNA.12950.1.S1_at	226931_at	TMTC1	transmembrane and tetratricopeptide repeat containing 1	chr12p11.22	83857	KRT18	Similar to Keratin, type I cytoskeletal 18 (Cytokeratin-18) (CK-18) (Keratin-18) (K18)	---	719942	MmugDNA.12950.1.S1_at_TMTC1
MmugDNA.12951.1.S1_at	229000_at	ZNF77	zinc finger protein 77	chr19p13.3	58492	LOC712142	similar to zinc finger protein 77	---	712142	MmugDNA.12951.1.S1_at_ZNF77
MmugDNA.1295.1.S1_at	203230_at	DVL1 /// LOC642469	dishevelled, dsh homolog 1 (Drosophila) /// hypothetical LOC642469	chr1p36	1855 /// 642469	---	---	---	---	MmugDNA.1295.1.S1_at_DVL1 /// LOC
MmugDNA.12952.1.S1_at	222075_s_at	OAZ3	ornithine decarboxylase antizyme 3	chr1q21.3	51686	OAZ3	ornithine decarboxylase antizyme 3	---	711313	MmugDNA.12952.1.S1_at_OAZ3
MmugDNA.12953.1.S1_at	227604_at	LOC151146	hypothetical protein LOC151146	chr2q14.2	151146	---	---	---	---	MmugDNA.12953.1.S1_at_LOC151146
MmugDNA.12954.1.S1_at	226281_at	DNER	delta/notch-like EGF repeat containing	chr2q36.3	92737	LOC710384	similar to delta-notch-like EGF repeat-containing transmembrane	---	710384	MmugDNA.12954.1.S1_at_DNER
MmugDNA.12956.1.S1_at	242510_at	---	---	---	---	---	---	---	---	MmugDNA.12956.1.S1_at_---
MmugDNA.12957.1.S1_at	1555145_at	C1orf96	chromosome 1 open reading frame 96	chr1q42.13	126731	---	---	---	---	MmugDNA.12957.1.S1_at_C1orf96
MmugDNA.12958.1.S1_at	226605_at	DGKQ	diacylglycerol kinase, theta 110kDa	chr4p16.3	1609	---	---	---	---	MmugDNA.12958.1.S1_at_DGKQ
MmugDNA.12959.1.S1_at	216545_at	---	---	---	---	---	---	---	---	MmugDNA.12959.1.S1_at_---
MmugDNA.12960.1.S1_at	233791_at	LOC644213	Glyceraldehyde-3-phosphate dehydrogenase pseudogene	chr1p12	644213	---	---	---	---	MmugDNA.12960.1.S1_at_LOC644213
MmugDNA.12961.1.S1_at	1560208_at	---	---	---	---	---	---	---	---	MmugDNA.12961.1.S1_at_---
MmugDNA.1296.1.S1_at	210833_at	PTGER3	prostaglandin E receptor 3 (subtype EP3)	chr1p31.2	5733	---	---	---	---	MmugDNA.1296.1.S1_at_PTGER3
MmugDNA.12962.1.S1_at	211255_x_at	DEDD	death effector domain containing	chr1q23.3	9191	LOC719875	similar to death effector domain-containing protein	---	719875	MmugDNA.12962.1.S1_at_DEDD
MmugDNA.12962.1.S1_s_at	NA	NA	NA	NA	NA	NA	NA	NA	NA	MmugDNA.12962.1.S1_s_at_NA
MmugDNA.12963.1.S1_at	1564841_at	---	---	---	---	---	---	---	---	MmugDNA.12963.1.S1_at_---
MmugDNA.12964.1.S1_at	235557_at	LOC150763	hypothetical protein LOC150763	chr2q11.2	150763	LOC707076	similar to glycerol-3-phosphate acyltransferase, mitochondrial	---	707076	MmugDNA.12964.1.S1_at_LOC150763
MmugDNA.12965.1.S1_at	225504_at	---	---	---	---	---	---	---	---	MmugDNA.12965.1.S1_at_---
MmugDNA.12966.1.S1_at	234103_at	KCNT2	Potassium channel, subfamily T, member 2	chr1q31.3	343450	---	---	---	---	MmugDNA.12966.1.S1_at_KCNT2
MmugDNA.12967.1.S1_at	1570289_at	LOC646736	similar to Alu subfamily SX sequence contamination warning entry	chr2q36.3	646736	---	---	---	---	MmugDNA.12967.1.S1_at_LOC646736
MmugDNA.12968.1.S1_at	224824_at	FAM36A	family with sequence similarity 36, member A	chr1q44	116228	LOC704298	similar to family with sequence similarity 36, member A	---	704298	MmugDNA.12968.1.S1_at_FAM36A
MmugDNA.12969.1.S1_at	227543_at	RNASEH2C	ribonuclease H2, subunit C	chr11q13.1	84153	---	---	---	---	MmugDNA.12969.1.S1_at_RNASEH2C
MmugDNA.12970.1.S1_at	224820_at	FAM36A	family with sequence similarity 36, member A	chr1q44	116228	LOC704298	similar to family with sequence similarity 36, member A	---	704298	MmugDNA.12970.1.S1_at_FAM36A
MmugDNA.12971.1.S1_at	223033_s_at	SCYL1	SCY1-like 1 (S. cerevisiae)	chr11q13	57410	LOC716345	similar to SCY1-like 1	---	716345	MmugDNA.12971.1.S1_at_SCYL1
MmugDNA.1297.1.S1_at	222729_at	FBXW7	F-box and WD repeat domain containing 7	chr4q31.3	55294	FBXW7	F-box and WD repeat domain containing 7	---	695101	MmugDNA.1297.1.S1_at_FBXW7
MmugDNA.12972.1.S1_at	1552875_a_at	CD200R1	CD200 receptor 1	chr3q13.2	131450	LOC708734	similar to CD200 receptor 1 isoform a	---	708734	MmugDNA.12972.1.S1_at_CD200R1
MmugDNA.12973.1.S1_at	202712_s_at	CKMT1A /// CKMT1B	creatine kinase, mitochondrial 1A /// creatine kinase, mitochondrial 1B	chr15q15	1159 /// 548596	LOC699802	Similar to melanoma antigen family C, 1	---	699802	MmugDNA.12973.1.S1_at_CKMT1A ///
MmugDNA.12974.1.S1_at	244581_at	ZBTB20	Zinc finger and BTB domain containing 20	chr3q13.2	26137	---	---	---	---	MmugDNA.12974.1.S1_at_ZBTB20
MmugDNA.12975.1.S1_at	205149_s_at	CLCN4	chloride channel 4	chrXp22.3	1183	---	---	---	---	MmugDNA.12975.1.S1_at_CLCN4
MmugDNA.12976.1.S1_at	1569785_at	---	---	---	---	---	---	---	---	MmugDNA.12976.1.S1_at_---
MmugDNA.12977.1.S1_at	207787_at	KRT33B	keratin 33B	chr17q12-q21	3884	LOC705939	similar to type I hair keratin 3B	---	705939	MmugDNA.12977.1.S1_at_KRT33B
MmugDNA.12978.1.S1_at	1553694_a_at	PIK3C2A	phosphoinositide-3-kinase, class 2, alpha polypeptide	chr11p15.5-p14	5286	LOC701569	Similar to retinoblastoma-binding protein 6 isoform 2	---	701569	MmugDNA.12978.1.S1_at_PIK3C2A
MmugDNA.12979.1.S1_s_at	223803_s_at	ZCCHC10	zinc finger, CCHC domain containing 10	chr5q31.1	54819	CDC37	Cell division cycle 37 homolog (S. cerevisiae)	---	715390	MmugDNA.12979.1.S1_s_at_ZCCHC10
MmugDNA.12980.1.S1_s_at	211526_s_at	RTEL1	regulator of telomere elongation helicase 1	chr20q13.3	51750	LOC574363 /// LOC7055	acidic ribosomal phosphoprotein PO /// similar to acidic ribosomal phosphoprotein P0 /// similar to 60S	---	574363 /// 705501 /// 718	MmugDNA.12980.1.S1_s_at_RTEL1
MmugDNA.12980.1.S1_x_at	201033_x_at	RPLP0	ribosomal protein, large, P0	chr12q24.2	6175	LOC718979	similar to 60S acidic ribosomal protein P0 (L10E)	---	718979	MmugDNA.12980.1.S1_x_at_RPLP0
MmugDNA.12981.1.S1_at	237856_at	RAP1GDS1	RAP1, GTP-GDP dissociation stimulator 1	chr4q23-q25	5910	---	---	---	---	MmugDNA.12981.1.S1_at_RAP1GDS1
MmugDNA.1298.1.S1_at	228487_s_at	---	---	---	---	---	---	---	---	MmugDNA.1298.1.S1_at_---
MmugDNA.12982.1.S1_at	211376_s_at	NSMCE4A	non-SMC element 4 homolog A (S. cerevisiae)	chr10q26.13	54780	NSMCE4A	non-SMC element 4 homolog A (S. cerevisiae)	---	705375	MmugDNA.12982.1.S1_at_NSMCE4A

MmugDNA.12983.1.S1_at	230032_at	OSGEPL1	O-sialoglycoprotein endopeptidase-like 1	chr2q32.2	64172	LOC709747	similar to O-sialoglycoprotein endopeptidase-like 1	---	709747	MmugDNA.12983.1.S1_at_OSGEPL1
MmugDNA.12984.1.S1_at	201363_s_at	IVNS1ABP	influenza virus NS1A binding protein	chr1q25.1-q31.1	10625	---	---	---	---	MmugDNA.12984.1.S1_at_IVNS1ABP
MmugDNA.12985.1.S1_at	1561849_at	PKD1L2	polycystic kidney disease 1-like 2	chr16q23.2	114780	---	---	---	---	MmugDNA.12985.1.S1_at_PKD1L2
MmugDNA.12986.1.S1_at	243604_at	---	---	---	---	---	---	---	---	MmugDNA.12986.1.S1_at_---
MmugDNA.12987.1.S1_at	1553954_at	ALG14	asparagine-linked glycosylation 14 homolog (S. cerevisiae)	chr1p21.3	199857	LOC709630	similar to asparagine-linked glycosylation 14 homolog	---	709630	MmugDNA.12987.1.S1_at_ALG14
MmugDNA.12988.1.S1_s_at	241712_at	CBY3	chibby homolog 3 (Drosophila)	chr5q35.3	646019	LOC703598	similar to high-mobility group box 3	---	703598	MmugDNA.12988.1.S1_s_at_CBY3
MmugDNA.12989.1.A1_at	NA	NA	NA	NA	NA	NA	NA	NA	NA	MmugDNA.12989.1.A1_at_NA
MmugDNA.12989.1.S1_at	1559018_at	PTPRE	protein tyrosine phosphatase, receptor type, E	chr10q26	5791	---	---	---	---	MmugDNA.12989.1.S1_at_PTPRE
MmugDNA.12990.1.S1_at	1562167_a_at	DEFB122	defensin, beta 122 (pseudogene)	chr20q11.1	245935	DEFB122	beta defensin 122	---	619506	MmugDNA.12990.1.S1_at_DEFB122
MmugDNA.12991.1.S1_at	243933_at	---	---	---	---	---	---	---	---	MmugDNA.12991.1.S1_at_---
MmugDNA.1299.1.S1_at	226935_s_at	CLPTM1L	CLPTM1-like	chr5pter-p15.3	81037	LOC709968	similar to cisplatin resistance related protein CRR9p	---	709968	MmugDNA.1299.1.S1_at_CLPTM1L
MmugDNA.12992.1.S1_at	241645_at	---	---	---	---	---	---	---	---	MmugDNA.12992.1.S1_at_---
MmugDNA.12993.1.S1_at	226118_at	CENPO	centromere protein O	chr2p23.3	79172	LOC693599	hypothetical protein LOC693599	---	693599	MmugDNA.12993.1.S1_at_CENPO
MmugDNA.12994.1.S1_at	223056_s_at	XPO5	exportin 5	chr6p21.1	57510	LOC700664	hypothetical protein LOC700664	---	700664	MmugDNA.12994.1.S1_at_XPO5
MmugDNA.12995.1.S1_at	238432_at	FLJ35776	hypothetical LOC649446	chr18p11.31	649446	---	---	---	---	MmugDNA.12995.1.S1_at_FLJ35776
MmugDNA.12996.1.S1_at	206397_x_at	GDF1 /// LASS1	growth differentiation factor 1 /// LAG1 homolog, ceramide synthase 1	chr19p12	10715 /// 2657	LOC720298	similar to longevity assurance gene 1 isoform 1	---	720298	MmugDNA.12996.1.S1_at_GDF1 /// LAS
MmugDNA.12997.1.S1_at	209465_x_at	PTN	pleiotrophin	chr7q33-q34	5764	LOC709496	similar to Pleiotrophin precursor (PTN) (Heparin-binding growth-associated molecule) (HB-GAM) (He	---	709496	MmugDNA.12997.1.S1_at_PTN
MmugDNA.12998.1.S1_at	222378_at	---	---	---	---	---	---	---	---	MmugDNA.12998.1.S1_at_---
MmugDNA.12999.1.S1_at	232201_at	NKD2	naked cuticle homolog 2 (Drosophila)	chr5p15.3	85409	LOC709500	similar to naked cuticle homolog 2	---	709500	MmugDNA.12999.1.S1_at_NKD2
MmugDNA.13000.1.S1_at	209145_s_at	CBFA2T2	core-binding factor, runt domain, alpha subunit 2; translocated to, 2	chr20q11	9139	---	---	---	---	MmugDNA.13000.1.S1_at_CBFA2T2
MmugDNA.13001.1.S1_at	1558612_a_at	ATP1A4	ATPase, Na+/K+ transporting, alpha 4 polypeptide	chr1q21-q23	480	ATP1A4	ATPase, Na+/K+ transporting, alpha 4 polypeptide	---	719623	MmugDNA.13001.1.S1_at_ATP1A4
MmugDNA.1300.1.S1_at	229274_at	GNAS	GNAS complex locus	chr20q13.3	2778	---	---	---	---	MmugDNA.1300.1.S1_at_GNAS
MmugDNA.13002.1.S1_at	237334_at	---	---	---	---	---	---	---	---	MmugDNA.13002.1.S1_at_---
MmugDNA.13003.1.S1_at	229546_at	LOC653602	hypothetical LOC653602	chr2p24.3	653602	---	---	---	---	MmugDNA.13003.1.S1_at_LOC653602
MmugDNA.13004.1.S1_at	215046_at	C2orf67	chromosome 2 open reading frame 67	chr2q34	151050	LOC713138	similar to CG4699-PA, isoform A	---	713138	MmugDNA.13004.1.S1_at_C2orf67
MmugDNA.13005.1.S1_at	242688_at	---	---	---	---	---	---	---	---	MmugDNA.13005.1.S1_at_---
MmugDNA.13006.1.S1_at	1565699_at	HECTD2	HECT domain containing 2	chr10q23.32	143279	---	---	---	---	MmugDNA.13006.1.S1_at_HECTD2
MmugDNA.13007.1.S1_at	1560934_at	LOC284669	hypothetical protein LOC284669	chr1q32.3	284669	---	---	---	---	MmugDNA.13007.1.S1_at_LOC284669
MmugDNA.13008.1.S1_at	240008_at	---	---	---	---	---	---	---	---	MmugDNA.13008.1.S1_at_---
MmugDNA.13010.1.S1_at	202429_s_at	PPP3CA	protein phosphatase 3 (formerly 2B), catalytic subunit, alpha isoform	chr4q21-q24	5530	HR	Hairless	---	574164	MmugDNA.13010.1.S1_at_PPP3CA
MmugDNA.13011.1.S1_at	219831_at	CDKL3	cyclin-dependent kinase-like 3	chr5q31	51265	CDKL3	cyclin-dependent kinase-like 3	---	710586	MmugDNA.13011.1.S1_at_CDKL3
MmugDNA.1301.1.S1_at	214493_s_at	INADL	InaD-like (Drosophila)	chr1p31.3	10207	LOC694408	similar to InaD-like protein isoform 1	---	694408	MmugDNA.1301.1.S1_at_INADL
MmugDNA.13012.1.S1_at	233560_x_at	MCM8	minichromosome maintenance complex component 8	chr20p12.3	84515	LOC718247	similar to minichromosome maintenance protein 8 isoform 2	---	718247	MmugDNA.13012.1.S1_at_MCM8
MmugDNA.13013.1.S1_at	239271_at	SMAD2	SMAD family member 2	chr18q21.1	4087	---	---	---	---	MmugDNA.13013.1.S1_at_SMAD2
MmugDNA.13014.1.S1_at	216344_at	NPHP4	nephronophthisis 4	chr1p36.22	261734	---	---	---	---	MmugDNA.13014.1.S1_at_NPHP4
MmugDNA.13015.1.S1_at	228674_s_at	EML4	echinoderm microtubule associated protein like 4	chr2p22-p21	27436	EML4	echinoderm microtubule associated protein like 4	---	715091	MmugDNA.13015.1.S1_at_EML4
MmugDNA.13015.1.S1_s_at	228673_s_at	EML4	Echinoderm microtubule associated protein like 4	chr2p22-p21	27436	EML4	echinoderm microtubule associated protein like 4	---	715091	MmugDNA.13015.1.S1_s_at_EML4
MmugDNA.13016.1.S1_at	210315_at	SYN2	synapsin II	chr3p25	6854	LOC695412	similar to synapsin II isoform IIa	---	695412	MmugDNA.13016.1.S1_at_SYN2
MmugDNA.13017.1.S1_at	1553852_at	VPS13B	vacuolar protein sorting 13 homolog B (yeast)	chr8q22.2	157680	VPS13B	vacuolar protein sorting 13 homolog B (yeast)	---	704477	MmugDNA.13017.1.S1_at_VPS13B
MmugDNA.13018.1.S1_at	217237_at	---	---	---	---	LOC698207	Similar to 40S ribosomal protein S26	---	698207	MmugDNA.13018.1.S1_at_---
MmugDNA.13019.1.S1_at	237477_at	PDCL2	phosducin-like 2	chr4q12	132954	PDCL2	phosducin-like 2	---	696014	MmugDNA.13019.1.S1_at_PDCL2
MmugDNA.130.1.S1_s_at	220019_s_at	ZNF224	zinc finger protein 224	chr19q13.2	7767	ZNF224	zinc finger protein 224	---	713143	MmugDNA.130.1.S1_s_at_ZNF224
MmugDNA.13020.1.S1_at	1554155_at	MCPH1	microcephalin 1	chr8p23.1	79648	---	---	---	---	MmugDNA.13020.1.S1_at_MCPH1
MmugDNA.13021.1.S1_at	210436_at	CCT8	chaperonin containing TCP1, subunit 8 (theta)	chr21q22.11	10694	---	---	---	---	MmugDNA.13021.1.S1_at_CCT8
MmugDNA.1302.1.S1_at	224665_at	C10orf104	chromosome 10 open reading frame 104	chr10q22.1	119504	LOC709147	hypothetical protein LOC709147	---	709147	MmugDNA.1302.1.S1_at_C10orf104
MmugDNA.13022.1.S1_at	237708_at	---	---	---	---	---	---	---	---	MmugDNA.13022.1.S1_at_---
MmugDNA.13023.1.S1_at	220496_at	CLEC1B	C-type lectin domain family 1, member B	chr12p13.2	51266	LOC717404	similar to C-type lectin domain family 1, member B	---	717404	MmugDNA.13023.1.S1_at_CLEC1B
MmugDNA.13024.1.S1_at	1554985_at	ZNF396	zinc finger protein 396	chr18q12	252884	---	---	---	---	MmugDNA.13024.1.S1_at_ZNF396
MmugDNA.13025.1.S1_at	NA	NA	NA	NA	NA	NA	NA	NA	NA	MmugDNA.13025.1.S1_at_NA
MmugDNA.13026.1.S1_at	1564446_at	LOC284930	hypothetical protein LOC284930	chr22q13.32	284930	---	---	---	---	MmugDNA.13026.1.S1_at_LOC284930
MmugDNA.13027.1.S1_at	1561423_at	LOC642924	hypothetical protein LOC642924	chr8q22.1	642924	---	---	---	---	MmugDNA.13027.1.S1_at_LOC642924
MmugDNA.13028.1.S1_at	1558378_a_at	AHNAK2	AHNAK nucleoprotein 2	chr14q32.33	113146	LOC707626	similar to periaxin	---	707626	MmugDNA.13028.1.S1_at_AHNAK2
MmugDNA.13029.1.S1_at	227450_at	ERP27	endoplasmic reticulum protein 27	chr12p12.3	121506	ERP27	endoplasmic reticulum protein 27	---	700186	MmugDNA.13029.1.S1_at_ERP27
MmugDNA.13030.1.S1_at	1553351_at	OTUD7A	OTU domain containing 7A	chr15q13.3	161725	---	---	---	---	MmugDNA.13030.1.S1_at_OTUD7A
MmugDNA.13031.1.S1_at	240546_at	LOC389043	hypothetical protein LOC389043	chr2q21.1	389043	---	---	---	---	MmugDNA.13031.1.S1_at_LOC389043
MmugDNA.1303.1.S1_at	NA	NA	NA	NA	NA	NA	NA	NA	NA	MmugDNA.1303.1.S1_at_NA
MmugDNA.1303.1.S1_s_at	NA	NA	NA	NA	NA	NA	NA	NA	NA	MmugDNA.1303.1.S1_s_at_NA
MmugDNA.13032.1.S1_at	207385_at	TFDP3	transcription factor Dp family, member 3	chrXq26.2	51270	TFDP1	transcription factor Dp-1	---	706557	MmugDNA.13032.1.S1_at_TFDP3
MmugDNA.13033.1.S1_at	204840_s_at	EEA1	early endosome antigen 1	chr12q22	8411	---	---	---	---	MmugDNA.13033.1.S1_at_EEA1
MmugDNA.13034.1.S1_at	222985_at	YWHAG	tyrosine 3-monooxygenase/tryptophan 5-monooxygenase activation protein, gamma polypeptide	chr7q11.23	7532	LOC722834	Similar to 60S ribosomal protein L21	---	722834	MmugDNA.13034.1.S1_at_YWHAG
MmugDNA.13035.1.S1_s_at	213702_x_at	ASAH1	N-acylsphingosine amidohydrolase (acid ceramidase) 1	chr8p22-p21.3	427	ASAH1	N-acylsphingosine amidohydrolase (acid ceramidase) 1	---	703699	MmugDNA.13035.1.S1_s_at_ASAH1
MmugDNA.13037.1.S1_at	227424_x_at	C21orf119	chromosome 21 open reading frame 119	chr21q22.11	84996	---	---	---	---	MmugDNA.13037.1.S1_at_C21orf119
MmugDNA.13038.1.S1_at	240569_at	---	---	---	---	---	---	---	---	MmugDNA.13038.1.S1_at_---
MmugDNA.13039.1.S1_at	222494_at	FOXN3	forkhead box N3	chr14q31.3	1112	FOXN3	forkhead box N3	---	694756	MmugDNA.13039.1.S1_at_FOXN3
MmugDNA.13040.1.S1_at	209720_s_at	SERPINB3	serpin peptidase inhibitor, clade B (ovalbumin), member 3	chr18q21.3	6317	SERPINB4	serpin peptidase inhibitor, clade B (ovalbumin), member 4	---	701189	MmugDNA.13040.1.S1_at_SERPINB3
MmugDNA.13041.1.S1_s_at	215382_x_at	TPSAB1	tryptase alpha/beta 1	chr16p13.3	7177	TPSB2	tryptase beta 2	---	699599	MmugDNA.13041.1.S1_s_at_TPSAB1
MmugDNA.1304.1.S1_at	224664_at	C10orf104	chromosome 10 open reading frame 104	chr10q22.1	119504	LOC709147	hypothetical protein LOC709147	---	709147	MmugDNA.1304.1.S1_at_C10orf104
MmugDNA.13042.1.S1_at	230465_at	HS2ST1	heparan sulfate 2-O-sulfotransferase 1	chr1p31.1-p22.1	9653	HS2ST1	heparan sulfate 2-O-sulfotransferase 1	---	712540	MmugDNA.13042.1.S1_at_HS2ST1
MmugDNA.13043.1.S1_at	210758_at	PSIP1	PC4 and SFRS1 interacting protein 1	chr9p22.3	11168	---	---	---	---	MmugDNA.13043.1.S1_at_PSIP1
MmugDNA.13044.1.S1_at	209065_at	UQCRB	ubiquinol-cytochrome c reductase binding protein	chr8q22	7381	UQCRB	ubiquinol-cytochrome c reductase binding protein	---	701223	MmugDNA.13044.1.S1_at_UQCRB
MmugDNA.13045.1.S1_at	240671_at	---	---	---	---	---	---	---	---	MmugDNA.13045.1.S1_at_---
MmugDNA.13046.1.S1_at	222016_s_at	ZNF323	zinc finger protein 323	chr6p21.31|6p22.3-p22.1	64288	ZSCAN12	zinc finger and SCAN domain containing 12	---	707473	MmugDNA.13046.1.S1_at_ZNF323
MmugDNA.13047.1.S1_at	231301_at	NUP54	nucleoporin 54kDa	chr4q21.1	53371	---	---	---	---	MmugDNA.13047.1.S1_at_NUP54
MmugDNA.13048.1.S1_at	241445_at	---	---	---	---	---	---	---	---	MmugDNA.13048.1.S1_at_---
MmugDNA.13049.1.S1_at	220470_at	BET1L	blocked early in transport 1 homolog (S. cerevisiae)-like	chr11p15.5	51272	BET1L	blocked early in transport 1 homolog (S. cerevisiae)-like	---	696599	MmugDNA.13049.1.S1_at_BET1L
MmugDNA.13050.1.S1_at	240575_at	---	---	---	---	---	---	---	---	MmugDNA.13050.1.S1_at_---
MmugDNA.13051.1.S1_at	240466_at	---	---	---	---	---	---	---	---	MmugDNA.13051.1.S1_at_---
MmugDNA.1305.1.S1_x_at	NA	NA	NA	NA	NA	NA	NA	NA	NA	MmugDNA.1305.1.S1_x_at_NA
MmugDNA.13052.1.S1_at	226459_at	PIK3AP1	phosphoinositide-3-kinase adaptor protein 1	chr10q24.1	118788	PIK3AP1	phosphoinositide-3-kinase adaptor protein 1	---	705017	MmugDNA.13052.1.S1_at_PIK3AP1
MmugDNA.13053.1.S1_at	228476_at	KIAA1407	KIAA1407	chr3q13.31	57577	LOC714466	Similar to HLA class I histocompatibility antigen, Cw-15 alpha chain precursor (MHC class I antigen C	---	714466	MmugDNA.13053.1.S1_at_KIAA1407
MmugDNA.13054.1.S1_at	230950_at	---	---	---	---	---	---	---	---	MmugDNA.13054.1.S1_at_---
MmugDNA.13055.1.S1_at	241252_at	ESCO2	establishment of cohesion 1 homolog 2 (S. cerevisiae)	chr8p21.1	157570	---	---	---	---	MmugDNA.13055.1.S1_at_ESCO2
MmugDNA.13056.1.S1_at	219817_at	C12orf47	chromosome 12 open reading frame 47	chr12q24.12	51275	LOC721887	Hypothetical protein LOC721887	---	721887	MmugDNA.13056.1.S1_at_C12orf47
MmugDNA.13057.1.S1_at	1552555_at	PRSS36	protease, serine, 36	chr16p11.2	146547	LOC714626	similar to protease, serine, 36	---	714626	MmugDNA.13057.1.S1_at_PRSS36
MmugDNA.13058.1.S1_at	216452_at	TRPM3	transient receptor potential cation channel, subfamily M, member 3	chr9q21.11-q21.12	80036	TRPM3	transient receptor potential cation channel, subfamily M, member 3	---	702898	MmugDNA.13058.1.S1_at_TRPM3
MmugDNA.13059.1.S1_at	1569777_a_at	ZPLD1	zona pellucida-like domain containing 1	chr3q12.3	131368	ZPLD1	zona pellucida-like domain containing 1	---	703200	MmugDNA.13059.1.S1_at_ZPLD1
MmugDNA.13060.1.S1_at	238524_at	---	---	---	---	DENND2D	DENN/MADD domain containing 2D	---	702568	MmugDNA.13060.1.S1_at_---
MmugDNA.13061.1.S1_at	227347_x_at	HES4	hairy and enhancer of split 4 (Drosophila)	chr1p36.33	57801	HES4	hairy and enhancer of split 4 (Drosophila)	---	698434	MmugDNA.13061.1.S1_at_HES4
MmugDNA.1306.1.S1_s_at	233868_x_at	ADAM33	ADAM metallopeptidase domain 33	chr20p13	80332	---	---	---	---	MmugDNA.1306.1.S1_s_at_ADAM33
MmugDNA.13062.1.S1_at	222783_s_at	SMOC1	SPARC related modular calcium binding 1	chr14q24.2	64093	LOC712994	similar to secreted modular calcium-binding protein 1 isoform 2	---	712994	MmugDNA.13062.1.S1_at_SMOC1
MmugDNA.13063.1.S1_at	242390_at	---	---	---	---	---	---	---	---	MmugDNA.13063.1.S1_at_---
MmugDNA.13064.1.S1_at	222784_at	SMOC1	SPARC related modular calcium binding 1	chr14q24.2	64093	LOC712994	similar to secreted modular calcium-binding protein 1 isoform 2	---	712994	MmugDNA.13064.1.S1_at_SMOC1
MmugDNA.13065.1.S1_at	239599_at	---	---	---	---	---	---	---	---	MmugDNA.13065.1.S1_at_---
MmugDNA.13066.1.S1_at	238882_at	---	---	---	---	---	---	---	---	MmugDNA.13066.1.S1_at_---
MmugDNA.13067.1.S1_at	223746_at	STK4	serine/threonine kinase 4	chr20q11.2-q13.2	6789	---	---	---	---	MmugDNA.13067.1.S1_at_STK4
MmugDNA.13068.1.S1_at	1554487_a_at	ATF6B	activating transcription factor 6 beta	chr6p21.3	1388	ATF6B	activating transcription factor 6 beta	---	717050	MmugDNA.13068.1.S1_at_ATF6B
MmugDNA.13069.1.S1_at	1555002_at	MGC39545	hypothetical protein LOC403312	chr11q24.2	403312	---	---	---	---	MmugDNA.13069.1.S1_at_MGC39545
MmugDNA.13070.1.S1_s_at	210826_x_at	RAD17	RAD17 homolog (S. pombe)	chr5q13	5884	RAD17	RAD17 homolog (S. pombe)	---	700116	MmugDNA.13070.1.S1_s_at_RAD17
MmugDNA.13071.1.S1_at	1564369_at	---	---	---	---	---	---	---	---	MmugDNA.13071.1.S1_at_---
MmugDNA.13072.1.S1_at	210722_at	PNLIPRP1	pancreatic lipase-related protein 1	chr10q25.3	5407	---	---	---	---	MmugDNA.13072.1.S1_at_PNLIPRP1
MmugDNA.13073.1.S1_at	1562906_at	FAM170A	family with sequence similarity 170, member A	chr5q23.1	340069	LOC712543 /// LOC7144	Similar to eukaryotic translation initiation factor 2C, 3 isoform a /// Similar to ribosomal protein L10	---	712543 /// 714462	MmugDNA.13073.1.S1_at_FAM170A
MmugDNA.13074.1.S1_at	218611_at	IER5	immediate early response 5	chr1q25.3	51278	LOC718339	similar to Immediate early response gene 5 protein	---	718339	MmugDNA.13074.1.S1_at_IER5
MmugDNA.13075.1.S1_at	1560207_at	C8orf81	chromosome 8 open reading frame 81	chr8q24.11	644660	LOC700878	hypothetical protein LOC700878	---	700878	MmugDNA.13075.1.S1_at_C8orf81
MmugDNA.13076.1.S1_at	218983_at	C1RL	complement component 1, r subcomponent-like	chr12p13.31	51279	---	---	---	---	MmugDNA.13076.1.S1_at_C1RL
MmugDNA.13077.1.S1_at	220280_s_at	ANKMY1	ankyrin repeat and MYND domain containing 1	chr2q37.3	51281	ANKMY1	ankyrin repeat and MYND domain containing 1	---	698447	MmugDNA.13077.1.S1_at_ANKMY1
MmugDNA.13078.1.S1_at	232700_at	---	---	---	---	---	---	---	---	MmugDNA.13078.1.S1_at_---
MmugDNA.13080.1.S1_at	235377_at	C6orf142	chromosome 6 open reading frame 142	chr6p12.1	90523	LOC711021	similar to Protein C6orf142 homolog	---	711021	MmugDNA.13080.1.S1_at_C6orf142
MmugDNA.13081.1.S1_at	243635_at	---	---	---	---	---	---	---	---	MmugDNA.13081.1.S1_at_---

MmugDNA.1308.1.S1_at	217121_at	TNKS	tankyrase, TRF1-interacting ankyrin-related ADP-ribose polymerase	chr8p23.1	8658	TNKS	tankyrase, TRF1-interacting ankyrin-related ADP-ribose polymerase	---	699004	MmugDNA.1308.1.S1_at_TNKS
MmugDNA.13082.1.S1_at	1569346_a_at	---	---	---	---	---	---	---	---	MmugDNA.13082.1.S1_at_---
MmugDNA.13083.1.S1_at	231825_x_at	ATF7IP	activating transcription factor 7 interacting protein	chr12p13.1	55729	ATF7IP	activating transcription factor 7 interacting protein	---	698815	MmugDNA.13083.1.S1_at_ATF7IP
MmugDNA.13084.1.S1_at	1555446_s_at	TRAPPC10	trafficking protein particle complex 10	chr21q22.3	7109	---	---	---	---	MmugDNA.13084.1.S1_at_TRAPPC10
MmugDNA.13085.1.S1_at	236833_at	TTC16	tetratricopeptide repeat domain 16	chr9q34.11	158248	LOC706784	similar to tetratricopeptide repeat domain 16	---	706784	MmugDNA.13085.1.S1_at_TTC16
MmugDNA.13086.1.S1_s_at	227006_at	PPP1R14A	protein phosphatase 1, regulatory (inhibitor) subunit 14A	chr19q13.1	94274	LOC719523	similar to protein phosphatase 1, regulatory (inhibitor) subunit 14A	---	719523	MmugDNA.13086.1.S1_s_at_PPP1R14
MmugDNA.13087.1.S1_at	238368_at	---	---	---	---	---	---	---	---	MmugDNA.13087.1.S1_at_---
MmugDNA.13088.1.S1_at	1556105_at	---	---	---	---	---	---	---	---	MmugDNA.13088.1.S1_at_---
MmugDNA.13089.1.S1_at	217314_at	---	---	---	---	CDC37	similar to Hsp90 co-chaperone Cdc37 (Hsp90 chaperone protein kinase-targeting subunit) (p50Cdc3	---	694008	MmugDNA.13089.1.S1_at_---
MmugDNA.13089.1.S1_x_at	NA	NA	NA	NA	NA	NA	NA	NA	NA	MmugDNA.13089.1.S1_x_at_NA
MmugDNA.13090.1.S1_at	228564_at	LOC375295	hypothetical protein LOC375295	chr2q31.1	375295	LOC701656	hypothetical protein LOC701656	---	701656	MmugDNA.13090.1.S1_at_LOC375295
MmugDNA.13091.1.S1_at	242105_at	CCNE1	Cyclin E1	chr19q12	898	---	---	---	---	MmugDNA.13091.1.S1_at_CCNE1
MmugDNA.1309.1.S1_at	240936_at	MFSD1	Major facilitator superfamily domain containing 1	chr3q25.33	64747	---	---	---	---	MmugDNA.1309.1.S1_at_MFSD1
MmugDNA.13092.1.S1_at	244825_at	SHROOM4	shroom family member 4	chrXp11.22	57477	---	---	---	---	MmugDNA.13092.1.S1_at_SHROOM4
MmugDNA.13093.1.S1_at	243687_at	---	---	---	---	LOC708259	hypothetical protein LOC708259	---	708259	MmugDNA.13093.1.S1_at_---
MmugDNA.13094.1.S1_at	210800_at	TIMM8A	translocase of inner mitochondrial membrane 8 homolog A (yeast)	chrXq22.1	1678	---	---	---	---	MmugDNA.13094.1.S1_at_TIMM8A
MmugDNA.13095.1.S1_at	1559692_at	---	---	---	---	---	---	---	---	MmugDNA.13095.1.S1_at_---
MmugDNA.13096.1.S1_x_at	NA	NA	NA	NA	NA	NA	NA	NA	NA	MmugDNA.13096.1.S1_x_at_NA
MmugDNA.13097.1.S1_at	218056_at	BFAR	bifunctional apoptosis regulator	chr16p13.12	51283	LOC695397	Similar to vacuolar protein sorting 54 isoform 1	---	695397	MmugDNA.13097.1.S1_at_BFAR
MmugDNA.13098.1.S1_at	241544_at	---	---	---	---	---	---	---	---	MmugDNA.13098.1.S1_at_---
MmugDNA.13099.1.S1_at	220146_at	TLR7	toll-like receptor 7	chrXp22.3	51284	---	---	---	---	MmugDNA.13099.1.S1_at_TLR7
MmugDNA.13100.1.S1_at	221782_at	DNAJC10	DnaJ (Hsp40) homolog, subfamily C, member 10	chr2q32.1	54431	LOC711450	Similar to crystallin, gamma D	---	711450	MmugDNA.13100.1.S1_at_DNAJC10
MmugDNA.13101.1.S1_at	1566624_at	---	---	---	---	---	---	---	---	MmugDNA.13101.1.S1_at_---
MmugDNA.1310.1.S1_at	230532_at	CXorf38	chromosome X open reading frame 38	chrXp11.4	159013	LOC699271	hypothetical protein LOC699271	---	699271	MmugDNA.1310.1.S1_at_CXorf38
MmugDNA.13102.1.S1_at	225174_at	DNAJC10	DnaJ (Hsp40) homolog, subfamily C, member 10	chr2q32.1	54431	LOC711450	Similar to crystallin, gamma D	---	711450	MmugDNA.13102.1.S1_at_DNAJC10
MmugDNA.13103.1.S1_at	1552785_at	ZNF781	zinc finger protein 781	chr19q13.12	163115	LOC719363	similar to zinc finger protein 110	---	719363	MmugDNA.13103.1.S1_at_ZNF781
MmugDNA.13104.1.S1_at	219591_at	CEND1	cell cycle exit and neuronal differentiation 1	chr11p15.5	51286	---	---	---	---	MmugDNA.13104.1.S1_at_CEND1
MmugDNA.13105.1.S1_at	237987_x_at	---	---	---	---	---	---	---	---	MmugDNA.13105.1.S1_at_---
MmugDNA.13106.1.S1_at	1562890_at	---	---	---	---	---	---	---	---	MmugDNA.13106.1.S1_at_---
MmugDNA.13107.1.S1_at	206476_s_at	NOVA2	neuro-oncological ventral antigen 2	chr19q13.3	4858	LOC716859	similar to neuro-oncological ventral antigen 2	---	716859	MmugDNA.13107.1.S1_at_NOVA2
MmugDNA.13108.1.S1_at	212723_at	JMJD6	jumonji domain containing 6	chr17q25	23210	---	---	---	---	MmugDNA.13108.1.S1_at_JMJD6
MmugDNA.13109.1.S1_at	237231_at	---	---	---	---	---	---	---	---	MmugDNA.13109.1.S1_at_---
MmugDNA.13110.1.S1_at	1555547_at	BPA-1	brain peptide A1	chr1p31.3	100131534	---	---	---	---	MmugDNA.13110.1.S1_at_BPA-1
MmugDNA.13111.1.S1_at	1569842_at	LOC728114	hypothetical protein LOC728114	chr12q13.11	728114	LOC704950	hypothetical protein LOC704950	---	704950	MmugDNA.13111.1.S1_at_LOC728114
MmugDNA.1311.1.S1_at	1555298_a_at	VWA3B	von Willebrand factor A domain containing 3B	chr2q11.2	200403	LOC707760	hypothetical protein LOC707760	---	707760	MmugDNA.1311.1.S1_at_VWA3B
MmugDNA.13112.1.S1_at	202489_s_at	FXYD3	FXYD domain containing ion transport regulator 3	chr19q13.11-q13.12	5349	FXYD3	FXYD domain containing ion transport regulator 3	---	707346	MmugDNA.13112.1.S1_at_FXYD3
MmugDNA.13113.1.S1_at	243607_at	---	---	---	---	---	---	---	---	MmugDNA.13113.1.S1_at_---
MmugDNA.13114.1.S1_at	1562689_at	LOC151484	hypothetical protein LOC151484	chr2q37.1	151484	---	---	---	---	MmugDNA.13114.1.S1_at_LOC151484
MmugDNA.13115.1.S1_at	217118_s_at	C22orf9	chromosome 22 open reading frame 9	chr22q13.31	23313	LOC717328	hypothetical protein LOC717328	---	717328	MmugDNA.13115.1.S1_at_C22orf9
MmugDNA.13116.1.S1_at	232260_at	LOC100009676	hypothetical LOC100009676	chr3q12.3	100009676	---	---	---	---	MmugDNA.13116.1.S1_at_LOC100009
MmugDNA.13117.1.S1_at	235315_at	TSC22D1	TSC22 domain family, member 1	chr13q14	8848	---	---	---	---	MmugDNA.13117.1.S1_at_TSC22D1
MmugDNA.13118.1.S1_at	211334_at	MRE11A	MRE11 meiotic recombination 11 homolog A (S. cerevisiae)	chr11q21	4361	---	---	---	---	MmugDNA.13118.1.S1_at_MRE11A
MmugDNA.13119.1.S1_at	1563290_at	DNAH3	dynein, axonemal, heavy chain 3	chr16p12.2	55567	LOC697227	similar to dynein, axonemal, heavy polypeptide 3	---	697227	MmugDNA.13119.1.S1_at_DNAH3
MmugDNA.131.1.S1_at	206175_x_at	ZNF222	zinc finger protein 222	chr19q13.2	7673	ZNF222	zinc finger protein 222	---	711652	MmugDNA.131.1.S1_at_ZNF222
MmugDNA.13120.1.S1_at	212523_s_at	KIAA0146	KIAA0146	chr8q11.21	23514	LOC714523	hypothetical protein LOC714523	---	714523	MmugDNA.13120.1.S1_at_KIAA0146
MmugDNA.13121.1.S1_at	1552327_at	ARMCX4	armadillo repeat containing, X-linked 4	chrXq22.1	158947	---	---	---	---	MmugDNA.13121.1.S1_at_ARMCX4
MmugDNA.1312.1.S1_at	236674_at	LOC388780	hypothetical LOC388780	chr20p13	388780	---	---	---	---	MmugDNA.1312.1.S1_at_LOC388780
MmugDNA.13123.1.S1_at	227931_at	INO80D	INO80 complex subunit D	chr2q33.3	54891	---	---	---	---	MmugDNA.13123.1.S1_at_INO80D
MmugDNA.13124.1.S1_at	227924_at	INO80D	INO80 complex subunit D	chr2q33.3	54891	---	---	---	---	MmugDNA.13124.1.S1_at_INO80D
MmugDNA.13125.1.S1_at	1563906_at	SOBP	sine oculis binding protein homolog (Drosophila)	chr6q21	55084	---	---	---	---	MmugDNA.13125.1.S1_at_SOBP
MmugDNA.13126.1.S1_at	1559638_at	C1orf200	chromosome 1 open reading frame 200	chr1p36.22	644997	LOC710638	hypothetical protein LOC710638	---	710638	MmugDNA.13126.1.S1_at_C1orf200
MmugDNA.13127.1.S1_at	203347_s_at	MTF2	metal response element binding transcription factor 2	chr1p22.1	22823	LOC702671	Similar to Aprataxin (Forkhead-associated domain histidine triad-like protein) (FHA-HIT)	---	702671	MmugDNA.13127.1.S1_at_MTF2
MmugDNA.13128.1.S1_at	221974_at	IPW	imprinted in Prader-Willi syndrome (non-protein coding)	chr15q11-q12	3653	---	---	---	---	MmugDNA.13128.1.S1_at_IPW
MmugDNA.13129.1.S1_at	1555019_at	PCDH21	protocadherin 21	chr10q22.1-q22.3	92211	PCDH21	protocadherin 21	---	698637	MmugDNA.13129.1.S1_at_PCDH21
MmugDNA.13130.1.S1_s_at	217077_s_at	GABBR2	gamma-aminobutyric acid (GABA) B receptor, 2	chr9q22.1-q22.3	9568	LOC699134	Similar to H3 histone, family 3B	---	699134	MmugDNA.13130.1.S1_s_at_GABBR2
MmugDNA.13131.1.S1_at	1553498_at	---	---	---	---	---	---	---	---	MmugDNA.13131.1.S1_at_---
MmugDNA.1313.1.S1_at	212441_at	KIAA0232	KIAA0232	chr4p16.1	9778	LOC710141	similar to Protein KIAA0232	---	710141	MmugDNA.1313.1.S1_at_KIAA0232
MmugDNA.13132.1.S1_at	227402_s_at	UTP23	UTP23, small subunit (SSU) processome component, homolog (yeast)	chr8q24.11	84294	LOC702915	similar to CG17652-PA	---	702915	MmugDNA.13132.1.S1_at_UTP23
MmugDNA.13133.1.S1_at	227951_s_at	FAM98C	family with sequence similarity 98, member C	chr19q13.2	147965	---	---	---	---	MmugDNA.13133.1.S1_at_FAM98C
MmugDNA.13134.1.S1_at	230055_at	KHDC1	KH homology domain containing 1	chr6q13	80759	LOC715576	similar to chromosome 6 open reading frame 148	---	715576	MmugDNA.13134.1.S1_at_KHDC1
MmugDNA.13135.1.S1_at	1558778_s_at	MKL2	MKL/myocardin-like 2	chr16p13.12	57496	---	---	---	---	MmugDNA.13135.1.S1_at_MKL2
MmugDNA.13136.1.S1_at	234160_at	---	---	---	---	---	---	---	---	MmugDNA.13136.1.S1_at_---
MmugDNA.13137.1.S1_at	244406_at	ZNF20 /// ZNF625	zinc finger protein 20 /// zinc finger protein 625	chr19p13.2 /// chr19p13.3-p13.2	7568 /// 90589	---	---	---	---	MmugDNA.13137.1.S1_at_ZNF20 /// ZN
MmugDNA.13138.1.S1_at	236866_at	GALNS	galactosamine (N-acetyl)-6-sulfate sulfatase	chr16q24.3	2588	---	---	---	---	MmugDNA.13138.1.S1_at_GALNS
MmugDNA.13139.1.S1_at	1552987_a_at	MGC42157	hypothetical locus MGC42157	chr4p14	439933	---	---	---	---	MmugDNA.13139.1.S1_at_MGC42157
MmugDNA.13140.1.S1_at	NA	NA	NA	NA	NA	NA	NA	NA	NA	MmugDNA.13140.1.S1_at_NA
MmugDNA.13141.1.S1_at	NA	NA	NA	NA	NA	NA	NA	NA	NA	MmugDNA.13141.1.S1_at_NA
MmugDNA.13141.1.S1_x_at	NA	NA	NA	NA	NA	NA	NA	NA	NA	MmugDNA.13141.1.S1_x_at_NA
MmugDNA.1314.1.S1_at	231979_at	---	---	---	---	---	---	---	---	MmugDNA.1314.1.S1_at_---
MmugDNA.13142.1.S1_at	238557_at	LOC100144603	hypothetical transcript	---	100144603	LOC720704	hypothetical protein LOC720704	---	720704	MmugDNA.13142.1.S1_at_LOC100144
MmugDNA.13143.1.S1_at	237638_at	---	---	---	---	---	---	---	---	MmugDNA.13143.1.S1_at_---
MmugDNA.13144.1.S1_at	214212_x_at	FERMT2	fermitin family homolog 2 (Drosophila)	chr14q22.2	10979	FERMT2	fermitin family homolog 2 (Drosophila)	---	693958	MmugDNA.13144.1.S1_at_FERMT2
MmugDNA.13145.1.S1_at	238772_at	ZNF207	zinc finger protein 207	chr17q11.2	7756	---	---	---	---	MmugDNA.13145.1.S1_at_ZNF207
MmugDNA.13146.1.S1_at	230954_at	C20orf112	chromosome 20 open reading frame 112	chr20q11.1-q11.23	140688	---	---	---	---	MmugDNA.13146.1.S1_at_C20orf112
MmugDNA.13147.1.S1_at	243428_at	KCNQ1OT1	KCNQ1 overlapping transcript 1 (non-protein coding)	chr11p15	10984	---	---	---	---	MmugDNA.13147.1.S1_at_KCNQ1OT1
MmugDNA.13148.1.S1_at	242191_at	NBPF10 /// NBPF11	neuroblastoma breakpoint family, member 10 /// neuroblastoma breakpoint family, member 11	chr1q21.1	100132406 /// 728912	---	---	---	---	MmugDNA.13148.1.S1_at_NBPF10 /// N
MmugDNA.13149.1.S1_at	1557796_at	---	---	---	---	---	---	---	---	MmugDNA.13149.1.S1_at_---
MmugDNA.13150.1.S1_at	230058_at	LOC646891 /// SDCCA	similar to serologically defined colon cancer antigen 3 /// serologically defined colon cancer antigen 3	chr1q25.1 /// chr9q34.3	10807 /// 646891	LOC721750	similar to serologically defined colon cancer antigen 3 isoform 1	---	721750	MmugDNA.13150.1.S1_at_LOC646891
MmugDNA.13151.1.S1_at	230252_at	LPAR5	lysophosphatidic acid receptor 5	chr12p13.31	57121	LPAR5	lysophosphatidic acid receptor 5	---	713283	MmugDNA.13151.1.S1_at_LPAR5
MmugDNA.1315.1.S1_at	214920_at	THSD7A	thrombospondin, type I, domain containing 7A	chr7p21.3	221981	---	---	---	---	MmugDNA.1315.1.S1_at_THSD7A
MmugDNA.13152.1.S1_at	221909_at	RNFT2	ring finger protein, transmembrane 2	chr12q24.22	84900	---	---	---	---	MmugDNA.13152.1.S1_at_RNFT2
MmugDNA.13153.1.S1_at	213266_at	TUBGCP4	Tubulin, gamma complex associated protein 4	chr15q15	27229	LOC710237	similar to tumor protein p53 binding protein, 1	---	710237	MmugDNA.13153.1.S1_at_TUBGCP4
MmugDNA.13154.1.S1_at	221908_at	RNFT2	ring finger protein, transmembrane 2	chr12q24.22	84900	---	---	---	---	MmugDNA.13154.1.S1_at_RNFT2
MmugDNA.13155.1.S1_at	204619_s_at	VCAN	versican	chr5q14.3	1462	LOC712365	similar to chondroitin sulfate proteoglycan 2 (versican)	---	712365	MmugDNA.13155.1.S1_at_VCAN
MmugDNA.13156.1.S1_at	212097_at	CAV1	caveolin 1, caveolae protein, 22kDa	chr7q31.1	857	CAV1	caveolin 1, caveolae protein, 22kDa	---	704449	MmugDNA.13156.1.S1_at_CAV1
MmugDNA.13157.1.S1_s_at	211211_x_at	SH2D1A	SH2 domain protein 1A	chrXq25-q26	4068	SAP	SAP protein	---	574156	MmugDNA.13157.1.S1_s_at_SH2D1A
MmugDNA.13158.1.S1_at	241061_at	---	---	---	---	---	---	---	---	MmugDNA.13158.1.S1_at_---
MmugDNA.13159.1.S1_at	235771_at	---	---	---	---	---	---	---	---	MmugDNA.13159.1.S1_at_---
MmugDNA.13160.1.S1_at	215917_at	SNPH	syntaphilin	chr20p13	9751	LOC717271	similar to RAD21 homolog	---	717271	MmugDNA.13160.1.S1_at_SNPH
MmugDNA.1316.1.S1_at	237514_at	---	---	---	---	---	---	---	---	MmugDNA.1316.1.S1_at_---
MmugDNA.13162.1.S1_at	1552633_at	ZNF101	zinc finger protein 101	chr19p13.11	94039	LOC720388	similar to zinc finger protein 101	---	720388	MmugDNA.13162.1.S1_at_ZNF101
MmugDNA.13164.1.S1_at	237715_at	AKNA	AT-hook transcription factor	chr9q32	80709	---	---	---	---	MmugDNA.13164.1.S1_at_AKNA
MmugDNA.13165.1.S1_at	1552671_a_at	SLC9A7	solute carrier family 9 (sodium/hydrogen exchanger), member 7	chrXp11.3-p11.23|Xp11.3	84679	LOC693271	similar to solute carrier family 9, member 7	---	693271	MmugDNA.13165.1.S1_at_SLC9A7
MmugDNA.13166.1.S1_at	232757_at	---	---	---	---	---	---	---	---	MmugDNA.13166.1.S1_at_---
MmugDNA.13167.1.S1_at	1566768_at	---	---	---	---	---	---	---	---	MmugDNA.13167.1.S1_at_---
MmugDNA.13168.1.S1_at	227166_at	DNAJC18	DnaJ (Hsp40) homolog, subfamily C, member 18	chr5q31.2	202052	---	---	---	---	MmugDNA.13168.1.S1_at_DNAJC18
MmugDNA.13169.1.S1_at	227169_at	DNAJC18	DnaJ (Hsp40) homolog, subfamily C, member 18	chr5q31.2	202052	TSPO	Translocator protein (18kDa)	---	711717	MmugDNA.13169.1.S1_at_DNAJC18
MmugDNA.13170.1.S1_at	230701_x_at	KIF9	kinesin family member 9	chr3p21.31	64147	LOC711742	similar to kinesin family member 9 isoform 2	---	711742	MmugDNA.13170.1.S1_at_KIF9
MmugDNA.13171.1.S1_at	1561721_a_at	C12orf40	chromosome 12 open reading frame 40	chr12q12	283461	LOC699770	hypothetical protein LOC699770	---	699770	MmugDNA.13171.1.S1_at_C12orf40
MmugDNA.1317.1.S1_at	210134_x_at	SHOX2	short stature homeobox 2	chr3q25-q26.1	6474	SHOX2	short stature homeobox 2	---	704327	MmugDNA.1317.1.S1_at_SHOX2
MmugDNA.13172.1.S1_at	212507_at	TMEM131	transmembrane protein 131	chr2q11.2	23505	LOC718306	Similar to cleft lip and palate associated transmembrane protein 1	---	718306	MmugDNA.13172.1.S1_at_TMEM131
MmugDNA.13173.1.S1_at	212875_s_at	C2CD2	C2 calcium-dependent domain containing 2	chr21q22.3	25966	LOC714860	hypothetical protein LOC714860	---	714860	MmugDNA.13173.1.S1_at_C2CD2
MmugDNA.13174.1.S1_at	223400_s_at	PBRM1	polybromo 1	chr3p21	55193	PB1	polybromo 1	---	696361	MmugDNA.13174.1.S1_at_PBRM1
MmugDNA.13175.1.S1_at	235213_at	---	---	---	---	---	---	---	---	MmugDNA.13175.1.S1_at_---
MmugDNA.13176.1.S1_at	1555431_a_at	IL31RA	interleukin 31 receptor A	chr5q11.2	133396	LOC709719	similar to gp130-like monocyte receptor	---	709719	MmugDNA.13176.1.S1_at_IL31RA
MmugDNA.13177.1.S1_at	227239_at	FAM126A	family with sequence similarity 126, member A	chr7p15.3	84668	AQP1	Aquaporin 1	---	694735	MmugDNA.13177.1.S1_at_FAM126A
MmugDNA.13178.1.S1_at	211347_at	CDC14B	CDC14 cell division cycle 14 homolog B (S. cerevisiae)	chr9q22.33	8555	---	---	---	---	MmugDNA.13178.1.S1_at_CDC14B
MmugDNA.13179.1.S1_at	230900_at	CCDC110	coiled-coil domain containing 110	chr4q35.1	256309	CCDC110	coiled-coil domain containing 110	---	695900	MmugDNA.13179.1.S1_at_CCDC110

MmugDNA.13180.1.S1_at	231506_at	---	---	---	---	LOC715985	Similar to 60S ribosomal protein L7a	---	715985	MmugDNA.13180.1.S1_at_---
MmugDNA.13181.1.S1_at	230044_at	NPB	Neuropeptide B	chr17q25.3	256933	---	---	---	---	MmugDNA.13181.1.S1_at_NPB
MmugDNA.1318.1.S1_at	236981_at	C17orf99	chromosome 17 open reading frame 99	---	100141515	LOC715098	hypothetical protein LOC715098	---	715098	MmugDNA.1318.1.S1_at_C17orf99
MmugDNA.13182.1.S1_at	244352_at	---	---	---	---	---	---	---	---	MmugDNA.13182.1.S1_at_---
MmugDNA.13183.1.S1_at	1557141_at	C7orf67	chromosome 7 open reading frame 67	chr7p15.1	84182	LOC694854	similar to CG7332-PA	---	694854	MmugDNA.13183.1.S1_at_C7orf67
MmugDNA.13185.1.S1_at	217031_at	KRT84	keratin 84	chr12q13	3890	KRT84	keratin 84	---	698573	MmugDNA.13185.1.S1_at_KRT84
MmugDNA.13186.1.S1_at	228492_at	USP9Y	ubiquitin specific peptidase 9, Y-linked	chrYq11.2	8287	---	---	---	---	MmugDNA.13186.1.S1_at_USP9Y
MmugDNA.13187.1.S1_at	222328_x_at	MEG3	Maternally expressed 3 (non-protein coding)	chr14q32	55384	---	---	---	---	MmugDNA.13187.1.S1_at_MEG3
MmugDNA.13188.1.S1_at	236631_at	C21orf125	chromosome 21 open reading frame 125	chr21q22.3	284836	LOC708882	Similar to RWD domain containing 4A	---	708882	MmugDNA.13188.1.S1_at_C21orf125
MmugDNA.13189.1.S1_at	228354_at	MORN4	MORN repeat containing 4	chr10q24.1	118812	MORN4	MORN repeat containing 4	---	706860	MmugDNA.13189.1.S1_at_MORN4
MmugDNA.13190.1.S1_at	236962_at	---	---	---	---	---	---	---	---	MmugDNA.13190.1.S1_at_---
MmugDNA.13191.1.S1_at	1568907_at	---	---	---	---	---	---	---	---	MmugDNA.13191.1.S1_at_---
MmugDNA.1319.1.S1_at	216230_x_at	SMPD1	sphingomyelin phosphodiesterase 1, acid lysosomal	chr11p15.4-p15.1	6609	SMPD1	sphingomyelin phosphodiesterase 1, acid lysosomal	---	711248	MmugDNA.1319.1.S1_at_SMPD1
MmugDNA.13192.1.S1_s_at	210502_s_at	PPIE	peptidylprolyl isomerase E (cyclophilin E)	chr1p32	10450	PPIE	peptidylprolyl isomerase E	---	716340	MmugDNA.13192.1.S1_s_at_PPIE
MmugDNA.13193.1.S1_at	1570272_at	---	---	---	---	---	---	---	---	MmugDNA.13193.1.S1_at_---
MmugDNA.13194.1.S1_at	1554334_a_at	DNAJA4	DnaJ (Hsp40) homolog, subfamily A, member 4	chr15q25.1	55466	DNAJA4	DnaJ (Hsp40) homolog, subfamily A, member 4	---	709872	MmugDNA.13194.1.S1_at_DNAJA4
MmugDNA.13194.1.S1_s_at	NA	NA	NA	NA	NA	NA	NA	NA	NA	MmugDNA.13194.1.S1_s_at_NA
MmugDNA.13195.1.S1_at	244478_at	LRRC37A3	leucine rich repeat containing 37, member A3	chr17q24.1	374819	---	---	---	---	MmugDNA.13195.1.S1_at_LRRC37A3
MmugDNA.13196.1.S1_at	232796_at	---	---	---	---	---	---	---	---	MmugDNA.13196.1.S1_at_---
MmugDNA.13197.1.S1_at	1558414_at	C9orf4	chromosome 9 open reading frame 4	chr9q31	23732	LOC710212	similar to CG8399-PA	---	710212	MmugDNA.13197.1.S1_at_C9orf4
MmugDNA.13198.1.S1_at	224362_at	LOC100128922	connexin	chr1p33	100128922	---	---	---	---	MmugDNA.13198.1.S1_at_LOC100128
MmugDNA.13199.1.S1_at	224363_at	---	---	---	---	---	---	---	---	MmugDNA.13199.1.S1_at_---
MmugDNA.13.1.S1_at	211435_at	---	---	---	---	---	---	---	---	MmugDNA.13.1.S1_at_---
MmugDNA.13200.1.S1_at	215763_at	---	---	---	---	---	---	---	---	MmugDNA.13200.1.S1_at_---
MmugDNA.13201.1.S1_at	224364_s_at	PPIL3	peptidylprolyl isomerase (cyclophilin)-like 3	chr2q33.1	53938	PPIL3	peptidylprolyl isomerase (cyclophilin)-like 3	---	700456	MmugDNA.13201.1.S1_at_PPIL3
MmugDNA.1320.1.S1_at	1569305_a_at	---	---	---	---	---	---	---	---	MmugDNA.1320.1.S1_at_---
MmugDNA.13202.1.S1_at	239050_s_at	---	---	---	---	---	---	---	---	MmugDNA.13202.1.S1_at_---
MmugDNA.13203.1.S1_at	226859_at	DNAJC25	DnaJ (Hsp40) homolog, subfamily C , member 25	chr9q31.3	548645	---	---	---	---	MmugDNA.13203.1.S1_at_DNAJC25
MmugDNA.13204.1.S1_at	214584_x_at	ACACB	acetyl-Coenzyme A carboxylase beta	chr12q24.11	32	LOC706909	similar to acetyl-Coenzyme A carboxylase beta	---	706909	MmugDNA.13204.1.S1_at_ACACB
MmugDNA.13205.1.S1_at	233240_at	---	---	---	---	---	---	---	---	MmugDNA.13205.1.S1_at_---
MmugDNA.13206.1.S1_at	1561294_a_at	hCG_2015435	hypothetical protein LOC100128554	chr12q24.32	100128554	---	---	---	---	MmugDNA.13206.1.S1_at_hCG_20154
MmugDNA.13207.1.S1_at	243345_at	---	---	---	---	---	---	---	---	MmugDNA.13207.1.S1_at_---
MmugDNA.13208.1.S1_at	1570596_at	---	---	---	---	---	---	---	---	MmugDNA.13208.1.S1_at_---
MmugDNA.13209.1.S1_at	243810_at	---	---	---	---	---	---	---	---	MmugDNA.13209.1.S1_at_---
MmugDNA.13210.1.S1_at	1559589_a_at	---	---	---	---	---	---	---	---	MmugDNA.13210.1.S1_at_---
MmugDNA.13211.1.S1_at	216048_s_at	RHOBTB3	Rho-related BTB domain containing 3	chr5q15	22836	LOC716022	Similar to Vasodilator-stimulated phosphoprotein (VASP)	---	716022	MmugDNA.13211.1.S1_at_RHOBTB3
MmugDNA.1321.1.S1_at	240907_at	---	---	---	---	---	---	---	---	MmugDNA.1321.1.S1_at_---
MmugDNA.13212.1.S1_at	1561288_at	---	---	---	---	---	---	---	---	MmugDNA.13212.1.S1_at_---
MmugDNA.13213.1.S1_at	NA	NA	NA	NA	NA	NA	NA	NA	NA	MmugDNA.13213.1.S1_at_NA
MmugDNA.13214.1.S1_at	1561091_at	---	---	---	---	---	---	---	---	MmugDNA.13214.1.S1_at_---
MmugDNA.13215.1.S1_at	225301_s_at	MYO5B	myosin VB	chr18q21	4645	MYO5B	myosin VB	---	699592	MmugDNA.13215.1.S1_at_MYO5B
MmugDNA.13216.1.S1_at	215800_at	DUOX1	dual oxidase 1	chr15q15.3	53905	---	---	---	---	MmugDNA.13216.1.S1_at_DUOX1
MmugDNA.13217.1.S1_at	227295_at	IKIP	IKK interacting protein	chr12q23.1	121457	IKIP	IKK interacting protein	---	693896	MmugDNA.13217.1.S1_at_IKIP
MmugDNA.13218.1.S1_at	222392_x_at	PERP	PERP, TP53 apoptosis effector	chr6q24	64065	PERP	PERP, TP53 apoptosis effector	---	703890	MmugDNA.13218.1.S1_at_PERP
MmugDNA.13219.1.S1_at	1552311_a_at	RAX2	retina and anterior neural fold homeobox 2	chr19p13.3	84839	LOC713929	similar to retina and anterior neural fold homeobox	---	713929	MmugDNA.13219.1.S1_at_RAX2
MmugDNA.132.1.S1_at	210033_s_at	SPAG6	sperm associated antigen 6	chr10p12.2	9576	SPAG6	sperm associated antigen 6	---	704807	MmugDNA.132.1.S1_at_SPAG6
MmugDNA.13220.1.S1_at	243958_at	---	---	---	---	---	---	---	---	MmugDNA.13220.1.S1_at_---
MmugDNA.13221.1.S1_at	227368_at	---	---	---	---	---	---	---	---	MmugDNA.13221.1.S1_at_---
MmugDNA.1322.1.S1_at	NA	NA	NA	NA	NA	NA	NA	NA	NA	MmugDNA.1322.1.S1_at_NA
MmugDNA.1322.1.S1_s_at	217448_s_at	LOC285412 /// TOX4	similar to KIAA0737 protein /// TOX high mobility group box family member 4	chr14q11.2 /// chr4q25	285412 /// 9878	LOC701204 /// LOC7069	similar to epidermal Langerhans cell protein LCP1 /// epidermal Langerhans cell protein LCP1	---	701204 /// 706950	MmugDNA.1322.1.S1_s_at_LOC285412
MmugDNA.13222.1.S1_s_at	NA	NA	NA	NA	NA	NA	NA	NA	NA	MmugDNA.13222.1.S1_s_at_NA
MmugDNA.13223.1.S1_at	1556332_at	---	---	---	---	---	---	---	---	MmugDNA.13223.1.S1_at_---
MmugDNA.13224.1.S1_at	221338_at	LY6G6E	lymphocyte antigen 6 complex, locus G6E	chr6p21.3	79136	---	---	---	---	MmugDNA.13224.1.S1_at_LY6G6E
MmugDNA.13225.1.S1_at	224365_s_at	TIGD7	tigger transposable element derived 7	chr16p13.3	91151	---	---	---	---	MmugDNA.13225.1.S1_at_TIGD7
MmugDNA.13226.1.S1_at	208200_at	IL1A	interleukin 1, alpha	chr2q14	3552	---	---	---	---	MmugDNA.13226.1.S1_at_IL1A
MmugDNA.13227.1.S1_at	224366_s_at	REPS1	RALBP1 associated Eps domain containing 1	---	85021	REPS1	RALBP1 associated Eps domain containing 1	---	702858	MmugDNA.13227.1.S1_at_REPS1
MmugDNA.13228.1.S1_at	232487_at	SFT2D1	SFT2 domain containing 1	chr6q27	113402	LOC716785	similar to CG5104-PB	---	716785	MmugDNA.13228.1.S1_at_SFT2D1
MmugDNA.13229.1.S1_at	222196_at	LOC286434	hypothetical protein LOC286434	chrXp22.33	286434	---	---	---	---	MmugDNA.13229.1.S1_at_LOC286434
MmugDNA.13230.1.S1_at	210938_at	PDX1	pancreatic and duodenal homeobox 1	chr13q12.1	3651	PDX1	pancreatic and duodenal homeobox 1	---	710936	MmugDNA.13230.1.S1_at_PDX1
MmugDNA.13231.1.S1_at	221362_at	HTR5A	5-hydroxytryptamine (serotonin) receptor 5A	chr7q36.1	3361	---	---	---	---	MmugDNA.13231.1.S1_at_HTR5A
MmugDNA.1323.1.S1_s_at	NA	NA	NA	NA	NA	NA	NA	NA	NA	MmugDNA.1323.1.S1_s_at_NA
MmugDNA.13232.1.S1_at	222865_x_at	TMEM204	transmembrane protein 204	chr16p13.3	79652	LOC701926	similar to Claudin-like protein 24	---	701926	MmugDNA.13232.1.S1_at_TMEM204
MmugDNA.13232.1.S1_s_at	228947_x_at	TMEM204	transmembrane protein 204	chr16p13.3	79652	LOC701926	similar to Claudin-like protein 24	---	701926	MmugDNA.13232.1.S1_s_at_TMEM204
MmugDNA.13233.1.S1_at	224367_at	BEX2	brain expressed X-linked 2	chrXq22	84707	BEX2	brain expressed X-linked 2	---	696048	MmugDNA.13233.1.S1_at_BEX2
MmugDNA.13235.1.S1_at	224368_s_at	NDRG3	NDRG family member 3	chr20q11.21-q11.23	57446	LOC702452	similar to N-myc downstream regulated gene 3	---	702452	MmugDNA.13235.1.S1_at_NDRG3
MmugDNA.13236.1.S1_at	235744_at	PPTC7	PTC7 protein phosphatase homolog (S. cerevisiae)	chr12q24.11	160760	LOC709471	similar to T-cell activation protein phosphatase 2C	---	709471	MmugDNA.13236.1.S1_at_PPTC7
MmugDNA.13237.1.S1_s_at	229640_x_at	---	---	---	---	LOC715015	similar to lethal (2) k00619 CG4775-PA	---	715015	MmugDNA.13237.1.S1_s_at_---
MmugDNA.13238.1.S1_at	1560514_at	LOC285205	hypothetical LOC285205	chr3q13.12	285205	LOC706875	hypothetical protein LOC706875	---	706875	MmugDNA.13238.1.S1_at_LOC285205
MmugDNA.13239.1.S1_at	233909_at	---	---	---	---	---	---	---	---	MmugDNA.13239.1.S1_at_---
MmugDNA.13240.1.S1_at	243019_at	LOC100132207	hypothetical LOC100132207	chr3q21.3	100132207	---	---	---	---	MmugDNA.13240.1.S1_at_LOC100132
MmugDNA.13241.1.S1_at	217357_at	---	---	---	---	---	---	---	---	MmugDNA.13241.1.S1_at_---
MmugDNA.1324.1.S1_s_at	214541_s_at	QKI	quaking homolog, KH domain RNA binding (mouse)	chr6q26|6q26-q27	9444	LOC712600	similar to quaking homolog, KH domain RNA binding isoform HQK-5	---	712600	MmugDNA.1324.1.S1_s_at_QKI
MmugDNA.13242.1.S1_at	219394_at	PGS1	phosphatidylglycerophosphate synthase 1	chr17q25.3	9489	LOC711720	Similar to family with sequence similarity 62 (C2 domain containing), member A	---	711720	MmugDNA.13242.1.S1_at_PGS1
MmugDNA.13243.1.S1_at	215040_at	RNASEH2B	Ribonuclease H2, subunit B	chr13q14.3	79621	LOC707995 /// LOC7116	similar to CG11164-PA /// hypothetical protein LOC711668	---	707995 /// 711668	MmugDNA.13243.1.S1_at_RNASEH2B
MmugDNA.13244.1.S1_at	234247_at	---	---	---	---	---	---	---	---	MmugDNA.13244.1.S1_at_---
MmugDNA.13245.1.S1_at	233117_at	---	---	---	---	---	---	---	---	MmugDNA.13245.1.S1_at_---
MmugDNA.13246.1.S1_at	242675_x_at	---	---	---	---	---	---	---	---	MmugDNA.13246.1.S1_at_---
MmugDNA.13247.1.S1_at	233132_at	LOC96610	BMS1 homolog, ribosome assembly protein (yeast) pseudogene	chr22q11.22	96610	LOC701772	similar to Immunoglobulin omega chain precursor (VpreB2 protein)	---	701772	MmugDNA.13247.1.S1_at_LOC96610
MmugDNA.13248.1.S1_at	237140_x_at	---	---	---	---	---	---	---	---	MmugDNA.13248.1.S1_at_---
MmugDNA.13249.1.S1_at	237510_at	MYNN	Myoneurin	chr3q26.2	55892	---	---	---	---	MmugDNA.13249.1.S1_at_MYNN
MmugDNA.13250.1.S1_at	224369_s_at	FBXO38	F-box protein 38	chr5q33.1	81545	FBXO38	F-box protein 38	---	709869	MmugDNA.13250.1.S1_at_FBXO38
MmugDNA.13251.1.S1_at	236726_at	RGS6	regulator of G-protein signaling 6	chr14q24.3	9628	---	---	---	---	MmugDNA.13251.1.S1_at_RGS6
MmugDNA.1325.1.S1_at	1557660_s_at	LOC728883	hypothetical LOC728883	chr10q11.22	728883	---	---	---	---	MmugDNA.1325.1.S1_at_LOC728883
MmugDNA.13252.1.S1_at	224370_s_at	CAPS2	calcyphosine 2	chr12q21.1	84698	---	---	---	---	MmugDNA.13252.1.S1_at_CAPS2
MmugDNA.13253.1.S1_at	NA	NA	NA	NA	NA	NA	NA	NA	NA	MmugDNA.13253.1.S1_at_NA
MmugDNA.13253.1.S1_x_at	1565065_at	OFCC1	orofacial cleft 1 candidate 1	chr6p24.3	266553	---	---	---	---	MmugDNA.13253.1.S1_x_at_OFCC1
MmugDNA.13254.1.S1_at	215473_at	---	---	---	---	---	---	---	---	MmugDNA.13254.1.S1_at_---
MmugDNA.13255.1.S1_at	224371_at	ADAMTSL1	ADAMTS-like 1	chr9p22.2-p22.1	92949	LOC712844	similar to ADAMTS-like 3	---	712844	MmugDNA.13255.1.S1_at_ADAMTSL1
MmugDNA.13256.1.S1_at	229280_s_at	FLJ22536	hypothetical locus LOC401237	chr6p22.3	401237	LOC706938	hypothetical protein LOC706938	---	706938	MmugDNA.13256.1.S1_at_FLJ22536
MmugDNA.13257.1.S1_at	238531_x_at	---	---	---	---	---	---	---	---	MmugDNA.13257.1.S1_at_---
MmugDNA.13258.1.S1_at	221696_s_at	STYK1	serine/threonine/tyrosine kinase 1	chr12p13.2	55359	LOC717844	similar to serine/threonine/tyrosine kinase 1	---	717844	MmugDNA.13258.1.S1_at_STYK1
MmugDNA.13259.1.S1_at	200828_s_at	ZNF207	zinc finger protein 207	chr17q11.2	7756	LOC705182	similar to zinc finger protein 207	---	705182	MmugDNA.13259.1.S1_at_ZNF207
MmugDNA.13260.1.S1_at	243762_at	---	---	---	---	---	---	---	---	MmugDNA.13260.1.S1_at_---
MmugDNA.13261.1.S1_at	243022_at	---	---	---	---	---	---	---	---	MmugDNA.13261.1.S1_at_---
MmugDNA.1326.1.S1_at	222965_at	PRO2214	hypothetical protein PRO2214	chr5q12.2	55387	---	---	---	---	MmugDNA.1326.1.S1_at_PRO2214
MmugDNA.13262.1.S1_at	243133_at	TSC22D1	TSC22 domain family, member 1	chr13q14	8848	LOC701076	similar to TSC22 domain family 1 isoform 1	---	701076	MmugDNA.13262.1.S1_at_TSC22D1
MmugDNA.13263.1.S1_at	236879_at	---	---	---	---	---	---	---	---	MmugDNA.13263.1.S1_at_---
MmugDNA.13264.1.S1_at	1555822_at	FAM138A /// FAM138B	family with sequence similarity 138, member A /// family with sequence similarity 138, member B /// fa	chr15q26.3 /// chr19p13.3 /// chr1	100124412 /// 641702 /	---	---	---	---	MmugDNA.13264.1.S1_at_FAM138A ///
MmugDNA.13265.1.S1_at	243630_at	NDUFB1	NADH dehydrogenase (ubiquinone) 1 beta subcomplex, 1, 7kDa	chr14q32.12	4707	---	---	---	---	MmugDNA.13265.1.S1_at_NDUFB1
MmugDNA.13266.1.S1_at	211535_s_at	FGFR1	fibroblast growth factor receptor 1	chr8p11.2-p11.1	2260	FGFR1	fibroblast growth factor receptor 1	---	700126	MmugDNA.13266.1.S1_at_FGFR1
MmugDNA.13267.1.S1_at	238609_at	C7orf38	chromosome 7 open reading frame 38	chr7q22.1	221786	LOC718782	hypothetical protein LOC718782	---	718782	MmugDNA.13267.1.S1_at_C7orf38
MmugDNA.13268.1.S1_at	226561_at	AGFG1	ArfGAP with FG repeats 1	chr2q36.3	3267	LOC694219	Similar to homeodomain-only protein	---	694219	MmugDNA.13268.1.S1_at_AGFG1
MmugDNA.13269.1.S1_at	211685_s_at	NCALD	neurocalcin delta	chr8q22.2	83988	NCALD	neurocalcin delta	---	706856	MmugDNA.13269.1.S1_at_NCALD
MmugDNA.13270.1.S1_at	211686_s_at	MAK16	MAK16 homolog (S. cerevisiae)	chr8p12	84549	---	---	---	---	MmugDNA.13270.1.S1_at_MAK16
MmugDNA.13271.1.S1_at	216925_s_at	TAL1	T-cell acute lymphocytic leukemia 1	chr1p32	6886	---	---	---	---	MmugDNA.13271.1.S1_at_TAL1
MmugDNA.1327.1.S1_at	NA	NA	NA	NA	NA	NA	NA	NA	NA	MmugDNA.1327.1.S1_at_NA
MmugDNA.13272.1.S1_at	242751_at	---	---	---	---	---	---	---	---	MmugDNA.13272.1.S1_at_---
MmugDNA.13273.1.S1_at	211592_s_at	CACNA1C	calcium channel, voltage-dependent, L type, alpha 1C subunit	chr12p13.3	775	---	---	---	---	MmugDNA.13273.1.S1_at_CACNA1C

MmugDNA.13274.1.S1_at	1570065_at	---	---	---	---	---	---	---	---	MmugDNA.13274.1.S1_at_---
MmugDNA.13275.1.S1_at	204124_at	SLC34A2	solute carrier family 34 (sodium phosphate), member 2	chr4p15.3-p15.1	10568	SLC34A2	solute carrier family 34 (sodium phosphate), member 2	---	694217	MmugDNA.13275.1.S1_at_SLC34A2
MmugDNA.13276.1.S1_at	1562644_at	MTHFD2L	methylenetetrahydrofolate dehydrogenase (NADP+ dependent) 2-like	chr4q13.3	441024	---	---	---	---	MmugDNA.13276.1.S1_at_MTHFD2L
MmugDNA.13277.1.S1_at	231386_at	C2orf84	chromosome 2 open reading frame 84	chr2p23.3	653140	LOC704748	hypothetical protein LOC704748	---	704748	MmugDNA.13277.1.S1_at_C2orf84
MmugDNA.13278.1.S1_at	228571_at	RBAK	RB-associated KRAB zinc finger	chr7p22.1	57786	LOC716026	similar to RB-associated KRAB repressor	---	716026	MmugDNA.13278.1.S1_at_RBAK
MmugDNA.13279.1.S1_at	201932_at	LRRC41	leucine rich repeat containing 41	chr1p33	10489	LRRC41	similar to MUF1 protein	---	708437	MmugDNA.13279.1.S1_at_LRRC41
MmugDNA.13280.1.S1_at	1556607_at	EHD4	EH-domain containing 4	chr15q11.1	30844	---	---	---	---	MmugDNA.13280.1.S1_at_EHD4
MmugDNA.13281.1.S1_at	1556240_at	---	---	---	---	---	---	---	---	MmugDNA.13281.1.S1_at_---
MmugDNA.1328.1.S1_at	224794_s_at	CERCAM	cerebral endothelial cell adhesion molecule	chr9q34.11	51148	LOC717531	similar to cerebral endothelial cell adhesion molecule 1	---	717531	MmugDNA.1328.1.S1_at_CERCAM
MmugDNA.13282.1.S1_at	231401_s_at	---	---	---	---	---	---	---	---	MmugDNA.13282.1.S1_at_---
MmugDNA.13283.1.S1_at	234615_at	---	---	---	---	---	---	---	---	MmugDNA.13283.1.S1_at_---
MmugDNA.13284.1.S1_at	213250_at	CCDC85B	Coiled-coil domain containing 85B	chr11q12.1	11007	CCDC85B	coiled-coil domain containing 85B	---	715413	MmugDNA.13284.1.S1_at_CCDC85B
MmugDNA.13285.1.S1_at	209193_at	PIM1	pim-1 oncogene	chr6p21.2	5292	PIM1	pim-1 oncogene	---	719232	MmugDNA.13285.1.S1_at_PIM1
MmugDNA.13286.1.S1_at	203912_s_at	DNASE1L1	deoxyribonuclease I-like 1	chrXq28	1774	---	---	---	---	MmugDNA.13286.1.S1_at_DNASE1L1
MmugDNA.13288.1.S1_s_at	208746_x_at	ATP5L	ATP synthase, H+ transporting, mitochondrial F0 complex, subunit G	chr11q23.3	10632	ATP5L	ATP synthase, H+ transporting, mitochondrial F0 complex, subunit G	---	699950	MmugDNA.13288.1.S1_s_at_ATP5L
MmugDNA.13289.1.S1_at	226761_at	IKZF4	IKAROS family zinc finger 4 (Eos)	chr12q13	64375	---	---	---	---	MmugDNA.13289.1.S1_at_IKZF4
MmugDNA.13290.1.S1_at	226759_at	IKZF4	IKAROS family zinc finger 4 (Eos)	chr12q13	64375	---	---	---	---	MmugDNA.13290.1.S1_at_IKZF4
MmugDNA.13291.1.S1_at	1569240_at	ZNF93	zinc finger protein 93	chr19p12	81931	---	---	---	---	MmugDNA.13291.1.S1_at_ZNF93
MmugDNA.1329.1.S1_at	238233_at	---	---	---	---	---	---	---	---	MmugDNA.1329.1.S1_at_---
MmugDNA.13292.1.S1_at	1569808_at	---	---	---	---	---	---	---	---	MmugDNA.13292.1.S1_at_---
MmugDNA.13293.1.S1_at	221819_at	RAB35	RAB35, member RAS oncogene family	chr12q24.31	11021	LOC698400	hypothetical protein LOC698400	---	698400	MmugDNA.13293.1.S1_at_RAB35
MmugDNA.13294.1.S1_at	237740_at	---	---	---	---	---	---	---	---	MmugDNA.13294.1.S1_at_---
MmugDNA.13295.1.S1_at	236921_at	---	---	---	---	---	---	---	---	MmugDNA.13295.1.S1_at_---
MmugDNA.13296.1.S1_at	231460_at	---	---	---	---	---	---	---	---	MmugDNA.13296.1.S1_at_---
MmugDNA.13297.1.S1_at	242472_x_at	FNBP4	Formin binding protein 4	chr11p11.2	23360	---	---	---	---	MmugDNA.13297.1.S1_at_FNBP4
MmugDNA.13298.1.S1_at	213124_at	ZNF473	zinc finger protein 473	chr19q13.33	25888	---	---	---	---	MmugDNA.13298.1.S1_at_ZNF473
MmugDNA.13299.1.S1_at	231583_at	KRT74	keratin 74	chr12q13.13	121391	LOC699069	similar to keratin 6 irs4	---	699069	MmugDNA.13299.1.S1_at_KRT74
MmugDNA.13300.1.S1_s_at	1559502_s_at	LRRC25	leucine rich repeat containing 25	chr19p13.11	126364	LOC719475	similar to leucine rich repeat containing 25	---	719475	MmugDNA.13300.1.S1_s_at_LRRC25
MmugDNA.13301.1.S1_at	222977_at	SURF4	surfeit 4	chr9q34.2	6836	SURF1	surfeit 1	---	722212	MmugDNA.13301.1.S1_at_SURF4
MmugDNA.1330.1.S1_at	1569322_at	LOC439990	hypothetical gene supported by BC009626; BC048265	chr10q22.3	439990	---	---	---	---	MmugDNA.1330.1.S1_at_LOC439990
MmugDNA.13302.1.S1_at	1558971_at	TSEPA	thymocyte selection pathway associated	chr6q22.33	387357	LOC718091	Similar to 60S acidic ribosomal protein P0 (L10E)	---	718091	MmugDNA.13302.1.S1_at_TSEPA
MmugDNA.13303.1.S1_s_at	222978_at	SURF4	surfeit 4	chr9q34.2	6836	---	---	---	---	MmugDNA.13303.1.S1_s_at_SURF4
MmugDNA.13304.1.S1_at	205283_at	FKTN	fukutin	chr9q31-q33	2218	---	---	---	---	MmugDNA.13304.1.S1_at_FKTN
MmugDNA.13305.1.S1_at	1556256_a_at	---	---	---	---	---	---	---	---	MmugDNA.13305.1.S1_at_---
MmugDNA.13306.1.S1_at	202768_at	FOSB	FBJ murine osteosarcoma viral oncogene homolog B	chr19q13.32	2354	---	---	---	---	MmugDNA.13306.1.S1_at_FOSB
MmugDNA.13307.1.S1_at	1563721_at	---	---	---	---	---	---	---	---	MmugDNA.13307.1.S1_at_---
MmugDNA.13308.1.S1_at	207590_s_at	CENPI	centromere protein I	chrXq22.1	2491	CENPI	centromere protein I	---	702316	MmugDNA.13308.1.S1_at_CENPI
MmugDNA.13309.1.S1_at	1563816_at	GAB4	GRB2-associated binding protein family, member 4	chr22q11.1	128954	---	---	---	---	MmugDNA.13309.1.S1_at_GAB4
MmugDNA.13310.1.S1_at	233391_at	CDH26	cadherin-like 26	chr20q13.2-q13.33	60437	---	---	---	---	MmugDNA.13310.1.S1_at_CDH26
MmugDNA.13311.1.S1_at	234558_at	---	---	---	---	---	---	---	---	MmugDNA.13311.1.S1_at_---
MmugDNA.1331.1.S1_at	1554065_at	FAM125B	family with sequence similarity 125, member B	chr9q33.3	89853	---	---	---	---	MmugDNA.1331.1.S1_at_FAM125B
MmugDNA.13312.1.S1_at	225075_at	PDRG1	p53 and DNA damage regulated 1	chr20q11.21	81572	LOC712622	similar to p53 and DNA damage-regulated protein	---	712622	MmugDNA.13312.1.S1_at_PDRG1
MmugDNA.13313.1.S1_at	234343_s_at	RASAL2	RAS protein activator like 2	chr1q24	9462	LOC718958	hypothetical protein LOC718958	---	718958	MmugDNA.13313.1.S1_at_RASAL2
MmugDNA.13314.1.S1_at	235149_at	PGM2L1	phosphoglucomutase 2-like 1	chr11q13.4	283209	---	---	---	---	MmugDNA.13314.1.S1_at_PGM2L1
MmugDNA.13315.1.S1_at	1570402_at	KLC3	kinesin light chain 3	chr19q13.2-q13.3	147700	LOC714331	similar to kinesin light chain 3	---	714331	MmugDNA.13315.1.S1_at_KLC3
MmugDNA.13316.1.S1_at	214888_at	CAPN2	calpain 2, (m/II) large subunit	chr1q41-q42	824	---	---	---	---	MmugDNA.13316.1.S1_at_CAPN2
MmugDNA.13317.1.S1_at	1557488_at	LOC645158	hypothetical protein LOC645158	chr18p11.32	645158	---	---	---	---	MmugDNA.13317.1.S1_at_LOC645158
MmugDNA.13318.1.S1_at	242243_at	TMF1	TATA element modulatory factor 1	chr3p21-p12	7110	---	---	---	---	MmugDNA.13318.1.S1_at_TMF1
MmugDNA.13319.1.S1_at	230594_at	---	---	---	---	---	---	---	---	MmugDNA.13319.1.S1_at_---
MmugDNA.133.1.S1_at	207128_s_at	ZNF223	zinc finger protein 223	chr19q13.2	7766	LOC711740	similar to zinc finger protein 223	---	711740	MmugDNA.133.1.S1_at_ZNF223
MmugDNA.13320.1.S1_at	200805_at	LMAN2	lectin, mannose-binding 2	chr5q35.3	10960	LOC706346	similar to Vesicular integral-membrane protein VIP36 precursor (GP36b glycoprotein) (Lectin, mannos	---	706346	MmugDNA.13320.1.S1_at_LMAN2
MmugDNA.13321.1.S1_at	226532_at	---	---	---	---	---	---	---	---	MmugDNA.13321.1.S1_at_---
MmugDNA.1332.1.S1_at	240956_at	---	---	---	---	---	---	---	---	MmugDNA.1332.1.S1_at_---
MmugDNA.13322.1.S1_at	235459_at	---	---	---	---	---	---	---	---	MmugDNA.13322.1.S1_at_---
MmugDNA.13323.1.S1_at	237746_at	SFRS11	Splicing factor, arginine/serine-rich 11	chr1p31	9295	---	---	---	---	MmugDNA.13323.1.S1_at_SFRS11
MmugDNA.13324.1.S1_at	NA	NA	NA	NA	NA	NA	NA	NA	NA	MmugDNA.13324.1.S1_at_NA
MmugDNA.13325.1.S1_at	204370_at	CLP1	CLP1, cleavage and polyadenylation factor I subunit, homolog (S. cerevisiae)	chr11q12	10978	HEAB	ATP/GTP-binding protein	---	705071	MmugDNA.13325.1.S1_at_CLP1
MmugDNA.13326.1.S1_at	1566974_at	---	---	---	---	---	---	---	---	MmugDNA.13326.1.S1_at_---
MmugDNA.13327.1.S1_at	1553340_s_at	AMAC1 /// AMAC1L2	acyl-malonyl condensing enzyme 1 /// acyl-malonyl condensing enzyme 1-like 2	chr17q12 /// chr8p23-p22	146861 /// 83650	LOC715658	similar to acyl-malonyl condensing enzyme	---	715658	MmugDNA.13327.1.S1_at_AMAC1 /// A
MmugDNA.13328.1.S1_at	201451_x_at	RHEB	Ras homolog enriched in brain	chr7q36	6009	---	---	---	---	MmugDNA.13328.1.S1_at_RHEB
MmugDNA.13329.1.S1_at	209110_s_at	RGL2	ral guanine nucleotide dissociation stimulator-like 2	chr6p21.3	5863	LOC718198	similar to ral guanine nucleotide dissociation stimulator-like 2	---	718198	MmugDNA.13329.1.S1_at_RGL2
MmugDNA.13330.1.S1_at	232110_at	---	---	---	---	GALNT5	UDP-N-acetyl-alpha-D-galactosamine:polypeptide N-acetylgalactosaminyltransferase 5 (GalNAc-T5)	---	697430	MmugDNA.13330.1.S1_at_---
MmugDNA.13331.1.S1_at	216117_at	EXOSC2	exosome component 2	chr9q34	23404	EXOSC2	exosome component 2	---	715960	MmugDNA.13331.1.S1_at_EXOSC2
MmugDNA.1333.1.S1_at	1566927_at	C21orf104	chromosome 21 open reading frame 104	---	54748	---	---	---	---	MmugDNA.1333.1.S1_at_C21orf104
MmugDNA.13332.1.S1_at	240426_at	FAM47B	family with sequence similarity 47, member B	chrXp21.1	170062	LOC694109	similar to Protein FAM47B	---	694109	MmugDNA.13332.1.S1_at_FAM47B
MmugDNA.13333.1.S1_at	242067_at	---	---	---	---	---	---	---	---	MmugDNA.13333.1.S1_at_---
MmugDNA.13334.1.S1_at	233816_at	---	---	---	---	---	---	---	---	MmugDNA.13334.1.S1_at_---
MmugDNA.13335.1.S1_at	235798_at	TMEM170B	transmembrane protein 170B	chr6p24.1	100113407	---	---	---	---	MmugDNA.13335.1.S1_at_TMEM170B
MmugDNA.13336.1.S1_at	1559222_at	---	---	---	---	---	---	---	---	MmugDNA.13336.1.S1_at_---
MmugDNA.13337.1.S1_at	236279_at	---	---	---	---	---	---	---	---	MmugDNA.13337.1.S1_at_---
MmugDNA.13338.1.S1_at	204504_s_at	HIRIP3	HIRA interacting protein 3	chr16p11.2	8479	LOC719419	Similar to heterogeneous nuclear ribonucleoproteins methyltransferase-like 2	---	719419	MmugDNA.13338.1.S1_at_HIRIP3
MmugDNA.13339.1.S1_at	225127_at	TMEM181	transmembrane protein 181	chr6q25.3	57583	LOC705039	similar to G protein-coupled receptor 178	---	705039	MmugDNA.13339.1.S1_at_TMEM181
MmugDNA.13340.1.S1_at	237065_s_at	---	---	---	---	---	---	---	---	MmugDNA.13340.1.S1_at_---
MmugDNA.13341.1.S1_at	240159_at	SLC15A2	solute carrier family 15 (H+/peptide transporter), member 2	chr3q13.33	6565	---	---	---	---	MmugDNA.13341.1.S1_at_SLC15A2
MmugDNA.1334.1.S1_at	216220_s_at	ADORA1	adenosine A1 receptor	chr1q32.1	134	ADORA1	adenosine A1 receptor	---	703638	MmugDNA.1334.1.S1_at_ADORA1
MmugDNA.13342.1.S1_s_at	1555713_at	PNKD	paroxysmal nonkinesigenic dyskinesia	chr2q35	25953	---	---	---	---	MmugDNA.13342.1.S1_s_at_PNKD
MmugDNA.13343.1.S1_at	212336_at	EPB41L1	erythrocyte membrane protein band 4.1-like 1	chr20q11.2-q12	2036	---	---	---	---	MmugDNA.13343.1.S1_at_EPB41L1
MmugDNA.13344.1.S1_at	209074_s_at	FAM107A	family with sequence similarity 107, member A	chr3p21.1	11170	FAM107A	family with sequence similarity 107, member A	---	701455	MmugDNA.13344.1.S1_at_FAM107A
MmugDNA.13345.1.S1_at	214067_at	C16orf42	chromosome 16 open reading frame 42	chr16p13.3	115939	---	---	---	---	MmugDNA.13345.1.S1_at_C16orf42
MmugDNA.13346.1.S1_at	212144_at	UNC84B	unc-84 homolog B (C. elegans)	chr22q13.1	25777	---	---	---	---	MmugDNA.13346.1.S1_at_UNC84B
MmugDNA.13347.1.S1_at	229455_at	---	---	---	---	---	---	---	---	MmugDNA.13347.1.S1_at_---
MmugDNA.13348.1.S1_at	238376_at	---	---	---	---	---	---	---	---	MmugDNA.13348.1.S1_at_---
MmugDNA.13349.1.S1_at	224328_s_at	LCE3D	late cornified envelope 3D	chr1q21	84648	LOC713158	hypothetical protein LOC713158	---	713158	MmugDNA.13349.1.S1_at_LCE3D
MmugDNA.13350.1.S1_at	1555540_at	TGFB3	transforming growth factor, beta 3	chr14q24	7043	TGFB3	transforming growth factor, beta 3	---	703239	MmugDNA.13350.1.S1_at_TGFB3
MmugDNA.13351.1.S1_at	1556725_a_at	LOC100128398	Hypothetical protein LOC100128398	chr19q13.43	100128398	---	---	---	---	MmugDNA.13351.1.S1_at_LOC100128
MmugDNA.1335.1.S1_at	229967_at	CMTM2	CKLF-like MARVEL transmembrane domain containing 2	chr16q21	146225	CMTM2	CKLF-like MARVEL transmembrane domain containing 2	---	693810	MmugDNA.1335.1.S1_at_CMTM2
MmugDNA.13352.1.S1_at	NA	NA	NA	NA	NA	NA	NA	NA	NA	MmugDNA.13352.1.S1_at_NA
MmugDNA.13352.1.S1_s_at	212815_at	ASCC3	activating signal cointegrator 1 complex subunit 3	chr6q16.1-q16.3	10973	LOC695429	similar to activating signal cointegrator 1 complex subunit 3 isoform a	---	695429	MmugDNA.13352.1.S1_s_at_ASCC3
MmugDNA.13353.1.S1_at	1554553_s_at	YIF1B	Yip1 interacting factor homolog B (S. cerevisiae)	chr19q13.2	90522	LOC719551	similar to Yip1 interacting factor homolog B isoform 2	---	719551	MmugDNA.13353.1.S1_at_YIF1B
MmugDNA.13354.1.S1_at	235885_at	P2RY12	purinergic receptor P2Y, G-protein coupled, 12	chr3q24-q25	64805	LOC710036	similar to purinergic receptor P2Y12	---	710036	MmugDNA.13354.1.S1_at_P2RY12
MmugDNA.13355.1.S1_at	240316_at	C9orf57	chromosome 9 open reading frame 57	chr9q21.13	138240	---	---	---	---	MmugDNA.13355.1.S1_at_C9orf57
MmugDNA.13356.1.S1_at	201652_at	COPS5	COP9 constitutive photomorphogenic homolog subunit 5 (Arabidopsis)	chr8q13.2	10987	COPS5	COP9 constitutive photomorphogenic homolog subunit 5 (Arabidopsis)	---	704584	MmugDNA.13356.1.S1_at_COPS5
MmugDNA.13357.1.S1_at	200922_at	KDELR1	KDEL (Lys-Asp-Glu-Leu) endoplasmic reticulum protein retention receptor 1	chr19q13.3	10945	KDELR1	KDEL (Lys-Asp-Glu-Leu) endoplasmic reticulum protein retention receptor 1	---	717928	MmugDNA.13357.1.S1_at_KDELR1
MmugDNA.13358.1.S1_at	234645_at	---	---	---	---	---	---	---	---	MmugDNA.13358.1.S1_at_---
MmugDNA.13359.1.S1_at	214104_at	GPR161	G protein-coupled receptor 161	chr1q24.2	23432	GPR161	G protein-coupled receptor 161	---	698934	MmugDNA.13359.1.S1_at_GPR161
MmugDNA.13360.1.S1_at	1555360_a_at	DNAJC11	DnaJ (Hsp40) homolog, subfamily C, member 11	chr1p36.31	55735	DNAJC11	DnaJ (Hsp40) homolog, subfamily C, member 11	---	705057	MmugDNA.13360.1.S1_at_DNAJC11
MmugDNA.13361.1.S1_at	238706_at	PAPD4	PAP associated domain containing 4	chr5q14.1	167153	LOC710044	similar to PAP associated domain containing 4	---	710044	MmugDNA.13361.1.S1_at_PAPD4
MmugDNA.1336.1.S1_at	226413_at	LOC400027	hypothetical protein LOC400027	chr12q12	400027	---	---	---	---	MmugDNA.1336.1.S1_at_LOC400027
MmugDNA.13363.1.S1_at	238899_at	---	---	---	---	---	---	---	---	MmugDNA.13363.1.S1_at_---
MmugDNA.13364.1.S1_at	243678_at	---	---	---	---	---	---	---	---	MmugDNA.13364.1.S1_at_---
MmugDNA.13365.1.S1_at	1560325_at	---	---	---	---	---	---	---	---	MmugDNA.13365.1.S1_at_---
MmugDNA.13366.1.S1_at	209791_at	PADI2	peptidyl arginine deiminase, type II	chr1p36.13	11240	PADI2	peptidyl arginine deiminase, type II	---	711044	MmugDNA.13366.1.S1_at_PADI2
MmugDNA.13368.1.S1_at	1562462_at	DNAH10	dynein, axonemal, heavy chain 10	chr12q24.31	196385	LOC710343	similar to Dynein heavy chain at 89D CG1842-PA	---	710343	MmugDNA.13368.1.S1_at_DNAH10
MmugDNA.13369.1.S1_at	241651_at	---	---	---	---	---	---	---	---	MmugDNA.13369.1.S1_at_---
MmugDNA.13370.1.S1_at	222172_at	NPAS3	neuronal PAS domain protein 3	chr14q12-q13	64067	---	---	---	---	MmugDNA.13370.1.S1_at_NPAS3
MmugDNA.13371.1.S1_at	1557598_at	---	---	---	---	---	---	---	---	MmugDNA.13371.1.S1_at_---
MmugDNA.1337.1.S1_at	214141_x_at	SFRS7	splicing factor, arginine/serine-rich 7, 35kDa	chr2p22.1	6432	LOC695281 /// LOC6984	Similar to FYVE, RhoGEF and PH domain containing 4 /// Hypothetical protein LOC698436	---	695281 /// 698436	MmugDNA.1337.1.S1_at_SFRS7
MmugDNA.13372.1.S1_at	231621_at	MPZL1	myelin protein zero-like 1	chr1q24.2	9019	---	---	---	---	MmugDNA.13372.1.S1_at_MPZL1

MmugDNA.13373.1.S1_at	234990_at	CBX5	chromobox homolog 5 (HP1 alpha homolog, Drosophila)	chr12q13.13		23468	LOC703477	similar to Chromobox protein homolog 5 (Heterochromatin protein 1 homolog alpha) (HP1 alpha) (Ant	---		703477	MmugDNA.13373.1.S1_at_CBX5
MmugDNA.13374.1.S1_at	214857_at	---	---	---	---		---	---	---	---		MmugDNA.13374.1.S1_at_---
MmugDNA.13375.1.S1_at	208325_s_at	AKAP13	A kinase (PRKA) anchor protein 13	chr15q24-q25		11214	LOC709502	Similar to junction-mediating and regulatory protein	---		709502	MmugDNA.13375.1.S1_at_AKAP13
MmugDNA.13376.1.S1_at	236591_at	---	---	---	---		---	---	---	---		MmugDNA.13376.1.S1_at_---
MmugDNA.13377.1.S1_at	233579_at	---	---	---	---		---	---	---	---		MmugDNA.13377.1.S1_at_---
MmugDNA.13378.1.S1_at	237679_at	TRIM66	tripartite motif-containing 66	chr11p15.4		9866	---	---	---	---		MmugDNA.13378.1.S1_at_TRIM66
MmugDNA.13379.1.S1_at	217295_at	MUC8	mucin 8	chr12q24.3		4590	---	---	---	---		MmugDNA.13379.1.S1_at_MUC8
MmugDNA.13380.1.S1_at	227411_at	WTIP	Wilms tumor 1 interacting protein	chr19q13.11		126374	LOC717999	similar to WT1-interacting protein	---		717999	MmugDNA.13380.1.S1_at_WTIP
MmugDNA.13381.1.S1_at	232825_s_at	DSEL	dermatan sulfate epimerase-like	chr18q22.1		92126	LOC709898	similar to squamous cell carcinoma antigen recognized by T cells 2	---		709898	MmugDNA.13381.1.S1_at_DSEL
MmugDNA.1338.1.S1_at	240012_at	---	---	---	---		---	---	---	---		MmugDNA.1338.1.S1_at_---
MmugDNA.13382.1.S1_at	229337_at	USP2	ubiquitin specific peptidase 2	chr11q23.3		9099	USP2	ubiquitin specific peptidase 2	---		705191	MmugDNA.13382.1.S1_at_USP2
MmugDNA.13383.1.S1_at	240979_at	LOC284100	Hypothetical protein LOC284100	chr17q12		284100	---	---	---	---		MmugDNA.13383.1.S1_at_LOC284100
MmugDNA.13384.1.S1_at	NA	NA	NA	NA	NA		NA	NA	NA	NA		MmugDNA.13384.1.S1_at_NA
MmugDNA.13385.1.S1_at	243889_at	---	---	---	---		---	---	---	---		MmugDNA.13385.1.S1_at_---
MmugDNA.13386.1.S1_at	226033_at	USP31	ubiquitin specific peptidase 31	chr16p12.1		57478	LOC704663	Similar to amine oxidase, flavin containing 1	---		704663	MmugDNA.13386.1.S1_at_USP31
MmugDNA.13387.1.S1_at	213450_s_at	ICOSLG	inducible T-cell co-stimulator ligand	chr21q22.3		23308	---	---	---	---		MmugDNA.13387.1.S1_at_ICOSLG
MmugDNA.13388.1.S1_at	237887_at	---	---	---	---		---	---	---	---		MmugDNA.13388.1.S1_at_---
MmugDNA.13389.1.S1_s_at	209131_s_at	SNAP23	synaptosomal-associated protein, 23kDa	chr15q15.1		8773	---	---	---	---		MmugDNA.13389.1.S1_s_at_SNAP23
MmugDNA.13390.1.S1_at	235486_at	C11orf41	chromosome 11 open reading frame 41	chr11p13		25758	LOC713533	Similar to selenophosphate synthetase	---		713533	MmugDNA.13390.1.S1_at_C11orf41
MmugDNA.13391.1.S1_at	206881_s_at	LILRA3	leukocyte immunoglobulin-like receptor, subfamily A (without TM domain), member 3	chr19q13.4		11026	LILRAD	leukocyte immunoglobulin-like receptor, subfamily A, member d	---		692337	MmugDNA.13391.1.S1_at_LILRA3
MmugDNA.1339.1.S1_at	222513_s_at	SORBS1	sorbin and SH3 domain containing 1	chr10q23.3-q24.1		10580	---	---	---	---		MmugDNA.1339.1.S1_at_SORBS1
MmugDNA.13392.1.S1_at	227793_at	---	---	---	---		---	---	---	---		MmugDNA.13392.1.S1_at_---
MmugDNA.13393.1.S1_at	203123_s_at	SLC11A2	solute carrier family 11 (proton-coupled divalent metal ion transporters), member 2	chr12q13		4891	---	---	---	---		MmugDNA.13393.1.S1_at_SLC11A2
MmugDNA.13394.1.S1_at	207872_s_at	LILRA1	leukocyte immunoglobulin-like receptor, subfamily A (with TM domain), member 1	chr19q13.4		11024	LILRA1 /// LILRAA	leukocyte immunoglobulin-like receptor, subfamily A (with TM domain), member 1 /// leukocyte immur	---	692342 /// 696552		MmugDNA.13394.1.S1_at_LILRA1
MmugDNA.13395.1.S1_at	NA	NA	NA	NA	NA		NA	NA	NA	NA		MmugDNA.13395.1.S1_at_NA
MmugDNA.13396.1.S1_at	1556192_x_at	---	---	---	---		---	---	---	---		MmugDNA.13396.1.S1_at_---
MmugDNA.13397.1.S1_x_at	206856_at	LILRB5	leukocyte immunoglobulin-like receptor, subfamily B (with TM and ITIM domains), member 5	chr19q13.4		10990	LOC722774	similar to Leukocyte immunoglobulin-like receptor subfamily B member 3 precursor (Leukocyte immur	---		722774	MmugDNA.13397.1.S1_x_at_LILRB5
MmugDNA.13398.1.S1_at	208209_s_at	C4BPB	complement component 4 binding protein, beta	chr1q32		725	C4BPB	complement component 4 binding protein, beta	---		693636	MmugDNA.13398.1.S1_at_C4BPB
MmugDNA.13399.1.S1_at	NA	NA	NA	NA	NA		NA	NA	NA	NA		MmugDNA.13399.1.S1_at_NA
MmugDNA.13400.1.S1_at	NA	NA	NA	NA	NA		NA	NA	NA	NA		MmugDNA.13400.1.S1_at_NA
MmugDNA.13400.1.S1_s_at	209240_at	OGT	O-linked N-acetylglucosamine (GlcNAc) transferase (UDP-N-acetylglucosamine:polypeptide-N-acetyl	chrXq13		8473	LOC717115	Similar to RNA-binding region (RNP1, RRM) containing 3	---		717115	MmugDNA.13400.1.S1_s_at_OGT
MmugDNA.13401.1.S1_s_at	224567_x_at	MALAT1	metastasis associated lung adenocarcinoma transcript 1 (non-protein coding)	chr11q13.1		378938	---	---	---	---		MmugDNA.13401.1.S1_s_at_MALAT1
MmugDNA.1340.1.S1_at	227802_at	RUFY3	RUN and FYVE domain containing 3	chr4q13.3		22902	---	---	---	---		MmugDNA.1340.1.S1_at_RUFY3
MmugDNA.13402.1.S1_at	236465_at	RNF175	ring finger protein 175	chr4q31.3		285533	RNF175	ring finger protein 175	---		697243	MmugDNA.13402.1.S1_at_RNF175
MmugDNA.13403.1.S1_at	220088_at	C5AR1	complement component 5a receptor 1	chr19q13.3-q13.4		728	C5AR1	complement component 5a receptor 1	---		718056	MmugDNA.13403.1.S1_at_C5AR1
MmugDNA.13404.1.S1_at	242535_at	---	---	---	---		---	---	---	---		MmugDNA.13404.1.S1_at_---
MmugDNA.13405.1.S1_at	214834_at	PAR5	Prader-Willi/Angelman syndrome-5	chr15q11-q13		8123	---	---	---	---		MmugDNA.13405.1.S1_at_PAR5
MmugDNA.13406.1.S1_at	215315_at	ZNF549	zinc finger protein 549	chr19q13.43		256051	ZNF304	similar to zinc finger protein 549	---		708201	MmugDNA.13406.1.S1_at_ZNF549
MmugDNA.13407.1.S1_at	244538_at	KCTD19	potassium channel tetramerisation domain containing 19	chr16q22.1		146212	KCTD19	potassium channel tetramerisation domain containing 19	---		699297	MmugDNA.13407.1.S1_at_KCTD19
MmugDNA.13408.1.S1_at	222490_at	POLR3E	polymerase (RNA) III (DNA directed) polypeptide E (80kD)	chr16p12.1		55718	POLR3E	polymerase (RNA) III (DNA directed) polypeptide E	---		697901	MmugDNA.13408.1.S1_at_POLR3E
MmugDNA.13409.1.S1_at	1561273_at	---	---	---	---		---	---	---	---		MmugDNA.13409.1.S1_at_---
MmugDNA.13410.1.S1_at	237584_at	---	---	---	---		---	---	---	---		MmugDNA.13410.1.S1_at_---
MmugDNA.13411.1.S1_at	1558166_at	MGC16275	hypothetical protein MGC16275	chr17q25.2		85001	---	---	---	---		MmugDNA.13411.1.S1_at_MGC16275
MmugDNA.1341.1.S1_at	228044_at	SERP2	stress-associated endoplasmic reticulum protein family member 2	chr13q14.11		387923	LOC705398	similar to ribosome associated membrane protein 4	---		705398	MmugDNA.1341.1.S1_at_SERP2
MmugDNA.13412.1.S1_at	230524_at	MPI	mannose phosphate isomerase	chr15q22-qter		4351	---	---	---	---		MmugDNA.13412.1.S1_at_MPI
MmugDNA.13413.1.S1_at	231993_at	ITGBL1	Integrin, beta-like 1 (with EGF-like repeat domains)	chr13q33		9358	---	---	---	---		MmugDNA.13413.1.S1_at_ITGBL1
MmugDNA.13414.1.S1_at	1561331_at	C1orf99	chromosome 1 open reading frame 99	chr1q31.1		339476	---	---	---	---		MmugDNA.13414.1.S1_at_C1orf99
MmugDNA.13415.1.S1_at	1563943_at	PPARGC1B	peroxisome proliferator-activated receptor gamma, coactivator 1 beta	chr5q33.1		133522	LOC711027	similar to PGC-1-related estrogen receptor alpha coactivator	---		711027	MmugDNA.13415.1.S1_at_PPARGC1B
MmugDNA.13415.1.S1_s_at	NA	NA	NA	NA	NA		NA	NA	NA	NA		MmugDNA.13415.1.S1_s_at_NA
MmugDNA.13416.1.S1_at	1559849_at	ZNF605	zinc finger protein 605	chr12q24.33		90462	---	---	---	---		MmugDNA.13416.1.S1_at_ZNF605
MmugDNA.13417.1.S1_at	224329_s_at	CNFN	cornifelin	chr19q13.2		84518	LOC722232	similar to cornifelin	---		722232	MmugDNA.13417.1.S1_at_CNFN
MmugDNA.13418.1.S1_at	203412_at	LZTR1	leucine-zipper-like transcription regulator 1	chr22q11.21|22q11.1-q11.2		8216	LZTR1	leucine-zipper-like transcription regulator 1	---		696330	MmugDNA.13418.1.S1_at_LZTR1
MmugDNA.13419.1.S1_at	237398_at	---	---	---	---		---	---	---	---		MmugDNA.13419.1.S1_at_---
MmugDNA.134.1.S1_at	226909_at	ZNF518B	zinc finger protein 518B	chr4p16.1		85460	LOC713122	similar to pleiomorphic adenoma gene-like 1	---		713122	MmugDNA.134.1.S1_at_ZNF518B
MmugDNA.13420.1.S1_at	203359_s_at	MYCBP	c-myc binding protein	chr1p33-p32.2		26292	LOC709242	Similar to phosphatidylethanolamine-binding protein 4	---		709242	MmugDNA.13420.1.S1_at_MYCBP
MmugDNA.13421.1.S1_at	204680_s_at	RAPGEF5	Rap guanine nucleotide exchange factor (GEF) 5	chr7p15.3		9771	LOC711698	similar to Rap guanine nucleotide exchange factor 5 (Guanine nucleotide exchange factor for Rap1) (	---		711698	MmugDNA.13421.1.S1_at_RAPGEF5
MmugDNA.1342.1.S1_at	234315_at	BACH1	BTB and CNC homology 1, basic leucine zipper transcription factor 1	chr21q22.11		571	---	---	---	---		MmugDNA.1342.1.S1_at_BACH1
MmugDNA.13422.1.S1_at	205819_at	MARCO	macrophage receptor with collagenous structure	chr2q12-q13		8685	MARCO	macrophage receptor with collagenous structure	---		694432	MmugDNA.13422.1.S1_at_MARCO
MmugDNA.13423.1.S1_at	221654_s_at	USP3	ubiquitin specific peptidase 3	chr15q22.3		9960	LOC696998	Hypothetical protein LOC696998	---		696998	MmugDNA.13423.1.S1_at_USP3
MmugDNA.13424.1.S1_a_at	NA	NA	NA	NA	NA		NA	NA	NA	NA		MmugDNA.13424.1.S1_a_at_NA
MmugDNA.13424.1.S1_at	232332_at	KIAA1210	KIAA1210	chrXq24		57481	USP21	Ubiquitin specific peptidase 21	---		719894	MmugDNA.13424.1.S1_at_KIAA1210
MmugDNA.13425.1.S1_at	1563452_at	AVL9	AVL9 homolog (S. cerevisiase)	chr7p14.3		23080	---	---	---	---		MmugDNA.13425.1.S1_at_AVL9
MmugDNA.13426.1.S1_at	229585_at	ADAMTSL1	ADAMTS-like 1	chr9p22.2-p22.1		92949	LOC712844	similar to ADAMTS-like 3	---		712844	MmugDNA.13426.1.S1_at_ADAMTSL1
MmugDNA.13427.1.S1_at	233526_at	---	---	---	---		---	---	---	---		MmugDNA.13427.1.S1_at_---
MmugDNA.13428.1.S1_at	236563_at	RD3	retinal degeneration 3	chr1q32.3		343035	---	---	---	---		MmugDNA.13428.1.S1_at_RD3
MmugDNA.13429.1.S1_at	228992_at	MED28	Mediator complex subunit 28	chr4p16		80306	---	---	---	---		MmugDNA.13429.1.S1_at_MED28
MmugDNA.13430.1.S1_at	215528_at	---	---	---	---		---	---	---	---		MmugDNA.13430.1.S1_at_---
MmugDNA.13431.1.S1_at	1553460_at	C2orf61	chromosome 2 open reading frame 61	chr2p21		285051	---	---	---	---		MmugDNA.13431.1.S1_at_C2orf61
MmugDNA.1343.1.S1_at	1556789_a_at	---	---	---	---		---	---	---	---		MmugDNA.1343.1.S1_at_---
MmugDNA.13432.1.S1_at	207421_at	CA5A	carbonic anhydrase VA, mitochondrial	chr16q24.3		763	LOC705278	similar to carbonic anhydrase VA, mitochondrial precursor	---		705278	MmugDNA.13432.1.S1_at_CA5A
MmugDNA.13433.1.S1_at	1553708_at	MGC16075	hypothetical protein MGC16075	chr7p12.3		84847	---	---	---	---		MmugDNA.13433.1.S1_at_MGC16075
MmugDNA.13434.1.S1_at	208071_s_at	LAIR1	leukocyte-associated immunoglobulin-like receptor 1	chr19q13.4		3903	LAIR1 /// LOC696069	similar to leukocyte-associated immunoglobulin-like receptor 1 isoform a /// similar to leukocyte-assoc	---	695533 /// 696069		MmugDNA.13434.1.S1_at_LAIR1
MmugDNA.13435.1.S1_at	208020_s_at	CACNA1C	calcium channel, voltage-dependent, L type, alpha 1C subunit	chr12p13.3		775	CACNA1C	calcium channel, voltage-dependent, L type, alpha 1C subunit	---		721721	MmugDNA.13435.1.S1_at_CACNA1C
MmugDNA.13436.1.S1_s_at	202991_at	STARD3	StAR-related lipid transfer (START) domain containing 3	chr17q11-q12		10948	STARD3	StAR-related lipid transfer (START) domain containing 3	---		697212	MmugDNA.13436.1.S1_s_at_STARD3
MmugDNA.13437.1.S1_at	241601_at	WIPF3	WAS/WASL interacting protein family, member 3	chr7p15.1		644150	WIPF3	WAS/WASL interacting protein family, member 3	---		696503	MmugDNA.13437.1.S1_at_WIPF3
MmugDNA.13438.1.S1_at	235736_at	---	---	---	---		LOC701685	similar to CG11670-PA	---		701685	MmugDNA.13438.1.S1_at_---
MmugDNA.13439.1.S1_at	207998_s_at	CACNA1D	calcium channel, voltage-dependent, L type, alpha 1D subunit	chr3p14.3		776	LOC693924	similar to calcium channel, voltage-dependent, L type, alpha 1D subunit	---		693924	MmugDNA.13439.1.S1_at_CACNA1D
MmugDNA.13440.1.S1_at	229970_at	KBTBD7	kelch repeat and BTB (POZ) domain containing 7	chr13q14.11		84078	KBTBD7	kelch repeat and BTB (POZ) domain containing 7	---		698337	MmugDNA.13440.1.S1_at_KBTBD7
MmugDNA.13441.1.S1_at	239536_at	---	---	---	---		LOC713638	Similar to S100 calcium binding protein A16	---		713638	MmugDNA.13441.1.S1_at_---
MmugDNA.1344.1.S1_at	230331_at	C13orf39	chromosome 13 open reading frame 39	chr13q33.1		196541	LOC701446	similar to RIKEN cDNA 4832428D23	---		701446	MmugDNA.1344.1.S1_at_C13orf39
MmugDNA.13442.1.S1_at	213106_at	ATP8A1	ATPase, aminophospholipid transporter (APLT), class I, type 8A, member 1	chr4p14-p12		10396	SERPINA5	Serpin peptidase inhibitor, clade A (alpha-1 antiproteinase, antitrypsin), member 5	---		702078	MmugDNA.13442.1.S1_at_ATP8A1
MmugDNA.13443.1.S1_at	208432_s_at	CACNA1E	calcium channel, voltage-dependent, R type, alpha 1E subunit	chr1q25-q31		777	CACNA1E	calcium channel, voltage-dependent, R type, alpha 1E subunit	---		718092	MmugDNA.13443.1.S1_at_CACNA1E
MmugDNA.13444.1.S1_at	210761_s_at	GRB7	growth factor receptor-bound protein 7	chr17q12		2886	LOC697951	similar to growth factor receptor-bound protein 7	---		697951	MmugDNA.13444.1.S1_at_GRB7
MmugDNA.13445.1.S1_at	1558820_a_at	C18orf34	chromosome 18 open reading frame 34	chr18q12.1		374864	C18H18orf34	similar to CLIP-190 CG5020-PA, isoform A	---		707566	MmugDNA.13445.1.S1_at_C18orf34
MmugDNA.13446.1.S1_at	234238_at	MAP1LC3B2	microtubule-associated protein 1 light chain 3 beta 2	chr12q24.22		643246	---	---	---	---		MmugDNA.13446.1.S1_at_MAP1LC3B2
MmugDNA.13447.1.S1_at	226271_at	GDAP1	ganglioside-induced differentiation-associated protein 1	chr8q21.11		54332	GDAP1	ganglioside-induced differentiation-associated protein 1	---		697136	MmugDNA.13447.1.S1_at_GDAP1
MmugDNA.13448.1.S1_at	226269_at	GDAP1	ganglioside-induced differentiation-associated protein 1	chr8q21.11		54332	GDAP1	ganglioside-induced differentiation-associated protein 1	---		697136	MmugDNA.13448.1.S1_at_GDAP1
MmugDNA.13449.1.S1_at	237183_at	GALNT5	UDP-N-acetyl-alpha-D-galactosamine:polypeptide N-acetylgalactosaminyltransferase 5 (GalNAc-T5)	chr2q24.1		11227	GALNT5	UDP-N-acetyl-alpha-D-galactosamine:polypeptide N-acetylgalactosaminyltransferase 5 (GalNAc-T5)	---		697430	MmugDNA.13449.1.S1_at_GALNT5
MmugDNA.13450.1.S1_at	1565162_s_at	MGST1	microsomal glutathione S-transferase 1	chr12p12.3-p12.1		4257	MGST1	microsomal glutathione S-transferase 1	---		701652	MmugDNA.13450.1.S1_at_MGST1
MmugDNA.13451.1.S1_at	215494_at	---	---	---	---		---	---	---	---		MmugDNA.13451.1.S1_at_---
MmugDNA.1345.1.S1_at	NA	NA	NA	NA	NA		NA	NA	NA	NA		MmugDNA.1345.1.S1_at_NA
MmugDNA.1345.1.S1_s_at	NA	NA	NA	NA	NA		NA	NA	NA	NA		MmugDNA.1345.1.S1_s_at_NA
MmugDNA.13452.1.S1_at	203363_s_at	KIAA0652	KIAA0652	chr11p11.2		9776	LOC714322	similar to CG7331-PA	---		714322	MmugDNA.13452.1.S1_at_KIAA0652
MmugDNA.13453.1.S1_at	222861_x_at	FBXO44	F-box protein 44	chr1p36.22		93611	---	---	---	---		MmugDNA.13453.1.S1_at_FBXO44
MmugDNA.13454.1.S1_at	244858_at	LOC100129041 /// TGIF	hypothetical protein LOC100129041 /// TGFB-induced factor homeobox 1	chr18p11.3 /// chr18p11.31	100129041 /// 7050		LOC713968	Similar to 60S ribosomal protein L23a	---		713968	MmugDNA.13454.1.S1_at_LOC100129(
MmugDNA.13455.1.S1_at	211596_s_at	LRIG1	leucine-rich repeats and immunoglobulin-like domains 1	chr3p14		26018	LRIG1	leucine-rich repeats and immunoglobulin-like domains 1	---		700033	MmugDNA.13455.1.S1_at_LRIG1
MmugDNA.13456.1.S1_at	239646_at	---	---	---	---		---	---	---	---		MmugDNA.13456.1.S1_at_---
MmugDNA.13457.1.S1_at	235701_at	---	---	---	---		---	---	---	---		MmugDNA.13457.1.S1_at_---
MmugDNA.13458.1.S1_at	1556121_at	NAP1L1	nucleosome assembly protein 1-like 1	chr12q21.2		4673	---	---	---	---		MmugDNA.13458.1.S1_at_NAP1L1
MmugDNA.13458.1.S1_s_at	NA	NA	NA	NA	NA		NA	NA	NA	NA		MmugDNA.13458.1.S1_s_at_NA
MmugDNA.13459.1.S1_at	244146_at	DTWD1	DTW domain containing 1	chr15q21.2		56986	---	---	---	---		MmugDNA.13459.1.S1_at_DTWD1
MmugDNA.13460.1.S1_at	NA	NA	NA	NA	NA		NA	NA	NA	NA		MmugDNA.13460.1.S1_at_NA
MmugDNA.13460.1.S1_s_at	211439_at	SFRS7	splicing factor, arginine/serine-rich 7, 35kDa	chr2p22.1		6432	LOC695281 /// LOC6984	Similar to FYVE, RhoGEF and PH domain containing 4 /// Hypothetical protein LOC698436	---	695281 /// 698436		MmugDNA.13460.1.S1_s_at_SFRS7
MmugDNA.1346.1.S1_at	1557838_at	---	---	---	---		---	---	---	---		MmugDNA.1346.1.S1_at_---
MmugDNA.13462.1.S1_at	227338_at	LOC440983	hypothetical gene supported by BC066916	chr3q25.1		440983	---	---	---	---		MmugDNA.13462.1.S1_at_LOC440983
MmugDNA.13463.1.S1_at	201405_s_at	COPS6	COP9 constitutive photomorphogenic homolog subunit 6 (Arabidopsis)	chr7q22.1		10980	LOC710667	similar to COP9 signalosome subunit 6	---		710667	MmugDNA.13463.1.S1_at_COPS6
MmugDNA.13465.1.S1_s_at	207776_s_at	CACNB2	calcium channel, voltage-dependent, beta 2 subunit	chr10p12		783	CACNB2	calcium channel, voltage-dependent, beta 2 subunit	---		701226	MmugDNA.13465.1.S1_s_at_CACNB2
MmugDNA.13466.1.S1_at	203952_at	ATF6	activating transcription factor 6	chr1q22-q23		22926	LOC720056	similar to activating transcription factor 6	---		720056	MmugDNA.13466.1.S1_at_ATF6

MmugDNA.13467.1.S1_at	212714_at	LARP4	La ribonucleoprotein domain family, member 4	chr12q13.13		113251	---	---	---	---		MmugDNA.13467.1.S1_at_LARP4
MmugDNA.13468.1.S1_at	210829_s_at	SSBP2	single-stranded DNA binding protein 2	chr5q14.1		23635	ZDHHC4	Zinc finger, DHHC-type containing 4	---		704660	MmugDNA.13468.1.S1_at_SSBP2
MmugDNA.13469.1.S1_at	238347_at	SLC38A10	solute carrier family 38, member 10	chr17q25.3		124565	LOC713556	similar to CG30394-PB, isoform B	---		713556	MmugDNA.13469.1.S1_at_SLC38A10
MmugDNA.13470.1.S1_at	238362_at	---	---	---	---		---	---	---	---		MmugDNA.13470.1.S1_at_---
MmugDNA.13471.1.S1_at	235053_at	---	---	---	---		---	---	---	---		MmugDNA.13471.1.S1_at_---
MmugDNA.1347.1.S1_at	239627_at	TMED9	Transmembrane emp24 protein transport domain containing 9	chr5q35.3		54732	---	---	---	---		MmugDNA.1347.1.S1_at_TMED9
MmugDNA.13472.1.S1_at	1554202_x_at	CABP4	calcium binding protein 4	chr11q13.1		57010	CABP4	calcium binding protein 4	---		711973	MmugDNA.13472.1.S1_at_CABP4
MmugDNA.13473.1.S1_at	232482_at	OR51E2	olfactory receptor, family 51, subfamily E, member 2	chr11p15		81285	LOC717504	similar to olfactory receptor, family 51, subfamily E, member 2	---		717504	MmugDNA.13473.1.S1_at_OR51E2
MmugDNA.13474.1.S1_at	1557280_s_at	---	---	---	---		---	---	---	---		MmugDNA.13474.1.S1_at_---
MmugDNA.13475.1.S1_at	1555981_at	C17orf65	chromosome 17 open reading frame 65	chr17q21.31		339201	---	---	---	---		MmugDNA.13475.1.S1_at_C17orf65
MmugDNA.13476.1.S1_at	1554994_at	RAG1	recombination activating gene 1	chr11p13		5896	---	---	---	---		MmugDNA.13476.1.S1_at_RAG1
MmugDNA.13477.1.S1_at	215442_s_at	TSHR	thyroid stimulating hormone receptor	chr14q31		7253	TSHR	thyroid stimulating hormone receptor	---		574251	MmugDNA.13477.1.S1_at_TSHR
MmugDNA.13478.1.S1_at	225811_at	C11orf58	chromosome 11 open reading frame 58	chr11p15.1		10944	---	---	---	---		MmugDNA.13478.1.S1_at_C11orf58
MmugDNA.13479.1.S1_at	1556781_at	---	---	---	---		---	---	---	---		MmugDNA.13479.1.S1_at_---
MmugDNA.13480.1.S1_at	1559124_at	LOC644135	hypothetical LOC644135	chr6q23.3		644135	---	---	---	---		MmugDNA.13480.1.S1_at_LOC644135
MmugDNA.13481.1.S1_at	208986_at	TCF12	transcription factor 12	chr15q21		6938	LOC698341	Similar to ADAM metallopeptidase with thrombospondin type 1 motif, 9 preproprotein	---		698341	MmugDNA.13481.1.S1_at_TCF12
MmugDNA.1348.1.S1_at	240502_at	---	---	---	---		LOC716872	Similar to homolog of yeast MON2	---		716872	MmugDNA.1348.1.S1_at_---
MmugDNA.13482.1.S1_at	208764_s_at	ATP5G2	ATP synthase, H+ transporting, mitochondrial F0 complex, subunit C2 (subunit 9)	chr12q13.13		517	ATP5G2	ATP synthase, H+ transporting, mitochondrial F0 complex, subunit C2 (subunit 9)	---		703751	MmugDNA.13482.1.S1_at_ATP5G2
MmugDNA.13483.1.S1_at	228412_at	LOC643072	hypothetical LOC643072	chr2q24.2		643072	---	---	---	---		MmugDNA.13483.1.S1_at_LOC643072
MmugDNA.13484.1.S1_at	226234_at	GDF11	growth differentiation factor 11	chr12q13.2		10220	---	---	---	---		MmugDNA.13484.1.S1_at_GDF11
MmugDNA.13485.1.S1_s_at	226232_at	GDF11	growth differentiation factor 11	chr12q13.2		10220	---	---	---	---		MmugDNA.13485.1.S1_s_at_GDF11
MmugDNA.13486.1.S1_at	204814_at	CADPS	Ca++-dependent secretion activator	chr3p14.2		8618	---	---	---	---		MmugDNA.13486.1.S1_at_CADPS
MmugDNA.13487.1.S1_at	232444_at	C6orf204	chromosome 6 open reading frame 204	chr6q22		387119	LOC714810	similar to chromosome 6 open reading frame 204	---		714810	MmugDNA.13487.1.S1_at_C6orf204
MmugDNA.13488.1.S1_at	242100_at	CHSY3	chondroitin sulfate synthase 3	chr5q23.3		337876	LOC705715	similar to chondroitin sulfate synthase 3	---		705715	MmugDNA.13488.1.S1_at_CHSY3
MmugDNA.13489.1.S1_at	231716_at	RC3H2	ring finger and CCCH-type zinc finger domains 2	chr9q34		54542	---	---	---	---		MmugDNA.13489.1.S1_at_RC3H2
MmugDNA.13490.1.S1_at	207886_s_at	CALCR	calcitonin receptor	chr7q21.3		799	CALCR	calcitonin receptor	---		701093	MmugDNA.13490.1.S1_at_CALCR
MmugDNA.13491.1.S1_at	222595_s_at	DIDO1	death inducer-obliterator 1	chr20q13.33		11083	LOC714153	Similar to exosome component 10 isoform 2	---		714153	MmugDNA.13491.1.S1_at_DIDO1
MmugDNA.1349.1.S1_at	1558119_at	TSPAN17	tetraspanin 17	chr5q35.3		26262	TSPAN17	tetraspanin 17	---		697739	MmugDNA.1349.1.S1_at_TSPAN17
MmugDNA.13492.1.S1_at	240351_at	---	---	---	---		---	---	---	---		MmugDNA.13492.1.S1_at_---
MmugDNA.13493.1.S1_at	244370_at	KIAA2022	KIAA2022	chrXq13.3		340533	LOC703920	similar to DNA polymerase zeta catalytic subunit (Seizure-related protein 4)	---		703920	MmugDNA.13493.1.S1_at_KIAA2022
MmugDNA.13494.1.S1_at	244579_at	---	---	---	---		---	---	---	---		MmugDNA.13494.1.S1_at_---
MmugDNA.13495.1.S1_at	239783_at	---	---	---	---		---	---	---	---		MmugDNA.13495.1.S1_at_---
MmugDNA.13496.1.S1_at	232522_at	---	---	---	---		---	---	---	---		MmugDNA.13496.1.S1_at_---
MmugDNA.13497.1.S1_at	236859_at	RUNX2	runt-related transcription factor 2	chr6p21		860	RUNX2	runt-related transcription factor 2	---		703331	MmugDNA.13497.1.S1_at_RUNX2
MmugDNA.13498.1.S1_at	227020_at	YPEL2	yippee-like 2 (Drosophila)	chr17q22		388403	---	---	---	---		MmugDNA.13498.1.S1_at_YPEL2
MmugDNA.13499.1.S1_at	222406_s_at	PNRC2	proline-rich nuclear receptor coactivator 2	chr1p36.11		55629	---	---	---	---		MmugDNA.13499.1.S1_at_PNRC2
MmugDNA.13500.1.S1_at	244678_at	---	---	---	---		---	---	---	---		MmugDNA.13500.1.S1_at_---
MmugDNA.13501.1.S1_at	227795_at	NDUFV1	NADH dehydrogenase (ubiquinone) flavoprotein 1, 51kDa	chr11q13		4723	---	---	---	---		MmugDNA.13501.1.S1_at_NDUFV1
MmugDNA.13502.1.S1_at	242665_at	FMNL2	formin-like 2	chr2q23.3		114793	---	---	---	---		MmugDNA.13502.1.S1_at_FMNL2
MmugDNA.13503.1.S1_at	230236_at	TDRG1	testis development related protein 1	chr6p21.2		732253	---	---	---	---		MmugDNA.13503.1.S1_at_TDRG1
MmugDNA.13504.1.S1_at	NA	NA	NA	NA	NA		NA	NA	NA	NA		MmugDNA.13504.1.S1_at_NA
MmugDNA.13504.1.S1_x_at	1554723_x_at	---	---	---	---		---	---	---	---		MmugDNA.13504.1.S1_x_at_---
MmugDNA.13505.1.S1_at	1554752_a_at	---	---	---	---		---	---	---	---		MmugDNA.13505.1.S1_at_---
MmugDNA.13506.1.S1_at	NA	NA	NA	NA	NA		NA	NA	NA	NA		MmugDNA.13506.1.S1_at_NA
MmugDNA.13507.1.S1_at	226741_at	SLC12A6	solute carrier family 12 (potassium/chloride transporters), member 6	chr15q13-q15		9990	---	---	---	---		MmugDNA.13507.1.S1_at_SLC12A6
MmugDNA.13508.1.S1_at	236391_at	---	---	---	---		---	---	---	---		MmugDNA.13508.1.S1_at_---
MmugDNA.13509.1.S1_at	208095_s_at	SRP72	signal recognition particle 72kDa	chr4q11		6731	LOC695614	Hypothetical protein LOC695614	---		695614	MmugDNA.13509.1.S1_at_SRP72
MmugDNA.13510.1.S1_at	217827_s_at	SPG21	spastic paraplegia 21 (autosomal recessive, Mast syndrome)	chr15q21-q22		51324	LOC712815	similar to acid cluster protein 33	---		712815	MmugDNA.13510.1.S1_at_SPG21
MmugDNA.13511.1.S1_at	1559036_at	C4A /// C4B	complement component 4A (Rodgers blood group) /// complement component 4B (Chido blood group	chr6p21.3	720 /// 721		---	---	---	---		MmugDNA.13511.1.S1_at_C4A /// C4B
MmugDNA.1351.1.S1_at	1559827_at	LOC401074	hypothetical LOC401074	chr3p12.3		401074	---	---	---	---		MmugDNA.1351.1.S1_at_LOC401074
MmugDNA.13512.1.S1_at	244395_at	FLJ41455	hypothetical gene supported by AK123449; BX641014	chr9q12		441441	---	---	---	---		MmugDNA.13512.1.S1_at_FLJ41455
MmugDNA.13513.1.S1_at	215696_s_at	SEC16A	SEC16 homolog A (S. cerevisiae)	chr9q34.3		9919	---	---	---	---		MmugDNA.13513.1.S1_at_SEC16A
MmugDNA.13514.1.S1_at	213989_x_at	SETD4	SET domain containing 4	chr21q22.13		54093	SETD4	SET domain containing 4	---		696003	MmugDNA.13514.1.S1_at_SETD4
MmugDNA.13515.1.S1_at	239003_at	---	---	---	---		---	---	---	---		MmugDNA.13515.1.S1_at_---
MmugDNA.13516.1.S1_at	239829_at	---	---	---	---		---	---	---	---		MmugDNA.13516.1.S1_at_---
MmugDNA.13517.1.S1_at	212306_at	CLASP2	cytoplasmic linker associated protein 2	chr3p22.3		23122	---	---	---	---		MmugDNA.13517.1.S1_at_CLASP2
MmugDNA.13518.1.S1_at	216089_at	---	---	---	---		LOC700155	similar to multiple coagulation factor deficiency 2	---		700155	MmugDNA.13518.1.S1_at_---
MmugDNA.13519.1.S1_at	212309_at	CLASP2	cytoplasmic linker associated protein 2	chr3p22.3		23122	LOC694117	Similar to dihydroxyacetone kinase 2	---		694117	MmugDNA.13519.1.S1_at_CLASP2
MmugDNA.135.1.S1_at	221805_at	NEFL	neurofilament, light polypeptide	chr8p21		4747	---	---	---	---		MmugDNA.135.1.S1_at_NEFL
MmugDNA.13520.1.S1_at	212308_at	CLASP2	cytoplasmic linker associated protein 2	chr3p22.3		23122	---	---	---	---		MmugDNA.13520.1.S1_at_CLASP2
MmugDNA.13521.1.S1_at	234861_at	LOC93463	hypothetical protein LOC93463	chr2q37.3		93463	---	---	---	---		MmugDNA.13521.1.S1_at_LOC93463
MmugDNA.1352.1.S1_at	213374_x_at	HIBCH	3-hydroxyisobutyryl-Coenzyme A hydrolase	chr2q32.2		26275	---	---	---	---		MmugDNA.1352.1.S1_at_HIBCH
MmugDNA.13522.1.S1_at	1564936_at	---	---	---	---		---	---	---	---		MmugDNA.13522.1.S1_at_---
MmugDNA.13523.1.S1_at	227996_at	FARP1	FERM, RhoGEF (ARHGEF) and pleckstrin domain protein 1 (chondrocyte-derived)	chr13q32.2		10160	---	---	---	---		MmugDNA.13523.1.S1_at_FARP1
MmugDNA.13524.1.S1_at	223146_at	WDR33	WD repeat domain 33	chr2q14.3		55339	WDR33	WD repeat domain 33	---		699696	MmugDNA.13524.1.S1_at_WDR33
MmugDNA.13525.1.S1_at	1569583_at	EREG	epiregulin	chr4q13.3		2069	---	---	---	---		MmugDNA.13525.1.S1_at_EREG
MmugDNA.13526.1.S1_at	240960_at	---	---	---	---		---	---	---	---		MmugDNA.13526.1.S1_at_---
MmugDNA.13527.1.S1_at	235460_at	SNX22	sorting nexin 22	chr15q22.31		79856	---	---	---	---		MmugDNA.13527.1.S1_at_SNX22
MmugDNA.13528.1.S1_at	240809_at	C21orf121	chromosome 21 open reading frame 121	chr21q22.3		150142	---	---	---	---		MmugDNA.13528.1.S1_at_C21orf121
MmugDNA.13529.1.S1_at	244688_at	---	---	---	---		---	---	---	---		MmugDNA.13529.1.S1_at_---
MmugDNA.13530.1.S1_at	241167_at	ITIH4	Inter-alpha (globulin) inhibitor H4 (plasma Kallikrein-sensitive glycoprotein)	chr3p21-p14		3700	---	---	---	---		MmugDNA.13530.1.S1_at_ITIH4
MmugDNA.13531.1.S1_at	201850_at	CAPG	capping protein (actin filament), gelsolin-like	chr2p11.2		822	CAPG	capping protein (actin filament), gelsolin-like	---		695288	MmugDNA.13531.1.S1_at_CAPG
MmugDNA.1353.1.S1_at	1552280_at	TIMD4	T-cell immunoglobulin and mucin domain containing 4	chr5q33.3		91937	LOC715236	similar to T-cell immunoglobulin and mucin domain containing 4	---		715236	MmugDNA.1353.1.S1_at_TIMD4
MmugDNA.13532.1.S1_at	221248_s_at	WHSC1L1	Wolf-Hirschhorn syndrome candidate 1-like 1	chr8p11.2		54904	WHSC1L1	Wolf-Hirschhorn syndrome candidate 1-like 1	---		700254	MmugDNA.13532.1.S1_at_WHSC1L1
MmugDNA.13533.1.S1_at	1555565_s_at	TAPBP	TAP binding protein (tapasin)	chr6p21.3		6892	TAPBP	TAP binding protein (tapasin)	---		718235	MmugDNA.13533.1.S1_at_TAPBP
MmugDNA.13534.1.S1_at	230740_at	---	---	---	---		---	---	---	---		MmugDNA.13534.1.S1_at_---
MmugDNA.13535.1.S1_at	1556008_a_at	---	---	---	---		---	---	---	---		MmugDNA.13535.1.S1_at_---
MmugDNA.13536.1.S1_at	200001_at	CAPNS1	calpain, small subunit 1	chr19q13.12		826	LOC712561	similar to Calpain small subunit 1 (CSS1) (Calcium-dependent protease small subunit 1) (Calcium-de	---		712561	MmugDNA.13536.1.S1_at_CAPNS1
MmugDNA.13537.1.S1_s_at	210272_at	CYP2B7P1	cytochrome P450, family 2, subfamily B, polypeptide 7 pseudogene 1	chr19q13.2		1556	---	---	---	---		MmugDNA.13537.1.S1_s_at_CYP2B7P
MmugDNA.13538.1.S1_at	210128_s_at	LTB4R	leukotriene B4 receptor	chr14q11.2-q12		1241	LTB4R	leukotriene B4 receptor	---		716055	MmugDNA.13538.1.S1_at_LTB4R
MmugDNA.13539.1.S1_at	NA	NA	NA	NA	NA		NA	NA	NA	NA		MmugDNA.13539.1.S1_at_NA
MmugDNA.13540.1.S1_at	234733_s_at	FANCM	Fanconi anemia, complementation group M	chr14q21.3		57697	FANCM	Fanconi anemia, complementation group M	---		702513	MmugDNA.13540.1.S1_at_FANCM
MmugDNA.13541.1.S1_at	229193_at	---	---	---	---		CROP	cisplatin resistance-associated overexpressed protein	---		703662	MmugDNA.13541.1.S1_at_---
MmugDNA.1354.1.S1_at	233682_at	---	---	---	---		---	---	---	---		MmugDNA.1354.1.S1_at_---
MmugDNA.13542.1.S1_at	236440_at	---	---	---	---		---	---	---	---		MmugDNA.13542.1.S1_at_---
MmugDNA.13543.1.S1_at	228656_at	PROX1	prospero homeobox 1	chr1q32.2-q32.3		5629	LOC709465	similar to prospero-related homeobox 1	---		709465	MmugDNA.13543.1.S1_at_PROX1
MmugDNA.13544.1.S1_at	226178_at	SOCS4	suppressor of cytokine signaling 4	chr14q22.3		122809	---	---	---	---		MmugDNA.13544.1.S1_at_SOCS4
MmugDNA.13545.1.S1_at	231696_x_at	---	---	---	---		---	---	---	---		MmugDNA.13545.1.S1_at_---
MmugDNA.13546.1.S1_at	242427_at	WAC	WW domain containing adaptor with coiled-coil	---		51322	---	---	---	---		MmugDNA.13546.1.S1_at_WAC
MmugDNA.13547.1.S1_at	231801_at	NFATC2	nuclear factor of activated T-cells, cytoplasmic, calcineurin-dependent 2	chr20q13.2-q13.3		4773	LOC710601	similar to nuclear factor of activated T-cells, cytoplasmic, calcineurin-dependent 2 isoform C	---		710601	MmugDNA.13547.1.S1_at_NFATC2
MmugDNA.13548.1.S1_at	229426_at	COX5A	cytochrome c oxidase subunit Va	chr15q24.1		9377	---	---	---	---		MmugDNA.13548.1.S1_at_COX5A
MmugDNA.13549.1.S1_at	243009_at	LOC441242	Hypothetical LOC441242	chr7q11.21		441242	---	---	---	---		MmugDNA.13549.1.S1_at_LOC441242
MmugDNA.13550.1.S1_at	210423_s_at	SLC11A1	solute carrier family 11 (proton-coupled divalent metal ion transporters), member 1	chr2q35		6556	SLC11A1	solute carrier family 11 (proton-coupled divalent metal ion transporters), member 1	---		701025	MmugDNA.13550.1.S1_at_SLC11A1
MmugDNA.13551.1.S1_at	215687_x_at	PLCB1	phospholipase C, beta 1 (phosphoinositide-specific)	chr20p12		23236	---	---	---	---		MmugDNA.13551.1.S1_at_PLCB1
MmugDNA.1355.1.S1_at	1557770_at	IPO11	importin 11	chr5q12.1		51194	---	---	---	---		MmugDNA.1355.1.S1_at_IPO11
MmugDNA.13552.1.S1_at	243856_at	---	---	---	---		---	---	---	---		MmugDNA.13552.1.S1_at_---
MmugDNA.13553.1.S1_at	1561488_at	---	---	---	---		---	---	---	---		MmugDNA.13553.1.S1_at_---
MmugDNA.13554.1.S1_at	1562973_at	---	---	---	---		---	---	---	---		MmugDNA.13554.1.S1_at_---
MmugDNA.13555.1.S1_at	242513_x_at	KIAA2018	KIAA2018	chr3q13.2		205717	---	---	---	---		MmugDNA.13555.1.S1_at_KIAA2018
MmugDNA.13556.1.S1_at	1561633_at	HMGA2	high mobility group AT-hook 2	chr12q15		8091	HMGA2	high mobility group AT-hook 2	---		717721	MmugDNA.13556.1.S1_at_HMGA2
MmugDNA.13556.1.S1_s_at	1558682_at	HMGA2	high mobility group AT-hook 2	chr12q15		8091	HMGA2	high mobility group AT-hook 2	---		717721	MmugDNA.13556.1.S1_s_at_HMGA2
MmugDNA.13557.1.S1_at	1562327_at	---	---	---	---		---	---	---	---		MmugDNA.13557.1.S1_at_---
MmugDNA.13558.1.S1_at	1560483_at	---	---	---	---		---	---	---	---		MmugDNA.13558.1.S1_at_---
MmugDNA.13559.1.S1_at	202298_at	NDUFA1	NADH dehydrogenase (ubiquinone) 1 alpha subcomplex, 1, 7.5kDa	chrXq24		4694	NDUFA1	NADH dehydrogenase (ubiquinone) 1 alpha subcomplex, 1, 7.5kDa	---		711626	MmugDNA.13559.1.S1_at_NDUFA1
MmugDNA.13561.1.S1_at	201304_at	NDUFA5	NADH dehydrogenase (ubiquinone) 1 alpha subcomplex, 5, 13kDa	chr7q32		4698	IFIT2	Interferon-induced protein with tetratricopeptide repeats 2	---		695452	MmugDNA.13561.1.S1_at_NDUFA5
MmugDNA.1356.1.S1_at	NA	NA	NA	NA	NA		NA	NA	NA	NA		MmugDNA.1356.1.S1_at_NA
MmugDNA.13562.1.S1_at	240908_at	---	---	---	---		---	---	---	---		MmugDNA.13562.1.S1_at_---
MmugDNA.13563.1.S1_at	239929_at	PM20D1	peptidase M20 domain containing 1	chr1q32.1		148811	---	---	---	---		MmugDNA.13563.1.S1_at_PM20D1

MmugDNA.13564.1.S1_at	1555546_a_at	POFUT2	protein O-fucosyltransferase 2	chr21q22.3		23275	LOC721991	similar to protein O-fucosyltransferase 2 isoform C	---	721991	MmugDNA.13564.1.S1_at_POFUT2
MmugDNA.13565.1.S1_at	204444_at	KIF11	kinesin family member 11	chr10q24.1		3832	---	---	---	---	MmugDNA.13565.1.S1_at_KIF11
MmugDNA.13566.1.S1_at	208050_s_at	CASP2	caspase 2, apoptosis-related cysteine peptidase	chr7q34-q35		835	---	---	---	---	MmugDNA.13566.1.S1_at_CASP2
MmugDNA.13567.1.S1_at	1560477_a_at	SAMD11	sterile alpha motif domain containing 11	chr1p36.33		148398	---	---	---	---	MmugDNA.13567.1.S1_at_SAMD11
MmugDNA.13568.1.S1_at	NA	NA	NA	NA	NA		NA	NA	NA	NA	MmugDNA.13568.1.S1_at_NA
MmugDNA.13568.1.S1_s_at	207682_s_at	KIF25	kinesin family member 25	chr6q27		3834	LOC697170	similar to kinesin family member 25 isoform 1	---	697170	MmugDNA.13568.1.S1_s_at_KIF25
MmugDNA.13569.1.S1_at	202169_s_at	AASDHPPT	aminoadipate-semialdehyde dehydrogenase-phosphopantetheinyl transferase	chr11q22		60496	---	---	---	---	MmugDNA.13569.1.S1_at_AASDHPPT
MmugDNA.13570.1.S1_at	NA	NA	NA	NA	NA		NA	NA	NA	NA	MmugDNA.13570.1.S1_at_NA
MmugDNA.13571.1.S1_at	207123_s_at	MATN4	matrilin 4	chr20q13.1-q13.2		8785	MATN4	matrilin 4	---	717652	MmugDNA.13571.1.S1_at_MATN4
MmugDNA.1357.1.S1_at	NA	NA	NA	NA	NA		NA	NA	NA	NA	MmugDNA.1357.1.S1_at_NA
MmugDNA.13572.1.S1_at	238985_at	---	---	---	---		---	---	---	---	MmugDNA.13572.1.S1_at_---
MmugDNA.13573.1.S1_at	241474_at	---	---	---	---		---	---	---	---	MmugDNA.13573.1.S1_at_---
MmugDNA.13574.1.S1_at	1557359_at	LOC285758	hypothetical protein LOC285758	chr6q21		285758	---	---	---	---	MmugDNA.13574.1.S1_at_LOC285758
MmugDNA.13575.1.S1_at	1569454_a_at	LOC283352	hypothetical protein LOC283352	chr12q24.33		283352	---	---	---	---	MmugDNA.13575.1.S1_at_LOC283352
MmugDNA.13576.1.S1_at	1568777_at	EML5	echinoderm microtubule associated protein like 5	chr14q31.3		161436	LOC694095	similar to echinoderm microtubule associated protein like 5	---	694095	MmugDNA.13576.1.S1_at_EML5
MmugDNA.13577.1.S1_at	240516_at	---	---	---	---		---	---	---	---	MmugDNA.13577.1.S1_at_---
MmugDNA.13578.1.S1_at	1554292_a_at	UHRF1BP1L	UHRF1 binding protein 1-like	chr12q23.1		23074	UHRF1BP1L	UHRF1 binding protein 1-like	---	694816	MmugDNA.13578.1.S1_at_UHRF1BP1L
MmugDNA.13579.1.S1_at	NA	NA	NA	NA	NA		NA	NA	NA	NA	MmugDNA.13579.1.S1_at_NA
MmugDNA.13579.1.S1_s_at	228334_x_at	KIAA1712	KIAA1712	chr4q34		80817	---	---	---	---	MmugDNA.13579.1.S1_s_at_KIAA1712
MmugDNA.13580.1.S1_at	NA	NA	NA	NA	NA		NA	NA	NA	NA	MmugDNA.13580.1.S1_at_NA
MmugDNA.13581.1.S1_at	1557244_a_at	---	---	---	---		---	---	---	---	MmugDNA.13581.1.S1_at_---
MmugDNA.1358.1.S1_at	1564274_at	C9orf47	chromosome 9 open reading frame 47	chr9q22.1		286223	LOC696731	hypothetical protein LOC696731	---	696731	MmugDNA.1358.1.S1_at_C9orf47
MmugDNA.13582.1.S1_at	235221_at	CBLN3	cerebellin 3 precursor	chr14q12		643866	LOC716357	similar to Cerebellin-3 precursor	---	716357	MmugDNA.13582.1.S1_at_CBLN3
MmugDNA.13583.1.S1_at	237047_at	LOC26080	hypothetical LOC26080	---		26080	LOC718805 /// LOC7188	hypothetical protein LOC718805 /// hypothetical protein LOC718817	---	718805 /// 718817	MmugDNA.13583.1.S1_at_LOC26080
MmugDNA.13584.1.S1_at	204709_s_at	KIF23	kinesin family member 23	chr15q23		9493	KIF23	kinesin family member 23	---	695229	MmugDNA.13584.1.S1_at_KIF23
MmugDNA.13585.1.S1_at	229170_s_at	TTC18	tetratricopeptide repeat domain 18	chr10q22.2		118491	LOC709315	Hypothetical protein LOC709315	---	709315	MmugDNA.13585.1.S1_at_TTC18
MmugDNA.13586.1.S1_at	213373_s_at	CASP8	caspase 8, apoptosis-related cysteine peptidase	chr2q33-q34		841	CASP8	caspase 8, apoptosis-related cysteine peptidase	---	702781	MmugDNA.13586.1.S1_at_CASP8
MmugDNA.13587.1.S1_at	1561540_at	LOC144920	hypothetical protein LOC144920	chr13q33.2		144920	---	---	---	---	MmugDNA.13587.1.S1_at_LOC144920
MmugDNA.13588.1.S1_at	1563744_a_at	DISC1	disrupted in schizophrenia 1	chr1q42.1		27185	---	---	---	---	MmugDNA.13588.1.S1_at_DISC1
MmugDNA.13589.1.S1_at	215514_at	---	---	---	---		---	---	---	---	MmugDNA.13589.1.S1_at_---
MmugDNA.13590.1.S1_at	208130_s_at	TBXAS1	thromboxane A synthase 1 (platelet)	chr7q34-q35		6916	TBXAS1	thromboxane A synthase 1 (platelet, cytochrome P450, family 5, subfamily A)	---	711748	MmugDNA.13590.1.S1_at_TBXAS1
MmugDNA.13591.1.S1_at	238497_at	TMEM136	transmembrane protein 136	chr11q23.3		219902	LOC706163	hypothetical protein LOC706163	---	706163	MmugDNA.13591.1.S1_at_TMEM136
MmugDNA.1359.1.S1_at	217579_x_at	---	---	---	---		---	---	---	---	MmugDNA.1359.1.S1_at_---
MmugDNA.13592.1.S1_at	1557555_at	---	---	---	---		---	---	---	---	MmugDNA.13592.1.S1_at_---
MmugDNA.13593.1.S1_at	231869_at	KIAA1586	KIAA1586	chr6p12.1		57691	LOC712568	similar to zinc finger protein 451	---	712568	MmugDNA.13593.1.S1_at_KIAA1586
MmugDNA.13594.1.S1_at	231781_s_at	LRRC2	leucine rich repeat containing 2	chr3p21.31		79442	LRRC2	leucine rich repeat containing 2	---	712980	MmugDNA.13594.1.S1_at_LRRC2
MmugDNA.13595.1.S1_at	NA	NA	NA	NA	NA		NA	NA	NA	NA	MmugDNA.13595.1.S1_at_NA
MmugDNA.13595.1.S1_s_at	224204_x_at	ARNTL2	aryl hydrocarbon receptor nuclear translocator-like 2	chr12p12.2-p11.2		56938	LOC709547	similar to aryl hydrocarbon receptor nuclear translocator-like 2	---	709547	MmugDNA.13595.1.S1_s_at_ARNTL2
MmugDNA.13596.1.S1_at	212411_at	IMP4	IMP4, U3 small nucleolar ribonucleoprotein, homolog (yeast)	chr2q21.1		92856	IMP4	IMP4, U3 small nucleolar ribonucleoprotein, homolog (yeast)	---	702144	MmugDNA.13596.1.S1_at_IMP4
MmugDNA.13597.1.S1_at	232410_at	---	---	---	---		---	---	---	---	MmugDNA.13597.1.S1_at_---
MmugDNA.13598.1.S1_at	229955_at	FBXO3	F-box protein 3	chr11p13		26273	---	---	---	---	MmugDNA.13598.1.S1_at_FBXO3
MmugDNA.13599.1.S1_at	NA	NA	NA	NA	NA		NA	NA	NA	NA	MmugDNA.13599.1.S1_at_NA
MmugDNA.13600.1.S1_at	233163_at	LOC731157	Hypothetical LOC731157	chr5q14.3		731157	---	---	---	---	MmugDNA.13600.1.S1_at_LOC731157
MmugDNA.13601.1.S1_at	210101_x_at	SH3GLB1	SH3-domain GRB2-like endophilin B1	chr1p22		51100	LOC707246	Hypothetical protein LOC707246	---	707246	MmugDNA.13601.1.S1_at_SH3GLB1
MmugDNA.1360.1.S1_at	204899_s_at	SAP30	Sin3A-associated protein, 30kDa	chr4q34.1		8819	---	---	---	---	MmugDNA.1360.1.S1_at_SAP30
MmugDNA.13602.1.S1_at	244867_at	---	---	---	---		---	---	---	---	MmugDNA.13602.1.S1_at_---
MmugDNA.13604.1.S1_s_at	208129_x_at	RUNX1	runt-related transcription factor 1	chr21q22.3		861	RUNX1	runt-related transcription factor 1	---	696749	MmugDNA.13604.1.S1_s_at_RUNX1
MmugDNA.13605.1.S1_at	238061_at	LGI3	leucine-rich repeat LGI family, member 3	chr8p21.3		203190	---	---	---	---	MmugDNA.13605.1.S1_at_LGI3
MmugDNA.13606.1.S1_at	242741_x_at	---	---	---	---		---	---	---	---	MmugDNA.13606.1.S1_at_---
MmugDNA.13607.1.S1_at	244277_at	---	---	---	---		---	---	---	---	MmugDNA.13607.1.S1_at_---
MmugDNA.13609.1.S1_at	232952_at	---	---	---	---		---	---	---	---	MmugDNA.13609.1.S1_at_---
MmugDNA.13610.1.S1_at	228333_at	---	---	---	---		---	---	---	---	MmugDNA.13610.1.S1_at_---
MmugDNA.13611.1.S1_at	221116_at	---	---	---	---		---	---	---	---	MmugDNA.13611.1.S1_at_---
MmugDNA.1361.1.S1_at	1564401_at	LOC400748	hypothetical gene supported by BC040627	chr1p35.2		400748	---	---	---	---	MmugDNA.1361.1.S1_at_LOC400748
MmugDNA.13612.1.S1_at	226522_at	PODN	podocan	chr1p32.3		127435	LOC714025	hypothetical protein LOC714025	---	714025	MmugDNA.13612.1.S1_at_PODN
MmugDNA.13613.1.S1_at	221066_at	RXFP3	relaxin/insulin-like family peptide receptor 3	chr5p15.1-p14		51289	RXFP3	relaxin/insulin-like family peptide receptor 3	---	698115	MmugDNA.13613.1.S1_at_RXFP3
MmugDNA.13614.1.S1_s_at	218135_at	ERGIC2	ERGIC and golgi 2	chr12p11.22		51290	LOC710837	similar to PTX1 protein	---	710837	MmugDNA.13614.1.S1_s_at_ERGIC2
MmugDNA.13615.1.S1_at	NA	NA	NA	NA	NA		NA	NA	NA	NA	MmugDNA.13615.1.S1_at_NA
MmugDNA.13616.1.S1_at	244541_x_at	---	---	---	---		---	---	---	---	MmugDNA.13616.1.S1_at_---
MmugDNA.13617.1.S1_at	218913_s_at	GMIP	GEM interacting protein	chr19p12-p11		51291	GMIP	GEM interacting protein	---	720380	MmugDNA.13617.1.S1_at_GMIP
MmugDNA.13618.1.S1_at	213259_s_at	SARM1	sterile alpha and TIR motif containing 1	chr17q11		23098	LOC715863	similar to sterile alpha and TIR motif containing 1	---	715863	MmugDNA.13618.1.S1_at_SARM1
MmugDNA.13619.1.S1_at	205310_at	FBXO46	F-box protein 46	chr19q13.3		23403	---	---	---	---	MmugDNA.13619.1.S1_at_FBXO46
MmugDNA.136.1.S1_at	NA	NA	NA	NA	NA		NA	NA	NA	NA	MmugDNA.136.1.S1_at_NA
MmugDNA.136.1.S1_s_at	221916_at	NEFL	neurofilament, light polypeptide	chr8p21		4747	---	---	---	---	MmugDNA.136.1.S1_s_at_NEFL
MmugDNA.13620.1.S1_at	213257_at	SARM1	sterile alpha and TIR motif containing 1	chr17q11		23098	---	---	---	---	MmugDNA.13620.1.S1_at_SARM1
MmugDNA.13621.1.S1_at	212534_at	ZNF24	zinc finger protein 24	chr18q12		7572	LOC702374	Similar to Protein KIAA0195 (Transmembrane protein 94)	---	702374	MmugDNA.13621.1.S1_at_ZNF24
MmugDNA.1362.1.S1_at	242703_at	---	---	---	---		---	---	---	---	MmugDNA.1362.1.S1_at_---
MmugDNA.13622.1.S1_at	216531_at	YY2	YY2 transcription factor	chrXp22.2-p22.1		404281	LOC696656	similar to YY2 transcription factor	---	696656	MmugDNA.13622.1.S1_at_YY2
MmugDNA.13623.1.S1_at	242339_at	---	---	---	---		---	---	---	---	MmugDNA.13623.1.S1_at_---
MmugDNA.13624.1.S1_at	231013_at	---	---	---	---		---	---	---	---	MmugDNA.13624.1.S1_at_---
MmugDNA.13625.1.S1_at	NA	NA	NA	NA	NA		NA	NA	NA	NA	MmugDNA.13625.1.S1_at_NA
MmugDNA.13625.1.S1_s_at	216396_s_at	EI24	etoposide induced 2.4 mRNA	chr11q24		9538	LOC713226	similar to etoposide induced 2.4 isoform 1	---	713226	MmugDNA.13625.1.S1_s_at_EI24
MmugDNA.13626.1.S1_at	208131_s_at	PTGIS	prostaglandin I2 (prostacyclin) synthase	chr20q13.13		5740	---	---	---	---	MmugDNA.13626.1.S1_at_PTGIS
MmugDNA.13627.1.S1_at	209598_at	PNMA2	paraneoplastic antigen MA2	chr8p21.2		10687	---	---	---	---	MmugDNA.13627.1.S1_at_PNMA2
MmugDNA.13628.1.S1_at	205917_at	ZNF264	zinc finger protein 264	chr19q13.4		9422	---	---	---	---	MmugDNA.13628.1.S1_at_ZNF264
MmugDNA.13629.1.S1_at	NA	NA	NA	NA	NA		NA	NA	NA	NA	MmugDNA.13629.1.S1_at_NA
MmugDNA.13629.1.S1_s_at	214118_x_at	PCM1	pericentriolar material 1	chr8p22-p21.3		5108	PCM1	pericentriolar material 1	---	703580	MmugDNA.13629.1.S1_s_at_PCM1
MmugDNA.13630.1.S1_at	231618_s_at	SUNC1	Sad1 and UNC84 domain containing 1	chr7p12.3		256979	LOC695546	similar to Sad1 and UNC84 domain containing 1	---	695546	MmugDNA.13630.1.S1_at_SUNC1
MmugDNA.13631.1.S1_at	NA	NA	NA	NA	NA		NA	NA	NA	NA	MmugDNA.13631.1.S1_at_NA
MmugDNA.13631.1.S1_s_at	218529_at	CD320	CD320 molecule	chr19p13.3-p13.2		51293	LOC711130	similar to 8D6 antigen	---	711130	MmugDNA.13631.1.S1_s_at_CD320
MmugDNA.1363.1.S1_at	1561653_at	---	---	---	---		---	---	---	---	MmugDNA.1363.1.S1_at_---
MmugDNA.13632.1.S1_at	238910_at	---	---	---	---		---	---	---	---	MmugDNA.13632.1.S1_at_---
MmugDNA.13633.1.S1_at	226888_at	CSNK1G1	casein kinase 1, gamma 1	chr15q22.1-q22.31		53944	LOC697717	Similar to cell cycle progression 2 protein isoform 1	---	697717	MmugDNA.13633.1.S1_at_CSNK1G1
MmugDNA.13634.1.S1_at	218225_at	ECSIT	ECSIT homolog (Drosophila)	chr19p13.2		51295	LOC714805	similar to evolutionarily conserved signaling intermediate in Toll pathway	---	714805	MmugDNA.13634.1.S1_at_ECSIT
MmugDNA.13635.1.S1_at	206469_x_at	AKR7A3	aldo-keto reductase family 7, member A3 (aflatoxin aldehyde reductase)	chr1p36.13		22977	AKR7A3	aldo-keto reductase family 7, member A3	---	715623	MmugDNA.13635.1.S1_at_AKR7A3
MmugDNA.13635.1.S1_s_at	NA	NA	NA	NA	NA		NA	NA	NA	NA	MmugDNA.13635.1.S1_s_at_NA
MmugDNA.13636.1.S1_at	209489_at	CUGBP1	CUG triplet repeat, RNA binding protein 1	chr11p11		10658	CUGBP1	CUG triplet repeat, RNA binding protein 1	---	713167	MmugDNA.13636.1.S1_at_CUGBP1
MmugDNA.13637.1.S1_at	230876_at	LOC169834	hypothetical protein LOC169834	chr9q32		169834	LOC706617	similar to zinc finger protein 135 (clone pHZ-17)	---	706617	MmugDNA.13637.1.S1_at_LOC169834
MmugDNA.13638.1.S1_at	219593_at	SLC15A3	solute carrier family 15, member 3	chr11q12.2		51296	SLC15A3	solute carrier family 15, member 3	---	696060	MmugDNA.13638.1.S1_at_SLC15A3
MmugDNA.13639.1.S1_at	219514_at	ANGPTL2	angiopoietin-like 2	chr9q34		23452	ANGPTL2	angiopoietin-like 2	---	701444	MmugDNA.13639.1.S1_at_ANGPTL2
MmugDNA.13640.1.S1_at	220542_s_at	PLUNC	palate, lung and nasal epithelium associated	chr20q11.2		51297	PLUNC	palate, lung and nasal epithelium associated	---	714509	MmugDNA.13640.1.S1_at_PLUNC
MmugDNA.13641.1.S1_at	237643_at	---	---	---	---		---	---	---	---	MmugDNA.13641.1.S1_at_---
MmugDNA.1364.1.S1_at	227069_at	---	---	---	---		---	---	---	---	MmugDNA.1364.1.S1_at_---
MmugDNA.13642.1.S1_at	225284_at	DNAJC3	DnaJ (Hsp40) homolog, subfamily C, member 3	chr13q32.1		5611	---	---	---	---	MmugDNA.13642.1.S1_at_DNAJC3
MmugDNA.13643.1.S1_at	224240_s_at	CCL28	chemokine (C-C motif) ligand 28	chr5p12		56477	CCL28	chemokine (C-C motif) ligand 28	---	574221	MmugDNA.13643.1.S1_at_CCL28
MmugDNA.13644.1.S1_at	207408_at	SLC22A14	solute carrier family 22, member 14	chr3p21.3		9389	LOC696278	Hypothetical protein LOC696278	---	696278	MmugDNA.13644.1.S1_at_SLC22A14
MmugDNA.13645.1.S1_at	236096_at	SIPA1L3	signal-induced proliferation-associated 1 like 3	chr19q13.13		23094	LOC714601	similar to signal-induced proliferation-associated 1 like 3	---	714601	MmugDNA.13645.1.S1_at_SIPA1L3
MmugDNA.13646.1.S1_at	222801_s_at	STAG3L4	stromal antigen 3-like 4	chr7p11.2-q11.2		64940	---	---	---	---	MmugDNA.13646.1.S1_at_STAG3L4
MmugDNA.13647.1.S1_at	215854_at	---	---	---	---		---	---	---	---	MmugDNA.13647.1.S1_at_---
MmugDNA.13648.1.S1_at	219516_at	TRPV4	transient receptor potential cation channel, subfamily V, member 4	chr12q24.1		59341	LOC709605	similar to transient receptor potential cation channel, subfamily V, member 4 isoform a	---	709605	MmugDNA.13648.1.S1_at_TRPV4
MmugDNA.13649.1.S1_at	NA	NA	NA	NA	NA		NA	NA	NA	NA	MmugDNA.13649.1.S1_at_NA
MmugDNA.13650.1.S1_at	220943_s_at	C2orf56	chromosome 2 open reading frame 56	chr2p22.2		55471	LOC709473	similar to CG17726-PA	---	709473	MmugDNA.13650.1.S1_at_C2orf56
MmugDNA.13651.1.S1_at	232292_at	---	---	---	---		---	---	---	---	MmugDNA.13651.1.S1_at_---
MmugDNA.1365.1.S1_at	242003_at	ERICH1	Glutamate-rich 1	chr8p23.3		157697	---	---	---	---	MmugDNA.1365.1.S1_at_ERICH1
MmugDNA.13652.1.S1_at	243141_at	SGMS2	sphingomyelin synthase 2	chr4q25		166929	---	---	---	---	MmugDNA.13652.1.S1_at_SGMS2
MmugDNA.13653.1.S1_at	244407_at	CYP39A1	cytochrome P450, family 39, subfamily A, polypeptide 1	chr6p21.1-p11.2		51302	---	---	---	---	MmugDNA.13653.1.S1_at_CYP39A1
MmugDNA.13654.1.S1_at	202309_at	MTHFD1	methylenetetrahydrofolate dehydrogenase (NADP+ dependent) 1, methenyltetrahydrofolate cyclohydr	chr14q24		4522	MTHFD1	methylenetetrahydrofolate dehydrogenase (NADP+ dependent) 1, methenyltetrahydrofolate cyclohydr	---	707059	MmugDNA.13654.1.S1_at_MTHFD1
MmugDNA.13655.1.S1_s_at	214037_s_at	CCDC22	coiled-coil domain containing 22	chrXp11.23		28952	LOC713063	similar to coiled-coil domain containing 22	---	713063	MmugDNA.13655.1.S1_s_at_CCDC22

MmugDNA.13656.1.S1_at	236828_at	---	---	---	---		---	---	---	---	MmugDNA.13656.1.S1_at_---
MmugDNA.13657.1.S1_at	1557888_at	ZNF787	zinc finger protein 787	chr19q13.42		126208	---	---	---	---	MmugDNA.13657.1.S1_at_ZNF787
MmugDNA.13658.1.S1_at	204409_s_at	EIF1AY	eukaryotic translation initiation factor 1A, Y-linked	chrYq11.222		9086	EIF1AY	eukaryotic translation initiation factor 1A, Y-linked	---	100270695	MmugDNA.13658.1.S1_at_EIF1AY
MmugDNA.13659.1.S1_at	232829_at	OR52K3P	olfactory receptor, family 52, subfamily K, member 3 pseudogene	chr11p15.4		390035	LOC717782	similar to Olfactory receptor 52K2	---	717782	MmugDNA.13659.1.S1_at_OR52K3P
MmugDNA.13660.1.S1_at	1563511_at	DKFZp451B082	hypothetical gene supported by AL831931	chr6q26		401282	---	---	---	---	MmugDNA.13660.1.S1_at_DKFZp451B(
MmugDNA.13661.1.S1_at	216253_s_at	PARVB	parvin, beta	chr22q13.2-q13.33		29780	LOC715859	similar to parvin, beta isoform b	---	715859	MmugDNA.13661.1.S1_at_PARVB
MmugDNA.1366.1.S1_at	242514_at	---	---	---	---		---	---	---	---	MmugDNA.1366.1.S1_at_---
MmugDNA.13662.1.S1_at	220808_at	THEG	Theg homolog (mouse)	chr19pter-p13		51298	THEG	Theg homolog (mouse)	---	701561	MmugDNA.13662.1.S1_at_THEG
MmugDNA.13663.1.S1_at	218158_s_at	APPL1	adaptor protein, phosphotyrosine interaction, PH domain and leucine zipper containing 1	chr3p21.1-p14.3		26060	LOC698461	Similar to COP9 constitutive photomorphogenic homolog subunit 3	---	698461	MmugDNA.13663.1.S1_at_APPL1
MmugDNA.13664.1.S1_at	218150_at	ARL5A	ADP-ribosylation factor-like 5A	chr2q23.3		26225	---	---	---	---	MmugDNA.13664.1.S1_at_ARL5A
MmugDNA.13665.1.S1_at	210859_x_at	CLN3	ceroid-lipofuscinosis, neuronal 3	chr16p12.1		1201	CLN3	CLN3 protein	---	705815	MmugDNA.13665.1.S1_at_CLN3
MmugDNA.13665.1.S1_s_at	NA	NA	NA	NA	NA		NA	NA	NA	NA	MmugDNA.13665.1.S1_s_at_NA
MmugDNA.13666.1.S1_at	220831_at	GCNT4	glucosaminyl (N-acetyl) transferase 4, core 2 (beta-1,6-N-acetylglucosaminyltransferase)	chr5q12		51301	LOC705107	similar to core 2 beta-1,6-N-acetylglucosaminyltransferase 3	---	705107	MmugDNA.13666.1.S1_at_GCNT4
MmugDNA.13667.1.S1_at	NA	NA	NA	NA	NA		NA	NA	NA	NA	MmugDNA.13667.1.S1_at_NA
MmugDNA.13667.1.S1_x_at	NA	NA	NA	NA	NA		NA	NA	NA	NA	MmugDNA.13667.1.S1_x_at_NA
MmugDNA.13669.1.S1_at	1559576_at	WDR78	WD repeat domain 78	chr1p31.3		79819	---	---	---	---	MmugDNA.13669.1.S1_at_WDR78
MmugDNA.13670.1.S1_at	38703_at	DNPEP	aspartyl aminopeptidase	chr2q35		23549	DNPEP	aspartyl aminopeptidase	---	703180	MmugDNA.13670.1.S1_at_DNPEP
MmugDNA.13671.1.S1_s_at	220432_s_at	CYP39A1	cytochrome P450, family 39, subfamily A, polypeptide 1	chr6p21.1-p11.2		51302	---	---	---	---	MmugDNA.13671.1.S1_s_at_CYP39A1
MmugDNA.1367.1.S1_at	238283_at	LOC151658	hypothetical LOC151658	chr3q13.12		151658	---	---	---	---	MmugDNA.1367.1.S1_at_LOC151658
MmugDNA.13672.1.S1_at	233464_at	BCL2L14	BCL2-like 14 (apoptosis facilitator)	chr12p13-p12		79370	---	---	---	---	MmugDNA.13672.1.S1_at_BCL2L14
MmugDNA.13673.1.S1_s_at	1553681_a_at	PRF1	perforin 1 (pore forming protein)	chr10q22		5551	---	---	---	---	MmugDNA.13673.1.S1_s_at_PRF1
MmugDNA.13674.1.S1_at	202504_at	TRIM29	tripartite motif-containing 29	chr11q22-q23		23650	TRIM29	tripartite motif-containing 29	---	705796	MmugDNA.13674.1.S1_at_TRIM29
MmugDNA.13675.1.S1_at	210955_at	CASP10	caspase 10, apoptosis-related cysteine peptidase	chr2q33-q34		843	---	---	---	---	MmugDNA.13675.1.S1_at_CASP10
MmugDNA.13676.1.S1_at	244198_at	RANBP17	RAN binding protein 17	chr5q34		64901	---	---	---	---	MmugDNA.13676.1.S1_at_RANBP17
MmugDNA.13677.1.S1_at	242046_at	---	---	---	---		---	---	---	---	MmugDNA.13677.1.S1_at_---
MmugDNA.13678.1.S1_at	238036_at	SHE	Src homology 2 domain containing E	chr1q21.3		126669	---	---	---	---	MmugDNA.13678.1.S1_at_SHE
MmugDNA.13679.1.S1_at	220414_at	CALML5	calmodulin-like 5	chr10p15.1		51806	CALML5	calmodulin-like 5	---	712159	MmugDNA.13679.1.S1_at_CALML5
MmugDNA.13680.1.S1_at	242383_at	---	---	---	---		---	---	---	---	MmugDNA.13680.1.S1_at_---
MmugDNA.13681.1.S1_at	241269_at	---	---	---	---		---	---	---	---	MmugDNA.13681.1.S1_at_---
MmugDNA.1368.1.S1_at	240910_at	---	---	---	---		---	---	---	---	MmugDNA.1368.1.S1_at_---
MmugDNA.13682.1.S1_at	217566_s_at	TGM4	transglutaminase 4 (prostate)	chr3p22-p21.33		7047	TGM4	transglutaminase 4 (prostate)	---	714883	MmugDNA.13682.1.S1_at_TGM4
MmugDNA.13683.1.S1_at	1570110_at	---	---	---	---		---	---	---	---	MmugDNA.13683.1.S1_at_---
MmugDNA.13684.1.S1_at	241278_at	---	---	---	---		---	---	---	---	MmugDNA.13684.1.S1_at_---
MmugDNA.13685.1.S1_at	240869_at	---	---	---	---		---	---	---	---	MmugDNA.13685.1.S1_at_---
MmugDNA.13686.1.S1_at	214941_s_at	PRPF40A	PRP40 pre-mRNA processing factor 40 homolog A (S. cerevisiae)	chr2q23.3		55660	PRPF40A	formin binding protein 3	---	696668	MmugDNA.13686.1.S1_at_PRPF40A
MmugDNA.13687.1.S1_at	1556043_a_at	LOC100131039	Hypothetical protein LOC100131039	chr2q31.2		100131039	---	---	---	---	MmugDNA.13687.1.S1_at_LOC100131(
MmugDNA.13688.1.S1_at	210634_at	KLHL20	kelch-like 20 (Drosophila)	chr1q24.1-q24.3		27252	LOC720187	similar to kelch-like 20	---	720187	MmugDNA.13688.1.S1_at_KLHL20
MmugDNA.13689.1.S1_at	204241_at	ACOX3	acyl-Coenzyme A oxidase 3, pristanoyl	chr4p15.3		8310	ACOX3	acyl-Coenzyme A oxidase 3, pristanoyl	---	722634	MmugDNA.13689.1.S1_at_ACOX3
MmugDNA.13690.1.S1_at	219118_at	FKBP11	FK506 binding protein 11, 19 kDa	chr12q13.12		51303	FKBP2	similar to FK506-binding protein 11 precursor (Peptidyl-prolyl cis-trans isomerase) (PPIase) (Rotamas	---	708598	MmugDNA.13690.1.S1_at_FKBP11
MmugDNA.13691.1.S1_at	239544_at	---	---	---	---		---	---	---	---	MmugDNA.13691.1.S1_at_---
MmugDNA.1369.1.S1_at	1558976_x_at	FAM100A	family with sequence similarity 100, member A	chr16p13.3		124402	---	---	---	---	MmugDNA.1369.1.S1_at_FAM100A
MmugDNA.13692.1.S1_at	200939_s_at	RERE	arginine-glutamic acid dipeptide (RE) repeats	chr1p36.2-p36.1		473	LOC708902	similar to atrophin-1 like protein	---	708902	MmugDNA.13692.1.S1_at_RERE
MmugDNA.13693.1.S1_at	225675_at	C14orf101	chromosome 14 open reading frame 101	chr14q23.1		54916	C7H14orf101	chromosome 14 open reading frame 101 ortholog	---	698254	MmugDNA.13693.1.S1_at_C14orf101
MmugDNA.13694.1.S1_at	218518_at	FAM13B	family with sequence similarity 13, member B	chr5q31		51306	---	---	---	---	MmugDNA.13694.1.S1_at_FAM13B
MmugDNA.13695.1.S1_at	217690_at	---	---	---	---		---	---	---	---	MmugDNA.13695.1.S1_at_---
MmugDNA.13696.1.S1_at	218023_s_at	FAM53C	family with sequence similarity 53, member C	chr5q31		51307	FAM53C	family with sequence similarity 53, member C	---	714176	MmugDNA.13696.1.S1_at_FAM53C
MmugDNA.13697.1.S1_at	208780_x_at	VAPA	VAMP (vesicle-associated membrane protein)-associated protein A, 33kDa	chr18p11.22		9218	LOC722335	similar to vesicle-associated membrane protein, associated protein a	---	722335	MmugDNA.13697.1.S1_at_VAPA
MmugDNA.13698.1.S1_at	1558770_a_at	PIK3R6	phosphoinositide-3-kinase, regulatory subunit 6	chr17p13.1		146850	LOC722023	similar to expressed sequence BB220380	---	722023	MmugDNA.13698.1.S1_at_PIK3R6
MmugDNA.13699.1.S1_at	NA	NA	NA	NA	NA		NA	NA	NA	NA	MmugDNA.13699.1.S1_at_NA
MmugDNA.13699.1.S1_s_at	220525_s_at	AUP1	ancient ubiquitous protein 1	chr2p13		550	LOC710020	similar to ancient ubiquitous protein 1	---	710020	MmugDNA.13699.1.S1_s_at_AUP1
MmugDNA.13700.1.S1_at	243814_at	ZMYND8	Zinc finger, MYND-type containing 8	chr20q13.12		23613	---	---	---	---	MmugDNA.13700.1.S1_at_ZMYND8
MmugDNA.13701.1.S1_at	NA	NA	NA	NA	NA		NA	NA	NA	NA	MmugDNA.13701.1.S1_at_NA
MmugDNA.1370.1.S1_at	214153_at	ELOVL5	ELOVL family member 5, elongation of long chain fatty acids (FEN1/Elo2, SUR4/Elo3-like, yeast)	chr6p21.1-p12.1		60481	---	---	---	---	MmugDNA.1370.1.S1_at_ELOVL5
MmugDNA.13702.1.S1_at	226177_at	GLTP	glycolipid transfer protein	chr12q24.11		51228	---	---	---	---	MmugDNA.13702.1.S1_at_GLTP
MmugDNA.13703.1.S1_at	229386_at	ID4	Inhibitor of DNA binding 4, dominant negative helix-loop-helix protein	chr6p22-p21		3400	ID4	inhibitor of DNA binding 4, dominant negative helix-loop-helix protein	---	705825	MmugDNA.13703.1.S1_at_ID4
MmugDNA.13704.1.S1_at	NA	NA	NA	NA	NA		NA	NA	NA	NA	MmugDNA.13704.1.S1_at_NA
MmugDNA.13705.1.S1_at	211548_s_at	HPGD	hydroxyprostaglandin dehydrogenase 15-(NAD)	chr4q34-q35		3248	HPGD	hydroxyprostaglandin dehydrogenase 15-(NAD)	---	697864	MmugDNA.13705.1.S1_at_HPGD
MmugDNA.13706.1.S1_at	237789_at	---	---	---	---		---	---	---	---	MmugDNA.13706.1.S1_at_---
MmugDNA.13707.1.S1_at	217424_at	---	---	---	---		---	---	---	---	MmugDNA.13707.1.S1_at_---
MmugDNA.13708.1.S1_at	209234_at	KIF1B	kinesin family member 1B	chr1p36.2		23095	---	---	---	---	MmugDNA.13708.1.S1_at_KIF1B
MmugDNA.13709.1.S1_at	238797_at	TRIM11	tripartite motif-containing 11	chr1q42.13		81559	---	---	---	---	MmugDNA.13709.1.S1_at_TRIM11
MmugDNA.13710.1.S1_at	1563946_at	---	---	---	---		---	---	---	---	MmugDNA.13710.1.S1_at_---
MmugDNA.13711.1.S1_at	1562710_at	---	---	---	---		---	---	---	---	MmugDNA.13711.1.S1_at_---
MmugDNA.1371.1.S1_at	227102_at	TRIM35	tripartite motif-containing 35	chr8p21.2		23087	LOC712720	similar to tripartite motif-containing 35	---	712720	MmugDNA.1371.1.S1_at_TRIM35
MmugDNA.13712.1.S1_at	1562909_at	C1orf98	chromosome 1 open reading frame 98	chr1q32.1		554279	---	---	---	---	MmugDNA.13712.1.S1_at_C1orf98
MmugDNA.13713.1.S1_at	209892_at	FUT4	fucosyltransferase 4 (alpha (1,3) fucosyltransferase, myeloid-specific)	chr11q21		2526	---	---	---	---	MmugDNA.13713.1.S1_at_FUT4
MmugDNA.13714.1.S1_at	209732_at	CLEC2B	C-type lectin domain family 2, member B	chr12p13-p12		9976	LOC719804	Similar to kallikrein 11 isoform 2 precursor	---	719804	MmugDNA.13714.1.S1_at_CLEC2B
MmugDNA.13715.1.S1_at	240948_at	---	---	---	---		---	---	---	---	MmugDNA.13715.1.S1_at_---
MmugDNA.13716.1.S1_s_at	NA	NA	NA	NA	NA		NA	NA	NA	NA	MmugDNA.13716.1.S1_s_at_NA
MmugDNA.13717.1.S1_at	NA	NA	NA	NA	NA		NA	NA	NA	NA	MmugDNA.13717.1.S1_at_NA
MmugDNA.13718.1.S1_at	205331_s_at	REEP2	receptor accessory protein 2	chr5q31		51308	LOC716686	similar to receptor expression enhancing protein 2	---	716686	MmugDNA.13718.1.S1_at_REEP2
MmugDNA.13719.1.S1_at	NA	NA	NA	NA	NA		NA	NA	NA	NA	MmugDNA.13719.1.S1_at_NA
MmugDNA.137.1.S1_at	209005_at	FBXL5	F-box and leucine-rich repeat protein 5	chr4p15.33		26234	---	---	---	---	MmugDNA.137.1.S1_at_FBXL5
MmugDNA.13720.1.S1_at	243445_at	BNC2	basonuclin 2	chr9p22.3-p22.2		54796	BNC2	basonuclin 2	---	713291	MmugDNA.13720.1.S1_at_BNC2
MmugDNA.13721.1.S1_at	244459_at	---	---	---	---		---	---	---	---	MmugDNA.13721.1.S1_at_---
MmugDNA.1372.1.S1_at	215155_at	HEXA	Hexosaminidase A (alpha polypeptide)	chr15q23-q24		3073	HEXA	hexosaminidase A	---	698251	MmugDNA.1372.1.S1_at_HEXA
MmugDNA.13722.1.S1_at	221777_at	C12orf52	chromosome 12 open reading frame 52	chr12q24.13		84934	LOC712837	hypothetical protein LOC712837	---	712837	MmugDNA.13722.1.S1_at_C12orf52
MmugDNA.13723.1.S1_at	NA	NA	NA	NA	NA		NA	NA	NA	NA	MmugDNA.13723.1.S1_at_NA
MmugDNA.13723.1.S1_s_at	218694_at	ARMCX1	armadillo repeat containing, X-linked 1	chrXq21.33-q22.2		51309	LOC714371	Similar to polypeptide N-acetylgalactosaminyltransferase 2	---	714371	MmugDNA.13723.1.S1_s_at_ARMCX1
MmugDNA.13724.1.S1_at	217867_x_at	BACE2	beta-site APP-cleaving enzyme 2	chr21q22.3		25825	---	---	---	---	MmugDNA.13724.1.S1_at_BACE2
MmugDNA.13725.1.S1_at	238670_at	---	---	---	---		LOC717415	Similar to putative aminopeptidase Fxna	---	717415	MmugDNA.13725.1.S1_at_---
MmugDNA.13726.1.S1_at	238533_at	EPHA7	EPH receptor A7	chr6q16.1		2045	---	---	---	---	MmugDNA.13726.1.S1_at_EPHA7
MmugDNA.13727.1.S1_at	217904_s_at	BACE1	beta-site APP-cleaving enzyme 1	chr11q23.2-q23.3		23621	LOC709640	Similar to NADH dehydrogenase (ubiquinone) 1 alpha subcomplex, 6, 14kDa	---	709640	MmugDNA.13727.1.S1_at_BACE1
MmugDNA.13728.1.S1_at	1562116_at	---	---	---	---		---	---	---	---	MmugDNA.13728.1.S1_at_---
MmugDNA.13729.1.S1_at	236082_at	PHLDB3	pleckstrin homology-like domain, family B, member 3	chr19q13.31		653583	LOC722275	similar to pleckstrin homology-like domain, family B, member 3	---	722275	MmugDNA.13729.1.S1_at_PHLDB3
MmugDNA.13730.1.S1_at	230781_at	---	---	---	---		---	---	---	---	MmugDNA.13730.1.S1_at_---
MmugDNA.13731.1.S1_at	229549_at	---	---	---	---		---	---	---	---	MmugDNA.13731.1.S1_at_---
MmugDNA.1373.1.S1_at	239751_at	---	---	---	---		---	---	---	---	MmugDNA.1373.1.S1_at_---
MmugDNA.13732.1.S1_at	231967_at	PHF20L1	PHD finger protein 20-like 1	chr8q24.22		51105	---	---	---	---	MmugDNA.13732.1.S1_at_PHF20L1
MmugDNA.13733.1.S1_at	221093_at	BRD7P3	bromodomain containing 7 pseudogene 3	chr6q22.1-q22.33		23629	BRD7 /// LOC711106	bromodomain containing 7 /// similar to bromodomain containing 7	---	694850 /// 711106	MmugDNA.13733.1.S1_at_BRD7P3
MmugDNA.13734.1.S1_at	226088_at	ZDHHC12	zinc finger, DHHC-type containing 12	chr9q34.11		84885	LOC722606	similar to zinc finger, DHHC domain containing 12	---	722606	MmugDNA.13734.1.S1_at_ZDHHC12
MmugDNA.13735.1.S1_at	239553_at	FLJ35379	similar to Alu subfamily J sequence contamination warning entry	chr13q22.2		729420	---	---	---	---	MmugDNA.13735.1.S1_at_FLJ35379
MmugDNA.13736.1.S1_at	233742_at	C16orf68	Chromosome 16 open reading frame 68	chr16p13.2		79091	---	---	---	---	MmugDNA.13736.1.S1_at_C16orf68
MmugDNA.13737.1.S1_at	201452_at	RHEB	Ras homolog enriched in brain	chr7q36		6009	LOC715378	similar to Ras homolog enriched in brain	---	715378	MmugDNA.13737.1.S1_at_RHEB
MmugDNA.13738.1.S1_at	216172_at	---	---	---	---		---	---	---	---	MmugDNA.13738.1.S1_at_---
MmugDNA.13739.1.S1_at	225101_s_at	SNX14	sorting nexin 14	chr6q14.3		57231	SNX14	sorting nexin 14	---	696632	MmugDNA.13739.1.S1_at_SNX14
MmugDNA.13740.1.S1_at	214254_at	MAGEA4	melanoma antigen family A, 4	chrXq28		4103	---	---	---	---	MmugDNA.13740.1.S1_at_MAGEA4
MmugDNA.13741.1.S1_at	223651_x_at	CDC23	cell division cycle 23 homolog (S. cerevisiae)	chr5q31		8697	---	---	---	---	MmugDNA.13741.1.S1_at_CDC23
MmugDNA.1374.1.S1_at	238769_at	---	---	---	---		---	---	---	---	MmugDNA.1374.1.S1_at_---
MmugDNA.13742.1.S1_at	NA	NA	NA	NA	NA		NA	NA	NA	NA	MmugDNA.13742.1.S1_at_NA
MmugDNA.13742.1.S1_s_at	223027_at	SNX9	sorting nexin 9	chr6q25.1-q26		51429	---	---	---	---	MmugDNA.13742.1.S1_s_at_SNX9
MmugDNA.13743.1.S1_at	202639_s_at	RANBP3	RAN binding protein 3	chr19p13.3		8498	LOC699953	Similar to solute carrier family 35, member B1	---	699953	MmugDNA.13743.1.S1_at_RANBP3
MmugDNA.13744.1.S1_at	1552675_at	DNAJB7	DnaJ (Hsp40) homolog, subfamily B, member 7	chr22q13.2		150353	DNAJB7	DnaJ (Hsp40) homolog, subfamily B, member 7	---	705916	MmugDNA.13744.1.S1_at_DNAJB7
MmugDNA.13745.1.S1_at	1556921_at	---	---	---	---		---	---	---	---	MmugDNA.13745.1.S1_at_---
MmugDNA.13746.1.S1_s_at	215977_x_at	GK	glycerol kinase	chrXp21.3		2710	GK	glycerol kinase	---	705001	MmugDNA.13746.1.S1_s_at_GK
MmugDNA.13747.1.S1_at	210616_s_at	SEC31A	SEC31 homolog A (S. cerevisiae)	chr4q21.22		22872	SEC31A	SEC31 homolog A (S. cerevisiae)	---	693687	MmugDNA.13747.1.S1_at_SEC31A
MmugDNA.13748.1.S1_at	206270_at	PRKCG	protein kinase C, gamma	chr19q13.4		5582	LOC720263	similar to protein kinase C, gamma	---	720263	MmugDNA.13748.1.S1_at_PRKCG
MmugDNA.13749.1.S1_s_at	205071_x_at	XRCC4	X-ray repair complementing defective repair in Chinese hamster cells 4	chr5q13-q14		7518	LOC712291	similar to X-ray repair cross complementing protein 4 isoform 1	---	712291	MmugDNA.13749.1.S1_s_at_XRCC4

MmugDNA.13750.1.S1_at	203733_at	DEXI	dexamethasone-induced transcript	chr16p13.13	28955	LOC716071	hypothetical protein LOC716071	---	716071	MmugDNA.13750.1.S1_at_DEXI
MmugDNA.13751.1.S1_at	219872_at	C4orf18	chromosome 4 open reading frame 18	chr4q32.1	51313	LOC700615	hypothetical protein LOC700615	---	700615	MmugDNA.13751.1.S1_at_C4orf18
MmugDNA.1375.1.S1_at	229652_s_at	LOC400236	hypothetical LOC400236	chr14q32.11	400236	---	---	---	---	MmugDNA.1375.1.S1_at_LOC400236
MmugDNA.13752.1.S1_at	219583_s_at	SPATA7	spermatogenesis associated 7	chr14q31.3	55812	SPATA7	spermatogenesis associated 7	---	693836	MmugDNA.13752.1.S1_at_SPATA7
MmugDNA.13753.1.S1_at	220384_at	TXNDC3	thioredoxin domain containing 3 (spermatozoa)	chr7p14.1	51314	TXNDC3	thioredoxin domain containing 3 (spermatozoa)	---	705583	MmugDNA.13753.1.S1_at_TXNDC3
MmugDNA.13754.1.S1_at	219005_at	TMEM59L	transmembrane protein 59-like	chr19p12	25789	LOC719572	brain-specific membrane-anchored protein	---	719572	MmugDNA.13754.1.S1_at_TMEM59L
MmugDNA.13755.1.S1_at	231128_at	FAM181B	family with sequence similarity 181, member B	chr11q14.1	220382	LOC701184	hypothetical protein LOC701184	---	701184	MmugDNA.13755.1.S1_at_FAM181B
MmugDNA.13756.1.S1_at	222585_x_at	KRCC1	lysine-rich coiled-coil 1	chr2p11.2	51315	---	---	---	---	MmugDNA.13756.1.S1_at_KRCC1
MmugDNA.13757.1.S1_at	NA	NA	NA	NA	NA	NA	NA	NA	NA	MmugDNA.13757.1.S1_at_NA
MmugDNA.13758.1.S1_at	218048_at	COMMD3	COMM domain containing 3	chr10pter-q22.1	23412	COMMD3	COMM domain containing 3	---	677699	MmugDNA.13758.1.S1_at_COMMD3
MmugDNA.13759.1.S1_at	1552944_a_at	PANX2	pannexin 2	chr22q13.33	56666	LOC720613	similar to pannexin 2	---	720613	MmugDNA.13759.1.S1_at_PANX2
MmugDNA.13760.1.S1_at	201491_at	AHSA1	AHA1, activator of heat shock 90kDa protein ATPase homolog 1 (yeast)	chr14q24	10598	AHSA1	AHA1, activator of heat shock 90kDa protein ATPase homolog 1	---	706547	MmugDNA.13760.1.S1_at_AHSA1
MmugDNA.13761.1.S1_at	233973_at	LYZL1 /// LYZL2	lysozyme-like 1 /// lysozyme-like 2	chr10p11.23	119180 /// 84569	LOC693706 /// LOC6981	similar to lysozyme-like 1 /// hypothetical protein LOC698176	---	693706 /// 698176	MmugDNA.13761.1.S1_at_LYZL1 /// LY
MmugDNA.1376.1.S1_at	234134_at	---	---	---	---	---	---	---	---	MmugDNA.1376.1.S1_at_---
MmugDNA.13762.1.S1_at	216839_at	LAMA2	Laminin, alpha 2	chr6q22-q23	3908	LOC715394	similar to laminin alpha 2 subunit precursor	---	715394	MmugDNA.13762.1.S1_at_LAMA2
MmugDNA.13762.1.S1_s_at	213519_s_at	LAMA2	laminin, alpha 2	chr6q22-q23	3908	LOC715394	similar to laminin alpha 2 subunit precursor	---	715394	MmugDNA.13762.1.S1_s_at_LAMA2
MmugDNA.13763.1.S1_at	1553314_a_at	KIF19	kinesin family member 19	chr17q25.1	124602	KIF19	kinesin family member 19	---	697090	MmugDNA.13763.1.S1_at_KIF19
MmugDNA.13764.1.S1_at	205525_at	CALD1	caldesmon 1	chr7q33	800	---	---	---	---	MmugDNA.13764.1.S1_at_CALD1
MmugDNA.13765.1.S1_at	243914_at	---	---	---	---	---	---	---	---	MmugDNA.13765.1.S1_at_---
MmugDNA.13766.1.S1_at	227362_at	SLC2A4RG	SLC2A4 regulator	chr20q13.33	56731	LOC695278	similar to SLC2A4 regulator	---	695278	MmugDNA.13766.1.S1_at_SLC2A4RG
MmugDNA.13767.1.S1_at	219936_s_at	GPR87	G protein-coupled receptor 87	chr3q24	53836	GPR87	G protein-coupled receptor 87	---	709766	MmugDNA.13767.1.S1_at_GPR87
MmugDNA.13768.1.S1_at	214036_at	EFNA5	ephrin-A5	chr5q21	1946	---	---	---	---	MmugDNA.13768.1.S1_at_EFNA5
MmugDNA.13769.1.S1_at	1559881_s_at	ZNF12	zinc finger protein 12	chr7p22.1	7559	ZNF12	zinc finger protein 12	---	705346	MmugDNA.13769.1.S1_at_ZNF12
MmugDNA.13770.1.S1_at	NA	NA	NA	NA	NA	NA	NA	NA	NA	MmugDNA.13770.1.S1_at_NA
MmugDNA.13771.1.S1_at	1552682_a_at	CASC5	cancer susceptibility candidate 5	chr15q14	57082	---	---	---	---	MmugDNA.13771.1.S1_at_CASC5
MmugDNA.1377.1.S1_at	216762_at	KANK1	KN motif and ankyrin repeat domains 1	chr9p24.3	23189	---	---	---	---	MmugDNA.1377.1.S1_at_KANK1
MmugDNA.13772.1.S1_at	239370_at	---	---	---	---	---	---	---	---	MmugDNA.13772.1.S1_at_---
MmugDNA.13773.1.S1_at	1557301_a_at	---	---	---	---	---	---	---	---	MmugDNA.13773.1.S1_at_---
MmugDNA.13774.1.S1_at	218011_at	UBL5	ubiquitin-like 5	chr19p13.3	59286	LOC711015	similar to ubiquitin-like 5	---	711015	MmugDNA.13774.1.S1_at_UBL5
MmugDNA.13775.1.S1_at	242820_at	---	---	---	---	---	---	---	---	MmugDNA.13775.1.S1_at_---
MmugDNA.13776.1.S1_s_at	209507_at	RPA3	replication protein A3, 14kDa	chr7p22	6119	RPA3	replication protein A3, 14kDa	---	696248	MmugDNA.13776.1.S1_s_at_RPA3
MmugDNA.13777.1.S1_at	212647_at	RRAS	related RAS viral (r-ras) oncogene homolog	chr19q13.3-qter	6237	RRAS	related RAS viral (r-ras) oncogene homolog	---	718939	MmugDNA.13777.1.S1_at_RRAS
MmugDNA.13778.1.S1_at	NA	NA	NA	NA	NA	NA	NA	NA	NA	MmugDNA.13778.1.S1_at_NA
MmugDNA.13778.1.S1_s_at	211878_at	IGHG1	Immunoglobulin heavy constant gamma 1 (G1m marker)	chr14q32.33	3500	---	---	---	---	MmugDNA.13778.1.S1_s_at_IGHG1
MmugDNA.13779.1.S1_at	235734_at	PACSIN3	protein kinase C and casein kinase substrate in neurons 3	chr11p12-p11.12	29763	LOC713919	similar to protein kinase C and casein kinase substrate in neurons 3	---	713919	MmugDNA.13779.1.S1_at_PACSIN3
MmugDNA.13780.1.S1_at	233633_at	TBL1XR1	Transducin (beta)-like 1 X-linked receptor 1	chr3q26.32	79718	---	---	---	---	MmugDNA.13780.1.S1_at_TBL1XR1
MmugDNA.13781.1.S1_at	218890_x_at	MRPL35	mitochondrial ribosomal protein L35	chr2p11.2	51318	LOC705494	hypothetical protein LOC705494	---	705494	MmugDNA.13781.1.S1_at_MRPL35
MmugDNA.1378.1.S1_at	210019_at	CALML3	calmodulin-like 3	chr10pter-p13	810	LOC722513	similar to Calmodulin-like protein 3 (Calmodulin-related protein NB-1) (CaM-like protein) (CLP)	---	722513	MmugDNA.1378.1.S1_at_CALML3
MmugDNA.13782.1.S1_at	219507_at	RSRC1	arginine/serine-rich coiled-coil 1	chr3q25.32	51319	---	---	---	---	MmugDNA.13782.1.S1_at_RSRC1
MmugDNA.13783.1.S1_at	241512_at	SPATC1	spermatogenesis and centriole associated 1	chr8q24.3	375686	LOC713021	similar to spermatogenesis and centriole associated 1	---	713021	MmugDNA.13783.1.S1_at_SPATC1
MmugDNA.13784.1.S1_at	216259_at	---	---	---	---	---	---	---	---	MmugDNA.13784.1.S1_at_---
MmugDNA.13785.1.S1_at	218167_at	AMZ2	archaelysin family metallopeptidase 2	chr17q24.2	51321	LOC718462	similar to archaemetzincins-2 isoform 1	---	718462	MmugDNA.13785.1.S1_at_AMZ2
MmugDNA.13786.1.S1_at	242717_at	---	---	---	---	---	---	---	---	MmugDNA.13786.1.S1_at_---
MmugDNA.13787.1.S1_at	228221_at	SLC44A3	solute carrier family 44, member 3	chr1p21.3	126969	SLC44A3	solute carrier family 44, member 3	---	714768	MmugDNA.13787.1.S1_at_SLC44A3
MmugDNA.13788.1.S1_s_at	210708_x_at	CASP10	caspase 10, apoptosis-related cysteine peptidase	chr2q33-q34	843	CASP10	caspase 10, apoptosis-related cysteine peptidase	---	701295	MmugDNA.13788.1.S1_s_at_CASP10
MmugDNA.13789.1.S1_at	228151_at	---	---	---	---	---	---	---	---	MmugDNA.13789.1.S1_at_---
MmugDNA.13790.1.S1_at	217742_s_at	WAC	WW domain containing adaptor with coiled-coil	---	51322	---	---	---	---	MmugDNA.13790.1.S1_at_WAC
MmugDNA.1379.1.S1_at	227638_at	KIAA1632	KIAA1632	chr18q12.3-q21.1	57724	LOC700766	similar to CG14299-PA, isoform A	---	700766	MmugDNA.1379.1.S1_at_KIAA1632
MmugDNA.13792.1.S1_at	220638_s_at	CBLC	Cas-Br-M (murine) ecotropic retroviral transforming sequence c	chr19q13.2	23624	LOC713112	similar to Signal transduction protein CBL-C (SH3-binding protein CBL-C) (CBL-3) (RING finger prote	---	713112	MmugDNA.13792.1.S1_at_CBLC
MmugDNA.13793.1.S1_at	235965_at	---	---	---	---	---	---	---	---	MmugDNA.13793.1.S1_at_---
MmugDNA.13794.1.S1_at	241255_at	---	---	---	---	---	---	---	---	MmugDNA.13794.1.S1_at_---
MmugDNA.13795.1.S1_at	218037_at	FAM134A	family with sequence similarity 134, member A	chr2q35	79137	LOC704378	similar to Protein C2orf17 homolog	---	704378	MmugDNA.13795.1.S1_at_FAM134A
MmugDNA.13796.1.S1_at	231353_at	---	---	---	---	---	---	---	---	MmugDNA.13796.1.S1_at_---
MmugDNA.13797.1.S1_at	205457_at	C6orf106	chromosome 6 open reading frame 106	chr6p21.31	64771	LOC718675	similar to chromosome 6 open reading frame 106 isoform a	---	718675	MmugDNA.13797.1.S1_at_C6orf106
MmugDNA.13797.1.S1_s_at	NA	NA	NA	NA	NA	NA	NA	NA	NA	MmugDNA.13797.1.S1_s_at_NA
MmugDNA.13798.1.S1_at	NA	NA	NA	NA	NA	NA	NA	NA	NA	MmugDNA.13798.1.S1_at_NA
MmugDNA.13798.1.S1_s_at	NA	NA	NA	NA	NA	NA	NA	NA	NA	MmugDNA.13798.1.S1_s_at_NA
MmugDNA.13799.1.S1_at	212980_at	USP34	ubiquitin specific peptidase 34	chr2p15	9736	---	---	---	---	MmugDNA.13799.1.S1_at_USP34
MmugDNA.13800.1.S1_at	219402_s_at	DERL1	Der1-like domain family, member 1	chr8q24.13	79139	DERL1	Der1-like domain family, member 1	---	703803	MmugDNA.13800.1.S1_at_DERL1
MmugDNA.13801.1.S1_at	219585_at	CCDC28B	coiled-coil domain containing 28B	chr1p35.1	79140	CCDC28B	coiled-coil domain containing 28B	---	707005	MmugDNA.13801.1.S1_at_CCDC28B
MmugDNA.1380.1.S1_at	1562286_at	---	---	---	---	---	---	---	---	MmugDNA.1380.1.S1_at_---
MmugDNA.13802.1.S1_at	209335_at	DCN	decorin	chr12q21.33	1634	DCN	decorin	---	709933	MmugDNA.13802.1.S1_at_DCN
MmugDNA.13803.1.S1_at	215634_at	---	---	---	---	---	---	---	---	MmugDNA.13803.1.S1_at_---
MmugDNA.13804.1.S1_at	235699_at	REM2	RAS (RAD and GEM)-like GTP binding 2	chr14q11.2	161253	LOC712572	similar to rad and gem related GTP binding protein 2	---	712572	MmugDNA.13804.1.S1_at_REM2
MmugDNA.13805.1.S1_at	217599_s_at	MDFIC	MyoD family inhibitor domain containing	chr7q31.1-q31.2	29969	LOC706236	hypothetical protein LOC706236	---	706236	MmugDNA.13805.1.S1_at_MDFIC
MmugDNA.13806.1.S1_at	215324_at	SEMA3D	sema domain, immunoglobulin domain (Ig), short basic domain, secreted, (semaphorin) 3D	chr7q21.11	223117	SEMA3D	sema domain, immunoglobulin domain (Ig), short basic domain, secreted, (semaphorin) 3D	---	707146	MmugDNA.13806.1.S1_at_SEMA3D
MmugDNA.13807.1.S1_at	236257_at	CD2AP	CD2-associated protein	chr6p12	23607	CD2AP	CD2-associated protein	---	705221	MmugDNA.13807.1.S1_at_CD2AP
MmugDNA.13808.1.S1_at	1559391_s_at	---	---	---	---	---	---	---	---	MmugDNA.13808.1.S1_at_---
MmugDNA.13809.1.S1_at	1555394_at	PIGK	phosphatidylinositol glycan anchor biosynthesis, class K	chr1p31.1	10026	---	---	---	---	MmugDNA.13809.1.S1_at_PIGK
MmugDNA.13810.1.S1_at	209303_at	NDUFS4	NADH dehydrogenase (ubiquinone) Fe-S protein 4, 18kDa (NADH-coenzyme Q reductase)	chr5q11.1	4724	NDUFS4	NADH dehydrogenase (ubiquinone) Fe-S protein 4, 18kDa (NADH-coenzyme Q reductase)	---	704137	MmugDNA.13810.1.S1_at_NDUFS4
MmugDNA.13811.1.S1_at	244415_at	---	---	---	---	---	---	---	---	MmugDNA.13811.1.S1_at_---
MmugDNA.1381.1.S1_at	235031_at	---	---	---	---	---	---	---	---	MmugDNA.1381.1.S1_at_---
MmugDNA.13812.1.S1_at	220671_at	CCRN4L	CCR4 carbon catabolite repression 4-like (S. cerevisiae)	chr4q31.1	25819	LOC717120	similar to CCR4 carbon catabolite repression 4-like	---	717120	MmugDNA.13812.1.S1_at_CCRN4L
MmugDNA.13813.1.S1_at	227614_at	HKDC1	hexokinase domain containing 1	chr10q21.3	80201	LOC711995	similar to hexokinase domain containing 1	---	711995	MmugDNA.13813.1.S1_at_HKDC1
MmugDNA.13814.1.S1_at	237325_at	C22orf43	chromosome 22 open reading frame 43	chr22q11.2	51233	---	---	---	---	MmugDNA.13814.1.S1_at_C22orf43
MmugDNA.13815.1.S1_at	218515_at	C21orf66	chromosome 21 open reading frame 66	chr21q21.3	94104	---	---	---	---	MmugDNA.13815.1.S1_at_C21orf66
MmugDNA.13816.1.S1_at	227995_at	---	---	---	---	---	---	---	---	MmugDNA.13816.1.S1_at_---
MmugDNA.13817.1.S1_at	217595_at	GSPT1	G1 to S phase transition 1	chr16p13.1	2935	---	---	---	---	MmugDNA.13817.1.S1_at_GSPT1
MmugDNA.13818.1.S1_at	1561899_at	CLECL1	C-type lectin-like 1	chr12p13.31	160365	---	---	---	---	MmugDNA.13818.1.S1_at_CLECL1
MmugDNA.13819.1.S1_at	1554992_at	RASGRF1	Ras protein-specific guanine nucleotide-releasing factor 1	chr15q24.2	5923	LOC717062	similar to Guanine nucleotide-releasing protein (GNRP) (Ras-specific nucleotide exchange factor CD(	---	717062	MmugDNA.13819.1.S1_at_RASGRF1
MmugDNA.138.1.S1_at	1561979_at	---	---	---	---	---	---	---	---	MmugDNA.138.1.S1_at_---
MmugDNA.13820.1.S1_at	242840_at	---	---	---	---	---	---	---	---	MmugDNA.13820.1.S1_at_---
MmugDNA.13821.1.S1_s_at	NA	NA	NA	NA	NA	NA	NA	NA	NA	MmugDNA.13821.1.S1_s_at_NA
MmugDNA.13821.1.S1_x_at	NA	NA	NA	NA	NA	NA	NA	NA	NA	MmugDNA.13821.1.S1_x_at_NA
MmugDNA.1382.1.S1_at	241696_at	CNTLN	centlein, centrosomal protein	chr9p22.2	54875	LOC713588	hypothetical protein LOC713588	---	713588	MmugDNA.1382.1.S1_at_CNTLN
MmugDNA.13823.1.S1_at	235613_at	---	---	---	---	---	---	---	---	MmugDNA.13823.1.S1_at_---
MmugDNA.13824.1.S1_at	226307_at	CRTC2	CREB regulated transcription coactivator 2	chr1q21.3	200186	LOC715908	similar to transducer of regulated cAMP response element-binding protein (CREB) 2	---	715908	MmugDNA.13824.1.S1_at_CRTC2
MmugDNA.13825.1.S1_at	236931_at	---	---	---	---	---	---	---	---	MmugDNA.13825.1.S1_at_---
MmugDNA.13826.1.S1_at	211625_s_at	DRD3	dopamine receptor D3	chr3q13.3	1814	---	---	---	---	MmugDNA.13826.1.S1_at_DRD3
MmugDNA.13827.1.S1_at	1554348_s_at	CDKN2AIPNL	CDKN2A interacting protein N-terminal like	chr5q31.1	91368	LOC710774	hypothetical protein LOC710774	---	710774	MmugDNA.13827.1.S1_at_CDKN2AIPN
MmugDNA.13828.1.S1_at	243610_at	C9orf135	chromosome 9 open reading frame 135	chr9q21.11	138255	LOC700202	hypothetical protein LOC700202	---	700202	MmugDNA.13828.1.S1_at_C9orf135
MmugDNA.13829.1.S1_s_at	227549_x_at	ZDHHC24	zinc finger, DHHC-type containing 24	chr11q13.1	254359	LOC713870	similar to zinc finger, DHHC domain containing 24	---	713870	MmugDNA.13829.1.S1_s_at_ZDHHC24
MmugDNA.13830.1.S1_at	221880_s_at	FAM174B	family with sequence similarity 174, member B	chr15q26.1	400451	---	---	---	---	MmugDNA.13830.1.S1_at_FAM174B
MmugDNA.13831.1.S1_at	239790_s_at	---	---	---	---	---	---	---	---	MmugDNA.13831.1.S1_at_---
MmugDNA.1383.1.S1_at	242382_at	---	---	---	---	---	---	---	---	MmugDNA.1383.1.S1_at_---
MmugDNA.13832.1.S1_s_at	218010_x_at	C20orf149	chromosome 20 open reading frame 149	chr20q13.33	79144	LOC719608	hypothetical protein LOC719608	---	719608	MmugDNA.13832.1.S1_s_at_C20orf149
MmugDNA.13833.1.S1_at	222701_s_at	CHCHD7	coiled-coil-helix-coiled-coil-helix domain containing 7	chr8q12.1	79145	CHCHD7	coiled-coil-helix-coiled-coil-helix domain containing 7	---	699551	MmugDNA.13833.1.S1_at_CHCHD7
MmugDNA.13834.1.S1_at	NA	NA	NA	NA	NA	NA	NA	NA	NA	MmugDNA.13834.1.S1_at_NA
MmugDNA.13835.1.S1_at	219853_at	FKRP	fukutin related protein	chr19q13.32	79147	LOC716804	similar to fukutin-related protein	---	716804	MmugDNA.13835.1.S1_at_FKRP
MmugDNA.13836.1.S1_at	227400_at	NFIX	nuclear factor I/X (CCAAT-binding transcription factor)	chr19p13.3	4784	---	---	---	---	MmugDNA.13836.1.S1_at_NFIX
MmugDNA.13837.1.S1_at	231862_at	CBX5	chromobox homolog 5 (HP1 alpha homolog, Drosophila)	chr12q13.13	23468	---	---	---	---	MmugDNA.13837.1.S1_at_CBX5
MmugDNA.13838.1.S1_at	229532_at	ZNF502	zinc finger protein 502	chr3p21.31	91392	LOC708167	Hypothetical protein LOC708167	---	708167	MmugDNA.13838.1.S1_at_ZNF502
MmugDNA.13839.1.S1_at	240500_at	LOC100132369	Hypothetical protein LOC100132369	chr12p13.33	100132369	---	---	---	---	MmugDNA.13839.1.S1_at_LOC100132
MmugDNA.13840.1.S1_at	223878_at	INPP4B	inositol polyphosphate-4-phosphatase, type II, 105kDa	chr4q31.21	8821	---	---	---	---	MmugDNA.13840.1.S1_at_INPP4B
MmugDNA.13841.1.S1_at	222137_at	CC2D1A	coiled-coil and C2 domain containing 1A	chr19p13.12	54862	LOC718483	similar to coiled-coil and C2 domain containing 1A	---	718483	MmugDNA.13841.1.S1_at_CC2D1A
MmugDNA.1384.1.S1_at	238863_x_at	---	---	---	---	---	---	---	---	MmugDNA.1384.1.S1_at_---
MmugDNA.13842.1.S1_at	218216_x_at	ARL6IP4	ADP-ribosylation-like factor 6 interacting protein 4	chr12q24.31	51329	ARL6IP4	similar to SRp25 nuclear protein isoform 2	---	707810	MmugDNA.13842.1.S1_at_ARL6IP4
MmugDNA.13843.1.S1_at	238522_at	USP54	Ubiquitin specific peptidase 54	chr10q22.2	159195	---	---	---	---	MmugDNA.13843.1.S1_at_USP54

MmugDNA.13844.1.S1_at	234431_at	---	---	---	---	---	---	---	---	MmugDNA.13844.1.S1_at_---
MmugDNA.13845.1.S1_at	1560094_at	---	---	---	---	---	---	---	---	MmugDNA.13845.1.S1_at_---
MmugDNA.13846.1.S1_at	218368_s_at	TNFRSF12A	tumor necrosis factor receptor superfamily, member 12A	chr16p13.3	51330	TNFRSF12A	tumor necrosis factor receptor superfamily, member 12A	---	699970	MmugDNA.13846.1.S1_at_TNFRSF12A
MmugDNA.13847.1.S1_at	1556255_a_at	LOC400756	hypothetical gene supported by BC030752	chr1p31.3	400756	---	---	---	---	MmugDNA.13847.1.S1_at_LOC400756
MmugDNA.13848.1.S1_at	220067_at	SPTBN5	spectrin, beta, non-erythrocytic 5	chr15q21	51332	SPTBN5	spectrin, beta, non-erythrocytic 5	---	707159	MmugDNA.13848.1.S1_at_SPTBN5
MmugDNA.13849.1.S1_at	214405_at	---	---	---	---	---	---	---	---	MmugDNA.13849.1.S1_at_---
MmugDNA.13850.1.S1_at	1568866_at	---	---	---	---	---	---	---	---	MmugDNA.13850.1.S1_at_---
MmugDNA.13851.1.S1_at	236682_at	---	---	---	---	---	---	---	---	MmugDNA.13851.1.S1_at_---
MmugDNA.1385.1.S1_at	235820_at	LOC100130219	Similar to hCG2041987	chr12p13.33	100130219	---	---	---	---	MmugDNA.1385.1.S1_at_LOC1001302
MmugDNA.13852.1.S1_at	219904_at	ZSCAN5A	zinc finger and SCAN domain containing 5A	chr19q13.43	79149	LOC705629	similar to zinc finger and SCAN domain containing 5	---	705629	MmugDNA.13852.1.S1_at_ZSCAN5A
MmugDNA.13853.1.S1_at	222721_at	DOCK5	Dedicator of cytokinesis 5	chr8p21.2	80005	LOC711359	similar to dedicator of cytokinesis 5	---	711359	MmugDNA.13853.1.S1_at_DOCK5
MmugDNA.13854.1.S1_at	217386_at	---	---	---	---	---	---	---	---	MmugDNA.13854.1.S1_at_---
MmugDNA.13855.1.S1_at	207775_at	MGC4859	hypothetical LOC79150	chr7p	79150	---	---	---	---	MmugDNA.13855.1.S1_at_MGC4859
MmugDNA.13856.1.S1_at	215668_s_at	PLXNB1	plexin B1	chr3p21.31	5364	LOC709802	similar to plexin B1	---	709802	MmugDNA.13856.1.S1_at_PLXNB1
MmugDNA.13857.1.S1_at	234842_at	---	---	---	---	---	---	---	---	MmugDNA.13857.1.S1_at_---
MmugDNA.13858.1.S1_at	NA	NA	NA	NA	NA	NA	NA	NA	NA	MmugDNA.13858.1.S1_at_NA
MmugDNA.13859.1.S1_at	244275_at	---	---	---	---	---	---	---	---	MmugDNA.13859.1.S1_at_---
MmugDNA.13860.1.S1_at	241364_at	TMEM57	transmembrane protein 57	chr1p36.11|1p36.11	55219	---	---	---	---	MmugDNA.13860.1.S1_at_TMEM57
MmugDNA.13861.1.S1_at	219429_at	FA2H	fatty acid 2-hydroxylase	chr16q23	79152	CRP	C-reactive protein, pentraxin-related	---	719454	MmugDNA.13861.1.S1_at_FA2H
MmugDNA.1386.1.S1_at	241459_at	LIMCH1	LIM and calponin homology domains 1	chr4p13	22998	---	---	---	---	MmugDNA.1386.1.S1_at_LIMCH1
MmugDNA.13862.1.S1_at	235021_at	KIAA2026	KIAA2026	chr9p24.1	158358	KIAA2026	KIAA2026 protein	---	717388	MmugDNA.13862.1.S1_at_KIAA2026
MmugDNA.13863.1.S1_at	228887_x_at	CCDC61	coiled-coil domain containing 61	chr19q13.32	729440	---	---	---	---	MmugDNA.13863.1.S1_at_CCDC61
MmugDNA.13864.1.S1_at	219722_s_at	GDPD3	glycerophosphodiester phosphodiesterase domain containing 3	chr16p11.2	79153	LOC708824	similar to glycerophosphodiester phosphodiesterase domain containing 3 isoform 2	---	708824	MmugDNA.13864.1.S1_at_GDPD3
MmugDNA.13865.1.S1_at	229295_at	LOC150166	hypothetical protein LOC150166	chr22q11.21	150166	---	---	---	---	MmugDNA.13865.1.S1_at_LOC150166
MmugDNA.13866.1.S1_at	236113_at	---	---	---	---	---	---	---	---	MmugDNA.13866.1.S1_at_---
MmugDNA.13867.1.S1_at	1570335_at	---	---	---	---	---	---	---	---	MmugDNA.13867.1.S1_at_---
MmugDNA.13868.1.S1_at	242650_at	---	---	---	---	---	---	---	---	MmugDNA.13868.1.S1_at_---
MmugDNA.13869.1.S1_x_at	NA	NA	NA	NA	NA	NA	NA	NA	NA	MmugDNA.13869.1.S1_x_at_NA
MmugDNA.13870.1.S1_at	230487_at	C6orf99 /// LOC10013	chromosome 6 open reading frame 99 /// similar to hCG2044932	chr6q25.3	100130967 /// 389440	---	---	---	---	MmugDNA.13870.1.S1_at_C6orf99 /// L
MmugDNA.13871.1.S1_at	240777_at	SYNE2	Spectrin repeat containing, nuclear envelope 2	chr14q23.2	23224	---	---	---	---	MmugDNA.13871.1.S1_at_SYNE2
MmugDNA.1387.1.S1_at	228243_at	---	---	---	---	---	---	---	---	MmugDNA.1387.1.S1_at_---
MmugDNA.13872.1.S1_at	220014_at	PRR16	proline rich 16	chr5q23.1	51334	---	---	---	---	MmugDNA.13872.1.S1_at_PRR16
MmugDNA.13873.1.S1_at	227359_at	C1orf102	chromosome 1 open reading frame 102	chr1p34.3	127700	LOC713350	similar to oxidored-nitro domain-containing protein isoform 1	---	713350	MmugDNA.13873.1.S1_at_C1orf102
MmugDNA.13874.1.S1_at	228782_at	SCGB3A2	secretoglobin, family 3A, member 2	chr5q32	117156	LOC709138	similar to secretoglobin, family 3A, member 2	---	709138	MmugDNA.13874.1.S1_at_SCGB3A2
MmugDNA.13875.1.S1_at	217722_s_at	NGRN	neugrin, neurite outgrowth associated	chr15q26.1	51335	LOC702187	similar to mesenchymal stem cell protein DSC92 isoform 2	---	702187	MmugDNA.13875.1.S1_at_NGRN
MmugDNA.13876.1.S1_at	242311_x_at	---	---	---	---	---	---	---	---	MmugDNA.13876.1.S1_at_---
MmugDNA.13877.1.S1_at	208154_at	---	---	---	---	---	---	---	---	MmugDNA.13877.1.S1_at_---
MmugDNA.13878.1.S1_at	1554608_at	TGOLN2	trans-golgi network protein 2	chr2p11.2	10618	---	---	---	---	MmugDNA.13878.1.S1_at_TGOLN2
MmugDNA.13879.1.S1_at	235103_at	MAN2A1	mannosidase, alpha, class 2A, member 1	chr5q21-q22	4124	LOC705480	similar to mannosidase, alpha, class 2A, member 1	---	705480	MmugDNA.13879.1.S1_at_MAN2A1
MmugDNA.13880.1.S1_at	217060_at	---	---	---	---	LOC701875	similar to T-cell receptor beta chain V region 3H.25 precursor	---	701875	MmugDNA.13880.1.S1_at_---
MmugDNA.13881.1.S1_at	218500_at	C8orf55	chromosome 8 open reading frame 55	chr8q24.3	51337	LOC703842	similar to mesenchymal stem cell protein DSCD75	---	703842	MmugDNA.13881.1.S1_at_C8orf55
MmugDNA.1388.1.S1_at	236670_s_at	---	---	---	---	---	---	---	---	MmugDNA.1388.1.S1_at_---
MmugDNA.13882.1.S1_at	218756_s_at	DHRS11	dehydrogenase/reductase (SDR family) member 11	chr17q12	79154	LOC713823	similar to short-chain dehydrogenase/reductase	---	713823	MmugDNA.13882.1.S1_at_DHRS11
MmugDNA.13883.1.S1_at	234635_at	KRTAP4-1	keratin associated protein 4-1	chr17q12-q21	85285	LOC702327	hypothetical protein LOC702327	---	702327	MmugDNA.13883.1.S1_at_KRTAP4-1
MmugDNA.13884.1.S1_at	232160_s_at	TNIP2	TNFAIP3 interacting protein 2	chr4p16.3	79155	LOC722308	similar to A20-binding inhibitor of NF-kappaB activation 2	---	722308	MmugDNA.13884.1.S1_at_TNIP2
MmugDNA.13885.1.S1_at	215387_x_at	---	---	---	---	---	---	---	---	MmugDNA.13885.1.S1_at_---
MmugDNA.13885.1.S1_x_at	NA	NA	NA	NA	NA	NA	NA	NA	NA	MmugDNA.13885.1.S1_x_at_NA
MmugDNA.13886.1.S1_at	219566_at	PLEKHF1	pleckstrin homology domain containing, family F (with FYVE domain) member 1	chr19q12	79156	LOC700343	similar to apoptosis-inducing protein D	---	700343	MmugDNA.13886.1.S1_at_PLEKHF1
MmugDNA.13887.1.S1_s_at	NA	NA	NA	NA	NA	NA	NA	NA	NA	MmugDNA.13887.1.S1_s_at_NA
MmugDNA.13887.1.S1_x_at	NA	NA	NA	NA	NA	NA	NA	NA	NA	MmugDNA.13887.1.S1_x_at_NA
MmugDNA.13888.1.S1_at	NA	NA	NA	NA	NA	NA	NA	NA	NA	MmugDNA.13888.1.S1_at_NA
MmugDNA.13889.1.S1_at	1563027_at	---	---	---	---	LOC715619	Hypothetical protein LOC715619	---	715619	MmugDNA.13889.1.S1_at_---
MmugDNA.13890.1.S1_at	215896_at	---	---	---	---	---	---	---	---	MmugDNA.13890.1.S1_at_---
MmugDNA.13891.1.S1_at	216289_at	GPR144	G protein-coupled receptor 144	chr9q33.3	347088	---	---	---	---	MmugDNA.13891.1.S1_at_GPR144
MmugDNA.1389.1.S1_at	217196_s_at	CAMSAP1L1	calmodulin regulated spectrin-associated protein 1-like 1	chr1q32.1	23271	---	---	---	---	MmugDNA.1389.1.S1_at_CAMSAP1L1
MmugDNA.13892.1.S1_at	229341_at	TFCP2L1	transcription factor CP2-like 1	chr2q14	29842	---	---	---	---	MmugDNA.13892.1.S1_at_TFCP2L1
MmugDNA.13893.1.S1_at	202634_at	POLR2K	polymerase (RNA) II (DNA directed) polypeptide K, 7.0kDa	chr8q22.2	5440	POLR2K	polymerase (RNA) II (DNA directed) polypeptide K, 7.0kDa	---	708464	MmugDNA.13893.1.S1_at_POLR2K
MmugDNA.13894.1.S1_at	1557705_a_at	---	---	---	---	---	---	---	---	MmugDNA.13894.1.S1_at_---
MmugDNA.13895.1.S1_at	210870_s_at	EPM2A	epilepsy, progressive myoclonus type 2A, Lafora disease (laforin)	chr6q24	7957	EPM2A	epilepsy, progressive myoclonus type 2A, Lafora disease (laforin)	---	699361	MmugDNA.13895.1.S1_at_EPM2A
MmugDNA.13896.1.S1_at	1563348_at	---	---	---	---	---	---	---	---	MmugDNA.13896.1.S1_at_---
MmugDNA.13897.1.S1_s_at	226460_at	FNIP2	folliculin interacting protein 2	chr4q32.1	57600	---	---	---	---	MmugDNA.13897.1.S1_s_at_FNIP2
MmugDNA.13898.1.S1_at	243947_s_at	---	---	---	---	---	---	---	---	MmugDNA.13898.1.S1_at_---
MmugDNA.13899.1.S1_at	NA	NA	NA	NA	NA	NA	NA	NA	NA	MmugDNA.13899.1.S1_at_NA
MmugDNA.13900.1.S1_at	1569080_at	RNF165	ring finger protein 165	chr18q21.1	494470	---	---	---	---	MmugDNA.13900.1.S1_at_RNF165
MmugDNA.13901.1.S1_at	34221_at	HMGXB3	HMG box domain containing 3	chr5q33.1	22993	LOC710180	similar to SMF protein	---	710180	MmugDNA.13901.1.S1_at_HMGXB3
MmugDNA.1390.1.S1_at	240252_at	---	---	---	---	---	---	---	---	MmugDNA.1390.1.S1_at_---
MmugDNA.13902.1.S1_at	213717_at	LDB3	LIM domain binding 3	chr10q22.3-q23.2	11155	---	---	---	---	MmugDNA.13902.1.S1_at_LDB3
MmugDNA.13903.1.S1_at	242009_at	SLC6A4	solute carrier family 6 (neurotransmitter transporter, serotonin), member 4	chr17q11.1-q12	6532	---	---	---	---	MmugDNA.13903.1.S1_at_SLC6A4
MmugDNA.13904.1.S1_at	60084_at	CYLD	cylindromatosis (turban tumor syndrome)	chr16q12.1	1540	---	---	---	---	MmugDNA.13904.1.S1_at_CYLD
MmugDNA.13905.1.S1_at	238989_at	C1GALT1C1	C1GALT1-specific chaperone 1	chrXq24	29071	---	---	---	---	MmugDNA.13905.1.S1_at_C1GALT1C1
MmugDNA.13906.1.S1_at	220398_at	GNPTAB	N-acetylglucosamine-1-phosphate transferase, alpha and beta subunits	chr12q23.2	79158	---	---	---	---	MmugDNA.13906.1.S1_at_GNPTAB
MmugDNA.13907.1.S1_at	219324_at	NOL12 /// TRIOBP	nucleolar protein 12 /// TRIO and F-actin binding protein	chr22q13.1	11078 /// 79159	LOC698318	hypothetical protein LOC698318	---	698318	MmugDNA.13907.1.S1_at_NOL12 /// TF
MmugDNA.13908.1.S1_at	227250_at	KREMEN1	kringle containing transmembrane protein 1	chr22q12.1	83999	---	---	---	---	MmugDNA.13908.1.S1_at_KREMEN1
MmugDNA.13909.1.S1_at	241600_at	---	---	---	---	---	---	---	---	MmugDNA.13909.1.S1_at_---
MmugDNA.13910.1.S1_at	220698_at	MGC4294	hypothetical protein MGC4294	chr20q13.32	79160	---	---	---	---	MmugDNA.13910.1.S1_at_MGC4294
MmugDNA.13911.1.S1_s_at	204215_at	C7orf23	chromosome 7 open reading frame 23	chr7q21.1-q21.2	79161	C3H7orf23	chromosome 7 open reading frame 23 ortholog	---	706405	MmugDNA.13911.1.S1_s_at_C7orf23
MmugDNA.1391.1.S1_at	206318_at	SPINLW1	serine peptidase inhibitor-like, with Kunitz and WAP domains 1 (eppin)	chr20q12-q13.2	57119	---	---	---	---	MmugDNA.1391.1.S1_at_SPINLW1
MmugDNA.13912.1.S1_at	1552585_s_at	GTF2A1L /// STON1	general transcription factor IIA, 1-like /// stonin 1	chr2p16.3	11036 /// 11037	LOC715957	similar to stoned B/TFIIA-alpha/beta-like factor	---	715957	MmugDNA.13912.1.S1_at_GTF2A1L ///
MmugDNA.13913.1.S1_at	219063_at	C1orf35	chromosome 1 open reading frame 35	chr1q42.13	79169	C1H1orf35	chromosome 1 open reading frame 35 ortholog	---	695723	MmugDNA.13913.1.S1_at_C1orf35
MmugDNA.13914.1.S1_at	226614_s_at	FAM167A	family with sequence similarity 167, member A	chr8p23-p22	83648	---	---	---	---	MmugDNA.13914.1.S1_at_FAM167A
MmugDNA.13915.1.S1_at	240234_at	---	---	---	---	---	---	---	---	MmugDNA.13915.1.S1_at_---
MmugDNA.13916.1.S1_at	233642_s_at	HEATR5B	HEAT repeat containing 5B	chr2p22.2	54497	LOC713230	Similar to prostate tumor overexpressed gene 1	---	713230	MmugDNA.13916.1.S1_at_HEATR5B
MmugDNA.13917.1.S1_at	228132_at	ABLIM2	actin binding LIM protein family, member 2	chr4p16.1	84448	---	---	---	---	MmugDNA.13917.1.S1_at_ABLIM2
MmugDNA.13918.1.S1_at	214825_at	FAM155A	family with sequence similarity 155, member A	chr13q33.3	728215	LOC698735	Similar to sex comb on midleg-like 4	---	698735	MmugDNA.13918.1.S1_at_FAM155A
MmugDNA.13919.1.S1_at	234599_at	---	---	---	---	---	---	---	---	MmugDNA.13919.1.S1_at_---
MmugDNA.139.1.S1_at	202297_s_at	RER1	RER1 retention in endoplasmic reticulum 1 homolog (S. cerevisiae)	chr1pter-q24	11079	RER1	RER1 retention in endoplasmic reticulum 1 homolog (S. cerevisiae)	---	694540	MmugDNA.139.1.S1_at_RER1
MmugDNA.13920.1.S1_at	228470_at	LOC731109	similar to ring finger protein 187	---	731109	RNF187	ring finger protein 187	---	694637	MmugDNA.13920.1.S1_at_LOC731109
MmugDNA.13921.1.S1_at	234179_at	---	---	---	---	---	---	---	---	MmugDNA.13921.1.S1_at_---
MmugDNA.1392.1.S1_at	207563_s_at	OGT	O-linked N-acetylglucosamine (GlcNAc) transferase (UDP-N-acetylglucosamine:polypeptide-N-acetyl	chrXq13	8473	LOC698268	similar to O-linked GlcNAc transferase isoform 1	---	698268	MmugDNA.1392.1.S1_at_OGT
MmugDNA.13922.1.S1_at	1561868_at	---	---	---	---	---	---	---	---	MmugDNA.13922.1.S1_at_---
MmugDNA.13924.1.S1_at	200904_at	HLA-E	major histocompatibility complex, class I, E	chr6p21.3	3133	---	---	---	---	MmugDNA.13924.1.S1_at_HLA-E
MmugDNA.13925.1.S1_at	NA	NA	NA	NA	NA	NA	NA	NA	NA	MmugDNA.13925.1.S1_at_NA
MmugDNA.13926.1.S1_at	225098_at	ABI2	abl interactor 2	chr2q33	10152	---	---	---	---	MmugDNA.13926.1.S1_at_ABI2
MmugDNA.13927.1.S1_at	236715_x_at	UACA	uveal autoantigen with coiled-coil domains and ankyrin repeats	chr15q22-q24	55075	UACA	uveal autoantigen with coiled-coil domains and ankyrin repeats	---	696026	MmugDNA.13927.1.S1_at_UACA
MmugDNA.13927.1.S1_x_at	NA	NA	NA	NA	NA	NA	NA	NA	NA	MmugDNA.13927.1.S1_x_at_NA
MmugDNA.13928.1.S1_at	1566182_at	---	---	---	---	---	---	---	---	MmugDNA.13928.1.S1_at_---
MmugDNA.13929.1.S1_at	242025_at	---	---	---	---	---	---	---	---	MmugDNA.13929.1.S1_at_---
MmugDNA.13930.1.S1_at	219127_at	ATAD4	ATPase family, AAA domain containing 4	chr17q21.32	79170	ATAD4	ATPase family, AAA domain containing 4	---	695068	MmugDNA.13930.1.S1_at_ATAD4
MmugDNA.13931.1.S1_at	205740_s_at	RBM42	RNA binding motif protein 42	chr19q13.12	79171	LOC709852	similar to Temporarily Assigned Gene name family member (tag-262)	---	709852	MmugDNA.13931.1.S1_at_RBM42
MmugDNA.1393.1.S1_at	231690_at	---	---	---	---	---	---	---	---	MmugDNA.1393.1.S1_at_---
MmugDNA.13932.1.S1_at	233880_at	RNF213	ring finger protein 213	chr17q25.3	57674	---	---	---	---	MmugDNA.13932.1.S1_at_RNF213
MmugDNA.13933.1.S1_at	211597_s_at	HOPX	HOP homeobox	chr4q11-q12	84525	HOPX	HOP homeobox	---	694219	MmugDNA.13933.1.S1_at_HOPX
MmugDNA.13934.1.S1_at	230545_at	---	---	---	---	---	---	---	---	MmugDNA.13934.1.S1_at_---
MmugDNA.13935.1.S1_at	238345_at	SLC38A10	Solute carrier family 38, member 10	chr17q25.3	124565	---	---	---	---	MmugDNA.13935.1.S1_at_SLC38A10
MmugDNA.13936.1.S1_at	1570169_at	CSMD2	CUB and Sushi multiple domains 2	chr1p35.1-p34.3	114784	LOC710468	hypothetical protein LOC710468	---	710468	MmugDNA.13936.1.S1_at_CSMD2
MmugDNA.13937.1.S1_at	206901_at	C19orf57	chromosome 19 open reading frame 57	chr19p13.12	79173	LOC718466	hypothetical protein LOC718466	---	718466	MmugDNA.13937.1.S1_at_C19orf57
MmugDNA.13938.1.S1_at	218358_at	CRELD2	cysteine-rich with EGF-like domains 2	chr22q13.33	79174	---	---	---	---	MmugDNA.13938.1.S1_at_CRELD2
MmugDNA.13939.1.S1_s_at	201520_s_at	GRSF1	G-rich RNA sequence binding factor 1	chr4q13	2926	GRSF1	G-rich RNA sequence binding factor 1	---	706301	MmugDNA.13939.1.S1_s_at_GRSF1

MmugDNA.13940.1.S1_at	215825_at	---	---	---	---	---	---	---	---	MmugDNA.13940.1.S1_at_---
MmugDNA.13941.1.S1_at	231611_at	---	---	---	---	---	---	---	---	MmugDNA.13941.1.S1_at_---
MmugDNA.1394.1.S1_at	213454_at	APITD1	apoptosis-inducing, TAF9-like domain 1	chr1p36.22	378708	LOC722674	similar to axonal transport of synaptic vesicles	---	722674	MmugDNA.1394.1.S1_at_APITD1
MmugDNA.13942.1.S1_at	1555475_x_at	TTLL3	tubulin tyrosine ligase-like family, member 3	chr3p25.3	26140	---	---	---	---	MmugDNA.13942.1.S1_at_TTLL3
MmugDNA.13943.1.S1_at	1554403_a_at	---	---	---	---	---	---	---	---	MmugDNA.13943.1.S1_at_---
MmugDNA.13944.1.S1_at	239500_at	EFCAB1	EF-hand calcium binding domain 1	chr8q11.21	79645	---	---	---	---	MmugDNA.13944.1.S1_at_EFCAB1
MmugDNA.13945.1.S1_at	1552524_at	ART5	ADP-ribosyltransferase 5	chr11p15.4	116969	---	---	---	---	MmugDNA.13945.1.S1_at_ART5
MmugDNA.13946.1.S1_at	1553077_at	SDR9C7	short chain dehydrogenase/reductase family 9C, member 7	chr12q13.3	121214	---	---	---	---	MmugDNA.13946.1.S1_at_SDR9C7
MmugDNA.13947.1.S1_at	1564836_at	---	---	---	---	---	---	---	---	MmugDNA.13947.1.S1_at_---
MmugDNA.13948.1.S1_at	NA	NA	NA	NA	NA	NA	NA	NA	NA	MmugDNA.13948.1.S1_at_NA
MmugDNA.13949.1.S1_at	235001_at	DNAJC21	DnaJ (Hsp40) homolog, subfamily C, member 21	chr5p13.2	134218	LOC699118	similar to DnaJ homology subfamily A member 5 isoform 1	---	699118	MmugDNA.13949.1.S1_at_DNAJC21
MmugDNA.13950.1.S1_at	1558541_at	C8orf79	chromosome 8 open reading frame 79	chr8p22	57604	---	---	---	---	MmugDNA.13950.1.S1_at_C8orf79
MmugDNA.13951.1.S1_at	1568791_s_at	---	---	---	---	---	---	---	---	MmugDNA.13951.1.S1_at_---
MmugDNA.1395.1.S1_at	224540_at	LACRT	lacritin	chr12q13	90070	LACRT	lacritin	---	654418	MmugDNA.1395.1.S1_at_LACRT
MmugDNA.13952.1.S1_at	207296_at	ZNF343	zinc finger protein 343	chr20p13	79175	LOC716613	similar to zinc finger protein 343	---	716613	MmugDNA.13952.1.S1_at_ZNF343
MmugDNA.13953.1.S1_at	231713_s_at	ELP2	elongation protein 2 homolog (S. cerevisiae)	chr18q12.2	55250	LOC709483	similar to elongator protein 2	---	709483	MmugDNA.13953.1.S1_at_ELP2
MmugDNA.13954.1.S1_at	218938_at	FBXL15	F-box and leucine-rich repeat protein 15	chr10q24.32	79176	LOC713188	similar to F-box and leucine-rich repeat protein 15	---	713188	MmugDNA.13954.1.S1_at_FBXL15
MmugDNA.13955.1.S1_at	207842_s_at	CASC3	cancer susceptibility candidate 3	chr17q11-q21.3	22794	LOC716899	Similar to ribosomal protein S6 kinase, 90kDa, polypeptide 2 isoform b	---	716899	MmugDNA.13955.1.S1_at_CASC3
MmugDNA.13956.1.S1_at	1560295_at	CYP19A1	Cytochrome P450, family 19, subfamily A, polypeptide 1	chr15q21.1	1588	---	---	---	---	MmugDNA.13956.1.S1_at_CYP19A1
MmugDNA.13957.1.S1_at	219089_s_at	ZNF576	zinc finger protein 576	chr19q13.31	79177	ZXDB	Zinc finger, X-linked, duplicated B	---	706216	MmugDNA.13957.1.S1_at_ZNF576
MmugDNA.13958.1.S1_at	214936_at	LRCH1	Leucine-rich repeats and calponin homology (CH) domain containing 1	chr13q14.13-q14.2	23143	---	---	---	---	MmugDNA.13958.1.S1_at_LRCH1
MmugDNA.13959.1.S1_at	240054_at	---	---	---	---	---	---	---	---	MmugDNA.13959.1.S1_at_---
MmugDNA.13960.1.S1_at	235199_at	RNF125	ring finger protein 125	chr18q12.1	54941	---	---	---	---	MmugDNA.13960.1.S1_at_RNF125
MmugDNA.13961.1.S1_at	1557757_at	---	---	---	---	---	---	---	---	MmugDNA.13961.1.S1_at_---
MmugDNA.1396.1.S1_at	231951_at	GNAO1	guanine nucleotide binding protein (G protein), alpha activating activity polypeptide O	chr16q13	2775	---	---	---	---	MmugDNA.1396.1.S1_at_GNAO1
MmugDNA.13962.1.S1_at	235435_at	AASDH	aminoadipate-semialdehyde dehydrogenase	chr4q12	132949	---	---	---	---	MmugDNA.13962.1.S1_at_AASDH
MmugDNA.13963.1.S1_at	238142_at	---	---	---	---	---	---	---	---	MmugDNA.13963.1.S1_at_---
MmugDNA.13964.1.S1_at	234989_at	NCRNA00084	non-protein coding RNA 84	chr11q13.1	283131	---	---	---	---	MmugDNA.13964.1.S1_at_NCRNA0008
MmugDNA.13965.1.S1_at	239795_at	---	---	---	---	---	---	---	---	MmugDNA.13965.1.S1_at_---
MmugDNA.13966.1.S1_s_at	209324_s_at	RGS16	regulator of G-protein signaling 16	chr1q25-q31	6004	RGS8	regulator of G-protein signaling 8	---	715599	MmugDNA.13966.1.S1_s_at_RGS16
MmugDNA.13967.1.S1_at	232277_at	---	---	---	---	---	---	---	---	MmugDNA.13967.1.S1_at_---
MmugDNA.13968.1.S1_at	228568_at	GCOM1	GRINL1A complex locus	chr15q21.3	145781	GCOM1	GRINL1A complex locus	---	700247	MmugDNA.13968.1.S1_at_GCOM1
MmugDNA.13969.1.S1_at	236741_at	WDR72	WD repeat domain 72	chr15q21.3	256764	WDR72	WD repeat domain 72	---	697128	MmugDNA.13969.1.S1_at_WDR72
MmugDNA.13970.1.S1_at	231917_at	GFM2	G elongation factor, mitochondrial 2	chr5q13	84340	GFM2	G elongation factor, mitochondrial 2	---	704247	MmugDNA.13970.1.S1_at_GFM2
MmugDNA.13971.1.S1_at	1560405_at	---	---	---	---	---	---	---	---	MmugDNA.13971.1.S1_at_---
MmugDNA.1397.1.S1_at	217342_x_at	FLJ11292	hypothetical protein FLJ11292	chr5q14.3	55338	---	---	---	---	MmugDNA.1397.1.S1_at_FLJ11292
MmugDNA.13972.1.S1_at	243330_at	---	---	---	---	---	---	---	---	MmugDNA.13972.1.S1_at_---
MmugDNA.13973.1.S1_at	61297_at	CASKIN2	CASK interacting protein 2	chr17q25.1	57513	CASKIN2	CASK interacting protein 2	---	702477	MmugDNA.13973.1.S1_at_CASKIN2
MmugDNA.13975.1.S1_at	1569741_at	---	---	---	---	---	---	---	---	MmugDNA.13975.1.S1_at_---
MmugDNA.13976.1.S1_at	1555884_at	PSMD6	Proteasome (prosome, macropain) 26S subunit, non-ATPase, 6	chr3p14.1	9861	PSMD6	proteasome (prosome, macropain) 26S subunit, non-ATPase, 6	---	698601	MmugDNA.13976.1.S1_at_PSMD6
MmugDNA.13977.1.S1_at	1562477_at	EBF2	early B-cell factor 2	chr8p21.2	64641	---	---	---	---	MmugDNA.13977.1.S1_at_EBF2
MmugDNA.13978.1.S1_at	227117_at	---	---	---	---	---	---	---	---	MmugDNA.13978.1.S1_at_---
MmugDNA.13979.1.S1_at	219633_at	TTPAL	tocopherol (alpha) transfer protein-like	chr20q13.12	79183	LOC712620	similar to Protein C20orf121	---	712620	MmugDNA.13979.1.S1_at_TTPAL
MmugDNA.13980.1.S1_at	215716_s_at	ATP2B1	ATPase, Ca++ transporting, plasma membrane 1	chr12q21.3	490	LOC709102	similar to plasma membrane calcium ATPase 1 isoform 1b	---	709102	MmugDNA.13980.1.S1_at_ATP2B1
MmugDNA.13981.1.S1_at	1560448_at	LOC340037	hypothetical protein LOC340037	chr5q35.3	340037	---	---	---	---	MmugDNA.13981.1.S1_at_LOC340037
MmugDNA.1398.1.S1_at	238898_at	---	---	---	---	---	---	---	---	MmugDNA.1398.1.S1_at_---
MmugDNA.13982.1.S1_s_at	221196_x_at	BRCC3	BRCA1/BRCA2-containing complex, subunit 3	chrXq28	79184	BRCC3	BRCA1/BRCA2-containing complex, subunit 3	---	702888	MmugDNA.13982.1.S1_s_at_BRCC3
MmugDNA.13983.1.S1_at	219170_at	FSD1	fibronectin type III and SPRY domain containing 1	chr19p13.3	79187	LOC718931	similar to fibronectin type III and SPRY domain containing 1	---	718931	MmugDNA.13983.1.S1_at_FSD1
MmugDNA.13984.1.S1_at	217559_at	RPL10L	ribosomal protein L10-like	chr14q13-q21	140801	LOC715330	Similar to beta-site APP-cleaving enzyme 2 isoform C preproprotein	---	715330	MmugDNA.13984.1.S1_at_RPL10L
MmugDNA.13985.1.S1_s_at	218848_at	THOC6	THO complex 6 homolog (Drosophila)	chr16p13.3	79228	THOC6	THO complex 6 homolog (Drosophila)	---	700217	MmugDNA.13985.1.S1_s_at_THOC6
MmugDNA.13987.1.S1_at	237636_at	PPP2R2A	protein phosphatase 2 (formerly 2A), regulatory subunit B, alpha isoform	chr8p21.2	5520	---	---	---	---	MmugDNA.13987.1.S1_at_PPP2R2A
MmugDNA.13988.1.S1_at	1556763_at	---	---	---	---	---	---	---	---	MmugDNA.13988.1.S1_at_---
MmugDNA.13989.1.S1_at	237243_at	---	---	---	---	---	---	---	---	MmugDNA.13989.1.S1_at_---
MmugDNA.13990.1.S1_at	217514_at	---	---	---	---	---	---	---	---	MmugDNA.13990.1.S1_at_---
MmugDNA.13991.1.S1_at	240062_at	FAM3C	family with sequence similarity 3, member C	chr7q31	10447	LOC694209	similar to Protein FAM3C precursor (Protein GS3786)	---	694209	MmugDNA.13991.1.S1_at_FAM3C
MmugDNA.1399.1.S1_at	242795_at	---	---	---	---	---	---	---	---	MmugDNA.1399.1.S1_at_---
MmugDNA.13992.1.S1_at	235096_at	LEO1	Leo1, Paf1/RNA polymerase II complex component, homolog (S. cerevisiae)	chr15q21.2	123169	LEO1	Leo1, Paf1/RNA polymerase II complex component, homolog	---	694874	MmugDNA.13992.1.S1_at_LEO1
MmugDNA.13993.1.S1_at	228257_at	ANKRD52	ankyrin repeat domain 52	chr12q13.2	283373	---	---	---	---	MmugDNA.13993.1.S1_at_ANKRD52
MmugDNA.13994.1.S1_at	1559432_at	CCBP2	chemokine binding protein 2	chr3p21.3	1238	---	---	---	---	MmugDNA.13994.1.S1_at_CCBP2
MmugDNA.13995.1.S1_at	215039_at	HS2ST1 /// LOC339524	heparan sulfate 2-O-sulfotransferase 1 /// hypothetical LOC339524	chr1p22.3 /// chr1p31.1-p22.1	339524 /// 9653	---	---	---	---	MmugDNA.13995.1.S1_at_HS2ST1 /// L
MmugDNA.13996.1.S1_at	216496_s_at	---	---	---	---	---	---	---	---	MmugDNA.13996.1.S1_at_---
MmugDNA.13997.1.S1_at	NA	NA	NA	NA	NA	NA	NA	NA	NA	MmugDNA.13997.1.S1_at_NA
MmugDNA.13997.1.S1_s_at	220292_at	ZNF434	zinc finger protein 434	chr16p13.3	54925	ZNF434	zinc finger protein 434	---	702733	MmugDNA.13997.1.S1_s_at_ZNF434
MmugDNA.13998.1.S1_at	230442_at	MTHFSD	methenyltetrahydrofolate synthetase domain containing	chr16q24.1	64779	---	---	---	---	MmugDNA.13998.1.S1_at_MTHFSD
MmugDNA.13999.1.S1_at	220444_at	ZNF557	zinc finger protein 557	chr19p13.2	79230	---	---	---	---	MmugDNA.13999.1.S1_at_ZNF557
MmugDNA.14000.1.S1_at	219731_at	---	---	---	---	---	---	---	---	MmugDNA.14000.1.S1_at_---
MmugDNA.14001.1.S1_at	NA	NA	NA	NA	NA	NA	NA	NA	NA	MmugDNA.14001.1.S1_at_NA
MmugDNA.1400.1.S1_at	1564107_at	---	---	---	---	---	---	---	---	MmugDNA.1400.1.S1_at_---
MmugDNA.14002.1.S1_at	219001_s_at	WDR32	WD repeat domain 32	chr9p13.2	79269	LOC716256	similar to WD repeat domain 32	---	716256	MmugDNA.14002.1.S1_at_WDR32
MmugDNA.14003.1.S1_at	228971_at	---	---	---	---	---	---	---	---	MmugDNA.14003.1.S1_at_---
MmugDNA.14004.1.S1_at	243386_at	CASZ1	castor zinc finger 1	chr1p36.22	54897	---	---	---	---	MmugDNA.14004.1.S1_at_CASZ1
MmugDNA.14005.1.S1_at	215796_at	---	---	---	---	LOC710276	similar to T-cell receptor alpha chain V region CTL-L17 precursor	---	710276	MmugDNA.14005.1.S1_at_---
MmugDNA.14006.1.S1_at	238773_at	METT5D1	methyltransferase 5 domain containing 1	chr11p14.1	196074	LOC698208	similar to methyltransferase 5 domain containing 1	---	698208	MmugDNA.14006.1.S1_at_METT5D1
MmugDNA.14007.1.S1_at	243423_at	TNIP1	TNFAIP3 interacting protein 1	chr5q32-q33.1	10318	---	---	---	---	MmugDNA.14007.1.S1_at_TNIP1
MmugDNA.14008.1.S1_s_at	236227_at	TMEM161B	transmembrane protein 161B	chr5q14.3	153396	TMEM161B	transmembrane protein 161B	---	694351	MmugDNA.14008.1.S1_s_at_TMEM161
MmugDNA.14009.1.S1_s_at	203476_at	TPBG	trophoblast glycoprotein	chr6q14-q15	7162	LOC693944	similar to 5T4 oncofetal trophoblast glycoprotein	---	693944	MmugDNA.14009.1.S1_s_at_TPBG
MmugDNA.14010.1.S1_at	1553356_at	---	---	---	---	---	---	---	---	MmugDNA.14010.1.S1_at_---
MmugDNA.14011.1.S1_s_at	230243_at	RG9MTD2	RNA (guanine-9-) methyltransferase domain containing 2	chr4q23	93587	---	---	---	---	MmugDNA.14011.1.S1_s_at_RG9MTD2
MmugDNA.1401.1.S1_at	1557051_s_at	---	---	---	---	---	---	---	---	MmugDNA.1401.1.S1_at_---
MmugDNA.14012.1.S1_at	243693_at	---	---	---	---	---	---	---	---	MmugDNA.14012.1.S1_at_---
MmugDNA.14013.1.S1_at	227901_at	LOC648987	hypothetical LOC648987	chr5p12	648987	---	---	---	---	MmugDNA.14013.1.S1_at_LOC648987
MmugDNA.14014.1.S1_at	241132_at	---	---	---	---	---	---	---	---	MmugDNA.14014.1.S1_at_---
MmugDNA.14015.1.S1_at	1558647_at	SH3D19	SH3 domain containing 19	chr4q31.3	152503	---	---	---	---	MmugDNA.14015.1.S1_at_SH3D19
MmugDNA.14016.1.S1_at	238140_at	ARV1	ARV1 homolog (S. cerevisiae)	chr1q42.2	64801	---	---	---	---	MmugDNA.14016.1.S1_at_ARV1
MmugDNA.14017.1.S1_at	215041_s_at	DOCK9	dedicator of cytokinesis 9	chr13q32.3	23348	DOCK9	dedicator of cytokinesis 9	---	702426	MmugDNA.14017.1.S1_at_DOCK9
MmugDNA.14018.1.S1_at	237557_at	---	---	---	---	---	---	---	---	MmugDNA.14018.1.S1_at_---
MmugDNA.14019.1.S1_at	244435_at	C10orf141	chromosome 10 open reading frame 141	chr10q26.2	642938	LOC697021	hypothetical protein LOC697021	---	697021	MmugDNA.14019.1.S1_at_C10orf141
MmugDNA.140.1.S1_at	222535_at	INF2	inverted formin, FH2 and WH2 domain containing	chr14q32.33	64423	INF2	inverted formin, FH2 and WH2 domain containing	---	706242	MmugDNA.140.1.S1_at_INF2
MmugDNA.14020.1.S1_at	208978_at	CRIP2	cysteine-rich protein 2	chr14q32.3	1397	LOC710186	similar to Cysteine-rich protein 2 (CRP2) (Protein ESP1)	---	710186	MmugDNA.14020.1.S1_at_CRIP2
MmugDNA.14021.1.S1_at	244684_at	PGGT1B	Protein geranylgeranyltransferase type I, beta subunit	chr5q22.3	5229	---	---	---	---	MmugDNA.14021.1.S1_at_PGGT1B
MmugDNA.1402.1.S1_at	1569323_at	PTPRG	protein tyrosine phosphatase, receptor type, G	chr3p21-p14	5793	---	---	---	---	MmugDNA.1402.1.S1_at_PTPRG
MmugDNA.14022.1.S1_at	217007_s_at	ADAM15	ADAM metallopeptidase domain 15	chr1q21.3	8751	LOC719030	similar to ADAM 15 precursor (A disintegrin and metalloproteinase domain 15) (Metalloproteinase-like	---	719030	MmugDNA.14022.1.S1_at_ADAM15
MmugDNA.14023.1.S1_at	200850_s_at	AHCYL1	S-adenosylhomocysteine hydrolase-like 1	chr1p13.2	10768	LOC700681	similar to S-adenosylhomocysteine hydrolase-like 1	---	700681	MmugDNA.14023.1.S1_at_AHCYL1
MmugDNA.14024.1.S1_at	241974_at	---	---	---	---	---	---	---	---	MmugDNA.14024.1.S1_at_---
MmugDNA.14025.1.S1_at	222368_at	---	---	---	---	---	---	---	---	MmugDNA.14025.1.S1_at_---
MmugDNA.14026.1.S1_at	241779_at	MTX3	metaxin 3	chr5q14.1	345778	LOC714732	similar to metaxin 3	---	714732	MmugDNA.14026.1.S1_at_MTX3
MmugDNA.14027.1.S1_at	244766_at	LOC440354 /// LOC595	PI-3-kinase-related kinase SMG-1 pseudogene /// PI-3-kinase-related kinase SMG-1 pseudogene ///	chr16p11.2 /// chr16p12.1 /// chr1	23049 /// 440354 /// 59	LOC693542	similar to PI-3-kinase-related kinase SMG-1	---	693542	MmugDNA.14027.1.S1_at_LOC440354
MmugDNA.14028.1.S1_at	233659_at	---	---	---	---	---	---	---	---	MmugDNA.14028.1.S1_at_---
MmugDNA.14029.1.S1_at	207690_at	ALX3	ALX homeobox 3	chr1p13.3	257	ALX3	ALX homeobox 3	---	700927	MmugDNA.14029.1.S1_at_ALX3
MmugDNA.14030.1.S1_at	1562845_at	EP400NL	EP400 N-terminal like	chr12q24.33	347918	LOC719386	similar to E1A-binding protein p400 (p400 kDa SWI2/SNF2-related protein) (Domino homolog) (mDor	---	719386	MmugDNA.14030.1.S1_at_EP400NL
MmugDNA.14031.1.S1_at	201687_s_at	API5	apoptosis inhibitor 5	chr11p11.2	8539	LOC722311	Similar to type II transmembrane protein DCAL1	---	722311	MmugDNA.14031.1.S1_at_API5
MmugDNA.1403.1.S1_at	234342_at	---	---	---	---	---	---	---	---	MmugDNA.1403.1.S1_at_---
MmugDNA.14032.1.S1_at	1558965_at	PHF21A	PHD finger protein 21A	chr11p11.2	51317	---	---	---	---	MmugDNA.14032.1.S1_at_PHF21A
MmugDNA.14033.1.S1_at	1555095_at	C6orf123	chromosome 6 open reading frame 123	chr6q27	26238	---	---	---	---	MmugDNA.14033.1.S1_at_C6orf123
MmugDNA.14034.1.S1_at	1561415_at	---	---	---	---	---	---	---	---	MmugDNA.14034.1.S1_at_---
MmugDNA.14035.1.S1_at	238991_at	---	---	---	---	ASB1	ankyrin repeat and SOCS box-containing 1	---	696716	MmugDNA.14035.1.S1_at_---
MmugDNA.14036.1.S1_at	236944_at	---	---	---	---	---	---	---	---	MmugDNA.14036.1.S1_at_---
MmugDNA.14037.1.S1_at	237765_at	C14orf68	chromosome 14 open reading frame 68	chr14q32.2	283600	---	---	---	---	MmugDNA.14037.1.S1_at_C14orf68

MmugDNA.14038.1.S1_at	242641_at	---	---	---	---	---	---	---	---		MmugDNA.14038.1.S1_at_---
MmugDNA.14039.1.S1_at	240007_at	---	---	---	---	---	---	---	---		MmugDNA.14039.1.S1_at_---
MmugDNA.14040.1.S1_at	240476_at	---	---	---	---	---	---	---	---		MmugDNA.14040.1.S1_at_---
MmugDNA.14041.1.S1_at	226877_at	RPL32P3	ribosomal protein L32 pseudogene 3	chr3q21.3	132241	---	---	---	---		MmugDNA.14041.1.S1_at_RPL32P3
MmugDNA.1404.1.S1_at	223359_s_at	PDE7A	phosphodiesterase 7A	chr8q13	5150	---	---	---	---		MmugDNA.1404.1.S1_at_PDE7A
MmugDNA.14042.1.S1_at	211502_s_at	PFTK1	PFTAIRE protein kinase 1	chr7q21-q22	5218	PFTK1	PFTAIRE protein kinase 1	---		703442	MmugDNA.14042.1.S1_at_PFTK1
MmugDNA.14043.1.S1_at	238040_at	---	---	---	---	---	---	---	---		MmugDNA.14043.1.S1_at_---
MmugDNA.14044.1.S1_at	1552347_at	CRYZL1	crystallin, zeta (quinone reductase)-like 1	chr21q21.3	9946	---	---	---	---		MmugDNA.14044.1.S1_at_CRYZL1
MmugDNA.14045.1.S1_at	205539_at	AVIL	advillin	chr12q14.1	10677	LOC712581	similar to advillin	---		712581	MmugDNA.14045.1.S1_at_AVIL
MmugDNA.14046.1.A1_at	NA	NA	NA	NA	NA	NA	NA	NA	NA		MmugDNA.14046.1.A1_at_NA
MmugDNA.14046.1.S1_at	1566039_a_at	LOC401220 /// LOC401	hypothetical gene supported by BC036933 /// hypothetical gene supported by BC036933	chr5q35.3	401220 /// 401222	---	---	---	---		MmugDNA.14046.1.S1_at_LOC401220
MmugDNA.14047.1.S1_at	228607_at	OAS2	2'-5'-oligoadenylate synthetase 2, 69/71kDa	chr12q24.2	4939	---	---	---	---		MmugDNA.14047.1.S1_at_OAS2
MmugDNA.14048.1.S1_at	201032_at	BLCAP	bladder cancer associated protein	chr20q11.2-q12	10904	LOC717399	Similar to ephrin A1 isoform a precursor	---		717399	MmugDNA.14048.1.S1_at_BLCAP
MmugDNA.14049.1.S1_at	242260_at	MATR3	Matrin 3	chr5q31.2	9782	---	---	---	---		MmugDNA.14049.1.S1_at_MATR3
MmugDNA.14050.1.S1_at	217265_at	PLLP	plasma membrane proteolipid (plasmolipin)	chr16q13	51090	PLLP	similar to Plasmolipin (Plasma membrane proteolipid)	---		709914	MmugDNA.14050.1.S1_at_PLLP
MmugDNA.14051.1.S1_at	208536_s_at	BCL2L11	BCL2-like 11 (apoptosis facilitator)	chr2q13	10018	BCL2L11	BCL2-like 11 (apoptosis facilitator)	---		697082	MmugDNA.14051.1.S1_at_BCL2L11
MmugDNA.1405.1.S1_at	1569791_at	STK4	serine/threonine kinase 4	chr20q11.2-q13.2	6789	---	---	---	---		MmugDNA.1405.1.S1_at_STK4
MmugDNA.14052.1.S1_at	210342_s_at	TPO	thyroid peroxidase	chr2p25	7173	---	---	---	---		MmugDNA.14052.1.S1_at_TPO
MmugDNA.14052.1.S1_s_at	NA	NA	NA	NA	NA	NA	NA	NA	NA		MmugDNA.14052.1.S1_s_at_NA
MmugDNA.14053.1.S1_at	235543_at	---	---	---	---	---	---	---	---		MmugDNA.14053.1.S1_at_---
MmugDNA.14054.1.S1_at	217982_s_at	MORF4L1	mortality factor 4 like 1	chr15q24	10933	LOC711357	similar to mortality factor 4 like 1 isoform b	---		711357	MmugDNA.14054.1.S1_at_MORF4L1
MmugDNA.14055.1.S1_at	235234_at	PATL1	protein associated with topoisomerase II homolog 1 (yeast)	chr11q12.1	219988	LOC699207	hypothetical protein LOC699207	---		699207	MmugDNA.14055.1.S1_at_PATL1
MmugDNA.14056.1.S1_at	1559548_at	ACVR2B	activin A receptor, type IIB	chr3p22	93	---	---	---	---		MmugDNA.14056.1.S1_at_ACVR2B
MmugDNA.14057.1.S1_at	1553895_at	C11orf42	chromosome 11 open reading frame 42	chr11p15.4	160298	LOC711815	hypothetical protein LOC711815	---		711815	MmugDNA.14057.1.S1_at_C11orf42
MmugDNA.14058.1.S1_at	228394_at	STK10	serine/threonine kinase 10	chr5q35.1	6793	---	---	---	---		MmugDNA.14058.1.S1_at_STK10
MmugDNA.14059.1.S1_at	207551_s_at	MSL3	male-specific lethal 3 homolog (Drosophila)	chrXp22.3	10943	MSL3	male-specific lethal 3 homolog (Drosophila)	---		720836	MmugDNA.14059.1.S1_at_MSL3
MmugDNA.14060.1.S1_at	232784_at	---	---	---	---	---	---	---	---		MmugDNA.14060.1.S1_at_---
MmugDNA.14061.1.S1_at	206948_at	NEU3	sialidase 3 (membrane sialidase)	chr11q13.5	10825	---	---	---	---		MmugDNA.14061.1.S1_at_NEU3
MmugDNA.1406.1.S1_at	238418_at	SLC35B4	solute carrier family 35, member B4	chr7q33	84912	LOC717185	Similar to Y45F3A.9	---		717185	MmugDNA.1406.1.S1_at_SLC35B4
MmugDNA.14063.1.S1_at	1557830_at	---	---	---	---	---	---	---	---		MmugDNA.14063.1.S1_at_---
MmugDNA.14064.1.S1_at	215248_at	GRB10	growth factor receptor-bound protein 10	chr7p12-p11.2	2887	---	---	---	---		MmugDNA.14064.1.S1_at_GRB10
MmugDNA.14065.1.S1_at	239318_at	FAM118B	family with sequence similarity 118, member B	chr11q24.2	79607	LOC714323	hypothetical protein LOC714323	---		714323	MmugDNA.14065.1.S1_at_FAM118B
MmugDNA.14066.1.S1_at	1558630_at	---	---	---	---	---	---	---	---		MmugDNA.14066.1.S1_at_---
MmugDNA.14067.1.S1_at	1552906_at	FMR1NB	fragile X mental retardation 1 neighbor	chrXq27.3-q28	158521	FMR1NB	similar to fragile X mental retardation 1 neighbor	---		704191	MmugDNA.14067.1.S1_at_FMR1NB
MmugDNA.14068.1.S1_at	45297_at	EHD2	EH-domain containing 2	chr19q13.3	30846	LOC711890	Similar to ribosomal protein L31	---		711890	MmugDNA.14068.1.S1_at_EHD2
MmugDNA.14069.1.S1_at	211550_at	EGFR	epidermal growth factor receptor (erythroblastic leukemia viral (v-erb-b) oncogene homolog, avian)	chr7p12	1956	---	---	---	---		MmugDNA.14069.1.S1_at_EGFR
MmugDNA.14070.1.S1_at	1559583_at	CD276	CD276 molecule	chr15q23-q24	80381	---	---	---	---		MmugDNA.14070.1.S1_at_CD276
MmugDNA.14071.1.S1_at	240120_at	---	---	---	---	---	---	---	---		MmugDNA.14071.1.S1_at_---
MmugDNA.1407.1.S1_at	233519_at	---	---	---	---	---	---	---	---		MmugDNA.1407.1.S1_at_---
MmugDNA.14072.1.S1_at	233603_at	---	---	---	---	---	---	---	---		MmugDNA.14072.1.S1_at_---
MmugDNA.14073.1.S1_at	228943_at	MAP6	microtubule-associated protein 6	chr11q13.5	4135	---	---	---	---		MmugDNA.14073.1.S1_at_MAP6
MmugDNA.14074.1.S1_at	241812_at	LOC26010	viral DNA polymerase-transactivated protein 6	chr2q33.1	26010	---	---	---	---		MmugDNA.14074.1.S1_at_LOC26010
MmugDNA.14075.1.S1_at	203755_at	BUB1B	budding uninhibited by benzimidazoles 1 homolog beta (yeast)	chr15q15	701	CLIP1	CAP-GLY domain containing linker protein 1	---		702137	MmugDNA.14075.1.S1_at_BUB1B
MmugDNA.14076.1.S1_at	NA	NA	NA	NA	NA	NA	NA	NA	NA		MmugDNA.14076.1.S1_at_NA
MmugDNA.14076.1.S1_s_at	235476_at	TRIM59	tripartite motif-containing 59	chr3q26.1	286827	LOC704829	similar to tripartite motif-containing 59	---		704829	MmugDNA.14076.1.S1_s_at_TRIM59
MmugDNA.14077.1.S1_at	1563327_a_at	CXorf31	chromosome X open reading frame 31	chrXp11.3	724087	---	---	---	---		MmugDNA.14077.1.S1_at_CXorf31
MmugDNA.14078.1.S1_at	201323_at	EBNA1BP2	EBNA1 binding protein 2	chr1p35-p33	10969	EBNA1BP2	EBNA1 binding protein 2	---		699672	MmugDNA.14078.1.S1_at_EBNA1BP2
MmugDNA.14079.1.S1_at	1558813_at	TMED5	transmembrane emp24 protein transport domain containing 5	chr1pter-q31.3	50999	---	---	---	---		MmugDNA.14079.1.S1_at_TMED5
MmugDNA.14080.1.S1_at	206691_s_at	PDIA2	protein disulfide isomerase family A, member 2	chr16p13.3	64714	PDIA2	protein disulfide isomerase family A, member 2	---		694964	MmugDNA.14080.1.S1_at_PDIA2
MmugDNA.14081.1.S1_at	221970_s_at	NOL11	nucleolar protein 11	chr17q24.2	25926	LOC718803	similar to nucleolar protein 11	---		718803	MmugDNA.14081.1.S1_at_NOL11
MmugDNA.1408.1.S1_at	230787_at	---	---	---	---	LOC716709	hypothetical protein LOC716709	---		716709	MmugDNA.1408.1.S1_at_---
MmugDNA.14082.1.S1_at	215327_at	---	---	---	---	---	---	---	---		MmugDNA.14082.1.S1_at_---
MmugDNA.14084.1.S1_at	234314_at	C20orf74	chromosome 20 open reading frame 74	chr20p11.22	57186	LOC708383	Similar to ankyrin repeat domain 13	---		708383	MmugDNA.14084.1.S1_at_C20orf74
MmugDNA.14085.1.S1_at	217043_s_at	MFN1	mitofusin 1	chr3q26.32	55669	LOC704692	similar to mitofusin 1	---		704692	MmugDNA.14085.1.S1_at_MFN1
MmugDNA.14086.1.S1_at	232383_at	TFEC	transcription factor EC	chr7q31.2	22797	TFEC	transcription factor EC	---		704124	MmugDNA.14086.1.S1_at_TFEC
MmugDNA.14087.1.S1_at	1554566_at	KCTD17	potassium channel tetramerisation domain containing 17	chr22q12.3	79734	KCTD17	potassium channel tetramerisation domain containing 17	---		696210	MmugDNA.14087.1.S1_at_KCTD17
MmugDNA.14088.1.S1_at	46323_at	CANT1	calcium activated nucleotidase 1	chr17q25.3	124583	LOC710935	similar to calcium activated nucleotidase 1	---		710935	MmugDNA.14088.1.S1_at_CANT1
MmugDNA.14089.1.S1_at	205804_s_at	TRAF3IP3	TRAF3 interacting protein 3	chr1q32.3-q41	80342	LOC712895	similar to TRAF3-interacting JNK-activating modulator	---		712895	MmugDNA.14089.1.S1_at_TRAF3IP3
MmugDNA.14090.1.S1_at	218608_at	ATP13A2	ATPase type 13A2	chr1p36	23400	ATP13A2	ATPase type 13A2	---		699678	MmugDNA.14090.1.S1_at_ATP13A2
MmugDNA.1409.1.S1_at	237797_at	DNM1L	Dynamin 1-like	chr12p11.21	10059	---	---	---	---		MmugDNA.1409.1.S1_at_DNM1L
MmugDNA.14092.1.S1_s_at	213836_s_at	WIPI1	WD repeat domain, phosphoinositide interacting 1	chr17q24.2	55062	---	---	---	---		MmugDNA.14092.1.S1_s_at_WIPI1
MmugDNA.14093.1.S1_at	202396_at	TCERG1	transcription elongation regulator 1	chr5q31	10915	---	---	---	---		MmugDNA.14093.1.S1_at_TCERG1
MmugDNA.14094.1.S1_at	240996_at	---	---	---	---	---	---	---	---		MmugDNA.14094.1.S1_at_---
MmugDNA.14095.1.S1_at	208063_s_at	CAPN9	calpain 9	chr1q42.11-q42.3	10753	CAPN9	calpain 9	---		719222	MmugDNA.14095.1.S1_at_CAPN9
MmugDNA.14096.1.S1_at	232891_at	SIRPD	signal-regulatory protein delta	chr20p13	128646	---	---	---	---		MmugDNA.14096.1.S1_at_SIRPD
MmugDNA.14097.1.S1_at	232678_at	SLC2A9	Solute carrier family 2 (facilitated glucose transporter), member 9	chr4p16-p15.3	56606	---	---	---	---		MmugDNA.14097.1.S1_at_SLC2A9
MmugDNA.14098.1.S1_at	207467_x_at	CAST	calpastatin	chr5q15	831	LOC707577	Similar to ring finger protein 137	---		707577	MmugDNA.14098.1.S1_at_CAST
MmugDNA.14099.1.S1_at	203857_s_at	PDIA5	protein disulfide isomerase family A, member 5	chr3q21.1	10954	PDIA5	protein disulfide isomerase family A, member 5	---		715226	MmugDNA.14099.1.S1_at_PDIA5
MmugDNA.14100.1.S1_at	238834_at	MYLK3	myosin light chain kinase 3	chr16q11.2	91807	---	---	---	---		MmugDNA.14100.1.S1_at_MYLK3
MmugDNA.14101.1.S1_at	201053_s_at	PSMF1	proteasome (prosome, macropain) inhibitor subunit 1 (PI31)	chr20p13	9491	PSMF1	proteasome (prosome, macropain) inhibitor subunit 1 (PI31)	---		715314	MmugDNA.14101.1.S1_at_PSMF1
MmugDNA.14102.1.S1_at	233539_at	NAPEPLD	N-acyl phosphatidylethanolamine phospholipase D	chr7q22.1	222236	---	---	---	---		MmugDNA.14102.1.S1_at_NAPEPLD
MmugDNA.14103.1.S1_at	217536_x_at	---	---	---	---	---	---	---	---		MmugDNA.14103.1.S1_at_---
MmugDNA.14104.1.S1_at	205478_at	PPP1R1A	protein phosphatase 1, regulatory (inhibitor) subunit 1A	chr12q13.2	5502	LOC708100	Similar to CG7872-PA	---		708100	MmugDNA.14104.1.S1_at_PPP1R1A
MmugDNA.14105.1.S1_at	234827_at	---	---	---	---	---	---	---	---		MmugDNA.14105.1.S1_at_---
MmugDNA.14106.1.S1_at	NA	NA	NA	NA	NA	NA	NA	NA	NA		MmugDNA.14106.1.S1_at_NA
MmugDNA.14107.1.S1_at	209597_s_at	PNMA2	paraneoplastic antigen MA2	chr8p21.2	10687	LOC712224	similar to paraneoplastic antigen MA2	---		712224	MmugDNA.14107.1.S1_at_PNMA2
MmugDNA.14108.1.S1_at	1561455_at	LOC284294	hypothetical protein LOC284294	chr18q22.1	284294	---	---	---	---		MmugDNA.14108.1.S1_at_LOC284294
MmugDNA.14109.1.S1_at	219601_s_at	C10orf12	chromosome 10 open reading frame 12	chr10q24.1	26148	C9H10orf12	chromosome 10 open reading frame 12 ortholog	---		705247	MmugDNA.14109.1.S1_at_C10orf12
MmugDNA.14110.1.S1_at	218826_at	SLC35F2	solute carrier family 35, member F2	chr11q22.3	54733	LOC707332	similar to solute carrier family 35, member F2	---		707332	MmugDNA.14110.1.S1_at_SLC35F2
MmugDNA.14111.1.S1_at	1564093_at	NEK1	NIMA (never in mitosis gene a)-related kinase 1	chr4q33	4750	---	---	---	---		MmugDNA.14111.1.S1_at_NEK1
MmugDNA.1411.1.S1_at	205491_s_at	GJB3	gap junction protein, beta 3, 31kDa	chr1p34	2707	LOC710834	similar to connexin 31	---		710834	MmugDNA.1411.1.S1_at_GJB3
MmugDNA.14112.1.S1_at	1555900_at	---	---	---	---	---	---	---	---		MmugDNA.14112.1.S1_at_---
MmugDNA.14113.1.S1_at	1557305_at	TACC1	Transforming, acidic coiled-coil containing protein 1	chr8p11.23	6867	TACC1	transforming, acidic coiled-coil containing protein 1	---		706243	MmugDNA.14113.1.S1_at_TACC1
MmugDNA.14114.1.S1_at	1566863_at	---	---	---	---	---	---	---	---		MmugDNA.14114.1.S1_at_---
MmugDNA.14115.1.S1_at	1561668_at	---	---	---	---	---	---	---	---		MmugDNA.14115.1.S1_at_---
MmugDNA.14116.1.S1_at	235135_at	TAF10	TAF10 RNA polymerase II, TATA box binding protein (TBP)-associated factor, 30kDa	chr11p15.3	6881	LOC710022	similar to Transcription initiation factor TFIID subunit 10 (Transcription initiation factor TFIID 30 kDa s	---		710022	MmugDNA.14116.1.S1_at_TAF10
MmugDNA.14117.1.S1_at	1557382_x_at	AGAP11	ankyrin repeat and GTPase domain Arf GTPase activating protein 11	chr10q23.2	119385	LOC697912	similar to centaurin, gamma-like family, member 1	---		697912	MmugDNA.14117.1.S1_at_AGAP11
MmugDNA.14118.1.S1_at	206587_at	CCT6B	chaperonin containing TCP1, subunit 6B (zeta 2)	chr17q12	10693	EEF1G	Similar to Elongation factor 1-gamma (EF-1-gamma) (eEF-1B gamma)	---		696871	MmugDNA.14118.1.S1_at_CCT6B
MmugDNA.14119.1.S1_at	1561983_at	---	---	---	---	---	---	---	---		MmugDNA.14119.1.S1_at_---
MmugDNA.141.1.S1_at	1561534_at	---	---	---	---	---	---	---	---		MmugDNA.141.1.S1_at_---
MmugDNA.14120.1.S1_at	222562_s_at	TNKS2	tankyrase, TRF1-interacting ankyrin-related ADP-ribose polymerase 2	chr10q23.3	80351	LOC698050	hypothetical protein LOC698050	---		698050	MmugDNA.14120.1.S1_at_TNKS2
MmugDNA.14121.1.S1_at	205937_at	CGREF1	cell growth regulator with EF-hand domain 1	chr2p23.3	10669	CGREF1	similar to Cell growth regulator with EF hand domain 1 (Cell growth regulatory gene 11 protein)	---		698449	MmugDNA.14121.1.S1_at_CGREF1
MmugDNA.1412.1.S1_at	232990_at	FAM104B	family with sequence similarity 104, member B	chrXp11.21	90736	---	---	---	---		MmugDNA.1412.1.S1_at_FAM104B
MmugDNA.14122.1.S1_at	234687_x_at	LIMD1	LIM domains containing 1	chr3p21.3	8994	LIMD1	LIM domains containing 1	---		714169	MmugDNA.14122.1.S1_at_LIMD1
MmugDNA.14123.1.S1_at	226839_at	NR2C2AP	nuclear receptor 2C2-associated protein	chr19p13.11	126382	LOC719729	similar to TR4 orphan receptor associated protein TRA16	---		719729	MmugDNA.14123.1.S1_at_NR2C2AP
MmugDNA.14124.1.S1_at	204605_at	CGRRF1	cell growth regulator with ring finger domain 1	chr14q22.2	10668	CGRRF1	cell growth regulator with ring finger domain 1	---		695231	MmugDNA.14124.1.S1_at_CGRRF1
MmugDNA.14125.1.S1_at	230973_at	SH2D5	SH2 domain containing 5	chr1p36.12	400745	LOC717509	hypothetical protein LOC717509	---		717509	MmugDNA.14125.1.S1_at_SH2D5
MmugDNA.14126.1.S1_at	222902_s_at	DEM1	defects in morphology 1 homolog (S. cerevisiae)	chr1p34.2	64789	LOC694536	hypothetical protein LOC694536	---		694536	MmugDNA.14126.1.S1_at_DEM1
MmugDNA.14127.1.S1_at	241477_at	NEIL1	nei endonuclease VIII-like 1 (E. coli)	chr15q24.2	79661	---	---	---	---		MmugDNA.14127.1.S1_at_NEIL1
MmugDNA.14128.1.S1_at	204591_at	CHL1	cell adhesion molecule with homology to L1CAM (close homolog of L1)	chr3p26.1	10752	---	---	---	---		MmugDNA.14128.1.S1_at_CHL1
MmugDNA.14129.1.S1_at	200085_s_at	TCEB2	transcription elongation factor B (SIII), polypeptide 2 (18kDa, elongin B)	chr16p12.3	6923	LOC713721	similar to Transcription elongation factor B polypeptide 2 (RNA polymerase II transcription factor SIII s	---		713721	MmugDNA.14129.1.S1_at_TCEB2
MmugDNA.14129.1.S1_x_at	NA	NA	NA	NA	NA	NA	NA	NA	NA		MmugDNA.14129.1.S1_x_at_NA
MmugDNA.14130.1.S1_at	217763_s_at	RAB31	RAB31, member RAS oncogene family	chr18p11.3	11031	RAB31	RAB31, member RAS oncogene family	---		704975	MmugDNA.14130.1.S1_at_RAB31
MmugDNA.14131.1.S1_at	222813_at	ZNF668	zinc finger protein 668	chr16p11.2	79759	LOC712709	similar to zinc finger protein 668	---		712709	MmugDNA.14131.1.S1_at_ZNF668
MmugDNA.1413.1.S1_at	NA	NA	NA	NA	NA	NA	NA	NA	NA		MmugDNA.1413.1.S1_at_NA
MmugDNA.14132.1.S1_at	204214_s_at	RAB32	RAB32, member RAS oncogene family	chr6q24.3	10981	LOC702431	similar to Ras-related protein Rab-32	---		702431	MmugDNA.14132.1.S1_at_RAB32
MmugDNA.14133.1.S1_at	205461_at	RAB35	RAB35, member RAS oncogene family	chr12q24.31	11021	LOC698400	hypothetical protein LOC698400	---		698400	MmugDNA.14133.1.S1_at_RAB35
MmugDNA.14134.1.S1_at	NA	NA	NA	NA	NA	NA	NA	NA	NA		MmugDNA.14134.1.S1_at_NA

MmugDNA.14135.1.S1_at	236346_at	---	---	---	---	---	---	---	---	MmugDNA.14135.1.S1_at_---
MmugDNA.14136.1.S1_at	240015_at	---	---	---	---	---	---	---	---	MmugDNA.14136.1.S1_at_---
MmugDNA.14137.1.S1_at	NA	NA	NA	NA	NA	NA	NA	NA	NA	MmugDNA.14137.1.S1_at_NA
MmugDNA.14138.1.S1_at	241139_at	---	---	---	---	---	---	---	---	MmugDNA.14138.1.S1_at_---
MmugDNA.14139.1.S1_at	219189_at	FBXL6	F-box and leucine-rich repeat protein 6	chr8q24.3	26233	FBXL6	F-box and leucine-rich repeat protein 6	---	701594	MmugDNA.14139.1.S1_at_FBXL6
MmugDNA.14140.1.S1_at	239158_at	---	---	---	---	---	---	---	---	MmugDNA.14140.1.S1_at_---
MmugDNA.14141.1.S1_at	219828_at	C9orf86	chromosome 9 open reading frame 86	chr9q34.3	55684	---	---	---	---	MmugDNA.14141.1.S1_at_C9orf86
MmugDNA.1414.1.S1_at	1557119_a_at	ZNF575	zinc finger protein 575	chr19q13.31	284346	LOC718118	similar to zinc finger protein 575	---	718118	MmugDNA.1414.1.S1_at_ZNF575
MmugDNA.14142.1.S1_at	216451_at	STK38	Serine/threonine kinase 38	chr6p21	11329	STK38	serine/threonine kinase 38	---	719169	MmugDNA.14142.1.S1_at_STK38
MmugDNA.14143.1.S1_at	233323_at	---	---	---	---	---	---	---	---	MmugDNA.14143.1.S1_at_---
MmugDNA.14144.1.S1_s_at	1553188_s_at	PARD3B	par-3 partitioning defective 3 homolog B (C. elegans)	chr2q33.3	117583	LOC707607	hypothetical protein LOC707607	---	707607	MmugDNA.14144.1.S1_s_at_PARD3B
MmugDNA.14145.1.S1_s_at	204577_s_at	CLUAP1	clusterin associated protein 1	chr16p13.3	23059	CLUAP1	clusterin associated protein 1	---	703676	MmugDNA.14145.1.S1_s_at_CLUAP1
MmugDNA.14147.1.S1_at	1553190_s_at	PARD3B	par-3 partitioning defective 3 homolog B (C. elegans)	chr2q33.3	117583	PARD3B	par-3 partitioning defective 3 homolog B (C. elegans)	---	707941	MmugDNA.14147.1.S1_at_PARD3B
MmugDNA.14148.1.S1_at	220159_at	ABCA11P	ATP-binding cassette, sub-family A (ABC1), member 11 (pseudogene)	chr4p16.3	79963	LOC714206	similar to ATP-binding cassette, sub-family A, member 10	---	714206	MmugDNA.14148.1.S1_at_ABCA11P
MmugDNA.14149.1.S1_at	219395_at	ESRP2	epithelial splicing regulatory protein 2	chr16q22.1	80004	LOC702287	similar to fusilli CG8205-PD, isoform D	---	702287	MmugDNA.14149.1.S1_at_ESRP2
MmugDNA.14150.1.S1_at	226519_s_at	AGXT2L2	alanine-glyoxylate aminotransferase 2-like 2	chr5q35.3	85007	LOC702302	similar to CG8745-PA	---	702302	MmugDNA.14150.1.S1_at_AGXT2L2
MmugDNA.14150.1.S1_s_at	NA	NA	NA	NA	NA	NA	NA	NA	NA	MmugDNA.14150.1.S1_s_at_NA
MmugDNA.14151.1.S1_at	213709_at	BHLHB9	basic helix-loop-helix domain containing, class B, 9	chrXq23	80823	LOC694634	similar to basic helix-loop-helix domain containing, class B, 9	---	694634	MmugDNA.14151.1.S1_at_BHLHB9
MmugDNA.1415.1.S1_at	240660_at	---	---	---	---	---	---	---	---	MmugDNA.1415.1.S1_at_---
MmugDNA.14152.1.S1_at	235293_at	---	---	---	---	---	---	---	---	MmugDNA.14152.1.S1_at_---
MmugDNA.14153.1.S1_at	208933_s_at	LGALS8	lectin, galactoside-binding, soluble, 8	chr1q42-q43	3964	LOC710375	similar to galectin 8 isoform a	---	710375	MmugDNA.14153.1.S1_at_LGALS8
MmugDNA.14154.1.S1_at	204085_s_at	CLN5	ceroid-lipofuscinosis, neuronal 5	chr13q21.1-q32	1203	LOC722516	Similar to Protein FAM3B precursor (Cytokine-like protein 2-21)	---	722516	MmugDNA.14154.1.S1_at_CLN5
MmugDNA.14155.1.S1_at	207270_x_at	CD300C	CD300c molecule	chr17q25.1	10871	CD300C	CD300c molecule	---	697839	MmugDNA.14155.1.S1_at_CD300C
MmugDNA.14156.1.S1_at	1562601_at	UNQ6975	NGNL6975	chr2p21	400952	---	---	---	---	MmugDNA.14156.1.S1_at_UNQ6975
MmugDNA.14157.1.S1_at	1553466_at	CXorf59	chromosome X open reading frame 59	chrXp21.1	286464	LOC695378	hypothetical protein LOC695378	---	695378	MmugDNA.14157.1.S1_at_CXorf59
MmugDNA.14158.1.S1_at	NA	NA	NA	NA	NA	NA	NA	NA	NA	MmugDNA.14158.1.S1_at_NA
MmugDNA.14158.1.S1_s_at	NA	NA	NA	NA	NA	NA	NA	NA	NA	MmugDNA.14158.1.S1_s_at_NA
MmugDNA.14159.1.S1_at	217684_at	TYMS	thymidylate synthetase	chr18p11.32	7298	TYMS	thymidylate synthetase	---	697580	MmugDNA.14159.1.S1_at_TYMS
MmugDNA.14160.1.S1_at	207836_s_at	RBPMS	RNA binding protein with multiple splicing	chr8p12-p11	11030	RBPMS	RNA binding protein with multiple splicing	---	694363	MmugDNA.14160.1.S1_at_RBPMS
MmugDNA.14161.1.S1_at	235428_at	---	---	---	---	---	---	---	---	MmugDNA.14161.1.S1_at_---
MmugDNA.1416.1.S1_at	238683_at	ZNF524	zinc finger protein 524	chr19q13.42	147807	ZNF524	zinc finger protein 524	---	701807	MmugDNA.1416.1.S1_at_ZNF524
MmugDNA.14162.1.S1_at	202845_s_at	RALBP1	ralA binding protein 1	chr18p11.3	10928	RALBP1	ralA binding protein 1	---	705571	MmugDNA.14162.1.S1_at_RALBP1
MmugDNA.14162.1.S1_s_at	NA	NA	NA	NA	NA	NA	NA	NA	NA	MmugDNA.14162.1.S1_s_at_NA
MmugDNA.14163.1.S1_at	235440_at	SPTY2D1	SPT2, Suppressor of Ty, domain containing 1 (S. cerevisiae)	chr11p15.1	144108	SPTY2D1	SPT2, Suppressor of Ty, domain containing 1 (S. cerevisiae)	---	693282	MmugDNA.14163.1.S1_at_SPTY2D1
MmugDNA.14164.1.S1_at	222074_at	UROD	uroporphyrinogen decarboxylase	chr1p34	7389	UROD	uroporphyrinogen decarboxylase	---	705166	MmugDNA.14164.1.S1_at_UROD
MmugDNA.14165.1.S1_at	215393_s_at	COBLL1	COBL-like 1	chr2q24.3	22837	COBLL1	COBL-like 1	---	702934	MmugDNA.14165.1.S1_at_COBLL1
MmugDNA.14166.1.S1_at	204426_at	TMED2	transmembrane emp24 domain trafficking protein 2	chr12q24.31	10959	LOC695168	similar to Transmembrane emp24 domain trafficking protein 2 precursor (Membrane protein p24A)	---	695168	MmugDNA.14166.1.S1_at_TMED2
MmugDNA.14167.1.S1_at	1569001_at	BMP1	bone morphogenetic protein 1	chr8p21.3	649	---	---	---	---	MmugDNA.14167.1.S1_at_BMP1
MmugDNA.14168.1.S1_at	221811_at	PERLD1	per1-like domain containing 1	chr17q12	93210	PERLD1	per1-like domain containing 1	---	697699	MmugDNA.14168.1.S1_at_PERLD1
MmugDNA.14169.1.S1_at	227747_at	MPZL3	myelin protein zero-like 3	chr11q23.3	196264	MPZL3	myelin protein zero-like 3	---	699211	MmugDNA.14169.1.S1_at_MPZL3
MmugDNA.14170.1.S1_at	222816_s_at	ZCCHC2	zinc finger, CCHC domain containing 2	chr18q21.33	54877	---	---	---	---	MmugDNA.14170.1.S1_at_ZCCHC2
MmugDNA.14171.1.S1_s_at	213341_at	FEM1C	fem-1 homolog c (C. elegans)	chr5q22	56929	FEM1C	fem-1 homolog c (C. elegans)	---	696022	MmugDNA.14171.1.S1_s_at_FEM1C
MmugDNA.1417.1.S1_at	228710_at	---	---	---	---	MYST1	MYST histone acetyltransferase 1	---	713117	MmugDNA.1417.1.S1_at_---
MmugDNA.14172.1.S1_at	233076_at	JAKMIP3	Janus kinase and microtubule interacting protein 3	chr10q26.3	282973	---	---	---	---	MmugDNA.14172.1.S1_at_JAKMIP3
MmugDNA.14173.1.S1_at	228047_at	SNORA72	small nucleolar RNA, H/ACA box 72	chr8q22	26775	LOC703120	similar to ribosomal protein L30	---	703120	MmugDNA.14173.1.S1_at_SNORA72
MmugDNA.14174.1.S1_at	1554706_at	OR2L13	olfactory receptor, family 2, subfamily L, member 13	chr1q44	284521	LOC706439	similar to olfactory receptor, family 2, subfamily L, member 13	---	706439	MmugDNA.14174.1.S1_at_OR2L13
MmugDNA.14175.1.S1_at	220763_at	HSPA12A	heat shock 70kDa protein 12A	chr10q26.12	259217	---	---	---	---	MmugDNA.14175.1.S1_at_HSPA12A
MmugDNA.14176.1.S1_s_at	1568720_at	ZNF506	zinc finger protein 506	chr19p13.11	440515	---	---	---	---	MmugDNA.14176.1.S1_s_at_ZNF506
MmugDNA.14177.1.S1_at	233968_at	CST11	cystatin 11	chr20p11.21	140880	CST11	cystatin 11	---	654419	MmugDNA.14177.1.S1_at_CST11
MmugDNA.14177.1.S1_s_at	233797_s_at	CST11	cystatin 11	chr20p11.21	140880	CST11	cystatin 11	---	654419	MmugDNA.14177.1.S1_s_at_CST11
MmugDNA.14178.1.S1_at	221176_x_at	WBSCR23	Williams-Beuren syndrome chromosome region 23	chr7q11.23	80112	---	---	---	---	MmugDNA.14178.1.S1_at_WBSCR23
MmugDNA.14179.1.S1_at	1553258_at	FLJ30679	hypothetical protein FLJ30679	chr16q24.1	146512	---	---	---	---	MmugDNA.14179.1.S1_at_FLJ30679
MmugDNA.14180.1.S1_at	220370_s_at	USP36	ubiquitin specific peptidase 36	chr17q25.3	57602	LOC710393	similar to ubiquitin specific protease 36	---	710393	MmugDNA.14180.1.S1_at_USP36
MmugDNA.14181.1.S1_at	231925_at	---	---	---	---	---	---	---	---	MmugDNA.14181.1.S1_at_---
MmugDNA.1418.1.S1_at	237227_at	NEK10	NIMA (never in mitosis gene a)- related kinase 10	chr3p24.1	152110	LOC701678	similar to NIMA (never in mitosis gene a)- related kinase 10 isoform 1	---	701678	MmugDNA.1418.1.S1_at_NEK10
MmugDNA.14182.1.S1_at	244769_at	---	---	---	---	---	---	---	---	MmugDNA.14182.1.S1_at_---
MmugDNA.14183.1.S1_at	206487_at	UNC84A	unc-84 homolog A (C. elegans)	chr7p22.3	23353	---	---	---	---	MmugDNA.14183.1.S1_at_UNC84A
MmugDNA.14184.1.S1_x_at	NA	NA	NA	NA	NA	NA	NA	NA	NA	MmugDNA.14184.1.S1_x_at_NA
MmugDNA.14185.1.S1_at	1561170_at	LOC100132025	transmembrane domain-containing protein ENSP00000320207-like	chr15q11.2	100132025	---	---	---	---	MmugDNA.14185.1.S1_at_LOC100132
MmugDNA.14186.1.S1_at	238748_at	---	---	---	---	LOC717415	Similar to putative aminopeptidase Fxna	---	717415	MmugDNA.14186.1.S1_at_---
MmugDNA.14187.1.S1_at	NA	NA	NA	NA	NA	NA	NA	NA	NA	MmugDNA.14187.1.S1_at_NA
MmugDNA.14187.1.S1_s_at	226963_at	BTF3L4	basic transcription factor 3-like 4	chr1p32.3	91408	LOC710271 /// LOC7129	hypothetical protein LOC710271 /// similar to basic transcription factor 3-like 4	---	710271 /// 712955	MmugDNA.14187.1.S1_s_at_BTF3L4
MmugDNA.14188.1.S1_at	205575_at	C1QL1	complement component 1, q subcomponent-like 1	chr17q21	10882	LOC715801	similar to C1q-related factor precursor (Complement component 1 Q subcomponent-like 1)	---	715801	MmugDNA.14188.1.S1_at_C1QL1
MmugDNA.14189.1.S1_at	234754_at	SLC37A1	solute carrier family 37 (glycerol-3-phosphate transporter), member 1	chr21q22.3	54020	---	---	---	---	MmugDNA.14189.1.S1_at_SLC37A1
MmugDNA.14190.1.S1_at	205344_at	CSPG5	chondroitin sulfate proteoglycan 5 (neuroglycan C)	chr3p21.3	10675	CSPG5	chondroitin sulfate proteoglycan 5 (neuroglycan C)	---	711213	MmugDNA.14190.1.S1_at_CSPG5
MmugDNA.14191.1.S1_at	204222_s_at	GLIPR1	GLI pathogenesis-related 1	chr12q21.2	11010	EPHA1	EPH receptor A1	---	706295	MmugDNA.14191.1.S1_at_GLIPR1
MmugDNA.1419.1.S1_at	232049_at	---	---	---	---	---	---	---	---	MmugDNA.1419.1.S1_at_---
MmugDNA.14192.1.S1_at	220190_s_at	GTF2A1L /// STON1 ///	general transcription factor IIA, 1-like /// stonin 1 /// STON1-GTF2A1L readthrough transcript	chr2p16.3	11036 /// 11037 /// 286	LOC715957	similar to stoned B/TFIIA-alpha/beta-like factor	---	715957	MmugDNA.14192.1.S1_at_GTF2A1L ///
MmugDNA.14193.1.S1_at	224955_at	TEAD1	TEA domain family member 1 (SV40 transcriptional enhancer factor)	chr11p15.2	7003	---	---	---	---	MmugDNA.14193.1.S1_at_TEAD1
MmugDNA.14194.1.S1_at	200619_at	SF3B2	splicing factor 3b, subunit 2, 145kDa	chr11q13.1	10992	LOC714747	similar to splicing factor 3B subunit 2	---	714747	MmugDNA.14194.1.S1_at_SF3B2
MmugDNA.14195.1.S1_at	1553446_at	FLJ37396	hypothetical protein FLJ37396	chr6q21	285754	LOC699986	hypothetical protein LOC699986	---	699986	MmugDNA.14195.1.S1_at_FLJ37396
MmugDNA.14196.1.S1_at	239287_at	---	---	---	---	---	---	---	---	MmugDNA.14196.1.S1_at_---
MmugDNA.14197.1.S1_at	229753_at	POU2F1	POU class 2 homeobox 1	chr1q22-q23	5451	---	---	---	---	MmugDNA.14197.1.S1_at_POU2F1
MmugDNA.14198.1.S1_at	229880_at	LOC643517	hypothetical LOC643517	chr2p25.3	643517	---	---	---	---	MmugDNA.14198.1.S1_at_LOC643517
MmugDNA.14199.1.S1_at	1556261_a_at	---	---	---	---	---	---	---	---	MmugDNA.14199.1.S1_at_---
MmugDNA.14.1.S1_at	1562984_at	---	---	---	---	---	---	---	---	MmugDNA.14.1.S1_at_---
MmugDNA.14200.1.S1_at	1555498_at	---	---	---	---	---	---	---	---	MmugDNA.14200.1.S1_at_---
MmugDNA.14201.1.S1_at	243710_at	---	---	---	---	---	---	---	---	MmugDNA.14201.1.S1_at_---
MmugDNA.1420.1.S1_s_at	231820_x_at	ZNF587	zinc finger protein 587	chr19q13.43	84914	---	---	---	---	MmugDNA.1420.1.S1_s_at_ZNF587
MmugDNA.14202.1.S1_at	1553346_a_at	TNRC6A	trinucleotide repeat containing 6A	chr16p11.2	27327	---	---	---	---	MmugDNA.14202.1.S1_at_TNRC6A
MmugDNA.14203.1.S1_at	209077_at	TXN2	thioredoxin 2	chr22q13.1	25828	---	---	---	---	MmugDNA.14203.1.S1_at_TXN2
MmugDNA.14204.1.S1_at	210164_at	GZMB	granzyme B (granzyme 2, cytotoxic T-lymphocyte-associated serine esterase 1)	chr14q11.2	3002	GZMB	granzyme B (granzyme 2, cytotoxic T-lymphocyte-associated serine esterase 1)	---	716518	MmugDNA.14204.1.S1_at_GZMB
MmugDNA.14205.1.S1_at	1553174_at	JPH2	junctophilin 2	chr20q13.12	57158	JPH2	junctophilin 2	---	694158	MmugDNA.14205.1.S1_at_JPH2
MmugDNA.14206.1.S1_at	220331_at	CYP46A1	cytochrome P450, family 46, subfamily A, polypeptide 1	chr14q32.1	10858	LOC705843	cytochrome P450, family 46	---	705843	MmugDNA.14206.1.S1_at_CYP46A1
MmugDNA.14207.1.S1_at	242640_at	C19orf55	chromosome 19 open reading frame 55	chr19q13.12	148137	---	---	---	---	MmugDNA.14207.1.S1_at_C19orf55
MmugDNA.14208.1.S1_at	237312_at	LOC731477	hypothetical protein LOC731477	---	731477	---	---	---	---	MmugDNA.14208.1.S1_at_LOC731477
MmugDNA.14209.1.S1_at	205695_at	SDS	serine dehydratase	chr12q24.13	10993	LOC713285	similar to serine dehydratase	---	713285	MmugDNA.14209.1.S1_at_SDS
MmugDNA.14210.1.S1_at	203133_at	SEC61B	Sec61 beta subunit	chr9q22.32-q31.3	10952	SEC61B	Sec61 beta subunit	---	717815	MmugDNA.14210.1.S1_at_SEC61B
MmugDNA.14211.1.S1_at	203818_s_at	SF3A3	splicing factor 3a, subunit 3, 60kDa	chr1p34.3	10946	LOC702443	similar to splicing factor 3a, subunit 3 (predicted)	---	702443	MmugDNA.14211.1.S1_at_SF3A3
MmugDNA.1421.1.S1_at	225968_at	PRICKLE2	prickle homolog 2 (Drosophila)	chr3p14.1	166336	LOC701254	similar to prickle-like 2	---	701254	MmugDNA.1421.1.S1_at_PRICKLE2
MmugDNA.14212.1.S1_at	206724_at	CBX4	chromobox homolog 4 (Pc class homolog, Drosophila)	chr17q25.3	8535	LOC717683	similar to chromobox homolog 4	---	717683	MmugDNA.14212.1.S1_at_CBX4
MmugDNA.14213.1.S1_at	227586_at	TMEM170A	transmembrane protein 170A	chr16q23.1	124491	---	---	---	---	MmugDNA.14213.1.S1_at_TMEM170A
MmugDNA.14214.1.S1_at	241620_at	SMCHD1	structural maintenance of chromosomes flexible hinge domain containing 1	chr18p11.32	23347	---	---	---	---	MmugDNA.14214.1.S1_at_SMCHD1
MmugDNA.14215.1.S1_at	230395_at	---	---	---	---	---	---	---	---	MmugDNA.14215.1.S1_at_---
MmugDNA.14216.1.S1_at	241841_at	---	---	---	---	---	---	---	---	MmugDNA.14216.1.S1_at_---
MmugDNA.14217.1.S1_at	240029_at	LOC143678	hypothetical LOC143678	chr11p11.2	143678	---	---	---	---	MmugDNA.14217.1.S1_at_LOC143678
MmugDNA.14218.1.S1_at	231264_at	TMCO2	transmembrane and coiled-coil domains 2	chr1p34.2	127391	TMCO2	similar to transmembrane and coiled-coil domains 2	---	693977	MmugDNA.14218.1.S1_at_TMCO2
MmugDNA.14219.1.S1_at	1564729_at	---	---	---	---	---	---	---	---	MmugDNA.14219.1.S1_at_---
MmugDNA.142.1.S1_at	240679_at	---	---	---	---	---	---	---	---	MmugDNA.142.1.S1_at_---
MmugDNA.14220.1.S1_s_at	210301_at	XDH	xanthine dehydrogenase	chr2p23.1	7498	XDH	xanthine dehydrogenase	---	708046	MmugDNA.14220.1.S1_s_at_XDH
MmugDNA.14221.1.S1_at	241247_at	---	---	---	---	---	---	---	---	MmugDNA.14221.1.S1_at_---
MmugDNA.1422.1.S1_at	232385_x_at	---	---	---	---	---	---	---	---	MmugDNA.1422.1.S1_at_---
MmugDNA.14222.1.S1_at	230951_at	---	---	---	---	---	---	---	---	MmugDNA.14222.1.S1_at_---
MmugDNA.14224.1.S1_at	225512_at	ZBTB38	zinc finger and BTB domain containing 38	chr3q23	253461	LOC715184	hypothetical protein LOC715184	---	715184	MmugDNA.14224.1.S1_at_ZBTB38
MmugDNA.14225.1.S1_s_at	211479_s_at	HTR2C	5-hydroxytryptamine (serotonin) receptor 2C	chrXq24	3358	---	---	---	---	MmugDNA.14225.1.S1_at_HTR2C
MmugDNA.14226.1.S1_at	225004_at	TMEM101	transmembrane protein 101	chr17q21.31	84336	LOC714166	hypothetical protein LOC714166	---	714166	MmugDNA.14226.1.S1_at_TMEM101
MmugDNA.14227.1.S1_at	203761_at	SLA	Src-like-adaptor	chr8q22.3-qter|8q24	6503	SLA	Src-like-adaptor	---	697377	MmugDNA.14227.1.S1_at_SLA
MmugDNA.14228.1.S1_at	233451_at	C20orf54	chromosome 20 open reading frame 54	chr20p13	113278	LOC715057	similar to Protein C20orf54 precursor	---	715057	MmugDNA.14228.1.S1_at_C20orf54

MmugDNA.14229.1.S1_at	210655_s_at	FOXO3 /// FOXO3B	forkhead box O3 /// forkhead box O3B pseudogene	chr17p11 /// chr6q21	2309 /// 2310	IL12RB2	Interleukin 12 receptor, beta 2	---	700677	MmugDNA.14229.1.S1_at_FOXO3 /// F
MmugDNA.14230.1.S1_at	205315_s_at	SNTB2	syntrophin, beta 2 (dystrophin-associated protein A1, 59kDa, basic component 2)	chr16q22-q23	6645	LOC699824	Similar to potassium channel tetramerisation domain containing 18	---	699824	MmugDNA.14230.1.S1_at_SNTB2
MmugDNA.14231.1.S1_at	205899_at	CCNA1	cyclin A1	chr13q12.3-q13	8900	CCNA1	cyclin A1	---	693797	MmugDNA.14231.1.S1_at_CCNA1
MmugDNA.1423.1.S1_at	1555309_a_at	CARD14	caspase recruitment domain family, member 14	chr17q25	79092	CARD14	caspase recruitment domain protein 14	---	712205	MmugDNA.1423.1.S1_at_CARD14
MmugDNA.14232.1.S1_at	238313_at	---	---	---	---	---	---	---	---	MmugDNA.14232.1.S1_at_---
MmugDNA.14233.1.S1_at	202506_at	SSFA2	sperm specific antigen 2	chr2q31.3	6744	SSFA2	sperm specific antigen 2	---	705181	MmugDNA.14233.1.S1_at_SSFA2
MmugDNA.14234.1.S1_at	200953_s_at	CCND2	cyclin D2	chr12p13	894	---	---	---	---	MmugDNA.14234.1.S1_at_CCND2
MmugDNA.14235.1.S1_at	229587_at	UBA2	ubiquitin-like modifier activating enzyme 2	chr19q12	10054	---	---	---	---	MmugDNA.14235.1.S1_at_UBA2
MmugDNA.14236.1.S1_at	217558_at	CYP2C9	cytochrome P450, family 2, subfamily C, polypeptide 9	chr10q24	1559	---	---	---	---	MmugDNA.14236.1.S1_at_CYP2C9
MmugDNA.14237.1.S1_at	215341_at	DNAH6	dynein, axonemal, heavy chain 6	chr2p11.2	1768	LOC694115	similar to Dynein heavy chain at 16F CG7092-PA	---	694115	MmugDNA.14237.1.S1_at_DNAH6
MmugDNA.14238.1.S1_at	232765_x_at	LOC146429	hypothetical protein LOC146429	chr16q24.3	146429	LOC712670	similar to Brain-type organic cation transporter (Solute carrier family 22 member 17)	---	712670	MmugDNA.14238.1.S1_at_LOC146429
MmugDNA.14239.1.S1_at	1555405_at	UBE3C	ubiquitin protein ligase E3C	chr7q36.3	9690	UBE3C	ubiquitin protein ligase E3C	---	719956	MmugDNA.14239.1.S1_at_UBE3C
MmugDNA.14240.1.S1_at	203514_at	MAP3K3	mitogen-activated protein kinase kinase kinase 3	chr17q23.3	4215	LOC717866	similar to mitogen-activated protein kinase kinase kinase 3 isoform 2	---	717866	MmugDNA.14240.1.S1_at_MAP3K3
MmugDNA.14241.1.S1_at	1568649_at	---	---	---	---	---	---	---	---	MmugDNA.14241.1.S1_at_---
MmugDNA.1424.1.S1_at	215367_at	KIAA1614	KIAA1614	chr1q25.3	57710	LOC716374 /// LOC7184	hypothetical protein LOC716374 /// hypothetical protein LOC718439	---	716374 /// 718439	MmugDNA.1424.1.S1_at_KIAA1614
MmugDNA.14242.1.S1_at	1556133_s_at	LOC100169752	hypothetical LOC100169752	chr10q	100169752	---	---	---	---	MmugDNA.14242.1.S1_at_LOC100169
MmugDNA.14243.1.S1_at	229106_at	DYNLL2	dynein, light chain, LC8-type 2	chr17q22	140735	---	---	---	---	MmugDNA.14243.1.S1_at_DYNLL2
MmugDNA.14244.1.S1_at	227079_at	DHX8	DEAH (Asp-Glu-Ala-His) box polypeptide 8	chr17q21.31	1659	---	---	---	---	MmugDNA.14244.1.S1_at_DHX8
MmugDNA.14245.1.S1_at	230246_at	PLAC9	placenta-specific 9	chr10q22.3	219348	PLAC9	placenta-specific 9	---	700881	MmugDNA.14245.1.S1_at_PLAC9
MmugDNA.14247.1.S1_at	229743_at	ZNF438	zinc finger protein 438	chr10p11.23	220929	LOC700170	similar to ZNF438 transcript variant 3	---	700170	MmugDNA.14247.1.S1_at_ZNF438
MmugDNA.14248.1.S1_at	1564697_a_at	hCG_1820661	hypothetical LOC400752	chr1p34.1	400752	---	---	---	---	MmugDNA.14248.1.S1_at_hCG_18206
MmugDNA.14249.1.S1_s_at	NA	NA	NA	NA	NA	NA	NA	NA	NA	MmugDNA.14249.1.S1_s_at_NA
MmugDNA.14250.1.S1_at	210849_s_at	VPS41	vacuolar protein sorting 41 homolog (S. cerevisiae)	chr7p14-p13	27072	LOC696746	Similar to THUMP domain containing 1	---	696746	MmugDNA.14250.1.S1_at_VPS41
MmugDNA.14251.1.S1_at	1561085_at	LOC153910	hypothetical LOC153910	chr6q24.1-q24.2	153910	---	---	---	---	MmugDNA.14251.1.S1_at_LOC153910
MmugDNA.1425.1.S1_at	222323_at	---	---	---	---	LOC711450	similar to crystallin, gamma D	---	711450	MmugDNA.1425.1.S1_at_---
MmugDNA.14252.1.S1_at	217576_x_at	SOS2	son of sevenless homolog 2 (Drosophila)	chr14q21	6655	LOC705601	similar to son of sevenless homolog 2	---	705601	MmugDNA.14252.1.S1_at_SOS2
MmugDNA.14253.1.S1_at	1553405_a_at	CSMD1	CUB and Sushi multiple domains 1	chr8p23.2	64478	LOC716670	similar to CUB and Sushi multiple domains 1	---	716670	MmugDNA.14253.1.S1_at_CSMD1
MmugDNA.14254.1.S1_at	243651_at	CPEB3 /// LOC1001307	cytoplasmic polyadenylation element binding protein 3 /// hypothetical protein LOC100130772	chr10q23.32	100130772 /// 22849	---	---	---	---	MmugDNA.14254.1.S1_at_CPEB3 /// L
MmugDNA.14255.1.S1_at	206593_s_at	MED22	mediator complex subunit 22	chr9q34.2	6837	LOC714097	similar to Surfeit locus protein 5	---	714097	MmugDNA.14255.1.S1_at_MED22
MmugDNA.14256.1.S1_at	201260_s_at	SYPL1	synaptophysin-like 1	chr7q22.2	6856	SYPL1	synaptophysin-like 1	---	698350	MmugDNA.14256.1.S1_at_SYPL1
MmugDNA.14257.1.S1_at	204826_at	CCNF	cyclin F	chr16p13.3	899	CCNF	cyclin F	---	696745	MmugDNA.14257.1.S1_at_CCNF
MmugDNA.14258.1.S1_s_at	201463_s_at	LOC100133665 /// TALI	similar to transaldolase /// transaldolase 1	chr11p15.5-p15.4	100133665 /// 6888	LOC720828	similar to transaldolase 1	---	720828	MmugDNA.14258.1.S1_s_at_LOC1001
MmugDNA.14259.1.S1_at	201660_at	ACSL3	Acyl-CoA synthetase long-chain family member 3	chr2q34-q35	2181	LOC703458	Similar to Histidine acid phosphatase domain containing 1	---	703458	MmugDNA.14259.1.S1_at_ACSL3
MmugDNA.14260.1.S1_at	NA	NA	NA	NA	NA	NA	NA	NA	NA	MmugDNA.14260.1.S1_at_NA
MmugDNA.14261.1.S1_at	1562651_at	ZSCAN1	zinc finger and SCAN domain containing 1	chr19q13.43	284312	---	---	---	---	MmugDNA.14261.1.S1_at_ZSCAN1
MmugDNA.14262.1.S1_at	232622_at	---	---	---	---	---	---	---	---	MmugDNA.14262.1.S1_at_---
MmugDNA.14263.1.S1_at	200976_s_at	TAX1BP1	Tax1 (human T-cell leukemia virus type I) binding protein 1	chr7p15	8887	LOC700533	Similar to developmental pluripotency associated 4	---	700533	MmugDNA.14263.1.S1_at_TAX1BP1
MmugDNA.14264.1.S1_at	NA	NA	NA	NA	NA	NA	NA	NA	NA	MmugDNA.14264.1.S1_at_NA
MmugDNA.14265.1.S1_at	236552_at	---	---	---	---	---	---	---	---	MmugDNA.14265.1.S1_at_---
MmugDNA.14266.1.S1_at	219273_at	CCNK	cyclin K	chr14q32	8812	---	---	---	---	MmugDNA.14266.1.S1_at_CCNK
MmugDNA.14267.1.S1_at	1557060_at	---	---	---	---	---	---	---	---	MmugDNA.14267.1.S1_at_---
MmugDNA.14268.1.S1_at	204645_at	CCNT2	cyclin T2	chr2q21.3	905	LOC708813	similar to cyclin T2 isoform b	---	708813	MmugDNA.14268.1.S1_at_CCNT2
MmugDNA.14269.1.S1_at	215366_at	SNX13	sorting nexin 13	chr7p21.1	23161	---	---	---	---	MmugDNA.14269.1.S1_at_SNX13
MmugDNA.14270.1.S1_at	243625_at	---	---	---	---	---	---	---	---	MmugDNA.14270.1.S1_at_---
MmugDNA.14271.1.S1_at	214033_at	ABCC6	ATP-binding cassette, sub-family C (CFTR/MRP), member 6	chr16p13.1	368	LOC717875	similar to ATP-binding cassette, sub-family C, member 6	---	717875	MmugDNA.14271.1.S1_at_ABCC6
MmugDNA.1427.1.S1_at	215457_at	---	---	---	---	---	---	---	---	MmugDNA.1427.1.S1_at_---
MmugDNA.14272.1.S1_at	214324_at	GP2	glycoprotein 2 (zymogen granule membrane)	chr16p12	2813	GP2	glycoprotein 2 (zymogen granule membrane)	---	695766	MmugDNA.14272.1.S1_at_GP2
MmugDNA.14273.1.S1_at	214325_at	GP2	glycoprotein 2 (zymogen granule membrane)	chr16p12	2813	GP2	glycoprotein 2 (zymogen granule membrane)	---	695766	MmugDNA.14273.1.S1_at_GP2
MmugDNA.14274.1.S1_at	1554212_s_at	KCNS2	potassium voltage-gated channel, delayed-rectifier, subfamily S, member 2	chr8q22	3788	KCNS2	potassium voltage-gated channel, delayed-rectifier, subfamily S, member 2	---	703802	MmugDNA.14274.1.S1_at_KCNS2
MmugDNA.14275.1.S1_at	1558473_at	---	---	---	---	---	---	---	---	MmugDNA.14275.1.S1_at_---
MmugDNA.14276.1.S1_at	1563391_at	---	---	---	---	---	---	---	---	MmugDNA.14276.1.S1_at_---
MmugDNA.14277.1.S1_at	1554343_a_at	STAP1	signal transducing adaptor family member 1	chr4q13.2	26228	BRDG1	BCR downstream signaling 1	---	711834	MmugDNA.14277.1.S1_at_STAP1
MmugDNA.14278.1.S1_at	216882_s_at	NEBL	nebulette	chr10p12	10529	NEBL	nebulette	---	702764	MmugDNA.14278.1.S1_at_NEBL
MmugDNA.14279.1.S1_at	1561690_at	---	---	---	---	---	---	---	---	MmugDNA.14279.1.S1_at_---
MmugDNA.14280.1.S1_at	202728_s_at	LTBP1	latent transforming growth factor beta binding protein 1	chr2p22-p21	4052	LOC709607	similar to latent transforming growth factor beta binding protein 1 isoform LTBP-1L	---	709607	MmugDNA.14280.1.S1_at_LTBP1
MmugDNA.14281.1.S1_at	222436_s_at	VPS24	vacuolar protein sorting 24 homolog (S. cerevisiae)	chr2p24.3-p24.1	51652	VPS24	similar to Charged multivesicular body protein 3 (Chromatin-modifying protein 3) (Vacuolar protein so	---	711833	MmugDNA.14281.1.S1_at_VPS24
MmugDNA.14281.1.S1_s_at	NA	NA	NA	NA	NA	NA	NA	NA	NA	MmugDNA.14281.1.S1_s_at_NA
MmugDNA.1428.1.S1_at	212481_s_at	TPM4	tropomyosin 4	chr19p13.1	7171	LOC695055	Similar to ARP6 actin-related protein 6 homolog	---	695055	MmugDNA.1428.1.S1_at_TPM4
MmugDNA.14282.1.S1_at	220051_at	PRSS21	protease, serine, 21 (testisin)	chr16p13.3	10942	PRSS21	protease, serine, 21 (testisin)	---	699296	MmugDNA.14282.1.S1_at_PRSS21
MmugDNA.14283.1.S1_at	205099_s_at	CCR1	chemokine (C-C motif) receptor 1	chr3p21	1230	---	---	---	---	MmugDNA.14283.1.S1_at_CCR1
MmugDNA.14284.1.S1_at	227711_at	GTSF1	gametocyte specific factor 1	chr12q13.2	121355	LOC696813	Similar to Alpha-1 catenin (Cadherin-associated protein) (Alpha E-catenin) (NY-REN-13 antigen)	---	696813	MmugDNA.14284.1.S1_at_GTSF1
MmugDNA.14285.1.S1_at	227255_at	PDIK1L	PDLIM1 interacting kinase 1 like	chr1p36.11	149420	---	---	---	---	MmugDNA.14285.1.S1_at_PDIK1L
MmugDNA.14286.1.S1_at	222417_s_at	SNX5	sorting nexin 5	chr20p11	27131	LOC699870	Similar to F49C5.4	---	699870	MmugDNA.14286.1.S1_at_SNX5
MmugDNA.14287.1.S1_at	224049_at	KCNK17	potassium channel, subfamily K, member 17	chr6p21.1	89822	LOC719574	similar to potassium channel, subfamily K, member 17	---	719574	MmugDNA.14287.1.S1_at_KCNK17
MmugDNA.14288.1.S1_at	226246_at	KCTD1	potassium channel tetramerisation domain containing 1	chr18q11.2	284252	---	---	---	---	MmugDNA.14288.1.S1_at_KCTD1
MmugDNA.14289.1.S1_at	226245_at	KCTD1	potassium channel tetramerisation domain containing 1	chr18q11.2	284252	---	---	---	---	MmugDNA.14289.1.S1_at_KCTD1
MmugDNA.14290.1.S1_at	NA	NA	NA	NA	NA	NA	NA	NA	NA	MmugDNA.14290.1.S1_at_NA
MmugDNA.14291.1.S1_at	226646_at	KLF2	Kruppel-like factor 2 (lung)	chr19p13.13-p13.11	10365	LOC719492	similar to Krueppel-like factor 2 (Lung krueppel-like factor)	---	719492	MmugDNA.14291.1.S1_at_KLF2
MmugDNA.1429.1.S1_at	228340_at	TLE3	transducin-like enhancer of split 3 (E(sp1) homolog, Drosophila)	chr15q22	7090	LOC698046	similar to transducin-like enhancer protein 3	---	698046	MmugDNA.1429.1.S1_at_TLE3
MmugDNA.14292.1.S1_at	1554345_a_at	GIN1	gypsy retrotransposon integrase 1	chr5q21.1	54826	LOC703340	hypothetical protein LOC703340	---	703340	MmugDNA.14292.1.S1_at_GIN1
MmugDNA.14294.1.S1_at	1553156_at	LHX4	LIM homeobox 4	chr1q25.2	89884	LOC716454	similar to LIM homeobox protein 4	---	716454	MmugDNA.14294.1.S1_at_LHX4
MmugDNA.14295.1.S1_at	222282_at	---	---	---	---	---	---	---	---	MmugDNA.14295.1.S1_at_---
MmugDNA.14296.1.S1_at	1560654_at	FLJ37201	tigger transposable element derived 2 pseudogene	chr10q23.31	283011	---	---	---	---	MmugDNA.14296.1.S1_at_FLJ37201
MmugDNA.14297.1.S1_at	NA	NA	NA	NA	NA	NA	NA	NA	NA	MmugDNA.14297.1.S1_at_NA
MmugDNA.14298.1.S1_at	206337_at	CCR7	chemokine (C-C motif) receptor 7	chr17q12-q21.2	1236	---	---	---	---	MmugDNA.14298.1.S1_at_CCR7
MmugDNA.14299.1.S1_at	225984_at	PRKAA1	protein kinase, AMP-activated, alpha 1 catalytic subunit	chr5p12	5562	LOC695558	similar to protein kinase, AMP-activated, alpha 1 catalytic subunit	---	695558	MmugDNA.14299.1.S1_at_PRKAA1
MmugDNA.14300.1.S1_at	225985_at	PRKAA1	protein kinase, AMP-activated, alpha 1 catalytic subunit	chr5p12	5562	LOC695558	similar to protein kinase, AMP-activated, alpha 1 catalytic subunit	---	695558	MmugDNA.14300.1.S1_at_PRKAA1
MmugDNA.14301.1.S1_at	201327_s_at	CCT6A	chaperonin containing TCP1, subunit 6A (zeta 1)	chr7p11.2	908	LOC717569	similar to chaperonin containing TCP1, subunit 6A isoform a	---	717569	MmugDNA.14301.1.S1_at_CCT6A
MmugDNA.1430.1.S1_at	221944_at	FLJ42627	hypothetical LOC645644	chr16p13.3	645644	LOC698663	similar to Potassium channel tetramerisation domain-containing protein 5	---	698663	MmugDNA.1430.1.S1_at_FLJ42627
MmugDNA.14302.1.S1_at	222410_s_at	SNX6	sorting nexin 6	chr14q13.2	58533	LOC693626	similar to sorting nexin 6	---	693626	MmugDNA.14302.1.S1_at_SNX6
MmugDNA.14303.1.S1_at	206749_at	CD1B	CD1b molecule	chr1q22-q23	910	CD1B	CD1b molecule	---	718955	MmugDNA.14303.1.S1_at_CD1B
MmugDNA.14304.1.S1_x_at	216552_x_at	KIR2DS4	killer cell immunoglobulin-like receptor, two domains, short cytoplasmic tail, 4	chr19q13.4	3809	---	---	---	---	MmugDNA.14304.1.S1_x_at_KIR2DS4
MmugDNA.14305.1.S1_at	1569502_s_at	KIAA0495	KIAA0495	chr1p36.32	57212	---	---	---	---	MmugDNA.14305.1.S1_at_KIAA0495
MmugDNA.14306.1.S1_at	205987_at	CD1C	CD1c molecule	chr1q22-q23	911	CD1C	CD1c molecule	---	717148	MmugDNA.14306.1.S1_at_CD1C
MmugDNA.14307.1.S1_at	223241_at	SNX8	sorting nexin 8	chr7p22.2	29886	SNX8	sorting nexin 8	---	698347	MmugDNA.14307.1.S1_at_SNX8
MmugDNA.14308.1.S1_at	205789_at	CD1D	CD1d molecule	chr1q22-q23	912	---	---	---	---	MmugDNA.14308.1.S1_at_CD1D
MmugDNA.14309.1.S1_at	201951_at	ALCAM	activated leukocyte cell adhesion molecule	chr3q13.1	214	ALCAM	activated leukocyte cell adhesion molecule	---	703777	MmugDNA.14309.1.S1_at_ALCAM
MmugDNA.14310.1.S1_at	201952_at	ALCAM	activated leukocyte cell adhesion molecule	chr3q13.1	214	ALCAM	activated leukocyte cell adhesion molecule	---	703777	MmugDNA.14310.1.S1_at_ALCAM
MmugDNA.14311.1.S1_at	239124_at	---	---	---	---	---	---	---	---	MmugDNA.14311.1.S1_at_---
MmugDNA.1431.1.S1_at	NA	NA	NA	NA	NA	NA	NA	NA	NA	MmugDNA.1431.1.S1_at_NA
MmugDNA.14312.1.S1_at	216334_s_at	CYP2A7P1	cytochrome P450, family 2, subfamily A, polypeptide 7 pseudogene 1	chr19q13.2	1550	LOC705143	similar to cytochrome P450, family 2, subfamily A, polypeptide 13	---	705143	MmugDNA.14312.1.S1_at_CYP2A7P1
MmugDNA.14313.1.S1_at	241241_at	RPS14	Ribosomal protein S14	chr5q31-q33	6208	---	---	---	---	MmugDNA.14313.1.S1_at_RPS14
MmugDNA.14314.1.S1_at	229964_at	C9orf152	chromosome 9 open reading frame 152	chr9q31.3	401546	---	---	---	---	MmugDNA.14314.1.S1_at_C9orf152
MmugDNA.14315.1.S1_at	228809_at	CXorf40A	chromosome X open reading frame 40A	chrXq28	91966	LOC702659	similar to chromosome X open reading frame 40	---	702659	MmugDNA.14315.1.S1_at_CXorf40A
MmugDNA.14316.1.S1_at	233599_at	hCG_2003663	hCG2003663	chr9q22.32	728061	---	---	---	---	MmugDNA.14316.1.S1_at_hCG_20036
MmugDNA.14317.1.S1_at	237660_at	C2orf21	chromosome 2 open reading frame 21	chr2q35	285175	---	---	---	---	MmugDNA.14317.1.S1_at_C2orf21
MmugDNA.14318.1.S1_at	228112_at	DNAH1	dynein, axonemal, heavy chain 1	chr3p21.1	25981	---	---	---	---	MmugDNA.14318.1.S1_at_DNAH1
MmugDNA.143.1.S1_at	241716_at	HSPD1	heat shock 60kDa protein 1 (chaperonin)	chr2q33.1	3329	---	---	---	---	MmugDNA.143.1.S1_at_HSPD1
MmugDNA.14320.1.S1_at	243327_at	---	---	---	---	---	---	---	---	MmugDNA.14320.1.S1_at_---
MmugDNA.14321.1.S1_at	225496_s_at	SYTL2	synaptotagmin-like 2	chr11q14	54843	SYTL2	synaptotagmin-like 2	---	703187	MmugDNA.14321.1.S1_at_SYTL2
MmugDNA.1432.1.S1_at	241017_at	TBC1D8	TBC1 domain family, member 8 (with GRAM domain)	chr2q11.2	11138	---	---	---	---	MmugDNA.1432.1.S1_at_TBC1D8
MmugDNA.14322.1.S1_at	242939_at	TFDP1	transcription factor Dp-1	chr13q34	7027	TFDP1	transcription factor Dp-1	---	715345	MmugDNA.14322.1.S1_at_TFDP1
MmugDNA.14323.1.S1_at	NA	NA	NA	NA	NA	NA	NA	NA	NA	MmugDNA.14323.1.S1_at_NA
MmugDNA.14323.1.S1_s_at	206520_x_at	SIGLEC6	sialic acid binding Ig-like lectin 6	chr19q13.3	946	SIGLEC6	sialic acid binding Ig-like lectin 6	---	719684	MmugDNA.14323.1.S1_s_at_SIGLEC6
MmugDNA.14324.1.S1_at	233768_at	---	---	---	---	---	---	---	---	MmugDNA.14324.1.S1_at_---
MmugDNA.14325.1.S1_at	235918_x_at	CEP97	centrosomal protein 97kDa	chr3q12.3	79598	---	---	---	---	MmugDNA.14325.1.S1_at_CEP97
MmugDNA.14325.1.S1_x_at	NA	NA	NA	NA	NA	NA	NA	NA	NA	MmugDNA.14325.1.S1_x_at_NA
MmugDNA.14326.1.S1_at	220000_at	SIGLEC5	sialic acid binding Ig-like lectin 5	chr19q13.3	8778	SIGLEC5	sialic acid binding Ig-like lectin 5	---	719730	MmugDNA.14326.1.S1_at_SIGLEC5

MmugDNA.14327.1.S1_at	226354_at	LACTB	lactamase, beta	chr15q22.1		114294	LACTB	lactamase, beta	---		705365	MmugDNA.14327.1.S1_at_LACTB
MmugDNA.14328.1.S1_at	NA	NA	NA	NA	NA		NA	NA	NA	NA		MmugDNA.14328.1.S1_at_NA
MmugDNA.14329.1.S1_at	217969_at	C11orf2	chromosome 11 open reading frame2	chr11q13		738	LOC719950	similar to Protein C11orf2 (Another new gene 2 protein)	---		719950	MmugDNA.14329.1.S1_at_C11orf2
MmugDNA.14330.1.S1_at	1562387_at	C5orf42	chromosome 5 open reading frame 42	chr5p13.2		65250	LOC701698	hypothetical protein LOC701698	---		701698	MmugDNA.14330.1.S1_at_C5orf42
MmugDNA.14331.1.S1_at	228225_at	PXMP3	peroxisomal membrane protein 3, 35kDa	chr8q21.1		5828	---	---	---	---		MmugDNA.14331.1.S1_at_PXMP3
MmugDNA.1433.1.S1_at	NA	NA	NA	NA	NA		NA	NA	NA	NA		MmugDNA.1433.1.S1_at_NA
MmugDNA.1433.1.S1_s_at	NA	NA	NA	NA	NA		NA	NA	NA	NA		MmugDNA.1433.1.S1_s_at_NA
MmugDNA.14332.1.S1_at	1560794_at	---	---	---	---		---	---	---	---		MmugDNA.14332.1.S1_at_---
MmugDNA.14333.1.S1_at	213586_at	CDKN2D	Cyclin-dependent kinase inhibitor 2D (p19, inhibits CDK4)	chr19p13		1032	CDKN2D	cyclin-dependent kinase inhibitor 2D (p19, inhibits CDK4)	---		713172	MmugDNA.14333.1.S1_at_CDKN2D
MmugDNA.14334.1.S1_at	241128_at	---	---	---	---		---	---	---	---		MmugDNA.14334.1.S1_at_---
MmugDNA.14336.1.S1_at	230391_at	---	---	---	---		---	---	---	---		MmugDNA.14336.1.S1_at_---
MmugDNA.14337.1.S1_at	232584_at	---	---	---	---		---	---	---	---		MmugDNA.14337.1.S1_at_---
MmugDNA.14338.1.S1_at	NA	NA	NA	NA	NA		NA	NA	NA	NA		MmugDNA.14338.1.S1_at_NA
MmugDNA.14339.1.S1_at	236019_at	RAB12	RAB12, member RAS oncogene family	chr18p11.22		201475	---	---	---	---		MmugDNA.14339.1.S1_at_RAB12
MmugDNA.14340.1.S1_at	NA	NA	NA	NA	NA		NA	NA	NA	NA		MmugDNA.14340.1.S1_at_NA
MmugDNA.14340.1.S1_s_at	223225_s_at	SEH1L	SEH1-like (S. cerevisiae)	chr18p11.21		81929	---	---	---	---		MmugDNA.14340.1.S1_s_at_SEH1L
MmugDNA.14341.1.S1_at	1557531_a_at	C10orf55	chromosome 10 open reading frame 55	chr10q22.2		414236	---	---	---	---		MmugDNA.14341.1.S1_at_C10orf55
MmugDNA.1434.1.S1_at	219144_at	DUSP26	dual specificity phosphatase 26 (putative)	chr8p12		78986	DUSP26	dual specificity phosphatase 26 (putative)	---		697134	MmugDNA.1434.1.S1_at_DUSP26
MmugDNA.14342.1.S1_at	204192_at	CD37	CD37 molecule	chr19q13.3		951	CD37	CD37 molecule	---		718718	MmugDNA.14342.1.S1_at_CD37
MmugDNA.14343.1.S1_at	1560512_at	---	---	---	---		---	---	---	---		MmugDNA.14343.1.S1_at_---
MmugDNA.14344.1.S1_at	1569061_at	IQGAP3	IQ motif containing GTPase activating protein 3	chr1q22-q23.1		128239	LOC719575	similar to IQ motif containing GTPase activating protein 3	---		719575	MmugDNA.14344.1.S1_at_IQGAP3
MmugDNA.14345.1.S1_at	201775_s_at	KIAA0494	KIAA0494	chr1pter-p22.1		9813	---	---	---	---		MmugDNA.14345.1.S1_at_KIAA0494
MmugDNA.14345.1.S1_s_at	NA	NA	NA	NA	NA		NA	NA	NA	NA		MmugDNA.14345.1.S1_s_at_NA
MmugDNA.14346.1.S1_at	240939_x_at	---	---	---	---		---	---	---	---		MmugDNA.14346.1.S1_at_---
MmugDNA.14347.1.S1_s_at	214574_x_at	LST1	leukocyte specific transcript 1	chr6p21.3		7940	LOC715042	similar to Leukocyte-specific transcript 1 protein (B144 protein)	---		715042	MmugDNA.14347.1.S1_s_at_LST1
MmugDNA.14348.1.S1_at	201776_s_at	KIAA0494	KIAA0494	chr1pter-p22.1		9813	---	---	---	---		MmugDNA.14348.1.S1_at_KIAA0494
MmugDNA.14349.1.S1_at	235049_at	ADCY1	adenylate cyclase 1 (brain)	chr7p13-p12		107	---	---	---	---		MmugDNA.14349.1.S1_at_ADCY1
MmugDNA.14350.1.S1_at	NA	NA	NA	NA	NA		NA	NA	NA	NA		MmugDNA.14350.1.S1_at_NA
MmugDNA.1435.1.S1_at	1554857_at	ELMO2	engulfment and cell motility 2	chr20q13		63916	ELMO2	engulfment and cell motility 2	---		707316	MmugDNA.1435.1.S1_at_ELMO2
MmugDNA.14352.1.S1_at	219482_at	SETD4	SET domain containing 4	chr21q22.13		54093	SETD4	SET domain containing 4	---		696003	MmugDNA.14352.1.S1_at_SETD4
MmugDNA.14354.1.S1_at	238276_at	---	---	---	---		---	---	---	---		MmugDNA.14354.1.S1_at_---
MmugDNA.14355.1.S1_at	218377_s_at	RWDD2B	RWD domain containing 2B	chr21q22.11		10069	RWDD2B	RWD domain containing 2B	---		706524	MmugDNA.14355.1.S1_at_RWDD2B
MmugDNA.14356.1.S1_at	201638_s_at	CPSF1	cleavage and polyadenylation specific factor 1, 160kDa	chr8q24.23		29894	CPSF1	cleavage and polyadenylation specific factor 1, 160kDa	---		702082	MmugDNA.14356.1.S1_at_CPSF1
MmugDNA.14357.1.S1_at	242218_at	PPARD	peroxisome proliferator-activated receptor delta	chr6p21.2-p21.1		5467	---	---	---	---		MmugDNA.14357.1.S1_at_PPARD
MmugDNA.14358.1.S1_at	241463_at	EIF2AK3	eukaryotic translation initiation factor 2-alpha kinase 3	chr2p12		9451	---	---	---	---		MmugDNA.14358.1.S1_at_EIF2AK3
MmugDNA.14359.1.S1_at	228327_x_at	MEIS3	Meis homeobox 3	chr19q13.32		56917	LOC718145	similar to Meis1, myeloid ecotropic viral integration site 1 homolog 3 isoform 2	---		718145	MmugDNA.14359.1.S1_at_MEIS3
MmugDNA.14360.1.S1_at	1558142_at	TNRC6B	trinucleotide repeat containing 6B	chr22q13.1		23112	---	---	---	---		MmugDNA.14360.1.S1_at_TNRC6B
MmugDNA.14361.1.S1_at	45572_s_at	GGA1	golgi associated, gamma adaptin ear containing, ARF binding protein 1	chr22q13.31		26088	GGA1	golgi associated, gamma adaptin ear containing, ARF binding protein 1	---		697929	MmugDNA.14361.1.S1_at_GGA1
MmugDNA.1436.1.S1_at	201164_s_at	PUM1	pumilio homolog 1 (Drosophila)	chr1p35.2		9698	PUM1	pumilio homolog 1	---		704848	MmugDNA.1436.1.S1_at_PUM1
MmugDNA.14362.1.S1_at	215577_at	---	---	---	---		---	---	---	---		MmugDNA.14362.1.S1_at_---
MmugDNA.14363.1.S1_at	222814_s_at	ZNHIT2	zinc finger, HIT type 2	chr11q13		741	LOC693305	Similar to DnaJ (Hsp40) homolog, subfamily B, member 6 isoform a	---		693305	MmugDNA.14363.1.S1_at_ZNHIT2
MmugDNA.14364.1.S1_at	215591_at	SATB2	SATB homeobox 2	chr2q33		23314	---	---	---	---		MmugDNA.14364.1.S1_at_SATB2
MmugDNA.14365.1.S1_at	222707_s_at	ACTR8	ARP8 actin-related protein 8 homolog (yeast)	---		93973	ACTR8	ARP8 actin-related protein 8 homolog (yeast)	---		693539	MmugDNA.14365.1.S1_at_ACTR8
MmugDNA.14366.1.S1_at	211611_s_at	ATF6B /// TNXB	activating transcription factor 6 beta /// tenascin XB	chr6p21.3	1388 /// 7148		ATF6B	activating transcription factor 6 beta	---		717050	MmugDNA.14366.1.S1_at_ATF6B /// TN
MmugDNA.14367.1.S1_at	229243_at	---	---	---	---		---	---	---	---		MmugDNA.14367.1.S1_at_---
MmugDNA.14368.1.S1_at	206465_at	ACSBG1	acyl-CoA synthetase bubblegum family member 1	chr15q23-q24		23205	LOC696641	Similar to ribosomal protein L27	---		696641	MmugDNA.14368.1.S1_at_ACSBG1
MmugDNA.14369.1.S1_at	231634_at	SLC12A3	solute carrier family 12 (sodium/chloride transporters), member 3	chr16q13		6559	---	---	---	---		MmugDNA.14369.1.S1_at_SLC12A3
MmugDNA.14370.1.S1_at	206466_at	ACSBG1	acyl-CoA synthetase bubblegum family member 1	chr15q23-q24		23205	---	---	---	---		MmugDNA.14370.1.S1_at_ACSBG1
MmugDNA.14371.1.S1_at	240425_x_at	ROBO2	Roundabout, axon guidance receptor, homolog 2 (Drosophila)	chr3p12.3		6092	---	---	---	---		MmugDNA.14371.1.S1_at_ROBO2
MmugDNA.1437.1.S1_at	1557429_s_at	C15orf33	chromosome 15 open reading frame 33	chr15q21.1-q21.2		196951	---	---	---	---		MmugDNA.1437.1.S1_at_C15orf33
MmugDNA.14372.1.S1_at	243392_at	USP49	ubiquitin specific peptidase 49	chr6p21		25862	USP49	ubiquitin specific peptidase 49	---		694862	MmugDNA.14372.1.S1_at_USP49
MmugDNA.14373.1.S1_at	205456_at	CD3E	CD3e molecule, epsilon (CD3-TCR complex)	chr11q23		916	---	---	---	---		MmugDNA.14373.1.S1_at_CD3E
MmugDNA.14374.1.S1_at	238417_at	PGM2L1	phosphoglucomutase 2-like 1	chr11q13.4		283209	---	---	---	---		MmugDNA.14374.1.S1_at_PGM2L1
MmugDNA.14375.1.S1_at	204118_at	CD48	CD48 molecule	chr1q21.3-q22		962	CD48	CD48 molecule	---		719762	MmugDNA.14375.1.S1_at_CD48
MmugDNA.14376.1.S1_at	238519_at	RSC1A1	regulatory solute carrier protein, family 1, member 1	chr1p36.1		6248	---	---	---	---		MmugDNA.14376.1.S1_at_RSC1A1
MmugDNA.14377.1.S1_s_at	205173_x_at	CD58	CD58 molecule	chr1p13		965	CD58	CD58 molecule	---		710732	MmugDNA.14377.1.S1_s_at_CD58
MmugDNA.14378.1.S1_at	228366_at	---	---	---	---		---	---	---	---		MmugDNA.14378.1.S1_at_---
MmugDNA.14379.1.S1_at	237816_at	---	---	---	---		---	---	---	---		MmugDNA.14379.1.S1_at_---
MmugDNA.14380.1.S1_at	200663_at	CD63	CD63 molecule	chr12q12-q13		967	CD63	CD63 molecule	---		709828	MmugDNA.14380.1.S1_at_CD63
MmugDNA.14381.1.S1_at	214213_x_at	LMNA	Lamin A/C	chr1q21.2-q21.3		4000	LMNA	lamin A/C	---		719414	MmugDNA.14381.1.S1_at_LMNA
MmugDNA.14381.1.S1_x_at	NA	NA	NA	NA	NA		NA	NA	NA	NA		MmugDNA.14381.1.S1_x_at_NA
MmugDNA.1438.1.S1_at	204387_x_at	MRP63	mitochondrial ribosomal protein 63	chr13q12.11		78988	LOC702952	similar to mitochondrial ribosomal protein 63	---		702952	MmugDNA.1438.1.S1_at_MRP63
MmugDNA.14382.1.S1_at	208605_s_at	NTRK1	neurotrophic tyrosine kinase, receptor, type 1	chr1q21-q22		4914	LOC719670	similar to neurotrophic tyrosine kinase, receptor, type 1 isoform 2	---		719670	MmugDNA.14382.1.S1_at_NTRK1
MmugDNA.14383.1.S1_at	231709_x_at	---	---	---	---		---	---	---	---		MmugDNA.14383.1.S1_at_---
MmugDNA.14384.1.S1_at	236996_at	---	---	---	---		---	---	---	---		MmugDNA.14384.1.S1_at_---
MmugDNA.14385.1.S1_at	205825_at	PCSK1	proprotein convertase subtilisin/kexin type 1	chr5q15-q21		5122	OTUD3	OTU domain containing 3	---		716815	MmugDNA.14385.1.S1_at_PCSK1
MmugDNA.14386.1.S1_s_at	218088_s_at	RRAGC	Ras-related GTP binding C	chr1p34		64121	LOC715549	similar to Ras-related GTP binding C	---		715549	MmugDNA.14386.1.S1_s_at_RRAGC
MmugDNA.14387.1.S1_at	204658_at	TRA2A	transformer 2 alpha homolog (Drosophila)	chr7p15.3		29896	LOC574247	transformer-2 alpha	---		574247	MmugDNA.14387.1.S1_at_TRA2A
MmugDNA.14388.1.S1_at	632_at	GSK3A	glycogen synthase kinase 3 alpha	chr19q13.2		2931	GSK3A	glycogen synthase kinase 3 alpha	---		707037	MmugDNA.14388.1.S1_at_GSK3A
MmugDNA.14389.1.S1_at	215698_at	KDM5A	lysine (K)-specific demethylase 5A	chr12p11		5927	LOC721599	hypothetical protein LOC721599	---		721599	MmugDNA.14389.1.S1_at_KDM5A
MmugDNA.14390.1.S1_at	240865_at	---	---	---	---		---	---	---	---		MmugDNA.14390.1.S1_at_---
MmugDNA.14391.1.S1_at	215028_at	SEMA6A	sema domain, transmembrane domain (TM), and cytoplasmic domain, (semaphorin) 6A	chr5q23.1		57556	---	---	---	---		MmugDNA.14391.1.S1_at_SEMA6A
MmugDNA.1439.1.S1_at	226572_at	SOCS7	suppressor of cytokine signaling 7	chr17q12		30837	---	---	---	---		MmugDNA.1439.1.S1_at_SOCS7
MmugDNA.14392.1.S1_at	225749_at	C16orf91	chromosome 16 open reading frame 91	chr16p13.3		283951	LOC701194	hypothetical protein LOC701194	---		701194	MmugDNA.14392.1.S1_at_C16orf91
MmugDNA.14393.1.S1_at	204594_s_at	SMCR7L	Smith-Magenis syndrome chromosome region, candidate 7-like	chr22q13		54471	LOC703747	hypothetical protein LOC703747	---		703747	MmugDNA.14393.1.S1_at_SMCR7L
MmugDNA.14394.1.S1_at	218566_s_at	CHORDC1	cysteine and histidine-rich domain (CHORD)-containing 1	chr11q14.3		26973	CHORDC1	cysteine and histidine-rich domain (CHORD)-containing 1	---		694312	MmugDNA.14394.1.S1_at_CHORDC1
MmugDNA.14395.1.S1_at	243095_at	---	---	---	---		---	---	---	---		MmugDNA.14395.1.S1_at_---
MmugDNA.14396.1.S1_at	205449_at	SAC3D1	SAC3 domain containing 1	chr11q13.1		29901	LOC717043	similar to SAC3 domain containing 1	---		717043	MmugDNA.14396.1.S1_at_SAC3D1
MmugDNA.14397.1.S1_at	230776_at	---	---	---	---		---	---	---	---		MmugDNA.14397.1.S1_at_---
MmugDNA.14399.1.S1_at	1553723_at	GPR97	G protein-coupled receptor 97	chr16q13		222487	GPR97	G protein-coupled receptor 97	---		705580	MmugDNA.14399.1.S1_at_GPR97
MmugDNA.14400.1.S1_at	NA	NA	NA	NA	NA		NA	NA	NA	NA		MmugDNA.14400.1.S1_at_NA
MmugDNA.14401.1.S1_at	1553221_at	ZNF583	zinc finger protein 583	chr19q13.43		147949	ZNF583	zinc finger protein 583	---		705205	MmugDNA.14401.1.S1_at_ZNF583
MmugDNA.1440.1.S1_at	244332_at	---	---	---	---		---	---	---	---		MmugDNA.1440.1.S1_at_---
MmugDNA.14402.1.S1_at	1560446_at	LOC100132815	hypothetical protein LOC100132815	chr19p12		100132815	---	---	---	---		MmugDNA.14402.1.S1_at_LOC100132
MmugDNA.14403.1.S1_at	1568943_at	INPP5D	inositol polyphosphate-5-phosphatase, 145kDa	chr2q37.1		3635	---	---	---	---		MmugDNA.14403.1.S1_at_INPP5D
MmugDNA.14404.1.S1_at	205988_at	CD84	CD84 molecule	chr1q24		8832	LOC719738	similar to CD84 antigen (leukocyte antigen)	---		719738	MmugDNA.14404.1.S1_at_CD84
MmugDNA.14405.1.S1_at	244412_at	---	---	---	---		---	---	---	---		MmugDNA.14405.1.S1_at_---
MmugDNA.14406.1.S1_at	241172_at	GRIA2	glutamate receptor, ionotropic, AMPA 2	chr4q32-q33		2891	---	---	---	---		MmugDNA.14406.1.S1_at_GRIA2
MmugDNA.14407.1.S1_at	53968_at	INTS5	integrator complex subunit 5	chr11q12.3		80789	LOC719124	similar to integrator complex subunit 5	---		719124	MmugDNA.14407.1.S1_at_INTS5
MmugDNA.14408.1.S1_at	204521_at	C12orf24	chromosome 12 open reading frame 24	chr12q24.11		29902	LOC709386	hypothetical protein LOC709386	---		709386	MmugDNA.14408.1.S1_at_C12orf24
MmugDNA.14409.1.S1_at	232960_at	NUP62CL	nucleoporin 62kDa C-terminal like	chrXq22.3		54830	LOC701722	similar to nucleoporin 62	---		701722	MmugDNA.14409.1.S1_at_NUP62CL
MmugDNA.14410.1.S1_at	207006_s_at	CCDC106	coiled-coil domain containing 106	chr19q13.42		29903	LOC702611	hypothetical protein LOC702611	---		702611	MmugDNA.14410.1.S1_at_CCDC106
MmugDNA.14411.1.S1_at	208042_at	AGGF1	angiogenic factor with G patch and FHA domains 1	chr5q13.3		55109	AGGF1	angiogenic factor with G patch and FHA domains 1	---		707686	MmugDNA.14411.1.S1_at_AGGF1
MmugDNA.1441.1.S1_at	226277_at	COL4A3BP	collagen, type IV, alpha 3 (Goodpasture antigen) binding protein	chr5q13.3		10087	---	---	---	---		MmugDNA.1441.1.S1_at_COL4A3BP
MmugDNA.14412.1.S1_at	213661_at	PAMR1	peptidase domain containing associated with muscle regeneration 1	chr11p13		25891	---	---	---	---		MmugDNA.14412.1.S1_at_PAMR1
MmugDNA.14413.1.S1_at	234444_at	---	---	---	---		---	---	---	---		MmugDNA.14413.1.S1_at_---
MmugDNA.14414.1.S1_at	1557208_at	LOC219731	hypothetical protein LOC219731	chr10p14		219731	---	---	---	---		MmugDNA.14414.1.S1_at_LOC219731
MmugDNA.14415.1.S1_at	237194_at	---	---	---	---		---	---	---	---		MmugDNA.14415.1.S1_at_---
MmugDNA.14416.1.S1_at	214829_at	AASS	aminoadipate-semialdehyde synthase	chr7q31.3		10157	---	---	---	---		MmugDNA.14416.1.S1_at_AASS
MmugDNA.14417.1.S1_at	242326_at	LOC100134109	similar to collagen, type XXII, alpha 1	---		100134109	---	---	---	---		MmugDNA.14417.1.S1_at_LOC100134
MmugDNA.14418.1.S1_at	NA	NA	NA	NA	NA		NA	NA	NA	NA		MmugDNA.14418.1.S1_at_NA
MmugDNA.14419.1.S1_at	1559762_at	---	---	---	---		---	---	---	---		MmugDNA.14419.1.S1_at_---
MmugDNA.144.1.S1_at	201576_s_at	GLB1	galactosidase, beta 1	chr3p21.33		2720	---	---	---	---		MmugDNA.144.1.S1_at_GLB1
MmugDNA.14420.1.S1_at	227771_at	LIFR	leukemia inhibitory factor receptor alpha	chr5p13-p12		3977	---	---	---	---		MmugDNA.14420.1.S1_at_LIFR
MmugDNA.14421.1.S1_at	239366_at	---	---	---	---		---	---	---	---		MmugDNA.14421.1.S1_at_---
MmugDNA.1442.1.S1_at	240446_at	---	---	---	---		---	---	---	---		MmugDNA.1442.1.S1_at_---
MmugDNA.14422.1.S1_at	214896_at	---	---	---	---		---	---	---	---		MmugDNA.14422.1.S1_at_---
MmugDNA.14423.1.S1_at	NA	NA	NA	NA	NA		NA	NA	NA	NA		MmugDNA.14423.1.S1_at_NA

MmugDNA.14424.1.S1_at	232218_at	---	---	---	---	---	---	---	---	MmugDNA.14424.1.S1_at_---
MmugDNA.14425.1.S1_at	240978_at	---	---	---	---	---	---	---	---	MmugDNA.14425.1.S1_at_---
MmugDNA.14426.1.S1_at	213758_at	COX4I1	cytochrome c oxidase subunit IV isoform 1	chr16q22-qter	1327	LOC693811 /// LOC7149	similar to Cytochrome c oxidase subunit 4 isoform 1, mitochondrial precursor (Cytochrome c oxidase	---	693811 /// 714951	MmugDNA.14426.1.S1_at_COX4I1
MmugDNA.14427.1.S1_at	229412_at	TAF8	TAF8 RNA polymerase II, TATA box binding protein (TBP)-associated factor, 43kDa	chr6p21.1	129685	TAF8	TAF8 RNA polymerase II, TATA box binding protein (TBP)-associated factor, 43kDa	---	696809	MmugDNA.14427.1.S1_at_TAF8
MmugDNA.14428.1.S1_s_at	208022_s_at	CDC14B	CDC14 cell division cycle 14 homolog B (S. cerevisiae)	chr9q22.33	8555	LOC710300	similar to CDC14 homolog B isoform 1	---	710300	MmugDNA.14428.1.S1_s_at_CDC14B
MmugDNA.14429.1.S1_at	1553391_at	CXorf58	chromosome X open reading frame 58	chrXp22.11	254158	LOC698265	hypothetical protein LOC698265	---	698265	MmugDNA.14429.1.S1_at_CXorf58
MmugDNA.14430.1.S1_at	NA	NA	NA	NA	NA	NA	NA	NA	NA	MmugDNA.14430.1.S1_at_NA
MmugDNA.14431.1.S1_at	215292_s_at	MKL1	megakaryoblastic leukemia (translocation) 1	chr22q13	57591	---	---	---	---	MmugDNA.14431.1.S1_at_MKL1
MmugDNA.1443.1.S1_at	232546_at	TP73	tumor protein p73	chr1p36.3	7161	---	---	---	---	MmugDNA.1443.1.S1_at_TP73
MmugDNA.14432.1.S1_at	242264_at	---	---	---	---	---	---	---	---	MmugDNA.14432.1.S1_at_---
MmugDNA.14433.1.S1_at	240357_at	---	---	---	---	---	---	---	---	MmugDNA.14433.1.S1_at_---
MmugDNA.14434.1.S1_at	201973_s_at	C7orf28A /// C7orf28B /	chromosome 7 open reading frame 28A /// chromosome 7 open reading frame 28B /// similar to Chro	chr7p22.1	100133775 /// 221960 /	---	---	---	---	MmugDNA.14434.1.S1_at_C7orf28A ///
MmugDNA.14435.1.S1_at	220522_at	CRB1	crumbs homolog 1 (Drosophila)	chr1q31-q32.1	23418	HNRPA1	Similar to Heterogeneous nuclear ribonucleoprotein A1 (Helix-destabilizing protein) (Single-strand bin	---	713437	MmugDNA.14435.1.S1_at_CRB1
MmugDNA.14436.1.S1_at	229516_at	WDR31	WD repeat domain 31	chr9q32	114987	---	---	---	---	MmugDNA.14436.1.S1_at_WDR31
MmugDNA.14437.1.S1_at	219293_s_at	OLA1	Obg-like ATPase 1	chr2q31.1	29789	PTD004	GTP-binding protein PTD004	---	697309	MmugDNA.14437.1.S1_at_OLA1
MmugDNA.14438.1.S1_at	203766_s_at	LMOD1	leiomodin 1 (smooth muscle)	chr1q32	25802	LMOD1	leiomodin 1 (smooth muscle)	---	706900	MmugDNA.14438.1.S1_at_LMOD1
MmugDNA.14439.1.S1_at	239723_at	---	---	---	---	---	---	---	---	MmugDNA.14439.1.S1_at_---
MmugDNA.14440.1.S1_at	225698_at	C5orf26	chromosome 5 open reading frame 26	chr5q21-q22	114915	---	---	---	---	MmugDNA.14440.1.S1_at_C5orf26
MmugDNA.14441.1.S1_at	NA	NA	NA	NA	NA	NA	NA	NA	NA	MmugDNA.14441.1.S1_at_NA
MmugDNA.1444.1.S1_s_at	214011_s_at	NOP16	NOP16 nucleolar protein homolog (yeast)	chr5q35.2	51491	NOP16	NOP16 nucleolar protein homolog (yeast)	---	696768	MmugDNA.1444.1.S1_s_at_NOP16
MmugDNA.14442.1.S1_at	231111_at	---	---	---	---	---	---	---	---	MmugDNA.14442.1.S1_at_---
MmugDNA.14443.1.S1_at	1553666_at	CCDC34	coiled-coil domain containing 34	chr11p14.1	91057	---	---	---	---	MmugDNA.14443.1.S1_at_CCDC34
MmugDNA.14444.1.S1_at	243670_at	---	---	---	---	---	---	---	---	MmugDNA.14444.1.S1_at_---
MmugDNA.14445.1.S1_at	228766_at	CD36	CD36 molecule (thrombospondin receptor)	chr7q11.2	948	---	---	---	---	MmugDNA.14445.1.S1_at_CD36
MmugDNA.14446.1.S1_at	1552779_a_at	SLC44A5	solute carrier family 44, member 5	chr1p31.1	204962	SLC44A5	solute carrier family 44, member 5	---	704729	MmugDNA.14446.1.S1_at_SLC44A5
MmugDNA.14447.1.S1_at	1552782_at	SLC44A5	solute carrier family 44, member 5	chr1p31.1	204962	SLC44A5	solute carrier family 44, member 5	---	704729	MmugDNA.14447.1.S1_at_SLC44A5
MmugDNA.14448.1.S1_at	241472_at	---	---	---	---	---	---	---	---	MmugDNA.14448.1.S1_at_---
MmugDNA.14449.1.S1_at	232813_s_at	GOLGA6 /// GOLGA6B	golgi autoantigen, golgin subfamily a, 6 /// golgi autoantigen, golgin subfamily a, 6B /// golgi autoantig	chr15q24.1 /// chr15q24.2	342096 /// 55889 /// 65	GOLGA6 /// LOC706794	golgi autoantigen, golgin subfamily a, 6 /// similar to Golgin subfamily A member 6 (Golgin linked to PI	---	706794 /// 707197	MmugDNA.14449.1.S1_at_GOLGA6 ///
MmugDNA.14450.1.S1_at	1564785_at	LOC196913	hypothetical protein LOC196913	chr14q22.1	196913	---	---	---	---	MmugDNA.14450.1.S1_at_LOC196913
MmugDNA.14451.1.S1_at	1559421_at	---	---	---	---	---	---	---	---	MmugDNA.14451.1.S1_at_---
MmugDNA.1445.1.S1_at	233502_at	---	---	---	---	---	---	---	---	MmugDNA.1445.1.S1_at_---
MmugDNA.14452.1.S1_at	1560204_at	NT5DC4	5'-nucleotidase domain containing 4	chr2q13	284958	LOC704273	similar to Cytosolic purine 5-nucleotidase (5-nucleotidase cytosolic II)	---	704273	MmugDNA.14452.1.S1_at_NT5DC4
MmugDNA.14453.1.S1_at	206897_at	PAGE1	P antigen family, member 1 (prostate associated)	chrXp11.23	8712	PAGE1	P antigen family, member 1 (prostate associated)	---	693588	MmugDNA.14453.1.S1_at_PAGE1
MmugDNA.14454.1.S1_at	222362_at	AGFG2	ArfGAP with FG repeats 2	chr7q22.1	3268	---	---	---	---	MmugDNA.14454.1.S1_at_AGFG2
MmugDNA.14455.1.S1_at	235417_at	SPOCD1	SPOC domain containing 1	chr1p35.2	90853	LOC706363	similar to SPOC domain containing 1	---	706363	MmugDNA.14455.1.S1_at_SPOCD1
MmugDNA.14456.1.S1_at	1552943_at	GABRG1	gamma-aminobutyric acid (GABA) A receptor, gamma 1	chr4p12	2565	---	---	---	---	MmugDNA.14456.1.S1_at_GABRG1
MmugDNA.14457.1.S1_at	203893_at	TAF9	TAF9 RNA polymerase II, TATA box binding protein (TBP)-associated factor, 32kDa	chr5q11.2-q13.1	6880	---	---	---	---	MmugDNA.14457.1.S1_at_TAF9
MmugDNA.14458.1.S1_at	200861_at	CNOT1	CCR4-NOT transcription complex, subunit 1	chr16q21	23019	LOC712880	similar to CCR4-NOT transcription complex, subunit 1 isoform a	---	712880	MmugDNA.14458.1.S1_at_CNOT1
MmugDNA.14459.1.S1_at	218275_at	SLC25A10	solute carrier family 25 (mitochondrial carrier; dicarboxylate transporter), member 10	chr17q25.3	1468	SLC25A10	solute carrier family 25 (mitochondrial carrier; dicarboxylate transporter), member 10	---	714494	MmugDNA.14459.1.S1_at_SLC25A10
MmugDNA.14460.1.S1_at	207785_s_at	RBPJ	recombination signal binding protein for immunoglobulin kappa J region	chr4p15.2	3516	RBPJ	recombination signal binding protein for immunoglobulin kappa J region	---	694601	MmugDNA.14460.1.S1_at_RBPJ
MmugDNA.14461.1.S1_at	1554787_at	C9orf96	chromosome 9 open reading frame 96	chr9q34.2	169436	MRPS18B	Mitochondrial ribosomal protein S18B	---	713694	MmugDNA.14461.1.S1_at_C9orf96
MmugDNA.1446.1.S1_at	239615_at	SLC22A5	solute carrier family 22 (organic cation/carnitine transporter), member 5	chr5q31	6584	SLC22A5	solute carrier family 22 (organic cation/carnitine transporter), member 5	---	707590	MmugDNA.1446.1.S1_at_SLC22A5
MmugDNA.14462.1.S1_at	214043_at	PTPRD	protein tyrosine phosphatase, receptor type, D	chr9p23-p24.3	5789	---	---	---	---	MmugDNA.14462.1.S1_at_PTPRD
MmugDNA.14463.1.S1_at	220125_at	DNAI1	dynein, axonemal, intermediate chain 1	chr9p21-p13	27019	DNAI1	dynein, axonemal, intermediate chain 1	---	701307	MmugDNA.14463.1.S1_at_DNAI1
MmugDNA.14464.1.S1_at	201772_at	AZIN1	antizyme inhibitor 1	chr8q22.3	51582	AZIN1	antizyme inhibitor 1	---	693581	MmugDNA.14464.1.S1_at_AZIN1
MmugDNA.14465.1.S1_at	230690_at	TUBB1	tubulin, beta 1	chr20q13.32	81027	---	---	---	---	MmugDNA.14465.1.S1_at_TUBB1
MmugDNA.14466.1.S1_at	244491_at	---	---	---	---	LOC698656	hypothetical protein LOC698656	---	698656	MmugDNA.14466.1.S1_at_---
MmugDNA.14467.1.S1_at	215521_at	PHC3	polyhomeotic homolog 3 (Drosophila)	chr3q26.2	80012	---	---	---	---	MmugDNA.14467.1.S1_at_PHC3
MmugDNA.14468.1.S1_at	217011_at	GBX1	gastrulation brain homeobox 1	chr7q36	2636	LOC714193	similar to gastrulation brain homeobox 1	---	714193	MmugDNA.14468.1.S1_at_GBX1
MmugDNA.14469.1.S1_at	1556154_a_at	MGC23284	hypothetical LOC197187	chr16q24.3	197187	---	---	---	---	MmugDNA.14469.1.S1_at_MGC23284
MmugDNA.14470.1.S1_at	239874_at	PDE8A	Phosphodiesterase 8A	chr15q25.3	5151	---	---	---	---	MmugDNA.14470.1.S1_at_PDE8A
MmugDNA.14471.1.S1_at	237787_at	---	---	---	---	---	---	---	---	MmugDNA.14471.1.S1_at_---
MmugDNA.1447.1.S1_at	209756_s_at	MYCN	v-myc myelocytomatosis viral related oncogene, neuroblastoma derived (avian)	chr2p24.1	4613	MYCN	v-myc myelocytomatosis viral related oncogene, neuroblastoma derived (avian)	---	698699	MmugDNA.1447.1.S1_at_MYCN
MmugDNA.14472.1.S1_at	1559578_at	---	---	---	---	LOC695525	hypothetical protein LOC695525	---	695525	MmugDNA.14472.1.S1_at_---
MmugDNA.14473.1.S1_at	239616_at	REXO2	REX2, RNA exonuclease 2 homolog (S. cerevisiae)	chr11q23.1-q23.2	25996	CAPN2	Calpain 2, (m/II) large subunit	---	703047	MmugDNA.14473.1.S1_at_REXO2
MmugDNA.14474.1.S1_s_at	NA	NA	NA	NA	NA	NA	NA	NA	NA	MmugDNA.14474.1.S1_s_at_NA
MmugDNA.14475.1.S1_at	208176_at	DUX1	double homeobox, 1	chr10q26.3	26584	LOC720104	similar to double homeobox, 4	---	720104	MmugDNA.14475.1.S1_at_DUX1
MmugDNA.14476.1.S1_s_at	1566040_at	---	---	---	---	---	---	---	---	MmugDNA.14476.1.S1_s_at_---
MmugDNA.14477.1.S1_at	1569713_at	---	---	---	---	---	---	---	---	MmugDNA.14477.1.S1_at_---
MmugDNA.14478.1.S1_at	242655_at	---	---	---	---	---	---	---	---	MmugDNA.14478.1.S1_at_---
MmugDNA.14479.1.S1_at	238218_at	OOEP	oocyte expressed protein homolog (dog)	chr6q13	441161	LOC717634	hypothetical protein LOC717634	---	717634	MmugDNA.14479.1.S1_at_OOEP
MmugDNA.14480.1.S1_at	203974_at	HDHD1A	haloacid dehalogenase-like hydrolase domain containing 1A	chrXp22.32	8226	LOC717603	Similar to SMG-7 homolog isoform 2	---	717603	MmugDNA.14480.1.S1_at_HDHD1A
MmugDNA.14481.1.S1_at	230952_at	LOC730092	RRN3 RNA polymerase I transcription factor homolog (S. cerevisiae) pseudogene	chr16p12.1	730092	---	---	---	---	MmugDNA.14481.1.S1_at_LOC730092
MmugDNA.1448.1.S1_s_at	210649_s_at	ARID1A	AT rich interactive domain 1A (SWI-like)	chr1p35.3	8289	LOC719949	similar to AT rich interactive domain 1A isoform a	---	719949	MmugDNA.1448.1.S1_s_at_ARID1A
MmugDNA.14482.1.S1_at	212502_at	ADO	2-aminoethanethiol (cysteamine) dioxygenase	chr10q21.2	84890	ADO	2-aminoethanethiol (cysteamine) dioxygenase	---	698259	MmugDNA.14482.1.S1_at_ADO
MmugDNA.14483.1.S1_at	212500_at	ADO	2-aminoethanethiol (cysteamine) dioxygenase	chr10q21.2	84890	ADO	2-aminoethanethiol (cysteamine) dioxygenase	---	698259	MmugDNA.14483.1.S1_at_ADO
MmugDNA.14484.1.S1_at	209313_at	GPN1	GPN-loop GTPase 1	chr2p23.3	11321	LOC701903	similar to XPA binding protein 1	---	701903	MmugDNA.14484.1.S1_at_GPN1
MmugDNA.14485.1.S1_at	243000_at	CDK6	cyclin-dependent kinase 6	chr7q21-q22	1021	---	---	---	---	MmugDNA.14485.1.S1_at_CDK6
MmugDNA.14486.1.S1_at	208868_s_at	GABARAPL1	GABA(A) receptor-associated protein like 1	chr12p13.2	23710	LOC702869	similar to gamma-aminobutyric acid (GABA(A)) receptor-associated protein-like 1	---	702869	MmugDNA.14486.1.S1_at_GABARAPL
MmugDNA.14487.1.S1_at	210180_s_at	TRA2B	transformer 2 beta homolog (Drosophila)	chr3q26.2-q27	6434	---	---	---	---	MmugDNA.14487.1.S1_at_TRA2B
MmugDNA.14488.1.S1_at	229442_at	C18orf54	chromosome 18 open reading frame 54	chr18q21.2	162681	LOC694192	similar to Protein C18orf54 precursor	---	694192	MmugDNA.14488.1.S1_at_C18orf54
MmugDNA.14489.1.S1_at	231272_at	---	---	---	---	---	---	---	---	MmugDNA.14489.1.S1_at_---
MmugDNA.14490.1.S1_at	211153_s_at	TNFSF11	tumor necrosis factor (ligand) superfamily, member 11	chr13q14	8600	TNFSF11	tumor necrosis factor (ligand) superfamily, member 11	---	699955	MmugDNA.14490.1.S1_at_TNFSF11
MmugDNA.14491.1.S1_at	1557848_at	---	---	---	---	---	---	---	---	MmugDNA.14491.1.S1_at_---
MmugDNA.1449.1.S1_at	224670_at	SYS1	SYS1 Golgi-localized integral membrane protein homolog (S. cerevisiae)	chr20q13.12	90196	LOC717604	similar to SCF apoptosis response protein 1 isoform 2	---	717604	MmugDNA.1449.1.S1_at_SYS1
MmugDNA.14492.1.S1_at	237427_at	---	---	---	---	---	---	---	---	MmugDNA.14492.1.S1_at_---
MmugDNA.14493.1.S1_at	1560469_at	NR5A2	nuclear receptor subfamily 5, group A, member 2	chr1q32.1	2494	---	---	---	---	MmugDNA.14493.1.S1_at_NR5A2
MmugDNA.14494.1.S1_at	1569664_at	---	---	---	---	---	---	---	---	MmugDNA.14494.1.S1_at_---
MmugDNA.14495.1.S1_at	229180_at	WWC1	WW and C2 domain containing 1	chr5q35.1	23286	---	---	---	---	MmugDNA.14495.1.S1_at_WWC1
MmugDNA.14496.1.S1_at	1562802_at	---	---	---	---	---	---	---	---	MmugDNA.14496.1.S1_at_---
MmugDNA.14497.1.S1_at	1557865_at	---	---	---	---	---	---	---	---	MmugDNA.14497.1.S1_at_---
MmugDNA.14498.1.S1_at	210025_s_at	CARD10	caspase recruitment domain family, member 10	chr22q13.1	29775	---	---	---	---	MmugDNA.14498.1.S1_at_CARD10
MmugDNA.14499.1.S1_at	232641_at	ZNF596	zinc finger protein 596	chr8p23.3	169270	LOC716013	similar to zinc finger protein 596	---	716013	MmugDNA.14499.1.S1_at_ZNF596
MmugDNA.14500.1.S1_at	1553780_at	MGC23270	hypothetical LOC196872	chr14q32.33	196872	---	---	---	---	MmugDNA.14500.1.S1_at_MGC23270
MmugDNA.14501.1.S1_at	201027_s_at	EIF5B	eukaryotic translation initiation factor 5B	chr2q11.2	9669	EIF5B	eukaryotic translation initiation factor 5B	---	714129	MmugDNA.14501.1.S1_at_EIF5B
MmugDNA.1450.1.S1_at	229584_at	LRRK2	leucine-rich repeat kinase 2	chr12q12	120892	LOC697930	similar to leucine-rich repeat kinase 2	---	697930	MmugDNA.1450.1.S1_at_LRRK2
MmugDNA.14502.1.S1_s_at	218287_s_at	EIF2C1	eukaryotic translation initiation factor 2C, 1	chr1p35-p34	26523	LOC712398	similar to eukaryotic translation initiation factor 2C, 1	---	712398	MmugDNA.14502.1.S1_s_at_EIF2C1
MmugDNA.14504.1.S1_at	230218_at	HIC1	hypermethylated in cancer 1	chr17p13.3	3090	---	---	---	---	MmugDNA.14504.1.S1_at_HIC1
MmugDNA.14505.1.S1_at	204398_s_at	EML2	echinoderm microtubule associated protein like 2	chr19q13.32	24139	EML2	similar to Echinoderm microtubule-associated protein-like 2 (EMAP-2) (HuEMAP-2)	---	703425	MmugDNA.14505.1.S1_at_EML2
MmugDNA.14506.1.S1_at	NA	NA	NA	NA	NA	NA	NA	NA	NA	MmugDNA.14506.1.S1_at_NA
MmugDNA.14507.1.S1_at	202926_at	NBAS	neuroblastoma amplified sequence	chr2p24	51594	LOC698203	similar to neuroblastoma-amplified protein	---	698203	MmugDNA.14507.1.S1_at_NBAS
MmugDNA.14508.1.S1_s_at	214771_x_at	MPRIP	myosin phosphatase Rho interacting protein	chr17p11.2	23164	---	---	---	---	MmugDNA.14508.1.S1_s_at_MPRIP
MmugDNA.14509.1.S1_at	241336_at	---	---	---	---	---	---	---	---	MmugDNA.14509.1.S1_at_---
MmugDNA.14510.1.S1_at	232150_at	---	---	---	---	---	---	---	---	MmugDNA.14510.1.S1_at_---
MmugDNA.1451.1.S1_at	224669_at	SYS1	SYS1 Golgi-localized integral membrane protein homolog (S. cerevisiae)	chr20q13.12	90196	LOC717604	similar to SCF apoptosis response protein 1 isoform 2	---	717604	MmugDNA.1451.1.S1_at_SYS1
MmugDNA.14512.1.S1_at	NA	NA	NA	NA	NA	NA	NA	NA	NA	MmugDNA.14512.1.S1_at_NA
MmugDNA.14513.1.S1_at	1554428_s_at	NLGN2	neuroligin 2	chr17p13.1	57555	---	---	---	---	MmugDNA.14513.1.S1_at_NLGN2
MmugDNA.14514.1.S1_at	240886_at	CASR	Calcium-sensing receptor	chr3q13	846	---	---	---	---	MmugDNA.14514.1.S1_at_CASR
MmugDNA.14515.1.S1_at	235522_at	CLEC2D	C-type lectin domain family 2, member D	chr12p13	29121	---	---	---	---	MmugDNA.14515.1.S1_at_CLEC2D
MmugDNA.14517.1.S1_at	226723_at	CCDC23	coiled-coil domain containing 23	chr1p34.2	374969	LOC698308	similar to coiled-coil domain containing 23	---	698308	MmugDNA.14517.1.S1_at_CCDC23
MmugDNA.14518.1.S1_at	222344_at	---	---	---	---	---	---	---	---	MmugDNA.14518.1.S1_at_---
MmugDNA.14519.1.S1_s_at	211355_x_at	LEPR	leptin receptor	chr1p31	3953	LOC574126	leptin receptor long insert isoform	---	574126	MmugDNA.14519.1.S1_s_at_LEPR
MmugDNA.145.1.S1_at	242399_at	---	---	---	---	---	---	---	---	MmugDNA.145.1.S1_at_---
MmugDNA.14520.1.S1_at	NA	NA	NA	NA	NA	NA	NA	NA	NA	MmugDNA.14520.1.S1_at_NA
MmugDNA.1452.1.S1_at	240361_at	---	---	---	---	---	---	---	---	MmugDNA.1452.1.S1_at_---
MmugDNA.14522.1.S1_at	234583_at	CHL1	Cell adhesion molecule with homology to L1CAM (close homolog of L1)	chr3p26.1	10752	---	---	---	---	MmugDNA.14522.1.S1_at_CHL1
MmugDNA.14523.1.S1_at	1553857_at	IGSF22	immunoglobulin superfamily, member 22	chr11p15.1	283284	IGSF22	immunoglobulin superfamily, member 22	---	703650	MmugDNA.14523.1.S1_at_IGSF22
MmugDNA.14524.1.S1_at	NA	NA	NA	NA	NA	NA	NA	NA	NA	MmugDNA.14524.1.S1_at_NA

MmugDNA.14524.1.S1_x_at	222136_x_at	ZNF43	zinc finger protein 43	chr19p13.1-p12	7594	---	---	---	---	MmugDNA.14524.1.S1_x_at_ZNF43
MmugDNA.14525.1.S1_at	228120_at	---	---	---	---	---	---	---	---	MmugDNA.14525.1.S1_at_---
MmugDNA.14526.1.S1_at	219603_s_at	ZNF226	zinc finger protein 226	chr19q13.2	7769	---	---	---	---	MmugDNA.14526.1.S1_at_ZNF226
MmugDNA.14527.1.S1_at	219305_x_at	FBXO2	F-box protein 2	chr1p36.22	26232	LOC722738	similar to F-box only protein 2	---	722738	MmugDNA.14527.1.S1_at_FBXO2
MmugDNA.14528.1.S1_s_at	211896_s_at	DCN	decorin	chr12q21.33	1634	DCN	decorin	---	709933	MmugDNA.14528.1.S1_s_at_DCN
MmugDNA.14529.1.S1_at	241822_at	LOC388553	similar to hCG1645909	chr19q13.32	388553	---	---	---	---	MmugDNA.14529.1.S1_at_LOC388553
MmugDNA.14530.1.S1_at	40850_at	FKBP8	FK506 binding protein 8, 38kDa	chr19p12	23770	LOC720232	similar to FK506-binding protein 8	---	720232	MmugDNA.14530.1.S1_at_FKBP8
MmugDNA.14531.1.S1_at	NA	NA	NA	NA	NA	NA	NA	NA	NA	MmugDNA.14531.1.S1_at_NA
MmugDNA.1453.1.S1_at	224668_at	SYS1	SYS1 Golgi-localized integral membrane protein homolog (S. cerevisiae)	chr20q13.12	90196	LOC717604	similar to SCF apoptosis response protein 1 isoform 2	---	717604	MmugDNA.1453.1.S1_at_SYS1
MmugDNA.14532.1.S1_at	224198_at	CELA1	chymotrypsin-like elastase family, member 1	chr12q13	1990	HNRPA1	Similar to Heterogeneous nuclear ribonucleoprotein A1 (Helix-destabilizing protein) (Single-strand RN	---	712227	MmugDNA.14532.1.S1_at_CELA1
MmugDNA.14533.1.S1_at	1554808_at	ACP1	acid phosphatase 1, soluble	chr2p25	52	---	---	---	---	MmugDNA.14533.1.S1_at_ACP1
MmugDNA.14534.1.S1_s_at	211417_x_at	GGT1	gamma-glutamyltransferase 1	chr22q11.23	2678	GGT1	gamma-glutamyltransferase 1	---	700936 /// 707545	MmugDNA.14534.1.S1_s_at_GGT1
MmugDNA.14535.1.S1_s_at	202216_x_at	NFYC	nuclear transcription factor Y, gamma	chr1p32|1p32	4802	---	---	---	---	MmugDNA.14535.1.S1_s_at_NFYC
MmugDNA.14536.1.S1_at	232381_s_at	DNAH5	dynein, axonemal, heavy chain 5	chr5p15.2	1767	LOC717339	similar to dynein, axonemal, heavy polypeptide 5	---	717339	MmugDNA.14536.1.S1_at_DNAH5
MmugDNA.14537.1.S1_s_at	208404_x_at	KCNJ5	potassium inwardly-rectifying channel, subfamily J, member 5	chr11q24	3762	---	---	---	---	MmugDNA.14537.1.S1_s_at_KCNJ5
MmugDNA.14538.1.S1_at	235672_at	MAP6	microtubule-associated protein 6	chr11q13.5	4135	LOC696223	similar to microtubule-associated protein 6 isoform 2	---	696223	MmugDNA.14538.1.S1_at_MAP6
MmugDNA.14539.1.S1_at	225924_at	FNIP2	folliculin interacting protein 2	chr4q32.1	57600	---	---	---	---	MmugDNA.14539.1.S1_at_FNIP2
MmugDNA.14540.1.S1_s_at	208716_s_at	TMCO1	transmembrane and coiled-coil domains 1	chr1q22-q25	54499	---	---	---	---	MmugDNA.14540.1.S1_s_at_TMCO1
MmugDNA.14541.1.S1_at	225922_at	FNIP2	folliculin interacting protein 2	chr4q32.1	57600	---	---	---	---	MmugDNA.14541.1.S1_at_FNIP2
MmugDNA.1454.1.S1_at	227421_at	C21orf57	chromosome 21 open reading frame 57	chr21q22.3	54059	LOC721807	similar to formin-like 1	---	721807	MmugDNA.1454.1.S1_at_C21orf57
MmugDNA.14542.1.S1_at	232365_at	SIAH1	seven in absentia homolog 1 (Drosophila)	chr16q12	6477	---	---	---	---	MmugDNA.14542.1.S1_at_SIAH1
MmugDNA.14543.1.S1_at	226884_at	LRRN1	leucine rich repeat neuronal 1	chr3p26.2	57633	LOC722192	Hypothetical protein LOC722192	---	722192	MmugDNA.14543.1.S1_at_LRRN1
MmugDNA.14544.1.S1_at	201212_at	LGMN	legumain	chr14q32.1	5641	LOC703986	Similar to Osteoclast-stimulating factor 1	---	703986	MmugDNA.14544.1.S1_at_LGMN
MmugDNA.14545.1.S1_at	233386_at	---	---	---	---	---	---	---	---	MmugDNA.14545.1.S1_at_---
MmugDNA.14546.1.S1_at	1553483_at	TSGA10IP	testis specific, 10 interacting protein	chr11q13.1	254187	LOC715217	similar to testis specific, 10 interacting protein	---	715217	MmugDNA.14546.1.S1_at_TSGA10IP
MmugDNA.14547.1.S1_at	240845_at	---	---	---	---	---	---	---	---	MmugDNA.14547.1.S1_at_---
MmugDNA.14548.1.S1_at	236840_at	C12orf56	chromosome 12 open reading frame 56	chr12q14.2	115749	LOC717259	hypothetical protein LOC717259	---	717259	MmugDNA.14548.1.S1_at_C12orf56
MmugDNA.14549.1.S1_at	240707_at	---	---	---	---	---	---	---	---	MmugDNA.14549.1.S1_at_---
MmugDNA.14550.1.S1_at	1570140_at	LOC653110	hypothetical LOC653110	chr10q11.22	653110	---	---	---	---	MmugDNA.14550.1.S1_at_LOC653110
MmugDNA.14551.1.S1_at	219505_at	CECR1	cat eye syndrome chromosome region, candidate 1	chr22q11.2	51816	LOC709295	similar to cat eye syndrome critical region protein 1 isoform a precursor	---	709295	MmugDNA.14551.1.S1_at_CECR1
MmugDNA.1455.1.S1_at	1569523_a_at	LOC100132767	hypothetical protein LOC100132767	chr16p11.2	100132767	LOC693865	hypothetical protein LOC693865	---	693865	MmugDNA.1455.1.S1_at_LOC1001327
MmugDNA.14552.1.S1_at	1568938_at	---	---	---	---	---	---	---	---	MmugDNA.14552.1.S1_at_---
MmugDNA.14553.1.S1_at	237917_at	NBPF8	neuroblastoma breakpoint family, member 8	chr1q21.1	728841	---	---	---	---	MmugDNA.14553.1.S1_at_NBPF8
MmugDNA.14554.1.S1_at	238068_at	ARIH2	Ariadne homolog 2 (Drosophila)	chr3p21.2-p21.3	10425	---	---	---	---	MmugDNA.14554.1.S1_at_ARIH2
MmugDNA.14555.1.S1_at	231306_at	LYZL4	lysozyme-like 4	chr3p22.1	131375	LOC716549	similar to lysozyme-like 4	---	716549	MmugDNA.14555.1.S1_at_LYZL4
MmugDNA.14556.1.S1_at	214945_at	FAM153A /// FAM153B	family with sequence similarity 153, member A /// family with sequence similarity 153, member B /// fa	chr5q35.2 /// chr5q35.3	202134 /// 285596 /// 6	---	---	---	---	MmugDNA.14556.1.S1_at_FAM153A ///
MmugDNA.14557.1.S1_at	222573_s_at	SAV1	salvador homolog 1 (Drosophila)	chr14q13-q23	60485	SAV1	salvador homolog 1 (Drosophila)	---	706424	MmugDNA.14557.1.S1_at_SAV1
MmugDNA.14559.1.S1_at	237842_at	MAP7D3	MAP7 domain containing 3	chrXq26.3	79649	---	---	---	---	MmugDNA.14559.1.S1_at_MAP7D3
MmugDNA.14560.1.S1_at	229492_at	VANGL1	vang-like 1 (van gogh, Drosophila)	chr1p11-p13.1	81839	---	---	---	---	MmugDNA.14560.1.S1_at_VANGL1
MmugDNA.14561.1.S1_at	227000_at	C7orf41	chromosome 7 open reading frame 41	chr7p15.1	222166	---	---	---	---	MmugDNA.14561.1.S1_at_C7orf41
MmugDNA.1456.1.S1_at	219873_at	COLEC11	collectin sub-family member 11	chr2p25.3	78989	LOC711529	Hypothetical protein LOC711529	---	711529	MmugDNA.1456.1.S1_at_COLEC11
MmugDNA.14562.1.S1_at	213208_at	KIAA0240	KIAA0240	chr6p21.1	23506	LOC698668	hypothetical protein LOC698668	---	698668	MmugDNA.14562.1.S1_at_KIAA0240
MmugDNA.14563.1.S1_at	208500_x_at	FOXD3	forkhead box D3	chr1p32-p31	27022	FOXD3	forkhead box D3	---	696085	MmugDNA.14563.1.S1_at_FOXD3
MmugDNA.14564.1.S1_at	227086_at	C22orf39	chromosome 22 open reading frame 39	chr22q11.21	128977	---	---	---	---	MmugDNA.14564.1.S1_at_C22orf39
MmugDNA.14565.1.S1_at	208239_at	FOXE1	forkhead box E1 (thyroid transcription factor 2)	chr9q22	2304	LOC715656	similar to forkhead box E1	---	715656	MmugDNA.14565.1.S1_at_FOXE1
MmugDNA.14566.1.S1_at	243381_at	---	---	---	---	---	---	---	---	MmugDNA.14566.1.S1_at_---
MmugDNA.14567.1.S1_at	206468_s_at	METTL13	methyltransferase like 13	chr1q24-q25.3	51603	METTL13	methyltransferase like 13	---	704943	MmugDNA.14567.1.S1_at_METTL13
MmugDNA.14568.1.S1_at	211195_s_at	TP63	tumor protein p63	chr3q28	8626	TP63	tumor protein p63	---	703997	MmugDNA.14568.1.S1_at_TP63
MmugDNA.14569.1.S1_at	1562791_at	MYCBPAP	MYCBP associated protein	chr17q21.33	84073	---	---	---	---	MmugDNA.14569.1.S1_at_MYCBPAP
MmugDNA.14570.1.S1_at	212404_s_at	UBE3B	ubiquitin protein ligase E3B	chr12q24.11	89910	LOC707324	similar to ubiquitin protein ligase E3B isoform a	---	707324	MmugDNA.14570.1.S1_at_UBE3B
MmugDNA.14571.1.S1_s_at	217770_at	PIGT	phosphatidylinositol glycan anchor biosynthesis, class T	chr20q12-q13.12	51604	LOC710556	similar to phosphatidylinositol glycan, class T precursor	---	710556	MmugDNA.14571.1.S1_s_at_PIGT
MmugDNA.1457.1.S1_at	223718_at	ACRBP	acrosin binding protein	chr12p13.31	84519	LOC713219	similar to proacrosin binding protein sp32 precursor	---	713219	MmugDNA.1457.1.S1_at_ACRBP
MmugDNA.14572.1.S1_at	212403_at	UBE3B	ubiquitin protein ligase E3B	chr12q24.11	89910	LOC707324 /// LOC7075	similar to ubiquitin protein ligase E3B isoform a /// hypothetical protein LOC707548	---	707324 /// 707548	MmugDNA.14572.1.S1_at_UBE3B
MmugDNA.14573.1.S1_at	219928_s_at	CABYR	calcium binding tyrosine-(Y)-phosphorylation regulated	chr18q11.2	26256	---	---	---	---	MmugDNA.14573.1.S1_at_CABYR
MmugDNA.14574.1.S1_at	230207_s_at	DOCK5	Dedicator of cytokinesis 5	chr8p21.2	80005	LOC711359	similar to dedicator of cytokinesis 5	---	711359	MmugDNA.14574.1.S1_at_DOCK5
MmugDNA.14575.1.S1_at	230047_at	FLJ32810	Rho-type GTPase-activating protein FLJ32810	chr11q22.1	143872	OPHN1	oligophrenin 1	---	703141	MmugDNA.14575.1.S1_at_FLJ32810
MmugDNA.14576.1.S1_at	1552391_at	C1orf65	chromosome 1 open reading frame 65	chr1q41	164127	LOC703637	hypothetical protein LOC703637	---	703637	MmugDNA.14576.1.S1_at_C1orf65
MmugDNA.14577.1.S1_s_at	210275_s_at	ZFAND5	zinc finger, AN1-type domain 5	chr9q13-q21	7763	ZFAND5	zinc finger, AN1-type domain 5	---	702149	MmugDNA.14577.1.S1_at_ZFAND5
MmugDNA.14578.1.S1_at	213498_at	CREB3L1	cAMP responsive element binding protein 3-like 1	chr11p11.2	90993	---	---	---	---	MmugDNA.14578.1.S1_at_CREB3L1
MmugDNA.14579.1.S1_at	1555352_at	FOXP2	forkhead box P2	chr7q31	93986	---	---	---	---	MmugDNA.14579.1.S1_at_FOXP2
MmugDNA.14580.1.S1_at	233284_at	---	---	---	---	---	---	---	---	MmugDNA.14580.1.S1_at_---
MmugDNA.14581.1.S1_at	230445_at	BTBD17	BTB (POZ) domain containing 17	chr17q25.1	388419	LOC702102	similar to Galectin-3-binding protein precursor (Lectin galactoside-binding soluble 3-binding protein) (I	---	702102	MmugDNA.14581.1.S1_at_BTBD17
MmugDNA.1458.1.S1_at	65086_at	YIPF2	Yip1 domain family, member 2	chr19p13.2	78992	LOC713753	similar to Yip1 domain family, member 2	---	713753	MmugDNA.1458.1.S1_at_YIPF2
MmugDNA.14582.1.S1_x_at	NA	NA	NA	NA	NA	NA	NA	NA	NA	MmugDNA.14582.1.S1_x_at_NA
MmugDNA.14583.1.S1_at	NA	NA	NA	NA	NA	NA	NA	NA	NA	MmugDNA.14583.1.S1_at_NA
MmugDNA.14584.1.S1_at	225969_at	ALKBH6	alkB, alkylation repair homolog 6 (E. coli)	chr19q13.12	84964	LOC719033	similar to calpain, small subunit 1	---	719033	MmugDNA.14584.1.S1_at_ALKBH6
MmugDNA.14585.1.S1_s_at	NA	NA	NA	NA	NA	NA	NA	NA	NA	MmugDNA.14585.1.S1_s_at_NA
MmugDNA.14586.1.S1_s_at	227567_at	LOC651250	hypothetical LOC651250	chr17q24.2	651250	---	---	---	---	MmugDNA.14586.1.S1_s_at_LOC6512
MmugDNA.14588.1.S1_at	209503_s_at	PSMC5	proteasome (prosome, macropain) 26S subunit, ATPase, 5	chr17q23-q25	5705	LOC717437	similar to for proteasomal ATPase (SUG1)	---	717437	MmugDNA.14588.1.S1_at_PSMC5
MmugDNA.14589.1.S1_at	NA	NA	NA	NA	NA	NA	NA	NA	NA	MmugDNA.14589.1.S1_at_NA
MmugDNA.14589.1.S1_s_at	224282_s_at	AGPAT3	1-acylglycerol-3-phosphate O-acyltransferase 3	chr21q22.3	56894	---	---	---	---	MmugDNA.14589.1.S1_s_at_AGPAT3
MmugDNA.14590.1.S1_at	1557472_a_at	FLJ30838	hypothetical gene supported by AL832565	chr2p16.1	400955	---	---	---	---	MmugDNA.14590.1.S1_at_FLJ30838
MmugDNA.14591.1.S1_at	241930_x_at	LOC442113	similar to protein-tyrosine phosphatase	chr4q21.22	442113	---	---	---	---	MmugDNA.14591.1.S1_at_LOC442113
MmugDNA.1459.1.S1_s_at	220305_at	MAVS	mitochondrial antiviral signaling protein	chr20p13	57506	---	---	---	---	MmugDNA.1459.1.S1_s_at_MAVS
MmugDNA.14592.1.S1_at	217981_s_at	FXC1	fracture callus 1 homolog (rat)	chr11p15.5-p15.3	26515	LOC713937	Similar to golgi phosphoprotein 4	---	713937	MmugDNA.14592.1.S1_at_FXC1
MmugDNA.14593.1.S1_at	218665_at	FZD4	frizzled homolog 4 (Drosophila)	chr11q14.2	8322	FZD4	frizzled homolog 4 (Drosophila)	---	704754	MmugDNA.14593.1.S1_at_FZD4
MmugDNA.14594.1.S1_at	230074_s_at	---	---	---	---	---	---	---	---	MmugDNA.14594.1.S1_at_---
MmugDNA.14595.1.S1_at	1563210_at	---	---	---	---	---	---	---	---	MmugDNA.14595.1.S1_at_---
MmugDNA.14596.1.S1_at	235323_at	---	---	---	---	---	---	---	---	MmugDNA.14596.1.S1_at_---
MmugDNA.14597.1.S1_at	207966_s_at	GLG1	golgi apparatus protein 1	chr16q22.3	2734	LOC705546	Similar to basic FGF-repressed Zic binding protein isoform a	---	705546	MmugDNA.14597.1.S1_at_GLG1
MmugDNA.14598.1.S1_s_at	225812_at	C6orf225	chromosome 6 open reading frame 225	chr6q21	619208	LOC722132	Hypothetical protein LOC722132	---	722132	MmugDNA.14598.1.S1_s_at_C6orf225
MmugDNA.14599.1.S1_at	231408_at	TMEM92	transmembrane protein 92	chr17q21.33	162461	LOC701913	hypothetical protein LOC701913	---	701913	MmugDNA.14599.1.S1_at_TMEM92
MmugDNA.14600.1.S1_at	237049_at	---	---	---	---	---	---	---	---	MmugDNA.14600.1.S1_at_---
MmugDNA.14601.1.S1_at	224913_s_at	TIMM50	translocase of inner mitochondrial membrane 50 homolog (S. cerevisiae)	chr19q13.2	92609	LOC697583	similar to translocase of inner mitochondrial membrane 50 homolog	---	697583	MmugDNA.14601.1.S1_at_TIMM50
MmugDNA.1460.1.S1_at	209781_s_at	KHDRBS3	KH domain containing, RNA binding, signal transduction associated 3	chr8q24.2	10656	LOC705416	similar to KH domain containing, RNA binding, signal transduction associated 3	---	705416	MmugDNA.1460.1.S1_at_KHDRBS3
MmugDNA.14602.1.S1_at	228603_at	---	---	---	---	ACTR3	similar to ARP3 actin-related protein 3 homolog	---	709836	MmugDNA.14602.1.S1_at_---
MmugDNA.14603.1.S1_at	214806_at	BICD1	bicaudal D homolog 1 (Drosophila)	chr12p11.2-p11.1	636	---	---	---	---	MmugDNA.14603.1.S1_at_BICD1
MmugDNA.14604.1.S1_s_at	211265_at	PTGER3	prostaglandin E receptor 3 (subtype EP3)	chr1p31.2	5733	---	---	---	---	MmugDNA.14604.1.S1_s_at_PTGER3
MmugDNA.14605.1.S1_at	222630_at	RFX7	regulatory factor X, 7	chr15q21.3	64864	RFX7	regulatory factor X, 7	---	698878	MmugDNA.14605.1.S1_at_RFX7
MmugDNA.14606.1.S1_at	NA	NA	NA	NA	NA	NA	NA	NA	NA	MmugDNA.14606.1.S1_at_NA
MmugDNA.14606.1.S1_s_at	NA	NA	NA	NA	NA	NA	NA	NA	NA	MmugDNA.14606.1.S1_s_at_NA
MmugDNA.14607.1.S1_at	225199_at	---	---	---	---	---	---	---	---	MmugDNA.14607.1.S1_at_---
MmugDNA.14608.1.S1_at	225197_at	---	---	---	---	---	---	---	---	MmugDNA.14608.1.S1_at_---
MmugDNA.14609.1.S1_at	228211_at	C9orf102	chromosome 9 open reading frame 102	chr9q22.32	375748	LOC695752	similar to stretch responsive protein 278 isoform a	---	695752	MmugDNA.14609.1.S1_at_C9orf102
MmugDNA.14610.1.S1_at	230592_at	NSL1	NSL1, MIND kinetochore complex component, homolog (S. cerevisiae)	chr1q41	25936	---	---	---	---	MmugDNA.14610.1.S1_at_NSL1
MmugDNA.14611.1.S1_at	243051_at	CNIH4	Cornichon homolog 4 (Drosophila)	chr1q42.11	29097	---	---	---	---	MmugDNA.14611.1.S1_at_CNIH4
MmugDNA.1461.1.S1_at	1570452_at	---	---	---	---	---	---	---	---	MmugDNA.1461.1.S1_at_---
MmugDNA.14612.1.S1_at	1555910_at	PTCD2	pentatricopeptide repeat domain 2	chr5q13.2	79810	---	---	---	---	MmugDNA.14612.1.S1_at_PTCD2
MmugDNA.14613.1.S1_at	210988_s_at	PRUNE	prune homolog (Drosophila)	chr1q21	58497	LOC708898	similar to prune homolog	---	708898	MmugDNA.14613.1.S1_at_PRUNE
MmugDNA.14614.1.S1_at	215067_x_at	PRDX2	peroxiredoxin 2	chr19p13.2	7001	---	---	---	---	MmugDNA.14614.1.S1_at_PRDX2
MmugDNA.14615.1.S1_s_at	NA	NA	NA	NA	NA	NA	NA	NA	NA	MmugDNA.14615.1.S1_s_at_NA
MmugDNA.14616.1.S1_at	1563495_at	SLC9A11	solute carrier family 9, member 11	chr1q25.1	284525	---	---	---	---	MmugDNA.14616.1.S1_at_SLC9A11
MmugDNA.14617.1.S1_at	NA	NA	NA	NA	NA	NA	NA	NA	NA	MmugDNA.14617.1.S1_at_NA
MmugDNA.14617.1.S1_s_at	214428_x_at	C4A /// C4B	complement component 4A (Rodgers blood group) /// complement component 4B (Chido blood group	chr6p21.3	720 /// 721	LOC723165	similar to complement component 4B preproprotein	---	723165	MmugDNA.14617.1.S1_s_at_C4A /// C4
MmugDNA.14618.1.S1_at	212759_s_at	TCF7L2	transcription factor 7-like 2 (T-cell specific, HMG-box)	chr10q25.3	6934	LOC701759	similar to Transcription factor 7-like 2 (HMG box transcription factor 4) (T-cell-specific transcription fa	---	701759	MmugDNA.14618.1.S1_at_TCF7L2
MmugDNA.14619.1.S1_at	212762_s_at	TCF7L2	transcription factor 7-like 2 (T-cell specific, HMG-box)	chr10q25.3	6934	LOC701759	similar to Transcription factor 7-like 2 (HMG box transcription factor 4) (T-cell-specific transcription fa	---	701759	MmugDNA.14619.1.S1_at_TCF7L2
MmugDNA.146.1.S1_at	1554183_s_at	TRIM73 /// TRIM74	tripartite motif-containing 73 /// tripartite motif-containing 74	chr7q11.23	375593 /// 378108	LOC716264	similar to tripartite motif protein 50A	---	716264	MmugDNA.146.1.S1_at_TRIM73 /// TRI
MmugDNA.14620.1.S1_at	212761_at	TCF7L2	transcription factor 7-like 2 (T-cell specific, HMG-box)	chr10q25.3	6934	LOC701759	similar to Transcription factor 7-like 2 (HMG box transcription factor 4) (T-cell-specific transcription fa	---	701759	MmugDNA.14620.1.S1_at_TCF7L2

MmugDNA.1462.1.S1_at	1570009_at	LOC732096	similar to hCG2040240	chr12q23.1	732096	---	---	---	---	MmugDNA.1462.1.S1_at_LOC732096
MmugDNA.14622.1.S1_at	206060_s_at	PTPN22	protein tyrosine phosphatase, non-receptor type 22 (lymphoid)	chr1p13.3-p13.1	26191	PTPN22	protein tyrosine phosphatase, non-receptor type 22 (lymphoid)	---	707433	MmugDNA.14622.1.S1_at_PTPN22
MmugDNA.14623.1.S1_at	219198_at	GTF3C4	general transcription factor IIIC, polypeptide 4, 90kDa	chr9q34.13	9329	GTF3C4	general transcription factor IIIC, polypeptide 4, 90kDa	---	714849	MmugDNA.14623.1.S1_at_GTF3C4
MmugDNA.14624.1.S1_at	226315_at	ZNF830	zinc finger protein 830	chr17q12	91603	LOC715130	similar to coiled-coil domain containing 16	---	715130	MmugDNA.14624.1.S1_at_ZNF830
MmugDNA.14625.1.S1_at	226323_at	ZNF830	zinc finger protein 830	chr17q12	91603	LOC715130	similar to coiled-coil domain containing 16	---	715130	MmugDNA.14625.1.S1_at_ZNF830
MmugDNA.14626.1.S1_at	215983_s_at	UBXN8	UBX domain protein 8	chr8p12-p11.2	7993	---	---	---	---	MmugDNA.14626.1.S1_at_UBXN8
MmugDNA.14627.1.S1_at	1552643_at	ZNF626	zinc finger protein 626	chr19p12	199777	---	---	---	---	MmugDNA.14627.1.S1_at_ZNF626
MmugDNA.14628.1.S1_at	227750_at	KALRN	kalirin, RhoGEF kinase	chr3q21.1-q21.2	8997	---	---	---	---	MmugDNA.14628.1.S1_at_KALRN
MmugDNA.14629.1.S1_at	236390_at	C20orf94	chromosome 20 open reading frame 94	chr20p12.2	128710	LOC718563	hypothetical protein LOC718563	---	718563	MmugDNA.14629.1.S1_at_C20orf94
MmugDNA.14630.1.S1_at	242456_at	MRE11A	MRE11 meiotic recombination 11 homolog A (S. cerevisiae)	chr11q21	4361	---	---	---	---	MmugDNA.14630.1.S1_at_MRE11A
MmugDNA.14631.1.S1_at	209586_s_at	PRUNE	prune homolog (Drosophila)	chr1q21	58497	LOC708898	similar to prune homolog	---	708898	MmugDNA.14631.1.S1_at_PRUNE
MmugDNA.1463.1.S1_s_at	218714_at	PRR14	proline rich 14	chr16p11.2	78994	LOC712594	hypothetical protein LOC712594	---	712594	MmugDNA.1463.1.S1_s_at_PRR14
MmugDNA.14633.1.S1_at	NA	NA	NA	NA	NA	NA	NA	NA	NA	MmugDNA.14633.1.S1_at_NA
MmugDNA.14634.1.S1_at	209612_s_at	ADH1B	alcohol dehydrogenase 1B (class I), beta polypeptide	chr4q21-q23	125	ADH1A /// ADH1B /// ADI	alcohol dehydrogenase 1A (class I), alpha polypeptide /// alcohol dehydrogenase 1B (class I), beta pc	---	707682 /// 707908 /// 711(	MmugDNA.14634.1.S1_at_ADH1B
MmugDNA.14635.1.S1_at	209613_s_at	ADH1B	alcohol dehydrogenase 1B (class I), beta polypeptide	chr4q21-q23	125	ADH1B	alcohol dehydrogenase 1B (class I), beta polypeptide	---	707908	MmugDNA.14635.1.S1_at_ADH1B
MmugDNA.14636.1.S1_at	217642_at	RNF40	ring finger protein 40	chr16p11.2-p11.1	9810	LOC713148	similar to ring finger protein 40	---	713148	MmugDNA.14636.1.S1_at_RNF40
MmugDNA.14637.1.S1_at	218623_at	HMP19	HMP19 protein	chr5q35.2	51617	HMP19	similar to Neuron-specific protein family member 2 (Protein p19) (8.5 protein)	---	693872	MmugDNA.14637.1.S1_at_HMP19
MmugDNA.14638.1.S1_at	207613_s_at	CAMK2A	calcium/calmodulin-dependent protein kinase II alpha	chr5q33.1	815	LOC710540	similar to calcium/calmodulin-dependent protein kinase IIA isoform 1	---	710540	MmugDNA.14638.1.S1_at_CAMK2A
MmugDNA.14640.1.S1_at	234349_at	SSPO	SCO-spondin homolog (Bos taurus)	chr7q36.1	23145	LOC712419	similar to subcommissural organ spondin	---	712419	MmugDNA.14640.1.S1_at_SSPO
MmugDNA.14641.1.S1_at	237405_at	---	---	---	---	---	---	---	---	MmugDNA.14641.1.S1_at_---
MmugDNA.1464.1.S1_at	225821_s_at	BOD1L	biorientation of chromosomes in cell division 1-like	chr4p16.1	259282	LOC713697	similar to CG5514-PB, isoform B	---	713697	MmugDNA.1464.1.S1_at_BOD1L
MmugDNA.14642.1.S1_at	219878_s_at	KLF13	Kruppel-like factor 13	chr15q12	51621	LOC720644	similar to Krueppel-like factor 13 (Transcription factor BTEB3) (Basic transcription element-binding pr	---	720644	MmugDNA.14642.1.S1_at_KLF13
MmugDNA.14643.1.S1_at	239819_at	---	---	---	---	---	---	---	---	MmugDNA.14643.1.S1_at_---
MmugDNA.14644.1.S1_at	213456_at	SOSTDC1	sclerostin domain containing 1	chr7p21.1	25928	LOC709577	similar to cystine knot-containing secreted protein	---	709577	MmugDNA.14644.1.S1_at_SOSTDC1
MmugDNA.14645.1.S1_at	242394_at	CNKSR3	CNKSR family member 3	chr6q25.2	154043	CNKSR3	CNKSR family member 3	---	702627	MmugDNA.14645.1.S1_at_CNKSR3
MmugDNA.14645.1.S1_s_at	NA	NA	NA	NA	NA	NA	NA	NA	NA	MmugDNA.14645.1.S1_s_at_NA
MmugDNA.14646.1.S1_at	209690_s_at	DOK4	docking protein 4	chr16q13	55715	DOK4	docking protein 4	---	704443	MmugDNA.14646.1.S1_at_DOK4
MmugDNA.14647.1.S1_at	1561469_at	---	---	---	---	---	---	---	---	MmugDNA.14647.1.S1_at_---
MmugDNA.14648.1.S1_at	1553323_a_at	CATSPER2	cation channel, sperm associated 2	chr15q15.3	117155	LOC710876	similar to sperm-associated cation channel 2 isoform 2	---	710876	MmugDNA.14648.1.S1_at_CATSPER2
MmugDNA.14649.1.S1_at	224388_s_at	COL25A1	collagen, type XXV, alpha 1	chr4q25	84570	COL25A1	collagen, type XXV, alpha 1	---	697122	MmugDNA.14649.1.S1_at_COL25A1
MmugDNA.14650.1.S1_at	241191_at	---	---	---	---	---	---	---	---	MmugDNA.14650.1.S1_at_---
MmugDNA.14651.1.S1_at	229228_at	CREB5	cAMP responsive element binding protein 5	chr7p15.1	9586	---	---	---	---	MmugDNA.14651.1.S1_at_CREB5
MmugDNA.1465.1.S1_at	242925_at	RNF148	ring finger protein 148	chr7q31.33	378925	RNF148	ring finger protein 148	---	695090	MmugDNA.1465.1.S1_at_RNF148
MmugDNA.14652.1.S1_at	241880_x_at	---	---	---	---	---	---	---	---	MmugDNA.14652.1.S1_at_---
MmugDNA.14653.1.S1_at	241621_at	SMCHD1	Structural maintenance of chromosomes flexible hinge domain containing 1	chr18p11.32	23347	---	---	---	---	MmugDNA.14653.1.S1_at_SMCHD1
MmugDNA.14654.1.S1_at	226365_at	---	---	---	---	---	---	---	---	MmugDNA.14654.1.S1_at_---
MmugDNA.14655.1.S1_at	226362_at	---	---	---	---	---	---	---	---	MmugDNA.14655.1.S1_at_---
MmugDNA.14656.1.S1_at	206410_at	NR0B2	nuclear receptor subfamily 0, group B, member 2	chr1p36.1	8431	LOC715170	similar to short heterodimer partner	---	715170	MmugDNA.14656.1.S1_at_NR0B2
MmugDNA.14657.1.S1_at	238252_at	---	---	---	---	---	---	---	---	MmugDNA.14657.1.S1_at_---
MmugDNA.14658.1.S1_at	213196_at	ZNF629	zinc finger protein 629	chr16p11.2	23361	LOC713331	similar to zinc finger protein 629	---	713331	MmugDNA.14658.1.S1_at_ZNF629
MmugDNA.14659.1.S1_at	208394_x_at	ESM1	endothelial cell-specific molecule 1	chr5q11.2	11082	ESM1	endothelial cell-specific molecule 1	---	705229	MmugDNA.14659.1.S1_at_ESM1
MmugDNA.14660.1.S1_at	200932_s_at	DCTN2	dynactin 2 (p50)	chr12q13.2-q13.3	10540	LOC694582	Similar to Nonhistone chromosomal protein HMG-17 (High-mobility group nucleosome-binding domai	---	694582	MmugDNA.14660.1.S1_at_DCTN2
MmugDNA.14661.1.S1_at	239283_at	TMED5	transmembrane emp24 protein transport domain containing 5	chr1p31.3	50999	---	---	---	---	MmugDNA.14661.1.S1_at_TMED5
MmugDNA.1466.1.S1_at	1568605_at	JRK	jerky homolog (mouse)	chr8q24.3	8629	---	---	---	---	MmugDNA.1466.1.S1_at_JRK
MmugDNA.14662.1.S1_at	240729_at	C3orf44	chromosome 3 open reading frame 44	chr3q25.1	131831	LOC710313	hypothetical protein LOC710313	---	710313	MmugDNA.14662.1.S1_at_C3orf44
MmugDNA.14663.1.S1_at	241204_at	---	---	---	---	---	---	---	---	MmugDNA.14663.1.S1_at_---
MmugDNA.14664.1.S1_at	204710_s_at	WIPI2	WD repeat domain, phosphoinositide interacting 2	chr7p22.1	26100	LOC702966	hypothetical protein LOC702966	---	702966	MmugDNA.14664.1.S1_at_WIPI2
MmugDNA.14665.1.S1_at	1561424_at	---	---	---	---	---	---	---	---	MmugDNA.14665.1.S1_at_---
MmugDNA.14666.1.S1_at	241288_at	---	---	---	---	---	---	---	---	MmugDNA.14666.1.S1_at_---
MmugDNA.14667.1.S1_at	1562455_at	---	---	---	---	---	---	---	---	MmugDNA.14667.1.S1_at_---
MmugDNA.14669.1.S1_at	1568820_a_at	BEND6	BEN domain containing 6	chr6p12.1	221336	---	---	---	---	MmugDNA.14669.1.S1_at_BEND6
MmugDNA.14670.1.S1_at	210977_s_at	HSF4	heat shock transcription factor 4	chr16q21	3299	HSF4	heat shock transcription factor 4	---	698412	MmugDNA.14670.1.S1_at_HSF4
MmugDNA.14671.1.S1_at	203762_s_at	DYNC2LI1	dynein, cytoplasmic 2, light intermediate chain 1	chr2p25.1-p24.1	51626	LOC716480	similar to dynein 2 light intermediate chain isoform 1	---	716480	MmugDNA.14671.1.S1_at_DYNC2LI1
MmugDNA.1467.1.S1_at	235983_at	---	---	---	---	---	---	---	---	MmugDNA.1467.1.S1_at_---
MmugDNA.14672.1.S1_at	1567591_at	---	---	---	---	---	---	---	---	MmugDNA.14672.1.S1_at_---
MmugDNA.14673.1.S1_at	226043_at	GPSM1	G-protein signaling modulator 1 (AGS3-like, C. elegans)	chr9q34.3	26086	LOC710652	similar to G-protein signalling modulator 1 (AGS3-like, C. elegans)	---	710652	MmugDNA.14673.1.S1_at_GPSM1
MmugDNA.14674.1.S1_at	NA	NA	NA	NA	NA	NA	NA	NA	NA	MmugDNA.14674.1.S1_at_NA
MmugDNA.14674.1.S1_s_at	216900_s_at	CHRNA4	cholinergic receptor, nicotinic, alpha 4	chr20q13.2-q13.3	1137	CHRNA4	cholinergic receptor, nicotinic, alpha 4	---	719678	MmugDNA.14674.1.S1_s_at_CHRNA4
MmugDNA.14675.1.S1_at	235874_at	PRSS35	protease, serine, 35	chr6q14.2	167681	---	---	---	---	MmugDNA.14675.1.S1_at_PRSS35
MmugDNA.14676.1.S1_at	242544_x_at	---	---	---	---	---	---	---	---	MmugDNA.14676.1.S1_at_---
MmugDNA.14677.1.S1_at	NA	NA	NA	NA	NA	NA	NA	NA	NA	MmugDNA.14677.1.S1_at_NA
MmugDNA.14678.1.S1_at	238445_x_at	MGAT5B	mannosyl (alpha-1,6-)-glycoprotein beta-1,6-N-acetyl-glucosaminyltransferase, isozyme B	chr17q25.2	146664	MGAT5	similar to beta(1,6)-N-acetylglucosaminyltransferase V isoform 2	---	708299	MmugDNA.14678.1.S1_at_MGAT5B
MmugDNA.14679.1.S1_at	227637_at	TFCP2	Transcription factor CP2	chr12q13	7024	LOC719124	Similar to integrator complex subunit 5	---	719124	MmugDNA.14679.1.S1_at_TFCP2
MmugDNA.14680.1.S1_at	214521_at	HES2	hairy and enhancer of split 2 (Drosophila)	chr1p36.31-p36.11	54626	---	---	---	---	MmugDNA.14680.1.S1_at_HES2
MmugDNA.14681.1.S1_at	228978_at	LOC645722	hypothetical LOC645722	chr17q23.2	645722	---	---	---	---	MmugDNA.14681.1.S1_at_LOC645722
MmugDNA.1468.1.S1_at	217631_at	GTPBP4 /// IDI2	GTP binding protein 4 /// isopentenyl-diphosphate delta isomerase 2	chr10p15-p14 /// chr10p15.3	23560 /// 91734	---	---	---	---	MmugDNA.1468.1.S1_at_GTPBP4 /// ID
MmugDNA.14682.1.S1_at	222134_at	DDO	D-aspartate oxidase	chr6q21	8528	---	---	---	---	MmugDNA.14682.1.S1_at_DDO
MmugDNA.14683.1.S1_at	1563115_at	---	---	---	---	---	---	---	---	MmugDNA.14683.1.S1_at_---
MmugDNA.14684.1.S1_at	203852_s_at	SMN1 /// SMN2	survival of motor neuron 1, telomeric /// survival of motor neuron 2, centromeric	chr5q13	6606 /// 6607	LOC708538	similar to survival of motor neuron 2, centromeric isoform a	---	708538	MmugDNA.14684.1.S1_at_SMN1 /// SM
MmugDNA.14685.1.S1_at	242861_at	---	---	---	---	---	---	---	---	MmugDNA.14685.1.S1_at_---
MmugDNA.14686.1.S1_s_at	204246_s_at	DCTN3	dynactin 3 (p22)	chr9p13	11258	DCTN3	dynactin 3 (p22)	---	700703	MmugDNA.14686.1.S1_s_at_DCTN3
MmugDNA.14687.1.S1_at	215515_at	---	---	---	---	---	---	---	---	MmugDNA.14687.1.S1_at_---
MmugDNA.14688.1.S1_at	213089_at	LOC100272216	hypothetical LOC100272216	---	100272216	---	---	---	---	MmugDNA.14688.1.S1_at_LOC100272:
MmugDNA.14689.1.S1_at	233693_at	C1orf201	chromosome 1 open reading frame 201	chr1p36.11	90529	---	---	---	---	MmugDNA.14689.1.S1_at_C1orf201
MmugDNA.14690.1.S1_at	222872_x_at	OBFC2A	oligonucleotide/oligosaccharide-binding fold containing 2A	chr2q32.3	64859	OBFC2A	oligonucleotide/oligosaccharide-binding fold containing 2A	---	694172	MmugDNA.14690.1.S1_at_OBFC2A
MmugDNA.14690.1.S1_s_at	233085_s_at	OBFC2A	oligonucleotide/oligosaccharide-binding fold containing 2A	chr2q32.3	64859	OBFC2A	oligonucleotide/oligosaccharide-binding fold containing 2A	---	694172	MmugDNA.14690.1.S1_s_at_OBFC2A
MmugDNA.14691.1.S1_at	213415_at	CLIC2	chloride intracellular channel 2	chrXq28	1193	CLIC2	chloride intracellular channel 2	---	703475	MmugDNA.14691.1.S1_at_CLIC2
MmugDNA.1469.1.S1_at	241218_at	---	---	---	---	LOC698240	hypothetical protein LOC698240	---	698240	MmugDNA.1469.1.S1_at_---
MmugDNA.14692.1.S1_at	NA	NA	NA	NA	NA	NA	NA	NA	NA	MmugDNA.14692.1.S1_at_NA
MmugDNA.14692.1.S1_s_at	212968_at	RFNG	RFNG O-fucosylpeptide 3-beta-N-acetylglucosaminyltransferase	chr17q25	5986	LOC719330	similar to radical fringe homolog	---	719330	MmugDNA.14692.1.S1_s_at_RFNG
MmugDNA.14693.1.S1_at	214545_s_at	PROSC	proline synthetase co-transcribed homolog (bacterial)	chr8p11.2	11212	PROSC	proline synthetase co-transcribed homolog	---	698507	MmugDNA.14693.1.S1_at_PROSC
MmugDNA.14695.1.S1_at	227149_at	TNRC6C	trinucleotide repeat containing 6C	chr17q25.3	57690	LOC714829	similar to trinucleotide repeat containing 6C	---	714829	MmugDNA.14695.1.S1_at_TNRC6C
MmugDNA.14696.1.S1_at	211977_at	GPR107	G protein-coupled receptor 107	chr9q34.11	57720	GPR107	G protein-coupled receptor 107	---	716428	MmugDNA.14696.1.S1_at_GPR107
MmugDNA.14697.1.S1_at	202455_at	HDAC5	histone deacetylase 5	chr17q21	10014	LOC711456	similar to Histone deacetylase 5 (HD5) (Antigen NY-CO-9)	---	711456	MmugDNA.14697.1.S1_at_HDAC5
MmugDNA.14698.1.S1_at	211979_at	GPR107	G protein-coupled receptor 107	chr9q34.11	57720	---	---	---	---	MmugDNA.14698.1.S1_at_GPR107
MmugDNA.14699.1.S1_at	232230_at	C10orf75	Chromosome 10 open reading frame 75	chr10q24.31	90271	---	---	---	---	MmugDNA.14699.1.S1_at_C10orf75
MmugDNA.14700.1.S1_at	229175_at	SMYD4	SET and MYND domain containing 4	chr17p13.3	114826	---	---	---	---	MmugDNA.14700.1.S1_at_SMYD4
MmugDNA.14701.1.S1_at	NA	NA	NA	NA	NA	NA	NA	NA	NA	MmugDNA.14701.1.S1_at_NA
MmugDNA.1470.1.S1_at	1554554_at	CCDC57	coiled-coil domain containing 57	chr17q25.3	284001	LOC719405	similar to coiled-coil domain containing 57	---	719405	MmugDNA.1470.1.S1_at_CCDC57
MmugDNA.14702.1.S1_at	217937_s_at	HDAC7	histone deacetylase 7	chr12q13.1	51564	---	---	---	---	MmugDNA.14702.1.S1_at_HDAC7
MmugDNA.14703.1.S1_at	232501_at	---	---	---	---	---	---	---	---	MmugDNA.14703.1.S1_at_---
MmugDNA.14704.1.S1_at	202582_s_at	RANBP9	RAN binding protein 9	chr6p23	10048	LOC706982	similar to RAN binding protein 9	---	706982	MmugDNA.14704.1.S1_at_RANBP9
MmugDNA.14705.1.S1_at	1570052_at	MIPOL1	mirror-image polydactyly 1	chr14q13.3-q21.1	145282	MIPOL1	mirror-image polydactyly 1	---	697617	MmugDNA.14705.1.S1_at_MIPOL1
MmugDNA.14706.1.S1_at	230622_at	MLLT4	myeloid/lymphoid or mixed-lineage leukemia (trithorax homolog, Drosophila); translocated to, 4	chr6q27	4301	MLLT4	myeloid/lymphoid or mixed-lineage leukemia (trithorax homolog, Drosophila); translocated to, 4	---	693560	MmugDNA.14706.1.S1_at_MLLT4
MmugDNA.14707.1.S1_at	NA	NA	NA	NA	NA	NA	NA	NA	NA	MmugDNA.14707.1.S1_at_NA
MmugDNA.14708.1.S1_at	NA	NA	NA	NA	NA	NA	NA	NA	NA	MmugDNA.14708.1.S1_at_NA
MmugDNA.14709.1.S1_at	244611_at	MED13	Mediator complex subunit 13	chr17q22-q23	9969	MED13	mediator complex subunit 13	---	712277	MmugDNA.14709.1.S1_at_MED13
MmugDNA.14710.1.S1_at	210079_x_at	KCNAB1	potassium voltage-gated channel, shaker-related subfamily, beta member 1	chr3q26.1	7881	KCNAB1	Potassium voltage-gated channel, shaker-related subfamily, beta member 1	---	706635	MmugDNA.14710.1.S1_at_KCNAB1
MmugDNA.14711.1.S1_at	210402_at	KCNJ1	potassium inwardly-rectifying channel, subfamily J, member 1	chr11q24	3758	KCNJ1	potassium inwardly-rectifying channel, subfamily J, member 1	---	714848	MmugDNA.14711.1.S1_at_KCNJ1
MmugDNA.1471.1.S1_at	1559272_at	EXOC3L	exocyst complex component 3-like	chr16q22.1	283849	EXOC3L	exocyst complex component 3-like	---	696499	MmugDNA.1471.1.S1_at_EXOC3L
MmugDNA.14712.1.S1_at	226627_at	SEPT8	septin 8	chr5q31	23176	---	---	---	---	MmugDNA.14712.1.S1_at_SEPT8
MmugDNA.14714.1.S1_at	237171_at	---	---	---	---	---	---	---	---	MmugDNA.14714.1.S1_at_---
MmugDNA.14715.1.S1_s_at	1569057_s_at	MIA3	melanoma inhibitory activity family, member 3	chr1q41	375056	MIA3	melanoma inhibitory activity family, member 3	---	704405	MmugDNA.14715.1.S1_s_at_MIA3
MmugDNA.14716.1.S1_at	1569146_s_at	C6orf174	chromosome 6 open reading frame 174	chr6q22.33	387104	---	---	---	---	MmugDNA.14716.1.S1_at_C6orf174
MmugDNA.14717.1.S1_at	229074_at	---	---	---	---	---	---	---	---	MmugDNA.14717.1.S1_at_---
MmugDNA.14718.1.S1_at	1564402_at	LOC146795	hypothetical protein LOC146795	chr17q24.3	146795	---	---	---	---	MmugDNA.14718.1.S1_at_LOC146795
MmugDNA.14719.1.S1_at	233783_at	---	---	---	---	---	---	---	---	MmugDNA.14719.1.S1_at_---
MmugDNA.147.1.S1_at	237054_at	ENPP5	ectonucleotide pyrophosphatase/phosphodiesterase 5 (putative function)	chr6p21.1-p11.2	59084	---	---	---	---	MmugDNA.147.1.S1_at_ENPP5

MmugDNA.14720.1.S1_at	223810_at	KLHL1	kelch-like 1 (Drosophila)	chr13q21	57626	KLHL1	kelch-like 1 (Drosophila)	---	708779	MmugDNA.14720.1.S1_at_KLHL1
MmugDNA.14721.1.S1_at	225868_at	TRIM47	tripartite motif-containing 47	chr17q24-q25	91107	TRIM47	tripartite motif-containing 47	---	704642	MmugDNA.14721.1.S1_at_TRIM47
MmugDNA.1472.1.S1_at	NA	NA	NA	NA	NA	NA	NA	NA	NA	MmugDNA.1472.1.S1_at_NA
MmugDNA.1472.1.S1_s_at	227758_at	RERG	RAS-like, estrogen-regulated, growth inhibitor	chr12p12.3	85004	RERG	RAS-like, estrogen-regulated, growth inhibitor	---	700567	MmugDNA.1472.1.S1_s_at_RERG
MmugDNA.14722.1.S1_at	213395_at	MLC1	megalencephalic leukoencephalopathy with subcortical cysts 1	chr22q13.33	23209	PLXNB2	Plexin B2	---	720646	MmugDNA.14722.1.S1_at_MLC1
MmugDNA.14723.1.S1_at	229435_at	GLIS3	GLIS family zinc finger 3	chr9p24.2	169792	GLIS3	GLIS family zinc finger 3	---	694900	MmugDNA.14723.1.S1_at_GLIS3
MmugDNA.14724.1.S1_at	1555294_a_at	ERC1	ELKS/RAB6-interacting/CAST family member 1	chr12p13.3	23085	LOC707010	similar to RAB6-interacting protein 2 isoform delta	---	707010	MmugDNA.14724.1.S1_at_ERC1
MmugDNA.14725.1.S1_at	1557143_at	CSMD2	CUB and Sushi multiple domains 2	chr1p35.1-p34.3	114784	---	---	---	---	MmugDNA.14725.1.S1_at_CSMD2
MmugDNA.14726.1.S1_at	207848_at	AVP	arginine vasopressin	chr20p13	551	AVP	arginine vasopressin	---	717101	MmugDNA.14726.1.S1_at_AVP
MmugDNA.14727.1.S1_at	1563586_at	LOC401324	acyloxyacyl hydrolase (neutrophil)	chr7p14.2	401324	---	---	---	---	MmugDNA.14727.1.S1_at_LOC401324
MmugDNA.14728.1.S1_at	1556844_at	LOC100132078	hypothetical protein LOC100132078	chr11q23.1	100132078	---	---	---	---	MmugDNA.14728.1.S1_at_LOC100132(
MmugDNA.14729.1.S1_at	203904_x_at	CD82	CD82 molecule	chr11p11.2	3732	CD82	CD82 molecule	---	715606	MmugDNA.14729.1.S1_at_CD82
MmugDNA.14730.1.S1_at	221384_at	UCP1	uncoupling protein 1 (mitochondrial, proton carrier)	chr4q28-q31	7350	UCP1	uncoupling protein 1	---	574124	MmugDNA.14730.1.S1_at_UCP1
MmugDNA.14731.1.S1_at	234915_s_at	DENR	density-regulated protein	chr12q24.31	8562	---	---	---	---	MmugDNA.14731.1.S1_at_DENR
MmugDNA.1473.1.S1_s_at	234679_at	KRTAP9-3	keratin associated protein 9-3	chr17q12-q21	83900	LOC697700 /// LOC7026	similar to Keratin-associated protein 9-8 (Keratin-associated protein 9.8) (Ultrahigh sulfur keratin-ass	---	697700 /// 702672 /// 705(	MmugDNA.1473.1.S1_s_at_KRTAP9-3
MmugDNA.14732.1.S1_at	223327_x_at	LOC727751 /// LOC727	similar to cis-Golgi matrix protein GM130 /// similar to cis-Golgi matrix protein GM130 /// hypothetical	chr15q25.2	727751 /// 727849 /// 8	---	---	---	---	MmugDNA.14732.1.S1_at_LOC727751
MmugDNA.14733.1.S1_at	213533_at	D4S234E	DNA segment on chromosome 4 (unique) 234 expressed sequence	chr4p16.3	27065	D4S234E	similar to Neuron-specific protein family member 1 (Brain neuron cytoplasmic protein 1) (D4S234)	---	712289	MmugDNA.14733.1.S1_at_D4S234E
MmugDNA.14734.1.S1_at	221471_at	SERINC3	serine incorporator 3	chr20q13.1-q13.3	10955	PRKX	Protein kinase, X-linked	---	706432	MmugDNA.14734.1.S1_at_SERINC3
MmugDNA.14735.1.S1_at	221472_at	SERINC3	serine incorporator 3	chr20q13.1-q13.3	10955	---	---	---	---	MmugDNA.14735.1.S1_at_SERINC3
MmugDNA.14736.1.S1_at	NA	NA	NA	NA	NA	NA	NA	NA	NA	MmugDNA.14736.1.S1_at_NA
MmugDNA.14737.1.S1_at	213109_at	TNIK	TRAF2 and NCK interacting kinase	chr3q26.2-q26.31	23043	TNIK	TRAF2 and NCK interacting kinase	---	695414	MmugDNA.14737.1.S1_at_TNIK
MmugDNA.14738.1.S1_at	213107_at	TNIK	TRAF2 and NCK interacting kinase	chr3q26.2-q26.31	23043	TNIK	TRAF2 and NCK interacting kinase	---	695414	MmugDNA.14738.1.S1_at_TNIK
MmugDNA.14739.1.S1_at	221939_at	YIPF2	Yip1 domain family, member 2	chr19p13.2	78992	LOC713753	similar to Yip1 domain family, member 2	---	713753	MmugDNA.14739.1.S1_at_YIPF2
MmugDNA.14740.1.S1_at	242174_at	---	---	---	---	---	---	---	---	MmugDNA.14740.1.S1_at_---
MmugDNA.14741.1.S1_at	238658_at	---	---	---	---	---	---	---	---	MmugDNA.14741.1.S1_at_---
MmugDNA.1474.1.S1_at	232778_at	---	---	---	---	---	---	---	---	MmugDNA.1474.1.S1_at_---
MmugDNA.14742.1.S1_at	217581_at	---	---	---	---	---	---	---	---	MmugDNA.14742.1.S1_at_---
MmugDNA.14743.1.S1_at	219827_at	UCP3	uncoupling protein 3 (mitochondrial, proton carrier)	chr11q13	7352	---	---	---	---	MmugDNA.14743.1.S1_at_UCP3
MmugDNA.14744.1.S1_at	NA	NA	NA	NA	NA	NA	NA	NA	NA	MmugDNA.14744.1.S1_at_NA
MmugDNA.14745.1.S1_at	227432_s_at	---	---	---	---	---	---	---	---	MmugDNA.14745.1.S1_at_---
MmugDNA.14746.1.S1_at	1556001_at	LOC284939	hypothetical protein LOC284939	chr22q13.33	284939	---	---	---	---	MmugDNA.14746.1.S1_at_LOC284939
MmugDNA.14747.1.S1_at	1564338_at	---	---	---	---	---	---	---	---	MmugDNA.14747.1.S1_at_---
MmugDNA.14748.1.S1_at	217695_x_at	---	---	---	---	---	---	---	---	MmugDNA.14748.1.S1_at_---
MmugDNA.14749.1.S1_at	233024_at	---	---	---	---	---	---	---	---	MmugDNA.14749.1.S1_at_---
MmugDNA.14750.1.S1_at	1553464_at	FLJ40288	hypothetical FLJ40288	chr7q32.3	286023	---	---	---	---	MmugDNA.14750.1.S1_at_FLJ40288
MmugDNA.14751.1.S1_at	223217_s_at	NFKBIZ	nuclear factor of kappa light polypeptide gene enhancer in B-cells inhibitor, zeta	chr3p12-q12	64332	NFKBIZ	nuclear factor of kappa light polypeptide gene enhancer in B-cells inhibitor, zeta	---	702846	MmugDNA.14751.1.S1_at_NFKBIZ
MmugDNA.1475.1.S1_at	225002_s_at	SUMF2	sulfatase modifying factor 2	chr7q11.1	25870	---	---	---	---	MmugDNA.1475.1.S1_at_SUMF2
MmugDNA.14752.1.S1_at	1566842_at	---	---	---	---	---	---	---	---	MmugDNA.14752.1.S1_at_---
MmugDNA.14753.1.S1_at	203173_s_at	C16orf62	chromosome 16 open reading frame 62	chr16p12.3	57020	LOC695027	similar to CG8202-PA	---	695027	MmugDNA.14753.1.S1_at_C16orf62
MmugDNA.14754.1.S1_at	1564064_a_at	ATP11B	ATPase, class VI, type 11B	chr3q27	23200	ATP11B	ATPase, Class VI, type 11B	---	708453	MmugDNA.14754.1.S1_at_ATP11B
MmugDNA.14755.1.S1_at	1566885_at	---	---	---	---	---	---	---	---	MmugDNA.14755.1.S1_at_---
MmugDNA.14756.1.S1_at	231923_at	FLJ12993	hypothetical LOC441027	chr4q21.22	441027	LOC694136	hypothetical protein LOC694136	---	694136	MmugDNA.14756.1.S1_at_FLJ12993
MmugDNA.14757.1.S1_at	215553_x_at	---	---	---	---	---	---	---	---	MmugDNA.14757.1.S1_at_---
MmugDNA.14758.1.S1_at	236017_at	CDKL3	cyclin-dependent kinase-like 3	chr5q31	51265	CDKL3	cyclin-dependent kinase-like 3	---	710586	MmugDNA.14758.1.S1_at_CDKL3
MmugDNA.14759.1.S1_at	NA	NA	NA	NA	NA	NA	NA	NA	NA	MmugDNA.14759.1.S1_at_NA
MmugDNA.14759.1.S1_s_at	202836_s_at	TXNL4A	thioredoxin-like 4A	chr18q23	10907	TXNL4A	thioredoxin-like 4A	---	698969	MmugDNA.14759.1.S1_s_at_TXNL4A
MmugDNA.14760.1.S1_at	241049_at	GRM7	glutamate receptor, metabotropic 7	chr3p26.1-p25.1	2917	ETV3	Similar to ETS translocation variant 3 (ETS-domain transcriptional repressor PE1) (PE-1) (Mitogenic	---	719741	MmugDNA.14760.1.S1_at_GRM7
MmugDNA.14761.1.S1_at	242767_at	LMCD1	LIM and cysteine-rich domains 1	chr3p26-p24	29995	---	---	---	---	MmugDNA.14761.1.S1_at_LMCD1
MmugDNA.1476.1.S1_at	1558590_at	LOC731602 /// METT11	similar to methyltransferase 11 domain containing 1 isoform 2 /// methyltransferase 11 domain contai	chr14q11.2	64745 /// 731602	LOC711935	Similar to Breakpoint cluster region protein (NY-REN-26 antigen)	---	711935	MmugDNA.1476.1.S1_at_LOC731602 //
MmugDNA.14762.1.S1_at	207315_at	CD226	CD226 molecule	chr18q22.3	10666	---	---	---	---	MmugDNA.14762.1.S1_at_CD226
MmugDNA.14763.1.S1_at	240681_at	---	---	---	---	---	---	---	---	MmugDNA.14763.1.S1_at_---
MmugDNA.14764.1.S1_at	222300_at	---	---	---	---	---	---	---	---	MmugDNA.14764.1.S1_at_---
MmugDNA.14765.1.S1_at	208789_at	PTRF	polymerase I and transcript release factor	chr17q21.31	284119	---	---	---	---	MmugDNA.14765.1.S1_at_PTRF
MmugDNA.14766.1.S1_at	226864_at	PKIA	Protein kinase (cAMP-dependent, catalytic) inhibitor alpha	chr8q21.12	5569	LOC698509	similar to cAMP-dependent protein kinase inhibitor alpha isoform 7	---	698509	MmugDNA.14766.1.S1_at_PKIA
MmugDNA.14767.1.S1_at	230964_at	FREM2	FRAS1 related extracellular matrix protein 2	chr13q13.3	341640	FREM2	FRAS1 related extracellular matrix protein 2	---	696113	MmugDNA.14767.1.S1_at_FREM2
MmugDNA.14768.1.S1_at	217107_at	---	---	---	---	LOC715988	similar to 40S ribosomal protein S4, X isoform	---	715988	MmugDNA.14768.1.S1_at_---
MmugDNA.14769.1.S1_at	219439_at	C1GALT1	core 1 synthase, glycoprotein-N-acetylgalactosamine 3-beta-galactosyltransferase, 1	chr7p14-p13	56913	LOC707587	similar to core 1 synthase, glycoprotein-N-acetylgalactosamine 3-beta-galactosyltransferase, 1	---	707587	MmugDNA.14769.1.S1_at_C1GALT1
MmugDNA.14770.1.S1_at	221211_s_at	C21orf7	chromosome 21 open reading frame 7	chr21q22.3	56911	LOC713717	similar to TAK1-like protein	---	713717	MmugDNA.14770.1.S1_at_C21orf7
MmugDNA.14771.1.S1_s_at	241523_at	---	---	---	---	---	---	---	---	MmugDNA.14771.1.S1_s_at_---
MmugDNA.1477.1.S1_at	219879_s_at	C17orf53	chromosome 17 open reading frame 53	chr17q21.31	78995	LOC711524	similar to ankyrin repeat and SOCS box-containing 16	---	711524	MmugDNA.1477.1.S1_at_C17orf53
MmugDNA.1477.1.S1_s_at	NA	NA	NA	NA	NA	NA	NA	NA	NA	MmugDNA.1477.1.S1_s_at_NA
MmugDNA.14772.1.S1_at	218541_s_at	C8orf4	chromosome 8 open reading frame 4	chr8p11.2	56892	C8H8orf4	chromosome 8 open reading frame 4 ortholog	---	702517	MmugDNA.14772.1.S1_at_C8orf4
MmugDNA.14773.1.S1_at	225712_at	GEMIN5	gem (nuclear organelle) associated protein 5	chr5q33.2	25929	LOC714864	similar to gemin 5	---	714864	MmugDNA.14773.1.S1_at_GEMIN5
MmugDNA.14774.1.S1_at	236770_at	SUGT1L1	SGT1, suppressor of G2 allele of SKP1 like 1 (S. cerevisiae)	chr13q14.11	283507	---	---	---	---	MmugDNA.14774.1.S1_at_SUGT1L1
MmugDNA.14775.1.S1_at	237895_at	---	---	---	---	---	---	---	---	MmugDNA.14775.1.S1_at_---
MmugDNA.14776.1.S1_at	214437_s_at	SHMT2	serine hydroxymethyltransferase 2 (mitochondrial)	chr12q12-q14	6472	LOC714433	similar to serine hydroxymethyltransferase 2 (mitochondrial)	---	714433	MmugDNA.14776.1.S1_at_SHMT2
MmugDNA.14777.1.S1_at	217554_at	---	---	---	---	---	---	---	---	MmugDNA.14777.1.S1_at_---
MmugDNA.14778.1.S1_at	204283_at	FARS2	phenylalanyl-tRNA synthetase 2, mitochondrial	chr6p25.1	10667	FARS2	phenylalanyl-tRNA synthetase 2, mitochondrial	---	710945	MmugDNA.14778.1.S1_at_FARS2
MmugDNA.14779.1.S1_at	232295_at	GFM1	G elongation factor, mitochondrial 1	chr3q25.1-q26.2	85476	GFM1	G elongation factor, mitochondrial 1	---	706456	MmugDNA.14779.1.S1_at_GFM1
MmugDNA.14780.1.S1_at	1555520_at	PTCH1	patched homolog 1 (Drosophila)	chr9q22.3	5727	LOC718751	similar to patched	---	718751	MmugDNA.14780.1.S1_at_PTCH1
MmugDNA.14781.1.S1_at	237201_at	---	---	---	---	---	---	---	---	MmugDNA.14781.1.S1_at_---
MmugDNA.1478.1.S1_at	1554216_at	CCDC132	coiled-coil domain containing 132	chr7q21.3	55610	---	---	---	---	MmugDNA.1478.1.S1_at_CCDC132
MmugDNA.14782.1.S1_at	235554_x_at	PACRGL	PARK2 co-regulated-like	chr4p15.31	133015	LOC715522	similar to CG15120-PA	---	715522	MmugDNA.14782.1.S1_at_PACRGL
MmugDNA.14783.1.S1_s_at	1561286_a_at	DIP2A	DIP2 disco-interacting protein 2 homolog A (Drosophila)	chr21q22.3	23181	DIP2A	DIP2-like protein	---	721788	MmugDNA.14783.1.S1_s_at_DIP2A
MmugDNA.14784.1.S1_at	243564_at	PDE1C	phosphodiesterase 1C, calmodulin-dependent 70kDa	chr7p15.1-p14.3	5137	---	---	---	---	MmugDNA.14784.1.S1_at_PDE1C
MmugDNA.14785.1.S1_at	1564212_at	---	---	---	---	---	---	---	---	MmugDNA.14785.1.S1_at_---
MmugDNA.14786.1.S1_at	236176_at	LOC645757	hypothetical LOC645757	chr1p32.1	645757	---	---	---	---	MmugDNA.14786.1.S1_at_LOC645757
MmugDNA.14787.1.S1_at	226141_at	CCDC149	coiled-coil domain containing 149	chr4p15.2	91050	LOC715968	similar to CG14868-PA	---	715968	MmugDNA.14787.1.S1_at_CCDC149
MmugDNA.14788.1.S1_at	226139_at	---	---	---	---	LOC715968	similar to CG14868-PA	---	715968	MmugDNA.14788.1.S1_at_---
MmugDNA.14789.1.S1_at	243615_at	---	---	---	---	---	---	---	---	MmugDNA.14789.1.S1_at_---
MmugDNA.14790.1.S1_at	230247_at	---	---	---	---	---	---	---	---	MmugDNA.14790.1.S1_at_---
MmugDNA.14791.1.S1_at	220031_at	OTUD7B	OTU domain containing 7B	chr1q21.2	56957	OTUD7B	OTU domain containing 7B	---	705774	MmugDNA.14791.1.S1_at_OTUD7B
MmugDNA.1479.1.S1_s_at	223289_s_at	USP38	ubiquitin specific peptidase 38	---	84640	USP38	ubiquitin specific peptidase 38	---	700235	MmugDNA.1479.1.S1_s_at_USP38
MmugDNA.14792.1.S1_at	1563797_at	---	---	---	---	---	---	---	---	MmugDNA.14792.1.S1_at_---
MmugDNA.14793.1.S1_at	1561146_at	VPS35	vacuolar protein sorting 35 homolog (S. cerevisiae)	chr16q12	55737	---	---	---	---	MmugDNA.14793.1.S1_at_VPS35
MmugDNA.14794.1.S1_at	1570212_a_at	LOC253724	Grp94 neighboring nucleotidase pseudogene	chr12q23.3	253724	LOC699683	similar to Grp94 neighboring nucleotidase isoform 3	---	699683	MmugDNA.14794.1.S1_at_LOC253724
MmugDNA.14795.1.S1_at	1563332_at	---	---	---	---	---	---	---	---	MmugDNA.14795.1.S1_at_---
MmugDNA.14796.1.S1_at	242013_at	BCL2L15	BCL2-like 15	chr1p13.2	440603	---	---	---	---	MmugDNA.14796.1.S1_at_BCL2L15
MmugDNA.14797.1.S1_at	240697_at	---	---	---	---	---	---	---	---	MmugDNA.14797.1.S1_at_---
MmugDNA.14798.1.S1_at	1561918_at	---	---	---	---	---	---	---	---	MmugDNA.14798.1.S1_at_---
MmugDNA.14799.1.S1_at	238664_s_at	MGC12916	hypothetical protein MGC12916	chr17p11.2	84815	LOC718334	hypothetical protein LOC718334	---	718334	MmugDNA.14799.1.S1_at_MGC12916
MmugDNA.14800.1.S1_s_at	202676_x_at	FASTK	Fas-activated serine/threonine kinase	chr7q35	10922	FASTK	Fas-activated serine/threonine kinase	---	714020	MmugDNA.14800.1.S1_s_at_FASTK
MmugDNA.14801.1.S1_at	235171_at	---	---	---	---	---	---	---	---	MmugDNA.14801.1.S1_at_---
MmugDNA.1480.1.S1_at	234071_at	DEPDC6	DEP domain containing 6	chr8q24.12	64798	---	---	---	---	MmugDNA.1480.1.S1_at_DEPDC6
MmugDNA.14802.1.S1_at	219840_s_at	TCL6	T-cell leukemia/lymphoma 6	chr14q32.1	27004	LOC710982	similar to T-cell leukemia/lymphoma 6 isoform TCL6a2	---	710982	MmugDNA.14802.1.S1_at_TCL6
MmugDNA.14803.1.S1_s_at	207501_s_at	FGF12	fibroblast growth factor 12	chr3q28	2257	LOC705813	similar to Fibroblast growth factor 12 (FGF-12) (Fibroblast growth factor homologous factor 1) (FHF-1	---	705813	MmugDNA.14803.1.S1_s_at_FGF12
MmugDNA.14804.1.S1_at	33148_at	ZFR	zinc finger RNA binding protein	chr5p13.3	51663	LOC696882	similar to zinc finger RNA binding protein	---	696882	MmugDNA.14804.1.S1_at_ZFR
MmugDNA.14805.1.S1_at	228376_at	GGTA1	glycoprotein, alpha-galactosyltransferase 1	chr9q33.2-q34.11	2681	---	---	---	---	MmugDNA.14805.1.S1_at_GGTA1
MmugDNA.14806.1.S1_at	243730_at	---	---	---	---	---	---	---	---	MmugDNA.14806.1.S1_at_---
MmugDNA.14807.1.S1_at	221624_at	TCL6	T-cell leukemia/lymphoma 6	chr14q32.1	27004	---	---	---	---	MmugDNA.14807.1.S1_at_TCL6
MmugDNA.14808.1.S1_at	224358_s_at	MS4A7	membrane-spanning 4-domains, subfamily A, member 7	chr11q12	58475	MS4A7	membrane-spanning 4-domains, subfamily A, member 7	---	697200	MmugDNA.14808.1.S1_at_MS4A7
MmugDNA.14809.1.S1_at	237602_at	---	---	---	---	---	---	---	---	MmugDNA.14809.1.S1_at_---
MmugDNA.14810.1.S1_at	242497_at	TRAFD1	TRAF-type zinc finger domain containing 1	chr12q	10906	LOC711784	Similar to aconitase 2, mitochondrial	---	711784	MmugDNA.14810.1.S1_at_TRAFD1
MmugDNA.14811.1.S1_at	237884_x_at	TRPM7	transient receptor potential cation channel, subfamily M, member 7	chr15q21	54822	---	---	---	---	MmugDNA.14811.1.S1_at_TRPM7
MmugDNA.1481.1.S1_at	219668_at	GDAP1L1	ganglioside-induced differentiation-associated protein 1-like 1	chr20q12	78997	LOC696811	Similar to zinc finger protein 341	---	696811	MmugDNA.1481.1.S1_at_GDAP1L1
MmugDNA.14812.1.S1_at	226738_at	WDR81	WD repeat domain 81	chr17p13.3	124997	LOC721254	similar to alpha-2-plasmin inhibitor	---	721254	MmugDNA.14812.1.S1_at_WDR81
MmugDNA.14813.1.S1_at	241042_at	---	---	---	---	---	---	---	---	MmugDNA.14813.1.S1_at_---
MmugDNA.14814.1.S1_at	1560290_at	LOC100129895	Hypothetical protein LOC100129895	chr16p13.2	100129895	---	---	---	---	MmugDNA.14814.1.S1_at_LOC100129(

MmugDNA.14815.1.S1_at	210641_at	CAPN9	calpain 9	chr1q42.11-q42.3		10753	CAPN9	calpain 9	---		719222	MmugDNA.14815.1.S1_at_CAPN9
MmugDNA.14816.1.S1_at	212040_at	TGOLN2	trans-golgi network protein 2	chr2p11.2		10618	LILRAA	Leukocyte immunoglobulin-like receptor, subfamily A, member a	---		692342	MmugDNA.14816.1.S1_at_TGOLN2
MmugDNA.14816.1.S1_x_at	NA	NA	NA	NA	NA		NA	NA	NA	NA		MmugDNA.14816.1.S1_x_at_NA
MmugDNA.14817.1.S1_at	212043_at	TGOLN2	trans-golgi network protein 2	chr2p11.2		10618	LILRAA	Leukocyte immunoglobulin-like receptor, subfamily A, member a	---		692342	MmugDNA.14817.1.S1_at_TGOLN2
MmugDNA.14818.1.S1_at	1570190_at	LSAMP	Limbic system-associated membrane protein	chr3q13.2-q21		4045	---	---	---	---		MmugDNA.14818.1.S1_at_LSAMP
MmugDNA.14819.1.S1_at	212693_at	MDN1	MDN1, midasin homolog (yeast)	chr6q15		23195	MDN1	MDN1, midasin homolog	---		703389	MmugDNA.14819.1.S1_at_MDN1
MmugDNA.148.1.S1_at	233939_at	REXO1	REX1, RNA exonuclease 1 homolog (S. cerevisiae)	chr19p13.3		57455	---	---	---	---		MmugDNA.148.1.S1_at_REXO1
MmugDNA.14820.1.S1_at	1553448_at	FLJ34503	hypothetical FLJ34503	chr6q22.1		285759	---	---	---	---		MmugDNA.14820.1.S1_at_FLJ34503
MmugDNA.14821.1.S1_at	207696_at	FUT9	fucosyltransferase 9 (alpha (1,3) fucosyltransferase)	chr6q16		10690	---	---	---	---		MmugDNA.14821.1.S1_at_FUT9
MmugDNA.1482.1.S1_at	207458_at	C8orf51	chromosome 8 open reading frame 51	chr8q24		78998	---	---	---	---		MmugDNA.1482.1.S1_at_C8orf51
MmugDNA.14822.1.S1_at	202326_at	EHMT2	euchromatic histone-lysine N-methyltransferase 2	chr6p21.31		10919	LOC715824	similar to HLA-B associated transcript 8 isoform a	---		715824	MmugDNA.14822.1.S1_at_EHMT2
MmugDNA.14823.1.S1_at	227982_at	SEPSECS	Sep (O-phosphoserine) tRNA:Sec (selenocysteine) tRNA synthase	chr4p15.2		51091	---	---	---	---		MmugDNA.14823.1.S1_at_SEPSECS
MmugDNA.14824.1.S1_at	209590_at	BMP7	bone morphogenetic protein 7	chr20q13		655	BMP7	bone morphogenetic protein 7	---		696948	MmugDNA.14824.1.S1_at_BMP7
MmugDNA.14825.1.S1_at	209309_at	AZGP1	alpha-2-glycoprotein 1, zinc-binding	chr7q22.1		563	LOC710136	similar to alpha-2-glycoprotein 1, zinc	---		710136	MmugDNA.14825.1.S1_at_AZGP1
MmugDNA.14826.1.S1_at	202477_s_at	TUBGCP2	tubulin, gamma complex associated protein 2	chr10q26.3		10844	TUBGCP2	tubulin, gamma complex associated protein 2	---		702201	MmugDNA.14826.1.S1_at_TUBGCP2
MmugDNA.14827.1.S1_at	240613_at	JAK1	Janus kinase 1 (a protein tyrosine kinase)	chr1p32.3-p31.3		3716	---	---	---	---		MmugDNA.14827.1.S1_at_JAK1
MmugDNA.14828.1.S1_at	200929_at	TMED10	transmembrane emp24-like trafficking protein 10 (yeast)	chr14q24.3		10972	TMED10	similar to Transmembrane emp24 domain-containing protein 10 precursor (Transmembrane protein T	---		705372	MmugDNA.14828.1.S1_at_TMED10
MmugDNA.14829.1.S1_at	215009_s_at	SEC31A	SEC31 homolog A (S. cerevisiae)	chr4q21.22		22872	---	---	---	---		MmugDNA.14829.1.S1_at_SEC31A
MmugDNA.14830.1.S1_at	229477_at	THRSP	thyroid hormone responsive (SPOT14 homolog, rat)	chr11q13.5		7069	---	---	---	---		MmugDNA.14830.1.S1_at_THRSP
MmugDNA.14831.1.S1_at	205693_at	TNNT3	troponin T type 3 (skeletal, fast)	chr11p15.5		7140	LOC704095	similar to Troponin T, fast skeletal muscle (TnTf) (Fast skeletal muscle troponin T) (fTnT) (Beta TnTF	---		704095	MmugDNA.14831.1.S1_at_TNNT3
MmugDNA.1483.1.S1_at	219491_at	LRFN4	leucine rich repeat and fibronectin type III domain containing 4	chr11q13.1		78999	LOC713166	similar to leucine rich repeat and fibronectin type III domain containing 4	---		713166	MmugDNA.1483.1.S1_at_LRFN4
MmugDNA.14832.1.S1_at	202858_at	U2AF1	U2 small nuclear RNA auxiliary factor 1	chr21q22.3		7307	LOC722383	similar to Splicing factor U2AF 35 kDa subunit (U2 auxiliary factor 35 kDa subunit) (U2 snRNP auxilia	---		722383	MmugDNA.14832.1.S1_at_U2AF1
MmugDNA.14833.1.S1_at	NA	NA	NA	NA	NA		NA	NA	NA	NA		MmugDNA.14833.1.S1_at_NA
MmugDNA.14834.1.S1_at	232206_at	ULK4	unc-51-like kinase 4 (C. elegans)	chr3p22.1		54986	LOC717956	similar to serine/threonine kinase 36 (fused homolog, Drosophila)	---		717956	MmugDNA.14834.1.S1_at_ULK4
MmugDNA.14835.1.S1_at	227900_at	CBLB	Cas-Br-M (murine) ecotropic retroviral transforming sequence b	chr3q13.11		868	---	---	---	---		MmugDNA.14835.1.S1_at_CBLB
MmugDNA.14836.1.S1_at	237737_at	LOC727770	similar to ankyrin repeat domain 20 family, member A1	chr1q12		727770	LOC718322	similar to F35D11.11b	---		718322	MmugDNA.14836.1.S1_at_LOC727770
MmugDNA.14837.1.S1_at	241292_at	---	---	---	---		---	---	---	---		MmugDNA.14837.1.S1_at_---
MmugDNA.14838.1.S1_at	1553361_x_at	FBXL18	F-box and leucine-rich repeat protein 18	chr7p22.2		80028	---	---	---	---		MmugDNA.14838.1.S1_at_FBXL18
MmugDNA.14839.1.S1_at	218981_at	ACN9	ACN9 homolog (S. cerevisiae)	chr7q21.3		57001	ACN9	ACN9 homolog (S. cerevisiae)	---		697593	MmugDNA.14839.1.S1_at_ACN9
MmugDNA.14840.1.S1_at	1560727_at	HEATR4	HEAT repeat containing 4	chr14q24.3		399671	ARIH2	Similar to Protein ariadne-2 homolog (ARI-2) (Triad1 protein)	---		704645	MmugDNA.14840.1.S1_at_HEATR4
MmugDNA.14841.1.S1_at	243899_at	ARL17P1	ADP-ribosylation factor-like 17 pseudogene 1	chr17q21.32		51326	---	---	---	---		MmugDNA.14841.1.S1_at_ARL17P1
MmugDNA.1484.1.S1_at	239892_at	---	---	---	---		---	---	---	---		MmugDNA.1484.1.S1_at_---
MmugDNA.14842.1.S1_at	223475_at	CRISPLD1	cysteine-rich secretory protein LCCL domain containing 1	chr8q21.11		83690	CRISPLD1	cysteine-rich secretory protein LCCL domain containing 1	---		697491	MmugDNA.14842.1.S1_at_CRISPLD1
MmugDNA.14843.1.S1_at	235977_at	LONRF2	LON peptidase N-terminal domain and ring finger 2	chr2q11.2		164832	---	---	---	---		MmugDNA.14843.1.S1_at_LONRF2
MmugDNA.14844.1.S1_at	200681_at	GLO1	glyoxalase I	chr6p21.3-p21.1		2739	TBR1	T-box, brain, 1	---		701176	MmugDNA.14844.1.S1_at_GLO1
MmugDNA.14845.1.S1_at	207928_s_at	GLRA3	glycine receptor, alpha 3	chr4q33-q34		8001	LOC697984	similar to glycine receptor, alpha 3	---		697984	MmugDNA.14845.1.S1_at_GLRA3
MmugDNA.14845.1.S1_s_at	NA	NA	NA	NA	NA		NA	NA	NA	NA		MmugDNA.14845.1.S1_s_at_NA
MmugDNA.14846.1.S1_at	229144_at	RP1-21O18.1	kazrin	chr1p36.21		23254	---	---	---	---		MmugDNA.14846.1.S1_at_RP1-21O18.
MmugDNA.14847.1.S1_at	241522_at	---	---	---	---		---	---	---	---		MmugDNA.14847.1.S1_at_---
MmugDNA.14848.1.S1_at	206917_at	GNA13	guanine nucleotide binding protein (G protein), alpha 13	chr17q24.3		10672	LOC718144	similar to guanine nucleotide binding protein (G protein), alpha 13	---		718144	MmugDNA.14848.1.S1_at_GNA13
MmugDNA.14849.1.S1_at	201181_at	GNAI3	guanine nucleotide binding protein (G protein), alpha inhibiting activity polypeptide 3	chr1p13		2773	GNAI3	guanine nucleotide binding protein (G protein), alpha inhibiting activity polypeptide 3	---		699310	MmugDNA.14849.1.S1_at_GNAI3
MmugDNA.14850.1.S1_at	243552_at	MBTD1	mbt domain containing 1	chr17q21.33		54799	LOC704544	similar to mbt domain containing 1	---		704544	MmugDNA.14850.1.S1_at_MBTD1
MmugDNA.14851.1.S1_at	1560557_at	---	---	---	---		---	---	---	---		MmugDNA.14851.1.S1_at_---
MmugDNA.1485.1.S1_at	208997_s_at	UCP2	uncoupling protein 2 (mitochondrial, proton carrier)	chr11q13		7351	UCP2	uncoupling protein 2	---		574198	MmugDNA.1485.1.S1_at_UCP2
MmugDNA.14852.1.S1_at	NA	NA	NA	NA	NA		NA	NA	NA	NA		MmugDNA.14852.1.S1_at_NA
MmugDNA.14853.1.S1_at	207862_at	UPK2	uroplakin 2	chr11q23		7379	UPK2	uroplakin 2	---		707184	MmugDNA.14853.1.S1_at_UPK2
MmugDNA.14854.1.S1_at	214352_s_at	KRAS	v-Ki-ras2 Kirsten rat sarcoma viral oncogene homolog	chr12p12.1		3845	KRAS	v-Ki-ras2 Kirsten rat sarcoma viral oncogene homolog	---		707977	MmugDNA.14854.1.S1_at_KRAS
MmugDNA.14855.1.S1_at	202090_s_at	UQCR	ubiquinol-cytochrome c reductase, 6.4kDa subunit	chr19p13.3		10975	UQCR	ubiquinol-cytochrome c reductase, 6.4kDa subunit	---		708396	MmugDNA.14855.1.S1_at_UQCR
MmugDNA.14856.1.S1_at	201678_s_at	C3orf37	chromosome 3 open reading frame 37	chr3q21.3		56941	C11H3orf37	chromosome 3 open reading frame 37 ortholog	---		703524	MmugDNA.14856.1.S1_at_C3orf37
MmugDNA.14857.1.S1_at	237378_at	FLJ46321	FAM75-like protein FLJ46321	chr9q21.32		389763	LOC707877	hypothetical protein LOC707877	---		707877	MmugDNA.14857.1.S1_at_FLJ46321
MmugDNA.14858.1.S1_at	205185_at	SPINK5	serine peptidase inhibitor, Kazal type 5	chr5q32		11005	SPINK5	serine peptidase inhibitor, Kazal type 5	---		709424	MmugDNA.14858.1.S1_at_SPINK5
MmugDNA.14859.1.S1_at	215552_s_at	ESR1	estrogen receptor 1	chr6q25.1		2099	---	---	---	---		MmugDNA.14859.1.S1_at_ESR1
MmugDNA.14860.1.S1_at	218447_at	C16orf61	chromosome 16 open reading frame 61	chr16q23.2		56942	---	---	---	---		MmugDNA.14860.1.S1_at_C16orf61
MmugDNA.14861.1.S1_at	218482_at	ENY2	enhancer of yellow 2 homolog (Drosophila)	chr8q23.1		56943	ENY2	enhancer of yellow 2 homolog (Drosophila)	---		699048	MmugDNA.14861.1.S1_at_ENY2
MmugDNA.1486.1.S1_at	1561014_at	---	---	---	---		---	---	---	---		MmugDNA.1486.1.S1_at_---
MmugDNA.14862.1.S1_at	1569469_a_at	LHX8	LIM homeobox 8	chr1p31.1		431707	LOC709153	similar to LIM homeobox 8	---		709153	MmugDNA.14862.1.S1_at_LHX8
MmugDNA.14863.1.S1_at	1555166_a_at	ZNF396	zinc finger protein 396	chr18q12		252884	ZNF396	similar to Zinc finger protein 396 (Zinc finger and SCAN domain-containing protein 14)	---		708731	MmugDNA.14863.1.S1_at_ZNF396
MmugDNA.14864.1.S1_s_at	225280_x_at	ARSD	arylsulfatase D	chrXp22.3		414	LOC708560	Similar to Sur-8 CG5407-PA, isoform A	---		708560	MmugDNA.14864.1.S1_s_at_ARSD
MmugDNA.14865.1.S1_at	225286_at	ARSD	arylsulfatase D	chrXp22.3		414	---	---	---	---		MmugDNA.14865.1.S1_at_ARSD
MmugDNA.14866.1.S1_at	226112_at	SGCB	sarcoglycan, beta (43kDa dystrophin-associated glycoprotein)	chr4q12		6443	SGCB	sarcoglycan, beta (43kDa dystrophin-associated glycoprotein)	---		702684	MmugDNA.14866.1.S1_at_SGCB
MmugDNA.14866.1.S1_s_at	NA	NA	NA	NA	NA		NA	NA	NA	NA		MmugDNA.14866.1.S1_s_at_NA
MmugDNA.14867.1.S1_at	244022_at	---	---	---	---		---	---	---	---		MmugDNA.14867.1.S1_at_---
MmugDNA.14868.1.S1_at	210317_s_at	YWHAE	tyrosine 3-monooxygenase/tryptophan 5-monooxygenase activation protein, epsilon polypeptide	chr17p13.3		7531	LOC720295	similar to tyrosine 3-monooxygenase/tryptophan 5-monooxygenase activation protein, epsilon polype	---		720295	MmugDNA.14868.1.S1_at_YWHAE
MmugDNA.14869.1.S1_at	242577_at	LOC389834	ankyrin repeat domain 57 pseudogene	---		389834	---	---	---	---		MmugDNA.14869.1.S1_at_LOC389834
MmugDNA.14870.1.S1_at	206620_at	GRAP	GRB2-related adaptor protein	chr17p11.2		10750	GRAP	GRB2-related adaptor protein	---		702473	MmugDNA.14870.1.S1_at_GRAP
MmugDNA.14871.1.S1_at	239052_at	HNRNPD	Heterogeneous nuclear ribonucleoprotein D (AU-rich element RNA binding protein 1, 37kDa)	chr4q21.1-q21.2		3184	---	---	---	---		MmugDNA.14871.1.S1_at_HNRNPD
MmugDNA.1487.1.S1_at	1559777_at	LOC731424	hypothetical LOC731424	---		731424	---	---	---	---		MmugDNA.1487.1.S1_at_LOC731424
MmugDNA.14872.1.S1_at	205732_s_at	NCOA2	nuclear receptor coactivator 2	chr8q13.3		10499	NCOA2	nuclear receptor coactivator 2	---		693455	MmugDNA.14872.1.S1_at_NCOA2
MmugDNA.14873.1.S1_at	242251_at	SPATA5	spermatogenesis associated 5	chr4q28.1		166378	---	---	---	---		MmugDNA.14873.1.S1_at_SPATA5
MmugDNA.14875.1.S1_at	215765_at	LRRC41	leucine rich repeat containing 41	chr1p33		10489	---	---	---	---		MmugDNA.14875.1.S1_at_LRRC41
MmugDNA.14876.1.S1_at	220231_at	C7orf16	chromosome 7 open reading frame 16	chr7p15		10842	C3H7orf16	chromosome 7 open reading frame 16 ortholog	---		694066	MmugDNA.14876.1.S1_at_C7orf16
MmugDNA.14877.1.S1_at	214872_at	RIF1	RAP1 interacting factor homolog (yeast)	chr2q23.3		55183	---	---	---	---		MmugDNA.14877.1.S1_at_RIF1
MmugDNA.14878.1.S1_at	241917_at	---	---	---	---		---	---	---	---		MmugDNA.14878.1.S1_at_---
MmugDNA.14879.1.S1_at	1552974_at	---	---	---	---		---	---	---	---		MmugDNA.14879.1.S1_at_---
MmugDNA.14880.1.S1_at	1568699_at	C14orf179	chromosome 14 open reading frame 179	chr14q24.3		112752	---	---	---	---		MmugDNA.14880.1.S1_at_C14orf179
MmugDNA.14881.1.S1_at	238032_at	---	---	---	---		---	---	---	---		MmugDNA.14881.1.S1_at_---
MmugDNA.1488.1.S1_at	1557677_a_at	TRIML1	tripartite motif family-like 1	chr4q35.2		339976	TRIML1	tripartite motif family-like 1	---		698114	MmugDNA.1488.1.S1_at_TRIML1
MmugDNA.14882.1.S1_s_at	218680_x_at	HYPK	Huntingtin interacting protein K	chr15q15.3		25764	LOC713266	hypothetical protein LOC713266	---		713266	MmugDNA.14882.1.S1_s_at_HYPK
MmugDNA.14883.1.S1_at	234485_at	LOC340947	similar to eukaryotic translation initiation factor 3, subunit 6 interacting protein	chr10p11.21		340947	LOC712118	similar to Eukaryotic translation initiation factor 3 subunit 6-interacting protein	---		712118	MmugDNA.14883.1.S1_at_LOC340947
MmugDNA.14883.1.S1_s_at	NA	NA	NA	NA	NA		NA	NA	NA	NA		MmugDNA.14883.1.S1_s_at_NA
MmugDNA.14884.1.S1_at	208265_at	C2orf83	chromosome 2 open reading frame 83	chr2q36.3		56918	LOC709301	similar to solute carrier family 19, member 3	---		709301	MmugDNA.14884.1.S1_at_C2orf83
MmugDNA.14885.1.S1_at	213064_at	ZC3H14	zinc finger CCCH-type containing 14	chr14q31.3		79882	ZC3H14	zinc finger CCCH-type containing 14	---		694233	MmugDNA.14885.1.S1_at_ZC3H14
MmugDNA.14886.1.S1_at	213063_at	ZC3H14	zinc finger CCCH-type containing 14	chr14q31.3		79882	ZC3H14	zinc finger CCCH-type containing 14	---		694233	MmugDNA.14886.1.S1_at_ZC3H14
MmugDNA.14887.1.S1_at	NA	NA	NA	NA	NA		NA	NA	NA	NA		MmugDNA.14887.1.S1_at_NA
MmugDNA.14888.1.S1_at	NA	NA	NA	NA	NA		NA	NA	NA	NA		MmugDNA.14888.1.S1_at_NA
MmugDNA.14889.1.S1_at	227589_at	PITPNC1	Phosphatidylinositol transfer protein, cytoplasmic 1	chr17q24.2		26207	LOC718773	similar to phosphatidylinositol transfer protein, cytoplasmic 1 isoform a	---		718773	MmugDNA.14889.1.S1_at_PITPNC1
MmugDNA.14890.1.S1_at	228248_at	RICTOR	rapamycin-insensitive companion of mTOR	chr5p13.1		253260	---	---	---	---		MmugDNA.14890.1.S1_at_RICTOR
MmugDNA.14891.1.S1_at	1568760_at	MYH11	myosin, heavy chain 11, smooth muscle	chr16p13.11		4629	---	---	---	---		MmugDNA.14891.1.S1_at_MYH11
MmugDNA.1489.1.S1_s_at	233292_s_at	ANKHD1 /// ANKHD1-E	ankyrin repeat and KH domain containing 1 /// ANKHD1-EIF4EBP3 readthrough transcript /// eukaryo	chr5q31.3	404734 /// 54882 /// 86		LOC698045	similar to MASK-4E-BP3 protein	---		698045	MmugDNA.1489.1.S1_s_at_ANKHD1 ///
MmugDNA.14892.1.S1_at	202438_x_at	IDS	iduronate 2-sulfatase	chrXq28		3423	IDS	iduronate 2-sulfatase	---		700892	MmugDNA.14892.1.S1_at_IDS
MmugDNA.14893.1.S1_at	206052_s_at	SLBP	stem-loop binding protein	chr4p16.3		7884	LOC722546	similar to histone stem-loop binding protein	---		722546	MmugDNA.14893.1.S1_at_SLBP
MmugDNA.14894.1.S1_at	1556913_a_at	GIT2	G protein-coupled receptor kinase interacting ArfGAP 2	chr12q24.1		9815	---	---	---	---		MmugDNA.14894.1.S1_at_GIT2
MmugDNA.14895.1.S1_at	1552719_at	CASC4	cancer susceptibility candidate 4	chr15q15.3		113201	---	---	---	---		MmugDNA.14895.1.S1_at_CASC4
MmugDNA.14896.1.S1_at	201934_at	WDR82	WD repeat domain 82	chr3p21.1		80335	WDR82P1	WD repeat domain 82 pseudogene 1	---		697708	MmugDNA.14896.1.S1_at_WDR82
MmugDNA.14897.1.S1_at	202686_s_at	AXL	AXL receptor tyrosine kinase	chr19q13.1		558	AXL	similar to AXL receptor tyrosine kinase isoform 2	---		706123	MmugDNA.14897.1.S1_at_AXL
MmugDNA.14898.1.S1_at	240622_at	---	---	---	---		---	---	---	---		MmugDNA.14898.1.S1_at_---
MmugDNA.14899.1.S1_at	221380_at	---	---	---	---		---	---	---	---		MmugDNA.14899.1.S1_at_---
MmugDNA.14900.1.S1_at	1556387_at	---	---	---	---		---	---	---	---		MmugDNA.14900.1.S1_at_---
MmugDNA.14901.1.S1_at	242049_s_at	NBAS	neuroblastoma amplified sequence	chr2p24		51594	LOC698203	similar to neuroblastoma-amplified protein	---		698203	MmugDNA.14901.1.S1_at_NBAS
MmugDNA.1490.1.S1_at	NA	NA	NA	NA	NA		NA	NA	NA	NA		MmugDNA.1490.1.S1_at_NA
MmugDNA.14902.1.S1_at	1557123_a_at	CHADL	chondroadherin-like	chr22q13.2		150356	LOC721158	similar to Chondroadherin precursor (Cartilage leucine-rich protein)	---		721158	MmugDNA.14902.1.S1_at_CHADL
MmugDNA.14903.1.S1_at	225073_at	PPHLN1	periphilin 1	chr12q12		51535	---	---	---	---		MmugDNA.14903.1.S1_at_PPHLN1
MmugDNA.14904.1.S1_at	227093_at	USP36	Ubiquitin specific peptidase 36	chr17q25.3		57602	---	---	---	---		MmugDNA.14904.1.S1_at_USP36
MmugDNA.14905.1.S1_at	1569674_at	BHLHB9	Basic helix-loop-helix domain containing, class B, 9	chrXq23		80823	LOC719383	Similar to Translocon-associated protein beta subunit precursor (TRAP-beta) (Signal sequence recep	---		719383	MmugDNA.14905.1.S1_at_BHLHB9
MmugDNA.14906.1.S1_s_at	211639_x_at	IGH@ /// IGHA1 /// IGH	immunoglobulin heavy locus /// immunoglobulin heavy constant alpha 1 /// immunoglobulin heavy con	chr14q32.33 /// chr15q11.2	100126583 /// 28396 //		LOC720935	similar to Ig heavy chain V-II region ARH-77 precursor	---		720935	MmugDNA.14906.1.S1_s_at_IGH@ /// I
MmugDNA.14907.1.S1_at	229013_at	LOC145783	hypothetical LOC145783	chr15q21.3		145783	---	---	---	---		MmugDNA.14907.1.S1_at_LOC145783
MmugDNA.14908.1.S1_at	208461_at	HIC1	hypermethylated in cancer 1	chr17p13.3		3090	LOC721284	similar to hypermethylated in cancer 1	---		721284	MmugDNA.14908.1.S1_at_HIC1
MmugDNA.14909.1.S1_at	214440_at	NAT1	N-acetyltransferase 1 (arylamine N-acetyltransferase)	chr8p23.1-p21.3		9	---	---	---	---		MmugDNA.14909.1.S1_at_NAT1

MmugDNA.14910.1.S1_at	219403_s_at	HPSE	heparanase	chr4q21.3	10855	HPSE	heparanase	---	707583	MmugDNA.14910.1.S1_at_HPSE
MmugDNA.14911.1.S1_at	213557_at	CRKRS	Cdc2-related kinase, arginine/serine-rich	chr17q12	51755	---	---	---	---	MmugDNA.14911.1.S1_at_CRKRS
MmugDNA.1491.1.S1_at	233784_at	---	---	---	---	---	---	---	---	MmugDNA.1491.1.S1_at_---
MmugDNA.14912.1.S1_at	NA	NA	NA	NA	NA	NA	NA	NA	NA	MmugDNA.14912.1.S1_at_NA
MmugDNA.14912.1.S1_s_at	207202_s_at	NR1I2	nuclear receptor subfamily 1, group I, member 2	chr3q12-q13.3	8856	LOC699220	Similar to CG15916-PA	---	699220	MmugDNA.14912.1.S1_s_at_NR1I2
MmugDNA.14913.1.S1_at	206976_s_at	HSPH1	heat shock 105kDa/110kDa protein 1	chr13q12.3	10808	LOC721956	hypothetical protein LOC721956	---	721956	MmugDNA.14913.1.S1_at_HSPH1
MmugDNA.14914.1.S1_at	1569910_at	---	---	---	---	---	---	---	---	MmugDNA.14914.1.S1_at_---
MmugDNA.14915.1.S1_at	239007_at	ZNF616	zinc finger protein 616	chr19q13.33	90317	---	---	---	---	MmugDNA.14915.1.S1_at_ZNF616
MmugDNA.14916.1.S1_at	239378_at	PITRM1	pitrilysin metallopeptidase 1	chr10p15.2	10531	---	---	---	---	MmugDNA.14916.1.S1_at_PITRM1
MmugDNA.14917.1.S1_at	242591_at	DNAH1	Dynein, axonemal, heavy chain 1	chr3p21.1	25981	---	---	---	---	MmugDNA.14917.1.S1_at_DNAH1
MmugDNA.14918.1.S1_at	212775_at	OBSL1	obscurin-like 1	chr2q35	23363	LOC705624	similar to obscurin, cytoskeletal calmodulin and titin-interacting RhoGEF	---	705624	MmugDNA.14918.1.S1_at_OBSL1
MmugDNA.14919.1.S1_at	1563802_at	LOC284551	hypothetical LOC284551	chr1p35.2	284551	---	---	---	---	MmugDNA.14919.1.S1_at_LOC284551
MmugDNA.149.1.S1_at	1563800_at	LOC283140	hypothetical protein LOC283140	chr11q23.1	283140	---	---	---	---	MmugDNA.149.1.S1_at_LOC283140
MmugDNA.14920.1.S1_at	1553172_at	ZNF777	zinc finger protein 777	chr7q36.1	27153	LOC711987	similar to zinc finger protein 629	---	711987	MmugDNA.14920.1.S1_at_ZNF777
MmugDNA.14921.1.S1_at	228999_at	CHD2	chromodomain helicase DNA binding protein 2	chr15q26	1106	---	---	---	---	MmugDNA.14921.1.S1_at_CHD2
MmugDNA.1492.1.S1_at	202671_s_at	PDXK	pyridoxal (pyridoxine, vitamin B6) kinase	chr21q22.3	8566	PDXK	pyridoxal (pyridoxine, vitamin B6) kinase	---	713564	MmugDNA.1492.1.S1_at_PDXK
MmugDNA.14922.1.S1_at	233265_at	---	---	---	---	---	---	---	---	MmugDNA.14922.1.S1_at_---
MmugDNA.14923.1.S1_at	227965_at	---	---	---	---	---	---	---	---	MmugDNA.14923.1.S1_at_---
MmugDNA.14924.1.S1_at	238774_at	---	---	---	---	---	---	---	---	MmugDNA.14924.1.S1_at_---
MmugDNA.14925.1.S1_at	235916_at	YPEL4	yippee-like 4 (Drosophila)	chr11q12.1	219539	LOC696002	Similar to trinucleotide repeat containing 9	---	696002	MmugDNA.14925.1.S1_at_YPEL4
MmugDNA.14926.1.S1_at	238279_x_at	---	---	---	---	---	---	---	---	MmugDNA.14926.1.S1_at_---
MmugDNA.14927.1.S1_at	236929_at	---	---	---	---	---	---	---	---	MmugDNA.14927.1.S1_at_---
MmugDNA.14928.1.S1_at	1556064_at	LOC284926	hypothetical protein LOC284926	chr22q13.31	284926	---	---	---	---	MmugDNA.14928.1.S1_at_LOC284926
MmugDNA.14929.1.S1_at	218847_at	IGF2BP2	insulin-like growth factor 2 mRNA binding protein 2	chr3q27.2	10644	LOC701536	similar to insulin-like growth factor 2 mRNA binding protein 2 isoform b	---	701536	MmugDNA.14929.1.S1_at_IGF2BP2
MmugDNA.14930.1.S1_at	1555778_a_at	POSTN	periostin, osteoblast specific factor	chr13q13.3	10631	---	---	---	---	MmugDNA.14930.1.S1_at_POSTN
MmugDNA.14931.1.S1_at	1552799_at	TSNARE1	t-SNARE domain containing 1	chr8q24.3	203062	TSNARE1	t-SNARE domain containing 1	---	701269	MmugDNA.14931.1.S1_at_TSNARE1
MmugDNA.1493.1.S1_at	NA	NA	NA	NA	NA	NA	NA	NA	NA	MmugDNA.1493.1.S1_at_NA
MmugDNA.14932.1.S1_at	1569167_at	---	---	---	---	---	---	---	---	MmugDNA.14932.1.S1_at_---
MmugDNA.14933.1.S1_at	200048_s_at	JTB	jumping translocation breakpoint	chr1q21	10899	LOC718537	hypothetical protein LOC718537	---	718537	MmugDNA.14933.1.S1_at_JTB
MmugDNA.14934.1.S1_at	224339_s_at	ANGPTL1	angiopoietin-like 1	chr1q25.2	9068	---	---	---	---	MmugDNA.14934.1.S1_at_ANGPTL1
MmugDNA.14935.1.S1_at	243642_x_at	---	---	---	---	---	---	---	---	MmugDNA.14935.1.S1_at_---
MmugDNA.14937.1.S1_at	1555131_a_at	PER3	period homolog 3 (Drosophila)	chr1p36.23	8863	PER3	period 3	---	722059	MmugDNA.14937.1.S1_at_PER3
MmugDNA.14938.1.S1_at	1555555_at	BBS9	Bardet-Biedl syndrome 9	chr7p14	27241	---	---	---	---	MmugDNA.14938.1.S1_at_BBS9
MmugDNA.14939.1.S1_at	229315_at	---	---	---	---	---	---	---	---	MmugDNA.14939.1.S1_at_---
MmugDNA.14940.1.S1_at	239752_at	---	---	---	---	---	---	---	---	MmugDNA.14940.1.S1_at_---
MmugDNA.14941.1.S1_at	1557328_at	LOC283665	hypothetical protein LOC283665	chr15q21.2	283665	---	---	---	---	MmugDNA.14941.1.S1_at_LOC283665
MmugDNA.1494.1.S1_at	200788_s_at	PEA15	phosphoprotein enriched in astrocytes 15	chr1q21.1	8682	LOC699404	Similar to ribosomal protein L15	---	699404	MmugDNA.1494.1.S1_at_PEA15
MmugDNA.14942.1.S1_at	203553_s_at	MAP4K5	mitogen-activated protein kinase kinase kinase kinase 5	chr14q11.2-q21	11183	---	---	---	---	MmugDNA.14942.1.S1_at_MAP4K5
MmugDNA.14943.1.S1_at	1561371_at	FLJ39080	hypothetical gene supported by AK096399	chr8q21.11	441355	---	---	---	---	MmugDNA.14943.1.S1_at_FLJ39080
MmugDNA.14944.1.S1_at	NA	NA	NA	NA	NA	NA	NA	NA	NA	MmugDNA.14944.1.S1_at_NA
MmugDNA.14944.1.S1_s_at	221858_at	TBC1D12	TBC1 domain family, member 12	chr10q23.33	23232	TBC1D12	TBC1 domain family, member 12	---	702000	MmugDNA.14944.1.S1_s_at_TBC1D12
MmugDNA.14945.1.S1_at	201176_s_at	ARCN1	archain 1	chr11q23.3	372	ARCN1	archain 1	---	700328	MmugDNA.14945.1.S1_at_ARCN1
MmugDNA.14946.1.S1_at	NA	NA	NA	NA	NA	NA	NA	NA	NA	MmugDNA.14946.1.S1_at_NA
MmugDNA.14947.1.S1_at	205407_at	RECK	reversion-inducing-cysteine-rich protein with kazal motifs	chr9p13.3	8434	RECK	reversion-inducing-cysteine-rich protein with kazal motifs	---	694043	MmugDNA.14947.1.S1_at_RECK
MmugDNA.14948.1.S1_at	1564684_at	BTN2A2	butyrophilin, subfamily 2, member A2	chr6p22.1	10385	---	---	---	---	MmugDNA.14948.1.S1_at_BTN2A2
MmugDNA.14949.1.S1_at	207229_at	KLRA1	killer cell lectin-like receptor subfamily A, member 1	chr12p13-p12	10748	---	---	---	---	MmugDNA.14949.1.S1_at_KLRA1
MmugDNA.14950.1.S1_at	202853_s_at	RYK	RYK receptor-like tyrosine kinase	chr3q22	6259	PSMA2	Proteasome (prosome, macropain) subunit, alpha type, 2	---	701683	MmugDNA.14950.1.S1_at_RYK
MmugDNA.14951.1.S1_s_at	225394_s_at	ZCRB1	zinc finger CCHC-type and RNA binding motif 1	chr12q12	85437	MADP-1	MADP-1 protein	---	699318	MmugDNA.14951.1.S1_s_at_ZCRB1
MmugDNA.1495.1.S1_at	210545_at	ITSN2	intersectin 2	chr2pter-p25.1	50618	---	---	---	---	MmugDNA.1495.1.S1_at_ITSN2
MmugDNA.14952.1.S1_at	NA	NA	NA	NA	NA	NA	NA	NA	NA	MmugDNA.14952.1.S1_at_NA
MmugDNA.14952.1.S1_s_at	208772_at	ANKHD1 /// ANKHD1-E	ankyrin repeat and KH domain containing 1 /// ANKHD1-EIF4EBP3 readthrough transcript /// eukaryo	chr5q31.3	404734 /// 54882 /// 86	LOC698045	similar to MASK-4E-BP3 protein	---	698045	MmugDNA.14952.1.S1_s_at_ANKHD1 /
MmugDNA.14953.1.S1_at	NA	NA	NA	NA	NA	NA	NA	NA	NA	MmugDNA.14953.1.S1_at_NA
MmugDNA.14954.1.S1_at	202717_s_at	CDC16	cell division cycle 16 homolog (S. cerevisiae)	chr13q34	8881	PRKRIR	Similar to 52 kDa repressor of the inhibitor of the protein kinase (p58IPK-interacting protein) (58 kDa	---	702534	MmugDNA.14954.1.S1_at_CDC16
MmugDNA.14955.1.S1_at	231906_at	HOXD8	homeobox D8	chr2q31.1	3234	LOC700455	similar to Homeobox protein Hox-D8 (Hox-4E) (Hox-5.4)	---	700455	MmugDNA.14955.1.S1_at_HOXD8
MmugDNA.14956.1.S1_at	203968_s_at	CDC6	cell division cycle 6 homolog (S. cerevisiae)	chr17q21.3	990	CDC6	cell division cycle 6 homolog (S. cerevisiae)	---	700081	MmugDNA.14956.1.S1_at_CDC6
MmugDNA.14957.1.S1_at	215756_at	LOC730227	hypothetical protein LOC730227	chr1q32.1	730227	---	---	---	---	MmugDNA.14957.1.S1_at_LOC730227
MmugDNA.14958.1.S1_at	204696_s_at	CDC25A	cell division cycle 25 homolog A (S. pombe)	chr3p21	993	LOC710858	similar to cell division cycle 25A isoform a	---	710858	MmugDNA.14958.1.S1_at_CDC25A
MmugDNA.14959.1.S1_at	203214_x_at	CDC2	cell division cycle 2, G1 to S and G2 to M	chr10q21.1	983	CDC2	cell division cycle 2, G1 to S and G2 to M	---	699269	MmugDNA.14959.1.S1_at_CDC2
MmugDNA.14960.1.S1_at	1562907_at	---	---	---	---	---	---	---	---	MmugDNA.14960.1.S1_at_---
MmugDNA.14961.1.S1_at	237306_at	ZNF829	zinc finger protein 829	chr19q13.12	374899	LOC719280	similar to zinc finger protein 383	---	719280	MmugDNA.14961.1.S1_at_ZNF829
MmugDNA.1496.1.S1_at	216080_s_at	FADS3	fatty acid desaturase 3	chr11q12-q13.1	3995	FADS3	fatty acid desaturase 3	---	722337	MmugDNA.1496.1.S1_at_FADS3
MmugDNA.1496.1.S1_s_at	NA	NA	NA	NA	NA	NA	NA	NA	NA	MmugDNA.1496.1.S1_s_at_NA
MmugDNA.14962.1.S1_at	201450_s_at	TIA1	TIA1 cytotoxic granule-associated RNA binding protein	chr2p13	7072	LOC700825	Similar to Small VCP/p97-interacting protein	---	700825	MmugDNA.14962.1.S1_at_TIA1
MmugDNA.14963.1.S1_at	1552641_s_at	ATAD3A /// ATAD3B ///	ATPase family, AAA domain containing 3A /// ATPase family, AAA domain containing 3B /// similar to	chr1p36.33	55210 /// 732419 /// 83	LOC713780	similar to ATPase family, AAA domain containing 3A	---	713780	MmugDNA.14963.1.S1_at_ATAD3A /// /
MmugDNA.14964.1.S1_at	228737_at	TOX2	TOX high mobility group box family member 2	chr20q13.12	84969	TOX2	TOX high mobility group box family member 2	---	694291	MmugDNA.14964.1.S1_at_TOX2
MmugDNA.14965.1.S1_at	238509_at	CUL1	Cullin 1	chr7q36.1	8454	---	---	---	---	MmugDNA.14965.1.S1_at_CUL1
MmugDNA.14966.1.S1_at	201383_s_at	LOC100133166 /// NBR	similar to neighbor of BRCA1 gene 1 /// neighbor of BRCA1 gene 1	chr17q21.31	100133166 /// 4077	LOC699964	Similar to vesicle transport through interaction with t-SNAREs 1B	---	699964	MmugDNA.14966.1.S1_at_LOC100133
MmugDNA.14967.1.S1_at	220865_s_at	PDSS1	prenyl (decaprenyl) diphosphate synthase, subunit 1	chr10p12.1	23590	PDSS1	prenyl (decaprenyl) diphosphate synthase, subunit 1	---	707598	MmugDNA.14967.1.S1_at_PDSS1
MmugDNA.14968.1.S1_at	243667_at	---	---	---	---	---	---	---	---	MmugDNA.14968.1.S1_at_---
MmugDNA.14969.1.S1_at	210087_s_at	MPZL1	myelin protein zero-like 1	chr1q24.2	9019	LOC700357	similar to myelin protein zero-like 1 isoform a	---	700357	MmugDNA.14969.1.S1_at_MPZL1
MmugDNA.14970.1.S1_at	214490_at	ARSF	arylsulfatase F	chrXp22.3	416	ARSF	arylsulfatase F	---	705983	MmugDNA.14970.1.S1_at_ARSF
MmugDNA.14971.1.S1_at	243409_at	FOXL1	forkhead box L1	chr16q24	2300	---	---	---	---	MmugDNA.14971.1.S1_at_FOXL1
MmugDNA.1497.1.S1_at	214852_x_at	VPS13A	vacuolar protein sorting 13 homolog A (S. cerevisiae)	chr9q21	23230	LOC705323	similar to vacuolar protein sorting 13A isoform A	---	705323	MmugDNA.1497.1.S1_at_VPS13A
MmugDNA.14972.1.S1_at	207319_s_at	CDC2L5	cell division cycle 2-like 5 (cholinesterase-related cell division controller)	chr7p13	8621	LOC711379	similar to cell division cycle 2-like 5 isoform 2	---	711379	MmugDNA.14972.1.S1_at_CDC2L5
MmugDNA.14973.1.S1_at	204126_s_at	CDC45L	CDC45 cell division cycle 45-like (S. cerevisiae)	chr22q11.21	8318	LOC711800	similar to CDC45-like	---	711800	MmugDNA.14973.1.S1_at_CDC45L
MmugDNA.14974.1.S1_at	221823_at	C5orf30	chromosome 5 open reading frame 30	chr5q21.1	90355	LOC703692	hypothetical protein LOC703692	---	703692	MmugDNA.14974.1.S1_at_C5orf30
MmugDNA.14975.1.S1_at	1569981_at	---	---	---	---	---	---	---	---	MmugDNA.14975.1.S1_at_---
MmugDNA.14976.1.S1_at	207172_s_at	CDH11	cadherin 11, type 2, OB-cadherin (osteoblast)	chr16q22.1	1009	LOC708826	similar to cadherin 11, type 2 preproprotein	---	708826	MmugDNA.14976.1.S1_at_CDH11
MmugDNA.14977.1.S1_at	NA	NA	NA	NA	NA	NA	NA	NA	NA	MmugDNA.14977.1.S1_at_NA
MmugDNA.14978.1.S1_at	232449_at	BCO2	beta-carotene oxygenase 2	chr11q22.3-q23.1	83875	BCO2	beta-carotene oxygenase 2	---	712095	MmugDNA.14978.1.S1_at_BCO2
MmugDNA.14979.1.S1_at	235806_at	ARAP3	ArfGAP with RhoGAP domain, ankyrin repeat and PH domain 3	chr5q31.3	64411	ARAP3	ArfGAP with RhoGAP domain, ankyrin repeat and PH domain 3	---	701400	MmugDNA.14979.1.S1_at_ARAP3
MmugDNA.14980.1.S1_at	242151_at	---	---	---	---	---	---	---	---	MmugDNA.14980.1.S1_at_---
MmugDNA.14981.1.S1_at	1558336_at	LOC100130268	Similar to hCG1648866	chr12q21.1	100130268	---	---	---	---	MmugDNA.14981.1.S1_at_LOC100130
MmugDNA.1498.1.S1_at	223140_s_at	DHX36	DEAH (Asp-Glu-Ala-His) box polypeptide 36	chr3p13-q23	170506	LOC707146	Similar to semaphorin 3D	---	707146	MmugDNA.1498.1.S1_at_DHX36
MmugDNA.14982.1.S1_at	225903_at	PIGU	phosphatidylinositol glycan anchor biosynthesis, class U	chr20q11.22	128869	PIGU	phosphatidylinositol glycan anchor biosynthesis, class U	---	707317	MmugDNA.14982.1.S1_at_PIGU
MmugDNA.14983.1.S1_at	242572_at	---	---	---	---	---	---	---	---	MmugDNA.14983.1.S1_at_---
MmugDNA.14984.1.S1_s_at	225818_s_at	TBRG1	transforming growth factor beta regulator 1	chr11q24.2	84897	LOC711895	similar to transforming growth factor beta regulated gene 1	---	711895	MmugDNA.14984.1.S1_s_at_TBRG1
MmugDNA.14985.1.S1_at	214617_at	PRF1	perforin 1 (pore forming protein)	chr10q22	5551	---	---	---	---	MmugDNA.14985.1.S1_at_PRF1
MmugDNA.14986.1.S1_at	238492_at	---	---	---	---	---	---	---	---	MmugDNA.14986.1.S1_at_---
MmugDNA.14987.1.S1_at	203441_s_at	CDH2	cadherin 2, type 1, N-cadherin (neuronal)	chr18q11.2	1000	LOC708820	Hypothetical protein LOC708820	---	708820	MmugDNA.14987.1.S1_at_CDH2
MmugDNA.14988.1.S1_at	201718_s_at	EPB41L2	erythrocyte membrane protein band 4.1-like 2	chr6q23	2037	EPB41L2	erythrocyte membrane protein band 4.1-like 2	---	714889	MmugDNA.14988.1.S1_at_EPB41L2
MmugDNA.14989.1.S1_at	NA	NA	NA	NA	NA	NA	NA	NA	NA	MmugDNA.14989.1.S1_at_NA
MmugDNA.14990.1.S1_at	237479_at	---	---	---	---	---	---	---	---	MmugDNA.14990.1.S1_at_---
MmugDNA.14991.1.S1_at	204750_s_at	DSC2	desmocollin 2	chr18q12.1	1824	LOC712942	similar to desmocollin 2 isoform Dsc2a preproprotein	---	712942	MmugDNA.14991.1.S1_at_DSC2
MmugDNA.1499.1.S1_at	202283_at	SERPINF1	serpin peptidase inhibitor, clade F (alpha-2 antiplasmin, pigment epithelium derived factor), member 1	chr17p13.1	5176	LOC721262	similar to Pigment epithelium-derived factor precursor (PEDF) (EPC-1)	---	721262	MmugDNA.1499.1.S1_at_SERPINF1
MmugDNA.14992.1.S1_at	206866_at	CDH4	cadherin 4, type 1, R-cadherin (retinal)	chr20q13.3	1002	LOC720209	similar to cadherin 4, type 1 preproprotein	---	720209	MmugDNA.14992.1.S1_at_CDH4
MmugDNA.14993.1.S1_at	230020_at	---	---	---	---	---	---	---	---	MmugDNA.14993.1.S1_at_---
MmugDNA.14994.1.S1_at	NA	NA	NA	NA	NA	NA	NA	NA	NA	MmugDNA.14994.1.S1_at_NA
MmugDNA.14995.1.S1_at	204677_at	CDH5	cadherin 5, type 2 (vascular endothelium)	chr16q22.1	1003	CDH5	cadherin 5, type 2	---	693297	MmugDNA.14995.1.S1_at_CDH5
MmugDNA.14996.1.S1_at	200969_at	SERP1	stress-associated endoplasmic reticulum protein 1	chr3q25.1	27230	---	---	---	---	MmugDNA.14996.1.S1_at_SERP1
MmugDNA.14997.1.S1_at	225363_at	PTEN	phosphatase and tensin homolog	chr10q23.3	5728	---	---	---	---	MmugDNA.14997.1.S1_at_PTEN
MmugDNA.14998.1.S1_at	244708_at	FLJ33996 /// LOC10012	hypothetical protein FLJ33996 /// hypothetical protein LOC100127971	chr12q13.13	100127971 /// 283401	---	---	---	---	MmugDNA.14998.1.S1_at_FLJ33996 ///
MmugDNA.14999.1.S1_at	1552724_at	RHOXF1	Rhox homeobox family, member 1	chrXq24	158800	RHOXF1	Rhox homeobox family, member 1	---	695129	MmugDNA.14999.1.S1_at_RHOXF1
MmugDNA.15000.1.S1_at	242877_at	---	---	---	---	---	---	---	---	MmugDNA.15000.1.S1_at_---
MmugDNA.15001.1.S1_at	230614_at	---	---	---	---	---	---	---	---	MmugDNA.15001.1.S1_at_---
MmugDNA.1500.1.S1_at	237764_at	---	---	---	---	LOC712310	hypothetical protein LOC712310	---	712310	MmugDNA.1500.1.S1_at_---
MmugDNA.15002.1.S1_at	228538_at	ZNF662	zinc finger protein 662	chr3p22.1	389114	LOC716008	similar to zinc finger protein 662	---	716008	MmugDNA.15002.1.S1_at_ZNF662
MmugDNA.15003.1.S1_at	224965_at	GNG2	guanine nucleotide binding protein (G protein), gamma 2	chr14q21	54331	---	---	---	---	MmugDNA.15003.1.S1_at_GNG2

MmugDNA.15004.1.S1_at	224964_s_at	GNG2	guanine nucleotide binding protein (G protein), gamma 2	chr14q21	54331	---	---	---	---	MmugDNA.15004.1.S1_at_GNG2
MmugDNA.15005.1.S1_at	203158_s_at	GLS	glutaminase	chr2q32-q34	2744	PCMT1	Protein-L-isoaspartate (D-aspartate) O-methyltransferase	---	695893	MmugDNA.15005.1.S1_at_GLS
MmugDNA.15006.1.S1_at	214869_x_at	GAPVD1	GTPase activating protein and VPS9 domains 1	chr9q33.3	26130	LOC712449	similar to GTPase activating protein and VPS9 domains 1	---	712449	MmugDNA.15006.1.S1_at_GAPVD1
MmugDNA.15007.1.S1_at	211414_s_at	GLS	glutaminase	chr2q32-q34	2744	---	---	---	---	MmugDNA.15007.1.S1_at_GLS
MmugDNA.15008.1.S1_at	239877_at	LOC157740	hypothetical protein C8orf9	chr8p23.1	157740	---	---	---	---	MmugDNA.15008.1.S1_at_LOC157740
MmugDNA.15009.1.S1_at	234912_at	DKFZP547L112	hypothetical protein DKFZp547L112	chr15q11.2	81787	---	---	---	---	MmugDNA.15009.1.S1_at_DKFZP547L
MmugDNA.15010.1.S1_at	235308_at	ZBTB20	zinc finger and BTB domain containing 20	chr3q13.2	26137	LOC712238	similar to zinc finger and BTB domain containing 20	---	712238	MmugDNA.15010.1.S1_at_ZBTB20
MmugDNA.15011.1.S1_at	223750_s_at	TLR10	toll-like receptor 10	chr4p14	81793	TLR10	toll-like receptor 10	---	697980	MmugDNA.15011.1.S1_at_TLR10
MmugDNA.1501.1.S1_at	209434_s_at	PPAT	phosphoribosyl pyrophosphate amidotransferase	chr4q12	5471	PPAT	phosphoribosyl pyrophosphate amidotransferase	---	694868	MmugDNA.1501.1.S1_at_PPAT
MmugDNA.15012.1.S1_at	240318_at	AFMID	arylformamidase	chr17q25.3	125061	---	---	---	---	MmugDNA.15012.1.S1_at_AFMID
MmugDNA.15013.1.S1_at	244393_x_at	---	---	---	---	---	---	---	---	MmugDNA.15013.1.S1_at_---
MmugDNA.15014.1.S1_at	203468_at	CDK10	cyclin-dependent kinase 10	chr16q24	8558	---	---	---	---	MmugDNA.15014.1.S1_at_CDK10
MmugDNA.15015.1.S1_at	239559_at	---	---	---	---	---	---	---	---	MmugDNA.15015.1.S1_at_---
MmugDNA.15016.1.S1_at	241196_at	LOC728777	hypothetical LOC728777	chr10p14	728777	---	---	---	---	MmugDNA.15016.1.S1_at_LOC728777
MmugDNA.15017.1.S1_s_at	208282_x_at	DAZ1 /// DAZ2 /// DAZ3	deleted in azoospermia 1 /// deleted in azoospermia 2 /// deleted in azoospermia 3 /// deleted in azoos	chrYq11.223	1617 /// 57054 /// 5705	DAZL	deleted in azoospermia-like	---	695763	MmugDNA.15017.1.S1_s_at_DAZ1 /// D
MmugDNA.15018.1.S1_at	229663_at	LONP2	lon peptidase 2, peroxisomal	chr16q12.1	83752	---	---	---	---	MmugDNA.15018.1.S1_at_LONP2
MmugDNA.15019.1.S1_at	243691_at	---	---	---	---	---	---	---	---	MmugDNA.15019.1.S1_at_---
MmugDNA.150.1.S1_at	216241_s_at	TCEA1	transcription elongation factor A (SII), 1	chr8q11.2	6917	LOC710829	Similar to stromal cell derived factor receptor 2 homolog	---	710829	MmugDNA.150.1.S1_at_TCEA1
MmugDNA.15020.1.S1_at	202246_s_at	CDK4	cyclin-dependent kinase 4	chr12q14	1019	CDK4	cyclin-dependent kinase 4	---	715650	MmugDNA.15020.1.S1_at_CDK4
MmugDNA.15021.1.S1_at	NA	NA	NA	NA	NA	NA	NA	NA	NA	MmugDNA.15021.1.S1_at_NA
MmugDNA.15021.1.S1_s_at	235085_at	PRAGMIN	homolog of rat pragma of Rnd2	chr8p23.1	157285	LOC706349	hypothetical protein LOC706349	---	706349	MmugDNA.15021.1.S1_s_at_PRAGMIN
MmugDNA.1502.1.S1_at	234597_at	---	---	---	---	---	---	---	---	MmugDNA.1502.1.S1_at_---
MmugDNA.15022.1.S1_at	226825_s_at	TMEM165	transmembrane protein 165	chr4q12	55858	TMEM165	transmembrane protein 165	---	696262	MmugDNA.15022.1.S1_at_TMEM165
MmugDNA.15023.1.S1_at	232174_at	---	---	---	---	---	---	---	---	MmugDNA.15023.1.S1_at_---
MmugDNA.15024.1.S1_at	231614_at	---	---	---	---	---	---	---	---	MmugDNA.15024.1.S1_at_---
MmugDNA.15025.1.S1_at	240771_at	C1orf101	chromosome 1 open reading frame 101	chr1q44	257044	LOC704627	hypothetical protein LOC704627	---	704627	MmugDNA.15025.1.S1_at_C1orf101
MmugDNA.15026.1.S1_at	229498_at	---	---	---	---	---	---	---	---	MmugDNA.15026.1.S1_at_---
MmugDNA.15027.1.S1_s_at	1569978_x_at	---	---	---	---	---	---	---	---	MmugDNA.15027.1.S1_s_at_---
MmugDNA.15028.1.S1_at	1566783_at	---	---	---	---	---	---	---	---	MmugDNA.15028.1.S1_at_---
MmugDNA.15029.1.S1_at	213637_at	---	---	---	---	---	---	---	---	MmugDNA.15029.1.S1_at_---
MmugDNA.15030.1.S1_at	NA	NA	NA	NA	NA	NA	NA	NA	NA	MmugDNA.15030.1.S1_at_NA
MmugDNA.15031.1.S1_s_at	1567242_at	OR2L1P	olfactory receptor, family 2, subfamily L, member 1 pseudogene	chr1q44	26247	LOC697549 /// LOC7068	similar to Olfactory receptor 2L5 (Olfactory receptor OR1-53) /// similar to Olfactory receptor 2L2 (HTI	---	697549 /// 706808	MmugDNA.15031.1.S1_s_at_OR2L1P
MmugDNA.1503.1.S1_at	215147_at	---	---	---	---	---	---	---	---	MmugDNA.1503.1.S1_at_---
MmugDNA.15032.1.S1_at	214053_at	ERBB4	v-erb-a erythroblastic leukemia viral oncogene homolog 4 (avian)	chr2q33.3-q34	2066	---	---	---	---	MmugDNA.15032.1.S1_at_ERBB4
MmugDNA.15034.1.S1_at	236238_at	---	---	---	---	---	---	---	---	MmugDNA.15034.1.S1_at_---
MmugDNA.15035.1.S1_at	222944_s_at	MAGIX	MAGI family member, X-linked	chrXp11.23	79917	LOC713456	Similar to CLIP-190 CG5020-PA, isoform A	---	713456	MmugDNA.15035.1.S1_at_MAGIX
MmugDNA.15036.1.S1_at	207143_at	CDK6	cyclin-dependent kinase 6	chr7q21-q22	1021	CDK6	cyclin-dependent kinase 6	---	701822	MmugDNA.15036.1.S1_at_CDK6
MmugDNA.15037.1.S1_at	225265_at	RBMS1	RNA binding motif, single stranded interacting protein 1	chr2q24.2	5937	LOC703608	similar to RNA binding motif, single stranded interacting protein 1 isoform c	---	703608	MmugDNA.15037.1.S1_at_RBMS1
MmugDNA.15038.1.S1_at	1562642_at	---	---	---	---	---	---	---	---	MmugDNA.15038.1.S1_at_---
MmugDNA.15039.1.S1_at	202855_s_at	SLC16A3	solute carrier family 16, member 3 (monocarboxylic acid transporter 4)	chr17q25	9123	LOC715842	similar to solute carrier family 16, member 3	---	715842	MmugDNA.15039.1.S1_at_SLC16A3
MmugDNA.15040.1.S1_at	225269_s_at	RBMS1	RNA binding motif, single stranded interacting protein 1	chr2q24.2	5937	LOC703608	similar to RNA binding motif, single stranded interacting protein 1 isoform c	---	703608	MmugDNA.15040.1.S1_at_RBMS1
MmugDNA.15041.1.S1_at	216581_at	---	---	---	---	KRT18	similar to Keratin, type I cytoskeletal 18 (Cytokeratin-18) (CK-18) (Keratin-18) (K18)	---	693413	MmugDNA.15041.1.S1_at_---
MmugDNA.1504.1.S1_at	234060_at	---	---	---	---	---	---	---	---	MmugDNA.1504.1.S1_at_---
MmugDNA.15042.1.S1_s_at	203048_s_at	TTC37	tetratricopeptide repeat domain 37	chr5q15	9652	LOC698247	similar to CG8777-PA	---	698247	MmugDNA.15042.1.S1_s_at_TTC37
MmugDNA.15043.1.S1_at	243366_s_at	---	---	---	---	---	---	---	---	MmugDNA.15043.1.S1_at_---
MmugDNA.15044.1.S1_s_at	211184_s_at	USH1C	Usher syndrome 1C (autosomal recessive, severe)	chr11p15.1-p14	10083	---	---	---	---	MmugDNA.15044.1.S1_s_at_USH1C
MmugDNA.15045.1.S1_at	207039_at	CDKN2A	cyclin-dependent kinase inhibitor 2A (melanoma, p16, inhibits CDK4)	chr9p21	1029	LOC709988	similar to Cyclin-dependent kinase inhibitor 2A, isoforms 1/2/3 (Cyclin-dependent kinase 4 inhibitor A)	---	709988	MmugDNA.15045.1.S1_at_CDKN2A
MmugDNA.15046.1.S1_at	235944_at	HMCN1	hemicentin 1	chr1q25.3-q31.1	83872	LOC714026	similar to hemicentin 1	---	714026	MmugDNA.15046.1.S1_at_HMCN1
MmugDNA.15047.1.S1_s_at	212547_at	BRD3	bromodomain containing 3	chr9q34	8019	LOC713107	similar to bromodomain containing protein 3	---	713107	MmugDNA.15047.1.S1_s_at_BRD3
MmugDNA.15048.1.S1_at	202019_s_at	LANCL1	LanC lantibiotic synthetase component C-like 1 (bacterial)	chr2q33-q35	10314	LANCL1	LanC lantibiotic synthetase component C-like 1 (bacterial)	---	711723	MmugDNA.15048.1.S1_at_LANCL1
MmugDNA.15049.1.S1_at	234734_s_at	TNRC6A	trinucleotide repeat containing 6A	chr16p11.2	27327	---	---	---	---	MmugDNA.15049.1.S1_at_TNRC6A
MmugDNA.15050.1.S1_at	228841_at	LYRM7	Lyrm7 homolog (mouse)	chr5q31.1	90624	LOC706196	hypothetical protein LOC706196	---	706196	MmugDNA.15050.1.S1_at_LYRM7
MmugDNA.15050.1.S1_x_at	NA	NA	NA	NA	NA	NA	NA	NA	NA	MmugDNA.15050.1.S1_x_at_NA
MmugDNA.15051.1.S1_at	202759_s_at	AKAP2 /// PALM2 /// PA	A kinase (PRKA) anchor protein 2 /// paralemmin 2 /// PALM2-AKAP2 readthrough transcript	chr9q31-q33	11217 /// 114299 /// 44	AKAP2	A kinase (PRKA) anchor protein 2	---	709841	MmugDNA.15051.1.S1_at_AKAP2 /// P/
MmugDNA.1505.1.S1_at	218097_s_at	CUEDC2	CUE domain containing 2	chr10q24.32	79004	CUEDC2	CUE domain containing 2	---	713243	MmugDNA.1505.1.S1_at_CUEDC2
MmugDNA.15052.1.S1_at	222571_at	ST6GALNAC6	ST6 (alpha-N-acetyl-neuraminyl-2,3-beta-galactosyl-1,3)-N-acetylgalactosaminide alpha-2,6-sialyltran	chr9q34.11	30815	---	---	---	---	MmugDNA.15052.1.S1_at_ST6GALNA(
MmugDNA.15053.1.S1_at	1563589_at	LOC340184	hypothetical protein LOC340184	chr6p24.1	340184	---	---	---	---	MmugDNA.15053.1.S1_at_LOC340184
MmugDNA.15054.1.S1_x_at	NA	NA	NA	NA	NA	NA	NA	NA	NA	MmugDNA.15054.1.S1_x_at_NA
MmugDNA.15055.1.S1_at	214476_at	TFF2	trefoil factor 2	chr21q22.3	7032	LOC714550	similar to Trefoil factor 2 precursor (Spasmolytic polypeptide) (SP)	---	714550	MmugDNA.15055.1.S1_at_TFF2
MmugDNA.15056.1.S1_at	1556602_at	---	---	---	---	---	---	---	---	MmugDNA.15056.1.S1_at_---
MmugDNA.15057.1.S1_at	1559928_at	PAPPA	pregnancy-associated plasma protein A, pappalysin 1	chr9q33.2	5069	---	---	---	---	MmugDNA.15057.1.S1_at_PAPPA
MmugDNA.15058.1.S1_at	228255_at	ALS2CR4	amyotrophic lateral sclerosis 2 (juvenile) chromosome region, candidate 4	---	65062	---	---	---	---	MmugDNA.15058.1.S1_at_ALS2CR4
MmugDNA.15059.1.S1_at	243294_at	ZNF780B	zinc finger protein 780B	chr19q13.2	163131	---	---	---	---	MmugDNA.15059.1.S1_at_ZNF780B
MmugDNA.15060.1.S1_at	242916_at	CEP110	centrosomal protein 110kDa	chr9q33-q34	11064	CEP110	centrosomal protein 110kDa	---	700200	MmugDNA.15060.1.S1_at_CEP110
MmugDNA.15061.1.S1_at	232113_at	---	---	---	---	---	---	---	---	MmugDNA.15061.1.S1_at_---
MmugDNA.1506.1.S1_at	1554690_a_at	TACC1	transforming, acidic coiled-coil containing protein 1	chr8p11.23	6867	TACC1	transforming, acidic coiled-coil containing protein 1	---	706243	MmugDNA.1506.1.S1_at_TACC1
MmugDNA.15062.1.S1_at	204154_at	CDO1	cysteine dioxygenase, type I	chr5q22-q23	1036	SSBP1	Single-stranded DNA binding protein 1	---	694459	MmugDNA.15062.1.S1_at_CDO1
MmugDNA.15063.1.S1_s_at	205709_s_at	CDS1	CDP-diacylglycerol synthase (phosphatidate cytidylyltransferase) 1	chr4q21.23	1040	LOC705086	Similar to Y53G8AR.8	---	705086	MmugDNA.15063.1.S1_s_at_CDS1
MmugDNA.15064.1.S1_at	206193_s_at	CDSN	corneodesmosin	chr6p21.3	1041	CDSN	corneodesmosin	---	714553	MmugDNA.15064.1.S1_at_CDSN
MmugDNA.15065.1.S1_at	236047_at	C8orf5	chromosome 8 open reading frame 5	chr8p23.1	100129441	---	---	---	---	MmugDNA.15065.1.S1_at_C8orf5
MmugDNA.15066.1.S1_at	1555470_a_at	PPM1F	protein phosphatase 1F (PP2C domain containing)	chr22q11.22	9647	PPM1F	protein phosphatase 1F	---	698441	MmugDNA.15066.1.S1_at_PPM1F
MmugDNA.15067.1.S1_at	1568931_at	---	---	---	---	---	---	---	---	MmugDNA.15067.1.S1_at_---
MmugDNA.15068.1.S1_at	242058_at	---	---	---	---	---	---	---	---	MmugDNA.15068.1.S1_at_---
MmugDNA.15069.1.S1_at	244546_at	CYCS	cytochrome c, somatic	chr7p15.2	54205	---	---	---	---	MmugDNA.15069.1.S1_at_CYCS
MmugDNA.15070.1.S1_at	1554261_at	KLHL29	kelch-like 29 (Drosophila)	chr2p24.1	114818	---	---	---	---	MmugDNA.15070.1.S1_at_KLHL29
MmugDNA.15071.1.S1_at	238308_at	LOC100133236	hypothetical protein LOC100133236	chr1q32.1	100133236	---	---	---	---	MmugDNA.15071.1.S1_at_LOC100133
MmugDNA.1507.1.S1_at	243324_x_at	TNPO1	transportin 1	chr5q13.2	3842	---	---	---	---	MmugDNA.1507.1.S1_at_TNPO1
MmugDNA.15072.1.S1_at	231447_at	MGC44328	hypothetical LOC440757	chr20p11.23	440757	LOC697674	hypothetical protein LOC697674	---	697674	MmugDNA.15072.1.S1_at_MGC44328
MmugDNA.15073.1.S1_at	210480_s_at	MYO6	myosin VI	chr6q13	4646	MYO6	myosin VI	---	716634	MmugDNA.15073.1.S1_at_MYO6
MmugDNA.15074.1.S1_at	1564545_a_at	---	---	---	---	---	---	---	---	MmugDNA.15074.1.S1_at_---
MmugDNA.15075.1.S1_at	231120_x_at	PKIB	protein kinase (cAMP-dependent, catalytic) inhibitor beta	chr6q22.31	5570	LOC716982	hypothetical protein LOC716982	---	716982	MmugDNA.15075.1.S1_at_PKIB
MmugDNA.15076.1.S1_at	1555575_a_at	KDELR1	KDEL (Lys-Asp-Glu-Leu) endoplasmic reticulum protein retention receptor 1	chr19q13.3	10945	KDELR1	KDEL (Lys-Asp-Glu-Leu) endoplasmic reticulum protein retention receptor 1	---	717928	MmugDNA.15076.1.S1_at_KDELR1
MmugDNA.15077.1.S1_at	235465_at	FAM123A	family with sequence similarity 123A	chr13q12.13	219287	LOC708730	hypothetical protein LOC708730	---	708730	MmugDNA.15077.1.S1_at_FAM123A
MmugDNA.15078.1.S1_at	1565857_at	---	---	---	---	---	---	---	---	MmugDNA.15078.1.S1_at_---
MmugDNA.15079.1.S1_at	NA	NA	NA	NA	NA	NA	NA	NA	NA	MmugDNA.15079.1.S1_at_NA
MmugDNA.15079.1.S1_s_at	1559964_at	FLJ38717	FLJ38717 protein	chr6p21.1	401261	---	---	---	---	MmugDNA.15079.1.S1_s_at_FLJ38717
MmugDNA.15080.1.S1_at	233023_at	---	---	---	---	---	---	---	---	MmugDNA.15080.1.S1_at_---
MmugDNA.15081.1.S1_at	230488_s_at	NCRNA00118	non-protein coding RNA 118	chr9q34.2	138948	---	---	---	---	MmugDNA.15081.1.S1_at_NCRNA0011
MmugDNA.1508.1.S1_at	1557829_at	---	---	---	---	---	---	---	---	MmugDNA.1508.1.S1_at_---
MmugDNA.15082.1.S1_at	1566465_at	LOC728987	Hypothetical LOC728987	chr1q42.2	728987	---	---	---	---	MmugDNA.15082.1.S1_at_LOC728987
MmugDNA.15083.1.S1_at	232701_at	---	---	---	---	---	---	---	---	MmugDNA.15083.1.S1_at_---
MmugDNA.15084.1.S1_s_at	204661_at	CD52	CD52 molecule	chr1p36	1043	LOC712017	Similar to PML-RARA regulated adaptor molecule 1	---	712017	MmugDNA.15084.1.S1_s_at_CD52
MmugDNA.15085.1.S1_at	244416_at	TTC17	tetratricopeptide repeat domain 17	chr11p12-p11.2	55761	---	---	---	---	MmugDNA.15085.1.S1_at_TTC17
MmugDNA.15086.1.S1_at	226011_at	CCDC12	coiled-coil domain containing 12	chr3p21.31	151903	LOC712061	similar to coiled-coil domain containing 12	---	712061	MmugDNA.15086.1.S1_at_CCDC12
MmugDNA.15087.1.S1_at	240861_at	---	---	---	---	---	---	---	---	MmugDNA.15087.1.S1_at_---
MmugDNA.15088.1.S1_at	1561776_at	---	---	---	---	---	---	---	---	MmugDNA.15088.1.S1_at_---
MmugDNA.15089.1.S1_at	236305_at	RFESD	Rieske (Fe-S) domain containing	chr5q15	317671	LOC698621	hypothetical protein LOC698621	---	698621	MmugDNA.15089.1.S1_at_RFESD
MmugDNA.15090.1.S1_at	1561454_at	---	---	---	---	---	---	---	---	MmugDNA.15090.1.S1_at_---
MmugDNA.15091.1.S1_at	1563950_at	---	---	---	---	---	---	---	---	MmugDNA.15091.1.S1_at_---
MmugDNA.1509.1.S1_s_at	218672_at	SCNM1	sodium channel modifier 1	chr1q21.2	79005	LOC709922	similar to sodium channel modifier 1 isoform 1	---	709922	MmugDNA.1509.1.S1_s_at_SCNM1
MmugDNA.15092.1.S1_at	206756_at	CHST7	carbohydrate (N-acetylglucosamine 6-O) sulfotransferase 7	chrXp11.23	56548	CHST7	carbohydrate (N-acetylglucosamine 6-O) sulfotransferase 7	---	704906	MmugDNA.15092.1.S1_at_CHST7
MmugDNA.15093.1.S1_at	243498_at	---	---	---	---	LOC717620	Similar to sterile alpha motif domain containing 1	---	717620	MmugDNA.15093.1.S1_at_---
MmugDNA.15094.1.S1_at	226943_at	C12orf73	chromosome 12 open reading frame 73	chr12q23.3	728568	LOC699533	hypothetical protein LOC699533	---	699533	MmugDNA.15094.1.S1_at_C12orf73
MmugDNA.15094.1.S1_s_at	NA	NA	NA	NA	NA	NA	NA	NA	NA	MmugDNA.15094.1.S1_s_at_NA
MmugDNA.15095.1.S1_at	226927_at	C12orf73	chromosome 12 open reading frame 73	chr12q23.3	728568	LOC699533	hypothetical protein LOC699533	---	699533	MmugDNA.15095.1.S1_at_C12orf73
MmugDNA.15096.1.S1_at	240032_at	---	---	---	---	---	---	---	---	MmugDNA.15096.1.S1_at_---
MmugDNA.15097.1.S1_at	1553997_a_at	ASPHD1	aspartate beta-hydroxylase domain containing 1	chr16p11.2	253982	LOC707467	hypothetical protein LOC707467	---	707467	MmugDNA.15097.1.S1_at_ASPHD1
MmugDNA.15098.1.S1_s_at	233173_x_at	GTF3C5	general transcription factor IIIC, polypeptide 5, 63kDa	chr9q34	9328	LOC710582	Hypothetical protein LOC710582	---	710582	MmugDNA.15098.1.S1_s_at_GTF3C5

MmugDNA.15099.1.S1_at	232624_at	ABTB2	ankyrin repeat and BTB (POZ) domain containing 2	chr11p13		25841	LOC717429	similar to ankyrin repeat and BTB (POZ) domain containing 2	---		717429	MmugDNA.15099.1.S1_at_ABTB2
MmugDNA.15100.1.S1_at	226211_at	MEG3	maternally expressed 3 (non-protein coding)	chr14q32		55384	LOC693289	Similar to ATP-binding cassette, sub-family A member 8	---		693289	MmugDNA.15100.1.S1_at_MEG3
MmugDNA.15101.1.S1_at	1564017_at	NCRNA00175	non-protein coding RNA 175	chr21q22.3		378832	---	---	---	---		MmugDNA.15101.1.S1_at_NCRNA0017
MmugDNA.15102.1.S1_at	222770_s_at	GUF1	GUF1 GTPase homolog (S. cerevisiae)	chr4p13		60558	GUF1	GUF1 GTPase homolog (S. cerevisiae)	---		703333	MmugDNA.15102.1.S1_at_GUF1
MmugDNA.15103.1.S1_at	242310_at	---	---	---	---		---	---	---	---		MmugDNA.15103.1.S1_at_---
MmugDNA.15104.1.S1_at	244720_at	---	---	---	---		---	---	---	---		MmugDNA.15104.1.S1_at_---
MmugDNA.15105.1.S1_at	238790_at	LOC374443	CLR pseudogene	chr12p13.31		374443	---	---	---	---		MmugDNA.15105.1.S1_at_LOC374443
MmugDNA.15106.1.S1_at	239265_at	TMEM20	transmembrane protein 20	chr10q23.33		159371	---	---	---	---		MmugDNA.15106.1.S1_at_TMEM20
MmugDNA.15107.1.S1_at	203946_s_at	ARG2	arginase, type II	chr14q24.1-q24.3		384	LILRBC	Leukocyte immunoglobulin-like receptor, subfamily B, member c	---		692336	MmugDNA.15107.1.S1_at_ARG2
MmugDNA.15108.1.S1_at	1556810_a_at	---	---	---	---		---	---	---	---		MmugDNA.15108.1.S1_at_---
MmugDNA.15109.1.S1_s_at	NA	NA	NA	NA	NA		NA	NA	NA	NA		MmugDNA.15109.1.S1_s_at_NA
MmugDNA.15110.1.S1_at	222823_at	IPPK	inositol 1,3,4,5,6-pentakisphosphate 2-kinase	chr9q21.33-q22.31		64768	IPPK	inositol 1,3,4,5,6-pentakisphosphate 2-kinase	---		705937	MmugDNA.15110.1.S1_at_IPPK
MmugDNA.15111.1.S1_at	207412_x_at	CELP	carboxyl ester lipase pseudogene	chr9q34.3		1057	---	---	---	---		MmugDNA.15111.1.S1_at_CELP
MmugDNA.1511.1.S1_at	228529_at	---	---	---	---		---	---	---	---		MmugDNA.1511.1.S1_at_---
MmugDNA.15112.1.S1_at	1559966_a_at	LOC100192378	hypothetical LOC100192378	---		100192378	---	---	---	---		MmugDNA.15112.1.S1_at_LOC100192
MmugDNA.15113.1.S1_at	220012_at	ERO1LB	ERO1-like beta (S. cerevisiae)	chr1q42.2-q43		56605	LOC710912	similar to endoplasmic reticulum oxidoreductin 1-Lbeta	---		710912	MmugDNA.15113.1.S1_at_ERO1LB
MmugDNA.15114.1.S1_at	204739_at	CENPC1	centromere protein C 1	chr4q12-q13.3		1060	LOC710620	similar to centromere protein C 1	---		710620	MmugDNA.15114.1.S1_at_CENPC1
MmugDNA.15115.1.S1_at	NA	NA	NA	NA	NA		NA	NA	NA	NA		MmugDNA.15115.1.S1_at_NA
MmugDNA.15115.1.S1_s_at	235027_at	---	---	---	---		---	---	---	---		MmugDNA.15115.1.S1_s_at_---
MmugDNA.15116.1.S1_at	242521_at	---	---	---	---		---	---	---	---		MmugDNA.15116.1.S1_at_---
MmugDNA.15117.1.S1_at	228817_at	ALG9	asparagine-linked glycosylation 9, alpha-1,2-mannosyltransferase homolog (S. cerevisiae)	chr11q23		79796	---	---	---	---		MmugDNA.15117.1.S1_at_ALG9
MmugDNA.15118.1.S1_at	244354_at	---	---	---	---		HMGN1	High-mobility group nucleosome binding domain 1	---		717112	MmugDNA.15118.1.S1_at_---
MmugDNA.15119.1.S1_at	1556969_at	CCDC108	coiled-coil domain containing 108	chr2q35		255101	---	---	---	---		MmugDNA.15119.1.S1_at_CCDC108
MmugDNA.151.1.S1_at	200983_x_at	CD59	CD59 molecule, complement regulatory protein	chr11p13		966	LOC715767	Similar to profilin 1	---		715767	MmugDNA.151.1.S1_at_CD59
MmugDNA.15120.1.A1_at	NA	NA	NA	NA	NA		NA	NA	NA	NA		MmugDNA.15120.1.A1_at_NA
MmugDNA.15120.1.S1_at	1556024_at	EME2	Essential meiotic endonuclease 1 homolog 2 (S. pombe)	chr16p13.3		197342	LOC708348	Similar to cardiomyopathy associated 3	---		708348	MmugDNA.15120.1.S1_at_EME2
MmugDNA.15121.1.S1_s_at	224426_s_at	LOC440888	ARP3 actin-related protein 3 homolog B pseudogene	chr2p11.1		440888	---	---	---	---		MmugDNA.15121.1.S1_s_at_LOC44088
MmugDNA.1512.1.S1_at	1552272_a_at	MGC24975	hypothetical protein MGC24975	chr19p13.3		163154	LOC697102	hypothetical protein LOC697102	---		697102	MmugDNA.1512.1.S1_at_MGC24975
MmugDNA.15123.1.S1_s_at	NA	NA	NA	NA	NA		NA	NA	NA	NA		MmugDNA.15123.1.S1_s_at_NA
MmugDNA.15126.1.S1_at	239390_at	GTDC1	glycosyltransferase-like domain containing 1	chr2q22.2-q22.3		79712	---	---	---	---		MmugDNA.15126.1.S1_at_GTDC1
MmugDNA.15127.1.S1_at	205657_at	HAAO	3-hydroxyanthranilate 3,4-dioxygenase	chr2p21		23498	HAAO	3-hydroxyanthranilate 3,4-dioxygenase	---		713101	MmugDNA.15127.1.S1_at_HAAO
MmugDNA.15128.1.S1_at	226358_at	APH1B	anterior pharynx defective 1 homolog B (C. elegans)	chr15q22.2		83464	APH1B	anterior pharynx defective 1 homolog B (C. elegans)	---		705718	MmugDNA.15128.1.S1_at_APH1B
MmugDNA.15129.1.S1_at	243380_at	---	---	---	---		---	---	---	---		MmugDNA.15129.1.S1_at_---
MmugDNA.15130.1.S1_at	1562739_at	LOC285593	hypothetical LOC285593	chr5q35.2		285593	---	---	---	---		MmugDNA.15130.1.S1_at_LOC285593
MmugDNA.15131.1.S1_at	230779_at	TNRC6B	trinucleotide repeat containing 6B	chr22q13.1		23112	TNRC6B	trinucleotide repeat containing 6B	---		704630	MmugDNA.15131.1.S1_at_TNRC6B
MmugDNA.1513.1.S1_at	238819_at	ZNF347	zinc finger protein 347	chr19q13.41		84671	LOC720141	similar to zinc finger protein 347	---		720141	MmugDNA.1513.1.S1_at_ZNF347
MmugDNA.15132.1.S1_at	207052_at	HAVCR1	hepatitis A virus cellular receptor 1	chr5q33.2		26762	LOC715333	similar to hepatitis A virus cellular receptor 1	---		715333	MmugDNA.15132.1.S1_at_HAVCR1
MmugDNA.15133.1.S1_at	243410_at	---	---	---	---		---	---	---	---		MmugDNA.15133.1.S1_at_---
MmugDNA.15134.1.S1_at	207361_at	HBP1	HMG-box transcription factor 1	chr7q22-q31		26959	---	---	---	---		MmugDNA.15134.1.S1_at_HBP1
MmugDNA.15135.1.S1_at	233916_at	KIAA1486	KIAA1486 protein	chr2q36.3		57624	---	---	---	---		MmugDNA.15135.1.S1_at_KIAA1486
MmugDNA.15136.1.S1_at	1559901_s_at	C21orf34	chromosome 21 open reading frame 34	chr21q21.1		388815	---	---	---	---		MmugDNA.15136.1.S1_at_C21orf34
MmugDNA.15137.1.S1_at	201495_x_at	MYH11	myosin, heavy chain 11, smooth muscle	chr16p13.11		4629	LOC713882	similar to smooth muscle myosin heavy chain 11 isoform SM1A	---		713882	MmugDNA.15137.1.S1_at_MYH11
MmugDNA.15138.1.S1_at	240799_at	SLC35F4	solute carrier family 35, member F4	chr14q22.2		341880	SLC35F3	similar to solute carrier family 35, member F4	---		701521	MmugDNA.15138.1.S1_at_SLC35F4
MmugDNA.15139.1.S1_at	244071_at	FBLL1	fibrillarin-like 1	chr5q35.1		345630	LOC703625	similar to fibrillarin	---		703625	MmugDNA.15139.1.S1_at_FBLL1
MmugDNA.15140.1.S1_at	244049_at	---	---	---	---		---	---	---	---		MmugDNA.15140.1.S1_at_---
MmugDNA.15141.1.S1_at	204965_at	GC	group-specific component (vitamin D binding protein)	chr4q12-q13		2638	GC	group-specific component (vitamin D binding protein)	---		705708	MmugDNA.15141.1.S1_at_GC
MmugDNA.15141.1.S1_s_at	NA	NA	NA	NA	NA		NA	NA	NA	NA		MmugDNA.15141.1.S1_s_at_NA
MmugDNA.1514.1.S1_at	NA	NA	NA	NA	NA		NA	NA	NA	NA		MmugDNA.1514.1.S1_at_NA
MmugDNA.15142.1.S1_at	1566837_at	---	---	---	---		---	---	---	---		MmugDNA.15142.1.S1_at_---
MmugDNA.15144.1.S1_at	1562577_at	---	---	---	---		---	---	---	---		MmugDNA.15144.1.S1_at_---
MmugDNA.15145.1.S1_at	239060_at	---	---	---	---		---	---	---	---		MmugDNA.15145.1.S1_at_---
MmugDNA.15146.1.S1_at	229446_at	---	---	---	---		---	---	---	---		MmugDNA.15146.1.S1_at_---
MmugDNA.15147.1.S1_at	NA	NA	NA	NA	NA		NA	NA	NA	NA		MmugDNA.15147.1.S1_at_NA
MmugDNA.15148.1.S1_at	208111_at	AVPR2	arginine vasopressin receptor 2	chrXq28		554	AVPR2	arginine vasopressin receptor 2	---		697763	MmugDNA.15148.1.S1_at_AVPR2
MmugDNA.15149.1.S1_at	242281_at	GLUL	glutamate-ammonia ligase (glutamine synthetase)	chr1q31		2752	---	---	---	---		MmugDNA.15149.1.S1_at_GLUL
MmugDNA.15150.1.S1_at	223345_at	HDAC8	histone deacetylase 8	chrXq13		55869	LOC699642	similar to Histone deacetylase 8 (HD8)	---		699642	MmugDNA.15150.1.S1_at_HDAC8
MmugDNA.1515.1.S1_at	210724_at	EMR3	egf-like module containing, mucin-like, hormone receptor-like 3	chr19p13.1		84658	LOC718975	similar to egf-like module-containing mucin-like receptor 3 isoform a	---		718975	MmugDNA.1515.1.S1_at_EMR3
MmugDNA.15152.1.S1_at	212612_at	RCOR1	REST corepressor 1	chr14q32.31-q32.32		23186	LOC694670	similar to REST corepressor 1	---		694670	MmugDNA.15152.1.S1_at_RCOR1
MmugDNA.15153.1.S1_at	219743_at	HEY2	hairy/enhancer-of-split related with YRPW motif 2	chr6q21		23493	LOC712988	similar to hairy/enhancer-of-split related with YRPW motif 2	---		712988	MmugDNA.15153.1.S1_at_HEY2
MmugDNA.15154.1.S1_at	238020_at	PSMC2	Proteasome (prosome, macropain) 26S subunit, ATPase, 2	chr7q22.1-q22.3		5701	---	---	---	---		MmugDNA.15154.1.S1_at_PSMC2
MmugDNA.15155.1.S1_at	234445_at	NCRNA00171	non-protein coding RNA 171	chr6p21.33		80862	---	---	---	---		MmugDNA.15155.1.S1_at_NCRNA0017
MmugDNA.15156.1.S1_at	236491_at	BCL2L10	BCL2-like 10 (apoptosis facilitator)	chr15q21		10017	BCL2L10	BCL2-like 10 (apoptosis facilitator)	---		695562	MmugDNA.15156.1.S1_at_BCL2L10
MmugDNA.15157.1.S1_at	238407_at	---	---	---	---		---	---	---	---		MmugDNA.15157.1.S1_at_---
MmugDNA.15158.1.S1_at	235609_at	---	---	---	---		---	---	---	---		MmugDNA.15158.1.S1_at_---
MmugDNA.15159.1.S1_at	233327_at	UBE2CBP	ubiquitin-conjugating enzyme E2C binding protein	chr6q14.1		90025	---	---	---	---		MmugDNA.15159.1.S1_at_UBE2CBP
MmugDNA.15160.1.S1_at	NA	NA	NA	NA	NA		NA	NA	NA	NA		MmugDNA.15160.1.S1_at_NA
MmugDNA.15160.1.S1_s_at	NA	NA	NA	NA	NA		NA	NA	NA	NA		MmugDNA.15160.1.S1_s_at_NA
MmugDNA.15161.1.S1_at	206861_s_at	CGGBP1	CGG triplet repeat binding protein 1	chr3p12-p11.1		8545	CGGBP1	CGG triplet repeat binding protein 1	---		693920	MmugDNA.15161.1.S1_at_CGGBP1
MmugDNA.1516.1.S1_at	214231_s_at	KIAA0564	KIAA0564	chr13q14.11		23078	KIAA0564	KIAA0564 protein	---		699086	MmugDNA.1516.1.S1_at_KIAA0564
MmugDNA.15162.1.S1_at	208052_x_at	CEACAM3	carcinoembryonic antigen-related cell adhesion molecule 3	chr19q13.2		1084	---	---	---	---		MmugDNA.15162.1.S1_at_CEACAM3
MmugDNA.15162.1.S1_s_at	NA	NA	NA	NA	NA		NA	NA	NA	NA		MmugDNA.15162.1.S1_s_at_NA
MmugDNA.15163.1.S1_at	215658_at	---	---	---	---		---	---	---	---		MmugDNA.15163.1.S1_at_---
MmugDNA.15164.1.S1_s_at	226734_at	EIF4E2	eukaryotic translation initiation factor 4E family member 2	chr2q37.1		9470	---	---	---	---		MmugDNA.15164.1.S1_s_at_EIF4E2
MmugDNA.15165.1.S1_at	239578_at	---	---	---	---		---	---	---	---		MmugDNA.15165.1.S1_at_---
MmugDNA.15166.1.S1_at	227430_at	ZC3H10	zinc finger CCCH-type containing 10	chr12q13.2		84872	LOC711633	similar to zinc finger CCCH-type containing 10	---		711633	MmugDNA.15166.1.S1_at_ZC3H10
MmugDNA.15167.1.S1_at	207205_at	CEACAM4	carcinoembryonic antigen-related cell adhesion molecule 4	chr19q13.2		1089	---	---	---	---		MmugDNA.15167.1.S1_at_CEACAM4
MmugDNA.15168.1.S1_at	232879_at	CRTC3	CREB regulated transcription coactivator 3	chr15q26.1		64784	---	---	---	---		MmugDNA.15168.1.S1_at_CRTC3
MmugDNA.15169.1.S1_at	240561_at	MBNL3	hypothetical LOC100130541	chrXq26.2		100130541	---	---	---	---		MmugDNA.15169.1.S1_at_MBNL3
MmugDNA.15170.1.S1_s_at	NA	NA	NA	NA	NA		NA	NA	NA	NA		MmugDNA.15170.1.S1_s_at_NA
MmugDNA.15171.1.S1_at	1565762_at	---	---	---	---		---	---	---	---		MmugDNA.15171.1.S1_at_---
MmugDNA.1517.1.S1_at	225836_s_at	C12orf32	chromosome 12 open reading frame 32	chr12p13.33		83695	LOC709008	hypothetical protein LOC709008	---		709008	MmugDNA.1517.1.S1_at_C12orf32
MmugDNA.15172.1.S1_at	235319_at	LOC100216546	hypothetical LOC100216546	---		100216546	---	---	---	---		MmugDNA.15172.1.S1_at_LOC100216
MmugDNA.15173.1.S1_s_at	210404_x_at	CAMK2B	calcium/calmodulin-dependent protein kinase II beta	chr22q12|7p14.3-p14.1		816	LOC706977	similar to calcium/calmodulin-dependent protein kinase IIB isoform 1	---		706977	MmugDNA.15173.1.S1_s_at_CAMK2B
MmugDNA.15174.1.S1_at	207453_s_at	DNAJB5	DnaJ (Hsp40) homolog, subfamily B, member 5	chr9p13.3		25822	DNAJB5	DnaJ (Hsp40) homolog, subfamily B, member 5	---		699081	MmugDNA.15174.1.S1_at_DNAJB5
MmugDNA.15175.1.S1_at	208855_s_at	STK24	serine/threonine kinase 24 (STE20 homolog, yeast)	chr13q31.2-q32.3		8428	STK24	serine/threonine kinase 24 (STE20 homolog, yeast)	---		697841	MmugDNA.15175.1.S1_at_STK24
MmugDNA.15176.1.S1_at	202415_s_at	HSPBP1	HSPA (heat shock 70kDa) binding protein, cytoplasmic cochaperone 1	chr19q13.42		23640	LOC699908	similar to hsp70-interacting protein	---		699908	MmugDNA.15176.1.S1_at_HSPBP1
MmugDNA.15177.1.S1_at	226186_at	TMOD2	tropomodulin 2 (neuronal)	chr15q21.1-q21.2		29767	TMOD2	tropomodulin 2 (neuronal)	---		694355	MmugDNA.15177.1.S1_at_TMOD2
MmugDNA.15178.1.S1_at	209430_at	BTAF1	BTAF1 RNA polymerase II, B-TFIID transcription factor-associated, 170kDa (Mot1 homolog, S. cerev	chr10q22-q23		9044	BTAF1	BTAF1 RNA polymerase II, B-TFIID transcription factor-associated, 170kDa	---		698008	MmugDNA.15178.1.S1_at_BTAF1
MmugDNA.15179.1.S1_at	220083_x_at	UCHL5	ubiquitin carboxyl-terminal hydrolase L5	chr1q32		51377	LOC712473	similar to ubiquitin C-terminal hydrolase UCH37	---		712473	MmugDNA.15179.1.S1_at_UCHL5
MmugDNA.15180.1.S1_at	1556656_at	---	---	---	---		---	---	---	---		MmugDNA.15180.1.S1_at_---
MmugDNA.15181.1.S1_at	226961_at	PRR15	proline rich 15	chr7p15.1		222171	PRR15	proline rich 15	---		696869	MmugDNA.15181.1.S1_at_PRR15
MmugDNA.1518.1.S1_at	225837_at	C12orf32	chromosome 12 open reading frame 32	chr12p13.33		83695	LOC709008	hypothetical protein LOC709008	---		709008	MmugDNA.1518.1.S1_at_C12orf32
MmugDNA.15182.1.S1_at	225640_at	LOC401504	hypothetical gene supported by AK091718	chr9p13.2		401504	LOC718554	hypothetical protein LOC718554	---		718554	MmugDNA.15182.1.S1_at_LOC401504
MmugDNA.15183.1.S1_at	242612_at	---	---	---	---		---	---	---	---		MmugDNA.15183.1.S1_at_---
MmugDNA.15184.1.S1_at	225635_s_at	LOC401504	Hypothetical gene supported by AK091718	chr9p13.2		401504	---	---	---	---		MmugDNA.15184.1.S1_at_LOC401504
MmugDNA.15185.1.S1_s_at	217842_at	LUC7L2	LUC7-like 2 (S. cerevisiae)	chr7q34		51631	LUC7L2	LUC7-like 2 (S. cerevisiae)	---		711104	MmugDNA.15185.1.S1_s_at_LUC7L2
MmugDNA.15186.1.S1_at	243376_at	TANK	TRAF family member-associated NFKB activator	chr2q24-q31		10010	---	---	---	---		MmugDNA.15186.1.S1_at_TANK
MmugDNA.15187.1.S1_at	1566994_at	---	---	---	---		---	---	---	---		MmugDNA.15187.1.S1_at_---
MmugDNA.15188.1.S1_at	229707_at	ZNF606	zinc finger protein 606	chr19q13.4		80095	---	---	---	---		MmugDNA.15188.1.S1_at_ZNF606
MmugDNA.15189.1.S1_at	1557580_at	---	---	---	---		---	---	---	---		MmugDNA.15189.1.S1_at_---
MmugDNA.15190.1.S1_at	228096_at	C1orf151	chromosome 1 open reading frame 151	chr1p36.13		440574	---	---	---	---		MmugDNA.15190.1.S1_at_C1orf151
MmugDNA.15191.1.S1_at	1555439_at	GTF3C3	general transcription factor IIIC, polypeptide 3, 102kDa	chr2q33.1		9330	GTF3C3	general transcription factor IIIC, polypeptide 3, 102kDa	---		696338	MmugDNA.15191.1.S1_at_GTF3C3
MmugDNA.1519.1.S1_at	231956_at	KIAA1618	KIAA1618	chr17q25.3		57714	---	---	---	---		MmugDNA.1519.1.S1_at_KIAA1618
MmugDNA.15192.1.S1_at	206324_s_at	DAPK2	death-associated protein kinase 2	chr15q22.31		23604	DAPK2	death-associated protein kinase 2	---		706421	MmugDNA.15192.1.S1_at_DAPK2
MmugDNA.15193.1.S1_at	223781_x_at	ADH4	alcohol dehydrogenase 4 (class II), pi polypeptide	chr4q21-q24|4q22		127	LOC707367	similar to class II alcohol dehydrogenase 4 pi subunit	---		707367	MmugDNA.15193.1.S1_at_ADH4
MmugDNA.15194.1.S1_at	219290_x_at	DAPP1	dual adaptor of phosphotyrosine and 3-phosphoinositides	chr4q25-q27		27071	---	---	---	---		MmugDNA.15194.1.S1_at_DAPP1
MmugDNA.15195.1.S1_at	NA	NA	NA	NA	NA		NA	NA	NA	NA		MmugDNA.15195.1.S1_at_NA
MmugDNA.15195.1.S1_s_at	205818_at	DBC1	deleted in bladder cancer 1	chr9q32-q33		1620	LOC701794	similar to deleted in bladder cancer 1	---		701794	MmugDNA.15195.1.S1_s_at_DBC1

MmugDNA.15196.1.S1_at	223874_at	ARP11	actin-related Arp11	chr7q36.1	653857	---	---	---	---	MmugDNA.15196.1.S1_at_ARP11
MmugDNA.15197.1.S1_at	1566099_at	CD6	CD6 molecule	chr11q13	923	---	---	---	---	MmugDNA.15197.1.S1_at_CD6
MmugDNA.15198.1.S1_at	231304_at	PPP3R2	protein phosphatase 3 (formerly 2B), regulatory subunit B, beta isoform	chr9q31.1	5535	LOC710900	Similar to signal peptidase complex subunit 2 homolog	---	710900	MmugDNA.15198.1.S1_at_PPP3R2
MmugDNA.15199.1.S1_at	NA	NA	NA	NA	NA	NA	NA	NA	NA	MmugDNA.15199.1.S1_at_NA
MmugDNA.15.1.S1_at	236487_at	SCLT1	sodium channel and clathrin linker 1	chr4q28.2	132320	SCLT1	sodium channel and clathrin linker 1	---	693822	MmugDNA.15.1.S1_at_SCLT1
MmugDNA.15200.1.S1_at	1559443_s_at	LOC283888	hypothetical protein LOC283888	chr16p12.2	283888	---	---	---	---	MmugDNA.15200.1.S1_at_LOC283888
MmugDNA.15201.1.S1_at	237116_at	LOC646903	hypothetical LOC646903	chr3q25.1	646903	---	---	---	---	MmugDNA.15201.1.S1_at_LOC646903
MmugDNA.1520.1.S1_at	228810_at	CCNYL1	cyclin Y-like 1	chr2q33.3	151195	---	---	---	---	MmugDNA.1520.1.S1_at_CCNYL1
MmugDNA.15202.1.S1_at	217826_s_at	UBE2J1	ubiquitin-conjugating enzyme E2, J1 (UBC6 homolog, yeast)	chr6q15	51465	UBE2J1	ubiquitin-conjugating enzyme E2, J1 (UBC6 homolog, yeast)	---	701100	MmugDNA.15202.1.S1_at_UBE2J1
MmugDNA.15203.1.S1_at	240076_at	---	---	---	---	---	---	---	---	MmugDNA.15203.1.S1_at_---
MmugDNA.15204.1.S1_at	214322_at	CAMK2G	calcium/calmodulin-dependent protein kinase II gamma	chr10q22	818	CAMK2G	calcium/calmodulin-dependent protein kinase II gamma	---	705980	MmugDNA.15204.1.S1_at_CAMK2G
MmugDNA.15205.1.S1_at	204098_at	RBMX2	RNA binding motif protein, X-linked 2	chrXq25	51634	RBMX2	RNA binding motif protein, X-linked 2	---	703472	MmugDNA.15205.1.S1_at_RBMX2
MmugDNA.15206.1.S1_at	1562559_at	C9orf89	chromosome 9 open reading frame 89	chr9q22.31	84270	---	---	---	---	MmugDNA.15206.1.S1_at_C9orf89
MmugDNA.15207.1.S1_at	1558111_at	MBNL1	muscleblind-like (Drosophila)	chr3q25	4154	---	---	---	---	MmugDNA.15207.1.S1_at_MBNL1
MmugDNA.15208.1.S1_at	242698_at	AKAP14	A kinase (PRKA) anchor protein 14	chrXq24	158798	LOC709295	Similar to cat eye syndrome critical region protein 1 isoform a precursor	---	709295	MmugDNA.15208.1.S1_at_AKAP14
MmugDNA.15209.1.S1_at	225376_at	C20orf11	chromosome 20 open reading frame 11	chr20q13.33	54994	C20orf11	chromosome 20 open reading frame 11	---	698189	MmugDNA.15209.1.S1_at_C20orf11
MmugDNA.15210.1.S1_at	1559132_at	TMEM80	transmembrane protein 80	chr11p15.5	283232	---	---	---	---	MmugDNA.15210.1.S1_at_TMEM80
MmugDNA.15211.1.S1_at	201104_x_at	NBPF10 /// NBPF11 ///	neuroblastoma breakpoint family, member 10 /// neuroblastoma breakpoint family, member 11 /// neu	chr1q12-q21.2 /// chr1q21.1	100132406 /// 200030	LOC708768 /// LOC7230	hypothetical protein LOC708768 /// hypothetical protein LOC723057	---	708768 /// 723057	MmugDNA.15211.1.S1_at_NBPF10 /// N
MmugDNA.1521.1.S1_s_at	239068_at	GNL1	guanine nucleotide binding protein-like 1	chr6p21.3	2794	---	---	---	---	MmugDNA.1521.1.S1_s_at_GNL1
MmugDNA.15212.1.S1_at	219825_at	CYP26B1	cytochrome P450, family 26, subfamily B, polypeptide 1	chr2p13.3	56603	---	---	---	---	MmugDNA.15212.1.S1_at_CYP26B1
MmugDNA.15213.1.S1_at	208847_s_at	ADH5	alcohol dehydrogenase 5 (class III), chi polypeptide	chr4q21-q25	128	ADH5	alcohol dehydrogenase 5 (class III), chi polypeptide	---	707258	MmugDNA.15213.1.S1_at_ADH5
MmugDNA.15214.1.S1_s_at	214549_x_at	SPRR1A	small proline-rich protein 1A	chr1q21-q22	6698	SPRR1B	small proline-rich protein 1B	---	613022	MmugDNA.15214.1.S1_s_at_SPRR1A
MmugDNA.15215.1.S1_s_at	204700_x_at	C1orf107	chromosome 1 open reading frame 107	chr1q32.2	27042	LOC712825	similar to digestive-organ expansion factor	---	712825	MmugDNA.15215.1.S1_s_at_C1orf107
MmugDNA.15216.1.S1_at	232978_at	---	---	---	---	---	---	---	---	MmugDNA.15216.1.S1_at_---
MmugDNA.15217.1.S1_at	1569538_at	---	---	---	---	---	---	---	---	MmugDNA.15217.1.S1_at_---
MmugDNA.15218.1.S1_at	203195_s_at	NUP98	nucleoporin 98kDa	chr11p15.5	4928	LOC717608	similar to nucleoporin 98kD isoform 1	---	717608	MmugDNA.15218.1.S1_at_NUP98
MmugDNA.15219.1.S1_at	NA	NA	NA	NA	NA	NA	NA	NA	NA	MmugDNA.15219.1.S1_at_NA
MmugDNA.15219.1.S1_s_at	214864_s_at	GRHPR	glyoxylate reductase/hydroxypyruvate reductase	chr9q12	9380	LOC718494	similar to Glyoxylate reductase/hydroxypyruvate reductase	---	718494	MmugDNA.15219.1.S1_s_at_GRHPR
MmugDNA.152.1.S1_at	200984_s_at	CD59	CD59 molecule, complement regulatory protein	chr11p13	966	LOC715767	Similar to profilin 1	---	715767	MmugDNA.152.1.S1_at_CD59
MmugDNA.15220.1.S1_s_at	207891_s_at	TREX2	HAUS augmin-like complex, subunit 7 /// three prime repair exonuclease 2	chrXq28	55559	TREX2	three prime repair exonuclease 2	---	695353	MmugDNA.15220.1.S1_s_at_HAUS7 ///
MmugDNA.15221.1.S1_at	240830_at	SCARNA17	small Cajal body-specific RNA 17	chr18q21.1	677769	---	---	---	---	MmugDNA.15221.1.S1_at_SCARNA17
MmugDNA.1522.1.S1_at	NA	NA	NA	NA	NA	NA	NA	NA	NA	MmugDNA.1522.1.S1_at_NA
MmugDNA.15222.1.S1_at	217768_at	C14orf166	chromosome 14 open reading frame 166	chr14q22.1	51637	LOC710094	similar to Protein C14orf166	---	710094	MmugDNA.15222.1.S1_at_C14orf166
MmugDNA.15223.1.S1_at	1564193_at	FLJ39061 /// LOC33980	hypothetical protein FLJ39061 /// KIAA2012 protein	chr2q33.1	165057 /// 339809	LOC704598	hypothetical protein LOC704598	---	704598	MmugDNA.15223.1.S1_at_FLJ39061 ///
MmugDNA.15224.1.S1_at	216146_at	---	---	---	---	---	---	---	---	MmugDNA.15224.1.S1_at_---
MmugDNA.15225.1.S1_at	220802_at	KCNH4	potassium voltage-gated channel, subfamily H (eag-related), member 4	chr17q21.2	23415	---	---	---	---	MmugDNA.15225.1.S1_at_KCNH4
MmugDNA.15226.1.S1_at	238183_at	---	---	---	---	---	---	---	---	MmugDNA.15226.1.S1_at_---
MmugDNA.15227.1.S1_at	201994_at	MORF4L2	mortality factor 4 like 2	chrXq22	9643	MORF4L2	similar to Mortality factor 4-like protein 2 (MORF-related gene X protein) (Transcription factor-like prot	---	697637	MmugDNA.15227.1.S1_at_MORF4L2
MmugDNA.15228.1.S1_at	1566656_a_at	---	---	---	---	---	---	---	---	MmugDNA.15228.1.S1_at_---
MmugDNA.15229.1.S1_at	213853_at	DNAJC24	DnaJ (Hsp40) homolog, subfamily C, member 24	chr11p13	120526	---	---	---	---	MmugDNA.15229.1.S1_at_DNAJC24
MmugDNA.15230.1.S1_at	226890_at	WDR35	WD repeat domain 35	chr2p24.1	57539	WDR35	WD repeat domain 35	---	716638	MmugDNA.15230.1.S1_at_WDR35
MmugDNA.15231.1.S1_at	209637_s_at	RGS12	regulator of G-protein signaling 12	chr4p16.3	6002	LOC699927	similar to regulator of G-protein signalling 12 isoform 1	---	699927	MmugDNA.15231.1.S1_at_RGS12
MmugDNA.1523.1.S1_at	240769_at	---	---	---	---	---	---	---	---	MmugDNA.1523.1.S1_at_---
MmugDNA.15232.1.S1_at	229812_at	USP48	ubiquitin specific peptidase 48	chr1p36.12	84196	---	---	---	---	MmugDNA.15232.1.S1_at_USP48
MmugDNA.15233.1.S1_at	226889_at	WDR35	WD repeat domain 35	chr2p24.1	57539	WDR35	WD repeat domain 35	---	716638	MmugDNA.15233.1.S1_at_WDR35
MmugDNA.15234.1.S1_at	202368_s_at	TRAM2	translocation associated membrane protein 2	chr6p21.1-p12	9697	LOC708479	Hypothetical protein LOC708479	---	708479	MmugDNA.15234.1.S1_at_TRAM2
MmugDNA.15235.1.S1_at	240208_at	---	---	---	---	---	---	---	---	MmugDNA.15235.1.S1_at_---
MmugDNA.15236.1.S1_at	240898_at	SPAG16	sperm associated antigen 16	chr2q34	79582	TIMM17A	Translocase of inner mitochondrial membrane 17 A-like protein	---	707001	MmugDNA.15236.1.S1_at_SPAG16
MmugDNA.15237.1.S1_at	1566860_at	LOC145663	hypothetical protein LOC145663	chr15q15.1	145663	---	---	---	---	MmugDNA.15237.1.S1_at_LOC145663
MmugDNA.15238.1.S1_at	206819_at	POM121L9P	POM121 membrane glycoprotein-like 9 (rat) pseudogene	chr22q11.22	29774	---	---	---	---	MmugDNA.15238.1.S1_at_POM121L9P
MmugDNA.15239.1.S1_at	204706_at	INPP5E	inositol polyphosphate-5-phosphatase, 72 kDa	chr9q34.3	56623	LOC710121	similar to inositol polyphosphate-5-phosphatase E	---	710121	MmugDNA.15239.1.S1_at_INPP5E
MmugDNA.15240.1.S1_at	206619_at	DKK4	dickkopf homolog 4 (Xenopus laevis)	chr8p11.2-p11.1	27121	DKK4	dickkopf homolog 4 (Xenopus laevis)	---	705011	MmugDNA.15240.1.S1_at_DKK4
MmugDNA.15241.1.S1_at	218599_at	REC8	REC8 homolog (yeast)	chr14q11.2-q12	9985	REC8	REC8 homolog (yeast)	---	715430	MmugDNA.15241.1.S1_at_REC8
MmugDNA.1524.1.S1_at	224609_at	SLC44A2	solute carrier family 44, member 2	chr19p13.1	57153	LOC713365 /// LOC7158	hypothetical protein LOC713365 /// similar to CTL2 protein	---	713365 /// 715866	MmugDNA.1524.1.S1_at_SLC44A2
MmugDNA.15242.1.S1_at	NA	NA	NA	NA	NA	NA	NA	NA	NA	MmugDNA.15242.1.S1_at_NA
MmugDNA.15243.1.S1_at	230285_at	SVIP	small VCP/p97-interacting protein	chr11p14.2	258010	SVIP	small VCP/p97-interacting protein	---	700825	MmugDNA.15243.1.S1_at_SVIP
MmugDNA.15244.1.S1_at	223596_at	SLC12A6	solute carrier family 12 (potassium/chloride transporters), member 6	chr15q13-q15	9990	SLC12A6	solute carrier family 12 (potassium/chloride transporters), member 6	---	697536	MmugDNA.15244.1.S1_at_SLC12A6
MmugDNA.15245.1.S1_at	219350_s_at	DIABLO	diablo homolog (Drosophila)	chr12q24.31	56616	DIABLO	diablo homolog (Drosophila)	---	701896	MmugDNA.15245.1.S1_at_DIABLO
MmugDNA.15246.1.S1_at	1566691_at	---	---	---	---	---	---	---	---	MmugDNA.15246.1.S1_at_---
MmugDNA.15247.1.S1_at	234478_at	LOC440863	hypothetical gene supported by AK026773	chr2p16.3	440863	---	---	---	---	MmugDNA.15247.1.S1_at_LOC440863
MmugDNA.15250.1.S1_at	204962_s_at	CENPA	centromere protein A	chr2p24-p21	1058	---	---	---	---	MmugDNA.15250.1.S1_at_CENPA
MmugDNA.15251.1.S1_at	NA	NA	NA	NA	NA	NA	NA	NA	NA	MmugDNA.15251.1.S1_at_NA
MmugDNA.1525.1.S1_at	242377_x_at	THUMPD3	THUMP domain containing 3	chr3p25.3	25917	---	---	---	---	MmugDNA.1525.1.S1_at_THUMPD3
MmugDNA.1525.1.S1_x_at	NA	NA	NA	NA	NA	NA	NA	NA	NA	MmugDNA.1525.1.S1_x_at_NA
MmugDNA.15252.1.S1_at	206678_at	GABRA1	gamma-aminobutyric acid (GABA) A receptor, alpha 1	chr5q34-q35	2554	---	---	---	---	MmugDNA.15252.1.S1_at_GABRA1
MmugDNA.15253.1.S1_at	1557080_s_at	ITGBL1	integrin, beta-like 1 (with EGF-like repeat domains)	chr13q33	9358	---	---	---	---	MmugDNA.15253.1.S1_at_ITGBL1
MmugDNA.15254.1.S1_at	217072_at	CD300A	CD300a molecule	chr17q25.1	11314	---	---	---	---	MmugDNA.15254.1.S1_at_CD300A
MmugDNA.15255.1.S1_at	241328_at	ZMAT1	zinc finger, matrin type 1	chrXq21	84460	ZMAT1	zinc finger, matrin type 1	---	704367	MmugDNA.15255.1.S1_at_ZMAT1
MmugDNA.15256.1.S1_at	1561392_at	---	---	---	---	---	---	---	---	MmugDNA.15256.1.S1_at_---
MmugDNA.15257.1.S1_at	230210_at	UNC84A	unc-84 homolog A (C. elegans)	chr7p22.3	23353	LOC708786	similar to unc-84 homolog A	---	708786	MmugDNA.15257.1.S1_at_UNC84A
MmugDNA.15258.1.S1_at	NA	NA	NA	NA	NA	NA	NA	NA	NA	MmugDNA.15258.1.S1_at_NA
MmugDNA.15259.1.S1_at	202417_at	KEAP1	kelch-like ECH-associated protein 1	chr19p13.2	9817	LOC720514	similar to Kelch-like ECH-associated protein 1 (Cytosolic inhibitor of Nrf2) (Kelch-like protein 19)	---	720514	MmugDNA.15259.1.S1_at_KEAP1
MmugDNA.15260.1.S1_at	204817_at	ESPL1	extra spindle pole bodies homolog 1 (S. cerevisiae)	chr12q	9700	LOC707481	Hypothetical protein LOC707481	---	707481	MmugDNA.15260.1.S1_at_ESPL1
MmugDNA.15261.1.S1_at	219206_x_at	TMBIM4	transmembrane BAX inhibitor motif containing 4	chr12q14.1|12q14.1-q15	51643	TMBIM4	transmembrane BAX inhibitor motif containing 4	---	717825	MmugDNA.15261.1.S1_at_TMBIM4
MmugDNA.1526.1.S1_at	36829_at	PER1	period homolog 1 (Drosophila)	chr17p13.1-p12	5187	LOC721948	similar to period 1	---	721948	MmugDNA.1526.1.S1_at_PER1
MmugDNA.15262.1.S1_at	204681_s_at	RAPGEF5	Rap guanine nucleotide exchange factor (GEF) 5	chr7p15.3	9771	---	---	---	---	MmugDNA.15262.1.S1_at_RAPGEF5
MmugDNA.15263.1.S1_at	203853_s_at	GAB2	GRB2-associated binding protein 2	chr11q14.1	9846	GAB2	GRB2-associated binding protein 2	---	700197	MmugDNA.15263.1.S1_at_GAB2
MmugDNA.15264.1.S1_at	222874_s_at	CLN8	ceroid-lipofuscinosis, neuronal 8 (epilepsy, progressive with mental retardation)	chr8p23	2055	LOC716441	similar to CLN8 protein	---	716441	MmugDNA.15264.1.S1_at_CLN8
MmugDNA.15265.1.S1_at	211189_x_at	CD84	CD84 molecule	chr1q24	8832	LOC719738	similar to CD84 antigen (leukocyte antigen)	---	719738	MmugDNA.15265.1.S1_at_CD84
MmugDNA.15266.1.S1_at	223912_s_at	CLN8	ceroid-lipofuscinosis, neuronal 8 (epilepsy, progressive with mental retardation)	chr8p23	2055	---	---	---	---	MmugDNA.15266.1.S1_at_CLN8
MmugDNA.15267.1.S1_at	228802_at	RBPMS2	RNA binding protein with multiple splicing 2	chr15q22.31	348093	LOC712536	similar to RNA binding protein with multiple splicing 2	---	712536	MmugDNA.15267.1.S1_at_RBPMS2
MmugDNA.15268.1.S1_s_at	NA	NA	NA	NA	NA	NA	NA	NA	NA	MmugDNA.15268.1.S1_s_at_NA
MmugDNA.15269.1.S1_at	219340_s_at	CLN8	ceroid-lipofuscinosis, neuronal 8 (epilepsy, progressive with mental retardation)	chr8p23	2055	---	---	---	---	MmugDNA.15269.1.S1_at_CLN8
MmugDNA.15270.1.S1_at	237703_at	---	---	---	---	---	---	---	---	MmugDNA.15270.1.S1_at_---
MmugDNA.15271.1.S1_at	207845_s_at	ANAPC10	anaphase promoting complex subunit 10	chr4q31	10393	---	---	---	---	MmugDNA.15271.1.S1_at_ANAPC10
MmugDNA.1527.1.S1_at	206351_s_at	PEX10	peroxisomal biogenesis factor 10	chr1p36.32	5192	PEX10	peroxisomal biogenesis factor 10	---	694412	MmugDNA.1527.1.S1_at_PEX10
MmugDNA.15272.1.S1_at	222149_x_at	GOLGA8C /// GOLGA8	golgi autoantigen, golgin subfamily a, 8C /// golgi autoantigen, golgin subfamily a, 8D /// golgi autoanti	chr15q11.2 /// chr15q13.1	100132565 /// 1001329	---	---	---	---	MmugDNA.15272.1.S1_at_GOLGA8C //
MmugDNA.15273.1.S1_at	242114_at	BOLA2	BolA homolog 2 (E. coli)	chr16p11.2	552900	---	---	---	---	MmugDNA.15273.1.S1_at_BOLA2
MmugDNA.15274.1.S1_at	230014_at	---	---	---	---	---	---	---	---	MmugDNA.15274.1.S1_at_---
MmugDNA.15275.1.S1_at	225119_at	CHMP4B	chromatin modifying protein 4B	chr20q11.22	128866	LOC709004	similar to Charged multivesicular body protein 4b (Chromatin-modifying protein 4b) (CHMP4b) (Vacuo	---	709004	MmugDNA.15275.1.S1_at_CHMP4B
MmugDNA.15276.1.S1_at	213247_at	SVEP1	sushi, von Willebrand factor type A, EGF and pentraxin domain containing 1	chr9q32	79987	LOC709478	similar to sushi, von Willebrand factor type A, EGF and pentraxin domain containing 1	---	709478	MmugDNA.15276.1.S1_at_SVEP1
MmugDNA.15277.1.S1_at	223981_at	NIN	ninein (GSK3B interacting protein)	chr14q22.1	51199	---	---	---	---	MmugDNA.15277.1.S1_at_NIN
MmugDNA.15278.1.S1_at	231156_at	---	---	---	---	---	---	---	---	MmugDNA.15278.1.S1_at_---
MmugDNA.15279.1.S1_at	218074_at	FAM96B	family with sequence similarity 96, member B	chr16q22.1-q22.3	51647	LOC697403	similar to Protein FAM96B	---	697403	MmugDNA.15279.1.S1_at_FAM96B
MmugDNA.15280.1.S1_at	207702_s_at	MAGI2	membrane associated guanylate kinase, WW and PDZ domain containing 2	chr7q21	9863	---	---	---	---	MmugDNA.15280.1.S1_at_MAGI2
MmugDNA.15281.1.S1_at	235718_at	SRL	sarcalumenin	chr16p13.3	6345	LOC704655	similar to sarcalumenin	---	704655	MmugDNA.15281.1.S1_at_SRL
MmugDNA.1528.1.S1_at	241703_at	RUNDC3B	RUN domain containing 3B	chr7q21.12	154661	---	---	---	---	MmugDNA.1528.1.S1_at_RUNDC3B
MmugDNA.15282.1.S1_at	213698_at	ZMYM6	zinc finger, MYM-type 6	chr1p34.2	9204	LOC711477	hypothetical protein LOC711477	---	711477	MmugDNA.15282.1.S1_at_ZMYM6
MmugDNA.15283.1.S1_at	227762_at	---	---	---	---	---	---	---	---	MmugDNA.15283.1.S1_at_---
MmugDNA.15284.1.S1_at	NA	NA	NA	NA	NA	NA	NA	NA	NA	MmugDNA.15284.1.S1_at_NA
MmugDNA.15285.1.S1_at	206953_s_at	LPHN2	latrophilin 2	chr1p31.1	23266	LOC708998	similar to latrophilin 2 precursor	---	708998	MmugDNA.15285.1.S1_at_LPHN2
MmugDNA.15286.1.S1_at	224701_at	PARP14	poly (ADP-ribose) polymerase family, member 14	chr3q21.1	54625	LOC715591	similar to poly (ADP-ribose) polymerase family, member 14	---	715591	MmugDNA.15286.1.S1_at_PARP14
MmugDNA.15287.1.S1_at	244287_at	SFRS12	splicing factor, arginine/serine-rich 12	chr5q12.3	140890	SFRS12	splicing factor, arginine/serine-rich 12	---	697731	MmugDNA.15287.1.S1_at_SFRS12
MmugDNA.15288.1.S1_at	221324_at	TAS2R1	taste receptor, type 2, member 1	chr5p15	50834	TAS2R1	taste receptor, type 2, member 1	---	696264	MmugDNA.15288.1.S1_at_TAS2R1
MmugDNA.15289.1.S1_at	223067_at	CWC15	CWC15 spliceosome-associated protein homolog (S. cerevisiae)	chr11q21	51503	CWC15	CWC15 spliceosome-associated protein homolog (S. cerevisiae)	---	698332	MmugDNA.15289.1.S1_at_CWC15
MmugDNA.15290.1.S1_at	211192_s_at	CD84	CD84 molecule	chr1q24	8832	LOC719738	similar to CD84 antigen (leukocyte antigen)	---	719738	MmugDNA.15290.1.S1_at_CD84
MmugDNA.15291.1.S1_at	203619_s_at	FAIM2	Fas apoptotic inhibitory molecule 2	chr12q13	23017	LOC711631	similar to Fas apoptotic inhibitory molecule 2	---	711631	MmugDNA.15291.1.S1_at_FAIM2
MmugDNA.1529.1.S1_at	205161_s_at	PEX11A	peroxisomal biogenesis factor 11 alpha	chr15q26.1	8800	PEX11A	peroxisomal biogenesis factor 11 alpha	---	700511	MmugDNA.1529.1.S1_at_PEX11A

MmugDNA.15292.1.S1_at	218654_s_at	MRPS33	mitochondrial ribosomal protein S33	chr7q32-q34	51650	MRPS33	mitochondrial ribosomal protein S33	---	693681	MmugDNA.15292.1.S1_at_MRPS33
MmugDNA.15293.1.S1_at	226960_at	CXCL17	chemokine (C-X-C motif) ligand 17	chr19q13.2	284340	LOC708108	similar to DMC	---	708108	MmugDNA.15293.1.S1_at_CXCL17
MmugDNA.15294.1.S1_at	236401_at	---	---	---	---	---	---	---	---	MmugDNA.15294.1.S1_at_---
MmugDNA.15295.1.S1_at	238273_at	PL-5283 /// SLC13A4	PL-5283 protein /// solute carrier family 13 (sodium/sulfate symporters), member 4	chr7q33	26266 /// 647087	LOC714071	hypothetical protein LOC714071	---	714071	MmugDNA.15295.1.S1_at_PL-5283 /// S
MmugDNA.15296.1.S1_at	217963_s_at	NGFRAP1	nerve growth factor receptor (TNFRSF16) associated protein 1	chrXq22.2	27018	---	---	---	---	MmugDNA.15296.1.S1_at_NGFRAP1
MmugDNA.15297.1.S1_at	1554649_at	---	---	---	---	---	---	---	---	MmugDNA.15297.1.S1_at_---
MmugDNA.15298.1.S1_at	NA	NA	NA	NA	NA	NA	NA	NA	NA	MmugDNA.15298.1.S1_at_NA
MmugDNA.15298.1.S1_x_at	202779_s_at	LOC731049 /// UBE2S	similar to Ubiquitin-conjugating enzyme E2S (Ubiquitin-conjugating enzyme E2-24 kDa) (Ubiquitin-pro	chr19q13.43	27338 /// 731049	UBE2S	ubiquitin-conjugating enzyme E2S	---	700592	MmugDNA.15298.1.S1_x_at_LOC73104
MmugDNA.15299.1.S1_at	217850_at	GNL3	guanine nucleotide binding protein-like 3 (nucleolar)	chr3p21.1	26354	GNL3	guanine nucleotide binding protein-like 3 (nucleolar)	---	695877	MmugDNA.15299.1.S1_at_GNL3
MmugDNA.15300.1.S1_at	217632_at	GNL3L	guanine nucleotide binding protein-like 3 (nucleolar)-like	chrXp11.22	54552	---	---	---	---	MmugDNA.15300.1.S1_at_GNL3L
MmugDNA.15301.1.S1_at	225497_at	ATE1	arginyltransferase 1	chr10q26.13	11101	---	---	---	---	MmugDNA.15301.1.S1_at_ATE1
MmugDNA.1530.1.S1_at	1566210_at	---	---	---	---	---	---	---	---	MmugDNA.1530.1.S1_at_---
MmugDNA.15302.1.S1_s_at	220942_x_at	FAM162A	family with sequence similarity 162, member A	chr3q21.1	26355	LOC714652	similar to growth and transformation-dependent protein	---	714652	MmugDNA.15302.1.S1_s_at_FAM162A
MmugDNA.15303.1.S1_at	NA	NA	NA	NA	NA	NA	NA	NA	NA	MmugDNA.15303.1.S1_at_NA
MmugDNA.15303.1.S1_s_at	224303_x_at	NIN	ninein (GSK3B interacting protein)	chr14q22.1	51199	LOC709532	similar to ninein isoform 5	---	709532	MmugDNA.15303.1.S1_s_at_NIN
MmugDNA.15305.1.S1_at	225898_at	WDR54	WD repeat domain 54	chr2p13.1	84058	LOC708605	similar to WD repeat domain 54	---	708605	MmugDNA.15305.1.S1_at_WDR54
MmugDNA.15306.1.S1_at	1552829_at	---	---	---	---	---	---	---	---	MmugDNA.15306.1.S1_at_---
MmugDNA.15307.1.S1_at	229598_at	COBLL1	COBL-like 1	chr2q24.3	22837	---	---	---	---	MmugDNA.15307.1.S1_at_COBLL1
MmugDNA.15308.1.S1_at	NA	NA	NA	NA	NA	NA	NA	NA	NA	MmugDNA.15308.1.S1_at_NA
MmugDNA.15308.1.S1_s_at	224755_at	TM9SF3	transmembrane 9 superfamily member 3	chr10q24.1	56889	---	---	---	---	MmugDNA.15308.1.S1_s_at_TM9SF3
MmugDNA.15309.1.S1_at	217837_s_at	VPS24	vacuolar protein sorting 24 homolog (S. cerevisiae)	chr2p24.3-p24.1	51652	---	---	---	---	MmugDNA.15309.1.S1_at_VPS24
MmugDNA.15310.1.S1_at	200897_s_at	PALLD	palladin, cytoskeletal associated protein	chr4q32.3	23022	PALLD	palladin, cytoskeletal associated protein	---	693824	MmugDNA.15310.1.S1_at_PALLD
MmugDNA.15311.1.S1_x_at	NA	NA	NA	NA	NA	NA	NA	NA	NA	MmugDNA.15311.1.S1_x_at_NA
MmugDNA.1531.1.S1_at	216989_at	SPAM1	sperm adhesion molecule 1 (PH-20 hyaluronidase, zona pellucida binding)	chr7q31.3	6677	---	---	---	---	MmugDNA.1531.1.S1_at_SPAM1
MmugDNA.15312.1.S1_at	1569466_at	C20orf152	chromosome 20 open reading frame 152	chr20q11.23	140894	---	---	---	---	MmugDNA.15312.1.S1_at_C20orf152
MmugDNA.15313.1.S1_at	237551_at	URB1	URB1 ribosome biogenesis 1 homolog (S. cerevisiae)	chr21q22.11	9875	---	---	---	---	MmugDNA.15313.1.S1_at_URB1
MmugDNA.15314.1.S1_at	204454_at	LDOC1	leucine zipper, down-regulated in cancer 1	chrXq27	23641	LDOC1	leucine zipper, down-regulated in cancer 1	---	696902	MmugDNA.15314.1.S1_at_LDOC1
MmugDNA.15315.1.S1_at	226424_at	CAPS	calcyphosine	chr19p13.3	828	LOC700767	similar to calcyphosine isoform b	---	700767	MmugDNA.15315.1.S1_at_CAPS
MmugDNA.15316.1.S1_at	213865_at	DCBLD2	discoidin, CUB and LCCL domain containing 2	chr3q12.1|3	131566	LOC700151	similar to discoidin, CUB and LCCL domain containing 2	---	700151	MmugDNA.15316.1.S1_at_DCBLD2
MmugDNA.15317.1.S1_at	235520_at	ZNF280C	zinc finger protein 280C	chrXq25	55609	---	---	---	---	MmugDNA.15317.1.S1_at_ZNF280C
MmugDNA.15318.1.S1_at	205341_at	EHD2	EH-domain containing 2	chr19q13.3	30846	LOC717477	similar to EH-domain containing 2	---	717477	MmugDNA.15318.1.S1_at_EHD2
MmugDNA.15319.1.S1_at	218935_at	EHD3	EH-domain containing 3	chr2p21	30845	LOC705316	similar to EH-domain containing 3	---	705316	MmugDNA.15319.1.S1_at_EHD3
MmugDNA.153.1.S1_at	NA	NA	NA	NA	NA	NA	NA	NA	NA	MmugDNA.153.1.S1_at_NA
MmugDNA.153.1.S1_s_at	213165_at	CEP350	centrosomal protein 350kDa	chr1p36.13-q41	9857	---	---	---	---	MmugDNA.153.1.S1_s_at_CEP350
MmugDNA.15320.1.S1_at	1568874_at	NCOA6	nuclear receptor coactivator 6	chr20q11	23054	---	---	---	---	MmugDNA.15320.1.S1_at_NCOA6
MmugDNA.15321.1.S1_at	232000_at	TTC39B	Tetratricopeptide repeat domain 39B	chr9p22.3	158219	---	---	---	---	MmugDNA.15321.1.S1_at_TTC39B
MmugDNA.1532.1.S1_at	238457_at	DGKE	diacylglycerol kinase, epsilon 64kDa	chr17q22	8526	---	---	---	---	MmugDNA.1532.1.S1_at_DGKE
MmugDNA.15322.1.S1_at	239848_at	---	---	---	---	---	---	---	---	MmugDNA.15322.1.S1_at_---
MmugDNA.15324.1.S1_at	212940_at	COL6A1	collagen, type VI, alpha 1	chr21q22.3	1291	---	---	---	---	MmugDNA.15324.1.S1_at_COL6A1
MmugDNA.15325.1.S1_at	212938_at	COL6A1	collagen, type VI, alpha 1	chr21q22.3	1291	LOC721855	similar to Collagen alpha-1(VI) chain precursor	---	721855	MmugDNA.15325.1.S1_at_COL6A1
MmugDNA.15326.1.S1_at	234656_x_at	---	---	---	---	---	---	---	---	MmugDNA.15326.1.S1_at_---
MmugDNA.15327.1.S1_at	1561314_at	---	---	---	---	LOC698240	hypothetical protein LOC698240	---	698240	MmugDNA.15327.1.S1_at_---
MmugDNA.15328.1.S1_at	228233_at	FREM1	FRAS1 related extracellular matrix 1	chr9p22.3	158326	FREM1	FRAS1 related extracellular matrix 1	---	713874	MmugDNA.15328.1.S1_at_FREM1
MmugDNA.15329.1.S1_at	219329_s_at	C2orf28	chromosome 2 open reading frame 28	chr2p23.3	51374	C13H2orf28	chromosome 2 open reading frame 28 ortholog	---	699201	MmugDNA.15329.1.S1_at_C2orf28
MmugDNA.15330.1.S1_at	233663_s_at	CDH26	cadherin-like 26	chr20q13.2-q13.33	60437	CDH26	cadherin-like 26	---	701165	MmugDNA.15330.1.S1_at_CDH26
MmugDNA.15331.1.S1_s_at	218939_at	LETM1	leucine zipper-EF-hand containing transmembrane protein 1	chr4p16.3	3954	---	---	---	---	MmugDNA.15331.1.S1_s_at_LETM1
MmugDNA.1533.1.S1_at	217478_s_at	HLA-DMA	major histocompatibility complex, class II, DM alpha	chr6p21.3	3108	LOC717870	similar to HLA class II histocompatibility antigen, DM beta chain precursor (MHC class II antigen DME	---	717870	MmugDNA.1533.1.S1_at_HLA-DMA
MmugDNA.15333.1.S1_at	214630_at	CYP11B2	cytochrome P450, family 11, subfamily B, polypeptide 2	chr8q21-q22	1585	LOC695914	cytochrome P450, subfamily XIB polypeptide 2	---	695914	MmugDNA.15333.1.S1_at_CYP11B2
MmugDNA.15334.1.S1_s_at	225414_at	RNF149	ring finger protein 149	chr2q11.2	284996	LOC710926	similar to ring finger protein 149	---	710926	MmugDNA.15334.1.S1_s_at_RNF149
MmugDNA.15335.1.S1_at	239361_at	---	---	---	---	---	---	---	---	MmugDNA.15335.1.S1_at_---
MmugDNA.15336.1.S1_at	212924_s_at	LSM4	LSM4 homolog, U6 small nuclear RNA associated (S. cerevisiae)	chr19p13.11	25804	LOC720192	similar to U6 snRNA-associated Sm-like protein LSm4 (Glycine-rich protein) (GRP)	---	720192	MmugDNA.15336.1.S1_at_LSM4
MmugDNA.15337.1.S1_s_at	202590_s_at	PDK2	pyruvate dehydrogenase kinase, isozyme 2	chr17q21.33	5164	LOC699222	Hypothetical protein LOC699222	---	699222	MmugDNA.15337.1.S1_s_at_PDK2
MmugDNA.15338.1.S1_at	240514_at	PGA3	Pepsinogen 3, group I (pepsinogen A)	chr11q12.2	643834	---	---	---	---	MmugDNA.15338.1.S1_at_PGA3
MmugDNA.15339.1.S1_at	202904_s_at	LSM5	LSM5 homolog, U6 small nuclear RNA associated (S. cerevisiae)	chr7p14.3	23658	LSM5	LSM5 homolog, U6 small nuclear RNA associated (S. cerevisiae)	---	693550	MmugDNA.15339.1.S1_at_LSM5
MmugDNA.15340.1.S1_at	1563502_at	ZDHHC2	Zinc finger, DHHC-type containing 2	chr8p21.3-p22	51201	---	---	---	---	MmugDNA.15340.1.S1_at_ZDHHC2
MmugDNA.15341.1.S1_at	232721_at	TRIM55	tripartite motif-containing 55	chr8q13.1	84675	LOC711707	similar to ring finger protein 29 isoform 2	---	711707	MmugDNA.15341.1.S1_at_TRIM55
MmugDNA.1534.1.S1_at	1558279_a_at	KDSR	3-ketodihydrosphingosine reductase	chr18q21.3	2531	KDSR	3-ketodihydrosphingosine reductase	---	700476	MmugDNA.1534.1.S1_at_KDSR
MmugDNA.15342.1.S1_at	227168_at	MIAT	myocardial infarction associated transcript (non-protein coding)	chr22q12.1	440823	LOC704767	Similar to MAP6 domain containing 1	---	704767	MmugDNA.15342.1.S1_at_MIAT
MmugDNA.15343.1.S1_at	223457_at	COPG2	coatomer protein complex, subunit gamma 2	chr7q32	26958	LOC707413	similar to Coatomer subunit gamma-2 (Gamma-2 coat protein) (Gamma-2 COP)	---	707413	MmugDNA.15343.1.S1_at_COPG2
MmugDNA.15344.1.S1_at	222386_s_at	COPZ1	coatomer protein complex, subunit zeta 1	chr12q13.2-q13.3	22818	LOC705176	similar to Coatomer subunit zeta-1 (Zeta-1 coat protein) (Zeta-1 COP)	---	705176	MmugDNA.15344.1.S1_at_COPZ1
MmugDNA.15345.1.S1_at	244783_at	YAF2	YY1 associated factor 2	chr12q12	10138	LOC702878	Similar to collagen, type XIV, alpha 1	---	702878	MmugDNA.15345.1.S1_at_YAF2
MmugDNA.15346.1.S1_at	NA	NA	NA	NA	NA	NA	NA	NA	NA	MmugDNA.15346.1.S1_at_NA
MmugDNA.15347.1.S1_at	243726_at	---	---	---	---	---	---	---	---	MmugDNA.15347.1.S1_at_---
MmugDNA.15348.1.S1_at	218498_s_at	ERO1L	ERO1-like (S. cerevisiae)	chr14q22.1	30001	ERO1L	ERO1-like	---	708329	MmugDNA.15348.1.S1_at_ERO1L
MmugDNA.15349.1.S1_s_at	219300_s_at	CNTNAP2	contactin associated protein-like 2	chr7q35-q36	26047	---	---	---	---	MmugDNA.15349.1.S1_s_at_CNTNAP2
MmugDNA.15350.1.S1_at	1555447_at	GPR114	G protein-coupled receptor 114	chr16q13	221188	LOC711459	similar to G-protein coupled receptor 114	---	711459	MmugDNA.15350.1.S1_at_GPR114
MmugDNA.15351.1.S1_at	219301_s_at	CNTNAP2	contactin associated protein-like 2	chr7q35-q36	26047	---	---	---	---	MmugDNA.15351.1.S1_at_CNTNAP2
MmugDNA.1535.1.S1_at	1563022_at	LOC347475	UPF0625 coiled-coil domain-containing protein ENSP00000359845	chrXq26.2	347475	LOC707070	hypothetical protein LOC707070	---	707070	MmugDNA.1535.1.S1_at_LOC347475
MmugDNA.15352.1.S1_at	NA	NA	NA	NA	NA	NA	NA	NA	NA	MmugDNA.15352.1.S1_at_NA
MmugDNA.15352.1.S1_s_at	224129_s_at	DPY30	dpy-30 homolog (C. elegans)	chr2p22.3	84661	DPY30	dpy-30 homolog (C. elegans)	---	706155	MmugDNA.15352.1.S1_s_at_DPY30
MmugDNA.15353.1.S1_at	227444_at	ARMCX4	Armadillo repeat containing, X-linked 4	chrXq22.1	158947	LOC704513	similar to ALEX3 protein	---	704513	MmugDNA.15353.1.S1_at_ARMCX4
MmugDNA.15354.1.S1_at	220556_at	ATP1B4	ATPase, (Na+)/K+ transporting, beta 4 polypeptide	chrXq24	23439	ATP1B4	ATPase, (Na+)/K+ transporting, beta 4 polypeptide	---	696160	MmugDNA.15354.1.S1_at_ATP1B4
MmugDNA.15355.1.S1_at	NA	NA	NA	NA	NA	NA	NA	NA	NA	MmugDNA.15355.1.S1_at_NA
MmugDNA.15355.1.S1_s_at	229120_s_at	CDC42SE1	CDC42 small effector 1	chr1q21.2	56882	LOC713638	Similar to S100 calcium binding protein A16	---	713638	MmugDNA.15355.1.S1_s_at_CDC42SE
MmugDNA.15356.1.S1_at	217621_at	---	---	---	---	---	---	---	---	MmugDNA.15356.1.S1_at_---
MmugDNA.15357.1.S1_at	205050_s_at	MAPK8IP2	mitogen-activated protein kinase 8 interacting protein 2	chr22q13.33	23542	MAPK8IP2	mitogen-activated protein kinase 8 interacting protein 2	---	720708	MmugDNA.15357.1.S1_at_MAPK8IP2
MmugDNA.15358.1.S1_at	201857_at	ZFR	zinc finger RNA binding protein	chr5p13.3	51663	LOC696882	similar to zinc finger RNA binding protein	---	696882	MmugDNA.15358.1.S1_at_ZFR
MmugDNA.15359.1.S1_at	NA	NA	NA	NA	NA	NA	NA	NA	NA	MmugDNA.15359.1.S1_at_NA
MmugDNA.15359.1.S1_s_at	200713_s_at	MAPRE1	microtubule-associated protein, RP/EB family, member 1	chr20q11.1-q11.23	22919	MAPRE1	microtubule-associated protein, RP/EB family, member 1	---	711404	MmugDNA.15359.1.S1_s_at_MAPRE1
MmugDNA.15360.1.S1_at	1554127_s_at	MSRB3	methionine sulfoxide reductase B3	chr12q14.3	253827	LOC717617	similar to methionine sulfoxide reductase B3 isoform 2	---	717617	MmugDNA.15360.1.S1_at_MSRB3
MmugDNA.15360.1.S1_s_at	1554126_at	MSRB3	methionine sulfoxide reductase B3	chr12q14.3	253827	LOC717617	similar to methionine sulfoxide reductase B3 isoform 2	---	717617	MmugDNA.15360.1.S1_s_at_MSRB3
MmugDNA.15361.1.S1_at	219048_at	PIGN	phosphatidylinositol glycan anchor biosynthesis, class N	chr18q21.33	23556	LOC699219	similar to phosphatidylinositol glycan, class N	---	699219	MmugDNA.15361.1.S1_at_PIGN
MmugDNA.1536.1.S1_at	237548_at	---	---	---	---	---	---	---	---	MmugDNA.1536.1.S1_at_---
MmugDNA.15362.1.S1_at	213069_at	HEG1	HEG homolog 1 (zebrafish)	chr3q21.2	57493	---	---	---	---	MmugDNA.15362.1.S1_at_HEG1
MmugDNA.15364.1.S1_at	219032_x_at	OPN3	opsin 3	chr1q43	23596	OPN3	opsin 3	---	707436	MmugDNA.15364.1.S1_at_OPN3
MmugDNA.15365.1.S1_at	217530_at	SLC34A1	solute carrier family 34 (sodium phosphate), member 1	chr5q35	6569	SLC34A2	similar to solute carrier family 34 (sodium phosphate), member 1	---	699746	MmugDNA.15365.1.S1_at_SLC34A1
MmugDNA.15366.1.S1_s_at	200751_s_at	HNRNPC	heterogeneous nuclear ribonucleoprotein C (C1/C2)	chr14q11.2	3183	HNRNPC	heterogeneous nuclear ribonucleoprotein C (C1/C2)	---	706423	MmugDNA.15366.1.S1_s_at_HNRNPC
MmugDNA.15368.1.S1_at	1554813_at	---	---	---	---	---	---	---	---	MmugDNA.15368.1.S1_at_---
MmugDNA.15369.1.S1_at	228034_x_at	ALKBH5	alkB, alkylation repair homolog 5 (E. coli)	chr17p11.2	54890	---	---	---	---	MmugDNA.15369.1.S1_at_ALKBH5
MmugDNA.15370.1.S1_at	219432_at	EVC	Ellis van Creveld syndrome	chr4p16	2121	---	---	---	---	MmugDNA.15370.1.S1_at_EVC
MmugDNA.15371.1.S1_at	233632_s_at	XRN1	5'-3' exoribonuclease 1	chr3q23	54464	---	---	---	---	MmugDNA.15371.1.S1_at_XRN1
MmugDNA.1537.1.S1_at	215216_at	VPS16A	vacuolar protein sorting 16 homolog A (S. cerevisiae)	chr20p13-p12	64601	---	---	---	---	MmugDNA.1537.1.S1_at_VPS16A
MmugDNA.15372.1.S1_at	239517_at	ITGB6	Integrin, beta 6	chr2q24.2	3694	---	---	---	---	MmugDNA.15372.1.S1_at_ITGB6
MmugDNA.15373.1.S1_at	222186_at	ZFAND6	Zinc finger, AN1-type domain 6	chr15q25.1	54469	---	---	---	---	MmugDNA.15373.1.S1_at_ZFAND6
MmugDNA.15374.1.S1_at	228633_s_at	CNTROB	centrobin, centrosomal BRCA2 interacting protein	chr17p13.1	116840	LOC716660	similar to LYST-interacting protein LIP8 isoform beta	---	716660	MmugDNA.15374.1.S1_at_CNTROB
MmugDNA.15375.1.S1_at	1563088_a_at	LOC284837	hypothetical LOC284837	chr21q22.3	284837	---	---	---	---	MmugDNA.15375.1.S1_at_LOC284837
MmugDNA.15376.1.S1_at	1562972_at	LOC503519	hypothetical LOC503519	chr15q11.2	503519	---	---	---	---	MmugDNA.15376.1.S1_at_LOC503519
MmugDNA.15377.1.S1_at	1553047_at	PIP4K2B	phosphatidylinositol-5-phosphate 4-kinase, type II, beta	chr17q12	8396	PIP4K2B	similar to phosphatidylinositol-4-phosphate 5-kinase, type II, beta	---	695366	MmugDNA.15377.1.S1_at_PIP4K2B
MmugDNA.15378.1.S1_at	NA	NA	NA	NA	NA	NA	NA	NA	NA	MmugDNA.15378.1.S1_at_NA
MmugDNA.15379.1.S1_at	226086_at	SYT13	synaptotagmin XIII	chr11p12-p11	57586	---	---	---	---	MmugDNA.15379.1.S1_at_SYT13
MmugDNA.15380.1.S1_at	221395_at	TAS2R13	taste receptor, type 2, member 13	chr12p13	50838	TAS2R13	taste receptor, type 2, member 13	---	718046	MmugDNA.15380.1.S1_at_TAS2R13
MmugDNA.15381.1.S1_at	234728_s_at	DHX35	DEAH (Asp-Glu-Ala-His) box polypeptide 35	chr20q11.22-q12	60625	DHX35	DEAH (Asp-Glu-Ala-His) box polypeptide 35	---	697673	MmugDNA.15381.1.S1_at_DHX35
MmugDNA.1538.1.S1_at	225625_at	ALKBH2	alkB, alkylation repair homolog 2 (E. coli)	chr12q24.11	121642	LOC706708	similar to alkB, alkylation repair homolog 2	---	706708	MmugDNA.1538.1.S1_at_ALKBH2
MmugDNA.15382.1.S1_at	217571_at	---	---	---	---	---	---	---	---	MmugDNA.15382.1.S1_at_---
MmugDNA.15383.1.S1_at	232520_s_at	NSFL1C	NSFL1 (p97) cofactor (p47)	chr20p13	55968	LOC715743	similar to p47 protein isoform a	---	715743	MmugDNA.15383.1.S1_at_NSFL1C
MmugDNA.15383.1.S1_s_at	220248_x_at	NSFL1C	NSFL1 (p97) cofactor (p47)	chr20p13	55968	LOC715743	similar to p47 protein isoform a	---	715743	MmugDNA.15383.1.S1_s_at_NSFL1C
MmugDNA.15384.1.S1_at	239779_at	---	---	---	---	---	---	---	---	MmugDNA.15384.1.S1_at_---
MmugDNA.15385.1.S1_at	1553539_at	KRT74	keratin 74	chr12q13.13	121391	LOC699069	similar to keratin 6 irs4	---	699069	MmugDNA.15385.1.S1_at_KRT74

MmugDNA.15386.1.S1_s_at	215691_x_at	HSPB11	heat shock protein family B (small), member 11	chr1p32.1-p33	51668	LOC714791	similar to Placental protein 25 (PP25)	---	714791	MmugDNA.15386.1.S1_s_at_HSPB11
MmugDNA.15387.1.S1_at	1557071_s_at	NUB1	negative regulator of ubiquitin-like proteins 1	chr7q36	51667	---	---	---	---	MmugDNA.15387.1.S1_at_NUB1
MmugDNA.15388.1.S1_at	228896_at	---	---	---	---	---	---	---	---	MmugDNA.15388.1.S1_at_---
MmugDNA.15389.1.S1_at	200847_s_at	TMEM66	transmembrane protein 66	chr8p12	51669	TMEM66	transmembrane protein 66	---	693452	MmugDNA.15389.1.S1_at_TMEM66
MmugDNA.15390.1.S1_s_at	208454_s_at	PGCP	plasma glutamate carboxypeptidase	chr8q22.2	10404	PGCP	plasma glutamate carboxypeptidase	---	701711	MmugDNA.15390.1.S1_s_at_PGCP
MmugDNA.15391.1.S1_at	239092_at	ITGA8	integrin, alpha 8	chr10p13	8516	---	---	---	---	MmugDNA.15391.1.S1_at_ITGA8
MmugDNA.1539.1.S1_at	217713_x_at	---	---	---	---	---	---	---	---	MmugDNA.1539.1.S1_at_---
MmugDNA.15392.1.S1_at	1562226_at	VWDE	von Willebrand factor D and EGF domains	chr7p21.3	221806	---	---	---	---	MmugDNA.15392.1.S1_at_VWDE
MmugDNA.15393.1.S1_at	NA	NA	NA	NA	NA	NA	NA	NA	NA	MmugDNA.15393.1.S1_at_NA
MmugDNA.15394.1.S1_at	236955_at	---	---	---	---	---	---	---	---	MmugDNA.15394.1.S1_at_---
MmugDNA.15395.1.S1_s_at	210380_s_at	CACNA1G	calcium channel, voltage-dependent, T type, alpha 1G subunit	chr17q22	8913	---	---	---	---	MmugDNA.15395.1.S1_s_at_CACNA1G
MmugDNA.15396.1.S1_at	230938_x_at	ATF5	activating transcription factor 5	chr19q13.3	22809	LOC719150	similar to activating transcription factor 5	---	719150	MmugDNA.15396.1.S1_at_ATF5
MmugDNA.15397.1.S1_at	225045_at	CCDC88A	coiled-coil domain containing 88A	chr2p16.1	55704	LOC717103	similar to Hook-related protein 1	---	717103	MmugDNA.15397.1.S1_at_CCDC88A
MmugDNA.15398.1.S1_at	238029_s_at	SLC16A14	solute carrier family 16, member 14 (monocarboxylic acid transporter 14)	chr2q36.3	151473	---	---	---	---	MmugDNA.15398.1.S1_at_SLC16A14
MmugDNA.15399.1.S1_at	224890_s_at	C7orf59	chromosome 7 open reading frame 59	chr7q22.1	389541	LOC719122	hypothetical protein LOC719122	---	719122	MmugDNA.15399.1.S1_at_C7orf59
MmugDNA.15400.1.S1_at	240793_at	TTN	Titin	chr2q31	7273	---	---	---	---	MmugDNA.15400.1.S1_at_TTN
MmugDNA.15401.1.S1_at	216689_x_at	ARHGAP1	Rho GTPase activating protein 1	chr11p12-q12	392	LOC698786	Similar to ribosomal protein L7-like 1	---	698786	MmugDNA.15401.1.S1_at_ARHGAP1
MmugDNA.1540.1.S1_at	226051_at	SELM	selenoprotein M	chr22q12.2	140606	SELM	selenoprotein M	---	718290	MmugDNA.1540.1.S1_at_SELM
MmugDNA.15402.1.S1_at	235482_at	LOC400960	hypothetical gene supported by BC040598	chr2p14	400960	---	---	---	---	MmugDNA.15402.1.S1_at_LOC400960
MmugDNA.15403.1.S1_at	1558409_at	---	---	---	---	---	---	---	---	MmugDNA.15403.1.S1_at_---
MmugDNA.15404.1.S1_at	219235_s_at	PHACTR4	phosphatase and actin regulator 4	chr1p35.3	65979	---	---	---	---	MmugDNA.15404.1.S1_at_PHACTR4
MmugDNA.15404.1.S1_s_at	NA	NA	NA	NA	NA	NA	NA	NA	NA	MmugDNA.15404.1.S1_s_at_NA
MmugDNA.15405.1.S1_at	1555377_at	OR4D2	olfactory receptor, family 4, subfamily D, member 2	chr17q22	124538	LOC709563	similar to Olfactory receptor 4D2 (Olfactory receptor OR17-24) (B-lymphocyte membrane protein BC2	---	709563	MmugDNA.15405.1.S1_at_OR4D2
MmugDNA.15406.1.S1_at	242525_at	---	---	---	---	---	---	---	---	MmugDNA.15406.1.S1_at_---
MmugDNA.15407.1.S1_at	214918_at	HNRNPM	heterogeneous nuclear ribonucleoprotein M	chr19p13.3-p13.2	4670	---	---	---	---	MmugDNA.15407.1.S1_at_HNRNPM
MmugDNA.15407.1.S1_x_at	NA	NA	NA	NA	NA	NA	NA	NA	NA	MmugDNA.15407.1.S1_x_at_NA
MmugDNA.15408.1.S1_at	241605_at	TRUB1	TruB pseudouridine (psi) synthase homolog 1 (E. coli)	chr10q25.3	142940	---	---	---	---	MmugDNA.15408.1.S1_at_TRUB1
MmugDNA.15409.1.S1_at	218876_at	TPPP3	tubulin polymerization-promoting protein family member 3	chr16q22.1	51673	---	---	---	---	MmugDNA.15409.1.S1_at_TPPP3
MmugDNA.15410.1.S1_at	217831_s_at	NSFL1C	NSFL1 (p97) cofactor (p47)	chr20p13	55968	LOC715743	similar to p47 protein isoform a	---	715743	MmugDNA.15410.1.S1_at_NSFL1C
MmugDNA.15411.1.S1_at	230336_at	---	---	---	---	---	---	---	---	MmugDNA.15411.1.S1_at_---
MmugDNA.1541.1.S1_at	242559_at	---	---	---	---	---	---	---	---	MmugDNA.1541.1.S1_at_---
MmugDNA.15412.1.S1_at	243757_at	LOC100129006	hypothetical protein LOC100129006	chr7q22.1	100129006	LOC694592	hypothetical protein LOC694592	---	694592	MmugDNA.15412.1.S1_at_LOC10012900
MmugDNA.15413.1.S1_s_at	218317_x_at	GIYD1 /// GIYD2	GIY-YIG domain containing 1 /// GIY-YIG domain containing 2	chr16p11.2	548593 /// 79008	LOC709225	similar to GIY-YIG domain containing 2 isoform 1	---	709225	MmugDNA.15413.1.S1_s_at_GIYD1 /// (
MmugDNA.15414.1.S1_at	1553112_s_at	CDK8	cyclin-dependent kinase 8	chr13q12	1024	CDK8	cyclin-dependent kinase 8	---	709761	MmugDNA.15414.1.S1_at_CDK8
MmugDNA.15415.1.S1_at	227478_at	SETBP1	SET binding protein 1	chr18q21.1	26040	---	---	---	---	MmugDNA.15415.1.S1_at_SETBP1
MmugDNA.15416.1.S1_at	241895_at	LOC440905	hypothetical LOC440905	chr2q21.1	440905	LOC721336	similar to male sterility domain containing 1	---	721336	MmugDNA.15416.1.S1_at_LOC440905
MmugDNA.15417.1.S1_at	NA	NA	NA	NA	NA	NA	NA	NA	NA	MmugDNA.15417.1.S1_at_NA
MmugDNA.15417.1.S1_s_at	1553113_s_at	CDK8	cyclin-dependent kinase 8	chr13q12	1024	CDK8	cyclin-dependent kinase 8	---	709761	MmugDNA.15417.1.S1_s_at_CDK8
MmugDNA.15418.1.S1_at	221404_at	IL1F6	interleukin 1 family, member 6 (epsilon)	chr2q12-q14.1	27179	IL1F6	interleukin 1 family, member 6 (epsilon)	---	705136	MmugDNA.15418.1.S1_at_IL1F6
MmugDNA.15419.1.S1_at	221470_s_at	IL1F7	interleukin 1 family, member 7 (zeta)	chr2q12-q14.1	27178	LOC700579	similar to interleukin 1 family, member 7 isoform 1 proprotein	---	700579	MmugDNA.15419.1.S1_at_IL1F7
MmugDNA.154.1.S1_at	207125_at	ZNF225	zinc finger protein 225	chr19q13.2	7768	ZNF225	zinc finger protein 225	---	711905	MmugDNA.154.1.S1_at_ZNF225
MmugDNA.15420.1.S1_at	243575_at	LOC100128443 /// MAS	hypothetical protein LOC100128443 /// microtubule associated serine/threonine kinase family membe	chr5q12.3	100128443 /// 375449	MAST2	microtubule associated serine/threonine kinase 2	---	701878	MmugDNA.15420.1.S1_at_LOC100128
MmugDNA.15421.1.S1_at	243616_at	---	---	---	---	---	---	---	---	MmugDNA.15421.1.S1_at_---
MmugDNA.1542.1.S1_at	244646_at	---	---	---	---	---	---	---	---	MmugDNA.1542.1.S1_at_---
MmugDNA.15422.1.S1_at	212543_at	AIM1	absent in melanoma 1	chr6q21	202	AIM1	absent in melanoma 1	---	697117	MmugDNA.15422.1.S1_at_AIM1
MmugDNA.15423.1.S1_at	228182_at	ADCY5	adenylate cyclase 5	chr3q13.2-q21	111	LOC715897	similar to adenylate cyclase 5	---	715897	MmugDNA.15423.1.S1_at_ADCY5
MmugDNA.15424.1.S1_at	218456_at	CAPRIN2	caprin family member 2	chr12p11	65981	---	---	---	---	MmugDNA.15424.1.S1_at_CAPRIN2
MmugDNA.15425.1.S1_at	232942_at	PYROXD2	pyridine nucleotide-disulphide oxidoreductase domain 2	chr10q24.2	84795	---	---	---	---	MmugDNA.15425.1.S1_at_PYROXD2
MmugDNA.15426.1.S1_at	218312_s_at	ZSCAN18	zinc finger and SCAN domain containing 18	chr19q13.43	65982	---	---	---	---	MmugDNA.15426.1.S1_at_ZSCAN18
MmugDNA.15427.1.S1_at	238613_at	ZAK	sterile alpha motif and leucine zipper containing kinase AZK	chr2q24.2	51776	---	---	---	---	MmugDNA.15427.1.S1_at_ZAK
MmugDNA.15428.1.S1_at	NA	NA	NA	NA	NA	NA	NA	NA	NA	MmugDNA.15428.1.S1_at_NA
MmugDNA.15428.1.S1_s_at	218434_s_at	AACS	acetoacetyl-CoA synthetase	chr12q24.31	65985	---	---	---	---	MmugDNA.15428.1.S1_s_at_AACS
MmugDNA.15429.1.S1_at	241913_at	---	---	---	---	---	---	---	---	MmugDNA.15429.1.S1_at_---
MmugDNA.15430.1.S1_at	222264_at	HNRNPUL2	heterogeneous nuclear ribonucleoprotein U-like 2	chr11q12.3	221092	LOC718602	similar to heterogeneous nuclear ribonucleoprotein U	---	718602	MmugDNA.15430.1.S1_at_HNRNPUL2
MmugDNA.15431.1.S1_at	213277_at	ZFP36L1	zinc finger protein 36, C3H type-like 1	chr14q22-q24	677	---	---	---	---	MmugDNA.15431.1.S1_at_ZFP36L1
MmugDNA.1543.1.S1_at	238977_at	MCM6	minichromosome maintenance complex component 6	chr2q21	4175	---	---	---	---	MmugDNA.1543.1.S1_at_MCM6
MmugDNA.15432.1.S1_at	215916_at	CHRNE	cholinergic receptor, nicotinic, epsilon	chr17p13-p12	1145	---	---	---	---	MmugDNA.15432.1.S1_at_CHRNE
MmugDNA.15433.1.S1_s_at	213284_at	ZFP36L1	zinc finger protein 36, C3H type-like 1	chr14q22-q24	677	LOC714309	hypothetical protein LOC714309	---	714309	MmugDNA.15433.1.S1_s_at_ZFP36L1
MmugDNA.15434.1.S1_at	243532_at	---	---	---	---	---	---	---	---	MmugDNA.15434.1.S1_at_---
MmugDNA.15435.1.S1_at	217135_x_at	---	---	---	---	---	---	---	---	MmugDNA.15435.1.S1_at_---
MmugDNA.15436.1.S1_at	223189_x_at	MLL5	myeloid/lymphoid or mixed-lineage leukemia 5 (trithorax homolog, Drosophila)	chr7q22.1	55904	MKLN1	Muskelin 1, intracellular mediator containing kelch motifs	---	704338	MmugDNA.15436.1.S1_at_MLL5
MmugDNA.15437.1.S1_at	240383_at	UBE2D3	ubiquitin-conjugating enzyme E2D 3 (UBC4/5 homolog, yeast)	chr4q24	7323	LOC717057	Similar to 60S ribosomal protein L12	---	717057	MmugDNA.15437.1.S1_at_UBE2D3
MmugDNA.15438.1.S1_at	240585_at	---	---	---	---	---	---	---	---	MmugDNA.15438.1.S1_at_---
MmugDNA.15439.1.S1_at	1554158_at	ZMYND11	zinc finger, MYND domain containing 11	chr10p14	10771	ZMYND11	zinc finger, MYND domain containing 11	---	708761	MmugDNA.15439.1.S1_at_ZMYND11
MmugDNA.15440.1.S1_at	237006_at	---	---	---	---	---	---	---	---	MmugDNA.15440.1.S1_at_---
MmugDNA.15441.1.S1_at	217481_x_at	---	---	---	---	---	---	---	---	MmugDNA.15441.1.S1_at_---
MmugDNA.1544.1.S1_at	204873_at	PEX1	peroxisomal biogenesis factor 1	chr7q21.2	5189	PEX1	peroxisomal biogenesis factor 1	---	702392	MmugDNA.1544.1.S1_at_PEX1
MmugDNA.15442.1.S1_at	1560298_at	---	---	---	---	---	---	---	---	MmugDNA.15442.1.S1_at_---
MmugDNA.15443.1.S1_at	219186_at	ZBTB7A	zinc finger and BTB domain containing 7A	chr19p13.3	51341	LOC721817	similar to Zinc finger and BTB domain-containing protein 7A (Leukemia/lymphoma related factor) (Fa	---	721817	MmugDNA.15443.1.S1_at_ZBTB7A
MmugDNA.15444.1.S1_at	243110_x_at	NPW	neuropeptide W	chr16p13.3	283869	LOC694262	similar to Neuropeptide W precursor (Preproprotein L8) (hPPL8)	---	694262	MmugDNA.15444.1.S1_at_NPW
MmugDNA.15445.1.S1_at	218820_at	C14orf132	chromosome 14 open reading frame 132	chr14q32.2	56967	---	---	---	---	MmugDNA.15445.1.S1_at_C14orf132
MmugDNA.15446.1.S1_at	222219_s_at	TLE6	transducin-like enhancer of split 6 (E(sp1) homolog, Drosophila)	chr19p13.3	79816	---	---	---	---	MmugDNA.15446.1.S1_at_TLE6
MmugDNA.15447.1.S1_at	1568803_at	---	---	---	---	---	---	---	---	MmugDNA.15447.1.S1_at_---
MmugDNA.15448.1.S1_at	236517_at	MEGF10	multiple EGF-like-domains 10	chr5q33	84466	MEGF10	multiple EGF-like-domains 10	---	703899	MmugDNA.15448.1.S1_at_MEGF10
MmugDNA.15449.1.S1_at	204917_s_at	MLLT3	myeloid/lymphoid or mixed-lineage leukemia (trithorax homolog, Drosophila); translocated to, 3	chr9p22	4300	MLLT3	myeloid/lymphoid or mixed-lineage leukemia (trithorax homolog, Drosophila); translocated to, 3	---	711732	MmugDNA.15449.1.S1_at_MLLT3
MmugDNA.15450.1.S1_at	1569818_at	---	---	---	---	---	---	---	---	MmugDNA.15450.1.S1_at_---
MmugDNA.15451.1.S1_at	1561242_at	---	---	---	---	---	---	---	---	MmugDNA.15451.1.S1_at_---
MmugDNA.1545.1.S1_at	236466_at	---	---	---	---	---	---	---	---	MmugDNA.1545.1.S1_at_---
MmugDNA.15452.1.S1_at	NA	NA	NA	NA	NA	NA	NA	NA	NA	MmugDNA.15452.1.S1_at_NA
MmugDNA.15453.1.S1_s_at	209956_s_at	CAMK2B	calcium/calmodulin-dependent protein kinase II beta	chr22q12|7p14.3-p14.1	816	LOC706977	similar to calcium/calmodulin-dependent protein kinase IIB isoform 1	---	706977	MmugDNA.15453.1.S1_s_at_CAMK2B
MmugDNA.15454.1.S1_at	1555691_a_at	KLRK1	killer cell lectin-like receptor subfamily K, member 1	chr12p13.2-p12.3	22914	NKG2D	NKG2D protein	---	574240	MmugDNA.15454.1.S1_at_KLRK1
MmugDNA.15455.1.S1_at	209253_at	SORBS3	sorbin and SH3 domain containing 3	chr8p21.3	10174	LOC693536	Similar to eukaryotic translation initiation factor 3, subunit 12	---	693536	MmugDNA.15455.1.S1_at_SORBS3
MmugDNA.15456.1.S1_at	240010_at	---	---	---	---	---	---	---	---	MmugDNA.15456.1.S1_at_---
MmugDNA.15457.1.S1_at	222141_at	KLHL22	kelch-like 22 (Drosophila)	chr22q11.21	84861	---	---	---	---	MmugDNA.15457.1.S1_at_KLHL22
MmugDNA.15458.1.S1_s_at	210756_s_at	NOTCH2	Notch homolog 2 (Drosophila)	chr1p13-p11	4853	LOC713798	similar to notch 2 preproprotein	---	713798	MmugDNA.15458.1.S1_at_NOTCH2
MmugDNA.15460.1.S1_at	223508_at	NOTCH1	Notch homolog 1, translocation-associated (Drosophila)	chr9q34.3	4851	---	---	---	---	MmugDNA.15460.1.S1_at_NOTCH1
MmugDNA.1546.1.S1_at	204545_at	PEX6	peroxisomal biogenesis factor 6	chr6p21.1	5190	PEX6	peroxisomal biogenesis factor 6	---	697597	MmugDNA.1546.1.S1_at_PEX6
MmugDNA.15462.1.S1_at	222713_s_at	FANCF	Fanconi anemia, complementation group F	chr11p15	2188	LOC704048	similar to Fanconi anemia, complementation group F	---	704048	MmugDNA.15462.1.S1_at_FANCF
MmugDNA.15463.1.S1_at	241036_at	---	---	---	---	---	---	---	---	MmugDNA.15463.1.S1_at_---
MmugDNA.15464.1.S1_at	231377_at	CXorf65	chromosome X open reading frame 65	chrXq13.1	158830	---	---	---	---	MmugDNA.15464.1.S1_at_CXorf65
MmugDNA.15465.1.S1_at	207731_at	---	---	---	---	---	---	---	---	MmugDNA.15465.1.S1_at_---
MmugDNA.15466.1.S1_at	225909_at	tcag7.1196	similar to GLI-Kruppel family member HKR1	chr7q36.1	728743	---	---	---	---	MmugDNA.15466.1.S1_at_tcag7.1196
MmugDNA.15467.1.S1_at	243540_at	---	---	---	---	---	---	---	---	MmugDNA.15467.1.S1_at_---
MmugDNA.15468.1.S1_at	225907_at	tcag7.1196	similar to GLI-Kruppel family member HKR1	chr7q36.1	728743	---	---	---	---	MmugDNA.15468.1.S1_at_tcag7.1196
MmugDNA.15469.1.S1_at	223778_at	KIF9	kinesin family member 9	chr3p21.31	64147	LOC711742	similar to kinesin family member 9 isoform 2	---	711742	MmugDNA.15469.1.S1_at_KIF9
MmugDNA.15470.1.S1_at	244339_at	---	---	---	---	---	---	---	---	MmugDNA.15470.1.S1_at_---
MmugDNA.15471.1.S1_at	207387_s_at	GK	glycerol kinase	chrXp21.3	2710	GK	glycerol kinase	---	705001	MmugDNA.15471.1.S1_at_GK
MmugDNA.1547.1.S1_at	NA	NA	NA	NA	NA	NA	NA	NA	NA	MmugDNA.1547.1.S1_at_NA
MmugDNA.1547.1.S1_s_at	228519_x_at	CIRBP	cold inducible RNA binding protein	chr19p13.3	1153	LOC706175	similar to cold inducible RNA binding protein	---	706175	MmugDNA.1547.1.S1_s_at_CIRBP
MmugDNA.15472.1.S1_at	223624_at	ANUBL1	AN1, ubiquitin-like, homolog (Xenopus laevis)	chr10q11.21	93550	LOC713181	Similar to heterogeneous nuclear ribonucleoprotein A0	---	713181	MmugDNA.15472.1.S1_at_ANUBL1
MmugDNA.15473.1.S1_at	208423_s_at	MSR1	macrophage scavenger receptor 1	chr8p22	4481	MSR1	macrophage scavenger receptor 1	---	709365	MmugDNA.15473.1.S1_at_MSR1
MmugDNA.15474.1.S1_at	224318_s_at	KIAA1310	KIAA1310	chr2p12-p11.2	55683	LOC706159	similar to CG8233-PC, isoform C	---	706159	MmugDNA.15474.1.S1_at_KIAA1310
MmugDNA.15475.1.S1_at	1561027_at	LOC646241	Hypothetical protein LOC646241	chr5p15.1	646241	---	---	---	---	MmugDNA.15475.1.S1_at_LOC646241
MmugDNA.15476.1.S1_at	227609_at	EPSTI1	epithelial stromal interaction 1 (breast)	chr13q13.3	94240	LOC700208	similar to epithelial stromal interaction 1 isoform 1	---	700208	MmugDNA.15476.1.S1_at_EPSTI1
MmugDNA.15477.1.S1_at	232469_x_at	chromosome 1 open rea	CDNA FLJ13821 fis, clone THYRO1000484	---	---	---	---	---	---	MmugDNA.15477.1.S1_at_chromosome
MmugDNA.15478.1.S1_at	233587_s_at	SIPA1L2	signal-induced proliferation-associated 1 like 2	chr1q42.2	57568	SIPA1L2	signal-induced proliferation-associated 1 like 2	---	641442	MmugDNA.15478.1.S1_at_SIPA1L2
MmugDNA.15479.1.S1_at	222145_at	---	---	---	---	---	---	---	---	MmugDNA.15479.1.S1_at_---
MmugDNA.15480.1.S1_at	220104_at	ZC3HAV1	zinc finger CCCH-type, antiviral 1	chr7q34	56829	---	---	---	---	MmugDNA.15480.1.S1_at_ZC3HAV1

MmugDNA.15481.1.S1_at	217810_x_at	LARS	leucyl-tRNA synthetase	chr5q32		51520	LARS	leucyl-tRNA synthetase	---		706792	MmugDNA.15481.1.S1_at_LARS
MmugDNA.1548.1.S1_at	1561328_at	---	---	---	---		---	---	---	---		MmugDNA.1548.1.S1_at_---
MmugDNA.15482.1.S1_at	219806_s_at	C11orf75	chromosome 11 open reading frame 75	chr11q13.3-q23.3		56935	LOC712524	similar to Protein FN5	---		712524	MmugDNA.15482.1.S1_at_C11orf75
MmugDNA.15483.1.S1_at	1561230_at	---	---	---	---		---	---	---	---		MmugDNA.15483.1.S1_at_---
MmugDNA.15484.1.S1_at	1559332_at	---	---	---	---		---	---	---	---		MmugDNA.15484.1.S1_at_---
MmugDNA.15485.1.S1_at	204836_at	GLDC	glycine dehydrogenase (decarboxylating)	chr9p22		2731	GLDC	glycine dehydrogenase (decarboxylating)	---		715563	MmugDNA.15485.1.S1_at_GLDC
MmugDNA.15486.1.S1_at	1570173_at	INTS7	integrator complex subunit 7	chr1p36.13-q42.3		25896	LOC694913	Similar to Prostaglandin E synthase 3 (Cytosolic prostaglandin E2 synthase) (cPGES) (Telomerase-b	---		694913	MmugDNA.15486.1.S1_at_INTS7
MmugDNA.15487.1.S1_at	213799_s_at	PTPRA	protein tyrosine phosphatase, receptor type, A	chr20p13		5786	BIRC6	Baculoviral IAP repeat-containing 6	---		709443	MmugDNA.15487.1.S1_at_PTPRA
MmugDNA.15488.1.S1_at	212616_at	CHD9	chromodomain helicase DNA binding protein 9	chr16q12.2		80205	LOC696367	similar to chromodomain helicase DNA binding protein 9	---		696367	MmugDNA.15488.1.S1_at_CHD9
MmugDNA.15489.1.S1_at	208390_s_at	GLP1R	glucagon-like peptide 1 receptor	chr6p21		2740	GLP1R	glucagon-like peptide 1 receptor	---		719548	MmugDNA.15489.1.S1_at_GLP1R
MmugDNA.15489.1.S1_s_at	211232_x_at	GLP1R	glucagon-like peptide 1 receptor	chr6p21		2740	GLP1R	glucagon-like peptide 1 receptor	---		719548	MmugDNA.15489.1.S1_s_at_GLP1R
MmugDNA.15490.1.S1_at	212615_at	CHD9	chromodomain helicase DNA binding protein 9	chr16q12.2		80205	HPS4	Hermansky-Pudlak syndrome 4	---		712549	MmugDNA.15490.1.S1_at_CHD9
MmugDNA.15491.1.S1_at	1556194_a_at	---	---	---	---		---	---	---	---		MmugDNA.15491.1.S1_at_---
MmugDNA.1549.1.S1_at	201732_s_at	CLCN3	chloride channel 3	chr4q33		1182	LOC694472	similar to chloride channel 3 isoform c	---		694472	MmugDNA.1549.1.S1_at_CLCN3
MmugDNA.15493.1.S1_at	239327_at	---	---	---	---		---	---	---	---		MmugDNA.15493.1.S1_at_---
MmugDNA.15494.1.S1_at	238746_at	PXMP4	peroxisomal membrane protein 4, 24kDa	chr20q11.22		11264	---	---	---	---		MmugDNA.15494.1.S1_at_PXMP4
MmugDNA.15495.1.S1_at	1557495_at	---	---	---	---		LOC707351	hypothetical protein LOC707351	---		707351	MmugDNA.15495.1.S1_at_---
MmugDNA.15496.1.S1_at	231857_s_at	AGBL5	ATP/GTP binding protein-like 5	chr2p23.3		60509	AGBL5	ATP/GTP binding protein-like 5	---		697940	MmugDNA.15496.1.S1_at_AGBL5
MmugDNA.15497.1.S1_at	234747_at	C4orf41	chromosome 4 open reading frame 41	chr4q35.1		60684	LOC708044	Hypothetical protein LOC708044	---		708044	MmugDNA.15497.1.S1_at_C4orf41
MmugDNA.15498.1.S1_at	240284_x_at	EMG1	EMG1 nucleolar protein homolog (S. cerevisiae)	chr12p13		10436	---	---	---	---		MmugDNA.15498.1.S1_at_EMG1
MmugDNA.15499.1.S1_at	232646_at	TTC17	Tetratricopeptide repeat domain 17	chr11p12-p11.2		55761	LOC701520	Similar to myosin IXA	---		701520	MmugDNA.15499.1.S1_at_TTC17
MmugDNA.15500.1.S1_at	231146_at	FAM24B	family with sequence similarity 24, member B	chr10q26.13		196792	CUZD1	CUB and zona pellucida-like domains 1	---		706861	MmugDNA.15500.1.S1_at_FAM24B
MmugDNA.15501.1.S1_at	221832_s_at	LUZP1	leucine zipper protein 1	chr1p36		7798	---	---	---	---		MmugDNA.15501.1.S1_at_LUZP1
MmugDNA.1550.1.S1_at	242697_at	ZNF540	zinc finger protein 540	chr19q13.12		163255	LOC708197	Similar to serine carboxypeptidase 1 precursor protein	---		708197	MmugDNA.1550.1.S1_at_ZNF540
MmugDNA.15502.1.S1_at	240369_at	TTC7A	Tetratricopeptide repeat domain 7A	chr2p21		57217	---	---	---	---		MmugDNA.15502.1.S1_at_TTC7A
MmugDNA.15503.1.S1_at	221831_at	LUZP1	leucine zipper protein 1	chr1p36		7798	---	---	---	---		MmugDNA.15503.1.S1_at_LUZP1
MmugDNA.15504.1.S1_at	218928_s_at	SLC37A1	solute carrier family 37 (glycerol-3-phosphate transporter), member 1	chr21q22.3		54020	SLC37A1	solute carrier family 37 (glycerol-3-phosphate transporter), member 1	---		714337	MmugDNA.15504.1.S1_at_SLC37A1
MmugDNA.15505.1.S1_at	236245_at	ODF3L1	outer dense fiber of sperm tails 3-like 1	chr15q24.2		161753	ODF3L1	outer dense fiber of sperm tails 3-like 1	---		706545	MmugDNA.15505.1.S1_at_ODF3L1
MmugDNA.15506.1.S1_at	214969_at	MAP3K9	mitogen-activated protein kinase kinase kinase 9	chr14q24.3-q31		4293	MAP3K9	mitogen-activated protein kinase kinase kinase 9	---		693835	MmugDNA.15506.1.S1_at_MAP3K9
MmugDNA.15507.1.S1_at	242596_at	---	---	---	---		---	---	---	---		MmugDNA.15507.1.S1_at_---
MmugDNA.15508.1.S1_at	1559284_at	---	---	---	---		---	---	---	---		MmugDNA.15508.1.S1_at_---
MmugDNA.15509.1.S1_at	207462_at	GLRA2	glycine receptor, alpha 2	chrXp22.1-p21.3		2742	GLRA2	glycine receptor, alpha 2	---		711926	MmugDNA.15509.1.S1_at_GLRA2
MmugDNA.15510.1.S1_s_at	211703_s_at	TM2D1	TM2 domain containing 1	chr1p31.3		83941	TM2D1	TM2 domain containing 1	---		694282	MmugDNA.15510.1.S1_s_at_TM2D1
MmugDNA.15511.1.S1_s_at	228089_x_at	TMEM179B	transmembrane protein 179B	chr11q12.3		374395	LOC718518	hypothetical protein LOC718518	---		718518	MmugDNA.15511.1.S1_s_at_TMEM179
MmugDNA.1551.1.S1_at	201733_at	CLCN3	chloride channel 3	chr4q33		1182	LOC694472	similar to chloride channel 3 isoform c	---		694472	MmugDNA.1551.1.S1_at_CLCN3
MmugDNA.1551.1.S1_s_at	NA	NA	NA	NA	NA		NA	NA	NA	NA		MmugDNA.1551.1.S1_s_at_NA
MmugDNA.15512.1.S1_at	NA	NA	NA	NA	NA		NA	NA	NA	NA		MmugDNA.15512.1.S1_at_NA
MmugDNA.15512.1.S1_s_at	205280_at	GLRB	glycine receptor, beta	chr4q31.3		2743	GLRB	glycine receptor, beta	---		700368	MmugDNA.15512.1.S1_s_at_GLRB
MmugDNA.15513.1.S1_at	234368_at	---	---	---	---		---	---	---	---		MmugDNA.15513.1.S1_at_---
MmugDNA.15514.1.S1_at	1553835_a_at	COL29A1	collagen, type XXIX, alpha 1	chr3q22.1		256076	LOC718067	hypothetical protein LOC718067	---		718067	MmugDNA.15514.1.S1_at_COL29A1
MmugDNA.15515.1.S1_at	206662_at	GLRX	glutaredoxin (thioltransferase)	chr5q14		2745	LOC698143	Similar to 60S ribosomal protein L12	---		698143	MmugDNA.15515.1.S1_at_GLRX
MmugDNA.15516.1.S1_at	234261_at	---	---	---	---		---	---	---	---		MmugDNA.15516.1.S1_at_---
MmugDNA.15517.1.S1_at	200648_s_at	GLUL	glutamate-ammonia ligase (glutamine synthetase)	chr1q31		2752	GLUL	glutamate-ammonia ligase (glutamine synthetase)	---		715952	MmugDNA.15517.1.S1_at_GLUL
MmugDNA.15518.1.S1_at	229275_at	IGFN1	immunoglobulin-like and fibronectin type III domain containing 1	chr1q32.1		91156	LOC710341	similar to eEF1A2 binding protein	---		710341	MmugDNA.15518.1.S1_at_IGFN1
MmugDNA.15519.1.S1_at	241467_at	---	---	---	---		---	---	---	---		MmugDNA.15519.1.S1_at_---
MmugDNA.155.1.S1_at	225785_at	REEP3	receptor accessory protein 3	chr10q21.3		221035	REEP3	receptor accessory protein 3	---		697494	MmugDNA.155.1.S1_at_REEP3
MmugDNA.15520.1.S1_at	203820_s_at	IGF2BP3	insulin-like growth factor 2 mRNA binding protein 3	chr7p11		10643	IGF2BP3	insulin-like growth factor 2 mRNA binding protein 3	---		704774	MmugDNA.15520.1.S1_at_IGF2BP3
MmugDNA.15520.1.S1_s_at	NA	NA	NA	NA	NA		NA	NA	NA	NA		MmugDNA.15520.1.S1_s_at_NA
MmugDNA.15521.1.S1_at	228245_s_at	LOC728715 /// OVOS //	similar to hCG38149 /// ovostatin /// ovostatin 2	chr12p11.21 /// chr12p13 /// chr1		144203 /// 408186 /// 7	LOC722305	similar to cDNA sequence BC048546	---		722305	MmugDNA.15521.1.S1_at_LOC728715
MmugDNA.1552.1.S1_at	201734_at	CLCN3	Chloride channel 3	chr4q33		1182	---	---	---	---		MmugDNA.1552.1.S1_at_CLCN3
MmugDNA.15522.1.S1_at	1569940_at	SLC6A16	Solute carrier family 6, member 16	chr19q13.1-q13.4		28968	---	---	---	---		MmugDNA.15522.1.S1_at_SLC6A16
MmugDNA.15523.1.S1_at	208450_at	LGALS2	lectin, galactoside-binding, soluble, 2	chr22q12-q13|22q13.1		3957	LGALS3	lectin, galactoside-binding, soluble, 3	---		697820	MmugDNA.15523.1.S1_at_LGALS2
MmugDNA.15524.1.S1_at	1558920_at	LOC100128590	hypothetical protein LOC100128590	chr2p22.1		100128590	---	---	---	---		MmugDNA.15524.1.S1_at_LOC1001285
MmugDNA.15525.1.S1_at	207910_at	SCGB1D1	secretoglobin, family 1D, member 1	chr11q13		10648	LOC718936	similar to lipophilin A precursor	---		718936	MmugDNA.15525.1.S1_at_SCGB1D1
MmugDNA.15526.1.S1_at	242278_at	---	---	---	---		---	---	---	---		MmugDNA.15526.1.S1_at_---
MmugDNA.15527.1.S1_at	231503_at	---	---	---	---		---	---	---	---		MmugDNA.15527.1.S1_at_---
MmugDNA.15528.1.S1_at	227760_at	IGFBPL1	insulin-like growth factor binding protein-like 1	chr9p13.1		347252	---	---	---	---		MmugDNA.15528.1.S1_at_IGFBPL1
MmugDNA.15529.1.S1_at	1558122_s_at	---	---	---	---		---	---	---	---		MmugDNA.15529.1.S1_at_---
MmugDNA.15530.1.S1_at	226472_at	PPIL4	peptidylprolyl isomerase (cyclophilin)-like 4	chr6q24-q25		85313	PPIL4	peptidylprolyl isomerase (cyclophilin)-like 4	---		696634	MmugDNA.15530.1.S1_at_PPIL4
MmugDNA.15531.1.S1_at	239085_at	JDP2	Jun dimerization protein 2	chr14q24.3		122953	---	---	---	---		MmugDNA.15531.1.S1_at_JDP2
MmugDNA.1553.1.S1_at	239542_at	ITPR3	Inositol 1,4,5-triphosphate receptor, type 3	chr6p21		3710	---	---	---	---		MmugDNA.1553.1.S1_at_ITPR3
MmugDNA.15532.1.S1_at	239867_at	---	---	---	---		---	---	---	---		MmugDNA.15532.1.S1_at_---
MmugDNA.15533.1.S1_at	1569041_at	---	---	---	---		---	---	---	---		MmugDNA.15533.1.S1_at_---
MmugDNA.15534.1.S1_at	228202_at	PLN	Phospholamban	chr6q22.1		5350	---	---	---	---		MmugDNA.15534.1.S1_at_PLN
MmugDNA.15535.1.S1_at	231616_at	---	---	---	---		---	---	---	---		MmugDNA.15535.1.S1_at_---
MmugDNA.15536.1.S1_at	224413_s_at	TM2D2	TM2 domain containing 2	chr8p11.23		83877	TM2D2	TM2 domain containing 2	---		701007	MmugDNA.15536.1.S1_at_TM2D2
MmugDNA.15537.1.S1_at	217814_at	CCDC47	coiled-coil domain containing 47	chr17q23.3		57003	---	---	---	---		MmugDNA.15537.1.S1_at_CCDC47
MmugDNA.15539.1.S1_at	225695_at	C2orf18	chromosome 2 open reading frame 18	chr2p23.3		54978	C13H2orf18	chromosome 2 open reading frame 18 ortholog	---		697195	MmugDNA.15539.1.S1_at_C2orf18
MmugDNA.15540.1.S1_at	226435_at	PAPLN	papilin, proteoglycan-like sulfated glycoprotein	chr14q24.2		89932	PAPLN	papilin, proteoglycan-like sulfated glycoprotein	---		696394	MmugDNA.15540.1.S1_at_PAPLN
MmugDNA.15541.1.S1_at	237753_at	---	---	---	---		---	---	---	---		MmugDNA.15541.1.S1_at_---
MmugDNA.1554.1.S1_at	217986_s_at	BAZ1A	bromodomain adjacent to zinc finger domain, 1A	chr14q12-q13		11177	BAZ1A	bromodomain adjacent to zinc finger domain, 1A	---		693697	MmugDNA.1554.1.S1_at_BAZ1A
MmugDNA.15543.1.S1_at	1569510_at	---	---	---	---		---	---	---	---		MmugDNA.15543.1.S1_at_---
MmugDNA.15544.1.S1_at	219012_s_at	C11orf30	chromosome 11 open reading frame 30	chr11q13.5		56946	C14H11orf30	chromosome 11 open reading frame 30 ortholog	---		697319	MmugDNA.15544.1.S1_at_C11orf30
MmugDNA.15545.1.S1_at	1553513_at	VNN3	vanin 3	chr6q23-q24		55350	VNN3	vanin 3	---	697384 /// 708748		MmugDNA.15545.1.S1_at_VNN3
MmugDNA.15546.1.S1_at	241684_at	---	---	---	---		---	---	---	---		MmugDNA.15546.1.S1_at_---
MmugDNA.15547.1.S1_at	222501_s_at	REPIN1	replication initiator 1	chr7q36.1		29803	LOC712920	similar to replication initiator 1 isoform 1	---		712920	MmugDNA.15547.1.S1_at_REPIN1
MmugDNA.15548.1.S1_at	219137_s_at	MFF	mitochondrial fission factor	chr2q36.3		56947	LOC708489	hypothetical protein LOC708489	---		708489	MmugDNA.15548.1.S1_at_MFF
MmugDNA.15549.1.S1_s_at	214106_s_at	GMDS	GDP-mannose 4,6-dehydratase	chr6p25		2762	LOC718841	Similar to Fc receptor-like 1	---		718841	MmugDNA.15549.1.S1_s_at_GMDS
MmugDNA.15550.1.S1_at	234399_at	---	---	---	---		LOC712031	similar to T-cell receptor alpha chain V region CTL-L17 precursor	---		712031	MmugDNA.15550.1.S1_at_---
MmugDNA.15551.1.S1_at	205001_s_at	DDX3Y	DEAD (Asp-Glu-Ala-Asp) box polypeptide 3, Y-linked	chrYq11		8653	---	---	---	---		MmugDNA.15551.1.S1_at_DDX3Y
MmugDNA.1555.1.S1_at	230976_at	C9orf98	chromosome 9 open reading frame 98	chr9q34.13		158067	LOC714698	similar to C29E4.8	---		714698	MmugDNA.1555.1.S1_at_C9orf98
MmugDNA.15552.1.S1_at	1562965_at	---	---	---	---		---	---	---	---		MmugDNA.15552.1.S1_at_---
MmugDNA.15553.1.S1_at	227783_at	CCDC57	coiled-coil domain containing 57	chr17q25.3		284001	---	---	---	---		MmugDNA.15553.1.S1_at_CCDC57
MmugDNA.15554.1.S1_at	204248_at	GNA11	guanine nucleotide binding protein (G protein), alpha 11 (Gq class)	chr19p13.3		2767	---	---	---	---		MmugDNA.15554.1.S1_at_GNA11
MmugDNA.15555.1.S1_at	216208_s_at	ATF6B	activating transcription factor 6 beta	chr6p21.3		1388	---	---	---	---		MmugDNA.15555.1.S1_at_ATF6B
MmugDNA.15556.1.S1_at	NA	NA	NA	NA	NA		NA	NA	NA	NA		MmugDNA.15556.1.S1_at_NA
MmugDNA.15556.1.S1_s_at	223370_at	PLEKHA3	pleckstrin homology domain containing, family A (phosphoinositide binding specific) member 3	chr2q31.2		65977	LOC710948	Similar to wolframin	---		710948	MmugDNA.15556.1.S1_s_at_PLEKHA3
MmugDNA.15557.1.S1_at	231497_at	LOC100131117	similar to hCG2045385	chr3q13.31		100131117	---	---	---	---		MmugDNA.15557.1.S1_at_LOC1001311
MmugDNA.15558.1.S1_at	212545_s_at	ZHX3	zinc fingers and homeoboxes 3	chr20q12		23051	SERPINB6	Serpin peptidase inhibitor, clade B (ovalbumin), member 6	---		701920	MmugDNA.15558.1.S1_at_ZHX3
MmugDNA.15559.1.S1_at	207041_at	MASP2	mannan-binding lectin serine peptidase 2	chr1p36.3-p36.2		10747	LOC722701	similar to mannan-binding lectin serine protease 2 isoform 1 precursor	---		722701	MmugDNA.15559.1.S1_at_MASP2
MmugDNA.15560.1.S1_at	204663_at	ME3	malic enzyme 3, NADP(+)-dependent, mitochondrial	chr11cen-q22.3		10873	ME3	malic enzyme 3, NADP(+)-dependent, mitochondrial	---		704422	MmugDNA.15560.1.S1_at_ME3
MmugDNA.15561.1.S1_at	244815_at	---	---	---	---		---	---	---	---		MmugDNA.15561.1.S1_at_---
MmugDNA.1556.1.S1_at	212928_at	TSPYL4	TSPY-like 4	chr6q22.1		23270	---	---	---	---		MmugDNA.1556.1.S1_at_TSPYL4
MmugDNA.15562.1.S1_s_at	212044_s_at	RPL27A	Ribosomal protein L27a	chr11p15		6157	LOC709769	similar to ribosomal protein L27a	---		709769	MmugDNA.15562.1.S1_s_at_RPL27A
MmugDNA.15563.1.S1_at	200028_s_at	STARD7	StAR-related lipid transfer (START) domain containing 7	chr2q11.2		56910	STARD7	StAR-related lipid transfer (START) domain containing 7	---		704860	MmugDNA.15563.1.S1_at_STARD7
MmugDNA.15564.1.S1_x_at	NA	NA	NA	NA	NA		NA	NA	NA	NA		MmugDNA.15564.1.S1_x_at_NA
MmugDNA.15565.1.S1_at	238528_at	UBR1	ubiquitin protein ligase E3 component n-recognin 1	chr15q13		197131	LOC709238	similar to ubiquitin protein ligase E3 component n-recognin 1	---		709238	MmugDNA.15565.1.S1_at_UBR1
MmugDNA.15566.1.S1_at	201040_at	GNAI2	guanine nucleotide binding protein (G protein), alpha inhibiting activity polypeptide 2	chr3p21		2771	---	---	---	---		MmugDNA.15566.1.S1_at_GNAI2
MmugDNA.15567.1.S1_at	225021_at	ZNF532	zinc finger protein 532	chr18q21.32		55205	ZNF532	zinc finger protein 532	---		701146	MmugDNA.15567.1.S1_at_ZNF532
MmugDNA.15568.1.S1_at	218110_at	XAB2	XPA binding protein 2	chr19p13.2		56949	XAB2	XPA binding protein 2	---		704109	MmugDNA.15568.1.S1_at_XAB2
MmugDNA.15569.1.S1_at	240176_at	---	---	---	---		---	---	---	---		MmugDNA.15569.1.S1_at_---
MmugDNA.15570.1.S1_at	241488_at	---	---	---	---		---	---	---	---		MmugDNA.15570.1.S1_at_---
MmugDNA.15571.1.S1_at	203992_s_at	KDM6A	lysine (K)-specific demethylase 6A	chrXp11.2		7403	LOC709727	similar to ubiquitously transcribed tetratricopeptide repeat, X chromosome	---		709727	MmugDNA.15571.1.S1_at_KDM6A
MmugDNA.1557.1.S1_at	243932_at	---	---	---	---		---	---	---	---		MmugDNA.1557.1.S1_at_---
MmugDNA.15572.1.S1_at	207514_s_at	GNAT1	guanine nucleotide binding protein (G protein), alpha transducing activity polypeptide 1	chr3p21		2779	GNAT1	guanine nucleotide binding protein (G protein), alpha transducing activity polypeptide 1	---		703881	MmugDNA.15572.1.S1_at_GNAT1
MmugDNA.15573.1.S1_s_at	NA	NA	NA	NA	NA		NA	NA	NA	NA		MmugDNA.15573.1.S1_s_at_NA
MmugDNA.15573.1.S1_x_at	234971_x_at	PLCD3	phospholipase C, delta 3	chr17q21.31		113026	LOC715924	similar to phospholipase C delta 3	---		715924	MmugDNA.15573.1.S1_x_at_PLCD3
MmugDNA.15574.1.S1_at	220043_s_at	MFI2	antigen p97 (melanoma associated) identified by monoclonal antibodies 133.2 and 96.5	chr3q28-q29		4241	MFI2	antigen p97 (melanoma associated) identified by monoclonal antibodies 133.2 and 96.5	---		707618	MmugDNA.15574.1.S1_at_MFI2

MmugDNA.15575.1.S1_at	206560_s_at	MIA	melanoma inhibitory activity	chr19q13.32-q13.33		8190	MIA	melanoma inhibitory activity	---		702844	MmugDNA.15575.1.S1_at_MIA
MmugDNA.15576.1.S1_at	NA	NA	NA	NA	NA		NA	NA	NA	NA		MmugDNA.15576.1.S1_at_NA
MmugDNA.15577.1.S1_s_at	221025_x_at	PUS7L	pseudouridylate synthase 7 homolog (S. cerevisiae)-like	chr12q12		83448	PUS7L	pseudouridylate synthase 7 homolog (S. cerevisiae)-like	---		700448	MmugDNA.15577.1.S1_s_at_PUS7L
MmugDNA.15578.1.S1_at	237102_at	---	---	---	---		---	---	---	---		MmugDNA.15578.1.S1_at_---
MmugDNA.15579.1.S1_at	216063_at	HBBP1	hemoglobin, beta pseudogene 1	chr11p15.5		3044	---	---	---	---		MmugDNA.15579.1.S1_at_HBBP1
MmugDNA.15580.1.S1_at	235038_at	KRR1	KRR1, small subunit (SSU) processome component, homolog (yeast)	chr12q21.2		11103	---	---	---	---		MmugDNA.15580.1.S1_at_KRR1
MmugDNA.15581.1.S1_at	237938_at	---	---	---	---		---	---	---	---		MmugDNA.15581.1.S1_at_---
MmugDNA.1558.1.S1_at	238396_at	---	---	---	---		---	---	---	---		MmugDNA.1558.1.S1_at_---
MmugDNA.15582.1.S1_s_at	215722_s_at	SNRPA1	small nuclear ribonucleoprotein polypeptide A'	chr15q26.3		6627	LOC711573	Similar to keratin 8	---		711573	MmugDNA.15582.1.S1_s_at_SNRPA1
MmugDNA.15583.1.S1_at	217511_at	KAZALD1	Kazal-type serine peptidase inhibitor domain 1	chr10q24.31		81621	LOC711518	similar to Kazal-type serine protease inhibitor domain 1	---		711518	MmugDNA.15583.1.S1_at_KAZALD1
MmugDNA.15584.1.S1_at	244467_at	C22:CTA-250D10.9	transmembrane protein 46-like	chr22q13.2		440829	---	---	---	---		MmugDNA.15584.1.S1_at_C22:CTA-25
MmugDNA.15585.1.S1_at	219306_at	KIF15	kinesin family member 15	chr3p21.31		56992	HSF4	Heat shock transcription factor 4	---		698412	MmugDNA.15585.1.S1_at_KIF15
MmugDNA.15586.1.S1_at	204993_at	GNAZ	guanine nucleotide binding protein (G protein), alpha z polypeptide	chr22q11.22		2781	---	---	---	---		MmugDNA.15586.1.S1_at_GNAZ
MmugDNA.15587.1.S1_at	206047_at	GNB3	guanine nucleotide binding protein (G protein), beta polypeptide 3	chr12p13		2784	GNB3	guanine nucleotide binding protein (G protein), beta polypeptide 3	---		713913	MmugDNA.15587.1.S1_at_GNB3
MmugDNA.15588.1.S1_at	210322_x_at	UTY	ubiquitously transcribed tetratricopeptide repeat gene, Y-linked	chrYq11		7404	LOC709727	similar to ubiquitously transcribed tetratricopeptide repeat, X chromosome	---		709727	MmugDNA.15588.1.S1_at_UTY
MmugDNA.15589.1.S1_at	218162_at	OLFML3	olfactomedin-like 3	chr1p13.2		56944	JAK2	Janus kinase 2	---		694184	MmugDNA.15589.1.S1_at_OLFML3
MmugDNA.15590.1.S1_at	1558742_at	---	---	---	---		---	---	---	---		MmugDNA.15590.1.S1_at_---
MmugDNA.15591.1.S1_at	240406_at	---	---	---	---		---	---	---	---		MmugDNA.15591.1.S1_at_---
MmugDNA.1559.1.S1_at	NA	NA	NA	NA	NA		NA	NA	NA	NA		MmugDNA.1559.1.S1_at_NA
MmugDNA.15592.1.S1_at	207987_s_at	GNRH1	gonadotropin-releasing hormone 1 (luteinizing-releasing hormone)	chr8p21-p11.2		2796	GNRH1	gonadotropin-releasing hormone 1 (luteinizing-releasing hormone)	---		613033	MmugDNA.15592.1.S1_at_GNRH1
MmugDNA.15593.1.S1_at	202767_at	ACP2	acid phosphatase 2, lysosomal	chr11p11.2-p11.11|11p12-p11		53	LOC701352	Similar to ubiquitin-specific protease 12-like 1	---		701352	MmugDNA.15593.1.S1_at_ACP2
MmugDNA.15594.1.S1_at	208519_x_at	GNRH2	gonadotropin-releasing hormone 2	chr20p13		2797	GNRH2	gonadotropin-releasing hormone 2	---		619516	MmugDNA.15594.1.S1_at_GNRH2
MmugDNA.15595.1.S1_at	216951_at	FCGR1A	Fc fragment of IgG, high affinity Ia, receptor (CD64)	chr1q21.2-q21.3		2209	HTRA1	HtrA serine peptidase 1	---		705855	MmugDNA.15595.1.S1_at_FCGR1A
MmugDNA.15596.1.S1_at	214954_at	SUSD5	sushi domain containing 5	chr3p22.3		26032	---	---	---	---		MmugDNA.15596.1.S1_at_SUSD5
MmugDNA.15597.1.S1_s_at	NA	NA	NA	NA	NA		NA	NA	NA	NA		MmugDNA.15597.1.S1_s_at_NA
MmugDNA.15598.1.S1_at	1559021_at	---	---	---	---		---	---	---	---		MmugDNA.15598.1.S1_at_---
MmugDNA.15599.1.S1_s_at	207425_s_at	SEPT9	septin 9	chr17q25		10801	---	---	---	---		MmugDNA.15599.1.S1_s_at_SEPT9
MmugDNA.15600.1.S1_at	202171_at	VEZF1	vascular endothelial zinc finger 1	chr17q22		7716	LOC713848	similar to zinc finger protein 161	---		713848	MmugDNA.15600.1.S1_at_VEZF1
MmugDNA.15601.1.S1_at	238931_at	METT10D	methyltransferase 10 domain containing	chr17p13.3		79066	---	---	---	---		MmugDNA.15601.1.S1_at_METT10D
MmugDNA.1560.1.S1_at	222209_s_at	TMEM135	transmembrane protein 135	chr11q14.2		65084	TMEM135	transmembrane protein 135	---		704963	MmugDNA.1560.1.S1_at_TMEM135
MmugDNA.15602.1.S1_at	1570071_at	---	---	---	---		---	---	---	---		MmugDNA.15602.1.S1_at_---
MmugDNA.15603.1.S1_at	202172_at	VEZF1	vascular endothelial zinc finger 1	chr17q22		7716	LOC713848	similar to zinc finger protein 161	---		713848	MmugDNA.15603.1.S1_at_VEZF1
MmugDNA.15604.1.S1_at	203517_at	MTX2	metaxin 2	chr2q31.1		10651	MTX2	metaxin 2	---		701294	MmugDNA.15604.1.S1_at_MTX2
MmugDNA.15605.1.S1_at	206688_s_at	CPSF4	cleavage and polyadenylation specific factor 4, 30kDa	chr7q22.1		10898	CPSF4	cleavage and polyadenylation specific factor 4, 30kDa	---		708119	MmugDNA.15605.1.S1_at_CPSF4
MmugDNA.15606.1.S1_at	1565881_at	---	---	---	---		---	---	---	---		MmugDNA.15606.1.S1_at_---
MmugDNA.15607.1.S1_at	1564052_at	TREML4	triggering receptor expressed on myeloid cells-like 4	chr6p21.1		285852	---	---	---	---		MmugDNA.15607.1.S1_at_TREML4
MmugDNA.15608.1.S1_at	220405_at	LOC100127998 /// SNT	hypothetical protein LOC100127998 /// syntrophin, gamma 1	chr8q11-q12 /// chr8q11.22		100127998 /// 54212	---	---	---	---		MmugDNA.15608.1.S1_at_LOC100127!
MmugDNA.15609.1.S1_at	231968_at	UGCGL1	UDP-glucose ceramide glucosyltransferase-like 1	chr2q14.3		56886	---	---	---	---		MmugDNA.15609.1.S1_at_UGCGL1
MmugDNA.15610.1.S1_at	220487_at	SNTG2	syntrophin, gamma 2	chr2p25.3		54221	SNTG2	syntrophin, gamma 2	---		706376	MmugDNA.15610.1.S1_at_SNTG2
MmugDNA.15611.1.S1_at	241742_at	PRAM1	PML-RARA regulated adaptor molecule 1	chr19p13.2		84106	LOC712017	similar to PML-RARA regulated adaptor molecule 1	---		712017	MmugDNA.15611.1.S1_at_PRAM1
MmugDNA.1561.1.S1_at	237164_at	---	---	---	---		---	---	---	---		MmugDNA.1561.1.S1_at_---
MmugDNA.15612.1.S1_at	240902_at	LOC283624	Hypothetical protein LOC283624	chr14q11.2		283624	---	---	---	---		MmugDNA.15612.1.S1_at_LOC283624
MmugDNA.15613.1.S1_at	203676_at	GNS	glucosamine (N-acetyl)-6-sulfatase	chr12q14		2799	GNS	glucosamine (N-acetyl)-6-sulfatase	---		717500	MmugDNA.15613.1.S1_at_GNS
MmugDNA.15614.1.S1_at	217881_s_at	CDC27	cell division cycle 27 homolog (S. cerevisiae)	chr17q12-q23.2		996	LOC716727	Similar to killer cell lectin-like receptor subfamily G, member 1	---		716727	MmugDNA.15614.1.S1_at_CDC27
MmugDNA.15615.1.S1_at	203024_s_at	C5orf15	chromosome 5 open reading frame 15	chr5q31.1		56951	---	---	---	---		MmugDNA.15615.1.S1_at_C5orf15
MmugDNA.15616.1.S1_at	203383_s_at	GOLGA1	golgi autoantigen, golgin subfamily a, 1	chr9q33.3		2800	LOC693285	similar to Golgin subfamily A member 1 (Golgin-97)	---		693285	MmugDNA.15616.1.S1_at_GOLGA1
MmugDNA.15617.1.S1_at	218257_s_at	UGCGL1	UDP-glucose ceramide glucosyltransferase-like 1	chr2q14.3		56886	UGCGL1	UDP-glucose ceramide glucosyltransferase-like 1	---		700462	MmugDNA.15617.1.S1_at_UGCGL1
MmugDNA.15618.1.S1_s_at	211122_s_at	CXCL11	chemokine (C-X-C motif) ligand 11	chr4q21.2		6373	---	---	---	---		MmugDNA.15618.1.S1_s_at_CXCL11
MmugDNA.15619.1.S1_at	NA	NA	NA	NA	NA		NA	NA	NA	NA		MmugDNA.15619.1.S1_at_NA
MmugDNA.15619.1.S1_s_at	230634_x_at	ADAT3	adenosine deaminase, tRNA-specific 3, TAD3 homolog (S. cerevisiae)	chr19p13.3		113179	LOC709219	similar to CG10927-PA	---		709219	MmugDNA.15619.1.S1_s_at_ADAT3
MmugDNA.156.1.S1_at	207792_at	OPRD1	opioid receptor, delta 1	chr1p36.1-p34.3		4985	OPRD1	opioid receptor, delta 1	---		717256	MmugDNA.156.1.S1_at_OPRD1
MmugDNA.15620.1.S1_at	200708_at	GOT2	glutamic-oxaloacetic transaminase 2, mitochondrial (aspartate aminotransferase 2)	chr16q21		2806	LOC700580	Similar to leucine rich repeat and death domain containing protein isoform 1	---		700580	MmugDNA.15620.1.S1_at_GOT2
MmugDNA.15621.1.S1_at	206023_at	NMU	neuromedin U	chr4q12		10874	LOC697171	similar to Neuromedin U-25 precursor (NmU-25)	---		697171	MmugDNA.15621.1.S1_at_NMU
MmugDNA.1562.1.S1_at	1562832_at	C3orf65	chromosome 3 open reading frame 65	chr3q27.2		646600	---	---	---	---		MmugDNA.1562.1.S1_at_C3orf65
MmugDNA.15622.1.S1_at	1570194_x_at	---	---	---	---		---	---	---	---		MmugDNA.15622.1.S1_at_---
MmugDNA.15623.1.S1_at	240127_at	---	---	---	---		---	---	---	---		MmugDNA.15623.1.S1_at_---
MmugDNA.15624.1.S1_at	240690_at	---	---	---	---		---	---	---	---		MmugDNA.15624.1.S1_at_---
MmugDNA.15625.1.S1_at	203246_s_at	TUSC4	tumor suppressor candidate 4	chr3p21.3		10641	TUSC4	tumor suppressor candidate 4	---		701924	MmugDNA.15625.1.S1_at_TUSC4
MmugDNA.15626.1.S1_at	1553326_at	RXFP2	relaxin/insulin-like family peptide receptor 2	chr13q13.1		122042	---	---	---	---		MmugDNA.15626.1.S1_at_RXFP2
MmugDNA.15627.1.S1_at	238950_at	---	---	---	---		---	---	---	---		MmugDNA.15627.1.S1_at_---
MmugDNA.15628.1.S1_at	240518_at	LOC729291	Hypothetical protein LOC729291	chr12p11.22		729291	---	---	---	---		MmugDNA.15628.1.S1_at_LOC729291
MmugDNA.15629.1.S1_at	1555259_at	ZAK	sterile alpha motif and leucine zipper containing kinase AZK	chr2q24.2		51776	---	---	---	---		MmugDNA.15629.1.S1_at_ZAK
MmugDNA.15630.1.S1_at	238511_at	LOC440288	similar to FLJ16518 protein	chr15q24.1		440288	---	---	---	---		MmugDNA.15630.1.S1_at_LOC440288
MmugDNA.15631.1.S1_at	205906_at	FOXJ1	forkhead box J1	chr17q22-q25		2302	FOXJ1	forkhead box J1	---		706275	MmugDNA.15631.1.S1_at_FOXJ1
MmugDNA.1563.1.S1_at	NA	NA	NA	NA	NA		NA	NA	NA	NA		MmugDNA.1563.1.S1_at_NA
MmugDNA.1563.1.S1_s_at	206390_x_at	PF4	platelet factor 4	chr4q12-q21		5196	PF4	platelet factor 4	---		703451	MmugDNA.1563.1.S1_s_at_PF4
MmugDNA.15632.1.S1_at	216261_at	ITGB3	integrin, beta 3 (platelet glycoprotein IIIa, antigen CD61)	chr17q21.32		3690	LOC717379	similar to integrin beta chain, beta 3 precursor	---		717379	MmugDNA.15632.1.S1_at_ITGB3
MmugDNA.15633.1.S1_at	218306_s_at	HERC1	hect (homologous to the E6-AP (UBE3A) carboxyl terminus) domain and RCC1 (CHC1)-like domain (	chr15q22		8925	LOC706308	similar to guanine nucleotide exchange factor p532	---		706308	MmugDNA.15633.1.S1_at_HERC1
MmugDNA.15634.1.S1_at	207389_at	GP1BA	glycoprotein Ib (platelet), alpha polypeptide	chr17pter-p12		2811	LOC721584	similar to Platelet glycoprotein Ib alpha chain precursor (Glycoprotein Ibalpha) (GP-Ib alpha) (GPIbA)	---		721584	MmugDNA.15634.1.S1_at_GP1BA
MmugDNA.15635.1.S1_at	206681_x_at	GP2	glycoprotein 2 (zymogen granule membrane)	chr16p12		2813	GP2	glycoprotein 2 (zymogen granule membrane)	---		695766	MmugDNA.15635.1.S1_at_GP2
MmugDNA.15636.1.S1_at	215371_at	MED27	mediator complex subunit 27	chr9q34.1-q34.3		9442	---	---	---	---		MmugDNA.15636.1.S1_at_MED27
MmugDNA.15637.1.S1_at	203866_at	NLE1	notchless homolog 1 (Drosophila)	chr17q12		54475	---	---	---	---		MmugDNA.15637.1.S1_at_NLE1
MmugDNA.15638.1.S1_at	203718_at	PNPLA6	patatin-like phospholipase domain containing 6	chr19p13.3-p13.2		10908	---	---	---	---		MmugDNA.15638.1.S1_at_PNPLA6
MmugDNA.15639.1.S1_s_at	201173_x_at	NUDC	nuclear distribution gene C homolog (A. nidulans)	chr1p35-p34		10726	---	---	---	---		MmugDNA.15639.1.S1_s_at_NUDC
MmugDNA.15640.1.S1_at	1561408_at	---	---	---	---		---	---	---	---		MmugDNA.15640.1.S1_at_---
MmugDNA.15641.1.S1_at	202900_s_at	NUP88	nucleoporin 88kDa	chr17p13.2		4927	LOC711709	Similar to inosine monophosphate dehydrogenase 1 isoform a	---		711709	MmugDNA.15641.1.S1_at_NUP88
MmugDNA.1564.1.S1_at	207815_at	PF4V1	platelet factor 4 variant 1	chr4q12-q21		5197	PF4	platelet factor 4	---		703451	MmugDNA.1564.1.S1_at_PF4V1
MmugDNA.15643.1.S1_at	1560445_x_at	ARHGEF1	Rho guanine nucleotide exchange factor (GEF) 1	chr19q13.13		9138	---	---	---	---		MmugDNA.15643.1.S1_at_ARHGEF1
MmugDNA.15644.1.S1_at	1562770_at	---	---	---	---		LOC707340	hypothetical protein LOC707340	---		707340	MmugDNA.15644.1.S1_at_---
MmugDNA.15645.1.S1_at	1562049_at	---	---	---	---		---	---	---	---		MmugDNA.15645.1.S1_at_---
MmugDNA.15646.1.S1_at	208521_at	OR5I1	olfactory receptor, family 5, subfamily I, member 1	chr11q11		10798	LOC707712	similar to Olfactory receptor 5I1 (Olfactory receptor-like protein OLF1) (Olfactory receptor OR11-159)	---		707712	MmugDNA.15646.1.S1_at_OR5I1
MmugDNA.15647.1.S1_at	227641_at	FBXL16	F-box and leucine-rich repeat protein 16	chr16p13.3		146330	---	---	---	---		MmugDNA.15647.1.S1_at_FBXL16
MmugDNA.15648.1.S1_at	1558631_at	PPARA	peroxisome proliferator-activated receptor alpha	chr22q12-q13.1|22q13.31		5465	---	---	---	---		MmugDNA.15648.1.S1_at_PPARA
MmugDNA.15649.1.S1_at	203768_s_at	STS	steroid sulfatase (microsomal), isozyme S	chrXp22.32		412	---	---	---	---		MmugDNA.15649.1.S1_at_STS
MmugDNA.15650.1.S1_s_at	224572_s_at	IRF2BP2	interferon regulatory factor 2 binding protein 2	chr1q42.3		359948	---	---	---	---		MmugDNA.15650.1.S1_s_at_IRF2BP2
MmugDNA.15651.1.S1_at	203767_s_at	STS	steroid sulfatase (microsomal), isozyme S	chrXp22.32		412	---	---	---	---		MmugDNA.15651.1.S1_at_STS
MmugDNA.1565.1.S1_at	241466_at	---	---	---	---		---	---	---	---		MmugDNA.1565.1.S1_at_---
MmugDNA.15652.1.S1_at	224569_s_at	IRF2BP2	interferon regulatory factor 2 binding protein 2	chr1q42.3		359948	---	---	---	---		MmugDNA.15652.1.S1_at_IRF2BP2
MmugDNA.15653.1.S1_at	1560946_at	---	---	---	---		---	---	---	---		MmugDNA.15653.1.S1_at_---
MmugDNA.15654.1.S1_at	224570_s_at	IRF2BP2	interferon regulatory factor 2 binding protein 2	chr1q42.3		359948	TNFAIP3	Tumor necrosis factor, alpha-induced protein 3	---		704130	MmugDNA.15654.1.S1_at_IRF2BP2
MmugDNA.15655.1.S1_at	211467_s_at	NFIB	nuclear factor I/B	chr9p24.1		4781	NFIB	nuclear factor I/B	---		714098	MmugDNA.15655.1.S1_at_NFIB
MmugDNA.15656.1.S1_at	224571_at	IRF2BP2	interferon regulatory factor 2 binding protein 2	chr1q42.3		359948	TNFAIP3	Tumor necrosis factor, alpha-induced protein 3	---		704130	MmugDNA.15656.1.S1_at_IRF2BP2
MmugDNA.15657.1.S1_at	214154_s_at	PKP2	plakophilin 2	chr12p11		5318	---	---	---	---		MmugDNA.15657.1.S1_at_PKP2
MmugDNA.15659.1.S1_at	237845_at	---	---	---	---		---	---	---	---		MmugDNA.15659.1.S1_at_---
MmugDNA.15660.1.S1_at	227667_at	CUEDC1	CUE domain containing 1	chr17q23.2		404093	---	---	---	---		MmugDNA.15660.1.S1_at_CUEDC1
MmugDNA.15661.1.S1_at	40284_at	FOXA2	forkhead box A2	chr20p11		3170	FOXA2	forkhead box A2	---		701771	MmugDNA.15661.1.S1_at_FOXA2
MmugDNA.1566.1.S1_at	243621_at	SPACA3	sperm acrosome associated 3	chr17q11.2		124912	LOC714491	similar to sperm acrosome associated 3	---		714491	MmugDNA.1566.1.S1_at_SPACA3
MmugDNA.15662.1.S1_at	207473_at	MLN	motilin	chr6p21.3		4295	MLN	motilin	---		574099	MmugDNA.15662.1.S1_at_MLN
MmugDNA.15663.1.S1_at	1555436_a_at	AFF4	AF4/FMR2 family, member 4	chr5q31		27125	LOC709137	similar to ALL1 fused gene from 5q31	---		709137	MmugDNA.15663.1.S1_at_AFF4
MmugDNA.15664.1.S1_at	232647_at	PROCA1	protein interacting with cyclin A1	chr17q11.2		147011	LOC709846	similar to proline-rich cyclin A1-interacting protein	---		709846	MmugDNA.15664.1.S1_at_PROCA1
MmugDNA.15665.1.S1_at	239067_s_at	PANX2	pannexin 2	chr22q13.33		56666	LOC720613	similar to pannexin 2	---		720613	MmugDNA.15665.1.S1_at_PANX2
MmugDNA.15666.1.S1_at	210297_s_at	MSMB	microseminoprotein, beta-	chr10q11.2		4477	MSMB	microseminoprotein, beta-	---		709332	MmugDNA.15666.1.S1_at_MSMB
MmugDNA.15667.1.S1_at	208308_s_at	GPI /// LOC100133951	glucose phosphate isomerase /// similar to Glucose phosphate isomerase	chr19q13.1		100133951 /// 2821	LOC717980	similar to Glucose-6-phosphate isomerase (GPI) (Phosphoglucose isomerase) (PGI) (Phosphohexose	---		717980	MmugDNA.15667.1.S1_at_GPI /// LOC1
MmugDNA.15668.1.S1_at	1569431_at	PAFAH1B2	platelet-activating factor acetylhydrolase, isoform Ib, subunit 2 (30kDa)	chr11q23		5049	---	---	---	---		MmugDNA.15668.1.S1_at_PAFAH1B2
MmugDNA.15669.1.S1_at	206266_at	GPLD1	glycosylphosphatidylinositol specific phospholipase D1	chr6p22.3-p22.2		2822	GPLD1	glycosylphosphatidylinositol specific phospholipase D1	---		707782	MmugDNA.15669.1.S1_at_GPLD1
MmugDNA.15670.1.S1_s_at	217496_s_at	IDE	insulin-degrading enzyme	chr10q23-q25		3416	IDE	insulin-degrading enzyme	---		698640	MmugDNA.15670.1.S1_s_at_IDE
MmugDNA.15671.1.S1_at	221344_at	OR12D2	olfactory receptor, family 12, subfamily D, member 2	chr6p22.2-p21.31		26529	LOC708163	similar to Olfactory receptor 12D2 (Hs6M1-20)	---		708163	MmugDNA.15671.1.S1_at_OR12D2
MmugDNA.1567.1.S1_at	206380_s_at	CFP	complement factor properdin	chrXp11.3-p11.23		5199	LOC707386	similar to properdin P factor, complement	---		707386	MmugDNA.1567.1.S1_at_CFP

MmugDNA.15672.1.S1_at	1553572_a_at	CYGB	cytoglobin	chr17q25.3	114757	CYGB	cytoglobin	---	707130	MmugDNA.15672.1.S1_at_CYGB
MmugDNA.15673.1.S1_at	232460_at	---	---	---	---	---	---	---	---	MmugDNA.15673.1.S1_at_---
MmugDNA.15674.1.S1_at	242653_at	DCC	Deleted in colorectal carcinoma	chr18q21.3	1630	---	---	---	---	MmugDNA.15674.1.S1_at_DCC
MmugDNA.15675.1.S1_at	215396_at	GPR98	G protein-coupled receptor 98	chr5q13	84059	---	---	---	---	MmugDNA.15675.1.S1_at_GPR98
MmugDNA.15676.1.S1_at	235173_at	hCG_1806964	hypothetical LOC401093	chr3q25.1	401093	---	---	---	---	MmugDNA.15676.1.S1_at_hCG_18069
MmugDNA.15677.1.S1_at	NA	NA	NA	NA	NA	NA	NA	NA	NA	MmugDNA.15677.1.S1_at_NA
MmugDNA.15678.1.S1_at	234269_at	---	---	---	---	---	---	---	---	MmugDNA.15678.1.S1_at_---
MmugDNA.15679.1.S1_at	206082_at	HCP5	HLA complex P5	chr6p21.3	10866	---	---	---	---	MmugDNA.15679.1.S1_at_HCP5
MmugDNA.15680.1.S1_at	216136_at	---	---	---	---	---	---	---	---	MmugDNA.15680.1.S1_at_---
MmugDNA.15681.1.S1_at	225622_at	PAG1	phosphoprotein associated with glycosphingolipid microdomains 1	chr8q21.13	55824	---	---	---	---	MmugDNA.15681.1.S1_at_PAG1
MmugDNA.1568.1.S1_at	203080_s_at	BAZ2B	bromodomain adjacent to zinc finger domain, 2B	chr2q23-q24	29994	BAZ2B	bromodomain adjacent to zinc finger domain, 2B	---	702780	MmugDNA.1568.1.S1_at_BAZ2B
MmugDNA.15682.1.S1_at	220332_at	CLDN16	claudin 16	chr3q28	10686	CLDN16	similar to claudin 16	---	704442	MmugDNA.15682.1.S1_at_CLDN16
MmugDNA.15683.1.S1_at	241096_at	---	---	---	---	---	---	---	---	MmugDNA.15683.1.S1_at_---
MmugDNA.15684.1.S1_at	1566658_at	---	---	---	---	---	---	---	---	MmugDNA.15684.1.S1_at_---
MmugDNA.15685.1.S1_at	229888_at	C12orf60	chromosome 12 open reading frame 60	chr12p12.3	144608	LOC699817	hypothetical protein LOC699817	---	699817	MmugDNA.15685.1.S1_at_C12orf60
MmugDNA.15686.1.S1_at	225507_at	SFRS18	splicing factor, arginine/serine-rich 18	chr6q16.3	25957	LOC694075	hypothetical protein LOC694075	---	694075	MmugDNA.15686.1.S1_at_SFRS18
MmugDNA.15688.1.S1_at	234941_s_at	GPHN	gephyrin	chr14q23.3	10243	GPHN	gephyrin	---	709677	MmugDNA.15688.1.S1_at_GPHN
MmugDNA.15689.1.S1_at	237919_at	RFFL	Ring finger and FYVE-like domain containing 1	chr17q12	117584	---	---	---	---	MmugDNA.15689.1.S1_at_RFFL
MmugDNA.15690.1.S1_at	233115_at	DEPDC1B	DEP domain containing 1B	chr5q12.1	55789	---	---	---	---	MmugDNA.15690.1.S1_at_DEPDC1B
MmugDNA.15691.1.S1_s_at	203859_s_at	PALM	paralemmin	chr19p13.3	5064	---	---	---	---	MmugDNA.15691.1.S1_s_at_PALM
MmugDNA.15692.1.S1_at	238644_at	MYSM1	Myb-like, SWIRM and MPN domains 1	chr1p32.1	114803	---	---	---	---	MmugDNA.15692.1.S1_at_MYSM1
MmugDNA.15693.1.S1_at	225573_at	ACAD11	acyl-Coenzyme A dehydrogenase family, member 11	chr3q22.1	84129	LOC717622	similar to nephronophthisis 3	---	717622	MmugDNA.15693.1.S1_at_ACAD11
MmugDNA.15694.1.S1_at	230821_at	ZNF148	zinc finger protein 148	chr3q21	7707	ZNF148	zinc finger protein 148	---	715925	MmugDNA.15694.1.S1_at_ZNF148
MmugDNA.15695.1.S1_at	NA	NA	NA	NA	NA	NA	NA	NA	NA	MmugDNA.15695.1.S1_at_NA
MmugDNA.15695.1.S1_s_at	222058_at	---	---	---	---	LOC715356	similar to ring finger protein 130	---	715356	MmugDNA.15695.1.S1_s_at_---
MmugDNA.15696.1.S1_at	207681_at	CXCR3	chemokine (C-X-C motif) receptor 3	chrXq13	2833	---	---	---	---	MmugDNA.15696.1.S1_at_CXCR3
MmugDNA.15697.1.S1_at	236393_at	---	---	---	---	---	---	---	---	MmugDNA.15697.1.S1_at_---
MmugDNA.15698.1.S1_at	242632_at	FGD2	FYVE, RhoGEF and PH domain containing 2	chr6p21.2	221472	LOC719329	similar to FYVE, RhoGEF and PH domain containing 2	---	719329	MmugDNA.15698.1.S1_at_FGD2
MmugDNA.15699.1.S1_at	201713_s_at	RANBP2	RAN binding protein 2	chr2q12.3	5903	LOC694823	similar to RAN binding protein 2	---	694823	MmugDNA.15699.1.S1_at_RANBP2
MmugDNA.15700.1.S1_s_at	201397_at	PHGDH	phosphoglycerate dehydrogenase	chr1p12	26227	PHGDH	phosphoglycerate dehydrogenase	---	713480	MmugDNA.15700.1.S1_s_at_PHGDH
MmugDNA.15701.1.S1_s_at	239919_at	LOC255480	hypothetical protein LOC255480	chr12q24.21	255480	---	---	---	---	MmugDNA.15701.1.S1_s_at_LOC2554
MmugDNA.1570.1.S1_at	240927_at	---	---	---	---	---	---	---	---	MmugDNA.1570.1.S1_at_---
MmugDNA.15702.1.S1_at	239329_at	---	---	---	---	---	---	---	---	MmugDNA.15702.1.S1_at_---
MmugDNA.15703.1.S1_at	205702_at	PHTF1	putative homeodomain transcription factor 1	chr1p13	10745	PHTF1	putative homeodomain transcription factor 1	---	707208	MmugDNA.15703.1.S1_at_PHTF1
MmugDNA.15704.1.S1_at	NA	NA	NA	NA	NA	NA	NA	NA	NA	MmugDNA.15704.1.S1_at_NA
MmugDNA.15704.1.S1_s_at	201549_x_at	KDM5B	lysine (K)-specific demethylase 5B	chr1q32.1	10765	---	---	---	---	MmugDNA.15704.1.S1_s_at_KDM5B
MmugDNA.15705.1.S1_at	1567030_at	SH3GLP2	SH3-domain GRB2-like pseudogene 2	chr17q11.2	6459	---	---	---	---	MmugDNA.15705.1.S1_at_SH3GLP2
MmugDNA.15706.1.S1_at	203515_s_at	PMVK	phosphomevalonate kinase	chr1q22	10654	PMVK	phosphomevalonate kinase	---	717014	MmugDNA.15706.1.S1_at_PMVK
MmugDNA.15707.1.S1_at	234308_at	TUBGCP6	tubulin, gamma complex associated protein 6	chr22q13.31-q13.33	85378	---	---	---	---	MmugDNA.15707.1.S1_at_TUBGCP6
MmugDNA.15708.1.S1_s_at	227132_at	ZNF706	zinc finger protein 706	chr8q22.3	51123	LOC710457	hypothetical protein LOC710457	---	710457	MmugDNA.15708.1.S1_s_at_ZNF706
MmugDNA.15709.1.S1_at	231572_at	LOC136242	Peptidase S1 domain-containing protein LOC136242	chr7q34	136242	LOC694908	similar to Anionic trypsin-1 precursor (Anionic trypsin I) (Pretrypsinogen I)	---	694908	MmugDNA.15709.1.S1_at_LOC136242
MmugDNA.15710.1.S1_at	212156_at	VPS39	vacuolar protein sorting 39 homolog (S. cerevisiae)	chr15q15.1	23339	VPS39	vacuolar protein sorting 39	---	707791	MmugDNA.15710.1.S1_at_VPS39
MmugDNA.15711.1.S1_at	235487_at	---	---	---	---	---	---	---	---	MmugDNA.15711.1.S1_at_---
MmugDNA.1571.1.S1_at	215843_s_at	TLL2	tolloid-like 2	chr10q23-q24	7093	---	---	---	---	MmugDNA.1571.1.S1_at_TLL2
MmugDNA.15712.1.S1_x_at	NA	NA	NA	NA	NA	NA	NA	NA	NA	MmugDNA.15712.1.S1_x_at_NA
MmugDNA.15713.1.S1_at	204585_s_at	L1CAM	L1 cell adhesion molecule	chrXq28	3897	L1CAM	L1 cell adhesion molecule	---	697504	MmugDNA.15713.1.S1_at_L1CAM
MmugDNA.15714.1.S1_at	238059_at	---	---	---	---	---	---	---	---	MmugDNA.15714.1.S1_at_---
MmugDNA.15715.1.S1_at	201145_at	HAX1	HCLS1 associated protein X-1	chr1q21.3	10456	LOC716495	similar to HCLS1 associated protein X-1 isoform a	---	716495	MmugDNA.15715.1.S1_at_HAX1
MmugDNA.15716.1.S1_at	212756_s_at	UBR2	ubiquitin protein ligase E3 component n-recognin 2	chr6p21.1	23304	LOC710500	Similar to RS21-C6 protein	---	710500	MmugDNA.15716.1.S1_at_UBR2
MmugDNA.15717.1.S1_at	230960_at	IGDCC3	immunoglobulin superfamily, DCC subclass, member 3	chr15q22.3-q23	9543	---	---	---	---	MmugDNA.15717.1.S1_at_IGDCC3
MmugDNA.15718.1.S1_at	216784_at	---	---	---	---	---	---	---	---	MmugDNA.15718.1.S1_at_---
MmugDNA.15719.1.S1_at	212760_at	UBR2	ubiquitin protein ligase E3 component n-recognin 2	chr6p21.1	23304	---	---	---	---	MmugDNA.15719.1.S1_at_UBR2
MmugDNA.157.1.S1_at	1554849_at	TOR1A	torsin family 1, member A (torsin A)	chr9q34	1861	---	---	---	---	MmugDNA.157.1.S1_at_TOR1A
MmugDNA.15720.1.S1_at	232240_at	DNHD1	dynein heavy chain domain 1	chr11p15.4	144132	LOC710482	similar to dynein, axonemal, heavy polypeptide 1	---	710482	MmugDNA.15720.1.S1_at_DNHD1
MmugDNA.15721.1.S1_at	219510_at	POLQ	polymerase (DNA directed), theta	chr3q13.33	10721	POLQ	polymerase (DNA directed), theta	---	713828	MmugDNA.15721.1.S1_at_POLQ
MmugDNA.1572.1.S1_at	1559882_at	SAMHD1	SAM domain and HD domain 1	chr20pter-q12	25939	---	---	---	---	MmugDNA.1572.1.S1_at_SAMHD1
MmugDNA.15722.1.S1_at	244081_at	---	---	---	---	---	---	---	---	MmugDNA.15722.1.S1_at_---
MmugDNA.15723.1.S1_at	202868_s_at	POP4	processing of precursor 4, ribonuclease P/MRP subunit (S. cerevisiae)	chr19q12	10775	POP4	processing of precursor 4, ribonuclease P/MRP subunit (S. cerevisiae)	---	700209	MmugDNA.15723.1.S1_at_POP4
MmugDNA.15724.1.S1_at	224226_at	PKD2L2	polycystic kidney disease 2-like 2	chr5q31	27039	---	---	---	---	MmugDNA.15724.1.S1_at_PKD2L2
MmugDNA.15725.1.S1_at	206174_s_at	PPP6C	protein phosphatase 6, catalytic subunit	chr9q33.3	5537	TSEN34	TRNA splicing endonuclease 34 homolog (S. cerevisiae)	---	693919	MmugDNA.15725.1.S1_at_PPP6C
MmugDNA.15726.1.S1_at	220356_at	CORIN	corin, serine peptidase	chr4p13-p12	10699	---	---	---	---	MmugDNA.15726.1.S1_at_CORIN
MmugDNA.15727.1.S1_at	231730_at	SEPSECS	Sep (O-phosphoserine) tRNA:Sec (selenocysteine) tRNA synthase	chr4p15.2	51091	SLA/LP	soluble liver antigen/liver pancreas antigen	---	693433	MmugDNA.15727.1.S1_at_SEPSECS
MmugDNA.15728.1.S1_at	241838_at	---	---	---	---	---	---	---	---	MmugDNA.15728.1.S1_at_---
MmugDNA.15729.1.S1_at	221988_at	C19orf42	chromosome 19 open reading frame 42	chr19p13.11	79086	---	---	---	---	MmugDNA.15729.1.S1_at_C19orf42
MmugDNA.15730.1.S1_at	1558568_a_at	---	---	---	---	---	---	---	---	MmugDNA.15730.1.S1_at_---
MmugDNA.15731.1.S1_at	236634_at	C8orf48	chromosome 8 open reading frame 48	chr8p22	157773	LOC700746	hypothetical protein LOC700746	---	700746	MmugDNA.15731.1.S1_at_C8orf48
MmugDNA.1573.1.S1_at	226799_at	FGD6	FYVE, RhoGEF and PH domain containing 6	chr12q22	55785	FGD6	FYVE, RhoGEF and PH domain containing 6	---	712132	MmugDNA.1573.1.S1_at_FGD6
MmugDNA.15732.1.S1_at	207324_s_at	DSC1	desmocollin 1	chr18q12.2|18q12.1	1823	DSC1	desmocollin 1	---	705332	MmugDNA.15732.1.S1_at_DSC1
MmugDNA.15733.1.S1_at	210810_s_at	SLC6A5	solute carrier family 6 (neurotransmitter transporter, glycine), member 5	chr11p15.2-p15.1	9152	SLC6A5	solute carrier family 6 (neurotransmitter transporter, glycine), member 5	---	704703	MmugDNA.15733.1.S1_at_SLC6A5
MmugDNA.15734.1.S1_at	1570533_at	CMPK1	cytidine monophosphate (UMP-CMP) kinase 1, cytosolic	chr1p32	51727	---	---	---	---	MmugDNA.15734.1.S1_at_CMPK1
MmugDNA.15735.1.S1_at	235670_at	STX11	syntaxin 11	chr6q24.2	8676	---	---	---	---	MmugDNA.15735.1.S1_at_STX11
MmugDNA.15736.1.S1_at	235415_at	RPRD2	regulation of nuclear pre-mRNA domain containing 2	chr1q21.2	23248	LOC715526	similar to CG15160-PA	---	715526	MmugDNA.15736.1.S1_at_RPRD2
MmugDNA.15737.1.S1_s_at	1553150_at	AOF1	amine oxidase (flavin containing) domain 1	chr6p22.3	221656	AOF1	similar to amine oxidase, flavin containing 1	---	704663	MmugDNA.15737.1.S1_s_at_AOF1
MmugDNA.15738.1.S1_at	209959_at	NR4A3	nuclear receptor subfamily 4, group A, member 3	chr9q22	8013	LOC701087	Similar to hepatocellular carcinoma antigen gene 520	---	701087	MmugDNA.15738.1.S1_at_NR4A3
MmugDNA.15739.1.S1_at	223243_s_at	EDEM3	ER degradation enhancer, mannosidase alpha-like 3	chr1q24-q25	80267	EDEM3	ER degradation enhancer, mannosidase alpha-like 3	---	714475	MmugDNA.15739.1.S1_at_EDEM3
MmugDNA.15740.1.S1_at	201252_at	PSMC4	proteasome (prosome, macropain) 26S subunit, ATPase, 4	chr19q13.11-q13.13	5704	PSMC4	proteasome (prosome, macropain) 26S subunit, ATPase, 4	---	699847	MmugDNA.15740.1.S1_at_PSMC4
MmugDNA.15741.1.S1_at	240380_at	---	---	---	---	LOC703730	hypothetical protein LOC703730	---	703730	MmugDNA.15741.1.S1_at_---
MmugDNA.1574.1.S1_at	230503_at	---	---	---	---	---	---	---	---	MmugDNA.1574.1.S1_at_---
MmugDNA.15742.1.S1_at	202102_s_at	BRD4	bromodomain containing 4	chr19p13.1	23476	LOC718195	similar to bromodomain-containing protein 4 isoform long	---	718195	MmugDNA.15742.1.S1_at_BRD4
MmugDNA.15743.1.S1_at	1553452_at	MYO1H	myosin IH	chr12q24.11	283446	---	---	---	---	MmugDNA.15743.1.S1_at_MYO1H
MmugDNA.15744.1.S1_at	207018_s_at	RAB27B	RAB27B, member RAS oncogene family	chr18q21.2	5874	RAB27B	RAB27B, member RAS oncogene family	---	694445	MmugDNA.15744.1.S1_at_RAB27B
MmugDNA.15745.1.S1_at	202103_at	BRD4	bromodomain containing 4	chr19p13.1	23476	---	---	---	---	MmugDNA.15745.1.S1_at_BRD4
MmugDNA.15746.1.S1_at	231719_at	IFRG15	interferon responsive gene 15	chr1q25.2	64163	LOC716691	similar to interferon responsive gene 15	---	716691	MmugDNA.15746.1.S1_at_IFRG15
MmugDNA.15747.1.S1_at	201628_s_at	RRAGA	Ras-related GTP binding A	chr9p22.1	10670	BRI3	Brain protein I3	---	706525	MmugDNA.15747.1.S1_at_RRAGA
MmugDNA.15748.1.S1_at	238619_at	---	---	---	---	---	---	---	---	MmugDNA.15748.1.S1_at_---
MmugDNA.15749.1.S1_at	213260_at	FOXC1	forkhead box C1	chr6p25	2296	FOXC1	forkhead box C1	---	722918	MmugDNA.15749.1.S1_at_FOXC1
MmugDNA.15750.1.S1_at	217096_at	PCLO	piccolo (presynaptic cytomatrix protein)	chr7q11.23-q21.3	27445	---	---	---	---	MmugDNA.15750.1.S1_at_PCLO
MmugDNA.15751.1.S1_at	206499_s_at	RCC1 /// SNHG3-RCC1	regulator of chromosome condensation 1 /// SNHG3-RCC1 readthrough transcript	chr1p36.1	1104 /// 751867	RCC1	regulator of chromosome condensation 1	---	711361	MmugDNA.15751.1.S1_at_RCC1 /// SN
MmugDNA.1575.1.S1_at	226467_at	TMCO7	transmembrane and coiled-coil domains 7	chr16q22.1	79613	TMCO7	transmembrane and coiled-coil domains 7	---	704499	MmugDNA.1575.1.S1_at_TMCO7
MmugDNA.15752.1.S1_at	243529_at	MARS2	methionyl-tRNA synthetase 2, mitochondrial	chr2q33.1	92935	LOC698275	similar to methionine-tRNA synthetase 2 precursor	---	698275	MmugDNA.15752.1.S1_at_MARS2
MmugDNA.15753.1.S1_at	232452_at	LOC148824	hypothetical LOC148824	chr1q44	148824	---	---	---	---	MmugDNA.15753.1.S1_at_LOC148824
MmugDNA.15754.1.S1_at	205393_s_at	CHEK1	CHK1 checkpoint homolog (S. pombe)	chr11q24-q24	1111	LOC713358	similar to CHK1 checkpoint homolog	---	713358	MmugDNA.15754.1.S1_at_CHEK1
MmugDNA.15755.1.S1_at	230234_at	---	---	---	---	---	---	---	---	MmugDNA.15755.1.S1_at_---
MmugDNA.15756.1.S1_at	201961_s_at	RNF41	ring finger protein 41	chr12q13.2	10193	MPDU1	Mannose-P-dolichol utilization defect 1	---	715962	MmugDNA.15756.1.S1_at_RNF41
MmugDNA.15757.1.S1_at	1561575_at	---	---	---	---	---	---	---	---	MmugDNA.15757.1.S1_at_---
MmugDNA.15758.1.S1_at	1556758_at	C10orf18	chromosome 10 open reading frame 18	chr10p15.1	54906	---	---	---	---	MmugDNA.15758.1.S1_at_C10orf18
MmugDNA.15759.1.S1_at	222572_at	PPM2C	protein phosphatase 2C, magnesium-dependent, catalytic subunit	chr8q22.1	54704	LOC699172	hypothetical protein LOC699172	---	699172	MmugDNA.15759.1.S1_at_PPM2C
MmugDNA.15760.1.S1_at	222994_at	PRDX5	peroxiredoxin 5	chr11q13	25824	LOC702978	Similar to 60S ribosomal protein L7a	---	702978	MmugDNA.15760.1.S1_at_PRDX5
MmugDNA.15761.1.S1_at	240177_at	---	---	---	---	---	---	---	---	MmugDNA.15761.1.S1_at_---
MmugDNA.1576.1.S1_at	NA	NA	NA	NA	NA	NA	NA	NA	NA	MmugDNA.1576.1.S1_at_NA
MmugDNA.15762.1.S1_at	220570_at	RETN	resistin	chr19p13.2	56729	RETN	resistin	---	708781	MmugDNA.15762.1.S1_at_RETN
MmugDNA.15763.1.S1_at	222755_s_at	CHD7	chromodomain helicase DNA binding protein 7	chr8q12.2	55636	LOC706471	similar to chromodomain helicase DNA binding protein 7	---	706471	MmugDNA.15763.1.S1_at_CHD7
MmugDNA.15764.1.S1_at	236625_at	---	---	---	---	---	---	---	---	MmugDNA.15764.1.S1_at_---
MmugDNA.15765.1.S1_at	239239_at	---	---	---	---	---	---	---	---	MmugDNA.15765.1.S1_at_---
MmugDNA.15766.1.S1_at	229126_at	TMEM19	transmembrane protein 19	chr12q21.1	55266	---	---	---	---	MmugDNA.15766.1.S1_at_TMEM19
MmugDNA.15767.1.S1_at	217373_x_at	MDM2	Mdm2 p53 binding protein homolog (mouse)	chr12q14.3-q15	4193	MDM2	Mdm2 p53 binding protein homolog (mouse)	---	718284	MmugDNA.15767.1.S1_at_MDM2
MmugDNA.15768.1.S1_at	215483_at	AKAP9	A kinase (PRKA) anchor protein (yotiao) 9	chr7q21-q22	10142	---	---	---	---	MmugDNA.15768.1.S1_at_AKAP9
MmugDNA.15769.1.S1_at	235152_at	---	---	---	---	---	---	---	---	MmugDNA.15769.1.S1_at_---

MmugDNA.15770.1.S1_at	230615_at	DUOXA2	dual oxidase maturation factor 2	chr15q15.1	405753	LOC712992	similar to Numb-interacting protein 2	---	712992	MmugDNA.15770.1.S1_at_DUOXA2
MmugDNA.15771.1.S1_at	1570259_at	LIMS1	LIM and senescent cell antigen-like domains 1	chr2q12.3-q13	3987	---	---	---	---	MmugDNA.15771.1.S1_at_LIMS1
MmugDNA.1577.1.S1_at	215643_at	---	---	---	---	---	---	---	---	MmugDNA.1577.1.S1_at_---
MmugDNA.15772.1.S1_at	206079_at	CHML	choroideremia-like (Rab escort protein 2)	chr1q42-qter	1122	---	---	---	---	MmugDNA.15772.1.S1_at_CHML
MmugDNA.15773.1.S1_at	208621_s_at	EZR	ezrin	chr6q25.2-q26	7430	LOC704716	similar to villin 2	---	704716	MmugDNA.15773.1.S1_at_EZR
MmugDNA.15774.1.S1_at	208622_s_at	EZR	ezrin	chr6q25.2-q26	7430	LOC704716	similar to villin 2	---	704716	MmugDNA.15774.1.S1_at_EZR
MmugDNA.15775.1.S1_at	217782_s_at	GPS1	G protein pathway suppressor 1	chr17q25.3	2873	LOC719343	similar to G protein pathway suppressor 1 isoform 1	---	719343	MmugDNA.15775.1.S1_at_GPS1
MmugDNA.15776.1.S1_at	236237_at	---	---	---	---	---	---	---	---	MmugDNA.15776.1.S1_at_---
MmugDNA.15777.1.S1_at	221229_s_at	TRMT61B	tRNA methyltransferase 61 homolog B (S. cerevisiae)	chr2p23.2	55006	LOC703419	hypothetical protein LOC703419	---	703419	MmugDNA.15777.1.S1_at_TRMT61B
MmugDNA.15778.1.S1_at	237368_at	C3orf35	Chromosome 3 open reading frame 35	chr3p22.2	339883	---	---	---	---	MmugDNA.15778.1.S1_at_C3orf35
MmugDNA.15779.1.S1_at	NA	NA	NA	NA	NA	NA	NA	NA	NA	MmugDNA.15779.1.S1_at_NA
MmugDNA.15780.1.S1_at	208066_s_at	GTF2B	general transcription factor IIB	chr1p22-p21	2959	GTF2B	general transcription factor IIB	---	694770	MmugDNA.15780.1.S1_at_GTF2B
MmugDNA.15781.1.S1_at	224909_s_at	PREX1	phosphatidylinositol-3,4,5-trisphosphate-dependent Rac exchange factor 1	chr20q13.13	57580	PREX1	PREX1 protein	---	714351	MmugDNA.15781.1.S1_at_PREX1
MmugDNA.1578.1.S1_s_at	213646_x_at	TUBA1B	tubulin, alpha 1b	chr12q13.12	10376	LOC710110	similar to tubulin alpha 6	---	710110	MmugDNA.1578.1.S1_s_at_TUBA1B
MmugDNA.15782.1.S1_at	224925_at	PREX1	phosphatidylinositol-3,4,5-trisphosphate-dependent Rac exchange factor 1	chr20q13.13	57580	PREX1	PREX1 protein	---	714351	MmugDNA.15782.1.S1_at_PREX1
MmugDNA.15783.1.S1_s_at	217319_x_at	CYP4A22	cytochrome P450, family 4, subfamily A, polypeptide 22	chr1p33	284541	---	---	---	---	MmugDNA.15783.1.S1_s_at_CYP4A22
MmugDNA.15784.1.S1_at	1557570_a_at	LOC285084	hypothetical protein LOC285084	chr2q31.1	285084	---	---	---	---	MmugDNA.15784.1.S1_at_LOC285084
MmugDNA.15785.1.S1_at	222332_at	---	---	---	---	---	---	---	---	MmugDNA.15785.1.S1_at_---
MmugDNA.15786.1.S1_at	241852_at	---	---	---	---	---	---	---	---	MmugDNA.15786.1.S1_at_---
MmugDNA.15787.1.S1_at	232396_at	---	---	---	---	---	---	---	---	MmugDNA.15787.1.S1_at_---
MmugDNA.15788.1.S1_at	232367_x_at	ZNF598	zinc finger protein 598	chr16p13.3	90850	---	---	---	---	MmugDNA.15788.1.S1_at_ZNF598
MmugDNA.15789.1.S1_at	239058_at	---	---	---	---	---	---	---	---	MmugDNA.15789.1.S1_at_---
MmugDNA.15790.1.S1_at	231972_at	---	---	---	---	---	---	---	---	MmugDNA.15790.1.S1_at_---
MmugDNA.15791.1.S1_x_at	216573_at	IGL	immunoglobulin lambda locus	chr22q11.1-q11.2	3535	LOC704043	similar to Ig lambda chain V-I region BL2 precursor	---	704043	MmugDNA.15791.1.S1_x_at_IGL@
MmugDNA.1579.1.S1_at	216767_at	---	---	---	---	---	---	---	---	MmugDNA.1579.1.S1_at_---
MmugDNA.15792.1.S1_at	1559567_at	---	---	---	---	---	---	---	---	MmugDNA.15792.1.S1_at_---
MmugDNA.15793.1.S1_at	1554022_at	CCDC13	coiled-coil domain containing 13	chr3p22.1	152206	LOC716174	similar to coiled-coil domain containing 13	---	716174	MmugDNA.15793.1.S1_at_CCDC13
MmugDNA.15794.1.S1_at	1560550_at	---	---	---	---	---	---	---	---	MmugDNA.15794.1.S1_at_---
MmugDNA.15795.1.S1_at	1563057_at	---	---	---	---	---	---	---	---	MmugDNA.15795.1.S1_at_---
MmugDNA.15796.1.S1_at	1554846_at	CCDC158	coiled-coil domain containing 158	chr4q21.1	339965	---	---	---	---	MmugDNA.15796.1.S1_at_CCDC158
MmugDNA.15797.1.S1_at	1561335_at	LOC440602	hypothetical gene supported by BC042100	chr1p13.3	440602	---	---	---	---	MmugDNA.15797.1.S1_at_LOC440602
MmugDNA.15798.1.S1_s_at	212671_s_at	HLA-DQA1 /// HLA-DQ	major histocompatibility complex, class II, DQ alpha 1 /// major histocompatibility complex, class II, D	chr6p21.3	3117 /// 3118	MAMU-DQA1	major histocompatibility complex, class II, DQ alpha 1	---	720582	MmugDNA.15798.1.S1_s_at_HLA-DQA
MmugDNA.15799.1.S1_at	1561167_at	---	---	---	---	---	---	---	---	MmugDNA.15799.1.S1_at_---
MmugDNA.15800.1.S1_at	229136_s_at	---	---	---	---	---	---	---	---	MmugDNA.15800.1.S1_at_---
MmugDNA.15801.1.S1_at	206533_at	CHRNA5	cholinergic receptor, nicotinic, alpha 5	chr15q24	1138	CHRNA5	cholinergic receptor, nicotinic, alpha 5	---	574191	MmugDNA.15801.1.S1_at_CHRNA5
MmugDNA.1580.1.S1_at	1553994_at	NT5E	5'-nucleotidase, ecto (CD73)	chr6q14-q21	4907	NT5E	5'-nucleotidase, ecto (CD73)	---	696509	MmugDNA.1580.1.S1_at_NT5E
MmugDNA.15802.1.S1_at	202406_s_at	TIAL1	TIA1 cytotoxic granule-associated RNA binding protein-like 1	chr10q	7073	TIAL1	TIA1 cytotoxic granule-associated RNA binding protein-like 1	---	703249	MmugDNA.15802.1.S1_at_TIAL1
MmugDNA.15803.1.S1_at	NA	NA	NA	NA	NA	NA	NA	NA	NA	MmugDNA.15803.1.S1_at_NA
MmugDNA.15804.1.S1_at	211975_at	ARFGAP2	ADP-ribosylation factor GTPase activating protein 2	chr11p11.2-p11.12	84364	LOC713871	similar to zinc finger protein 289, ID1 regulated	---	713871	MmugDNA.15804.1.S1_at_ARFGAP2
MmugDNA.15805.1.S1_at	242791_at	FBXO3	F-box protein 3	chr11p13	26273	---	---	---	---	MmugDNA.15805.1.S1_at_FBXO3
MmugDNA.15806.1.S1_at	236935_at	---	---	---	---	---	---	---	---	MmugDNA.15806.1.S1_at_---
MmugDNA.15807.1.S1_at	243926_at	---	---	---	---	---	---	---	---	MmugDNA.15807.1.S1_at_---
MmugDNA.15808.1.S1_s_at	202443_x_at	NOTCH2	Notch homolog 2 (Drosophila)	chr1p13-p11	4853	LOC713798	similar to notch 2 preproprotein	---	713798	MmugDNA.15808.1.S1_s_at_NOTCH2
MmugDNA.15809.1.S1_at	223828_s_at	LGALS12	lectin, galactoside-binding, soluble, 12	chr11q13	85329	LOC718300	similar to lectin, galactoside-binding, soluble, 12 (galectin 12)	---	718300	MmugDNA.15809.1.S1_at_LGALS12
MmugDNA.15810.1.S1_at	229050_s_at	SNHG7	small nucleolar RNA host gene 7 (non-protein coding)	chr9q34.3	84973	---	---	---	---	MmugDNA.15810.1.S1_at_SNHG7
MmugDNA.15811.1.S1_at	225235_at	TSPAN17	tetraspanin 17	chr5q35.3	26262	TSPAN17	tetraspanin 17	---	697739	MmugDNA.15811.1.S1_at_TSPAN17
MmugDNA.1581.1.S1_at	NA	NA	NA	NA	NA	NA	NA	NA	NA	MmugDNA.1581.1.S1_at_NA
MmugDNA.1581.1.S1_s_at	207132_x_at	PFDN5	prefoldin subunit 5	chr12q12	5204	LOC719420 /// PFDN5	similar to prefoldin 5 isoform 1 /// prefoldin subunit 5	---	702355 /// 719420	MmugDNA.1581.1.S1_s_at_PFDN5
MmugDNA.15812.1.S1_at	233887_at	GPR126	G protein-coupled receptor 126	chr6q24.1	57211	LOC712378	Similar to ribosomal protein L19	---	712378	MmugDNA.15812.1.S1_at_GPR126
MmugDNA.15813.1.S1_at	234245_at	---	---	---	---	---	---	---	---	MmugDNA.15813.1.S1_at_---
MmugDNA.15814.1.S1_at	216742_at	---	---	---	---	---	---	---	---	MmugDNA.15814.1.S1_at_---
MmugDNA.15815.1.S1_at	213683_at	ACSL6	acyl-CoA synthetase long-chain family member 6	chr5q31	23305	LOC706847	similar to acyl-CoA synthetase long-chain family member 6 isoform b	---	706847	MmugDNA.15815.1.S1_at_ACSL6
MmugDNA.15816.1.S1_at	225432_s_at	CSRP2BP	CSRP2 binding protein	chr20p11.23	57325	CSRP2BP	CSRP2 binding protein	---	697187	MmugDNA.15816.1.S1_at_CSRP2BP
MmugDNA.15817.1.S1_at	207859_s_at	CHRNB3	cholinergic receptor, nicotinic, beta 3	chr8p11.2	1142	CHRNB3	cholinergic receptor, nicotinic, beta 3	---	720301	MmugDNA.15817.1.S1_at_CHRNB3
MmugDNA.15818.1.S1_at	207516_at	CHRNB4	cholinergic receptor, nicotinic, beta 4	chr15q24	1143	LOC716649	similar to Neuronal acetylcholine receptor protein, beta-4 subunit precursor	---	716649	MmugDNA.15818.1.S1_at_CHRNB4
MmugDNA.15819.1.S1_s_at	210097_s_at	NOL7	nucleolar protein 7, 27kDa	chr6p23	51406	NOL7	nucleolar protein 7, 27kDa	---	701335	MmugDNA.15819.1.S1_s_at_NOL7
MmugDNA.158.1.S1_at	NA	NA	NA	NA	NA	NA	NA	NA	NA	MmugDNA.158.1.S1_at_NA
MmugDNA.158.1.S1_s_at	232209_x_at	HM13	histocompatibility (minor) 13	chr20q11.21	81502	LOC713216	similar to minor histocompatibility antigen 13 isoform 3	---	713216	MmugDNA.158.1.S1_s_at_HM13
MmugDNA.15820.1.S1_at	242999_at	ARHGEF7	Rho guanine nucleotide exchange factor (GEF) 7	chr13q34	8874	ARHGEF7	Rho guanine nucleotide exchange factor 7	---	695530	MmugDNA.15820.1.S1_at_ARHGEF7
MmugDNA.15821.1.S1_at	231899_at	ZC3H12C	zinc finger CCCH-type containing 12C	chr11q22.3	85463	LOC708921	similar to zinc finger CCCH type containing 12A	---	708921	MmugDNA.15821.1.S1_at_ZC3H12C
MmugDNA.1582.1.S1_at	229520_s_at	C14orf118	chromosome 14 open reading frame 118	chr14q22.1-q24.3	55668	LOC703697	similar to G patch domain containing 2 (predicted)	---	703697	MmugDNA.1582.1.S1_at_C14orf118
MmugDNA.15822.1.S1_s_at	NA	NA	NA	NA	NA	NA	NA	NA	NA	MmugDNA.15822.1.S1_s_at_NA
MmugDNA.15823.1.S1_at	1560513_at	LOC400568	hypothetical LOC400568	chr17p13.2	400568	---	---	---	---	MmugDNA.15823.1.S1_at_LOC400568
MmugDNA.15824.1.S1_at	244655_at	LOC100132798	Similar to hCG1774772	chr15q26.2	100132798	---	---	---	---	MmugDNA.15824.1.S1_at_LOC100132
MmugDNA.15825.1.S1_at	1558794_at	LOC728190	Hypothetical LOC728190	chr10q23.2	728190	---	---	---	---	MmugDNA.15825.1.S1_at_LOC728190
MmugDNA.15826.1.S1_at	238003_at	HEPACAM	hepatocyte cell adhesion molecule	chr11q24.2	220296	LOC716321	similar to hepatocyte cell adhesion molecule	---	716321	MmugDNA.15826.1.S1_at_HEPACAM
MmugDNA.15827.1.S1_at	229428_at	LOC100132418	Uncharacterized PRO1102-like protein LOC100132418	chr10q11.23	100132418	---	---	---	---	MmugDNA.15827.1.S1_at_LOC100132
MmugDNA.15828.1.S1_at	1553535_a_at	RANGAP1	Ran GTPase activating protein 1	chr22q13	5905	RANGAP1	Ran GTPase activating protein 1	---	710698	MmugDNA.15828.1.S1_at_RANGAP1
MmugDNA.15829.1.S1_at	226148_at	ZBTB44	zinc finger and BTB domain containing 44	chr11q24.3	29068	LOC715415	similar to BTB (POZ) domain containing 15	---	715415	MmugDNA.15829.1.S1_at_ZBTB44
MmugDNA.15830.1.S1_at	241606_s_at	TRUB1	TruB pseudouridine (psi) synthase homolog 1 (E. coli)	chr10q25.3	142940	---	---	---	---	MmugDNA.15830.1.S1_at_TRUB1
MmugDNA.15831.1.S1_at	230077_at	LOC220729 /// SDHA //	succinate dehydrogenase complex, subunit A, flavoprotein pseudogene /// succinate dehydrogenase	chr3q29 /// chr5p15	220729 /// 255812 /// 6	---	---	---	---	MmugDNA.15831.1.S1_at_LOC220729
MmugDNA.1583.1.S1_at	207537_at	PFKFB1	6-phosphofructo-2-kinase/fructose-2,6-biphosphatase 1	chrXp11.21	5207	PFKFB1	6-phosphofructo-2-kinase/fructose-2,6-biphosphatase 1	---	701969	MmugDNA.1583.1.S1_at_PFKFB1
MmugDNA.15832.1.S1_at	239450_at	---	---	---	---	---	---	---	---	MmugDNA.15832.1.S1_at_---
MmugDNA.15833.1.S1_at	NA	NA	NA	NA	NA	NA	NA	NA	NA	MmugDNA.15833.1.S1_at_NA
MmugDNA.15834.1.S1_at	231463_at	CNTD1	cyclin N-terminal domain containing 1	chr17q21.31	124817	LOC707820	similar to WNK lysine deficient protein kinase 4	---	707820	MmugDNA.15834.1.S1_at_CNTD1
MmugDNA.15835.1.S1_at	205567_at	CHST1	carbohydrate (keratan sulfate Gal-6) sulfotransferase 1	chr11p11.2-p11.1	8534	LOC715176	similar to carbohydrate (keratan sulfate Gal-6) sulfotransferase 1	---	715176	MmugDNA.15835.1.S1_at_CHST1
MmugDNA.15836.1.S1_at	227782_at	ZBTB7C	zinc finger and BTB domain containing 7C	chr18q21.1	201501	ZBTB7A	similar to zinc finger and BTB domain containing 36	---	705033	MmugDNA.15836.1.S1_at_ZBTB7C
MmugDNA.15837.1.S1_at	215134_at	PI4K2A	phosphatidylinositol 4-kinase type 2 alpha	chr10q24	55361	PI4K2A	similar to phosphatidylinositol 4-kinase type II	---	707201	MmugDNA.15837.1.S1_at_PI4K2A
MmugDNA.15838.1.S1_at	235324_at	SFRS3	splicing factor, arginine/serine-rich 3	chr6p21	6428	---	---	---	---	MmugDNA.15838.1.S1_at_SFRS3
MmugDNA.15839.1.S1_at	1555871_at	LOC284900	hypothetical LOC284900	chr22q12.1	284900	---	---	---	---	MmugDNA.15839.1.S1_at_LOC284900
MmugDNA.15840.1.S1_at	238726_at	---	---	---	---	---	---	---	---	MmugDNA.15840.1.S1_at_---
MmugDNA.15841.1.S1_at	200811_at	CIRBP	cold inducible RNA binding protein	chr19p13.3	1153	LOC709217	Similar to cDNA sequence BC021395	---	709217	MmugDNA.15841.1.S1_at_CIRBP
MmugDNA.1584.1.S1_at	226498_at	---	---	---	---	---	---	---	---	MmugDNA.1584.1.S1_at_---
MmugDNA.15842.1.S1_at	228869_at	---	---	---	---	---	---	---	---	MmugDNA.15842.1.S1_at_---
MmugDNA.15844.1.S1_at	1558118_at	DGCR5	DiGeorge syndrome critical region gene 5 (non-protein coding)	chr22q11	26220	---	---	---	---	MmugDNA.15844.1.S1_at_DGCR5
MmugDNA.15845.1.S1_at	230300_at	---	---	---	---	---	---	---	---	MmugDNA.15845.1.S1_at_---
MmugDNA.15846.1.S1_at	225064_at	RABEP1	rabaptin, RAB GTPase binding effector protein 1	chr17p13.2	9135	LOC711646	similar to rabaptin, RAB GTPase binding effector protein 1	---	711646	MmugDNA.15846.1.S1_at_RABEP1
MmugDNA.15847.1.S1_at	225092_at	RABEP1	rabaptin, RAB GTPase binding effector protein 1	chr17p13.2	9135	LOC711646	similar to rabaptin, RAB GTPase binding effector protein 1	---	711646	MmugDNA.15847.1.S1_at_RABEP1
MmugDNA.15848.1.S1_at	235575_at	---	---	---	---	---	---	---	---	MmugDNA.15848.1.S1_at_---
MmugDNA.15849.1.S1_s_at	214042_s_at	RPL22	ribosomal protein L22	chr1p36.3-p36.2	6146	LOC703050	similar to 60S ribosomal protein L22 (Heparin-binding protein HBp15)	---	703050	MmugDNA.15849.1.S1_s_at_RPL22
MmugDNA.15850.1.S1_at	214202_at	---	---	---	---	---	---	---	---	MmugDNA.15850.1.S1_at_---
MmugDNA.15851.1.S1_at	212624_s_at	CHN1	chimerin (chimaerin) 1	chr2q31.1	1123	CHN1	chimerin (chimaerin) 1	---	698697	MmugDNA.15851.1.S1_at_CHN1
MmugDNA.1585.1.S1_at	229514_at	C14orf118	chromosome 14 open reading frame 118	chr14q22.1-q24.3	55668	---	---	---	---	MmugDNA.1585.1.S1_at_C14orf118
MmugDNA.15852.1.S1_at	244160_at	---	---	---	---	---	---	---	---	MmugDNA.15852.1.S1_at_---
MmugDNA.15854.1.S1_at	242799_at	---	---	---	---	---	---	---	---	MmugDNA.15854.1.S1_at_---
MmugDNA.15855.1.S1_at	1564701_at	---	---	---	---	---	---	---	---	MmugDNA.15855.1.S1_at_---
MmugDNA.15856.1.S1_at	228653_at	SAMD5	sterile alpha motif domain containing 5	chr6q24.3	389432	---	---	---	---	MmugDNA.15856.1.S1_at_SAMD5
MmugDNA.15857.1.S1_s_at	209735_at	ABCG2	ATP-binding cassette, sub-family G (WHITE), member 2	chr4q22	9429	ABCG3	breast cancer resistance protein	---	574307	MmugDNA.15857.1.S1_s_at_ABCG2
MmugDNA.15858.1.S1_at	237121_at	---	---	---	---	---	---	---	---	MmugDNA.15858.1.S1_at_---
MmugDNA.15859.1.S1_at	216996_s_at	FASTKD2	FAST kinase domains 2	chr2q33.3	22868	LOC709702	hypothetical protein LOC709702	---	709702	MmugDNA.15859.1.S1_at_FASTKD2
MmugDNA.15859.1.S1_s_at	NA	NA	NA	NA	NA	NA	NA	NA	NA	MmugDNA.15859.1.S1_s_at_NA
MmugDNA.15860.1.S1_at	205295_at	CKMT2	creatine kinase, mitochondrial 2 (sarcomeric)	chr5q13.3	1160	CKMT2	creatine kinase, mitochondrial 2 (sarcomeric)	---	711434	MmugDNA.15860.1.S1_at_CKMT2
MmugDNA.15861.1.S1_s_at	201897_s_at	CKS1B	CDC28 protein kinase regulatory subunit 1B	chr1q21.2	1163	LOC717164	similar to Cyclin-dependent kinases regulatory subunit 1 (CKS-1) (Sid 1334)	---	717164	MmugDNA.15861.1.S1_s_at_CKS1B
MmugDNA.15862.1.S1_at	204170_s_at	CKS2	CDC28 protein kinase regulatory subunit 2	chr9q22	1164	LOC697324	similar to CDC28 protein kinase 2	---	697324	MmugDNA.15862.1.S1_at_CKS2
MmugDNA.15863.1.S1_at	225337_at	ABHD2	abhydrolase domain containing 2	chr15q26.1	11057	---	---	---	---	MmugDNA.15863.1.S1_at_ABHD2
MmugDNA.15864.1.S1_at	1554771_at	---	---	---	---	---	---	---	---	MmugDNA.15864.1.S1_at_---
MmugDNA.15865.1.S1_at	209831_x_at	DNASE2	deoxyribonuclease II, lysosomal	chr19p13.2	1777	LOC716802	hypothetical protein LOC716802	---	716802	MmugDNA.15865.1.S1_at_DNASE2
MmugDNA.15866.1.S1_at	1569723_a_at	SPIRE2	spire homolog 2 (Drosophila)	chr16q24	84501	---	---	---	---	MmugDNA.15866.1.S1_at_SPIRE2

MmugDNA.15867.1.S1_at	220114_s_at	STAB2	stabilin 2	chr12q23.3		55576	LOC699023	similar to stabilin 2 precursor	---		699023	MmugDNA.15867.1.S1_at_STAB2
MmugDNA.15868.1.S1_at	1560225_at	CNR1	Cannabinoid receptor 1 (brain)	chr6q14-q15		1268	---	---	---	---		MmugDNA.15868.1.S1_at_CNR1
MmugDNA.15869.1.S1_at	208969_at	NDUFA9	NADH dehydrogenase (ubiquinone) 1 alpha subcomplex, 9, 39kDa	chr12p13.3		4704	LOC693776	hypothetical protein LOC693776	---		693776	MmugDNA.15869.1.S1_at_NDUFA9
MmugDNA.15870.1.S1_at	49679_s_at	EDEM2	ER degradation enhancer, mannosidase alpha-like 2	chr20q11.22		55741	---	---	---	---		MmugDNA.15870.1.S1_at_EDEM2
MmugDNA.15871.1.S1_at	222200_s_at	BSDC1	BSD domain containing 1	chr1p35.1		55108	LOC708083	similar to BSD domain containing 1	---		708083	MmugDNA.15871.1.S1_at_BSDC1
MmugDNA.1587.1.S1_at	211580_s_at	PIK3R3	phosphoinositide-3-kinase, regulatory subunit 3 (gamma)	chr1p34.1		8503	PIK3R3	phosphoinositide-3-kinase, regulatory subunit 3 (gamma)	---		707310	MmugDNA.1587.1.S1_at_PIK3R3
MmugDNA.15872.1.S1_at	1570433_at	TMPRSS2	transmembrane protease, serine 2	chr21q22.3		7113	---	---	---	---		MmugDNA.15872.1.S1_at_TMPRSS2
MmugDNA.15873.1.S1_at	1557439_at	---	---	---	---		---	---	---	---		MmugDNA.15873.1.S1_at_---
MmugDNA.15874.1.S1_at	230444_at	---	---	---	---		---	---	---	---		MmugDNA.15874.1.S1_at_---
MmugDNA.15875.1.S1_at	210959_s_at	SRD5A1	steroid-5-alpha-reductase, alpha polypeptide 1 (3-oxo-5 alpha-steroid delta 4-dehydrogenase alpha 1	chr5p15		6715	LOC712079	Similar to barrier to autointegration factor 1	---		712079	MmugDNA.15875.1.S1_at_SRD5A1
MmugDNA.15876.1.S1_at	241780_at	---	---	---	---		---	---	---	---		MmugDNA.15876.1.S1_at_---
MmugDNA.15877.1.S1_at	232016_at	MTMR15	Myotubularin related protein 15	chr15q13.2-q13.3		22909	---	---	---	---		MmugDNA.15877.1.S1_at_MTMR15
MmugDNA.15878.1.S1_at	202442_at	AP3S1	adaptor-related protein complex 3, sigma 1 subunit	chr5q22		1176	AP3S1	adaptor-related protein complex 3, sigma 1 subunit	---		696644	MmugDNA.15878.1.S1_at_AP3S1
MmugDNA.15879.1.S1_at	208730_x_at	RAB2A	RAB2A, member RAS oncogene family	chr8q12.1		5862	LOC705899	hypothetical protein LOC705899	---		705899	MmugDNA.15879.1.S1_at_RAB2A
MmugDNA.15880.1.S1_at	1559049_a_at	---	---	---	---		---	---	---	---		MmugDNA.15880.1.S1_at_---
MmugDNA.15881.1.S1_at	208731_at	RAB2A	RAB2A, member RAS oncogene family	chr8q12.1		5862	LOC699383	similar to RAB2, member RAS oncogene family	---		699383	MmugDNA.15881.1.S1_at_RAB2A
MmugDNA.1588.1.S1_at	213976_at	CIZ1	CDKN1A interacting zinc finger protein 1	chr9q34.1		25792	LOC717725	similar to Cip1-interacting zinc finger protein	---		717725	MmugDNA.1588.1.S1_at_CIZ1
MmugDNA.15882.1.S1_at	208732_at	RAB2A	RAB2A, member RAS oncogene family	chr8q12.1		5862	LOC699383	similar to RAB2, member RAS oncogene family	---		699383	MmugDNA.15882.1.S1_at_RAB2A
MmugDNA.15883.1.S1_at	208733_at	RAB2A	RAB2A, member RAS oncogene family	chr8q12.1		5862	LOC699383	similar to RAB2, member RAS oncogene family	---		699383	MmugDNA.15883.1.S1_at_RAB2A
MmugDNA.15884.1.S1_at	1552998_at	DEFB125	defensin, beta 125	chr20p13		245938	LOC714029	similar to beta defensin 125 preproprotein	---		714029	MmugDNA.15884.1.S1_at_DEFB125
MmugDNA.15885.1.S1_at	217312_s_at	COL7A1	collagen, type VII, alpha 1	chr3p21.1		1294	COL7A1	collagen, type VII, alpha 1	---		709354	MmugDNA.15885.1.S1_at_COL7A1
MmugDNA.15886.1.S1_at	242690_at	ATP11C	ATPase, class VI, type 11C	chrXq27.1		286410	LOC695802	similar to ATPase, Class VI, type 11C isoform b	---		695802	MmugDNA.15886.1.S1_at_ATP11C
MmugDNA.15887.1.S1_at	201735_s_at	CLCN3	chloride channel 3	chr4q33		1182	LOC694472	similar to chloride channel 3 isoform c	---		694472	MmugDNA.15887.1.S1_at_CLCN3
MmugDNA.15888.1.S1_at	227475_at	FOXQ1	forkhead box Q1	chr6p25		94234	FOXQ1	forkhead box Q1	---		722908	MmugDNA.15888.1.S1_at_FOXQ1
MmugDNA.15889.1.S1_at	1557065_at	YLPM1	YLP motif containing 1	chr14q24.3		56252	---	---	---	---		MmugDNA.15889.1.S1_at_YLPM1
MmugDNA.15890.1.S1_at	215119_at	MYO16	myosin XVI	chr13q33.3		23026	---	---	---	---		MmugDNA.15890.1.S1_at_MYO16
MmugDNA.15891.1.S1_at	1554719_at	NDUFA10	NADH dehydrogenase (ubiquinone) 1 alpha subcomplex, 10, 42kDa	chr2q37.3		4705	---	---	---	---		MmugDNA.15891.1.S1_at_NDUFA10
MmugDNA.1589.1.S1_at	228123_s_at	ABHD12	abhydrolase domain containing 12	chr20p11.21		26090	LOC700604	Similar to 60S ribosomal protein L21	---		700604	MmugDNA.1589.1.S1_at_ABHD12
MmugDNA.15892.1.S1_at	241250_at	---	---	---	---		---	---	---	---		MmugDNA.15892.1.S1_at_---
MmugDNA.15893.1.S1_at	213538_at	SON	SON DNA binding protein	chr21q22.1-q22.2|21q22.11		6651	---	---	---	---		MmugDNA.15893.1.S1_at_SON
MmugDNA.15894.1.S1_at	206005_s_at	KIAA1009	KIAA1009	chr6q14.3		22832	KIAA1009	KIAA1009	---		695775	MmugDNA.15894.1.S1_at_KIAA1009
MmugDNA.15895.1.S1_at	212777_at	SOS1	son of sevenless homolog 1 (Drosophila)	chr2p22-p21		6654	LOC713777	similar to son of sevenless homolog 1	---		713777	MmugDNA.15895.1.S1_at_SOS1
MmugDNA.15896.1.S1_at	215637_at	TSGA14	testis specific, 14	chr7q32		95681	---	---	---	---		MmugDNA.15896.1.S1_at_TSGA14
MmugDNA.15897.1.S1_at	212780_at	SOS1	son of sevenless homolog 1 (Drosophila)	chr2p22-p21		6654	LOC713777	similar to son of sevenless homolog 1	---		713777	MmugDNA.15897.1.S1_at_SOS1
MmugDNA.15898.1.S1_at	NA	NA	NA	NA	NA		NA	NA	NA	NA		MmugDNA.15898.1.S1_at_NA
MmugDNA.15898.1.S1_s_at	201972_at	ATP6V1A	ATPase, H+ transporting, lysosomal 70kDa, V1 subunit A	chr3q13.2-q13.31		523	LOC696878	similar to ATPase, H+ transporting, lysosomal 70kD, V1 subunit A, isoform 1	---		696878	MmugDNA.15898.1.S1_s_at_ATP6V1A
MmugDNA.15899.1.S1_at	227474_at	LOC654433	hypothetical LOC654433	chr2q13		654433	---	---	---	---		MmugDNA.15899.1.S1_at_LOC654433
MmugDNA.15900.1.S1_at	1561055_at	---	---	---	---		---	---	---	---		MmugDNA.15900.1.S1_at_---
MmugDNA.15901.1.S1_at	229594_at	SPTY2D1	SPT2, Suppressor of Ty, domain containing 1 (S. cerevisiae)	chr11p15.1		144108	SPTY2D1	SPT2, Suppressor of Ty, domain containing 1 (S. cerevisiae)	---		693282	MmugDNA.15901.1.S1_at_SPTY2D1
MmugDNA.1590.1.S1_at	1568878_at	---	---	---	---		---	---	---	---		MmugDNA.1590.1.S1_at_---
MmugDNA.15902.1.S1_at	241647_x_at	---	---	---	---		---	---	---	---		MmugDNA.15902.1.S1_at_---
MmugDNA.15903.1.S1_at	234853_s_at	EFCAB6	EF-hand calcium binding domain 6	chr22q13.1-q13.33		64800	---	---	---	---		MmugDNA.15903.1.S1_at_EFCAB6
MmugDNA.15904.1.S1_at	222918_at	RAB9B	RAB9B, member RAS oncogene family	chrXq22.1-q22.3		51209	RAB9B	RAB9B, member RAS oncogene family	---		698014	MmugDNA.15904.1.S1_at_RAB9B
MmugDNA.15905.1.S1_at	239636_at	MCF2L	MCF.2 cell line derived transforming sequence-like	chr13q34		23263	---	---	---	---		MmugDNA.15905.1.S1_at_MCF2L
MmugDNA.15906.1.S1_at	213117_at	KLHL9	kelch-like 9 (Drosophila)	chr9p22		55958	LOC710804	similar to kelch-like 9	---		710804	MmugDNA.15906.1.S1_at_KLHL9
MmugDNA.15907.1.S1_at	232961_at	LOC26010	Viral DNA polymerase-transactivated protein 6	chr2q33.1		26010	---	---	---	---		MmugDNA.15907.1.S1_at_LOC26010
MmugDNA.15908.1.S1_at	1566689_at	---	---	---	---		---	---	---	---		MmugDNA.15908.1.S1_at_---
MmugDNA.15909.1.S1_at	235496_at	HRCT1	histidine rich carboxyl terminus 1	chr9p13.3		646962	---	---	---	---		MmugDNA.15909.1.S1_at_HRCT1
MmugDNA.15910.1.S1_at	215624_at	TSC2	tuberous sclerosis 2	chr16p13.3		7249	---	---	---	---		MmugDNA.15910.1.S1_at_TSC2
MmugDNA.15911.1.S1_at	240336_at	HBM	hemoglobin, mu	chr16p13.3		3042	LOC694058	similar to hemoglobin mu chain	---		694058	MmugDNA.15911.1.S1_at_HBM
MmugDNA.1591.1.S1_at	228694_at	---	---	---	---		---	---	---	---		MmugDNA.1591.1.S1_at_---
MmugDNA.15912.1.S1_at	1562852_at	---	---	---	---		LOC719329	similar to FYVE, RhoGEF and PH domain containing 2	---		719329	MmugDNA.15912.1.S1_at_---
MmugDNA.15913.1.S1_at	226643_s_at	NUDCD2	NudC domain containing 2	chr5q34		134492	NUDCD2	NudC domain containing 2	---		697869	MmugDNA.15913.1.S1_at_NUDCD2
MmugDNA.15914.1.S1_at	1560141_at	LOC100133039	hypothetical protein LOC100133039	chr3p25.2		100133039	---	---	---	---		MmugDNA.15914.1.S1_at_LOC1001330
MmugDNA.15915.1.S1_at	236642_at	---	---	---	---		---	---	---	---		MmugDNA.15915.1.S1_at_---
MmugDNA.15916.1.S1_at	208384_s_at	MID2	midline 2	chrXq22.3		11043	LOC703281	similar to midline 2 isoform 2	---		703281	MmugDNA.15916.1.S1_at_MID2
MmugDNA.15917.1.S1_at	220863_at	MIP	major intrinsic protein of lens fiber	chr12q13		4284	MIP	major intrinsic protein of lens fiber	---		712901	MmugDNA.15917.1.S1_at_MIP
MmugDNA.15918.1.S1_at	203414_at	MMD	monocyte to macrophage differentiation-associated	chr17q		23531	LOC706723	similar to monocyte to macrophage differentiation-associated precursor	---		706723	MmugDNA.15918.1.S1_at_MMD
MmugDNA.15919.1.S1_at	229369_at	VSIG2	V-set and immunoglobulin domain containing 2	chr11q24		23584	LOC712047	similar to V-set and immunoglobulin domain containing 2	---		712047	MmugDNA.15919.1.S1_at_VSIG2
MmugDNA.159.1.S1_at	244364_at	MYO3A	myosin IIIA	chr10p11.1		53904	---	---	---	---		MmugDNA.159.1.S1_at_MYO3A
MmugDNA.15920.1.S1_at	239069_s_at	---	---	---	---		---	---	---	---		MmugDNA.15920.1.S1_at_---
MmugDNA.15921.1.S1_at	NA	NA	NA	NA	NA		NA	NA	NA	NA		MmugDNA.15921.1.S1_at_NA
MmugDNA.1592.1.S1_at	1553363_at	C6orf195	chromosome 6 open reading frame 195	chr6p25.2		154386	---	---	---	---		MmugDNA.1592.1.S1_at_C6orf195
MmugDNA.15922.1.S1_at	233676_at	---	---	---	---		---	---	---	---		MmugDNA.15922.1.S1_at_---
MmugDNA.15923.1.S1_at	229405_at	KIF7	kinesin family member 7	chr15q26.1		374654	KIF7	kinesin family member 7	---		706132	MmugDNA.15923.1.S1_at_KIF7
MmugDNA.15924.1.S1_at	244302_at	---	---	---	---		---	---	---	---		MmugDNA.15924.1.S1_at_---
MmugDNA.15925.1.S1_at	242689_at	RALGPS1	Ral GEF with PH domain and SH3 binding motif 1	chr9q33.3		9649	---	---	---	---		MmugDNA.15925.1.S1_at_RALGPS1
MmugDNA.15926.1.S1_at	240285_at	AQP2	aquaporin 2 (collecting duct)	chr12q12-q13		359	LOC711719	similar to aquaporin 2	---		711719	MmugDNA.15926.1.S1_at_AQP2
MmugDNA.15927.1.S1_at	229402_at	SAMD13	sterile alpha motif domain containing 13	chr1p31.1		148418	LOC713946	similar to dnaj-like protein	---		713946	MmugDNA.15927.1.S1_at_SAMD13
MmugDNA.15928.1.S1_at	240216_at	---	---	---	---		---	---	---	---		MmugDNA.15928.1.S1_at_---
MmugDNA.15929.1.S1_at	206538_at	MRAS	muscle RAS oncogene homolog	chr3q22.3		22808	MRAS	muscle RAS oncogene homolog	---		716352	MmugDNA.15929.1.S1_at_MRAS
MmugDNA.15930.1.S1_at	1564242_at	---	---	---	---		---	---	---	---		MmugDNA.15930.1.S1_at_---
MmugDNA.15931.1.S1_at	1552715_a_at	RXFP1	relaxin/insulin-like family peptide receptor 1	chr4q32.1		59350	RXFP1	relaxin/insulin-like family peptide receptor 1	---		701107	MmugDNA.15931.1.S1_at_RXFP1
MmugDNA.1593.1.S1_at	235870_at	LOC339524	hypothetical LOC339524	chr1p22.3		339524	LOC712613	hypothetical protein LOC712613	---		712613	MmugDNA.1593.1.S1_at_LOC339524
MmugDNA.15932.1.S1_at	207727_s_at	MUTYH	mutY homolog (E. coli)	chr1p34.3-p32.1		4595	MUTYH	mutY homolog	---		705651	MmugDNA.15932.1.S1_at_MUTYH
MmugDNA.15933.1.S1_at	213031_s_at	WDR73	WD repeat domain 73	chr15q25.2		84942	LOC696186	similar to WD repeat domain 73	---		696186	MmugDNA.15933.1.S1_at_WDR73
MmugDNA.15935.1.S1_s_at	NA	NA	NA	NA	NA		NA	NA	NA	NA		MmugDNA.15935.1.S1_s_at_NA
MmugDNA.15936.1.S1_at	226216_at	INSR	insulin receptor	chr19p13.3-p13.2		3643	---	---	---	---		MmugDNA.15936.1.S1_at_INSR
MmugDNA.15936.1.S1_s_at	NA	NA	NA	NA	NA		NA	NA	NA	NA		MmugDNA.15936.1.S1_s_at_NA
MmugDNA.15937.1.S1_at	1555056_at	CCNG2	cyclin G2	chr4q21.1		901	---	---	---	---		MmugDNA.15937.1.S1_at_CCNG2
MmugDNA.15938.1.S1_at	222627_at	VPS54	vacuolar protein sorting 54 homolog (S. cerevisiae)	chr2p13-p14		51542	VPS54	vacuolar protein sorting 54 homolog (S. cerevisiae)	---		695397	MmugDNA.15938.1.S1_at_VPS54
MmugDNA.15939.1.S1_at	226212_s_at	INSR	insulin receptor	chr19p13.3-p13.2		3643	---	---	---	---		MmugDNA.15939.1.S1_at_INSR
MmugDNA.15940.1.S1_at	205553_s_at	CSRP3	cysteine and glycine-rich protein 3 (cardiac LIM protein)	chr11p15.1		8048	CSRP3	cysteine and glycine-rich protein 3 (cardiac LIM protein)	---		702466	MmugDNA.15940.1.S1_at_CSRP3
MmugDNA.15941.1.S1_at	238957_at	PDXDC2	Pyridoxal-dependent decarboxylase domain containing 2	chr16q22.1		283970	---	---	---	---		MmugDNA.15941.1.S1_at_PDXDC2
MmugDNA.1594.1.S1_at	224999_at	---	---	---	---		---	---	---	---		MmugDNA.1594.1.S1_at_---
MmugDNA.15942.1.S1_at	228451_at	TSSK3	Testis-specific serine kinase 3	chr1p35-p34		81629	LOC708770	hypothetical protein LOC708770	---		708770	MmugDNA.15942.1.S1_at_TSSK3
MmugDNA.15942.1.S1_s_at	1556560_a_at	LOC100128071	similar to hCG41624	chr1p35.1		100128071	LOC708770	hypothetical protein LOC708770	---		708770	MmugDNA.15942.1.S1_s_at_LOC10012
MmugDNA.15943.1.S1_at	NA	NA	NA	NA	NA		NA	NA	NA	NA		MmugDNA.15943.1.S1_at_NA
MmugDNA.15943.1.S1_s_at	201640_x_at	CLPTM1	cleft lip and palate associated transmembrane protein 1	chr19q13.2-q13.3		1209	LOC718306	similar to cleft lip and palate associated transmembrane protein 1	---		718306	MmugDNA.15943.1.S1_s_at_CLPTM1
MmugDNA.15943.1.S1_x_at	NA	NA	NA	NA	NA		NA	NA	NA	NA		MmugDNA.15943.1.S1_x_at_NA
MmugDNA.15944.1.S1_at	NA	NA	NA	NA	NA		NA	NA	NA	NA		MmugDNA.15944.1.S1_at_NA
MmugDNA.15945.1.S1_at	1556552_a_at	ACSF3	acyl-CoA synthetase family member 3	chr16q24.3		197322	---	---	---	---		MmugDNA.15945.1.S1_at_ACSF3
MmugDNA.15946.1.S1_at	242059_at	---	---	---	---		---	---	---	---		MmugDNA.15946.1.S1_at_---
MmugDNA.15947.1.S1_at	241136_at	---	---	---	---		LOC699658	Similar to 60S ribosomal protein L7a	---		699658	MmugDNA.15947.1.S1_at_---
MmugDNA.15948.1.S1_at	223926_at	KIF2B	kinesin family member 2B	chr17q22		84643	LOC706977	Similar to calcium/calmodulin-dependent protein kinase IIB isoform 1	---		706977	MmugDNA.15948.1.S1_at_KIF2B
MmugDNA.15949.1.S1_at	220781_at	DEC1	deleted in esophageal cancer 1	chr9q32		50514	---	---	---	---		MmugDNA.15949.1.S1_at_DEC1
MmugDNA.15950.1.S1_at	212095_s_at	MTUS1	mitochondrial tumor suppressor 1	chr8p22		57509	LOC702990	similar to mitochondrial tumor suppressor 1 isoform 1	---		702990	MmugDNA.15950.1.S1_at_MTUS1
MmugDNA.15951.1.S1_at	210968_s_at	RTN4	reticulon 4	chr2p16.3		57142	BTBD3	BTB (POZ) domain containing 3	---		693983	MmugDNA.15951.1.S1_at_RTN4
MmugDNA.1595.1.S1_at	1570490_at	---	---	---	---		---	---	---	---		MmugDNA.1595.1.S1_at_---
MmugDNA.15952.1.S1_at	212093_s_at	MTUS1	mitochondrial tumor suppressor 1	chr8p22		57509	LOC702990	similar to mitochondrial tumor suppressor 1 isoform 1	---		702990	MmugDNA.15952.1.S1_at_MTUS1
MmugDNA.15953.1.S1_at	215295_at	DTNB	dystrobrevin, beta	chr2p24		1838	---	---	---	---		MmugDNA.15953.1.S1_at_DTNB
MmugDNA.15954.1.S1_at	212096_s_at	MTUS1	mitochondrial tumor suppressor 1	chr8p22		57509	LOC702990	similar to mitochondrial tumor suppressor 1 isoform 1	---		702990	MmugDNA.15954.1.S1_at_MTUS1
MmugDNA.15955.1.S1_at	204986_s_at	TAOK2	TAO kinase 2	chr16p11.2		9344	TAOK2	TAO kinase 2	---		707777	MmugDNA.15955.1.S1_at_TAOK2
MmugDNA.15956.1.S1_at	240951_at	RORA	RAR-related orphan receptor A	chr15q21.3		6095	---	---	---	---		MmugDNA.15956.1.S1_at_RORA
MmugDNA.15957.1.S1_at	205066_s_at	ENPP1	ectonucleotide pyrophosphatase/phosphodiesterase 1	chr6q22-q23		5167	ENPP1	ectonucleotide pyrophosphatase/phosphodiesterase 1	---		710140	MmugDNA.15957.1.S1_at_ENPP1
MmugDNA.15958.1.S1_at	1557276_at	---	---	---	---		---	---	---	---		MmugDNA.15958.1.S1_at_---
MmugDNA.15959.1.S1_at	205429_s_at	MPP6	membrane protein, palmitoylated 6 (MAGUK p55 subfamily member 6)	chr7p15		51678	MPP6	membrane protein, palmitoylated 6 (MAGUK p55 subfamily member 6)	---		703560	MmugDNA.15959.1.S1_at_MPP6
MmugDNA.15960.1.S1_at	209533_s_at	PLAA	phospholipase A2-activating protein	chr9p21		9373	MAN2B2	Mannosidase, alpha, class 2B, member 2	---		710870	MmugDNA.15960.1.S1_at_PLAA

MmugDNA.15961.1.S1_at	219862_s_at	NARF	nuclear prelamin A recognition factor	chr17q25.3	26502	NARF	nuclear prelamin A recognition factor	---	716259	MmugDNA.15961.1.S1_at_NARF
MmugDNA.1596.1.S1_at	204992_s_at	PFN2	profilin 2	chr3q25.1-q25.2	5217	LOC713460	similar to Profilin-2 (Profilin II)	---	713460	MmugDNA.1596.1.S1_at_PFN2
MmugDNA.15962.1.S1_at	220308_at	CCDC19	coiled-coil domain containing 19	chr1q22	25790	ETNK2	Ethanolamine kinase 2	---	700683	MmugDNA.15962.1.S1_at_CCDC19
MmugDNA.15963.1.S1_at	239597_at	---	---	---	---	---	---	---	---	MmugDNA.15963.1.S1_at_---
MmugDNA.15964.1.S1_at	243945_at	TRIM2	tripartite motif-containing 2	chr4q31.3	23321	TRIM2	tripartite motif-containing 2	---	696517	MmugDNA.15964.1.S1_at_TRIM2
MmugDNA.15966.1.S1_at	225778_at	RBMS2	RNA binding motif, single stranded interacting protein 2	chr12q13.3	5939	---	---	---	---	MmugDNA.15966.1.S1_at_RBMS2
MmugDNA.15967.1.S1_at	1560538_at	---	---	---	---	---	---	---	---	MmugDNA.15967.1.S1_at_---
MmugDNA.15968.1.S1_at	225776_at	RBMS2	RNA binding motif, single stranded interacting protein 2	chr12q13.3	5939	---	---	---	---	MmugDNA.15968.1.S1_at_RBMS2
MmugDNA.15970.1.S1_at	1563853_at	LOC283045	hypothetical protein LOC283045	chr10q21.3	283045	---	---	---	---	MmugDNA.15970.1.S1_at_LOC283045
MmugDNA.15971.1.S1_at	1554623_x_at	DGKE	diacylglycerol kinase, epsilon 64kDa	chr17q22	8526	---	---	---	---	MmugDNA.15971.1.S1_at_DGKE
MmugDNA.1597.1.S1_at	1567253_at	OR10D3P	olfactory receptor, family 10, subfamily D, member 3 pseudogene	chr11q24.2	26497	LOC715199	similar to olfactory receptor 958	---	715199	MmugDNA.1597.1.S1_at_OR10D3P
MmugDNA.15972.1.S1_at	233145_at	CSMD2	CUB and Sushi multiple domains 2	chr1p35.1-p34.3	114784	---	---	---	---	MmugDNA.15972.1.S1_at_CSMD2
MmugDNA.15973.1.S1_s_at	211328_x_at	HFE	hemochromatosis	chr6p21.3	3077	HFE	hemochromatosis	---	696129	MmugDNA.15973.1.S1_s_at_HFE
MmugDNA.15975.1.S1_at	232468_at	---	---	---	---	---	---	---	---	MmugDNA.15975.1.S1_at_---
MmugDNA.15976.1.S1_at	219312_s_at	ZBTB10	zinc finger and BTB domain containing 10	chr8q13-q21.1	65986	---	---	---	---	MmugDNA.15976.1.S1_at_ZBTB10
MmugDNA.15977.1.S1_at	230212_at	LOC729345	hypothetical LOC729345	chr4q28.1	729345	---	---	---	---	MmugDNA.15977.1.S1_at_LOC729345
MmugDNA.15978.1.S1_s_at	220790_s_at	MS4A5	membrane-spanning 4-domains, subfamily A, member 5	chr11q12	64232	MS4A5	membrane-spanning 4-domains, subfamily A, member 5	---	696967	MmugDNA.15978.1.S1_s_at_MS4A5
MmugDNA.15979.1.S1_at	236380_at	---	---	---	---	---	---	---	---	MmugDNA.15979.1.S1_at_---
MmugDNA.15980.1.S1_at	236142_at	PPIH	Peptidylprolyl isomerase H (cyclophilin H)	chr1p34.1	10465	---	---	---	---	MmugDNA.15980.1.S1_at_PPIH
MmugDNA.15981.1.S1_at	225094_at	SETD8	SET domain containing (lysine methyltransferase) 8	chr12q24.31	387893	SETD8	SET domain containing (lysine methyltransferase) 8	---	709345	MmugDNA.15981.1.S1_at_SETD8
MmugDNA.1598.1.S1_at	1563639_a_at	FHAD1	forkhead-associated (FHA) phosphopeptide binding domain 1	chr1p36.21	114827	LOC699900	similar to forkhead-associated (FHA) phosphopeptide binding domain 1	---	699900	MmugDNA.1598.1.S1_at_FHAD1
MmugDNA.15982.1.S1_at	241658_at	---	---	---	---	---	---	---	---	MmugDNA.15982.1.S1_at_---
MmugDNA.15983.1.S1_at	202751_at	TFIP11	tuftelin interacting protein 11	chr22q12.1	24144	---	---	---	---	MmugDNA.15983.1.S1_at_TFIP11
MmugDNA.15984.1.S1_at	225118_at	SETD8	SET domain containing (lysine methyltransferase) 8	chr12q24.31	387893	SETD8	SET domain containing (lysine methyltransferase) 8	---	709345	MmugDNA.15984.1.S1_at_SETD8
MmugDNA.15985.1.S1_s_at	201245_s_at	OTUB1	OTU domain, ubiquitin aldehyde binding 1	chr11q13.1	55611	OTUB1	OTU domain, ubiquitin aldehyde binding 1	---	717989	MmugDNA.15985.1.S1_s_at_OTUB1
MmugDNA.15988.1.S1_s_at	224342_x_at	IGL@ /// LOC96610	immunoglobulin lambda locus /// BMS1 homolog, ribosome assembly protein (yeast) pseudogene	chr22q11.1-q11.2 /// chr22q11.2	3535 /// 96610	LOC704043 /// LOC7043	similar to Ig lambda chain V-I region BL2 precursor /// similar to Ig lambda chain V-I region BL2 precu	---	704043 /// 704375 /// 7046	MmugDNA.15988.1.S1_s_at_IGL@ /// L
MmugDNA.15989.1.S1_s_at	NA	NA	NA	NA	NA	NA	NA	NA	NA	MmugDNA.15989.1.S1_s_at_NA
MmugDNA.15990.1.S1_s_at	211634_x_at	IGHM /// LOC10013386	immunoglobulin heavy constant mu /// similar to hCG1773549	chr14q32.33	100133862 /// 3507	LOC721043	similar to Ig heavy chain V-I region HG3 precursor	---	721043	MmugDNA.15990.1.S1_s_at_IGHM /// L
MmugDNA.15991.1.S1_at	236280_at	---	---	---	---	---	---	---	---	MmugDNA.15991.1.S1_at_---
MmugDNA.1599.1.S1_at	200886_s_at	PGAM1	phosphoglycerate mutase 1 (brain)	chr10q25.3	5223	PGAM1	phosphoglycerate mutase 1 (brain)	---	696959	MmugDNA.1599.1.S1_at_PGAM1
MmugDNA.15993.1.S1_at	218238_at	GTPBP4	GTP binding protein 4	chr10p15-p14	23560	GTPBP4	G protein-binding protein CRFG	---	709873	MmugDNA.15993.1.S1_at_GTPBP4
MmugDNA.15994.1.S1_at	218090_s_at	BRWD2	bromodomain and WD repeat domain containing 2	chr10q26	55717	F13B	Coagulation factor XIII, B polypeptide	---	711237	MmugDNA.15994.1.S1_at_BRWD2
MmugDNA.15996.1.S1_s_at	NA	NA	NA	NA	NA	NA	NA	NA	NA	MmugDNA.15996.1.S1_s_at_NA
MmugDNA.15997.1.S1_s_at	NA	NA	NA	NA	NA	NA	NA	NA	NA	MmugDNA.15997.1.S1_s_at_NA
MmugDNA.15998.1.S1_at	236479_at	---	---	---	---	---	---	---	---	MmugDNA.15998.1.S1_at_---
MmugDNA.15999.1.S1_at	202784_s_at	NNT	nicotinamide nucleotide transhydrogenase	chr5p13.1-cen	23530	NNT	nicotinamide nucleotide transhydrogenase	---	701110	MmugDNA.15999.1.S1_at_NNT
MmugDNA.16000.1.S1_at	236166_at	LOC285147	hypothetical protein LOC285147	chr2p25.2	285147	---	---	---	---	MmugDNA.16000.1.S1_at_LOC285147
MmugDNA.16001.1.S1_s_at	212187_x_at	PTGDS	prostaglandin D2 synthase 21kDa (brain)	chr9q34.2-q34.3	5730	LOC708788	Similar to 60S ribosomal protein L7a	---	708788	MmugDNA.16001.1.S1_s_at_PTGDS
MmugDNA.1600.1.S1_at	242358_at	---	---	---	---	---	---	---	---	MmugDNA.1600.1.S1_at_---
MmugDNA.16002.1.S1_at	218227_at	NUBP2	nucleotide binding protein 2 (MinD homolog, E. coli)	chr16p13.3	10101	NUBP2	nucleotide binding protein 2 (MinD homolog, E. coli)	---	702962	MmugDNA.16002.1.S1_at_NUBP2
MmugDNA.16003.1.S1_at	204800_s_at	DHRS12	dehydrogenase/reductase (SDR family) member 12	chr13q14.3	79758	LOC713457	hypothetical protein LOC713457	---	713457	MmugDNA.16003.1.S1_at_DHRS12
MmugDNA.16004.1.S1_at	239969_at	---	---	---	---	---	---	---	---	MmugDNA.16004.1.S1_at_---
MmugDNA.16005.1.S1_at	1562000_at	LOC400620	Hypothetical LOC400620	chr17q25.1	400620	---	---	---	---	MmugDNA.16005.1.S1_at_LOC400620
MmugDNA.16006.1.S1_at	211855_s_at	SLC25A14	solute carrier family 25 (mitochondrial carrier, brain), member 14	chrXq24	9016	SLC25A14	solute carrier family 25 (mitochondrial carrier, brain), member 14	---	703255	MmugDNA.16006.1.S1_at_SLC25A14
MmugDNA.16007.1.S1_at	230680_at	---	---	---	---	---	---	---	---	MmugDNA.16007.1.S1_at_---
MmugDNA.16008.1.S1_at	243404_at	---	---	---	---	---	---	---	---	MmugDNA.16008.1.S1_at_---
MmugDNA.16009.1.S1_at	1557386_at	---	---	---	---	---	---	---	---	MmugDNA.16009.1.S1_at_---
MmugDNA.16010.1.S1_at	207116_s_at	GAPDHS	glyceraldehyde-3-phosphate dehydrogenase, spermatogenic	chr19q13.12	26330	LOC707919	Similar to nicotinamide nucleotide adenylyltransferase 1	---	707919	MmugDNA.16010.1.S1_at_GAPDHS
MmugDNA.16011.1.S1_at	228391_at	CYP4V2	cytochrome P450, family 4, subfamily V, polypeptide 2	chr4q35.2	285440	LOC696520	similar to cytochrome P450, family 4, subfamily v, polypeptide 2	---	696520	MmugDNA.16011.1.S1_at_CYP4V2
MmugDNA.1601.1.S1_at	205736_at	PGAM2	phosphoglycerate mutase 2 (muscle)	chr7p13-p12	5224	PGAM2	phosphoglycerate mutase 2 (muscle)	---	720615	MmugDNA.1601.1.S1_at_PGAM2
MmugDNA.16012.1.S1_at	205164_at	GCAT	glycine C-acetyltransferase (2-amino-3-ketobutyrate coenzyme A ligase)	chr22q13.1	23464	LOC693561	Similar to phosphatidylinositol 4-kinase type-II beta	---	693561	MmugDNA.16012.1.S1_at_GCAT
MmugDNA.16013.1.S1_at	219537_x_at	DLL3	delta-like 3 (Drosophila)	chr19q13	10683	DLL3	delta-like 3 (Drosophila)	---	697453	MmugDNA.16013.1.S1_at_DLL3
MmugDNA.16014.1.S1_at	240665_at	---	---	---	---	---	---	---	---	MmugDNA.16014.1.S1_at_---
MmugDNA.16015.1.S1_at	1552672_a_at	IGSF3	immunoglobulin superfamily, member 3	chr1p13	3321	LOC713579	similar to immunoglobulin superfamily, member 3 isoform 1	---	713579	MmugDNA.16015.1.S1_at_IGSF3
MmugDNA.16016.1.S1_at	226644_at	MIB2	mindbomb homolog 2 (Drosophila)	chr1p36.33	142678	---	---	---	---	MmugDNA.16016.1.S1_at_MIB2
MmugDNA.16017.1.S1_at	243981_at	STK4	serine/threonine kinase 4	chr20q11.2-q13.2	6789	STK4	serine/threonine kinase 4	---	717730	MmugDNA.16017.1.S1_at_STK4
MmugDNA.16018.1.S1_at	NA	NA	NA	NA	NA	NA	NA	NA	NA	MmugDNA.16018.1.S1_at_NA
MmugDNA.16019.1.S1_at	1566499_at	---	---	---	---	---	---	---	---	MmugDNA.16019.1.S1_at_---
MmugDNA.160.1.S1_at	236956_at	---	---	---	---	---	---	---	---	MmugDNA.160.1.S1_at_---
MmugDNA.16021.1.S1_at	231642_at	C6orf201	chromosome 6 open reading frame 201	chr6p25.2	404220	LOC697442	Similar to SECIS binding protein 2	---	697442	MmugDNA.16021.1.S1_at_C6orf201
MmugDNA.1602.1.S1_at	240854_x_at	---	---	---	---	---	---	---	---	MmugDNA.1602.1.S1_at_---
MmugDNA.16022.1.S1_at	228844_at	SLC13A5	solute carrier family 13 (sodium-dependent citrate transporter), member 5	chr17p13.2	284111	LOC713292	similar to solute carrier family 13 (sodium-dependent citrate transporter), member 5	---	713292	MmugDNA.16022.1.S1_at_SLC13A5
MmugDNA.16023.1.S1_at	225680_at	LRWD1	leucine-rich repeats and WD repeat domain containing 1	chr7q22.1	222229	LOC715187	similar to C56E6.6	---	715187	MmugDNA.16023.1.S1_at_LRWD1
MmugDNA.16024.1.S1_at	204559_s_at	LSM7	LSM7 homolog, U6 small nuclear RNA associated (S. cerevisiae)	chr19p13.3	51690	LOC717609	similar to U6 snRNA-associated Sm-like protein LSm7	---	717609	MmugDNA.16024.1.S1_at_LSM7
MmugDNA.16025.1.S1_at	205136_s_at	NUFIP1	nuclear fragile X mental retardation protein interacting protein 1	chr13q14	26747	---	---	---	---	MmugDNA.16025.1.S1_at_NUFIP1
MmugDNA.16026.1.S1_at	231576_at	---	---	---	---	---	---	---	---	MmugDNA.16026.1.S1_at_---
MmugDNA.16027.1.S1_at	207740_s_at	NUP62	nucleoporin 62kDa	chr19q13.33	23636	AP3B1	Adaptor-related protein complex 3, beta 1 subunit	---	708754	MmugDNA.16027.1.S1_at_NUP62
MmugDNA.16028.1.S1_at	219119_at	LSM8	LSM8 homolog, U6 small nuclear RNA associated (S. cerevisiae)	chr7q31.1-q31.3	51691	LOC702661	Similar to RP11-506B15.1 protein isoform 1	---	702661	MmugDNA.16028.1.S1_at_LSM8
MmugDNA.16029.1.S1_at	1561526_at	---	---	---	---	---	---	---	---	MmugDNA.16029.1.S1_at_---
MmugDNA.16030.1.S1_at	221382_at	---	---	---	---	LOC707518	similar to olfactory receptor 134	---	707518	MmugDNA.16030.1.S1_at_---
MmugDNA.16031.1.S1_at	218354_at	TRAPPC2L	trafficking protein particle complex 2-like	chr16q24.3	51693	TRAPPC2L	trafficking protein particle complex 2-like	---	697970	MmugDNA.16031.1.S1_at_TRAPPC2L
MmugDNA.1603.1.S1_at	205261_at	PGC	progastricsin (pepsinogen C)	chr6p21.3-p21.1	5225	PGC	similar to Gastricsin precursor (Pepsinogen C)	---	695713	MmugDNA.1603.1.S1_at_PGC
MmugDNA.16032.1.S1_at	1564672_at	---	---	---	---	---	---	---	---	MmugDNA.16032.1.S1_at_---
MmugDNA.16033.1.S1_at	1568618_a_at	GALNT1	UDP-N-acetyl-alpha-D-galactosamine:polypeptide N-acetylgalactosaminyltransferase 1 (GalNAc-T1)	chr18q12.1	2589	LOC718845	Similar to differentially expressed in FDCP 6 homolog	---	718845	MmugDNA.16033.1.S1_at_GALNT1
MmugDNA.16034.1.S1_at	1562122_at	---	---	---	---	---	---	---	---	MmugDNA.16034.1.S1_at_---
MmugDNA.16035.1.S1_at	240227_at	MIER1	mesoderm induction early response 1 homolog (Xenopus laevis)	chr1p31.3	57708	---	---	---	---	MmugDNA.16035.1.S1_at_MIER1
MmugDNA.16036.1.S1_at	1563082_at	LOC285045	hypothetical LOC285045	chr2p22.3	285045	---	---	---	---	MmugDNA.16036.1.S1_at_LOC285045
MmugDNA.16037.1.S1_at	234034_at	---	---	---	---	---	---	---	---	MmugDNA.16037.1.S1_at_---
MmugDNA.16038.1.S1_at	1564389_at	---	---	---	---	---	---	---	---	MmugDNA.16038.1.S1_at_---
MmugDNA.16039.1.S1_at	237308_at	---	---	---	---	---	---	---	---	MmugDNA.16039.1.S1_at_---
MmugDNA.16040.1.S1_at	208296_x_at	TNFAIP8	tumor necrosis factor, alpha-induced protein 8	chr5q23.1	25816	LOC700778	similar to tumor necrosis factor, alpha-induced protein 8	---	700778	MmugDNA.16040.1.S1_at_TNFAIP8
MmugDNA.16041.1.S1_at	220780_at	PLA2G3	phospholipase A2, group III	chr22q11.2-q13.2	50487	---	---	---	---	MmugDNA.16041.1.S1_at_PLA2G3
MmugDNA.1604.1.S1_at	NA	NA	NA	NA	NA	NA	NA	NA	NA	MmugDNA.1604.1.S1_at_NA
MmugDNA.16042.1.S1_at	1561542_at	---	---	---	---	---	---	---	---	MmugDNA.16042.1.S1_at_---
MmugDNA.16043.1.S1_at	1556411_s_at	KY	kyphoscoliosis peptidase	chr3q22.2	339855	LOC717233	similar to kyphoscoliosis peptidase	---	717233	MmugDNA.16043.1.S1_at_KY
MmugDNA.16044.1.S1_at	214480_at	ETV3	ets variant 3	chr1q21-q23	2117	ETV3	similar to ETS translocation variant 3 (ETS-domain transcriptional repressor PE1) (PE-1) (Mitogenic E	---	719741	MmugDNA.16044.1.S1_at_ETV3
MmugDNA.16045.1.S1_at	217807_s_at	GLTSCR2	glioma tumor suppressor candidate region gene 2	chr19q13.3	29997	GLTSCR2	glioma tumor suppressor candidate region gene 2	---	717454	MmugDNA.16045.1.S1_at_GLTSCR2
MmugDNA.16046.1.S1_at	216388_s_at	LTB4R	leukotriene B4 receptor	chr14q11.2-q12	1241	LTB4R	leukotriene B4 receptor	---	716055	MmugDNA.16046.1.S1_at_LTB4R
MmugDNA.16047.1.S1_at	235240_at	ATXN3	ataxin 3	chr14q24.3-q32.2	4287	---	---	---	---	MmugDNA.16047.1.S1_at_ATXN3
MmugDNA.16048.1.S1_at	226247_at	PLEKHA1	pleckstrin homology domain containing, family A (phosphoinositide binding specific) member 1	chr10q26.13	59338	PLEKHA1	pleckstrin homology domain containing, family A (phosphoinositide binding specific) member 1	---	705731	MmugDNA.16048.1.S1_at_PLEKHA1
MmugDNA.16049.1.S1_at	227572_at	USP30	ubiquitin specific peptidase 30	chr12q24.11	84749	USP30	ubiquitin specific peptidase 30	---	706607	MmugDNA.16049.1.S1_at_USP30
MmugDNA.16050.1.S1_at	203732_at	TRIP4	thyroid hormone receptor interactor 4	chr15q22.31	9325	---	---	---	---	MmugDNA.16050.1.S1_at_TRIP4
MmugDNA.16051.1.S1_at	221445_at	OR1A2	olfactory receptor, family 1, subfamily A, member 2	chr17p13.3	26189	---	---	---	---	MmugDNA.16051.1.S1_at_OR1A2
MmugDNA.1605.1.S1_at	238180_at	---	---	---	---	---	---	---	---	MmugDNA.1605.1.S1_at_---
MmugDNA.16052.1.S1_at	228418_at	EXOC5	exocyst complex component 5	chr14q23.1	10640	EXOC5	exocyst complex component 5	---	699001	MmugDNA.16052.1.S1_at_EXOC5
MmugDNA.16053.1.S1_at	218603_at	HECA	headcase homolog (Drosophila)	chr6q23-q24	51696	HECA	similar to Headcase protein homolog (hHDC)	---	702628	MmugDNA.16053.1.S1_at_HECA
MmugDNA.16054.1.S1_at	226784_at	TWISTNB	TWIST neighbor	chr7p15.3	221830	LOC702153	Similar to keratin 10	---	702153	MmugDNA.16054.1.S1_at_TWISTNB
MmugDNA.16055.1.S1_at	233594_at	---	---	---	---	---	---	---	---	MmugDNA.16055.1.S1_at_---
MmugDNA.16056.1.S1_at	243124_at	LOC653390	RRN3 RNA polymerase I transcription factor homolog (S. cerevisiae) pseudogene	chr16p11.2	653390	---	---	---	---	MmugDNA.16056.1.S1_at_LOC653390
MmugDNA.16057.1.S1_at	221460_at	OR2C1	olfactory receptor, family 2, subfamily C, member 1	chr16p13.3	4993	---	---	---	---	MmugDNA.16057.1.S1_at_OR2C1
MmugDNA.16058.1.S1_at	218636_s_at	MAN1B1	mannosidase, alpha, class 1B, member 1	chr9q34	11253	MAN1B1	mannosidase, alpha, class 1B, member 1	---	706918	MmugDNA.16058.1.S1_at_MAN1B1
MmugDNA.16059.1.S1_at	208526_at	OR2F1	olfactory receptor, family 2, subfamily F, member 1	chr7q35	26211	LOC707900	similar to Olfactory receptor 2F1 (Olfactory receptor-like protein OLF3)	---	707900	MmugDNA.16059.1.S1_at_OR2F1
MmugDNA.16060.1.S1_at	221329_at	OR52A1	olfactory receptor, family 52, subfamily A, member 1	chr11p15.5	23538	LOC718205	Hypothetical protein LOC718205	---	718205	MmugDNA.16060.1.S1_at_OR52A1
MmugDNA.16061.1.S1_at	240245_at	---	---	---	---	---	---	---	---	MmugDNA.16061.1.S1_at_---
MmugDNA.1606.1.S1_at	231961_at	RBPMS	RNA binding protein with multiple splicing	chr8p12-p11	11030	---	---	---	---	MmugDNA.1606.1.S1_at_RBPMS
MmugDNA.16062.1.S1_at	201956_s_at	GNPAT	glyceronephosphate O-acyltransferase	chr1q42	8443	LOC702417	Similar to High mobility group protein B1 (High mobility group protein 1) (HMG-1) (Amphoterin) (Hepa	---	702417	MmugDNA.16062.1.S1_at_GNPAT
MmugDNA.16063.1.S1_at	215440_s_at	BEX4	brain expressed, X-linked 4	chrXq22.1-q22.3	56271	LOC695153	hypothetical protein LOC695153	---	695153	MmugDNA.16063.1.S1_at_BEX4
MmugDNA.16064.1.S1_at	1564472_at	LOC100132080	hypothetical LOC100132080	chr7p22.3	100132080	---	---	---	---	MmugDNA.16064.1.S1_at_LOC100132

MmugDNA.16065.1.S1_at	1553211_at	ANKFN1	ankyrin-repeat and fibronectin type III domain containing 1	chr17q22		162282	LOC707235	similar to CG6954-PA	---		707235	MmugDNA.16065.1.S1_at_ANKFN1
MmugDNA.16066.1.S1_at	221393_at	TAAR3	trace amine associated receptor 3 (gene/pseudogene)	chr6q23-q24		9288	LOC709130	similar to trace amine-associated receptor 3	---		709130	MmugDNA.16066.1.S1_at_TAAR3
MmugDNA.16067.1.S1_at	225569_at	EIF2C2	Eukaryotic translation initiation factor 2C, 2	chr8q24		27161	---	---	---	---		MmugDNA.16067.1.S1_at_EIF2C2
MmugDNA.16068.1.S1_at	239371_at	---	---	---	---		---	---	---	---		MmugDNA.16068.1.S1_at_---
MmugDNA.16069.1.S1_at	231807_at	KIAA1217	KIAA1217	chr10p12.2-p12.1		56243	LOC705851	similar to p130Cas-associated protein (p140Cap) (SNAP-25-interacting protein) (SNIP)	---		705851	MmugDNA.16069.1.S1_at_KIAA1217
MmugDNA.16070.1.S1_at	235847_at	---	---	---	---		---	---	---	---		MmugDNA.16070.1.S1_at_---
MmugDNA.16071.1.S1_at	212016_s_at	PTBP1	polypyrimidine tract binding protein 1	chr19p13.3		5725	PTBP1	polypyrimidine tract binding protein 1	---		703430	MmugDNA.16071.1.S1_at_PTBP1
MmugDNA.1607.1.S1_at	225167_at	FRMD4A	FERM domain containing 4A	chr10p13		55691	---	---	---	---		MmugDNA.1607.1.S1_at_FRMD4A
MmugDNA.16072.1.S1_at	1554063_at	C8orf76	chromosome 8 open reading frame 76	chr8q24.13		84933	LOC704150	hypothetical protein LOC704150	---		704150	MmugDNA.16072.1.S1_at_C8orf76
MmugDNA.16074.1.S1_at	NA	NA	NA	NA	NA		NA	NA	NA	NA		MmugDNA.16074.1.S1_at_NA
MmugDNA.16075.1.S1_at	215289_at	ZNF749	zinc finger protein 749	chr19q13.43		388567	LOC710445	similar to zinc finger protein 749	---		710445	MmugDNA.16075.1.S1_at_ZNF749
MmugDNA.16076.1.S1_at	1553467_at	FLJ32742	hypothetical locus FLJ32742	chrXp21.3		439944	---	---	---	---		MmugDNA.16076.1.S1_at_FLJ32742
MmugDNA.16077.1.S1_at	235267_at	---	---	---	---		---	---	---	---		MmugDNA.16077.1.S1_at_---
MmugDNA.16078.1.S1_at	225724_at	FLJ31306	hypothetical protein FLJ31306	chr14q23.1		379025	---	---	---	---		MmugDNA.16078.1.S1_at_FLJ31306
MmugDNA.16079.1.S1_at	204479_at	OSTF1	osteoclast stimulating factor 1	chr9q13-q21.2		26578	OSTF1	osteoclast stimulating factor 1	---		703986	MmugDNA.16079.1.S1_at_OSTF1
MmugDNA.16080.1.S1_at	233986_s_at	PLEKHG2	pleckstrin homology domain containing, family G (with RhoGef domain) member 2	chr19q13.2		64857	PLEKHG2	pleckstrin homology domain containing, family G (with RhoGef domain) member 2	---		698408	MmugDNA.16080.1.S1_at_PLEKHG2
MmugDNA.16081.1.S1_at	224557_x_at	P2RX2	purinergic receptor P2X, ligand-gated ion channel, 2	chr12q24.33		22953	P2RX2	purinergic receptor P2X, ligand-gated ion channel, 2	---		693390	MmugDNA.16081.1.S1_at_P2RX2
MmugDNA.1608.1.S1_at	214436_at	FBXL2	F-box and leucine-rich repeat protein 2	chr3p22.3		25827	LOC709902	similar to F-box and leucine-rich repeat protein 2	---		709902	MmugDNA.1608.1.S1_at_FBXL2
MmugDNA.16082.1.S1_at	NA	NA	NA	NA	NA		NA	NA	NA	NA		MmugDNA.16082.1.S1_at_NA
MmugDNA.16083.1.S1_at	238191_at	---	---	---	---		---	---	---	---		MmugDNA.16083.1.S1_at_---
MmugDNA.16084.1.S1_at	243062_at	FLCN	folliculin	chr17p11.2		201163	FLCN	folliculin	---		698082	MmugDNA.16084.1.S1_at_FLCN
MmugDNA.16085.1.S1_at	1554306_at	ITPKB	inositol 1,4,5-trisphosphate 3-kinase B	chr1q42.13		3707	---	---	---	---		MmugDNA.16085.1.S1_at_ITPKB
MmugDNA.16086.1.S1_at	221394_at	TAAR2	trace amine associated receptor 2	chr6q24		9287	LOC714074	hypothetical protein LOC714074	---		714074	MmugDNA.16086.1.S1_at_TAAR2
MmugDNA.16087.1.S1_at	239907_at	---	---	---	---		---	---	---	---		MmugDNA.16087.1.S1_at_---
MmugDNA.16088.1.S1_at	237699_at	---	---	---	---		---	---	---	---		MmugDNA.16088.1.S1_at_---
MmugDNA.16089.1.S1_at	208552_at	GRIK4	glutamate receptor, ionotropic, kainate 4	chr11q22.3		2900	---	---	---	---		MmugDNA.16089.1.S1_at_GRIK4
MmugDNA.16090.1.S1_at	NA	NA	NA	NA	NA		NA	NA	NA	NA		MmugDNA.16090.1.S1_at_NA
MmugDNA.16091.1.S1_at	225881_at	SLC35B4	solute carrier family 35, member B4	chr7q33		84912	CLIC5	Chloride intracellular channel 5	---		703565	MmugDNA.16091.1.S1_at_SLC35B4
MmugDNA.1609.1.S1_at	201118_at	PGD	phosphogluconate dehydrogenase	chr1p36.3-p36.13		5226	LOC722674	similar to axonal transport of synaptic vesicles	---		722674	MmugDNA.1609.1.S1_at_PGD
MmugDNA.16092.1.S1_at	225882_at	SLC35B4	solute carrier family 35, member B4	chr7q33		84912	CLIC5	Chloride intracellular channel 5	---		703565	MmugDNA.16092.1.S1_at_SLC35B4
MmugDNA.16093.1.S1_at	216908_x_at	LOC730092	RRN3 RNA polymerase I transcription factor homolog (S. cerevisiae) pseudogene	chr16p12.1		730092	LOC713500	similar to Tif-IA CG3278-PA	---		713500	MmugDNA.16093.1.S1_at_LOC730092
MmugDNA.16094.1.S1_at	238034_at	CANX	calnexin	chr5q35		821	---	---	---	---		MmugDNA.16094.1.S1_at_CANX
MmugDNA.16095.1.S1_at	202250_s_at	WDR42A	WD repeat domain 42A	chr1q22-q23		50717	LOC719645	similar to H326	---		719645	MmugDNA.16095.1.S1_at_WDR42A
MmugDNA.16096.1.S1_at	232768_at	CCNB2	Cyclin B2	chr15q22.2		9133	---	---	---	---		MmugDNA.16096.1.S1_at_CCNB2
MmugDNA.16097.1.S1_at	223319_at	GPHN	gephyrin	chr14q23.3		10243	GPHN	gephyrin	---		709677	MmugDNA.16097.1.S1_at_GPHN
MmugDNA.16098.1.S1_at	1555356_a_at	SCML4	sex comb on midleg-like 4 (Drosophila)	chr6q21		256380	---	---	---	---		MmugDNA.16098.1.S1_at_SCML4
MmugDNA.16099.1.S1_at	241331_at	SKAP2	Src kinase associated phosphoprotein 2	chr7p21-p15		8935	---	---	---	---		MmugDNA.16099.1.S1_at_SKAP2
MmugDNA.16100.1.S1_at	209400_at	SLC12A4	Solute carrier family 12 (potassium/chloride transporters), member 4	chr16q22.1		6560	---	---	---	---		MmugDNA.16100.1.S1_at_SLC12A4
MmugDNA.16101.1.S1_at	206580_s_at	EFEMP2	EGF-containing fibulin-like extracellular matrix protein 2	chr11q13.1		30008	EFEMP2	EGF-containing fibulin-like extracellular matrix protein 2	---		721877	MmugDNA.16101.1.S1_at_EFEMP2
MmugDNA.1610.1.S1_at	225168_at	FRMD4A	FERM domain containing 4A	chr10p13		55691	---	---	---	---		MmugDNA.1610.1.S1_at_FRMD4A
MmugDNA.16102.1.S1_at	234493_at	LOC116437	hypothetical LOC116437	chr12q24.33		116437	---	---	---	---		MmugDNA.16102.1.S1_at_LOC116437
MmugDNA.16103.1.S1_at	211379_x_at	B3GALNT1	beta-1,3-N-acetylgalactosaminyltransferase 1 (globoside blood group)	chr3q25		8706	---	---	---	---		MmugDNA.16103.1.S1_at_B3GALNT1
MmugDNA.16104.1.S1_at	228067_at	C2orf55	chromosome 2 open reading frame 55	chr2q11.2		343990	---	---	---	---		MmugDNA.16104.1.S1_at_C2orf55
MmugDNA.16105.1.S1_at	203632_s_at	GPRC5B	G protein-coupled receptor, family C, group 5, member B	chr16p12		51704	GPRC5B	G protein-coupled receptor, family C, group 5, member B	---		695204	MmugDNA.16105.1.S1_at_GPRC5B
MmugDNA.16106.1.S1_at	223374_s_at	B3GALNT1	beta-1,3-N-acetylgalactosaminyltransferase 1 (globoside blood group)	chr3q25		8706	LOC704055	similar to UDP-Gal:betaGlcNAc beta 1,3-galactosyltransferase 1	---		704055	MmugDNA.16106.1.S1_at_B3GALNT1
MmugDNA.16107.1.S1_at	219436_s_at	EMCN	endomucin	chr4q23		51705	LOC709580	similar to endomucin	---		709580	MmugDNA.16107.1.S1_at_EMCN
MmugDNA.16108.1.S1_at	1560100_at	DLX1	distal-less homeobox 1	chr2q32		1745	DLX1	distal-less homeobox 1	---		695620	MmugDNA.16108.1.S1_at_DLX1
MmugDNA.16110.1.S1_at	210562_at	GREB1	GREB1 protein	chr2p25.1		9687	---	---	---	---		MmugDNA.16110.1.S1_at_GREB1
MmugDNA.16111.1.S1_at	237012_at	---	---	---	---		---	---	---	---		MmugDNA.16111.1.S1_at_---
MmugDNA.1611.1.S1_at	1569995_at	LOC152586	similar to RIKEN cDNA 4933434I20	chr4q31.1		152586	LOC701630	similar to mannosyl (alpha-1,3-)-glycoprotein beta-1,4-N-acetylglucosaminyltransferase, isoenzyme A	---		701630	MmugDNA.1611.1.S1_at_LOC152586
MmugDNA.16112.1.S1_at	210855_at	GREB1	GREB1 protein	chr2p25.1		9687	---	---	---	---		MmugDNA.16112.1.S1_at_GREB1
MmugDNA.16113.1.S1_at	203156_at	AKAP11	A kinase (PRKA) anchor protein 11	chr13q14.11		11215	---	---	---	---		MmugDNA.16113.1.S1_at_AKAP11
MmugDNA.16114.1.S1_at	229287_at	PCNX	pecanex homolog (Drosophila)	chr14q24.2		22990	---	---	---	---		MmugDNA.16114.1.S1_at_PCNX
MmugDNA.16115.1.S1_at	1559817_at	---	---	---	---		LOC705132	hypothetical protein LOC705132	---		705132	MmugDNA.16115.1.S1_at_---
MmugDNA.16116.1.S1_at	220192_x_at	SPDEF	SAM pointed domain containing ets transcription factor	chr6p21.3		25803	LOC718648	similar to SAM pointed domain containing ets transcription factor	---		718648	MmugDNA.16116.1.S1_at_SPDEF
MmugDNA.16117.1.S1_at	1565651_at	ARF1	ADP-ribosylation factor 1	chr1q42		375	---	---	---	---		MmugDNA.16117.1.S1_at_ARF1
MmugDNA.16118.1.S1_at	206183_s_at	HERC3	hect domain and RLD 3	chr4q21		8916	HERC3	hect domain and RLD 3	---		703108	MmugDNA.16118.1.S1_at_HERC3
MmugDNA.16119.1.S1_at	220662_s_at	HEYL	hairy/enhancer-of-split related with YRPW motif-like	chr1p34.3		26508	LOC716117	similar to hairy/enhancer-of-split related with YRPW motif-like	---		716117	MmugDNA.16119.1.S1_at_HEYL
MmugDNA.161.1.S1_at	228914_at	---	---	---	---		---	---	---	---		MmugDNA.161.1.S1_at_---
MmugDNA.16120.1.S1_at	228693_at	CCDC50	coiled-coil domain containing 50	chr3q28		152137	---	---	---	---		MmugDNA.16120.1.S1_at_CCDC50
MmugDNA.16121.1.S1_at	1554071_at	CCDC67	coiled-coil domain containing 67	chr11q21		159989	CCDC67	coiled-coil domain containing 67	---		695855	MmugDNA.16121.1.S1_at_CCDC67
MmugDNA.1612.1.S1_at	233529_at	---	---	---	---		---	---	---	---		MmugDNA.1612.1.S1_at_---
MmugDNA.16122.1.S1_at	207963_at	C6orf54	chromosome 6 open reading frame 54	chr6q27		26236	---	---	---	---		MmugDNA.16122.1.S1_at_C6orf54
MmugDNA.16123.1.S1_at	242115_at	---	---	---	---		---	---	---	---		MmugDNA.16123.1.S1_at_---
MmugDNA.16124.1.S1_at	207698_at	C6orf123	chromosome 6 open reading frame 123	chr6q27		26238	---	---	---	---		MmugDNA.16124.1.S1_at_C6orf123
MmugDNA.16125.1.S1_at	203711_s_at	HIBCH	3-hydroxyisobutyryl-Coenzyme A hydrolase	chr2q32.2		26275	LOC696182	similar to 3-hydroxyisobutyryl-Coenzyme A hydrolase isoform 1	---		696182	MmugDNA.16125.1.S1_at_HIBCH
MmugDNA.16126.1.S1_at	237112_at	---	---	---	---		---	---	---	---		MmugDNA.16126.1.S1_at_---
MmugDNA.16127.1.S1_at	214523_at	CEBPE	CCAAT/enhancer binding protein (C/EBP), epsilon	chr14q11.2		1053	LOC713442	similar to CCAAT/enhancer binding protein epsilon	---		713442	MmugDNA.16127.1.S1_at_CEBPE
MmugDNA.16129.1.S1_at	235299_at	SLC41A2	solute carrier family 41, member 2	chr12q23.3		84102	---	---	---	---		MmugDNA.16129.1.S1_at_SLC41A2
MmugDNA.16130.1.S1_at	229668_at	---	---	---	---		---	---	---	---		MmugDNA.16130.1.S1_at_---
MmugDNA.16131.1.S1_at	217923_at	PEF1	penta-EF-hand domain containing 1	chr1p34		553115	PEF1	penta-EF-hand domain containing 1	---		706038	MmugDNA.16131.1.S1_at_PEF1
MmugDNA.1613.1.S1_at	210256_s_at	PIP5K1A	phosphatidylinositol-4-phosphate 5-kinase, type I, alpha	chr1q22-q24		8394	LOC695322	PIP5K1A pseudogene	---		695322	MmugDNA.1613.1.S1_at_PIP5K1A
MmugDNA.16132.1.S1_at	206810_at	ZNF44	zinc finger protein 44	chr19p13.2		51710	---	---	---	---		MmugDNA.16132.1.S1_at_ZNF44
MmugDNA.16133.1.S1_at	NA	NA	NA	NA	NA		NA	NA	NA	NA		MmugDNA.16133.1.S1_at_NA
MmugDNA.16134.1.S1_at	221628_s_at	N-PAC	cytokine-like nuclear factor n-pac	chr16p13.3		84656	LOC713844	similar to cytokine-like nuclear factor n-pac	---		713844	MmugDNA.16134.1.S1_at_N-PAC
MmugDNA.16135.1.S1_at	232176_at	SLITRK6	SLIT and NTRK-like family, member 6	chr13q31.1		84189	SLITRK6	SLIT and NTRK-like family, member 6	---		699338	MmugDNA.16135.1.S1_at_SLITRK6
MmugDNA.16136.1.S1_at	215204_at	---	---	---	---		---	---	---	---		MmugDNA.16136.1.S1_at_---
MmugDNA.16137.1.S1_at	204604_at	PFTK1	PFTAIRE protein kinase 1	chr7q21-q22		5218	PFTK1	PFTAIRE protein kinase 1	---		703442	MmugDNA.16137.1.S1_at_PFTK1
MmugDNA.16138.1.S1_at	219571_s_at	ZNF12	zinc finger protein 12	chr7p22.1		7559	ZNF12	zinc finger protein 12	---		705346	MmugDNA.16138.1.S1_at_ZNF12
MmugDNA.16139.1.S1_at	1559287_at	---	---	---	---		---	---	---	---		MmugDNA.16139.1.S1_at_---
MmugDNA.16140.1.S1_at	218388_at	PGLS	6-phosphogluconolactonase	chr19p13.2		25796	LOC719149	similar to 6-phosphogluconolactonase	---		719149	MmugDNA.16140.1.S1_at_PGLS
MmugDNA.16141.1.S1_at	NA	NA	NA	NA	NA		NA	NA	NA	NA		MmugDNA.16141.1.S1_at_NA
MmugDNA.16141.1.S1_s_at	214678_x_at	ZFX	zinc finger protein, X-linked	chrXp21.3		7543	ZFX	zinc finger protein, X-linked	---		698892	MmugDNA.16141.1.S1_s_at_ZFX
MmugDNA.1614.1.S1_at	205122_at	TMEFF1	transmembrane protein with EGF-like and two follistatin-like domains 1	chr9q31		8577	LOC714325	similar to transmembrane protein with EGF-like and two follistatin-like domains 1	---		714325	MmugDNA.1614.1.S1_at_TMEFF1
MmugDNA.16142.1.S1_at	226645_at	KLF2	Kruppel-like factor 2 (lung)	chr19p13.13-p13.11		10365	LOC719492	similar to Krueppel-like factor 2 (Lung krueppel-like factor)	---		719492	MmugDNA.16142.1.S1_at_KLF2
MmugDNA.16143.1.S1_at	203314_at	GTPBP6	GTP binding protein 6 (putative)	chrXp22.33; Yp11.32		8225	LOC719390	similar to pseudoautosomal GTP-binding protein-like protein	---		719390	MmugDNA.16143.1.S1_at_GTPBP6
MmugDNA.16144.1.S1_at	218634_at	PHLDA3	pleckstrin homology-like domain, family A, member 3	chr1q31		23612	LOC709782	similar to pleckstrin homology-like domain, family A, member 3	---		709782	MmugDNA.16144.1.S1_at_PHLDA3
MmugDNA.16145.1.S1_at	217563_at	CLOCK	clock homolog (mouse)	chr4q12		9575	---	---	---	---		MmugDNA.16145.1.S1_at_CLOCK
MmugDNA.16146.1.S1_at	205389_s_at	ANK1	ankyrin 1, erythrocytic	chr8p11.1		286	LOC710904	similar to ankyrin 1 isoform 1	---		710904	MmugDNA.16146.1.S1_at_ANK1
MmugDNA.16147.1.S1_at	240782_at	---	---	---	---		---	---	---	---		MmugDNA.16147.1.S1_at_---
MmugDNA.16148.1.S1_at	226668_at	WDSUB1	WD repeat, sterile alpha motif and U-box domain containing 1	chr2q24.2		151525	WDSUB1	WD repeat, sterile alpha motif and U-box domain containing 1	---		699456	MmugDNA.16148.1.S1_at_WDSUB1
MmugDNA.16149.1.S1_at	237034_at	---	---	---	---		---	---	---	---		MmugDNA.16149.1.S1_at_---
MmugDNA.16150.1.S1_at	1553622_a_at	FSIP1	fibrous sheath interacting protein 1	chr15q14		161835	LOC711746	Similar to paired immunoglobulin-like type 2 receptor alpha isoform 2 precursor	---		711746	MmugDNA.16150.1.S1_at_FSIP1
MmugDNA.16151.1.S1_at	238316_at	ZNF567	zinc finger protein 567	chr19q13.12		163081	LOC713173	similar to zinc finger protein 567	---		713173	MmugDNA.16151.1.S1_at_ZNF567
MmugDNA.1615.1.S1_at	211205_x_at	PIP5K1A	phosphatidylinositol-4-phosphate 5-kinase, type I, alpha	chr1q22-q24		8394	---	---	---	---		MmugDNA.1615.1.S1_at_PIP5K1A
MmugDNA.16152.1.S1_at	217811_at	SELT	selenoprotein T	chr3q25.1		51714	SELT	selenoprotein T	---		710572	MmugDNA.16152.1.S1_at_SELT
MmugDNA.16153.1.S1_at	243261_at	LOC154822	hypothetical LOC154822	chr7q36.3		154822	---	---	---	---		MmugDNA.16153.1.S1_at_LOC154822
MmugDNA.16154.1.S1_at	218773_s_at	MSRB2	methionine sulfoxide reductase B2	chr10p12		22921	LOC711305	similar to Methionine-R-sulfoxide reductase B2	---		711305	MmugDNA.16154.1.S1_at_MSRB2
MmugDNA.16155.1.S1_at	231195_at	KLRG2	killer cell lectin-like receptor subfamily G, member 2	chr7q34		346689	---	---	---	---		MmugDNA.16155.1.S1_at_KLRG2
MmugDNA.16156.1.S1_at	206824_at	CES4	carboxylesterase 4-like	chr16q12.2		51716	---	---	---	---		MmugDNA.16156.1.S1_at_CES4
MmugDNA.16157.1.S1_at	206966_s_at	KLF12	Kruppel-like factor 12	chr13q22		11278	KLF12	Kruppel-like factor 12	---		694575	MmugDNA.16157.1.S1_at_KLF12
MmugDNA.16158.1.S1_at	220423_at	PLA2G2D	phospholipase A2, group IID	chr1p36.12		26279	PLA2G2D	phospholipase A2, group IID	---		704735	MmugDNA.16158.1.S1_at_PLA2G2D
MmugDNA.16159.1.S1_at	232760_at	TEX15	testis expressed 15	chr8p12		56154	---	---	---	---		MmugDNA.16159.1.S1_at_TEX15
MmugDNA.16160.1.S1_at	NA	NA	NA	NA	NA		NA	NA	NA	NA		MmugDNA.16160.1.S1_at_NA
MmugDNA.16161.1.S1_at	1566954_at	---	---	---	---		---	---	---	---		MmugDNA.16161.1.S1_at_---
MmugDNA.1616.1.S1_at	242846_at	---	---	---	---		---	---	---	---		MmugDNA.1616.1.S1_at_---
MmugDNA.16162.1.S1_at	230480_at	PIWIL4	piwi-like 4 (Drosophila)	chr11q21		143689	PIWIL4	piwi-like 4 (Drosophila)	---		697947	MmugDNA.16162.1.S1_at_PIWIL4
MmugDNA.16163.1.S1_at	225567_at	---	---	---	---		---	---	---	---		MmugDNA.16163.1.S1_at_---

MmugDNA.16164.1.S1_at	242012_at	LOC100132625	hypothetical protein LOC100132625	chrXq26.3		100132625	LOC694279	hypothetical protein LOC694279	---		694279	MmugDNA.16164.1.S1_at_LOC1001326
MmugDNA.16165.1.S1_at	239020_at	FUT10	fucosyltransferase 10 (alpha (1,3) fucosyltransferase)	chr8p12		84750	FUT10	fucosyltransferase 10 (alpha (1,3) fucosyltransferase)	---		697659	MmugDNA.16165.1.S1_at_FUT10
MmugDNA.16166.1.S1_at	204856_at	B3GNT3	UDP-GlcNAc:betaGal beta-1,3-N-acetylglucosaminyltransferase 3	chr19p13.1		10331	B3GNT3	UDP-GlcNAc:betaGal beta-1,3-N-acetylglucosaminyltransferase 3	---		719221	MmugDNA.16166.1.S1_at_B3GNT3
MmugDNA.16167.1.S1_at	237109_at	TOM1L2	target of myb1-like 2 (chicken)	chr17p11.2		146691	TOM1L2	target of myb1-like 2 (chicken)	---		699471	MmugDNA.16167.1.S1_at_TOM1L2
MmugDNA.16168.1.S1_at	NA	NA	NA	NA	NA		NA	NA	NA	NA		MmugDNA.16168.1.S1_at_NA
MmugDNA.16168.1.S1_s_at	209259_s_at	SMC3	structural maintenance of chromosomes 3	chr10q25		9126	SMC3	structural maintenance of chromosomes 3	---		695123	MmugDNA.16168.1.S1_s_at_SMC3
MmugDNA.16169.1.S1_at	207995_s_at	CLEC4M	C-type lectin domain family 4, member M	chr19p13		10332	---	---	---	---		MmugDNA.16169.1.S1_at_CLEC4M
MmugDNA.16170.1.S1_s_at	202857_at	CNPY2	canopy 2 homolog (zebrafish)	chr12q15		10330	LOC712552	similar to MIR-interacting saposin-like protein precursor (Transmembrane protein 4) (Putative secrete	---		712552	MmugDNA.16170.1.S1_s_at_CNPY2
MmugDNA.16171.1.S1_at	1570099_at	---	---	---	---		---	---	---	---		MmugDNA.16171.1.S1_at_---
MmugDNA.1617.1.S1_at	NA	NA	NA	NA	NA		NA	NA	NA	NA		MmugDNA.1617.1.S1_at_NA
MmugDNA.16172.1.S1_at	204808_s_at	TMEM5	transmembrane protein 5	chr12q14.2		10329	TMEM5	transmembrane protein 5	---		717102	MmugDNA.16172.1.S1_at_TMEM5
MmugDNA.16173.1.S1_at	213694_at	RSBN1	round spermatid basic protein 1	chr1p13.2		54665	LOC702307	Similar to JNK1-associated membrane protein	---		702307	MmugDNA.16173.1.S1_at_RSBN1
MmugDNA.16174.1.S1_at	208998_at	UCP2	uncoupling protein 2 (mitochondrial, proton carrier)	chr11q13		7351	---	---	---	---		MmugDNA.16174.1.S1_at_UCP2
MmugDNA.16175.1.S1_at	216865_at	COL14A1	collagen, type XIV, alpha 1	chr8q23		7373	LOC702878	similar to collagen, type XIV, alpha 1	---		702878	MmugDNA.16175.1.S1_at_COL14A1
MmugDNA.16176.1.S1_at	243425_at	---	---	---	---		---	---	---	---		MmugDNA.16176.1.S1_at_---
MmugDNA.16177.1.S1_at	1552496_a_at	COBL	cordon-bleu homolog (mouse)	chr7p12.1		23242	COBL	similar to cordon-bleu homolog	---		693867	MmugDNA.16177.1.S1_at_COBL
MmugDNA.16178.1.S1_at	1563745_a_at	LOC283050	hypothetical LOC283050	chr10q22.3		283050	---	---	---	---		MmugDNA.16178.1.S1_at_LOC283050
MmugDNA.16179.1.S1_at	NA	NA	NA	NA	NA		NA	NA	NA	NA		MmugDNA.16179.1.S1_at_NA
MmugDNA.16180.1.S1_at	217823_s_at	UBE2J1	ubiquitin-conjugating enzyme E2, J1 (UBC6 homolog, yeast)	chr6q15		51465	UBE2J1	ubiquitin-conjugating enzyme E2, J1 (UBC6 homolog, yeast)	---		701100	MmugDNA.16180.1.S1_at_UBE2J1
MmugDNA.16181.1.S1_at	225500_x_at	SCAF1	SR-related CTD-associated factor 1	chr19q13.3-q13.4		58506	---	---	---	---		MmugDNA.16181.1.S1_at_SCAF1
MmugDNA.1618.1.S1_at	242883_at	OTOS	otospiralin	chr2q37.3		150677	OTOS	otospiralin	---		698202	MmugDNA.1618.1.S1_at_OTOS
MmugDNA.16182.1.S1_at	229568_at	MOBKL2B	MOB1, Mps One Binder kinase activator-like 2B (yeast)	chr9p21.2		79817	LOC706919	similar to MOB1, Mps One Binder kinase activator-like 2B	---		706919	MmugDNA.16182.1.S1_at_MOBKL2B
MmugDNA.16183.1.S1_at	217824_at	UBE2J1	ubiquitin-conjugating enzyme E2, J1 (UBC6 homolog, yeast)	chr6q15		51465	UBE2J1	ubiquitin-conjugating enzyme E2, J1 (UBC6 homolog, yeast)	---		701100	MmugDNA.16183.1.S1_at_UBE2J1
MmugDNA.16184.1.S1_at	232371_at	MARCH7	Membrane-associated ring finger (C3HC4) 7	chr2q24.2		64844	---	---	---	---		MmugDNA.16184.1.S1_at_MARCH7
MmugDNA.16185.1.S1_at	217873_at	CAB39	calcium binding protein 39	chr2q37.1		51719	CAB39	calcium binding protein 39	---		711166	MmugDNA.16185.1.S1_at_CAB39
MmugDNA.16186.1.S1_at	220746_s_at	UIMC1	ubiquitin interaction motif containing 1	chr5q35.2		51720	UIMC1	ubiquitin interaction motif containing 1	---		705412	MmugDNA.16186.1.S1_at_UIMC1
MmugDNA.16187.1.S1_at	201391_at	TRAP1	TNF receptor-associated protein 1	chr16p13.3		10131	TRAP1	TNF receptor-associated protein 1	---		706234	MmugDNA.16187.1.S1_at_TRAP1
MmugDNA.16188.1.S1_at	56829_at	TRAPPC9	trafficking protein particle complex 9	chr8q24.3		83696	LOC699757	similar to NIK and IKK(beta) binding protein	---		699757	MmugDNA.16188.1.S1_at_TRAPPC9
MmugDNA.16189.1.S1_at	NA	NA	NA	NA	NA		NA	NA	NA	NA		MmugDNA.16189.1.S1_at_NA
MmugDNA.16190.1.S1_at	215076_s_at	COL3A1	collagen, type III, alpha 1	chr2q31		1281	---	---	---	---		MmugDNA.16190.1.S1_at_COL3A1
MmugDNA.16191.1.S1_at	236687_at	---	---	---	---		---	---	---	---		MmugDNA.16191.1.S1_at_---
MmugDNA.1619.1.S1_at	243533_x_at	---	---	---	---		---	---	---	---		MmugDNA.1619.1.S1_at_---
MmugDNA.16192.1.S1_s_at	244532_x_at	---	---	---	---		---	---	---	---		MmugDNA.16192.1.S1_s_at_---
MmugDNA.16193.1.S1_at	203529_at	PPP6C	protein phosphatase 6, catalytic subunit	chr9q33.3		5537	TSEN34	TRNA splicing endonuclease 34 homolog (S. cerevisiae)	---		693919	MmugDNA.16193.1.S1_at_PPP6C
MmugDNA.16194.1.S1_at	237136_at	---	---	---	---		---	---	---	---		MmugDNA.16194.1.S1_at_---
MmugDNA.16195.1.S1_at	1552432_at	MFSD6L	major facilitator superfamily domain containing 6-like	chr17p13.1		162387	LOC717575	similar to CG12858-PA	---		717575	MmugDNA.16195.1.S1_at_MFSD6L
MmugDNA.16196.1.S1_at	241748_x_at	DGCR14	DiGeorge syndrome critical region gene 14	chr22q11.21|22q11.2		8220	---	---	---	---		MmugDNA.16196.1.S1_at_DGCR14
MmugDNA.16197.1.S1_at	224306_at	NPC1L1	NPC1 (Niemann-Pick disease, type C1, gene)-like 1	chr7p13		29881	---	---	---	---		MmugDNA.16197.1.S1_at_NPC1L1
MmugDNA.16197.1.S1_s_at	224305_s_at	NPC1L1	NPC1 (Niemann-Pick disease, type C1, gene)-like 1	chr7p13		29881	NPC1L1	NPC1 (Niemann-Pick disease, type C1, gene)-like 1	---		699235	MmugDNA.16197.1.S1_s_at_NPC1L1
MmugDNA.16198.1.S1_at	215802_at	---	---	---	---		---	---	---	---		MmugDNA.16198.1.S1_at_---
MmugDNA.16199.1.S1_at	1556999_at	LOC100271832	hypothetical LOC100271832	---		100271832	---	---	---	---		MmugDNA.16199.1.S1_at_LOC1002718
MmugDNA.16.1.S1_at	211935_at	ARL6IP1	ADP-ribosylation factor-like 6 interacting protein 1	chr16p12-p11.2		23204	LOC693347	similar to ARL-6-interacting protein 1 (ADP-ribosylation-like factor 6-interacting protein 1) (Aip-1)	---		693347	MmugDNA.16.1.S1_at_ARL6IP1
MmugDNA.16200.1.S1_at	203520_s_at	ZNF318	zinc finger protein 318	chr6pter-p12.1		24149	---	---	---	---		MmugDNA.16200.1.S1_at_ZNF318
MmugDNA.16201.1.S1_at	1561574_at	---	---	---	---		---	---	---	---		MmugDNA.16201.1.S1_at_---
MmugDNA.1620.1.S1_s_at	1558148_x_at	FLJ90757	hypothetical LOC440465	chr17q25.3		440465	---	---	---	---		MmugDNA.1620.1.S1_s_at_FLJ90757
MmugDNA.16202.1.S1_at	227915_at	ASB2	ankyrin repeat and SOCS box-containing 2	chr14q31-q32		51676	ASB2	ankyrin repeat and SOCS box-containing 2	---		699882	MmugDNA.16202.1.S1_at_ASB2
MmugDNA.16203.1.S1_at	207215_at	GSTTP1	glutathione S-transferase theta pseudogene 1	chr22q12		25774	GSTT1	glutathione S-transferase theta 1	---		699679	MmugDNA.16203.1.S1_at_GSTTP1
MmugDNA.16204.1.S1_at	1569895_at	LOC100133612	similar to hCG1815312	---		100133612	---	---	---	---		MmugDNA.16204.1.S1_at_LOC1001336
MmugDNA.16205.1.S1_at	1562558_at	LOC440704	hypothetical gene supported by BC042042	chr1q31.1-q31.2		440704	---	---	---	---		MmugDNA.16205.1.S1_at_LOC440704
MmugDNA.16206.1.S1_at	1561004_at	---	---	---	---		---	---	---	---		MmugDNA.16206.1.S1_at_---
MmugDNA.16207.1.S1_at	1554616_at	SERPINB8	serpin peptidase inhibitor, clade B (ovalbumin), member 8	chr18q21.3		5271	SERPINB6	serpin peptidase inhibitor, clade B (ovalbumin), member 6	---		702046	MmugDNA.16207.1.S1_at_SERPINB8
MmugDNA.16208.1.S1_at	1562162_at	---	---	---	---		---	---	---	---		MmugDNA.16208.1.S1_at_---
MmugDNA.16209.1.S1_at	239896_at	---	---	---	---		---	---	---	---		MmugDNA.16209.1.S1_at_---
MmugDNA.16210.1.S1_at	209834_at	CHST3	carbohydrate (chondroitin 6) sulfotransferase 3	chr10q22.1		9469	CCL8	Chemokine (C-C motif) ligand 8	---		574179	MmugDNA.16210.1.S1_at_CHST3
MmugDNA.16211.1.S1_at	1553690_at	SGOL1	shugoshin-like 1 (S. pombe)	chr3p24.3		151648	SGOL1	shugoshin-like 1 (S. pombe)	---		698231	MmugDNA.16211.1.S1_at_SGOL1
MmugDNA.1621.1.S1_at	NA	NA	NA	NA	NA		NA	NA	NA	NA		MmugDNA.1621.1.S1_at_NA
MmugDNA.16212.1.S1_at	208473_s_at	GP2	glycoprotein 2 (zymogen granule membrane)	chr16p12		2813	GP2	glycoprotein 2 (zymogen granule membrane)	---		695766	MmugDNA.16212.1.S1_at_GP2
MmugDNA.16213.1.S1_at	220164_s_at	FBXO40	F-box protein 40	chr3q13.33		51725	FBXO40	F-box protein 40	---		713933	MmugDNA.16213.1.S1_at_FBXO40
MmugDNA.16214.1.S1_at	217870_s_at	CMPK1	cytidine monophosphate (UMP-CMP) kinase 1, cytosolic	chr1p32		51727	PDZD2	PDZ domain containing 2	---		696141	MmugDNA.16214.1.S1_at_CMPK1
MmugDNA.16215.1.S1_at	218866_s_at	POLR3K	polymerase (RNA) III (DNA directed) polypeptide K, 12.3 kDa	chr16p13.3		51728	POLR3K	similar to polymerase (RNA) III (DNA directed) polypeptide K	---		697361	MmugDNA.16215.1.S1_at_POLR3K
MmugDNA.16216.1.S1_at	228073_at	NANP	N-acetylneuraminic acid phosphatase	chr20p11.1		140838	NANP	N-acetylneuraminic acid phosphatase	---		706491	MmugDNA.16216.1.S1_at_NANP
MmugDNA.16217.1.S1_at	215522_at	SORCS3	sortilin-related VPS10 domain containing receptor 3	chr10q23-q25		22986	SORCS3	sortilin-related VPS10 domain containing receptor 3	---		693500	MmugDNA.16217.1.S1_at_SORCS3
MmugDNA.16218.1.S1_s_at	206798_x_at	DLEC1	deleted in lung and esophageal cancer 1	chr3p22-p21.3		9940	LOC697163	hypothetical protein LOC697163	---		697163	MmugDNA.16218.1.S1_s_at_DLEC1
MmugDNA.16219.1.S1_at	216159_s_at	---	---	---	---		---	---	---	---		MmugDNA.16219.1.S1_at_---
MmugDNA.162.1.S1_at	238988_at	---	---	---	---		---	---	---	---		MmugDNA.162.1.S1_at_---
MmugDNA.16220.1.S1_at	214501_s_at	H2AFY	H2A histone family, member Y	chr5q31.3-q32		9555	LOC715746	Similar to Nanog homeobox	---		715746	MmugDNA.16220.1.S1_at_H2AFY
MmugDNA.16221.1.S1_at	NA	NA	NA	NA	NA		NA	NA	NA	NA		MmugDNA.16221.1.S1_at_NA
MmugDNA.1622.1.S1_at	1555313_a_at	MCF2	MCF.2 cell line derived transforming sequence	chrXq27		4168	---	---	---	---		MmugDNA.1622.1.S1_at_MCF2
MmugDNA.16222.1.S1_at	244454_at	---	---	---	---		---	---	---	---		MmugDNA.16222.1.S1_at_---
MmugDNA.16223.1.S1_at	232048_at	FAM76B	family with sequence similarity 76, member B	chr11q21		143684	LOC699581	similar to family with sequence similarity 76, member A	---		699581	MmugDNA.16223.1.S1_at_FAM76B
MmugDNA.16224.1.S1_s_at	224997_x_at	H19	H19, imprinted maternally expressed transcript (non-protein coding)	chr11p15.5		283120	H19	H19, imprinted maternally expressed transcript (non-protein coding)	---		704308	MmugDNA.16224.1.S1_s_at_H19
MmugDNA.16225.1.S1_at	224203_at	SUFU	suppressor of fused homolog (Drosophila)	chr10q24.32		51684	---	---	---	---		MmugDNA.16225.1.S1_at_SUFU
MmugDNA.16226.1.S1_at	1568598_at	KAZALD1	Kazal-type serine peptidase inhibitor domain 1	chr10q24.31		81621	---	---	---	---		MmugDNA.16226.1.S1_at_KAZALD1
MmugDNA.16227.1.S1_at	236683_at	---	---	---	---		---	---	---	---		MmugDNA.16227.1.S1_at_---
MmugDNA.16228.1.S1_at	1557501_a_at	---	---	---	---		---	---	---	---		MmugDNA.16228.1.S1_at_---
MmugDNA.16229.1.S1_at	200637_s_at	PTPRF	protein tyrosine phosphatase, receptor type, F	chr1p34		5792	PTPRF	protein tyrosine phosphatase, receptor type, F	---		701160	MmugDNA.16229.1.S1_at_PTPRF
MmugDNA.16230.1.S1_at	225956_at	C5orf41	chromosome 5 open reading frame 41	chr5q35.2		153222	C6H5orf41	chromosome 5 open reading frame 41 ortholog	---		703459	MmugDNA.16230.1.S1_at_C5orf41
MmugDNA.16231.1.S1_at	200635_s_at	PTPRF	protein tyrosine phosphatase, receptor type, F	chr1p34		5792	PTPRF	protein tyrosine phosphatase, receptor type, F	---		701160	MmugDNA.16231.1.S1_at_PTPRF
MmugDNA.1623.1.S1_at	219839_x_at	TCL6	T-cell leukemia/lymphoma 6	chr14q32.1		27004	---	---	---	---		MmugDNA.1623.1.S1_at_TCL6
MmugDNA.16232.1.S1_at	238993_at	MATR3	Matrin 3	chr5q31.2		9782	---	---	---	---		MmugDNA.16232.1.S1_at_MATR3
MmugDNA.16233.1.S1_at	217049_x_at	PCDH11Y	protocadherin 11 Y-linked	chrYp11.2		83259	---	---	---	---		MmugDNA.16233.1.S1_at_PCDH11Y
MmugDNA.16234.1.S1_at	225957_at	C5orf41	chromosome 5 open reading frame 41	chr5q35.2		153222	C6H5orf41	chromosome 5 open reading frame 41 ortholog	---		703459	MmugDNA.16234.1.S1_at_C5orf41
MmugDNA.16235.1.S1_at	237345_at	---	---	---	---		---	---	---	---		MmugDNA.16235.1.S1_at_---
MmugDNA.16236.1.S1_at	242256_x_at	---	---	---	---		---	---	---	---		MmugDNA.16236.1.S1_at_---
MmugDNA.16237.1.S1_at	239313_at	LOC401320	Hypothetical LOC401320	chr7p15.1		401320	---	---	---	---		MmugDNA.16237.1.S1_at_LOC401320
MmugDNA.16238.1.S1_at	1557615_a_at	SLIT1	Slit homolog 1 (Drosophila)	chr10q23.3-q24		6585	---	---	---	---		MmugDNA.16238.1.S1_at_SLIT1
MmugDNA.16239.1.S1_at	NA	NA	NA	NA	NA		NA	NA	NA	NA		MmugDNA.16239.1.S1_at_NA
MmugDNA.16239.1.S1_s_at	227415_at	LOC283508	hypothetical protein LOC283508	chr13q13.3		283508	---	---	---	---		MmugDNA.16239.1.S1_s_at_LOC28350
MmugDNA.16240.1.S1_at	217286_s_at	NDRG3	NDRG family member 3	chr20q11.21-q11.23		57446	LOC702452	similar to N-myc downstream regulated gene 3	---		702452	MmugDNA.16240.1.S1_at_NDRG3
MmugDNA.16241.1.S1_at	239372_at	---	---	---	---		---	---	---	---		MmugDNA.16241.1.S1_at_---
MmugDNA.1624.1.S1_at	238686_at	FBXO3	F-box protein 3	chr11p13		26273	---	---	---	---		MmugDNA.1624.1.S1_at_FBXO3
MmugDNA.16242.1.S1_at	227985_at	---	---	---	---		---	---	---	---		MmugDNA.16242.1.S1_at_---
MmugDNA.16244.1.S1_at	236454_at	RNF212	ring finger protein 212	chr4p16.3		285498	LOC713795	hypothetical protein LOC713795	---		713795	MmugDNA.16244.1.S1_at_RNF212
MmugDNA.16245.1.S1_at	210333_at	NR5A1	nuclear receptor subfamily 5, group A, member 1	chr9q33		2516	LOC694183	similar to nuclear receptor subfamily 5, group A, member 1	---		694183	MmugDNA.16245.1.S1_at_NR5A1
MmugDNA.16246.1.S1_at	242540_at	DNHD1	dynein heavy chain domain 1	chr11p15.4		144132	---	---	---	---		MmugDNA.16246.1.S1_at_DNHD1
MmugDNA.16247.1.S1_at	239736_at	---	---	---	---		---	---	---	---		MmugDNA.16247.1.S1_at_---
MmugDNA.16248.1.S1_at	229063_s_at	CCDC107	coiled-coil domain containing 107	chr9p13.3		203260	CCDC107	similar to coiled-coil domain containing 107	---		696972	MmugDNA.16248.1.S1_at_CCDC107
MmugDNA.16248.1.S1_s_at	NA	NA	NA	NA	NA		NA	NA	NA	NA		MmugDNA.16248.1.S1_s_at_NA
MmugDNA.16249.1.S1_at	244017_at	---	---	---	---		---	---	---	---		MmugDNA.16249.1.S1_at_---
MmugDNA.16250.1.S1_at	231691_at	C3P1	complement component 3 precursor pseudogene	chr19p13.2		388503	LOC714567	similar to Complement C3 precursor	---		714567	MmugDNA.16250.1.S1_at_C3P1
MmugDNA.16251.1.S1_at	215682_at	LOC440792	proline dehydrogenase (oxidase) 1 pseudogene	chr22q11.21		440792	---	---	---	---		MmugDNA.16251.1.S1_at_LOC440792
MmugDNA.1625.1.S1_at	209799_at	PRKAA1	protein kinase, AMP-activated, alpha 1 catalytic subunit	chr5p12		5562	LOC695558	similar to protein kinase, AMP-activated, alpha 1 catalytic subunit	---		695558	MmugDNA.1625.1.S1_at_PRKAA1
MmugDNA.16252.1.S1_at	235443_at	LOC100131067	hypothetical protein LOC100131067	chr5q14.1		100131067	LOC711511	hypothetical protein LOC711511	---		711511	MmugDNA.16252.1.S1_at_LOC1001310
MmugDNA.16253.1.S1_at	NA	NA	NA	NA	NA		NA	NA	NA	NA		MmugDNA.16253.1.S1_at_NA
MmugDNA.16253.1.S1_s_at	209504_s_at	PLEKHB1	pleckstrin homology domain containing, family B (evectins) member 1	chr11q13.5-q14.1		58473	LOC718702	similar to pleckstrin homology domain containing, family B (evectins) member 1	---		718702	MmugDNA.16253.1.S1_s_at_PLEKHB1
MmugDNA.16254.1.S1_at	235115_at	PDE8B	phosphodiesterase 8B	chr5q14.1		8622	LOC712674	similar to phosphodiesterase 8B isoform 1	---		712674	MmugDNA.16254.1.S1_at_PDE8B
MmugDNA.16255.1.S1_at	219725_at	TREM2	triggering receptor expressed on myeloid cells 2	chr6p21.1		54209	TREM2	triggering receptor expressed on myeloid cells 2	---		719740	MmugDNA.16255.1.S1_at_TREM2
MmugDNA.16256.1.S1_at	231203_at	LOC100129633	similar to hCG1651427	chr9q32		100129633	---	---	---	---		MmugDNA.16256.1.S1_at_LOC1001296

MmugDNA.16257.1.S1_at	218426_s_at	RNF216	ring finger protein 216	chr7p22.1		54476	LOC701819	similar to TRIAD3 protein isoform a	---		701819	MmugDNA.16257.1.S1_at_RNF216
MmugDNA.16258.1.S1_at	232791_at	---	---	---	---		---	---	---	---		MmugDNA.16258.1.S1_at_---
MmugDNA.16259.1.S1_at	239962_at	---	---	---	---		---	---	---	---		MmugDNA.16259.1.S1_at_---
MmugDNA.16261.1.S1_at	228983_at	---	---	---	---		---	---	---	---		MmugDNA.16261.1.S1_at_---
MmugDNA.1626.1.S1_at	1559884_at	CDKN2BAS	CDKN2B antisense RNA (non-protein coding)	chr9p21.3		100048912	---	---	---	---		MmugDNA.1626.1.S1_at_CDKN2BAS
MmugDNA.16262.1.S1_at	NA	NA	NA	NA	NA		NA	NA	NA	NA		MmugDNA.16262.1.S1_at_NA
MmugDNA.16263.1.S1_at	236537_at	---	---	---	---		---	---	---	---		MmugDNA.16263.1.S1_at_---
MmugDNA.16264.1.S1_at	NA	NA	NA	NA	NA		NA	NA	NA	NA		MmugDNA.16264.1.S1_at_NA
MmugDNA.16265.1.S1_at	216118_at	---	---	---	---		---	---	---	---		MmugDNA.16265.1.S1_at_---
MmugDNA.16266.1.S1_at	NA	NA	NA	NA	NA		NA	NA	NA	NA		MmugDNA.16266.1.S1_at_NA
MmugDNA.16266.1.S1_s_at	224423_x_at	PMCHL2	pro-melanin-concentrating hormone-like 2	chr5q13		5370	---	---	---	---		MmugDNA.16266.1.S1_s_at_PMCHL2
MmugDNA.16267.1.S1_at	223725_at	C3orf42	chromosome 3 open reading frame 42	chr3p26-p25		84657	---	---	---	---		MmugDNA.16267.1.S1_at_C3orf42
MmugDNA.16268.1.S1_at	NA	NA	NA	NA	NA		NA	NA	NA	NA		MmugDNA.16268.1.S1_at_NA
MmugDNA.16268.1.S1_s_at	201106_at	GPX4	glutathione peroxidase 4 (phospholipid hydroperoxidase)	chr19p13.3		2879	GPX4	glutathione peroxidase 4	---		705333	MmugDNA.16268.1.S1_s_at_GPX4
MmugDNA.16269.1.S1_at	1559458_at	---	---	---	---		---	---	---	---		MmugDNA.16269.1.S1_at_---
MmugDNA.16270.1.S1_at	1553335_x_at	LOC285696	hypothetical LOC285696	chr5p15.1		285696	---	---	---	---		MmugDNA.16270.1.S1_at_LOC285696
MmugDNA.16270.1.S1_x_at	NA	NA	NA	NA	NA		NA	NA	NA	NA		MmugDNA.16270.1.S1_x_at_NA
MmugDNA.16271.1.S1_at	1561050_a_at	---	---	---	---		---	---	---	---		MmugDNA.16271.1.S1_at_---
MmugDNA.1627.1.S1_at	231751_at	ABCG8	ATP-binding cassette, sub-family G (WHITE), member 8	chr2p21		64241	LOC713676	similar to sterolin 2	---		713676	MmugDNA.1627.1.S1_at_ABCG8
MmugDNA.16272.1.S1_at	227910_at	XPNPEP3	X-prolyl aminopeptidase (aminopeptidase P) 3, putative	chr22q13.31-q13.33		63929	---	---	---	---		MmugDNA.16272.1.S1_at_XPNPEP3
MmugDNA.16273.1.S1_at	240810_at	---	---	---	---		---	---	---	---		MmugDNA.16273.1.S1_at_---
MmugDNA.16274.1.S1_at	NA	NA	NA	NA	NA		NA	NA	NA	NA		MmugDNA.16274.1.S1_at_NA
MmugDNA.16275.1.S1_at	1555412_at	FBXL21	F-box and leucine-rich repeat protein 21	chr5q31		26223	---	---	---	---		MmugDNA.16275.1.S1_at_FBXL21
MmugDNA.16276.1.S1_at	224854_s_at	SLAIN2	SLAIN motif family, member 2	chr4p12		57606	SLAIN2	SLAIN motif family, member 2	---		711866	MmugDNA.16276.1.S1_at_SLAIN2
MmugDNA.16277.1.S1_at	224845_s_at	SLAIN2	SLAIN motif family, member 2	chr4p12		57606	SLAIN2	SLAIN motif family, member 2	---		711866	MmugDNA.16277.1.S1_at_SLAIN2
MmugDNA.16278.1.S1_at	224844_at	SLAIN2	SLAIN motif family, member 2	chr4p12		57606	SLAIN2	SLAIN motif family, member 2	---		711866	MmugDNA.16278.1.S1_at_SLAIN2
MmugDNA.16279.1.S1_at	224843_at	SLAIN2	SLAIN motif family, member 2	chr4p12		57606	SLAIN2	SLAIN motif family, member 2	---		711866	MmugDNA.16279.1.S1_at_SLAIN2
MmugDNA.16280.1.S1_at	226853_at	BMP2K	BMP2 inducible kinase	chr4q21.21		55589	BMP2K	BMP-2 inducible kinase	---		697001	MmugDNA.16280.1.S1_at_BMP2K
MmugDNA.16281.1.S1_at	1555323_at	ABCB9	ATP-binding cassette, sub-family B (MDR/TAP), member 9	chr12q24		23457	---	---	---	---		MmugDNA.16281.1.S1_at_ABCB9
MmugDNA.1628.1.S1_at	200738_s_at	PGK1	phosphoglycerate kinase 1	chrXq13		5230	LOC701144	Hypothetical protein LOC701144	---		701144	MmugDNA.1628.1.S1_at_PGK1
MmugDNA.16282.1.S1_x_at	215852_x_at	C20orf117	chromosome 20 open reading frame 117	chr20q11.23		140710	---	---	---	---		MmugDNA.16282.1.S1_x_at_C20orf117
MmugDNA.16283.1.S1_s_at	215118_s_at	IGHG1	Immunoglobulin heavy constant gamma 1 (G1m marker)	chr14q32.33		3500	IGHA2	similar to Ig alpha-2 chain C region	---		720839	MmugDNA.16283.1.S1_s_at_IGHG1
MmugDNA.16284.1.S1_at	229757_at	NAF1	Nuclear assembly factor 1 homolog (S. cerevisiae)	chr4q32.2		92345	LOC718077	Similar to PMS1 protein homolog 2 (DNA mismatch repair protein PMS2)	---		718077	MmugDNA.16284.1.S1_at_NAF1
MmugDNA.16285.1.S1_at	208032_s_at	GRIA3	glutamate receptor, ionotrophic, AMPA 3	chrXq25-q26		2892	BPHL	Biphenyl hydrolase-like	---		708121	MmugDNA.16285.1.S1_at_GRIA3
MmugDNA.16285.1.S1_s_at	NA	NA	NA	NA	NA		NA	NA	NA	NA		MmugDNA.16285.1.S1_s_at_NA
MmugDNA.16286.1.S1_at	1568978_s_at	C11orf21	chromosome 11 open reading frame 21	chr11p15.5		29125	---	---	---	---		MmugDNA.16286.1.S1_at_C11orf21
MmugDNA.16287.1.S1_at	207242_s_at	GRIK1	glutamate receptor, ionotropic, kainate 1	chr21q22.11		2897	LOC721585	Similar to annexin A2 isoform 1	---		721585	MmugDNA.16287.1.S1_at_GRIK1
MmugDNA.16288.1.S1_at	241143_at	---	---	---	---		---	---	---	---		MmugDNA.16288.1.S1_at_---
MmugDNA.16289.1.S1_at	236733_at	---	---	---	---		---	---	---	---		MmugDNA.16289.1.S1_at_---
MmugDNA.16290.1.S1_at	240530_at	---	---	---	---		---	---	---	---		MmugDNA.16290.1.S1_at_---
MmugDNA.16291.1.S1_at	NA	NA	NA	NA	NA		NA	NA	NA	NA		MmugDNA.16291.1.S1_at_NA
MmugDNA.16291.1.S1_s_at	221690_s_at	NLRP2	NLR family, pyrin domain containing 2	chr19q13.42		55655	LOC708701	similar to NACHT-, LRR- and PYD-containing protein 2 (PYRIN domain- and NACHT domain-contain	---		708701	MmugDNA.16291.1.S1_s_at_NLRP2
MmugDNA.1629.1.S1_at	1558987_at	---	---	---	---		---	---	---	---		MmugDNA.1629.1.S1_at_---
MmugDNA.16292.1.S1_at	234980_at	TMEM56	transmembrane protein 56	chr1p21.3		148534	LOC709729	similar to transmembrane protein 56	---		709729	MmugDNA.16292.1.S1_at_TMEM56
MmugDNA.16293.1.S1_at	1555689_at	CD80	CD80 molecule	chr3q13.3-q21		941	CD80	CD80 molecule	---		732518	MmugDNA.16293.1.S1_at_CD80
MmugDNA.16294.1.S1_at	213615_at	LPCAT3	Lysophosphatidylcholine acyltransferase 3	chr12p13		10162	---	---	---	---		MmugDNA.16294.1.S1_at_LPCAT3
MmugDNA.16295.1.S1_at	209644_x_at	CDKN2A	cyclin-dependent kinase inhibitor 2A (melanoma, p16, inhibits CDK4)	chr9p21		1029	---	---	---	---		MmugDNA.16295.1.S1_at_CDKN2A
MmugDNA.16295.1.S1_s_at	NA	NA	NA	NA	NA		NA	NA	NA	NA		MmugDNA.16295.1.S1_s_at_NA
MmugDNA.16296.1.S1_at	1557476_at	PFN4	profilin family, member 4	chr2p23.3		375189	---	---	---	---		MmugDNA.16296.1.S1_at_PFN4
MmugDNA.16297.1.S1_at	225510_at	OAF	OAF homolog (Drosophila)	chr11q23.3		220323	---	---	---	---		MmugDNA.16297.1.S1_at_OAF
MmugDNA.16298.1.S1_at	230366_at	LOC100134119	similar to hCG2018924	---		100134119	---	---	---	---		MmugDNA.16298.1.S1_at_LOC1001341
MmugDNA.16299.1.S1_at	214358_at	ACACA	acetyl-Coenzyme A carboxylase alpha	chr17q21		31	LOC714218	similar to acetyl-Coenzyme A carboxylase alpha isoform 1	---		714218	MmugDNA.16299.1.S1_at_ACACA
MmugDNA.16300.1.S1_at	NA	NA	NA	NA	NA		NA	NA	NA	NA		MmugDNA.16300.1.S1_at_NA
MmugDNA.16301.1.S1_at	232966_at	LPIN3	lipin 3	chr20q12		64900	LPIN3	lipin 3	---		696329	MmugDNA.16301.1.S1_at_LPIN3
MmugDNA.1630.1.S1_at	233925_at	---	---	---	---		---	---	---	---		MmugDNA.1630.1.S1_at_---
MmugDNA.16302.1.S1_at	215476_at	---	---	---	---		---	---	---	---		MmugDNA.16302.1.S1_at_---
MmugDNA.16303.1.S1_at	226680_at	IKZF5	IKAROS family zinc finger 5 (Pegasus)	chr10q26		64376	---	---	---	---		MmugDNA.16303.1.S1_at_IKZF5
MmugDNA.16304.1.S1_at	236882_at	---	---	---	---		---	---	---	---		MmugDNA.16304.1.S1_at_---
MmugDNA.16305.1.S1_s_at	235438_at	---	---	---	---		---	---	---	---		MmugDNA.16305.1.S1_s_at_---
MmugDNA.16306.1.S1_at	237840_at	LOC388948	hypothetical protein LOC388948	chr2p21		388948	LOC715061	hypothetical protein LOC715061	---		715061	MmugDNA.16306.1.S1_at_LOC388948
MmugDNA.16307.1.S1_at	235531_at	---	---	---	---		---	---	---	---		MmugDNA.16307.1.S1_at_---
MmugDNA.16308.1.S1_at	244637_at	---	---	---	---		---	---	---	---		MmugDNA.16308.1.S1_at_---
MmugDNA.16309.1.S1_at	217036_at	---	---	---	---		---	---	---	---		MmugDNA.16309.1.S1_at_---
MmugDNA.16309.1.S1_x_at	NA	NA	NA	NA	NA		NA	NA	NA	NA		MmugDNA.16309.1.S1_x_at_NA
MmugDNA.16310.1.S1_at	243471_at	---	---	---	---		---	---	---	---		MmugDNA.16310.1.S1_at_---
MmugDNA.1631.1.S1_at	NA	NA	NA	NA	NA		NA	NA	NA	NA		MmugDNA.1631.1.S1_at_NA
MmugDNA.16312.1.S1_at	221615_at	BMP8A /// BMP8B	bone morphogenetic protein 8a /// bone morphogenetic protein 8b	chr1p34.2 /// chr1p35-p32	353500 /// 656		---	---	---	---		MmugDNA.16312.1.S1_at_BMP8A /// B
MmugDNA.16313.1.S1_at	240413_at	PYHIN1	pyrin and HIN domain family, member 1	chr1q23.1		149628	LOC719241	similar to pyrin and HIN domain family, member 1 beta 1 isoform	---		719241	MmugDNA.16313.1.S1_at_PYHIN1
MmugDNA.16314.1.S1_at	227335_at	DIDO1	death inducer-obliterator 1	chr20q13.33		11083	DIDO1	death inducer-obliterator 1	---		698313	MmugDNA.16314.1.S1_at_DIDO1
MmugDNA.16315.1.S1_at	228775_at	---	---	---	---		---	---	---	---		MmugDNA.16315.1.S1_at_---
MmugDNA.16316.1.S1_at	241462_at	---	---	---	---		---	---	---	---		MmugDNA.16316.1.S1_at_---
MmugDNA.16317.1.S1_at	244327_at	---	---	---	---		---	---	---	---		MmugDNA.16317.1.S1_at_---
MmugDNA.16318.1.S1_at	230981_at	CATSPER3	cation channel, sperm associated 3	chr5q31.1		347732	LOC713905	similar to cation channel, sperm associated 3	---		713905	MmugDNA.16318.1.S1_at_CATSPER3
MmugDNA.16319.1.S1_at	202124_s_at	TRAK2	trafficking protein, kinesin binding 2	chr2q33		66008	LOC697531	Similar to Importin alpha-2 subunit (Karyopherin alpha-2 subunit) (SRP1-alpha) (RAG cohort protein 1	---		697531	MmugDNA.16319.1.S1_at_TRAK2
MmugDNA.163.1.S1_at	224547_at	---	---	---	---		---	---	---	---		MmugDNA.163.1.S1_at_---
MmugDNA.16320.1.S1_at	221304_at	UGT1A10 /// UGT1A6 /	UDP glucuronosyltransferase 1 family, polypeptide A10 /// UDP glucuronosyltransferase 1 family, poly	chr2q37	54575 /// 54576 /// 545		LOC718141 /// UGT1A7 /	similar to UDP glycosyltransferase 1 family, polypeptide A9 precursor /// similar to UDP-glucuronosylt	---	714532 /// 718123 /// 7181		MmugDNA.16320.1.S1_at_UGT1A10 ///
MmugDNA.16321.1.S1_at	203995_at	C21orf2	chromosome 21 open reading frame 2	chr21q22.3		755	LOC722168	similar to Protein C21orf2 (C21orf-HUMF09G8.5) (YF5/A2)	---		722168	MmugDNA.16321.1.S1_at_C21orf2
MmugDNA.1632.1.S1_at	208305_at	PGR	progesterone receptor	chr11q22-q23		5241	PR	progesterone receptor	---		613024	MmugDNA.1632.1.S1_at_PGR
MmugDNA.16322.1.S1_at	236718_at	MYO10	myosin X	chr5p15.1-p14.3		4651	---	---	---	---		MmugDNA.16322.1.S1_at_MYO10
MmugDNA.16323.1.S1_at	211950_at	UBR4	ubiquitin protein ligase E3 component n-recognin 4	chr1p36.13		23352	LOC702449	similar to purity of essence CG14472-PA	---		702449	MmugDNA.16323.1.S1_at_UBR4
MmugDNA.16324.1.S1_at	227874_at	EMCN	endomucin	chr4q23		51705	LOC709580	similar to endomucin	---		709580	MmugDNA.16324.1.S1_at_EMCN
MmugDNA.16325.1.S1_at	235673_at	TAF1B	TATA box binding protein (TBP)-associated factor, RNA polymerase I, B, 63kDa	chr2p25		9014	---	---	---	---		MmugDNA.16325.1.S1_at_TAF1B
MmugDNA.16326.1.S1_at	203491_s_at	CEP57	centrosomal protein 57kDa	chr11q21		9702	CEP57	centrosomal protein 57kDa	---		699835	MmugDNA.16326.1.S1_at_CEP57
MmugDNA.16327.1.S1_at	205248_at	DOPEY2	dopey family member 2	chr21q22.2		9980	DOPEY2	dopey family member 2	---		695420	MmugDNA.16327.1.S1_at_DOPEY2
MmugDNA.16328.1.S1_at	NA	NA	NA	NA	NA		NA	NA	NA	NA		MmugDNA.16328.1.S1_at_NA
MmugDNA.16328.1.S1_s_at	203492_x_at	CEP57	centrosomal protein 57kDa	chr11q21		9702	CEP57	centrosomal protein 57kDa	---		699835	MmugDNA.16328.1.S1_s_at_CEP57
MmugDNA.16329.1.S1_at	NA	NA	NA	NA	NA		NA	NA	NA	NA		MmugDNA.16329.1.S1_at_NA
MmugDNA.16330.1.S1_at	43934_at	GPR137	G protein-coupled receptor 137	chr11cen-q22.3		56834	LOC717722	similar to expressed sequence AI428855	---		717722	MmugDNA.16330.1.S1_at_GPR137
MmugDNA.16331.1.S1_at	211349_at	SLC15A1	solute carrier family 15 (oligopeptide transporter), member 1	chr13q33-q34		6564	---	---	---	---		MmugDNA.16331.1.S1_at_SLC15A1
MmugDNA.1633.1.S1_at	230396_at	SAMM50	sorting and assembly machinery component 50 homolog (S. cerevisiae)	chr22q13.31		25813	---	---	---	---		MmugDNA.1633.1.S1_at_SAMM50
MmugDNA.16332.1.S1_at	221349_at	VPREB1	pre-B lymphocyte 1	chr22q11.2|22q11.22		7441	VPREB1	pre-B lymphocyte 1	---		698810	MmugDNA.16332.1.S1_at_VPREB1
MmugDNA.16333.1.S1_at	203493_s_at	CEP57	centrosomal protein 57kDa	chr11q21		9702	CEP57	centrosomal protein 57kDa	---		699835	MmugDNA.16333.1.S1_at_CEP57
MmugDNA.16334.1.S1_at	217898_at	C15orf24	chromosome 15 open reading frame 24	chr15q14		56851	LOC695440	similar to chromosome 15 open reading frame 24	---		695440	MmugDNA.16334.1.S1_at_C15orf24
MmugDNA.16335.1.S1_at	206106_at	MAPK12	mitogen-activated protein kinase 12	chr22q13.33		6300	PSME2	Similar to Proteasome activator complex subunit 2 (Proteasome activator 28-beta subunit) (PA28beta	---		701695	MmugDNA.16335.1.S1_at_MAPK12
MmugDNA.16336.1.S1_at	240923_at	---	---	---	---		---	---	---	---		MmugDNA.16336.1.S1_at_---
MmugDNA.16337.1.S1_at	220158_at	LGALS14	lectin, galactoside-binding, soluble, 14	chr19q13.2		56891	LGALS14	lectin, galactoside-binding, soluble, 14	---		698227	MmugDNA.16337.1.S1_at_LGALS14
MmugDNA.16338.1.S1_at	201950_x_at	CAPZB	capping protein (actin filament) muscle Z-line, beta	chr1p36.1		832	LOC723282	Similar to Zinc finger protein 432	---		723282	MmugDNA.16338.1.S1_at_CAPZB
MmugDNA.16339.1.S1_at	1569578_at	---	---	---	---		---	---	---	---		MmugDNA.16339.1.S1_at_---
MmugDNA.16340.1.S1_s_at	201866_s_at	NR3C1	nuclear receptor subfamily 3, group C, member 1 (glucocorticoid receptor)	chr5q31.3		2908	NR3C1	nuclear receptor subfamily 3, group C, member 1	---		706198	MmugDNA.16340.1.S1_s_at_NR3C1
MmugDNA.16341.1.S1_at	218015_s_at	WRNIP1	Werner helicase interacting protein 1	chr6p25.2		56897	---	---	---	---		MmugDNA.16341.1.S1_at_WRNIP1
MmugDNA.1634.1.S1_at	228692_at	---	---	---	---		---	---	---	---		MmugDNA.1634.1.S1_at_---
MmugDNA.16342.1.S1_at	240422_at	FMO5	Flavin containing monooxygenase 5	chr1q21.1		2330	FMO5	flavin containing monooxygenase 5	---		709691	MmugDNA.16342.1.S1_at_FMO5
MmugDNA.16343.1.S1_at	213242_x_at	KIAA0284	KIAA0284	chr14q32.33		283638	---	---	---	---		MmugDNA.16343.1.S1_at_KIAA0284
MmugDNA.16344.1.S1_at	1561854_at	---	---	---	---		---	---	---	---		MmugDNA.16344.1.S1_at_---
MmugDNA.16345.1.S1_at	NA	NA	NA	NA	NA		NA	NA	NA	NA		MmugDNA.16345.1.S1_at_NA
MmugDNA.16345.1.S1_s_at	1555881_s_at	LZTS2	leucine zipper, putative tumor suppressor 2	chr10q24		84445	LOC711279	similar to leucine zipper, putative tumor suppressor 2	---		711279	MmugDNA.16345.1.S1_s_at_LZTS2
MmugDNA.16346.1.S1_at	222849_s_at	SCRN3	secernin 3	chr2q31.1		79634	SCRN3	secernin 3	---		698199	MmugDNA.16346.1.S1_at_SCRN3
MmugDNA.16347.1.S1_at	208465_at	GRM2	glutamate receptor, metabotropic 2	chr3p21.1		2912	LOC702244	Similar to ATPase family, AAA domain containing 1	---		702244	MmugDNA.16347.1.S1_at_GRM2

MmugDNA.16348.1.S1_at	1561673_at	---	---	---	---	---	---	---	---	MmugDNA.16348.1.S1_at_---
MmugDNA.16349.1.S1_at	205814_at	GRM3	glutamate receptor, metabotropic 3	chr7q21.1-q21.2	2913	LOC722846	Similar to F-box and leucine-rich repeat protein 5 isoform 1	---	722846	MmugDNA.16349.1.S1_at_GRM3
MmugDNA.16350.1.S1_at	235629_at	---	---	---	---	---	---	---	---	MmugDNA.16350.1.S1_at_---
MmugDNA.16351.1.S1_at	209286_at	CDC42EP3	CDC42 effector protein (Rho GTPase binding) 3	chr2p21	10602	---	---	---	---	MmugDNA.16351.1.S1_at_CDC42EP3
MmugDNA.1635.1.S1_at	241781_at	C9orf41	chromosome 9 open reading frame 41	chr9q21.13	138199	C15H9orf41	chromosome 9 open reading frame 41 ortholog	---	706229	MmugDNA.1635.1.S1_at_C9orf41
MmugDNA.16352.1.S1_at	1556821_x_at	DLEU2	deleted in lymphocytic leukemia 2 (non-protein coding)	chr13q14.3	8847	---	---	---	---	MmugDNA.16352.1.S1_at_DLEU2
MmugDNA.16353.1.S1_at	1554827_a_at	ADCY7	adenylate cyclase 7	chr16q12-q13	113	ADCY7	adenylate cyclase 7	---	694730	MmugDNA.16353.1.S1_at_ADCY7
MmugDNA.16354.1.S1_at	1564907_s_at	MATR3 /// SNHG4	matrin 3 /// small nucleolar RNA host gene 4 (non-protein coding)	chr5q31.2	724102 /// 9782	MATR3	matrin 3	---	714812	MmugDNA.16354.1.S1_at_MATR3 /// SI
MmugDNA.16355.1.S1_at	244863_at	---	---	---	---	---	---	---	---	MmugDNA.16355.1.S1_at_---
MmugDNA.16356.1.S1_at	223474_at	C14orf4	chromosome 14 open reading frame 4	chr14q24.3	64207	---	---	---	---	MmugDNA.16356.1.S1_at_C14orf4
MmugDNA.16357.1.S1_at	210181_s_at	CABP1	calcium binding protein 1	chr12q24.31	9478	LOC697825	similar to calcium binding protein 1 isoform 3	---	697825	MmugDNA.16357.1.S1_at_CABP1
MmugDNA.16357.1.S1_s_at	208321_s_at	CABP1	calcium binding protein 1	chr12q24.31	9478	LOC697825	similar to calcium binding protein 1 isoform 3	---	697825	MmugDNA.16357.1.S1_s_at_CABP1
MmugDNA.16358.1.S1_at	241653_x_at	---	---	---	---	---	---	---	---	MmugDNA.16358.1.S1_at_---
MmugDNA.16359.1.S1_at	210762_s_at	DLC1	deleted in liver cancer 1	chr8p22	10395	DLC1	deleted in liver cancer 1	---	700630	MmugDNA.16359.1.S1_at_DLC1
MmugDNA.16360.1.S1_at	236626_at	ALG1	asparagine-linked glycosylation 1, beta-1,4-mannosyltransferase homolog (S. cerevisiae)	chr16p13.3	56052	---	---	---	---	MmugDNA.16360.1.S1_at_ALG1
MmugDNA.16361.1.S1_at	229417_at	---	---	---	---	STAU2	staufen, RNA binding protein, homolog 2 (Drosophila)	---	696034	MmugDNA.16361.1.S1_at_---
MmugDNA.1636.1.S1_at	1570155_at	---	---	---	---	---	---	---	---	MmugDNA.1636.1.S1_at_---
MmugDNA.16362.1.S1_at	207007_at	NR1I3	nuclear receptor subfamily 1, group I, member 3	chr1q23.3	9970	CAR	constitutive androstane receptor	---	574249	MmugDNA.16362.1.S1_at_NR1I3
MmugDNA.16363.1.S1_at	218813_s_at	SH3GLB2	SH3-domain GRB2-like endophilin B2	chr9q34	56904	SH3GLB2	SH3-domain GRB2-like endophilin B2	---	716994	MmugDNA.16363.1.S1_at_SH3GLB2
MmugDNA.16364.1.S1_at	207625_s_at	CBFA2T2	core-binding factor, runt domain, alpha subunit 2; translocated to, 2	chr20q11	9139	LOC709738	hypothetical protein LOC709738	---	709738	MmugDNA.16364.1.S1_at_CBFA2T2
MmugDNA.16365.1.S1_at	229678_at	LOC728431	Hypothetical LOC728431	chr1p34.3	728431	---	---	---	---	MmugDNA.16365.1.S1_at_LOC728431
MmugDNA.16366.1.S1_at	219596_at	THAP10	THAP domain containing 10	chr15q23	56906	LOC705360	Hypothetical protein LOC705360	---	705360	MmugDNA.16366.1.S1_at_THAP10
MmugDNA.16367.1.S1_at	207979_s_at	CD8B	CD8b molecule	chr2p12	926	CD8B	similar to T-cell surface glycoprotein CD8 beta chain precursor (CD8b antigen)	---	699409	MmugDNA.16367.1.S1_at_CD8B
MmugDNA.16368.1.S1_at	206327_s_at	CDH15	cadherin 15, type 1, M-cadherin (myotubule)	chr16q24.3	1013	CDH15	cadherin 15	---	712526	MmugDNA.16368.1.S1_at_CDH15
MmugDNA.16369.1.S1_at	218483_s_at	C11orf60	chromosome 11 open reading frame 60	chr11q23.3	56912	LOC700582	similar to F46F6.4	---	700582	MmugDNA.16369.1.S1_at_C11orf60
MmugDNA.16370.1.S1_at	228113_at	RAB37	RAB37, member RAS oncogene family	chr17q25.1	326624	RAB37	RAB37, member RAS oncogene family	---	698842	MmugDNA.16370.1.S1_at_RAB37
MmugDNA.16371.1.S1_at	207235_s_at	GRM5	glutamate receptor, metabotropic 5	chr11q14.2-q14.3	2915	---	---	---	---	MmugDNA.16371.1.S1_at_GRM5
MmugDNA.1637.1.S1_at	244218_at	---	---	---	---	---	---	---	---	MmugDNA.1637.1.S1_at_---
MmugDNA.16372.1.S1_at	218481_at	EXOSC5	exosome component 5	chr19q13.1	56915	EXOSC5	exosome component 5	---	705203	MmugDNA.16372.1.S1_at_EXOSC5
MmugDNA.16373.1.S1_at	216131_at	FRMD4B	FERM domain containing 4B	chr3p14.1	23150	---	---	---	---	MmugDNA.16373.1.S1_at_FRMD4B
MmugDNA.16374.1.S1_at	1568706_s_at	AVIL	Advillin	chr12q14.1	10677	---	---	---	---	MmugDNA.16374.1.S1_at_AVIL
MmugDNA.16375.1.S1_at	216765_at	MAP2K5	Mitogen-activated protein kinase kinase 5	chr15q23	5607	---	---	---	---	MmugDNA.16375.1.S1_at_MAP2K5
MmugDNA.16376.1.S1_at	210172_at	SF1	splicing factor 1	chr11q13	7536	SF1	splicing factor 1	---	717428	MmugDNA.16376.1.S1_at_SF1
MmugDNA.16377.1.S1_at	204860_s_at	NAIP	NLR family, apoptosis inhibitory protein	chr5q13.1	4671	NAIP	NLR family, apoptosis inhibitory protein	---	705157	MmugDNA.16377.1.S1_at_NAIP
MmugDNA.16378.1.S1_s_at	1569138_a_at	SRCAP	Snf2-related CREBBP activator protein	chr16p11.2	10847	LOC708729	similar to DnaJ (Hsp40) homolog, subfamily C, member 7	---	708729	MmugDNA.16378.1.S1_s_at_SRCAP
MmugDNA.16379.1.S1_at	1570621_at	---	---	---	---	---	---	---	---	MmugDNA.16379.1.S1_at_---
MmugDNA.16380.1.S1_at	1562017_at	LOC100130654	hypothetical protein LOC100130654	chr17q21.2	100130654	---	---	---	---	MmugDNA.16380.1.S1_at_LOC100130(
MmugDNA.16381.1.S1_at	213322_at	C6orf130	chromosome 6 open reading frame 130	chr6p21.1	221443	LOC719685	similar to Protein C6orf130 homolog	---	719685	MmugDNA.16381.1.S1_at_C6orf130
MmugDNA.1638.1.S1_at	229842_at	ELF3	E74-like factor 3 (ets domain transcription factor, epithelial-specific)	chr1q32.2	1999	---	---	---	---	MmugDNA.1638.1.S1_at_ELF3
MmugDNA.16382.1.S1_at	1563532_at	HMCN2	hemicentin 2	chr9q34.11	256158	---	---	---	---	MmugDNA.16382.1.S1_at_HMCN2
MmugDNA.16383.1.S1_at	1554946_at	---	---	---	---	---	---	---	---	MmugDNA.16383.1.S1_at_---
MmugDNA.16384.1.S1_at	1553702_at	ZNF697	zinc finger protein 697	chr1p12	90874	LOC715582	similar to zinc finger protein 697	---	715582	MmugDNA.16384.1.S1_at_ZNF697
MmugDNA.16385.1.S1_at	1557719_at	PIKFYVE	phosphoinositide kinase, FYVE finger containing	chr2q33.3	200576	LOC710115	similar to phosphatidylinositol-3-phosphate/phosphatidylinositol 5-kinase, type III isoform 2	---	710115	MmugDNA.16385.1.S1_at_PIKFYVE
MmugDNA.16386.1.S1_at	1569141_a_at	PPARGC1A	peroxisome proliferator-activated receptor gamma, coactivator 1 alpha	chr4p15.1	10891	---	---	---	---	MmugDNA.16386.1.S1_at_PPARGC1A
MmugDNA.16387.1.S1_at	219689_at	SEMA3G	sema domain, immunoglobulin domain (Ig), short basic domain, secreted, (semaphorin) 3G	chr3p21.1	56920	---	---	---	---	MmugDNA.16387.1.S1_at_SEMA3G
MmugDNA.16388.1.S1_at	1560199_x_at	LOC728153	similar to FAM133B protein	chr5q12.1	728153	---	---	---	---	MmugDNA.16388.1.S1_at_LOC728153
MmugDNA.16389.1.S1_at	207284_s_at	ASPH	aspartate beta-hydroxylase	chr8q12.1	444	LOC699884	similar to aspartate beta-hydroxylase isoform b	---	699884	MmugDNA.16389.1.S1_at_ASPH
MmugDNA.16390.1.S1_at	204247_s_at	CDK5	cyclin-dependent kinase 5	chr7q36	1020	RTN1	Reticulon 1	---	619184	MmugDNA.16390.1.S1_at_CDK5
MmugDNA.16391.1.S1_at	1569073_x_at	SMARCA4	SWI/SNF related, matrix associated, actin dependent regulator of chromatin, subfamily a, member 4	chr19p13.2	6597	---	---	---	---	MmugDNA.16391.1.S1_at_SMARCA4
MmugDNA.1639.1.S1_at	203945_at	ARG2	arginase, type II	chr14q24.1-q24.3	384	LILRBC	Leukocyte immunoglobulin-like receptor, subfamily B, member c	---	692336	MmugDNA.1639.1.S1_at_ARG2
MmugDNA.16392.1.S1_at	1559634_at	CHRM3	cholinergic receptor, muscarinic 3	chr1q43	1131	---	---	---	---	MmugDNA.16392.1.S1_at_CHRM3
MmugDNA.16393.1.S1_at	235948_at	RIMKLA	ribosomal modification protein rimK-like family member A	chr1p34.2	284716	LOC699767	similar to family with sequence similarity 80, member A	---	699767	MmugDNA.16393.1.S1_at_RIMKLA
MmugDNA.16394.1.S1_s_at	217095_x_at	NCR1	natural cytotoxicity triggering receptor 1	chr19q13.42	9437	---	---	---	---	MmugDNA.16394.1.S1_s_at_NCR1
MmugDNA.16395.1.S1_at	220889_s_at	CA10	carbonic anhydrase X	chr17q21.33	56934	CA10	carbonic anhydrase X	---	704971	MmugDNA.16395.1.S1_at_CA10
MmugDNA.16396.1.S1_at	204059_s_at	ME1	malic enzyme 1, NADP(+)-dependent, cytosolic	chr6q12	4199	ME1	malic enzyme 1, NADP(+)-dependent, cytosolic	---	694739	MmugDNA.16396.1.S1_at_ME1
MmugDNA.16397.1.S1_s_at	216041_x_at	GRN	granulin	chr17q21.32	2896	LOC700880	Similar to Serine/threonine-protein kinase tousled-like 2 (Tousled-like kinase 2)	---	700880	MmugDNA.16397.1.S1_s_at_GRN
MmugDNA.16398.1.S1_at	220658_s_at	ARNTL2	aryl hydrocarbon receptor nuclear translocator-like 2	chr12p12.2-p11.2	56938	LOC709547	similar to aryl hydrocarbon receptor nuclear translocator-like 2	---	709547	MmugDNA.16398.1.S1_at_ARNTL2
MmugDNA.16399.1.S1_s_at	219767_s_at	CRYZL1	crystallin, zeta (quinone reductase)-like 1	chr21q21.3	9946	CRYZL1	crystallin, zeta (quinone reductase)-like 1	---	698723	MmugDNA.16399.1.S1_s_at_CRYZL1
MmugDNA.16400.1.S1_at	205579_at	HRH1	histamine receptor H1	chr3p25	3269	HRH1	histamine receptor H1	---	696359	MmugDNA.16400.1.S1_at_HRH1
MmugDNA.1640.1.S1_at	235427_at	---	---	---	---	---	---	---	---	MmugDNA.1640.1.S1_at_---
MmugDNA.16402.1.S1_at	218900_at	CNNM4	cyclin M4	chr2q11	26504	LOC710164	similar to cyclin M4	---	710164	MmugDNA.16402.1.S1_at_CNNM4
MmugDNA.16403.1.S1_at	1555286_at	CDKL1	cyclin-dependent kinase-like 1 (CDC2-related kinase)	chr14q22.1	8814	CDKL1	cyclin-dependent kinase-like 1 (CDC2-related kinase)	---	705975	MmugDNA.16403.1.S1_at_CDKL1
MmugDNA.16404.1.S1_at	229372_at	GOLT1A	golgi transport 1 homolog A (S. cerevisiae)	chr1q32.1	127845	LOC700305	similar to golgi transport 1 homolog A	---	700305	MmugDNA.16404.1.S1_at_GOLT1A
MmugDNA.16405.1.S1_a_at	NA	NA	NA	NA	NA	NA	NA	NA	NA	MmugDNA.16405.1.S1_a_at_NA
MmugDNA.16405.1.S1_at	229709_at	ATP1B3	ATPase, Na+/K+ transporting, beta 3 polypeptide	chr3q23	483	---	---	---	---	MmugDNA.16405.1.S1_at_ATP1B3
MmugDNA.16406.1.S1_at	244800_x_at	---	---	---	---	---	---	---	---	MmugDNA.16406.1.S1_at_---
MmugDNA.16407.1.S1_at	206326_at	GRP	gastrin-releasing peptide	chr18q21.1-q21.32	2922	LOC696983	similar to gastrin-releasing peptide isoform 3 preproprotein	---	696983	MmugDNA.16407.1.S1_at_GRP
MmugDNA.16408.1.S1_at	1557782_s_at	C21orf49	chromosome 21 open reading frame 49	chr21q22.11	54067	---	---	---	---	MmugDNA.16408.1.S1_at_C21orf49
MmugDNA.16409.1.S1_at	1558076_at	ANKRD32	ankyrin repeat domain 32	chr5q15	84250	NFKBIL2	similar to ankyrin repeat domain 32	---	701994	MmugDNA.16409.1.S1_at_ANKRD32
MmugDNA.16410.1.S1_at	239215_at	---	---	---	---	---	---	---	---	MmugDNA.16410.1.S1_at_---
MmugDNA.16411.1.S1_at	222897_s_at	ZFP64	zinc finger protein 64 homolog (mouse)	chr20q13.2	55734	LOC700183	similar to Zinc finger protein 64, isoforms 3 and 4 (Zinc finger protein 338)	---	700183	MmugDNA.16411.1.S1_at_ZFP64
MmugDNA.1641.1.S1_at	1570624_at	---	---	---	---	---	---	---	---	MmugDNA.1641.1.S1_at_---
MmugDNA.16412.1.S1_at	231425_at	PLCZ1	phospholipase C, zeta 1	chr12p12.3	89869	SUMO2	SMT3 suppressor of mif two 3 homolog 2 (S. cerevisiae)	---	711990	MmugDNA.16412.1.S1_at_PLCZ1
MmugDNA.16413.1.S1_at	240319_at	---	---	---	---	---	---	---	---	MmugDNA.16413.1.S1_at_---
MmugDNA.16414.1.S1_at	233750_s_at	C1orf25	chromosome 1 open reading frame 25	chr1q25.2	81627	LOC714319	similar to N2,N2-dimethylguanosine tRNA methyltransferase-like	---	714319	MmugDNA.16414.1.S1_at_C1orf25
MmugDNA.16416.1.S1_at	236582_at	---	---	---	---	---	---	---	---	MmugDNA.16416.1.S1_at_---
MmugDNA.16417.1.S1_at	229257_at	TNRC18	trinucleotide repeat containing 18	chr7p22.1	84629	---	---	---	---	MmugDNA.16417.1.S1_at_TNRC18
MmugDNA.16418.1.S1_at	1562220_at	---	---	---	---	---	---	---	---	MmugDNA.16418.1.S1_at_---
MmugDNA.16419.1.S1_at	1554869_at	WDR37	WD repeat domain 37	chr10p15.3	22884	WDR37	WD repeat domain 37	---	709975	MmugDNA.16419.1.S1_at_WDR37
MmugDNA.164.1.S1_at	235804_at	---	---	---	---	---	---	---	---	MmugDNA.164.1.S1_at_---
MmugDNA.16420.1.S1_at	201501_s_at	GRSF1	G-rich RNA sequence binding factor 1	chr4q13	2926	GRSF1	G-rich RNA sequence binding factor 1	---	706301	MmugDNA.16420.1.S1_at_GRSF1
MmugDNA.16421.1.S1_s_at	NA	NA	NA	NA	NA	NA	NA	NA	NA	MmugDNA.16421.1.S1_s_at_NA
MmugDNA.1642.1.S1_at	237270_at	---	---	---	---	---	---	---	---	MmugDNA.1642.1.S1_at_---
MmugDNA.16422.1.S1_at	216949_s_at	PKD1	polycystic kidney disease 1 (autosomal dominant)	chr16p13.3	5310	PKD1	polycystic kidney disease 1 (autosomal dominant)	---	694905	MmugDNA.16422.1.S1_at_PKD1
MmugDNA.16423.1.S1_at	NA	NA	NA	NA	NA	NA	NA	NA	NA	MmugDNA.16423.1.S1_at_NA
MmugDNA.16424.1.S1_at	200696_s_at	GSN	gelsolin (amyloidosis, Finnish type)	chr9q33	2934	GSN	gelsolin	---	699705	MmugDNA.16424.1.S1_at_GSN
MmugDNA.16425.1.S1_at	243405_at	---	---	---	---	---	---	---	---	MmugDNA.16425.1.S1_at_---
MmugDNA.16426.1.S1_at	201415_at	GSS	glutathione synthetase	chr20q11.2	2937	LOC697879	Similar to 60S ribosomal protein L32	---	697879	MmugDNA.16426.1.S1_at_GSS
MmugDNA.16427.1.S1_at	NA	NA	NA	NA	NA	NA	NA	NA	NA	MmugDNA.16427.1.S1_at_NA
MmugDNA.16428.1.S1_at	240353_s_at	C12orf54	chromosome 12 open reading frame 54	chr12q13.11	121273	---	---	---	---	MmugDNA.16428.1.S1_at_C12orf54
MmugDNA.16429.1.S1_at	239978_at	---	---	---	---	---	---	---	---	MmugDNA.16429.1.S1_at_---
MmugDNA.16430.1.S1_at	222347_at	LOC644450	hypothetical protein LOC644450	chr1q12	644450	---	---	---	---	MmugDNA.16430.1.S1_at_LOC644450
MmugDNA.16430.1.S1_s_at	215625_at	LOC644450	hypothetical protein LOC644450	chr1q12	644450	---	---	---	---	MmugDNA.16430.1.S1_s_at_LOC6444!
MmugDNA.16431.1.S1_at	212032_s_at	PTOV1	prostate tumor overexpressed 1	chr19q13.33	53635	LOC719449	similar to prostate tumor overexpressed gene 1	---	719449	MmugDNA.16431.1.S1_at_PTOV1
MmugDNA.1643.1.S1_at	226717_at	LRTOMT	leucine rich transmembrane and 0-methyltransferase domain containing	chr11q13.4	220074	LRCC51	leucine rich repeat containing 51	---	718286	MmugDNA.1643.1.S1_at_LRTOMT
MmugDNA.16432.1.S1_at	228230_at	PRIC285	peroxisomal proliferator-activated receptor A interacting complex 285	chr20q13.33	85441	DHX8	DEAH (Asp-Glu-Ala-His) box polypeptide 8	---	713228	MmugDNA.16432.1.S1_at_PRIC285
MmugDNA.16433.1.S1_at	1563559_at	FMN1	formin 1	chr15q13.3	342184	---	---	---	---	MmugDNA.16433.1.S1_at_FMN1
MmugDNA.16434.1.S1_at	215758_x_at	ZNF93	zinc finger protein 93	chr19p12	81931	---	---	---	---	MmugDNA.16434.1.S1_at_ZNF93
MmugDNA.16435.1.S1_at	236211_at	---	---	---	---	---	---	---	---	MmugDNA.16435.1.S1_at_---
MmugDNA.16436.1.S1_at	1561059_a_at	LOC152024	hypothetical LOC152024	chr3p24.2	152024	---	---	---	---	MmugDNA.16436.1.S1_at_LOC152024
MmugDNA.16437.1.S1_at	1557542_at	---	---	---	---	---	---	---	---	MmugDNA.16437.1.S1_at_---
MmugDNA.16438.1.S1_at	225377_at	C9orf86	chromosome 9 open reading frame 86	chr9q34.3	55684	LOC709111	similar to F08G12.1	---	709111	MmugDNA.16438.1.S1_at_C9orf86
MmugDNA.16439.1.S1_at	240789_at	---	---	---	---	---	---	---	---	MmugDNA.16439.1.S1_at_---
MmugDNA.16440.1.S1_at	1561202_at	---	---	---	---	---	---	---	---	MmugDNA.16440.1.S1_at_---
MmugDNA.16441.1.S1_at	237686_at	RNF219	Ring finger protein 219	chr13q31.1	79596	---	---	---	---	MmugDNA.16441.1.S1_at_RNF219
MmugDNA.1644.1.S1_at	214313_s_at	EIF5B	Eukaryotic translation initiation factor 5B	chr2q11.2	9669	EIF5B	eukaryotic translation initiation factor 5B	---	714129	MmugDNA.1644.1.S1_at_EIF5B
MmugDNA.16442.1.S1_at	1562659_at	LOC400548	hypothetical gene supported by BC040918	chr16q24.1	400548	---	---	---	---	MmugDNA.16442.1.S1_at_LOC400548
MmugDNA.16443.1.S1_at	217960_s_at	TOMM22	translocase of outer mitochondrial membrane 22 homolog (yeast)	chr22q12-q13	56993	LOC693766	similar to Mitochondrial import receptor subunit TOM22 homolog (Translocase of outer membrane 22	---	693766	MmugDNA.16443.1.S1_at_TOMM22

MmugDNA.16444.1.S1_at	242014_at	---	---	---	---		---	---	---	---		MmugDNA.16444.1.S1_at_---
MmugDNA.16445.1.S1_at	1562100_at	---	---	---	---		---	---	---	---		MmugDNA.16445.1.S1_at_---
MmugDNA.16446.1.S1_at	244053_at	---	---	---	---		---	---	---	---		MmugDNA.16446.1.S1_at_---
MmugDNA.16447.1.S1_at	220371_s_at	SLC12A9	solute carrier family 12 (potassium/chloride transporters), member 9	chr7q22		56996	LOC713119	similar to solute carrier family 12 (potassium/chloride transporters), member 9	---		713119	MmugDNA.16447.1.S1_at_SLC12A9
MmugDNA.16448.1.S1_at	205554_s_at	DNASE1L3	deoxyribonuclease I-like 3	chr3p21.1-p14.3		1776	DNASE1L3	deoxyribonuclease I-like 3	---		702613	MmugDNA.16448.1.S1_at_DNASE1L3
MmugDNA.16449.1.S1_at	218168_s_at	CABC1	chaperone, ABC1 activity of bc1 complex homolog (S. pombe)	chr1q42.13		56997	CABC1	chaperone, ABC1 activity of bc1 complex homolog (S. pombe)	---		697920	MmugDNA.16449.1.S1_at_CABC1
MmugDNA.16450.1.S1_at	202253_s_at	DNM2	dynamin 2	chr19p13.2		1785	DNM2	dynamin 2	---		713558	MmugDNA.16450.1.S1_at_DNM2
MmugDNA.16451.1.S1_s_at	201210_at	DDX3X	DEAD (Asp-Glu-Ala-Asp) box polypeptide 3, X-linked	chrXp11.3-p11.23		1654	---	---	---	---		MmugDNA.16451.1.S1_s_at_DDX3X
MmugDNA.1645.1.S1_at	204288_s_at	SORBS2	sorbin and SH3 domain containing 2	chr4q35.1		8470	SORBS2	sorbin and SH3 domain containing 2	---		696139	MmugDNA.1645.1.S1_at_SORBS2
MmugDNA.16452.1.S1_s_at	NA	NA	NA	NA	NA		NA	NA	NA	NA		MmugDNA.16452.1.S1_s_at_NA
MmugDNA.16453.1.S1_at	NA	NA	NA	NA	NA		NA	NA	NA	NA		MmugDNA.16453.1.S1_at_NA
MmugDNA.16453.1.S1_s_at	NA	NA	NA	NA	NA		NA	NA	NA	NA		MmugDNA.16453.1.S1_s_at_NA
MmugDNA.16454.1.S1_s_at	NA	NA	NA	NA	NA		NA	NA	NA	NA		MmugDNA.16454.1.S1_s_at_NA
MmugDNA.16455.1.S1_at	236868_at	---	---	---	---		LOC700542	hypothetical protein LOC700542	---		700542	MmugDNA.16455.1.S1_at_---
MmugDNA.16456.1.S1_at	NA	NA	NA	NA	NA		NA	NA	NA	NA		MmugDNA.16456.1.S1_at_NA
MmugDNA.16457.1.S1_at	1562010_x_at	---	---	---	---		---	---	---	---		MmugDNA.16457.1.S1_at_---
MmugDNA.16458.1.S1_at	213155_at	WSCD1	WSC domain containing 1	chr17p13.2		23302	LOC712488	similar to CG9164-PA, isoform A	---		712488	MmugDNA.16458.1.S1_at_WSCD1
MmugDNA.16459.1.S1_at	221502_at	KPNA3	karyopherin alpha 3 (importin alpha 4)	chr13q14.3		3839	LOC711922	Similar to hexokinase 1 isoform HKI-R	---		711922	MmugDNA.16459.1.S1_at_KPNA3
MmugDNA.16460.1.S1_at	239607_at	GPR156	G protein-coupled receptor 156	chr3q13.33		165829	---	---	---	---		MmugDNA.16460.1.S1_at_GPR156
MmugDNA.16461.1.S1_at	231263_at	C6orf81	chromosome 6 open reading frame 81	chr6p21.31		221481	LOC718882	hypothetical protein LOC718882	---		718882	MmugDNA.16461.1.S1_at_C6orf81
MmugDNA.1646.1.S1_at	216599_x_at	SLC22A6	solute carrier family 22 (organic anion transporter), member 6	chr11q13.1-q13.2		9356	LOC712100	Similar to transcription factor-like nuclear regulator	---		712100	MmugDNA.1646.1.S1_at_SLC22A6
MmugDNA.16462.1.S1_at	216017_s_at	NAB2	NGFI-A binding protein 2 (EGR1 binding protein 2)	chr12q13.3-q14.1		4665	---	---	---	---		MmugDNA.16462.1.S1_at_NAB2
MmugDNA.16463.1.S1_at	233250_x_at	FOXRED2	FAD-dependent oxidoreductase domain containing 2	chr22q12.3		80020	LOC703292	hypothetical protein LOC703292	---		703292	MmugDNA.16463.1.S1_at_FOXRED2
MmugDNA.16464.1.S1_at	226387_at	RSBN1L	round spermatid basic protein 1-like	chr7q11.23		222194	RSBN1L	round spermatid basic protein 1-like	---		693814	MmugDNA.16464.1.S1_at_RSBN1L
MmugDNA.16465.1.S1_at	233619_at	---	---	---	---		---	---	---	---		MmugDNA.16465.1.S1_at_---
MmugDNA.16466.1.S1_at	231269_at	ASCC3	activating signal cointegrator 1 complex subunit 3	chr6q16.1-q16.3		10973	---	---	---	---		MmugDNA.16466.1.S1_at_ASCC3
MmugDNA.16467.1.S1_at	216715_at	---	---	---	---		LOC715538	similar to eukaryotic translation elongation factor 1 delta isoform 1	---		715538	MmugDNA.16467.1.S1_at_---
MmugDNA.16468.1.S1_at	1554986_a_at	SNX19	sorting nexin 19	chr11q25		399979	SNX19	sorting nexin 19	---		715704	MmugDNA.16468.1.S1_at_SNX19
MmugDNA.16469.1.S1_at	219693_at	AGPAT4	1-acylglycerol-3-phosphate O-acyltransferase 4 (lysophosphatidic acid acyltransferase, delta)	chr6q26		56895	AGPAT4	1-acylglycerol-3-phosphate O-acyltransferase 4	---		704132	MmugDNA.16469.1.S1_at_AGPAT4
MmugDNA.16470.1.S1_at	239630_at	---	---	---	---		---	---	---	---		MmugDNA.16470.1.S1_at_---
MmugDNA.16471.1.S1_at	NA	NA	NA	NA	NA		NA	NA	NA	NA		MmugDNA.16471.1.S1_at_NA
MmugDNA.1647.1.S1_at	243907_at	---	---	---	---		---	---	---	---		MmugDNA.1647.1.S1_at_---
MmugDNA.16473.1.S1_at	237696_at	---	---	---	---		---	---	---	---		MmugDNA.16473.1.S1_at_---
MmugDNA.16474.1.S1_at	NA	NA	NA	NA	NA		NA	NA	NA	NA		MmugDNA.16474.1.S1_at_NA
MmugDNA.16474.1.S1_s_at	218729_at	LXN	latexin	chr3q25.32		56925	LXN	latexin	---		703996	MmugDNA.16474.1.S1_s_at_LXN
MmugDNA.16475.1.S1_at	214464_at	CDC42BPA	CDC42 binding protein kinase alpha (DMPK-like)	chr1q42.11		8476	CDC42BPA	CDC42 binding protein kinase alpha (DMPK-like)	---		697811	MmugDNA.16475.1.S1_at_CDC42BPA
MmugDNA.16476.1.S1_at	227575_s_at	C14orf102	chromosome 14 open reading frame 102	chr14q32.11		55051	C7H14orf102	chromosome 14 open reading frame 102 ortholog	---		695676	MmugDNA.16476.1.S1_at_C14orf102
MmugDNA.16477.1.S1_at	205752_s_at	GSTM5	glutathione S-transferase mu 5	chr1p13.3		2949	GZMA	Granzyme A (granzyme 1, cytotoxic T-lymphocyte-associated serine esterase 3)	---		705712	MmugDNA.16477.1.S1_at_GSTM5
MmugDNA.16478.1.S1_at	200033_at	DDX5	DEAD (Asp-Glu-Ala-Asp) box polypeptide 5	chr17q21		1655	LOC705787	Similar to DnaJ (Hsp40) homolog, subfamily C, member 10	---		705787	MmugDNA.16478.1.S1_at_DDX5
MmugDNA.16479.1.S1_at	237792_at	---	---	---	---		---	---	---	---		MmugDNA.16479.1.S1_at_---
MmugDNA.16480.1.S1_at	232014_at	ZNF30	zinc finger protein 30	chr19q13.11		90075	LOC718310	similar to zinc finger protein 30	---		718310	MmugDNA.16480.1.S1_at_ZNF30
MmugDNA.16481.1.S1_at	1556762_a_at	---	---	---	---		---	---	---	---		MmugDNA.16481.1.S1_at_---
MmugDNA.1648.1.S1_at	226705_at	FGFR1	fibroblast growth factor receptor 1	chr8p11.2-p11.1		2260	---	---	---	---		MmugDNA.1648.1.S1_at_FGFR1
MmugDNA.16482.1.S1_at	212974_at	DENND3	DENN/MADD domain containing 3	chr8q24.3		22898	DENND3	DENN/MADD domain containing 3	---		693759	MmugDNA.16482.1.S1_at_DENND3
MmugDNA.16483.1.S1_at	NA	NA	NA	NA	NA		NA	NA	NA	NA		MmugDNA.16483.1.S1_at_NA
MmugDNA.16483.1.S1_s_at	242661_x_at	ALS2CR12	amyotrophic lateral sclerosis 2 (juvenile) chromosome region, candidate 12	chr2q33.1		130540	CTAGE5	CTAGE family, member 5	---		699832	MmugDNA.16483.1.S1_s_at_ALS2CR1
MmugDNA.16484.1.S1_at	212975_at	DENND3	DENN/MADD domain containing 3	chr8q24.3		22898	DENND3	DENN/MADD domain containing 3	---		693759	MmugDNA.16484.1.S1_at_DENND3
MmugDNA.16485.1.S1_at	1569181_x_at	---	---	---	---		---	---	---	---		MmugDNA.16485.1.S1_at_---
MmugDNA.16486.1.S1_at	228122_at	CCDC66	coiled-coil domain containing 66	chr3p14.3		285331	LOC708564	hypothetical protein LOC708564	---		708564	MmugDNA.16486.1.S1_at_CCDC66
MmugDNA.16487.1.S1_at	234660_s_at	DIS3	DIS3 mitotic control homolog (S. cerevisiae)	chr13q22.1		22894	DIS3	DIS3 mitotic control homolog (S. cerevisiae)	---		694048	MmugDNA.16487.1.S1_at_DIS3
MmugDNA.16488.1.S1_at	NA	NA	NA	NA	NA		NA	NA	NA	NA		MmugDNA.16488.1.S1_at_NA
MmugDNA.16488.1.S1_s_at	227278_at	---	---	---	---		---	---	---	---		MmugDNA.16488.1.S1_s_at_---
MmugDNA.16489.1.S1_at	238829_at	---	---	---	---		---	---	---	---		MmugDNA.16489.1.S1_at_---
MmugDNA.16490.1.S1_at	NA	NA	NA	NA	NA		NA	NA	NA	NA		MmugDNA.16490.1.S1_at_NA
MmugDNA.16491.1.S1_at	1556086_at	C4orf27	chromosome 4 open reading frame 27	chr4q33		54969	C5H4orf27	chromosome 4 open reading frame 27 ortholog	---		694607	MmugDNA.16491.1.S1_at_C4orf27
MmugDNA.1649.1.S1_at	243786_at	ZDHHC20	zinc finger, DHHC-type containing 20	chr13q12.11		253832	LOC705802	similar to DHHC-containing protein 20	---		705802	MmugDNA.1649.1.S1_at_ZDHHC20
MmugDNA.16492.1.S1_at	224803_s_at	LOC642393 /// MRPL20	similar to mitochondrial ribosomal protein L20 /// mitochondrial ribosomal protein L20	chr1p36.3-p36.2		55052 /// 642393	---	---	---	---		MmugDNA.16492.1.S1_at_LOC642393
MmugDNA.16493.1.S1_at	233537_at	KRTAP3-1	keratin associated protein 3-1	chr17q12-q21		83896	LOC697574	similar to keratin associated protein 3.1	---		697574	MmugDNA.16493.1.S1_at_KRTAP3-1
MmugDNA.16494.1.S1_s_at	216705_s_at	ADA	adenosine deaminase	chr20q12-q13.11		100	ADA	adenosine deaminase	---		717897	MmugDNA.16494.1.S1_s_at_ADA
MmugDNA.16495.1.S1_at	202680_at	GTF2E2	general transcription factor IIE, polypeptide 2, beta 34kDa	chr8p21-p12		2961	LOC707503	Similar to PRP4 pre-mRNA processing factor 4 homolog B (yeast) (predicted)	---		707503	MmugDNA.16495.1.S1_at_GTF2E2
MmugDNA.16496.1.S1_at	235172_at	---	---	---	---		---	---	---	---		MmugDNA.16496.1.S1_at_---
MmugDNA.16497.1.S1_at	202356_s_at	GTF2F1	general transcription factor IIF, polypeptide 1, 74kDa	chr19p13.3		2962	---	---	---	---		MmugDNA.16497.1.S1_at_GTF2F1
MmugDNA.16498.1.S1_at	1560172_at	INTS10	integrator complex subunit 10	chr8p21.3		55174	INTS10	integrator complex subunit 10	---		705240	MmugDNA.16498.1.S1_at_INTS10
MmugDNA.16499.1.S1_at	240272_at	---	---	---	---		---	---	---	---		MmugDNA.16499.1.S1_at_---
MmugDNA.16500.1.S1_at	230394_at	TCP10L	t-complex 10 (mouse)-like	chr21q22.11		140290	TCP10L	similar to T-complex 10A-2	---		700847	MmugDNA.16500.1.S1_at_TCP10L
MmugDNA.16501.1.S1_at	212551_at	CAP2	CAP, adenylate cyclase-associated protein, 2 (yeast)	chr6p22.3		10486	LOC699013	Similar to tumor protein, translationally-controlled 1	---		699013	MmugDNA.16501.1.S1_at_CAP2
MmugDNA.1650.1.S1_at	210010_s_at	SLC25A1	solute carrier family 25 (mitochondrial carrier; citrate transporter), member 1	chr22q11.21		6576	SLC25A1	solute carrier family 25 (mitochondrial carrier; citrate transporter), member 1	---		719075	MmugDNA.1650.1.S1_at_SLC25A1
MmugDNA.16502.1.S1_at	222277_at	LOC100133696 /// PCO	hypothetical protein LOC100133696 /// prostate collagen triple helix	chr13q12		100133696 /// 542767	---	---	---	---		MmugDNA.16502.1.S1_at_LOC100133
MmugDNA.16503.1.S1_at	233772_at	---	---	---	---		---	---	---	---		MmugDNA.16503.1.S1_at_---
MmugDNA.16504.1.S1_at	1557383_a_at	---	---	---	---		---	---	---	---		MmugDNA.16504.1.S1_at_---
MmugDNA.16505.1.S1_at	214996_at	---	---	---	---		---	---	---	---		MmugDNA.16505.1.S1_at_---
MmugDNA.16506.1.S1_at	238052_at	---	---	---	---		---	---	---	---		MmugDNA.16506.1.S1_at_---
MmugDNA.16507.1.S1_at	229481_at	LOC283859	hypothetical protein LOC283859	chr16q12.1		283859	---	---	---	---		MmugDNA.16507.1.S1_at_LOC283859
MmugDNA.16508.1.S1_at	1556536_at	hCG_2044152	hCG2044152	chr2q33.1		729224	---	---	---	---		MmugDNA.16508.1.S1_at_hCG_20441
MmugDNA.16509.1.S1_at	241333_x_at	---	---	---	---		---	---	---	---		MmugDNA.16509.1.S1_at_---
MmugDNA.16510.1.S1_at	241138_at	---	---	---	---		---	---	---	---		MmugDNA.16510.1.S1_at_---
MmugDNA.16511.1.S1_at	1556541_s_at	---	---	---	---		---	---	---	---		MmugDNA.16511.1.S1_at_---
MmugDNA.16512.1.S1_at	234654_at	---	---	---	---		---	---	---	---		MmugDNA.16512.1.S1_at_---
MmugDNA.16513.1.S1_at	1555090_x_at	TMEM182	transmembrane protein 182	chr2q12.1		130827	LOC712344	hypothetical protein LOC712344	---		712344	MmugDNA.16513.1.S1_at_TMEM182
MmugDNA.16514.1.S1_at	241892_at	---	---	---	---		---	---	---	---		MmugDNA.16514.1.S1_at_---
MmugDNA.16515.1.S1_at	1559213_at	---	---	---	---		---	---	---	---		MmugDNA.16515.1.S1_at_---
MmugDNA.16516.1.S1_x_at	NA	NA	NA	NA	NA		NA	NA	NA	NA		MmugDNA.16516.1.S1_x_at_NA
MmugDNA.16517.1.S1_at	236125_at	---	---	---	---		LOC698413	hypothetical protein LOC698413	---		698413	MmugDNA.16517.1.S1_at_---
MmugDNA.16518.1.S1_at	236485_at	LOC100129837	similar to ZNF124 protein	chr19p13.2		100129837	---	---	---	---		MmugDNA.16518.1.S1_at_LOC100129
MmugDNA.16519.1.S1_at	1555151_s_at	TDH	L-threonine dehydrogenase	chr8p23.1		157739	LOC697254	similar to L-threonine dehydrogenase	---		697254	MmugDNA.16519.1.S1_at_TDH
MmugDNA.165.1.S1_at	206244_at	CR1	complement component (3b/4b) receptor 1 (Knops blood group)	chr1q32		1378	---	---	---	---		MmugDNA.165.1.S1_at_CR1
MmugDNA.16520.1.S1_at	1564015_at	C18orf58	chromosome 18 open reading frame 58	chr18p11.22		284222	LOC703772	hypothetical protein LOC703772	---		703772	MmugDNA.16520.1.S1_at_C18orf58
MmugDNA.16521.1.S1_at	213486_at	COPG2IT1	COPG2 imprinted transcript 1 (non-protein coding)	chr7q32		53844	---	---	---	---		MmugDNA.16521.1.S1_at_COPG2IT1
MmugDNA.1652.1.S1_at	1553295_at	ABCA13	ATP-binding cassette, sub-family A (ABC1), member 13	chr7p12.3		154664	LOC695208	similar to ATP binding cassette, sub-family A (ABC1), member 13	---		695208	MmugDNA.1652.1.S1_at_ABCA13
MmugDNA.16522.1.S1_at	1563105_at	---	---	---	---		---	---	---	---		MmugDNA.16522.1.S1_at_---
MmugDNA.16523.1.S1_at	1558723_at	LOC284014	hypothetical protein LOC284014	chr17p13.3		284014	---	---	---	---		MmugDNA.16523.1.S1_at_LOC284014
MmugDNA.16524.1.S1_at	215870_s_at	PLA2G5	phospholipase A2, group V	chr1p36-p34		5322	LOC704629	similar to phospholipase A2, group V	---		704629	MmugDNA.16524.1.S1_at_PLA2G5
MmugDNA.16526.1.S1_at	209401_s_at	SLC12A4	solute carrier family 12 (potassium/chloride transporters), member 4	chr16q22.1		6560	SLC12A4	solute carrier family 12 (potassium/chloride transporters), member 4	---		701196	MmugDNA.16526.1.S1_at_SLC12A4
MmugDNA.16527.1.S1_at	242351_at	---	---	---	---		---	---	---	---		MmugDNA.16527.1.S1_at_---
MmugDNA.16528.1.S1_at	215540_at	TRA@	T cell receptor alpha locus	chr14q11.2		6955	LOC711787	similar to T-cell receptor alpha chain V region CTL-L17 precursor	---		711787	MmugDNA.16528.1.S1_at_TRA@
MmugDNA.16529.1.S1_at	225406_at	TWSG1	twisted gastrulation homolog 1 (Drosophila)	chr18p11.3		57045	---	---	---	---		MmugDNA.16529.1.S1_at_TWSG1
MmugDNA.16530.1.S1_at	233454_at	POLN	polymerase (DNA directed) nu	chr4p16.3		353497	LOC722353	similar to polymerase (DNA directed) nu	---		722353	MmugDNA.16530.1.S1_at_POLN
MmugDNA.16531.1.S1_at	1559352_a_at	LOC92659	hypothetical LOC92659	chr17q25.3		92659	---	---	---	---		MmugDNA.16531.1.S1_at_LOC92659
MmugDNA.1653.1.S1_at	200030_s_at	SLC25A3	solute carrier family 25 (mitochondrial carrier; phosphate carrier), member 3	chr12q23		5250	SLC25A3	solute carrier family 25 (mitochondrial carrier; phosphate carrier), member 3	---		693780	MmugDNA.1653.1.S1_at_SLC25A3
MmugDNA.16532.1.S1_at	1563300_at	LOC387826	Similar to hCG2001114	chr12p13.32		387826	---	---	---	---		MmugDNA.16532.1.S1_at_LOC387826
MmugDNA.16533.1.S1_at	221564_at	PRMT2	protein arginine methyltransferase 2	chr21q22.3		3275	---	---	---	---		MmugDNA.16533.1.S1_at_PRMT2
MmugDNA.16534.1.S1_at	213365_at	ERI2	exoribonuclease 2	chr16p12.2		112479	ERI2	similar to exonuclease domain containing 1	---		696072	MmugDNA.16534.1.S1_at_ERI2
MmugDNA.16535.1.S1_at	1558651_at	---	---	---	---		---	---	---	---		MmugDNA.16535.1.S1_at_---
MmugDNA.16536.1.S1_at	201888_s_at	IL13RA1	interleukin 13 receptor, alpha 1	chrXq24		3597	LOC710986	similar to Interleukin-13 receptor alpha-1 chain precursor (IL-13R-alpha-1) (IL-13RA-1) (CD213a1 ant	---		710986	MmugDNA.16536.1.S1_at_IL13RA1
MmugDNA.16537.1.S1_at	236805_at	C9orf96	chromosome 9 open reading frame 96	chr9q34.2		169436	LOC722205	similar to cyclin G associated kinase	---		722205	MmugDNA.16537.1.S1_at_C9orf96
MmugDNA.16540.1.S1_at	229865_at	FNDC3B	fibronectin type III domain containing 3B	chr3q26.31		64778	---	---	---	---		MmugDNA.16540.1.S1_at_FNDC3B
MmugDNA.16541.1.S1_at	212159_x_at	AP2A2	adaptor-related protein complex 2, alpha 2 subunit	chr11p15.5		161	---	---	---	---		MmugDNA.16541.1.S1_at_AP2A2
MmugDNA.1654.1.S1_at	1563546_at	---	---	---	---		---	---	---	---		MmugDNA.1654.1.S1_at_---

MmugDNA.16542.1.S1_at	1556238_at	---	---	---	---	---	---	---	---	MmugDNA.16542.1.S1_at_---
MmugDNA.16543.1.S1_x_at	NA	NA	NA	NA	NA	NA	NA	NA	NA	MmugDNA.16543.1.S1_x_at_NA
MmugDNA.16544.1.S1_at	1557248_at	ZNF587	Zinc finger protein 587	chr19q13.43	84914	---	---	---	---	MmugDNA.16544.1.S1_at_ZNF587
MmugDNA.16545.1.S1_at	203838_s_at	TNK2	tyrosine kinase, non-receptor, 2	chr3q29	10188	TNK2	tyrosine kinase, non-receptor, 2	---	711655	MmugDNA.16545.1.S1_at_TNK2
MmugDNA.16546.1.S1_at	205518_s_at	CMAH	cytidine monophosphate-N-acetylneuraminic acid hydroxylase (CMP-N-acetylneuraminate monooxyg	chr6p21.32	8418	CMAH	CMP-N-acetylneuraminic acid hydroxylase	---	574186	MmugDNA.16546.1.S1_at_CMAH
MmugDNA.16547.1.S1_at	234701_at	ANKRD11	ankyrin repeat domain 11	chr16q24.3	29123	ANKRD11	ankyrin repeat domain 11	---	698722	MmugDNA.16547.1.S1_at_ANKRD11
MmugDNA.16548.1.S1_at	240821_at	---	---	---	---	---	---	---	---	MmugDNA.16548.1.S1_at_---
MmugDNA.16549.1.S1_at	238643_at	---	---	---	---	---	---	---	---	MmugDNA.16549.1.S1_at_---
MmugDNA.16550.1.S1_at	237893_at	---	---	---	---	---	---	---	---	MmugDNA.16550.1.S1_at_---
MmugDNA.16551.1.S1_at	243875_at	CCDC158	coiled-coil domain containing 158	chr4q21.1	339965	LOC699735	similar to Myosin-1 (Myosin heavy chain D) (MHC D)	---	699735	MmugDNA.16551.1.S1_at_CCDC158
MmugDNA.1655.1.S1_at	1556486_at	---	---	---	---	---	---	---	---	MmugDNA.1655.1.S1_at_---
MmugDNA.16552.1.S1_x_at	NA	NA	NA	NA	NA	NA	NA	NA	NA	MmugDNA.16552.1.S1_x_at_NA
MmugDNA.16553.1.S1_at	1560522_at	LOC201477	hypothetical protein LOC201477	chr18p11.31	201477	---	---	---	---	MmugDNA.16553.1.S1_at_LOC201477
MmugDNA.16554.1.S1_at	236532_at	C11orf87	chromosome 11 open reading frame 87	chr11q22.3	399947	---	---	---	---	MmugDNA.16554.1.S1_at_C11orf87
MmugDNA.16555.1.S1_at	202663_at	WIPF1	WAS/WASL interacting protein family, member 1	chr2q31.1	7456	WIPF1	WAS/WASL interacting protein family, member 1	---	698446	MmugDNA.16555.1.S1_at_WIPF1
MmugDNA.16556.1.S1_at	202664_at	WIPF1	WAS/WASL interacting protein family, member 1	chr2q31.1	7456	WIPF1	WAS/WASL interacting protein family, member 1	---	698446	MmugDNA.16556.1.S1_at_WIPF1
MmugDNA.16557.1.S1_at	236567_at	---	---	---	---	---	---	---	---	MmugDNA.16557.1.S1_at_---
MmugDNA.16558.1.S1_at	209993_at	ABCB1	ATP-binding cassette, sub-family B (MDR/TAP), member 1	chr7q21.1	5243	ABCB1	ATP-binding cassette, sub-family B (MDR/TAP), member 1	---	574235	MmugDNA.16558.1.S1_at_ABCB1
MmugDNA.16559.1.S1_at	222915_s_at	BANK1	B-cell scaffold protein with ankyrin repeats 1	chr4q24	55024	LOC710040	similar to B-cell scaffold protein with ankyrin repeats 1	---	710040	MmugDNA.16559.1.S1_at_BANK1
MmugDNA.16560.1.S1_at	206887_at	CCBP2	chemokine binding protein 2	chr3p21.3	1238	LOC716097	hypothetical protein LOC716097	---	716097	MmugDNA.16560.1.S1_at_CCBP2
MmugDNA.16561.1.S1_at	243870_at	---	---	---	---	---	---	---	---	MmugDNA.16561.1.S1_at_---
MmugDNA.1656.1.S1_at	205450_at	PHKA1	phosphorylase kinase, alpha 1 (muscle)	chrXq12-q13	5255	PHKA1	phosphorylase kinase, alpha 1 (muscle)	---	699764	MmugDNA.1656.1.S1_at_PHKA1
MmugDNA.16562.1.S1_at	1556204_a_at	ZNF814	Zinc finger protein 814	chr19q13.43	730051	---	---	---	---	MmugDNA.16562.1.S1_at_ZNF814
MmugDNA.16563.1.S1_at	207342_at	CNGB1	cyclic nucleotide gated channel beta 1	chr16q13	1258	CNGB1	cyclic nucleotide gated channel beta 1	---	706073	MmugDNA.16563.1.S1_at_CNGB1
MmugDNA.16564.1.S1_at	1562718_at	---	---	---	---	---	---	---	---	MmugDNA.16564.1.S1_at_---
MmugDNA.16565.1.S1_at	240294_at	HIPK2	homeodomain interacting protein kinase 2	chr7q32-q34	28996	---	---	---	---	MmugDNA.16565.1.S1_at_HIPK2
MmugDNA.16566.1.S1_at	1556685_at	---	---	---	---	---	---	---	---	MmugDNA.16566.1.S1_at_---
MmugDNA.16567.1.S1_at	213985_s_at	C19orf6	Chromosome 19 open reading frame 6	chr19p13.3	91304	LOC721161	similar to membralin isoform 2	---	721161	MmugDNA.16567.1.S1_at_C19orf6
MmugDNA.16568.1.S1_at	242445_at	FGD4	FYVE, RhoGEF and PH domain containing 4	chr12p11.21	121512	---	---	---	---	MmugDNA.16568.1.S1_at_FGD4
MmugDNA.16569.1.S1_s_at	1556175_at	MTSS1L	metastasis suppressor 1-like	chr16q22.1	92154	---	---	---	---	MmugDNA.16569.1.S1_s_at_MTSS1L
MmugDNA.16570.1.S1_at	1557601_s_at	KIAA1257 /// LOC10013	KIAA1257 /// hypothetical protein LOC100132731	chr3q21.3	100132731 /// 57501	---	---	---	---	MmugDNA.16570.1.S1_at_KIAA1257 ///
MmugDNA.16571.1.S1_at	237426_at	SP100	SP100 nuclear antigen	chr2q37.1	6672	---	---	---	---	MmugDNA.16571.1.S1_at_SP100
MmugDNA.1657.1.S1_at	244722_at	---	---	---	---	---	---	---	---	MmugDNA.1657.1.S1_at_---
MmugDNA.16572.1.S1_at	202693_s_at	STK17A	serine/threonine kinase 17a	chr7p12-p14	9263	STK17A	serine/threonine kinase 17a	---	701091	MmugDNA.16572.1.S1_at_STK17A
MmugDNA.16573.1.S1_s_at	210592_s_at	SAT1	spermidine/spermine N1-acetyltransferase 1	chrXp22.1	6303	SAT1	spermidine/spermine N1-acetyltransferase 1	---	697884	MmugDNA.16573.1.S1_s_at_SAT1
MmugDNA.16574.1.S1_at	202694_at	STK17A	serine/threonine kinase 17a	chr7p12-p14	9263	---	---	---	---	MmugDNA.16574.1.S1_at_STK17A
MmugDNA.16575.1.S1_at	230804_at	NKAPL	NFKB activating protein-like	chr6p22.1	222698	LOC693565	Similar to progesterone membrane binding protein	---	693565	MmugDNA.16575.1.S1_at_NKAPL
MmugDNA.16576.1.S1_at	NA	NA	NA	NA	NA	NA	NA	NA	NA	MmugDNA.16576.1.S1_at_NA
MmugDNA.16576.1.S1_s_at	242422_at	G3BP1	GTPase activating protein (SH3 domain) binding protein 1	chr5q33.1	10146	---	---	---	---	MmugDNA.16576.1.S1_s_at_G3BP1
MmugDNA.16577.1.S1_at	1556812_a_at	---	---	---	---	---	---	---	---	MmugDNA.16577.1.S1_at_---
MmugDNA.16578.1.S1_at	237995_at	RIMBP2	RIMS binding protein 2	chr12q24.33	23504	---	---	---	---	MmugDNA.16578.1.S1_at_RIMBP2
MmugDNA.16579.1.S1_at	NA	NA	NA	NA	NA	NA	NA	NA	NA	MmugDNA.16579.1.S1_at_NA
MmugDNA.16579.1.S1_s_at	227449_at	EPHA4	EPH receptor A4	chr2q36.1	2043	HN1	Hematological and neurological expressed 1	---	664729	MmugDNA.16579.1.S1_s_at_EPHA4
MmugDNA.16580.1.S1_at	227751_at	PDCD5	programmed cell death 5	chr19q12-q13.1	9141	PDCD5	programmed cell death 5	---	702277	MmugDNA.16580.1.S1_at_PDCD5
MmugDNA.16581.1.S1_at	201445_at	CNN3	calponin 3, acidic	chr1p22-p21	1266	LOC696772	Similar to discs large homolog 7	---	696772	MmugDNA.16581.1.S1_at_CNN3
MmugDNA.1658.1.S1_at	226762_at	PURB	purine-rich element binding protein B	chr7p13	5814	LOC705519	similar to purine rich element binding protein B	---	705519	MmugDNA.1658.1.S1_at_PURB
MmugDNA.16582.1.S1_at	222471_s_at	KCMF1	potassium channel modulatory factor 1	chr2p11.2	56888	KCMF1	potassium channel modulatory factor 1	---	694645	MmugDNA.16582.1.S1_at_KCMF1
MmugDNA.16583.1.S1_at	NA	NA	NA	NA	NA	NA	NA	NA	NA	MmugDNA.16583.1.S1_at_NA
MmugDNA.16584.1.S1_at	233713_at	---	---	---	---	---	---	---	---	MmugDNA.16584.1.S1_at_---
MmugDNA.16585.1.S1_at	240716_at	TTC23	tetratricopeptide repeat domain 23	chr15q26.3	64927	LOC708530	similar to tetratricopeptide repeat domain 23	---	708530	MmugDNA.16585.1.S1_at_TTC23
MmugDNA.16586.1.S1_at	206586_at	CNR2	cannabinoid receptor 2 (macrophage)	chr1p36.11	1269	LOC711155	similar to cannabinoid receptor 2 (macrophage)	---	711155	MmugDNA.16586.1.S1_at_CNR2
MmugDNA.16587.1.S1_at	231677_at	---	---	---	---	---	---	---	---	MmugDNA.16587.1.S1_at_---
MmugDNA.16588.1.S1_at	234106_s_at	FLYWCH1	FLYWCH-type zinc finger 1	chr16p13.3	84256	---	---	---	---	MmugDNA.16588.1.S1_at_FLYWCH1
MmugDNA.16589.1.S1_at	235026_at	C12orf66	chromosome 12 open reading frame 66	chr12q14.2	144577	LOC717230	hypothetical protein LOC717230	---	717230	MmugDNA.16589.1.S1_at_C12orf66
MmugDNA.16590.1.S1_at	235678_at	GM2A	GM2 ganglioside activator	chr5q31.3-q33.1	2760	---	---	---	---	MmugDNA.16590.1.S1_at_GM2A
MmugDNA.16591.1.S1_at	225443_at	DCP1A	DCP1 decapping enzyme homolog A (S. cerevisiae)	chr3p21.1	55802	---	---	---	---	MmugDNA.16591.1.S1_at_DCP1A
MmugDNA.1659.1.S1_at	1559245_at	---	---	---	---	---	---	---	---	MmugDNA.1659.1.S1_at_---
MmugDNA.16592.1.S1_at	230517_at	tcag7.1196	similar to GLI-Kruppel family member HKR1	chr7q36.1	728743	LOC713120	similar to GLI-Kruppel family member HKR1	---	713120	MmugDNA.16592.1.S1_at_tcag7.1196
MmugDNA.16593.1.S1_at	222681_at	KTELC1	KTEL (Lys-Tyr-Glu-Leu) containing 1	chr3q13.33	56983	---	---	---	---	MmugDNA.16593.1.S1_at_KTELC1
MmugDNA.16594.1.S1_at	240705_at	CYP19A1	cytochrome P450, family 19, subfamily A, polypeptide 1	chr15q21.1	1588	---	---	---	---	MmugDNA.16594.1.S1_at_CYP19A1
MmugDNA.16595.1.S1_at	NA	NA	NA	NA	NA	NA	NA	NA	NA	MmugDNA.16595.1.S1_at_NA
MmugDNA.16596.1.S1_at	228533_at	---	---	---	---	---	---	---	---	MmugDNA.16596.1.S1_at_---
MmugDNA.16597.1.S1_at	205723_at	CNTFR	ciliary neurotrophic factor receptor	chr9p13	1271	CNTFR	ciliary neurotrophic factor receptor	---	700959	MmugDNA.16597.1.S1_at_CNTFR
MmugDNA.16598.1.S1_at	NA	NA	NA	NA	NA	NA	NA	NA	NA	MmugDNA.16598.1.S1_at_NA
MmugDNA.16598.1.S1_s_at	242462_at	LOC100131741	hypothetical protein LOC100131741	chr19q13.12	100131741	---	---	---	---	MmugDNA.16598.1.S1_s_at_LOC10013
MmugDNA.16599.1.S1_at	205048_s_at	PSPH	phosphoserine phosphatase	chr7p15.2-p15.1	5723	---	---	---	---	MmugDNA.16599.1.S1_at_PSPH
MmugDNA.16600.1.S1_at	221844_x_at	SPCS3	signal peptidase complex subunit 3 homolog (S. cerevisiae)	chr4q34.2	60559	---	---	---	---	MmugDNA.16600.1.S1_at_SPCS3
MmugDNA.16601.1.S1_at	214197_s_at	SETDB1	SET domain, bifurcated 1	chr1q21	9869	SETDB1	SET domain, bifurcated 1	---	716141	MmugDNA.16601.1.S1_at_SETDB1
MmugDNA.1660.1.S1_at	240469_at	---	---	---	---	---	---	---	---	MmugDNA.1660.1.S1_at_---
MmugDNA.16602.1.S1_at	210720_s_at	NECAB3	N-terminal EF-hand calcium binding protein 3	chr20q11.22	63941	LOC714183	similar to amyloid beta (A4) precursor protein-binding, family A, member 2 binding protein isoform 1	---	714183	MmugDNA.16602.1.S1_at_NECAB3
MmugDNA.16603.1.S1_at	224739_at	PIM3	pim-3 oncogene	chr22q13	415116	PIM3	pim-3 oncogene	---	720591	MmugDNA.16603.1.S1_at_PIM3
MmugDNA.16604.1.S1_at	227932_at	ARIH2	ariadne homolog 2 (Drosophila)	chr3p21.2-p21.3	10425	---	---	---	---	MmugDNA.16604.1.S1_at_ARIH2
MmugDNA.16605.1.S1_at	211266_s_at	GPR4	G protein-coupled receptor 4	chr19q13.3	2828	GPR4	G protein-coupled receptor 4	---	714913	MmugDNA.16605.1.S1_at_GPR4
MmugDNA.16606.1.S1_s_at	202514_at	DLG1	discs, large homolog 1 (Drosophila)	chr3q29	1739	DLG1	discs, large homolog 1 (Drosophila)	---	709572	MmugDNA.16606.1.S1_s_at_DLG1
MmugDNA.16607.1.S1_at	202515_at	DLG1	discs, large homolog 1 (Drosophila)	chr3q29	1739	DLG1	discs, large homolog 1 (Drosophila)	---	709572	MmugDNA.16607.1.S1_at_DLG1
MmugDNA.16608.1.S1_at	226982_at	ELL2	elongation factor, RNA polymerase II, 2	chr5q15	22936	---	---	---	---	MmugDNA.16608.1.S1_at_ELL2
MmugDNA.16609.1.S1_at	1558401_at	---	---	---	---	---	---	---	---	MmugDNA.16609.1.S1_at_---
MmugDNA.16610.1.S1_at	213017_at	ABHD3	abhydrolase domain containing 3	chr18q11.2	171586	LOC701145	similar to alpha/beta hydrolase domain containing protein 3	---	701145	MmugDNA.16610.1.S1_at_ABHD3
MmugDNA.16611.1.S1_at	234076_at	---	---	---	---	---	---	---	---	MmugDNA.16611.1.S1_at_---
MmugDNA.1661.1.S1_s_at	210962_s_at	AKAP9	A kinase (PRKA) anchor protein (yotiao) 9	chr7q21-q22	10142	AKAP9	A kinase (PRKA) anchor protein (yotiao) 9	---	702970	MmugDNA.1661.1.S1_s_at_AKAP9
MmugDNA.16612.1.S1_at	216204_at	COMT	Catechol-O-methyltransferase	chr22q11.21-q11.23|22q11.21	1312	---	---	---	---	MmugDNA.16612.1.S1_at_COMT
MmugDNA.16613.1.S1_at	226844_at	MOBKL2B	MOB1, Mps One Binder kinase activator-like 2B (yeast)	chr9p21.2	79817	LOC706919	similar to MOB1, Mps One Binder kinase activator-like 2B	---	706919	MmugDNA.16613.1.S1_at_MOBKL2B
MmugDNA.16614.1.S1_at	205972_at	SLC38A3	solute carrier family 38, member 3	chr3p21.3	10991	SLC38A3	solute carrier family 38, member 3	---	703671	MmugDNA.16614.1.S1_at_SLC38A3
MmugDNA.16615.1.S1_at	242475_at	---	---	---	---	---	---	---	---	MmugDNA.16615.1.S1_at_---
MmugDNA.16616.1.S1_at	1563486_at	---	---	---	---	LOC712600	similar to quaking homolog, KH domain RNA binding isoform HQK-5	---	712600	MmugDNA.16616.1.S1_at_---
MmugDNA.16617.1.S1_at	204636_at	COL17A1	collagen, type XVII, alpha 1	chr10q24.3	1308	---	---	---	---	MmugDNA.16617.1.S1_at_COL17A1
MmugDNA.16618.1.S1_at	1561700_at	---	---	---	---	---	---	---	---	MmugDNA.16618.1.S1_at_---
MmugDNA.16619.1.S1_at	236597_at	UGT3A1	UDP glycosyltransferase 3 family, polypeptide A1	chr5p13.2	133688	UGT3A1	UDP glycosyltransferase 3 family, polypeptide A1	---	700115	MmugDNA.16619.1.S1_at_UGT3A1
MmugDNA.166.1.S1_at	239353_at	---	---	---	---	STK32B	similar to serine/threonine kinase 32A	---	708524	MmugDNA.166.1.S1_at_---
MmugDNA.16620.1.S1_at	202312_s_at	COL1A1	collagen, type I, alpha 1	chr17q21.33	1277	---	---	---	---	MmugDNA.16620.1.S1_at_COL1A1
MmugDNA.16621.1.S1_at	1563295_at	---	---	---	---	---	---	---	---	MmugDNA.16621.1.S1_at_---
MmugDNA.1662.1.S1_at	223230_at	PRPF38A	PRP38 pre-mRNA processing factor 38 (yeast) domain containing A	chr1p33-p32.1	84950	LOC713344	similar to PRP38 pre-mRNA processing factor 38 (yeast) domain containing A	---	713344	MmugDNA.1662.1.S1_at_PRPF38A
MmugDNA.16622.1.S1_at	1562314_at	---	---	---	---	---	---	---	---	MmugDNA.16622.1.S1_at_---
MmugDNA.16623.1.S1_at	244733_at	---	---	---	---	---	---	---	---	MmugDNA.16623.1.S1_at_---
MmugDNA.16624.1.S1_at	233981_at	---	---	---	---	---	---	---	---	MmugDNA.16624.1.S1_at_---
MmugDNA.16625.1.S1_at	212369_at	ZNF384	zinc finger protein 384	chr12p12	171017	ZNF384	zinc finger protein 384	---	722077	MmugDNA.16625.1.S1_at_ZNF384
MmugDNA.16626.1.S1_at	227228_s_at	CCDC88C	coiled-coil domain containing 88C	chr14q32.12	440193	---	---	---	---	MmugDNA.16626.1.S1_at_CCDC88C
MmugDNA.16627.1.S1_at	NA	NA	NA	NA	NA	NA	NA	NA	NA	MmugDNA.16627.1.S1_at_NA
MmugDNA.16628.1.S1_at	243673_at	---	---	---	---	---	---	---	---	MmugDNA.16628.1.S1_at_---
MmugDNA.16629.1.S1_at	212861_at	MFSD5	major facilitator superfamily domain containing 5	chr12q13.13	84975	MFSD5	major facilitator superfamily domain containing 5	---	702021	MmugDNA.16629.1.S1_at_MFSD5
MmugDNA.16630.1.S1_at	244355_at	AVL9	AVL9 homolog (S. cerevisiase)	chr7p14.3	23080	---	---	---	---	MmugDNA.16630.1.S1_at_AVL9
MmugDNA.16631.1.S1_at	1564198_a_at	C10orf90	chromosome 10 open reading frame 90	chr10q26.2	118611	C9H10orf90	similar to Alstrom syndrome 1	---	696655	MmugDNA.16631.1.S1_at_C10orf90
MmugDNA.1663.1.S1_at	233140_s_at	C14orf133	chromosome 14 open reading frame 133	chr14q24.3-q31	63894	VPS16A	vacuolar protein sorting 16 homolog A (S. cerevisiae)	---	706315	MmugDNA.1663.1.S1_at_C14orf133
MmugDNA.16632.1.S1_at	1561948_at	COQ10B	Coenzyme Q10 homolog B (S. cerevisiae)	chr2q33.1	80219	---	---	---	---	MmugDNA.16632.1.S1_at_COQ10B
MmugDNA.16633.1.S1_at	241958_at	---	---	---	---	---	---	---	---	MmugDNA.16633.1.S1_at_---
MmugDNA.16634.1.S1_at	241035_s_at	---	---	---	---	---	---	---	---	MmugDNA.16634.1.S1_at_---
MmugDNA.16635.1.S1_at	214225_at	PIN4	protein (peptidylprolyl cis/trans isomerase) NIMA-interacting, 4 (parvulin)	chrXq13	5303	LOC711706 /// LOC7233	similar to protein (peptidyl-prolyl cis/trans isomerase) NIMA-interacting, 4 (parvulin) /// similar to Pepti	---	699273 /// 711706 /// 7233	MmugDNA.16635.1.S1_at_PIN4
MmugDNA.16636.1.S1_at	240780_at	---	---	---	---	---	---	---	---	MmugDNA.16636.1.S1_at_---

MmugDNA.16637.1.S1_at	1564479_a_at	---	---	---	---	---	---	---	---	MmugDNA.16637.1.S1_at_---
MmugDNA.16638.1.S1_at	215124_at	ZNF550	zinc finger protein 550	chr19q13.43	162972	LOC708304	hypothetical protein LOC708304	---	708304	MmugDNA.16638.1.S1_at_ZNF550
MmugDNA.16639.1.S1_at	230587_at	STGC3	hypothetical STGC3	---	474171	---	---	---	---	MmugDNA.16639.1.S1_at_STGC3
MmugDNA.16640.1.S1_at	1560402_at	GAS5	growth arrest-specific 5 (non-protein coding)	chr1q25.1	60674	---	---	---	---	MmugDNA.16640.1.S1_at_GAS5
MmugDNA.16641.1.S1_at	216857_at	---	---	---	---	---	---	---	---	MmugDNA.16641.1.S1_at_---
MmugDNA.1664.1.S1_at	233100_at	EEPD1	endonuclease/exonuclease/phosphatase family domain containing 1	chr7p14.2	80820	---	---	---	---	MmugDNA.1664.1.S1_at_EEPD1
MmugDNA.16642.1.S1_at	225892_at	IREB2	iron-responsive element binding protein 2	chr15q25.1	3658	ZC3H13	Zinc finger CCCH-type containing 13	---	703196	MmugDNA.16642.1.S1_at_IREB2
MmugDNA.16643.1.S1_at	225146_at	C9orf25	chromosome 9 open reading frame 25	chr9p13.3	203259	---	---	---	---	MmugDNA.16643.1.S1_at_C9orf25
MmugDNA.16644.1.S1_at	232465_at	---	---	---	---	---	---	---	---	MmugDNA.16644.1.S1_at_---
MmugDNA.16645.1.S1_at	232373_at	NOXA1	NADPH oxidase activator 1	chr9q34.3	10811	LOC721516	similar to NADPH oxidase activator 1	---	721516	MmugDNA.16645.1.S1_at_NOXA1
MmugDNA.16646.1.S1_at	235165_at	PARD6B	par-6 partitioning defective 6 homolog beta (C. elegans)	chr20q13.13	84612	---	---	---	---	MmugDNA.16646.1.S1_at_PARD6B
MmugDNA.16647.1.S1_at	232948_at	---	---	---	---	---	---	---	---	MmugDNA.16647.1.S1_at_---
MmugDNA.16648.1.S1_at	215370_at	---	---	---	---	---	---	---	---	MmugDNA.16648.1.S1_at_---
MmugDNA.16649.1.S1_at	215103_at	CYP2C18	cytochrome P450, family 2, subfamily C, polypeptide 18	chr10q24	1562	LOC701855	similar to cytochrome P450, family 2, subfamily C, polypeptide 18	---	701855	MmugDNA.16649.1.S1_at_CYP2C18
MmugDNA.16650.1.S1_at	1556540_a_at	---	---	---	---	---	---	---	---	MmugDNA.16650.1.S1_at_---
MmugDNA.16651.1.S1_at	1568997_at	---	---	---	---	---	---	---	---	MmugDNA.16651.1.S1_at_---
MmugDNA.1665.1.S1_at	208561_at	ABCC9	ATP-binding cassette, sub-family C (CFTR/MRP), member 9	chr12p12.1	10060	---	---	---	---	MmugDNA.1665.1.S1_at_ABCC9
MmugDNA.16652.1.S1_at	223920_s_at	TP53AIP1	tumor protein p53 regulated apoptosis inducing protein 1	chr11q24	63970	---	---	---	---	MmugDNA.16652.1.S1_at_TP53AIP1
MmugDNA.16653.1.S1_at	235876_at	---	---	---	---	---	---	---	---	MmugDNA.16653.1.S1_at_---
MmugDNA.16654.1.S1_at	242907_at	---	---	---	---	---	---	---	---	MmugDNA.16654.1.S1_at_---
MmugDNA.16655.1.S1_at	1557895_at	FLJ35934	FLJ35934 protein	chr17p11.2	400579	---	---	---	---	MmugDNA.16655.1.S1_at_FLJ35934
MmugDNA.16656.1.S1_at	212150_at	EFR3A	EFR3 homolog A (S. cerevisiae)	chr8q24.22	23167	GM-CSF /// LOC696036	Granulocyte-macrophage colony-stimulating factor /// Similar to Protein KIAA0143	---	574371 /// 696036	MmugDNA.16656.1.S1_at_EFR3A
MmugDNA.16657.1.S1_at	212149_at	EFR3A	EFR3 homolog A (S. cerevisiae)	chr8q24.22	23167	LOC696036	Similar to Protein KIAA0143	---	696036	MmugDNA.16657.1.S1_at_EFR3A
MmugDNA.16658.1.S1_at	1554778_at	PHLDB2	pleckstrin homology-like domain, family B, member 2	chr3q13.2	90102	LOC709353	similar to pleckstrin homology-like domain, family B, member 2	---	709353	MmugDNA.16658.1.S1_at_PHLDB2
MmugDNA.16659.1.S1_at	238216_at	LOC100129386	hypothetical protein LOC100129386	chr11p15.4	100129386	---	---	---	---	MmugDNA.16659.1.S1_at_LOC100129
MmugDNA.16660.1.S1_at	1555516_at	FOXP2	forkhead box P2	chr7q31	93986	---	---	---	---	MmugDNA.16660.1.S1_at_FOXP2
MmugDNA.16661.1.S1_at	237385_at	---	---	---	---	---	---	---	---	MmugDNA.16661.1.S1_at_---
MmugDNA.1666.1.S1_at	1552752_a_at	CADM2	cell adhesion molecule 2	chr3p12.1	253559	LOC719312	similar to immunoglobulin superfamily, member 4D	---	719312	MmugDNA.1666.1.S1_at_CADM2
MmugDNA.16662.1.S1_at	244761_at	FLJ44606	Hypothetical gene supported by AK126569	chr5q23.2	401207	---	---	---	---	MmugDNA.16662.1.S1_at_FLJ44606
MmugDNA.16663.1.S1_at	204136_at	COL7A1	collagen, type VII, alpha 1	chr3p21.1	1294	COL7A1	collagen, type VII, alpha 1	---	709354	MmugDNA.16663.1.S1_at_COL7A1
MmugDNA.16664.1.S1_at	231631_at	---	---	---	---	---	---	---	---	MmugDNA.16664.1.S1_at_---
MmugDNA.16665.1.S1_s_at	236983_at	TMC5	transmembrane channel-like 5	chr16p12.3	79838	LOC694659	hypothetical protein LOC694659	---	694659	MmugDNA.16665.1.S1_s_at_TMC5
MmugDNA.16666.1.S1_at	211568_at	BAI3	brain-specific angiogenesis inhibitor 3	chr6q12	577	---	---	---	---	MmugDNA.16666.1.S1_at_BAI3
MmugDNA.16667.1.S1_at	215065_at	PHF8	PHD finger protein 8	chrXp11.22	23133	---	---	---	---	MmugDNA.16667.1.S1_at_PHF8
MmugDNA.16668.1.S1_at	201153_s_at	MBNL1	muscleblind-like (Drosophila)	chr3q25	4154	B4GALNT1	Beta-1,4-N-acetyl-galactosaminyl transferase 1	---	715317	MmugDNA.16668.1.S1_at_MBNL1
MmugDNA.16669.1.S1_at	236867_at	---	---	---	---	---	---	---	---	MmugDNA.16669.1.S1_at_---
MmugDNA.16670.1.S1_at	1554555_a_at	SETD6	SET domain containing 6	chr16q21	79918	LOC707353	hypothetical protein LOC707353	---	707353	MmugDNA.16670.1.S1_at_SETD6
MmugDNA.16671.1.S1_at	210100_s_at	ABCA2	ATP-binding cassette, sub-family A (ABC1), member 2	chr9q34	20	LOC721631	similar to ATP-binding cassette, sub-family A, member 2 isoform b	---	721631	MmugDNA.16671.1.S1_at_ABCA2
MmugDNA.1667.1.S1_at	1552754_a_at	CADM2	cell adhesion molecule 2	chr3p12.1	253559	LOC719312	similar to immunoglobulin superfamily, member 4D	---	719312	MmugDNA.1667.1.S1_at_CADM2
MmugDNA.16672.1.S1_at	233343_at	DEFB127	defensin, beta 127	chr20p13	140850	LOC714155	similar to beta defensin 127 preproprotein	---	714155	MmugDNA.16672.1.S1_at_DEFB127
MmugDNA.16673.1.S1_at	237960_at	---	---	---	---	---	---	---	---	MmugDNA.16673.1.S1_at_---
MmugDNA.16674.1.S1_at	229949_at	---	---	---	---	---	---	---	---	MmugDNA.16674.1.S1_at_---
MmugDNA.16675.1.S1_at	1559348_a_at	---	---	---	---	---	---	---	---	MmugDNA.16675.1.S1_at_---
MmugDNA.16676.1.S1_at	226110_at	PTAR1	protein prenyltransferase alpha subunit repeat containing 1	chr9q21.11	375743	---	---	---	---	MmugDNA.16676.1.S1_at_PTAR1
MmugDNA.16677.1.S1_at	208957_at	ERP44	endoplasmic reticulum protein 44	chr9q31.1	23071	---	---	---	---	MmugDNA.16677.1.S1_at_ERP44
MmugDNA.16678.1.S1_at	208958_at	ERP44	endoplasmic reticulum protein 44	chr9q31.1	23071	---	---	---	---	MmugDNA.16678.1.S1_at_ERP44
MmugDNA.16679.1.S1_x_at	NA	NA	NA	NA	NA	NA	NA	NA	NA	MmugDNA.16679.1.S1_x_at_NA
MmugDNA.16680.1.S1_at	205517_at	GATA4	GATA binding protein 4	chr8p23.1-p22	2626	GATA4	GATA binding protein 4	---	696775	MmugDNA.16680.1.S1_at_GATA4
MmugDNA.16681.1.S1_at	239455_at	---	---	---	---	---	---	---	---	MmugDNA.16681.1.S1_at_---
MmugDNA.1668.1.S1_at	1559224_at	LCE1E	late cornified envelope 1E	chr1q21.3	353135	---	---	---	---	MmugDNA.1668.1.S1_at_LCE1E
MmugDNA.16682.1.S1_at	237484_at	C12orf69	chromosome 12 open reading frame 69	chr12p12.3	440087	LOC699680	hypothetical protein LOC699680	---	699680	MmugDNA.16682.1.S1_at_C12orf69
MmugDNA.16683.1.S1_at	57588_at	SLC24A3	solute carrier family 24 (sodium/potassium/calcium exchanger), member 3	chr20p13	57419	SLC24A3	solute carrier family 24 (sodium/potassium/calcium exchanger), member 3	---	702212	MmugDNA.16683.1.S1_at_SLC24A3
MmugDNA.16684.1.S1_at	208876_s_at	PAK2	p21 protein (Cdc42/Rac)-activated kinase 2	chr3q29	5062	LOC715969	Similar to Protein C14orf21 homolog	---	715969	MmugDNA.16684.1.S1_at_PAK2
MmugDNA.16685.1.S1_at	208158_s_at	OSBPL1A	oxysterol binding protein-like 1A	chr18q11.1	114876	OSBPL1A	oxysterol binding protein-like 1A	---	677867	MmugDNA.16685.1.S1_at_OSBPL1A
MmugDNA.16686.1.S1_at	208877_at	PAK2	p21 protein (Cdc42/Rac)-activated kinase 2	chr3q29	5062	---	---	---	---	MmugDNA.16686.1.S1_at_PAK2
MmugDNA.16686.1.S1_s_at	236283_x_at	LOC646214	p21-activated kinase 2 pseudogene	chr15q11.2	646214	---	---	---	---	MmugDNA.16686.1.S1_s_at_LOC64621
MmugDNA.16687.1.S1_at	217444_at	---	---	---	---	---	---	---	---	MmugDNA.16687.1.S1_at_---
MmugDNA.16688.1.S1_at	222596_s_at	LGR4	leucine-rich repeat-containing G protein-coupled receptor 4	chr11p14-p13	55366	LGR4	leucine-rich repeat-containing G protein-coupled receptor 4	---	701736	MmugDNA.16688.1.S1_at_LGR4
MmugDNA.16689.1.S1_at	240866_at	---	---	---	---	---	---	---	---	MmugDNA.16689.1.S1_at_---
MmugDNA.16690.1.S1_at	224427_s_at	PAPOLG	poly(A) polymerase gamma	chr2p16.1	64895	LOC717781	similar to poly(A) polymerase gamma	---	717781	MmugDNA.16690.1.S1_at_PAPOLG
MmugDNA.16691.1.S1_at	1562669_at	---	---	---	---	---	---	---	---	MmugDNA.16691.1.S1_at_---
MmugDNA.1669.1.S1_s_at	217909_s_at	MLX	MAX-like protein X	chr17q21.1	6945	MLX	MAX-like protein X	---	710570	MmugDNA.1669.1.S1_s_at_MLX
MmugDNA.16692.1.S1_at	1562287_at	SNTG1	Syntrophin, gamma 1	chr8q11-q12	54212	---	---	---	---	MmugDNA.16692.1.S1_at_SNTG1
MmugDNA.16693.1.S1_at	1565073_at	---	---	---	---	---	---	---	---	MmugDNA.16693.1.S1_at_---
MmugDNA.16694.1.S1_at	202109_at	ARFIP2	ADP-ribosylation factor interacting protein 2	chr11p15	23647	ARFIP2	ADP-ribosylation factor interacting protein 2	---	710929	MmugDNA.16694.1.S1_at_ARFIP2
MmugDNA.16695.1.S1_at	236756_at	LOC389857	hypothetical protein	chrXp11.22	389857	LOC717718	similar to proline rich 6	---	717718	MmugDNA.16695.1.S1_at_LOC389857
MmugDNA.16696.1.S1_s_at	201043_s_at	ANP32A /// LOC100128	acidic (leucine-rich) nuclear phosphoprotein 32 family, member A /// hypothetical protein LOC100128	chr15q22.3-q23 /// chr15q23	100128146 /// 8125	LOC712768	Hypothetical protein LOC712768	---	712768	MmugDNA.16696.1.S1_s_at_ANP32A //
MmugDNA.16697.1.S1_at	205434_s_at	AAK1	AP2 associated kinase 1	chr2p14	22848	LOC708047	hypothetical protein LOC708047	---	708047	MmugDNA.16697.1.S1_at_AAK1
MmugDNA.16698.1.S1_at	223716_s_at	ZRANB2	zinc finger, RAN-binding domain containing 2	chr1p31	9406	ZRANB2	zinc finger, RAN-binding domain containing 2	---	703513	MmugDNA.16698.1.S1_at_ZRANB2
MmugDNA.16699.1.S1_at	223016_x_at	ZRANB2	zinc finger, RAN-binding domain containing 2	chr1p31	9406	ZRANB2	zinc finger, RAN-binding domain containing 2	---	703513	MmugDNA.16699.1.S1_at_ZRANB2
MmugDNA.16700.1.S1_at	NA	NA	NA	NA	NA	NA	NA	NA	NA	MmugDNA.16700.1.S1_at_NA
MmugDNA.16700.1.S1_s_at	226389_s_at	RAPGEF1	Rap guanine nucleotide exchange factor (GEF) 1	chr9q34.3	2889	LOC715272	similar to guanine nucleotide-releasing factor 2 isoform b	---	715272	MmugDNA.16700.1.S1_s_at_RAPGEF1
MmugDNA.16701.1.S1_at	205527_s_at	GEMIN4	gem (nuclear organelle) associated protein 4	chr17p13	50628	GEMIN4	gem (nuclear organelle) associated protein 4	---	699841	MmugDNA.16701.1.S1_at_GEMIN4
MmugDNA.1670.1.S1_at	228665_at	CYYR1	cysteine/tyrosine-rich 1	chr21q21.2	116159	CYYR1	similar to cysteine and tyrosine-rich 1 protein precursor	---	708008	MmugDNA.1670.1.S1_at_CYYR1
MmugDNA.16702.1.S1_x_at	1570266_x_at	---	---	---	---	---	---	---	---	MmugDNA.16702.1.S1_x_at_---
MmugDNA.16703.1.S1_at	238864_at	VWA3A	von Willebrand factor A domain containing 3A	chr16p12.1	146177	---	---	---	---	MmugDNA.16703.1.S1_at_VWA3A
MmugDNA.16704.1.S1_at	NA	NA	NA	NA	NA	NA	NA	NA	NA	MmugDNA.16704.1.S1_at_NA
MmugDNA.16705.1.S1_at	NA	NA	NA	NA	NA	NA	NA	NA	NA	MmugDNA.16705.1.S1_at_NA
MmugDNA.16705.1.S1_s_at	233528_s_at	GATSL3	GATS protein-like 3	chr22q12	652968	LOC715407	similar to TBC1 domain family, member 10A	---	715407	MmugDNA.16705.1.S1_s_at_GATSL3
MmugDNA.16706.1.S1_at	241573_at	---	---	---	---	---	---	---	---	MmugDNA.16706.1.S1_at_---
MmugDNA.16708.1.S1_at	241854_at	DNASE1	deoxyribonuclease I	chr16p13.3	1773	---	---	---	---	MmugDNA.16708.1.S1_at_DNASE1
MmugDNA.16709.1.S1_s_at	204570_at	COX7A1	cytochrome c oxidase subunit VIIa polypeptide 1 (muscle)	chr19q13.1	1346	COX7AH	cytochrome c oxidase subunit 7AH	---	692060	MmugDNA.16709.1.S1_s_at_COX7A1
MmugDNA.16710.1.S1_at	201597_at	COX7A2	cytochrome c oxidase subunit VIIa polypeptide 2 (liver)	chr6q12	1347	LOC703896	similar to Cytochrome c oxidase polypeptide VIIa-liver/heart, mitochondrial precursor (Cytochrome c c	---	703896	MmugDNA.16710.1.S1_at_COX7A2
MmugDNA.16711.1.S1_at	NA	NA	NA	NA	NA	NA	NA	NA	NA	MmugDNA.16711.1.S1_at_NA
MmugDNA.16711.1.S1_x_at	NA	NA	NA	NA	NA	NA	NA	NA	NA	MmugDNA.16711.1.S1_x_at_NA
MmugDNA.1671.1.S1_at	213585_s_at	PDCD2	Programmed cell death 2	chr6q27	5134	---	---	---	---	MmugDNA.1671.1.S1_at_PDCD2
MmugDNA.16712.1.S1_at	NA	NA	NA	NA	NA	NA	NA	NA	NA	MmugDNA.16712.1.S1_at_NA
MmugDNA.16713.1.S1_at	239787_at	KCTD4	potassium channel tetramerisation domain containing 4	chr13q14.12	386618	LOC710651	Similar to Phenylalanyl-tRNA synthetase, mitochondrial precursor (Phenylalanine--tRNA ligase) (PheI	---	710651	MmugDNA.16713.1.S1_at_KCTD4
MmugDNA.16714.1.S1_at	243751_at	---	---	---	---	---	---	---	---	MmugDNA.16714.1.S1_at_---
MmugDNA.16715.1.S1_at	211642_at	C12orf32 /// IGHG1	Chromosome 12 open reading frame 32 /// Immunoglobulin heavy constant gamma 1 (G1m marker)	chr12p13.33 /// chr14q32.33	3500 /// 83695	IGHV@	similar to Ig heavy chain V-III region VH26 precursor	---	715594	MmugDNA.16715.1.S1_at_C12orf32 ///
MmugDNA.16716.1.S1_at	216752_at	PIK3R4	Phosphoinositide-3-kinase, regulatory subunit 4	chr3q22.1	30849	---	---	---	---	MmugDNA.16716.1.S1_at_PIK3R4
MmugDNA.16717.1.S1_s_at	218720_x_at	LOC652900 /// SEZ6L2	similar to seizure related 6 homolog (mouse)-like 2 isoform 1 /// seizure related 6 homolog (mouse)-lik	chr16p11.2	26470 /// 652900	LOC707244	similar to seizure related 6 homolog (mouse)-like 2 isoform 2	---	707244	MmugDNA.16717.1.S1_s_at_LOC6529(
MmugDNA.16718.1.S1_at	208011_at	PTPN22	protein tyrosine phosphatase, non-receptor type 22 (lymphoid)	chr1p13.3-p13.1	26191	PTPN22	protein tyrosine phosphatase, non-receptor type 22 (lymphoid)	---	707433	MmugDNA.16718.1.S1_at_PTPN22
MmugDNA.16719.1.S1_at	1557689_at	---	---	---	---	---	---	---	---	MmugDNA.16719.1.S1_at_---
MmugDNA.167.1.S1_at	200736_s_at	GPX1	glutathione peroxidase 1	chr3p21.3	2876	GPX1	glutathione peroxidase 1	---	706732	MmugDNA.167.1.S1_at_GPX1
MmugDNA.16720.1.S1_at	240087_at	---	---	---	---	---	---	---	---	MmugDNA.16720.1.S1_at_---
MmugDNA.16721.1.S1_at	241921_x_at	---	---	---	---	---	---	---	---	MmugDNA.16721.1.S1_at_---
MmugDNA.1672.1.S1_at	206025_s_at	TNFAIP6	tumor necrosis factor, alpha-induced protein 6	chr2q23.3	7130	TNFAIP6	tumor necrosis factor, alpha-induced protein 6	---	694699	MmugDNA.1672.1.S1_at_TNFAIP6
MmugDNA.16722.1.S1_at	202487_s_at	H2AFV	H2A histone family, member V	chr7p13	94239	LOC705636	similar to H2A histone family, member V isoform 1	---	705636	MmugDNA.16722.1.S1_at_H2AFV
MmugDNA.16723.1.S1_at	1558855_at	FARP2	FERM, RhoGEF and pleckstrin domain protein 2	chr2q37.3	9855	---	---	---	---	MmugDNA.16723.1.S1_at_FARP2
MmugDNA.16724.1.S1_at	202374_s_at	RAB3GAP2	RAB3 GTPase activating protein subunit 2 (non-catalytic)	chr1q41	25782	RAB3GAP2	RAB3 GTPase activating protein subunit 2 (non-catalytic)	---	706252	MmugDNA.16724.1.S1_at_RAB3GAP2
MmugDNA.16725.1.S1_at	1553228_at	CCDC89	coiled-coil domain containing 89	chr11q14.1	220388	TCHH	similar to coiled-coil domain containing 89	---	703071	MmugDNA.16725.1.S1_at_CCDC89
MmugDNA.16726.1.S1_at	1554772_at	C17orf57	chromosome 17 open reading frame 57	chr17q21.32	124989	---	---	---	---	MmugDNA.16726.1.S1_at_C17orf57
MmugDNA.16727.1.S1_at	215734_at	C19orf36	chromosome 19 open reading frame 36	chr19p13.3	113177	LOC709954	hypothetical protein LOC709954	---	709954	MmugDNA.16727.1.S1_at_C19orf36
MmugDNA.16728.1.S1_at	1552613_s_at	CDC42SE2	CDC42 small effector 2	chr5q31.1	56990	---	---	---	---	MmugDNA.16728.1.S1_at_CDC42SE2
MmugDNA.16728.1.S1_s_at	NA	NA	NA	NA	NA	NA	NA	NA	NA	MmugDNA.16728.1.S1_s_at_NA
MmugDNA.16729.1.S1_at	NA	NA	NA	NA	NA	NA	NA	NA	NA	MmugDNA.16729.1.S1_at_NA
MmugDNA.16730.1.S1_at	226520_at	---	---	---	---	---	---	---	---	MmugDNA.16730.1.S1_at_---

MmugDNA.16731.1.S1_s_at	201781_s_at	AIP	aryl hydrocarbon receptor interacting protein	chr11q13.3	9049	LOC711814	similar to aryl hydrocarbon receptor interacting protein	---	711814	MmugDNA.16731.1.S1_s_at_AIP
MmugDNA.1673.1.S1_at	239878_at	IPMK	inositol polyphosphate multikinase	chr10q21.1	253430	IPMK	inositol polyphosphate multikinase	---	703630	MmugDNA.1673.1.S1_at_IPMK
MmugDNA.16732.1.S1_at	NA	NA	NA	NA	NA	NA	NA	NA	NA	MmugDNA.16732.1.S1_at_NA
MmugDNA.16733.1.S1_at	207399_at	BFSP2	beaded filament structural protein 2, phakinin	chr3q21-q25	8419	BFSP2	similar to Phakinin (Beaded filament structural protein 2) (Lens fiber cell beaded filament protein CP 4	---	717551	MmugDNA.16733.1.S1_at_BFSP2
MmugDNA.16734.1.S1_at	228913_at	LOC100190939	hypothetical LOC100190939	---	100190939	---	---	---	---	MmugDNA.16734.1.S1_at_LOC1001909
MmugDNA.16735.1.S1_at	1569325_at	ARPC5	Actin related protein 2/3 complex, subunit 5, 16kDa	chr1q25.3	10092	---	---	---	---	MmugDNA.16735.1.S1_at_ARPC5
MmugDNA.16736.1.S1_at	205615_at	CPA1	carboxypeptidase A1 (pancreatic)	chr7q32	1357	CPA1	carboxypeptidase A1 (pancreatic)	---	703095	MmugDNA.16736.1.S1_at_CPA1
MmugDNA.16737.1.S1_at	237504_at	INTS10	integrator complex subunit 10	chr8p21.3	55174	INTS10	integrator complex subunit 10	---	705240	MmugDNA.16737.1.S1_at_INTS10
MmugDNA.16738.1.S1_at	206212_at	CPA2	carboxypeptidase A2 (pancreatic)	chr7q32	1358	CPA2	carboxypeptidase A2 (pancreatic)	---	706684	MmugDNA.16738.1.S1_at_CPA2
MmugDNA.16739.1.S1_at	236405_at	FBXO41	F-box protein 41	chr2p13.2	150726	---	---	---	---	MmugDNA.16739.1.S1_at_FBXO41
MmugDNA.16740.1.S1_at	1570250_at	RP11-35N6.1	plasticity related gene 3	chr9q31.1	54886	---	---	---	---	MmugDNA.16740.1.S1_at_RP11-35N6.
MmugDNA.16741.1.S1_at	203220_s_at	TLE1	transducin-like enhancer of split 1 (E(sp1) homolog, Drosophila)	chr9q21.32	7088	LOC707336	similar to transducin-like enhancer protein 1	---	707336	MmugDNA.16741.1.S1_at_TLE1
MmugDNA.1674.1.S1_at	229920_at	---	---	---	---	---	---	---	---	MmugDNA.1674.1.S1_at_---
MmugDNA.16742.1.S1_at	244256_at	CACNA1E	Calcium channel, voltage-dependent, R type, alpha 1E subunit	chr1q25-q31	777	---	---	---	---	MmugDNA.16742.1.S1_at_CACNA1E
MmugDNA.16743.1.S1_at	244280_at	---	---	---	---	---	---	---	---	MmugDNA.16743.1.S1_at_---
MmugDNA.16744.1.S1_at	235792_x_at	PIK3C2A	phosphoinositide-3-kinase, class 2, alpha polypeptide	chr11p15.5-p14	5286	LOC701569	Similar to retinoblastoma-binding protein 6 isoform 2	---	701569	MmugDNA.16744.1.S1_at_PIK3C2A
MmugDNA.16745.1.S1_s_at	211809_x_at	COL13A1	collagen, type XIII, alpha 1	chr10q22	1305	LOC716986	similar to alpha 1 type XIII collagen isoform 5	---	716986	MmugDNA.16745.1.S1_s_at_COL13A1
MmugDNA.16746.1.S1_at	244623_at	KCNQ5	potassium voltage-gated channel, KQT-like subfamily, member 5	chr6q14	56479	---	---	---	---	MmugDNA.16746.1.S1_at_KCNQ5
MmugDNA.16747.1.S1_at	217822_at	WBP11	WW domain binding protein 11	chr12p12.3	51729	WBP11	WW domain binding protein 11	---	699451	MmugDNA.16747.1.S1_at_WBP11
MmugDNA.16748.1.S1_at	219155_at	PITPNC1	phosphatidylinositol transfer protein, cytoplasmic 1	chr17q24.2	26207	LOC718773	similar to phosphatidylinositol transfer protein, cytoplasmic 1 isoform a	---	718773	MmugDNA.16748.1.S1_at_PITPNC1
MmugDNA.16749.1.S1_at	235830_at	---	---	---	---	---	---	---	---	MmugDNA.16749.1.S1_at_---
MmugDNA.16750.1.S1_at	212873_at	HMHA1	histocompatibility (minor) HA-1	chr19p13.3	23526	LOC721173	similar to minor histocompatibility antigen HA-1	---	721173	MmugDNA.16750.1.S1_at_HMHA1
MmugDNA.16751.1.S1_at	207204_at	FSCN2	fascin homolog 2, actin-bundling protein, retinal (Strongylocentrotus purpuratus)	chr17q25	25794	FSCN2	fascin homolog 2, actin-bundling protein, retinal (Strongylocentrotus purpuratus)	---	713824	MmugDNA.16751.1.S1_at_FSCN2
MmugDNA.1675.1.S1_at	202738_s_at	PHKB	phosphorylase kinase, beta	chr16q12-q13	5257	PHKB	phosphorylase kinase, beta	---	716390	MmugDNA.1675.1.S1_at_PHKB
MmugDNA.16752.1.S1_at	1557287_at	---	---	---	---	---	---	---	---	MmugDNA.16752.1.S1_at_---
MmugDNA.16753.1.S1_at	225331_at	CCDC50	coiled-coil domain containing 50	chr3q28	152137	CCDC50	coiled-coil domain containing 50	---	705459	MmugDNA.16753.1.S1_at_CCDC50
MmugDNA.16754.1.S1_at	225345_s_at	---	---	---	---	FBXO32	F-box protein 32	---	704804	MmugDNA.16754.1.S1_at_---
MmugDNA.16754.1.S1_s_at	NA	NA	NA	NA	NA	NA	NA	NA	NA	MmugDNA.16754.1.S1_s_at_NA
MmugDNA.16755.1.S1_at	220507_s_at	UPB1	ureidopropionase, beta	chr22q11.2	51733	UPB1	ureidopropionase, beta	---	707752	MmugDNA.16755.1.S1_at_UPB1
MmugDNA.16756.1.S1_at	232035_at	HIST1H4B	Histone cluster 1, H4b	chr6p21.3	8366	---	---	---	---	MmugDNA.16756.1.S1_at_HIST1H4B
MmugDNA.16757.1.S1_at	225328_at	---	---	---	---	FBXO32	F-box protein 32	---	704804	MmugDNA.16757.1.S1_at_---
MmugDNA.16758.1.S1_at	236152_at	PAGE5	P antigen family, member 5 (prostate associated)	chrXp11.21	90737	PAGE5	P antigen family, member 5 (prostate associated)	---	703127	MmugDNA.16758.1.S1_at_PAGE5
MmugDNA.16759.1.S1_at	232946_s_at	NADSYN1	NAD synthetase 1	chr11q13.4	55191	NADSYN1	NAD synthetase 1	---	707652	MmugDNA.16759.1.S1_at_NADSYN1
MmugDNA.16760.1.S1_at	241556_at	---	---	---	---	---	---	---	---	MmugDNA.16760.1.S1_at_---
MmugDNA.16761.1.S1_at	233543_s_at	FAM175A	family with sequence similarity 175, member A	chr4q21.21-q21.23	84142	LOC720625	similar to coiled-coil domain containing 98	---	720625	MmugDNA.16761.1.S1_at_FAM175A
MmugDNA.1676.1.S1_at	241151_at	---	---	---	---	---	---	---	---	MmugDNA.1676.1.S1_at_---
MmugDNA.16762.1.S1_at	1560184_at	TMCO5A	transmembrane and coiled-coil domains 5A	chr15q14	145942	LOC700246	hypothetical protein LOC700246	---	700246	MmugDNA.16762.1.S1_at_TMCO5A
MmugDNA.16763.1.S1_at	202776_at	DNTTIP2	deoxynucleotidyltransferase, terminal, interacting protein 2	chr1p22.1	30836	LOC713888	similar to estrogen receptor binding protein	---	713888	MmugDNA.16763.1.S1_at_DNTTIP2
MmugDNA.16764.1.S1_at	228608_at	NALCN	sodium leak channel, non-selective	chr13q32.3	259232	---	---	---	---	MmugDNA.16764.1.S1_at_NALCN
MmugDNA.16765.1.S1_at	205176_s_at	ITGB3BP	integrin beta 3 binding protein (beta3-endonexin)	chr1p31.3	23421	ITGB3BP	integrin beta 3 binding protein (beta3-endonexin)	---	699279	MmugDNA.16765.1.S1_at_ITGB3BP
MmugDNA.16766.1.S1_at	226958_s_at	MED11	mediator complex subunit 11	chr17p13.2	400569	LOC709561	similar to Mediator complex subunit 11 CG6884-PA	---	709561	MmugDNA.16766.1.S1_at_MED11
MmugDNA.16767.1.S1_at	1560148_at	---	---	---	---	---	---	---	---	MmugDNA.16767.1.S1_at_---
MmugDNA.16768.1.S1_s_at	212995_x_at	FAM128B	family with sequence similarity 128, member B	chr2q21.1	80097	LOC701787	hypothetical protein LOC701787	---	701787	MmugDNA.16768.1.S1_s_at_FAM128B
MmugDNA.16769.1.S1_at	237609_at	---	---	---	---	---	---	---	---	MmugDNA.16769.1.S1_at_---
MmugDNA.16770.1.S1_at	1553873_at	KLHL34	kelch-like 34 (Drosophila)	chrXp22.12	257240	KLHL34	kelch-like 34 (Drosophila)	---	696156	MmugDNA.16770.1.S1_at_KLHL34
MmugDNA.16771.1.S1_at	1553239_at	FAM124A	family with sequence similarity 124A	chr13q14.3	220108	LOC708395	hypothetical protein LOC708395	---	708395	MmugDNA.16771.1.S1_at_FAM124A
MmugDNA.1677.1.S1_at	203709_at	PHKG2	phosphorylase kinase, gamma 2 (testis)	chr16p12.1-p11.2	5261	---	---	---	---	MmugDNA.1677.1.S1_at_PHKG2
MmugDNA.16772.1.S1_at	212592_at	IGJ	immunoglobulin J polypeptide, linker protein for immunoglobulin alpha and mu polypeptides	chr4q21	3512	IGJ	immunoglobulin J polypeptide, linker protein for immunoglobulin alpha and mu polypeptides	---	706650	MmugDNA.16772.1.S1_at_IGJ
MmugDNA.16773.1.S1_at	219112_at	RAPGEF6	Rap guanine nucleotide exchange factor (GEF) 6	chr5q31.1	51735	RAPGEF6	Rap guanine nucleotide exchange factor (GEF) 6	---	706541	MmugDNA.16773.1.S1_at_RAPGEF6
MmugDNA.16774.1.S1_s_at	200716_x_at	RPL13A	ribosomal protein L13a	chr19q13.3	23521	LOC698713	similar to ribosomal protein L13a	---	698713	MmugDNA.16774.1.S1_s_at_RPL13A
MmugDNA.16775.1.S1_at	1570622_at	---	---	---	---	---	---	---	---	MmugDNA.16775.1.S1_at_---
MmugDNA.16776.1.S1_at	224530_s_at	KCNIP4	Kv channel interacting protein 4	chr4p15.31	80333	LOC715577	similar to Kv channel interacting protein 4 isoform 4	---	715577	MmugDNA.16776.1.S1_at_KCNIP4
MmugDNA.16777.1.S1_s_at	214077_x_at	MEIS3P1	Meis homeobox 3 pseudogene 1	chr17p12	4213	---	---	---	---	MmugDNA.16777.1.S1_s_at_MEIS3P1
MmugDNA.16778.1.S1_at	239938_x_at	---	---	---	---	---	---	---	---	MmugDNA.16778.1.S1_at_---
MmugDNA.16779.1.S1_at	219911_s_at	LOC100134295 /// SLC	similar to Solute carrier organic anion transporter family, member 4A1 /// solute carrier organic anion t	chr20q13.33	100134295 /// 28231	SLCO4A1	solute carrier organic anion transporter family, member 4A1	---	699062	MmugDNA.16779.1.S1_at_LOC100134
MmugDNA.16780.1.S1_at	236105_at	---	---	---	---	---	---	---	---	MmugDNA.16780.1.S1_at_---
MmugDNA.16781.1.S1_at	1560292_a_at	TMCO4	transmembrane and coiled-coil domains 4	chr1p36.13	255104	TMCO4	transmembrane and coiled-coil domains 4	---	703851	MmugDNA.16781.1.S1_at_TMCO4
MmugDNA.1678.1.S1_at	210204_s_at	CNOT4	CCR4-NOT transcription complex, subunit 4	chr7q22-qter	4850	CNOT4	CCR4-NOT transcription complex, subunit 4	---	708011	MmugDNA.1678.1.S1_at_CNOT4
MmugDNA.16782.1.S1_at	241284_at	---	---	---	---	---	---	---	---	MmugDNA.16782.1.S1_at_---
MmugDNA.16783.1.S1_at	201846_s_at	RYBP	RING1 and YY1 binding protein	chr3p13	23429	LOC694390	similar to RING1 and YY1 binding protein	---	694390	MmugDNA.16783.1.S1_at_RYBP
MmugDNA.16784.1.S1_at	1568665_at	RNF103	ring finger protein 103	chr2p11.2	7844	---	---	---	---	MmugDNA.16784.1.S1_at_RNF103
MmugDNA.16785.1.S1_s_at	NA	NA	NA	NA	NA	NA	NA	NA	NA	MmugDNA.16785.1.S1_s_at_NA
MmugDNA.16786.1.S1_at	239432_at	FLJ31306	hypothetical protein FLJ31306	chr14q23.1	379025	---	---	---	---	MmugDNA.16786.1.S1_at_FLJ31306
MmugDNA.16787.1.S1_at	237457_at	EIF3B	eukaryotic translation initiation factor 3, subunit B	chr7p22.2	8662	EIF3B	eukaryotic translation initiation factor 3, subunit B	---	698481	MmugDNA.16787.1.S1_at_EIF3B
MmugDNA.16788.1.S1_at	203942_s_at	MARK2	MAP/microtubule affinity-regulating kinase 2	chr11q12-q13	2011	MARK2	MAP/microtubule affinity-regulating kinase 2	---	718065	MmugDNA.16788.1.S1_at_MARK2
MmugDNA.16789.1.S1_at	239775_at	LOC100131512	hypothetical protein LOC100131512	chr7p21.1	100131512	---	---	---	---	MmugDNA.16789.1.S1_at_LOC1001315
MmugDNA.16790.1.S1_s_at	244112_x_at	---	---	---	---	---	---	---	---	MmugDNA.16790.1.S1_s_at_---
MmugDNA.16791.1.S1_at	227613_at	ZNF331	zinc finger protein 331	chr19q13.41	55422	---	---	---	---	MmugDNA.16791.1.S1_at_ZNF331
MmugDNA.1679.1.S1_at	203691_at	PI3	peptidase inhibitor 3, skin-derived	chr20q12-q13	5266	PI3	peptidase inhibitor 3, skin-derived	---	574237	MmugDNA.1679.1.S1_at_PI3
MmugDNA.16792.1.S1_at	215574_at	---	---	---	---	---	---	---	---	MmugDNA.16792.1.S1_at_---
MmugDNA.16794.1.S1_at	237556_at	LOC728804	Hypothetical LOC728804	chr16p11.2	728804	---	---	---	---	MmugDNA.16794.1.S1_at_LOC728804
MmugDNA.16795.1.S1_at	207279_s_at	NEBL	nebulette	chr10p12	10529	NEBL	nebulette	---	702764	MmugDNA.16795.1.S1_at_NEBL
MmugDNA.16796.1.S1_at	219077_s_at	WWOX	WW domain containing oxidoreductase	chr16q23.3-q24.1	51741	LOC709897	Similar to SSXT protein (Synovial sarcoma, translocated to X chromosome) (SYT protein)	---	709897	MmugDNA.16796.1.S1_at_WWOX
MmugDNA.16797.1.S1_at	231802_at	CLEC3A	C-type lectin domain family 3, member A	chr16q23	10143	CLEC3A	C-type lectin domain family 3, member A	---	713054	MmugDNA.16797.1.S1_at_CLEC3A
MmugDNA.16798.1.S1_at	233011_at	ANXA1	Annexin A1	chr9q12-q21.2|9q12-q21.2	301	ANXA1	annexin A1	---	702946	MmugDNA.16798.1.S1_at_ANXA1
MmugDNA.16799.1.S1_at	235537_at	OCIAD1	OCIA domain containing 1	chr4p11	54940	---	---	---	---	MmugDNA.16799.1.S1_at_OCIAD1
MmugDNA.16800.1.S1_at	1557056_at	C5orf47	chromosome 5 open reading frame 47	chr5q35.2	133491	---	---	---	---	MmugDNA.16800.1.S1_at_C5orf47
MmugDNA.16801.1.S1_at	220307_at	CD244	CD244 molecule, natural killer cell receptor 2B4	chr1q23.3	51744	LOC719800	similar to Natural killer cell receptor 2B4 precursor (NKR2B4) (NK cell type I receptor protein 2B4) (h	---	719800	MmugDNA.16801.1.S1_at_CD244
MmugDNA.1680.1.S1_at	1561195_at	---	---	---	---	---	---	---	---	MmugDNA.1680.1.S1_at_---
MmugDNA.16802.1.S1_at	218703_at	SEC22A	SEC22 vesicle trafficking protein homolog A (S. cerevisiae)	chr3q21.1	26984	LOC715277	similar to SEC22 vesicle trafficking protein homolog A	---	715277	MmugDNA.16802.1.S1_at_SEC22A
MmugDNA.16803.1.S1_at	236721_at	ALKBH1	alkB, alkylation repair homolog 1 (E. coli)	chr14q24.3	8846	ALKBH1	alkylated DNA repair protein alkB homolog	---	706760	MmugDNA.16803.1.S1_at_ALKBH1
MmugDNA.16804.1.S1_at	206941_x_at	SEMA3E	sema domain, immunoglobulin domain (Ig), short basic domain, secreted, (semaphorin) 3E	chr7q21.11	9723	---	---	---	---	MmugDNA.16804.1.S1_at_SEMA3E
MmugDNA.16806.1.S1_at	1569433_at	SAMD5	sterile alpha motif domain containing 5	chr6q24.3	389432	---	---	---	---	MmugDNA.16806.1.S1_at_SAMD5
MmugDNA.16807.1.S1_at	233905_at	SPAG4L	sperm associated antigen 4-like	chr20q11.21	140732	LOC710995	similar to sperm associated antigen 4-like	---	710995	MmugDNA.16807.1.S1_at_SPAG4L
MmugDNA.16809.1.S1_at	221835_at	DTX3	deltex homolog 3 (Drosophila)	chr12q13.3	196403	LOC715175	similar to deltex 3 homolog	---	715175	MmugDNA.16809.1.S1_at_DTX3
MmugDNA.16810.1.S1_at	202202_s_at	LAMA4	laminin, alpha 4	chr6q21	3910	LOC696685	hypothetical protein LOC696685	---	696685	MmugDNA.16810.1.S1_at_LAMA4
MmugDNA.16811.1.S1_at	229504_at	---	---	---	---	---	---	---	---	MmugDNA.16811.1.S1_at_---
MmugDNA.1681.1.S1_at	241471_at	LOC730236	hypothetical LOC730236	chr13q21.32	730236	---	---	---	---	MmugDNA.1681.1.S1_at_LOC730236
MmugDNA.16812.1.S1_at	220273_at	IL17B	interleukin 17B	chr5q32-q34	27190	IL17B	interleukin 17B	---	710679	MmugDNA.16812.1.S1_at_IL17B
MmugDNA.16813.1.S1_at	241711_at	---	---	---	---	---	---	---	---	MmugDNA.16813.1.S1_at_---
MmugDNA.16814.1.S1_at	200771_at	LAMC1	laminin, gamma 1 (formerly LAMB2)	chr1q31	3915	LOC715265	Hypothetical protein LOC715265	---	715265	MmugDNA.16814.1.S1_at_LAMC1
MmugDNA.16815.1.S1_x_at	NA	NA	NA	NA	NA	NA	NA	NA	NA	MmugDNA.16815.1.S1_x_at_NA
MmugDNA.16816.1.S1_at	205707_at	IL17RA	interleukin 17 receptor A	chr22q11.1	23765	LOC709005	similar to interleukin 17A receptor precursor	---	709005	MmugDNA.16816.1.S1_at_IL17RA
MmugDNA.16817.1.S1_at	207517_at	LAMC2	laminin, gamma 2	chr1q25-q31	3918	LAMC2	laminin, gamma 2	---	715212	MmugDNA.16817.1.S1_at_LAMC2
MmugDNA.16818.1.S1_at	201279_s_at	DAB2	disabled homolog 2, mitogen-responsive phosphoprotein (Drosophila)	chr5p13	1601	DAB2	disabled homolog 2	---	694349	MmugDNA.16818.1.S1_at_DAB2
MmugDNA.16819.1.S1_at	1559672_a_at	C9orf93	chromosome 9 open reading frame 93	chr9p22.3	203238	LOC714370	Similar to decay accelerating factor for complement	---	714370	MmugDNA.16819.1.S1_at_C9orf93
MmugDNA.168.1.S1_at	243013_at	---	---	---	---	---	---	---	---	MmugDNA.168.1.S1_at_---
MmugDNA.16820.1.S1_at	219683_at	FZD3	frizzled homolog 3 (Drosophila)	chr8p21	7976	FZD3	frizzled homolog 3 (Drosophila)	---	713839	MmugDNA.16820.1.S1_at_FZD3
MmugDNA.16821.1.S1_at	233978_at	PTPRE	Protein tyrosine phosphatase, receptor type, E	chr10q26	5791	---	---	---	---	MmugDNA.16821.1.S1_at_PTPRE
MmugDNA.1682.1.S1_at	1554503_a_at	OSCAR	osteoclast associated, immunoglobulin-like receptor	chr19q13.42	126014	---	---	---	---	MmugDNA.1682.1.S1_at_OSCAR
MmugDNA.16822.1.S1_at	222236_s_at	ASAP3	ArfGAP with SH3 domain, ankyrin repeat and PH domain 3	chr1p36.12	55616	LOC718875	similar to development and differentiation enhancing factor-like 1	---	718875	MmugDNA.16822.1.S1_at_ASAP3
MmugDNA.16823.1.S1_at	229400_at	HOXD10	homeobox D10	chr2q31.1	3236	HOXD10	similar to Homeobox protein Hox-D10 (Hox-4D) (Hox-4E)	---	700318	MmugDNA.16823.1.S1_at_HOXD10
MmugDNA.16824.1.S1_at	236066_at	---	---	---	---	---	---	---	---	MmugDNA.16824.1.S1_at_---
MmugDNA.16826.1.S1_at	220044_x_at	CROP	cisplatin resistance-associated overexpressed protein	chr17q21.33	51747	LEMD3	LEM domain containing 3	---	717597	MmugDNA.16826.1.S1_at_CROP
MmugDNA.16826.1.S1_s_at	NA	NA	NA	NA	NA	NA	NA	NA	NA	MmugDNA.16826.1.S1_s_at_NA
MmugDNA.16827.1.S1_at	239538_at	ZRANB3	zinc finger, RAN-binding domain containing 3	chr2q21.3	84083	LOC712445	similar to zinc finger, RAN-binding domain containing 3	---	712445	MmugDNA.16827.1.S1_at_ZRANB3
MmugDNA.16828.1.S1_at	226316_at	---	---	---	---	POLD3	Polymerase (DNA-directed), delta 3, accessory subunit	---	693601	MmugDNA.16828.1.S1_at_---
MmugDNA.16829.1.S1_at	208603_s_at	MAPK8IP2	mitogen-activated protein kinase 8 interacting protein 2	chr22q13.33	23542	MAPK8IP2	mitogen-activated protein kinase 8 interacting protein 2	---	720708	MmugDNA.16829.1.S1_at_MAPK8IP2

MmugDNA.16830.1.S1_at	1561347_a_at	---	---	---	---		---	---	---	---	MmugDNA.16830.1.S1_at_---
MmugDNA.16831.1.S1_s_at	208584_at	SNCG	synuclein, gamma (breast cancer-specific protein 1)	chr10q23.2-q23.3		6623	---	---	---	---	MmugDNA.16831.1.S1_s_at_SNCG
MmugDNA.1683.1.S1_at	236481_at	FAH	fumarylacetoacetate hydrolase (fumarylacetoacetase)	chr15q23-q25		2184	---	---	---	---	MmugDNA.1683.1.S1_at_FAH
MmugDNA.16832.1.S1_s_at	1569383_s_at	ZFYVE28	zinc finger, FYVE domain containing 28	chr4p16.3		57732	---	---	---	---	MmugDNA.16832.1.S1_s_at_ZFYVE28
MmugDNA.16833.1.S1_at	220663_at	IL1RAPL1	interleukin 1 receptor accessory protein-like 1	chrXp22.1-p21.3		11141	IL1RAPL1	interleukin 1 receptor accessory protein-like 1	---	706588	MmugDNA.16833.1.S1_at_IL1RAPL1
MmugDNA.16834.1.S1_at	238213_at	---	---	---	---		---	---	---	---	MmugDNA.16834.1.S1_at_---
MmugDNA.16835.1.S1_at	223131_s_at	TRIM8	tripartite motif-containing 8	chr10q24.3		81603	NOC2L	Nucleolar complex associated 2 homolog	---	697809	MmugDNA.16835.1.S1_at_TRIM8
MmugDNA.16836.1.S1_at	225448_at	NAPG	N-ethylmaleimide-sensitive factor attachment protein, gamma	chr18p11.22		8774	---	---	---	---	MmugDNA.16836.1.S1_at_NAPG
MmugDNA.16837.1.S1_at	201784_s_at	C11orf58	chromosome 11 open reading frame 58	chr11p15.1		10944	LOC697690	similar to small acidic protein	---	697690	MmugDNA.16837.1.S1_at_C11orf58
MmugDNA.16838.1.S1_at	1562051_at	LOC284757	hypothetical protein LOC284757	chr20q13.33		284757	---	---	---	---	MmugDNA.16838.1.S1_at_LOC284757
MmugDNA.16839.1.S1_at	230494_at	SLC20A1	Solute carrier family 20 (phosphate transporter), member 1	chr2q11-q14		6574	LOC699462	similar to solute carrier family 20 (phosphate transporter), member 1	---	699462	MmugDNA.16839.1.S1_at_SLC20A1
MmugDNA.16840.1.S1_at	211576_s_at	SLC19A1	solute carrier family 19 (folate transporter), member 1	chr21q22.3		6573	LOC721917	similar to Folate transporter 1 (Solute carrier family 19 member 1) (Placental folate transporter) (FOL	---	721917	MmugDNA.16840.1.S1_at_SLC19A1
MmugDNA.16841.1.S1_at	240315_at	---	---	---	---		---	---	---	---	MmugDNA.16841.1.S1_at_---
MmugDNA.1684.1.S1_at	236758_at	LOC100129407	similar to hCG1993257	chrXq13.1		100129407	---	---	---	---	MmugDNA.1684.1.S1_at_LOC100129(
MmugDNA.16842.1.S1_at	220755_s_at	C6orf48	chromosome 6 open reading frame 48	chr6p21.3		50854	---	---	---	---	MmugDNA.16842.1.S1_at_C6orf48
MmugDNA.16843.1.S1_at	231493_at	---	---	---	---		---	---	---	---	MmugDNA.16843.1.S1_at_---
MmugDNA.16844.1.S1_at	1561429_a_at	C3orf15	chromosome 3 open reading frame 15	chr3q12-q13.3		89876	LOC697219	Similar to 40S ribosomal protein S16	---	697219	MmugDNA.16844.1.S1_at_C3orf15
MmugDNA.16845.1.S1_at	241797_at	---	---	---	---		---	---	---	---	MmugDNA.16845.1.S1_at_---
MmugDNA.16846.1.S1_at	240187_at	PPP1R3C	protein phosphatase 1, regulatory (inhibitor) subunit 3C	chr10q23-q24		5507	---	---	---	---	MmugDNA.16846.1.S1_at_PPP1R3C
MmugDNA.16847.1.S1_at	238271_x_at	---	---	---	---		---	---	---	---	MmugDNA.16847.1.S1_at_---
MmugDNA.16848.1.S1_at	NA	NA	NA	NA	NA		NA	NA	NA	NA	MmugDNA.16848.1.S1_at_NA
MmugDNA.16848.1.S1_s_at	213829_x_at	RTEL1	regulator of telomere elongation helicase 1	chr20q13.3		51750	LOC719519	similar to regulator of telomere elongation helicase 1	---	719519	MmugDNA.16848.1.S1_s_at_RTEL1
MmugDNA.16849.1.S1_at	220047_at	SIRT4	sirtuin (silent mating type information regulation 2 homolog) 4 (S. cerevisiae)	chr12q		23409	LOC720498	similar to NAD-dependent deacetylase sirtuin-4 (SIR2-like protein 4)	---	720498	MmugDNA.16849.1.S1_at_SIRT4
MmugDNA.16850.1.S1_at	219719_at	HIGD1B	HIG1 hypoxia inducible domain family, member 1B	chr17q21.31		51751	HIGD1B	HIG1 hypoxia inducible domain family, member 1B	---	715566	MmugDNA.16850.1.S1_at_HIGD1B
MmugDNA.16851.1.S1_at	231397_at	PAP2D	phosphatidic acid phosphatase type 2	chr1p21.3		163404	---	---	---	---	MmugDNA.16851.1.S1_at_PAP2D
MmugDNA.1685.1.S1_at	207605_x_at	ZNF117	zinc finger protein 117	chr7q11.21		51351	LOC719936	similar to zinc finger protein 682	---	719936	MmugDNA.1685.1.S1_at_ZNF117
MmugDNA.16852.1.S1_at	236172_at	LTB4R	leukotriene B4 receptor	chr14q11.2-q12		1241	LTB4R	leukotriene B4 receptor	---	716055	MmugDNA.16852.1.S1_at_LTB4R
MmugDNA.16853.1.S1_at	1561518_at	LOC283914	hypothetical protein LOC283914	chr16p11.1		283914	---	---	---	---	MmugDNA.16853.1.S1_at_LOC283914
MmugDNA.16854.1.S1_at	235253_at	RAD1	RAD1 homolog (S. pombe)	chr5p13.2		5810	LOC698993	similar to RAD1 homolog isoform 1	---	698993	MmugDNA.16854.1.S1_at_RAD1
MmugDNA.16855.1.S1_at	207839_s_at	C9orf127	chromosome 9 open reading frame 127	chr9p13.3		51754	C15H9orf127	chromosome 9 open reading frame 127 ortholog	---	695699	MmugDNA.16855.1.S1_at_C9orf127
MmugDNA.16856.1.S1_at	206770_s_at	SLC35A3	solute carrier family 35 (UDP-N-acetylglucosamine (UDP-GlcNAc) transporter), member A3	chr1p21		23443	LOC711077	similar to solute carrier family 35 (UDP-N-acetylglucosamine (UDP-GlcNAc) transporter), member A3	---	711077	MmugDNA.16856.1.S1_at_SLC35A3
MmugDNA.16857.1.S1_at	202607_at	NDST1	N-deacetylase/N-sulfotransferase (heparan glucosaminyl) 1	chr5q33.1		3340	NDST1	N-deacetylase/N-sulfotransferase (heparan glucosaminyl) 1	---	712425	MmugDNA.16857.1.S1_at_NDST1
MmugDNA.16858.1.S1_at	NA	NA	NA	NA	NA		NA	NA	NA	NA	MmugDNA.16858.1.S1_at_NA
MmugDNA.16858.1.S1_s_at	229036_at	TNRC6B	trinucleotide repeat containing 6B	chr22q13.1		23112	TNRC6B	trinucleotide repeat containing 6B	---	704630	MmugDNA.16858.1.S1_s_at_TNRC6B
MmugDNA.16859.1.S1_at	1554274_a_at	SSH1	slingshot homolog 1 (Drosophila)	chr12q24.11		54434	SSH1	slingshot homolog 1 (Drosophila)	---	707809	MmugDNA.16859.1.S1_at_SSH1
MmugDNA.16860.1.S1_at	NA	NA	NA	NA	NA		NA	NA	NA	NA	MmugDNA.16860.1.S1_at_NA
MmugDNA.16861.1.S1_at	1557985_s_at	CEP78	centrosomal protein 78kDa	chr9q21.2		84131	CEP78	centrosomal protein 78kDa	---	706167	MmugDNA.16861.1.S1_at_CEP78
MmugDNA.1686.1.S1_at	240052_at	ITPR1	inositol 1,4,5-triphosphate receptor, type 1	chr3p26-p25		3708	ITPR1	inositol 1,4,5-triphosphate receptor, type 1	---	703669	MmugDNA.1686.1.S1_at_ITPR1
MmugDNA.16862.1.S1_at	214383_x_at	KLHDC3	kelch domain containing 3	chr6p21.1		116138	LOC698107	similar to testis intracellular mediator protein	---	698107	MmugDNA.16862.1.S1_at_KLHDC3
MmugDNA.16863.1.S1_at	221307_at	KCNIP1	Kv channel interacting protein 1	chr5q35.1		30820	LOC706021	similar to Kv channel-interacting protein 1 (KChIP1) (A-type potassium channel modulatory protein 1)	---	706021	MmugDNA.16863.1.S1_at_KCNIP1
MmugDNA.16864.1.S1_at	226268_at	RAB21	RAB21, member RAS oncogene family	chr12q21.1		23011	---	---	---	---	MmugDNA.16864.1.S1_at_RAB21
MmugDNA.16865.1.S1_at	235858_at	CREBBP	CREB binding protein	chr16p13.3		1387	---	---	---	---	MmugDNA.16865.1.S1_at_CREBBP
MmugDNA.16866.1.S1_at	221656_s_at	ARHGEF10L	Rho guanine nucleotide exchange factor (GEF) 10-like	chr1p36.13		55160	ARHGEF10L	Rho guanine nucleotide exchange factor (GEF) 10-like	---	701164	MmugDNA.16866.1.S1_at_ARHGEF10L
MmugDNA.16868.1.S1_at	1556558_s_at	FLJ36665	hypothetical protein FLJ36665	chr3p21.33		285266	TBC1D19	TBC1 domain family, member 19	---	694865	MmugDNA.16868.1.S1_at_FLJ36665
MmugDNA.16869.1.S1_at	223065_s_at	STARD3NL	STARD3 N-terminal like	chr7p14-p13		83930	LOC705212	similar to MLN64 N-terminal homolog	---	705212	MmugDNA.16869.1.S1_at_STARD3NL
MmugDNA.16870.1.S1_at	213072_at	CYHR1	cysteine/histidine-rich 1	chr8q24.3		50626	LOC702522	similar to cysteine and histidine rich 1 isoform 1	---	702522	MmugDNA.16870.1.S1_at_CYHR1
MmugDNA.16871.1.S1_at	240773_at	---	---	---	---		---	---	---	---	MmugDNA.16871.1.S1_at_---
MmugDNA.1687.1.S1_at	236705_at	TMEM196	transmembrane protein 196	chr7p15.3		256130	---	---	---	---	MmugDNA.1687.1.S1_at_TMEM196
MmugDNA.16873.1.S1_at	225582_at	ITPRIP	inositol 1,4,5-triphosphate receptor interacting protein	chr10q25.1		85450	---	---	---	---	MmugDNA.16873.1.S1_at_ITPRIP
MmugDNA.16874.1.S1_s_at	210130_s_at	TM7SF2	transmembrane 7 superfamily member 2	chr11q13		7108	TM7SF2	transmembrane 7 superfamily member 2	---	721985	MmugDNA.16874.1.S1_s_at_TM7SF2
MmugDNA.16875.1.S1_at	234141_s_at	LOC286059	hypothetical protein LOC286059	chr8p21.2		286059	---	---	---	---	MmugDNA.16875.1.S1_at_LOC286059
MmugDNA.16876.1.S1_at	205613_at	SYT17	synaptotagmin XVII	chr16p12.3		51760	SYT17	synaptotagmin XVII	---	694130	MmugDNA.16876.1.S1_at_SYT17
MmugDNA.16877.1.S1_at	212766_s_at	ISG20L2	interferon stimulated exonuclease gene 20kDa-like 2	chr1q23.1		81875	LOC718621	similar to interferon stimulated exonuclease gene 20kDa-like 2	---	718621	MmugDNA.16877.1.S1_at_ISG20L2
MmugDNA.16878.1.S1_at	227949_at	PHACTR3	phosphatase and actin regulator 3	chr20q13.32		116154	PHACTR3	phosphatase and actin regulator 3	---	701648	MmugDNA.16878.1.S1_at_PHACTR3
MmugDNA.16879.1.S1_at	235364_at	TSC22D3	TSC22 domain family, member 3	chrXq22.3		1831	---	---	---	---	MmugDNA.16879.1.S1_at_TSC22D3
MmugDNA.16880.1.S1_at	1569604_at	LOC100133612	similar to hCG1815312	---		100133612	---	---	---	---	MmugDNA.16880.1.S1_at_LOC100133(
MmugDNA.16881.1.S1_at	238469_at	OGFRL1	opioid growth factor receptor-like 1	chr6q13		79627	OSBP	Oxysterol binding protein	---	699325	MmugDNA.16881.1.S1_at_OGFRL1
MmugDNA.1688.1.S1_at	228414_at	KCNMA1	potassium large conductance calcium-activated channel, subfamily M, alpha member 1	chr10q22.3		3778	---	---	---	---	MmugDNA.1688.1.S1_at_KCNMA1
MmugDNA.16882.1.S1_at	241786_at	---	---	---	---		---	---	---	---	MmugDNA.16882.1.S1_at_---
MmugDNA.16883.1.S1_at	200662_s_at	TOMM20	translocase of outer mitochondrial membrane 20 homolog (yeast)	chr1q42		9804	TOMM20	translocase of outer mitochondrial membrane 20 homolog (yeast)	---	712039	MmugDNA.16883.1.S1_at_TOMM20
MmugDNA.16884.1.S1_s_at	221597_s_at	TMEM208	transmembrane protein 208	chr16q22.1		29100	TMEM208	transmembrane protein 208	---	697228	MmugDNA.16884.1.S1_s_at_TMEM208
MmugDNA.16885.1.S1_at	203144_s_at	KIAA0040	KIAA0040	chr1q24-q25		9674	---	---	---	---	MmugDNA.16885.1.S1_at_KIAA0040
MmugDNA.16886.1.S1_at	1556158_at	FAM154B	family with sequence similarity 154, member B	chr15q25.2		283726	LOC713942 /// LOC7181	hypothetical protein LOC713942 /// similar to ADAM metallopeptidase with thrombospondin type 1 mo	---	713942 /// 718170	MmugDNA.16886.1.S1_at_FAM154B
MmugDNA.16887.1.S1_at	1557706_at	ZHX2	zinc fingers and homeoboxes 2	chr8q24.13		22882	---	---	---	---	MmugDNA.16887.1.S1_at_ZHX2
MmugDNA.16888.1.S1_at	202235_at	SLC16A1	solute carrier family 16, member 1 (monocarboxylic acid transporter 1)	chr1p12		6566	SLC16A1	solute carrier family 16, member 1	---	706593	MmugDNA.16888.1.S1_at_SLC16A1
MmugDNA.16889.1.S1_at	238031_at	---	---	---	---		---	---	---	---	MmugDNA.16889.1.S1_at_---
MmugDNA.16890.1.S1_at	237841_at	---	---	---	---		---	---	---	---	MmugDNA.16890.1.S1_at_---
MmugDNA.16891.1.S1_at	226847_at	FST	follistatin	chr5q11.2		10468	FST	follistatin	---	707420	MmugDNA.16891.1.S1_at_FST
MmugDNA.1689.1.S1_at	1556804_s_at	POLR3B	polymerase (RNA) III (DNA directed) polypeptide B	chr12q23.3		55703	---	---	---	---	MmugDNA.1689.1.S1_at_POLR3B
MmugDNA.16892.1.S1_at	242744_s_at	CASR	calcium-sensing receptor	chr3q13		846	---	---	---	---	MmugDNA.16892.1.S1_at_CASR
MmugDNA.16893.1.S1_at	219660_s_at	ATP8A2	ATPase, aminophospholipid transporter-like, class I, type 8A, member 2	chr13q12		51761	ATP8A2	ATPase, aminophospholipid transporter-like, class I, type 8A, member 2	---	709311	MmugDNA.16893.1.S1_at_ATP8A2
MmugDNA.16894.1.S1_at	1570122_at	---	---	---	---		---	---	---	---	MmugDNA.16894.1.S1_at_---
MmugDNA.16895.1.S1_at	218638_s_at	SPON2	spondin 2, extracellular matrix protein	chr4p16.3		10417	SPON2	spondin 2, extracellular matrix protein	---	702450	MmugDNA.16895.1.S1_at_SPON2
MmugDNA.16896.1.S1_at	219753_at	STAG3	stromal antigen 3	chr7q22.1		10734	LOC711345	similar to stromal antigen 3	---	711345	MmugDNA.16896.1.S1_at_STAG3
MmugDNA.16897.1.S1_x_at	220806_x_at	GNG13	guanine nucleotide binding protein (G protein), gamma 13	chr16p13.3		51764	---	---	---	---	MmugDNA.16897.1.S1_x_at_GNG13
MmugDNA.16898.1.S1_at	238754_at	---	---	---	---		---	---	---	---	MmugDNA.16898.1.S1_at_---
MmugDNA.16899.1.S1_at	236093_at	---	---	---	---		---	---	---	---	MmugDNA.16899.1.S1_at_---
MmugDNA.16900.1.S1_at	231940_at	ZNF529	zinc finger protein 529	chr19q13.13		57711	---	---	---	---	MmugDNA.16900.1.S1_at_ZNF529
MmugDNA.16901.1.S1_at	205026_at	STAT5B	signal transducer and activator of transcription 5B	chr17q11.2		6777	LOC709760	similar to signal transducer and activator of transcription 5B	---	709760	MmugDNA.16901.1.S1_at_STAT5B
MmugDNA.1690.1.S1_at	214299_at	TOP3A	topoisomerase (DNA) III alpha	chr17p12-p11.2		7156	---	---	---	---	MmugDNA.1690.1.S1_at_TOP3A
MmugDNA.16902.1.S1_at	218499_at	RP6-213H19.1	serine/threonine protein kinase MST4	chrXq26.2		51765	---	---	---	---	MmugDNA.16902.1.S1_at_RP6-213H19
MmugDNA.16903.1.S1_at	225418_at	PVRL2	poliovirus receptor-related 2 (herpesvirus entry mediator B)	chr19q13.2		5819	---	---	---	---	MmugDNA.16903.1.S1_at_PVRL2
MmugDNA.16904.1.S1_at	219824_at	SLC13A4	solute carrier family 13 (sodium/sulfate symporters), member 4	chr7q33		26266	SLC13A4	solute carrier family 13 (sodium/sulfate symporters), member 4	---	708744	MmugDNA.16904.1.S1_at_SLC13A4
MmugDNA.16905.1.S1_at	210577_at	CASR	calcium-sensing receptor	chr3q13		846	---	---	---	---	MmugDNA.16905.1.S1_at_CASR
MmugDNA.16906.1.S1_at	203097_s_at	RAPGEF2	Rap guanine nucleotide exchange factor (GEF) 2	chr4q32.1		9693	RAPGEF2	Rap guanine nucleotide exchange factor (GEF) 2	---	704717	MmugDNA.16906.1.S1_at_RAPGEF2
MmugDNA.16907.1.S1_at	244509_at	GPR155	G protein-coupled receptor 155	chr2q31.1		151556	GPR155	G protein-coupled receptor 155	---	698323	MmugDNA.16907.1.S1_at_GPR155
MmugDNA.16908.1.S1_at	1556457_s_at	FLJ39739	hypothetical FLJ39739	chr1q21.1		388685	---	---	---	---	MmugDNA.16908.1.S1_at_FLJ39739
MmugDNA.16908.1.S1_x_at	NA	NA	NA	NA	NA		NA	NA	NA	NA	MmugDNA.16908.1.S1_x_at_NA
MmugDNA.16909.1.S1_at	213175_s_at	SNRPB	small nuclear ribonucleoprotein polypeptides B and B1	chr20p13		6628	SNRPB	small nuclear ribonucleoprotein polypeptides B and B1	---	716578	MmugDNA.16909.1.S1_at_SNRPB
MmugDNA.16910.1.S1_at	201997_s_at	SPEN	spen homolog, transcriptional regulator (Drosophila)	chr1p36.33-p36.11		23013	LOC696205	similar to spen homolog, transcriptional regulator	---	696205	MmugDNA.16910.1.S1_at_SPEN
MmugDNA.16911.1.S1_at	210369_at	SWAP70	SWAP-70 protein	chr11p15		23075	---	---	---	---	MmugDNA.16911.1.S1_at_SWAP70
MmugDNA.1691.1.S1_at	239246_at	FARP1	FERM, RhoGEF (ARHGEF) and pleckstrin domain protein 1 (chondrocyte-derived)	chr13q32.2		10160	---	---	---	---	MmugDNA.1691.1.S1_at_FARP1
MmugDNA.16912.1.S1_s_at	204742_s_at	PDS5B	PDS5, regulator of cohesion maintenance, homolog B (S. cerevisiae)	chr13q12.3		23047	---	---	---	---	MmugDNA.16912.1.S1_s_at_PDS5B
MmugDNA.16913.1.S1_at	223034_s_at	C1orf43	chromosome 1 open reading frame 43	chr1q21.2		25912	LOC716453	similar to Protein C1orf43 (Hepatitis C virus NS5A-transactivated protein 4) (Protein NICE-3) (S863-3	---	716453	MmugDNA.16913.1.S1_at_C1orf43
MmugDNA.16914.1.S1_at	232382_s_at	PCMTD1	protein-L-isoaspartate (D-aspartate) O-methyltransferase domain containing 1	chr8q11.23		115294	---	---	---	---	MmugDNA.16914.1.S1_at_PCMTD1
MmugDNA.16915.1.S1_at	1559707_at	---	---	---	---		---	---	---	---	MmugDNA.16915.1.S1_at_---
MmugDNA.16916.1.S1_at	223994_s_at	SLC12A9	solute carrier family 12 (potassium/chloride transporters), member 9	chr7q22		56996	LOC713119	similar to solute carrier family 12 (potassium/chloride transporters), member 9	---	713119	MmugDNA.16916.1.S1_at_SLC12A9
MmugDNA.16917.1.S1_at	1562675_at	LOC100128003	hypothetical protein LOC100128003	chr1p36.33		100128003	---	---	---	---	MmugDNA.16917.1.S1_at_LOC100128(
MmugDNA.16918.1.S1_at	234344_at	RAP2C	RAP2C, member of RAS oncogene family	chrXq25		57826	---	---	---	---	MmugDNA.16918.1.S1_at_RAP2C
MmugDNA.16919.1.S1_at	1554950_at	ACAN	aggrecan	chr15q26.1		176	AGC1	aggrecan 1	---	698498	MmugDNA.16919.1.S1_at_ACAN
MmugDNA.169.1.S1_at	216720_at	CYP2U1	cytochrome P450, family 2, subfamily U, polypeptide 1	chr4q25		113612	---	---	---	---	MmugDNA.169.1.S1_at_CYP2U1
MmugDNA.16920.1.S1_at	206719_at	SYNGR4	synaptogyrin 4	chr19q13.3		23546	LOC717913	similar to synaptogyrin 4	---	717913	MmugDNA.16920.1.S1_at_SYNGR4
MmugDNA.16921.1.S1_s_at	217855_x_at	SDF4	stromal cell derived factor 4	chr1p36.33		51150	SDF4	stromal cell derived factor 4	---	699804	MmugDNA.16921.1.S1_s_at_SDF4
MmugDNA.1692.1.S1_at	1566461_at	---	---	---	---		---	---	---	---	MmugDNA.1692.1.S1_at_---
MmugDNA.16922.1.S1_at	1563061_at	---	---	---	---		---	---	---	---	MmugDNA.16922.1.S1_at_---
MmugDNA.16923.1.S1_at	NA	NA	NA	NA	NA		NA	NA	NA	NA	MmugDNA.16923.1.S1_at_NA
MmugDNA.16924.1.S1_at	232236_at	---	---	---	---		---	---	---	---	MmugDNA.16924.1.S1_at_---
MmugDNA.16925.1.S1_at	1560075_at	ZNF622	Zinc finger protein 622	chr5p15.1		90441	---	---	---	---	MmugDNA.16925.1.S1_at_ZNF622

MmugDNA.16926.1.S1_at	243233_at	---	---	---	---	---	---	---	---	MmugDNA.16926.1.S1_at_---
MmugDNA.16927.1.S1_at	208456_s_at	RRAS2	related RAS viral (r-ras) oncogene homolog 2	chr11p15.2	22800	LOC699700	similar to related RAS viral (r-ras) oncogene homolog 2 (predicted)	---	699700	MmugDNA.16927.1.S1_at_RRAS2
MmugDNA.16928.1.S1_at	224922_at	CSNK2A2	casein kinase 2, alpha prime polypeptide	chr16q21	1459	---	---	---	---	MmugDNA.16928.1.S1_at_CSNK2A2
MmugDNA.16929.1.S1_at	1555384_a_at	LARP4	La ribonucleoprotein domain family, member 4	chr12q13.13	113251	LOC693337	similar to c-Mpl binding protein isoform c	---	693337	MmugDNA.16929.1.S1_at_LARP4
MmugDNA.16930.1.S1_x_at	211389_x_at	KIR3DS1	killer cell immunoglobulin-like receptor, three domains, short cytoplasmic tail, 1	chr19q13.4	3813	KIR2DL5 /// KIR3DL /// KI	killer immunoglobulin-like receptor KIR2DL5 /// killer immunoglobulin-like receptor KIR3DL /// killer imı	---	100125559 /// 100125575	MmugDNA.16930.1.S1_x_at_KIR3DS1
MmugDNA.16931.1.S1_at	1558600_a_at	---	---	---	---	---	---	---	---	MmugDNA.16931.1.S1_at_---
MmugDNA.1693.1.S1_at	1561676_at	---	---	---	---	---	---	---	---	MmugDNA.1693.1.S1_at_---
MmugDNA.16932.1.S1_at	1570213_at	---	---	---	---	---	---	---	---	MmugDNA.16932.1.S1_at_---
MmugDNA.16933.1.S1_at	1570267_at	---	---	---	---	---	---	---	---	MmugDNA.16933.1.S1_at_---
MmugDNA.16934.1.S1_at	214492_at	SGCD	sarcoglycan, delta (35kDa dystrophin-associated glycoprotein)	chr5q33-q34	6444	LOC715190	similar to delta-sarcoglycan isoform 2	---	715190	MmugDNA.16934.1.S1_at_SGCD
MmugDNA.16935.1.S1_at	215929_at	LOC283079	hypothetical protein LOC283079	chr10p12.1	283079	---	---	---	---	MmugDNA.16935.1.S1_at_LOC283079
MmugDNA.16936.1.S1_at	1554643_at	RGS11	regulator of G-protein signaling 11	chr16p13.3	8786	---	---	---	---	MmugDNA.16936.1.S1_at_RGS11
MmugDNA.16937.1.S1_at	225699_at	C7orf40	chromosome 7 open reading frame 40	chr7p13	285958	---	---	---	---	MmugDNA.16937.1.S1_at_C7orf40
MmugDNA.16938.1.S1_at	229073_at	PRTG	protogenin homolog (Gallus gallus)	chr15q21.3	283659	---	---	---	---	MmugDNA.16938.1.S1_at_PRTG
MmugDNA.16939.1.S1_at	242359_at	---	---	---	---	---	---	---	---	MmugDNA.16939.1.S1_at_---
MmugDNA.16940.1.S1_at	218264_at	BCCIP	BRCA2 and CDKN1A interacting protein	chr10q26.1	56647	---	---	---	---	MmugDNA.16940.1.S1_at_BCCIP
MmugDNA.16941.1.S1_at	237918_at	---	---	---	---	---	---	---	---	MmugDNA.16941.1.S1_at_---
MmugDNA.1694.1.S1_at	224621_at	MAPK1	mitogen-activated protein kinase 1	chr22q11.2|22q11.21	5594	---	---	---	---	MmugDNA.1694.1.S1_at_MAPK1
MmugDNA.16942.1.S1_at	236856_x_at	---	---	---	---	---	---	---	---	MmugDNA.16942.1.S1_at_---
MmugDNA.16943.1.S1_at	227173_s_at	BACH2	BTB and CNC homology 1, basic leucine zipper transcription factor 2	chr6q15	60468	---	---	---	---	MmugDNA.16943.1.S1_at_BACH2
MmugDNA.16944.1.S1_at	229392_s_at	PIK3R2	Phosphoinositide-3-kinase, regulatory subunit 2 (beta)	chr19q13.2-q13.4	5296	---	---	---	---	MmugDNA.16944.1.S1_at_PIK3R2
MmugDNA.16945.1.S1_at	230064_at	---	---	---	---	---	---	---	---	MmugDNA.16945.1.S1_at_---
MmugDNA.16946.1.S1_at	215202_at	LOC91316	glucuronidase, beta/ immunoglobulin lambda-like polypeptide 1 pseudogene	chr22q11.23	91316	---	---	---	---	MmugDNA.16946.1.S1_at_LOC91316
MmugDNA.16947.1.S1_at	213466_at	RAB40C	RAB40C, member RAS oncogene family	chr16p13.3	57799	LOC722336	similar to Rab40c, member RAS oncogene family	---	722336	MmugDNA.16947.1.S1_at_RAB40C
MmugDNA.16948.1.S1_at	211695_x_at	MUC1	mucin 1, cell surface associated	chr1q21	4582	LOC717546	similar to MUC1 mucin isoform 1 precursor	---	717546	MmugDNA.16948.1.S1_at_MUC1
MmugDNA.16949.1.S1_at	243987_at	---	---	---	---	---	---	---	---	MmugDNA.16949.1.S1_at_---
MmugDNA.16950.1.S1_at	220294_at	KCNV1	potassium channel, subfamily V, member 1	chr8q22.3-q24.1	27012	---	---	---	---	MmugDNA.16950.1.S1_at_KCNV1
MmugDNA.16951.1.S1_at	232790_at	---	---	---	---	---	---	---	---	MmugDNA.16951.1.S1_at_---
MmugDNA.1695.1.S1_at	NA	NA	NA	NA	NA	NA	NA	NA	NA	MmugDNA.1695.1.S1_at_NA
MmugDNA.16952.1.S1_at	232101_s_at	PIGN	phosphatidylinositol glycan anchor biosynthesis, class N	chr18q21.33	23556	LOC699219	similar to phosphatidylinositol glycan, class N	---	699219	MmugDNA.16952.1.S1_at_PIGN
MmugDNA.16954.1.S1_at	235550_at	MAP9	microtubule-associated protein 9	chr4q32.1	79884	LOC699247	hypothetical protein LOC699247	---	699247	MmugDNA.16954.1.S1_at_MAP9
MmugDNA.16955.1.S1_at	NA	NA	NA	NA	NA	NA	NA	NA	NA	MmugDNA.16955.1.S1_at_NA
MmugDNA.16955.1.S1_s_at	224640_at	UNQ1887	signal peptide peptidase 3	chr12q24.31	121665	SPPL3	signal peptide peptidase 3	---	700898	MmugDNA.16955.1.S1_s_at_UNQ1887
MmugDNA.16956.1.S1_at	207148_x_at	MYOZ2	myozenin 2	chr4q26-q27	51778	MYOZ2	myozenin 2	---	705000	MmugDNA.16956.1.S1_at_MYOZ2
MmugDNA.16956.1.S1_s_at	213782_s_at	MYOZ2	myozenin 2	chr4q26-q27	51778	MYOZ2	myozenin 2	---	705000	MmugDNA.16956.1.S1_s_at_MYOZ2
MmugDNA.16957.1.S1_at	224639_at	UNQ1887	signal peptide peptidase 3	chr12q24.31	121665	SPPL3	signal peptide peptidase 3	---	700898	MmugDNA.16957.1.S1_at_UNQ1887
MmugDNA.16958.1.S1_at	224638_at	UNQ1887	signal peptide peptidase 3	chr12q24.31	121665	SPPL3	signal peptide peptidase 3	---	700898	MmugDNA.16958.1.S1_at_UNQ1887
MmugDNA.16959.1.S1_s_at	201643_x_at	KDM3B	lysine (K)-specific demethylase 3B	chr5q31	51780	LOC716648	similar to jumonji domain containing 1B	---	716648	MmugDNA.16959.1.S1_s_at_KDM3B
MmugDNA.16960.1.S1_at	240637_at	WDR41	WD repeat domain 41	chr5q14.1	55255	---	---	---	---	MmugDNA.16960.1.S1_at_WDR41
MmugDNA.16961.1.S1_at	1556864_at	TCTN1	tectonic family member 1	chr12q24.11	79600	---	---	---	---	MmugDNA.16961.1.S1_at_TCTN1
MmugDNA.1696.1.S1_at	234023_s_at	CENPJ	centromere protein J	chr13q12.12	55835	CENPJ	centromere protein J	---	721498	MmugDNA.1696.1.S1_at_CENPJ
MmugDNA.16962.1.S1_at	235645_at	ESCO1	establishment of cohesion 1 homolog 1 (S. cerevisiae)	chr18q11.2	114799	ESCO1	establishment of cohesion 1 homolog 1 (S. cerevisiae)	---	698845	MmugDNA.16962.1.S1_at_ESCO1
MmugDNA.16963.1.S1_at	1554560_at	PGM5	phosphoglucomutase 5	chr9q13	5239	---	---	---	---	MmugDNA.16963.1.S1_at_PGM5
MmugDNA.16964.1.S1_at	236815_at	---	---	---	---	---	---	---	---	MmugDNA.16964.1.S1_at_---
MmugDNA.16965.1.S1_at	220428_at	CD207	CD207 molecule, langerin	chr2p13	50489	CD207	CD207 molecule, langerin	---	703420	MmugDNA.16965.1.S1_at_CD207
MmugDNA.16966.1.S1_at	1557465_at	RP11-327P2.4	hypothetical LOC283521	chr13q14.3	283521	LOC713644	hypothetical protein LOC713644	---	713644	MmugDNA.16966.1.S1_at_RP11-327P2
MmugDNA.16967.1.S1_at	1564499_at	C14orf81	chromosome 14 open reading frame 81	chr14q32.33	153514	---	---	---	---	MmugDNA.16967.1.S1_at_C14orf81
MmugDNA.16968.1.S1_at	1553051_s_at	ODF3	outer dense fiber of sperm tails 3	chr11p15.5	113746	ODF3	outer dense fiber of sperm tails 3	---	696480	MmugDNA.16968.1.S1_at_ODF3
MmugDNA.16969.1.S1_at	1558702_at	TEX10	Testis expressed 10	chr9q31.1	54881	LOC714490	similar to inversin isoform b	---	714490	MmugDNA.16969.1.S1_at_TEX10
MmugDNA.16970.1.S1_at	228812_at	---	---	---	---	---	---	---	---	MmugDNA.16970.1.S1_at_---
MmugDNA.16971.1.S1_at	1567055_at	OR1C1	olfactory receptor, family 1, subfamily C, member 1	chr1q44	26188	LOC708042	similar to olfactory receptor, family 1, subfamily C, member 1	---	708042	MmugDNA.16971.1.S1_at_OR1C1
MmugDNA.1697.1.S1_at	1557938_s_at	PTRF	polymerase I and transcript release factor	chr17q21.31	284119	---	---	---	---	MmugDNA.1697.1.S1_at_PTRF
MmugDNA.16972.1.S1_at	1553527_at	NLRP9	NLR family, pyrin domain containing 9	chr19q13.42	338321	---	---	---	---	MmugDNA.16972.1.S1_at_NLRP9
MmugDNA.16973.1.S1_at	244744_at	LOC100130502	hypothetical LOC100130502	chr2p21	100130502	---	---	---	---	MmugDNA.16973.1.S1_at_LOC100130!
MmugDNA.16974.1.S1_at	NA	NA	NA	NA	NA	NA	NA	NA	NA	MmugDNA.16974.1.S1_at_NA
MmugDNA.16975.1.S1_at	204901_at	BTRC	beta-transducin repeat containing	chr10q24.32	8945	LOC696403	Similar to aldo-keto reductase family 1, member B10	---	696403	MmugDNA.16975.1.S1_at_BTRC
MmugDNA.16976.1.S1_at	240959_at	---	---	---	---	---	---	---	---	MmugDNA.16976.1.S1_at_---
MmugDNA.16977.1.S1_at	1556012_at	KLHDC7A	kelch domain containing 7A	chr1p36.13	127707	---	---	---	---	MmugDNA.16977.1.S1_at_KLHDC7A
MmugDNA.16978.1.S1_at	234689_at	C6orf138	chromosome 6 open reading frame 138	chr6p12.3	442213	LOC708060	similar to Ptc-related CG11212-PA	---	708060	MmugDNA.16978.1.S1_at_C6orf138
MmugDNA.16979.1.S1_at	239539_at	---	---	---	---	---	---	---	---	MmugDNA.16979.1.S1_at_---
MmugDNA.16980.1.S1_at	1552615_at	ACACB	acetyl-Coenzyme A carboxylase beta	chr12q24.11	32	LOC706909	similar to acetyl-Coenzyme A carboxylase beta	---	706909	MmugDNA.16980.1.S1_at_ACACB
MmugDNA.16981.1.S1_at	234449_at	---	---	---	---	---	---	---	---	MmugDNA.16981.1.S1_at_---
MmugDNA.1698.1.S1_at	207622_s_at	ABCF2	ATP-binding cassette, sub-family F (GCN20), member 2	chr7q36	10061	LOC714518	similar to ATP-binding cassette, sub-family F, member 2 isoform b	---	714518	MmugDNA.1698.1.S1_at_ABCF2
MmugDNA.16982.1.S1_at	1552381_at	SRrp35	serine-arginine repressor protein (35 kDa)	chr6q15	135295	SRRP35	serine-arginine repressor protein (35 kDa)	---	700497	MmugDNA.16982.1.S1_at_SRrp35
MmugDNA.16983.1.S1_at	1553249_at	SMC1B	structural maintenance of chromosomes 1B	chr22q13.31	27127	LOC713486	similar to SMC1 structural maintenance of chromosomes 1-like 2	---	713486	MmugDNA.16983.1.S1_at_SMC1B
MmugDNA.16984.1.S1_at	NA	NA	NA	NA	NA	NA	NA	NA	NA	MmugDNA.16984.1.S1_at_NA
MmugDNA.16984.1.S1_s_at	NA	NA	NA	NA	NA	NA	NA	NA	NA	MmugDNA.16984.1.S1_s_at_NA
MmugDNA.16985.1.S1_at	206501_x_at	ETV1	ets variant 1	chr7p21.3	2115	ETV1	ets variant gene 1	---	710318	MmugDNA.16985.1.S1_at_ETV1
MmugDNA.16986.1.S1_at	1561137_s_at	GYPE	glycophorin E	chr4q31.1	2996	GYPA	glycophorin A	---	574101	MmugDNA.16986.1.S1_at_GYPE
MmugDNA.16988.1.S1_at	227991_x_at	ZBTB43	zinc finger and BTB domain containing 43	chr9q33-q34	23099	ZBTB43	zinc finger and BTB domain containing 43	---	701797	MmugDNA.16988.1.S1_at_ZBTB43
MmugDNA.16989.1.S1_at	230892_at	---	---	---	---	---	---	---	---	MmugDNA.16989.1.S1_at_---
MmugDNA.16990.1.S1_at	1570238_at	ZNF527	zinc finger protein 527	chr19q13.1	84503	---	---	---	---	MmugDNA.16990.1.S1_at_ZNF527
MmugDNA.16991.1.S1_at	209663_s_at	ITGA7	integrin, alpha 7	chr12q13	3679	LOC707279	similar to integrin alpha 7 precursor	---	707279	MmugDNA.16991.1.S1_at_ITGA7
MmugDNA.1699.1.S1_at	206177_s_at	ARG1	arginase, liver	chr6q23	383	LOC710410	similar to arginase, type I	---	710410	MmugDNA.1699.1.S1_at_ARG1
MmugDNA.16992.1.S1_at	231382_at	FGF18	Fibroblast growth factor 18	chr5q34	8817	LOC702072	similar to Fibroblast growth factor 18 precursor (FGF-18)	---	702072	MmugDNA.16992.1.S1_at_FGF18
MmugDNA.16993.1.S1_at	213437_at	RUFY3	RUN and FYVE domain containing 3	chr4q13.3	22902	RUFY3	RUN and FYVE domain containing 3	---	706417	MmugDNA.16993.1.S1_at_RUFY3
MmugDNA.16994.1.S1_at	238080_at	B4GALNT4	beta-1,4-N-acetyl-galactosaminyl transferase 4	chr11p15.5	338707	LOC720768	similar to beta1,4-N-acetylgalactosaminyltransferases IV	---	720768	MmugDNA.16994.1.S1_at_B4GALNT4
MmugDNA.16995.1.S1_at	215229_at	LOC100129973	hypothetical protein LOC100129973	chr15q21.2	100129973	---	---	---	---	MmugDNA.16995.1.S1_at_LOC100129!
MmugDNA.16996.1.S1_at	213430_at	RUFY3	RUN and FYVE domain containing 3	chr4q13.3	22902	RUFY3	RUN and FYVE domain containing 3	---	706417	MmugDNA.16996.1.S1_at_RUFY3
MmugDNA.16997.1.S1_at	236379_at	---	---	---	---	---	---	---	---	MmugDNA.16997.1.S1_at_---
MmugDNA.16998.1.S1_at	232279_at	PHF15	PHD finger protein 15	chr5q31.1	23338	---	---	---	---	MmugDNA.16998.1.S1_at_PHF15
MmugDNA.16999.1.S1_at	234199_at	---	---	---	---	---	---	---	---	MmugDNA.16999.1.S1_at_---
MmugDNA.17000.1.S1_at	244473_at	---	---	---	---	---	---	---	---	MmugDNA.17000.1.S1_at_---
MmugDNA.17001.1.S1_at	215227_x_at	ACP1	acid phosphatase 1, soluble	chr2p25	52	---	---	---	---	MmugDNA.17001.1.S1_at_ACP1
MmugDNA.1700.1.S1_at	217760_at	TRIM44	tripartite motif-containing 44	chr11p13	54765	LOC711615	Hypothetical protein LOC711615	---	711615	MmugDNA.1700.1.S1_at_TRIM44
MmugDNA.17002.1.S1_at	202783_at	NNT	nicotinamide nucleotide transhydrogenase	chr5p13.1-cen	23530	NNT	nicotinamide nucleotide transhydrogenase	---	701110	MmugDNA.17002.1.S1_at_NNT
MmugDNA.17003.1.S1_at	244874_at	---	---	---	---	---	---	---	---	MmugDNA.17003.1.S1_at_---
MmugDNA.17004.1.S1_at	202945_at	FPGS	folylpolyglutamate synthase	chr9q34.1	2356	FPGS	folylpolyglutamate synthase	---	699587	MmugDNA.17004.1.S1_at_FPGS
MmugDNA.17005.1.S1_at	212850_s_at	LRP4	low density lipoprotein receptor-related protein 4	chr11p11.2-p12	4038	LOC714038	similar to low density lipoprotein receptor-related protein 4	---	714038	MmugDNA.17005.1.S1_at_LRP4
MmugDNA.17006.1.S1_at	203577_at	GTF2H4	general transcription factor IIH, polypeptide 4, 52kDa	chr6p21.3	2968	GTF2H4	general transcription factor IIH, polypeptide 4, 52kDa	---	714285	MmugDNA.17006.1.S1_at_GTF2H4
MmugDNA.17007.1.S1_at	1564539_at	LOC647323	hypothetical protein LOC647323	chr3q29	647323	---	---	---	---	MmugDNA.17007.1.S1_at_LOC647323
MmugDNA.17008.1.S1_at	211315_s_at	CACNA1G	calcium channel, voltage-dependent, T type, alpha 1G subunit	chr17q22	8913	---	---	---	---	MmugDNA.17008.1.S1_at_CACNA1G
MmugDNA.17009.1.S1_at	201065_s_at	GTF2I /// GTF2IP1 /// L	general transcription factor II, i /// general transcription factor II, i, pseudogene 1 /// general transcripti	chr7q11.23	100093631 /// 2969 ///	LOC714356	Hypothetical protein LOC714356	---	714356	MmugDNA.17009.1.S1_at_GTF2I /// GT
MmugDNA.17010.1.S1_at	200753_x_at	SFRS2	splicing factor, arginine/serine-rich 2	chr17q25.2	6427	LOC701187	Similar to high-mobility group box 3	---	701187	MmugDNA.17010.1.S1_at_SFRS2
MmugDNA.17011.1.S1_at	242909_at	---	---	---	---	---	---	---	---	MmugDNA.17011.1.S1_at_---
MmugDNA.1701.1.S1_at	230498_at	MCHR1	melanin-concentrating hormone receptor 1	chr22q13.2	2847	---	---	---	---	MmugDNA.1701.1.S1_at_MCHR1
MmugDNA.17012.1.S1_at	1558397_at	---	---	---	---	---	---	---	---	MmugDNA.17012.1.S1_at_---
MmugDNA.17013.1.S1_at	207003_at	GUCA2A	guanylate cyclase activator 2A (guanylin)	chr1p35-p34	2980	GUCA2A	guanylate cyclase activator 2A (guanylin)	---	696455	MmugDNA.17013.1.S1_at_GUCA2A
MmugDNA.17014.1.S1_at	1568888_at	---	---	---	---	---	---	---	---	MmugDNA.17014.1.S1_at_---
MmugDNA.17015.1.S1_at	238897_at	LRRC28	Leucine rich repeat containing 28	chr15q26.3	123355	---	---	---	---	MmugDNA.17015.1.S1_at_LRRC28
MmugDNA.17016.1.S1_at	NA	NA	NA	NA	NA	NA	NA	NA	NA	MmugDNA.17016.1.S1_at_NA
MmugDNA.17017.1.S1_at	242128_at	OTX2	orthodenticle homeobox 2	chr14q21-q22	5015	---	---	---	---	MmugDNA.17017.1.S1_at_OTX2
MmugDNA.17018.1.S1_s_at	203944_x_at	BTN2A1	butyrophilin, subfamily 2, member A1	chr6p22.1	11120	LOC700495	similar to butyrophilin, subfamily 2, member A1 isoform 1 precursor	---	700495	MmugDNA.17018.1.S1_s_at_BTN2A1
MmugDNA.17019.1.S1_at	1560163_at	LOC100129510	hypothetical protein LOC100129510	chr17q25.3	100129510	---	---	---	---	MmugDNA.17019.1.S1_at_LOC100129!
MmugDNA.170.1.S1_at	208193_at	IL9	interleukin 9	chr5q31.1	3578	LOC712292	similar to interleukin 9 precursor	---	712292	MmugDNA.170.1.S1_at_IL9
MmugDNA.17020.1.S1_at	214216_s_at	LARP5	La ribonucleoprotein domain family, member 5	chr10p15.3	23185	---	---	---	---	MmugDNA.17020.1.S1_at_LARP5
MmugDNA.17021.1.S1_at	208684_at	COPA	coatomer protein complex, subunit alpha	chr1q23-q25	1314	LOC707839	Hypothetical protein LOC707839	---	707839	MmugDNA.17021.1.S1_at_COPA
MmugDNA.1702.1.S1_at	206034_at	SERPINB8	serpin peptidase inhibitor, clade B (ovalbumin), member 8	chr18q21.3	5271	SERPINB6	serpin peptidase inhibitor, clade B (ovalbumin), member 6	---	702046	MmugDNA.1702.1.S1_at_SERPINB8

MmugDNA.17022.1.S1_at	241321_at	---	---	---	---		---	---	---	---		MmugDNA.17022.1.S1_at_---
MmugDNA.17023.1.S1_at	241587_at	---	---	---	---		---	---	---	---		MmugDNA.17023.1.S1_at_---
MmugDNA.17024.1.S1_at	241761_at	---	---	---	---		---	---	---	---		MmugDNA.17024.1.S1_at_---
MmugDNA.17025.1.S1_at	218467_at	PSMG2	proteasome (prosome, macropain) assembly chaperone 2	chr18p11.21		56984	LOC717110	similar to tumor necrosis factor superfamily, member 5-induced protein 1	---		717110	MmugDNA.17025.1.S1_at_PSMG2
MmugDNA.17026.1.S1_at	244667_at	---	---	---	---		---	---	---	---		MmugDNA.17026.1.S1_at_---
MmugDNA.17027.1.S1_at	212535_at	MEF2A	myocyte enhancer factor 2A	chr15q26		4205	MEF2A	myocyte enhancer factor 2A	---		708956	MmugDNA.17027.1.S1_at_MEF2A
MmugDNA.17028.1.S1_s_at	220606_s_at	C17orf48	chromosome 17 open reading frame 48	chr17p13.1		56985	LOC717991	hypothetical protein LOC717991	---		717991	MmugDNA.17028.1.S1_s_at_C17orf48
MmugDNA.17029.1.S1_at	200959_at	FUS	fusion (involved in t(12;16) in malignant liposarcoma)	chr16p11.2		2521	LOC713433	similar to fusion (involved in t(12;16) in malignant liposarcoma) isoform a	---		713433	MmugDNA.17029.1.S1_at_FUS
MmugDNA.17029.1.S1_s_at	NA	NA	NA	NA	NA		NA	NA	NA	NA		MmugDNA.17029.1.S1_s_at_NA
MmugDNA.17030.1.S1_at	238138_at	CBY3	Chibby homolog 3 (Drosophila)	chr5q35.3		646019	---	---	---	---		MmugDNA.17030.1.S1_at_CBY3
MmugDNA.17031.1.S1_at	219291_at	DTWD1	DTW domain containing 1	chr15q21.2		56986	LOC715084	similar to DTW domain containing 1	---		715084	MmugDNA.17031.1.S1_at_DTWD1
MmugDNA.1703.1.S1_at	227193_at	---	---	---	---		---	---	---	---		MmugDNA.1703.1.S1_at_---
MmugDNA.17032.1.S1_at	201637_s_at	FXR1	fragile X mental retardation, autosomal homolog 1	chr3q28		8087	---	---	---	---		MmugDNA.17032.1.S1_at_FXR1
MmugDNA.17033.1.S1_at	218587_s_at	KTELC1	KTEL (Lys-Tyr-Glu-Leu) containing 1	chr3q13.33		56983	LOC712357	similar to RIKEN cDNA 9630046K23	---		712357	MmugDNA.17033.1.S1_at_KTELC1
MmugDNA.17034.1.S1_at	206927_s_at	GUCY1A2	guanylate cyclase 1, soluble, alpha 2	chr11q21-q22		2977	LOC706615	similar to guanylate cyclase 1, soluble, alpha 2	---		706615	MmugDNA.17034.1.S1_at_GUCY1A2
MmugDNA.17035.1.S1_at	1569948_at	---	---	---	---		---	---	---	---		MmugDNA.17035.1.S1_at_---
MmugDNA.17036.1.S1_at	221150_at	MEPE	matrix extracellular phosphoglycoprotein	chr4q21.1		56955	MEPE	matrix extracellular phosphoglycoprotein	---		701943	MmugDNA.17036.1.S1_at_MEPE
MmugDNA.17037.1.S1_at	241308_at	LOC729589	hypothetical LOC729589	chr18p11.21		729589	---	---	---	---		MmugDNA.17037.1.S1_at_LOC729589
MmugDNA.17038.1.S1_at	207884_at	GUCY2D	guanylate cyclase 2D, membrane (retina-specific)	chr17p13.1		3000	GUCY2D	guanylate cyclase 2D, membrane (retina-specific)	---		716734	MmugDNA.17038.1.S1_at_GUCY2D
MmugDNA.17039.1.S1_at	208053_at	GUCY2F	guanylate cyclase 2F, retinal	chrXq22		2986	GUCY2F	guanylate cyclase 2F	---		704485	MmugDNA.17039.1.S1_at_GUCY2F
MmugDNA.17040.1.S1_at	235846_at	---	---	---	---		---	---	---	---		MmugDNA.17040.1.S1_at_---
MmugDNA.17041.1.S1_at	241110_at	---	---	---	---		---	---	---	---		MmugDNA.17041.1.S1_at_---
MmugDNA.1704.1.S1_at	NA	NA	NA	NA	NA		NA	NA	NA	NA		MmugDNA.1704.1.S1_at_NA
MmugDNA.17042.1.S1_at	235960_at	---	---	---	---		---	---	---	---		MmugDNA.17042.1.S1_at_---
MmugDNA.17043.1.S1_at	241846_at	---	---	---	---		---	---	---	---		MmugDNA.17043.1.S1_at_---
MmugDNA.17044.1.S1_at	238650_x_at	WDR89	WD repeat domain 89	chr14q23.2		112840	---	---	---	---		MmugDNA.17044.1.S1_at_WDR89
MmugDNA.17045.1.S1_at	243925_at	---	---	---	---		---	---	---	---		MmugDNA.17045.1.S1_at_---
MmugDNA.17046.1.S1_at	1569765_at	---	---	---	---		---	---	---	---		MmugDNA.17046.1.S1_at_---
MmugDNA.17047.1.S1_at	1563112_at	FAM120AOS	Family with sequence similarity 120A opposite strand	chr9q22.31		158293	---	---	---	---		MmugDNA.17047.1.S1_at_FAM120AOS
MmugDNA.17048.1.S1_at	225760_at	MYSM1	Myb-like, SWIRM and MPN domains 1	chr1p32.1		114803	---	---	---	---		MmugDNA.17048.1.S1_at_MYSM1
MmugDNA.17049.1.S1_at	207033_at	GIF	gastric intrinsic factor (vitamin B synthesis)	chr11q13		2694	GIF	gastric intrinsic factor (vitamin B synthesis)	---		698704	MmugDNA.17049.1.S1_at_GIF
MmugDNA.17050.1.S1_at	206896_s_at	GNG7	guanine nucleotide binding protein (G protein), gamma 7	chr19p13.3		2788	LOC711335	similar to guanine nucleotide binding protein (G protein), gamma 7	---		711335	MmugDNA.17050.1.S1_at_GNG7
MmugDNA.17051.1.S1_at	218241_at	GOLGA5	golgi autoantigen, golgin subfamily a, 5	chr14q32.12-q32.13		9950	---	---	---	---		MmugDNA.17051.1.S1_at_GOLGA5
MmugDNA.1705.1.S1_at	1556429_a_at	WDR67	WD repeat domain 67	chr8q24.13		93594	---	---	---	---		MmugDNA.1705.1.S1_at_WDR67
MmugDNA.17052.1.S1_at	242750_at	MMAA	methylmalonic aciduria (cobalamin deficiency) cblA type	chr4q31.22		166785	---	---	---	---		MmugDNA.17052.1.S1_at_MMAA
MmugDNA.17053.1.S1_at	241734_at	SRFBP1	serum response factor binding protein 1	chr5q23.1		153443	SRFBP1	serum response factor binding protein 1	---		699872	MmugDNA.17053.1.S1_at_SRFBP1
MmugDNA.17054.1.S1_at	234435_at	---	---	---	---		---	---	---	---		MmugDNA.17054.1.S1_at_---
MmugDNA.17055.1.S1_at	1570089_at	---	---	---	---		---	---	---	---		MmugDNA.17055.1.S1_at_---
MmugDNA.17056.1.S1_at	228550_at	RTN4R	reticulon 4 receptor	chr22q11.21		65078	RTN4R	reticulon 4 receptor	---		694382	MmugDNA.17056.1.S1_at_RTN4R
MmugDNA.17056.1.S1_s_at	NA	NA	NA	NA	NA		NA	NA	NA	NA		MmugDNA.17056.1.S1_s_at_NA
MmugDNA.17057.1.S1_at	209227_at	TUSC3	tumor suppressor candidate 3	chr8p22		7991	LOC694043	Similar to RECK protein precursor	---		694043	MmugDNA.17057.1.S1_at_TUSC3
MmugDNA.17057.1.S1_s_at	209228_x_at	TUSC3	tumor suppressor candidate 3	chr8p22		7991	TUSC3	tumor suppressor candidate 3	---		701123	MmugDNA.17057.1.S1_s_at_TUSC3
MmugDNA.17058.1.S1_at	NA	NA	NA	NA	NA		NA	NA	NA	NA		MmugDNA.17058.1.S1_at_NA
MmugDNA.17060.1.S1_s_at	242338_at	TMEM64	transmembrane protein 64	chr8q21.3		169200	LOC695826	similar to transmembrane protein 64	---		695826	MmugDNA.17060.1.S1_s_at_TMEM64
MmugDNA.17061.1.S1_at	1566931_at	TFB2M	Transcription factor B2, mitochondrial	chr1q44		64216	---	---	---	---		MmugDNA.17061.1.S1_at_TFB2M
MmugDNA.1706.1.S1_at	1554397_s_at	UEVLD	UEV and lactate/malate dehyrogenase domains	chr11p15.1		55293	---	---	---	---		MmugDNA.1706.1.S1_at_UEVLD
MmugDNA.17062.1.S1_at	242921_at	SGSM1	small G protein signaling modulator 1	chr22q11.23		129049	LOC714057	similar to RUN and TBC1 domain containing 2	---		714057	MmugDNA.17062.1.S1_at_SGSM1
MmugDNA.17063.1.S1_at	240288_at	KCNRG	potassium channel regulator	chr13q14.3		283518	LOC707341	similar to potassium channel regulator isoform 1	---		707341	MmugDNA.17063.1.S1_at_KCNRG
MmugDNA.17064.1.S1_at	237022_at	---	---	---	---		---	---	---	---		MmugDNA.17064.1.S1_at_---
MmugDNA.17065.1.S1_at	221508_at	TAOK3	TAO kinase 3	chr12q		51347	TAOK3	TAO kinase 3	---		677710	MmugDNA.17065.1.S1_at_TAOK3
MmugDNA.17066.1.S1_at	226698_at	FCHSD1	FCH and double SH3 domains 1	chr5q31.3		89848	FCHSD1	similar to FCH and double SH3 domains 1	---		701267	MmugDNA.17066.1.S1_at_FCHSD1
MmugDNA.17067.1.S1_at	242642_at	---	---	---	---		---	---	---	---		MmugDNA.17067.1.S1_at_---
MmugDNA.17068.1.S1_at	226699_at	FCHSD1	FCH and double SH3 domains 1	chr5q31.3		89848	---	---	---	---		MmugDNA.17068.1.S1_at_FCHSD1
MmugDNA.17069.1.S1_at	222619_at	ZNF281	zinc finger protein 281	chr1q32.1		23528	---	---	---	---		MmugDNA.17069.1.S1_at_ZNF281
MmugDNA.17070.1.S1_at	1560286_s_at	---	---	---	---		---	---	---	---		MmugDNA.17070.1.S1_at_---
MmugDNA.17071.1.S1_at	230978_at	---	---	---	---		---	---	---	---		MmugDNA.17071.1.S1_at_---
MmugDNA.1707.1.S1_at	205077_s_at	PIGF	phosphatidylinositol glycan anchor biosynthesis, class F	chr2p21-p16		5281	LOC714844	similar to phosphatidylinositol glycan, class F isoform 1	---		714844	MmugDNA.1707.1.S1_at_PIGF
MmugDNA.17072.1.S1_at	NA	NA	NA	NA	NA		NA	NA	NA	NA		MmugDNA.17072.1.S1_at_NA
MmugDNA.17072.1.S1_x_at	230029_x_at	UBR3	ubiquitin protein ligase E3 component n-recognin 3 (putative)	chr2q31.1		130507	LOC710524	similar to zinc finger protein 650	---		710524	MmugDNA.17072.1.S1_x_at_UBR3
MmugDNA.17073.1.S1_at	200944_s_at	HMGN1	high-mobility group nucleosome binding domain 1	chr21q22.3|21q22.2		3150	LOC705517	hypothetical protein LOC705517	---		705517	MmugDNA.17073.1.S1_at_HMGN1
MmugDNA.17074.1.S1_at	230262_at	ST8SIA3	ST8 alpha-N-acetyl-neuraminide alpha-2,8-sialyltransferase 3	chr18q21.31		51046	---	---	---	---		MmugDNA.17074.1.S1_at_ST8SIA3
MmugDNA.17075.1.S1_at	208266_at	C8orf17	chromosome 8 open reading frame 17	chr8q24.3		56988	---	---	---	---		MmugDNA.17075.1.S1_at_C8orf17
MmugDNA.17076.1.S1_s_at	207854_at	GYPE	glycophorin E	chr4q31.1		2996	---	---	---	---		MmugDNA.17076.1.S1_s_at_GYPE
MmugDNA.17077.1.S1_at	NA	NA	NA	NA	NA		NA	NA	NA	NA		MmugDNA.17077.1.S1_at_NA
MmugDNA.17078.1.S1_at	242960_at	EPC2	Enhancer of polycomb homolog 2 (Drosophila)	chr2q23.1		26122	---	---	---	---		MmugDNA.17078.1.S1_at_EPC2
MmugDNA.17079.1.S1_at	242353_at	---	---	---	---		---	---	---	---		MmugDNA.17079.1.S1_at_---
MmugDNA.17080.1.S1_at	221072_at	C9orf31	chromosome 9 open reading frame 31	chr9q33.2		57000	---	---	---	---		MmugDNA.17080.1.S1_at_C9orf31
MmugDNA.17081.1.S1_at	NA	NA	NA	NA	NA		NA	NA	NA	NA		MmugDNA.17081.1.S1_at_NA
MmugDNA.1708.1.S1_at	243497_at	---	---	---	---		---	---	---	---		MmugDNA.1708.1.S1_at_---
MmugDNA.17082.1.S1_at	244777_at	DCP2	DCP2 decapping enzyme homolog (S. cerevisiae)	chr5q22.2		167227	---	---	---	---		MmugDNA.17082.1.S1_at_DCP2
MmugDNA.17083.1.S1_at	225371_at	GLE1	GLE1 RNA export mediator homolog (yeast)	chr9q34.11		2733	LOC717474	similar to GLE1-like, RNA export mediator isoform 1	---		717474	MmugDNA.17083.1.S1_at_GLE1
MmugDNA.17084.1.S1_at	206666_at	GZMK	granzyme K (granzyme 3; tryptase II)	chr5q11-q12		3003	---	---	---	---		MmugDNA.17084.1.S1_at_GZMK
MmugDNA.17085.1.S1_at	1553901_x_at	ZNF486	zinc finger protein 486	chr19p12		90649	---	---	---	---		MmugDNA.17085.1.S1_at_ZNF486
MmugDNA.17086.1.S1_at	225313_at	C20orf177	chromosome 20 open reading frame 177	chr20q13.2-q13.33		63939	LOC720556	similar to Protein C20orf177	---		720556	MmugDNA.17086.1.S1_at_C20orf177
MmugDNA.17087.1.S1_at	1556662_at	---	---	---	---		---	---	---	---		MmugDNA.17087.1.S1_at_---
MmugDNA.17089.1.S1_at	NA	NA	NA	NA	NA		NA	NA	NA	NA		MmugDNA.17089.1.S1_at_NA
MmugDNA.17090.1.S1_at	236862_at	GOPC	Golgi associated PDZ and coiled-coil motif containing	chr6q21		57120	---	---	---	---		MmugDNA.17090.1.S1_at_GOPC
MmugDNA.17091.1.S1_at	1556421_at	LOC286189	hypothetical protein LOC286189	chr8q13.1		286189	---	---	---	---		MmugDNA.17091.1.S1_at_LOC286189
MmugDNA.1709.1.S1_at	1556679_at	---	---	---	---		---	---	---	---		MmugDNA.1709.1.S1_at_---
MmugDNA.17092.1.S1_at	228650_at	---	---	---	---		---	---	---	---		MmugDNA.17092.1.S1_at_---
MmugDNA.17093.1.S1_at	206273_at	SLMO1	slowmo homolog 1 (Drosophila)	chr18p11.21		10650	---	---	---	---		MmugDNA.17093.1.S1_at_SLMO1
MmugDNA.17094.1.S1_at	1554589_at	LENG9	leukocyte receptor cluster (LRC) member 9	chr19q13.4		94059	LOC695874	similar to leukocyte receptor cluster (LRC) member 9	---		695874	MmugDNA.17094.1.S1_at_LENG9
MmugDNA.17095.1.S1_at	236548_at	GIPC2	GIPC PDZ domain containing family, member 2	chr1p31.1		54810	---	---	---	---		MmugDNA.17095.1.S1_at_GIPC2
MmugDNA.17096.1.S1_at	244837_at	---	---	---	---		---	---	---	---		MmugDNA.17096.1.S1_at_---
MmugDNA.17097.1.S1_at	1555004_a_at	RBL1	retinoblastoma-like 1 (p107)	chr20q11.2		5933	LOC701650	similar to retinoblastoma-like protein 1 isoform a	---		701650	MmugDNA.17097.1.S1_at_RBL1
MmugDNA.17098.1.S1_at	230808_at	FNTA	farnesyltransferase, CAAX box, alpha	chr8p11		2339	---	---	---	---		MmugDNA.17098.1.S1_at_FNTA
MmugDNA.17099.1.S1_at	213493_at	SNED1	sushi, nidogen and EGF-like domains 1	chr2q37.3		25992	---	---	---	---		MmugDNA.17099.1.S1_at_SNED1
MmugDNA.17100.1.S1_at	205466_s_at	HS3ST1	heparan sulfate (glucosamine) 3-O-sulfotransferase 1	chr4p16		9957	LOC713374	similar to heparan sulfate D-glucosaminyl 3-O-sulfotransferase 1 precursor	---		713374	MmugDNA.17100.1.S1_at_HS3ST1
MmugDNA.17101.1.S1_at	1553069_at	C21orf87	chromosome 21 open reading frame 87	chr21q22.2		257357	---	---	---	---		MmugDNA.17101.1.S1_at_C21orf87
MmugDNA.1710.1.S1_at	1562399_at	---	---	---	---		---	---	---	---		MmugDNA.1710.1.S1_at_---
MmugDNA.17102.1.S1_s_at	207949_s_at	ICA1	islet cell autoantigen 1, 69kDa	chr7p22		3382	ICA1	islet cell autoantigen 1, 69kDa	---		695889	MmugDNA.17102.1.S1_s_at_ICA1
MmugDNA.17104.1.S1_at	1558762_a_at	ZNF789	zinc finger protein 789	chr7q22.1		285989	---	---	---	---		MmugDNA.17104.1.S1_at_ZNF789
MmugDNA.17105.1.S1_at	218557_at	NIT2	nitrilase family, member 2	chr3q12.2		56954	LOC695195	Similar to carnosinase 1	---		695195	MmugDNA.17105.1.S1_at_NIT2
MmugDNA.17106.1.S1_s_at	217437_s_at	TACC1	transforming, acidic coiled-coil containing protein 1	chr8p11.23		6867	TACC1	transforming, acidic coiled-coil containing protein 1	---		706243	MmugDNA.17106.1.S1_s_at_TACC1
MmugDNA.17107.1.S1_at	1552263_at	MAPK1	mitogen-activated protein kinase 1	chr22q11.2|22q11.21		5594	MAPK1	mitogen-activated protein kinase 1	---		698569	MmugDNA.17107.1.S1_at_MAPK1
MmugDNA.17108.1.S1_at	233074_at	---	---	---	---		CHCHD4	Similar to Mitochondrial intermembrane space import and assembly protein 40 (Coiled-coil-helix-coile	---		696492	MmugDNA.17108.1.S1_at_---
MmugDNA.17109.1.S1_at	1552562_at	ZNF570	zinc finger protein 570	chr19q13.12		148268	LOC713879	similar to zinc finger protein 570	---		713879	MmugDNA.17109.1.S1_at_ZNF570
MmugDNA.17110.1.S1_at	211359_s_at	OPRM1	opioid receptor, mu 1	chr6q24-q25		4988	OPRM1	opioid receptor, mu 1	---		574141	MmugDNA.17110.1.S1_at_OPRM1
MmugDNA.17111.1.S1_at	231681_x_at	HIST3H2A	Histone cluster 3, H2a	chr1q42.13		92815	LOC695551	similar to histone 2a	---		695551	MmugDNA.17111.1.S1_at_HIST3H2A
MmugDNA.1711.1.S1_at	238504_at	C6orf57	chromosome 6 open reading frame 57	chr6q13		135154	LOC717008	similar to CG7224-PA, isoform A	---		717008	MmugDNA.1711.1.S1_at_C6orf57
MmugDNA.17112.1.S1_at	228086_at	STK33	serine/threonine kinase 33	chr11p15.3		65975	STK33	serine/threonine kinase 33	---		705791	MmugDNA.17112.1.S1_at_STK33
MmugDNA.17113.1.S1_at	228035_at	STK33	serine/threonine kinase 33	chr11p15.3		65975	STK33	serine/threonine kinase 33	---		705791	MmugDNA.17113.1.S1_at_STK33
MmugDNA.17114.1.S1_at	241759_at	MGC5566	Hypothetical protein MGC5566	chr20q13.12		79015	---	---	---	---		MmugDNA.17114.1.S1_at_MGC5566
MmugDNA.17115.1.S1_at	242214_at	RPS27A	Ribosomal protein S27a	chr2p16		6233	---	---	---	---		MmugDNA.17115.1.S1_at_RPS27A
MmugDNA.17116.1.S1_at	239563_at	---	---	---	---		---	---	---	---		MmugDNA.17116.1.S1_at_---
MmugDNA.17117.1.S1_at	1553630_at	C10orf107	chromosome 10 open reading frame 107	chr10q21.2		219621	LOC700172	hypothetical protein LOC700172	---		700172	MmugDNA.17117.1.S1_at_C10orf107
MmugDNA.17118.1.S1_at	205483_s_at	ISG15	ISG15 ubiquitin-like modifier	chr1p36.33		9636	ISG15	ISG15 ubiquitin-like modifier	---		700141	MmugDNA.17118.1.S1_at_ISG15
MmugDNA.17119.1.S1_at	1559573_at	---	---	---	---		---	---	---	---		MmugDNA.17119.1.S1_at_---

MmugDNA.171.1.S1_at	236010_at	---	---	---	---		---	---	---	---		MmugDNA.171.1.S1_at_---
MmugDNA.17120.1.S1_at	220740_s_at	SLC12A6	solute carrier family 12 (potassium/chloride transporters), member 6	chr15q13-q15		9990	SLC12A6	solute carrier family 12 (potassium/chloride transporters), member 6	---		697536	MmugDNA.17120.1.S1_at_SLC12A6
MmugDNA.17121.1.S1_at	200803_s_at	TMBIM6	transmembrane BAX inhibitor motif containing 6	chr12q12-q13		7009	LOC701925	Similar to solute carrier family 4, sodium bicarbonate cotransporter, member 7	---		701925	MmugDNA.17121.1.S1_at_TMBIM6
MmugDNA.1712.1.S1_at	1556352_at	---	---	---	---		---	---	---	---		MmugDNA.1712.1.S1_at_---
MmugDNA.17122.1.S1_at	221209_s_at	OTOR	otoraplin	chr20p12.1-p11.23		56914	OTOR	otoraplin	---		695505	MmugDNA.17122.1.S1_at_OTOR
MmugDNA.17123.1.S1_at	234096_at	---	---	---	---		---	---	---	---		MmugDNA.17123.1.S1_at_---
MmugDNA.17124.1.S1_at	241920_x_at	SPG11	spastic paraplegia 11 (autosomal recessive)	chr15q14		80208	LOC712221	hypothetical protein LOC712221	---		712221	MmugDNA.17124.1.S1_at_SPG11
MmugDNA.17125.1.S1_at	214793_at	DUSP7	dual specificity phosphatase 7	chr3p21		1849	LOC698473	similar to dual specificity phosphatase 7	---		698473	MmugDNA.17125.1.S1_at_DUSP7
MmugDNA.17126.1.S1_at	235298_at	WDR27	WD repeat domain 27	chr6q27		253769	LOC695097	similar to WD repeat domain 27	---		695097	MmugDNA.17126.1.S1_at_WDR27
MmugDNA.17127.1.S1_at	NA	NA	NA	NA	NA		NA	NA	NA	NA		MmugDNA.17127.1.S1_at_NA
MmugDNA.17127.1.S1_s_at	214270_s_at	MAPRE3	microtubule-associated protein, RP/EB family, member 3	chr2p23.3-p23.1		22924	LOC699648	similar to dihydropyrimidinase-like 5	---		699648	MmugDNA.17127.1.S1_s_at_MAPRE3
MmugDNA.17128.1.S1_at	1561149_at	---	---	---	---		---	---	---	---		MmugDNA.17128.1.S1_at_---
MmugDNA.17129.1.S1_at	222173_s_at	TBC1D2	TBC1 domain family, member 2	chr9q22.33		55357	TBC1D2	TBC1 domain family, member 2	---		715322	MmugDNA.17129.1.S1_at_TBC1D2
MmugDNA.17130.1.S1_at	1569407_at	---	---	---	---		---	---	---	---		MmugDNA.17130.1.S1_at_---
MmugDNA.17131.1.S1_at	240721_at	---	---	---	---		---	---	---	---		MmugDNA.17131.1.S1_at_---
MmugDNA.1713.1.S1_at	1556873_at	MEX3C	mex-3 homolog C (C. elegans)	chr18q21.2		51320	---	---	---	---		MmugDNA.1713.1.S1_at_MEX3C
MmugDNA.17132.1.S1_at	242237_at	---	---	---	---		---	---	---	---		MmugDNA.17132.1.S1_at_---
MmugDNA.17133.1.S1_at	1560750_at	LOC151121	hypothetical LOC151121	chr2q21.1		151121	---	---	---	---		MmugDNA.17133.1.S1_at_LOC151121
MmugDNA.17134.1.S1_at	1555358_a_at	ENTPD4	ectonucleoside triphosphate diphosphohydrolase 4	chr8p21.3		9583	ENTPD4	ectonucleoside triphosphate diphosphohydrolase 4	---		717197	MmugDNA.17134.1.S1_at_ENTPD4
MmugDNA.17135.1.S1_at	209236_at	SLC23A2	solute carrier family 23 (nucleobase transporters), member 2	chr20p13		9962	---	---	---	---		MmugDNA.17135.1.S1_at_SLC23A2
MmugDNA.17136.1.S1_at	242736_at	---	---	---	---		---	---	---	---		MmugDNA.17136.1.S1_at_---
MmugDNA.17137.1.S1_at	235466_s_at	DISP1	Dispatched homolog 1 (Drosophila)	chr1q41		84976	---	---	---	---		MmugDNA.17137.1.S1_at_DISP1
MmugDNA.17138.1.S1_at	1559429_a_at	C3orf43	chromosome 3 open reading frame 43	chr3q29		255798	---	---	---	---		MmugDNA.17138.1.S1_at_C3orf43
MmugDNA.17139.1.S1_s_at	221059_s_at	COTL1	coactosin-like 1 (Dictyostelium)	chr16q24.1		23406	LOC716455	Similar to syntaxin 12	---		716455	MmugDNA.17139.1.S1_s_at_COTL1
MmugDNA.17140.1.S1_at	207600_at	KCNC3	potassium voltage-gated channel, Shaw-related subfamily, member 3	chr19q13.3-q13.4		3748	---	---	---	---		MmugDNA.17140.1.S1_at_KCNC3
MmugDNA.17141.1.S1_at	243317_at	---	---	---	---		---	---	---	---		MmugDNA.17141.1.S1_at_---
MmugDNA.1714.1.S1_at	1552348_at	PRSS33	protease, serine, 33	chr16p13.3		260429	---	---	---	---		MmugDNA.1714.1.S1_at_PRSS33
MmugDNA.17142.1.S1_at	220894_x_at	PRDM12	PR domain containing 12	chr9q33-q34		59335	---	---	---	---		MmugDNA.17142.1.S1_at_PRDM12
MmugDNA.17143.1.S1_x_at	208490_x_at	HIST1H2BF	histone cluster 1, H2bf	chr6p21.3		8343	LOC697469	similar to Histone H2B 291B	---		697469	MmugDNA.17143.1.S1_x_at_HIST1H2E
MmugDNA.17144.1.S1_at	1560940_at	---	---	---	---		---	---	---	---		MmugDNA.17144.1.S1_at_---
MmugDNA.17146.1.S1_at	207293_s_at	AGTR2	angiotensin II receptor, type 2	chrXq22-q23		186	AGTR2	angiotensin II receptor, type 2	---		709513	MmugDNA.17146.1.S1_at_AGTR2
MmugDNA.17147.1.S1_at	240633_at	DOK7	docking protein 7	chr4p16.2		285489	---	---	---	---		MmugDNA.17147.1.S1_at_DOK7
MmugDNA.17148.1.S1_at	1562349_at	TRAPPC2	trafficking protein particle complex 2	chrXp22		6399	---	---	---	---		MmugDNA.17148.1.S1_at_TRAPPC2
MmugDNA.17149.1.S1_at	240570_at	INADL	InaD-like (Drosophila)	chr1p31.3		10207	---	---	---	---		MmugDNA.17149.1.S1_at_INADL
MmugDNA.17150.1.S1_at	1553070_a_at	MYOZ3	myozenin 3	chr5q33.1		91977	LOC712647	similar to myozenin 3	---		712647	MmugDNA.17150.1.S1_at_MYOZ3
MmugDNA.17151.1.S1_at	1560180_at	GALNT14	UDP-N-acetyl-alpha-D-galactosamine:polypeptide N-acetylgalactosaminyltransferase 14 (GalNAc-T1	chr2p23.1		79623	---	---	---	---		MmugDNA.17151.1.S1_at_GALNT14
MmugDNA.1715.1.S1_at	219854_at	ZNF14	zinc finger protein 14	chr19p13.3-p13.2		7561	---	---	---	---		MmugDNA.1715.1.S1_at_ZNF14
MmugDNA.17152.1.S1_at	244696_at	---	---	---	---		---	---	---	---		MmugDNA.17152.1.S1_at_---
MmugDNA.17153.1.S1_at	1561686_at	---	---	---	---		---	---	---	---		MmugDNA.17153.1.S1_at_---
MmugDNA.17154.1.S1_at	229739_s_at	FAM116B	family with sequence similarity 116, member B	chr22q13.33		414918	LOC715877	similar to CG3309-PA	---		715877	MmugDNA.17154.1.S1_at_FAM116B
MmugDNA.17155.1.S1_at	233431_x_at	---	---	---	---		---	---	---	---		MmugDNA.17155.1.S1_at_---
MmugDNA.17156.1.S1_at	231222_at	---	---	---	---		---	---	---	---		MmugDNA.17156.1.S1_at_---
MmugDNA.17157.1.S1_at	216269_s_at	ELN	elastin	chr7q11.23		2006	---	---	---	---		MmugDNA.17157.1.S1_at_ELN
MmugDNA.17157.1.S1_x_at	NA	NA	NA	NA	NA		NA	NA	NA	NA		MmugDNA.17157.1.S1_x_at_NA
MmugDNA.17158.1.S1_at	221337_s_at	ADAM29	ADAM metallopeptidase domain 29	chr4q34		11086	LOC697067	Similar to Rabring 7	---		697067	MmugDNA.17158.1.S1_at_ADAM29
MmugDNA.17159.1.S1_at	204702_s_at	NFE2L3	nuclear factor (erythroid-derived 2)-like 3	chr7p15-p14		9603	NFE2L3	nuclear factor (erythroid-derived 2)-like 3	---		702171	MmugDNA.17159.1.S1_at_NFE2L3
MmugDNA.17159.1.S1_s_at	NA	NA	NA	NA	NA		NA	NA	NA	NA		MmugDNA.17159.1.S1_s_at_NA
MmugDNA.17160.1.S1_at	228954_at	LYSMD4	LysM, putative peptidoglycan-binding, domain containing 4	chr15q26.3		145748	LOC709141	similar to LysM, putative peptidoglycan-binding, domain containing 4	---		709141	MmugDNA.17160.1.S1_at_LYSMD4
MmugDNA.17161.1.S1_at	1552621_at	POLR2J2	polymerase (RNA) II (DNA directed) polypeptide J2	chr7q22.1		246721	---	---	---	---		MmugDNA.17161.1.S1_at_POLR2J2
MmugDNA.1716.1.S1_at	235066_at	MAP4	microtubule-associated protein 4	chr3p21		4134	PRKCB	Protein kinase C, beta	---		701195	MmugDNA.1716.1.S1_at_MAP4
MmugDNA.17162.1.S1_at	230383_x_at	---	---	---	---		---	---	---	---		MmugDNA.17162.1.S1_at_---
MmugDNA.17163.1.S1_at	219024_at	PLEKHA1	pleckstrin homology domain containing, family A (phosphoinositide binding specific) member 1	chr10q26.13		59338	PLEKHA1	pleckstrin homology domain containing, family A (phosphoinositide binding specific) member 1	---		705731	MmugDNA.17163.1.S1_at_PLEKHA1
MmugDNA.17164.1.S1_s_at	226926_at	DMKN	dermokine	chr19q13.12		93099	LOC718709	similar to dermokine isoform beta	---		718709	MmugDNA.17164.1.S1_s_at_DMKN
MmugDNA.17165.1.S1_at	221169_s_at	HRH4	histamine receptor H4	chr18q11.2		59340	LOC712018	Similar to immunity-related GTPase family, cinema 1	---		712018	MmugDNA.17165.1.S1_at_HRH4
MmugDNA.17167.1.S1_at	1558938_at	---	---	---	---		---	---	---	---		MmugDNA.17167.1.S1_at_---
MmugDNA.17168.1.S1_at	1554736_at	ARHGAP29	Rho GTPase activating protein 29	chr1p22.1		9411	---	---	---	---		MmugDNA.17168.1.S1_at_ARHGAP29
MmugDNA.17169.1.S1_at	1554102_a_at	TMTC4	transmembrane and tetratricopeptide repeat containing 4	chr13q32.3		84899	---	---	---	---		MmugDNA.17169.1.S1_at_TMTC4
MmugDNA.17170.1.S1_at	204583_x_at	KLK3	kallikrein-related peptidase 3	chr19q13.41		354	LOC719444	prostate specific antigen	---		719444	MmugDNA.17170.1.S1_at_KLK3
MmugDNA.17171.1.S1_at	242458_at	RALGPS2	Ral GEF with PH domain and SH3 binding motif 2	chr1q25.2		55103	LOC717165	similar to Ral GEF with PH domain and SH3 binding motif 2 isoform 2	---		717165	MmugDNA.17171.1.S1_at_RALGPS2
MmugDNA.1717.1.S1_at	233882_s_at	SEMA6D	sema domain, transmembrane domain (TM), and cytoplasmic domain, (semaphorin) 6D	chr15q21.1		80031	LOC713885	similar to semaphorin 6D isoform 3 precursor	---		713885	MmugDNA.1717.1.S1_at_SEMA6D
MmugDNA.17172.1.S1_at	230141_at	ARID4A	AT rich interactive domain 4A (RBP1-like)	chr14q23.1		5926	ARID4A	AT rich interactive domain 4A (RBP1-like)	---		702688	MmugDNA.17172.1.S1_at_ARID4A
MmugDNA.17173.1.S1_at	1564813_at	---	---	---	---		---	---	---	---		MmugDNA.17173.1.S1_at_---
MmugDNA.17175.1.S1_at	235444_at	FOXP1	forkhead box P1	chr3p14.1		27086	---	---	---	---		MmugDNA.17175.1.S1_at_FOXP1
MmugDNA.17176.1.S1_at	NA	NA	NA	NA	NA		NA	NA	NA	NA		MmugDNA.17176.1.S1_at_NA
MmugDNA.17177.1.S1_at	1552359_at	C8orf45	chromosome 8 open reading frame 45	chr8q13.1		157777	---	---	---	---		MmugDNA.17177.1.S1_at_C8orf45
MmugDNA.17178.1.S1_at	224775_at	IWS1	IWS1 homolog (S. cerevisiae)	chr2q14.3		55677	IWS1	IWS1 homolog (S. cerevisiae)	---		699205	MmugDNA.17178.1.S1_at_IWS1
MmugDNA.17179.1.S1_at	206454_s_at	RHO	rhodopsin	chr3q21-q24		6010	LOC712087	Similar to 60S ribosomal protein L29 (P23)	---		712087	MmugDNA.17179.1.S1_at_RHO
MmugDNA.17180.1.S1_at	224768_at	IWS1	IWS1 homolog (S. cerevisiae)	chr2q14.3		55677	IWS1	IWS1 homolog (S. cerevisiae)	---		699205	MmugDNA.17180.1.S1_at_IWS1
MmugDNA.17181.1.S1_at	229858_at	---	---	---	---		---	---	---	---		MmugDNA.17181.1.S1_at_---
MmugDNA.1718.1.S1_at	201593_s_at	ZC3H15	zinc finger CCCH-type containing 15	chr2q32.1		55854	---	---	---	---		MmugDNA.1718.1.S1_at_ZC3H15
MmugDNA.17182.1.S1_at	234903_at	OR2B3	olfactory receptor, family 2, subfamily B, member 3	chr6p22.1		442184	LOC708747	similar to Olfactory receptor 2B3 (Olfactory receptor 6-4) (OR6-4) (Olfactory receptor OR6-14) (Hs6M	---		708747	MmugDNA.17182.1.S1_at_OR2B3
MmugDNA.17183.1.S1_at	222445_at	SLC39A9	solute carrier family 39 (zinc transporter), member 9	chr14q24.1		55334	---	---	---	---		MmugDNA.17183.1.S1_at_SLC39A9
MmugDNA.17184.1.S1_at	215010_s_at	BRSK2	BR serine/threonine kinase 2	chr11p15.5		9024	MGP	Matrix Gla protein	---		574116	MmugDNA.17184.1.S1_at_BRSK2
MmugDNA.17185.1.S1_at	242915_at	ZNF682	zinc finger protein 682	chr19p12		91120	LOC719936	similar to zinc finger protein 682	---		719936	MmugDNA.17185.1.S1_at_ZNF682
MmugDNA.17186.1.S1_at	241938_at	QKI	Quaking homolog, KH domain RNA binding (mouse)	chr6q26|6q26-q27		9444	---	---	---	---		MmugDNA.17186.1.S1_at_QKI
MmugDNA.17187.1.S1_at	218122_s_at	SENP2	SUMO1/sentrin/SMT3 specific peptidase 2	chr3q27.2		59343	SENP2	SUMO1/sentrin/SMT3 specific peptidase 2	---		700717	MmugDNA.17187.1.S1_at_SENP2
MmugDNA.17188.1.S1_at	202366_at	ACADS	acyl-Coenzyme A dehydrogenase, C-2 to C-3 short chain	chr12q22-qter		35	ACADS	acyl-Coenzyme A dehydrogenase, C-2 to C-3 short chain	---		698196	MmugDNA.17188.1.S1_at_ACADS
MmugDNA.17189.1.S1_at	220887_at	C14orf162	chromosome 14 open reading frame 162	chr14q24.1		56936	---	---	---	---		MmugDNA.17189.1.S1_at_C14orf162
MmugDNA.17190.1.S1_at	236923_x_at	---	---	---	---		---	---	---	---		MmugDNA.17190.1.S1_at_---
MmugDNA.17191.1.S1_at	1555852_at	---	---	---	---		---	---	---	---		MmugDNA.17191.1.S1_at_---
MmugDNA.1719.1.S1_at	1559835_at	---	---	---	---		---	---	---	---		MmugDNA.1719.1.S1_at_---
MmugDNA.17192.1.S1_at	NA	NA	NA	NA	NA		NA	NA	NA	NA		MmugDNA.17192.1.S1_at_NA
MmugDNA.17192.1.S1_s_at	217875_s_at	PMEPA1	prostate transmembrane protein, androgen induced 1	chr20q13.31|20q13.31-q13.33		56937	LOC695968	similar to transmembrane prostate androgen-induced protein isoform b	---		695968	MmugDNA.17192.1.S1_s_at_PMEPA1
MmugDNA.17193.1.S1_at	208575_at	HIST1H3A	histone cluster 1, H3a	chr6p21.3		8350	LOC695092	similar to histone 1, H2ai (predicted)	---		695092	MmugDNA.17193.1.S1_at_HIST1H3A
MmugDNA.17194.1.S1_at	211500_at	MAPK11	mitogen-activated protein kinase 11	chr22q13.33		5600	LOC700942	Similar to TRAF interacting protein TANK isoform a	---		700942	MmugDNA.17194.1.S1_at_MAPK11
MmugDNA.17195.1.S1_at	1555853_at	---	---	---	---		---	---	---	---		MmugDNA.17195.1.S1_at_---
MmugDNA.17196.1.S1_at	205412_at	ACAT1	acetyl-Coenzyme A acetyltransferase 1	chr11q22.3-q23.1		38	LOC701705	Similar to 40S ribosomal protein S12	---		701705	MmugDNA.17196.1.S1_at_ACAT1
MmugDNA.17197.1.S1_at	219165_at	PDLIM2	PDZ and LIM domain 2 (mystique)	chr8p21.2		64236	LOC708652	similar to PDZ and LIM domain 2 isoform 2	---		708652	MmugDNA.17197.1.S1_at_PDLIM2
MmugDNA.17198.1.S1_at	224242_at	GALP	galanin-like peptide	chr19q13.42		85569	---	---	---	---		MmugDNA.17198.1.S1_at_GALP
MmugDNA.17199.1.S1_at	1558208_at	---	---	---	---		TARDBP	TAR DNA binding protein	---		713860	MmugDNA.17199.1.S1_at_---
MmugDNA.17.1.S1_at	221526_x_at	PARD3	par-3 partitioning defective 3 homolog (C. elegans)	chr10p11.22-p11.21		56288	---	---	---	---		MmugDNA.17.1.S1_at_PARD3
MmugDNA.17200.1.S1_at	1552846_s_at	RAB42	RAB42, member RAS oncogene family	chr1p35.3		115273	LOC720282	similar to RAB42, member RAS homolog family	---		720282	MmugDNA.17200.1.S1_at_RAB42
MmugDNA.17201.1.S1_at	NA	NA	NA	NA	NA		NA	NA	NA	NA		MmugDNA.17201.1.S1_at_NA
MmugDNA.1720.1.S1_at	215305_at	PDGFRA	platelet-derived growth factor receptor, alpha polypeptide	chr4q11-q13		5156	---	---	---	---		MmugDNA.1720.1.S1_at_PDGFRA
MmugDNA.17202.1.S1_at	244850_at	---	---	---	---		---	---	---	---		MmugDNA.17202.1.S1_at_---
MmugDNA.17203.1.S1_at	224812_at	HIBADH	3-hydroxyisobutyrate dehydrogenase	chr7p15.2		11112	LOC702794	hypothetical protein LOC702794	---		702794	MmugDNA.17203.1.S1_at_HIBADH
MmugDNA.17204.1.S1_at	240000_at	---	---	---	---		---	---	---	---		MmugDNA.17204.1.S1_at_---
MmugDNA.17205.1.S1_at	210159_s_at	TRIM31	tripartite motif-containing 31	chr6p21.3		11074	LOC709807	similar to tripartite motif protein 31 isoform alpha	---		709807	MmugDNA.17205.1.S1_at_TRIM31
MmugDNA.17206.1.S1_at	1562674_at	---	---	---	---		---	---	---	---		MmugDNA.17206.1.S1_at_---
MmugDNA.17207.1.S1_at	1563059_at	LOC340581	hypothetical protein LOC340581	chrXq26.3		340581	---	---	---	---		MmugDNA.17207.1.S1_at_LOC340581
MmugDNA.17209.1.S1_at	206891_at	ACTN3	actinin, alpha 3	chr11q13.1		89	LOC713811	similar to skeletal muscle specific actinin, alpha 3	---		713811	MmugDNA.17209.1.S1_at_ACTN3
MmugDNA.17210.1.S1_at	1565888_at	---	---	---	---		---	---	---	---		MmugDNA.17210.1.S1_at_---
MmugDNA.17211.1.S1_at	222925_at	DCDC2	doublecortin domain containing 2	chr6p22.1		51473	DCDC2	doublecortin domain containing 2	---		707472	MmugDNA.17211.1.S1_at_DCDC2
MmugDNA.1721.1.S1_at	208081_s_at	ZNF442	zinc finger protein 442	chr19p13.2		79973	LOC717463	similar to zinc finger protein 442	---		717463	MmugDNA.1721.1.S1_at_ZNF442
MmugDNA.17212.1.S1_at	222926_at	DCDC2	doublecortin domain containing 2	chr6p22.1		51473	---	---	---	---		MmugDNA.17212.1.S1_at_DCDC2
MmugDNA.17213.1.S1_at	210248_at	WNT7A	wingless-type MMTV integration site family, member 7A	chr3p25		7476	WNT7A	wingless-type MMTV integration site family, member 7A	---		693419	MmugDNA.17213.1.S1_at_WNT7A
MmugDNA.17214.1.S1_at	237663_at	---	---	---	---		---	---	---	---		MmugDNA.17214.1.S1_at_---
MmugDNA.17215.1.S1_at	1557089_at	KIF5C	kinesin family member 5C	chr2q23.1		3800	KIF5C	kinesin family member 5C	---		693340	MmugDNA.17215.1.S1_at_KIF5C

MmugDNA.17216.1.S1_at	208496_x_at	HIST1H3G	histone cluster 1, H3g	chr6p21.3		8355	LOC698731	similar to histone 1, H2ai (predicted)	---		698731	MmugDNA.17216.1.S1_at_HIST1H3G
MmugDNA.17217.1.S1_at	206068_s_at	ACADL	acyl-Coenzyme A dehydrogenase, long chain	chr2q34-q35		33	LOC711485	similar to acyl-Coenzyme A dehydrogenase, long chain precursor	---		711485	MmugDNA.17217.1.S1_at_ACADL
MmugDNA.17218.1.S1_at	204639_at	ADA	adenosine deaminase	chr20q12-q13.11		100	ADA	adenosine deaminase	---		717897	MmugDNA.17218.1.S1_at_ADA
MmugDNA.17219.1.S1_at	206110_at	---	---	---	---		LOC703889	similar to histone 1, H2ai (predicted)	---		703889	MmugDNA.17219.1.S1_at_---
MmugDNA.172.1.S1_s_at	204053_x_at	PTEN	phosphatase and tensin homolog	chr10q23.3		5728	LOC718005	Hypothetical protein LOC718005	---		718005	MmugDNA.172.1.S1_s_at_PTEN
MmugDNA.17220.1.S1_at	231420_at	GGN	gametogenetin	chr19q13.2		199720	LOC715072	similar to gametogenetin	---		715072	MmugDNA.17220.1.S1_at_GGN
MmugDNA.17221.1.S1_at	214128_at	DAGLA	diacylglycerol lipase, alpha	chr11q12.2		747	LOC719100	hypothetical protein LOC719100	---		719100	MmugDNA.17221.1.S1_at_DAGLA
MmugDNA.1722.1.S1_at	237808_at	ST7OT2	ST7 overlapping transcript 2 (non-protein coding)	chr7q31.1-q31.2		93654	---	---	---	---		MmugDNA.1722.1.S1_at_ST7OT2
MmugDNA.17222.1.S1_at	NA	NA	NA	NA	NA		NA	NA	NA	NA		MmugDNA.17222.1.S1_at_NA
MmugDNA.17223.1.S1_at	213328_at	NEK1	NIMA (never in mitosis gene a)-related kinase 1	chr4q33		4750	NEK1	NIMA (never in mitosis gene a)-related kinase 1	---		694345	MmugDNA.17223.1.S1_at_NEK1
MmugDNA.17224.1.A1_at	NA	NA	NA	NA	NA		NA	NA	NA	NA		MmugDNA.17224.1.A1_at_NA
MmugDNA.17224.1.S1_at	231349_at	GPR150	G protein-coupled receptor 150	chr5q15		285601	---	---	---	---		MmugDNA.17224.1.S1_at_GPR150
MmugDNA.17225.1.S1_at	213331_s_at	NEK1	NIMA (never in mitosis gene a)-related kinase 1	chr4q33		4750	NEK1	NIMA (never in mitosis gene a)-related kinase 1	---		694345	MmugDNA.17225.1.S1_at_NEK1
MmugDNA.17226.1.S1_at	231103_at	---	---	---	---		---	---	---	---		MmugDNA.17226.1.S1_at_---
MmugDNA.17228.1.S1_at	211002_s_at	TRIM29	tripartite motif-containing 29	chr11q22-q23		23650	TRIM29	tripartite motif-containing 29	---		705796	MmugDNA.17228.1.S1_at_TRIM29
MmugDNA.17229.1.S1_at	211916_s_at	MYO1A	myosin IA	chr12q13-q14		4640	MYO1A	myosin IA	---		714033	MmugDNA.17229.1.S1_at_MYO1A
MmugDNA.17230.1.S1_at	225645_at	EHF	Ets homologous factor	chr11p12		26298	LOC717350	similar to ets homologous factor	---		717350	MmugDNA.17230.1.S1_at_EHF
MmugDNA.17231.1.S1_at	1554685_a_at	KIAA1199	KIAA1199	chr15q24		57214	LOC717880	similar to KIAA1199	---		717880	MmugDNA.17231.1.S1_at_KIAA1199
MmugDNA.1723.1.S1_at	237945_at	---	---	---	---		---	---	---	---		MmugDNA.1723.1.S1_at_---
MmugDNA.17232.1.S1_at	215220_s_at	TPR	translocated promoter region (to activated MET oncogene)	chr1q25		7175	TPR	translocated promoter region (to activated MET oncogene)	---		713910	MmugDNA.17232.1.S1_at_TPR
MmugDNA.17233.1.S1_at	234573_at	---	---	---	---		---	---	---	---		MmugDNA.17233.1.S1_at_---
MmugDNA.17234.1.S1_at	1561152_a_at	---	---	---	---		---	---	---	---		MmugDNA.17234.1.S1_at_---
MmugDNA.17235.1.S1_at	238571_at	---	---	---	---		---	---	---	---		MmugDNA.17235.1.S1_at_---
MmugDNA.17236.1.S1_at	205017_s_at	MBNL2	muscleblind-like 2 (Drosophila)	chr13q32.1		10150	LOC697097	similar to muscleblind-like 2 isoform 1	---		697097	MmugDNA.17236.1.S1_at_MBNL2
MmugDNA.17237.1.S1_at	233210_at	FLJ12120	hypothetical LOC388439	chr17p13.3		388439	---	---	---	---		MmugDNA.17237.1.S1_at_FLJ12120
MmugDNA.17238.1.S1_at	203741_s_at	ADCY7	adenylate cyclase 7	chr16q12-q13		113	---	---	---	---		MmugDNA.17238.1.S1_at_ADCY7
MmugDNA.17239.1.S1_at	243732_at	---	---	---	---		---	---	---	---		MmugDNA.17239.1.S1_at_---
MmugDNA.17240.1.S1_at	241808_at	FAM164A	family with sequence similarity 164, member A	chr8q21.12		51101	LOC698760	similar to T03G11.3	---		698760	MmugDNA.17240.1.S1_at_FAM164A
MmugDNA.17241.1.S1_at	206811_at	ADCY8 /// LOC1001340	adenylate cyclase 8 (brain) /// similar to adenylyl cyclase	chr8q24		100134077 /// 114	ADCY8	adenylate cyclase 8 (brain)	---		695792	MmugDNA.17241.1.S1_at_ADCY8 /// L
MmugDNA.1724.1.S1_at	240764_at	---	---	---	---		---	---	---	---		MmugDNA.1724.1.S1_at_---
MmugDNA.17242.1.S1_at	227911_at	ARHGAP28	Rho GTPase activating protein 28	chr18p11.31		79822	LOC718264	Similar to progestin and adipoQ receptor family member VI isoform 2	---		718264	MmugDNA.17242.1.S1_at_ARHGAP28
MmugDNA.17243.1.S1_at	239463_at	---	---	---	---		---	---	---	---		MmugDNA.17243.1.S1_at_---
MmugDNA.17244.1.S1_at	1553268_at	RHBDL3	rhomboid, veinlet-like 3 (Drosophila)	chr17q11.2		162494	---	---	---	---		MmugDNA.17244.1.S1_at_RHBDL3
MmugDNA.17245.1.S1_at	230419_at	FLJ37644 /// LOC72797	hypothetical gene supported by AK094963 /// hypothetical protein LOC727973	chr17q24.3		400618 /// 727973	LOC694951	hypothetical protein LOC694951	---		694951	MmugDNA.17245.1.S1_at_FLJ37644 ///
MmugDNA.17247.1.S1_at	1553886_at	CCDC108	coiled-coil domain containing 108	chr2q35		255101	---	---	---	---		MmugDNA.17247.1.S1_at_CCDC108
MmugDNA.17248.1.S1_at	240929_at	---	---	---	---		---	---	---	---		MmugDNA.17248.1.S1_at_---
MmugDNA.17249.1.S1_at	210541_s_at	TRIM27	tripartite motif-containing 27	chr6p22		5987	TRIM27	tripartite motif-containing 27	---		706129	MmugDNA.17249.1.S1_at_TRIM27
MmugDNA.17250.1.S1_at	239461_at	GALNTL2	UDP-N-acetyl-alpha-D-galactosamine:polypeptide N-acetylgalactosaminyltransferase-like 2	chr3p24.3		117248	GALNTL2	similar to UDP-N-acetyl-alpha-D-galactosamine:polypeptide	---		694784	MmugDNA.17250.1.S1_at_GALNTL2
MmugDNA.17251.1.S1_at	210214_s_at	BMPR2	bone morphogenetic protein receptor, type II (serine/threonine kinase)	chr2q33-q34		659	---	---	---	---		MmugDNA.17251.1.S1_at_BMPR2
MmugDNA.1725.1.S1_at	236330_at	---	---	---	---		---	---	---	---		MmugDNA.1725.1.S1_at_---
MmugDNA.17252.1.S1_at	1557551_at	---	---	---	---		---	---	---	---		MmugDNA.17252.1.S1_at_---
MmugDNA.17253.1.S1_at	223513_at	CENPJ	centromere protein J	chr13q12.12		55835	CENPJ	centromere protein J	---		721498	MmugDNA.17253.1.S1_at_CENPJ
MmugDNA.17254.1.S1_at	208214_at	ADRB1	adrenergic, beta-1-, receptor	chr10q24-q26		153	---	---	---	---		MmugDNA.17254.1.S1_at_ADRB1
MmugDNA.17255.1.S1_at	225896_at	---	---	---	---		---	---	---	---		MmugDNA.17255.1.S1_at_---
MmugDNA.17256.1.S1_at	223836_at	FGFBP2	fibroblast growth factor binding protein 2	chr4p16		83888	LOC714554	similar to Ksp37 protein	---		714554	MmugDNA.17256.1.S1_at_FGFBP2
MmugDNA.17257.1.S1_at	215989_at	CBX2	chromobox homolog 2 (Pc class homolog, Drosophila)	chr17q25.3		84733	LOC717462	similar to chromobox homolog 2 isoform 1	---		717462	MmugDNA.17257.1.S1_at_CBX2
MmugDNA.17258.1.S1_at	223625_at	FAM126A	family with sequence similarity 126, member A	chr7p15.3		84668	FAM126A	family with sequence similarity 126, member A	---		705467	MmugDNA.17258.1.S1_at_FAM126A
MmugDNA.17259.1.S1_at	202144_s_at	ADSL	adenylosuccinate lyase	chr22q13.1|22q13.2		158	ADSL	adenylosuccinate lyase	---		709259	MmugDNA.17259.1.S1_at_ADSL
MmugDNA.17260.1.S1_at	235877_at	---	---	---	---		---	---	---	---		MmugDNA.17260.1.S1_at_---
MmugDNA.17261.1.S1_at	223498_at	CYTSB	cytospin B	chr17p11.2		92521	CYTSB	cytospin B	---		704221	MmugDNA.17261.1.S1_at_CYTSB
MmugDNA.1726.1.S1_at	241090_at	---	---	---	---		---	---	---	---		MmugDNA.1726.1.S1_at_---
MmugDNA.17262.1.S1_at	235371_at	GLT8D4	glycosyltransferase 8 domain containing 4	chr3p13		727936	GLT8D3	similar to CG9996-PA	---		693538	MmugDNA.17262.1.S1_at_GLT8D4
MmugDNA.17263.1.S1_at	239362_at	NAPA	N-ethylmaleimide-sensitive factor attachment protein, alpha	chr19q13.32		8775	---	---	---	---		MmugDNA.17263.1.S1_at_NAPA
MmugDNA.17264.1.S1_at	233419_at	---	---	---	---		---	---	---	---		MmugDNA.17264.1.S1_at_---
MmugDNA.17265.1.S1_at	217484_at	CR1	complement component (3b/4b) receptor 1 (Knops blood group)	chr1q32		1378	---	---	---	---		MmugDNA.17265.1.S1_at_CR1
MmugDNA.17266.1.S1_at	1562281_at	---	---	---	---		---	---	---	---		MmugDNA.17266.1.S1_at_---
MmugDNA.17267.1.S1_at	NA	NA	NA	NA	NA		NA	NA	NA	NA		MmugDNA.17267.1.S1_at_NA
MmugDNA.17267.1.S1_s_at	210995_s_at	TRIM23	tripartite motif-containing 23	chr5q12.3		373	TRIM23	tripartite motif-containing 23	---		696386	MmugDNA.17267.1.S1_s_at_TRIM23
MmugDNA.17268.1.S1_at	240423_at	hCG_2009921 /// LOC1	hypothetical locus LOC441204 /// similar to hCG2009921	chr7p15.2		100134569 /// 441204	LOC706576	hypothetical protein LOC706576	---		706576	MmugDNA.17268.1.S1_at_hCG_200992
MmugDNA.17269.1.S1_at	1553030_a_at	SUOX	sulfite oxidase	chr12q13.2		6821	LOC711164	similar to sulfite oxidase	---		711164	MmugDNA.17269.1.S1_at_SUOX
MmugDNA.17270.1.S1_at	NA	NA	NA	NA	NA		NA	NA	NA	NA		MmugDNA.17270.1.S1_at_NA
MmugDNA.17271.1.S1_at	1569714_at	ZFYVE20	zinc finger, FYVE domain containing 20	chr3p24.3		64145	LOC702356	similar to FYVE-finger-containing Rab5 effector protein rabenosyn-5	---		702356	MmugDNA.17271.1.S1_at_ZFYVE20
MmugDNA.1727.1.S1_at	233255_s_at	BIVM	basic, immunoglobulin-like variable motif containing	chr13q32-q33.1		54841	ERCC5	excision repair cross-complementing rodent repair deficiency, complementation group 5	---		707822	MmugDNA.1727.1.S1_at_BIVM
MmugDNA.17272.1.S1_at	1556504_at	---	---	---	---		---	---	---	---		MmugDNA.17272.1.S1_at_---
MmugDNA.17273.1.S1_at	NA	NA	NA	NA	NA		NA	NA	NA	NA		MmugDNA.17273.1.S1_at_NA
MmugDNA.17274.1.S1_at	1569121_at	SLC25A24	solute carrier family 25 (mitochondrial carrier; phosphate carrier), member 24	chr1p13.3		29957	---	---	---	---		MmugDNA.17274.1.S1_at_SLC25A24
MmugDNA.17275.1.S1_s_at	213441_x_at	SPDEF	SAM pointed domain containing ets transcription factor	chr6p21.3		25803	LOC718648	similar to SAM pointed domain containing ets transcription factor	---		718648	MmugDNA.17275.1.S1_s_at_SPDEF
MmugDNA.17276.1.S1_at	1554026_a_at	MYO10	myosin X	chr5p15.1-p14.3		4651	---	---	---	---		MmugDNA.17276.1.S1_at_MYO10
MmugDNA.17277.1.S1_at	204333_s_at	AGA	aspartylglucosaminidase	chr4q32-q33		175	AGA	aspartylglucosaminidase	---		699740	MmugDNA.17277.1.S1_at_AGA
MmugDNA.17278.1.S1_at	235920_at	---	---	---	---		---	---	---	---		MmugDNA.17278.1.S1_at_---
MmugDNA.17279.1.S1_at	226603_at	SAMD9L	sterile alpha motif domain containing 9-like	chr7q21.2-q21.3		219285	LOC701572	similar to sterile alpha motif domain containing 9-like	---		701572	MmugDNA.17279.1.S1_at_SAMD9L
MmugDNA.17280.1.S1_at	241404_at	---	---	---	---		---	---	---	---		MmugDNA.17280.1.S1_at_---
MmugDNA.17281.1.S1_at	243247_at	LOC100129525	Hypothetical protein LOC100129525	chr8q24.21		100129525	---	---	---	---		MmugDNA.17281.1.S1_at_LOC100129
MmugDNA.1728.1.S1_at	216959_x_at	NRCAM	neuronal cell adhesion molecule	chr7q31.1-q31.2		4897	---	---	---	---		MmugDNA.1728.1.S1_at_NRCAM
MmugDNA.17282.1.S1_at	244523_at	MMD	monocyte to macrophage differentiation-associated	chr17q		23531	---	---	---	---		MmugDNA.17282.1.S1_at_MMD
MmugDNA.17283.1.S1_at	NA	NA	NA	NA	NA		NA	NA	NA	NA		MmugDNA.17283.1.S1_at_NA
MmugDNA.17284.1.S1_at	242007_at	---	---	---	---		---	---	---	---		MmugDNA.17284.1.S1_at_---
MmugDNA.17285.1.S1_at	239305_at	---	---	---	---		---	---	---	---		MmugDNA.17285.1.S1_at_---
MmugDNA.17286.1.S1_at	223008_s_at	C9orf5	chromosome 9 open reading frame 5	chr9q31		23731	---	---	---	---		MmugDNA.17286.1.S1_at_C9orf5
MmugDNA.17287.1.S1_at	230533_at	ZMYND8	zinc finger, MYND-type containing 8	chr20q13.12		23613	---	---	---	---		MmugDNA.17287.1.S1_at_ZMYND8
MmugDNA.17288.1.S1_at	240682_at	BEYLA	hypothetical LOC497634	---		497634	---	---	---	---		MmugDNA.17288.1.S1_at_BEYLA
MmugDNA.17289.1.S1_at	243102_at	---	---	---	---		---	---	---	---		MmugDNA.17289.1.S1_at_---
MmugDNA.17290.1.S1_at	224221_s_at	VAV3	vav 3 guanine nucleotide exchange factor	chr1p13.3		10451	VAV3	vav 3 guanine nucleotide exchange factor	---		694685	MmugDNA.17290.1.S1_at_VAV3
MmugDNA.17291.1.S1_at	233832_at	---	---	---	---		---	---	---	---		MmugDNA.17291.1.S1_at_---
MmugDNA.17292.1.S1_at	214933_at	CACNA1A	calcium channel, voltage-dependent, P/Q type, alpha 1A subunit	chr19p13.2-p13.1		773	---	---	---	---		MmugDNA.17292.1.S1_at_CACNA1A
MmugDNA.17293.1.S1_s_at	218806_s_at	VAV3	vav 3 guanine nucleotide exchange factor	chr1p13.3		10451	VAV3	vav 3 guanine nucleotide exchange factor	---		694685	MmugDNA.17293.1.S1_s_at_VAV3
MmugDNA.17294.1.S1_at	228831_s_at	GNG7	guanine nucleotide binding protein (G protein), gamma 7	chr19p13.3		2788	---	---	---	---		MmugDNA.17294.1.S1_at_GNG7
MmugDNA.17295.1.S1_at	203566_s_at	AGL	amylo-1, 6-glucosidase, 4-alpha-glucanotransferase	chr1p21		178	LOC710910	similar to amylo-1,6-glucosidase, 4-alpha-glucanotransferase isoform 2	---		710910	MmugDNA.17295.1.S1_at_AGL
MmugDNA.17296.1.S1_at	243951_at	ABCB1	ATP-binding cassette, sub-family B (MDR/TAP), member 1	chr7q21.1		5243	---	---	---	---		MmugDNA.17296.1.S1_at_ABCB1
MmugDNA.17297.1.S1_at	1569620_s_at	LOC100128079	hypothetical protein LOC100128079	chr16p11.2		100128079	---	---	---	---		MmugDNA.17297.1.S1_at_LOC100128
MmugDNA.17298.1.S1_at	1559663_at	---	---	---	---		---	---	---	---		MmugDNA.17298.1.S1_at_---
MmugDNA.17299.1.S1_at	1553436_at	MUC19	mucin 19, oligomeric	chr12q12		283463	---	---	---	---		MmugDNA.17299.1.S1_at_MUC19
MmugDNA.17300.1.S1_s_at	223859_at	EPB41L4B	erythrocyte membrane protein band 4.1 like 4B	chr9q31-q32		54566	LOC713324	similar to erythrocyte membrane protein band 4.1 like 4B isoform 2	---		713324	MmugDNA.17300.1.S1_s_at_EPB41L4
MmugDNA.17301.1.S1_at	NA	NA	NA	NA	NA		NA	NA	NA	NA		MmugDNA.17301.1.S1_at_NA
MmugDNA.1730.1.S1_at	203099_s_at	CDYL	chromodomain protein, Y-like	chr6p25.1		9425	LOC714887	Similar to F-box and WD-40 domain protein 7, archipelago homolog	---		714887	MmugDNA.1730.1.S1_at_CDYL
MmugDNA.17303.1.S1_at	223426_s_at	EPB41L4B	erythrocyte membrane protein band 4.1 like 4B	chr9q31-q32		54566	---	---	---	---		MmugDNA.17303.1.S1_at_EPB41L4B
MmugDNA.17304.1.S1_at	1566991_at	ARID1B	AT rich interactive domain 1B (SWI1-like)	chr6q25.1		57492	---	---	---	---		MmugDNA.17304.1.S1_at_ARID1B
MmugDNA.17305.1.S1_at	1554763_at	UBE2DNL	ubiquitin-conjugating enzyme E2D N-terminal like pseudogene	chrXq21.1		100131816	---	---	---	---		MmugDNA.17305.1.S1_at_UBE2DNL
MmugDNA.17306.1.S1_at	232939_at	---	---	---	---		---	---	---	---		MmugDNA.17306.1.S1_at_---
MmugDNA.17307.1.S1_at	237771_s_at	---	---	---	---		---	---	---	---		MmugDNA.17307.1.S1_at_---
MmugDNA.17308.1.S1_at	239404_at	---	---	---	---		---	---	---	---		MmugDNA.17308.1.S1_at_---
MmugDNA.17309.1.S1_at	1557139_at	---	---	---	---		---	---	---	---		MmugDNA.17309.1.S1_at_---
MmugDNA.17310.1.S1_at	224886_at	JMJD8	jumonji domain containing 8	chr16p13.3		339123	LOC697226	hypothetical protein LOC697226	---		697226	MmugDNA.17310.1.S1_at_JMJD8
MmugDNA.17311.1.S1_at	216390_at	LCAT	Lecithin-cholesterol acyltransferase	chr16q22.1		3931	---	---	---	---		MmugDNA.17311.1.S1_at_LCAT
MmugDNA.1731.1.S1_at	226116_at	---	---	---	---		---	---	---	---		MmugDNA.1731.1.S1_at_---
MmugDNA.17312.1.S1_at	1555469_a_at	CLASP2	cytoplasmic linker associated protein 2	chr3p22.3		23122	LOC695424	Similar to Wiskott-Aldrich syndrome-like	---		695424	MmugDNA.17312.1.S1_at_CLASP2
MmugDNA.17313.1.S1_at	1564144_at	---	---	---	---		---	---	---	---		MmugDNA.17313.1.S1_at_---
MmugDNA.17314.1.S1_at	237616_at	---	---	---	---		---	---	---	---		MmugDNA.17314.1.S1_at_---

MmugDNA.17315.1.S1_at	216312_at	ATP2B3	ATPase, Ca++ transporting, plasma membrane 3	chrXq28		492	---	---	---	---		MmugDNA.17315.1.S1_at_ATP2B3
MmugDNA.17316.1.S1_at	NA	NA	NA	NA	NA		NA	NA	NA	NA		MmugDNA.17316.1.S1_at_NA
MmugDNA.17317.1.S1_at	1556847_s_at	---	---	---	---		---	---	---	---		MmugDNA.17317.1.S1_at_---
MmugDNA.17318.1.S1_at	230305_at	---	---	---	---		HNRPH1	Heterogeneous nuclear ribonucleoprotein H1	---		704469	MmugDNA.17318.1.S1_at_---
MmugDNA.17319.1.S1_at	208896_at	DDX18	DEAD (Asp-Glu-Ala-Asp) box polypeptide 18	chr2q14.1		8886	---	---	---	---		MmugDNA.17319.1.S1_at_DDX18
MmugDNA.173.1.S1_at	206564_at	OPRL1	opiate receptor-like 1	chr20q13.33		4987	---	---	---	---		MmugDNA.173.1.S1_at_OPRL1
MmugDNA.17320.1.S1_at	1562169_at	---	---	---	---		---	---	---	---		MmugDNA.17320.1.S1_at_---
MmugDNA.17321.1.S1_at	232252_at	DUSP27	dual specificity phosphatase 27 (putative)	chr1q22-q24		92235	DUSP27	dual specificity phosphatase 27 (putative)	---		697299	MmugDNA.17321.1.S1_at_DUSP27
MmugDNA.1732.1.S1_at	203783_x_at	POLRMT	polymerase (RNA) mitochondrial (DNA directed)	chr19p13.3		5442	---	---	---	---		MmugDNA.1732.1.S1_at_POLRMT
MmugDNA.17322.1.S1_at	208090_s_at	AIRE	autoimmune regulator	chr21q22.3		326	AIRE	autoimmune regulator	---		712858	MmugDNA.17322.1.S1_at_AIRE
MmugDNA.17323.1.S1_at	242084_at	LOC339316	hypothetical protein LOC339316	chr19q12		339316	---	---	---	---		MmugDNA.17323.1.S1_at_LOC339316
MmugDNA.17324.1.S1_at	1556842_at	LOC286087	hypothetical protein LOC286087	chr8p23.2		286087	---	---	---	---		MmugDNA.17324.1.S1_at_LOC286087
MmugDNA.17325.1.S1_at	218489_s_at	ALAD	aminolevulinate, delta-, dehydratase	chr9q33.1		210	ALAD	aminolevulinate, delta-, dehydratase	---		705193	MmugDNA.17325.1.S1_at_ALAD
MmugDNA.17326.1.S1_at	1569465_at	FAM160A1	family with sequence similarity 160, member A1	chr4q31.3		729830	---	---	---	---		MmugDNA.17326.1.S1_at_FAM160A1
MmugDNA.17327.1.S1_at	231495_at	---	---	---	---		---	---	---	---		MmugDNA.17327.1.S1_at_---
MmugDNA.17328.1.S1_at	239356_at	LOC100129122	Hypothetical protein LOC100129122	chr13q32.3		100129122	---	---	---	---		MmugDNA.17328.1.S1_at_LOC100129
MmugDNA.17329.1.S1_at	224428_s_at	CDCA7	cell division cycle associated 7	chr2q31		83879	---	---	---	---		MmugDNA.17329.1.S1_at_CDCA7
MmugDNA.17330.1.S1_at	237826_at	---	---	---	---		---	---	---	---		MmugDNA.17330.1.S1_at_---
MmugDNA.17331.1.S1_s_at	211924_s_at	PLAUR	plasminogen activator, urokinase receptor	chr19q13		5329	LOC710662	similar to plasminogen activator, urokinase receptor isoform 1 precursor	---		710662	MmugDNA.17331.1.S1_s_at_PLAUR
MmugDNA.1733.1.S1_at	NA	NA	NA	NA	NA		NA	NA	NA	NA		MmugDNA.1733.1.S1_at_NA
MmugDNA.1733.1.S1_s_at	210951_x_at	RAB27A	RAB27A, member RAS oncogene family	chr15q15-q21.1		5873	LOC697741	similar to Ras-related protein Rab-27A	---		697741	MmugDNA.1733.1.S1_s_at_RAB27A
MmugDNA.17332.1.S1_at	239225_at	---	---	---	---		---	---	---	---		MmugDNA.17332.1.S1_at_---
MmugDNA.17333.1.S1_x_at	1560391_at	---	---	---	---		---	---	---	---		MmugDNA.17333.1.S1_x_at_---
MmugDNA.17334.1.S1_at	1561468_at	FAM45B	Family with sequence similarity 45, member B	chrXq25		55855	---	---	---	---		MmugDNA.17334.1.S1_at_FAM45B
MmugDNA.17335.1.S1_at	216036_x_at	WDTC1	WD and tetratricopeptide repeats 1	chr1p36.11		23038	LOC715790	similar to WD and tetratricopeptide repeats 1	---		715790	MmugDNA.17335.1.S1_at_WDTC1
MmugDNA.17336.1.S1_s_at	224575_at	C3orf10	chromosome 3 open reading frame 10	chr3p25.3		55845	LOC717243	similar to chromosome 3 open reading frame 10	---		717243	MmugDNA.17336.1.S1_s_at_C3orf10
MmugDNA.17337.1.S1_at	228545_at	ZNF148	zinc finger protein 148	chr3q21		7707	ZNF148	zinc finger protein 148	---		715925	MmugDNA.17337.1.S1_at_ZNF148
MmugDNA.17338.1.S1_at	1560372_at	---	---	---	---		---	---	---	---		MmugDNA.17338.1.S1_at_---
MmugDNA.17339.1.S1_at	209840_s_at	LRRN3	leucine rich repeat neuronal 3	chr7q31.1		54674	LOC698135	Hypothetical protein LOC698135	---		698135	MmugDNA.17339.1.S1_at_LRRN3
MmugDNA.17339.1.S1_s_at	NA	NA	NA	NA	NA		NA	NA	NA	NA		MmugDNA.17339.1.S1_s_at_NA
MmugDNA.17340.1.S1_at	1562057_at	---	---	---	---		---	---	---	---		MmugDNA.17340.1.S1_at_---
MmugDNA.17341.1.S1_at	215190_at	EIF3M	eukaryotic translation initiation factor 3, subunit M	chr11p13		10480	---	---	---	---		MmugDNA.17341.1.S1_at_EIF3M
MmugDNA.1734.1.S1_at	219888_at	SPAG4	sperm associated antigen 4	chr20q11.21		6676	SPAG4	sperm associated antigen 4	---		704522	MmugDNA.1734.1.S1_at_SPAG4
MmugDNA.17342.1.S1_s_at	214894_x_at	MACF1	microtubule-actin crosslinking factor 1	chr1p32-p31		23499	LOC718651	similar to microfilament and actin filament cross-linker protein isoform a	---		718651	MmugDNA.17342.1.S1_s_at_MACF1
MmugDNA.17343.1.S1_at	239735_at	---	---	---	---		---	---	---	---		MmugDNA.17343.1.S1_at_---
MmugDNA.17344.1.S1_at	206706_at	NTF3	neurotrophin 3	chr12p13		4908	NTF3	neurotrophin 3	---		721988	MmugDNA.17344.1.S1_at_NTF3
MmugDNA.17345.1.S1_s_at	214113_s_at	RBM8A	RNA binding motif protein 8A	chr1q12		9939	LDHA	Similar to L-lactate dehydrogenase A chain (LDH-A) (LDH muscle subunit) (LDH-M) (Proliferation-ind	---		693898	MmugDNA.17345.1.S1_s_at_RBM8A
MmugDNA.17346.1.S1_at	238967_at	---	---	---	---		---	---	---	---		MmugDNA.17346.1.S1_at_---
MmugDNA.17347.1.S1_at	226650_at	ZFAND2A	zinc finger, AN1-type domain 2A	chr7p22.3		90637	---	---	---	---		MmugDNA.17347.1.S1_at_ZFAND2A
MmugDNA.17348.1.S1_at	201365_at	OAZ2	ornithine decarboxylase antizyme 2	chr15q22.31		4947	OAZ2	ornithine decarboxylase antizyme 2	---		707593	MmugDNA.17348.1.S1_at_OAZ2
MmugDNA.17349.1.S1_at	1557553_at	PPP1R12B	Protein phosphatase 1, regulatory (inhibitor) subunit 12B	chr1q32.1		4660	---	---	---	---		MmugDNA.17349.1.S1_at_PPP1R12B
MmugDNA.17350.1.S1_at	225417_at	EPC1	enhancer of polycomb homolog 1 (Drosophila)	chr10p11		80314	---	---	---	---		MmugDNA.17350.1.S1_at_EPC1
MmugDNA.17351.1.S1_at	1570299_at	---	---	---	---		---	---	---	---		MmugDNA.17351.1.S1_at_---
MmugDNA.1735.1.S1_at	235843_at	---	---	---	---		---	---	---	---		MmugDNA.1735.1.S1_at_---
MmugDNA.17352.1.S1_at	1562168_at	---	---	---	---		---	---	---	---		MmugDNA.17352.1.S1_at_---
MmugDNA.17353.1.S1_at	1554891_at	---	---	---	---		---	---	---	---		MmugDNA.17353.1.S1_at_---
MmugDNA.17354.1.S1_at	222928_s_at	FAM160B2	family with sequence similarity 160, member B2	chr8p21.3		64760	---	---	---	---		MmugDNA.17354.1.S1_at_FAM160B2
MmugDNA.17355.1.S1_at	1552716_at	SPEF2	sperm flagellar 2	chr5p13.2		79925	LOC703498	similar to KPL2 protein isoform 1	---		703498	MmugDNA.17355.1.S1_at_SPEF2
MmugDNA.17356.1.S1_x_at	NA	NA	NA	NA	NA		NA	NA	NA	NA		MmugDNA.17356.1.S1_x_at_NA
MmugDNA.17357.1.S1_at	228800_x_at	AURKAIP1	aurora kinase A interacting protein 1	chr1p36.33		54998	IRF6	Interferon regulatory factor 6	---		712958	MmugDNA.17357.1.S1_at_AURKAIP1
MmugDNA.17358.1.S1_at	240400_at	---	---	---	---		---	---	---	---		MmugDNA.17358.1.S1_at_---
MmugDNA.17359.1.S1_at	227885_at	LOC400236	Hypothetical LOC400236	chr14q32.11		400236	---	---	---	---		MmugDNA.17359.1.S1_at_LOC400236
MmugDNA.17360.1.S1_at	1555238_at	PTH2	parathyroid hormone 2	chr19q13.33		113091	LOC718758	similar to tuberoinfundibular 39 residue protein precursor	---		718758	MmugDNA.17360.1.S1_at_PTH2
MmugDNA.17361.1.S1_at	243197_at	---	---	---	---		---	---	---	---		MmugDNA.17361.1.S1_at_---
MmugDNA.1736.1.S1_at	210273_at	PCDH7	protocadherin 7	chr4p15		5099	CUTL1	Cut-like 1	---		574319	MmugDNA.1736.1.S1_at_PCDH7
MmugDNA.17362.1.S1_at	227268_at	RNFT1	ring finger protein, transmembrane 1	chr17q23.1		51136	LOC707102	Similar to far upstream element-binding protein	---		707102	MmugDNA.17362.1.S1_at_RNFT1
MmugDNA.17363.1.S1_at	207419_s_at	RAC2	ras-related C3 botulinum toxin substrate 2 (rho family, small GTP binding protein Rac2)	chr22q13.1		5880	RAC2	ras-related C3 botulinum toxin substrate 2 (rho family, small GTP binding protein Rac2)	---		696709	MmugDNA.17363.1.S1_at_RAC2
MmugDNA.17364.1.S1_at	233923_at	FLJ32810	Rho-type GTPase-activating protein FLJ32810	chr11q22.1		143872	---	---	---	---		MmugDNA.17364.1.S1_at_FLJ32810
MmugDNA.17365.1.S1_at	203186_s_at	S100A4	S100 calcium binding protein A4	chr1q21		6275	S100A4	S100 calcium binding protein A4	---		715115	MmugDNA.17365.1.S1_at_S100A4
MmugDNA.17366.1.S1_s_at	NA	NA	NA	NA	NA		NA	NA	NA	NA		MmugDNA.17366.1.S1_s_at_NA
MmugDNA.17367.1.S1_at	215068_s_at	FBXL18	F-box and leucine-rich repeat protein 18	chr7p22.2		80028	LOC716863	similar to F-box and leucine-rich repeat protein 18	---		716863	MmugDNA.17367.1.S1_at_FBXL18
MmugDNA.17368.1.S1_at	234211_at	---	---	---	---		---	---	---	---		MmugDNA.17368.1.S1_at_---
MmugDNA.17369.1.S1_at	239575_at	OPALIN	oligodendrocytic myelin paranodal and inner loop protein	chr10q23-q24		93377	---	---	---	---		MmugDNA.17369.1.S1_at_OPALIN
MmugDNA.17370.1.S1_at	217728_at	S100A6	S100 calcium binding protein A6	chr1q21		6277	S100A6	S100 calcium binding protein A6	---		715169	MmugDNA.17370.1.S1_at_S100A6
MmugDNA.17370.1.S1_s_at	228923_at	S100A6	S100 calcium binding protein A6	chr1q21		6277	S100A6	S100 calcium binding protein A6	---		715169	MmugDNA.17370.1.S1_s_at_S100A6
MmugDNA.17371.1.S1_at	244204_at	---	---	---	---		---	---	---	---		MmugDNA.17371.1.S1_at_---
MmugDNA.1737.1.S1_at	1564733_at	---	---	---	---		---	---	---	---		MmugDNA.1737.1.S1_at_---
MmugDNA.17372.1.S1_at	242687_at	FAM160A1	family with sequence similarity 160, member A1	chr4q31.3		729830	---	---	---	---		MmugDNA.17372.1.S1_at_FAM160A1
MmugDNA.17373.1.S1_at	205916_at	S100A7	S100 calcium binding protein A7	chr1q21		6278	S100A7	S100 calcium binding protein A7	---		714943	MmugDNA.17373.1.S1_at_S100A7
MmugDNA.17374.1.S1_at	230823_at	---	---	---	---		---	---	---	---		MmugDNA.17374.1.S1_at_---
MmugDNA.17375.1.S1_at	1559921_at	PECAM1	platelet/endothelial cell adhesion molecule	chr17q23		5175	---	---	---	---		MmugDNA.17375.1.S1_at_PECAM1
MmugDNA.17376.1.S1_at	202917_s_at	S100A8	S100 calcium binding protein A8	chr1q21		6279	S100A8	S100 calcium binding protein A8	---		714740	MmugDNA.17376.1.S1_at_S100A8
MmugDNA.17377.1.S1_s_at	214786_at	MAP3K1	mitogen-activated protein kinase kinase kinase 1	chr5q11.2		4214	MAP3K1	mitogen-activated protein kinase kinase kinase 1	---		710979	MmugDNA.17377.1.S1_s_at_MAP3K1
MmugDNA.17378.1.S1_at	231069_at	---	---	---	---		---	---	---	---		MmugDNA.17378.1.S1_at_---
MmugDNA.17379.1.S1_at	239912_at	---	---	---	---		---	---	---	---		MmugDNA.17379.1.S1_at_---
MmugDNA.17380.1.S1_at	244778_x_at	---	---	---	---		---	---	---	---		MmugDNA.17380.1.S1_at_---
MmugDNA.17381.1.S1_at	221617_at	TAF9B	TAF9B RNA polymerase II, TATA box binding protein (TBP)-associated factor, 31kDa	chrXq13.1-q21.1		51616	---	---	---	---		MmugDNA.17381.1.S1_at_TAF9B
MmugDNA.1738.1.S1_at	233315_at	---	---	---	---		---	---	---	---		MmugDNA.1738.1.S1_at_---
MmugDNA.17382.1.S1_at	1554121_at	HSD17B12	hydroxysteroid (17-beta) dehydrogenase 12	chr11p11.2		51144	---	---	---	---		MmugDNA.17382.1.S1_at_HSD17B12
MmugDNA.17383.1.S1_at	NA	NA	NA	NA	NA		NA	NA	NA	NA		MmugDNA.17383.1.S1_at_NA
MmugDNA.17384.1.S1_at	228787_s_at	BCAS4	breast carcinoma amplified sequence 4	chr20q13.13		55653	BCAS4	breast carcinoma amplified sequence 4	---		701649	MmugDNA.17384.1.S1_at_BCAS4
MmugDNA.17385.1.S1_at	229540_at	RBPJ	recombination signal binding protein for immunoglobulin kappa J region	chr4p15.2		3516	RBPJ	recombination signal binding protein for immunoglobulin kappa J region	---		694601	MmugDNA.17385.1.S1_at_RBPJ
MmugDNA.17386.1.S1_at	1563693_at	---	---	---	---		---	---	---	---		MmugDNA.17386.1.S1_at_---
MmugDNA.17387.1.S1_at	1568822_at	GTPBP5	GTP binding protein 5 (putative)	chr20q13.33		26164	GTPBP5	GTP binding protein 5 (putative)	---		700182	MmugDNA.17387.1.S1_at_GTPBP5
MmugDNA.17388.1.S1_at	1553689_s_at	METTL6	methyltransferase like 6	chr3p24.3		131965	METTL6	methyltransferase like 6	---		694202	MmugDNA.17388.1.S1_at_METTL6
MmugDNA.17389.1.S1_at	234943_at	CSNK1E /// LOC40092	casein kinase 1, epsilon /// TPTE and PTEN homologous inositol lipid phosphatase pseudogene /// T	chr22q11.1 /// chr22q13.1	1454 /// 387590 /// 400		LOC704870	similar to TPTE and PTEN homologous inositol lipid phosphatase isoform gamma	---		704870	MmugDNA.17389.1.S1_at_CSNK1E /// I
MmugDNA.17390.1.S1_at	1553930_at	TAAR1	trace amine associated receptor 1	chr6q23.2		134864	TAR1	trace amine receptor 1	---		708944	MmugDNA.17390.1.S1_at_TAAR1
MmugDNA.17391.1.S1_at	226077_at	RNF145	ring finger protein 145	chr5q33.3		153830	RNF145	ring finger protein 145	---		694354	MmugDNA.17391.1.S1_at_RNF145
MmugDNA.1739.1.S1_at	242011_at	---	---	---	---		---	---	---	---		MmugDNA.1739.1.S1_at_---
MmugDNA.17392.1.S1_at	1559686_a_at	---	---	---	---		LOC702332	hypothetical protein LOC702332	---		702332	MmugDNA.17392.1.S1_at_---
MmugDNA.17393.1.S1_at	210028_s_at	ORC3L	origin recognition complex, subunit 3-like (yeast)	chr6q14.3-q16.1		23595	ORC3L	origin recognition complex, subunit 3-like (yeast)	---		700773	MmugDNA.17393.1.S1_at_ORC3L
MmugDNA.17394.1.S1_at	1569953_at	---	---	---	---		---	---	---	---		MmugDNA.17394.1.S1_at_---
MmugDNA.17395.1.S1_at	202808_at	C10orf26	chromosome 10 open reading frame 26	chr10q24.32		54838	LOC713887	hypothetical protein LOC713887	---		713887	MmugDNA.17395.1.S1_at_C10orf26
MmugDNA.17396.1.S1_at	238824_at	---	---	---	---		---	---	---	---		MmugDNA.17396.1.S1_at_---
MmugDNA.17397.1.S1_at	233134_at	RPH3AL	rabphilin 3A-like (without C2 domains)	chr17p13.3		9501	---	---	---	---		MmugDNA.17397.1.S1_at_RPH3AL
MmugDNA.17398.1.S1_at	1557224_at	---	---	---	---		---	---	---	---		MmugDNA.17398.1.S1_at_---
MmugDNA.17399.1.S1_at	1558804_at	---	---	---	---		---	---	---	---		MmugDNA.17399.1.S1_at_---
MmugDNA.17400.1.S1_at	218313_s_at	GALNT7	UDP-N-acetyl-alpha-D-galactosamine:polypeptide N-acetylgalactosaminyltransferase 7 (GalNAc-T7)	chr4q31.1		51809	GALNT7	UDP-N-acetyl-alpha-D-galactosamine:polypeptide N-acetylgalactosaminyltransferase 7 (GalNAc-T7)	---		696924	MmugDNA.17400.1.S1_at_GALNT7
MmugDNA.17401.1.S1_at	244841_at	SEC24A	SEC24 family, member A (S. cerevisiae)	chr5q31.1		10802	SEC24A	SEC24 family, member A (S. cerevisiae)	---		711109	MmugDNA.17401.1.S1_at_SEC24A
MmugDNA.1740.1.S1_at	230650_at	---	---	---	---		---	---	---	---		MmugDNA.1740.1.S1_at_---
MmugDNA.17402.1.S1_at	239930_at	GALNT2	UDP-N-acetyl-alpha-D-galactosamine:polypeptide N-acetylgalactosaminyltransferase 2 (GalNAc-T2)	chr1q41-q42		2590	---	---	---	---		MmugDNA.17402.1.S1_at_GALNT2
MmugDNA.17403.1.S1_at	224246_at	---	---	---	---		---	---	---	---		MmugDNA.17403.1.S1_at_---
MmugDNA.17404.1.S1_s_at	201981_at	PAPPA	pregnancy-associated plasma protein A, pappalysin 1	chr9q33.2		5069	---	---	---	---		MmugDNA.17404.1.S1_s_at_PAPPA
MmugDNA.17405.1.S1_at	236282_at	---	---	---	---		---	---	---	---		MmugDNA.17405.1.S1_at_---
MmugDNA.17406.1.S1_at	NA	NA	NA	NA	NA		NA	NA	NA	NA		MmugDNA.17406.1.S1_at_NA
MmugDNA.17407.1.S1_at	228864_at	ZNF653	zinc finger protein 653	chr19p13.2		115950	LOC714754	similar to zinc finger protein 653	---		714754	MmugDNA.17407.1.S1_at_ZNF653
MmugDNA.17408.1.S1_at	1558154_at	---	---	---	---		---	---	---	---		MmugDNA.17408.1.S1_at_---
MmugDNA.17409.1.S1_s_at	NA	NA	NA	NA	NA		NA	NA	NA	NA		MmugDNA.17409.1.S1_s_at_NA

MmugDNA.17410.1.S1_at	1554595_at	SYMPK	symplekin	chr19q13.3	8189	---	---	---	---	MmugDNA.17410.1.S1_at_SYMPK
MmugDNA.17411.1.S1_at	228537_at	GLI2	GLI family zinc finger 2	chr2q14	2736	---	---	---	---	MmugDNA.17411.1.S1_at_GLI2
MmugDNA.1741.1.S1_at	222655_s_at	IMPAD1	inositol monophosphatase domain containing 1	chr8q12.1	54928	---	---	---	---	MmugDNA.1741.1.S1_at_IMPAD1
MmugDNA.17412.1.S1_at	237356_at	---	---	---	---	---	---	---	---	MmugDNA.17412.1.S1_at_---
MmugDNA.17413.1.S1_at	225401_at	C1orf85	chromosome 1 open reading frame 85	chr1q22	112770	LOC719468	similar to kidney predominant protein NCU-G1	---	719468	MmugDNA.17413.1.S1_at_C1orf85
MmugDNA.17414.1.S1_at	226940_at	FAM69B	Family with sequence similarity 69, member B	chr9q34.3	138311	---	---	---	---	MmugDNA.17414.1.S1_at_FAM69B
MmugDNA.17414.1.S1_s_at	229003_x_at	FAM69B	Family with sequence similarity 69, member B	chr9q34.3	138311	---	---	---	---	MmugDNA.17414.1.S1_s_at_FAM69B
MmugDNA.17415.1.S1_at	242476_at	---	---	---	---	---	---	---	---	MmugDNA.17415.1.S1_at_---
MmugDNA.17416.1.S1_at	216377_x_at	ALPPL2	alkaline phosphatase, placental-like 2	chr2q37	251	ALPP	placental alkaline phosphatase	---	712965	MmugDNA.17416.1.S1_at_ALPPL2
MmugDNA.17417.1.S1_at	1565927_s_at	---	---	---	---	---	---	---	---	MmugDNA.17417.1.S1_at_---
MmugDNA.17418.1.S1_at	1569975_at	---	---	---	---	---	---	---	---	MmugDNA.17418.1.S1_at_---
MmugDNA.17419.1.S1_at	220929_at	GALNT8	UDP-N-acetyl-alpha-D-galactosamine:polypeptide N-acetylgalactosaminyltransferase 8 (GalNAc-T8)	chr12p13.3	26290	LOC721915	similar to Probable polypeptide N-acetylgalactosaminyltransferase 8 (Protein-UDP acetylgalactosami	---	721915	MmugDNA.17419.1.S1_at_GALNT8
MmugDNA.174.1.S1_at	226737_at	SLC25A42	solute carrier family 25, member 42	chr19p13.11	284439	---	---	---	---	MmugDNA.174.1.S1_at_SLC25A42
MmugDNA.17420.1.S1_at	238502_at	---	---	---	---	---	---	---	---	MmugDNA.17420.1.S1_at_---
MmugDNA.17421.1.S1_at	231295_at	ME3	malic enzyme 3, NADP(+)-dependent, mitochondrial	chr11cen-q22.3	10873	ME3	malic enzyme 3, NADP(+)-dependent, mitochondrial	---	704422	MmugDNA.17421.1.S1_at_ME3
MmugDNA.1742.1.S1_at	222654_at	IMPAD1	inositol monophosphatase domain containing 1	chr8q12.1	54928	---	---	---	---	MmugDNA.1742.1.S1_at_IMPAD1
MmugDNA.17422.1.S1_at	216239_at	BBS9	Bardet-Biedl syndrome 9	chr7p14	27241	BBS9	similar to parathyroid hormone-responsive B1 isoform 2	---	708412	MmugDNA.17422.1.S1_at_BBS9
MmugDNA.17423.1.S1_at	234378_at	---	---	---	---	---	---	---	---	MmugDNA.17423.1.S1_at_---
MmugDNA.17424.1.S1_at	1562805_at	LOC349408	hypothetical protein LOC349408	chrXp22.31	349408	---	---	---	---	MmugDNA.17424.1.S1_at_LOC349408
MmugDNA.17425.1.S1_at	226618_at	FLJ25076	probable ubiquitin-conjugating enzyme E2 FLJ25076	chr5p15.31	134111	---	---	---	---	MmugDNA.17425.1.S1_at_FLJ25076
MmugDNA.17426.1.S1_at	226612_at	FLJ25076	probable ubiquitin-conjugating enzyme E2 FLJ25076	chr5p15.31	134111	---	---	---	---	MmugDNA.17426.1.S1_at_FLJ25076
MmugDNA.17427.1.S1_at	235190_at	---	---	---	---	---	---	---	---	MmugDNA.17427.1.S1_at_---
MmugDNA.17428.1.S1_at	210530_s_at	NR2C1	nuclear receptor subfamily 2, group C, member 1	chr12q22	7181	---	---	---	---	MmugDNA.17428.1.S1_at_NR2C1
MmugDNA.17429.1.S1_at	1570469_at	---	---	---	---	---	---	---	---	MmugDNA.17429.1.S1_at_---
MmugDNA.17430.1.S1_at	212457_at	TFE3	transcription factor binding to IGHM enhancer 3	chrXp11.22	7030	LOC712161	similar to Transcription factor E3	---	712161	MmugDNA.17430.1.S1_at_TFE3
MmugDNA.17430.1.S1_s_at	NA	NA	NA	NA	NA	NA	NA	NA	NA	MmugDNA.17430.1.S1_s_at_NA
MmugDNA.17431.1.S1_at	1561692_at	---	---	---	---	---	---	---	---	MmugDNA.17431.1.S1_at_---
MmugDNA.1743.1.S1_at	239920_at	UBTF	Upstream binding transcription factor, RNA polymerase I	chr17q21.3	7343	---	---	---	---	MmugDNA.1743.1.S1_at_UBTF
MmugDNA.17432.1.S1_at	1563456_at	CCDC141 /// LOC28502	coiled-coil domain containing 141 /// hypothetical protein LOC285026	chr2q31.2	285025 /// 285026	---	---	---	---	MmugDNA.17432.1.S1_at_CCDC141 ///
MmugDNA.17433.1.S1_at	226729_at	USP37	ubiquitin specific peptidase 37	chr2q35	57695	---	---	---	---	MmugDNA.17433.1.S1_at_USP37
MmugDNA.17434.1.S1_at	1557843_at	---	---	---	---	---	---	---	---	MmugDNA.17434.1.S1_at_---
MmugDNA.17435.1.S1_at	1558451_at	LOC285500	hypothetical protein LOC285500	chr4q34.3	285500	---	---	---	---	MmugDNA.17435.1.S1_at_LOC285500
MmugDNA.17436.1.S1_at	242660_at	C10orf112	chromosome 10 open reading frame 112	chr10p12.33	340895	LOC707769	Similar to junction plakoglobin	---	707769	MmugDNA.17436.1.S1_at_C10orf112
MmugDNA.17437.1.S1_at	232085_at	MAPK8IP3	mitogen-activated protein kinase 8 interacting protein 3	chr16p13.3	23162	MAPK8IP3	mitogen-activated protein kinase 8 interacting protein 3	---	702388	MmugDNA.17437.1.S1_at_MAPK8IP3
MmugDNA.17438.1.S1_at	235210_s_at	C8orf84	chromosome 8 open reading frame 84	chr8q21.11	157869	---	---	---	---	MmugDNA.17438.1.S1_at_C8orf84
MmugDNA.17439.1.S1_at	230379_x_at	C2orf56	chromosome 2 open reading frame 56	chr2p22.2	55471	LOC709473	similar to CG17726-PA	---	709473	MmugDNA.17439.1.S1_at_C2orf56
MmugDNA.17440.1.S1_at	NA	NA	NA	NA	NA	NA	NA	NA	NA	MmugDNA.17440.1.S1_at_NA
MmugDNA.17441.1.S1_at	240750_at	---	---	---	---	---	---	---	---	MmugDNA.17441.1.S1_at_---
MmugDNA.1744.1.S1_at	233198_at	GOLGA2L1	golgi autoantigen, golgin subfamily a, 2-like 1	chr12q23.1	55592	---	---	---	---	MmugDNA.1744.1.S1_at_GOLGA2L1
MmugDNA.17442.1.S1_at	229298_at	KBTBD7	kelch repeat and BTB (POZ) domain containing 7	chr13q14.11	84078	KBTBD7	kelch repeat and BTB (POZ) domain containing 7	---	698337	MmugDNA.17442.1.S1_at_KBTBD7
MmugDNA.17443.1.S1_at	239507_at	LOC151300	hypothetical LOC151300	chr2q35	151300	---	---	---	---	MmugDNA.17443.1.S1_at_LOC151300
MmugDNA.17444.1.S1_at	230977_at	NPM2	nucleophosmin/nucleoplasmin, 2	chr8p21.3	10361	LOC715448	similar to nucleoplasmin 2	---	715448	MmugDNA.17444.1.S1_at_NPM2
MmugDNA.17445.1.S1_at	233413_at	---	---	---	---	---	---	---	---	MmugDNA.17445.1.S1_at_---
MmugDNA.17446.1.S1_at	232326_at	C8orf56	chromosome 8 open reading frame 56	chr8q22.3	157556	---	---	---	---	MmugDNA.17446.1.S1_at_C8orf56
MmugDNA.17447.1.S1_at	213666_at	SEPT6	septin 6	chrXq24	23157	---	---	---	---	MmugDNA.17447.1.S1_at_SEPT6
MmugDNA.17448.1.S1_at	212513_s_at	USP33	ubiquitin specific peptidase 33	chr1p31.1	23032	LOC710693	hypothetical protein LOC710693	---	710693	MmugDNA.17448.1.S1_at_USP33
MmugDNA.17449.1.S1_at	206715_at	TFEC	transcription factor EC	chr7q31.2	22797	TFEC	transcription factor EC	---	704124	MmugDNA.17449.1.S1_at_TFEC
MmugDNA.17450.1.S1_at	222942_s_at	TIAM2	T-cell lymphoma invasion and metastasis 2	chr6q25.2	26230	LOC701688	similar to T-cell lymphoma invasion and metastasis 2 isoform a	---	701688	MmugDNA.17450.1.S1_at_TIAM2
MmugDNA.17451.1.S1_at	203317_at	PSD4	pleckstrin and Sec7 domain containing 4	chr2q13	23550	---	---	---	---	MmugDNA.17451.1.S1_at_PSD4
MmugDNA.1745.1.S1_at	1554821_a_at	ZBED1	zinc finger, BED-type containing 1	chrXp22.33;Yp11	9189	---	---	---	---	MmugDNA.1745.1.S1_at_ZBED1
MmugDNA.17452.1.S1_s_at	210792_x_at	SIVA1	SIVA1, apoptosis-inducing factor	chr14q32.33	10572	SIVA1	SIVA1, apoptosis-inducing factor	---	706688	MmugDNA.17452.1.S1_s_at_SIVA1
MmugDNA.17453.1.S1_at	1555235_s_at	IQCF3	IQ motif containing F3	chr3p21.1	401067	---	---	---	---	MmugDNA.17453.1.S1_at_IQCF3
MmugDNA.17454.1.S1_s_at	209719_x_at	SERPINB3	serpin peptidase inhibitor, clade B (ovalbumin), member 3	chr18q21.3	6317	SERPINB3 /// SERPINB4	serpin peptidase inhibitor, clade B (ovalbumin), member 3 /// serpin peptidase inhibitor, clade B (ovalt	---	701189 /// 701315	MmugDNA.17454.1.S1_s_at_SERPINB:
MmugDNA.17455.1.S1_at	237452_at	---	---	---	---	---	---	---	---	MmugDNA.17455.1.S1_at_---
MmugDNA.17456.1.S1_at	233853_at	---	---	---	---	---	---	---	---	MmugDNA.17456.1.S1_at_---
MmugDNA.17457.1.S1_at	229784_at	MGC16121	hypothetical protein MGC16121	chrXq26.3	84848	LOC707702	hypothetical protein LOC707702	---	707702	MmugDNA.17457.1.S1_at_MGC16121
MmugDNA.17458.1.S1_at	211507_s_at	MTMR3	myotubularin related protein 3	chr22q12.2	8897	LOC715214	similar to myotubularin-related protein 3 isoform c	---	715214	MmugDNA.17458.1.S1_at_MTMR3
MmugDNA.17458.1.S1_s_at	NA	NA	NA	NA	NA	NA	NA	NA	NA	MmugDNA.17458.1.S1_s_at_NA
MmugDNA.17459.1.S1_at	1570425_s_at	LATS1	LATS, large tumor suppressor, homolog 1 (Drosophila)	chr6q24-q25.1	9113	LOC696131	similar to LATS homolog 1	---	696131	MmugDNA.17459.1.S1_at_LATS1
MmugDNA.17460.1.S1_at	202198_s_at	MTMR3	myotubularin related protein 3	chr22q12.2	8897	LOC715214	similar to myotubularin-related protein 3 isoform c	---	715214	MmugDNA.17460.1.S1_at_MTMR3
MmugDNA.17461.1.S1_at	211617_at	ALDOAP2	aldolase A, fructose-bisphosphate pseudogene 2	chr10q26.13	228	---	---	---	---	MmugDNA.17461.1.S1_at_ALDOAP2
MmugDNA.1746.1.S1_at	1559103_s_at	---	---	---	---	---	---	---	---	MmugDNA.1746.1.S1_at_---
MmugDNA.17462.1.S1_at	227005_at	RPP14	ribonuclease P/MRP 14kDa subunit	chr3p14.3	11102	---	---	---	---	MmugDNA.17462.1.S1_at_RPP14
MmugDNA.17463.1.S1_at	223423_at	GPR160	G protein-coupled receptor 160	chr3q26.2-q27	26996	LOC696917	hypothetical protein LOC696917	---	696917	MmugDNA.17463.1.S1_at_GPR160
MmugDNA.17464.1.S1_at	240846_at	PCBD2	pterin-4 alpha-carbinolamine dehydratase/dimerization cofactor of hepatocyte nuclear factor 1 alpha (	chr5q31.1	84105	LOC713854	similar to Pterin-4-alpha-carbinolamine dehydratase 2 (PHS 2) (4-alpha-hydroxy-tetrahydropterin deh	---	713854	MmugDNA.17464.1.S1_at_PCBD2
MmugDNA.17465.1.S1_s_at	208615_s_at	PTP4A2	protein tyrosine phosphatase type IVA, member 2	chr1p35	8073	LOC706489	similar to Protein tyrosine phosphatase type IVA protein 2 (Protein-tyrosine phosphatase 4a2) (Protei	---	706489	MmugDNA.17465.1.S1_s_at_PTP4A2
MmugDNA.17466.1.S1_at	243556_at	NGEF	neuronal guanine nucleotide exchange factor	chr2q37	25791	LOC713917	similar to neuronal guanine nucleotide exchange factor	---	713917	MmugDNA.17466.1.S1_at_NGEF
MmugDNA.17467.1.S1_at	230697_at	BBS5	Bardet-Biedl syndrome 5	chr2q31.1	129880	LOC709701	similar to Bardet-Biedl syndrome 5	---	709701	MmugDNA.17467.1.S1_at_BBS5
MmugDNA.17468.1.S1_at	239443_at	PCDHB6	protocadherin beta 6	chr5q31	56130	---	---	---	---	MmugDNA.17468.1.S1_at_PCDHB6
MmugDNA.17469.1.S1_at	NA	NA	NA	NA	NA	NA	NA	NA	NA	MmugDNA.17469.1.S1_at_NA
MmugDNA.17470.1.S1_at	218316_at	TIMM9	translocase of inner mitochondrial membrane 9 homolog (yeast)	chr14q21	26520	TIMM9	translocase of inner mitochondrial membrane 9 homolog (yeast)	---	701005	MmugDNA.17470.1.S1_at_TIMM9
MmugDNA.17471.1.S1_at	235343_at	VASH2	vasohibin 2	chr1q32.3	79805	LOC709924	similar to Vasohibin-like protein	---	709924	MmugDNA.17471.1.S1_at_VASH2
MmugDNA.1747.1.S1_at	1563913_at	---	---	---	---	---	---	---	---	MmugDNA.1747.1.S1_at_---
MmugDNA.17472.1.S1_at	237498_at	---	---	---	---	LOC716094	Similar to adaptor-related protein complex 2, beta 1 subunit	---	716094	MmugDNA.17472.1.S1_at_---
MmugDNA.17473.1.S1_x_at	NA	NA	NA	NA	NA	NA	NA	NA	NA	MmugDNA.17473.1.S1_x_at_NA
MmugDNA.17474.1.S1_at	205704_s_at	ATP6V0A2	ATPase, H+ transporting, lysosomal V0 subunit a2	chr12q24.31	23545	AFP	Alpha-fetoprotein	---	704786	MmugDNA.17474.1.S1_at_ATP6V0A2
MmugDNA.17475.1.S1_s_at	210606_x_at	KLRD1	killer cell lectin-like receptor subfamily D, member 1	chr12p13	3824	LOC574145	CD94	---	574145	MmugDNA.17475.1.S1_s_at_KLRD1
MmugDNA.17476.1.S1_at	226347_at	---	---	---	---	---	---	---	---	MmugDNA.17476.1.S1_at_---
MmugDNA.17477.1.S1_at	216330_s_at	POU6F1	POU class 6 homeobox 1	chr12q13.13	5463	LOC694640	similar to POU domain, class 6, transcription factor 1 (Brain-specific homeobox/POU domain protein !	---	694640	MmugDNA.17477.1.S1_at_POU6F1
MmugDNA.17478.1.S1_at	242919_at	ZNF253	zinc finger protein 253	chr19p13.11	56242	ZNF85	zinc finger protein 85	---	720418	MmugDNA.17478.1.S1_at_ZNF253
MmugDNA.17479.1.S1_at	226348_at	---	---	---	---	---	---	---	---	MmugDNA.17479.1.S1_at_---
MmugDNA.17480.1.S1_at	1560034_a_at	---	---	---	---	---	---	---	---	MmugDNA.17480.1.S1_at_---
MmugDNA.17481.1.S1_at	231123_at	TRIM36	tripartite motif-containing 36	chr5q22.3	55521	---	---	---	---	MmugDNA.17481.1.S1_at_TRIM36
MmugDNA.1748.1.S1_at	223784_at	TMEM27	transmembrane protein 27	chrXp22	57393	LOC712928	similar to transmembrane protein 27	---	712928	MmugDNA.1748.1.S1_at_TMEM27
MmugDNA.17483.1.S1_at	226953_at	DARC	Duffy blood group, chemokine receptor	chr1q21-q22	2532	LOC719284 /// LOC7199	similar to Duffy blood group /// similar to immunoglobulin superfamily, member 4B	---	719284 /// 719985	MmugDNA.17483.1.S1_at_DARC
MmugDNA.17485.1.S1_at	1552755_at	C9orf66	chromosome 9 open reading frame 66	chr9p24.3	157983	---	---	---	---	MmugDNA.17485.1.S1_at_C9orf66
MmugDNA.17486.1.S1_at	1558571_at	ZMYND8	Zinc finger, MYND-type containing 8	chr20q13.12	23613	---	---	---	---	MmugDNA.17486.1.S1_at_ZMYND8
MmugDNA.17487.1.S1_at	1552757_s_at	C9orf66	chromosome 9 open reading frame 66	chr9p24.3	157983	---	---	---	---	MmugDNA.17487.1.S1_at_C9orf66
MmugDNA.17488.1.S1_at	239408_at	---	---	---	---	---	---	---	---	MmugDNA.17488.1.S1_at_---
MmugDNA.17490.1.S1_at	1560495_at	---	---	---	---	---	---	---	---	MmugDNA.17490.1.S1_at_---
MmugDNA.17491.1.S1_at	1563691_at	LOC285300	hypothetical protein LOC285300	chr3p14.1	285300	---	---	---	---	MmugDNA.17491.1.S1_at_LOC285300
MmugDNA.1749.1.S1_at	1566852_at	TRIM42	tripartite motif-containing 42	chr3q23	287015	---	---	---	---	MmugDNA.1749.1.S1_at_TRIM42
MmugDNA.17492.1.S1_at	210431_at	ALPPL2	alkaline phosphatase, placental-like 2	chr2q37	251	---	---	---	---	MmugDNA.17492.1.S1_at_ALPPL2
MmugDNA.17493.1.S1_at	215376_at	---	---	---	---	---	---	---	---	MmugDNA.17493.1.S1_at_---
MmugDNA.17494.1.S1_at	226430_at	RELL1	RELT-like 1	chr4p14	768211	---	---	---	---	MmugDNA.17494.1.S1_at_RELL1
MmugDNA.17495.1.S1_at	233907_s_at	SERTAD4	SERTA domain containing 4	chr1q32.1-q41	56256	LOC712616	similar to SERTA domain containing 4	---	712616	MmugDNA.17495.1.S1_at_SERTAD4
MmugDNA.17496.1.S1_at	218833_at	ZAK	sterile alpha motif and leucine zipper containing kinase AZK	chr2q24.2	51776	ZAK	MLK-related kinase	---	698274	MmugDNA.17496.1.S1_at_ZAK
MmugDNA.17497.1.S1_at	220314_at	HSFX1 /// HSFX2	heat shock transcription factor family, X linked 1 /// heat shock transcription factor family, X linked 2	chrXq28	100130086 /// 51402	TMEM185A	similar to ee3_2	---	705538	MmugDNA.17497.1.S1_at_HSFX1 /// HS
MmugDNA.17498.1.S1_at	208027_s_at	TLL2	tolloid-like 2	chr10q23-q24	7093	---	---	---	---	MmugDNA.17498.1.S1_at_TLL2
MmugDNA.17499.1.S1_at	220062_s_at	MAGEC2	melanoma antigen family C, 2	chrXq27	51438	MAGEC2	melanoma antigen family C, 2	---	697762	MmugDNA.17499.1.S1_at_MAGEC2
MmugDNA.17501.1.S1_at	223910_at	ERN2	endoplasmic reticulum to nucleus signaling 2	chr16p12.2	10595	LOC698026	similar to Serine/threonine-protein kinase/endoribonuclease IRE2 precursor (Inositol-requiring protein	---	698026	MmugDNA.17501.1.S1_at_ERN2
MmugDNA.1750.1.S1_at	1556491_at	---	---	---	---	---	---	---	---	MmugDNA.1750.1.S1_at_---
MmugDNA.17502.1.S1_at	229511_at	SMARCE1	SWI/SNF related, matrix associated, actin dependent regulator of chromatin, subfamily e, member 1	chr17q21.2	6605	---	---	---	---	MmugDNA.17502.1.S1_at_SMARCE1
MmugDNA.17503.1.S1_at	218604_at	LEMD3	LEM domain containing 3	chr12q14	23592	---	---	---	---	MmugDNA.17503.1.S1_at_LEMD3
MmugDNA.17504.1.S1_at	229605_at	CYB5RL	cytochrome b5 reductase-like	chr1p32.3	606495	LOC715211	similar to cytochrome b5 reductase 1 (B5R.1)	---	715211	MmugDNA.17504.1.S1_at_CYB5RL
MmugDNA.17505.1.S1_at	220339_s_at	TPSG1	tryptase gamma 1	chr16p13.3	25823	---	---	---	---	MmugDNA.17505.1.S1_at_TPSG1
MmugDNA.17506.1.S1_at	217906_at	KLHDC2	kelch domain containing 2	chr14q22.1	23588	KLHDC2	kelch domain containing 2	---	704580	MmugDNA.17506.1.S1_at_KLHDC2
MmugDNA.17507.1.S1_at	215314_at	---	---	---	---	---	---	---	---	MmugDNA.17507.1.S1_at_---

MmugDNA.17508.1.S1_at	210867_at	CNOT4	CCR4-NOT transcription complex, subunit 4	chr7q22-qter	4850	CNOT4	CCR4-NOT transcription complex, subunit 4	---	708011	MmugDNA.17508.1.S1_at_CNOT4
MmugDNA.17509.1.S1_at	215178_x_at	NAAA	N-acylethanolamine acid amidase	chr4q21.1	27163	---	---	---	---	MmugDNA.17509.1.S1_at_NAAA
MmugDNA.17510.1.S1_at	229045_at	SNX20	sorting nexin 20	chr16q12.1	124460	SNX20	sorting nexin 20	---	695418	MmugDNA.17510.1.S1_at_SNX20
MmugDNA.17511.1.S1_at	215263_at	ZXDA /// ZXDB	zinc finger, X-linked, duplicated A /// zinc finger, X-linked, duplicated B	chrXp11.1 /// chrXp11.21	158586 /// 7789	ZXDB	zinc finger, X-linked, duplicated B	---	706216	MmugDNA.17511.1.S1_at_ZXDA /// ZXI
MmugDNA.1751.1.S1_at	211272_s_at	DGKA	diacylglycerol kinase, alpha 80kDa	chr12q13.3	1606	LOC710559	similar to diacylglycerol kinase, alpha 80kDa	---	710559	MmugDNA.1751.1.S1_at_DGKA
MmugDNA.17512.1.S1_at	218850_s_at	LIMD1	LIM domains containing 1	chr3p21.3	8994	---	---	---	---	MmugDNA.17512.1.S1_at_LIMD1
MmugDNA.17513.1.S1_at	227317_at	LMCD1	LIM and cysteine-rich domains 1	chr3p26-p24	29995	LOC707235	Similar to CG6954-PA	---	707235	MmugDNA.17513.1.S1_at_LMCD1
MmugDNA.17514.1.S1_at	1565669_at	---	---	---	---	---	---	---	---	MmugDNA.17514.1.S1_at_---
MmugDNA.17515.1.S1_at	223258_s_at	G2E3	G2/M-phase specific E3 ubiquitin ligase	chr14q12	55632	LOC701130	Similar to Protein C10orf57 homolog	---	701130	MmugDNA.17515.1.S1_at_G2E3
MmugDNA.17516.1.S1_at	216639_at	SRPX2	sushi-repeat-containing protein, X-linked 2	chrXq21.33-q23	27286	---	---	---	---	MmugDNA.17516.1.S1_at_SRPX2
MmugDNA.17517.1.S1_at	239444_at	CXorf56	chromosome X open reading frame 56	chrXq23	63932	LOC698255	similar to CG16865-PA	---	698255	MmugDNA.17517.1.S1_at_CXorf56
MmugDNA.17518.1.S1_at	217804_s_at	ILF3	interleukin enhancer binding factor 3, 90kDa	chr19p13.2	3609	LOC713427	similar to interleukin enhancer binding factor 3 isoform a	---	713427	MmugDNA.17518.1.S1_at_ILF3
MmugDNA.17519.1.S1_at	214210_at	SLC25A17	solute carrier family 25 (mitochondrial carrier; peroxisomal membrane protein, 34kDa), member 17	chr22q13.2	10478	LOC703517	similar to Peroxisomal membrane protein PMP34 (34 kDa peroxisomal membrane protein) (Solute ca	---	703517	MmugDNA.17519.1.S1_at_SLC25A17
MmugDNA.175.1.S1_at	242865_at	---	---	---	---	---	---	---	---	MmugDNA.175.1.S1_at_---
MmugDNA.17520.1.S1_at	229048_at	---	---	---	---	---	---	---	---	MmugDNA.17520.1.S1_at_---
MmugDNA.17521.1.S1_at	233689_at	---	---	---	---	---	---	---	---	MmugDNA.17521.1.S1_at_---
MmugDNA.1752.1.S1_s_at	221236_s_at	STMN4	stathmin-like 4	chr8p21.2	81551	STMN4	stathmin-like 4	---	712651	MmugDNA.1752.1.S1_s_at_STMN4
MmugDNA.17522.1.S1_at	236095_at	NTRK2	neurotrophic tyrosine kinase, receptor, type 2	chr9q22.1	4915	LOC718482	Hypothetical protein LOC718482	---	718482	MmugDNA.17522.1.S1_at_NTRK2
MmugDNA.17523.1.S1_at	1552535_at	CLDN19	claudin 19	chr1p34.2	149461	CLDN19	claudin 19	---	697807	MmugDNA.17523.1.S1_at_CLDN19
MmugDNA.17524.1.S1_at	232243_at	MCPH1	microcephalin 1	chr8p23.1	79648	---	---	---	---	MmugDNA.17524.1.S1_at_MCPH1
MmugDNA.17525.1.S1_s_at	202593_s_at	GDE1	glycerophosphodiester phosphodiesterase 1	chr16p12-p11.2	51573	GDE1	glycerophosphodiester phosphodiesterase 1	---	694849	MmugDNA.17525.1.S1_s_at_GDE1
MmugDNA.17526.1.S1_at	206234_s_at	MMP17	matrix metallopeptidase 17 (membrane-inserted)	chr12q24.3	4326	---	---	---	---	MmugDNA.17526.1.S1_at_MMP17
MmugDNA.17527.1.S1_at	225024_at	RPRD1B	regulation of nuclear pre-mRNA domain containing 1B	chr20q11.21-q12	58490	RPRD1B	regulation of nuclear pre-mRNA domain containing 1B	---	700056	MmugDNA.17527.1.S1_at_RPRD1B
MmugDNA.17528.1.S1_at	215837_x_at	---	---	---	---	---	---	---	---	MmugDNA.17528.1.S1_at_---
MmugDNA.17529.1.S1_at	1561961_at	DKFZp686A1627	PHD finger protein 2 pseudogene-like	chr13q11	266695	---	---	---	---	MmugDNA.17529.1.S1_at_DKFZp686A
MmugDNA.17530.1.S1_at	224724_at	SULF2	sulfatase 2	chr20q12-q13.2	55959	LOC715937	similar to sulfatase 2 isoform a precursor	---	715937	MmugDNA.17530.1.S1_at_SULF2
MmugDNA.17531.1.S1_at	203534_at	LSM1	LSM1 homolog, U6 small nuclear RNA associated (S. cerevisiae)	chr8p11.2	27257	LSM1	LSM1 homolog, U6 small nuclear RNA associated (S. cerevisiae)	---	699754	MmugDNA.17531.1.S1_at_LSM1
MmugDNA.1753.1.S1_at	1555437_at	EARS2	glutamyl-tRNA synthetase 2, mitochondrial (putative)	chr16p12.1	124454	EARS2	glutamyl-tRNA synthetase 2, mitochondrial (putative)	---	700487	MmugDNA.1753.1.S1_at_EARS2
MmugDNA.17532.1.S1_at	1562608_at	---	---	---	---	---	---	---	---	MmugDNA.17532.1.S1_at_---
MmugDNA.17533.1.S1_at	202209_at	LSM3	LSM3 homolog, U6 small nuclear RNA associated (S. cerevisiae)	chr3p25.1	27258	LSM3	LSM3 homolog, U6 small nuclear RNA associated (S. cerevisiae)	---	701060	MmugDNA.17533.1.S1_at_LSM3
MmugDNA.17534.1.S1_at	201454_s_at	NPEPPS	aminopeptidase puromycin sensitive	chr17q21	9520	LOC697915	Similar to synaptotagmin-like 5	---	697915	MmugDNA.17534.1.S1_at_NPEPPS
MmugDNA.17534.1.S1_s_at	NA	NA	NA	NA	NA	NA	NA	NA	NA	MmugDNA.17534.1.S1_s_at_NA
MmugDNA.17535.1.S1_at	201455_s_at	NPEPPS	aminopeptidase puromycin sensitive	chr17q21	9520	---	---	---	---	MmugDNA.17535.1.S1_at_NPEPPS
MmugDNA.17536.1.S1_at	231891_at	STAMBPL1	STAM binding protein-like 1	chr10q23.31	57559	LOC694629	similar to associated molecule with the SH3 domain of STAM (AMSH) like protein	---	694629	MmugDNA.17536.1.S1_at_STAMBPL1
MmugDNA.17537.1.S1_at	215960_at	SLC5A4	solute carrier family 5 (low affinity glucose cotransporter), member 4	chr22q12.2-q12.3	6527	LOC717137	similar to solute carrier family 5 (low affinity glucose cotransporter), member 4	---	717137	MmugDNA.17537.1.S1_at_SLC5A4
MmugDNA.17538.1.S1_at	212700_x_at	PLEKHM1	pleckstrin homology domain containing, family M (with RUN domain) member 1	chr17q21.31	9842	LOC716464	similar to pleckstrin homology domain containing, family M (with RUN domain) member 1	---	716464	MmugDNA.17538.1.S1_at_PLEKHM1
MmugDNA.17539.1.S1_at	212717_at	PLEKHM1	pleckstrin homology domain containing, family M (with RUN domain) member 1	chr17q21.31	9842	LOC716464	similar to pleckstrin homology domain containing, family M (with RUN domain) member 1	---	716464	MmugDNA.17539.1.S1_at_PLEKHM1
MmugDNA.17540.1.S1_at	202141_s_at	COPS8	COP9 constitutive photomorphogenic homolog subunit 8 (Arabidopsis)	chr2q37.3	10920	LOC702097	similar to COP9 signalosome complex subunit 8 (Signalosome subunit 8) (SGN8) (JAB1-containing s	---	702097	MmugDNA.17540.1.S1_at_COPS8
MmugDNA.17541.1.S1_at	240798_at	---	---	---	---	---	---	---	---	MmugDNA.17541.1.S1_at_---
MmugDNA.1754.1.S1_at	221237_s_at	OSBP2	oxysterol binding protein 2	chr22q12.2	23762	LOC716252	similar to oxysterol binding protein 2 isoform a	---	716252	MmugDNA.1754.1.S1_at_OSBP2
MmugDNA.17542.1.S1_at	221089_at	NOX3	NADPH oxidase 3	chr6q25.1-q26	50508	NOX3	NADPH oxidase 3	---	701576	MmugDNA.17542.1.S1_at_NOX3
MmugDNA.17543.1.S1_at	239720_at	GLP-1	GATA like protein-1	chr19p13.2	100125288	LOC712411	similar to GATA binding protein 2	---	712411	MmugDNA.17543.1.S1_at_GLP-1
MmugDNA.17544.1.S1_at	207410_s_at	TLX2	T-cell leukemia homeobox 2	chr2p13.1-p12	3196	TLX2	T-cell leukemia homeobox 2	---	709649	MmugDNA.17544.1.S1_at_TLX2
MmugDNA.17545.1.S1_at	238820_at	C14orf83	chromosome 14 open reading frame 83	chr14q24.1	161145	LOC710681	hypothetical protein LOC710681	---	710681	MmugDNA.17545.1.S1_at_C14orf83
MmugDNA.17546.1.S1_at	218367_x_at	USP21	ubiquitin specific peptidase 21	chr1q22	27005	USP21	ubiquitin specific peptidase 21	---	719894	MmugDNA.17546.1.S1_at_USP21
MmugDNA.17547.1.S1_at	219594_at	NINJ2	ninjurin 2	chr12p13	4815	NINJ2	ninjurin 2	---	706368	MmugDNA.17547.1.S1_at_NINJ2
MmugDNA.17548.1.S1_at	235404_at	ARID5B	AT rich interactive domain 5B (MRF1-like)	chr10q21.2	84159	---	---	---	---	MmugDNA.17548.1.S1_at_ARID5B
MmugDNA.17549.1.S1_s_at	202069_s_at	IDH3A	isocitrate dehydrogenase 3 (NAD+) alpha	chr15q25.1-q25.2	3419	LOC712230	Similar to myosin, heavy polypeptide 7B, cardiac muscle, beta	---	712230	MmugDNA.17549.1.S1_s_at_IDH3A
MmugDNA.17550.1.S1_at	208530_s_at	RARB	retinoic acid receptor, beta	chr3p24	5915	RARB	retinoic acid receptor, beta	---	700485	MmugDNA.17550.1.S1_at_RARB
MmugDNA.17551.1.S1_at	1556325_at	FILIP1	filamin A interacting protein 1	chr6q14.1	27145	LOC716439	similar to filamin A interacting protein 1	---	716439	MmugDNA.17551.1.S1_at_FILIP1
MmugDNA.1755.1.S1_at	244099_at	---	---	---	---	LOC700803	LOC57821	---	700803	MmugDNA.1755.1.S1_at_---
MmugDNA.17552.1.S1_at	213824_at	OLIG2	oligodendrocyte lineage transcription factor 2	chr21q22.11	10215	LOC700098	similar to oligodendrocyte lineage transcription factor 2	---	700098	MmugDNA.17552.1.S1_at_OLIG2
MmugDNA.17553.1.S1_at	204575_s_at	MMP19	matrix metallopeptidase 19	chr12q14	4327	MMP19	matrix metallopeptidase 19	---	710111	MmugDNA.17553.1.S1_at_MMP19
MmugDNA.17554.1.S1_at	213825_at	OLIG2	oligodendrocyte lineage transcription factor 2	chr21q22.11	10215	LOC700098	similar to oligodendrocyte lineage transcription factor 2	---	700098	MmugDNA.17554.1.S1_at_OLIG2
MmugDNA.17555.1.S1_at	238731_at	SETDB2	SET domain, bifurcated 2	chr13q14	83852	SETDB2	SET domain, bifurcated 2	---	706277	MmugDNA.17555.1.S1_at_SETDB2
MmugDNA.17556.1.S1_at	218061_at	MEA1	male-enhanced antigen 1	chr6p21.3-p21.1	4201	LOC696610	Similar to ATPase family, AAA domain containing 1	---	696610	MmugDNA.17556.1.S1_at_MEA1
MmugDNA.17557.1.S1_at	230813_at	LEPREL1	leprecan-like 1	chr3q28	55214	LEPREL1	leprecan-like 1	---	704118	MmugDNA.17557.1.S1_at_LEPREL1
MmugDNA.17558.1.S1_at	218250_s_at	CNOT7	CCR4-NOT transcription complex, subunit 7	chr8p22-p21.3	29883	CNOT7	CCR4-NOT transcription complex, subunit 7	---	702192	MmugDNA.17558.1.S1_at_CNOT7
MmugDNA.17559.1.S1_at	210371_s_at	RBBP4	retinoblastoma binding protein 4	chr1p35.1	5928	LOC694736	similar to Histone-binding protein RBBP4 (Retinoblastoma-binding protein 4) (RBBP-4) (Retinoblastom	---	694736	MmugDNA.17559.1.S1_at_RBBP4
MmugDNA.17560.1.S1_at	211618_s_at	ALPI	alkaline phosphatase, intestinal	chr2q37.1	248	ALPI	intestinal alkaline phosphatase	---	721052	MmugDNA.17560.1.S1_at_ALPI
MmugDNA.17561.1.S1_at	244262_x_at	---	---	---	---	---	---	---	---	MmugDNA.17561.1.S1_at_---
MmugDNA.1756.1.S1_at	217687_at	ADCY2	adenylate cyclase 2 (brain)	chr5p15.3	108	---	---	---	---	MmugDNA.1756.1.S1_at_ADCY2
MmugDNA.17562.1.S1_at	NA	NA	NA	NA	NA	NA	NA	NA	NA	MmugDNA.17562.1.S1_at_NA
MmugDNA.17562.1.S1_s_at	204669_s_at	RNF24	ring finger protein 24	chr20p13-p12.1	11237	---	---	---	---	MmugDNA.17562.1.S1_s_at_RNF24
MmugDNA.17563.1.S1_at	1561491_at	LOC283214	hypothetical protein LOC283214	chr11q13.5	283214	---	---	---	---	MmugDNA.17563.1.S1_at_LOC283214
MmugDNA.17564.1.S1_at	211564_s_at	PDLIM4	PDZ and LIM domain 4	chr5q31.1	8572	PDLIM4	PDZ and LIM domain 4	---	707372	MmugDNA.17564.1.S1_at_PDLIM4
MmugDNA.17565.1.S1_at	228731_at	---	---	---	---	LOC709260	Similar to sno, strawberry notch homolog 1	---	709260	MmugDNA.17565.1.S1_at_---
MmugDNA.17566.1.S1_at	228732_at	---	---	---	---	---	---	---	---	MmugDNA.17566.1.S1_at_---
MmugDNA.17567.1.S1_at	222557_at	STMN3	stathmin-like 3	chr20q13.3	50861	LOC719538	similar to SCG10-like-protein	---	719538	MmugDNA.17567.1.S1_at_STMN3
MmugDNA.17568.1.S1_at	204484_at	PIK3C2B	phosphoinositide-3-kinase, class 2, beta polypeptide	chr1q32	5287	LOC701974	similar to phosphoinositide-3-kinase, class 2, beta polypeptide	---	701974	MmugDNA.17568.1.S1_at_PIK3C2B
MmugDNA.17569.1.S1_at	1559174_at	RAB6A	RAB6A, member RAS oncogene family	chr11q13.3	5870	---	---	---	---	MmugDNA.17569.1.S1_at_RAB6A
MmugDNA.17570.1.S1_at	230090_at	GDNF	glial cell derived neurotrophic factor	chr5p13.1-p12	2668	---	---	---	---	MmugDNA.17570.1.S1_at_GDNF
MmugDNA.17571.1.S1_at	220090_at	CRNN	cornulin	chr1q21	49860	LOC712894	similar to Repetin	---	712894	MmugDNA.17571.1.S1_at_CRNN
MmugDNA.1757.1.S1_at	214975_s_at	MTMR1	myotubularin related protein 1	chrXq28	8776	LOC708892	similar to myotubularin-related protein 1	---	708892	MmugDNA.1757.1.S1_at_MTMR1
MmugDNA.17572.1.S1_s_at	229475_at	MAEL	maelstrom homolog (Drosophila)	chr1q24.1	84944	MAEL	maelstrom homolog (Drosophila)	---	697185	MmugDNA.17572.1.S1_s_at_MAEL
MmugDNA.17573.1.S1_at	223051_at	SSU72	SSU72 RNA polymerase II CTD phosphatase homolog (S. cerevisiae)	chr1p36.33	29101	LOC702348	similar to Ssu72 RNA polymerase II CTD phosphatase homolog	---	702348	MmugDNA.17573.1.S1_at_SSU72
MmugDNA.17574.1.S1_at	221061_at	PKD2L1	polycystic kidney disease 2-like 1	chr10q24	9033	PKD2L1	polycystic kidney disease 2-like 1	---	710056	MmugDNA.17574.1.S1_at_PKD2L1
MmugDNA.17575.1.S1_at	226364_at	HIP1	Huntingtin interacting protein 1	chr7q11.23	3092	---	---	---	---	MmugDNA.17575.1.S1_at_HIP1
MmugDNA.17576.1.S1_at	230100_x_at	PAK1	p21 protein (Cdc42/Rac)-activated kinase 1	chr11q13-q14	5058	LOC698585	similar to p21-activated kinase 1	---	698585	MmugDNA.17576.1.S1_at_PAK1
MmugDNA.17577.1.S1_at	242471_at	---	---	---	---	---	---	---	---	MmugDNA.17577.1.S1_at_---
MmugDNA.17578.1.S1_at	243628_at	COPS7B	COP9 constitutive photomorphogenic homolog subunit 7B (Arabidopsis)	chr2q37.1	64708	LOC712697	similar to COP9 constitutive photomorphogenic homolog subunit 7B	---	712697	MmugDNA.17578.1.S1_at_COPS7B
MmugDNA.17579.1.S1_at	221051_s_at	ITGB1BP3	integrin beta 1 binding protein 3	chr19p13.3	27231	---	---	---	---	MmugDNA.17579.1.S1_at_ITGB1BP3
MmugDNA.17580.1.S1_at	1555804_a_at	YSK4	YSK4 Sps1/Ste20-related kinase homolog (S. cerevisiae)	chr2q21.3	80122	LOC712728	similar to Yeast Sps1/Ste20-related kinase 4 isoform 1	---	712728	MmugDNA.17580.1.S1_at_YSK4
MmugDNA.17581.1.S1_at	228551_at	DENND5B	DENN/MADD domain containing 5B	chr12p11.21	160518	---	---	---	---	MmugDNA.17581.1.S1_at_DENND5B
MmugDNA.1758.1.S1_at	NA	NA	NA	NA	NA	NA	NA	NA	NA	MmugDNA.1758.1.S1_at_NA
MmugDNA.17582.1.S1_at	1553163_at	ADPRHL1	ADP-ribosylhydrolase like 1	chr13q34	113622	LOC697842	similar to ADP-ribosylhydrolase like 1 isoform 1	---	697842	MmugDNA.17582.1.S1_at_ADPRHL1
MmugDNA.17583.1.S1_at	212544_at	ZNHIT3	zinc finger, HIT type 3	chr17q12	9326	LOC707869	similar to thyroid hormone receptor interactor 3 isoform 2	---	707869	MmugDNA.17583.1.S1_at_ZNHIT3
MmugDNA.17584.1.S1_s_at	NA	NA	NA	NA	NA	NA	NA	NA	NA	MmugDNA.17584.1.S1_s_at_NA
MmugDNA.17585.1.S1_at	204838_s_at	MLH3	mutL homolog 3 (E. coli)	chr14q24.3	27030	MLH3	mutL homolog 3	---	701482	MmugDNA.17585.1.S1_at_MLH3
MmugDNA.17586.1.S1_s_at	1554894_a_at	PCBD2	pterin-4 alpha-carbinolamine dehydratase/dimerization cofactor of hepatocyte nuclear factor 1 alpha (	chr5q31.1	84105	LOC713854	similar to Pterin-4-alpha-carbinolamine dehydratase 2 (PHS 2) (4-alpha-hydroxy-tetrahydropterin deh	---	713854	MmugDNA.17586.1.S1_s_at_PCBD2
MmugDNA.17587.1.S1_at	206141_at	MOCS3	molybdenum cofactor synthesis 3	chr20q13.13	27304	MOCS3	molybdenum cofactor synthesis 3	---	701166	MmugDNA.17587.1.S1_at_MOCS3
MmugDNA.17588.1.S1_at	211920_at	CFB	complement factor B	chr6p21.3	629	CFB	complement factor B	---	716809	MmugDNA.17588.1.S1_at_CFB
MmugDNA.17589.1.S1_at	230284_at	MYOM3	myomesin family, member 3	chr1p36.11	127294	LOC719256	similar to myomesin family, member 3	---	719256	MmugDNA.17589.1.S1_at_MYOM3
MmugDNA.17590.1.S1_at	226924_at	LOC400657	hypothetical LOC400657	chr18q22.3	400657	---	---	---	---	MmugDNA.17590.1.S1_at_LOC400657
MmugDNA.17591.1.S1_at	232547_at	SNIP	SNAP25-interacting protein	chr17q12	80725	SNIP	SNAP25-interacting protein	---	693662	MmugDNA.17591.1.S1_at_SNIP
MmugDNA.1759.1.S1_at	NA	NA	NA	NA	NA	NA	NA	NA	NA	MmugDNA.1759.1.S1_at_NA
MmugDNA.1759.1.S1_s_at	233743_x_at	S1PR5	sphingosine-1-phosphate receptor 5	chr19p13.2	53637	LOC712977	similar to endothelial differentiation, sphingolipid G-protein-coupled receptor, 8	---	712977	MmugDNA.1759.1.S1_s_at_S1PR5
MmugDNA.17592.1.S1_at	224411_at	PLA2G12B	phospholipase A2, group XIIB	chr10q22.1	84647	PLA2G12B	phospholipase A2, group XIIB	---	708031	MmugDNA.17592.1.S1_at_PLA2G12B
MmugDNA.17593.1.S1_at	1555705_a_at	CMTM3	CKLF-like MARVEL transmembrane domain containing 3	chr16q21-q22.1	123920	CMTM3	CKLF-like MARVEL transmembrane domain containing 3	---	695592	MmugDNA.17593.1.S1_at_CMTM3
MmugDNA.17593.1.S1_s_at	224733_at	CMTM3	CKLF-like MARVEL transmembrane domain containing 3	chr16q21-q22.1	123920	CMTM3	CKLF-like MARVEL transmembrane domain containing 3	---	695592	MmugDNA.17593.1.S1_s_at_CMTM3
MmugDNA.17594.1.S1_at	231003_at	SLC35B3	solute carrier family 35, member B3	chr6p24.3	51000	---	---	---	---	MmugDNA.17594.1.S1_at_SLC35B3
MmugDNA.17595.1.S1_at	236749_at	MNT	MAX binding protein	chr17p13.3	4335	MNT	MAX binding protein	---	703659	MmugDNA.17595.1.S1_at_MNT
MmugDNA.17596.1.S1_at	239769_at	CDH11	Cadherin 11, type 2, OB-cadherin (osteoblast)	chr16q22.1	1009	---	---	---	---	MmugDNA.17596.1.S1_at_CDH11
MmugDNA.17597.1.S1_at	215780_s_at	hCG_1644608 /// SET	SET translocation (myeloid leukemia-associated) pseudogene /// SET nuclear oncogene	chr9q34 /// chrXq21.1	6418 /// 642869	LOC717352 /// SET /// SI	similar to Protein SET (Phosphatase 2A inhibitor I2PP2A) (I-2PP2A) (Template-activating factor I) (T/	---	693331 /// 705771 /// 713(	MmugDNA.17597.1.S1_at_hCG_16446
MmugDNA.17598.1.S1_at	243237_at	---	---	---	---	---	---	---	---	MmugDNA.17598.1.S1_at_---
MmugDNA.17599.1.S1_at	228228_at	DACT3	dapper, antagonist of beta-catenin, homolog 3 (Xenopus laevis)	chr19q13.32	147906	LOC717565	hypothetical protein LOC717565	---	717565	MmugDNA.17599.1.S1_at_DACT3
MmugDNA.17600.1.S1_at	237322_at	MIAT	myocardial infarction associated transcript (non-protein coding)	chr22q12.1	440823	---	---	---	---	MmugDNA.17600.1.S1_at_MIAT
MmugDNA.17601.1.S1_at	218644_at	PLEK2	pleckstrin 2	chr14q23.3	26499	PLEK2	pleckstrin 2	---	710419	MmugDNA.17601.1.S1_at_PLEK2

MmugDNA.1760.1.S1_s_at	214260_at	COPS8	COP9 constitutive photomorphogenic homolog subunit 8 (Arabidopsis)	chr2q37.3	10920	LOC702097	similar to COP9 signalosome complex subunit 8 (Signalosome subunit 8) (SGN8) (JAB1-containing s	---	702097	MmugDNA.1760.1.S1_s_at_COPS8
MmugDNA.17602.1.S1_at	215130_s_at	IQCK	IQ motif containing K	chr16p12.3	124152	LOC695417	hypothetical protein LOC695417	---	695417	MmugDNA.17602.1.S1_at_IQCK
MmugDNA.17603.1.S1_at	49077_at	PPME1	protein phosphatase methylesterase 1	chr11q13.4	51400	---	---	---	---	MmugDNA.17603.1.S1_at_PPME1
MmugDNA.17604.1.S1_at	211009_s_at	ZNF271	zinc finger protein 271	chr18q12	10778	ZNF397	zinc finger protein 397	---	714878	MmugDNA.17604.1.S1_at_ZNF271
MmugDNA.17605.1.S1_at	230881_at	CCDC42	coiled-coil domain containing 42	chr17p13.1	146849	LOC717532	similar to F59A2.6	---	717532	MmugDNA.17605.1.S1_at_CCDC42
MmugDNA.17606.1.S1_at	228570_at	BTBD11	BTB (POZ) domain containing 11	chr12q23.3	121551	BTBD11	BTB (POZ) domain containing 11	---	704916	MmugDNA.17606.1.S1_at_BTBD11
MmugDNA.17607.1.S1_at	217936_at	ARHGAP5	Rho GTPase activating protein 5	chr14q12	394	---	---	---	---	MmugDNA.17607.1.S1_at_ARHGAP5
MmugDNA.17608.1.S1_at	225605_at	TP53I13	tumor protein p53 inducible protein 13	chr17q11.2	90313	LOC716918	similar to CG12093-PA	---	716918	MmugDNA.17608.1.S1_at_TP53I13
MmugDNA.17609.1.S1_at	239653_at	---	---	---	---	---	---	---	---	MmugDNA.17609.1.S1_at_---
MmugDNA.17610.1.S1_at	1562925_at	---	---	---	---	---	---	---	---	MmugDNA.17610.1.S1_at_---
MmugDNA.17611.1.S1_at	205592_at	SLC4A1	solute carrier family 4, anion exchanger, member 1 (erythrocyte membrane protein band 3, Diego blo	chr17q21-q22	6521	---	---	---	---	MmugDNA.17611.1.S1_at_SLC4A1
MmugDNA.1761.1.S1_at	218319_at	PELI1	pellino homolog 1 (Drosophila)	chr2p13.3	57162	LOC696416	similar to pellino protein	---	696416	MmugDNA.1761.1.S1_at_PELI1
MmugDNA.17612.1.S1_at	220850_at	MORC1	MORC family CW-type zinc finger 1	chr3q13	27136	---	---	---	---	MmugDNA.17612.1.S1_at_MORC1
MmugDNA.17613.1.S1_at	244492_at	---	---	---	---	---	---	---	---	MmugDNA.17613.1.S1_at_---
MmugDNA.17614.1.S1_at	240715_at	TBX5	T-box 5	chr12q24.1	6910	TBX5	T-box 5	---	713550	MmugDNA.17614.1.S1_at_TBX5
MmugDNA.17615.1.S1_at	233691_at	---	---	---	---	---	---	---	---	MmugDNA.17615.1.S1_at_---
MmugDNA.17616.1.S1_at	234404_at	---	---	---	---	---	---	---	---	MmugDNA.17616.1.S1_at_---
MmugDNA.17617.1.S1_s_at	211700_s_at	TRO	trophinin	chrXp11.22-p11.21	7216	TRO	trophinin	---	704512	MmugDNA.17617.1.S1_s_at_TRO
MmugDNA.17618.1.S1_at	241335_at	---	---	---	---	---	---	---	---	MmugDNA.17618.1.S1_at_---
MmugDNA.17619.1.S1_at	217772_s_at	MTCH2	mitochondrial carrier homolog 2 (C. elegans)	chr11p11.2	23788	---	---	---	---	MmugDNA.17619.1.S1_at_MTCH2
MmugDNA.176.1.S1_at	207989_at	OPRM1	opioid receptor, mu 1	chr6q24-q25	4988	---	---	---	---	MmugDNA.176.1.S1_at_OPRM1
MmugDNA.17620.1.S1_at	211701_s_at	TRO	trophinin	chrXp11.22-p11.21	7216	TRO	trophinin	---	704512	MmugDNA.17620.1.S1_at_TRO
MmugDNA.17621.1.S1_at	210991_s_at	RIMS3	regulating synaptic membrane exocytosis 3	chr1pter-p22.2	9783	RIMS3	regulating synaptic membrane exocytosis 3	---	694926	MmugDNA.17621.1.S1_at_RIMS3
MmugDNA.1762.1.S1_at	1570136_at	---	---	---	---	---	---	---	---	MmugDNA.1762.1.S1_at_---
MmugDNA.17622.1.S1_at	214753_at	N4BP2L2	NEDD4 binding protein 2-like 2	chr13q13.1	10443	---	---	---	---	MmugDNA.17622.1.S1_at_N4BP2L2
MmugDNA.17623.1.S1_at	1555345_at	SLC38A4	solute carrier family 38, member 4	chr12q13	55089	SLC38A4	solute carrier family 38, member 4	---	702354	MmugDNA.17623.1.S1_at_SLC38A4
MmugDNA.17624.1.S1_at	209695_at	PTP4A3	protein tyrosine phosphatase type IVA, member 3	chr8q24.3	11156	LOC693703	similar to protein tyrosine phosphatase type IVA, member 3 isoform 2	---	693703	MmugDNA.17624.1.S1_at_PTP4A3
MmugDNA.17625.1.S1_at	203702_s_at	TTLL4	tubulin tyrosine ligase-like family, member 4	chr2p24.3-p24.1	9654	TTLL4	tubulin tyrosine ligase-like family, member 4	---	700064	MmugDNA.17625.1.S1_at_TTLL4
MmugDNA.17626.1.S1_at	207331_at	---	---	---	---	---	---	---	---	MmugDNA.17626.1.S1_at_---
MmugDNA.17627.1.S1_at	243873_at	---	---	---	---	---	---	---	---	MmugDNA.17627.1.S1_at_---
MmugDNA.17628.1.S1_at	207779_at	---	---	---	---	---	---	---	---	MmugDNA.17628.1.S1_at_---
MmugDNA.17629.1.S1_at	218536_at	MRS2	MRS2 magnesium homeostasis factor homolog (S. cerevisiae)	chr6p22.3-p22.1	57380	---	---	---	---	MmugDNA.17629.1.S1_at_MRS2
MmugDNA.17630.1.S1_at	203377_s_at	CDC40	cell division cycle 40 homolog (S. cerevisiae)	chr6q21	51362	---	---	---	---	MmugDNA.17630.1.S1_at_CDC40
MmugDNA.17631.1.S1_at	244016_at	---	---	---	---	---	---	---	---	MmugDNA.17631.1.S1_at_---
MmugDNA.1763.1.S1_at	206090_s_at	DISC1	disrupted in schizophrenia 1	chr1q42.1	27185	LOC722194	Similar to ankyrin repeat domain 26	---	722194	MmugDNA.1763.1.S1_at_DISC1
MmugDNA.17632.1.S1_at	214766_s_at	AHCTF1	AT hook containing transcription factor 1	chr1q44	25909	AHCTF1	AT hook containing transcription factor 1	---	702705	MmugDNA.17632.1.S1_at_AHCTF1
MmugDNA.17633.1.S1_at	1563203_at	---	---	---	---	---	---	---	---	MmugDNA.17633.1.S1_at_---
MmugDNA.17634.1.S1_at	229119_s_at	ZSWIM7	zinc finger, SWIM-type containing 7	chr17p12	125150	---	---	---	---	MmugDNA.17634.1.S1_at_ZSWIM7
MmugDNA.17635.1.S1_at	243408_at	---	---	---	---	---	---	---	---	MmugDNA.17635.1.S1_at_---
MmugDNA.17636.1.S1_at	219098_at	MYBBP1A	MYB binding protein (P160) 1a	chr17p13.3	10514	MYBBP1A	MYB binding protein (P160) 1a	---	708817	MmugDNA.17636.1.S1_at_MYBBP1A
MmugDNA.17637.1.S1_at	NA	NA	NA	NA	NA	NA	NA	NA	NA	MmugDNA.17637.1.S1_at_NA
MmugDNA.17637.1.S1_s_at	229429_x_at	LOC728855	hypothetical LOC728855	chr1q21.1	728855	---	---	---	---	MmugDNA.17637.1.S1_s_at_LOC72885
MmugDNA.17638.1.S1_at	225095_at	SPTLC2	Serine palmitoyltransferase, long chain base subunit 2	chr14q24.3-q31	9517	---	---	---	---	MmugDNA.17638.1.S1_at_SPTLC2
MmugDNA.17639.1.S1_at	1567248_at	OR9A1P	olfactory receptor, family 9, subfamily A, member 1 pseudogene	chr7q34	26495	LOC695212	similar to olfactory receptor 461	---	695212	MmugDNA.17639.1.S1_at_OR9A1P
MmugDNA.17640.1.S1_at	218064_s_at	AKAP8L	A kinase (PRKA) anchor protein 8-like	chr19p13.12	26993	AKAP8L	A kinase (PRKA) anchor protein 8-like	---	719202	MmugDNA.17640.1.S1_at_AKAP8L
MmugDNA.17641.1.S1_at	211619_s_at	ALPP /// ALPPL2	alkaline phosphatase, placental (Regan isozyme) /// alkaline phosphatase, placental-like 2	chr2q37	250 /// 251	---	---	---	---	MmugDNA.17641.1.S1_at_ALPP /// ALF
MmugDNA.1764.1.S1_at	238459_x_at	SPATA6	spermatogenesis associated 6	chr1p33	54558	---	---	---	---	MmugDNA.1764.1.S1_at_SPATA6
MmugDNA.17642.1.S1_at	214495_at	CACNG2	calcium channel, voltage-dependent, gamma subunit 2	chr22q13.1	10369	CACNG2	similar to voltage-dependent calcium channel gamma-2 subunit	---	696294	MmugDNA.17642.1.S1_at_CACNG2
MmugDNA.17643.1.S1_at	207452_s_at	CNTN5	contactin 5	chr11q21-q22.2	53942	CNTN5	contactin 5	---	700826	MmugDNA.17643.1.S1_at_CNTN5
MmugDNA.17644.1.S1_s_at	212063_at	CD44	CD44 molecule (Indian blood group)	chr11p13	960	---	---	---	---	MmugDNA.17644.1.S1_s_at_CD44
MmugDNA.17645.1.S1_at	1556667_at	LOC348751	hypothetical protein LOC348751	chr2q33.1	348751	---	---	---	---	MmugDNA.17645.1.S1_at_LOC348751
MmugDNA.17646.1.S1_at	244116_at	---	---	---	---	---	---	---	---	MmugDNA.17646.1.S1_at_---
MmugDNA.17647.1.S1_at	1562006_at	---	---	---	---	---	---	---	---	MmugDNA.17647.1.S1_at_---
MmugDNA.17648.1.S1_at	211096_at	PBX2	pre-B-cell leukemia homeobox 2	chr6p21.3	5089	LOC716239	similar to Pre-B-cell leukemia transcription factor 2 (Homeobox protein PBX2) (Protein G17)	---	716239	MmugDNA.17648.1.S1_at_PBX2
MmugDNA.17649.1.S1_at	216403_at	SP3P	Sp3 transcription factor pseudogene	chr13q31.3	160824	SP3	Sp3 transcription factor	---	699901	MmugDNA.17649.1.S1_at_SP3P
MmugDNA.17650.1.S1_at	217150_s_at	NF2	neurofibromin 2 (merlin)	chr22q12.2	4771	LOC714945	similar to neurofibromin 2 isoform 2	---	714945	MmugDNA.17650.1.S1_at_NF2
MmugDNA.17651.1.S1_at	215961_at	F12	coagulation factor XII (Hageman factor)	chr5q33-qter	2161	F12	coagulation factor XII	---	699875	MmugDNA.17651.1.S1_at_F12
MmugDNA.1765.1.S1_at	1556322_a_at	---	---	---	---	---	---	---	---	MmugDNA.1765.1.S1_at_---
MmugDNA.17652.1.S1_at	233997_at	---	---	---	---	---	---	---	---	MmugDNA.17652.1.S1_at_---
MmugDNA.17652.1.S1_x_at	NA	NA	NA	NA	NA	NA	NA	NA	NA	MmugDNA.17652.1.S1_x_at_NA
MmugDNA.17653.1.S1_at	240156_at	---	---	---	---	---	---	---	---	MmugDNA.17653.1.S1_at_---
MmugDNA.17654.1.S1_at	218439_s_at	COMMD10	COMM domain containing 10	chr5q23.1	51397	---	---	---	---	MmugDNA.17654.1.S1_at_COMMD10
MmugDNA.17655.1.S1_at	217780_at	C19orf56	chromosome 19 open reading frame 56	chr19p13.13	51398	LOC716302	similar to UPF0139 protein CGI-140	---	716302	MmugDNA.17655.1.S1_at_C19orf56
MmugDNA.17656.1.S1_at	236046_at	FLJ44896	FLJ44896 protein	chr5p15.33	401166	---	---	---	---	MmugDNA.17656.1.S1_at_FLJ44896
MmugDNA.17657.1.S1_at	217958_at	TRAPPC4	trafficking protein particle complex 4	chr11q23.3	51399	TRAPPC4	trafficking protein particle complex 4	---	702469	MmugDNA.17657.1.S1_at_TRAPPC4
MmugDNA.17658.1.S1_at	219598_s_at	RWDD1	RWD domain containing 1	chr6q13-q22.33	51389	RWDD1	RWD domain containing 1	---	715760	MmugDNA.17658.1.S1_at_RWDD1
MmugDNA.17659.1.S1_at	212041_at	ATP6V0D1	ATPase, H+ transporting, lysosomal 38kDa, V0 subunit d1	chr16q22	9114	---	---	---	---	MmugDNA.17659.1.S1_at_ATP6V0D1
MmugDNA.17660.1.S1_at	204160_s_at	ENPP4	ectonucleotide pyrophosphatase/phosphodiesterase 4 (putative function)	chr6p12.3	22875	LOC699511	Similar to CTAGE family, member 5 isoform 1	---	699511	MmugDNA.17660.1.S1_at_ENPP4
MmugDNA.17660.1.S1_s_at	NA	NA	NA	NA	NA	NA	NA	NA	NA	MmugDNA.17660.1.S1_s_at_NA
MmugDNA.17661.1.S1_at	226156_at	AKT2	v-akt murine thymoma viral oncogene homolog 2	chr19q13.1-q13.2	208	---	---	---	---	MmugDNA.17661.1.S1_at_AKT2
MmugDNA.1766.1.S1_at	243021_at	LOC730496	hypothetical protein LOC730496	---	730496	LOC707173	hypothetical protein LOC707173	---	707173	MmugDNA.1766.1.S1_at_LOC730496
MmugDNA.17662.1.S1_s_at	212211_at	ANKRD17	ankyrin repeat domain 17	chr4q13.3	26057	---	---	---	---	MmugDNA.17662.1.S1_s_at_ANKRD17
MmugDNA.17663.1.S1_at	207195_at	CNTN6	contactin 6	chr3p26-p25	27255	CNTN6	contactin 6	---	705207	MmugDNA.17663.1.S1_at_CNTN6
MmugDNA.17664.1.S1_at	208009_s_at	ARHGEF16	Rho guanine exchange factor (GEF) 16	chr1p36.3	27237	ARHGEF16	Rho guanine exchange factor (GEF) 16	---	694808	MmugDNA.17664.1.S1_at_ARHGEF16
MmugDNA.17665.1.S1_s_at	218266_s_at	FREQ	frequenin homolog (Drosophila)	chr9q34	23413	LOC722507	similar to neuronal calcium sensor-1	---	722507	MmugDNA.17665.1.S1_s_at_FREQ
MmugDNA.17666.1.S1_at	1569716_at	---	---	---	---	---	---	---	---	MmugDNA.17666.1.S1_at_---
MmugDNA.17667.1.S1_at	239558_at	---	---	---	---	---	---	---	---	MmugDNA.17667.1.S1_at_---
MmugDNA.17668.1.S1_at	243890_at	---	---	---	---	---	---	---	---	MmugDNA.17668.1.S1_at_---
MmugDNA.17669.1.S1_s_at	NA	NA	NA	NA	NA	NA	NA	NA	NA	MmugDNA.17669.1.S1_s_at_NA
MmugDNA.17670.1.S1_at	242875_at	---	---	---	---	---	---	---	---	MmugDNA.17670.1.S1_at_---
MmugDNA.17671.1.S1_at	223953_s_at	ZBTB37	zinc finger and BTB domain containing 37	chr1q25.1	84614	ZBTB37	zinc finger and BTB domain containing 37	---	708769	MmugDNA.17671.1.S1_at_ZBTB37
MmugDNA.1767.1.S1_at	1556436_at	C8orf50	chromosome 8 open reading frame 50	chr8p21.1	340414	---	---	---	---	MmugDNA.1767.1.S1_at_C8orf50
MmugDNA.17672.1.S1_at	242617_at	TMED8	Transmembrane emp24 protein transport domain containing 8	chr14q24.3	283578	---	---	---	---	MmugDNA.17672.1.S1_at_TMED8
MmugDNA.17673.1.S1_at	200764_s_at	CTNNA1	catenin (cadherin-associated protein), alpha 1, 102kDa	chr5q31	1495	CTNNA1	catenin (cadherin-associated protein), alpha 1, 102kDa	---	574385	MmugDNA.17673.1.S1_at_CTNNA1
MmugDNA.17674.1.S1_at	1570653_at	---	---	---	---	---	---	---	---	MmugDNA.17674.1.S1_at_---
MmugDNA.17675.1.S1_at	1570087_at	---	---	---	---	---	---	---	---	MmugDNA.17675.1.S1_at_---
MmugDNA.17676.1.S1_at	219642_s_at	PEX5L	peroxisomal biogenesis factor 5-like	chr3q26.33	51555	LOC715853	Similar to interphase cytoplasmic foci protein 45	---	715853	MmugDNA.17676.1.S1_at_PEX5L
MmugDNA.17677.1.S1_at	232685_at	---	---	---	---	---	---	---	---	MmugDNA.17677.1.S1_at_---
MmugDNA.17679.1.S1_at	236973_at	LOC100131662	hypothetical LOC100131662	chr2q11.1	100131662	---	---	---	---	MmugDNA.17679.1.S1_at_LOC10013
MmugDNA.17680.1.S1_at	228273_at	---	---	---	---	---	---	---	---	MmugDNA.17680.1.S1_at_---
MmugDNA.17681.1.S1_at	227716_at	UBXN11	UBX domain protein 11	chr1p36.11	91544	LOC719826	similar to socius isoform 2	---	719826	MmugDNA.17681.1.S1_at_UBXN11
MmugDNA.1768.1.S1_at	237575_at	---	---	---	---	---	---	---	---	MmugDNA.1768.1.S1_at_---
MmugDNA.17682.1.S1_at	205856_at	SLC14A1	solute carrier family 14 (urea transporter), member 1 (Kidd blood group)	chr18q11-q12	6563	SLC14A1	similar to Urea transporter, erythrocyte	---	695193	MmugDNA.17682.1.S1_at_SLC14A1
MmugDNA.17683.1.S1_at	1555140_a_at	BCL2L2	BCL2-like 2	chr14q11.2-q12	599	---	---	---	---	MmugDNA.17683.1.S1_at_BCL2L2
MmugDNA.17684.1.S1_at	233888_s_at	SRGAP1	SLIT-ROBO Rho GTPase activating protein 1	chr12q14.2	57522	LOC717169	similar to SLIT-ROBO Rho GTPase-activating protein 1	---	717169	MmugDNA.17684.1.S1_at_SRGAP1
MmugDNA.17685.1.S1_at	234357_at	---	---	---	---	---	---	---	---	MmugDNA.17685.1.S1_at_---
MmugDNA.17686.1.S1_at	217979_at	TSPAN13	tetraspanin 13	chr7p21.1	27075	LOC719260	Similar to absent in melanoma 2	---	719260	MmugDNA.17686.1.S1_at_TSPAN13
MmugDNA.17687.1.S1_at	1563473_at	---	---	---	---	---	---	---	---	MmugDNA.17687.1.S1_at_---
MmugDNA.17688.1.S1_at	232907_at	UBR4	ubiquitin protein ligase E3 component n-recognin 4	chr1p36.13	23352	---	---	---	---	MmugDNA.17688.1.S1_at_UBR4
MmugDNA.17689.1.S1_at	207451_at	NKX2-8	NK2 homeobox 8	chr14q13.3	26257	LOC696564	similar to NK2 transcription factor related, locus 8	---	696564	MmugDNA.17689.1.S1_at_NKX2-8
MmugDNA.17690.1.S1_at	229542_at	C20orf85	chromosome 20 open reading frame 85	chr20q13.32	128602	LOC709796	Similar to 60S ribosomal protein L17 (L23) (Amino acid starvation-induced protein) (ASI)	---	709796	MmugDNA.17690.1.S1_at_C20orf85
MmugDNA.17691.1.S1_at	239992_at	---	---	---	---	---	---	---	---	MmugDNA.17691.1.S1_at_---
MmugDNA.1769.1.S1_at	231252_at	C2orf67	chromosome 2 open reading frame 67	chr2q34	151050	LOC713138	similar to CG4699-PA, isoform A	---	713138	MmugDNA.1769.1.S1_at_C2orf67
MmugDNA.17692.1.S1_at	NA	NA	NA	NA	NA	NA	NA	NA	NA	MmugDNA.17692.1.S1_at_NA
MmugDNA.17692.1.S1_s_at	222578_s_at	UBA5	ubiquitin-like modifier activating enzyme 5	chr3q22.1	79876	LOC717647	similar to ubiquitin-activating enzyme E1-domain containing 1 isoform 1	---	717647	MmugDNA.17692.1.S1_s_at_UBA5
MmugDNA.17693.1.S1_at	222579_at	UBA5	ubiquitin-like modifier activating enzyme 5	chr3q22.1	79876	LOC717647	similar to ubiquitin-activating enzyme E1-domain containing 1 isoform 1	---	717647	MmugDNA.17693.1.S1_at_UBA5
MmugDNA.17694.1.S1_at	226000_at	CTTNBP2NL	CTTNBP2 N-terminal like	chr1p13.2	55917	CTTNBP2NL	CTTNBP2 N-terminal like	---	705295	MmugDNA.17694.1.S1_at_CTTNBP2NL
MmugDNA.17695.1.S1_at	1566882_at	---	---	---	---	---	---	---	---	MmugDNA.17695.1.S1_at_---

MmugDNA.17696.1.S1_at	235296_at	EIF5A2	eukaryotic translation initiation factor 5A2	chr3q26.2		56648	LOC695647	similar to eukaryotic translation initiation factor 5A2	---		695647	MmugDNA.17696.1.S1_at_EIF5A2
MmugDNA.17696.1.S1_s_at	NA	NA	NA	NA	NA		NA	NA	NA	NA		MmugDNA.17696.1.S1_s_at_NA
MmugDNA.17697.1.S1_at	244052_at	CBR4	carbonyl reductase 4	chr4q32.3		84869	---	---	---	---		MmugDNA.17697.1.S1_at_CBR4
MmugDNA.17698.1.S1_at	NA	NA	NA	NA	NA		NA	NA	NA	NA		MmugDNA.17698.1.S1_at_NA
MmugDNA.17699.1.S1_at	219554_at	RHCG	Rh family, C glycoprotein	chr15q25		51458	RHCG	Rh family, C glycoprotein	---		664731	MmugDNA.17699.1.S1_at_RHCG
MmugDNA.17700.1.S1_at	1556818_at	---	---	---	---		---	---	---	---		MmugDNA.17700.1.S1_at_---
MmugDNA.17701.1.S1_at	233247_x_at	ZDHHC21	zinc finger, DHHC-type containing 21	chr9p22.3		340481	---	---	---	---		MmugDNA.17701.1.S1_at_ZDHHC21
MmugDNA.1770.1.S1_at	232431_at	---	---	---	---		---	---	---	---		MmugDNA.1770.1.S1_at_---
MmugDNA.17702.1.S1_at	1562004_x_at	MYOZ3	myozenin 3	chr5q33.1		91977	LOC712647	similar to myozenin 3	---		712647	MmugDNA.17702.1.S1_at_MYOZ3
MmugDNA.17703.1.S1_at	236885_at	MEX3A	mex-3 homolog A (C. elegans)	chr1q22		92312	LOC719403	similar to ring finger and KH domain containing 2	---		719403	MmugDNA.17703.1.S1_at_MEX3A
MmugDNA.17703.1.S1_x_at	NA	NA	NA	NA	NA		NA	NA	NA	NA		MmugDNA.17703.1.S1_x_at_NA
MmugDNA.17704.1.S1_at	217838_s_at	EVL	Enah/Vasp-like	chr14q32.2		51466	EVL	Enah/Vasp-like	---		706316	MmugDNA.17704.1.S1_at_EVL
MmugDNA.17705.1.S1_s_at	210063_at	SARDH	sarcosine dehydrogenase	chr9q33-q34		1757	LOC713289	similar to sarcosine dehydrogenase	---		713289	MmugDNA.17705.1.S1_s_at_SARDH
MmugDNA.17706.1.S1_at	237555_at	CARS2	cysteinyl-tRNA synthetase 2, mitochondrial (putative)	chr13q34		79587	---	---	---	---		MmugDNA.17706.1.S1_at_CARS2
MmugDNA.17707.1.S1_at	232060_at	ROR1	receptor tyrosine kinase-like orphan receptor 1	chr1p32-p31		4919	---	---	---	---		MmugDNA.17707.1.S1_at_ROR1
MmugDNA.17708.1.S1_at	212409_s_at	TOR1AIP1	torsin A interacting protein 1	chr1q24.2		26092	TICAM2	Toll-like receptor adaptor molecule 2	---		696269	MmugDNA.17708.1.S1_at_TOR1AIP1
MmugDNA.17709.1.S1_at	212408_at	TOR1AIP1	torsin A interacting protein 1	chr1q24.2		26092	---	---	---	---		MmugDNA.17709.1.S1_at_TOR1AIP1
MmugDNA.17710.1.S1_at	236977_at	LOC646588	hypothetical protein LOC646588	chr7p15.2		646588	LOC702495	hypothetical protein LOC702495	---		702495	MmugDNA.17710.1.S1_at_LOC646588
MmugDNA.17711.1.S1_at	1566186_at	---	---	---	---		---	---	---	---		MmugDNA.17711.1.S1_at_---
MmugDNA.1771.1.S1_at	1554356_at	GINS4	GINS complex subunit 4 (Sld5 homolog)	chr8p11.21		84296	---	---	---	---		MmugDNA.1771.1.S1_at_GINS4
MmugDNA.17712.1.S1_at	203103_s_at	PRPF19	PRP19/PSO4 pre-mRNA processing factor 19 homolog (S. cerevisiae)	chr11q12.2		27339	PRPF19	PRP19/PSO4 pre-mRNA processing factor 19 homolog	---		696170	MmugDNA.17712.1.S1_at_PRPF19
MmugDNA.17713.1.S1_at	216659_at	LOC1720	dihydrofolate reductase pseudogene	chr2p12		1720	LOC695198	similar to dihydrofolate reductase	---		695198	MmugDNA.17713.1.S1_at_LOC1720
MmugDNA.17714.1.S1_at	237095_at	ASXL2	additional sex combs like 2 (Drosophila)	chr2p24.1		55252	---	---	---	---		MmugDNA.17714.1.S1_at_ASXL2
MmugDNA.17715.1.S1_at	201194_at	SEPW1	selenoprotein W, 1	chr19q13.3		6415	SEPW1	selenoprotein W, 1	---		718370	MmugDNA.17715.1.S1_at_SEPW1
MmugDNA.17716.1.S1_at	226906_s_at	ARHGAP9	Rho GTPase activating protein 9	chr12q14		64333	ARHGAP9	Rho GTPase activating protein 9	---		711684	MmugDNA.17716.1.S1_at_ARHGAP9
MmugDNA.17716.1.S1_s_at	NA	NA	NA	NA	NA		NA	NA	NA	NA		MmugDNA.17716.1.S1_s_at_NA
MmugDNA.17717.1.S1_at	244271_at	---	---	---	---		---	---	---	---		MmugDNA.17717.1.S1_at_---
MmugDNA.17718.1.S1_at	237681_at	---	---	---	---		---	---	---	---		MmugDNA.17718.1.S1_at_---
MmugDNA.17719.1.S1_x_at	NA	NA	NA	NA	NA		NA	NA	NA	NA		MmugDNA.17719.1.S1_x_at_NA
MmugDNA.177.1.S1_at	227564_at	HGSNAT	heparan-alpha-glucosaminide N-acetyltransferase	chr8p11.1		138050	LOC712888	similar to CG6903-PA	---		712888	MmugDNA.177.1.S1_at_HGSNAT
MmugDNA.17721.1.S1_at	238539_at	HPS3	Hermansky-Pudlak syndrome 3	chr3q24		84343	LOC712282	similar to Hermansky-Pudlak syndrome 3 protein	---		712282	MmugDNA.17721.1.S1_at_HPS3
MmugDNA.1772.1.S1_at	230267_at	---	---	---	---		---	---	---	---		MmugDNA.1772.1.S1_at_---
MmugDNA.17722.1.S1_at	241306_at	---	---	---	---		---	---	---	---		MmugDNA.17722.1.S1_at_---
MmugDNA.17723.1.S1_at	242929_at	---	---	---	---		---	---	---	---		MmugDNA.17723.1.S1_at_---
MmugDNA.17724.1.S1_at	233066_at	---	---	---	---		---	---	---	---		MmugDNA.17724.1.S1_at_---
MmugDNA.17725.1.S1_at	203036_s_at	MTSS1	metastasis suppressor 1	chr8p22		9788	MTSS1	metastasis suppressor 1	---		711465	MmugDNA.17725.1.S1_at_MTSS1
MmugDNA.17726.1.S1_at	NA	NA	NA	NA	NA		NA	NA	NA	NA		MmugDNA.17726.1.S1_at_NA
MmugDNA.17726.1.S1_x_at	216584_at	---	---	---	---		LOC695773	Similar to 60S ribosomal protein L23a	---		695773	MmugDNA.17726.1.S1_x_at_---
MmugDNA.17727.1.S1_at	206473_at	MBTPS2	membrane-bound transcription factor peptidase, site 2	chrXp22.13		51360	MBTPS2	membrane-bound transcription factor peptidase, site 2	---		696540	MmugDNA.17727.1.S1_at_MBTPS2
MmugDNA.17728.1.S1_at	227946_at	OSBPL7	oxysterol binding protein-like 7	chr17q21		114881	OSBPL7	oxysterol-binding protein-like protein 7	---		694186	MmugDNA.17728.1.S1_at_OSBPL7
MmugDNA.17729.1.S1_at	218207_s_at	STMN3	stathmin-like 3	chr20q13.3		50861	LOC719538	similar to SCG10-like-protein	---		719538	MmugDNA.17729.1.S1_at_STMN3
MmugDNA.17730.1.S1_at	226089_at	RABL3	RAB, member of RAS oncogene family-like 3	chr3q13.33		285282	LOC713621	similar to RAB, member of RAS oncogene family-like 3	---		713621	MmugDNA.17730.1.S1_at_RABL3
MmugDNA.17731.1.S1_at	219808_at	SCLY	selenocysteine lyase	chr2q37.3		51540	LOC695511	similar to selenocysteine lyase	---		695511	MmugDNA.17731.1.S1_at_SCLY
MmugDNA.1773.1.S1_at	229090_at	LOC220930	hypothetical LOC220930	chr10p11.23		220930	---	---	---	---		MmugDNA.1773.1.S1_at_LOC220930
MmugDNA.17732.1.S1_at	222309_at	C6orf62	Chromosome 6 open reading frame 62	chr6p22.2		81688	---	---	---	---		MmugDNA.17732.1.S1_at_C6orf62
MmugDNA.17733.1.S1_at	226090_x_at	RABL3	RAB, member of RAS oncogene family-like 3	chr3q13.33		285282	LOC713621	similar to RAB, member of RAS oncogene family-like 3	---		713621	MmugDNA.17733.1.S1_at_RABL3
MmugDNA.17734.1.S1_at	1556666_a_at	TTC6	tetratricopeptide repeat domain 6	chr14q21.1		115669	TTC6	tetratricopeptide repeat domain 6	---		698673	MmugDNA.17734.1.S1_at_TTC6
MmugDNA.17735.1.S1_at	226632_at	CYGB	cytoglobin	chr17q25.3		114757	CYGB	cytoglobin	---		707130	MmugDNA.17735.1.S1_at_CYGB
MmugDNA.17736.1.S1_at	213316_at	KIAA1462	KIAA1462	chr10p11.23		57608	---	---	---	---		MmugDNA.17736.1.S1_at_KIAA1462
MmugDNA.17737.1.S1_at	220357_s_at	SGK2	serum/glucocorticoid regulated kinase 2	chr20q13.2		10110	LOC695356	similar to serum/glucocorticoid regulated kinase 2 isoform beta	---		695356	MmugDNA.17737.1.S1_at_SGK2
MmugDNA.17738.1.S1_at	213445_at	ZC3H3	zinc finger CCCH-type containing 3	chr8q24.3		23144	ZC3H3	zinc finger CCCH-type containing 3	---		708584	MmugDNA.17738.1.S1_at_ZC3H3
MmugDNA.17739.1.S1_at	1569422_at	FAM129C	family with sequence similarity 129, member C	chr19p13.11		199786	LOC719972	similar to B-cell novel protein 1	---		719972	MmugDNA.17739.1.S1_at_FAM129C
MmugDNA.17740.1.S1_at	1561714_a_at	B3GALNT2	beta-1,3-N-acetylgalactosaminyltransferase 2	chr1q42.3		148789	---	---	---	---		MmugDNA.17740.1.S1_at_B3GALNT2
MmugDNA.17741.1.S1_at	NA	NA	NA	NA	NA		NA	NA	NA	NA		MmugDNA.17741.1.S1_at_NA
MmugDNA.1774.1.S1_at	237233_at	---	---	---	---		---	---	---	---		MmugDNA.1774.1.S1_at_---
MmugDNA.17742.1.S1_at	1559650_at	LOC100128081	Hypothetical LOC100128081	chr7p15.1		100128081	LOC706902	Similar to exonuclease 1 isoform b	---		706902	MmugDNA.17742.1.S1_at_LOC100128(
MmugDNA.17743.1.S1_at	205304_s_at	KCNJ8	potassium inwardly-rectifying channel, subfamily J, member 8	chr12p11.23		3764	---	---	---	---		MmugDNA.17743.1.S1_at_KCNJ8
MmugDNA.17744.1.S1_at	NA	NA	NA	NA	NA		NA	NA	NA	NA		MmugDNA.17744.1.S1_at_NA
MmugDNA.17744.1.S1_s_at	234318_x_at	NDOR1	NADPH dependent diflavin oxidoreductase 1	chr9q34.3		27158	LOC706056	similar to NADPH dependent diflavin oxidoreductase 1	---		706056	MmugDNA.17744.1.S1_s_at_NDOR1
MmugDNA.17745.1.S1_at	242385_at	RORB	RAR-related orphan receptor B	chr9q22		6096	---	---	---	---		MmugDNA.17745.1.S1_at_RORB
MmugDNA.17746.1.S1_at	229597_s_at	WDFY4	WDFY family member 4	chr10q11.22		57705	LOC719472	similar to WD repeat and FYVE domain containing 3	---		719472	MmugDNA.17746.1.S1_at_WDFY4
MmugDNA.17747.1.S1_at	1559535_s_at	---	---	---	---		---	---	---	---		MmugDNA.17747.1.S1_at_---
MmugDNA.17748.1.S1_at	208388_at	NR2E3	nuclear receptor subfamily 2, group E, member 3	chr15q22.32		10002	NR2E3	nuclear receptor subfamily 2, group E, member 3	---		701402	MmugDNA.17748.1.S1_at_NR2E3
MmugDNA.17749.1.S1_at	242234_at	XAF1	XIAP associated factor 1	chr17p13.2		54739	---	---	---	---		MmugDNA.17749.1.S1_at_XAF1
MmugDNA.17750.1.S1_at	212930_at	ATP2B1	ATPase, Ca++ transporting, plasma membrane 1	chr12q21.3		490	LOC709102	similar to plasma membrane calcium ATPase 1 isoform 1b	---		709102	MmugDNA.17750.1.S1_at_ATP2B1
MmugDNA.17751.1.S1_at	235889_at	---	---	---	---		---	---	---	---		MmugDNA.17751.1.S1_at_---
MmugDNA.1775.1.S1_at	211156_at	CDKN2A	cyclin-dependent kinase inhibitor 2A (melanoma, p16, inhibits CDK4)	chr9p21		1029	LOC709988	similar to Cyclin-dependent kinase inhibitor 2A, isoforms 1/2/3 (Cyclin-dependent kinase 4 inhibitor A)	---		709988	MmugDNA.1775.1.S1_at_CDKN2A
MmugDNA.17753.1.S1_at	1562820_at	---	---	---	---		---	---	---	---		MmugDNA.17753.1.S1_at_---
MmugDNA.17754.1.S1_at	231773_at	ANGPTL1	angiopoietin-like 1	chr1q25.2		9068	---	---	---	---		MmugDNA.17754.1.S1_at_ANGPTL1
MmugDNA.17755.1.S1_at	1552898_a_at	KCNG3	potassium voltage-gated channel, subfamily G, member 3	chr2p21		170850	LOC712838	similar to potassium voltage-gated channel, subfamily G, member 3 isoform 1	---		712838	MmugDNA.17755.1.S1_at_KCNG3
MmugDNA.17756.1.S1_at	NA	NA	NA	NA	NA		NA	NA	NA	NA		MmugDNA.17756.1.S1_at_NA
MmugDNA.17757.1.S1_at	239932_at	---	---	---	---		---	---	---	---		MmugDNA.17757.1.S1_at_---
MmugDNA.17758.1.S1_at	1552897_a_at	KCNG3	potassium voltage-gated channel, subfamily G, member 3	chr2p21		170850	LOC712838	similar to potassium voltage-gated channel, subfamily G, member 3 isoform 1	---		712838	MmugDNA.17758.1.S1_at_KCNG3
MmugDNA.17759.1.S1_at	239727_at	DIO3OS	DIO3 opposite strand (non-protein coding)	chr14q32.31		64150	---	---	---	---		MmugDNA.17759.1.S1_at_DIO3OS
MmugDNA.17760.1.S1_at	227374_at	EARS2	glutamyl-tRNA synthetase 2, mitochondrial (putative)	chr16p12.1		124454	EARS2	glutamyl-tRNA synthetase 2, mitochondrial (putative)	---		700487	MmugDNA.17760.1.S1_at_EARS2
MmugDNA.17761.1.S1_at	1560570_a_at	---	---	---	---		---	---	---	---		MmugDNA.17761.1.S1_at_---
MmugDNA.1776.1.S1_at	221238_at	NSBP1	nucleosomal binding protein 1	chrXq13.3		79366	LOC709253	similar to nucleosomal binding protein 1	---		709253	MmugDNA.1776.1.S1_at_NSBP1
MmugDNA.17762.1.S1_at	NA	NA	NA	NA	NA		NA	NA	NA	NA		MmugDNA.17762.1.S1_at_NA
MmugDNA.17762.1.S1_s_at	1554795_a_at	FBLIM1	filamin binding LIM protein 1	chr1p36.21		54751	LOC695727	similar to filamin-binding LIM protein-1 isoform a	---		695727	MmugDNA.17762.1.S1_s_at_FBLIM1
MmugDNA.17763.1.S1_at	205430_at	BMP5	bone morphogenetic protein 5	chr6p12.1		653	LOC718630	Similar to large subunit ribosomal protein L36a	---		718630	MmugDNA.17763.1.S1_at_BMP5
MmugDNA.17764.1.S1_at	226488_at	RCCD1	RCC1 domain containing 1	chr15q26.1		91433	---	---	---	---		MmugDNA.17764.1.S1_at_RCCD1
MmugDNA.17765.1.S1_at	222806_s_at	C11orf30	chromosome 11 open reading frame 30	chr11q13.5		56946	C14H11orf30	chromosome 11 open reading frame 30 ortholog	---		697319	MmugDNA.17765.1.S1_at_C11orf30
MmugDNA.17766.1.S1_at	236326_at	HDAC7	histone deacetylase 7	chr12q13.1		51564	---	---	---	---		MmugDNA.17766.1.S1_at_HDAC7
MmugDNA.17767.1.S1_s_at	222807_at	C11orf30	chromosome 11 open reading frame 30	chr11q13.5		56946	C14H11orf30	chromosome 11 open reading frame 30 ortholog	---		697319	MmugDNA.17767.1.S1_s_at_C11orf30
MmugDNA.17768.1.S1_at	215911_x_at	ATP2B3	ATPase, Ca++ transporting, plasma membrane 3	chrXq28		492	LOC720457	similar to Plasma membrane calcium-transporting ATPase 3 (PMCA3) (Plasma membrane calcium p	---		720457	MmugDNA.17768.1.S1_at_ATP2B3
MmugDNA.17769.1.S1_at	240342_at	TRIM61	tripartite motif-containing 61	chr4q32.3		391712	LOC704463	similar to tripartite motif-containing 61	---		704463	MmugDNA.17769.1.S1_at_TRIM61
MmugDNA.17770.1.S1_at	228727_at	ANXA11	annexin A11	chr10q23		311	---	---	---	---		MmugDNA.17770.1.S1_at_ANXA11
MmugDNA.17771.1.S1_at	204010_s_at	KRAS	v-Ki-ras2 Kirsten rat sarcoma viral oncogene homolog	chr12p12.1		3845	---	---	---	---		MmugDNA.17771.1.S1_at_KRAS
MmugDNA.1777.1.S1_at	221239_s_at	FCRL2	Fc receptor-like 2	chr1q21		79368	LOC718824	similar to Fc receptor-like 2 isoform b	---		718824	MmugDNA.1777.1.S1_at_FCRL2
MmugDNA.17773.1.S1_at	219515_at	PRDM10	PR domain containing 10	chr11q25		56980	LOC715177	similar to PR domain containing 10 isoform 1	---		715177	MmugDNA.17773.1.S1_at_PRDM10
MmugDNA.17774.1.S1_at	231983_at	C1orf69	chromosome 1 open reading frame 69	chr1q42.13		200205	C1H1orf69	chromosome 1 open reading frame 69 ortholog	---		695043	MmugDNA.17774.1.S1_at_C1orf69
MmugDNA.17775.1.S1_s_at	202412_s_at	USP1	ubiquitin specific peptidase 1	chr1p31.3		7398	USP1	ubiquitin specific peptidase 1	---		695157	MmugDNA.17775.1.S1_s_at_USP1
MmugDNA.17776.1.S1_at	219835_at	PRDM8	PR domain containing 8	chr4q21		56978	PRDM8	PR domain containing 8	---		696013	MmugDNA.17776.1.S1_at_PRDM8
MmugDNA.17777.1.S1_at	203671_at	TPMT	thiopurine S-methyltransferase	chr6p22.3		7172	TPMT	thiopurine S-methyltransferase	---		704564	MmugDNA.17777.1.S1_at_TPMT
MmugDNA.17778.1.S1_at	1560813_at	---	---	---	---		---	---	---	---		MmugDNA.17778.1.S1_at_---
MmugDNA.17779.1.S1_at	226066_at	MITF	microphthalmia-associated transcription factor	chr3p14.2-p14.1		4286	---	---	---	---		MmugDNA.17779.1.S1_at_MITF
MmugDNA.17780.1.S1_at	1560489_at	---	---	---	---		---	---	---	---		MmugDNA.17780.1.S1_at_---
MmugDNA.17781.1.S1_at	201829_at	NET1	neuroepithelial cell transforming 1	chr10p15		10276	NET1	neuroepithelial cell transforming gene 1	---		712080	MmugDNA.17781.1.S1_at_NET1
MmugDNA.1778.1.S1_at	234726_s_at	TMEM168	transmembrane protein 168	chr7q31.32		64418	LOC702972	hypothetical protein LOC702972	---		702972	MmugDNA.1778.1.S1_at_TMEM168
MmugDNA.17782.1.S1_at	226652_at	USP3	ubiquitin specific peptidase 3	chr15q22.3		9960	LOC696998	Hypothetical protein LOC696998	---		696998	MmugDNA.17782.1.S1_at_USP3
MmugDNA.17783.1.S1_at	234217_at	---	---	---	---		---	---	---	---		MmugDNA.17783.1.S1_at_---
MmugDNA.17784.1.S1_at	233678_at	---	---	---	---		---	---	---	---		MmugDNA.17784.1.S1_at_---
MmugDNA.17785.1.S1_at	236899_at	LOC729397	hypothetical LOC729397	chr1q42.3		729397	---	---	---	---		MmugDNA.17785.1.S1_at_LOC729397
MmugDNA.17786.1.S1_at	217565_at	GRIA3	glutamate receptor, ionotrophic, AMPA 3	chrXq25-q26		2892	---	---	---	---		MmugDNA.17786.1.S1_at_GRIA3
MmugDNA.17787.1.S1_at	1556082_a_at	---	---	---	---		---	---	---	---		MmugDNA.17787.1.S1_at_---
MmugDNA.17788.1.S1_at	230189_x_at	NELF	Nasal embryonic LHRH factor	chr9q34.3		26012	---	---	---	---		MmugDNA.17788.1.S1_at_NELF
MmugDNA.17789.1.S1_at	1559524_at	---	---	---	---		---	---	---	---		MmugDNA.17789.1.S1_at_---
MmugDNA.17790.1.S1_at	230738_at	LOC730631	Hypothetical LOC730631	chr1q21.2		730631	---	---	---	---		MmugDNA.17790.1.S1_at_LOC730631

MmugDNA.17791.1.S1_at	NA	NA	NA	NA	NA		NA	NA	NA	NA		MmugDNA.17791.1.S1_at_NA
MmugDNA.1779.1.S1_at	221240_s_at	B3GNT4	UDP-GlcNAc:betaGal beta-1,3-N-acetylglucosaminyltransferase 4	chr12q24		79369	B3GNT4	UDP-GlcNAc:betaGal beta-1,3-N-acetylglucosaminyltransferase 4	---		702023	MmugDNA.1779.1.S1_at_B3GNT4
MmugDNA.17792.1.S1_at	215544_s_at	UBOX5	U-box domain containing 5	chr20p13		22888	UBOX5	U-box domain containing 5	---		717166	MmugDNA.17792.1.S1_at_UBOX5
MmugDNA.17793.1.S1_at	207325_x_at	MAGEA1	melanoma antigen family A, 1 (directs expression of antigen MZ2-E)	chrXq28		4100	LOC695155	similar to melanoma antigen family A, 11 isoform a	---		695155	MmugDNA.17793.1.S1_at_MAGEA1
MmugDNA.17794.1.S1_at	1557122_s_at	GABRB2	gamma-aminobutyric acid (GABA) A receptor, beta 2	chr5q34		2561	LOC712612	Similar to tubulin-specific chaperone a	---		712612	MmugDNA.17794.1.S1_at_GABRB2
MmugDNA.17795.1.S1_at	203579_s_at	SLC7A6	solute carrier family 7 (cationic amino acid transporter, y+ system), member 6	chr16q22.1		9057	---	---	---	---		MmugDNA.17795.1.S1_at_SLC7A6
MmugDNA.17796.1.S1_at	213954_at	FAM169A	family with sequence similarity 169, member A	chr5q13.3		26049	LOC710539	hypothetical protein LOC710539	---		710539	MmugDNA.17796.1.S1_at_FAM169A
MmugDNA.17797.1.S1_at	201475_x_at	MARS	methionyl-tRNA synthetase	chr12q13.2		4141	LOC714744	similar to methionine-tRNA synthetase	---		714744	MmugDNA.17797.1.S1_at_MARS
MmugDNA.17798.1.S1_at	208434_at	MDS1	myelodysplasia syndrome 1	chr3q26		4197	---	---	---	---		MmugDNA.17798.1.S1_at_MDS1
MmugDNA.17799.1.S1_at	1553907_a_at	EXD1	exonuclease 3'-5' domain containing 1	chr15q15.1		161829	EXD1	exonuclease 3'-5' domain containing 1	---		705364	MmugDNA.17799.1.S1_at_EXD1
MmugDNA.17800.1.S1_at	1553686_at	C18orf25	chromosome 18 open reading frame 25	chr18q21.1		147339	C18H18orf25	similar to chromosome 18 open reading frame 25 isoform b	---		695993	MmugDNA.17800.1.S1_at_C18orf25
MmugDNA.17801.1.S1_at	238431_at	---	---	---	---		---	---	---	---		MmugDNA.17801.1.S1_at_---
MmugDNA.1780.1.S1_at	235354_s_at	RSRC1	arginine/serine-rich coiled-coil 1	chr3q25.32		51319	---	---	---	---		MmugDNA.1780.1.S1_at_RSRC1
MmugDNA.17802.1.S1_at	NA	NA	NA	NA	NA		NA	NA	NA	NA		MmugDNA.17802.1.S1_at_NA
MmugDNA.17802.1.S1_s_at	222867_s_at	MED31	mediator complex subunit 31	chr17p13.2		51003	LOC712971	similar to Mediator of RNA polymerase II transcription subunit 31 (Mediator complex subunit SOH1) (I	---		712971	MmugDNA.17802.1.S1_s_at_MED31
MmugDNA.17803.1.S1_at	223283_s_at	TSHZ1	teashirt zinc finger homeobox 1	chr18q22.3		10194	LOC703018	similar to teashirt family zinc finger 1	---		703018	MmugDNA.17803.1.S1_at_TSHZ1
MmugDNA.17804.1.S1_at	238747_at	---	---	---	---		LOC714829	Similar to trinucleotide repeat containing 6C	---		714829	MmugDNA.17804.1.S1_at_---
MmugDNA.17805.1.S1_at	211116_at	SLC9A2	solute carrier family 9 (sodium/hydrogen exchanger), member 2	chr2q11.2		6549	LOC712199	similar to solute carrier family 9 (sodium/hydrogen exchanger), isoform 2	---		712199	MmugDNA.17805.1.S1_at_SLC9A2
MmugDNA.17806.1.S1_at	NA	NA	NA	NA	NA		NA	NA	NA	NA		MmugDNA.17806.1.S1_at_NA
MmugDNA.17807.1.S1_at	222929_at	FAM160B2	family with sequence similarity 160, member B2	chr8p21.3		64760	LOC715595	similar to CG3558-PA, isoform A	---		715595	MmugDNA.17807.1.S1_at_FAM160B2
MmugDNA.17808.1.S1_at	228510_at	C6orf134	chromosome 6 open reading frame 134	chr6p21.33		79969	LOC713625	similar to CG3967-PC, isoform C	---		713625	MmugDNA.17808.1.S1_at_C6orf134
MmugDNA.17809.1.S1_at	1558254_s_at	SRPK2	SFRS protein kinase 2	chr7q22-q31.1		6733	SRPK3	SFRS protein kinase 3	---		697351	MmugDNA.17809.1.S1_at_SRPK2
MmugDNA.17810.1.S1_at	NA	NA	NA	NA	NA		NA	NA	NA	NA		MmugDNA.17810.1.S1_at_NA
MmugDNA.17811.1.S1_at	215561_s_at	IL1R1	interleukin 1 receptor, type I	chr2q12		3554	LOC714817	Similar to peptidase (prosome, macropain) 26S subunit, ATPase 1	---		714817	MmugDNA.17811.1.S1_at_IL1R1
MmugDNA.1781.1.S1_at	217701_x_at	---	---	---	---		---	---	---	---		MmugDNA.1781.1.S1_at_---
MmugDNA.17812.1.S1_at	214992_s_at	DNASE2	deoxyribonuclease II, lysosomal	chr19p13.2		1777	LOC716802	hypothetical protein LOC716802	---		716802	MmugDNA.17812.1.S1_at_DNASE2
MmugDNA.17813.1.S1_at	243758_at	CCDC37	coiled-coil domain containing 37	chr3q21.2		348807	LOC716433	hypothetical protein LOC716433	---		716433	MmugDNA.17813.1.S1_at_CCDC37
MmugDNA.17814.1.S1_at	236747_at	---	---	---	---		---	---	---	---		MmugDNA.17814.1.S1_at_---
MmugDNA.17815.1.S1_at	1554441_a_at	WAPAL	wings apart-like homolog (Drosophila)	chr10q23.2		23063	LOC700171	similar to wings apart-like homolog	---		700171	MmugDNA.17815.1.S1_at_WAPAL
MmugDNA.17816.1.S1_at	237448_at	---	---	---	---		---	---	---	---		MmugDNA.17816.1.S1_at_---
MmugDNA.17817.1.S1_at	227887_at	LOC400236	hypothetical LOC400236	chr14q32.11		400236	LOC694879	hypothetical protein LOC694879	---		694879	MmugDNA.17817.1.S1_at_LOC400236
MmugDNA.17818.1.S1_at	1556689_a_at	WNT4	wingless-type MMTV integration site family, member 4	chr1p36.23-p35.1		54361	---	---	---	---		MmugDNA.17818.1.S1_at_WNT4
MmugDNA.17819.1.S1_at	203936_s_at	MMP9	matrix metallopeptidase 9 (gelatinase B, 92kDa gelatinase, 92kDa type IV collagenase)	chr20q11.2-q13.1		4318	MMP9	matrix metallopeptidase 9 (gelatinase B, 92kDa gelatinase, 92kDa type IV collagenase)	---		708084	MmugDNA.17819.1.S1_at_MMP9
MmugDNA.178.1.S1_at	238904_at	---	---	---	---		---	---	---	---		MmugDNA.178.1.S1_at_---
MmugDNA.17820.1.S1_at	211702_s_at	USP32	ubiquitin specific peptidase 32	chr17q23.2		84669	LOC716857	similar to ubiquitin specific protease 32	---		716857	MmugDNA.17820.1.S1_at_USP32
MmugDNA.17821.1.S1_at	230492_s_at	RP5-1022P6.2	hypothetical protein KIAA1434	chr20p12.3		56261	---	---	---	---		MmugDNA.17821.1.S1_at_RP5-1022P6
MmugDNA.1782.1.S1_at	1564295_at	FLJ25917	hypothetical gene supported by AK098783	chrXp11.3		401585	---	---	---	---		MmugDNA.1782.1.S1_at_FLJ25917
MmugDNA.17822.1.S1_at	1563272_at	TBC1D8B	TBC1 domain family, member 8B (with GRAM domain)	chrXq22.3		54885	---	---	---	---		MmugDNA.17822.1.S1_at_TBC1D8B
MmugDNA.17823.1.S1_at	1557296_at	FLJ12825	hypothetical LOC440101	chr12q13.13		440101	---	---	---	---		MmugDNA.17823.1.S1_at_FLJ12825
MmugDNA.17824.1.S1_at	1558760_at	---	---	---	---		---	---	---	---		MmugDNA.17824.1.S1_at_---
MmugDNA.17825.1.S1_at	1563173_at	---	---	---	---		---	---	---	---		MmugDNA.17825.1.S1_at_---
MmugDNA.17826.1.S1_at	228458_at	C6orf226	chromosome 6 open reading frame 226	chr6p21.1		441150	LOC698914	hypothetical protein LOC698914	---		698914	MmugDNA.17826.1.S1_at_C6orf226
MmugDNA.17827.1.S1_at	234304_s_at	IPO11	importin 11	chr5q12.1		51194	IPO11	importin 11	---		694610	MmugDNA.17827.1.S1_at_IPO11
MmugDNA.17828.1.S1_at	228399_at	OSR1	odd-skipped related 1 (Drosophila)	chr2p24.1		130497	---	---	---	---		MmugDNA.17828.1.S1_at_OSR1
MmugDNA.17829.1.S1_at	206304_at	MYBPH	myosin binding protein H	chr1q32.1		4608	MYBPH	myosin binding protein H	---		703516	MmugDNA.17829.1.S1_at_MYBPH
MmugDNA.17830.1.S1_at	1568748_at	---	---	---	---		---	---	---	---		MmugDNA.17830.1.S1_at_---
MmugDNA.17831.1.S1_at	236284_at	KIAA0146	KIAA0146	chr8q11.21		23514	---	---	---	---		MmugDNA.17831.1.S1_at_KIAA0146
MmugDNA.1783.1.S1_s_at	NA	NA	NA	NA	NA		NA	NA	NA	NA		MmugDNA.1783.1.S1_s_at_NA
MmugDNA.17832.1.S1_at	202785_at	NDUFA7	NADH dehydrogenase (ubiquinone) 1 alpha subcomplex, 7, 14.5kDa	chr19p13.2		4701	LOC711212	similar to NADH dehydrogenase (ubiquinone) 1 alpha subcomplex, 7, 14.5kDa	---		711212	MmugDNA.17832.1.S1_at_NDUFA7
MmugDNA.17833.1.S1_at	236598_at	---	---	---	---		---	---	---	---		MmugDNA.17833.1.S1_at_---
MmugDNA.17834.1.S1_at	205485_at	RYR1	ryanodine receptor 1 (skeletal)	chr19q13.1		6261	---	---	---	---		MmugDNA.17834.1.S1_at_RYR1
MmugDNA.17835.1.S1_at	207046_at	HIST2H4A /// HIST2H4I	histone cluster 2, H4a /// histone cluster 2, H4b	chr1q21	554313 /// 8370		---	---	---	---		MmugDNA.17835.1.S1_at_HIST2H4A //
MmugDNA.17836.1.S1_at	203084_at	TGFB1	transforming growth factor, beta 1	chr19q13.2|19q13.1		7040	---	---	---	---		MmugDNA.17836.1.S1_at_TGFB1
MmugDNA.17837.1.S1_at	NA	NA	NA	NA	NA		NA	NA	NA	NA		MmugDNA.17837.1.S1_at_NA
MmugDNA.17838.1.S1_at	201007_at	HADHB	hydroxyacyl-Coenzyme A dehydrogenase/3-ketoacyl-Coenzyme A thiolase/enoyl-Coenzyme A hydrat	chr2p23		3032	LOC707168	similar to hydroxyacyl dehydrogenase, subunit B	---		707168	MmugDNA.17838.1.S1_at_HADHB
MmugDNA.17839.1.S1_at	215579_at	APOBEC3G	apolipoprotein B mRNA editing enzyme, catalytic polypeptide-like 3G	chr22q13.1-q13.2		60489	---	---	---	---		MmugDNA.17839.1.S1_at_APOBEC3G
MmugDNA.17840.1.S1_at	202826_at	SPINT1	serine peptidase inhibitor, Kunitz type 1	chr15q15.1		6692	LOC702605	similar to hepatocyte growth factor activator inhibitor 1 isoform 1 precursor	---		702605	MmugDNA.17840.1.S1_at_SPINT1
MmugDNA.17841.1.S1_at	233648_at	---	---	---	---		---	---	---	---		MmugDNA.17841.1.S1_at_---
MmugDNA.1784.1.S1_at	1568644_at	ZNF208	zinc finger protein 208	chr19p12		7757	LOC721428	similar to Zinc finger protein 254 (Bone marrow zinc finger 5) (BMZF-5) (Hematopoietic cell-derived zi	---		721428	MmugDNA.1784.1.S1_at_ZNF208
MmugDNA.17842.1.S1_at	204087_s_at	SLC5A6	solute carrier family 5 (sodium-dependent vitamin transporter), member 6	chr2p23		8884	SLC5A6	solute carrier family 5 (sodium-dependent vitamin transporter), member 6	---		699074	MmugDNA.17842.1.S1_at_SLC5A6
MmugDNA.17843.1.S1_at	224542_s_at	NFATC2	nuclear factor of activated T-cells, cytoplasmic, calcineurin-dependent 2	chr20q13.2-q13.3		4773	LOC710601	similar to nuclear factor of activated T-cells, cytoplasmic, calcineurin-dependent 2 isoform C	---		710601	MmugDNA.17843.1.S1_at_NFATC2
MmugDNA.17844.1.S1_at	232630_at	APIP	APAF1 interacting protein	chr11p13		51074	LOC717291	similar to APAF1 interacting protein	---		717291	MmugDNA.17844.1.S1_at_APIP
MmugDNA.17845.1.S1_at	1558489_at	---	---	---	---		---	---	---	---		MmugDNA.17845.1.S1_at_---
MmugDNA.17846.1.S1_at	223771_at	TMEM87A	Transmembrane protein 87A	chr15q15.1		25963	---	---	---	---		MmugDNA.17846.1.S1_at_TMEM87A
MmugDNA.17847.1.S1_at	244180_at	ZNF793	zinc finger protein 793	chr19q13.12		390927	LOC713931	similar to Zinc finger protein 90 (Zfp-90) (Zinc finger protein NK10)	---		713931	MmugDNA.17847.1.S1_at_ZNF793
MmugDNA.17848.1.S1_at	212180_at	CRKL	v-crk sarcoma virus CT10 oncogene homolog (avian)-like	chr22q11|22q11.21		1399	CRKL	v-crk sarcoma virus CT10 oncogene homolog (avian)-like	---		696584	MmugDNA.17848.1.S1_at_CRKL
MmugDNA.17849.1.S1_at	209261_s_at	NR2F6	nuclear receptor subfamily 2, group F, member 6	chr19p13.1		2063	LOC719699	similar to nuclear receptor subfamily 2, group F, member 6	---		719699	MmugDNA.17849.1.S1_at_NR2F6
MmugDNA.17850.1.S1_at	232626_at	---	---	---	---		---	---	---	---		MmugDNA.17850.1.S1_at_---
MmugDNA.17851.1.S1_at	202077_at	NDUFAB1	NADH dehydrogenase (ubiquinone) 1, alpha/beta subcomplex, 1, 8kDa	chr16p12.1		4706	NDUFAB1	NADH dehydrogenase (ubiquinone) 1, alpha/beta subcomplex, 1, 8kDa	---		715222	MmugDNA.17851.1.S1_at_NDUFAB1
MmugDNA.1785.1.S1_at	240366_at	LOC100129562	hypothetical protein LOC100129562	chr7q22.1		100129562	---	---	---	---		MmugDNA.1785.1.S1_at_LOC1001295(
MmugDNA.17852.1.S1_at	201543_s_at	SAR1A	SAR1 homolog A (S. cerevisiae)	chr10q22.1		56681	---	---	---	---		MmugDNA.17852.1.S1_at_SAR1A
MmugDNA.17853.1.S1_at	1560451_at	---	---	---	---		LOC717407	hypothetical protein LOC717407	---		717407	MmugDNA.17853.1.S1_at_---
MmugDNA.17854.1.S1_at	1557795_s_at	NTRK3	neurotrophic tyrosine kinase, receptor, type 3	chr15q25		4916	NTRK3	neurotrophic tyrosine kinase, receptor, type 3	---		697615	MmugDNA.17854.1.S1_at_NTRK3
MmugDNA.17855.1.S1_at	201227_s_at	NDUFB8	NADH dehydrogenase (ubiquinone) 1 beta subcomplex, 8, 19kDa	chr10q23.2-q23.33		4714	LOC710521 /// LOC7130:	similar to NADH dehydrogenase (ubiquinone) 1 beta subcomplex, 8, 19kDa /// similar to NADH dehyc	---	710521 /// 713028		MmugDNA.17855.1.S1_at_NDUFB8
MmugDNA.17856.1.S1_at	203039_s_at	NDUFS1	NADH dehydrogenase (ubiquinone) Fe-S protein 1, 75kDa (NADH-coenzyme Q reductase)	chr2q33-q34		4719	NDUFS1	NADH dehydrogenase (ubiquinone) Fe-S protein 1, 75kDa (NADH-coenzyme Q reductase)	---		706912	MmugDNA.17856.1.S1_at_NDUFS1
MmugDNA.17857.1.S1_at	1556896_at	LOC284751	hypothetical LOC284751	chr20q13.13		284751	---	---	---	---		MmugDNA.17857.1.S1_at_LOC284751
MmugDNA.17858.1.S1_at	205866_at	FCN3	ficolin (collagen/fibrinogen domain containing) 3 (Hakata antigen)	chr1p36.11		8547	LOC720154	similar to ficolin 3 isoform 1 precursor	---		720154	MmugDNA.17858.1.S1_at_FCN3
MmugDNA.17859.1.S1_at	213325_at	PVRL3	poliovirus receptor-related 3	chr3q13		25945	LOC706731	similar to nectin 3	---		706731	MmugDNA.17859.1.S1_at_PVRL3
MmugDNA.17860.1.S1_at	201076_at	NHP2L1	NHP2 non-histone chromosome protein 2-like 1 (S. cerevisiae)	chr22q13.2-q13.31		4809	LOC708283	similar to Nhp2 non-histone chromosome protein 2-like 1	---		708283	MmugDNA.17860.1.S1_at_NHP2L1
MmugDNA.17861.1.S1_at	208299_at	CACNA1I	calcium channel, voltage-dependent, T type, alpha 1I subunit	chr22q13.1		8911	---	---	---	---		MmugDNA.17861.1.S1_at_CACNA1I
MmugDNA.1786.1.S1_at	226795_at	---	---	---	---		---	---	---	---		MmugDNA.1786.1.S1_at_---
MmugDNA.17862.1.A1_at	NA	NA	NA	NA	NA		NA	NA	NA	NA		MmugDNA.17862.1.A1_at_NA
MmugDNA.17862.1.S1_at	240818_at	---	---	---	---		---	---	---	---		MmugDNA.17862.1.S1_at_---
MmugDNA.17863.1.S1_at	221283_at	RUNX2	runt-related transcription factor 2	chr6p21		860	RUNX2	runt-related transcription factor 2	---		703331	MmugDNA.17863.1.S1_at_RUNX2
MmugDNA.17864.1.S1_at	218182_s_at	CLDN1	claudin 1	chr3q28-q29		9076	LOC704330	similar to claudin 1	---		704330	MmugDNA.17864.1.S1_at_CLDN1
MmugDNA.17865.1.S1_at	207316_at	HAS1	hyaluronan synthase 1	chr19q13.4		3036	LOC719773	similar to hyaluronan synthase 1	---		719773	MmugDNA.17865.1.S1_at_HAS1
MmugDNA.17866.1.S1_at	225911_at	NPNT	nephronectin	chr4q24		255743	NPNT	nephronectin	---		694470	MmugDNA.17866.1.S1_at_NPNT
MmugDNA.17867.1.S1_at	200051_at	SART1	squamous cell carcinoma antigen recognized by T cells	chr11q13.1		9092	LOC721873	similar to squamous cell carcinoma antigen recognized by T cells 1	---		721873	MmugDNA.17867.1.S1_at_SART1
MmugDNA.17868.1.S1_at	1554768_a_at	MAD2L1	MAD2 mitotic arrest deficient-like 1 (yeast)	chr4q27		4085	LOC708574	similar to Mitotic spindle assembly checkpoint protein MAD2A (MAD2-like 1) (HsMAD2)	---		708574	MmugDNA.17868.1.S1_at_MAD2L1
MmugDNA.17869.1.S1_at	244469_at	---	---	---	---		---	---	---	---		MmugDNA.17869.1.S1_at_---
MmugDNA.17870.1.S1_at	241022_at	---	---	---	---		---	---	---	---		MmugDNA.17870.1.S1_at_---
MmugDNA.17871.1.S1_at	1562030_at	LOC284898	hypothetical protein LOC284898	chr22q12.1		284898	---	---	---	---		MmugDNA.17871.1.S1_at_LOC284898
MmugDNA.1787.1.S1_at	233917_s_at	MOV10	Mov10, Moloney leukemia virus 10, homolog (mouse)	chr1p13.2		4343	MOV10	Mov10, Moloney leukemia virus 10, homolog (mouse)	---		705910	MmugDNA.1787.1.S1_at_MOV10
MmugDNA.17872.1.S1_at	222226_at	SAA3P	serum amyloid A3 pseudogene	chr11p15.1-p14		6290	SAA3P	similar to Putative serum amyloid A-3 protein	---		695134	MmugDNA.17872.1.S1_at_SAA3P
MmugDNA.17873.1.S1_at	232142_at	ISY1	ISY1 splicing factor homolog (S. cerevisiae)	chr3q21.3		57461	---	---	---	---		MmugDNA.17873.1.S1_at_ISY1
MmugDNA.17874.1.S1_at	209640_at	PML	promyelocytic leukemia	chr15q22		5371	PML	promyelocytic leukemia	---		700379	MmugDNA.17874.1.S1_at_PML
MmugDNA.17875.1.S1_at	228183_s_at	RPAIN	RPA interacting protein	chr17p13.2		84268	LOC711897	similar to RPA interacting protein isoform 1	---		711897	MmugDNA.17875.1.S1_at_RPAIN
MmugDNA.17876.1.S1_at	217359_s_at	NCAM1	neural cell adhesion molecule 1	chr11q23.1		4684	NCAM1	neural cell adhesion molecule 1	---		693789	MmugDNA.17876.1.S1_at_NCAM1
MmugDNA.17877.1.S1_at	NA	NA	NA	NA	NA		NA	NA	NA	NA		MmugDNA.17877.1.S1_at_NA
MmugDNA.17877.1.S1_s_at	NA	NA	NA	NA	NA		NA	NA	NA	NA		MmugDNA.17877.1.S1_s_at_NA
MmugDNA.17878.1.S1_at	228606_at	TCTEX1D2	Tctex1 domain containing 2	chr3q29		255758	LOC711098	similar to CG5359-PA	---		711098	MmugDNA.17878.1.S1_at_TCTEX1D2
MmugDNA.17879.1.S1_at	238035_at	SP3	Sp3 transcription factor	chr2q31		6670	SP3	Sp3 transcription factor	---		699901	MmugDNA.17879.1.S1_at_SP3
MmugDNA.17880.1.S1_at	NA	NA	NA	NA	NA		NA	NA	NA	NA		MmugDNA.17880.1.S1_at_NA
MmugDNA.17880.1.S1_s_at	NA	NA	NA	NA	NA		NA	NA	NA	NA		MmugDNA.17880.1.S1_s_at_NA
MmugDNA.17881.1.S1_at	221697_at	MAP1LC3C	microtubule-associated protein 1 light chain 3 gamma	chr1q43		440738	MAP1LC3B2 /// MAP1LC	similar to microtubule-associated protein 1 light chain 3 gamma /// microtubule-associated protein 1 li	---	706487 /// 707563		MmugDNA.17881.1.S1_at_MAP1LC3C
MmugDNA.1788.1.S1_at	231852_at	ERI1	exoribonuclease 1	chr8p23.1		90459	---	---	---	---		MmugDNA.1788.1.S1_at_ERI1
MmugDNA.17882.1.S1_at	241194_at	---	---	---	---		---	---	---	---		MmugDNA.17882.1.S1_at_---
MmugDNA.17883.1.S1_at	1564021_at	LOC100130456	hypothetical LOC100130456	chr7p22.3		100130456	---	---	---	---		MmugDNA.17883.1.S1_at_LOC100130(

MmugDNA.17884.1.S1_at	204652_s_at	NRF1	nuclear respiratory factor 1	chr7q32	4899	LOC701933	similar to Nuclear respiratory factor 1 (NRF-1) (Alpha palindromic-binding protein) (Alpha-pal)	---	701933	MmugDNA.17884.1.S1_at_NRF1
MmugDNA.17885.1.S1_at	222062_at	IL27RA	interleukin 27 receptor, alpha	chr19p13.11	9466	LOC717549	similar to class I cytokine receptor	---	717549	MmugDNA.17885.1.S1_at_IL27RA
MmugDNA.17886.1.S1_at	1559807_at	---	---	---	---	---	---	---	---	MmugDNA.17886.1.S1_at_---
MmugDNA.17887.1.S1_s_at	203675_at	NUCB2	nucleobindin 2	chr11p15.1-p14	4925	NUCB2	nucleobindin 2	---	697084	MmugDNA.17887.1.S1_s_at_NUCB2
MmugDNA.17888.1.S1_at	221390_s_at	MTMR8	myotubularin related protein 8	chrXq11.1	55613	MTMR9	myotubularin related protein 9	---	708037	MmugDNA.17888.1.S1_at_MTMR8
MmugDNA.17889.1.S1_at	208337_s_at	NR5A2	nuclear receptor subfamily 5, group A, member 2	chr1q32.1	2494	LOC709735	similar to nuclear receptor subfamily 5, group A, member 2 isoform 2	---	709735	MmugDNA.17889.1.S1_at_NR5A2
MmugDNA.17890.1.S1_at	1553141_at	C13orf31	chromosome 13 open reading frame 31	chr13q14.11	144811	C17H13orf31	chromosome 13 open reading frame 31 ortholog	---	700834	MmugDNA.17890.1.S1_at_C13orf31
MmugDNA.17891.1.S1_at	201736_s_at	MARCH6	membrane-associated ring finger (C3HC4) 6	chr5p15.2	10299	---	---	---	---	MmugDNA.17891.1.S1_at_MARCH6
MmugDNA.1789.1.S1_at	239464_at	---	---	---	---	---	---	---	---	MmugDNA.1789.1.S1_at_---
MmugDNA.17892.1.S1_at	1553142_at	C13orf31	chromosome 13 open reading frame 31	chr13q14.11	144811	---	---	---	---	MmugDNA.17892.1.S1_at_C13orf31
MmugDNA.17893.1.S1_at	217733_s_at	TMSB10	thymosin beta 10	chr2p11.2	9168	LOC694305	similar to thymosin, beta 10	---	694305	MmugDNA.17893.1.S1_at_TMSB10
MmugDNA.17894.1.S1_at	1555680_a_at	SMOX	spermine oxidase	chr20p13	54498	LOC718761	similar to polyamine oxidase isoform 4	---	718761	MmugDNA.17894.1.S1_at_SMOX
MmugDNA.17895.1.S1_at	240713_s_at	---	---	---	---	---	---	---	---	MmugDNA.17895.1.S1_at_---
MmugDNA.17896.1.S1_s_at	211013_x_at	PML	promyelocytic leukemia	chr15q22	5371	PML	promyelocytic leukemia	---	700379	MmugDNA.17896.1.S1_s_at_PML
MmugDNA.17897.1.S1_at	237732_at	---	---	---	---	---	---	---	---	MmugDNA.17897.1.S1_at_---
MmugDNA.17898.1.S1_at	242240_at	---	---	---	---	---	---	---	---	MmugDNA.17898.1.S1_at_---
MmugDNA.17899.1.S1_at	227085_at	H2AFV	H2A histone family, member V	chr7p13	94239	---	---	---	---	MmugDNA.17899.1.S1_at_H2AFV
MmugDNA.17900.1.S1_at	1558078_at	---	---	---	---	---	---	---	---	MmugDNA.17900.1.S1_at_---
MmugDNA.17901.1.S1_at	241479_at	---	---	---	---	---	---	---	---	MmugDNA.17901.1.S1_at_---
MmugDNA.1790.1.S1_at	214610_at	CYP11B1	cytochrome P450, family 11, subfamily B, polypeptide 1	chr8q21	1584	LOC695793	similar to cytochrome P450, family 11, subfamily B, polypeptide 1 isoform 1 precursor	---	695793	MmugDNA.1790.1.S1_at_CYP11B1
MmugDNA.17902.1.S1_at	217476_at	NR1D1 /// THRA	nuclear receptor subfamily 1, group D, member 1 /// thyroid hormone receptor, alpha (erythroblastic le	chr17q11.2	7067 /// 9572	NR1D1	nuclear receptor subfamily 1, group D, member 1	---	699472	MmugDNA.17902.1.S1_at_NR1D1 /// TH
MmugDNA.17903.1.S1_at	244213_at	---	---	---	---	---	---	---	---	MmugDNA.17903.1.S1_at_---
MmugDNA.17904.1.S1_at	1552482_at	RAPH1	Ras association (RalGDS/AF-6) and pleckstrin homology domains 1	chr2q33	65059	LOC705065	similar to Ras association and pleckstrin homology domains 1 isoform 1	---	705065	MmugDNA.17904.1.S1_at_RAPH1
MmugDNA.17905.1.S1_at	235095_at	CCDC64B	coiled-coil domain containing 64B	chr16p13.3	146439	CCDC64B	coiled-coil domain containing 64B	---	700092	MmugDNA.17905.1.S1_at_CCDC64B
MmugDNA.17906.1.S1_at	202097_at	NUP153	nucleoporin 153kDa	chr6p22.3	9972	LOC702648	Hypothetical protein LOC702648	---	702648	MmugDNA.17906.1.S1_at_NUP153
MmugDNA.17907.1.S1_at	202155_s_at	NUP214	nucleoporin 214kDa	chr9q34.1	8021	---	---	---	---	MmugDNA.17907.1.S1_at_NUP214
MmugDNA.17907.1.S1_s_at	NA	NA	NA	NA	NA	NA	NA	NA	NA	MmugDNA.17907.1.S1_s_at_NA
MmugDNA.17908.1.S1_at	200894_s_at	FKBP4	FK506 binding protein 4, 59kDa	chr12p13.33	2288	FKBP4	FK506 binding protein 4, 59kDa	---	708481	MmugDNA.17908.1.S1_at_FKBP4
MmugDNA.17909.1.S1_at	205908_s_at	OMD	osteomodulin	chr9q22.31	4958	OMD	osteomodulin	---	706272	MmugDNA.17909.1.S1_at_OMD
MmugDNA.17910.1.S1_at	202054_s_at	ALDH3A2	aldehyde dehydrogenase 3 family, member A2	chr17p11.2	224	ALDH3A2	aldehyde dehydrogenase 3 family, member A2	---	703661	MmugDNA.17910.1.S1_at_ALDH3A2
MmugDNA.17911.1.S1_at	NA	NA	NA	NA	NA	NA	NA	NA	NA	MmugDNA.17911.1.S1_at_NA
MmugDNA.1791.1.S1_at	1562918_at	---	---	---	---	---	---	---	---	MmugDNA.1791.1.S1_at_---
MmugDNA.17912.1.S1_at	240915_at	IGHG1	Immunoglobulin heavy constant gamma 1 (G1m marker)	chr14q32.33	3500	LOC721043	similar to Ig heavy chain V-I region HG3 precursor	---	721043	MmugDNA.17912.1.S1_at_IGHG1
MmugDNA.17913.1.S1_at	228580_at	HTRA3	HtrA serine peptidase 3	chr4p16.1	94031	---	---	---	---	MmugDNA.17913.1.S1_at_HTRA3
MmugDNA.17914.1.S1_at	231417_at	---	---	---	---	---	---	---	---	MmugDNA.17914.1.S1_at_---
MmugDNA.17915.1.S1_at	204705_x_at	ALDOB	aldolase B, fructose-bisphosphate	chr9q21.3-q22.2	229	ALDOB	aldolase B	---	713818	MmugDNA.17915.1.S1_at_ALDOB
MmugDNA.17916.1.S1_at	209744_x_at	ITCH	itchy E3 ubiquitin protein ligase homolog (mouse)	chr20q11.22-q11.23	83737	LOC709524	Similar to Aldose reductase (AR) (Aldehyde reductase)	---	709524	MmugDNA.17916.1.S1_at_ITCH
MmugDNA.17918.1.S1_at	232084_at	SGTB	small glutamine-rich tetratricopeptide repeat (TPR)-containing, beta	chr5q12.3	54557	SGTB	small glutamine-rich tetratricopeptide repeat (TPR)-containing, beta	---	696763	MmugDNA.17918.1.S1_at_SGTB
MmugDNA.17919.1.S1_at	206714_at	ALOX15B	arachidonate 15-lipoxygenase, type B	chr17p13.1	247	ALOX15B	arachidonate 15-lipoxygenase, type B	---	716764	MmugDNA.17919.1.S1_at_ALOX15B
MmugDNA.179.1.S1_at	200667_at	UBE2D3	ubiquitin-conjugating enzyme E2D 3 (UBC4/5 homolog, yeast)	chr4q24	7323	LOC710772	similar to ubiquitin-conjugating enzyme E2D 3 (UBC4/5 homolog, yeast)	---	710772	MmugDNA.179.1.S1_at_UBE2D3
MmugDNA.17920.1.S1_at	222763_s_at	WDR33	WD repeat domain 33	chr2q14.3	55339	WDR33	WD repeat domain 33	---	699696	MmugDNA.17920.1.S1_at_WDR33
MmugDNA.17921.1.S1_at	235246_at	---	---	---	---	---	---	---	---	MmugDNA.17921.1.S1_at_---
MmugDNA.1792.1.S1_at	1555226_s_at	C1orf43	chromosome 1 open reading frame 43	chr1q21.2	25912	LOC716453	similar to Protein C1orf43 (Hepatitis C virus NS5A-transactivated protein 4) (Protein NICE-3) (S863-3	---	716453	MmugDNA.1792.1.S1_at_C1orf43
MmugDNA.17922.1.S1_at	217413_s_at	TNXB	tenascin XB	chr6p21.3	7148	LOC716998	similar to tenascin XB isoform 1	---	716998	MmugDNA.17922.1.S1_at_TNXB
MmugDNA.17923.1.S1_at	231485_at	---	---	---	---	---	---	---	---	MmugDNA.17923.1.S1_at_---
MmugDNA.17924.1.S1_at	1556580_a_at	---	---	---	---	---	---	---	---	MmugDNA.17924.1.S1_at_---
MmugDNA.17925.1.S1_at	213269_at	ZNF248	zinc finger protein 248	---	57209	ZNF248	zinc finger protein 248	---	700749	MmugDNA.17925.1.S1_at_ZNF248
MmugDNA.17926.1.S1_at	1561266_at	---	---	---	---	---	---	---	---	MmugDNA.17926.1.S1_at_---
MmugDNA.17927.1.S1_at	234189_at	---	---	---	---	---	---	---	---	MmugDNA.17927.1.S1_at_---
MmugDNA.17928.1.S1_at	227805_at	---	---	---	---	---	---	---	---	MmugDNA.17928.1.S1_at_---
MmugDNA.17929.1.S1_at	1558467_a_at	---	---	---	---	---	---	---	---	MmugDNA.17929.1.S1_at_---
MmugDNA.17930.1.S1_at	1556958_at	---	---	---	---	---	---	---	---	MmugDNA.17930.1.S1_at_---
MmugDNA.17931.1.S1_at	225672_at	GOLGA2	golgi autoantigen, golgin subfamily a, 2	chr9q34.11	2801	LOC717677	similar to Golgi autoantigen, golgin subfamily a, 2	---	717677	MmugDNA.17931.1.S1_at_GOLGA2
MmugDNA.1793.1.S1_at	230177_at	---	---	---	---	---	---	---	---	MmugDNA.1793.1.S1_at_---
MmugDNA.17932.1.S1_at	215306_at	---	---	---	---	---	---	---	---	MmugDNA.17932.1.S1_at_---
MmugDNA.17933.1.S1_at	243817_at	---	---	---	---	---	---	---	---	MmugDNA.17933.1.S1_at_---
MmugDNA.17934.1.S1_at	226582_at	LOC400043	hypothetical LOC400043	chr12q13.13	400043	---	---	---	---	MmugDNA.17934.1.S1_at_LOC400043
MmugDNA.17935.1.S1_at	213455_at	FAM114A1	family with sequence similarity 114, member A1	chr4p14	92689	---	---	---	---	MmugDNA.17935.1.S1_at_FAM114A1
MmugDNA.17936.1.S1_at	1563724_at	---	---	---	---	---	---	---	---	MmugDNA.17936.1.S1_at_---
MmugDNA.17937.1.S1_at	235467_s_at	KCNC4	potassium voltage-gated channel, Shaw-related subfamily, member 4	chr1p21	3749	LOC701275	similar to Shaw-related voltage-gated potassium channel protein 4 isoform a	---	701275	MmugDNA.17937.1.S1_at_KCNC4
MmugDNA.17938.1.S1_at	206214_at	PLA2G7	phospholipase A2, group VII (platelet-activating factor acetylhydrolase, plasma)	chr6p21.2-p12	7941	PLA2G7	phospholipase A2, group VII	---	704566	MmugDNA.17938.1.S1_at_PLA2G7
MmugDNA.17939.1.S1_at	206121_at	AMPD1	adenosine monophosphate deaminase 1 (isoform M)	chr1p13	270	AMPD1	adenosine monophosphate deaminase 1 (isoform M)	---	708801	MmugDNA.17939.1.S1_at_AMPD1
MmugDNA.17940.1.S1_at	204031_s_at	PCBP2	poly(rC) binding protein 2	chr12q13.12-q13.13	5094	LOC694744	similar to poly(rC) binding protein 2 (predicted)	---	694744	MmugDNA.17940.1.S1_at_PCBP2
MmugDNA.17941.1.S1_at	205141_at	ANG	angiogenin, ribonuclease, RNase A family, 5	chr14q11.1-q11.2	283	RNASE4	ribonuclease, RNase A family, 4	---	703903	MmugDNA.17941.1.S1_at_ANG
MmugDNA.1794.1.S1_at	1569838_at	---	---	---	---	---	---	---	---	MmugDNA.1794.1.S1_at_---
MmugDNA.17942.1.S1_at	224396_s_at	ASPN	asporin	chr9q22	54829	LOC706404	Similar to anillin, actin binding protein (scraps homolog, Drosophila)	---	706404	MmugDNA.17942.1.S1_at_ASPN
MmugDNA.17943.1.S1_at	228762_at	LFNG	LFNG O-fucosylpeptide 3-beta-N-acetylglucosaminyltransferase	chr7p22.2	3955	---	---	---	---	MmugDNA.17943.1.S1_at_LFNG
MmugDNA.17944.1.S1_at	204210_s_at	PCYT1A	phosphate cytidylyltransferase 1, choline, alpha	chr3q29	5130	---	---	---	---	MmugDNA.17944.1.S1_at_PCYT1A
MmugDNA.17945.1.S1_at	206200_s_at	ANXA11	annexin A11	chr10q23	311	LOC702407	Hypothetical protein LOC702407	---	702407	MmugDNA.17945.1.S1_at_ANXA11
MmugDNA.17946.1.S1_s_at	204741_at	BICD1	bicaudal D homolog 1 (Drosophila)	chr12p11.2-p11.1	636	LOC695170	similar to bicaudal D homolog 1 isoform 1	---	695170	MmugDNA.17946.1.S1_s_at_BICD1
MmugDNA.17947.1.S1_at	NA	NA	NA	NA	NA	NA	NA	NA	NA	MmugDNA.17947.1.S1_at_NA
MmugDNA.17948.1.S1_at	1562844_at	LOC339822	hypothetical protein LOC339822	chr2p25.3	339822	---	---	---	---	MmugDNA.17948.1.S1_at_LOC339822
MmugDNA.17949.1.S1_at	1564878_at	---	---	---	---	---	---	---	---	MmugDNA.17949.1.S1_at_---
MmugDNA.17950.1.S1_s_at	204603_at	EXO1	exonuclease 1	chr1q42-q43	9156	EXO1	exonuclease 1	---	706902	MmugDNA.17950.1.S1_s_at_EXO1
MmugDNA.17951.1.S1_at	222908_at	FAM38B	family with sequence similarity 38, member B	chr18p11.22	63895	LOC722206	similar to Protein FAM38B	---	722206	MmugDNA.17951.1.S1_at_FAM38B
MmugDNA.1795.1.S1_at	221424_s_at	OR51E2	olfactory receptor, family 51, subfamily E, member 2	chr11p15	81285	LOC717504	similar to olfactory receptor, family 51, subfamily E, member 2	---	717504	MmugDNA.1795.1.S1_at_OR51E2
MmugDNA.17952.1.S1_at	207445_s_at	CCR9	chemokine (C-C motif) receptor 9	chr3p21.3	10803	---	---	---	---	MmugDNA.17952.1.S1_at_CCR9
MmugDNA.17953.1.S1_at	1557527_at	---	---	---	---	---	---	---	---	MmugDNA.17953.1.S1_at_---
MmugDNA.17954.1.S1_at	244151_at	LOC285733	hypothetical LOC285733	chr6q23.1	285733	LOC710673	hypothetical protein LOC710673	---	710673	MmugDNA.17954.1.S1_at_LOC285733
MmugDNA.17955.1.S1_at	1558306_at	THADA	thyroid adenoma associated	chr2p21	63892	---	---	---	---	MmugDNA.17955.1.S1_at_THADA
MmugDNA.17957.1.S1_at	1560460_at	---	---	---	---	---	---	---	---	MmugDNA.17957.1.S1_at_---
MmugDNA.17958.1.S1_at	208396_s_at	PDE1A	phosphodiesterase 1A, calmodulin-dependent	chr2q32.1	5136	LOC705555	similar to phosphodiesterase 1A, calmodulin-dependent isoform 1	---	705555	MmugDNA.17958.1.S1_at_PDE1A
MmugDNA.17959.1.S1_at	228755_at	GIGYF1	GRB10 interacting GYF protein 1	chr7q22	64599	LOC712775	similar to PERQ amino acid rich, with GYF domain 1	---	712775	MmugDNA.17959.1.S1_at_GIGYF1
MmugDNA.17961.1.S1_at	224685_at	MLLT4	myeloid/lymphoid or mixed-lineage leukemia (trithorax homolog, Drosophila); translocated to, 4	chr6q27	4301	MLLT4	myeloid/lymphoid or mixed-lineage leukemia (trithorax homolog, Drosophila); translocated to, 4	---	693560	MmugDNA.17961.1.S1_at_MLLT4
MmugDNA.1796.1.S1_at	1559879_a_at	---	---	---	---	---	---	---	---	MmugDNA.1796.1.S1_at_---
MmugDNA.17962.1.S1_at	1554170_a_at	MBLAC1	metallo-beta-lactamase domain containing 1	chr7q22.1	255374	LOC711102	similar to B0432.9	---	711102	MmugDNA.17962.1.S1_at_MBLAC1
MmugDNA.17963.1.S1_at	219912_s_at	---	---	---	---	---	---	---	---	MmugDNA.17963.1.S1_at_---
MmugDNA.17964.1.S1_at	NA	NA	NA	NA	NA	NA	NA	NA	NA	MmugDNA.17964.1.S1_at_NA
MmugDNA.17964.1.S1_s_at	205820_s_at	APOC3	apolipoprotein C-III	chr11q23.1-q23.2	345	APOC3	apolipoprotein C-III	---	696726	MmugDNA.17964.1.S1_s_at_APOC3
MmugDNA.17965.1.S1_at	200634_at	PFN1	profilin 1	chr17p13.3	5216	PFN1	profilin 1	---	710753	MmugDNA.17965.1.S1_at_PFN1
MmugDNA.17966.1.S1_x_at	203382_s_at	APOE	apolipoprotein E	chr19q13.2	348	APOE	Apolipoprotein E	---	714623	MmugDNA.17966.1.S1_x_at_APOE
MmugDNA.17967.1.S1_at	231202_at	ALDH1L2	Aldehyde dehydrogenase 1 family, member L2	chr12q23.3	160428	ALDH1L2	aldehyde dehydrogenase 1 family, member L2	---	701242	MmugDNA.17967.1.S1_at_ALDH1L2
MmugDNA.17968.1.S1_at	206288_at	PGGT1B	protein geranylgeranyltransferase type I, beta subunit	chr5q22.3	5229	LOC696437	similar to Geranylgeranyl transferase type-1 beta subunit (Geranylgeranyl transferase type I beta sub	---	696437	MmugDNA.17968.1.S1_at_PGGT1B
MmugDNA.17969.1.S1_at	205216_s_at	APOH	apolipoprotein H (beta-2-glycoprotein I)	chr17q23-qter	350	APOH /// APOH	similar to Beta-2-glycoprotein 1 precursor (Beta-2-glycoprotein I) (Apolipoprotein H) (Apo-H) (B2GPI)	---	695967 /// 718645	MmugDNA.17969.1.S1_at_APOH
MmugDNA.17969.1.S1_s_at	NA	NA	NA	NA	NA	NA	NA	NA	NA	MmugDNA.17969.1.S1_s_at_NA
MmugDNA.17970.1.S1_at	230851_x_at	C16orf13	Chromosome 16 open reading frame 13	chr16p13.3	84326	LOC696495	similar to CG18661-PA	---	696495	MmugDNA.17970.1.S1_at_C16orf13
MmugDNA.17971.1.S1_at	1559603_at	GPR12	G protein-coupled receptor 12	chr13q12	2835	GPR12	G protein-coupled receptor 12	---	710030	MmugDNA.17971.1.S1_at_GPR12
MmugDNA.1797.1.S1_at	226341_at	---	---	---	---	---	---	---	---	MmugDNA.1797.1.S1_at_---
MmugDNA.17972.1.S1_at	216465_at	---	---	---	---	---	---	---	---	MmugDNA.17972.1.S1_at_---
MmugDNA.17973.1.S1_s_at	206087_x_at	HFE	hemochromatosis	chr6p21.3	3077	---	---	---	---	MmugDNA.17973.1.S1_s_at_HFE
MmugDNA.17974.1.S1_at	1553247_a_at	ZNF709	zinc finger protein 709	chr19p13.2	163051	LOC717515	similar to zinc finger protein 709	---	717515	MmugDNA.17974.1.S1_at_ZNF709
MmugDNA.17975.1.S1_s_at	226462_at	STXBP6	syntaxin binding protein 6 (amisyn)	chr14q12	29091	LOC715357	similar to amisyn	---	715357	MmugDNA.17975.1.S1_s_at_STXBP6
MmugDNA.17976.1.S1_at	230810_at	JMJD4	jumonji domain containing 4	chr1q42.13	65094	---	---	---	---	MmugDNA.17976.1.S1_at_JMJD4
MmugDNA.17977.1.S1_at	228547_at	NRXN1	neurexin 1	chr2p16.3	9378	EP300	E1A binding protein p300	---	706258	MmugDNA.17977.1.S1_at_NRXN1
MmugDNA.17978.1.S1_at	215761_at	DMXL2	Dmx-like 2	chr15q21.2	23312	---	---	---	---	MmugDNA.17978.1.S1_at_DMXL2
MmugDNA.17979.1.S1_at	226187_at	CDS1	CDP-diacylglycerol synthase (phosphatidate cytidylyltransferase) 1	chr4q21.23	1040	---	---	---	---	MmugDNA.17979.1.S1_at_CDS1
MmugDNA.17980.1.S1_at	226185_at	CDS1	CDP-diacylglycerol synthase (phosphatidate cytidylyltransferase) 1	chr4q21.23	1040	---	---	---	---	MmugDNA.17980.1.S1_at_CDS1
MmugDNA.17981.1.S1_at	211360_s_at	ITPR2	inositol 1,4,5-triphosphate receptor, type 2	chr12p11	3709	ITPR2	inositol 1,4,5-triphosphate receptor, type 2	---	708714	MmugDNA.17981.1.S1_at_ITPR2

MmugDNA.1798.1.S1_at	232399_at	DCLK3	doublecortin-like kinase 3	chr3p22.2		85443	DCLK3	doublecortin-like kinase 3	---		699654	MmugDNA.1798.1.S1_at_DCLK3
MmugDNA.17982.1.S1_at	229502_at	CHDH	choline dehydrogenase	chr3p21.1		55349	CHDH	choline dehydrogenase	---		693805	MmugDNA.17982.1.S1_at_CHDH
MmugDNA.17983.1.S1_at	215831_at	---	---	---	---		---	---	---	---		MmugDNA.17983.1.S1_at_---
MmugDNA.17984.1.S1_at	205734_s_at	AFF3	AF4/FMR2 family, member 3	chr2q11.2-q12		3899	LOC714408	similar to AF4/FMR2 family, member 3 isoform 2	---		714408	MmugDNA.17984.1.S1_at_AFF3
MmugDNA.17985.1.S1_at	225274_at	PCYOX1	prenylcysteine oxidase 1	chr2p13.3		51449	---	---	---	---		MmugDNA.17985.1.S1_at_PCYOX1
MmugDNA.17986.1.S1_at	NA	NA	NA	NA	NA		NA	NA	NA	NA		MmugDNA.17986.1.S1_at_NA
MmugDNA.17986.1.S1_s_at	244050_at	PTPLAD2	protein tyrosine phosphatase-like A domain containing 2	chr9p21.3		401494	LOC711051	similar to CG9267-PA	---		711051	MmugDNA.17986.1.S1_s_at_PTPLAD2
MmugDNA.17987.1.S1_at	243424_at	---	---	---	---		---	---	---	---		MmugDNA.17987.1.S1_at_---
MmugDNA.17988.1.S1_at	217677_at	PLEKHA2	pleckstrin homology domain containing, family A (phosphoinositide binding specific) member 2	chr8p11.23		59339	---	---	---	---		MmugDNA.17988.1.S1_at_PLEKHA2
MmugDNA.17990.1.S1_at	NA	NA	NA	NA	NA		NA	NA	NA	NA		MmugDNA.17990.1.S1_at_NA
MmugDNA.17991.1.S1_at	205807_s_at	TUFT1	tuftelin 1	chr1q21		7286	---	---	---	---		MmugDNA.17991.1.S1_at_TUFT1
MmugDNA.1799.1.S1_at	230796_at	LOC440900	hypothetical LOC440900	chr2q14.1		440900	---	---	---	---		MmugDNA.1799.1.S1_at_LOC440900
MmugDNA.17992.1.S1_at	206030_at	ASPA	aspartoacylase (Canavan disease)	chr17pter-p13		443	ASPA	aspartoacylase	---		706170	MmugDNA.17992.1.S1_at_ASPA
MmugDNA.17993.1.S1_at	1564868_a_at	FAM117B	family with sequence similarity 117, member B	chr2q33.1		150864	LOC705134	similar to amyotrophic lateral sclerosis 2 (juvenile) chromosome region, candidate 13	---		705134	MmugDNA.17993.1.S1_at_FAM117B
MmugDNA.17994.1.S1_at	205221_at	HGD	homogentisate 1,2-dioxygenase (homogentisate oxidase)	chr3q13.33		3081	HGD	homogentisate 1,2-dioxygenase	---		713499	MmugDNA.17994.1.S1_at_HGD
MmugDNA.17995.1.S1_at	209643_s_at	PLD2	phospholipase D2	chr17p13.1		5338	PLD2	phospholipase D2	---		721573	MmugDNA.17995.1.S1_at_PLD2
MmugDNA.17996.1.S1_at	207027_at	HGFAC	HGF activator	chr4p16		3083	LOC719952	similar to HGF activator preproprotein	---		719952	MmugDNA.17996.1.S1_at_HGFAC
MmugDNA.17997.1.S1_at	207546_at	ATP4B	ATPase, H+/K+ exchanging, beta polypeptide	chr13q34		496	ATP4B	ATPase, H+/K+ exchanging, beta polypeptide	---		698222	MmugDNA.17997.1.S1_at_ATP4B
MmugDNA.17998.1.S1_at	200989_at	HIF1A	hypoxia inducible factor 1, alpha subunit (basic helix-loop-helix transcription factor)	chr14q21-q24		3091	LOC695964	Similar to 40S ribosomal protein S3a (V-fos transformation effector protein)	---		695964	MmugDNA.17998.1.S1_at_HIF1A
MmugDNA.17999.1.S1_at	211263_s_at	PCSK6	proprotein convertase subtilisin/kexin type 6	chr15q26.3		5046	---	---	---	---		MmugDNA.17999.1.S1_at_PCSK6
MmugDNA.18000.1.S1_at	1568807_a_at	---	---	---	---		---	---	---	---		MmugDNA.18000.1.S1_at_---
MmugDNA.18001.1.S1_at	236029_at	FAT3	FAT tumor suppressor homolog 3 (Drosophila)	chr11q14.3		120114	FAT3	FAT tumor suppressor homolog 3 (Drosophila)	---		695935	MmugDNA.18001.1.S1_at_FAT3
MmugDNA.1800.1.S1_at	227070_at	GLT8D2	glycosyltransferase 8 domain containing 2	chr12q		83468	GLT8D2	glycosyltransferase 8 domain containing 2	---		699937	MmugDNA.1800.1.S1_at_GLT8D2
MmugDNA.18002.1.S1_at	230882_at	FLJ34048	hypothetical transcript	chr7q21.3		285987	---	---	---	---		MmugDNA.18002.1.S1_at_FLJ34048
MmugDNA.18003.1.S1_at	227632_at	TBC1D24	TBC1 domain family, member 24	chr16p13.3		57465	TBC1D24	TBC1 domain family, member 24	---		697106	MmugDNA.18003.1.S1_at_TBC1D24
MmugDNA.18004.1.S1_at	227225_at	---	---	---		439985	---	---	---	---		MmugDNA.18004.1.S1_at_---
MmugDNA.18006.1.S1_at	204274_at	EBAG9	estrogen receptor binding site associated, antigen, 9	chr8q23		9166	EBAG9	estrogen receptor binding site associated, antigen, 9	---		699305	MmugDNA.18006.1.S1_at_EBAG9
MmugDNA.18007.1.S1_at	NA	NA	NA	NA	NA		NA	NA	NA	NA		MmugDNA.18007.1.S1_at_NA
MmugDNA.18007.1.S1_s_at	226568_at	FAM102B	family with sequence similarity 102, member B	chr1p13.3		284611	---	---	---	---		MmugDNA.18007.1.S1_s_at_FAM102B
MmugDNA.18008.1.S1_at	1556822_s_at	ZNF837	Zinc finger protein 837	chr19q13.43		116412	---	---	---	---		MmugDNA.18008.1.S1_at_ZNF837
MmugDNA.18009.1.S1_at	230752_at	---	---	---	---		---	---	---	---		MmugDNA.18009.1.S1_at_---
MmugDNA.18010.1.S1_at	210237_at	ARTN	artemin	chr1p33-p32		9048	ARTN	artemin	---		701641	MmugDNA.18010.1.S1_at_ARTN
MmugDNA.18011.1.S1_at	229017_s_at	DSTYK	dual serine/threonine and tyrosine protein kinase	chr1q32.1		25778	DSTYK	dual serine/threonine and tyrosine protein kinase	---		664732	MmugDNA.18011.1.S1_at_DSTYK
MmugDNA.1801.1.S1_at	232216_at	YME1L1	YME1-like 1 (S. cerevisiae)	chr10p14		10730	---	---	---	---		MmugDNA.1801.1.S1_at_YME1L1
MmugDNA.18012.1.S1_at	1562081_a_at	---	---	---	---		---	---	---	---		MmugDNA.18012.1.S1_at_---
MmugDNA.18013.1.S1_at	205936_s_at	HK3	hexokinase 3 (white cell)	chr5q35.2		3101	HK3	hexokinase 3	---		698120	MmugDNA.18013.1.S1_at_HK3
MmugDNA.18014.1.S1_at	240515_at	---	---	---	---		---	---	---	---		MmugDNA.18014.1.S1_at_---
MmugDNA.18015.1.S1_at	203932_at	HLA-DMB	major histocompatibility complex, class II, DM beta	chr6p21.3		3109	LOC717870	similar to HLA class II histocompatibility antigen, DM beta chain precursor (MHC class II antigen DMB	---		717870	MmugDNA.18015.1.S1_at_HLA-DMB
MmugDNA.18016.1.S1_at	228683_s_at	KCTD15	potassium channel tetramerisation domain containing 15	chr19q13.11		79047	LOC704761	similar to potassium channel tetramerisation domain containing 15	---		704761	MmugDNA.18016.1.S1_at_KCTD15
MmugDNA.18017.1.S1_at	1554747_a_at	SEPT2	septin 2	chr2q37		4735	SEPT2	septin 2	---		717682	MmugDNA.18017.1.S1_at_SEPT2
MmugDNA.18018.1.S1_at	239186_at	MGC39372	hypothetical protein MGC39372	chr6p25.2		221756	---	---	---	---		MmugDNA.18018.1.S1_at_MGC39372
MmugDNA.18019.1.S1_at	215487_x_at	FAM182B	family with sequence similarity 182, member B	chr20p11.1		728882	---	---	---	---		MmugDNA.18019.1.S1_at_FAM182B
MmugDNA.180.1.S1_at	221464_at	OR1D2	olfactory receptor, family 1, subfamily D, member 2	chr17p13-p12|17p13.3		4991	LOC721417	similar to olfactory receptor, family 1, subfamily D, member 2	---		721417	MmugDNA.180.1.S1_at_OR1D2
MmugDNA.18020.1.S1_at	242205_at	---	---	---	---		---	---	---	---		MmugDNA.18020.1.S1_at_---
MmugDNA.18021.1.S1_at	241875_at	---	---	---	---		---	---	---	---		MmugDNA.18021.1.S1_at_---
MmugDNA.1802.1.S1_at	206753_at	RDH16	retinol dehydrogenase 16 (all-trans)	chr12q13.3		8608	LOC713672	similar to microsomal NAD+-dependent retinol dehydrogenase 4	---		713672	MmugDNA.1802.1.S1_at_RDH16
MmugDNA.18022.1.S1_at	235719_at	CYP4V2	cytochrome P450, family 4, subfamily V, polypeptide 2	chr4q35.2		285440	---	---	---	---		MmugDNA.18022.1.S1_at_CYP4V2
MmugDNA.18023.1.S1_at	234954_at	---	---	---	---		LOC712109	similar to Glyceraldehyde-3-phosphate dehydrogenase (GAPDH)	---		712109	MmugDNA.18023.1.S1_at_---
MmugDNA.18024.1.S1_at	213888_s_at	LOC100133233 /// TRA	hypothetical protein LOC100133233 /// TRAF3 interacting protein 3	chr1q32.2 /// chr1q32.3-q41	100133233 /// 80342		LOC712895	similar to TRAF3-interacting JNK-activating modulator	---		712895	MmugDNA.18024.1.S1_at_LOC1001332
MmugDNA.18025.1.S1_at	1559471_s_at	D21S2088E	D21S2088E	---		266917	---	---	---	---		MmugDNA.18025.1.S1_at_D21S2088E
MmugDNA.18026.1.S1_at	223700_at	MND1	meiotic nuclear divisions 1 homolog (S. cerevisiae)	chr4q31.3		84057	---	---	---	---		MmugDNA.18026.1.S1_at_MND1
MmugDNA.18027.1.S1_at	242078_at	---	---	---	---		---	---	---	---		MmugDNA.18027.1.S1_at_---
MmugDNA.18028.1.S1_at	211673_s_at	MOCS1	molybdenum cofactor synthesis 1	chr6p21.3		4337	LOC719625	similar to molybdenum cofactor synthesis-step 1 protein isoform 2	---		719625	MmugDNA.18028.1.S1_at_MOCS1
MmugDNA.18029.1.S1_at	207004_at	BCL2	B-cell CLL/lymphoma 2	chr18q21.33|18q21.3		596	LOC707407	similar to B-cell lymphoma protein 2 beta isoform	---		707407	MmugDNA.18029.1.S1_at_BCL2
MmugDNA.18030.1.S1_at	232762_at	KIAA1217	KIAA1217	chr10p12.2-p12.1		56243	LOC705851	similar to p130Cas-associated protein (p140Cap) (SNAP-25-interacting protein) (SNIP)	---		705851	MmugDNA.18030.1.S1_at_KIAA1217
MmugDNA.18031.1.S1_at	205870_at	BDKRB2	bradykinin receptor B2	chr14q32.1-q32.2		624	BDKRB2	bradykinin receptor B2	---		703698	MmugDNA.18031.1.S1_at_BDKRB2
MmugDNA.1803.1.S1_at	243432_at	LOC642891	hypothetical LOC642891	chr3p26.3		642891	---	---	---	---		MmugDNA.1803.1.S1_at_LOC642891
MmugDNA.18032.1.S1_at	235075_at	DSG3	desmoglein 3 (pemphigus vulgaris antigen)	chr18q12.1-q12.2		1830	---	---	---	---		MmugDNA.18032.1.S1_at_DSG3
MmugDNA.18033.1.S1_at	233155_at	UPP2	uridine phosphorylase 2	chr2q24.1		151531	LOC698067	similar to uridine phosphorylase 2	---		698067	MmugDNA.18033.1.S1_at_UPP2
MmugDNA.18034.1.S1_at	243295_at	RBM27	RNA binding motif protein 27	chr5q32		54439	RBM27	RNA binding motif protein 27	---		707688	MmugDNA.18034.1.S1_at_RBM27
MmugDNA.18035.1.S1_at	1563241_at	---	---	---	---		---	---	---	---		MmugDNA.18035.1.S1_at_---
MmugDNA.18036.1.S1_at	1561247_at	LOC283682	Hypothetical protein LOC283682	chr15q26.1-q26.2		283682	---	---	---	---		MmugDNA.18036.1.S1_at_LOC283682
MmugDNA.18037.1.S1_at	213537_at	HLA-DPA1	major histocompatibility complex, class II, DP alpha 1	chr6p21.3		3113	---	---	---	---		MmugDNA.18037.1.S1_at_HLA-DPA1
MmugDNA.18038.1.S1_at	240181_at	---	---	---	---		---	---	---	---		MmugDNA.18038.1.S1_at_---
MmugDNA.18039.1.S1_at	222640_at	DNMT3A	DNA (cytosine-5-)-methyltransferase 3 alpha	chr2p23		1788	DNMT3A	DNA (cytosine-5-)-methyltransferase 3 alpha	---		694822	MmugDNA.18039.1.S1_at_DNMT3A
MmugDNA.18040.1.S1_at	NA	NA	NA	NA	NA		NA	NA	NA	NA		MmugDNA.18040.1.S1_at_NA
MmugDNA.18040.1.S1_s_at	232812_at	LOC401052	hypothetical LOC401052	chr3p25.3		401052	---	---	---	---		MmugDNA.18040.1.S1_s_at_LOC40105
MmugDNA.18041.1.S1_at	NA	NA	NA	NA	NA		NA	NA	NA	NA		MmugDNA.18041.1.S1_at_NA
MmugDNA.1804.1.S1_at	NA	NA	NA	NA	NA		NA	NA	NA	NA		MmugDNA.1804.1.S1_at_NA
MmugDNA.18042.1.S1_at	1560618_at	---	---	---	---		---	---	---	---		MmugDNA.18042.1.S1_at_---
MmugDNA.18043.1.S1_at	225963_at	KLHDC5	kelch domain containing 5	chr12p11.22		57542	KLHDC5	kelch domain containing 5	---		711593	MmugDNA.18043.1.S1_at_KLHDC5
MmugDNA.18044.1.S1_at	228807_at	---	---	---	---		---	---	---	---		MmugDNA.18044.1.S1_at_---
MmugDNA.18045.1.S1_at	225961_at	KLHDC5	kelch domain containing 5	chr12p11.22		57542	KLHDC5	kelch domain containing 5	---		711593	MmugDNA.18045.1.S1_at_KLHDC5
MmugDNA.18046.1.S1_at	205733_at	BLM	Bloom syndrome, RecQ helicase-like	chr15q26.1		641	BLM	Bloom syndrome	---		703460	MmugDNA.18046.1.S1_at_BLM
MmugDNA.18047.1.S1_at	221623_at	BCAN	brevican	chr1q31		63827	LOC718545	similar to brevican isoform 1	---		718545	MmugDNA.18047.1.S1_at_BCAN
MmugDNA.18048.1.S1_at	1564933_at	---	---	---	---		---	---	---	---		MmugDNA.18048.1.S1_at_---
MmugDNA.18049.1.S1_s_at	233126_s_at	OLAH	oleoyl-ACP hydrolase	chr10p13		55301	OLAH	oleoyl-ACP hydrolase	---		697753	MmugDNA.18049.1.S1_s_at_OLAH
MmugDNA.18050.1.S1_at	243882_at	---	---	---	---		---	---	---	---		MmugDNA.18050.1.S1_at_---
MmugDNA.18051.1.S1_at	215930_s_at	CTAGE5	CTAGE family, member 5	chr14q13.3		4253	LOC699511	similar to CTAGE family, member 5 isoform 1	---		699511	MmugDNA.18051.1.S1_at_CTAGE5
MmugDNA.1805.1.S1_at	236277_at	---	---	---	---		---	---	---	---		MmugDNA.1805.1.S1_at_---
MmugDNA.18052.1.S1_at	1561487_at	---	---	---	---		---	---	---	---		MmugDNA.18052.1.S1_at_---
MmugDNA.18053.1.S1_at	243855_at	---	---	---	---		---	---	---	---		MmugDNA.18053.1.S1_at_---
MmugDNA.18054.1.S1_at	237574_at	---	---	---	---		---	---	---	---		MmugDNA.18054.1.S1_at_---
MmugDNA.18055.1.S1_at	212552_at	HPCAL1	hippocalcin-like 1	chr2p25.1		3241	LOC695395	similar to hippocalcin-like 1	---		695395	MmugDNA.18055.1.S1_at_HPCAL1
MmugDNA.18056.1.S1_at	242505_at	---	---	---	---		---	---	---	---		MmugDNA.18056.1.S1_at_---
MmugDNA.18057.1.S1_at	231415_at	WBSCR22	Williams Beuren syndrome chromosome region 22	---		114049	---	---	---	---		MmugDNA.18057.1.S1_at_WBSCR22
MmugDNA.18058.1.S1_at	238870_at	KCNK9	Potassium channel, subfamily K, member 9	chr8q24.3		51305	---	---	---	---		MmugDNA.18058.1.S1_at_KCNK9
MmugDNA.18059.1.S1_at	232840_at	---	---	---	---		---	---	---	---		MmugDNA.18059.1.S1_at_---
MmugDNA.18060.1.S1_at	1553115_at	NKD1	naked cuticle homolog 1 (Drosophila)	chr16q12		85407	LOC698036	similar to naked cuticle homolog 1	---		698036	MmugDNA.18060.1.S1_at_NKD1
MmugDNA.18061.1.S1_a_at	NA	NA	NA	NA	NA		NA	NA	NA	NA		MmugDNA.18061.1.S1_a_at_NA
MmugDNA.18061.1.S1_at	239137_x_at	C6orf223	chromosome 6 open reading frame 223	chr6p21.1		221416	---	---	---	---		MmugDNA.18061.1.S1_at_C6orf223
MmugDNA.1806.1.S1_at	225607_at	CCDC43	coiled-coil domain containing 43	chr17q21.31		124808	LOC715325	similar to CG9586-PB	---		715325	MmugDNA.1806.1.S1_at_CCDC43
MmugDNA.18062.1.S1_at	244093_at	---	---	---	---		---	---	---	---		MmugDNA.18062.1.S1_at_---
MmugDNA.18063.1.S1_at	242924_at	---	---	---	---		---	---	---	---		MmugDNA.18063.1.S1_at_---
MmugDNA.18064.1.S1_at	228205_at	TKT	transketolase	chr3p14.3		7086	---	---	---	---		MmugDNA.18064.1.S1_at_TKT
MmugDNA.18065.1.S1_at	208244_at	BMP3	bone morphogenetic protein 3	chr4q21		651	BMP3	bone morphogenetic protein 3	---		695432	MmugDNA.18065.1.S1_at_BMP3
MmugDNA.18066.1.S1_at	228235_at	MGC16121	hypothetical protein MGC16121	chrXq26.3		84848	---	---	---	---		MmugDNA.18066.1.S1_at_MGC16121
MmugDNA.18067.1.S1_at	1558368_s_at	C1orf187	chromosome 1 open reading frame 187	chr1p36.22		374946	---	---	---	---		MmugDNA.18067.1.S1_at_C1orf187
MmugDNA.18068.1.S1_at	236851_x_at	---	---	---	---		---	---	---	---		MmugDNA.18068.1.S1_at_---
MmugDNA.18069.1.S1_at	204167_at	BTD	biotinidase	chr3p25		686	BTD	biotinidase	---		677722	MmugDNA.18069.1.S1_at_BTD
MmugDNA.18070.1.S1_at	203543_s_at	KLF9	Kruppel-like factor 9	chr9q13		687	---	---	---	---		MmugDNA.18070.1.S1_at_KLF9
MmugDNA.18070.1.S1_s_at	NA	NA	NA	NA	NA		NA	NA	NA	NA		MmugDNA.18070.1.S1_s_at_NA
MmugDNA.18071.1.S1_at	1563519_at	---	---	---	---		---	---	---	---		MmugDNA.18071.1.S1_at_---
MmugDNA.1807.1.S1_at	244177_at	---	---	---	---		---	---	---	---		MmugDNA.1807.1.S1_at_---
MmugDNA.18072.1.S1_s_at	NA	NA	NA	NA	NA		NA	NA	NA	NA		MmugDNA.18072.1.S1_s_at_NA
MmugDNA.18073.1.S1_at	1560628_at	KRT18P33	Keratin 18 pseudogene 33	chr2p14		647162	---	---	---	---		MmugDNA.18073.1.S1_at_KRT18P33
MmugDNA.18074.1.S1_at	237524_at	---	---	---	---		---	---	---	---		MmugDNA.18074.1.S1_at_---
MmugDNA.18075.1.S1_at	1557436_at	XKR6	XK, Kell blood group complex subunit-related family, member 6	chr8p23.1		286046	---	---	---	---		MmugDNA.18075.1.S1_at_XKR6

MmugDNA.18076.1.S1_at	1561311_at	---	---	---	---		---	---	---	---		MmugDNA.18076.1.S1_at_---
MmugDNA.18077.1.S1_at	1570422_at	SNTG2	syntrophin, gamma 2	chr2p25.3		54221	---	---	---	---		MmugDNA.18077.1.S1_at_SNTG2
MmugDNA.18078.1.S1_at	232561_at	ZNF771	Zinc finger protein 771	chr16p11.2		51333	---	---	---	---		MmugDNA.18078.1.S1_at_ZNF771
MmugDNA.18079.1.S1_at	1562414_at	---	---	---	---		---	---	---	---		MmugDNA.18079.1.S1_at_---
MmugDNA.18080.1.S1_at	221925_s_at	CSPP1	centrosome and spindle pole associated protein 1	chr8q13.2		79848	CSPP1	centrosome and spindle pole associated protein 1	---		704476	MmugDNA.18080.1.S1_at_CSPP1
MmugDNA.18081.1.S1_at	227105_at	CSPP1	centrosome and spindle pole associated protein 1	chr8q13.2		79848	CSPP1	centrosome and spindle pole associated protein 1	---		704476	MmugDNA.18081.1.S1_at_CSPP1
MmugDNA.1808.1.S1_at	232830_at	RNF32	ring finger protein 32	chr7q36		140545	LOC716703	similar to ring finger protein 32	---		716703	MmugDNA.1808.1.S1_at_RNF32
MmugDNA.18082.1.S1_at	233846_at	CCDC85A	coiled-coil domain containing 85A	chr2p16.1		114800	LOC717376	similar to coiled-coil domain containing 85A	---		717376	MmugDNA.18082.1.S1_at_CCDC85A
MmugDNA.18083.1.S1_at	225554_s_at	ANAPC7	anaphase promoting complex subunit 7	chr12q24.11		51434	ANAPC7	anaphase promoting complex subunit 7	---		709012	MmugDNA.18083.1.S1_at_ANAPC7
MmugDNA.18084.1.S1_at	235685_at	---	---	---	---		---	---	---	---		MmugDNA.18084.1.S1_at_---
MmugDNA.18085.1.S1_at	1560977_a_at	BCL2L13	BCL2-like 13 (apoptosis facilitator)	chr22q11.1		23786	---	---	---	---		MmugDNA.18085.1.S1_at_BCL2L13
MmugDNA.18086.1.S1_at	1556327_a_at	---	---	---	---		---	---	---	---		MmugDNA.18086.1.S1_at_---
MmugDNA.18087.1.S1_at	212607_at	AKT3	v-akt murine thymoma viral oncogene homolog 3 (protein kinase B, gamma)	chr1q43-q44		10000	---	---	---	---		MmugDNA.18087.1.S1_at_AKT3
MmugDNA.18088.1.S1_at	1569405_at	---	---	---	---		---	---	---	---		MmugDNA.18088.1.S1_at_---
MmugDNA.18089.1.S1_at	212609_s_at	AKT3	V-akt murine thymoma viral oncogene homolog 3 (protein kinase B, gamma)	chr1q43-q44		10000	---	---	---	---		MmugDNA.18089.1.S1_at_AKT3
MmugDNA.18090.1.S1_at	1557491_at	hCG_2038692	hypothetical LOC100240728	---		100240728	---	---	---	---		MmugDNA.18090.1.S1_at_hCG_20386
MmugDNA.18091.1.S1_at	235785_at	---	---	---	---		---	---	---	---		MmugDNA.18091.1.S1_at_---
MmugDNA.1809.1.S1_at	235730_at	---	---	---	---		---	---	---	---		MmugDNA.1809.1.S1_at_---
MmugDNA.18092.1.S1_at	236635_at	ZNF667	zinc finger protein 667	chr19q13.43		63934	---	---	---	---		MmugDNA.18092.1.S1_at_ZNF667
MmugDNA.18093.1.S1_at	225464_at	FRMD6	FERM domain containing 6	chr14q22.1		122786	LOC707266	similar to FERM domain containing 6	---		707266	MmugDNA.18093.1.S1_at_FRMD6
MmugDNA.18094.1.S1_at	208247_at	C3orf51	chromosome 3 open reading frame 51	chr3p14.3		711	---	---	---	---		MmugDNA.18094.1.S1_at_C3orf51
MmugDNA.18095.1.S1_at	225481_at	FRMD6	FERM domain containing 6	chr14q22.1		122786	LOC707266	similar to FERM domain containing 6	---		707266	MmugDNA.18095.1.S1_at_FRMD6
MmugDNA.18096.1.S1_at	228164_at	AP4E1	adaptor-related protein complex 4, epsilon 1 subunit	chr15q21.2		23431	AP4E1	adaptor-related protein complex 4, epsilon 1 subunit	---		693444	MmugDNA.18096.1.S1_at_AP4E1
MmugDNA.18097.1.S1_at	235671_at	---	---	---	---		---	---	---	---		MmugDNA.18097.1.S1_at_---
MmugDNA.18098.1.S1_s_at	1556709_a_at	LOC283887	hypothetical protein LOC283887	chr16p12.1		283887	---	---	---	---		MmugDNA.18098.1.S1_s_at_LOC28388
MmugDNA.18099.1.S1_at	217767_at	C3	complement component 3	chr19p13.3-p13.2		718	ADAM18	ADAM metallopeptidase domain 18	---		701958	MmugDNA.18099.1.S1_at_C3
MmugDNA.18100.1.S1_at	202992_at	C7	complement component 7	chr5p13		730	---	---	---	---		MmugDNA.18100.1.S1_at_C7
MmugDNA.18101.1.S1_at	229934_at	---	---	---	---		---	---	---	---		MmugDNA.18101.1.S1_at_---
MmugDNA.1810.1.S1_at	237217_at	ADAMTSL1	ADAMTS-like 1	chr9p22.2-p22.1		92949	ADAMTSL1	ADAMTS-like 1	---		712909	MmugDNA.1810.1.S1_at_ADAMTSL1
MmugDNA.18102.1.S1_at	234757_at	LOC100129884	Hypothetical protein LOC100129884	chr13q22.1		100129884	---	---	---	---		MmugDNA.18102.1.S1_at_LOC100129
MmugDNA.18103.1.S1_at	216692_at	---	---	---	---		---	---	---	---		MmugDNA.18103.1.S1_at_---
MmugDNA.18103.1.S1_x_at	1562853_x_at	---	---	---	---		---	---	---	---		MmugDNA.18103.1.S1_x_at_---
MmugDNA.18104.1.S1_at	239584_at	---	---	---	---		---	---	---	---		MmugDNA.18104.1.S1_at_---
MmugDNA.18105.1.S1_at	210073_at	ST8SIA1	ST8 alpha-N-acetyl-neuraminide alpha-2,8-sialyltransferase 1	chr12p12.1-p11.2		6489	ST8SIA1	ST8 alpha-N-acetyl-neuraminide alpha-2,8-sialyltransferase 1	---		706707	MmugDNA.18105.1.S1_at_ST8SIA1
MmugDNA.18106.1.S1_at	1554614_a_at	PTBP2	polypyrimidine tract binding protein 2	chr1p22.1-p21.3		58155	LOC710101	similar to polypyrimidine tract binding protein 2	---		710101	MmugDNA.18106.1.S1_at_PTBP2
MmugDNA.18107.1.S1_at	1570185_at	---	---	---	---		---	---	---	---		MmugDNA.18107.1.S1_at_---
MmugDNA.18108.1.S1_at	240557_at	---	---	---	---		---	---	---	---		MmugDNA.18108.1.S1_at_---
MmugDNA.18109.1.S1_at	240308_at	---	---	---	---		---	---	---	---		MmugDNA.18109.1.S1_at_---
MmugDNA.18110.1.S1_at	238085_at	---	---	---	---		---	---	---	---		MmugDNA.18110.1.S1_at_---
MmugDNA.18111.1.S1_s_at	206979_at	C8B	complement component 8, beta polypeptide	chr1p32		732	C8B	complement component 8, beta polypeptide	---		716155	MmugDNA.18111.1.S1_s_at_C8B
MmugDNA.1811.1.S1_at	234466_at	---	---	---	---		FAM20A	similar to family with sequence similarity 20, member C	---		695088	MmugDNA.1811.1.S1_at_---
MmugDNA.18112.1.S1_at	240760_at	CDRT15	CMT1A duplicated region transcript 15	chr17p12		146822	LOC697209	hypothetical protein LOC697209	---		697209	MmugDNA.18112.1.S1_at_CDRT15
MmugDNA.18113.1.S1_s_at	212674_s_at	DHX30	DEAH (Asp-Glu-Ala-His) box polypeptide 30	chr3p21.31		22907	LOC711178	similar to DEAH (Asp-Glu-Ala-His) box polypeptide 30 isoform 1	---		711178	MmugDNA.18113.1.S1_s_at_DHX30
MmugDNA.18114.1.S1_at	206873_at	CA6	carbonic anhydrase VI	chr1p36.2		765	LOC709540	similar to carbonic anhydrase VI precursor	---		709540	MmugDNA.18114.1.S1_at_CA6
MmugDNA.18115.1.S1_at	238319_at	LOC644090	hypothetical LOC644090	chr7q36.1		644090	---	---	---	---		MmugDNA.18115.1.S1_at_LOC644090
MmugDNA.18116.1.S1_s_at	1564280_x_at	LOC100133920	hypothetical protein LOC100133920	---		100133920	---	---	---	---		MmugDNA.18116.1.S1_s_at_LOC10013
MmugDNA.18117.1.S1_at	217273_at	PRAMEF10	PRAME family member 10	chr1p36.21		343071	PRAMEF10	PRAME family member 10	---		715953	MmugDNA.18117.1.S1_at_PRAMEF10
MmugDNA.18118.1.S1_at	1562850_at	---	---	---	---		---	---	---	---		MmugDNA.18118.1.S1_at_---
MmugDNA.18119.1.S1_at	1552314_a_at	EYA3	eyes absent homolog 3 (Drosophila)	chr1p36		2140	LOC716724	similar to eyes absent 3 homolog isoform 1	---		716724	MmugDNA.18119.1.S1_at_EYA3
MmugDNA.181.1.S1_at	1552980_at	HAS3	hyaluronan synthase 3	chr16q22.1		3038	HAS3	hyaluronan synthase 3	---		704121	MmugDNA.181.1.S1_at_HAS3
MmugDNA.18120.1.S1_at	1557590_at	LOC100130522	similar to hCG1996578	chr18q23		100130522	---	---	---	---		MmugDNA.18120.1.S1_at_LOC100130
MmugDNA.18121.1.S1_at	226374_at	---	---	---	---		---	---	---	---		MmugDNA.18121.1.S1_at_---
MmugDNA.1812.1.S1_at	208087_s_at	ZBP1	Z-DNA binding protein 1	chr20q13.31		81030	ZBP1	Z-DNA binding protein 1	---		696090	MmugDNA.1812.1.S1_at_ZBP1
MmugDNA.18122.1.S1_at	238622_at	RAP2B	RAP2B, member of RAS oncogene family	chr3q25.2		5912	---	---	---	---		MmugDNA.18122.1.S1_at_RAP2B
MmugDNA.18123.1.S1_at	204127_at	RFC3	replication factor C (activator 1) 3, 38kDa	chr13q12.3-q13		5983	LOC708774	Hypothetical protein LOC708774	---		708774	MmugDNA.18123.1.S1_at_RFC3
MmugDNA.18124.1.S1_at	220026_at	CLCA4	chloride channel accessory 4	chr1p31-p22		22802	LOC712123	similar to calcium activated chloride channel 4	---		712123	MmugDNA.18124.1.S1_at_CLCA4
MmugDNA.18125.1.S1_at	231247_s_at	LOC727820	Hypothetical protein LOC727820	chr1q21.1		727820	---	---	---	---		MmugDNA.18125.1.S1_at_LOC727820
MmugDNA.18126.1.S1_at	NA	NA	NA	NA	NA		NA	NA	NA	NA		MmugDNA.18126.1.S1_at_NA
MmugDNA.18127.1.S1_at	204713_s_at	F5	coagulation factor V (proaccelerin, labile factor)	chr1q23		2153	F5	coagulation factor V (proaccelerin, labile factor)	---		704695	MmugDNA.18127.1.S1_at_F5
MmugDNA.18128.1.S1_at	207300_s_at	F7	coagulation factor VII (serum prothrombin conversion accelerator)	chr13q34		2155	F7	coagulation factor VII	---		721933	MmugDNA.18128.1.S1_at_F7
MmugDNA.18129.1.S1_at	210435_at	ARL17	ADP-ribosylation factor-like 17	chr17q21.31		641522	LOC717420	similar to Enhancer of bithorax CG32346-PB, isoform B	---		717420	MmugDNA.18129.1.S1_at_ARL17
MmugDNA.18130.1.S1_at	215104_at	NRIP2	nuclear receptor interacting protein 2	chr12p13.33		83714	---	---	---	---		MmugDNA.18130.1.S1_at_NRIP2
MmugDNA.18131.1.S1_at	230993_s_at	C6orf118	chromosome 6 open reading frame 118	chr6q27		168090	LOC714890	similar to Protein C6orf118	---		714890	MmugDNA.18131.1.S1_at_C6orf118
MmugDNA.1813.1.S1_at	241185_at	SAE1	SUMO1 activating enzyme subunit 1	chr19q13.32		10055	---	---	---	---		MmugDNA.1813.1.S1_at_SAE1
MmugDNA.18132.1.S1_at	217662_x_at	---	---	---	---		---	---	---	---		MmugDNA.18132.1.S1_at_---
MmugDNA.18134.1.S1_at	205756_s_at	F8	coagulation factor VIII, procoagulant component	chrXq28		2157	---	---	---	---		MmugDNA.18134.1.S1_at_F8
MmugDNA.18135.1.S1_at	227172_at	TMEM116	transmembrane protein 116	chr12q24.13		89894	---	---	---	---		MmugDNA.18135.1.S1_at_TMEM116
MmugDNA.18136.1.S1_at	207218_at	F9	coagulation factor IX	chrXq27.1-q27.2		2158	LOC695578	coagulation factor IX protein	---		695578	MmugDNA.18136.1.S1_at_F9
MmugDNA.18137.1.S1_at	239013_at	SEC22C	SEC22 vesicle trafficking protein homolog C (S. cerevisiae)	chr3p22.1		9117	---	---	---	---		MmugDNA.18137.1.S1_at_SEC22C
MmugDNA.18138.1.S1_at	202790_at	CLDN7	claudin 7	chr17p13		1366	CLDN7	claudin 7	---		714647	MmugDNA.18138.1.S1_at_CLDN7
MmugDNA.18139.1.S1_at	1557632_at	---	---	---	---		---	---	---	---		MmugDNA.18139.1.S1_at_---
MmugDNA.18140.1.S1_at	227630_at	---	---	---	---		---	---	---	---		MmugDNA.18140.1.S1_at_---
MmugDNA.18141.1.S1_at	208407_s_at	CTNND1	catenin (cadherin-associated protein), delta 1	chr11q11		1500	CTNND1	catenin (cadherin-associated protein), delta 1	---		704212	MmugDNA.18141.1.S1_at_CTNND1
MmugDNA.1814.1.S1_at	1558836_at	---	---	---	---		---	---	---	---		MmugDNA.1814.1.S1_at_---
MmugDNA.18142.1.S1_at	228708_at	RAB27B	RAB27B, member RAS oncogene family	chr18q21.2		5874	---	---	---	---		MmugDNA.18142.1.S1_at_RAB27B
MmugDNA.18143.1.S1_at	235796_at	---	---	---	---		---	---	---	---		MmugDNA.18143.1.S1_at_---
MmugDNA.18144.1.S1_at	234153_at	---	---	---	---		---	---	---	---		MmugDNA.18144.1.S1_at_---
MmugDNA.18145.1.S1_at	237926_s_at	---	---	---	---		---	---	---	---		MmugDNA.18145.1.S1_at_---
MmugDNA.18146.1.S1_at	233154_at	---	---	---	---		---	---	---	---		MmugDNA.18146.1.S1_at_---
MmugDNA.18147.1.S1_at	243522_at	---	---	---	---		---	---	---	---		MmugDNA.18147.1.S1_at_---
MmugDNA.18148.1.S1_at	228372_at	C10orf128	chromosome 10 open reading frame 128	chr10q11.22		170371	LOC710423	hypothetical protein LOC710423	---		710423	MmugDNA.18148.1.S1_at_C10orf128
MmugDNA.18149.1.S1_at	231403_at	TRIO	Triple functional domain (PTPRF interacting)	chr5p15.2		7204	---	---	---	---		MmugDNA.18149.1.S1_at_TRIO
MmugDNA.18150.1.S1_at	214014_at	CDC42EP2	CDC42 effector protein (Rho GTPase binding) 2	chr11q13		10435	LOC716504	similar to Cdc42 effector protein 2	---		716504	MmugDNA.18150.1.S1_at_CDC42EP2
MmugDNA.18151.1.S1_at	210388_at	PLCB2	phospholipase C, beta 2	chr15q15		5330	---	---	---	---		MmugDNA.18151.1.S1_at_PLCB2
MmugDNA.1815.1.S1_at	240209_at	C16orf78	chromosome 16 open reading frame 78	chr16q12.1		123970	LOC693672	hypothetical protein LOC693672	---		693672	MmugDNA.1815.1.S1_at_C16orf78
MmugDNA.18152.1.S1_at	208372_s_at	LIMK1	LIM domain kinase 1	chr7q11.23		3984	LOC699494	similar to LIM domain kinase 1	---		699494	MmugDNA.18152.1.S1_at_LIMK1
MmugDNA.18153.1.S1_at	210193_at	MOBP	myelin-associated oligodendrocyte basic protein	chr3p22.1		4336	LOC698803	Similar to vacuolar H+ ATPase E1	---		698803	MmugDNA.18153.1.S1_at_MOBP
MmugDNA.18154.1.S1_at	1558088_a_at	UBE2I	ubiquitin-conjugating enzyme E2I (UBC9 homolog, yeast)	chr16p13.3		7329	---	---	---	---		MmugDNA.18154.1.S1_at_UBE2I
MmugDNA.18155.1.S1_at	1569129_s_at	C3orf38	chromosome 3 open reading frame 38	chr3p11.2		285237	---	---	---	---		MmugDNA.18155.1.S1_at_C3orf38
MmugDNA.18156.1.S1_at	226059_at	TOMM40L	translocase of outer mitochondrial membrane 40 homolog (yeast)-like	chr1q23.3		84134	---	---	---	---		MmugDNA.18156.1.S1_at_TOMM40L
MmugDNA.18157.1.S1_at	1559402_a_at	C1orf61	chromosome 1 open reading frame 61	chr1q22		10485	---	---	---	---		MmugDNA.18157.1.S1_at_C1orf61
MmugDNA.18158.1.S1_at	205973_at	FEZ1	fasciculation and elongation protein zeta 1 (zygin I)	chr11q24.2		9638	---	---	---	---		MmugDNA.18158.1.S1_at_FEZ1
MmugDNA.18159.1.S1_at	209920_at	BMPR2	bone morphogenetic protein receptor, type II (serine/threonine kinase)	chr2q33-q34		659	---	---	---	---		MmugDNA.18159.1.S1_at_BMPR2
MmugDNA.18160.1.S1_at	216873_s_at	ATP8B2	ATPase, class I, type 8B, member 2	chr1q21.3		57198	ATP8B2	ATPase, class I, type 8B, member 2	---		716652	MmugDNA.18160.1.S1_at_ATP8B2
MmugDNA.18161.1.S1_at	225116_at	HIPK2	homeodomain interacting protein kinase 2	chr7q32-q34		28996	HIPK2	homeodomain interacting protein kinase 2	---		716152	MmugDNA.18161.1.S1_at_HIPK2
MmugDNA.1816.1.S1_at	228002_at	IDI2	isopentenyl-diphosphate delta isomerase 2	chr10p15.3		91734	---	---	---	---		MmugDNA.1816.1.S1_at_IDI2
MmugDNA.18162.1.S1_at	235781_at	CACNA1B	calcium channel, voltage-dependent, N type, alpha 1B subunit	chr9q34		774	---	---	---	---		MmugDNA.18162.1.S1_at_CACNA1B
MmugDNA.18163.1.S1_at	203146_s_at	GABBR1	gamma-aminobutyric acid (GABA) B receptor, 1	chr6p21.31		2550	---	---	---	---		MmugDNA.18163.1.S1_at_GABBR1
MmugDNA.18164.1.S1_at	225115_at	HIPK2	homeodomain interacting protein kinase 2	chr7q32-q34		28996	HIPK2	homeodomain interacting protein kinase 2	---		716152	MmugDNA.18164.1.S1_at_HIPK2
MmugDNA.18165.1.S1_at	215135_at	DNPEP	aspartyl aminopeptidase	chr2q35		23549	DNPEP	aspartyl aminopeptidase	---		703180	MmugDNA.18165.1.S1_at_DNPEP
MmugDNA.18166.1.S1_at	225097_at	HIPK2	homeodomain interacting protein kinase 2	chr7q32-q34		28996	HIPK2	homeodomain interacting protein kinase 2	---		716152	MmugDNA.18166.1.S1_at_HIPK2
MmugDNA.18167.1.S1_at	242748_at	SREBF2	sterol regulatory element binding transcription factor 2	chr22q13		6721	LOC712307	similar to sterol regulatory element-binding transcription factor 2	---		712307	MmugDNA.18167.1.S1_at_SREBF2
MmugDNA.18168.1.S1_at	212550_at	STAT5B	signal transducer and activator of transcription 5B	chr17q11.2		6777	LOC709760	similar to signal transducer and activator of transcription 5B	---		709760	MmugDNA.18168.1.S1_at_STAT5B
MmugDNA.18169.1.S1_at	212549_at	STAT5B	signal transducer and activator of transcription 5B	chr17q11.2		6777	LOC709760	similar to signal transducer and activator of transcription 5B	---		709760	MmugDNA.18169.1.S1_at_STAT5B
MmugDNA.18170.1.S1_at	NA	NA	NA	NA	NA		NA	NA	NA	NA		MmugDNA.18170.1.S1_at_NA
MmugDNA.18171.1.S1_at	NA	NA	NA	NA	NA		NA	NA	NA	NA		MmugDNA.18171.1.S1_at_NA
MmugDNA.1817.1.S1_at	244149_at	---	---	---	---		---	---	---	---		MmugDNA.1817.1.S1_at_---
MmugDNA.18172.1.S1_at	243902_at	---	---	---	---		---	---	---	---		MmugDNA.18172.1.S1_at_---

MmugDNA.18173.1.S1_at	238940_at	KLF12	Kruppel-like factor 12	chr13q22		11278	KLF12	Kruppel-like factor 12	---		694575	MmugDNA.18173.1.S1_at_KLF12
MmugDNA.18174.1.S1_at	230226_s_at	KDM5A	lysine (K)-specific demethylase 5A	chr12p11		5927	---	---	---	---		MmugDNA.18174.1.S1_at_KDM5A
MmugDNA.18175.1.S1_at	209505_at	NR2F1	Nuclear receptor subfamily 2, group F, member 1	chr5q14		7025	NR2F1	nuclear receptor subfamily 2, group F, member 1	---		700777	MmugDNA.18175.1.S1_at_NR2F1
MmugDNA.18176.1.S1_at	203562_at	FEZ1	fasciculation and elongation protein zeta 1 (zygin I)	chr11q24.2		9638	FEZ1	fasciculation and elongation protein zeta 1 (zygin I)	---		713106	MmugDNA.18176.1.S1_at_FEZ1
MmugDNA.18177.1.S1_at	211337_s_at	TUBGCP4	tubulin, gamma complex associated protein 4	chr15q15		27229	76P	gamma tubulin ring complex protein (76p gene)	---		710327	MmugDNA.18177.1.S1_at_TUBGCP4
MmugDNA.18178.1.S1_at	210839_s_at	ENPP2	ectonucleotide pyrophosphatase/phosphodiesterase 2	chr8q24.1		5168	ENPP2	ectonucleotide pyrophosphatase/phosphodiesterase 2	---		705287	MmugDNA.18178.1.S1_at_ENPP2
MmugDNA.18178.1.S1_x_at	NA	NA	NA	NA	NA		NA	NA	NA	NA		MmugDNA.18178.1.S1_x_at_NA
MmugDNA.18179.1.S1_at	238607_at	ZNF296	zinc finger protein 296	chr19q13.32		162979	LOC713620	similar to zinc finger protein 342	---		713620	MmugDNA.18179.1.S1_at_ZNF296
MmugDNA.18180.1.S1_at	242211_x_at	WDR90	WD repeat domain 90	chr16p13.3		197335	---	---	---	---		MmugDNA.18180.1.S1_at_WDR90
MmugDNA.18181.1.S1_at	209076_s_at	WDR45L	WDR45-like	chr17q25.3		56270	---	---	---	---		MmugDNA.18181.1.S1_at_WDR45L
MmugDNA.1818.1.S1_at	NA	NA	NA	NA	NA		NA	NA	NA	NA		MmugDNA.1818.1.S1_at_NA
MmugDNA.18182.1.S1_at	214932_at	KIDINS220	kinase D-interacting substrate, 220kDa	chr2p24		57498	KIDINS220	kinase D-interacting substrate, 220kDa	---		693522	MmugDNA.18182.1.S1_at_KIDINS220
MmugDNA.18183.1.S1_at	235709_at	GAS2L3	growth arrest-specific 2 like 3	chr12q23.1		283431	GAS2L3	growth arrest-specific 2 like 3	---		694895	MmugDNA.18183.1.S1_at_GAS2L3
MmugDNA.18184.1.S1_at	214078_at	---	---	---	---		---	---	---	---		MmugDNA.18184.1.S1_at_---
MmugDNA.18185.1.S1_at	209174_s_at	QRICH1	glutamine-rich 1	chr3p21.31		54870	LOC708204	hypothetical protein LOC708204	---		708204	MmugDNA.18185.1.S1_at_QRICH1
MmugDNA.18186.1.S1_at	1552286_at	ATP6V1E2	ATPase, H+ transporting, lysosomal 31kDa, V1 subunit E2	chr2p21		90423	LOC714746	similar to ATPase, H+ transporting, lysosomal 31kDa, V1 subunit E isoform 2	---		714746	MmugDNA.18186.1.S1_at_ATP6V1E2
MmugDNA.18187.1.S1_s_at	NA	NA	NA	NA	NA		NA	NA	NA	NA		MmugDNA.18187.1.S1_s_at_NA
MmugDNA.18188.1.S1_at	227724_at	---	---	---	---		---	---	---	---		MmugDNA.18188.1.S1_at_---
MmugDNA.18189.1.S1_at	201508_at	IGFBP4	insulin-like growth factor binding protein 4	chr17q12-q21.1		3487	IGFBP4	insulin-like growth factor binding protein 4	---		700963	MmugDNA.18189.1.S1_at_IGFBP4
MmugDNA.18190.1.S1_at	213305_s_at	PPP2R5C	protein phosphatase 2, regulatory subunit B', gamma isoform	chr14q32		5527	LOC706718	similar to gamma isoform of regulatory subunit B56, protein phosphatase 2A isoform b	---		706718	MmugDNA.18190.1.S1_at_PPP2R5C
MmugDNA.18191.1.S1_at	203710_at	ITPR1	inositol 1,4,5-triphosphate receptor, type 1	chr3p26-p25		3708	ITPR1	inositol 1,4,5-triphosphate receptor, type 1	---		703669	MmugDNA.18191.1.S1_at_ITPR1
MmugDNA.1819.1.S1_at	1563638_at	FAM18A	family with sequence similarity 18, member A	chr16p13.13		780776	LOC720712	Similar to chromodomain helicase DNA binding protein 5	---		720712	MmugDNA.1819.1.S1_at_FAM18A
MmugDNA.18192.1.S1_at	202941_at	NDUFV2	NADH dehydrogenase (ubiquinone) flavoprotein 2, 24kDa	chr18p11.31-p11.2		4729	NDUFV2	NADH dehydrogenase (ubiquinone) flavoprotein 2, 24kDa	---		706067	MmugDNA.18192.1.S1_at_NDUFV2
MmugDNA.18193.1.S1_at	228434_at	BTNL9	butyrophilin-like 9	chr5q35.3		153579	---	---	---	---		MmugDNA.18193.1.S1_at_BTNL9
MmugDNA.18194.1.S1_at	205698_s_at	MAP2K6	mitogen-activated protein kinase kinase 6	chr17q24.3		5608	PTBP1	Polypyrimidine tract binding protein 1	---		703430	MmugDNA.18194.1.S1_at_MAP2K6
MmugDNA.18195.1.S1_at	220506_at	GUCY1B2	guanylate cyclase 1, soluble, beta 2	chr13q14.2-q14.3		2974	LOC712734	hypothetical protein LOC712734	---		712734	MmugDNA.18195.1.S1_at_GUCY1B2
MmugDNA.18196.1.S1_at	241646_s_at	BCAP29	B-cell receptor-associated protein 29	chr7q22-q31		55973	---	---	---	---		MmugDNA.18196.1.S1_at_BCAP29
MmugDNA.18197.1.S1_at	237786_at	MAGI2	Membrane associated guanylate kinase, WW and PDZ domain containing 2	chr7q21		9863	---	---	---	---		MmugDNA.18197.1.S1_at_MAGI2
MmugDNA.18198.1.S1_at	241232_x_at	---	---	---	---		---	---	---	---		MmugDNA.18198.1.S1_at_---
MmugDNA.18199.1.S1_at	226678_at	UNC13D	unc-13 homolog D (C. elegans)	chr17q25.1		201294	UNC13D	unc-13 homolog D (C. elegans)	---		704431	MmugDNA.18199.1.S1_at_UNC13D
MmugDNA.18.1.S1_at	240362_at	BRP44L	Brain protein 44-like	chr6q27		51660	---	---	---	---		MmugDNA.18.1.S1_at_BRP44L
MmugDNA.18200.1.S1_at	216885_s_at	WDR42A	WD repeat domain 42A	chr1q22-q23		50717	LOC719645	similar to H326	---		719645	MmugDNA.18200.1.S1_at_WDR42A
MmugDNA.18201.1.S1_at	235595_at	ARHGEF2	Rho/rac guanine nucleotide exchange factor (GEF) 2	chr1q21-q22		9181	---	---	---	---		MmugDNA.18201.1.S1_at_ARHGEF2
MmugDNA.1820.1.S1_at	204181_s_at	ZBTB43	zinc finger and BTB domain containing 43	chr9q33-q34		23099	ZBTB43	zinc finger and BTB domain containing 43	---		701797	MmugDNA.1820.1.S1_at_ZBTB43
MmugDNA.18202.1.S1_at	1568899_at	---	---	---	---		---	---	---	---		MmugDNA.18202.1.S1_at_---
MmugDNA.18203.1.S1_at	211676_s_at	IFNGR1	interferon gamma receptor 1	chr6q23.3		3459	IFNGR1	interferon gamma receptor 1	---		704571	MmugDNA.18203.1.S1_at_IFNGR1
MmugDNA.18204.1.S1_s_at	214366_s_at	ALOX5	arachidonate 5-lipoxygenase	chr10q11.2		240	ALOX5	arachidonate 5-lipoxygenase	---		707926	MmugDNA.18204.1.S1_s_at_ALOX5
MmugDNA.18205.1.S1_at	236904_x_at	TECTA	tectorin alpha	chr11q22-q24		7007	---	---	---	---		MmugDNA.18205.1.S1_at_TECTA
MmugDNA.18206.1.S1_at	1561122_a_at	RAD51L1	RAD51-like 1 (S. cerevisiae)	chr14q23-q24.2		5890	---	---	---	---		MmugDNA.18206.1.S1_at_RAD51L1
MmugDNA.18207.1.S1_at	210711_at	C1orf217	chromosome 1 open reading frame 217	chr1q32.1		84719	---	---	---	---		MmugDNA.18207.1.S1_at_C1orf217
MmugDNA.18208.1.S1_at	1569782_at	---	---	---	---		---	---	---	---		MmugDNA.18208.1.S1_at_---
MmugDNA.18209.1.S1_at	NA	NA	NA	NA	NA		NA	NA	NA	NA		MmugDNA.18209.1.S1_at_NA
MmugDNA.18210.1.S1_at	244350_at	MYO10	myosin X	chr5p15.1-p14.3		4651	---	---	---	---		MmugDNA.18210.1.S1_at_MYO10
MmugDNA.18211.1.S1_at	1570269_at	---	---	---	---		---	---	---	---		MmugDNA.18211.1.S1_at_---
MmugDNA.1821.1.S1_at	NA	NA	NA	NA	NA		NA	NA	NA	NA		MmugDNA.1821.1.S1_at_NA
MmugDNA.1821.1.S1_s_at	210987_x_at	TPM1	tropomyosin 1 (alpha)	chr15q22.1		7168	TPM1	tropomyosin 1 (alpha)	---		705236	MmugDNA.1821.1.S1_s_at_TPM1
MmugDNA.18212.1.S1_at	235702_at	---	---	---	---		---	---	---	---		MmugDNA.18212.1.S1_at_---
MmugDNA.18213.1.S1_at	1568749_at	---	---	---	---		---	---	---	---		MmugDNA.18213.1.S1_at_---
MmugDNA.18214.1.S1_at	237790_at	LOC100127971	hypothetical protein LOC100127971	chr12q13.13		100127971	---	---	---	---		MmugDNA.18214.1.S1_at_LOC1001279
MmugDNA.18215.1.S1_s_at	208073_x_at	TTC3	tetratricopeptide repeat domain 3	chr21q22.2		7267	TTC3	tetratricopeptide repeat domain 3	---		704690	MmugDNA.18215.1.S1_s_at_TTC3
MmugDNA.18216.1.S1_at	207513_s_at	ZNF189	zinc finger protein 189	chr9q22-q31		7743	LOC713873	similar to zinc finger protein 189 isoform 1	---		713873	MmugDNA.18216.1.S1_at_ZNF189
MmugDNA.18217.1.S1_at	230574_at	LOC100130938	hypothetical protein LOC100130938	chr18q21.33		100130938	---	---	---	---		MmugDNA.18217.1.S1_at_LOC1001309
MmugDNA.18218.1.S1_at	213652_at	PCSK5	Proprotein convertase subtilisin/kexin type 5	chr9q21.3		5125	---	---	---	---		MmugDNA.18218.1.S1_at_PCSK5
MmugDNA.18219.1.S1_at	238675_x_at	BTF3L4	basic transcription factor 3-like 4	chr1p32.3		91408	---	---	---	---		MmugDNA.18219.1.S1_at_BTF3L4
MmugDNA.182.1.S1_at	221353_at	OR3A1	olfactory receptor, family 3, subfamily A, member 1	chr17p13.3		4994	LOC721435	similar to Olfactory receptor 3A1 (Olfactory receptor 17-40) (OR17-40)	---		721435	MmugDNA.182.1.S1_at_OR3A1
MmugDNA.18220.1.S1_at	215862_at	---	---	---	---		---	---	---	---		MmugDNA.18220.1.S1_at_---
MmugDNA.18221.1.S1_at	1566424_at	---	---	---	---		---	---	---	---		MmugDNA.18221.1.S1_at_---
MmugDNA.1822.1.S1_at	239100_x_at	PCNX	pecanex homolog (Drosophila)	chr14q24.2		22990	PCNX	pecanex homolog (Drosophila)	---		694094	MmugDNA.1822.1.S1_at_PCNX
MmugDNA.18222.1.S1_at	233105_at	---	---	---	---		---	---	---	---		MmugDNA.18222.1.S1_at_---
MmugDNA.18223.1.S1_at	230571_at	---	---	---	---		---	---	---	---		MmugDNA.18223.1.S1_at_---
MmugDNA.18224.1.S1_at	214467_at	GPR65	G protein-coupled receptor 65	chr14q31-q32.1		8477	GPR65	G protein-coupled receptor 65	---		693449	MmugDNA.18224.1.S1_at_GPR65
MmugDNA.18225.1.S1_at	1570385_at	---	---	---	---		---	---	---	---		MmugDNA.18225.1.S1_at_---
MmugDNA.18226.1.S1_at	1569934_at	PREX2	Phosphatidylinositol-3,4,5-trisphosphate-dependent Rac exchange factor 2	chr8q13.2		80243	---	---	---	---		MmugDNA.18226.1.S1_at_PREX2
MmugDNA.18227.1.S1_at	209553_at	VPS8	vacuolar protein sorting 8 homolog (S. cerevisiae)	chr3q27.2		23355	VPS8	vacuolar protein sorting 8 homolog (S. cerevisiae)	---		701567	MmugDNA.18227.1.S1_at_VPS8
MmugDNA.18228.1.S1_at	206904_at	MATN1	matrilin 1, cartilage matrix protein	chr1p35		4146	MATN1	matrilin 1, cartilage matrix protein	---		704521	MmugDNA.18228.1.S1_at_MATN1
MmugDNA.18229.1.S1_at	1563770_at	ZBTB8B	zinc finger and BTB domain containing 8B	chr1p35.1		728116	---	---	---	---		MmugDNA.18229.1.S1_at_ZBTB8B
MmugDNA.18230.1.S1_at	1554700_at	CDH7	cadherin 7, type 2	chr18q22-q23		1005	---	---	---	---		MmugDNA.18230.1.S1_at_CDH7
MmugDNA.18231.1.S1_at	1560347_at	---	---	---	---		---	---	---	---		MmugDNA.18231.1.S1_at_---
MmugDNA.18232.1.S1_at	212455_at	YTHDC1	YTH domain containing 1	chr4q13.2		91746	LOC709717	similar to YTH domain-containing protein 1 (Putative splicing factor YT521)	---		709717	MmugDNA.18232.1.S1_at_YTHDC1
MmugDNA.18233.1.S1_at	231481_at	CCNB3	cyclin B3	chrXp11		85417	CCNB3	cyclin B3	---		694534	MmugDNA.18233.1.S1_at_CCNB3
MmugDNA.18234.1.S1_at	NA	NA	NA	NA	NA		NA	NA	NA	NA		MmugDNA.18234.1.S1_at_NA
MmugDNA.18234.1.S1_s_at	216994_s_at	RUNX2	runt-related transcription factor 2	chr6p21		860	RUNX2	runt-related transcription factor 2	---		703331	MmugDNA.18234.1.S1_s_at_RUNX2
MmugDNA.18235.1.S1_at	216262_s_at	TGIF2	TGFB-induced factor homeobox 2	chr20q11.2-q12		60436	---	---	---	---		MmugDNA.18235.1.S1_at_TGIF2
MmugDNA.18236.1.S1_at	206222_at	TNFRSF10C	tumor necrosis factor receptor superfamily, member 10c, decoy without an intracellular domain	chr8p22-p21		8794	---	---	---	---		MmugDNA.18236.1.S1_at_TNFRSF10C
MmugDNA.18237.1.S1_at	1558103_a_at	---	---	---	---		LOC714613	Similar to peroxisomal short-chain alcohol dehydrogenase	---		714613	MmugDNA.18237.1.S1_at_---
MmugDNA.18238.1.S1_at	207015_s_at	ALDH1A2	aldehyde dehydrogenase 1 family, member A2	chr15q22.1		8854	ALDH1A2	aldehyde dehydrogenase 1 family, member A2	---		702235	MmugDNA.18238.1.S1_at_ALDH1A2
MmugDNA.18239.1.S1_at	233081_at	---	---	---	---		---	---	---	---		MmugDNA.18239.1.S1_at_---
MmugDNA.18240.1.S1_at	1556331_a_at	---	---	---	---		---	---	---	---		MmugDNA.18240.1.S1_at_---
MmugDNA.18241.1.S1_at	204688_at	SGCE	sarcoglycan, epsilon	chr7q21-q22		8910	SGCE	sarcoglycan, epsilon	---		700104	MmugDNA.18241.1.S1_at_SGCE
MmugDNA.1824.1.S1_at	1553961_s_at	SNX21	sorting nexin family member 21	chr20q13.12		90203	LOC709196	similar to sorting nexin 21 isoform a	---		709196	MmugDNA.1824.1.S1_at_SNX21
MmugDNA.18242.1.S1_at	1562916_at	---	---	---	---		---	---	---	---		MmugDNA.18242.1.S1_at_---
MmugDNA.18243.1.S1_at	237129_at	---	---	---	---		---	---	---	---		MmugDNA.18243.1.S1_at_---
MmugDNA.18244.1.S1_at	1557623_at	---	---	---	---		---	---	---	---		MmugDNA.18244.1.S1_at_---
MmugDNA.18245.1.S1_at	227476_at	LPGAT1	lysophosphatidylglycerol acyltransferase 1	chr1q32		9926	---	---	---	---		MmugDNA.18245.1.S1_at_LPGAT1
MmugDNA.18245.1.S1_s_at	NA	NA	NA	NA	NA		NA	NA	NA	NA		MmugDNA.18245.1.S1_s_at_NA
MmugDNA.18246.1.S1_at	234239_at	---	---	---	---		---	---	---	---		MmugDNA.18246.1.S1_at_---
MmugDNA.18247.1.S1_at	209203_s_at	BICD2	bicaudal D homolog 2 (Drosophila)	chr9q22.31		23299	BICD2	bicaudal D homolog 2 (Drosophila)	---		706621	MmugDNA.18247.1.S1_at_BICD2
MmugDNA.18248.1.S1_s_at	217284_x_at	SERHL2	serine hydrolase-like 2	chr22q13		253190	LOC713772	similar to serine hydrolase-like 2	---		713772	MmugDNA.18248.1.S1_s_at_SERHL2
MmugDNA.18249.1.S1_at	241527_at	---	---	---	---		---	---	---	---		MmugDNA.18249.1.S1_at_---
MmugDNA.18250.1.S1_at	1558803_at	---	---	---	---		---	---	---	---		MmugDNA.18250.1.S1_at_---
MmugDNA.18251.1.S1_at	202402_s_at	CARS	cysteinyl-tRNA synthetase	chr11p15.5		833	LOC696937	Similar to keratin 8	---		696937	MmugDNA.18251.1.S1_at_CARS
MmugDNA.18251.1.S1_s_at	NA	NA	NA	NA	NA		NA	NA	NA	NA		MmugDNA.18251.1.S1_s_at_NA
MmugDNA.1825.1.S1_at	214415_at	PLGLB1 /// PLGLB2	plasminogen-like B1 /// plasminogen-like B2	chr2p11-q11 /// chr2p11.2	5342 /// 5343		---	---	---	---		MmugDNA.1825.1.S1_at_PLGLB1 /// PL
MmugDNA.18252.1.S1_at	243558_at	---	---	---	---		---	---	---	---		MmugDNA.18252.1.S1_at_---
MmugDNA.18253.1.S1_at	238413_at	LOC100131998	RRN3 RNA polymerase I transcription factor homolog (S. cerevisiae) pseudogene	chr16p12.1		100131998	---	---	---	---		MmugDNA.18253.1.S1_at_LOC1001319
MmugDNA.18254.1.S1_at	206398_s_at	CD19	CD19 molecule	chr16p11.2		930	---	---	---	---		MmugDNA.18254.1.S1_at_CD19
MmugDNA.18255.1.S1_at	235573_at	---	---	---	---		LOC721956	hypothetical protein LOC721956	---		721956	MmugDNA.18255.1.S1_at_---
MmugDNA.18257.1.S1_at	210572_at	PCDHA2	protocadherin alpha 2	chr5q31		56146	---	---	---	---		MmugDNA.18257.1.S1_at_PCDHA2
MmugDNA.18258.1.S1_at	237186_at	---	---	---	---		---	---	---	---		MmugDNA.18258.1.S1_at_---
MmugDNA.18259.1.S1_at	203025_at	ARD1A	ARD1 homolog A, N-acetyltransferase (S. cerevisiae)	chrXq28		8260	ARD1A	ARD1 homolog A, N-acetyltransferase (S. cerevisiae)	---		698017	MmugDNA.18259.1.S1_at_ARD1A
MmugDNA.18260.1.S1_at	244294_at	GTF2H5	general transcription factor IIH, polypeptide 5	chr6q25.3		404672	---	---	---	---		MmugDNA.18260.1.S1_at_GTF2H5
MmugDNA.18261.1.S1_at	225478_at	MFHAS1	malignant fibrous histiocytoma amplified sequence 1	chr8p23.1		9258	MFHAS1	malignant fibrous histiocytoma amplified sequence 1	---		699633	MmugDNA.18261.1.S1_at_MFHAS1
MmugDNA.1826.1.S1_at	242723_at	---	---	---	---		---	---	---	---		MmugDNA.1826.1.S1_at_---
MmugDNA.18262.1.S1_at	204764_at	FNTB	farnesyltransferase, CAAX box, beta	chr14q23-q24		2342	LOC708129	similar to farnesyltransferase, CAAX box, beta	---		708129	MmugDNA.18262.1.S1_at_FNTB
MmugDNA.18263.1.S1_at	222451_s_at	ZDHHC9	zinc finger, DHHC-type containing 9	chrXq26.1		51114	LOC717474	Similar to GLE1-like, RNA export mediator isoform 1	---		717474	MmugDNA.18263.1.S1_at_ZDHHC9
MmugDNA.18264.1.S1_at	NA	NA	NA	NA	NA		NA	NA	NA	NA		MmugDNA.18264.1.S1_at_NA
MmugDNA.18265.1.S1_at	238489_at	PRKAA2	protein kinase, AMP-activated, alpha 2 catalytic subunit	chr1p31		5563	---	---	---	---		MmugDNA.18265.1.S1_at_PRKAA2
MmugDNA.18266.1.S1_at	235064_s_at	C20orf196	chromosome 20 open reading frame 196	chr20p12.3		149840	LOC718152	hypothetical protein LOC718152	---		718152	MmugDNA.18266.1.S1_at_C20orf196

MmugDNA.18267.1.S1_at	223048_at	C11orf79	chromosome 11 open reading frame 79	chr11q12.2		54949	C14H11orf79	chromosome 11 open reading frame 79 ortholog	---		693401	MmugDNA.18267.1.S1_at_C11orf79
MmugDNA.18268.1.S1_at	238256_at	---	---	---	---		---	---	---	---		MmugDNA.18268.1.S1_at_---
MmugDNA.18269.1.S1_at	233455_at	---	---	---	---		---	---	---	---		MmugDNA.18269.1.S1_at_---
MmugDNA.18270.1.S1_at	236686_at	---	---	---	---		MVK	mevalonate kinase	---		707645	MmugDNA.18270.1.S1_at_---
MmugDNA.18271.1.S1_at	227061_at	---	---	---	---		---	---	---	---		MmugDNA.18271.1.S1_at_---
MmugDNA.1827.1.S1_at	1555567_s_at	LMOD3	leiomodin 3 (fetal)	chr3p14.1		56203	LOC696239	similar to leiomodin 3 (fetal)	---		696239	MmugDNA.1827.1.S1_at_LMOD3
MmugDNA.18272.1.S1_at	1558673_s_at	ZNF77	zinc finger protein 77	chr19p13.3		58492	LOC712142	similar to zinc finger protein 77	---		712142	MmugDNA.18272.1.S1_at_ZNF77
MmugDNA.18273.1.S1_at	241203_at	---	---	---	---		---	---	---	---		MmugDNA.18273.1.S1_at_---
MmugDNA.18274.1.S1_at	241414_at	ANKRD10	ankyrin repeat domain 10	chr13q34		55608	---	---	---	---		MmugDNA.18274.1.S1_at_ANKRD10
MmugDNA.18275.1.S1_at	244365_at	---	---	---	---		---	---	---	---		MmugDNA.18275.1.S1_at_---
MmugDNA.18276.1.S1_at	243396_at	---	---	---	---		---	---	---	---		MmugDNA.18276.1.S1_at_---
MmugDNA.18277.1.S1_at	231623_at	TMEM174	transmembrane protein 174	chr5q13.2		134288	LOC703111	hypothetical protein LOC703111	---		703111	MmugDNA.18277.1.S1_at_TMEM174
MmugDNA.18278.1.S1_at	201139_s_at	SSB	Sjogren syndrome antigen B (autoantigen La)	chr2q31.1		6741	LOC710344	similar to autoantigen La	---		710344	MmugDNA.18278.1.S1_at_SSB
MmugDNA.18279.1.S1_at	225912_at	TP53INP1	tumor protein p53 inducible nuclear protein 1	chr8q22		94241	EIF2S2	Similar to Eukaryotic translation initiation factor 2 subunit 2 (Eukaryotic translation initiation factor 2 b	---		708374	MmugDNA.18279.1.S1_at_TP53INP1
MmugDNA.18280.1.S1_at	1553794_at	STOML3	stomatin (EPB72)-like 3	chr13q13.3		161003	LOC696490	similar to stomatin-like 3	---		696490	MmugDNA.18280.1.S1_at_STOML3
MmugDNA.18281.1.S1_s_at	NA	NA	NA	NA	NA		NA	NA	NA	NA		MmugDNA.18281.1.S1_s_at_NA
MmugDNA.1828.1.S1_at	209734_at	NCKAP1L	NCK-associated protein 1-like	chr12q13.1		3071	LOC705782	similar to hematopoietic protein 1	---		705782	MmugDNA.1828.1.S1_at_NCKAP1L
MmugDNA.18282.1.S1_at	227382_at	CYB5B	cytochrome b5 type B (outer mitochondrial membrane)	chr16q22.1		80777	LOC705093	similar to Cytochrome b5 outer mitochondrial membrane isoform precursor	---		705093	MmugDNA.18282.1.S1_at_CYB5B
MmugDNA.18283.1.S1_at	1559067_a_at	---	---	---	---		---	---	---	---		MmugDNA.18283.1.S1_at_---
MmugDNA.18284.1.S1_at	237203_at	---	---	---	---		---	---	---	---		MmugDNA.18284.1.S1_at_---
MmugDNA.18285.1.S1_at	236795_at	---	---	---	---		---	---	---	---		MmugDNA.18285.1.S1_at_---
MmugDNA.18286.1.S1_at	201196_s_at	AMD1	adenosylmethionine decarboxylase 1	chr6q21-q22		262	AMD1	adenosylmethionine decarboxylase 1	---		698042	MmugDNA.18286.1.S1_at_AMD1
MmugDNA.18287.1.S1_at	222276_at	---	---	---	---		---	---	---	---		MmugDNA.18287.1.S1_at_---
MmugDNA.18288.1.S1_at	231014_at	TRIM50	tripartite motif-containing 50	chr7q11.23		135892	LOC716264	similar to tripartite motif protein 50A	---		716264	MmugDNA.18288.1.S1_at_TRIM50
MmugDNA.18289.1.S1_at	214189_s_at	GGA2	Golgi associated, gamma adaptin ear containing, ARF binding protein 2	chr16p12		23062	---	---	---	---		MmugDNA.18289.1.S1_at_GGA2
MmugDNA.18290.1.S1_at	227297_at	ITGA9	integrin, alpha 9	chr3p21.3		3680	ITGA9	integrin, alpha 9	---		698035	MmugDNA.18290.1.S1_at_ITGA9
MmugDNA.18291.1.S1_at	1554351_a_at	TIPRL	TIP41, TOR signaling pathway regulator-like (S. cerevisiae)	chr1q23.2		261726	TIPRL	TIP41, TOR signaling pathway regulator-like (S. cerevisiae)	---		699058	MmugDNA.18291.1.S1_at_TIPRL
MmugDNA.1829.1.S1_at	1557763_at	P2RY5	purinergic receptor P2Y, G-protein coupled, 5	chr13q14		10161	---	---	---	---		MmugDNA.1829.1.S1_at_P2RY5
MmugDNA.18292.1.S1_at	208098_at	OR12D3 /// OR5V1	olfactory receptor, family 12, subfamily D, member 3 /// olfactory receptor, family 5, subfamily V, mem	chr6p22.1	81696 /// 81797		---	---	---	---		MmugDNA.18292.1.S1_at_OR12D3 /// (
MmugDNA.18293.1.S1_at	1560047_s_at	---	---	---	---		---	---	---	---		MmugDNA.18293.1.S1_at_---
MmugDNA.18294.1.S1_at	221017_s_at	LRRC3	leucine rich repeat containing 3	chr21q22.3		81543	LOC712418	similar to leucine-rich repeat-containing 3 precursor	---		712418	MmugDNA.18294.1.S1_at_LRRC3
MmugDNA.18295.1.S1_at	227532_at	LRRC39	leucine rich repeat containing 39	chr1p21.2		127495	LOC711471	similar to leucine rich repeat containing 39	---		711471	MmugDNA.18295.1.S1_at_LRRC39
MmugDNA.18296.1.S1_at	NA	NA	NA	NA	NA		NA	NA	NA	NA		MmugDNA.18296.1.S1_at_NA
MmugDNA.18297.1.S1_at	209838_at	COPS2	COP9 constitutive photomorphogenic homolog subunit 2 (Arabidopsis)	chr15q21.2		9318	---	---	---	---		MmugDNA.18297.1.S1_at_COPS2
MmugDNA.18298.1.S1_at	216165_at	GARNL3	GTPase activating Rap/RanGAP domain-like 3	chr9q33.3		84253	---	---	---	---		MmugDNA.18298.1.S1_at_GARNL3
MmugDNA.18299.1.S1_at	240829_at	---	---	---	---		---	---	---	---		MmugDNA.18299.1.S1_at_---
MmugDNA.18300.1.S1_at	1559577_at	FCN2	ficolin (collagen/fibrinogen domain containing lectin) 2 (hucolin)	chr9q34.3		2220	---	---	---	---		MmugDNA.18300.1.S1_at_FCN2
MmugDNA.18301.1.S1_at	212055_at	C18orf10	chromosome 18 open reading frame 10	chr18q12.2		25941	LOC709953	C18orf10	---		709953	MmugDNA.18301.1.S1_at_C18orf10
MmugDNA.1830.1.S1_at	221020_s_at	SLC25A32	solute carrier family 25, member 32	chr8q22.3		81034	---	---	---	---		MmugDNA.1830.1.S1_at_SLC25A32
MmugDNA.18302.1.S1_at	200914_x_at	KTN1	kinectin 1 (kinesin receptor)	chr14q22.1		3895	LOC697873	hypothetical protein LOC697873	---		697873	MmugDNA.18302.1.S1_at_KTN1
MmugDNA.18303.1.S1_at	209987_s_at	ASCL1	achaete-scute complex homolog 1 (Drosophila)	chr12q23.2		429	---	---	---	---		MmugDNA.18303.1.S1_at_ASCL1
MmugDNA.18304.1.S1_at	232776_at	---	---	---	---		---	---	---	---		MmugDNA.18304.1.S1_at_---
MmugDNA.18305.1.S1_at	217253_at	SH3BP2	SH3-domain binding protein 2	chr4p16.3		6452	---	---	---	---		MmugDNA.18305.1.S1_at_SH3BP2
MmugDNA.18306.1.S1_at	236490_at	---	---	---	---		---	---	---	---		MmugDNA.18306.1.S1_at_---
MmugDNA.18308.1.S1_at	232079_s_at	PVRL2	poliovirus receptor-related 2 (herpesvirus entry mediator B)	chr19q13.2		5819	---	---	---	---		MmugDNA.18308.1.S1_at_PVRL2
MmugDNA.18309.1.S1_at	218959_at	HOXC10	homeobox C10	chr12q13.3		3226	HOXC10	homeobox C10	---		702321	MmugDNA.18309.1.S1_at_HOXC10
MmugDNA.18311.1.S1_at	227156_at	CASK	calcium/calmodulin-dependent serine protein kinase (MAGUK family)	chrXp11.4		8573	---	---	---	---		MmugDNA.18311.1.S1_at_CASK
MmugDNA.1831.1.S1_at	241388_at	---	---	---	---		LOC700292	hypothetical protein LOC700292	---		700292	MmugDNA.1831.1.S1_at_---
MmugDNA.18312.1.S1_at	208099_x_at	TTLL5	tubulin tyrosine ligase-like family, member 5	chr14q24.3		23093	---	---	---	---		MmugDNA.18312.1.S1_at_TTLL5
MmugDNA.18313.1.S1_at	228444_at	---	---	---	---		LOC695250	similar to arrestin beta 1 isoform A	---		695250	MmugDNA.18313.1.S1_at_---
MmugDNA.18314.1.S1_at	227907_at	---	---	---	---		---	---	---	---		MmugDNA.18314.1.S1_at_---
MmugDNA.18315.1.S1_at	239710_at	FIGN	fidgetin	chr2q24.3		55137	---	---	---	---		MmugDNA.18315.1.S1_at_FIGN
MmugDNA.18316.1.S1_at	224871_at	TPRG1L	tumor protein p63 regulated 1-like	chr1p36.32		127262	LOC695045	hypothetical protein LOC695045	---		695045	MmugDNA.18316.1.S1_at_TPRG1L
MmugDNA.18317.1.S1_at	233469_at	psiTPTE22	TPTE pseudogene	chr22q11.1		387590	---	---	---	---		MmugDNA.18317.1.S1_at_psiTPTE22
MmugDNA.18318.1.S1_at	240749_at	---	---	---	---		---	---	---	---		MmugDNA.18318.1.S1_at_---
MmugDNA.183.1.S1_at	233335_at	---	---	---	---		---	---	---	---		MmugDNA.183.1.S1_at_---
MmugDNA.18320.1.S1_at	225762_x_at	LOC284801	hypothetical protein LOC284801	chr20p11.1		284801	---	---	---	---		MmugDNA.18320.1.S1_at_LOC284801
MmugDNA.18321.1.S1_at	225767_at	LOC284801	hypothetical protein LOC284801	chr20p11.1		284801	---	---	---	---		MmugDNA.18321.1.S1_at_LOC284801
MmugDNA.1832.1.S1_at	209949_at	NCF2	neutrophil cytosolic factor 2	chr1q25		4688	NCF2	neutrophil cytosolic factor 2	---		715052	MmugDNA.1832.1.S1_at_NCF2
MmugDNA.18322.1.S1_at	230772_at	---	---	---	---		---	---	---	---		MmugDNA.18322.1.S1_at_---
MmugDNA.18323.1.S1_at	220830_at	IMPG2	interphotoreceptor matrix proteoglycan 2	chr3q12.2-q12.3		50939	IMPG2	interphotoreceptor matrix proteoglycan 2	---		701319	MmugDNA.18323.1.S1_at_IMPG2
MmugDNA.18324.1.S1_at	221676_s_at	CORO1C	coronin, actin binding protein, 1C	chr12q24.1		23603	CORO1C	coronin, actin binding protein, 1C	---		707498	MmugDNA.18324.1.S1_at_CORO1C
MmugDNA.18325.1.S1_at	231454_at	PLAC4	placenta-specific 4	chr21q22.3		191585	---	---	---	---		MmugDNA.18325.1.S1_at_PLAC4
MmugDNA.18326.1.S1_at	207435_s_at	SRRM2	serine/arginine repetitive matrix 2	chr16p13.3		23524	---	---	---	---		MmugDNA.18326.1.S1_at_SRRM2
MmugDNA.18327.1.S1_at	214649_s_at	MTMR2	myotubularin related protein 2	chr11q22		8898	MTMR2	myotubularin related protein 2	---		699703	MmugDNA.18327.1.S1_at_MTMR2
MmugDNA.18328.1.S1_at	232874_at	DOCK9	dedicator of cytokinesis 9	chr13q32.3		23348	---	---	---	---		MmugDNA.18328.1.S1_at_DOCK9
MmugDNA.18329.1.S1_at	237150_at	---	---	---	---		---	---	---	---		MmugDNA.18329.1.S1_at_---
MmugDNA.18330.1.S1_at	206929_s_at	NFIC	nuclear factor I/C (CCAAT-binding transcription factor)	chr19p13.3		4782	NFIC	nuclear factor I/C	---		721702	MmugDNA.18330.1.S1_at_NFIC
MmugDNA.18330.1.S1_x_at	NA	NA	NA	NA	NA		NA	NA	NA	NA		MmugDNA.18330.1.S1_x_at_NA
MmugDNA.18331.1.S1_at	241199_x_at	DPPA4	developmental pluripotency associated 4	chr3q13.13		55211	---	---	---	---		MmugDNA.18331.1.S1_at_DPPA4
MmugDNA.1833.1.S1_at	221005_s_at	PTDSS2	phosphatidylserine synthase 2	chr11p15		81490	LOC698582	similar to phosphatidylserine synthase 2	---		698582	MmugDNA.1833.1.S1_at_PTDSS2
MmugDNA.18332.1.S1_at	207363_at	RS1	retinoschisin 1	chrXp22.13		6247	---	---	---	---		MmugDNA.18332.1.S1_at_RS1
MmugDNA.18333.1.S1_at	211456_x_at	MT1P2	metallothionein 1 pseudogene 2	chr1q43		645745	LOC708874	hypothetical protein LOC708874	---		708874	MmugDNA.18333.1.S1_at_MT1P2
MmugDNA.18333.1.S1_x_at	NA	NA	NA	NA	NA		NA	NA	NA	NA		MmugDNA.18333.1.S1_x_at_NA
MmugDNA.18334.1.S1_at	213741_s_at	KPNA1	karyopherin alpha 1 (importin alpha 5)	chr3q21		3836	---	---	---	---		MmugDNA.18334.1.S1_at_KPNA1
MmugDNA.18335.1.S1_at	208892_s_at	DUSP6	dual specificity phosphatase 6	chr12q22-q23		1848	LOC699107	Similar to paraoxonase 2 isoform 1	---		699107	MmugDNA.18335.1.S1_at_DUSP6
MmugDNA.18336.1.S1_at	207369_at	BRS3	bombesin-like receptor 3	chrXq26-q28		680	---	---	---	---		MmugDNA.18336.1.S1_at_BRS3
MmugDNA.18337.1.S1_at	33767_at	NEFH	neurofilament, heavy polypeptide	chr22q12.2		4744	LOC717705	similar to neurofilament, heavy polypeptide 200kDa	---		717705	MmugDNA.18337.1.S1_at_NEFH
MmugDNA.18338.1.S1_at	230118_at	---	---	---	---		---	---	---	---		MmugDNA.18338.1.S1_at_---
MmugDNA.18339.1.S1_at	206434_at	SPOCK3	sparc/osteonectin, cwcv and kazal-like domains proteoglycan (testican) 3	chr4q32.3		50859	SPOCK3	sparc/osteonectin, cwcv and kazal-like domains proteoglycan (testican) 3	---		705964	MmugDNA.18339.1.S1_at_SPOCK3
MmugDNA.18340.1.S1_at	1569003_at	TMEM49	transmembrane protein 49	chr17q23.1		81671	---	---	---	---		MmugDNA.18340.1.S1_at_TMEM49
MmugDNA.18341.1.S1_at	NA	NA	NA	NA	NA		NA	NA	NA	NA		MmugDNA.18341.1.S1_at_NA
MmugDNA.1834.1.S1_at	238190_at	TUFM	Tu translation elongation factor, mitochondrial	chr16p11.2		7284	TUFM	Tu translation elongation factor, mitochondrial	---		705462	MmugDNA.1834.1.S1_at_TUFM
MmugDNA.18342.1.S1_at	215890_at	GM2A	GM2 ganglioside activator	chr5q31.3-q33.1		2760	LOC713375	similar to GM2 ganglioside activator precursor	---		713375	MmugDNA.18342.1.S1_at_GM2A
MmugDNA.18343.1.S1_at	NA	NA	NA	NA	NA		NA	NA	NA	NA		MmugDNA.18343.1.S1_at_NA
MmugDNA.18343.1.S1_x_at	234517_at	---	---	---	---		---	---	---	---		MmugDNA.18343.1.S1_x_at_---
MmugDNA.18344.1.S1_at	232406_at	---	---	---	---		---	---	---	---		MmugDNA.18344.1.S1_at_---
MmugDNA.18345.1.S1_at	211802_x_at	CACNA1G	calcium channel, voltage-dependent, T type, alpha 1G subunit	chr17q22		8913	---	---	---	---		MmugDNA.18345.1.S1_at_CACNA1G
MmugDNA.18346.1.S1_at	239430_at	IGFL1	IGF-like family member 1	chr19q13.32		374918	LOC717124	similar to insulin growth factor-like family member 1	---		717124	MmugDNA.18346.1.S1_at_IGFL1
MmugDNA.18347.1.S1_at	209621_s_at	PDLIM3	PDZ and LIM domain 3	chr4q35		27295	PDLIM3	PDZ and LIM domain 3	---		695779	MmugDNA.18347.1.S1_at_PDLIM3
MmugDNA.18348.1.S1_at	219968_at	ZNF589	zinc finger protein 589	chr3p21		51385	---	---	---	---		MmugDNA.18348.1.S1_at_ZNF589
MmugDNA.18349.1.S1_at	211093_at	PDE6C	phosphodiesterase 6C, cGMP-specific, cone, alpha prime	chr10q24		5146	PDE6C	phosphodiesterase 6C, cGMP-specific, cone, alpha prime	---		700261	MmugDNA.18349.1.S1_at_PDE6C
MmugDNA.18350.1.S1_at	224241_s_at	LOC100132388	hypothetical protein LOC100132388	chr17q21.33		100132388	---	---	---	---		MmugDNA.18350.1.S1_at_LOC100132:
MmugDNA.18351.1.S1_at	1552400_a_at	C15orf27	chromosome 15 open reading frame 27	chr15q24.2		123591	LOC707482	hypothetical protein LOC707482	---		707482	MmugDNA.18351.1.S1_at_C15orf27
MmugDNA.1835.1.S1_at	239083_at	ZNF786	zinc finger protein 786	chr7q36.1		136051	LOC709906	similar to zinc finger protein 111	---		709906	MmugDNA.1835.1.S1_at_ZNF786
MmugDNA.18352.1.S1_s_at	201544_x_at	PABPN1	poly(A) binding protein, nuclear 1	chr14q11.2-q13		8106	LOC698012	similar to Polyadenylate-binding protein 2 (Poly(A)-binding protein 2) (PolyA binding protein II) (PABII)	---		698012	MmugDNA.18352.1.S1_s_at_PABPN1
MmugDNA.18353.1.S1_at	208995_s_at	PPIG	peptidylprolyl isomerase G (cyclophilin G)	chr2q31.1		9360	LOC722763	Similar to lysyl hydroxylase precursor	---		722763	MmugDNA.18353.1.S1_at_PPIG
MmugDNA.18354.1.S1_at	205431_s_at	BMP5	bone morphogenetic protein 5	chr6p12.1		653	LOC702904	Similar to T-cell receptor beta chain C region	---		702904	MmugDNA.18354.1.S1_at_BMP5
MmugDNA.18355.1.S1_at	220503_at	SLC13A1	solute carrier family 13 (sodium/sulfate symporters), member 1	chr7q31-q32		6561	SLC13A1	solute carrier family 13 (sodium/sulfate symporters), member 1	---		695320	MmugDNA.18355.1.S1_at_SLC13A1
MmugDNA.18356.1.S1_at	212492_s_at	KDM4B	lysine (K)-specific demethylase 4B	chr19p13.3		23030	KDM4B	lysine (K)-specific demethylase 4B	---		695760	MmugDNA.18356.1.S1_at_KDM4B
MmugDNA.18357.1.S1_at	219105_x_at	ORC6L	origin recognition complex, subunit 6 like (yeast)	chr16q12		23594	---	---	---	---		MmugDNA.18357.1.S1_at_ORC6L
MmugDNA.18358.1.S1_at	212495_at	KDM4B	lysine (K)-specific demethylase 4B	chr19p13.3		23030	KDM4B	lysine (K)-specific demethylase 4B	---		695760	MmugDNA.18358.1.S1_at_KDM4B
MmugDNA.18359.1.S1_at	1553768_a_at	DCBLD1	discoidin, CUB and LCCL domain containing 1	chr6q22.2		285761	DCBLD1	discoidin, CUB and LCCL domain containing 1	---		715234	MmugDNA.18359.1.S1_at_DCBLD1
MmugDNA.18360.1.S1_at	1570268_at	---	---	---	---		---	---	---	---		MmugDNA.18360.1.S1_at_---
MmugDNA.18361.1.S1_s_at	213982_s_at	RABGAP1L	RAB GTPase activating protein 1-like	chr1q24		9910	RABGAP1L	similar to RAB GTPase activating protein 1-like isoform A	---		708179	MmugDNA.18361.1.S1_s_at_RABGAP1
MmugDNA.1836.1.S1_at	244360_at	FBXL17 /// LOC10013(	F-box and leucine-rich repeat protein 17 /// similar to FBXL17 protein	chr5q21.3	100130089 /// 64839		---	---	---	---		MmugDNA.1836.1.S1_at_FBXL17 /// LC
MmugDNA.18362.1.S1_at	201702_s_at	PPP1R10	protein phosphatase 1, regulatory (inhibitor) subunit 10	chr6p21.3		5514	PPP1R10	protein phosphatase 1, regulatory (inhibitor) subunit 10	---		711949	MmugDNA.18362.1.S1_at_PPP1R10
MmugDNA.18363.1.S1_at	225955_at	LOC653506 /// METRNI	similar to meteorin, glial cell differentiation regulator-like /// meteorin, glial cell differentiation regulator-	chr17q25.3	284207 /// 653506		LOC719751	similar to meteorin, glial cell differentiation regulator-like	---		719751	MmugDNA.18363.1.S1_at_LOC653506

MmugDNA.18364.1.S1_at	1555527_at	COL9A1	collagen, type IX, alpha 1	chr6q12-q14		1297	LOC714809	similar to alpha 1 type IX collagen isoform 2 precursor	---		714809	MmugDNA.18364.1.S1_at_COL9A1
MmugDNA.18365.1.S1_at	241030_at	---	---	---	---		---	---	---	---		MmugDNA.18365.1.S1_at_---
MmugDNA.18366.1.S1_at	209731_at	NTHL1	nth endonuclease III-like 1 (E. coli)	chr16p13.3		4913	NTHL1	nth endonuclease III-like 1	---		694848	MmugDNA.18366.1.S1_at_NTHL1
MmugDNA.18367.1.S1_at	1552386_at	GAPT	GRB2-binding adaptor protein, transmembrane	chr5q11.2		202309	LOC708948	hypothetical protein LOC708948	---		708948	MmugDNA.18367.1.S1_at_GAPT
MmugDNA.18368.1.S1_at	235914_at	SYNPO	synaptopodin	chr5q33.1		11346	---	---	---	---		MmugDNA.18368.1.S1_at_SYNPO
MmugDNA.18369.1.S1_at	NA	NA	NA	NA	NA		NA	NA	NA	NA		MmugDNA.18369.1.S1_at_NA
MmugDNA.18370.1.S1_at	229309_at	ADRB1	adrenergic, beta-1-, receptor	chr10q24-q26		153	---	---	---	---		MmugDNA.18370.1.S1_at_ADRB1
MmugDNA.18371.1.S1_at	209331_s_at	MAX	MYC associated factor X	chr14q23		4149	MAX	similar to MAX protein isoform c	---		708228	MmugDNA.18371.1.S1_at_MAX
MmugDNA.1837.1.S1_at	220965_s_at	RSHL1	radial spokehead-like 1	chr19q13.3		81492	LOC722433	similar to radial spokehead-like 1	---		722433	MmugDNA.1837.1.S1_at_RSHL1
MmugDNA.18372.1.S1_at	229277_at	ADRB1	adrenergic, beta-1-, receptor	chr10q24-q26		153	---	---	---	---		MmugDNA.18372.1.S1_at_ADRB1
MmugDNA.18373.1.S1_at	213942_at	MEGF6	multiple EGF-like-domains 6	chr1p36.3		1953	LOC697150	hypothetical protein LOC697150	---		697150	MmugDNA.18373.1.S1_at_MEGF6
MmugDNA.18374.1.S1_at	206521_s_at	GTF2A1	general transcription factor IIA, 1, 19/37kDa	chr14q31.1		2957	GTF2A1	general transcription factor IIA, 1, 19/37kDa	---		708130	MmugDNA.18374.1.S1_at_GTF2A1
MmugDNA.18375.1.S1_at	242784_at	---	---	---	---		---	---	---	---		MmugDNA.18375.1.S1_at_---
MmugDNA.18376.1.S1_at	206957_at	AGXT	alanine-glyoxylate aminotransferase	chr2q36-q37		189	---	---	---	---		MmugDNA.18376.1.S1_at_AGXT
MmugDNA.18377.1.S1_at	239465_at	---	---	---	---		---	---	---	---		MmugDNA.18377.1.S1_at_---
MmugDNA.18378.1.S1_at	226657_at	C17orf103	chromosome 17 open reading frame 103	chr17p11.2		256302	---	---	---	---		MmugDNA.18378.1.S1_at_C17orf103
MmugDNA.18379.1.S1_at	232229_at	SETX	senataxin	chr9q34.13		23064	LOC715065	similar to senataxin	---		715065	MmugDNA.18379.1.S1_at_SETX
MmugDNA.18380.1.S1_at	243180_at	---	---	---	---		---	---	---	---		MmugDNA.18380.1.S1_at_---
MmugDNA.18381.1.S1_at	242538_at	TFDP1	Transcription factor Dp-1	chr13q34		7027	---	---	---	---		MmugDNA.18381.1.S1_at_TFDP1
MmugDNA.1838.1.S1_at	240980_at	---	---	---	---		---	---	---	---		MmugDNA.1838.1.S1_at_---
MmugDNA.18382.1.S1_at	226493_at	KCTD18	potassium channel tetramerisation domain containing 18	chr2q33.1		130535	KCTD18	potassium channel tetramerisation domain containing 18	---		699824	MmugDNA.18382.1.S1_at_KCTD18
MmugDNA.18383.1.S1_at	235194_at	TPCN2	two pore segment channel 2	chr11q13.2		219931	---	---	---	---		MmugDNA.18383.1.S1_at_TPCN2
MmugDNA.18384.1.S1_at	239592_at	---	---	---	---		---	---	---	---		MmugDNA.18384.1.S1_at_---
MmugDNA.18385.1.S1_at	222794_x_at	MTPAP	mitochondrial poly(A) polymerase	chr10p11.23		55149	---	---	---	---		MmugDNA.18385.1.S1_at_MTPAP
MmugDNA.18386.1.S1_at	244230_at	---	---	---	---		---	---	---	---		MmugDNA.18386.1.S1_at_---
MmugDNA.18387.1.S1_at	1563274_at	---	---	---	---		---	---	---	---		MmugDNA.18387.1.S1_at_---
MmugDNA.18388.1.S1_at	1569551_at	---	---	---	---		---	---	---	---		MmugDNA.18388.1.S1_at_---
MmugDNA.18389.1.S1_at	1561679_at	---	---	---	---		---	---	---	---		MmugDNA.18389.1.S1_at_---
MmugDNA.18390.1.S1_at	214708_at	SNTB1	syntrophin, beta 1 (dystrophin-associated protein A1, 59kDa, basic component 1)	chr8q23-q24		6641	SNTB1	syntrophin, beta 1 (dystrophin-associated protein A1, 59kDa, basic component 1)	---		703245	MmugDNA.18390.1.S1_at_SNTB1
MmugDNA.18391.1.S1_at	218346_s_at	SESN1	sestrin 1	chr6q21		27244	SESN1	sestrin 1	---		700232	MmugDNA.18391.1.S1_at_SESN1
MmugDNA.1839.1.S1_at	230205_at	ZNF561	zinc finger protein 561	chr19p13.2		93134	LOC714188	similar to zinc finger protein 561	---		714188	MmugDNA.1839.1.S1_at_ZNF561
MmugDNA.18392.1.S1_at	230688_at	---	---	---	---		---	---	---	---		MmugDNA.18392.1.S1_at_---
MmugDNA.18393.1.S1_at	226574_at	PSPC1	paraspeckle component 1	chr13q12.11		55269	---	---	---	---		MmugDNA.18393.1.S1_at_PSPC1
MmugDNA.18394.1.S1_at	NA	NA	NA	NA	NA		NA	NA	NA	NA		MmugDNA.18394.1.S1_at_NA
MmugDNA.18395.1.S1_at	242050_at	LRRC7	Leucine rich repeat containing 7	chr1p31.1		57554	---	---	---	---		MmugDNA.18395.1.S1_at_LRRC7
MmugDNA.18396.1.S1_at	238084_at	PCGF3	polycomb group ring finger 3	chr4p16.3		10336	---	---	---	---		MmugDNA.18396.1.S1_at_PCGF3
MmugDNA.18397.1.S1_at	213700_s_at	---	---	---	---		---	---	---	---		MmugDNA.18397.1.S1_at_---
MmugDNA.18398.1.S1_at	1552440_at	GPR182	G protein-coupled receptor 182	chr12q13.3		11318	GPR182	G protein-coupled receptor 182	---		713866	MmugDNA.18398.1.S1_at_GPR182
MmugDNA.18399.1.S1_at	236995_x_at	TFEC	transcription factor EC	chr7q31.2		22797	TFEC	transcription factor EC	---		704124	MmugDNA.18399.1.S1_at_TFEC
MmugDNA.18400.1.S1_at	236266_at	RORA	RAR-related orphan receptor A	chr15q21.3		6095	---	---	---	---		MmugDNA.18400.1.S1_at_RORA
MmugDNA.18401.1.S1_s_at	218786_at	NT5DC3	5'-nucleotidase domain containing 3	chr12q22-q23.1		51559	---	---	---	---		MmugDNA.18401.1.S1_s_at_NT5DC3
MmugDNA.1840.1.S1_at	225169_at	INTS4	integrator complex subunit 4	chr11q14.1		92105	INTS4	integrator complex subunit 4	---		699466	MmugDNA.1840.1.S1_at_INTS4
MmugDNA.18402.1.S1_at	1553162_x_at	C19orf55	chromosome 19 open reading frame 55	chr19q13.12		148137	---	---	---	---		MmugDNA.18402.1.S1_at_C19orf55
MmugDNA.18403.1.S1_at	241907_at	---	---	---	---		---	---	---	---		MmugDNA.18403.1.S1_at_---
MmugDNA.18404.1.S1_at	202290_at	PDAP1	PDGFA associated protein 1	chr7q22.1		11333	LOC707779	similar to 28 kDa heat- and acid-stable phosphoprotein (PDGF-associated protein) (PAP) (PDGFA-as	---		707779	MmugDNA.18404.1.S1_at_PDAP1
MmugDNA.18405.1.S1_s_at	236123_at	ST7L	Suppression of tumorigenicity 7 like	chr1p13.2		54879	LOC703434	Similar to source of immunodominant MHC-associated peptides	---		703434	MmugDNA.18405.1.S1_s_at_ST7L
MmugDNA.18406.1.S1_at	208366_at	PCDH11X	protocadherin 11 X-linked	chrXq21.3		27328	---	---	---	---		MmugDNA.18406.1.S1_at_PCDH11X
MmugDNA.18407.1.S1_at	35148_at	TJP3	tight junction protein 3 (zona occludens 3)	chr19p13.3		27134	LOC721760	similar to tight junction protein 3 (zona occludens 3)	---		721760	MmugDNA.18407.1.S1_at_TJP3
MmugDNA.18408.1.S1_at	242935_at	SBF2	SET binding factor 2	chr11p15.4		81846	LOC706782	similar to SET binding factor 2	---		706782	MmugDNA.18408.1.S1_at_SBF2
MmugDNA.18409.1.S1_at	234151_at	---	---	---	---		---	---	---	---		MmugDNA.18409.1.S1_at_---
MmugDNA.18410.1.S1_s_at	228055_at	NAPSB	napsin B aspartic peptidase pseudogene	chr19q13.33		256236	LOC719243	similar to napsin A preproprotein	---		719243	MmugDNA.18410.1.S1_s_at_NAPSB
MmugDNA.18411.1.S1_at	202730_s_at	PDCD4	programmed cell death 4 (neoplastic transformation inhibitor)	chr10q24		27250	LOC701340	Similar to human immunodeficiency virus type I enhancer binding protein 2	---		701340	MmugDNA.18411.1.S1_at_PDCD4
MmugDNA.1841.1.S1_at	208990_s_at	HNRNPH3	heterogeneous nuclear ribonucleoprotein H3 (2H9)	chr10q22		3189	HNRNPH3	heterogeneous nuclear ribonucleoprotein H3 (2H9)	---		694760	MmugDNA.1841.1.S1_at_HNRNPH3
MmugDNA.18412.1.S1_at	226786_at	RFX1	regulatory factor X, 1 (influences HLA class II expression)	chr19p13.1		5989	---	---	---	---		MmugDNA.18412.1.S1_at_RFX1
MmugDNA.18413.1.S1_at	212896_at	SKIV2L2	superkiller viralicidic activity 2-like 2 (S. cerevisiae)	chr5q11.2		23517	---	---	---	---		MmugDNA.18413.1.S1_at_SKIV2L2
MmugDNA.18414.1.S1_at	222552_at	GOLT1B	golgi transport 1 homolog B (S. cerevisiae)	chr12p12.1		51026	LOC705997	similar to Golgi transport 1 protein B homolog	---		705997	MmugDNA.18414.1.S1_at_GOLT1B
MmugDNA.18415.1.S1_at	227195_at	ZNF503	zinc finger protein 503	chr10q22.2		84858	NOLZ1	similar to zinc-finger protein NOLZ1	---		703912	MmugDNA.18415.1.S1_at_ZNF503
MmugDNA.18416.1.S1_at	1555317_at	POLK	polymerase (DNA directed) kappa	chr5q13		51426	---	---	---	---		MmugDNA.18416.1.S1_at_POLK
MmugDNA.18417.1.S1_at	1562696_at	---	---	---	---		---	---	---	---		MmugDNA.18417.1.S1_at_---
MmugDNA.18418.1.S1_at	1560136_at	ARL5C	ADP-ribosylation factor-like 5C	chr17q12		390790	---	---	---	---		MmugDNA.18418.1.S1_at_ARL5C
MmugDNA.18419.1.S1_at	235336_at	---	---	---	---		---	---	---	---		MmugDNA.18419.1.S1_at_---
MmugDNA.184.1.S1_at	243248_at	---	---	---	---		---	---	---	---		MmugDNA.184.1.S1_at_---
MmugDNA.18421.1.S1_at	201014_s_at	PAICS	phosphoribosylaminoimidazole carboxylase, phosphoribosylaminoimidazole succinocarboxamide synt	chr4q12		10606	LOC716893	Similar to peptidylprolyl isomerase A isoform 1	---		716893	MmugDNA.18421.1.S1_at_PAICS
MmugDNA.1842.1.S1_at	NA	NA	NA	NA	NA		NA	NA	NA	NA		MmugDNA.1842.1.S1_at_NA
MmugDNA.1842.1.S1_s_at	210110_x_at	HNRNPH3	heterogeneous nuclear ribonucleoprotein H3 (2H9)	chr10q22		3189	HNRNPH3	heterogeneous nuclear ribonucleoprotein H3 (2H9)	---		694760	MmugDNA.1842.1.S1_s_at_HNRNPH3
MmugDNA.18422.1.S1_at	230686_s_at	SLC13A3	solute carrier family 13 (sodium-dependent dicarboxylate transporter), member 3	chr20q12-q13.1		64849	---	---	---	---		MmugDNA.18422.1.S1_at_SLC13A3
MmugDNA.18423.1.S1_at	241426_at	KIAA1712	KIAA1712	chr4q34		80817	LOC697478	hypothetical protein LOC697478	---		697478	MmugDNA.18423.1.S1_at_KIAA1712
MmugDNA.18424.1.S1_at	234964_at	TRD@	T cell receptor delta locus	chr14q11.2		6964	LOC710821	similar to T-cell receptor alpha chain V region CTL-L17 precursor	---		710821	MmugDNA.18424.1.S1_at_TRD@
MmugDNA.18425.1.S1_at	207214_at	SPINK4	serine peptidase inhibitor, Kazal type 4	chr9p13.3		27290	SPINK4	serine peptidase inhibitor, Kazal type 4	---		702218	MmugDNA.18425.1.S1_at_SPINK4
MmugDNA.18426.1.S1_at	202212_at	PES1	pescadillo homolog 1, containing BRCT domain (zebrafish)	chr22q12.1		23481	PES1	pescadillo homolog 1, containing BRCT domain	---		718122	MmugDNA.18426.1.S1_at_PES1
MmugDNA.18427.1.S1_at	227483_s_at	---	---	---	---		---	---	---	---		MmugDNA.18427.1.S1_at_---
MmugDNA.18428.1.S1_at	241515_at	---	---	---	---		---	---	---	---		MmugDNA.18428.1.S1_at_---
MmugDNA.18429.1.S1_at	210692_s_at	SLC43A3	solute carrier family 43, member 3	chr11q11		29015	SLC43A3	solute carrier family 43, member 3	---		706267	MmugDNA.18429.1.S1_at_SLC43A3
MmugDNA.18430.1.S1_at	1559489_a_at	LOC257358	hypothetical LOC257358	chr5q35.1		257358	---	---	---	---		MmugDNA.18430.1.S1_at_LOC257358
MmugDNA.18431.1.S1_at	221118_at	PKD2L2	polycystic kidney disease 2-like 2	chr5q31		27039	---	---	---	---		MmugDNA.18431.1.S1_at_PKD2L2
MmugDNA.1843.1.S1_at	227277_at	MTDH	metadherin	chr8q22.1		92140	---	---	---	---		MmugDNA.1843.1.S1_at_MTDH
MmugDNA.18432.1.S1_at	210029_at	IDO1	indoleamine 2,3-dioxygenase 1	chr8p12-p11		3620	IDO1	indoleamine 2,3-dioxygenase 1	---		574370	MmugDNA.18432.1.S1_at_IDO1
MmugDNA.18433.1.S1_at	1554259_at	GPSM1	G-protein signaling modulator 1 (AGS3-like, C. elegans)	chr9q34.3		26086	---	---	---	---		MmugDNA.18433.1.S1_at_GPSM1
MmugDNA.18434.1.S1_at	209179_s_at	MBOAT7	membrane bound O-acyltransferase domain containing 7	chr19q13.4		79143	ATP5B	ATP synthase, H+ transporting, mitochondrial F1 complex, beta polypeptide	---		705465	MmugDNA.18434.1.S1_at_MBOAT7
MmugDNA.18435.1.S1_x_at	NA	NA	NA	NA	NA		NA	NA	NA	NA		MmugDNA.18435.1.S1_x_at_NA
MmugDNA.18436.1.S1_at	240069_at	---	---	---	---		---	---	---	---		MmugDNA.18436.1.S1_at_---
MmugDNA.18437.1.S1_at	209346_s_at	PI4K2A	phosphatidylinositol 4-kinase type 2 alpha	chr10q24		55361	PI4K2A	similar to phosphatidylinositol 4-kinase type II	---		707201	MmugDNA.18437.1.S1_at_PI4K2A
MmugDNA.18438.1.S1_at	201333_s_at	ARHGEF12	Rho guanine nucleotide exchange factor (GEF) 12	chr11q23.3		23365	ARHGEF12	Rho guanine nucleotide exchange factor (GEF) 12	---		712096	MmugDNA.18438.1.S1_at_ARHGEF12
MmugDNA.18439.1.S1_at	201335_s_at	ARHGEF12	Rho guanine nucleotide exchange factor (GEF) 12	chr11q23.3		23365	---	---	---	---		MmugDNA.18439.1.S1_at_ARHGEF12
MmugDNA.18440.1.S1_at	230416_at	---	---	---	---		---	---	---	---		MmugDNA.18440.1.S1_at_---
MmugDNA.18441.1.S1_at	NA	NA	NA	NA	NA		NA	NA	NA	NA		MmugDNA.18441.1.S1_at_NA
MmugDNA.1844.1.S1_at	1569974_x_at	SEPT13	Septin 13	chr7p13		641977	---	---	---	---		MmugDNA.1844.1.S1_at_SEPT13
MmugDNA.18442.1.S1_at	210294_at	TAPBP	TAP binding protein (tapasin)	chr6p21.3		6892	TAPBP	TAP binding protein (tapasin)	---		718235	MmugDNA.18442.1.S1_at_TAPBP
MmugDNA.18443.1.S1_at	230104_s_at	TPPP	tubulin polymerization promoting protein	chr5p15.3		11076	---	---	---	---		MmugDNA.18443.1.S1_at_TPPP
MmugDNA.18444.1.S1_at	78383_at	LOC100129250	similar to hCG1811779	chr9p21.1		100129250	LOC703827	hypothetical protein LOC703827	---		703827	MmugDNA.18444.1.S1_at_LOC1001292
MmugDNA.18445.1.S1_at	219282_s_at	TRPV2	transient receptor potential cation channel, subfamily V, member 2	chr17p11.2		51393	LOC695986	similar to transient receptor potential cation channel, subfamily V, member 2	---		695986	MmugDNA.18445.1.S1_at_TRPV2
MmugDNA.18446.1.S1_s_at	NA	NA	NA	NA	NA		NA	NA	NA	NA		MmugDNA.18446.1.S1_s_at_NA
MmugDNA.18447.1.S1_at	233264_at	---	---	---	---		NPY5R	Neuropeptide Y receptor Y5	---		574150	MmugDNA.18447.1.S1_at_---
MmugDNA.18448.1.S1_at	NA	NA	NA	NA	NA		NA	NA	NA	NA		MmugDNA.18448.1.S1_at_NA
MmugDNA.18449.1.S1_s_at	205591_at	OLFM1	olfactomedin 1	chr9q34.3		10439	LOC721518	Similar to zinc finger, ZZ type with EF hand domain 1	---		721518	MmugDNA.18449.1.S1_s_at_OLFM1
MmugDNA.18450.1.S1_s_at	215719_x_at	FAS	Fas (TNF receptor superfamily, member 6)	chr10q24.1		355	FAS	Fas (TNF receptor superfamily, member 6)	---		574332	MmugDNA.18450.1.S1_s_at_FAS
MmugDNA.18451.1.S1_at	201923_at	PRDX4	peroxiredoxin 4	chrXp22.11		10549	LOC697635	similar to Peroxiredoxin-4 (Prx-IV) (Thioredoxin peroxidase AO372) (Thioredoxin-dependent peroxide	---		697635	MmugDNA.18451.1.S1_at_PRDX4
MmugDNA.1845.1.S1_at	232076_at	ZNF707	zinc finger protein 707	chr8q24.3		286075	---	---	---	---		MmugDNA.1845.1.S1_at_ZNF707
MmugDNA.18452.1.S1_at	1562400_at	LOC283112	hypothetical protein LOC283112	chr11p15.1		283112	---	---	---	---		MmugDNA.18452.1.S1_at_LOC283112
MmugDNA.18453.1.S1_at	221389_at	PLA2G2E	phospholipase A2, group IIE	chr1p36.13		30814	PLA2G2E	phospholipase A2, group IIE	---		704299	MmugDNA.18453.1.S1_at_PLA2G2E
MmugDNA.18454.1.S1_at	236627_at	---	---	---	---		---	---	---	---		MmugDNA.18454.1.S1_at_---
MmugDNA.18455.1.S1_at	219152_at	PODXL2	podocalyxin-like 2	chr3q21.3		50512	LOC700452	similar to endoglycan	---		700452	MmugDNA.18455.1.S1_at_PODXL2
MmugDNA.18456.1.S1_at	1558512_at	---	---	---	---		---	---	---	---		MmugDNA.18456.1.S1_at_---
MmugDNA.18457.1.S1_at	210944_s_at	CAPN3	calpain 3, (p94)	chr15q15.1-q21.1		825	PSMD7	Similar to 26S proteasome non-ATPase regulatory subunit 7 (26S proteasome regulatory subunit rpn	---		718396	MmugDNA.18457.1.S1_at_CAPN3
MmugDNA.18458.1.S1_at	228065_at	BCL9L	B-cell CLL/lymphoma 9-like	chr11q23.3		283149	---	---	---	---		MmugDNA.18458.1.S1_at_BCL9L
MmugDNA.18459.1.S1_at	NA	NA	NA	NA	NA		NA	NA	NA	NA		MmugDNA.18459.1.S1_at_NA
MmugDNA.18460.1.S1_s_at	201461_s_at	MAPKAPK2	mitogen-activated protein kinase-activated protein kinase 2	chr1q32		9261	MAPKAPK2	mitogen-activated protein kinase-activated protein kinase 2	---		695044	MmugDNA.18460.1.S1_s_at_MAPKAPK2
MmugDNA.18461.1.S1_at	209947_at	UBAP2L	ubiquitin associated protein 2-like	chr1q21.3		9898	UBAP2L	ubiquitin associated protein 2-like	---		716417	MmugDNA.18461.1.S1_at_UBAP2L

MmugDNA.1846.1.S1_at	238236_at	---	---	---	---	---	---	---	---		MmugDNA.1846.1.S1_at_---
MmugDNA.18462.1.S1_at	240522_at	---	---	---	---	---	---	---	---		MmugDNA.18462.1.S1_at_---
MmugDNA.18463.1.S1_at	228783_at	BVES	blood vessel epicardial substance	chr6q21	11149	---	---	---	---		MmugDNA.18463.1.S1_at_BVES
MmugDNA.18464.1.S1_at	1564631_at	---	---	---	---	---	---	---	---		MmugDNA.18464.1.S1_at_---
MmugDNA.18465.1.S1_at	206954_at	WIT1	Wilms tumor upstream neighbor 1	chr11p13	51352	---	---	---	---		MmugDNA.18465.1.S1_at_WIT1
MmugDNA.18466.1.S1_at	221113_s_at	WNT16	wingless-type MMTV integration site family, member 16	chr7q31	51384	WNT16	wingless-type MMTV integration site family, member 16	---		694069	MmugDNA.18466.1.S1_at_WNT16
MmugDNA.18467.1.S1_at	204643_s_at	ENOX2	ecto-NOX disulfide-thiol exchanger 2	chrXq25-q26.2	10495	LOC703816	similar to cytosolic ovarian carcinoma antigen 1 isoform b	---		703816	MmugDNA.18467.1.S1_at_ENOX2
MmugDNA.18468.1.S1_at	1569756_at	---	---	---	---	---	---	---	---		MmugDNA.18468.1.S1_at_---
MmugDNA.18469.1.S1_at	205739_x_at	ZNF107	zinc finger protein 107	chr7q11.2	51427	ZNF107	zinc finger protein 107	---		710268	MmugDNA.18469.1.S1_at_ZNF107
MmugDNA.18470.1.S1_at	202631_s_at	APPBP2	amyloid beta precursor protein (cytoplasmic tail) binding protein 2	chr17q21-q23	10513	LOC716680	similar to amyloid beta precursor protein-binding protein 2	---		716680	MmugDNA.18470.1.S1_at_APPBP2
MmugDNA.18471.1.S1_at	219104_at	RNF141	ring finger protein 141	chr11p15.4	50862	---	---	---	---		MmugDNA.18471.1.S1_at_RNF141
MmugDNA.1847.1.S1_at	231579_s_at	TIMP2	TIMP metallopeptidase inhibitor 2	chr17q25	7077	---	---	---	---		MmugDNA.1847.1.S1_at_TIMP2
MmugDNA.18472.1.S1_at	229103_at	WNT3	wingless-type MMTV integration site family, member 3	chr17q21	7473	LOC716909	similar to wingless-type MMTV integration site family, member 3	---		716909	MmugDNA.18472.1.S1_at_WNT3
MmugDNA.18473.1.S1_at	221201_s_at	ZNF155	zinc finger protein 155	chr19q13.2-q13.32	7711	ZNF221	zinc finger protein 221	---		712736	MmugDNA.18473.1.S1_at_ZNF155
MmugDNA.18474.1.S1_s_at	201230_s_at	ARIH2	ariadne homolog 2 (Drosophila)	chr3p21.2-p21.3	10425	LOC701459	Similar to nucleophosmin 1 isoform 1	---		701459	MmugDNA.18474.1.S1_s_at_ARIH2
MmugDNA.18475.1.S1_at	1560622_at	---	---	---	---	---	---	---	---		MmugDNA.18475.1.S1_at_---
MmugDNA.18476.1.S1_at	242341_x_at	GLYCTK	glycerate kinase	chr3p21.1	132158	LOC714616	Similar to ribosomal protein L5	---		714616	MmugDNA.18476.1.S1_at_GLYCTK
MmugDNA.18477.1.S1_at	209871_s_at	APBA2	amyloid beta (A4) precursor protein-binding, family A, member 2	chr15q11-q12	321	---	---	---	---		MmugDNA.18477.1.S1_at_APBA2
MmugDNA.18478.1.S1_at	206064_s_at	PPIL2	peptidylprolyl isomerase (cyclophilin)-like 2	chr22q11.21	23759	---	---	---	---		MmugDNA.18478.1.S1_at_PPIL2
MmugDNA.18479.1.S1_at	237825_x_at	---	---	---	---	---	---	---	---		MmugDNA.18479.1.S1_at_---
MmugDNA.18480.1.S1_at	229728_at	---	---	---	---	---	---	---	---		MmugDNA.18480.1.S1_at_---
MmugDNA.18481.1.S1_at	220683_at	RDH8	retinol dehydrogenase 8 (all-trans)	chr19p13.2-p13.3	50700	LOC711496	similar to retinol dehydrogenase 8 (all-trans)	---		711496	MmugDNA.18481.1.S1_at_RDH8
MmugDNA.1848.1.S1_at	230647_at	TMEM53	transmembrane protein 53	chr1p34.1	79639	---	---	---	---		MmugDNA.1848.1.S1_at_TMEM53
MmugDNA.18482.1.S1_at	227883_at	FLJ36031	hypothetical protein FLJ36031	chr7q22.3	168455	LOC698727	hypothetical protein LOC698727	---		698727	MmugDNA.18482.1.S1_at_FLJ36031
MmugDNA.18483.1.S1_at	209883_at	GLT25D2	glycosyltransferase 25 domain containing 2	chr1q25	23127	---	---	---	---		MmugDNA.18483.1.S1_at_GLT25D2
MmugDNA.18484.1.S1_at	1569561_at	LOC389328	hypothetical LOC389328	chr5q31.1	389328	PITX1	paired-like homeodomain 1	---		711666	MmugDNA.18484.1.S1_at_LOC389328
MmugDNA.18485.1.S1_at	1555125_at	C21orf66	chromosome 21 open reading frame 66	chr21q21.3	94104	LOC700489	GC-rich sequence DNA-binding factor candidate	---		700489	MmugDNA.18485.1.S1_at_C21orf66
MmugDNA.18486.1.S1_s_at	206310_at	SPINK2	serine peptidase inhibitor, Kazal type 2 (acrosin-trypsin inhibitor)	chr4q12	6691	LOC712251	Similar to bradykinin receptor B1	---		712251	MmugDNA.18486.1.S1_s_at_SPINK2
MmugDNA.18487.1.S1_at	214704_at	TCF25	transcription factor 25 (basic helix-loop-helix)	chr16q24.3	22980	---	---	---	---		MmugDNA.18487.1.S1_at_TCF25
MmugDNA.18488.1.S1_at	224239_at	DEFB103A /// DEFB103	defensin, beta 103A /// defensin, beta 103B	chr8p23	414325 /// 55894	DEFB103A	defensin, beta 103A	---		694235	MmugDNA.18488.1.S1_at_DEFB103A /
MmugDNA.18489.1.S1_at	231051_at	---	---	---	---	---	---	---	---		MmugDNA.18489.1.S1_at_---
MmugDNA.18490.1.S1_at	227307_at	TSPAN18	Tetraspanin 18	chr11p11.2	90139	LOC715506	similar to Tetraspanin-18 (Tspan-18)	---		715506	MmugDNA.18490.1.S1_at_TSPAN18
MmugDNA.18491.1.S1_at	207873_x_at	SEZ6L	seizure related 6 homolog (mouse)-like	chr22q12.1	23544	SEZ6L	seizure related 6 homolog (mouse)-like	---		714925	MmugDNA.18491.1.S1_at_SEZ6L
MmugDNA.18491.1.S1_s_at	NA	NA	NA	NA	NA	NA	NA	NA	NA		MmugDNA.18491.1.S1_s_at_NA
MmugDNA.1849.1.S1_at	244596_at	---	---	---	---	---	---	---	---		MmugDNA.1849.1.S1_at_---
MmugDNA.18492.1.S1_at	1569788_at	ST8SIA1	ST8 alpha-N-acetyl-neuraminide alpha-2,8-sialyltransferase 1	chr12p12.1-p11.2	6489	---	---	---	---		MmugDNA.18492.1.S1_at_ST8SIA1
MmugDNA.18493.1.S1_at	244281_at	REXO1	REX1, RNA exonuclease 1 homolog (S. cerevisiae)	chr19p13.3	57455	---	---	---	---		MmugDNA.18493.1.S1_at_REXO1
MmugDNA.18494.1.S1_at	242018_at	---	---	---	---	---	---	---	---		MmugDNA.18494.1.S1_at_---
MmugDNA.18495.1.S1_at	225475_at	MIER1	mesoderm induction early response 1 homolog (Xenopus laevis)	chr1p31.3	57708	LOC704161	similar to mesoderm induction early response 1	---		704161	MmugDNA.18495.1.S1_at_MIER1
MmugDNA.18497.1.S1_at	1563321_s_at	MLLT10	myeloid/lymphoid or mixed-lineage leukemia (trithorax homolog, Drosophila); translocated to, 10	chr10p12	8028	MLL	myeloid/lymphoid or mixed-lineage leukemia (trithorax homolog, Drosophila)	---		705511	MmugDNA.18497.1.S1_at_MLLT10
MmugDNA.18497.1.S1_s_at	1565254_s_at	ELL	elongation factor RNA polymerase II	chr19p13.1	8178	MLL	myeloid/lymphoid or mixed-lineage leukemia (trithorax homolog, Drosophila)	---		705511	MmugDNA.18497.1.S1_s_at_ELL
MmugDNA.18498.1.S1_at	205965_at	BATF	basic leucine zipper transcription factor, ATF-like	chr14q24.3	10538	BATF	basic leucine zipper transcription factor, ATF-like	---		702646	MmugDNA.18498.1.S1_at_BATF
MmugDNA.18499.1.S1_at	226078_at	RPUSD1	RNA pseudouridylate synthase domain containing 1	chr16p13.3	113000	---	---	---	---		MmugDNA.18499.1.S1_at_RPUSD1
MmugDNA.18500.1.S1_at	237120_at	KRT77	keratin 77	chr12q13.13	374454	---	---	---	---		MmugDNA.18500.1.S1_at_KRT77
MmugDNA.18501.1.S1_at	242343_x_at	---	---	---	---	---	---	---	---		MmugDNA.18501.1.S1_at_---
MmugDNA.1850.1.S1_s_at	212999_x_at	HLA-DQB1	major histocompatibility complex, class II, DQ beta 1	chr6p21.3	3119	LOC720578	similar to HLA class II histocompatibility antigen, DQ(1) beta chain precursor (DC-3 beta chain)	---		720578	MmugDNA.1850.1.S1_s_at_HLA-DQB1
MmugDNA.18502.1.S1_at	203166_at	CFDP1	craniofacial development protein 1	chr16q22.2-q22.3	10428	LOC711343	similar to craniofacial development protein 1	---		711343	MmugDNA.18502.1.S1_at_CFDP1
MmugDNA.18503.1.S1_at	1562480_at	---	---	---	---	---	---	---	---		MmugDNA.18503.1.S1_at_---
MmugDNA.18504.1.S1_at	204911_s_at	TRIM3	tripartite motif-containing 3	chr11p15.5	10612	LOC710846	similar to tripartite motif-containing 3	---		710846	MmugDNA.18504.1.S1_at_TRIM3
MmugDNA.18505.1.S1_at	1562214_at	LOC151171	hypothetical protein LOC151171	chr2q37.3	151171	---	---	---	---		MmugDNA.18505.1.S1_at_LOC151171
MmugDNA.18506.1.S1_at	226438_at	SNTB1	syntrophin, beta 1 (dystrophin-associated protein A1, 59kDa, basic component 1)	chr8q23-q24	6641	SNTB1	syntrophin, beta 1 (dystrophin-associated protein A1, 59kDa, basic component 1)	---		703245	MmugDNA.18506.1.S1_at_SNTB1
MmugDNA.18507.1.S1_at	215445_x_at	---	---	---	---	---	---	---	---		MmugDNA.18507.1.S1_at_---
MmugDNA.18508.1.S1_at	219654_at	PTPLA	protein tyrosine phosphatase-like (proline instead of catalytic arginine), member A	chr10p14-p13	9200	LOC700635	similar to protein tyrosine phosphatase-like, member A	---		700635	MmugDNA.18508.1.S1_at_PTPLA
MmugDNA.18509.1.S1_at	1559490_at	LRCH3	leucine-rich repeats and calponin homology (CH) domain containing 3	chr3q29	84859	---	---	---	---		MmugDNA.18509.1.S1_at_LRCH3
MmugDNA.18510.1.S1_at	203555_at	PTPN18	protein tyrosine phosphatase, non-receptor type 18 (brain-derived)	chr2q21.1	26469	---	---	---	---		MmugDNA.18510.1.S1_at_PTPN18
MmugDNA.18511.1.S1_at	217967_s_at	FAM129A	family with sequence similarity 129, member A	chr1q25	116496	---	---	---	---		MmugDNA.18511.1.S1_at_FAM129A
MmugDNA.1851.1.S1_at	215238_s_at	DOCK9	dedicator of cytokinesis 9	chr13q32.3	23348	DOCK9	dedicator of cytokinesis 9	---		702426	MmugDNA.1851.1.S1_at_DOCK9
MmugDNA.18512.1.S1_at	234862_at	---	---	---	---	---	---	---	---		MmugDNA.18512.1.S1_at_---
MmugDNA.18513.1.S1_s_at	201568_at	UQCRQ	ubiquinol-cytochrome c reductase, complex III subunit VII, 9.5kDa	chr5q31.1	27089	UQCRQ	ubiquinol-cytochrome c reductase, complex III subunit VII, 9.5kDa	---		708950	MmugDNA.18513.1.S1_s_at_UQCRQ
MmugDNA.18514.1.S1_at	231877_at	RG9MTD2	RNA (guanine-9-) methyltransferase domain containing 2	chr4q23	93587	LOC696764 /// LOC7087	similar to RNA (guanine-9-) methyltransferase domain containing 2 /// similar to RNA (guanine-9-) me	---	696764 /// 708751		MmugDNA.18514.1.S1_at_RG9MTD2
MmugDNA.18515.1.S1_at	223548_at	C1orf26	chromosome 1 open reading frame 26	chr1q25	54823	LOC714083	similar to CG7206-PA, isoform A	---		714083	MmugDNA.18515.1.S1_at_C1orf26
MmugDNA.18516.1.S1_at	212752_at	CLASP1	cytoplasmic linker associated protein 1	chr2q14.2-q14.3	23332	LOC696719	similar to CLIP-associating protein 1	---		696719	MmugDNA.18516.1.S1_at_CLASP1
MmugDNA.18517.1.S1_at	1566950_at	---	---	---	---	---	---	---	---		MmugDNA.18517.1.S1_at_---
MmugDNA.18518.1.S1_at	NA	NA	NA	NA	NA	NA	NA	NA	NA		MmugDNA.18518.1.S1_at_NA
MmugDNA.18519.1.S1_at	209755_at	NMNAT2	nicotinamide nucleotide adenylyltransferase 2	chr1q25	23057	LOC717648	similar to nicotinamide mononucleotide adenylyltransferase 2 isoform 1	---		717648	MmugDNA.18519.1.S1_at_NMNAT2
MmugDNA.185.1.S1_at	222143_s_at	MTMR14	myotubularin related protein 14	chr3p26	64419	MTMR14	myotubularin related protein 14	---		703936	MmugDNA.185.1.S1_at_MTMR14
MmugDNA.18520.1.S1_at	203196_at	ABCC4	ATP-binding cassette, sub-family C (CFTR/MRP), member 4	chr13q32	10257	LOC697160	similar to ATP-binding cassette, sub-family C, member 4	---		697160	MmugDNA.18520.1.S1_at_ABCC4
MmugDNA.18521.1.S1_at	238317_x_at	---	---	---	---	---	---	---	---		MmugDNA.18521.1.S1_at_---
MmugDNA.1852.1.S1_at	223897_at	ZNF765	zinc finger protein 765	chr19q13.41	91661	---	---	---	---		MmugDNA.1852.1.S1_at_ZNF765
MmugDNA.18523.1.S1_at	242102_at	---	---	---	---	---	---	---	---		MmugDNA.18523.1.S1_at_---
MmugDNA.18524.1.S1_at	232662_x_at	C10orf58	chromosome 10 open reading frame 58	chr10q23.1	84293	---	---	---	---		MmugDNA.18524.1.S1_at_C10orf58
MmugDNA.18525.1.S1_at	1559347_at	---	---	---	---	---	---	---	---		MmugDNA.18525.1.S1_at_---
MmugDNA.18526.1.S1_at	221287_at	RNASEL	ribonuclease L (2',5'-oligoisoadenylate synthetase-dependent)	chr1q25	6041	RNASEL	ribonuclease L	---		715647	MmugDNA.18526.1.S1_at_RNASEL
MmugDNA.18527.1.S1_at	201793_x_at	SMG7	Smg-7 homolog, nonsense mediated mRNA decay factor (C. elegans)	chr1q25	9887	---	---	---	---		MmugDNA.18527.1.S1_at_SMG7
MmugDNA.18528.1.S1_at	202421_at	IGSF3	immunoglobulin superfamily, member 3	chr1p13	3321	LOC713579	similar to immunoglobulin superfamily, member 3 isoform 1	---		713579	MmugDNA.18528.1.S1_at_IGSF3
MmugDNA.18529.1.S1_at	215481_s_at	PEX5	peroxisomal biogenesis factor 5	chr12p13.31	5830	PEX5	peroxisomal biogenesis factor 5	---		715173	MmugDNA.18529.1.S1_at_PEX5
MmugDNA.18530.1.S1_at	239129_at	---	---	---	---	---	---	---	---		MmugDNA.18530.1.S1_at_---
MmugDNA.18531.1.S1_at	240958_at	UNC5C	Unc-5 homolog C (C. elegans)	chr4q21-q23	8633	---	---	---	---		MmugDNA.18531.1.S1_at_UNC5C
MmugDNA.1853.1.S1_at	233604_at	FLJ22763	hypothetical gene supported by AK026416	chr3q13.13	401081	LOC707722	hypothetical protein LOC707722	---		707722	MmugDNA.1853.1.S1_at_FLJ22763
MmugDNA.18532.1.S1_at	216911_s_at	HIC2	hypermethylated in cancer 2	chr22q11.21	23119	---	---	---	---		MmugDNA.18532.1.S1_at_HIC2
MmugDNA.18533.1.S1_at	1563933_a_at	PLD5	phospholipase D family, member 5	chr1q43	200150	PLD5	phospholipase D family, member 5	---		706256	MmugDNA.18533.1.S1_at_PLD5
MmugDNA.18534.1.S1_at	227042_at	YDJC	YdjC homolog (bacterial)	chr22q11.21	150223	---	---	---	---		MmugDNA.18534.1.S1_at_YDJC
MmugDNA.18535.1.S1_at	240577_at	LOC643749	hypothetical LOC643749	chr6q21	643749	---	---	---	---		MmugDNA.18535.1.S1_at_LOC643749
MmugDNA.18536.1.S1_at	231568_at	CT47A1 /// CT47A10 ///	cancer/testis antigen family 47, member A1 /// cancer/testis antigen family 47, member A10 /// cancer	chrXq24	255313 /// 653282 /// 7	LOC696321 /// LOC6971	hypothetical protein LOC696321 /// hypothetical protein LOC697148	---	696321 /// 697148		MmugDNA.18536.1.S1_at_CT47A1 /// C
MmugDNA.18537.1.S1_at	1558621_at	CABLES1	Cdk5 and Abl enzyme substrate 1	chr18q11.2	91768	SLC4A2	Solute carrier family 4, anion exchanger, member 2 (erythrocyte membrane protein band 3-like 1)	---		713966	MmugDNA.18537.1.S1_at_CABLES1
MmugDNA.18538.1.S1_at	NA	NA	NA	NA	NA	NA	NA	NA	NA		MmugDNA.18538.1.S1_at_NA
MmugDNA.18538.1.S1_s_at	205352_at	SERPINI1	serpin peptidase inhibitor, clade I (neuroserpin), member 1	chr3q26.1	5274	SERPINI1	serpin peptidase inhibitor, clade I (neuroserpin), member 1	---		699483	MmugDNA.18538.1.S1_s_at_SERPINI1
MmugDNA.18539.1.S1_at	206530_at	RAB30	RAB30, member RAS oncogene family	chr11q12-q14	27314	RAB30	RAB30, member RAS oncogene family	---		701550	MmugDNA.18539.1.S1_at_RAB30
MmugDNA.18540.1.S1_at	230411_at	---	---	---	---	---	---	---	---		MmugDNA.18540.1.S1_at_---
MmugDNA.18541.1.S1_at	218310_at	RABGEF1	RAB guanine nucleotide exchange factor (GEF) 1	chr7q11.21	27342	LOC695887	similar to RAB guanine nucleotide exchange factor (GEF) 1	---		695887	MmugDNA.18541.1.S1_at_RABGEF1
MmugDNA.1854.1.S1_at	1570255_s_at	ANKRD20A1 /// ANKRD	ankyrin repeat domain 20 family, member A1 /// ankyrin repeat domain 20 family, member A2 /// anky	chr1q12 /// chr2q11.1 /// chr4p11	375010 /// 441425 /// 4	LOC707318	similar to ankyrin repeat domain 20 family, member A2	---		707318	MmugDNA.1854.1.S1_at_ANKRD20A1 /
MmugDNA.18542.1.S1_at	244074_at	LOC100129104	similar to hydroxyproline-rich glycoprotein VSP-3	chr8q24.22	100129104	LOC703163	hypothetical protein LOC703163	---		703163	MmugDNA.18542.1.S1_at_LOC100129
MmugDNA.18543.1.S1_at	1558579_at	FLJ37786	hypothetical LOC642691	chr2p11.1	642691	---	---	---	---		MmugDNA.18543.1.S1_at_FLJ37786
MmugDNA.18544.1.S1_at	205130_at	RAGE	renal tumor antigen	chr14q32	5891	RAGE	renal tumor antigen	---		719012	MmugDNA.18544.1.S1_at_RAGE
MmugDNA.18545.1.S1_at	1557873_at	---	---	---	---	---	---	---	---		MmugDNA.18545.1.S1_at_---
MmugDNA.18546.1.S1_at	207105_s_at	PIK3R2	phosphoinositide-3-kinase, regulatory subunit 2 (beta)	chr19q13.2-q13.4	5296	---	---	---	---		MmugDNA.18546.1.S1_at_PIK3R2
MmugDNA.18547.1.S1_at	239555_at	---	---	---	---	---	---	---	---		MmugDNA.18547.1.S1_at_---
MmugDNA.18548.1.S1_at	203485_at	RTN1	reticulon 1	chr14q23.1	6252	---	---	---	---		MmugDNA.18548.1.S1_at_RTN1
MmugDNA.18549.1.S1_at	234745_at	---	---	---	---	---	---	---	---		MmugDNA.18549.1.S1_at_---
MmugDNA.18550.1.S1_at	1563036_at	---	---	---	---	---	---	---	---		MmugDNA.18550.1.S1_at_---
MmugDNA.18551.1.S1_at	213804_at	INPP5B	inositol polyphosphate-5-phosphatase, 75kDa	chr1p34	3633	INPP5B	inositol polyphosphate-5-phosphatase, 75kDa	---		716851	MmugDNA.18551.1.S1_at_INPP5B
MmugDNA.1855.1.S1_at	222792_s_at	CCDC59	coiled-coil domain containing 59	chr12q21.31	29080	CCDC59	coiled-coil domain containing 59	---		698443	MmugDNA.1855.1.S1_at_CCDC59
MmugDNA.18552.1.S1_at	201207_at	TNFAIP1	tumor necrosis factor, alpha-induced protein 1 (endothelial)	chr17q22-q23	7126	---	---	---	---		MmugDNA.18552.1.S1_at_TNFAIP1
MmugDNA.18553.1.S1_at	NA	NA	NA	NA	NA	NA	NA	NA	NA		MmugDNA.18553.1.S1_at_NA
MmugDNA.18553.1.S1_s_at	224629_at	LMAN1	lectin, mannose-binding, 1	chr18q21.3-q22	3998	LMAN1	lectin, mannose-binding, 1	---		697449	MmugDNA.18553.1.S1_s_at_LMAN1
MmugDNA.18554.1.S1_at	1552937_s_at	ATRIP	ATR interacting protein	chr3p21.31	84126	LOC713631	Hypothetical protein LOC713631	---		713631	MmugDNA.18554.1.S1_at_ATRIP
MmugDNA.18555.1.S1_at	204413_at	TRAF2	TNF receptor-associated factor 2	chr9q34	7186	---	---	---	---		MmugDNA.18555.1.S1_at_TRAF2
MmugDNA.18556.1.S1_at	228407_at	SCUBE3	signal peptide, CUB domain, EGF-like 3	chr6p21.3	222663	LOC718726	similar to signal peptide, CUB domain, EGF-like 3	---		718726	MmugDNA.18556.1.S1_at_SCUBE3

MmugDNA.18557.1.S1_at	208280_at	CDRT1	CMT1A duplicated region transcript 1	chr17p12	374286	---	---	---	---	MmugDNA.18557.1.S1_at_CDRT1
MmugDNA.18559.1.S1_at	200056_s_at	C1D	C1D nuclear receptor co-repressor	chr2p13-p12	10438	LOC698450	similar to nuclear DNA-binding protein	---	698450	MmugDNA.18559.1.S1_at_C1D
MmugDNA.18560.1.S1_at	233325_at	SLC35D2	solute carrier family 35, member D2	chr9q22.32	11046	---	---	---	---	MmugDNA.18560.1.S1_at_SLC35D2
MmugDNA.18561.1.S1_at	1557335_at	---	---	---	---	---	---	---	---	MmugDNA.18561.1.S1_at_---
MmugDNA.1856.1.S1_at	243759_at	SFRS15	Splicing factor, arginine/serine-rich 15	chr21q22.1	57466	---	---	---	---	MmugDNA.1856.1.S1_at_SFRS15
MmugDNA.18562.1.S1_at	207164_s_at	ZNF238	zinc finger protein 238	chr1q44-qter	10472	LOC714294	similar to zinc finger protein 238 isoform 1	---	714294	MmugDNA.18562.1.S1_at_ZNF238
MmugDNA.18563.1.S1_at	234403_at	OR1I1	olfactory receptor, family 1, subfamily I, member 1	chr19p13.12	126370	LOC719077	similar to Olfactory receptor 1I1 (Olfactory receptor 19-20) (OR19-20)	---	719077	MmugDNA.18563.1.S1_at_OR1I1
MmugDNA.18564.1.S1_at	220616_at	ZNF384	zinc finger protein 384	chr12p12	171017	---	---	---	---	MmugDNA.18564.1.S1_at_ZNF384
MmugDNA.18565.1.S1_at	205342_s_at	SULT1C2	sulfotransferase family, cytosolic, 1C, member 2	chr2q11.1-q11.2	6819	SULT1C4	sulfotransferase family, cytosolic, 1C, member 4	---	693398	MmugDNA.18565.1.S1_at_SULT1C2
MmugDNA.18566.1.S1_at	208277_at	PITX3	paired-like homeodomain 3	chr10q25	5309	---	---	---	---	MmugDNA.18566.1.S1_at_PITX3
MmugDNA.18567.1.S1_at	238514_at	TMEM25	transmembrane protein 25	chr11q23.3	84866	TMEM25	transmembrane protein 25	---	700466	MmugDNA.18567.1.S1_at_TMEM25
MmugDNA.18568.1.S1_at	NA	NA	NA	NA	NA	NA	NA	NA	NA	MmugDNA.18568.1.S1_at_NA
MmugDNA.18568.1.S1_s_at	228320_x_at	CCDC64	coiled-coil domain containing 64	chr12q24.23	92558	LOC698147	similar to coiled-coil domain containing 64	---	698147	MmugDNA.18568.1.S1_s_at_CCDC64
MmugDNA.18569.1.S1_at	219095_at	JMJD7-PLA2G4B /// PL	JMJD7-PLA2G4B readthrough transcript /// phospholipase A2, group IVB (cytosolic)	chr15q11.2-q21.3	100137049 /// 8681	JMJD7-PLA2G4B	JMJD7-PLA2G4B readthrough transcript	---	707262	MmugDNA.18569.1.S1_at_JMJD7-PLA2
MmugDNA.18570.1.S1_at	242990_at	---	---	---	---	---	---	---	---	MmugDNA.18570.1.S1_at_---
MmugDNA.18571.1.S1_at	NA	NA	NA	NA	NA	NA	NA	NA	NA	MmugDNA.18571.1.S1_at_NA
MmugDNA.1857.1.S1_at	208088_s_at	CFHR5	complement factor H-related 5	chr1q22-q23	81494	---	---	---	---	MmugDNA.1857.1.S1_at_CFHR5
MmugDNA.18572.1.S1_at	222818_at	OSBPL10	oxysterol binding protein-like 10	chr3p22.3	114884	OSBPL10	oxysterol binding protein-like 10	---	703555	MmugDNA.18572.1.S1_at_OSBPL10
MmugDNA.18573.1.S1_at	243978_at	FAM65C	family with sequence similarity 65, member C	chr20q13.13	140876	LOC712375	similar to C27H2.3	---	712375	MmugDNA.18573.1.S1_at_FAM65C
MmugDNA.18574.1.S1_at	206505_at	UGT2B4	UDP glucuronosyltransferase 2 family, polypeptide B4	chr4q13	7363	LOC711590	Similar to guanine nucleotide binding protein (G protein), q polypeptide	---	711590	MmugDNA.18574.1.S1_at_UGT2B4
MmugDNA.18575.1.S1_at	1560915_at	DPY19L1	dpy-19-like 1 (C. elegans)	chr7p14.3	23333	LOC716109	similar to dpy-19-like 1, like	---	716109	MmugDNA.18575.1.S1_at_DPY19L1
MmugDNA.18576.1.S1_at	210032_s_at	SPAG6	sperm associated antigen 6	chr10p12.2	9576	SPAG6	sperm associated antigen 6	---	704807	MmugDNA.18576.1.S1_at_SPAG6
MmugDNA.18577.1.S1_at	240917_at	---	---	---	---	---	---	---	---	MmugDNA.18577.1.S1_at_---
MmugDNA.18578.1.S1_at	203991_s_at	KDM6A	lysine (K)-specific demethylase 6A	chrXp11.2	7403	LOC709727	similar to ubiquitously transcribed tetratricopeptide repeat, X chromosome	---	709727	MmugDNA.18578.1.S1_at_KDM6A
MmugDNA.18579.1.S1_at	237319_at	C2orf53	chromosome 2 open reading frame 53	chr2p23.3	339779	LOC698826	hypothetical protein LOC698826	---	698826	MmugDNA.18579.1.S1_at_C2orf53
MmugDNA.18580.1.S1_at	223038_s_at	FAM60A	family with sequence similarity 60, member A	chr12p11	58516	LOC695854	similar to Protein FAM60A (Tera protein)	---	695854	MmugDNA.18580.1.S1_at_FAM60A
MmugDNA.18581.1.S1_at	NA	NA	NA	NA	NA	NA	NA	NA	NA	MmugDNA.18581.1.S1_at_NA
MmugDNA.1858.1.S1_at	217648_at	---	---	---	---	---	---	---	---	MmugDNA.1858.1.S1_at_---
MmugDNA.18582.1.S1_at	1554082_a_at	NOL9	nucleolar protein 9	chr1p36.31	79707	LOC703850	similar to K09B11.2	---	703850	MmugDNA.18582.1.S1_at_NOL9
MmugDNA.18583.1.S1_at	1554084_a_at	NOL9	nucleolar protein 9	chr1p36.31	79707	LOC703850	similar to K09B11.2	---	703850	MmugDNA.18583.1.S1_at_NOL9
MmugDNA.18584.1.S1_at	203301_s_at	DMTF1	cyclin D binding myb-like transcription factor 1	chr7q21	9988	DMTF1	cyclin D binding myb-like transcription factor 1	---	706292	MmugDNA.18584.1.S1_at_DMTF1
MmugDNA.18585.1.S1_at	206423_at	ANGPTL7	angiopoietin-like 7	chr1p36.3-p36.2	10218	---	---	---	---	MmugDNA.18585.1.S1_at_ANGPTL7
MmugDNA.18586.1.S1_at	204240_s_at	SMC2	structural maintenance of chromosomes 2	chr9q31.1	10592	SMC2	structural maintenance of chromosomes 2	---	713359	MmugDNA.18586.1.S1_at_SMC2
MmugDNA.18587.1.S1_at	238479_at	---	---	---	---	---	---	---	---	MmugDNA.18587.1.S1_at_---
MmugDNA.18588.1.S1_at	242293_at	ING3	inhibitor of growth family, member 3	chr7q31	54556	ING3	inhibitor of growth family, member 3	---	694005	MmugDNA.18588.1.S1_at_ING3
MmugDNA.18589.1.S1_at	242417_at	LOC283278	hypothetical protein LOC283278	chr11p15.1	283278	---	---	---	---	MmugDNA.18589.1.S1_at_LOC283278
MmugDNA.18590.1.S1_at	208374_s_at	CAPZA1	capping protein (actin filament) muscle Z-line, alpha 1	chr1p13.2	829	LOC699124	Hypothetical protein LOC699124	---	699124	MmugDNA.18590.1.S1_at_CAPZA1
MmugDNA.18591.1.S1_at	214393_at	RND2	Rho family GTPase 2	chr17q21	8153	---	---	---	---	MmugDNA.18591.1.S1_at_RND2
MmugDNA.1859.1.S1_at	241505_at	---	---	---	---	---	---	---	---	MmugDNA.1859.1.S1_at_---
MmugDNA.18592.1.S1_at	232124_at	LOC729085	hypothetical protein LOC729085	chr3p22.1	729085	---	---	---	---	MmugDNA.18592.1.S1_at_LOC729085
MmugDNA.18593.1.S1_at	205061_s_at	EXOSC9	exosome component 9	chr4q27	5393	EXOSC9	exosome component 9	---	707368	MmugDNA.18593.1.S1_at_EXOSC9
MmugDNA.18594.1.S1_at	202635_s_at	POLR2K	polymerase (RNA) II (DNA directed) polypeptide K, 7.0kDa	chr8q22.2	5440	POLR2K	polymerase (RNA) II (DNA directed) polypeptide K, 7.0kDa	---	708464	MmugDNA.18594.1.S1_at_POLR2K
MmugDNA.18595.1.S1_at	203782_s_at	POLRMT	polymerase (RNA) mitochondrial (DNA directed)	chr19p13.3	5442	---	---	---	---	MmugDNA.18595.1.S1_at_POLRMT
MmugDNA.18596.1.S1_at	1563945_at	LOC284100	hypothetical protein LOC284100	chr17q12	284100	---	---	---	---	MmugDNA.18596.1.S1_at_LOC284100
MmugDNA.18597.1.S1_at	206313_at	HLA-DOA	major histocompatibility complex, class II, DO alpha	chr6p21.3	3111	LOC717947	similar to HLA class II histocompatibility antigen, DO alpha chain precursor (MHC class II antigen DO	---	717947	MmugDNA.18597.1.S1_at_HLA-DOA
MmugDNA.18598.1.S1_at	1563078_at	---	---	---	---	---	---	---	---	MmugDNA.18598.1.S1_at_---
MmugDNA.18599.1.S1_at	215478_at	RIMS2	regulating synaptic membrane exocytosis 2	chr8q22.3	9699	---	---	---	---	MmugDNA.18599.1.S1_at_RIMS2
MmugDNA.18600.1.S1_at	211923_s_at	ZNF471	zinc finger protein 471	chr19q13.43	57573	LOC706874	similar to zinc finger protein 471	---	706874	MmugDNA.18600.1.S1_at_ZNF471
MmugDNA.18601.1.S1_at	201137_s_at	HLA-DPB1	major histocompatibility complex, class II, DP beta 1	chr6p21.3	3115	MAMU-DPB	major histocompatibility complex, class II, DP beta	---	717969	MmugDNA.18601.1.S1_at_HLA-DPB1
MmugDNA.1860.1.S1_at	221266_s_at	TM7SF4	transmembrane 7 superfamily member 4	chr8q23	81501	TM7SF4	transmembrane 7 superfamily member 4	---	695237	MmugDNA.1860.1.S1_at_TM7SF4
MmugDNA.18602.1.S1_s_at	220892_s_at	PSAT1	phosphoserine aminotransferase 1	chr9q21.2	29968	LOC700836	Similar to acidic ribosomal phosphoprotein P0	---	700836	MmugDNA.18602.1.S1_s_at_PSAT1
MmugDNA.18603.1.S1_at	203290_at	HLA-DQA1	major histocompatibility complex, class II, DQ alpha 1	chr6p21.3	3117	LOC695437 /// LOC7052	Similar to C03A3.3 /// Similar to peptidylprolyl isomerase A isoform 1	---	695437 /// 705266	MmugDNA.18603.1.S1_at_HLA-DQA1
MmugDNA.18604.1.S1_at	242482_at	PRKAR1A	protein kinase, cAMP-dependent, regulatory, type I, alpha (tissue specific extinguisher 1)	chr17q23-q24	5573	PRKAR1A /// PRKAR1A	similar to cAMP-dependent protein kinase type I-alpha regulatory subunit (Tissue-specific extinguishe	---	707275 /// 719009	MmugDNA.18604.1.S1_at_PRKAR1A
MmugDNA.18605.1.S1_at	NA	NA	NA	NA	NA	NA	NA	NA	NA	MmugDNA.18605.1.S1_at_NA
MmugDNA.18606.1.S1_at	241994_at	XDH	xanthine dehydrogenase	chr2p23.1	7498	---	---	---	---	MmugDNA.18606.1.S1_at_XDH
MmugDNA.18607.1.S1_at	225250_at	STIM2	stromal interaction molecule 2	chr4p15.2	57620	STIM2	stromal interaction molecule 2	---	694980	MmugDNA.18607.1.S1_at_STIM2
MmugDNA.18608.1.S1_at	236690_at	RHBDD1	Rhomboid domain containing 1	chr2q36.3	84236	---	---	---	---	MmugDNA.18608.1.S1_at_RHBDD1
MmugDNA.18609.1.S1_at	214166_at	SDHB	succinate dehydrogenase complex, subunit B, iron sulfur (Ip)	chr1p36.1-p35	6390	SDHB	succinate dehydrogenase complex, subunit B, iron sulfur (Ip)	---	699810 /// 705183	MmugDNA.18609.1.S1_at_SDHB
MmugDNA.18610.1.S1_at	230710_at	---	---	---	---	---	---	---	---	MmugDNA.18610.1.S1_at_---
MmugDNA.18611.1.S1_at	NA	NA	NA	NA	NA	NA	NA	NA	NA	MmugDNA.18611.1.S1_at_NA
MmugDNA.18611.1.S1_x_at	204185_x_at	PPID	peptidylprolyl isomerase D	chr4q31.3	5481	LOC701677	Similar to NMD3 homolog	---	701677	MmugDNA.18611.1.S1_x_at_PPID
MmugDNA.1861.1.S1_at	221449_s_at	ITFG1	integrin alpha FG-GAP repeat containing 1	chr16q12.1	81533	LOC716435	similar to T-cell immunomodulatory protein	---	716435	MmugDNA.1861.1.S1_at_ITFG1
MmugDNA.18612.1.S1_at	225212_at	SLC25A25	solute carrier family 25 (mitochondrial carrier; phosphate carrier), member 25	chr9q34.11	114789	LOC698216	similar to mitochondrial Ca2+-dependent solute carrier	---	698216	MmugDNA.18612.1.S1_at_SLC25A25
MmugDNA.18613.1.S1_at	NA	NA	NA	NA	NA	NA	NA	NA	NA	MmugDNA.18613.1.S1_at_NA
MmugDNA.18614.1.S1_at	201594_s_at	PPP4R1	protein phosphatase 4, regulatory subunit 1	chr18p11.22	9989	PPP4R1	protein phosphatase 4, regulatory subunit 1	---	705451	MmugDNA.18614.1.S1_at_PPP4R1
MmugDNA.18615.1.S1_at	212378_at	GART	phosphoribosylglycinamide formyltransferase, phosphoribosylglycinamide synthetase, phosphoribosyl	chr21q22.1|21q22.11	2618	GART	phosphoribosylglycinamide formyltransferase, phosphoribosylglycinamide synthetase, phosphoribosyl	---	704926	MmugDNA.18615.1.S1_at_GART
MmugDNA.18616.1.S1_at	232794_at	---	---	---	---	---	---	---	---	MmugDNA.18616.1.S1_at_---
MmugDNA.18617.1.S1_at	227157_at	CCDC111	coiled-coil domain containing 111	chr4q35.1	201973	LOC694608	hypothetical protein LOC694608	---	694608	MmugDNA.18617.1.S1_at_CCDC111
MmugDNA.18618.1.S1_at	216228_s_at	WDHD1	WD repeat and HMG-box DNA binding protein 1	chr14q22.3	11169	WDHD1	WD repeat and HMG-box DNA binding protein 1	---	695910	MmugDNA.18618.1.S1_at_WDHD1
MmugDNA.186.1.S1_at	210706_s_at	RNF24	ring finger protein 24	chr20p13-p12.1	11237	LOC717693	similar to RING finger protein 24	---	717693	MmugDNA.186.1.S1_at_RNF24
MmugDNA.18620.1.S1_at	1563692_at	---	---	---	---	---	---	---	---	MmugDNA.18620.1.S1_at_---
MmugDNA.18621.1.S1_at	220727_at	KCNK10	potassium channel, subfamily K, member 10	chr14q31.3	54207	LOC694669	similar to potassium channel, subfamily K, member 10 isoform 3	---	694669	MmugDNA.18621.1.S1_at_KCNK10
MmugDNA.1862.1.S1_at	221268_s_at	SGPP1	sphingosine-1-phosphate phosphatase 1	chr14q23.2	81537	LOC709364	similar to sphingosine-1-phosphatase	---	709364	MmugDNA.1862.1.S1_at_SGPP1
MmugDNA.18622.1.S1_at	NA	NA	NA	NA	NA	NA	NA	NA	NA	MmugDNA.18622.1.S1_at_NA
MmugDNA.18622.1.S1_s_at	NA	NA	NA	NA	NA	NA	NA	NA	NA	MmugDNA.18622.1.S1_s_at_NA
MmugDNA.18623.1.S1_at	236637_at	---	---	---	---	---	---	---	---	MmugDNA.18623.1.S1_at_---
MmugDNA.18624.1.S1_at	208503_s_at	GATAD1	GATA zinc finger domain containing 1	chr7q21-q22	57798	ODAG	ocular development associated	---	700726	MmugDNA.18624.1.S1_at_GATAD1
MmugDNA.18625.1.S1_s_at	214756_x_at	PMS2L1	postmeiotic segregation increased 2-like 1 pseudogene	chr7q22.1	5379	LOC717589	similar to PMS1 protein homolog 2 (DNA mismatch repair protein PMS2)	---	717589	MmugDNA.18625.1.S1_s_at_PMS2L1
MmugDNA.18626.1.S1_at	207566_at	MR1	major histocompatibility complex, class I-related	chr1q25.3	3140	LOC699759	Similar to zinc finger protein 33B	---	699759	MmugDNA.18626.1.S1_at_MR1
MmugDNA.18627.1.S1_at	207833_s_at	HLCS	holocarboxylase synthetase (biotin-(proprionyl-Coenzyme A-carboxylase (ATP-hydrolysing)) ligase)	chr21q22.1|21q22.13	3141	HLCS	holocarboxylase synthetase	---	694733	MmugDNA.18627.1.S1_at_HLCS
MmugDNA.18628.1.S1_at	234963_s_at	FA2H	fatty acid 2-hydroxylase	chr16q23	79152	CRP	C-reactive protein, pentraxin-related	---	719454	MmugDNA.18628.1.S1_at_FA2H
MmugDNA.18629.1.S1_at	242072_at	---	---	---	---	---	---	---	---	MmugDNA.18629.1.S1_at_---
MmugDNA.18630.1.S1_at	202996_at	POLD4	polymerase (DNA-directed), delta 4	chr11q13	57804	POLD4	polymerase (DNA-directed), delta 4	---	712632	MmugDNA.18630.1.S1_at_POLD4
MmugDNA.18631.1.S1_at	228300_at	CCDC103	coiled-coil domain containing 103	chr17q21.31	388389	LOC715661	hypothetical protein LOC715661	---	715661	MmugDNA.18631.1.S1_at_CCDC103
MmugDNA.1863.1.S1_at	232851_at	FBXO3	F-box protein 3	chr11p13	26273	---	---	---	---	MmugDNA.1863.1.S1_at_FBXO3
MmugDNA.18632.1.S1_at	232301_at	UBE3B	ubiquitin protein ligase E3B	chr12q24.11	89910	---	---	---	---	MmugDNA.18632.1.S1_at_UBE3B
MmugDNA.18633.1.S1_at	243661_at	ZNF273	zinc finger protein 273	chr7q11.21	10793	---	---	---	---	MmugDNA.18633.1.S1_at_ZNF273
MmugDNA.18634.1.S1_at	205082_s_at	AOX1	aldehyde oxidase 1	chr2q33	316	LOC707078	Similar to peptidylprolyl isomerase A isoform 1	---	707078	MmugDNA.18634.1.S1_at_AOX1
MmugDNA.18634.1.S1_s_at	NA	NA	NA	NA	NA	NA	NA	NA	NA	MmugDNA.18634.1.S1_s_at_NA
MmugDNA.18635.1.S1_at	218226_s_at	NDUFB4	NADH dehydrogenase (ubiquinone) 1 beta subcomplex, 4, 15kDa	chr3q13.33	4710	LOC713428	similar to NADH dehydrogenase (ubiquinone) 1 beta subcomplex, 4, 15kDa	---	713428	MmugDNA.18635.1.S1_at_NDUFB4
MmugDNA.18636.1.S1_s_at	229711_s_at	MDM2	Mdm2 p53 binding protein homolog (mouse)	chr12q14.3-q15	4193	LOC718298	similar to Probable ribosome biogenesis protein RLP24	---	718298	MmugDNA.18636.1.S1_s_at_MDM2
MmugDNA.18637.1.S1_at	1562121_at	---	---	---	---	---	---	---	---	MmugDNA.18637.1.S1_at_---
MmugDNA.18638.1.S1_s_at	NA	NA	NA	NA	NA	NA	NA	NA	NA	MmugDNA.18638.1.S1_s_at_NA
MmugDNA.18639.1.S1_at	1564323_at	---	---	---	---	---	---	---	---	MmugDNA.18639.1.S1_at_---
MmugDNA.18640.1.S1_at	NA	NA	NA	NA	NA	NA	NA	NA	NA	MmugDNA.18640.1.S1_at_NA
MmugDNA.18641.1.S1_at	1561450_at	---	---	---	---	---	---	---	---	MmugDNA.18641.1.S1_at_---
MmugDNA.1864.1.S1_at	237107_at	PRKRA /// PRKRAP1	protein kinase, interferon-inducible double stranded RNA dependent activator /// protein kinase, interf	chr2q31.2	731716 /// 8575	LOC709385 /// PRKRA	similar to protein kinase, interferon-inducible double stranded RNA dependent activator /// protein kin	---	703063 /// 709385	MmugDNA.1864.1.S1_at_PRKRA /// PR
MmugDNA.18642.1.S1_at	233808_at	---	---	---	---	---	---	---	---	MmugDNA.18642.1.S1_at_---
MmugDNA.18643.1.S1_s_at	201526_at	ARF5	ADP-ribosylation factor 5	chr7q31.3	381	LOC699426	similar to ADP-ribosylation factor 5	---	699426	MmugDNA.18643.1.S1_s_at_ARF5
MmugDNA.18644.1.S1_at	221070_s_at	KIAA1967	KIAA1967	chr8p22	57805	LOC716288	similar to p30 DBC protein	---	716288	MmugDNA.18644.1.S1_at_KIAA1967
MmugDNA.18645.1.S1_at	213591_at	ALDH7A1	aldehyde dehydrogenase 7 family, member A1	chr5q31	501	ALDH7A1	aldehyde dehydrogenase 7 family, member A1	---	702749	MmugDNA.18645.1.S1_at_ALDH7A1
MmugDNA.18646.1.S1_at	NA	NA	NA	NA	NA	NA	NA	NA	NA	MmugDNA.18646.1.S1_at_NA
MmugDNA.18646.1.S1_s_at	236970_s_at	BCAP29	B-cell receptor-associated protein 29	chr7q22-q31	55973	---	---	---	---	MmugDNA.18646.1.S1_s_at_BCAP29
MmugDNA.18647.1.S1_s_at	220491_at	HAMP	hepcidin antimicrobial peptide	chr19q13.1	57817	HAMP	hepcidin antimicrobial peptide	---	708397	MmugDNA.18647.1.S1_s_at_HAMP
MmugDNA.18648.1.S1_at	235356_at	NHLRC2	NHL repeat containing 2	chr10q25.3	374354	NHLRC2	NHL repeat containing 2	---	698009	MmugDNA.18648.1.S1_at_NHLRC2
MmugDNA.18649.1.S1_at	232256_s_at	LOC401321	hypothetical LOC401321	chr7p14.3	401321	---	---	---	---	MmugDNA.18649.1.S1_at_LOC401321
MmugDNA.18650.1.S1_at	221453_at	G6PC2	glucose-6-phosphatase, catalytic, 2	chr2q24.3	57818	LOC719987	similar to glucose-6-phosphatase, catalytic, 2	---	719987	MmugDNA.18650.1.S1_at_G6PC2
MmugDNA.18651.1.S1_at	205822_s_at	HMGCS1	3-hydroxy-3-methylglutaryl-Coenzyme A synthase 1 (soluble)	chr5p14-p13	3157	LOC699853	Similar to WD repeat domain 49	---	699853	MmugDNA.18651.1.S1_at_HMGCS1

MmugDNA.1865.1.S1_at	213300_at	ATG2A	ATG2 autophagy related 2 homolog A (S. cerevisiae)	chr11q13.1		23130	LOC717290	similar to Autophagy-specific gene 2 CG1241-PA	---		717290	MmugDNA.1865.1.S1_at_ATG2A
MmugDNA.18652.1.S1_at	NA	NA	NA	NA	NA		NA	NA	NA	NA		MmugDNA.18652.1.S1_at_NA
MmugDNA.18653.1.S1_at	217988_at	CCNB1IP1	cyclin B1 interacting protein 1	chr14q11.2		57820	CCNB1IP1	cyclin B1 interacting protein 1	---		703502	MmugDNA.18653.1.S1_at_CCNB1IP1
MmugDNA.18654.1.S1_at	213340_s_at	KIAA0495	KIAA0495	chr1p36.32		57212	---	---	---	---		MmugDNA.18654.1.S1_at_KIAA0495
MmugDNA.18655.1.S1_at	222843_at	FIGNL1	fidgetin-like 1	chr7p12.2		63979	---	---	---	---		MmugDNA.18655.1.S1_at_FIGNL1
MmugDNA.18656.1.S1_at	208480_s_at	ABCC6	ATP-binding cassette, sub-family C (CFTR/MRP), member 6	chr16p13.1		368	LOC717875	similar to ATP-binding cassette, sub-family C, member 6	---		717875	MmugDNA.18656.1.S1_at_ABCC6
MmugDNA.18657.1.S1_s_at	208220_x_at	AMELY	amelogenin, Y-linked	chrYp11.2		266	AMELX	amelogenin	---		710057	MmugDNA.18657.1.S1_s_at_AMELY
MmugDNA.18658.1.S1_at	202425_x_at	PPP3CA	protein phosphatase 3 (formerly 2B), catalytic subunit, alpha isoform	chr4q21-q24		5530	HR	Hairless	---		574164	MmugDNA.18658.1.S1_at_PPP3CA
MmugDNA.18659.1.S1_at	209220_at	GPC3	glypican 3	chrXq26.1		2719	GPC3	glypican 3	---		706773	MmugDNA.18659.1.S1_at_GPC3
MmugDNA.18660.1.S1_at	232774_x_at	ZIK1	zinc finger protein interacting with K protein 1 homolog (mouse)	chr19q13.43		284307	LOC708621	similar to zinc finger protein interacting with K protein 1	---		708621	MmugDNA.18660.1.S1_at_ZIK1
MmugDNA.18661.1.S1_at	216645_at	---	---	---	---		---	---	---	---		MmugDNA.18661.1.S1_at_---
MmugDNA.1866.1.S1_at	212213_x_at	OPA1	optic atrophy 1 (autosomal dominant)	chr3q28-q29|3q28-q29		4976	OPA1	optic atrophy 1 (autosomal dominant)	---		698540	MmugDNA.1866.1.S1_at_OPA1
MmugDNA.18662.1.S1_at	206772_at	PTH2R	parathyroid hormone 2 receptor	chr2q33		5746	PTH2R	parathyroid hormone 2 receptor	---		710383	MmugDNA.18662.1.S1_at_PTH2R
MmugDNA.18663.1.S1_at	228850_s_at	---	---	---	---		---	---	---	---		MmugDNA.18663.1.S1_at_---
MmugDNA.18664.1.S1_at	203982_s_at	ABCD4	ATP-binding cassette, sub-family D (ALD), member 4	chr14q24.3		5826	ABCD4	ATP-binding cassette, sub-family D (ALD), member 4	---		699380	MmugDNA.18664.1.S1_at_ABCD4
MmugDNA.18665.1.S1_at	217846_at	QARS	glutaminyl-tRNA synthetase	chr3p21.3-p21.1		5859	LOC696591	Similar to hematological and neurological expressed 1 isoform 2	---		696591	MmugDNA.18665.1.S1_at_QARS
MmugDNA.18666.1.S1_at	235592_at	---	---	---	---		---	---	---	---		MmugDNA.18666.1.S1_at_---
MmugDNA.18667.1.S1_at	206074_s_at	HMGA1	high mobility group AT-hook 1	chr6p21		3159	LOC718536	similar to high mobility group AT-hook 1	---		718536	MmugDNA.18667.1.S1_at_HMGA1
MmugDNA.18668.1.S1_at	208302_at	HMHB1	histocompatibility (minor) HB-1	chr5q32		57824	---	---	---	---		MmugDNA.18668.1.S1_at_HMHB1
MmugDNA.18669.1.S1_at	228147_at	---	---	---	---		---	---	---	---		MmugDNA.18669.1.S1_at_---
MmugDNA.18670.1.S1_s_at	204968_at	C6orf47	chromosome 6 open reading frame 47	chr6p21.3		57827	LOC715966	similar to Protein C6orf47 (Protein G4)	---		715966	MmugDNA.18670.1.S1_s_at_C6orf47
MmugDNA.18671.1.S1_at	224631_at	ZFP91	zinc finger protein 91 homolog (mouse)	chr11q12		80829	---	---	---	---		MmugDNA.18671.1.S1_at_ZFP91
MmugDNA.1867.1.S1_at	216867_s_at	PDGFA	platelet-derived growth factor alpha polypeptide	chr7p22		5154	PDGFA	platelet-derived growth factor alpha polypeptide	---		707725	MmugDNA.1867.1.S1_at_PDGFA
MmugDNA.18672.1.S1_at	211918_x_at	PAPPA2	pappalysin 2	chr1q23-q25		60676	PAPPA2	pappalysin 2	---		709193	MmugDNA.18672.1.S1_at_PAPPA2
MmugDNA.18673.1.S1_at	224636_at	ZFP91	zinc finger protein 91 homolog (mouse)	chr11q12		80829	LOC711250	Similar to ubiquitin associated domain containing 1	---		711250	MmugDNA.18673.1.S1_at_ZFP91
MmugDNA.18674.1.S1_at	1559065_a_at	CLEC4G	C-type lectin domain family 4, member G	chr19p13.2		339390	LOC704872	similar to C-type lectin superfamily 4, member G	---		704872	MmugDNA.18674.1.S1_at_CLEC4G
MmugDNA.18675.1.S1_at	232963_at	---	---	---	---		---	---	---	---		MmugDNA.18675.1.S1_at_---
MmugDNA.18676.1.S1_at	232688_at	BMP2K	BMP2 inducible kinase	chr4q21.21		55589	---	---	---	---		MmugDNA.18676.1.S1_at_BMP2K
MmugDNA.18677.1.S1_at	237004_at	---	---	---	---		---	---	---	---		MmugDNA.18677.1.S1_at_---
MmugDNA.18678.1.S1_at	240483_at	ZNF717	zinc finger protein 717	chr3p12.3		100131827	---	---	---	---		MmugDNA.18678.1.S1_at_ZNF717
MmugDNA.18679.1.S1_at	244018_at	---	---	---	---		---	---	---	---		MmugDNA.18679.1.S1_at_---
MmugDNA.18680.1.S1_at	238382_x_at	---	---	---	---		---	---	---	---		MmugDNA.18680.1.S1_at_---
MmugDNA.18681.1.S1_at	206103_at	RAC3	ras-related C3 botulinum toxin substrate 3 (rho family, small GTP binding protein Rac3)	chr17q25.3		5881	LOC719305	similar to RAS-related C3 botulinum substrate 3	---		719305	MmugDNA.18681.1.S1_at_RAC3
MmugDNA.1868.1.S1_at	215092_s_at	NFAT5	nuclear factor of activated T-cells 5, tonicity-responsive	chr16q22.1		10725	NFAT5	nuclear factor of activated T-cells 5, tonicity-responsive	---		705518	MmugDNA.1868.1.S1_at_NFAT5
MmugDNA.18682.1.S1_at	201046_s_at	RAD23A	RAD23 homolog A (S. cerevisiae)	chr19p13.2		5886	LOC720675	similar to UV excision repair protein RAD23 homolog A (mHR23A)	---		720675	MmugDNA.18682.1.S1_at_RAD23A
MmugDNA.18683.1.S1_at	243475_at	CBL	Cas-Br-M (murine) ecotropic retroviral transforming sequence	chr11q23.3		867	---	---	---	---		MmugDNA.18683.1.S1_at_CBL
MmugDNA.18684.1.S1_at	230200_at	NSUN6	NOL1/NOP2/Sun domain family, member 6	chr10p12.33		221078	LOC701490	similar to NOL1/NOP2/Sun domain family, member 6	---		701490	MmugDNA.18684.1.S1_at_NSUN6
MmugDNA.18685.1.S1_at	NA	NA	NA	NA	NA		NA	NA	NA	NA		MmugDNA.18685.1.S1_at_NA
MmugDNA.18685.1.S1_s_at	202340_x_at	NR4A1	nuclear receptor subfamily 4, group A, member 1	chr12q13		3164	NR4A1	nuclear receptor subfamily 4, group A, member 1	---		696797	MmugDNA.18685.1.S1_s_at_NR4A1
MmugDNA.18686.1.S1_at	237013_at	---	---	---	---		---	---	---	---		MmugDNA.18686.1.S1_at_---
MmugDNA.18687.1.S1_at	205169_at	RBBP5	retinoblastoma binding protein 5	chr1q32		5929	LOC699057	similar to retinoblastoma binding protein 5	---		699057	MmugDNA.18687.1.S1_at_RBBP5
MmugDNA.18688.1.S1_at	229886_at	C5orf34	chromosome 5 open reading frame 34	chr5p12		375444	LOC700618	hypothetical protein LOC700618	---		700618	MmugDNA.18688.1.S1_at_C5orf34
MmugDNA.18689.1.S1_at	208429_x_at	HNF4A	hepatocyte nuclear factor 4, alpha	chr20q12-q13.1		3172	NOS3	Nitric oxide synthase 3 (endothelial cell)	---		714231	MmugDNA.18689.1.S1_at_HNF4A
MmugDNA.18690.1.S1_at	206153_at	CYP4F11	cytochrome P450, family 4, subfamily F, polypeptide 11	chr19p13.1		57834	LOC719353	similar to Cytochrome P450 4F11 (CYPIVF11)	---		719353	MmugDNA.18690.1.S1_at_CYP4F11
MmugDNA.18691.1.S1_at	NA	NA	NA	NA	NA		NA	NA	NA	NA		MmugDNA.18691.1.S1_at_NA
MmugDNA.18691.1.S1_x_at	NA	NA	NA	NA	NA		NA	NA	NA	NA		MmugDNA.18691.1.S1_x_at_NA
MmugDNA.1869.1.S1_at	228242_at	N4BP2	NEDD4 binding protein 2	chr4p14		55728	---	---	---	---		MmugDNA.1869.1.S1_at_N4BP2
MmugDNA.18692.1.S1_at	NA	NA	NA	NA	NA		NA	NA	NA	NA		MmugDNA.18692.1.S1_at_NA
MmugDNA.18692.1.S1_x_at	214120_at	RFPL1S	RFPL1 antisense RNA (non-protein coding)	chr22q12		10740	---	---	---	---		MmugDNA.18692.1.S1_x_at_RFPL1S
MmugDNA.18693.1.S1_at	216813_at	---	---	---	---		---	---	---	---		MmugDNA.18693.1.S1_at_---
MmugDNA.18694.1.S1_at	1555814_a_at	RHOA	ras homolog gene family, member A	chr3p21.3		387	---	---	---	---		MmugDNA.18694.1.S1_at_RHOA
MmugDNA.18695.1.S1_at	202010_s_at	ZNF410	zinc finger protein 410	chr14q24.3		57862	LOC697978	Hypothetical protein LOC697978	---		697978	MmugDNA.18695.1.S1_at_ZNF410
MmugDNA.18696.1.S1_at	205292_s_at	HNRNPA2B1	heterogeneous nuclear ribonucleoprotein A2/B1	chr7p15		3181	LOC702053	hypothetical protein LOC702053	---		702053	MmugDNA.18696.1.S1_at_HNRNPA2B
MmugDNA.18697.1.S1_at	231097_at	---	---	---	---		---	---	---	---		MmugDNA.18697.1.S1_at_---
MmugDNA.18698.1.S1_at	218683_at	PTBP2	polypyrimidine tract binding protein 2	chr1p22.1-p21.3		58155	LOC710101	similar to polypyrimidine tract binding protein 2	---		710101	MmugDNA.18698.1.S1_at_PTBP2
MmugDNA.18699.1.S1_at	210328_at	GNMT	glycine N-methyltransferase	chr6p12		27232	GNMT	glycine N-methyltransferase	---		697722	MmugDNA.18699.1.S1_at_GNMT
MmugDNA.18700.1.S1_at	205183_at	HNRNPD	heterogeneous nuclear ribonucleoprotein D (AU-rich element RNA binding protein 1, 37kDa)	chr4q21.1-q21.2		3184	---	---	---	---		MmugDNA.18700.1.S1_at_HNRNPD
MmugDNA.18701.1.S1_at	237507_at	KRT73	keratin 73	chr12q13.3		319101	LOC699452	similar to keratin 6 irs3	---		699452	MmugDNA.18701.1.S1_at_KRT73
MmugDNA.18702.1.S1_at	230179_at	LOC285812	hypothetical protein LOC285812	chr6p23		285812	---	---	---	---		MmugDNA.18702.1.S1_at_LOC285812
MmugDNA.18703.1.S1_at	239692_at	---	---	---	---		---	---	---	---		MmugDNA.18703.1.S1_at_---
MmugDNA.18704.1.S1_at	205357_s_at	AGTR1	angiotensin II receptor, type 1	chr3q21-q25		185	LOC712773	similar to angiotensin II receptor, type 1	---		712773	MmugDNA.18704.1.S1_at_AGTR1
MmugDNA.18705.1.S1_at	234932_s_at	CDCP1	CUB domain containing protein 1	chr3p21.31		64866	LOC714333	similar to CUB domain-containing protein 1 isoform 1	---		714333	MmugDNA.18705.1.S1_at_CDCP1
MmugDNA.18706.1.S1_at	NA	NA	NA	NA	NA		NA	NA	NA	NA		MmugDNA.18706.1.S1_at_NA
MmugDNA.18706.1.S1_s_at	238853_at	RAB3IP	RAB3A interacting protein (rabin3)	chr12q14.3		117177	---	---	---	---		MmugDNA.18706.1.S1_s_at_RAB3IP
MmugDNA.18707.1.S1_at	208212_s_at	ALK	anaplastic lymphoma receptor tyrosine kinase	chr2p23		238	ALK	anaplastic lymphoma receptor tyrosine kinase	---		704601	MmugDNA.18707.1.S1_at_ALK
MmugDNA.18708.1.S1_at	238272_at	TPMT	Thiopurine S-methyltransferase	chr6p22.3		7172	---	---	---	---		MmugDNA.18708.1.S1_at_TPMT
MmugDNA.18709.1.S1_at	227319_at	---	---	---	---		LOC715423	similar to Ring canal kelch protein	---		715423	MmugDNA.18709.1.S1_at_---
MmugDNA.18711.1.S1_at	237989_at	IGFBP1	Insulin-like growth factor binding protein 1	chr7p13-p12		3484	---	---	---	---		MmugDNA.18711.1.S1_at_IGFBP1
MmugDNA.1871.1.S1_at	216790_at	---	---	---	---		---	---	---	---		MmugDNA.1871.1.S1_at_---
MmugDNA.18712.1.S1_at	217857_s_at	RBM8A	RNA binding motif protein 8A	chr1q12		9939	LDHA	Similar to L-lactate dehydrogenase A chain (LDH-A) (LDH muscle subunit) (LDH-M) (Proliferation-ind	---		693898	MmugDNA.18712.1.S1_at_RBM8A
MmugDNA.18713.1.S1_at	211886_s_at	TBX5	T-box 5	chr12q24.1		6910	TBX5	T-box 5	---		713550	MmugDNA.18713.1.S1_at_TBX5
MmugDNA.18714.1.S1_at	231094_s_at	MTHFD1L	methylenetetrahydrofolate dehydrogenase (NADP+ dependent) 1-like	chr6q25.1		25902	---	---	---	---		MmugDNA.18714.1.S1_at_MTHFD1L
MmugDNA.18715.1.S1_at	205333_s_at	RCE1	RCE1 homolog, prenyl protein peptidase (S. cerevisiae)	chr11q13		9986	RCE1	RCE1 homolog, prenyl protein peptidase (S. cerevisiae)	---		713224	MmugDNA.18715.1.S1_at_RCE1
MmugDNA.18716.1.S1_at	203588_s_at	TFDP2	transcription factor Dp-2 (E2F dimerization partner 2)	chr3q23		7029	TFDP2	transcription factor Dp-2 (E2F dimerization partner 2)	---		714655	MmugDNA.18716.1.S1_at_TFDP2
MmugDNA.18717.1.S1_at	219645_at	CASQ1	calsequestrin 1 (fast-twitch, skeletal muscle)	chr1q21		844	LOC720233	similar to skeletal muscle calsequestrin 1	---		720233	MmugDNA.18717.1.S1_at_CASQ1
MmugDNA.18718.1.S1_at	240207_at	---	---	---	---		---	---	---	---		MmugDNA.18718.1.S1_at_---
MmugDNA.18719.1.S1_at	1569337_at	SLC5A9	solute carrier family 5 (sodium/glucose cotransporter), member 9	chr1p33		200010	---	---	---	---		MmugDNA.18719.1.S1_at_SLC5A9
MmugDNA.187.1.S1_at	210791_s_at	RICS	Rho GTPase-activating protein	chr11q24-q25		9743	LOC718464	similar to Rho GTPase-activating protein	---		718464	MmugDNA.187.1.S1_at_RICS
MmugDNA.18721.1.S1_at	1557203_at	PABPC1L2B	poly(A) binding protein, cytoplasmic 1-like 2B	chrXq13.1		645974	LOC705906	similar to poly(A) binding protein, cytoplasmic 1	---		705906	MmugDNA.18721.1.S1_at_PABPC1L2B
MmugDNA.1872.1.S1_at	239745_at	---	---	---	---		---	---	---	---		MmugDNA.1872.1.S1_at_---
MmugDNA.18722.1.S1_at	207317_s_at	CASQ2	calsequestrin 2 (cardiac muscle)	chr1p13.3-p11		845	CASQ2	calsequestrin 2 (cardiac muscle)	---		712373	MmugDNA.18722.1.S1_at_CASQ2
MmugDNA.18723.1.S1_at	242626_at	SAMD5	sterile alpha motif domain containing 5	chr6q24.3		389432	---	---	---	---		MmugDNA.18723.1.S1_at_SAMD5
MmugDNA.18724.1.S1_at	206233_at	B4GALT6	UDP-Gal:betaGlcNAc beta 1,4- galactosyltransferase, polypeptide 6	chr18q11		9331	B4GALT6	UDP-Gal:betaGlcNAc beta 1,4- galactosyltransferase, polypeptide 6	---		706068	MmugDNA.18724.1.S1_at_B4GALT6
MmugDNA.18724.1.S1_s_at	NA	NA	NA	NA	NA		NA	NA	NA	NA		MmugDNA.18724.1.S1_s_at_NA
MmugDNA.18724.1.S1_x_at	NA	NA	NA	NA	NA		NA	NA	NA	NA		MmugDNA.18724.1.S1_x_at_NA
MmugDNA.18725.1.S1_at	206289_at	HOXA4	homeobox A4	chr7p15-p14		3201	---	---	---	---		MmugDNA.18725.1.S1_at_HOXA4
MmugDNA.18726.1.S1_at	237774_at	---	---	---	---		---	---	---	---		MmugDNA.18726.1.S1_at_---
MmugDNA.18727.1.S1_at	241636_x_at	---	---	---	---		---	---	---	---		MmugDNA.18727.1.S1_at_---
MmugDNA.18728.1.S1_at	230174_at	LYPLAL1	lysophospholipase-like 1	chr1q41		127018	TMEM33	Transmembrane protein 33	---		702063	MmugDNA.18728.1.S1_at_LYPLAL1
MmugDNA.18729.1.S1_at	238098_at	---	---	---	---		---	---	---	---		MmugDNA.18729.1.S1_at_---
MmugDNA.18730.1.S1_at	203356_at	CAPN7	calpain 7	chr3p24		23473	---	---	---	---		MmugDNA.18730.1.S1_at_CAPN7
MmugDNA.18731.1.S1_at	238846_at	TNFRSF11A	tumor necrosis factor receptor superfamily, member 11a, NFKB activator	chr18q22.1		8792	---	---	---	---		MmugDNA.18731.1.S1_at_TNFRSF11A
MmugDNA.1873.1.S1_at	228448_at	MAP6	microtubule-associated protein 6	chr11q13.5		4135	---	---	---	---		MmugDNA.1873.1.S1_at_MAP6
MmugDNA.18732.1.S1_at	207896_s_at	DLEC1	deleted in lung and esophageal cancer 1	chr3p22-p21.3		9940	LOC697163	hypothetical protein LOC697163	---		697163	MmugDNA.18732.1.S1_at_DLEC1
MmugDNA.18733.1.S1_at	226215_s_at	KDM2B	lysine (K)-specific demethylase 2B	chr12q24.31		84678	KDM2B	lysine (K)-specific demethylase 2B	---		699938	MmugDNA.18733.1.S1_at_KDM2B
MmugDNA.18734.1.S1_at	221314_at	GDF9	growth differentiation factor 9	chr5q31.1		2661	---	---	---	---		MmugDNA.18734.1.S1_at_GDF9
MmugDNA.18735.1.S1_at	201567_s_at	GOLGA4	golgi autoantigen, golgin subfamily a, 4	chr3p22-p21.3		2803	---	---	---	---		MmugDNA.18735.1.S1_at_GOLGA4
MmugDNA.18737.1.S1_at	237849_at	---	---	---	---		---	---	---	---		MmugDNA.18737.1.S1_at_---
MmugDNA.18738.1.S1_at	229226_at	---	---	---	---		---	---	---	---		MmugDNA.18738.1.S1_at_---
MmugDNA.18739.1.S1_at	207223_s_at	ROD1	ROD1 regulator of differentiation 1 (S. pombe)	chr9q32		9991	LOC711210	similar to ROD1 regulator of differentiation 1	---		711210	MmugDNA.18739.1.S1_at_ROD1
MmugDNA.18740.1.S1_at	NA	NA	NA	NA	NA		NA	NA	NA	NA		MmugDNA.18740.1.S1_at_NA
MmugDNA.18740.1.S1_s_at	203324_s_at	CAV2	caveolin 2	chr7q31.1		858	LOC710021	Similar to neuromedin S	---		710021	MmugDNA.18740.1.S1_s_at_CAV2
MmugDNA.18741.1.S1_at	227053_at	PACSIN1	protein kinase C and casein kinase substrate in neurons 1	chr6p21.3		29993	PACSIN1	protein kinase C and casein kinase substrate in neurons 1	---		718624	MmugDNA.18741.1.S1_at_PACSIN1
MmugDNA.1874.1.S1_at	NA	NA	NA	NA	NA		NA	NA	NA	NA		MmugDNA.1874.1.S1_at_NA
MmugDNA.1874.1.S1_s_at	1554482_a_at	SAR1B	SAR1 homolog B (S. cerevisiae)	chr5q31.1		51128	SAR1B	SAR1 homolog B (S. cerevisiae)	---		711269	MmugDNA.1874.1.S1_s_at_SAR1B
MmugDNA.18742.1.S1_at	215851_at	EVI1	ecotropic viral integration site 1	chr3q24-q28		2122	---	---	---	---		MmugDNA.18742.1.S1_at_EVI1
MmugDNA.18744.1.S1_at	206768_at	RPL3L	ribosomal protein L3-like	chr16p13.3		6123	LOC693424	similar to 60S ribosomal protein L3-like	---		693424	MmugDNA.18744.1.S1_at_RPL3L
MmugDNA.18745.1.S1_at	214994_at	APOBEC3F	apolipoprotein B mRNA editing enzyme, catalytic polypeptide-like 3F	chr22q13.1		200316	---	---	---	---		MmugDNA.18745.1.S1_at_APOBEC3F

MmugDNA.18746.1.S1_at	1557484_at	---	---	---	---		---	---	---	---	MmugDNA.18746.1.S1_at_---
MmugDNA.18747.1.S1_at	206488_s_at	CD36	CD36 molecule (thrombospondin receptor)	chr7q11.2		948	CD36	CD36 molecule (thrombospondin receptor)	---	574296	MmugDNA.18747.1.S1_at_CD36
MmugDNA.18748.1.S1_at	205453_at	HOXB2	homeobox B2	chr17q21-q22		3212	---	---	---	---	MmugDNA.18748.1.S1_at_HOXB2
MmugDNA.18749.1.S1_at	244349_at	---	---	---	---		---	---	---	---	MmugDNA.18749.1.S1_at_---
MmugDNA.18750.1.S1_at	215985_at	NCRNA00171	Non-protein coding RNA 171	chr6p21.33		80862	---	---	---	---	MmugDNA.18750.1.S1_at_NCRNA0017
MmugDNA.18751.1.S1_at	205601_s_at	HOXB5	homeobox B5	chr17q21.3		3215	HOXB5	homeobox B5	---	696732	MmugDNA.18751.1.S1_at_HOXB5
MmugDNA.1875.1.S1_at	209646_x_at	ALDH1B1	aldehyde dehydrogenase 1 family, member B1	chr9p11.1		219	LOC716092	similar to aldehyde dehydrogenase 1B1 precursor	---	716092	MmugDNA.1875.1.S1_at_ALDH1B1
MmugDNA.18752.1.S1_at	213116_at	NEK3	NIMA (never in mitosis gene a)-related kinase 3	chr13q14.13		4752	NEK3	NIMA (never in mitosis gene a)-related kinase 3	---	710127	MmugDNA.18752.1.S1_at_NEK3
MmugDNA.18753.1.S1_s_at	231948_s_at	UBE2F	ubiquitin-conjugating enzyme E2F (putative)	chr2q37.3		140739	LOC697992 /// UBE2F	similar to NEDD8-conjugating enzyme /// ubiquitin-conjugating enzyme E2F (putative)	---	695394 /// 697992	MmugDNA.18753.1.S1_s_at_UBE2F
MmugDNA.18754.1.S1_at	244289_at	LOC134466	zinc finger protein 300 pseudogene	chr5q33.1		134466	LOC712991	similar to zinc finger protein 300	---	712991	MmugDNA.18754.1.S1_at_LOC134466
MmugDNA.18755.1.S1_at	226740_x_at	NBPF1	Neuroblastoma breakpoint family, member 1	chr1p36.13		55672	---	---	---	---	MmugDNA.18755.1.S1_at_NBPF1
MmugDNA.18756.1.S1_at	209903_s_at	ATR	ataxia telangiectasia and Rad3 related	chr3q22-q24		545	---	---	---	---	MmugDNA.18756.1.S1_at_ATR
MmugDNA.18757.1.S1_at	225689_at	C3orf39	chromosome 3 open reading frame 39	chr3p22.1		84892	LOC717032	similar to glycosyltransferase	---	717032	MmugDNA.18757.1.S1_at_C3orf39
MmugDNA.18758.1.S1_at	242074_at	---	---	---	---		---	---	---	---	MmugDNA.18758.1.S1_at_---
MmugDNA.18759.1.S1_at	212834_at	DDX52	DEAD (Asp-Glu-Ala-Asp) box polypeptide 52	chr17q21.1		11056	---	---	---	---	MmugDNA.18759.1.S1_at_DDX52
MmugDNA.18760.1.S1_at	224749_at	ITFG3	integrin alpha FG-GAP repeat containing 3	chr16p13.3		83986	ITFG3	integrin alpha FG-GAP repeat containing 3	---	694729	MmugDNA.18760.1.S1_at_ITFG3
MmugDNA.18761.1.S1_at	213349_at	TMCC1	transmembrane and coiled-coil domain family 1	chr3q21.3		23023	TMCC1	transmembrane and coiled-coil domain family 1	---	702576	MmugDNA.18761.1.S1_at_TMCC1
MmugDNA.1876.1.S1_at	227163_at	GSTO2	glutathione S-transferase omega 2	chr10q25.1		119391	GSTO2	glutathione S-transferase omega 2	---	715291	MmugDNA.1876.1.S1_at_GSTO2
MmugDNA.18762.1.S1_at	213351_s_at	TMCC1	transmembrane and coiled-coil domain family 1	chr3q21.3		23023	TMCC1	transmembrane and coiled-coil domain family 1	---	702576	MmugDNA.18762.1.S1_at_TMCC1
MmugDNA.18763.1.S1_at	241482_at	---	---	---	---		---	---	---	---	MmugDNA.18763.1.S1_at_---
MmugDNA.18764.1.S1_at	242349_at	HECTD1	HECT domain containing 1	chr14q12		25831	LOC717177	similar to HECT domain containing 1	---	717177	MmugDNA.18764.1.S1_at_HECTD1
MmugDNA.18765.1.S1_at	1560851_at	C10orf136	chromosome 10 open reading frame 136	chr10q11.21		414260	---	---	---	---	MmugDNA.18765.1.S1_at_C10orf136
MmugDNA.18766.1.S1_at	207372_s_at	ENTPD2	ectonucleoside triphosphate diphosphohydrolase 2	chr9q34		954	ENTPD2	ectonucleoside triphosphate diphosphohydrolase 2	---	721617	MmugDNA.18766.1.S1_at_ENTPD2
MmugDNA.18767.1.S1_at	236469_at	---	---	---	---		---	---	---	---	MmugDNA.18767.1.S1_at_---
MmugDNA.18768.1.S1_at	202062_s_at	SEL1L	sel-1 suppressor of lin-12-like (C. elegans)	chr14q24.3-q31		6400	---	---	---	---	MmugDNA.18768.1.S1_at_SEL1L
MmugDNA.18769.1.S1_at	212233_at	MAP1B	microtubule-associated protein 1B	chr5q13		4131	---	---	---	---	MmugDNA.18769.1.S1_at_MAP1B
MmugDNA.18770.1.S1_at	201586_s_at	SFPQ	splicing factor proline/glutamine-rich (polypyrimidine tract binding protein associated)	chr1p34.3		6421	LOC712439	similar to Splicing factor, proline- and glutamine-rich (Polypyrimidine tract-binding protein-associated s	---	712439	MmugDNA.18770.1.S1_at_SFPQ
MmugDNA.18771.1.S1_at	207373_at	HOXD10	homeobox D10	chr2q31.1		3236	HOXD10	similar to Homeobox protein Hox-D10 (Hox-4D) (Hox-4E)	---	700318	MmugDNA.18771.1.S1_at_HOXD10
MmugDNA.1877.1.S1_at	218044_x_at	PTMS	parathymosin	chr12p13		5763	LOC722084	hypothetical protein LOC722084	---	722084	MmugDNA.1877.1.S1_at_PTMS
MmugDNA.18772.1.S1_at	231278_at	---	---	---	---		---	---	---	---	MmugDNA.18772.1.S1_at_---
MmugDNA.18773.1.S1_at	238074_at	WDR27	WD repeat domain 27	chr6q27		253769	LOC711727	Similar to potassium channel tetramerisation domain containing 9	---	711727	MmugDNA.18773.1.S1_at_WDR27
MmugDNA.18774.1.S1_at	206804_at	CD3G	CD3g molecule, gamma (CD3-TCR complex)	chr11q23		917	LOC715904	Hypothetical protein LOC715904	---	715904	MmugDNA.18774.1.S1_at_CD3G
MmugDNA.18775.1.S1_at	205462_s_at	HPCAL1	hippocalcin-like 1	chr2p25.1		3241	LOC695395	similar to hippocalcin-like 1	---	695395	MmugDNA.18775.1.S1_at_HPCAL1
MmugDNA.18776.1.S1_at	206024_at	HPD	4-hydroxyphenylpyruvate dioxygenase	chr12q24-qter		3242	HPD	4-hydroxyphenylpyruvate dioxygenase	---	700940	MmugDNA.18776.1.S1_at_HPD
MmugDNA.18777.1.S1_at	209046_s_at	GABARAPL2	GABA(A) receptor-associated protein-like 2	chr16q22.3-q24.1		11345	LOC714413	similar to GABA(A) receptor-associated protein like 2	---	714413	MmugDNA.18777.1.S1_at_GABARAPL2
MmugDNA.18778.1.S1_at	NA	NA	NA	NA	NA		NA	NA	NA	NA	MmugDNA.18778.1.S1_at_NA
MmugDNA.18778.1.S1_s_at	203914_x_at	HPGD	hydroxyprostaglandin dehydrogenase 15-(NAD)	chr4q34-q35		3248	HPGD	hydroxyprostaglandin dehydrogenase 15-(NAD)	---	697864	MmugDNA.18778.1.S1_s_at_HPGD
MmugDNA.18779.1.S1_at	231547_at	ZBTB37	zinc finger and BTB domain containing 37	chr1q25.1		84614	---	---	---	---	MmugDNA.18779.1.S1_at_ZBTB37
MmugDNA.18780.1.S1_at	244794_at	---	---	---	---		---	---	---	---	MmugDNA.18780.1.S1_at_---
MmugDNA.18781.1.S1_at	239131_at	---	---	---	---		---	---	---	---	MmugDNA.18781.1.S1_at_---
MmugDNA.1878.1.S1_at	225339_at	SPAG9	sperm associated antigen 9	chr17q21.33		9043	---	---	---	---	MmugDNA.1878.1.S1_at_SPAG9
MmugDNA.18782.1.S1_at	236806_at	---	---	---	---		---	---	---	---	MmugDNA.18782.1.S1_at_---
MmugDNA.18784.1.S1_at	244609_at	---	---	---	---		---	---	---	---	MmugDNA.18784.1.S1_at_---
MmugDNA.18785.1.S1_at	201848_s_at	BNIP3	BCL2/adenovirus E1B 19kDa interacting protein 3	chr10q26.3		664	---	---	---	---	MmugDNA.18785.1.S1_at_BNIP3
MmugDNA.18786.1.S1_at	234749_s_at	WDR51A	WD repeat domain 51A	chr3p21.1		25886	LOC698157	similar to WD repeat domain 51A	---	698157	MmugDNA.18786.1.S1_at_WDR51A
MmugDNA.18787.1.S1_at	215587_x_at	---	---	---	---		---	---	---	---	MmugDNA.18787.1.S1_at_---
MmugDNA.18788.1.S1_at	240146_at	---	---	---	---		---	---	---	---	MmugDNA.18788.1.S1_at_---
MmugDNA.18789.1.S1_at	232763_at	TLN1	Talin 1	chr9p13		7094	---	---	---	---	MmugDNA.18789.1.S1_at_TLN1
MmugDNA.18790.1.S1_at	239836_at	ECHDC2	Enoyl Coenzyme A hydratase domain containing 2	chr1p32.3		55268	---	---	---	---	MmugDNA.18790.1.S1_at_ECHDC2
MmugDNA.18791.1.S1_at	NA	NA	NA	NA	NA		NA	NA	NA	NA	MmugDNA.18791.1.S1_at_NA
MmugDNA.1879.1.S1_s_at	NA	NA	NA	NA	NA		NA	NA	NA	NA	MmugDNA.1879.1.S1_s_at_NA
MmugDNA.18792.1.S1_at	1561527_at	---	---	---	---		---	---	---	---	MmugDNA.18792.1.S1_at_---
MmugDNA.18793.1.S1_at	204934_s_at	HPN	hepsin	chr19q11-q13.2		3249	HPN	hepsin	---	707242	MmugDNA.18793.1.S1_at_HPN
MmugDNA.18794.1.S1_at	202870_s_at	CDC20	cell division cycle 20 homolog (S. cerevisiae)	chr1p34.1		991	---	---	---	---	MmugDNA.18794.1.S1_at_CDC20
MmugDNA.18796.1.S1_at	1553491_at	KSR2	kinase suppressor of ras 2	chr12q24.22-q24.23		283455	KSR2	kinase suppressor of ras 2	---	694642	MmugDNA.18796.1.S1_at_KSR2
MmugDNA.18797.1.S1_at	1561290_at	LOC339622	hypothetical protein LOC339622	chr21q21.1-q21.2		339622	---	---	---	---	MmugDNA.18797.1.S1_at_LOC339622
MmugDNA.18798.1.S1_at	207188_at	CDK3	cyclin-dependent kinase 3	chr17q22-qter		1018	CDK3	cyclin-dependent kinase 3	---	705330	MmugDNA.18798.1.S1_at_CDK3
MmugDNA.18799.1.S1_at	203308_x_at	HPS1	Hermansky-Pudlak syndrome 1	chr10q23.1-q23.3		3257	---	---	---	---	MmugDNA.18799.1.S1_at_HPS1
MmugDNA.18800.1.S1_at	1562332_at	---	---	---	---		---	---	---	---	MmugDNA.18800.1.S1_at_---
MmugDNA.18801.1.S1_at	1559658_at	C15orf29	chromosome 15 open reading frame 29	chr15q14		79768	LOC719386	Similar to E1A-binding protein p400 (p400 kDa SWI2/SNF2-related protein) (Domino homolog) (mDo	---	719386	MmugDNA.18801.1.S1_at_C15orf29
MmugDNA.1880.1.S1_at	221257_x_at	FBXO38	F-box protein 38	chr5q33.1		81545	FBXO38	F-box protein 38	---	709869	MmugDNA.1880.1.S1_at_FBXO38
MmugDNA.18802.1.S1_at	207066_at	HRC	histidine rich calcium binding protein	chr19q13.3		3270	HRC	histidine rich calcium binding protein	---	718647	MmugDNA.18802.1.S1_at_HRC
MmugDNA.18803.1.S1_at	1562137_at	---	---	---	---		---	---	---	---	MmugDNA.18803.1.S1_at_---
MmugDNA.18804.1.S1_at	209693_at	ASTN2	astrotactin 2	chr9q33.1		23245	ASTN2	astrotactin 2	---	702370	MmugDNA.18804.1.S1_at_ASTN2
MmugDNA.18805.1.S1_at	241502_x_at	---	---	---	---		---	---	---	---	MmugDNA.18805.1.S1_at_---
MmugDNA.18806.1.S1_at	231602_at	---	---	---	---		---	---	---	---	MmugDNA.18806.1.S1_at_---
MmugDNA.18807.1.S1_at	243439_at	ZNF418	zinc finger protein 418	chr19q13.43		147686	---	---	---	---	MmugDNA.18807.1.S1_at_ZNF418
MmugDNA.18808.1.S1_at	234908_s_at	DSCAML1	Down syndrome cell adhesion molecule like 1	chr11q23		57453	LOC698212	similar to Down syndrome cell adhesion molecule like 1	---	698212	MmugDNA.18808.1.S1_at_DSCAML1
MmugDNA.18809.1.S1_at	243922_at	---	---	---	---		---	---	---	---	MmugDNA.18809.1.S1_at_---
MmugDNA.18810.1.S1_at	215664_s_at	EPHA5	EPH receptor A5	chr4q13.1		2044	LOC712074	similar to ephrin receptor EphA5 isoform b	---	712074	MmugDNA.18810.1.S1_at_EPHA5
MmugDNA.18811.1.S1_at	213156_at	---	---	---	---		---	---	---	---	MmugDNA.18811.1.S1_at_---
MmugDNA.1881.1.S1_at	1552660_a_at	C5orf22	chromosome 5 open reading frame 22	chr5p13.3		55322	C6H5orf22	chromosome 5 open reading frame 22 ortholog	---	695901	MmugDNA.1881.1.S1_at_C5orf22
MmugDNA.18812.1.S1_at	213158_at	---	---	---	---		---	---	---	---	MmugDNA.18812.1.S1_at_---
MmugDNA.18813.1.S1_at	230372_at	HAS2	Hyaluronan synthase 2	chr8q24.12		3037	HAS2	hyaluronan synthase 2	---	703353	MmugDNA.18813.1.S1_at_HAS2
MmugDNA.18815.1.S1_at	1555460_a_at	SLC39A6	solute carrier family 39 (zinc transporter), member 6	chr18q12.2		25800	LOC709313	similar to solute carrier family 39 (zinc transporter), member 6	---	709313	MmugDNA.18815.1.S1_at_SLC39A6
MmugDNA.18816.1.S1_at	1557919_a_at	LOC648232	Hypothetical protein LOC648232	---		648232	---	---	---	---	MmugDNA.18816.1.S1_at_LOC648232
MmugDNA.18817.1.S1_at	206210_s_at	CETP	cholesteryl ester transfer protein, plasma	chr16q21		1071	LOC710267	similar to cholesteryl ester transfer protein, plasma precursor	---	710267	MmugDNA.18817.1.S1_at_CETP
MmugDNA.18818.1.S1_at	215526_at	---	---	---	---		---	---	---	---	MmugDNA.18818.1.S1_at_---
MmugDNA.18819.1.S1_at	238794_at	C10orf78	chromosome 10 open reading frame 78	chr10q25.1		119392	LOC715145	similar to Protein C10orf78 homolog	---	715145	MmugDNA.18819.1.S1_at_C10orf78
MmugDNA.188.1.S1_at	1569525_s_at	---	---	---	---		BMS1	BMS1 homolog, ribosome assembly protein (yeast)	---	703355	MmugDNA.188.1.S1_at_---
MmugDNA.18820.1.S1_at	233894_x_at	EMID2	EMI domain containing 2	chr7q22.1		136227	LOC719721	similar to putative emu2	---	719721	MmugDNA.18820.1.S1_at_EMID2
MmugDNA.18821.1.S1_at	217020_at	RARB	retinoic acid receptor, beta	chr3p24		5915	---	---	---	---	MmugDNA.18821.1.S1_at_RARB
MmugDNA.1882.1.S1_s_at	NA	NA	NA	NA	NA		NA	NA	NA	NA	MmugDNA.1882.1.S1_s_at_NA
MmugDNA.18822.1.S1_at	233712_at	ZNF44	zinc finger protein 44	chr19p13.2		51710	---	---	---	---	MmugDNA.18822.1.S1_at_ZNF44
MmugDNA.18823.1.S1_at	206864_s_at	HRK	harakiri, BCL2 interacting protein (contains only BH3 domain)	chr12q24.22		8739	---	---	---	---	MmugDNA.18823.1.S1_at_HRK
MmugDNA.18825.1.S1_at	237075_at	---	---	---	---		---	---	---	---	MmugDNA.18825.1.S1_at_---
MmugDNA.18826.1.S1_at	203461_at	CHD2	chromodomain helicase DNA binding protein 2	chr15q26		1106	---	---	---	---	MmugDNA.18826.1.S1_at_CHD2
MmugDNA.18827.1.S1_at	NA	NA	NA	NA	NA		NA	NA	NA	NA	MmugDNA.18827.1.S1_at_NA
MmugDNA.18828.1.S1_at	206445_s_at	PRMT1	protein arginine methyltransferase 1	chr19q13.3		3276	LOC719419	similar to heterogeneous nuclear ribonucleoproteins methyltransferase-like 2	---	719419	MmugDNA.18828.1.S1_at_PRMT1
MmugDNA.18829.1.S1_at	1565026_a_at	OFCC1	orofacial cleft 1 candidate 1	chr6p24.3		266553	---	---	---	---	MmugDNA.18829.1.S1_at_OFCC1
MmugDNA.18829.1.S1_s_at	NA	NA	NA	NA	NA		NA	NA	NA	NA	MmugDNA.18829.1.S1_s_at_NA
MmugDNA.18830.1.S1_at	204130_at	HSD11B2	hydroxysteroid (11-beta) dehydrogenase 2	chr16q22		3291	---	---	---	---	MmugDNA.18830.1.S1_at_HSD11B2
MmugDNA.18831.1.S1_at	226363_at	ABCC5	ATP-binding cassette, sub-family C (CFTR/MRP), member 5	chr3q27		10057	---	---	---	---	MmugDNA.18831.1.S1_at_ABCC5
MmugDNA.1883.1.S1_at	208091_s_at	ECOP	EGFR-coamplified and overexpressed protein	chr7p11.2		81552	LOC716151	similar to EGFR-coamplified and overexpressed protein	---	716151	MmugDNA.1883.1.S1_at_ECOP
MmugDNA.18832.1.S1_at	205415_s_at	ATXN3	ataxin 3	chr14q24.3-q32.2		4287	LOC699321	similar to ataxin 3 isoform 1	---	699321	MmugDNA.18832.1.S1_at_ATXN3
MmugDNA.18833.1.S1_at	229685_at	LOC100134937	hypothetical LOC100134937	---		100134937	---	---	---	---	MmugDNA.18833.1.S1_at_LOC100134
MmugDNA.18834.1.S1_at	223343_at	MS4A7	membrane-spanning 4-domains, subfamily A, member 7	chr11q12		58475	MS4A7	membrane-spanning 4-domains, subfamily A, member 7	---	697200	MmugDNA.18834.1.S1_at_MS4A7
MmugDNA.18835.1.S1_at	1557371_a_at	LOC158376	hypothetical LOC158376	chr9p13.3		158376	---	---	---	---	MmugDNA.18835.1.S1_at_LOC158376
MmugDNA.18836.1.S1_at	229278_at	---	---	---	---		---	---	---	---	MmugDNA.18836.1.S1_at_---
MmugDNA.18837.1.S1_at	232477_at	---	---	---	---		---	---	---	---	MmugDNA.18837.1.S1_at_---
MmugDNA.18838.1.S1_at	1569878_at	CCNYL2	cyclin Y-like 2	chr10q11.21		414194	---	---	---	---	MmugDNA.18838.1.S1_at_CCNYL2
MmugDNA.18839.1.S1_at	228290_at	NCRNA00153	Non-protein coding RNA 153	chr20pter-q11.23		55857	---	---	---	---	MmugDNA.18839.1.S1_at_NCRNA0015
MmugDNA.18840.1.S1_at	235569_at	VPS37D	vacuolar protein sorting 37 homolog D (S. cerevisiae)	chr7q11.23		155382	LOC720111	similar to vacuolar protein sorting 37D	---	720111	MmugDNA.18840.1.S1_at_VPS37D
MmugDNA.18841.1.S1_at	1570394_at	XRN1	5'-3' exoribonuclease 1	chr3q23		54464	---	---	---	---	MmugDNA.18841.1.S1_at_XRN1
MmugDNA.1884.1.S1_at	1566079_at	RPS16P5	ribosomal protein S16 pseudogene 5	chr6p12.1		647190	---	---	---	---	MmugDNA.1884.1.S1_at_RPS16P5
MmugDNA.18842.1.S1_at	207461_at	---	---	---	---		---	---	---	---	MmugDNA.18842.1.S1_at_---
MmugDNA.18843.1.S1_at	209050_s_at	RALGDS	ral guanine nucleotide dissociation stimulator	chr9q34.3		5900	RALGDS	ral guanine nucleotide dissociation stimulator	---	714436	MmugDNA.18843.1.S1_at_RALGDS
MmugDNA.18844.1.S1_at	206633_at	CHRNA1	cholinergic receptor, nicotinic, alpha 1 (muscle)	chr2q24-q32		1134	CHRNA1	cholinergic receptor, nicotinic, alpha 1 (muscle)	---	698578	MmugDNA.18844.1.S1_at_CHRNA1

MmugDNA.18845.1.S1_at	238689_at	GPR110	G protein-coupled receptor 110	chr6p12.3	266977	GPR110	G protein-coupled receptor 110	---	704995	MmugDNA.18845.1.S1_at_GPR110
MmugDNA.18846.1.S1_at	206703_at	CHRNB1	cholinergic receptor, nicotinic, beta 1 (muscle)	chr17p13.1	1140	CHRNB1	cholinergic receptor, nicotinic, beta 1 (muscle)	---	715509	MmugDNA.18846.1.S1_at_CHRNB1
MmugDNA.18847.1.S1_at	1553002_at	DEFB105A /// DEFB105	defensin, beta 105A /// defensin, beta 105B	chr8p23.1	245908 /// 504180	LOC694797	similar to defensin, beta 105A	---	694797	MmugDNA.18847.1.S1_at_DEFB105A /
MmugDNA.18848.1.S1_at	243443_at	---	---	---	---	---	---	---	---	MmugDNA.18848.1.S1_at_---
MmugDNA.18849.1.S1_at	237795_s_at	SP2	Sp2 transcription factor	chr17q21.32	6668	---	---	---	---	MmugDNA.18849.1.S1_at_SP2
MmugDNA.18850.1.S1_at	221827_at	RBCK1	RanBP-type and C3HC4-type zinc finger containing 1	chr20p13	10616	LOC714692	similar to ubiquitin conjugating enzyme 7 interacting protein 3 isoform 2	---	714692	MmugDNA.18850.1.S1_at_RBCK1
MmugDNA.18851.1.S1_at	200951_s_at	CCND2	cyclin D2	chr12p13	894	---	---	---	---	MmugDNA.18851.1.S1_at_CCND2
MmugDNA.18852.1.S1_at	243357_at	NEGR1	neuronal growth regulator 1	chr1p31.1	257194	LOC703636	similar to neuronal growth regulator 1	---	703636	MmugDNA.18852.1.S1_at_NEGR1
MmugDNA.18853.1.S1_at	227119_at	CNOT6L	CCR4-NOT transcription complex, subunit 6-like	chr4q13.3	246175	IKBKB	Inhibitor of kappa light polypeptide gene enhancer in B-cells, kinase beta	---	704683	MmugDNA.18853.1.S1_at_CNOT6L
MmugDNA.18854.1.S1_at	200952_s_at	CCND2	cyclin D2	chr12p13	894	---	---	---	---	MmugDNA.18854.1.S1_at_CCND2
MmugDNA.18855.1.S1_at	231368_at	BOD1P	biorientation of chromosomes in cell division 1 pseudogene	chr18q21.31	284257	LOC695409	similar to CG5514-PB, isoform B	---	695409	MmugDNA.18855.1.S1_at_BOD1P
MmugDNA.18856.1.S1_at	239222_at	C9orf9	chromosome 9 open reading frame 9	chr9q34	11092	LOC714749	similar to Rsb-66 protein	---	714749	MmugDNA.18856.1.S1_at_C9orf9
MmugDNA.18857.1.S1_at	203900_at	KIAA0467	KIAA0467	chr1p34.2	23334	LOC702661	similar to RP11-506B15.1 protein isoform 1	---	702661	MmugDNA.18857.1.S1_at_KIAA0467
MmugDNA.18858.1.S1_at	210815_s_at	CALCRL	calcitonin receptor-like	chr2q32.1	10203	CALCRL	similar to Calcitonin gene-related peptide type 1 receptor precursor (CGRP type 1 receptor) (Calcitoni	---	708187	MmugDNA.18858.1.S1_at_CALCRL
MmugDNA.18859.1.S1_at	1557430_at	LOC147670	hypothetical protein LOC147670	chr19q13.43	147670	---	---	---	---	MmugDNA.18859.1.S1_at_LOC147670
MmugDNA.18860.1.S1_at	1553620_at	TRIM42	tripartite motif-containing 42	chr3q23	287015	LOC715465	similar to tripartite motif-containing 42	---	715465	MmugDNA.18860.1.S1_at_TRIM42
MmugDNA.18861.1.S1_at	222087_at	PVT1	Pvt1 oncogene (non-protein coding)	chr8q24	5820	---	---	---	---	MmugDNA.18861.1.S1_at_PVT1
MmugDNA.1886.1.S1_at	1554181_at	SNX32	sorting nexin 32	chr11q13.1	254122	LOC721888	similar to Snx6 CG8282-PA	---	721888	MmugDNA.1886.1.S1_at_SNX32
MmugDNA.18862.1.S1_at	240880_at	---	---	---	---	LOC710271	Hypothetical protein LOC710271	---	710271	MmugDNA.18862.1.S1_at_---
MmugDNA.18863.1.S1_at	231612_at	C4orf35	chromosome 4 open reading frame 35	chr4q13.3	85438	OVGP1	Oviductal glycoprotein 1	---	703848	MmugDNA.18863.1.S1_at_C4orf35
MmugDNA.18864.1.S1_at	207024_at	CHRND	cholinergic receptor, nicotinic, delta	chr2q33-q34	1144	LOC713354	similar to nicotinic acetylcholine receptor delta polypeptide precursor	---	713354	MmugDNA.18864.1.S1_at_CHRND
MmugDNA.18865.1.S1_at	1564251_at	EMID1	EMI domain containing 1	chr22q12.2	129080	LOC717414	similar to EMI domain containing 1	---	717414	MmugDNA.18865.1.S1_at_EMID1
MmugDNA.18866.1.S1_at	1561960_at	KIAA1920	KIAA1920 protein	chr15q25.2	114817	---	---	---	---	MmugDNA.18866.1.S1_at_KIAA1920
MmugDNA.18867.1.S1_at	NA	NA	NA	NA	NA	NA	NA	NA	NA	MmugDNA.18867.1.S1_at_NA
MmugDNA.18868.1.S1_at	207274_at	CHRNE	cholinergic receptor, nicotinic, epsilon	chr17p13-p12	1145	CHRNE	cholinergic receptor, nicotinic, epsilon	---	710301	MmugDNA.18868.1.S1_at_CHRNE
MmugDNA.18869.1.S1_at	221295_at	CIDEA	cell death-inducing DFFA-like effector a	chr18p11.21|18	1149	CIDEA	cell death-inducing DFFA-like effector a	---	703318	MmugDNA.18869.1.S1_at_CIDEA
MmugDNA.18870.1.S1_at	229793_at	ASAH2B	N-acylsphingosine amidohydrolase (non-lysosomal ceramidase) 2B	chr10q11.23	653308	LOC703913	similar to N-acylsphingosine amidohydrolase 2	---	703913	MmugDNA.18870.1.S1_at_ASAH2B
MmugDNA.18871.1.S1_at	216916_s_at	DLGAP2	discs, large (Drosophila) homolog-associated protein 2	chr8p23	9228	DLGAP2	discs large-associated protein 2	---	722734	MmugDNA.18871.1.S1_at_DLGAP2
MmugDNA.1887.1.S1_at	208092_s_at	FAM49A	family with sequence similarity 49, member A	chr2p24.3	81553	LOC698947	similar to zinc finger protein 395	---	698947	MmugDNA.1887.1.S1_at_FAM49A
MmugDNA.18872.1.S1_at	200612_s_at	AP2B1	adaptor-related protein complex 2, beta 1 subunit	chr17q11.2-q12	163	LOC716094	similar to adaptor-related protein complex 2, beta 1 subunit	---	716094	MmugDNA.18872.1.S1_at_AP2B1
MmugDNA.18873.1.S1_at	40148_at	APBB2	amyloid beta (A4) precursor protein-binding, family B, member 2	chr4p14-p13	323	APBB2	amyloid beta (A4) precursor protein-binding, family B, member 2	---	701342	MmugDNA.18873.1.S1_at_APBB2
MmugDNA.18874.1.S1_at	219081_at	ANKHD1	ankyrin repeat and KH domain containing 1	chr5q31.3	54882	LOC698045	similar to MASK-4E-BP3 protein	---	698045	MmugDNA.18874.1.S1_at_ANKHD1
MmugDNA.18875.1.S1_at	240590_at	LOC348761	hypothetical protein LOC348761	chr2q37.1	348761	---	---	---	---	MmugDNA.18875.1.S1_at_LOC348761
MmugDNA.18876.1.S1_at	1557116_at	APOL6	apolipoprotein L, 6	chr22q12.3	80830	---	---	---	---	MmugDNA.18876.1.S1_at_APOL6
MmugDNA.18877.1.S1_at	1561923_a_at	SF3B14	Splicing factor 3B, 14 kDa subunit	chr2pter-p25.1	51639	SF3B14	splicing factor 3B, 14 kDa subunit	---	704090	MmugDNA.18877.1.S1_at_SF3B14
MmugDNA.18878.1.S1_at	220917_s_at	WDR19	WD repeat domain 19	chr4p14	57728	WDR19	WD repeat domain 19	---	698866	MmugDNA.18878.1.S1_at_WDR19
MmugDNA.18879.1.S1_at	204157_s_at	KIAA0999	KIAA0999 protein	chr11q23.3	23387	---	---	---	---	MmugDNA.18879.1.S1_at_KIAA0999
MmugDNA.18880.1.S1_at	235572_at	SPC24	SPC24, NDC80 kinetochore complex component, homolog (S. cerevisiae)	chr19p13.2	147841	LOC714043	similar to spindle pole body component 24 homolog	---	714043	MmugDNA.18880.1.S1_at_SPC24
MmugDNA.18881.1.S1_at	239165_at	---	---	---	---	---	---	---	---	MmugDNA.18881.1.S1_at_---
MmugDNA.1888.1.S1_at	236287_at	---	---	---	---	---	---	---	---	MmugDNA.1888.1.S1_at_---
MmugDNA.18882.1.S1_at	232442_at	LOC100131601	similar to hCG1980470	chr16q23.1	100131601	---	---	---	---	MmugDNA.18882.1.S1_at_LOC100131€
MmugDNA.18883.1.S1_at	236736_at	---	---	---	---	---	---	---	---	MmugDNA.18883.1.S1_at_---
MmugDNA.18884.1.S1_at	227704_at	C19orf12	chromosome 19 open reading frame 12	chr19q12	83636	LOC710444	similar to CG3740-PA	---	710444	MmugDNA.18884.1.S1_at_C19orf12
MmugDNA.18885.1.S1_at	242224_at	GPATCH2	G patch domain containing 2	chr1q41	55105	GPATCH2	G patch domain containing 2	---	708081	MmugDNA.18885.1.S1_at_GPATCH2
MmugDNA.18886.1.S1_at	216966_at	ITGA2B	integrin, alpha 2b (platelet glycoprotein IIb of IIb/IIIa complex, antigen CD41)	chr17q21.32	3674	---	---	---	---	MmugDNA.18886.1.S1_at_ITGA2B
MmugDNA.18887.1.S1_at	215278_at	---	---	---	---	---	---	---	---	MmugDNA.18887.1.S1_at_---
MmugDNA.18888.1.S1_at	243360_at	---	---	---	---	---	---	---	---	MmugDNA.18888.1.S1_at_---
MmugDNA.18889.1.S1_s_at	205195_at	AP1S1	adaptor-related protein complex 1, sigma 1 subunit	chr7q22.1	1174	LOC719569	similar to AP-1 complex subunit sigma-1A (Adapter-related protein complex 1 sigma-1A subunit) (Sigı	---	719569	MmugDNA.18889.1.S1_s_at_AP1S1
MmugDNA.18890.1.S1_at	206704_at	CLCN5	chloride channel 5	chrXp11.23	1184	CLCN5	chloride channel 5	---	694497	MmugDNA.18890.1.S1_at_CLCN5
MmugDNA.18891.1.S1_s_at	231135_at	LOC151174	hypothetical LOC151174	chr2q37.3	151174	LOC706446	hypothetical protein LOC706446	---	706446	MmugDNA.18891.1.S1_s_at_LOC15117
MmugDNA.1889.1.S1_at	232309_at	LOC202181	hypothetical protein LOC202181	chr5q35.3	202181	LOC701880	hypothetical protein LOC701880	---	701880	MmugDNA.1889.1.S1_at_LOC202181
MmugDNA.18892.1.S1_s_at	205985_x_at	CLCNKB	chloride channel Kb	chr1p36	1188	CLCNKA	chloride channel Ka	---	703259	MmugDNA.18892.1.S1_s_at_CLCNKB
MmugDNA.18893.1.S1_at	1559144_x_at	LOC100130581	hypothetical LOC100130581	chr17q21.31	100130581	---	---	---	---	MmugDNA.18893.1.S1_at_LOC100130!
MmugDNA.18894.1.S1_at	218699_at	RAB7L1	RAB7, member RAS oncogene family-like 1	chr1q32	8934	RAB7L1	RAB7, member RAS oncogene family-like 1	---	697184	MmugDNA.18894.1.S1_at_RAB7L1
MmugDNA.18895.1.S1_at	233473_x_at	---	---	---	---	---	---	---	---	MmugDNA.18895.1.S1_at_---
MmugDNA.18896.1.S1_at	238879_at	---	---	---	---	---	---	---	---	MmugDNA.18896.1.S1_at_---
MmugDNA.18897.1.S1_at	1553499_s_at	SERPINA9	serpin peptidase inhibitor, clade A (alpha-1 antiproteinase, antitrypsin), member 9	chr14q32.13	327657	SERPINA9	serpin peptidase inhibitor, clade A (alpha-1 antiproteinase, antitrypsin), member 9	---	707962	MmugDNA.18897.1.S1_at_SERPINA9
MmugDNA.18898.1.S1_at	240635_at	---	---	---	---	---	---	---	---	MmugDNA.18898.1.S1_at_---
MmugDNA.18899.1.S1_at	219638_at	FBXO22	F-box protein 22	chr15q24.2	26263	---	---	---	---	MmugDNA.18899.1.S1_at_FBXO22
MmugDNA.18900.1.S1_at	217741_s_at	ZFAND5	zinc finger, AN1-type domain 5	chr9q13-q21	7763	ZFAND5	zinc finger, AN1-type domain 5	---	702149	MmugDNA.18900.1.S1_at_ZFAND5
MmugDNA.18901.1.S1_at	239135_at	CPPED1	calcineurin-like phosphoesterase domain containing 1	chr16p13.12	55313	LOC716978	hypothetical protein LOC716978	---	716978	MmugDNA.18901.1.S1_at_CPPED1
MmugDNA.1890.1.S1_at	217542_at	MDM2	Mdm2 p53 binding protein homolog (mouse)	chr12q14.3-q15	4193	---	---	---	---	MmugDNA.1890.1.S1_at_MDM2
MmugDNA.18902.1.S1_at	1561844_at	---	---	---	---	---	---	---	---	MmugDNA.18902.1.S1_at_---
MmugDNA.18903.1.S1_at	230378_at	SCGB3A1	secretoglobin, family 3A, member 1	chr5q35-qter	92304	LOC716331	similar to Uteroglobin-related protein 2 precursor (Cytokine HIN-1) (High in normal-1) (Secretoglobin f	---	716331	MmugDNA.18903.1.S1_at_SCGB3A1
MmugDNA.18904.1.S1_s_at	211127_x_at	EDA	ectodysplasin A	chrXq12-q13.1	1896	EDA	ectodysplasin A	---	694110	MmugDNA.18904.1.S1_s_at_EDA
MmugDNA.18905.1.S1_at	219742_at	PRR7	proline rich 7 (synaptic)	chr5q35.3	80758	PRR7	proline rich 7 (synaptic)	---	700123	MmugDNA.18905.1.S1_at_PRR7
MmugDNA.18906.1.S1_at	230197_s_at	TPPP	Tubulin polymerization promoting protein	chr5p15.3	11076	---	---	---	---	MmugDNA.18906.1.S1_at_TPPP
MmugDNA.18907.1.S1_at	223572_at	HHATL	hedgehog acyltransferase-like	chr3p22.1	57467	HHATL	hedgehog acyltransferase-like	---	717333	MmugDNA.18907.1.S1_at_HHATL
MmugDNA.18908.1.S1_at	211128_at	EDA	ectodysplasin A	chrXq12-q13.1	1896	EDA	ectodysplasin A	---	694110	MmugDNA.18908.1.S1_at_EDA
MmugDNA.18909.1.S1_at	220513_at	RP11-257K9.7	hypothetical protein LOC100129128	chr6q13	100129128	LOC715519	similar to chromosome 6 open reading frame 148	---	715519	MmugDNA.18909.1.S1_at_RP11-257K9
MmugDNA.18910.1.S1_at	201747_s_at	SAFB	scaffold attachment factor B	chr19p13.3-p13.2	6294	SAFB	scaffold attachment factor B	---	696356	MmugDNA.18910.1.S1_at_SAFB
MmugDNA.18911.1.S1_at	NA	NA	NA	NA	NA	NA	NA	NA	NA	MmugDNA.18911.1.S1_at_NA
MmugDNA.1891.1.S1_at	214740_at	LOC100134053 /// POL	similar to POLR2J4 protein /// polymerase (RNA) II (DNA directed) polypeptide J, 13.3kDa /// polymer	chr7p13 /// chr7q22.1	100134053 /// 246721 /	---	---	---	---	MmugDNA.1891.1.S1_at_LOC1001340!
MmugDNA.18912.1.S1_at	219064_at	ITIH5	inter-alpha (globulin) inhibitor H5	chr10p14	80760	LOC722614	similar to inter-alpha trypsin inhibitor heavy chain precursor 5 isoform 2	---	722614	MmugDNA.18912.1.S1_at_ITIH5
MmugDNA.18913.1.S1_at	209411_s_at	GGA3	golgi associated, gamma adaptin ear containing, ARF binding protein 3	chr17q25.1	23163	LOC701558	hypothetical protein LOC701558	---	701558	MmugDNA.18913.1.S1_at_GGA3
MmugDNA.18914.1.S1_at	NA	NA	NA	NA	NA	NA	NA	NA	NA	MmugDNA.18914.1.S1_at_NA
MmugDNA.18915.1.S1_at	1555736_a_at	AGTRAP	angiotensin II receptor-associated protein	chr1p36.22	57085	LOC722746	similar to Type-1 angiotensin II receptor-associated protein (AT1 receptor-associated protein)	---	722746	MmugDNA.18915.1.S1_at_AGTRAP
MmugDNA.18916.1.S1_at	NA	NA	NA	NA	NA	NA	NA	NA	NA	MmugDNA.18916.1.S1_at_NA
MmugDNA.18916.1.S1_s_at	217800_s_at	NDFIP1	Nedd4 family interacting protein 1	chr5q31.3	80762	---	---	---	---	MmugDNA.18916.1.S1_s_at_NDFIP1
MmugDNA.18917.1.S1_at	225351_at	FAM45A	family with sequence similarity 45, member A	chr10q25	404636	LOC711519	hypothetical protein LOC711519	---	711519	MmugDNA.18917.1.S1_at_FAM45A
MmugDNA.18918.1.S1_at	210305_at	PDE4DIP	phosphodiesterase 4D interacting protein	chr1q12	9659	---	---	---	---	MmugDNA.18918.1.S1_at_PDE4DIP
MmugDNA.18919.1.S1_at	222034_at	GNB2L1	Guanine nucleotide binding protein (G protein), beta polypeptide 2-like 1	chr5q35.3	10399	LOC708526	similar to Guanine nucleotide-binding protein beta subunit 2-like 1 (Receptor of activated protein kina:	---	708526	MmugDNA.18919.1.S1_at_GNB2L1
MmugDNA.189.1.S1_at	203425_s_at	IGFBP5	insulin-like growth factor binding protein 5	chr2q33-q36	3488	LOC716001	Similar to steroid dehydrogenase homolog	---	716001	MmugDNA.189.1.S1_at_IGFBP5
MmugDNA.18920.1.S1_at	243441_at	---	---	---	---	---	---	---	---	MmugDNA.18920.1.S1_at_---
MmugDNA.1892.1.S1_at	231068_at	SLC47A2	solute carrier family 47, member 2	chr17p11.2	146802	LOC703767	hypothetical protein LOC703767	---	703767	MmugDNA.1892.1.S1_at_SLC47A2
MmugDNA.18922.1.S1_at	239091_at	---	---	---	---	---	---	---	---	MmugDNA.18922.1.S1_at_---
MmugDNA.18923.1.S1_at	205713_s_at	COMP	cartilage oligomeric matrix protein	chr19p13.1	1311	LOC720286	similar to cartilage oligomeric matrix protein precursor	---	720286	MmugDNA.18923.1.S1_at_COMP
MmugDNA.18924.1.S1_at	229501_s_at	USP8	ubiquitin specific peptidase 8	chr15q21.2	9101	---	---	---	---	MmugDNA.18924.1.S1_at_USP8
MmugDNA.18925.1.S1_at	204846_at	CP	ceruloplasmin (ferroxidase)	chr3q23-q25	1356	CP	ceruloplasmin (ferroxidase)	---	712210	MmugDNA.18925.1.S1_at_CP
MmugDNA.18926.1.S1_at	214258_x_at	KAT5	K(lysine) acetyltransferase 5	chr11q13	10524	KAT5	K(lysine) acetyltransferase 5	---	715880	MmugDNA.18926.1.S1_at_KAT5
MmugDNA.18927.1.S1_at	232297_at	---	---	---	---	---	---	---	---	MmugDNA.18927.1.S1_at_---
MmugDNA.18928.1.S1_at	1553395_a_at	CD200R1	CD200 receptor 1	chr3q13.2	131450	LOC708734	similar to CD200 receptor 1 isoform a	---	708734	MmugDNA.18928.1.S1_at_CD200R1
MmugDNA.18929.1.S1_at	231166_at	GPR155	G protein-coupled receptor 155	chr2q31.1	151556	---	---	---	---	MmugDNA.18929.1.S1_at_GPR155
MmugDNA.18930.1.S1_at	232095_at	---	---	---	---	---	---	---	---	MmugDNA.18930.1.S1_at_---
MmugDNA.18931.1.S1_at	238668_at	---	---	---	---	---	---	---	---	MmugDNA.18931.1.S1_at_---
MmugDNA.1893.1.S1_at	204207_s_at	RNGTT	RNA guanylyltransferase and 5'-phosphatase	chr6q16	8732	LOC721442	similar to RNA guanylyltransferase and 5-phosphatase	---	721442	MmugDNA.1893.1.S1_at_RNGTT
MmugDNA.18933.1.S1_at	221001_at	C15orf49	chromosome 15 open reading frame 49	chr15q11.2	63969	---	---	---	---	MmugDNA.18933.1.S1_at_C15orf49
MmugDNA.18934.1.S1_at	NA	NA	NA	NA	NA	NA	NA	NA	NA	MmugDNA.18934.1.S1_at_NA
MmugDNA.18935.1.S1_at	230268_at	---	---	---	---	---	---	---	---	MmugDNA.18935.1.S1_at_---
MmugDNA.18936.1.S1_at	241242_at	---	---	---	---	---	---	---	---	MmugDNA.18936.1.S1_at_---
MmugDNA.18937.1.S1_at	1553759_at	MCM9	minichromosome maintenance complex component 9	chr6q22.31	254394	---	---	---	---	MmugDNA.18937.1.S1_at_MCM9
MmugDNA.18939.1.S1_at	221067_s_at	C12orf39	chromosome 12 open reading frame 39	chr12p12.1	80763	LOC706042	hypothetical protein LOC706042	---	706042	MmugDNA.18939.1.S1_at_C12orf39
MmugDNA.18940.1.S1_at	1553086_at	C11orf40	chromosome 11 open reading frame 40	chr11p15.5	143501	---	---	---	---	MmugDNA.18940.1.S1_at_C11orf40
MmugDNA.18941.1.S1_at	1553679_s_at	VKORC1L1	vitamin K epoxide reductase complex, subunit 1-like 1	chr7q11.21	154807	LOC705761 /// VKORC1L	similar to vitamin K epoxide reductase complex, subunit 1-like 1 /// vitamin K epoxide reductase comp	---	697591 /// 705761	MmugDNA.18941.1.S1_at_VKORC1L1
MmugDNA.1894.1.S1_s_at	217287_s_at	TRPC6	transient receptor potential cation channel, subfamily C, member 6	chr11q21-q22	7225	---	---	---	---	MmugDNA.1894.1.S1_s_at_TRPC6
MmugDNA.18942.1.S1_at	218492_s_at	THAP7	THAP domain containing 7	chr22q11.2	80764	THAP7	THAP domain containing 7	---	696208	MmugDNA.18942.1.S1_at_THAP7
MmugDNA.18943.1.S1_at	232982_at	AP1GBP1	AP1 gamma subunit binding protein 1	chr17q12	11276	LOC717750	similar to AP1 gamma subunit binding protein 1 isoform 2	---	717750	MmugDNA.18943.1.S1_at_AP1GBP1
MmugDNA.18944.1.S1_at	220236_at	PDPR	pyruvate dehydrogenase phosphatase regulatory subunit	chr16q22.1	55066	LOC709861	similar to pyruvate dehydrogenase phosphatase regulatory subunit	---	709861	MmugDNA.18944.1.S1_at_PDPR

MmugDNA.18945.1.S1_at	220734_s_at	GLTPD1 /// LOC727825	glycolipid transfer protein domain containing 1 /// hypothetical LOC727825	chr1p36.33	727825 /// 80772	GLTPD1	glycolipid transfer protein domain containing 1	---	700679	MmugDNA.18945.1.S1_at_GLTPD1 /// L
MmugDNA.18946.1.S1_at	236753_at	LOC154822	hypothetical LOC154822	chr7q36.3	154822	---	---	---	---	MmugDNA.18946.1.S1_at_LOC154822
MmugDNA.18947.1.S1_at	1559275_x_at	hCG_1771830	Zinc finger protein LOC654254	chr4p16.3	654254	---	---	---	---	MmugDNA.18947.1.S1_at_hCG_177183
MmugDNA.18948.1.S1_at	236249_at	IKIP	IKK interacting protein	chr12q23.1	121457	IKIP	IKK interacting protein	---	693896	MmugDNA.18948.1.S1_at_IKIP
MmugDNA.18949.1.S1_at	221246_x_at	TNS1	tensin 1	chr2q35-q36	7145	LOC696960	similar to tensin	---	696960	MmugDNA.18949.1.S1_at_TNS1
MmugDNA.18950.1.S1_at	NA	NA	NA	NA	NA	NA	NA	NA	NA	MmugDNA.18950.1.S1_at_NA
MmugDNA.18950.1.S1_s_at	218600_at	LIMD2	LIM domain containing 2	chr17q23.3	80774	LOC717889	similar to LIM domain containing 2	---	717889	MmugDNA.18950.1.S1_s_at_LIMD2
MmugDNA.18951.1.S1_at	234999_at	C19orf47	chromosome 19 open reading frame 47	chr19q13.2	126526	C19H19orf47	chromosome 19 open reading frame 47 ortholog	---	701191	MmugDNA.18951.1.S1_at_C19orf47
MmugDNA.1895.1.S1_at	211387_x_at	RNGTT	RNA guanylyltransferase and 5'-phosphatase	chr6q16	8732	RNGTT	RNA guanylyltransferase and 5'-phosphatase	---	702334	MmugDNA.1895.1.S1_at_RNGTT
MmugDNA.18952.1.S1_at	242879_x_at	---	---	---	---	---	---	---	---	MmugDNA.18952.1.S1_at_---
MmugDNA.18953.1.S1_at	204459_at	CSTF2	cleavage stimulation factor, 3' pre-RNA, subunit 2, 64kDa	chrXq22.1	1478	LOC701235	similar to cleavage stimulation factor subunit 2	---	701235	MmugDNA.18953.1.S1_at_CSTF2
MmugDNA.18954.1.S1_at	1556950_s_at	SERPINB6	serpin peptidase inhibitor, clade B (ovalbumin), member 6	chr6p25	5269	---	---	---	---	MmugDNA.18954.1.S1_at_SERPINB6
MmugDNA.18954.1.S1_x_at	NA	NA	NA	NA	NA	NA	NA	NA	NA	MmugDNA.18954.1.S1_x_at_NA
MmugDNA.18955.1.S1_at	203947_at	CSTF3	cleavage stimulation factor, 3' pre-RNA, subunit 3, 77kDa	chr11p13	1479	---	---	---	---	MmugDNA.18955.1.S1_at_CSTF3
MmugDNA.18956.1.S1_s_at	203392_s_at	CTBP1	C-terminal binding protein 1	chr4p16	1487	LOC694507	similar to C-terminal binding protein 1 isoform 1	---	694507	MmugDNA.18956.1.S1_s_at_CTBP1
MmugDNA.18957.1.S1_at	223472_at	WHSC1	Wolf-Hirschhorn syndrome candidate 1	chr4p16.3	7468	---	---	---	---	MmugDNA.18957.1.S1_at_WHSC1
MmugDNA.18958.1.S1_at	235177_at	FAM119A	family with sequence similarity 119, member A	chr2q33.3	151194	LOC710435	similar to CG7889-PA	---	710435	MmugDNA.18958.1.S1_at_FAM119A
MmugDNA.18959.1.S1_at	233826_at	---	---	---	---	---	---	---	---	MmugDNA.18959.1.S1_at_---
MmugDNA.18960.1.S1_at	NA	NA	NA	NA	NA	NA	NA	NA	NA	MmugDNA.18960.1.S1_at_NA
MmugDNA.18961.1.S1_at	208604_s_at	HOXA3	homeobox A3	chr7p15-p14	3200	HOXA3	similar to homeobox A3 isoform a	---	699979	MmugDNA.18961.1.S1_at_HOXA3
MmugDNA.1896.1.S1_at	241536_at	---	---	---	---	---	---	---	---	MmugDNA.1896.1.S1_at_---
MmugDNA.18962.1.S1_at	208601_s_at	TUBB1	tubulin, beta 1	chr20q13.32	81027	LOC693889	similar to beta tubulin 1, class VI	---	693889	MmugDNA.18962.1.S1_at_TUBB1
MmugDNA.18963.1.S1_at	220999_s_at	CYFIP2	cytoplasmic FMR1 interacting protein 2	chr5q33.3	26999	LOC714984	similar to cytoplasmic FMR1 interacting protein 2	---	714984	MmugDNA.18963.1.S1_at_CYFIP2
MmugDNA.18964.1.S1_at	218897_at	TMEM177	transmembrane protein 177	chr2q14.2	80775	LOC695399	similar to CG33506-PA	---	695399	MmugDNA.18964.1.S1_at_TMEM177
MmugDNA.18965.1.S1_at	1565346_a_at	ATP1A4	ATPase, Na+/K+ transporting, alpha 4 polypeptide	chr1q21-q23	480	ATP1A4	ATPase, Na+/K+ transporting, alpha 4 polypeptide	---	719623	MmugDNA.18965.1.S1_at_ATP1A4
MmugDNA.18966.1.S1_at	219586_at	WDR59	WD repeat domain 59	chr16q23.1	79726	LOC710576	similar to WD repeat domain 59	---	710576	MmugDNA.18966.1.S1_at_WDR59
MmugDNA.18967.1.S1_at	208136_s_at	MGC3771	hypothetical LOC81854	chr16p13.3	81854	---	---	---	---	MmugDNA.18967.1.S1_at_MGC3771
MmugDNA.18968.1.S1_at	238623_at	---	---	---	---	LOC708420	hypothetical protein LOC708420	---	708420	MmugDNA.18968.1.S1_at_---
MmugDNA.18969.1.S1_at	225395_s_at	FAM120AOS	family with sequence similarity 120A opposite strand	chr9q22.31	158293	LOC717436	hypothetical protein LOC717436	---	717436	MmugDNA.18969.1.S1_at_FAM120AOS
MmugDNA.18970.1.S1_at	208137_x_at	ZNF611	zinc finger protein 611	chr19q13.41	81856	---	---	---	---	MmugDNA.18970.1.S1_at_ZNF611
MmugDNA.1897.1.S1_at	1553545_at	ILDR1	immunoglobulin-like domain containing receptor 1	chr3q13.33	286676	LOC714332	similar to immunoglobulin-like domain containing receptor 1	---	714332	MmugDNA.1897.1.S1_at_ILDR1
MmugDNA.18972.1.S1_at	208139_s_at	---	---	---	---	---	---	---	---	MmugDNA.18972.1.S1_at_---
MmugDNA.18973.1.S1_at	200649_at	NUCB1	nucleobindin 1	chr19q13.2-q13.4	4924	LOC718380	similar to nucleobindin 1	---	718380	MmugDNA.18973.1.S1_at_NUCB1
MmugDNA.18974.1.S1_at	228601_at	LOC401022	hypothetical LOC401022	chr2q31.2	401022	---	---	---	---	MmugDNA.18974.1.S1_at_LOC401022
MmugDNA.18975.1.S1_at	242633_x_at	LOC100128510	hypothetical protein LOC100128510	chr16p13.3	100128510	---	---	---	---	MmugDNA.18975.1.S1_at_LOC1001285
MmugDNA.18976.1.S1_s_at	1559072_a_at	ELFN2	extracellular leucine-rich repeat and fibronectin type III domain containing 2	chr22q13.1	114794	LOC697071	similar to slit homolog 1	---	697071	MmugDNA.18976.1.S1_s_at_ELFN2
MmugDNA.18977.1.S1_at	230716_at	LOC285733	hypothetical LOC285733	chr6q23.1	285733	LOC710673	hypothetical protein LOC710673	---	710673	MmugDNA.18977.1.S1_at_LOC285733
MmugDNA.18978.1.S1_at	214124_x_at	---	---	---	---	---	---	---	---	MmugDNA.18978.1.S1_at_---
MmugDNA.18979.1.S1_at	219922_s_at	LTBP3	latent transforming growth factor beta binding protein 3	chr11q13.1	4054	LTBP2	latent transforming growth factor beta binding protein 2	---	721941	MmugDNA.18979.1.S1_at_LTBP3
MmugDNA.18980.1.S1_at	205204_at	NMB	neuromedin B	chr15q22-qter	4828	---	---	---	---	MmugDNA.18980.1.S1_at_NMB
MmugDNA.18981.1.S1_at	235614_at	TMEM151A	transmembrane protein 151A	chr11q13.1	256472	LOC721836	similar to ZK1067.4	---	721836	MmugDNA.18981.1.S1_at_TMEM151A
MmugDNA.1898.1.S1_at	201907_x_at	DVL3	dishevelled, dsh homolog 3 (Drosophila)	chr3q27	1857	LOC705758	similar to dishevelled 3	---	705758	MmugDNA.1898.1.S1_at_DVL3
MmugDNA.18982.1.S1_s_at	226675_s_at	MALAT1	metastasis associated lung adenocarcinoma transcript 1 (non-protein coding)	chr11q13.1	378938	---	---	---	---	MmugDNA.18982.1.S1_s_at_MALAT1
MmugDNA.18983.1.S1_at	231537_at	---	---	---	---	---	---	---	---	MmugDNA.18983.1.S1_at_---
MmugDNA.18984.1.S1_s_at	232510_s_at	DPP3	dipeptidyl-peptidase 3	chr11q12-q13.1	10072	LOC705395 /// LOC7200	similar to dipeptidyl peptidase III /// similar to dipeptidyl peptidase III	---	705395 /// 720063	MmugDNA.18984.1.S1_s_at_DPP3
MmugDNA.18985.1.S1_at	1554119_at	C16orf57	chromosome 16 open reading frame 57	chr16q13	79650	---	---	---	---	MmugDNA.18985.1.S1_at_C16orf57
MmugDNA.18986.1.S1_at	221243_s_at	---	---	---	---	---	---	---	---	MmugDNA.18986.1.S1_at_---
MmugDNA.18987.1.S1_at	234148_at	---	---	---	---	---	---	---	---	MmugDNA.18987.1.S1_at_---
MmugDNA.18988.1.S1_at	221448_s_at	TEX15	testis expressed 15	chr8p12	56154	---	---	---	---	MmugDNA.18988.1.S1_at_TEX15
MmugDNA.18989.1.S1_at	1557112_a_at	VPS53	vacuolar protein sorting 53 homolog (S. cerevisiae)	chr17p13.3	55275	---	---	---	---	MmugDNA.18989.1.S1_at_VPS53
MmugDNA.18990.1.S1_at	221035_s_at	TEX14	testis expressed 14	chr17q22	56155	LOC714776	similar to testis expressed sequence 14 isoform b	---	714776	MmugDNA.18990.1.S1_at_TEX14
MmugDNA.18991.1.S1_at	214934_at	ATP9B	ATPase, class II, type 9B	chr18q23	374868	ATP9B	ATPase, class II, type 9B	---	697957	MmugDNA.18991.1.S1_at_ATP9B
MmugDNA.1899.1.S1_at	NA	NA	NA	NA	NA	NA	NA	NA	NA	MmugDNA.1899.1.S1_at_NA
MmugDNA.18992.1.S1_at	221034_s_at	TEX13B	testis expressed 13B	chrXq22.3	56156	---	---	---	---	MmugDNA.18992.1.S1_at_TEX13B
MmugDNA.18993.1.S1_at	1558561_at	HM13	histocompatibility (minor) 13	chr20q11.21	81502	---	---	---	---	MmugDNA.18993.1.S1_at_HM13
MmugDNA.18994.1.S1_at	210990_s_at	LAMA4	laminin, alpha 4	chr6q21	3910	---	---	---	---	MmugDNA.18994.1.S1_at_LAMA4
MmugDNA.18995.1.S1_at	NA	NA	NA	NA	NA	NA	NA	NA	NA	MmugDNA.18995.1.S1_at_NA
MmugDNA.18996.1.S1_at	222398_s_at	EFTUD2	elongation factor Tu GTP binding domain containing 2	chr17q21.31	9343	EFTUD2	elongation factor Tu GTP binding domain containing 2	---	715512	MmugDNA.18996.1.S1_at_EFTUD2
MmugDNA.18997.1.S1_at	243244_at	CACNB4	calcium channel, voltage-dependent, beta 4 subunit	chr2q22-q23	785	---	---	---	---	MmugDNA.18997.1.S1_at_CACNB4
MmugDNA.18998.1.S1_at	209820_s_at	TBL3	transducin (beta)-like 3	chr16p13.3	10607	TBL3	transducin (beta)-like 3	---	693864	MmugDNA.18998.1.S1_at_TBL3
MmugDNA.18999.1.S1_at	226817_at	DSC2	desmocollin 2	chr18q12.1	1824	LOC712942	similar to desmocollin 2 isoform Dsc2a preproprotein	---	712942	MmugDNA.18999.1.S1_at_DSC2
MmugDNA.19000.1.S1_at	1552917_at	IL29	interleukin 29 (interferon, lambda 1)	chr19q13.13	282618	LOC697161	similar to interleukin 29	---	697161	MmugDNA.19000.1.S1_at_IL29
MmugDNA.19001.1.S1_at	240609_at	---	---	---	---	---	---	---	---	MmugDNA.19001.1.S1_at_---
MmugDNA.1900.1.S1_at	NA	NA	NA	NA	NA	NA	NA	NA	NA	MmugDNA.1900.1.S1_at_NA
MmugDNA.1900.1.S1_s_at	210548_at	CCL23	chemokine (C-C motif) ligand 23	chr17q12	6368	---	---	---	---	MmugDNA.1900.1.S1_s_at_CCL23
MmugDNA.19002.1.S1_at	NA	NA	NA	NA	NA	NA	NA	NA	NA	MmugDNA.19002.1.S1_at_NA
MmugDNA.19003.1.S1_at	211545_at	GHRHR	growth hormone releasing hormone receptor	chr7p14	2692	---	---	---	---	MmugDNA.19003.1.S1_at_GHRHR
MmugDNA.19004.1.S1_at	1563572_at	LOC152274	hypothetical protein LOC152274	chr3p25.1	152274	---	---	---	---	MmugDNA.19004.1.S1_at_LOC152274
MmugDNA.19005.1.S1_at	1553193_at	ZNF441	zinc finger protein 441	chr19p13.2	126068	LOC716945	similar to zinc finger protein 441	---	716945	MmugDNA.19005.1.S1_at_ZNF441
MmugDNA.19006.1.S1_at	64440_at	IL17RC	interleukin 17 receptor C	chr3p25.3|3p25.3-p24.1	84818	IL17RC	interleukin 17 receptor C	---	699223	MmugDNA.19006.1.S1_at_IL17RC
MmugDNA.19007.1.S1_at	211922_s_at	CAT	catalase	chr11p13	847	IL2	Interleukin 2	---	708017	MmugDNA.19007.1.S1_at_CAT
MmugDNA.19008.1.S1_at	209267_s_at	SLC39A8	solute carrier family 39 (zinc transporter), member 8	chr4q22-q24	64116	LOC710142	similar to solute carrier family 39 (zinc transporter), member 8	---	710142	MmugDNA.19008.1.S1_at_SLC39A8
MmugDNA.19009.1.S1_at	235036_at	LIX1L	Lix1 homolog (mouse)-like	chr1q21.1	128077	---	---	---	---	MmugDNA.19009.1.S1_at_LIX1L
MmugDNA.19010.1.S1_at	1553747_at	MGC16025	hypothetical LOC85009	chr2q37.3	85009	---	---	---	---	MmugDNA.19010.1.S1_at_MGC16025
MmugDNA.19011.1.S1_at	NA	NA	NA	NA	NA	NA	NA	NA	NA	MmugDNA.19011.1.S1_at_NA
MmugDNA.1901.1.S1_at	223804_s_at	THUMPD3	THUMP domain containing 3	chr3p25.3	25917	THUMPD3	THUMP domain containing 3	---	701564	MmugDNA.1901.1.S1_at_THUMPD3
MmugDNA.19012.1.S1_a_at	NA	NA	NA	NA	NA	NA	NA	NA	NA	MmugDNA.19012.1.S1_a_at_NA
MmugDNA.19012.1.S1_at	1564421_at	---	---	---	---	---	---	---	---	MmugDNA.19012.1.S1_at_---
MmugDNA.19013.1.S1_s_at	221438_s_at	TEX12	testis expressed 12	chr11q22	56158	LOC711488	similar to testis expressed sequence 12	---	711488	MmugDNA.19013.1.S1_s_at_TEX12
MmugDNA.19014.1.S1_at	222275_at	---	---	---	---	---	---	---	---	MmugDNA.19014.1.S1_at_---
MmugDNA.19015.1.S1_at	232710_at	---	---	---	---	---	---	---	---	MmugDNA.19015.1.S1_at_---
MmugDNA.19017.1.S1_at	221033_s_at	RNF17	ring finger protein 17	chr13q12.12	56163	---	---	---	---	MmugDNA.19017.1.S1_at_RNF17
MmugDNA.19018.1.S1_at	226485_at	FLJ20674	hypothetical protein FLJ20674	chr12q24.23	54621	---	---	---	---	MmugDNA.19018.1.S1_at_FLJ20674
MmugDNA.190.1.S1_at	227551_at	FAM108B1	family with sequence similarity 108, member B1	chr9q21.13	51104	LOC703828	similar to chromosome 9 open reading frame 77 isoform 2	---	703828	MmugDNA.190.1.S1_at_FAM108B1
MmugDNA.19020.1.S1_at	221018_s_at	TDRD1	tudor domain containing 1	chr10q25.3	56165	---	---	---	---	MmugDNA.19020.1.S1_at_TDRD1
MmugDNA.19021.1.S1_at	212152_x_at	ARID1A	AT rich interactive domain 1A (SWI-like)	chr1p35.3	8289	LOC719949	similar to AT rich interactive domain 1A isoform a	---	719949	MmugDNA.19021.1.S1_at_ARID1A
MmugDNA.1902.1.S1_s_at	215157_x_at	PABPC1	poly(A) binding protein, cytoplasmic 1	chr8q22.2-q23	26986	PABPC1	poly(A) binding protein, cytoplasmic 1	---	708443	MmugDNA.1902.1.S1_s_at_PABPC1
MmugDNA.19022.1.S1_at	221008_s_at	AGXT2L1	alanine-glyoxylate aminotransferase 2-like 1	chr4q25	64850	LOC696880	similar to alanine-glyoxylate aminotransferase 2-like 1	---	696880	MmugDNA.19022.1.S1_at_AGXT2L1
MmugDNA.19023.1.S1_at	1569436_at	LOC400128	hypothetical gene supported by BC025370	chr13q14.11	400128	---	---	---	---	MmugDNA.19023.1.S1_at_LOC400128
MmugDNA.19024.1.S1_at	224661_at	PIGY	phosphatidylinositol glycan anchor biosynthesis, class Y	chr4q22.1	84992	LOC702862	hypothetical protein LOC702862	---	702862	MmugDNA.19024.1.S1_at_PIGY
MmugDNA.19025.1.S1_at	243787_at	---	---	---	---	---	---	---	---	MmugDNA.19025.1.S1_at_---
MmugDNA.19026.1.S1_at	1552857_a_at	HTR6	5-hydroxytryptamine (serotonin) receptor 6	chr1p36-p35	3362	LOC713945	Similar to High mobility group protein B1 (High mobility group protein 1) (HMG-1) (Amphoterin) (Hepa	---	713945	MmugDNA.19026.1.S1_at_HTR6
MmugDNA.19027.1.S1_at	242982_x_at	ITGB8	integrin, beta 8	chr7p15.3	3696	ITGB8	integrin, beta 8	---	707247	MmugDNA.19027.1.S1_at_ITGB8
MmugDNA.19028.1.S1_at	224660_at	PIGY	phosphatidylinositol glycan anchor biosynthesis, class Y	chr4q22.1	84992	LOC702862	hypothetical protein LOC702862	---	702862	MmugDNA.19028.1.S1_at_PIGY
MmugDNA.19029.1.S1_at	241719_at	---	---	---	---	---	---	---	---	MmugDNA.19029.1.S1_at_---
MmugDNA.19030.1.S1_at	207858_s_at	PKLR	pyruvate kinase, liver and RBC	chr1q21	5313	LOC719152	similar to pyruvate kinase, liver and RBC isoform 1	---	719152	MmugDNA.19030.1.S1_at_PKLR
MmugDNA.19031.1.S1_at	235789_at	KDM4B	lysine (K)-specific demethylase 4B	chr19p13.3	23030	---	---	---	---	MmugDNA.19031.1.S1_at_KDM4B
MmugDNA.1903.1.S1_at	NA	NA	NA	NA	NA	NA	NA	NA	NA	MmugDNA.1903.1.S1_at_NA
MmugDNA.1903.1.S1_s_at	221247_s_at	WBSCR16	Williams-Beuren syndrome chromosome region 16	chr7q11.23	81554	WBSCR16	Williams-Beuren syndrome chromosome region 16	---	694334	MmugDNA.1903.1.S1_s_at_WBSCR16
MmugDNA.19032.1.S1_s_at	206155_at	ABCC2	ATP-binding cassette, sub-family C (CFTR/MRP), member 2	chr10q24	1244	ABCC2	ATP-binding cassette, sub-family C (CFTR/MRP), member 2	---	574169	MmugDNA.19032.1.S1_s_at_ABCC2
MmugDNA.19033.1.S1_at	204850_s_at	DCX	doublecortin	chrXq22.3-q23	1641	LOC706556	similar to doublecortin isoform a	---	706556	MmugDNA.19033.1.S1_at_DCX
MmugDNA.19034.1.S1_at	211599_x_at	MET	met proto-oncogene (hepatocyte growth factor receptor)	chr7q31	4233	MET	met proto-oncogene	---	704562	MmugDNA.19034.1.S1_at_MET
MmugDNA.19035.1.S1_at	1565617_at	---	---	---	---	---	---	---	---	MmugDNA.19035.1.S1_at_---
MmugDNA.19037.1.S1_at	240201_at	---	---	---	---	---	---	---	---	MmugDNA.19037.1.S1_at_---
MmugDNA.19039.1.S1_at	220177_s_at	TMPRSS3	transmembrane protease, serine 3	chr21q22.3	64699	TMPRSS3	transmembrane protease, serine 3	---	714500	MmugDNA.19039.1.S1_at_TMPRSS3
MmugDNA.19040.1.S1_at	203011_at	IMPA1	inositol(myo)-1(or 4)-monophosphatase 1	chr8q21.13-q21.3	3612	IMPA1	inositol(myo)-1(or 4)-monophosphatase 1	---	701959	MmugDNA.19040.1.S1_at_IMPA1
MmugDNA.19041.1.S1_at	1559863_a_at	AFG3L1	AFG3 ATPase family gene 3-like 1 (S. cerevisiae)	chr16q24.3	172	LOC715685	similar to AFG3-like protein 1	---	715685	MmugDNA.19041.1.S1_at_AFG3L1
MmugDNA.1904.1.S1_at	237138_at	---	---	---	---	---	---	---	---	MmugDNA.1904.1.S1_at_---

MmugDNA.19042.1.S1_at	244463_at	ADAM23	ADAM metallopeptidase domain 23	chr2q33	8745	---	---	---	---	MmugDNA.19042.1.S1_at_ADAM23
MmugDNA.19043.1.S1_at	230493_at	SHISA2	shisa homolog 2 (Xenopus laevis)	chr13q12.13	387914	LOC709404	similar to Transmembrane protein 46 precursor	---	709404	MmugDNA.19043.1.S1_at_SHISA2
MmugDNA.19044.1.S1_at	NA	NA	NA	NA	NA	NA	NA	NA	NA	MmugDNA.19044.1.S1_at_NA
MmugDNA.19044.1.S1_s_at	205296_at	RBL1	retinoblastoma-like 1 (p107)	chr20q11.2	5933	---	---	---	---	MmugDNA.19044.1.S1_s_at_RBL1
MmugDNA.19045.1.S1_at	221437_s_at	MRPS15	mitochondrial ribosomal protein S15	chr1p35-p34.1	64960	LOC718596	similar to 28S ribosomal protein S15, mitochondrial precursor (S15mt) (MRP-S15)	---	718596	MmugDNA.19045.1.S1_at_MRPS15
MmugDNA.19046.1.S1_at	235195_at	FBXW2	F-box and WD repeat domain containing 2	chr9q34	26190	FBXW2	F-box and WD repeat domain containing 2	---	700958	MmugDNA.19046.1.S1_at_FBXW2
MmugDNA.19047.1.S1_at	220980_s_at	ADPGK	ADP-dependent glucokinase	chr15q24.1	83440	LOC697850	Similar to N-acetylgalactosamine-6-sulfatase precursor	---	697850	MmugDNA.19047.1.S1_at_ADPGK
MmugDNA.19048.1.S1_at	228408_s_at	SDAD1	SDA1 domain containing 1	chr4q21.1	55153	LOC717921	similar to SDA1 domain containing 1	---	717921	MmugDNA.19048.1.S1_at_SDAD1
MmugDNA.19049.1.S1_at	220968_s_at	TSPAN9	tetraspanin 9	chr12p13.33-p13.32	10867	TSPAN9	tetraspanin 9	---	721790	MmugDNA.19049.1.S1_at_TSPAN9
MmugDNA.19050.1.S1_at	221269_s_at	SH3BGRL3	SH3 domain binding glutamic acid-rich protein like 3	chr1p35-p34.3	83442	LOC702794	Hypothetical protein LOC702794	---	702794	MmugDNA.19050.1.S1_at_SH3BGRL3
MmugDNA.19051.1.S1_at	221263_s_at	SF3B5	splicing factor 3b, subunit 5, 10kDa	chr6q24.2	83443	SF3B5	splicing factor 3b, subunit 5, 10kDa	---	699863	MmugDNA.19051.1.S1_at_SF3B5
MmugDNA.1905.1.S1_at	221423_s_at	YIPF5	Yip1 domain family, member 5	chr5q32	81555	LOC706656	similar to smooth muscle cell associated protein 5	---	706656	MmugDNA.1905.1.S1_at_YIPF5
MmugDNA.19052.1.S1_at	231519_at	---	---	---	---	---	---	---	---	MmugDNA.19052.1.S1_at_---
MmugDNA.19053.1.S1_at	240332_at	---	---	---	---	---	---	---	---	MmugDNA.19053.1.S1_at_---
MmugDNA.19054.1.S1_at	1561973_at	SMARCC2	SWI/SNF related, matrix associated, actin dependent regulator of chromatin, subfamily c, member 2	chr12q13-q14	6601	LOC709158	similar to SWI/SNF-related matrix-associated actin-dependent regulator of chromatin c2 isoform b	---	709158	MmugDNA.19054.1.S1_at_SMARCC2
MmugDNA.19055.1.S1_at	223963_s_at	IGF2BP2	insulin-like growth factor 2 mRNA binding protein 2	chr3q27.2	10644	LOC700598	similar to insulin-like growth factor 2 mRNA binding protein 2 isoform a	---	700598	MmugDNA.19055.1.S1_at_IGF2BP2
MmugDNA.19056.1.S1_at	235591_at	SSTR1	somatostatin receptor 1	chr14q13	6751	SSTR1	somatostatin receptor 1	---	698123	MmugDNA.19056.1.S1_at_SSTR1
MmugDNA.19057.1.S1_at	232289_at	KCNJ12	potassium inwardly-rectifying channel, subfamily J, member 12	chr17p11.2	3768	LOC705326	similar to potassium inwardly-rectifying channel J12	---	705326	MmugDNA.19057.1.S1_at_KCNJ12
MmugDNA.19058.1.S1_at	1563162_at	---	---	---	---	---	---	---	---	MmugDNA.19058.1.S1_at_---
MmugDNA.19059.1.S1_at	1560869_a_at	---	---	---	---	---	---	---	---	MmugDNA.19059.1.S1_at_---
MmugDNA.19060.1.S1_at	1560035_at	C2orf85	chromosome 2 open reading frame 85	chr2q37.3	285093	LOC723474	hypothetical protein LOC723474	---	723474	MmugDNA.19060.1.S1_at_C2orf85
MmugDNA.19061.1.S1_at	1555164_at	KIF26B	kinesin family member 26B	chr1q44	55083	---	---	---	---	MmugDNA.19061.1.S1_at_KIF26B
MmugDNA.1906.1.S1_at	221265_s_at	C15orf44	chromosome 15 open reading frame 44	chr15q22.31	81556	---	---	---	---	MmugDNA.1906.1.S1_at_C15orf44
MmugDNA.19062.1.S1_at	209293_x_at	ID4	inhibitor of DNA binding 4, dominant negative helix-loop-helix protein	chr6p22-p21	3400	ID4	inhibitor of DNA binding 4, dominant negative helix-loop-helix protein	---	705825	MmugDNA.19062.1.S1_at_ID4
MmugDNA.19063.1.S1_at	1563185_at	---	---	---	---	---	---	---	---	MmugDNA.19063.1.S1_at_---
MmugDNA.19064.1.S1_at	239302_s_at	---	---	---	---	---	---	---	---	MmugDNA.19064.1.S1_at_---
MmugDNA.19065.1.S1_at	231742_at	CRX	cone-rod homeobox	chr19q13.3	1406	CRX	cone-rod homeobox	---	717550	MmugDNA.19065.1.S1_at_CRX
MmugDNA.19066.1.S1_at	204006_s_at	FCGR3A /// FCGR3B	Fc fragment of IgG, low affinity IIIa, receptor (CD16a) /// Fc fragment of IgG, low affinity IIIb, receptor	chr1q23	2214 /// 2215	LOC720006	Fc gamma RIIIa	---	720006	MmugDNA.19066.1.S1_at_FCGR3A ///
MmugDNA.19067.1.S1_at	1552718_at	DTD1	D-tyrosyl-tRNA deacylase 1 homolog (S. cerevisiae)	chr20p11.23	92675	---	---	---	---	MmugDNA.19067.1.S1_at_DTD1
MmugDNA.19068.1.S1_x_at	NA	NA	NA	NA	NA	NA	NA	NA	NA	MmugDNA.19068.1.S1_x_at_NA
MmugDNA.19069.1.S1_at	223324_s_at	TRPM7	transient receptor potential cation channel, subfamily M, member 7	chr15q21	54822	LOC716755	similar to transient receptor potential cation channel, subfamily M, member 7	---	716755	MmugDNA.19069.1.S1_at_TRPM7
MmugDNA.19070.1.S1_at	220244_at	LOH3CR2A	loss of heterozygosity, 3, chromosomal region 2, gene A	chr3p25.3	29931	---	---	---	---	MmugDNA.19070.1.S1_at_LOH3CR2A
MmugDNA.19071.1.S1_at	226382_at	LOC283070	hypothetical LOC283070	chr10p14	283070	---	---	---	---	MmugDNA.19071.1.S1_at_LOC283070
MmugDNA.19072.1.S1_at	1554362_at	BTG4	B-cell translocation gene 4	chr11q23	54766	---	---	---	---	MmugDNA.19072.1.S1_at_BTG4
MmugDNA.19073.1.S1_at	206401_s_at	MAPT	microtubule-associated protein tau	chr17q21.1	4137	LOC712421	Similar to microtubule-associated protein tau isoform 4	---	712421	MmugDNA.19073.1.S1_at_MAPT
MmugDNA.19074.1.S1_at	223284_at	NAT14	N-acetyltransferase 14 (GCN5-related, putative)	chr19q13.42	57106	ZNF628	similar to zinc finger protein 628	---	701621	MmugDNA.19074.1.S1_at_NAT14
MmugDNA.19075.1.S1_at	230668_at	NKAIN4	Na+/K+ transporting ATPase interacting 4	chr20q13.33	128414	NKAIN4	Na+/K+ transporting ATPase interacting 4	---	697551	MmugDNA.19075.1.S1_at_NKAIN4
MmugDNA.19076.1.S1_at	201573_s_at	ETF1	eukaryotic translation termination factor 1	chr5q31.1	2107	LOC714449	similar to eukaryotic translation termination factor 1 (predicted)	---	714449	MmugDNA.19076.1.S1_at_ETF1
MmugDNA.19076.1.S1_s_at	NA	NA	NA	NA	NA	NA	NA	NA	NA	MmugDNA.19076.1.S1_s_at_NA
MmugDNA.19077.1.S1_at	214373_at	---	---	---	---	---	---	---	---	MmugDNA.19077.1.S1_at_---
MmugDNA.19078.1.S1_at	233433_at	---	---	---	---	---	---	---	---	MmugDNA.19078.1.S1_at_---
MmugDNA.19079.1.S1_at	NA	NA	NA	NA	NA	NA	NA	NA	NA	MmugDNA.19079.1.S1_at_NA
MmugDNA.19079.1.S1_s_at	202058_s_at	KPNA1	karyopherin alpha 1 (importin alpha 5)	chr3q21	3836	---	---	---	---	MmugDNA.19079.1.S1_s_at_KPNA1
MmugDNA.19080.1.S1_at	237232_at	---	---	---	---	---	---	---	---	MmugDNA.19080.1.S1_at_---
MmugDNA.19081.1.S1_at	216027_at	TMX4	thioredoxin-related transmembrane protein 4	chr20p12	56255	TMX4	thioredoxin-related transmembrane protein 4	---	718375	MmugDNA.19081.1.S1_at_TMX4
MmugDNA.1908.1.S1_at	NA	NA	NA	NA	NA	NA	NA	NA	NA	MmugDNA.1908.1.S1_at_NA
MmugDNA.1908.1.S1_s_at	NA	NA	NA	NA	NA	NA	NA	NA	NA	MmugDNA.1908.1.S1_s_at_NA
MmugDNA.19082.1.S1_at	237460_x_at	C14orf182	chromosome 14 open reading frame 182	chr14q22.1	283551	---	---	---	---	MmugDNA.19082.1.S1_at_C14orf182
MmugDNA.19083.1.S1_at	240287_at	IRG1	immunoresponsive 1 homolog (mouse)	chr13q22.3	730249	LOC695991	similar to Immune-responsive protein 1	---	695991	MmugDNA.19083.1.S1_at_IRG1
MmugDNA.19084.1.S1_at	230572_at	C4orf38	chromosome 4 open reading frame 38	chr4q35.1	152641	---	---	---	---	MmugDNA.19084.1.S1_at_C4orf38
MmugDNA.19085.1.S1_at	228100_at	C1orf88	chromosome 1 open reading frame 88	chr1p13.2	128344	LOC703515	hypothetical protein LOC703515	---	703515	MmugDNA.19085.1.S1_at_C1orf88
MmugDNA.19086.1.S1_at	207433_at	IL10	interleukin 10	chr1q31-q32	3586	---	---	---	---	MmugDNA.19086.1.S1_at_IL10
MmugDNA.19087.1.S1_at	216121_at	---	---	---	---	---	---	---	---	MmugDNA.19087.1.S1_at_---
MmugDNA.19088.1.S1_at	232464_at	TRIMP1	tripartite motif-containing pseudogene 1	chr11p15	117852	---	---	---	---	MmugDNA.19088.1.S1_at_TRIMP1
MmugDNA.19089.1.S1_s_at	1555833_a_at	IRGQ	immunity-related GTPase family, Q	chr19q13.31	126298	---	---	---	---	MmugDNA.19089.1.S1_s_at_IRGQ
MmugDNA.19090.1.S1_at	232648_at	PSMA3	proteasome (prosome, macropain) subunit, alpha type, 3	chr14q23	5684	PSMA3	proteasome (prosome, macropain) subunit, alpha type, 3	---	700251	MmugDNA.19090.1.S1_at_PSMA3
MmugDNA.19091.1.S1_at	212438_at	SNRNP27	small nuclear ribonucleoprotein 27kDa (U4/U6.U5)	chr2p14	11017	LOC708670	similar to putative nucleic acid binding protein RY-1	---	708670	MmugDNA.19091.1.S1_at_SNRNP27
MmugDNA.1909.1.S1_at	240408_at	LOC100134937	Hypothetical LOC100134937	---	100134937	---	---	---	---	MmugDNA.1909.1.S1_at_LOC10013493
MmugDNA.19092.1.S1_at	212440_at	SNRNP27	small nuclear ribonucleoprotein 27kDa (U4/U6.U5)	chr2p14	11017	LOC708670	similar to putative nucleic acid binding protein RY-1	---	708670	MmugDNA.19092.1.S1_at_SNRNP27
MmugDNA.19093.1.S1_at	240053_x_at	---	---	---	---	---	---	---	---	MmugDNA.19093.1.S1_at_---
MmugDNA.19094.1.S1_at	1561864_at	---	---	---	---	---	---	---	---	MmugDNA.19094.1.S1_at_---
MmugDNA.19095.1.S1_at	1554050_at	SMPDL3B	sphingomyelin phosphodiesterase, acid-like 3B	chr1p35.3	27293	LOC716655	similar to acid sphingomyelinase-like phosphodiesterase 3B isoform 1	---	716655	MmugDNA.19095.1.S1_at_SMPDL3B
MmugDNA.19096.1.S1_at	1552858_at	MAGEB6	melanoma antigen family B, 6	chrXp21.3	158809	LOC700754	similar to melanoma antigen family B, 6	---	700754	MmugDNA.19096.1.S1_at_MAGEB6
MmugDNA.19097.1.S1_at	232404_at	SHROOM4	shroom family member 4	chrXp11.22	57477	---	---	---	---	MmugDNA.19097.1.S1_at_SHROOM4
MmugDNA.19098.1.S1_at	242898_at	EIF2AK2	eukaryotic translation initiation factor 2-alpha kinase 2	chr2p22-p21	5610	---	---	---	---	MmugDNA.19098.1.S1_at_EIF2AK2
MmugDNA.19099.1.S1_at	244034_at	ZCCHC2	Zinc finger, CCHC domain containing 2	chr18q21.33	54877	---	---	---	---	MmugDNA.19099.1.S1_at_ZCCHC2
MmugDNA.19100.1.S1_at	235143_at	SLC10A7	solute carrier family 10 (sodium/bile acid cotransporter family), member 7	chr4q31.22	84068	SLC10A7	solute carrier family 10 (sodium/bile acid cotransporter family), member 7	---	703452	MmugDNA.19100.1.S1_at_SLC10A7
MmugDNA.19101.1.S1_at	231987_at	LOC728264	hypothetical LOC728264	chr5q33.1	728264	---	---	---	---	MmugDNA.19101.1.S1_at_LOC728264
MmugDNA.1910.1.S1_at	221867_at	N4BP1	NEDD4 binding protein 1	chr16q12.1	9683	N4BP1	Nedd4 binding protein 1	---	717876	MmugDNA.1910.1.S1_at_N4BP1
MmugDNA.19102.1.S1_at	225781_at	MAPK9	mitogen-activated protein kinase 9	chr5q35	5601	---	---	---	---	MmugDNA.19102.1.S1_at_MAPK9
MmugDNA.19103.1.S1_at	215297_at	hCG_2009921	hypothetical locus LOC441204	chr7p15.2	441204	---	---	---	---	MmugDNA.19103.1.S1_at_hCG_200992
MmugDNA.19104.1.S1_at	228963_at	RSBN1L	Round spermatid basic protein 1-like	chr7q11.23	222194	---	---	---	---	MmugDNA.19104.1.S1_at_RSBN1L
MmugDNA.19105.1.S1_at	239891_x_at	RAB12	RAB12, member RAS oncogene family	chr18p11.22	201475	LOC707131	Similar to mitochondrial intermediate peptidase	---	707131	MmugDNA.19105.1.S1_at_RAB12
MmugDNA.19106.1.S1_at	221550_at	COX15	COX15 homolog, cytochrome c oxidase assembly protein (yeast)	chr10q24	1355	---	---	---	---	MmugDNA.19106.1.S1_at_COX15
MmugDNA.19106.1.S1_x_at	NA	NA	NA	NA	NA	NA	NA	NA	NA	MmugDNA.19106.1.S1_x_at_NA
MmugDNA.19107.1.S1_at	221474_at	MYL12B	myosin, light chain 12B, regulatory	chr18p11.31	103910	LOC711953	similar to Myosin regulatory light chain 2-B, smooth muscle isoform (Myosin RLC-B)	---	711953	MmugDNA.19107.1.S1_at_MYL12B
MmugDNA.19108.1.S1_at	216182_at	SYNJ2	Synaptojanin 2	chr6q25.3	8871	---	---	---	---	MmugDNA.19108.1.S1_at_SYNJ2
MmugDNA.19109.1.S1_at	205992_s_at	IL15	interleukin 15	chr4q31	3600	IL15	interleukin 15	---	699616	MmugDNA.19109.1.S1_at_IL15
MmugDNA.19110.1.S1_at	225477_s_at	NR2C2	nuclear receptor subfamily 2, group C, member 2	chr3p25	7182	---	---	---	---	MmugDNA.19110.1.S1_at_NR2C2
MmugDNA.19111.1.S1_at	202718_at	IGFBP2	insulin-like growth factor binding protein 2, 36kDa	chr2q33-q34	3485	IGFBP2	insulin-like growth factor binding protein 2, 36kDa	---	696214	MmugDNA.19111.1.S1_at_IGFBP2
MmugDNA.1911.1.S1_at	221249_s_at	FAM117A	family with sequence similarity 117, member A	chr17q21.33	81558	FAM117A	family with sequence similarity 117, member A	---	700079	MmugDNA.1911.1.S1_at_FAM117A
MmugDNA.19112.1.S1_at	227873_at	TXNDC15	thioredoxin domain containing 15	chr5q31.1	79770	---	---	---	---	MmugDNA.19112.1.S1_at_TXNDC15
MmugDNA.19113.1.S1_at	202628_s_at	SERPINE1	serpin peptidase inhibitor, clade E (nexin, plasminogen activator inhibitor type 1), member 1	chr7q21.3-q22	5054	---	---	---	---	MmugDNA.19113.1.S1_at_SERPINE1
MmugDNA.19114.1.S1_at	236658_at	---	---	---	---	---	---	---	---	MmugDNA.19114.1.S1_at_---
MmugDNA.19115.1.S1_at	1559494_at	---	---	---	---	---	---	---	---	MmugDNA.19115.1.S1_at_---
MmugDNA.19116.1.S1_at	240129_at	---	---	---	---	---	---	---	---	MmugDNA.19116.1.S1_at_---
MmugDNA.19117.1.S1_at	1554717_a_at	PDE4D	phosphodiesterase 4D, cAMP-specific (phosphodiesterase E3 dunce homolog, Drosophila)	chr5q12	5144	---	---	---	---	MmugDNA.19117.1.S1_at_PDE4D
MmugDNA.19118.1.S1_at	225710_at	GNB4	guanine nucleotide binding protein (G protein), beta polypeptide 4	chr3q26.32-q26.33	59345	---	---	---	---	MmugDNA.19118.1.S1_at_GNB4
MmugDNA.19119.1.S1_at	227095_at	---	---	---	---	---	---	---	---	MmugDNA.19119.1.S1_at_---
MmugDNA.19119.1.S1_s_at	NA	NA	NA	NA	NA	NA	NA	NA	NA	MmugDNA.19119.1.S1_s_at_NA
MmugDNA.191.1.S1_at	1569758_at	---	---	---	---	---	---	---	---	MmugDNA.191.1.S1_at_---
MmugDNA.19120.1.S1_at	231798_at	NOG	noggin	chr17q21-q22	9241	---	---	---	---	MmugDNA.19120.1.S1_at_NOG
MmugDNA.19121.1.S1_at	237734_s_at	---	---	---	---	---	---	---	---	MmugDNA.19121.1.S1_at_---
MmugDNA.1912.1.S1_at	206303_s_at	NUDT4	nudix (nucleoside diphosphate linked moiety X)-type motif 4	chr12q21	11163	LOC715385	similar to nudix-type motif 4 isoform alpha	---	715385	MmugDNA.1912.1.S1_at_NUDT4
MmugDNA.19122.1.S1_at	214867_at	---	---	---	---	---	---	---	---	MmugDNA.19122.1.S1_at_---
MmugDNA.19123.1.S1_at	NA	NA	NA	NA	NA	NA	NA	NA	NA	MmugDNA.19123.1.S1_at_NA
MmugDNA.19124.1.S1_at	237011_at	LOC643837	Hypothetical LOC643837	chr1p36.33	643837	---	---	---	---	MmugDNA.19124.1.S1_at_LOC643837
MmugDNA.19125.1.S1_at	223609_at	ROPN1L	ropporin 1-like	chr5p15.2	83853	ROPN1L	ropporin 1-like	---	697006	MmugDNA.19125.1.S1_at_ROPN1L
MmugDNA.19126.1.S1_at	210163_at	CXCL11	chemokine (C-X-C motif) ligand 11	chr4q21.2	6373	---	---	---	---	MmugDNA.19126.1.S1_at_CXCL11
MmugDNA.19127.1.S1_s_at	218269_at	RNASEN	ribonuclease type III, nuclear	chr5p13.3	29102	---	---	---	---	MmugDNA.19127.1.S1_s_at_RNASEN
MmugDNA.19128.1.S1_at	234058_at	---	---	---	---	---	---	---	---	MmugDNA.19128.1.S1_at_---
MmugDNA.19129.1.S1_at	235029_at	GINS4	GINS complex subunit 4 (Sld5 homolog)	chr8p11.21	84296	GINS4	GINS complex subunit 4 (Sld5 homolog)	---	703350	MmugDNA.19129.1.S1_at_GINS4
MmugDNA.19130.1.S1_at	234099_at	---	---	---	---	---	---	---	---	MmugDNA.19130.1.S1_at_---
MmugDNA.19131.1.S1_at	NA	NA	NA	NA	NA	NA	NA	NA	NA	MmugDNA.19131.1.S1_at_NA
MmugDNA.1913.1.S1_at	227525_at	GLCCI1	glucocorticoid induced transcript 1	chr7p21.3	113263	LOC696004	similar to glucocorticoid induced transcript 1	---	696004	MmugDNA.1913.1.S1_at_GLCCI1
MmugDNA.19132.1.S1_at	227358_at	ZBTB46	zinc finger and BTB domain containing 46	chr20q13.33	140685	ZBTB46	zinc finger and BTB domain containing 46	---	695166	MmugDNA.19132.1.S1_at_ZBTB46
MmugDNA.19133.1.S1_at	244172_at	---	---	---	---	---	---	---	---	MmugDNA.19133.1.S1_at_---
MmugDNA.19134.1.S1_at	208753_s_at	NAP1L1	nucleosome assembly protein 1-like 1	chr12q21.2	4673	LOC695614 /// LOC7043	hypothetical protein LOC695614 /// similar to Nucleosome assembly protein 1-like 1 (NAP-1-related p	---	695614 /// 704319 /// 7146	MmugDNA.19134.1.S1_at_NAP1L1

MmugDNA.19135.1.S1_at	226377_at	---	---	---	---	---	---	---	---		MmugDNA.19135.1.S1_at_---
MmugDNA.19136.1.S1_at	213294_at	EIF2AK2	eukaryotic translation initiation factor 2-alpha kinase 2	chr2p22-p21	5610	---	---	---	---		MmugDNA.19136.1.S1_at_EIF2AK2
MmugDNA.19137.1.S1_at	1561132_at	DHX35	DEAH (Asp-Glu-Ala-His) box polypeptide 35	chr20q11.22-q12	60625	---	---	---	---		MmugDNA.19137.1.S1_at_DHX35
MmugDNA.19138.1.S1_at	205581_s_at	NOS3	nitric oxide synthase 3 (endothelial cell)	chr7q36	4846	NOS3	nitric oxide synthase 3 (endothelial cell)	---		714231	MmugDNA.19138.1.S1_at_NOS3
MmugDNA.19139.1.S1_at	231649_at	---	---	---	---	---	---	---	---		MmugDNA.19139.1.S1_at_---
MmugDNA.19140.1.S1_at	1555613_a_at	ZAP70	zeta-chain (TCR) associated protein kinase 70kDa	chr2q12	7535	ZAP70	zeta-chain (TCR) associated protein kinase 70kDa	---		707555	MmugDNA.19140.1.S1_at_ZAP70
MmugDNA.19141.1.S1_at	237414_at	F7	coagulation factor VII (serum prothrombin conversion accelerator)	chr13q34	2155	---	---	---	---		MmugDNA.19141.1.S1_at_F7
MmugDNA.1914.1.S1_at	236725_at	WWC1	WW and C2 domain containing 1	chr5q35.1	23286	WWC1	WW and C2 domain containing 1	---		698875	MmugDNA.1914.1.S1_at_WWC1
MmugDNA.19142.1.S1_at	1552985_at	SLC30A8	solute carrier family 30 (zinc transporter), member 8	chr8q24.11	169026	SLC30A8	solute carrier family 30 (zinc transporter), member 8	---		701127	MmugDNA.19142.1.S1_at_SLC30A8
MmugDNA.19143.1.S1_at	229879_at	---	---	---	---	---	---	---	---		MmugDNA.19143.1.S1_at_---
MmugDNA.19144.1.S1_at	241356_at	---	---	---	---	---	---	---	---		MmugDNA.19144.1.S1_at_---
MmugDNA.19145.1.S1_at	235871_at	LIPH	lipase, member H	chr3q27	200879	LIPH	similar to lipase, member H precursor	---		701743	MmugDNA.19145.1.S1_at_LIPH
MmugDNA.19146.1.S1_at	1568742_at	---	---	---	---	---	---	---	---		MmugDNA.19146.1.S1_at_---
MmugDNA.19147.1.S1_at	242771_at	TTN	Titin	chr2q31	7273	---	---	---	---		MmugDNA.19147.1.S1_at_TTN
MmugDNA.19148.1.S1_at	228515_at	LOC90784	hypothetical protein LOC90784	chr2p11.2	90784	---	---	---	---		MmugDNA.19148.1.S1_at_LOC90784
MmugDNA.19149.1.S1_at	242425_at	---	---	---	---	---	---	---	---		MmugDNA.19149.1.S1_at_---
MmugDNA.19150.1.S1_at	1557883_a_at	---	---	---	---	---	---	---	---		MmugDNA.19150.1.S1_at_---
MmugDNA.19151.1.S1_at	243358_at	IGF1R	insulin-like growth factor 1 receptor	chr15q26.3	3480	---	---	---	---		MmugDNA.19151.1.S1_at_IGF1R
MmugDNA.1915.1.S1_at	229360_at	ZNF280B	zinc finger protein 280B	chr22q11.22	140883	---	---	---	---		MmugDNA.1915.1.S1_at_ZNF280B
MmugDNA.19152.1.S1_at	240964_at	---	---	---	---	---	---	---	---		MmugDNA.19152.1.S1_at_---
MmugDNA.19153.1.S1_at	230717_at	LCN12	lipocalin 12	chr9q34.3	286256	LCN2	similar to Epididymal-specific lipocalin-12 precursor	---		708099	MmugDNA.19153.1.S1_at_LCN12
MmugDNA.19154.1.S1_at	231053_at	---	---	---	---	---	---	---	---		MmugDNA.19154.1.S1_at_---
MmugDNA.19155.1.S1_at	1558636_s_at	ADAMTS5	ADAM metallopeptidase with thrombospondin type 1 motif, 5	chr21q21.3	11096	ADAMTS5	ADAM metallopeptidase with thrombospondin type 1 motif, 5	---		707672	MmugDNA.19155.1.S1_at_ADAMTS5
MmugDNA.19156.1.S1_s_at	231319_x_at	KIF9	kinesin family member 9	chr3p21.31	64147	LOC711742	similar to kinesin family member 9 isoform 2	---		711742	MmugDNA.19156.1.S1_s_at_KIF9
MmugDNA.19157.1.S1_at	1554837_a_at	CYP4A11 /// CYP4A22	cytochrome P450, family 4, subfamily A, polypeptide 11 /// cytochrome P450, family 4, subfamily A, p	chr1p33	1579 /// 284541	---	---	---	---		MmugDNA.19157.1.S1_at_CYP4A11 ///
MmugDNA.19158.1.S1_at	201831_s_at	USO1	USO1 homolog, vesicle docking protein (yeast)	chr4q21.1	8615	---	---	---	---		MmugDNA.19158.1.S1_at_USO1
MmugDNA.19159.1.S1_at	238875_at	---	---	---	---	---	---	---	---		MmugDNA.19159.1.S1_at_---
MmugDNA.19160.1.S1_at	221648_s_at	---	---	---	---	---	---	---	---		MmugDNA.19160.1.S1_at_---
MmugDNA.19161.1.S1_at	229251_s_at	TPCN2	two pore segment channel 2	chr11q13.2	219931	LOC709391	similar to two pore segment channel 2	---		709391	MmugDNA.19161.1.S1_at_TPCN2
MmugDNA.1916.1.S1_at	210109_at	C7orf54	chromosome 7 open reading frame 54	chr7q31	27099	---	---	---	---		MmugDNA.1916.1.S1_at_C7orf54
MmugDNA.19162.1.S1_at	207309_at	NOS1	nitric oxide synthase 1 (neuronal)	chr12q24.2-q24.31	4842	---	---	---	---		MmugDNA.19162.1.S1_at_NOS1
MmugDNA.19163.1.S1_at	206767_at	RBMS3	RNA binding motif, single stranded interacting protein	chr3p24-p23	27303	LOC702852	similar to RNA binding motif, single stranded interacting protein 3 isoform 1	---		702852	MmugDNA.19163.1.S1_at_RBMS3
MmugDNA.19164.1.S1_at	227500_at	FBXL18	F-box and leucine-rich repeat protein 18	chr7p22.2	80028	---	---	---	---		MmugDNA.19164.1.S1_at_FBXL18
MmugDNA.19165.1.S1_at	211845_at	PVRL1	poliovirus receptor-related 1 (herpesvirus entry mediator C)	chr11q23.3	5818	---	---	---	---		MmugDNA.19165.1.S1_at_PVRL1
MmugDNA.19166.1.S1_at	218117_at	RBX1	ring-box 1	chr22q13.2	9978	LOC708350	similar to ring-box 1	---		708350	MmugDNA.19166.1.S1_at_RBX1
MmugDNA.19167.1.S1_at	215432_at	ACSM1	acyl-CoA synthetase medium-chain family member 1	chr16p12.2	116285	LOC696498	similar to acyl-CoA synthetase medium-chain family member 1	---		696498	MmugDNA.19167.1.S1_at_ACSM1
MmugDNA.19168.1.S1_at	202496_at	EDC4	enhancer of mRNA decapping 4	chr16q22.1	23644	EDC4	similar to autoantigen RCD8	---		701393	MmugDNA.19168.1.S1_at_EDC4
MmugDNA.19169.1.S1_at	NA	NA	NA	NA	NA	NA	NA	NA	NA		MmugDNA.19169.1.S1_at_NA
MmugDNA.19170.1.S1_at	209748_at	SPAST	spastin	chr2p24-p21	6683	LOC706264	similar to spastin isoform 1	---		706264	MmugDNA.19170.1.S1_at_SPAST
MmugDNA.19171.1.S1_at	1565846_at	---	---	---	---	---	---	---	---		MmugDNA.19171.1.S1_at_---
MmugDNA.19172.1.S1_at	242659_at	---	---	---	---	---	---	---	---		MmugDNA.19172.1.S1_at_---
MmugDNA.19173.1.S1_at	1555895_at	DNM2	dynamin 2	chr19p13.2	1785	DNM2	dynamin 2	---		713558	MmugDNA.19173.1.S1_at_DNM2
MmugDNA.19174.1.S1_at	215002_at	LOC100132247 /// LOC	similar to Uncharacterized protein KIAA0220 /// hypothetical LOC642778 /// NPIP-like protein ENSP0	chr16p11.2 /// chr16p12.1 /// chr1	100132247 /// 23117 //	---	---	---	---		MmugDNA.19174.1.S1_at_LOC100132
MmugDNA.19175.1.S1_at	1556649_at	---	---	---	---	---	---	---	---		MmugDNA.19175.1.S1_at_---
MmugDNA.19176.1.S1_at	238144_s_at	---	---	---	---	---	---	---	---		MmugDNA.19176.1.S1_at_---
MmugDNA.19177.1.S1_at	1559474_at	SPEG	SPEG complex locus	chr2q35	10290	---	---	---	---		MmugDNA.19177.1.S1_at_SPEG
MmugDNA.19178.1.S1_at	217120_s_at	MED14	mediator complex subunit 14	chrXp11.4-p11.2	9282	MED14	mediator complex subunit 14	---		699395	MmugDNA.19178.1.S1_at_MED14
MmugDNA.19179.1.S1_at	217659_at	---	---	---	---	---	---	---	---		MmugDNA.19179.1.S1_at_---
MmugDNA.19180.1.S1_at	232755_at	UBE2G2	ubiquitin-conjugating enzyme E2G 2 (UBC7 homolog, yeast)	chr21q22.3	7327	---	---	---	---		MmugDNA.19180.1.S1_at_UBE2G2
MmugDNA.1918.1.S1_at	238094_at	---	---	---	---	---	---	---	---		MmugDNA.1918.1.S1_at_---
MmugDNA.19182.1.S1_at	1555033_a_at	RGS12	regulator of G-protein signaling 12	chr4p16.3	6002	LOC699927	similar to regulator of G-protein signalling 12 isoform 1	---		699927	MmugDNA.19182.1.S1_at_RGS12
MmugDNA.19183.1.S1_at	1554579_a_at	MYO18B	myosin XVIIIB	chr22q11.2-q12.1	84700	MYO18B	myosin XVIIIB	---		714823	MmugDNA.19183.1.S1_at_MYO18B
MmugDNA.19184.1.S1_at	237756_at	KLHL23	Kelch-like 23 (Drosophila)	chr2q31.1	151230	---	---	---	---		MmugDNA.19184.1.S1_at_KLHL23
MmugDNA.19185.1.S1_at	229681_at	---	---	---	---	---	---	---	---		MmugDNA.19185.1.S1_at_---
MmugDNA.19186.1.S1_at	242431_at	---	---	---	---	---	---	---	---		MmugDNA.19186.1.S1_at_---
MmugDNA.19187.1.S1_at	229444_at	LOC729776	hypothetical LOC729776	chr6p21.31	729776	---	---	---	---		MmugDNA.19187.1.S1_at_LOC729776
MmugDNA.19188.1.S1_at	204191_at	IFNAR1	interferon (alpha, beta and omega) receptor 1	chr21q22.1|21q22.11	3454	LOC709795	Similar to Keratin, type II cytoskeletal 8 (Cytokeratin-8) (CK-8) (Keratin-8) (K8)	---		709795	MmugDNA.19188.1.S1_at_IFNAR1
MmugDNA.19189.1.S1_at	218943_s_at	DDX58	DEAD (Asp-Glu-Ala-Asp) box polypeptide 58	chr9p12	23586	DDX58	DEAD (Asp-Glu-Ala-Asp) box polypeptide 58	---		704638	MmugDNA.19189.1.S1_at_DDX58
MmugDNA.19190.1.S1_at	215590_x_at	LOC100128640	Hypothetical protein LOC100128640	chr3p22.2	100128640	---	---	---	---		MmugDNA.19190.1.S1_at_LOC100128
MmugDNA.19191.1.S1_at	208031_s_at	RFX2	regulatory factor X, 2 (influences HLA class II expression)	chr19p13.3-p13.2	5990	---	---	---	---		MmugDNA.19191.1.S1_at_RFX2
MmugDNA.1919.1.S1_at	1569891_at	ATP5A1	ATP synthase, H+ transporting, mitochondrial F1 complex, alpha subunit 1, cardiac muscle	chr18q12-q21	498	---	---	---	---		MmugDNA.1919.1.S1_at_ATP5A1
MmugDNA.19192.1.S1_at	237421_at	---	---	---	---	---	---	---	---		MmugDNA.19192.1.S1_at_---
MmugDNA.19193.1.S1_at	230390_at	---	---	---	---	---	---	---	---		MmugDNA.19193.1.S1_at_---
MmugDNA.19194.1.S1_at	234911_at	---	---	---	---	---	---	---	---		MmugDNA.19194.1.S1_at_---
MmugDNA.19195.1.S1_at	205770_at	GSR	glutathione reductase	chr8p21.1	2936	---	---	---	---		MmugDNA.19195.1.S1_at_GSR
MmugDNA.19196.1.S1_at	NA	NA	NA	NA	NA	NA	NA	NA	NA		MmugDNA.19196.1.S1_at_NA
MmugDNA.19197.1.S1_at	241524_at	---	---	---	---	---	---	---	---		MmugDNA.19197.1.S1_at_---
MmugDNA.19198.1.S1_at	244235_at	IVNS1ABP	influenza virus NS1A binding protein	chr1q25.1-q31.1	10625	---	---	---	---		MmugDNA.19198.1.S1_at_IVNS1ABP
MmugDNA.19199.1.S1_at	241632_x_at	---	---	---	---	---	---	---	---		MmugDNA.19199.1.S1_at_---
MmugDNA.19.1.S1_at	1552367_a_at	SCIN	scinderin	chr7p21.3	85477	SCIN	scinderin	---		693551	MmugDNA.19.1.S1_at_SCIN
MmugDNA.19200.1.S1_at	1559252_a_at	C20orf29	Chromosome 20 open reading frame 29	chr20p13	55317	---	---	---	---		MmugDNA.19200.1.S1_at_C20orf29
MmugDNA.19201.1.S1_at	201380_at	CRTAP	cartilage associated protein	chr3p22.3	10491	CRTAP	cartilage associated protein	---		705949	MmugDNA.19201.1.S1_at_CRTAP
MmugDNA.1920.1.S1_at	223211_at	HACL1	2-hydroxyacyl-CoA lyase 1	chr3p24.3	26061	HACL1	2-hydroxyacyl-CoA lyase 1	---		694261	MmugDNA.1920.1.S1_at_HACL1
MmugDNA.19202.1.S1_at	236153_at	---	---	---	---	---	---	---	---		MmugDNA.19202.1.S1_at_---
MmugDNA.19203.1.S1_at	201947_s_at	CCT2	chaperonin containing TCP1, subunit 2 (beta)	chr12q15	10576	CCT2	chaperonin containing TCP1, subunit 2	---		717182	MmugDNA.19203.1.S1_at_CCT2
MmugDNA.19204.1.S1_at	208636_at	ACTN1	actinin, alpha 1	chr14q24.1-q24.2|14q24|14q22-	87	LOC712933	similar to Alpha-actinin-1 (Alpha-actinin cytoskeletal isoform) (Non-muscle alpha-actinin-1) (F-actin cr	---		712933	MmugDNA.19204.1.S1_at_ACTN1
MmugDNA.19205.1.S1_at	1566445_at	---	---	---	---	---	---	---	---		MmugDNA.19205.1.S1_at_---
MmugDNA.19206.1.S1_at	1564772_at	---	---	---	---	---	---	---	---		MmugDNA.19206.1.S1_at_---
MmugDNA.19207.1.S1_at	200877_at	CCT4	chaperonin containing TCP1, subunit 4 (delta)	chr2p15	10575	LOC719649	similar to chaperonin containing TCP1, subunit 4 (delta)	---		719649	MmugDNA.19207.1.S1_at_CCT4
MmugDNA.19208.1.S1_at	200812_at	CCT7	chaperonin containing TCP1, subunit 7 (eta)	chr2p13.2	10574	LOC713340	similar to T-complex protein 1 subunit eta (TCP-1-eta) (CCT-eta) (HIV-1 Nef-interacting protein)	---		713340	MmugDNA.19208.1.S1_at_CCT7
MmugDNA.19209.1.S1_s_at	212531_at	LCN2	lipocalin 2	chr9q34	3934	LCN2	lipocalin 2	---		697208	MmugDNA.19209.1.S1_s_at_LCN2
MmugDNA.19210.1.S1_at	232147_at	BTBD12	BTB (POZ) domain containing 12	chr16p13.3	84464	---	---	---	---		MmugDNA.19210.1.S1_at_BTBD12
MmugDNA.19211.1.S1_at	230747_s_at	TTC39C	tetratricopeptide repeat domain 39C	chr18q11.2	125488	---	---	---	---		MmugDNA.19211.1.S1_at_TTC39C
MmugDNA.1921.1.S1_at	228647_at	LOC100049716	hypothetical protein LOC100049716	chr12p13.33	100049716	---	---	---	---		MmugDNA.1921.1.S1_at_LOC1000497
MmugDNA.19213.1.S1_at	227248_at	PLEKHH3	pleckstrin homology domain containing, family H (with MyTH4 domain) member 3	chr17q21.31	79990	LOC711013	similar to unconventional myosin class XV CG2174-PA	---		711013	MmugDNA.19213.1.S1_at_PLEKHH3
MmugDNA.19214.1.S1_at	204534_at	VTN	vitronectin	chr17q11	7448	VTN	vitronectin	---		708499	MmugDNA.19214.1.S1_at_VTN
MmugDNA.19215.1.S1_at	238185_at	---	---	---	---	---	---	---	---		MmugDNA.19215.1.S1_at_---
MmugDNA.19216.1.S1_at	210755_at	HGF	hepatocyte growth factor (hepapoietin A; scatter factor)	chr7q21.1	3082	---	---	---	---		MmugDNA.19216.1.S1_at_HGF
MmugDNA.19217.1.S1_at	226336_at	PPIA	peptidylprolyl isomerase A (cyclophilin A)	chr7p13	5478	---	---	---	---		MmugDNA.19217.1.S1_at_PPIA
MmugDNA.19218.1.S1_at	240386_at	TRPM1	Transient receptor potential cation channel, subfamily M, member 1	chr15q13-q14	4308	---	---	---	---		MmugDNA.19218.1.S1_at_TRPM1
MmugDNA.19219.1.S1_at	207012_at	MMP16	matrix metallopeptidase 16 (membrane-inserted)	chr8q21.3	4325	LOC695120	similar to matrix metalloproteinase 16 isoform 2 preproprotein	---		695120	MmugDNA.19219.1.S1_at_MMP16
MmugDNA.192.1.S1_at	1559214_at	---	---	---	---	---	---	---	---		MmugDNA.192.1.S1_at_---
MmugDNA.19220.1.S1_at	220105_at	RTDR1	rhabdoid tumor deletion region gene 1	chr22q11.2	27156	RTDR1	rhabdoid tumor deletion region gene 1	---		706814	MmugDNA.19220.1.S1_at_RTDR1
MmugDNA.1922.1.S1_at	222658_s_at	APTX	aprataxin	chr9p13.3	54840	APTX	aprataxin	---		702671	MmugDNA.1922.1.S1_at_APTX
MmugDNA.19222.1.S1_at	229768_at	OR51E1	olfactory receptor, family 51, subfamily E, member 1	chr11p15.4	143503	LOC717598	similar to olfactory receptor, family 51, subfamily E, member 1	---		717598	MmugDNA.19222.1.S1_at_OR51E1
MmugDNA.19223.1.S1_at	1563376_at	---	---	---	---	---	---	---	---		MmugDNA.19223.1.S1_at_---
MmugDNA.19224.1.S1_at	208935_s_at	LGALS8	lectin, galactoside-binding, soluble, 8	chr1q42-q43	3964	LOC710375	similar to galectin 8 isoform a	---		710375	MmugDNA.19224.1.S1_at_LGALS8
MmugDNA.19225.1.S1_at	228621_at	HFE2	hemochromatosis type 2 (juvenile)	chr1q21.1	148738	HFE2	hemochromatosis type 2 (juvenile)	---		698805	MmugDNA.19225.1.S1_at_HFE2
MmugDNA.19226.1.S1_at	212272_at	LPIN1	lipin 1	chr2p25.1	23175	---	---	---	---		MmugDNA.19226.1.S1_at_LPIN1
MmugDNA.19227.1.S1_at	212274_at	LPIN1	lipin 1	chr2p25.1	23175	---	---	---	---		MmugDNA.19227.1.S1_at_LPIN1
MmugDNA.19228.1.S1_at	1562861_at	---	---	---	---	---	---	---	---		MmugDNA.19228.1.S1_at_---
MmugDNA.19229.1.S1_at	212276_at	LPIN1	lipin 1	chr2p25.1	23175	---	---	---	---		MmugDNA.19229.1.S1_at_LPIN1
MmugDNA.19230.1.S1_at	221739_at	C19orf10	chromosome 19 open reading frame 10	chr19p13.3	56005	C19H19orf10	similar to chromosome 19 open reading frame 10	---		695477	MmugDNA.19230.1.S1_at_C19orf10
MmugDNA.19230.1.S1_s_at	NA	NA	NA	NA	NA	NA	NA	NA	NA		MmugDNA.19230.1.S1_s_at_NA
MmugDNA.19231.1.S1_at	206545_at	CD28	CD28 molecule	chr2q33	940	---	---	---	---		MmugDNA.19231.1.S1_at_CD28
MmugDNA.19232.1.S1_at	229340_at	NKAPL	NFKB activating protein-like	chr6p22.1	222698	LOC693565	Similar to progesterone membrane binding protein	---		693565	MmugDNA.19232.1.S1_at_NKAPL
MmugDNA.19233.1.S1_at	225403_at	C9orf23	chromosome 9 open reading frame 23	chr9p13.3	138716	C15H9orf23	chromosome 9 open reading frame 23 ortholog	---		700829	MmugDNA.19233.1.S1_at_C9orf23
MmugDNA.19234.1.S1_at	232845_at	CDH23	cadherin-like 23	chr10q21-q22	64072	CDH23	cadherin related 23	---		709433	MmugDNA.19234.1.S1_at_CDH23

MmugDNA.19235.1.S1_at	240912_x_at	TMEM84	transmembrane protein 84	chr15q23	283673	---	---	---	---	MmugDNA.19235.1.S1_at_TMEM84
MmugDNA.19236.1.S1_at	201541_s_at	ZNHIT1	zinc finger, HIT type 1	chr7q22.1	10467	ZNHIT1	zinc finger, HIT type 1	---	714339	MmugDNA.19236.1.S1_at_ZNHIT1
MmugDNA.19237.1.S1_at	1557529_at	C12orf51	chromosome 12 open reading frame 51	chr12q24.13	283450	LOC711805	hypothetical protein LOC711805	---	711805	MmugDNA.19237.1.S1_at_C12orf51
MmugDNA.19238.1.S1_at	215017_s_at	FNBP1L	formin binding protein 1-like	chr1p22.1	54874	---	---	---	---	MmugDNA.19238.1.S1_at_FNBP1L
MmugDNA.19239.1.S1_at	209599_s_at	PRUNE	prune homolog (Drosophila)	chr1q21	58497	LOC708898	similar to prune homolog	---	708898	MmugDNA.19239.1.S1_at_PRUNE
MmugDNA.19240.1.S1_at	238616_at	QDPR	Quinoid dihydropteridine reductase	chr4p15.31	5860	---	---	---	---	MmugDNA.19240.1.S1_at_QDPR
MmugDNA.19241.1.S1_at	243170_at	---	---	---	---	---	---	---	---	MmugDNA.19241.1.S1_at_---
MmugDNA.1924.1.S1_at	205471_s_at	DACH1	dachshund homolog 1 (Drosophila)	chr13q22	1602	DACH1	dachshund homolog 1 (Drosophila)	---	693532	MmugDNA.1924.1.S1_at_DACH1
MmugDNA.19242.1.S1_at	NA	NA	NA	NA	NA	NA	NA	NA	NA	MmugDNA.19242.1.S1_at_NA
MmugDNA.19243.1.S1_at	231124_x_at	LY9	lymphocyte antigen 9	chr1q21.3-q22	4063	---	---	---	---	MmugDNA.19243.1.S1_at_LY9
MmugDNA.19244.1.S1_at	215901_at	ZNF81	zinc finger protein 81	chrXp11.23	347344	ZNF81	zinc finger protein 81	---	708036	MmugDNA.19244.1.S1_at_ZNF81
MmugDNA.19245.1.S1_at	1552798_a_at	TLR4	toll-like receptor 4	chr9q32-q33	7099	TLR4	toll-like receptor 4	---	574360	MmugDNA.19245.1.S1_at_TLR4
MmugDNA.19246.1.S1_s_at	204030_s_at	SCHIP1	schwannomin interacting protein 1	chr3q25.33	29970	SCHIP1	schwannomin interacting protein 1	---	703321	MmugDNA.19246.1.S1_s_at_SCHIP1
MmugDNA.19247.1.S1_at	217381_s_at	TRGV5	T cell receptor gamma variable 5	chr7p14	6978	LOC712882	similar to Ig kappa chain V-II region 17S29.1	---	712882	MmugDNA.19247.1.S1_at_TRGV5
MmugDNA.19248.1.S1_at	230814_at	LOC342918	similar to mCG134545	chr19q13.33	342918	LOC719661	hypothetical protein LOC719661	---	719661	MmugDNA.19248.1.S1_at_LOC342918
MmugDNA.19249.1.S1_at	NA	NA	NA	NA	NA	NA	NA	NA	NA	MmugDNA.19249.1.S1_at_NA
MmugDNA.19250.1.S1_at	232485_at	RUNDC2A	RUN domain containing 2A	chr16p13.13	84127	LOC716896	similar to RUN domain containing 2A	---	716896	MmugDNA.19250.1.S1_at_RUNDC2A
MmugDNA.19251.1.S1_at	228116_at	---	---	---	---	---	---	---	---	MmugDNA.19251.1.S1_at_---
MmugDNA.1925.1.S1_at	221245_s_at	FZD5	frizzled homolog 5 (Drosophila)	chr2q33-q34	7855	LOC710796	similar to frizzled 5	---	710796	MmugDNA.1925.1.S1_at_FZD5
MmugDNA.19252.1.S1_at	203306_s_at	SLC35A1	solute carrier family 35 (CMP-sialic acid transporter), member A1	chr6q15	10559	SLC35A1	solute carrier family 35 (CMP-sialic acid transporter), member A1	---	699113	MmugDNA.19252.1.S1_at_SLC35A1
MmugDNA.19253.1.S1_at	1554818_s_at	C20orf12	chromosome 20 open reading frame 12	chr20p11.23	55184	C10H20orf12	chromosome 20 open reading frame 12 ortholog	---	700143	MmugDNA.19253.1.S1_at_C20orf12
MmugDNA.19254.1.S1_at	213609_s_at	SEZ6L	seizure related 6 homolog (mouse)-like	chr22q12.1	23544	SEZ6L	seizure related 6 homolog (mouse)-like	---	714925	MmugDNA.19254.1.S1_at_SEZ6L
MmugDNA.19255.1.S1_at	205103_at	C1orf61	chromosome 1 open reading frame 61	chr1q22	10485	LOC715758	Similar to Down syndrome critical region protein 2 isoform a	---	715758	MmugDNA.19255.1.S1_at_C1orf61
[truncated: 6,757,260 more chars]
